# Proceedings of the 28th European Paediatric Rheumatology Congress (PReS 2022): Prague, Czech Republic. 20-23 September 2022

**DOI:** 10.1186/s12969-022-00729-z

**Published:** 2022-09-07

## Oral Communications Session 1 - Autoinflammation/SJIA/MAS

### O01. Modeling HLH & MAS susceptibility identifies the characteristics of hyperinflammatory CD8 T-cells

#### E. Landy^1^, V. Dang^2^, J. Varghese^2^, P. Tsoukas^3^, S. Canna^2^

##### ^1^University of Pittsburgh, Pittsburgh, ^2^The Children’s Hospital of Philadelphia, Philadelphia, United States, ^3^Hospital for Sick Children, Toronto, Canada

###### **Correspondence:** S. Canna

**Introduction:** Life-threatening hyperinflammatory Syndromes like Hemophagocytic Lymphohistiocytosis (HLH) and Macrophage Activation Syndrome (MAS) often result from the interactions of multiple susceptibility factors and environmental insults. Recent studies in both HLH and MAS patients demonstrate CD8 T-cell activation profiles in peripheral blood.

**Objectives:** We wished to test how two independent and synergistic susceptibility factors, perforin insufficiency and excess IL-18, drove pathology in a spontaneous murine model of HLH/MAS.

**Methods:** We bred and examined transgenic mice bearing knock-out alleles in perforin (*Prf1*^*+/-*^ and *-/-*) as well as transgenic expression of mature murine IL-18 (*Il18tg*) by “clinical” measures, cytokine levels, flow cytometry, bulk RNA- and TCR-sequencing, and in functional studies.

**Results:** Mice bearing dual susceptibility factors (DS mice, *Prf1*^*-/-*^*Il18tg*) develop spontaneous HLH/MAS - even *Prf1*^*+/-*^*Il18tg* mice develop spontaneous HLH-like immunopathology - in a manner dependent on IFNg. Detailed flow cytometric organ phenotyping reveals a dense expansion of CD8 T-cells bearing high levels of the IL-18 receptor as well as multiple markers associated with exhaustion (PD-1, Lag-3, CD39), yet overproduce IFNg. Such “hyperinflammatory CD8 T-cells” are present in the reticuloendothelial organs (spleen, bone marrow, and liver) but not lymph nodes, peripheral blood, or thymus. RNAseq analyses of hyperinflammatory CD8 T-cells alongside well-described exhausted cells reflects a pattern of activation distinct from acute effector, effector-memory, tissue-resident memory, or exhaustion. Multiple features suggest cells that have received recent antigen stimulation but are terminally-differentiated. TCR sequencing of bulk splenic CD8 T-cells shows oligoclonal expansion in DS mice. Attempts to circumvent CD8 T-cell activation, using inducible deletion of Il18r1 only on CD8 T-cells, or by promoting allelic exclusion by fixing the T-cell receptor of DS mice, shows that IL-18 responsive, oligoclonal CD8 T-cells circumvent these efforts to hinder their ability to expand. In vitro studies show that the effects of IL-18 require recent TCR stimulation, but do not inhibit activation-induced cell death.

**Conclusion:** Both perforin deficiency and excess IL-18 seem to exert preferential effects on post-thymically activated CD8 T-cells in reticuloendothelial organs. These are precisely the sites where hemophagocytosis is most commonly observed. These data suggest a requirement for TCR stimulation reminiscent of the infectious triggers common to HLH and MAS, demonstrate these cells remarkable resilience, and identify potentially-targetable nodes of T-cell activation.

**Patient Consent:** Not applicable (there are no patient data)

**Disclosure of Interest**: E. Landy: None declared, V. Dang: None declared, J. Varghese: None declared, P. Tsoukas: None declared, S. Canna Grant / Research Support with: AB2Bio, Novartis, SOBI, IMMvention Therapeutix, Consultant with: Simcha Therapeutics, Speaker Bureau with: Clinical Viewpoints

### O02. Characteristics and disease course of patients with systemic juvenile idiopathic arthritis without arthritis in the German AID-NET cohort

#### C. Hinze^1^, H. Wittkowski^1^, E. Lainka^2^, J.-P. Haas^3^, T. Kallinich^4^, D. Föll^1^ on behalf of for the AID-registry investigators

##### ^1^Pediatric Rheumatology and Immunology, University Hospital Münster, Münster, ^2^Pediatrics II, University Hospital of Essen, Essen, ^3^German Centre for Paediatric and Adolescent Rheumatology, Garmisch-Partenkirchen, ^4^Paediatric Rheumatology, Charity-University Medicine, Berlin, Germany

###### **Correspondence:** C. Hinze

**Introduction:** In systemic juvenile idiopathic arthritis (SJIA), arthritis is often absent during the initial presentation and may not develop at all in some patients (pts). It is unclear how SJIA pts with and without arthritis differ otherwise.

**Objectives:** To evaluate the clinical characteristics and disease courses in pts diagnosed with SJIA and compare those between pts who had or developed chronic arthritis and those who did not.

**Methods:** The German AID-Net cohort enrolled pts between 2009 and 2018, some of them retrospectively. Pts with physician-diagnosed SJIA were analyzed in regards to clinical and laboratory parameters at study inclusion and during the disease course. Pts were considered in the arthritis group if arthritis was recorded at the time of enrolment and/or if it was recorded during the disease course for a duration of at least 6 weeks. Distributions and frequencies of clinical parameters were compared between the groups.

**Results:** The study included 262 pts with SJIA, with a median (interquartile range) age at inclusion of 10.2 (6.0-14.4) yrs, a median age at diagnosis of 7.6 (3.7-12.0) yrs and a median follow-up of 4.1 (1.4-8.1) yrs. At baseline, 147 pts (56%) had arthritis recorded, and of the remaining 115 pts (44%), 30 later developed arthritis (11% of all patients) after 11 (2-20) months, i.e., 85 (32%) pts never had arthritis according to the case definition. Demographic data and clinical parameters that differed significantly between the groups (arthritis versus no arthritis) at baseline are shown in the table. The following parameters did not show significant differences in those for whom the parameters was available: sex, time from onset to diagnosis, adenopathy, macrophage activation syndrome (MAS) (11%:14%), leukocyte count, CRP, S100A8/A9, interleukin-18 or CXCL9 at the time of highest S100A12 level. Medication use was more variable in SJIA patients with arthritis, and the following medications were used more frequently in SJIA with arthritis versus never arthritis (chi square p<0.05): methotrexate (81%:48%), anakinra (47%:27%), etanercept (34%:2%), ciclosporin A (27%:12%), azathioprine (19%:4%), adalimumab (18%:1%), and leflunomide (11%:0%). Concerning the outcomes during the disease, significant differences were seen in the proportion of active disease at the last study visit (25%:13%), and the proportion of pts on glucocorticoids (GC) (23%:11%). There were no significant differences concerning MAS (13%:8%) or death (3 [2%]:0 [0%]).

**Conclusion:** In this German registry-based cohort of patients with physician-diagnosed SJIA, about one third of pts did not have chronic arthritis. SJIA pts without arthritis on average were older and inflammatory signs were more frequent at baseline, while MAS was similarly frequent. The pharmacologic therapy was substantially more variable in SJIA with arthritis. During the follow-up, SJIA pts without arthritis tended to have better outcomes, i.e., less frequently active disease, less GC use, and no significant difference in MAS occurrence. Limitations include overall shorter follow-up for SJIA pts without arthritis.

**Patient Consent:** Yes, I received consent

**Disclosure of Interest**: None declared

**Table 1 (abstract O02).** See text for description

Parameter	Arthritis (n=177)	Data available	No arthritis (n=85)	Data available	P value
Age at diagnosis, median (IQR) yrs	5.0 (2.8-9.3)	177	7.8 (3.9-12.5)	85	<0.01*
Duration of follow-up, median (IQR) yrs	5.2 (2.1-9.5)	177	2.4 (1.0-5.1)	85	<0.0001*
Number of visits, median (IQR)	16 (8-29)	177	9 (5-17)	85	0.0001*
Rash at baseline, n (%)	119 (77%)§	155	75 (100%)§	75	<0.0001†§
Serositis at baseline, n (%)	17 (13%)§	133	16 (24%)§	68	0.05†§
(Hepato) splenomegaly at baseline, n (%)	33 (19%)§	146	26 (31%)§	73	0.04†§

*Mann-Whitney U; †chi square; §applied only to available data; IQR, interquartile distance

### O03. Impact of interferon signalling on response to canakinumab treatment in systemic juvenile idiopathic arthritis as revealed by whole blood RNA sequencing

#### T. Hinze, C. Hinze^2^, C. Kessel^2^, A. Huge^2^, C. Farady^3^, S. McCreddin^4^, K. Gandhi^5^, D. Foell^2^

##### ^1^Department of Pediatric Rheumatology and Immunology, ^2^University of Münster, Münster, Germany, ^3^Novartis Pharma AG, Basel, Switzerland, ^4^Novartis Ireland Limited, Dublin, Ireland, ^5^Novartis Pharmaceuticals Corporation, East Hanover, NJ, United States

###### **Correspondence:** T. Hinze

**Introduction:** Active systemic juvenile idiopathic arthritis (SJIA) is characterized by marked innate immune overactivation and dysregulation of immune-related peripheral blood gene expression. Canakinumab (CAN), an interleukin (IL)-1β blocking antibody has shown to be effective in patients with SJIA. We had previously observed that patients with a higher serum protein IL-18:CXCL9 ratio responded better to CAN than those with a lower ratio. However, it is unclear which factors specifically govern differences in treatment response.

**Objectives:** The objective of this study was to identify differences in peripheral blood gene expression patterns between patients with active SJIA who subsequently showed sustained complete response to CAN and those that did not.

**Methods:** Whole blood RNA samples from (1) CAN-naïve patients from an open-label randomized CAN trial (NCT02296424) in active disease (AD), (2) CAN-treated patients enrolled in a randomized controlled CAN trial (NCT00891046) in inactive disease (ID), and (3) paediatric healthy controls (HC) were studied via whole RNAseq on the Illumina NextSeq 2000 platform, assessing 58395 genes overall. For a proof-of-principle analysis, gene expression patterns were compared across SJIA patients with AD (n=10), ID (n=10), and HC (n=10). CAN-naïve patients were further categorized according to their subsequent treatment response. We specifically considered patients with subsequent sustained complete response (SCR) (n=5), i.e., inactive disease or ACR100 response within 4 weeks of treatment, no subsequent flares or macrophage activation syndrome [MAS] during the study, and non-responders (NR) who did not have an ACR30 response (n=4). Differential gene expression was analysed using the R package deseq2. Adjustment of p-value for multiple comparisons was achieved via a false discovery rate (FDR) <0.05 or <0.1.

**Results:** The proof-of-principle analysis demonstrated marked differential gene expression when comparing AD versus both ID and HC, assuming an FDR<0.05 (AD versus HC: 1872 genes up, 326 down; AD versus ID: 2269 up, 618 down). The prominently upregulated genes included multiple neutrophil-related genes (e.g., CD177, CD93, HK3, MMP9, NLRC4, PADI4, NCF4, NCF1B, PSTPIP2, S100A8, S100A9). When comparing 5 patients with SCR and 4 with NR, assuming an FDR <0.1, 98 genes were differentially regulated (14 up in SCR, 84 up in NR). The 14 genes up in SCR included several type 1 interferon (IFN)-regulated genes, including ERAP2, RSAD2 and SIGLEC1. The 84 genes up in NR included multiple erythropoiesis-related genes, including EPB42, GYPC, PRDX2, RHAG, SPTA1, SPTB, TFR2, TMOD1, TFRC, and TRIM58.

**Conclusion:** As expected, there was marked dysregulation of peripheral blood gene expression in patients with active SJIA, prominently including the overexpression or overrepresentation of neutrophil-related genes. Relative overexpression of peripheral blood type 1 IFN-stimulated genes may correlate with an excellent response to CAN. In contrast, relative overexpression of erythropoiesis-related genes, which in turn may correlate with ineffective erythropoiesis/occult MAS and IFN-γ activity, may correlate with a poor response to CAN. In summary, these findings support the notion that dysregulation of the type 1 IFN-IL-18-IFN-γ axis may play a role in the treatment response to CAN in SJIA.

**Patient Consent:** Not applicable (there are no patient data)

**Disclosure of Interest**: T. Hinze Grant / Research Support with: Novartis Pharma AG, C. Hinze: None declared, C. Kessel Grant / Research Support with: Novartis, Consultant with: Novartis, Sobi, A. Huge: None declared, C. Farady Employee with: Novartis Pharma AG, S. McCreddin Employee with: Novartis Ireland Limited, K. Gandhi Employee with: Novartis Pharmaceuticals Corporation, D. Foell Grant / Research Support with: Novartis, Consultant with: Novartis

### O04. Systemic juvenile idiopathic arthritis associated lung disease in Europe

#### C. Bracaglia^1^, F. Minoia^2^, C. Kessel^3^, S. Vastert^4^, M. Pardeo^1^, A. Arduini^1^, O. Basaran^5^, N. Kipper^6^, M. Kostik^7^, M. Glerup^8^, S. Fingerhutova^9^, R. Caorsi^10^, A. Horne^11^, G. Filocamo^2^, H. Wittkowski^3^, M. Jelusic^12^, J. Anton^13^, S. Khaldi-Plassart^14^, A. Belot^14^, G. Horneff^15^, S. Palmer Sarott^16^, E. Cannizzaro Schneider^16^, P. Dolezalova^9^, A. Ravelli^17^, S. Ozen^5^, F. De Benedetti^1^ on behalf of MAS/sJIA working party

##### ^1^Division of Rheumatology, IRCCS Ospedale Pediatrico Bambino Gesù, Roma, ^2^Fondazione IRCCS Ca’ Grande Ospedale Maggiore Policlinico, Milano, Italy, ^3^Department of Pediatric Rheumatology & Immunology, WWU Medical Center (UKM), Munster, Germany, ^4^Pediatric Rheumatology & Immunology, University Medical Center Utrecht, Utrecht, Netherlands, ^5^Department of Pediatrics, Division of Pediatric Rheumatology, ^6^Department of Pediatrics, Division of Pediatric Pulmonology, Hacettepe University, Ankara, Turkey, ^7^Saint-Petersburg State Pediatric Medical University, Saint-Petersburg, Russian Federation, ^8^Department of Pediatrics, Aarhus University Hospital, Aarhus, Denmark, ^9^Centre for Paediatric Rheumatology and Autoinflammatory Diseases, Department of Paediatrics and Inherited Metabolic Disorders, 1st Faculty of Medicine, Charles University and General University Hospital in Prague, Prague, Czech Republic, ^10^Department of pediatrics and Rheumatology, IRRCS Istituto G. Gaslini, Genova, Italy, ^11^Department of pediatric rheumathology, Karolinska University Hospital and Department of pediatrics, Karolinska Institute, Stockholm, Sweden, ^12^Department of Paediatrics, University of Zagreb School of Medicine, University Hospital Centre, Zagreb, Croatia, ^13^Pediatric Rheumatology, Hospital Sant Joan de Déu, Universitat de Barcelona, Barcelona, Spain, ^14^Pediatric Nephrology, Rheumatology, Dermatology Unit, Hôpital Femme Mère Enfant, Hospices Civils de Lyon, Lyon, France, ^15^Pediatrics, Asklepios Clinic Sankt Augustin, Sank Augustin, Germany, ^16^Paediatric Rheumatology, University Children’s Hospital Zurich, Zurich, Switzerland, ^17^IRRCS Istituto Giannina Gaslini and Università degli Studi di Genova, Genova, Italy

###### **Correspondence:** C. Bracaglia

**Introduction:** Chronic parenchymal lung disease (LD) is a new emerging severe life-threatening complication of sJIA. The number of sJIA patients with LD is apparently increasing and interestingly they are reported more frequently in North America. Data regarding frequency and features of sJIA-LD in Europe are not available.

**Objectives:** To evaluate the burden of sJIA-LD in Europe.

**Methods:** Patients with diagnosis of sJIA with LD, including pulmonary alveolar proteinosis (PAP), interstitial lung disease (ILD) and pulmonary arterial hypertension (PAH), followed in European paediatric rheumatology centres were identified through a survey sent to the members of the MAS/SJIA Working Party.

**Results:** Data from 34 sJIA-LD patients, diagnosed in 15 European paediatric rheumatology centres between 2007 and 2022, were collected. 33 patients were Caucasian and 1 was African-American; 21 were female. The median age at sJIA onset was 6 years and LD occurred after a median time of 2 years. 19 patients had a chronic persistent sJIA course, 14 had a polycyclic course and only 1 patient had a monocyclic course; 29 (85%) had active sJIA at time of LD diagnosis. During the disease course, 28 (82%) patients developed MAS, 12 (35%) of whom had MAS at sJIA onset and 19 (56%) had full-blown MAS at time of LD diagnosis; 23 (68%) patients had >1 MAS episode. 28 (82 %) patients were treated with at least one IL-1 or IL-6 inhibitor before LD diagnosis: 15 with canakinumab, 24 with anakinra and 13 with tocilizumab; 13 (38%) patients experienced drug adverse reaction to a cytokine inhibitor: 9 to tocilizumab and 4 to anakinra. 24 (70%) patients developed ILD, 6 (18%) PAP and 4 (12%) PAH. 15 (44%) patients presented acute digital clubbing; 16 (47%) patients developed hypoxia and 9 (26%) developed pulmonary hypertension. A chest CT scan was performed in all patients with evidence of septal thickening, peri-bronchovascular thickening and ground glass opacities in the majority of patients (26, 18 and 18 respectively). In 17 patients a bronchoalveolar lavage was performed and 12 underwent a lung biopsy. The histopathological pattern was alveolar proteinosis in 5 patients, endogenous lipoid pneumonia in 3, vasculitis in 1 and fibrosis in 1. Half of the patients (17) required ICU admission and 6 (18%) died. All the patients were treated with glucocorticoids (GCs) at time of diagnosis, and 26 received IL-1 or IL-6 inhibitor after the diagnosis (13 canakinumab, 20 anakinra, 14 tocilizumab).

**Conclusion:** Lung involvement is an emerging life-threatening complication of sJIA and patients are also diagnosed in Europe. Prompt recognition is crucial and new therapeutic strategies are needed to reduce the risk and improve the outcome of this complication.

**Patient Consent:** Yes, I received consent

**Disclosure of Interest**: C. Bracaglia Consultant with: Sobi, Novartis, F. Minoia Consultant with: Sobi, C. Kessel Grant / Research Support with: Novartis, Consultant with: Novartis, Speaker Bureau with: Sobi, S. Vastert: None declared, M. Pardeo: None declared, A. Arduini: None declared, O. Basaran: None declared, N. Kipper: None declared, M. Kostik: None declared, M. Glerup: None declared, S. Fingerhutova: None declared, R. Caorsi Consultant with: Sobi, Novartis, A. Horne: None declared, G. Filocamo Consultant with: Sobi, H. Wittkowski: None declared, M. Jelusic: None declared, J. Anton Grant / Research Support with: Sobi, Novimmune, Novartis, Abbvie, Pfizer, GSK, Roche, Amgen, Lilly, BMS, Sanofi, Consultant with: Sobi, Novimmune, Novartis, Pfizer, GSK, Speaker Bureau with: Sobi, Novimmune, Novartis, GSK, Pfizer, S. Khaldi-Plassart: None declared, A. Belot Consultant with: Sobi, Novartis, Roche, Pfizer, G. Horneff Grant / Research Support with: MSD, Novartis, Roche, Speaker Bureau with: Abbvie, Chugai, Lilly, Sanofi, Novartis, Pfizer, S. Palmer Sarott: None declared, E. Cannizzaro Schneider: None declared, P. Dolezalova: None declared, A. Ravelli: None declared, S. Ozen Consultant with: Sobi, Novartis, Speaker Bureau with: Sobi, Novartis, F. De Benedetti Consultant with: Abbvie, Sobi, Novimmune, Novartis, Roche, Pfizer

### O05. Analysis of pyrin inflammasome activation defines surf patients from FMF and other recurrent fevers

#### S. Palmeri^1^, A. Bertoni^2^, R. Papa^2^, F. Penco^2^, A. Corcione^2^, R. Caorsi^2^, C. Matucci-Cerinic^1^, M. Bustaffa^2^, F. Schena^2^, P. Bocca^2^, S. Volpi^1,2^, A. Rubartelli^2^, M. Gattorno^2^, I. Prigione^2^

##### ^1^Department of Neuroscience, Rehabilitation, Ophthalmology, Genetics, Maternal and Child Health, University of Genova, ^2^Center for Autoinflammatory Diseases and Immunodeficiencies, IRCCS Istituto Giannina Gaslini, Genova, Italy

###### **Correspondence:** S. Palmeri

**Introduction:** The best known of recurrent fevers, familial Mediterranean fever (FMF), is genetically determined and its pathogenetic mechanism has already been extensively investigated, revealing a role of the pyrin inflammasome (1). However, cohorts of patients with undifferentiated, genetically negative forms of relapsing fever (SURF) are still poorly studied. These patients are genetically negative, and their clinical picture resemble FMF, including the good response to colchicine. However, the underlying inflammatory mechanisms of SURF are not yet known (2).

**Objectives:** To assess the *in vitro* activation of the pyrin inflammasome in a cohort of patients with SURF, compared with FMF and Periodic Fever, Aphthous Stomatitis, Pharyngitis, Adenitis (PFAPA) patients; to stratify the response between *in vivo* colchicine-treated and untreated individuals; to dissect the *in vitro* response to colchicine in the same cohort of patients.

**Methods:** Peripheral blood mononuclear cells (PBMC) of a subset of SURF (N=15, colchicine-treated and untreated), FMF(N=8), PFAPA (N=8) and HD (N=13) were tested for ASC speck formation and IL-1beta production in response to different stimuli. Clostridium difficile toxin A (TcdA) and PKN1/2inhibitor (UCN-01) were used to trigger pyrin inflammasome. Colchicine was added in vitro to evaluate the pyrin inflammasome inhibition. We used a flow cytometric method to measure the percentage of ASC speck formation in monocytes and ELISA assay to quantify the secretion of IL-1beta on cell supernatants. We performed non-parametric Mann-Whitney test (MW) for the analysis of differences between groups.

**Results:** in SURF patients, we did not observe spontaneous activation of the inflammasome in the absence of clinical flare. FMF, SURF, PFAPA and HD displayed no differences in TcdA-induced activation of pyrin inflammasome (% of ASC speck formation). However, *in vivo* colchicine-untreated SURF patients showed a reduced response to TcdA, with a normalization of values in SURF patients after treatment (% of ASC speck formation, MW, *p<0,05*; IL1-beta (pg/mL) MW, *p<0,05*). In contrast with FMF, SURF, PFAPA patients and HD showed the following features: i) UCN-01 mediated-pyrin dephosphorylation was not sufficient to trigger Pyrin inflammasome activation; ii) the *in vitro* colchicine administration caused a huge inhibition of TcdA-induced pyrin inflammasome activation.

**Conclusion:** we applied functional *in vitro* tests for pyrin inflammasome activation analysis to a clinically homogeneous group of subjects with SURF. These preliminary data show that SURF patients differ from FMF, PFAPA, and HD. SURF subjects showed a different response to TcdA, with a normalization after colchicine therapy, suggesting an involvement of pyrin inflammasome in the physiopathology of SURF.

References:

1) Magnotti F. et al. “Fast diagnostic test for familial Mediterranean fever based on a kinase inhibitor.” Annals of the rheumatic diseases vol. 80,1 (2021): 128-132.

2) Papa R. et al. “Syndrome of Undifferentiated Recurrent Fever (SURF): An Emerging Group of Autoinflammatory Recurrent Fevers.” Journal of clinical medicine vol. 10,9 1963.

**Trial registration identifying number:**

**Patient Consent:** Yes, I received consent

**Disclosure of Interest**: None declared

### O06. MIS-C phenotypes vary between SARS-COV-2 variants

#### G. Mastrangelo^1^, E. Go^1,2,3^, P. Tsoukas^1,2^, H. Lu^1,2^, A. Hoi Hin Cheng^1,2^, R. S. Yeung^1,2,4^ on behalf of on behalf of SickKids MIS-C Working Group

##### ^1^Division of Rheumatology, Department of Paediatrics, The Hospital for Sick Children, University of Toronto, ^2^Cell Biology Program, The Hospital for Sick Children Research Institute, Toronto, Canada, ^3^Division of Pediatric Rheumatology, Riley Hospital for Children, Indianapolis, United States, ^4^Department of Immunology and Institute of Medical Science, University of Toronto, Toronto, Canada

###### **Correspondence:** G. Mastrangelo

**Introduction:** Multisystem Inflammatory Syndrome in Children (MIS-C) is a serious complication associated with COVID-19, presenting as a hyperinflammatory disorder characterized by fever and multiorgan dysfunction. Whether the MIS-C phenotype varies accordingly to the SARS-CoV-2 variants is still unclear.

**Objectives:** We aim to compare MIS-C clinical features, treatments, and outcomes across the various waves of COVID-19, dividing the patient population into three cohorts according to the Alpha/Beta/Gamma, Delta, and Omicron MIS-C waves. Our secondary objective is to evaluate if the clinical phenotype (shock, Kawasaki Disease (KD), fever with hyperinflammation) varies across the three cohorts.

**Methods:** We performed a prospective cohort study of 252 patients with MIS-C, at a tertiary care pediatric center from March 2020 to March 2022. Clinical and laboratory features, complications, treatments, and outcomes were evaluated. The association with SARS-CoV-2 variants and MIS-C cohorts was assumed based on local epidemiology and sequencing data, representing the predominant strain across the three-time periods. The starting date of each MIS-C wave for study purposes was set at two weeks after the first case of COVID-19 from that respective variant in the community, as the actual time lag for developing MIS-C is within 2-6 weeks after the acute infection. Descriptive statistics were performed to assess differences between the three MIS-C cohorts and clinical phenotypes.

**Results:** Of the 252 patients (150 with Alpha/Beta/Gamma variants, 59 with Delta, 43 with Omicron), the median age was 5.2 years, 58.7% were male, and 50% had SARS-CoV-2 exposure. The three cohorts showed a significant difference in MIS-C phenotype distribution (p=0.003). Fever and hyperinflammation was the predominant phenotype (20%) in the Alpha/Beta/Gamma cohort; shock represented the majority (39%) in the Delta cohort; and the KD phenotype was prevalent (67%) in the Omicron cohort. Cardiac and gastrointestinal involvements were the most common features in all the cohorts, whereas, neurological involvement was the least prevalent. The Omicron cohort had more mucocutaneous involvement and renal abnormalities compared to the others. The main difference between the various waves was reflected in measures of complications and outcome with the MIS-C cohort associated with the Delta variant capturing the most severe phenotype with a higher incidence of shock (39%), MAS (22%), and PICU admission (34%). The proportion of children developing coronary artery lesions was similar in all groups. Among all the three MIS-C cohorts, the majority of patients received either IVIG alone or together with upfront steroids. Pulsed high-dose steroids and anticoagulation therapy were more commonly used among children in the Delta MIS-C cohort, findings in keeping with the prevalence of the MIS-C shock phenotype in this group.

**Conclusion:** The MIS-C phenotype varies accordingly to the SARS-CoV-2 variants, and patients with the Delta variant had a more severe phenotype with a greater proportion of complications. This is the first study that compares MIS-C phenotypes stratified by virus variant waves, including the Omicron wave. These findings provide new insights into disease phenotype and SARS-CoV-2 variants and may have important implications for diagnosis and management.

**Patient Consent:** Not applicable (there are no patient data)

**Disclosure of Interest**: None declared

### O07. Development of the oncoreum score for differential diagnosis between childhood cancer with arthropathy and juvenile idiopathic arthritis

#### A. Civino^1^, F. Bovis^2^, M. Ponzano^2^, S. Magni-Manzoni^3^, S. Sorrentino^4^, L. Vinti^5^, M. Romano^6^, N. Santoro^7^, G. Filocamo^8^, C. Gorio^9^, F. Santarelli^10^, M. Cattalini^11^, F. Diomeda^12^, G. Alighieri^13^, E. Prete^14^, G. Stabile^15^, R. Rondelli^16^, V. Conter^17^, A. Pession^18^, A. Ravelli^19,20^ on behalf of ONCOREUM Study group

##### ^1^Reumatologia e Immunologia Pediatrica, Ospedale “Vito Fazzi”, Lecce, ^2^Dipartimento di Scienze della Salute, Sezione di Biostatistica, Università di Genova , Genova, ^3^Reumatologia Pediatrica, Ospedale Pediatrico Bambino Gesù IRCCS, Roma, ^4^Dipartimento di Oncoematologia Pediatrica, IRCCS Istituto ‘Giannina Gaslini’, Genova, ^5^Dipartimento di Oncoematologia Pediatrica, Ospedale Pediatrico Bambino Gesù IRCCS, Roma, ^6^Divisione di Reumatologia, ASST G. Pini-CT, Milano, ^7^Oncoematologia Pediatrica, Azienda Ospedaliero Universitaria Consorziale Policlinico di Bari, Bari, ^8^Fondazione IRCCS Cà Granda Ospedale Maggiore Policlinico, Milano, ^9^Unità di Oncoematologia Pediatrica e TMO, “Spedali Civili”, Brescia, ^10^Dipartimento di Pediatria, Ospedale Pediatrico “Regina Margherita”, Torino, ^11^Clinica Pediatrica, Università degli Studi di Brescia e ASST Spedali Civili, Brescia, ^12^Clinica Pediatrica, Università degli Studi di Bari Aldo Moro, Bari, ^13^UO Neonatologia-UTIN, Azienda Ospedaliera “A. Perrino”, Brindisi, ^14^Dipartimento di Ematologia, Azienda Ospedaliera “Cardinale G. Panico”, Tricase (Lecce), ^15^Consorzio Interuniversitario Cineca, Casalecchio di Reno, ^16^Oncoematologia Pediatrica, IRCCS Azienda Ospedaliero-Universitaria di Bologna, Bologna, ^17^Oncoematologia Pediatrica, Fondazione MBBM, Università Milano Bicocca, Monza, ^18^Clinica Pediatrica, IRCCS Azienda Ospedaliero-Universitaria di Bologna, Bologna, ^19^Direzione Scientifica, IRCCS Istituto Giannina Gaslini, ^20^Dipartimento di Neuroscienze, Riabilitazione, Oftalmologia, Genetica e Scienze Materno-Infantili (DiNOGMI), Università degli Studi di Genova, Genova, Italy

###### **Correspondence:** A. Civino

**Introduction:** Pediatric cancer with musculoskeletal symptoms at onset can mimic rheumatic diseases, particularly juvenile idiopathic arthritis (JIA). This could lead to inappropriate steroid treatment or immunosuppressive therapy with a delay in the diagnosis.

**Objectives:** To develop and validate a weighted score, named ONCOREUM Score, that aids physicians in timely differentiation of cancer with arthropathy from JIA.

**Methods:** Data were extracted from the ONCOREUM Study, a multicenter, prospective, cross-sectional study aimed to compare patients with cancer and arthropathy with those affected by JIA. Patients were younger than 16 years and were newly diagnosed with cancer at 25 Italian pediatric hemato-oncology centers or with JIA at 22 Italian pediatric rheumatology centers. Details concerning study design have been described in detail previously.^1^

A multiple imputation by chained equations approach with 10 imputations was first performed. Then, 80% of patients were assigned to the developmental data set and 20% to the validation data set.

Three statistical approaches were applied to develop the ONCOREUM Score, two based on multivariable analysis of different sets of variables (Models 1 and 2) and one based on a Bayesian Model Averaging method (Model 3). The β coefficients estimated in the models were used to assign points to the scores. Discriminating performance was evaluated by calculating sensitivity, specificity and AUC in the validation sample.

**Results:** The study dataset included 772 patients, 95 with cancer and arthropathy and 677 with JIA. The highest AUC in the validation data set was yielded by Model 1, which was selected to constitute the ONCOREUM Score. Sensitivity, specificity, and AUC of the cutoff in the validation sample were 81.3%, 96.4%, and 0.89, respectively. The formula used to calculate the score includes 9 variables weighted according to the estimated coefficients, as follows (clinical features are included in the model only if present): ONCOREUM score = - 4.1 + 8.4 x limb bone pain + 4.7 x weight loss + 5.1 x thrombocytopenia - 5.2 x morning stiffness - 3.7 x joint swelling - 5.0 x small hand joint involvement + 4.1 x monoarticular involvement + 2.4 x hip involvement + 1.2 x male sex.

The score ranges from - 18 to 21.8 and the optimal cutoff obtained through ROC analysis was – 2, with patients being classified at higher risk of having cancer and arthropathy if score is ≥ - 2, and at higher risk of having JIA if score is < - 2.

**Conclusion:** The ONCOREUM score is composed of 9 clinical features that can easily be recorded at initial encounter with the patient. It is a powerful tool that may facilitate early differentiation of malignancies with arthropathy from JIA and timely referral of the child to the appropriate pediatric specialist.

1. Civino A, Alighieri G, Prete E et al. Musculoskeletal manifestations of childhood cancer and differential diagnosis with juvenile idiopathic arthritis (ONCOREUM): a multicentre, cross-sectional study. Lancet Rheumatol. 2021; 3 (7): e507-e516.

**Trial registration identifying number:** ONCOREUM study group members

Italian Association of Paediatric Haematology and Oncology (AIEOP) Centres

Massimo Eraldo Abate (Bologna), Annalisa Arlotta (Parma), Catia Atzeni (Cagliari), Tamara Belotti (Bologna) Patrizia Bertolini (Parma), Barbara Bigucci (Rimini), Andrea Biondi (Monza), Roberta Burnelli (Ferrara), Maurizio Caniglia (Perugia), Ilaria Capolsini (Perugia), Anna Maria Caroleo (Genova), Maria Giusepina Cefalo (Roma), Monica Cellini (Modena), Simone Cesaro (Verona), Adele Civino (Tricase, Lecce), Elisa Coassin (Aviano), Antonella Colombini (Monza), Valentino Conter (Monza), Carmela De Fusco (Napoli), Raffaela De Santis (San Giovanni Rotondo), Andrea Di Cataldo (Catania), Elena Fabbri (Rimini), Franca Fagioli (Torino), Monica Ficara (Modena), Ilaria Fontanili (Parma), Alberto Garaventa (Genova), Chiara Gorio (Brescia), Saverio Ladogana (San Giovanni Rotondo), Franco Locatelli (Roma), Chiara Mainardi (Padova), Maurizio Mascarin (Aviano), Chiara Messina (Padova), Concetta Micalizzi (Genova), Rossella Mura (Cagliari), Daniela Onofrillo (Pescara), Roberta Pericoli (Rimini), Andrea Pession (Bologna), Cristina Pizzato (Treviso), Fulvio Porta (Brescia), Carmelo Rizzari (Monza), Andrea Roncadori (Bologna), Roberto Rondelli (Bologna), Elisa Rossi (Bologna), Giovanna Russo (Catania), Nicola Santoro (Bari), Giulia Stabile (Bologna), Elisa Tirtei (Torino), Assunta Tornesello (Lecce), Federico Verzegnassi (Trieste), Luciana Vinti (Roma)

Italian Paediatric Rheumatology Study Group (IPRSG) Centres

Giovanni Alighieri (Tricase), Martina Amatruda (Roma), Patrizia Barone (Catania), Luciana Breda (Chieti), Michela Cappella (Reggio Emilia), Marco Cattalini (Brescia), Adele Civino (Tricase, Lecce), Rita Consolini (Pisa), Elisabetta Cortis (Orvieto), Sergio Davì (Genova), Alessandro De Fanti (Reggio Emilia), Fabrizio De Benedetti (Roma), Giovanni Filocamo (Milano), Romina Gallizzi (Messina), Maria Francesca Gicchino (Napoli), Francesco La Torre (Brindisi), Bianca Lattanzi (Ancona), Loredana Lepore (Trieste), Maria Cristina Maggio (Palermo), Silvia Magni-Manzoni (Roma), Andrea Magnolato (Trieste), Manuela Marsili (Chieti), Silvana Martino (Torino), Angela Miniaci (Bologna), Alma Nunzia Olivieri (Napoli), Serena Pastore (Trieste), Maria Antonietta Pelagatti (Monza), Rosa Anna Podda (Cagliari), Eleonora Prete (Tricase), Angelo Ravelli (Genova), Francesca Ricci (Brescia), Donato Rigante (Roma), Micol Romano (Milano), Francesca Santarelli (Torino), Francesca Soscia (Orvieto)

**Patient Consent:** Not applicable (there are no patient data)

**Disclosure of Interest**: None declared

### O08. NLRP3 splicing variants as a regulatory mechanism of inflammasome priming in auto-inflammation

#### R. Erkens^1,2^, M. van Haaren^2^, R. Sanchez Rodriguez^2^, J. Calis^2^, S. Vastert^1,2^, J. van Loosdregt^2^

##### ^1^Pediatric Rheumatology, Wilhelmina Children’s Hospital, ^2^Center for Translational Immunology, UMC Utrecht, Utrecht, Netherlands

###### **Correspondence:** R. Erkens

**Introduction:**

The activation of the NLRP3 Inflammasome is both transcriptionally and post-translationally regulated. Recently, it has been demonstrated that various isoforms of NLRP3 are expressed in human macrophages and that isoforms that lack certain exons due to alternative splicing are not able to form a functional inflammasome by being unable to bind to NEK7, an essential mediator of NLRP3 activation. Inflammasome activation seem to play an important role in systemic Juvenile Idiopathic Arthritis (SJIA) pathophysiology, characterized by high levels of IL-18 and IL-1 pathway activation. Here, we assessed whether NLRP3 splicing changes upon neutrophil and monocyte activation and investigated the role of alternative splicing in the auto-inflammatory setting of SJIA.

**Objectives:**

To explore the effects of alternative RNA splicing products of NLRP3 in neutrophils and monocytes in healthy individuals and SJIA patients on the activation of the NLRP3 inflammasome.

**Methods:**

We assessed NLRP3 isoform expression using third generation mRNA Nanopore sequencing, qPCR and western blot. The compared conditions included *ex-vivo* and 3 hours 100ng/ml LPS stimulated neutrophils and monocytes isolated from peripheral blood of healthy donors and SJIA patients. Inflammasome activation was assessed by measuring IL-1β production using ELISA after LPS priming and subsequent nigericin activation.

**Results:**

We identified various NLRP3 RNA isoforms in both neutrophils and monocytes using Nanopore sequencing and qPCR. Full length NLRP3 and NLRP3 δexon5 isoforms were found to be the most dominant. In monocytes and neutrophils, LPS mediated priming induces NLRP3 expression and a shift to full length NLRP3 expression due to a decrease in NLRP3 δexon5. *Ex-vivo* neutrophils and monocytes of biological and steroid naïve patients with active SJIA express relatively more full length NLRP3 compared to samples from healthy donors. After 3 days of treatment with anakinra, exon 5 inclusion decreased in both neutrophils and monocytes of the SJIA patients, but remains increased in neutrophils compared to healthy controls. These data suggest that neutrophils of active SJIA patients are more primed for NLRP3 inflammasome activation.

**Conclusion:**

Priming of neutrophils and monocytes induces profound changes in NLRP3 isoform expression. These changes after LPS priming might increase the potential of the cell to form an active inflammasome upon a second stimulation. Similarly, we demonstrated that neutrophils and monocytes from patients with active SJIA express more full-length NLRP3, able to form inflammasomes, compared to healthy controls. This observation could contribute to the increased inflammasome activation found in SJIA. Taken together, these results suggest an important level of regulation of inflammasome activation by alternative splicing. Further research is necessary to elucidate the pathophysiology of inflammasome activation in auto-inflammation in general as this opens a window for new therapeutic options.

**Patient Consent:** Yes, I received consent

**Disclosure of Interest**: R. Erkens: None declared, M. van Haaren: None declared, R. Sanchez Rodriguez: None declared, J. Calis: None declared, S. Vastert Consultant with: Sobi and Novartis, J. van Loosdregt: None declared

### O09. Candidate gene sequencing in systemic juvenile idiopathic arthritis implicates rare variation in hereditary periodic fever and familial hemophagocytic lymphohistiocytosis genes

#### M. Correia Marques, D. Rubin, E. Shuldiner, E. Schmitz, E. Baskin, A. Patt, M. Ombrello on behalf of Incharge Consortium

##### Translational Genetics and Genomics Section, NIAMS, National Institutes of Health, Bethesda, United States

###### **Correspondence:** M. Correia Marques

**Introduction:** Systemic juvenile idiopathic arthritis (sJIA) is a genetically complex inflammatory condition. It can be marked by severe systemic inflammation that resembles the hereditary periodic fever syndromes (HPF). Sometimes that inflammation leads to macrophage activation syndrome (MAS), a secondary form of hemophagocytic lymphohistiocytosis (HLH). The HPFs and familial forms of HLH (fHLH) are caused by rare genetic mutations, and it has been hypothesized that genetic variants of HPF or fHLH genes are involved in the pathophysiology of sJIA. Several studies have examined this question, but none have had the statistical power to provide an unequivocal answer.

**Objectives:** We used targeted sequencing of HPF and fHLH genes in a large patient cohort to determine whether rare variation in these genes contributes to the risk of developing sJIA.

**Methods:** Targeted sequencing of HPF (*MEFV, MVK, NLRP12, NLRP3, NOD2, PSTPIP1, TNFRSF1A*) and fHLH (*LYST, PRF1, RAB27A, STX11, STXBP2, UNC13D*) genes was performed in sJIA cases and control subjects from the International Childhood Arthritis Genetics Consortium cohort using Illumina Nextera Custom Capture Assays and Illumina sequencers. Sequence reads were aligned to human genome assembly hg19 with the Burrows-Wheeler Aligner. Data processing and quality control was performed using the Genome Analysis Toolkit. Variants were filtered to retain rare (minor allele frequency < 0.01), protein-altering variation that mapped to Ensembl canonical transcripts. The distribution of rare variants among sJIA cases was compared to the distributions among INCHARGE healthy controls or simulated data from the 33,370 Non-Finnish European (NFE) reference subjects from the Exome Aggregation Consortium (ExAC) using rare variant association testing (RVT). RVT was performed in R using the data-adaptive sum test and the sequence kernel association test (SKAT). Significance was evaluated at a threshold of p < 0.05 after 100,000 permutations.

**Results:** Targeted sequencing was performed in 525 sJIA cases and 366 control subjects. Six sJIA cases were discovered to have a genetic diagnosis of either an HPF (n=4) or fHLH (n=2) and were excluded. We also found that 39 cases and 1 control subject were ancestrally dissimilar from the larger cohort by principal component analysis, leading to their exclusion. Sequencing of the remining 480 sJIA cases identified 78 rare fHLH gene variants and 62 rare HPF gene variants. RVT comparing the distribution of rare variants of sJIA cases with that of the ExAC NFE population revealed significant rare variant associations between sJIA and *LYST, STXBP2, UNC13D* and *MEFV.* We also discovered recurrent mutations of *STXBP2, UNC13D,* and *MEFV* among the sJIA cohort that were not observed in the ExAC population*.* A sub-analysis of 123 sJIA cases with known MAS status (32 with MAS, 91 without MAS) identified a significant association between rare variation of *UNC13D* and the development of MAS in sJIA (SKAT p=0.024).

**Conclusion:** The observations of this study connect HPF and fHLH genes to the pathophysiology of sJIA. The distributions of rare genetic variants of *LYST, STXBP2, UNC13D* and *MEFV* were statistically different in children with sJIA than in the general population. We also identified novel, recurrent mutations in 3 of these 4 genes in children with sJIA. These results highlight the potential value of studying rare genetic variation in sJIA. To expand this approach, we have established a collaborative infrastructure to perform an exome sequencing-based study of rare variation across all protein-coding genes in sJIA.

**Patient Consent:** Not applicable (there are no patient data)

**Disclosure of Interest**: None declared

## Oral Communications Session 2 - CTD, Vasculitis Oral Communications

### O10. CD14+ Monocyte-Derived oxidised mitochondrial DNA amplifies the inflammatory interferon type 1 signature in Juvenile dermatomyositis

#### M. Wilkinson^1,2,3^, D. Moulding^4^, T. C. R. McDonnell^5^, M. Orford^4^, C. Wincup^1,5^, J. Y. J. Ting^3^, G. W. Otto^2,6^, R. Restuadi^2,3^, D. Kelberman^2,7^, S. Castellano^2,6^, S. Eaton^4^, C. Deakin^1,2,3^, E. C. Rosser^1,5^, L. R. Wedderburn^1,2,3^ on behalf of UK Juvenile Dermatomyositis Research Group and the Centre for Adolescent Rheumatology Versus Arthritis

##### ^1^Centre for Adolescent Rheumatology Versus Arthritis at UCL UCLH and GOSH, UCL, ^2^NIHR GOSH BRC, ^3^Infection, Immunity and Inflammation Research and Teaching Department, ^4^Developmental Biology and Cancer Research & Teaching Department;, UCL GOS Institute of Child Health, ^5^Centre for Rheumatology Research, UCL, ^6^Genetics and Genomic Medicine Research & Teaching Department, UCL GOS Institute of Child Health, ^7^Genetics and Genomic Medicine Research & Teaching Department, UCL, London, United Kingdom

###### **Correspondence:** M. Wilkinson

**Introduction:** JDM is a rare childhood autoimmune myositis that presents with proximal muscle weakness and associated skin changes. There is an unmet need to develop new targeted treatments.

**Objectives:** This study aimed to identify dysregulated biological processes up-stream of the known, pathological interferon (IFN) type 1 signature in JDM by RNA-sequencing and develop functional assays to confirm these pathways.

**Methods:** Peripheral blood samples were obtained from JDM patients [pre-n=10 on-n=11 treatment] and age/sex-matched child healthy controls [n=8]. CD4^+^, CD8^+^, CD14^+^ and CD19^+^ cells were sorted by flowcytometry from PBMC, and RNA was extracted and RNA-sequenced. Mitochondrial morphology and mitochondrial superoxide was assessed in CD14+ monocytes by fluorescence microscopy using MitoTracker and MitoSox dyes quantified by volume, JDM [n=6] and control [n=9]. Oxidised mitochondrial DNA (oxmtDNA) from CD14+ monocytes was measured by western dot-blot, JDM [n=10] and control [n=11]. Healthy control PBMC samples [n=6] were cultured with IFN-α or oxmtDNA (+ LL37) with or without TLR-9 antagonist or *n*-acetyl cysteine (NAC). Post-culture, IFN type 1 gene expression was measured by qPCR.

**Results:** RNA-seq confirmed a strong IFN type 1 signature pre-treatment, and genes involved in mitochondrial function were abnormally expressed in both pre- and on-treatment CD14+ monocytes vs. controls, suggesting that mitochondrial dysfunction is not corrected by current treatment strategies. Investigating abnormal mitochondrial biology in JDM CD14+ monocytes by microscopy, we identified that the mitochondria were significantly more fragmented in JDM vs. control (p=0.0044) and evidence of megamitochondria. Analysis of the RNA-seq data showed that the oxidative phosphorylation pathway the gene expression of superoxide dismutase (*SOD1*) were downregulated in JDM pre- and on-treatment vs. controls. We showed an increase in mitochondrial superoxide in CD14+ monocytes JDM vs. control (p=0.0005, p=0.017). By western dot-blot there was an increase in oxmtDNA in CD14+ monocytes JDM vs. control (p=0.0178). *In vitro*, oxmtDNA and IFN-α induced a comparative up-regulation IFN1 genes compared to unstimulated control (MX1 (p<0.0001, p<0.0001); RSAD2 (p=0.1508, p=0.001)). Both TLR-9 antagonist and NAC were able to down-regulate IFN type 1 genes after 24hr of oxmtDNA stimulation, suggesting that both could translate to therapeutic targets (TLR-9 (MX1, p=0.0001; RSAD2, p=0.0374); NAC (MX1, p<0.0001; RSAD2, p=0.00014)).

**Conclusion:** Here, we show that dysregulated mitochondrial biology in JDM CD14+ monocytes is associated with increased oxidised mitochondria DNA (oxmtDNA) and which amplifies the interferon type 1 signature which characterises JDM, which represents a therapeutically targetable mechanism in JDM and potentially other IFN type 1-driven autoimmune diseases.

**Patient Consent:** Yes, I received consent

**Disclosure of Interest**: M. Wilkinson Grant / Research Support with: CureJM, NIHR GOSH BRC fellowship grants, non-renumerated collaboration with Novartis, D. Moulding: None declared, T. McDonnell: None declared, M. Orford: None declared, C. Wincup: None declared, J. Ting: None declared, G. Otto: None declared, R. Restuadi: None declared, D. Kelberman: None declared, S. Castellano: None declared, S. Eaton: None declared, C. Deakin: None declared, E. Rosser: None declared, L. Wedderburn Grant / Research Support with: NIHR (Senior Investigator award and the NIHR GOSH BRC), Myositis UK, Versus Arthritis and Great Ormond Street Childrens Charity. This research was supported by the NIHR Biomedical Research Centre at Great Ormond Street Hospital for Children NHS Foundation Trust (GOSH). The JDCBS is also supported by grants from Cure JM, Versus Arthritis (21593, 21552), Great Ormond Street Childrens Charity (W1143), Myositis UK, the NIHR GOSH BRC and the Remission Charity. Non-renumerated collaboration with Novartis

### O11. A novel in vitro model to study precision targeting of IFN-mediated responses

#### S. R. Veldkamp^1^, M. Reugebrink^1^, B. Lerkvaleekul^1,2^, E. P. A. H. Hoppenreijs^3^, S. Kamphuis^4^, W. Armbrust^5^, J. M. van den Berg^6^, P. C. E. Hissink Muller^7^, J. Wienke^1^, M. H. A. Jansen^2^, A. van Royen-Kerkhof^2^, F. van Wijk^1^

##### ^1^Center for Translational Immunology, University Medical Center Utrecht, ^2^Pediatric Rheumatology and Immunology, Wilhelmina Children’s Hospital, University Medical Center Utrecht, Utrecht, ^3^Department of Paediatrics, Paediatric Rheumatology, Amalia Children’s Hospital, Radboud University Medical Centre Nijmegen, Nijmegen, ^4^Paediatric Rheumatology, Sophia Children’s Hospital, Erasmus University Medical Centre, Rotterdam, ^5^Department of Pediatric Rheumatology and Immunology, Beatrix Children’s Hospital, University Medical Center Groningen, Groningen, ^6^Department of Pediatric Immunology, Rheumatology and Infectious Diseases, Emma Children’s Hospital, Amsterdam University Medical Centers, Amsterdam, ^7^Department of Paediatric Rheumatology, Leiden University Medical Centre, Leiden, Netherlands

###### **Correspondence:** S. R. Veldkamp

**Introduction:** A dysregulated interferon (IFN) pathway is an important hallmark of JDM pathogenesis. Siglec-1, a macrophage/monocyte-restricted surface marker, was recently identified as a novel type I IFN-related activation marker in patients with JDM that correlated with clinical disease activity and could predict treatment response.

**Objectives:** Our aim was to develop an *in vitro* model to study the inhibition of IFN-mediated responses with Siglec-1 as a read-out.

**Methods:** PBMCs from healthy donors were stimulated *in vitro* for 0, 6 and 18 h with various TLR agonists (TLR-3, -4, -7, -9) or cytokines (IFNα/β/γ, TNFα, IL-1β). Pre-incubation for 1 h with various concentrations of JAK-inhibitors ruxolitinib (JAK1/2), baricitinib (JAK1/2), tofacitinib (JAK1/3), or filgotinib (JAK1), a TYK2-inhibitor (deucravacitinib) or an anti-IFNα/βR2 blocking antibody was performed to study their inhibitory effect on Siglec-1 induction. Furthermore, PBMCs were treated for 18 h with plasma of JDM patients or healthy donors (20% v/v), with or without anti-IFNα/βR2 blocking antibody. In all experiments, Siglec-1 expression was measured in CD14^+^ PBMCs by flow cytometry.

**Results:** Siglec-1 expression was induced after 18 h by IFNα, IFNβ, and agonists of TLR-7 (imiquimod), TLR-9 (Class A CPG ODNs), and, most potently, TLR-3 (poly I:C). Induction by all these stimuli could be completely inhibited by IFNα/β receptor blockade. Pre-incubation with 1 μM ruxolitinib, baricitinib, or tofacitinib, or 10 μM filgotinib fully prevented induction of Siglec-1 by poly I:C. For full inhibition of IFNα-induced Siglec-1 expression, however, 10 μM of all JAK inhibitors was required. In contrast, deucravacitinib, a TYK2-inhibitor, was able to inhibit Siglec-1 induction by either poly I:C or IFNα at a concentration of 0.1 μM. Treating healthy donor PBMCs with JDM patient plasma induced Siglec-1 expression, while healthy donor plasma did not. The effect of the patient plasma could be inhibited by IFNα/β receptor blockade.

**Conclusion:** Siglec-1 expression on monocytes was previously shown to be increased *ex vivo* in JDM patients and correlate with disease activity. These *in vitro* studies show that Siglec-1 expression can be induced in PBMCs by type I IFNs as well as certain TLR ligands, the latter of which mediate through type I IFN secretion. Siglec-1 induction can be inhibited to different extents by JAK/TYK inhibitors. Compared to the JAK-inhibitors, the TYK2-inhibitor deucravacitinib showed the strongest effect on inhibiting the IFN-mediated monocyte activation. This *in vitro* model has the potential to provide a biological basis for precision treatment of IFN-related systemic inflammatory diseases such as JDM and to study underlying mechanisms of derailed IFN responses.

**Patient Consent:** Not applicable (there are no patient data)

**Disclosure of Interest**: None declared

### O12. Predicting immunoglobulin resistance in Kawasaki disease in multi-ethnic populations in europe: a multicenter cohort study

#### N. Ouldali^1,2^, R. M. Dellepiane^3^, S. Torreggiani^3^, L. Mauri^4^, G. Beaujour^2^, C. Beyler^5^, M. Cucchetti^6^, C. Dumaine^2^, A. La Vecchia^7^, I. Melki^2^, R. Stracquadaino^7^, C. Vinit^2^, R. Cimaz^8^, U. Meinzer^1,2^

##### ^1^Université Paris Cité, Center of Reserach on Inflammation, Inserm 1149, ^2^National Reference Centre for Rare Paediatric Inflammatory Rheumatisms and Systemic Autoimmune diseases RAISE, Hôpital Robert Debré, APHP, Paris, Paris, France, ^3^Pediatric Intermediate Care Unit, ^4^Department of Paediatric Cardiology, Fondazione IRCCS Ca’ Granda Ospedale Maggiore Policlinico, Milan, Italy, ^5^Department of Paediatric Cardiology, Hôpital Robert Debré, APHP, Paris, Paris, France, ^6^Department of Clinical Sciences and Community Health, Research Center for Adult and Pediatric Rheumatic Diseases, University of Milan, ^7^University of Milan, ^8^Department of Clinical Sciences and Community Health, Research Center for Adult and Pediatric Rheumatic Diseases, University of Milan, Milan, Italy

###### **Correspondence:** U. Meinzer

**Introduction:** Early identification of high-risk patients is essential to stratify treatment algorithms of Kawasaki disease (KD) and to appropriately select patients at risk for complicated disease who would benefit from intensified first-line treatment. Several scores have been developed and validated in Asian populations but have shown low sensitivity in predicting intravenous immunoglobulin (IVIG) resistance in non-Asian populations.

**Objectives:** We sought methods to predict the need for secondary treatment after initial IVIG in non-Asian populations.

**Methods:** We conducted a retrospective, multicenter study including consecutive patients with KD admitted to two tertiary pediatric hospitals in France and Italy from 2005 to 2019. We evaluated the performance of the Kawanet-score and compared it with the performances of initial echocardiography findings, and of a newly proposed score combining the Kawanet-score and initial echocardiography findings. For each score, we assessed the AUC, sensitivity and specificity for predicting the need for second-line treatment.

**Results:** We included 363 children with KD, 186 from France and 177 from Italy, of whom 57 (16%) required second-line therapy after the first IVIG dose. The Kawanet score, coronary artery dilation or aneurysm with maximal Z-score ≥2.0 at baseline, and abnormal initial echocardiography had a sensitivity of 43%, 55% and 65% and a specificity of 73%, 78%, 73%, respectively, for predicting the need for second-line treatment. The Kawanet-score was significantly improved by combining it with initial echocardiography findings. The best predictive performance (Sensitivity 76%, Specificity 54%) was obtained by combining the Kawanet-score with abnormal initial echocardiography, defined by the presence of either coronary artery maximal Z-score ≥2.0, pericarditis, myocarditis and/or ventricular dysfunction. This score predicted the need for second-line treatment in European, African/Afro-Caribbean and Asian ethnicity with a sensitivity of 80%, 65% and 100%, respectively, and a specificity of 56%, 51% and 61%, respectively.

**Conclusion:** Our study proposes a score that we named the Kawanet-echo score, which allows early identification of children with KD who require a second-line treatment in multi-ethnic populations in Europe.

**Patient Consent:** Yes, I received consent

**Disclosure of Interest**: None declared

### O13. TIF1-GAMMA and NXP2 autoantibodies in children with JDM are underrepresented when assessed by immunoblot compared to immunoprecipitation

#### H. D. Nguyen^1^, C. Papadopoulou^2^, D. Cancemi^1^, L. R. Wedderburn^1,2,3^, S. L. Tansley^4,5^ on behalf of on behalf of the UK Juvenile Dermatomyositis Cohort and Biomarker Study

##### ^1^UCL Great Ormond Street Institute of Child Health, ^2^NIHR Biomedical Research Centre at Great Ormond Street Hospital for Children, ^3^Centre for Adolescent Rheumatology Versus Arthritis at UCL, UCLH and GOSH, London, ^4^Royal United Hospitals, ^5^The University of Bath, Bath, United Kingdom

###### **Correspondence:** H. D. Nguyen

**Introduction:**

Juvenile Dermatomyositis (JDM) is a rare chronic autoimmune disease that causes proximal muscle weakness and skin rash in children and adolescents. Myositis specific and associated autoantibodies (MSA and MAA) are important prognostic biomarkers for JDM, yet the screening process of MSA and MAA is not standardised across healthcare centres, raising concerns about reliability or inter assay validity for this important prognostic tool. Although immunoprecipitation is considered the reference standard method to detect relevant autoantibodies, most autoantibody-testing laboratories use blotting-based immunoassays, for reasons of practicality and cost. A recent study suggested that immunoblot can be limited at detecting certain clinically important MSA subtypes ^1^.

**Objectives:**

To compare relevant autoantibody frequencies detected by immunoprecipitation (IP) with autoantibody frequencies detected in immunoblots, and to determine the different levels of sensitivity of these techniques in detecting different myositis relevant autoantibody subtypes, in the UK Juvenile Dermatomyositis Cohort and Biomarker Study (JDCBS).

**Methods:**

A total of 472 JDM patients recruited to JDCBS were included. MSA/MAA status of each patient was determined by radio-labelled protein immunoprecipitation at the University of Bath (383 patients) or by immunoblot assays provided via the patients’ centre of care (89 patients).

**Results:**

The female distribution was 70.9% in immunoprecipitation cohort and 72.7% in immunoblot cohort. Regarding ethnicity, most patients in both cohorts identified as White: 77.1% of immunoprecipitation cohort and 65.9% of immunoblot cohort. Immunoprecipitation and immunoblot methods detected a myositis relevant autoantibody in 225 of 383 (58%) and 47 of 89 (53%) of samples respectively. The frequency of autoantibodies detected however, varied between the two cohorts. Anti-TIF1γ was identified in 70 patients (18.1%) in the immunoprecipitation cohort, followed by anti-NXP2 in 61 (15.8%), and anti-MDA5 in 23 (5.9%) patients. In contrast, in the immunoblot cohort, anti-MDA5 was the most prevalent autoantibody found in 15 (15.5%) patients, followed by anti-TIF1γ in 11 (11.3%), and anti-NXP2 in 9 (9.3%) of patients.

Excluding anti-Ro52, which is not detected by immunoprecipitation, the identification of more than one autoantibody in a single patient was rare in both groups. Detection of more than one autoantibody was more prevalent in the immunoblot cohort (2.2%) versus immunoprecipitation (0.5%). Anti-Ro52 was present in 8 (9%) patients in the immunoblot cohort, and most commonly occurred in conjunction with anti-MDA5 (7 patients (7.9%)).

**Conclusion:**

The differing prevalence of autoantibodies in our cohort when analysed by immunoprecipitation and immunoblot raises concerns regarding the sensitivity and specificity of immunoblot to detect key autoantibodies relevant to patients with JDM. Poor sensitivity for immunoblot to detect anti-TIF1γ, in keeping with our data, has previously been reported but this is the first report of a potential reduction in sensitivity for anti-NXP2. The titre of anti-MDA5 has been shown to reduce with treatment ^2^. The increase in prevalence of anti-MDA5 in the immunoblot cohort could relate to the analysis of earlier pre-treatment samples in this group, rather than false positive results. Further work is required to understand the reliability of immunoblot to detect myositis autoantibodies in JDM.

**References:**

1. Tansley SL, Li D, Betteridge ZE, McHugh NJ. Rheumatology (Oxford). 59(8), 2109-2114 (2020).

2. Sato S, Kuwana M, Fujita T, Suzuki Y. Mod Rheumatol. 23, 496–502 (2012).

**Patient Consent:** Yes, I received consent

**Disclosure of Interest**: H. Nguyen Grant / Research Support with: NIHR Biomedical Research Centre at GOSH, C. Papadopoulou Grant / Research Support with: NIHR Biomedical Research Centre at GOSH, D. Cancemi Grant / Research Support with: NIHR Biomedical Research Centre at GOSH, L. Wedderburn Shareholder with: non-remunerated collaboration with Novartis, Grant / Research Support with: NIHR Biomedical Research Centre at GOSH; The JDCBS is supported by Cure JM, GOSCC, Myositis Remission, and the NIHR, S. Tansley: None declared

### O14. Comparing the use of 1g/kg intravenous immunoglobulin (IVIG) dose against the standard dose of 2g/Kg IVIG in patients with juvenile dermatomyositis (JDM): a retrospective cohort study

#### R. Ebrahim^1^, M. Al-Obaidi^2^, C. Papadopoulou^2^

##### ^1^University College London, ^2^Great Ormond Street Hospital, London, United Kingdom

###### **Correspondence:** R. Ebrahim

**Introduction:** Juvenile Dermatomyositis (JDM) is a rare childhood inflammatory disease affecting skin and muscle usually treated with corticosteroids alongside adjunctive therapies including intravenous immunoglobulins (IVIG). While typically dosed at 2g/kg on the recommendation of the Childhood Arthritis and Rheumatology Research Alliance (CARRA), national shortages in 2019 have meant JDM patients now receive a dose of 1g/kg IVIG.

**Objectives:** This study aims to evaluate differences in remission odds, disease activity improvement and reduction of concomitant medication use in JDM patients receiving either dose.

**Methods:** Data was collected from 48 JDM patients receiving IVIG for at least 6 months seen at London’s Great Ormond Street Hospital (GOSH) and part of the UK Juvenile Dermatomyositis Cohort and Biomarker Study (JDCBS). The primary outcomes were odds of remission at 0, 6 and 12 months post-IVIG initiation while disease improvement and reduction of concomitant medication were secondary outcomes measured at the same timepoints. Remission was defined using the Paediatric Rheumatology International Trials Organisation (PRINTO) criteria by meeting at least three of the following: CK ≤150U/I, CMAS >50/52, MMT8 >78/80, PGVAS ≤0.2/10. Logistic regression analysis was used to demonstrate effect of dose on remission odds while Friedman’s ANOVA and McNemar’s test measured disease activity improvement and reduction of medication.

**Results:** Of the 48 patients, 41 were taking 2g/kg IVIG while only 7 were taking 1g/kg IVIG. There was no significant difference in remission odds after 6 (p=0.957) and 12 months (p=0.894) between IVIG doses after adjusting for confounders including age, sex, ethnicity, disease onset age (years) IVIG start age (years) time starting IVIG post diagnosis (weeks) Myositis Specific Antibodies (MSA) presence, MSA subtype, and treatment received. Improvements were noted at 6 and 12 months for both groups in skin, muscle and global disease activity, the most notable of which were significant improvements in the modified skin disease activity score in both the 2g/kg group (χ2=10.150, p=0.006) and 1g/kg group (χ2=7.176, p=0.028). There was a significant reduction in corticosteroid use after 12 months (p=0.035) but no significant difference between IVIG doses after 6 months (p=0.241) and 12 months (p=0.253).

**Conclusion:** This study is the first to describe the effects of different IVIG doses in a large cohort of JDM patients and found that there is no significant difference in treatment outcomes and effect between JDM patients taking 1g/kg IVIG and 2g/kg IVIG. The results of this study further confirmed findings in the wider literature surrounding IVIG treatment in JDM with many patients achieving remission and improvement in muscle, skin and systemic disease. Meanwhile, the overall result of this study has important financial and clinical implications to reduce treatment cost and patients’ hospitalisation and thus presents an attractive proposition to healthcare providers to change their dose from 2g/kg IVIG to 1g/kg IVIG.

**Patient Consent:** Yes, I received consent

**Disclosure of Interest**: None declared

### O15. Measuring IFNA2 levels by a single-molecule array in clinical practice of childhood-onset SLE patients does matter; results from a single center longitudinal study

#### M. J. Wahadat^1,2^, H. Qi^3^, C. van Helden-Meeuwsen^2^, E. Huijser^2^, L. van den Berg^1^, J. Göpfert^4^, M. Verkaaik^1^, M. Schreurs^2^, S. Kamphuis^1^, M. Versnel^2^

##### ^1^Department of Paediatric Rheumatology, Sophia Children’s hospital, Erasmus University Medical Center, ^2^Department of Immunology, ^3^Department of Biostatistics, Erasmus University Medical Center, Rotterdam, Netherlands, ^4^Department of Applied, Biomarkers and Immunoassays, NMI Natural and Medical Sciences Institute at the University of Tübingen, Reutlingen, Germany

###### **Correspondence:** M. J. Wahadat

**Introduction:** Type-I interferon (IFN-I) pathway activation plays a pivotal role in the pathogenesis of SLE and has been proposed as biomarker for disease activity. IFN-I pathway activation can be measured by determining the expression of IFN-I stimulated genes or a so-called IFN signature. Ultrasensitive single-molecule array (Simoa) technology enables measurement of IFN protein concentrations at subfemtomolar concentrations. Parallel use of these measuring methods in longitudinal cohorts of childhood-onset SLE (cSLE) patients in relation to disease activity could help in translating the most relevant technique for use in clinical practice.

**Objectives:** To determine the association of serum IFNa2 levels and whole blood IFN-I stimulated gene expression with disease activity and study their potential to mark specific disease activity states in a longitudinal cohort of cSLE patients.

**Methods:** Serum IFNa2 levels were measured in 338 samples from 48 cSLE patients and 67 healthy controls using an IFN-a2 Simoa assay (Quanterix) on an HD-X analyser. A 5 gene IFN-I signature was measured by RT-PCR in paired whole blood samples. Disease activity was assessed by the clinical SELENA-SLEDAI (cSLEDAI) and BILAG-2004. Low disease activity was defined by the Low Lupus Disease Activity State (LLDAS) and flares were characterized by the SELENA-SLEDAI flare index. Analysis was performed using linear mixed effect models.

**Results:** A clear positive correlation was present between serum IFNa2 levels and the IFN-I gene signature (r=0.78, p<0.0001). Serum IFNa2 levels and the IFN-I gene signature showed the same significant negative trend in the first three years after diagnosis. In this timeframe, mean baseline serum IFNa2 levels decreased with 55.1% (delta 172 fg/mL, p<0.001) to a mean value of 164 fg/mL, which was below the calculated threshold of 219.4 fg/mL. In the linear mixed model, serum IFNa2 levels were significantly associated with both the cSLEDAI and the BILAG-2004 (p<0.001 and p<0.01), while the IFN-I gene signature did not show this association (p=0.35 and p=0.23). Moreover, 69.7% of the time points in LLDAS had a serum IFNa2 level under the calculated threshold, while only 31.9% of the time points in LLDAS reached an IFN-I gene signature below the calculated threshold. Both techniques were equally capable of marking disease flares (79.2% above threshold vs 87.5% above threshold).

**Conclusion:** Serum IFNa2 levels measured by Simoa, but not the type-I IFN gene signature, are associated with disease activity scores and characterize disease activity states in cSLE patients. Hence, this technique has the potential to be implemented in clinical practice.

**Patient Consent:** Yes, I received consent

**Disclosure of Interest**: None declared

### O16. Panel sequencing links rare, possibly damaging genetic variants to a subset of patients with juvenile-onset SLE with distinct clinical phenotypes and outcomes

#### A. Charras^1^, S. Haldenby^2^, E. Smith^1,3^, C. Roberts^1^, M. Beresford^1,3^, C. Hedrich^1,3^ on behalf of On behalf of the UK JSLE Cohort Study

##### ^1^Department of Women’s and Children’s Health, Institute of Life Course and Medical Sciences, University of Liverpool, ^2^Institute of Infection, Veterinary & Ecological Sciences, University of Liverpool, Centre for Genomic Research, ^3^Alder Hey Children’s NHS Foundation Trust Hospital, Department of Paediatric Rheumatology, Liverpool, United Kingdom

###### **Correspondence:** A. Charras

**Introduction:** Juvenile-onset systemic lupus erythematosus (jSLE) affects 15-20% of lupus patients. Clinical heterogeneity across ethnicities, age groups and individual patients suggests a variable pathophysiology.

**Objectives:** The aim of this study was to identify damaging high-penetrance mutations in genes associated with SLE or SLE-like diseases in a large national cohort (UK JSLE Cohort Study and Repository) and to compare demographic, clinical and laboratory characteristics in sub-cohorts of patients with “genetic” SLE versus other SLE patients.

**Methods:** Based on a 2018 literature search targeting known Mendelian disease causes and risk alleles (using PubMed, OMIM and Ensembl), target enrichment and next generation sequencing was performed in 348 patients. Using *in silico* screening (SnpEFF) for ‘high impact’ variants with low minor allele frequencies (MAF <5%) followed by inheritance pattern analysis (using OMIM, ClinVar and SNPnexus), patients with predicted ‘high impact’ variants and autosomal dominant (AD) inheritance were included. Furthermore, patients with variants following autosomal recessive inheritance were included, if ≥1 additional “high impact” mutations were present on the second allele (compound heterozygote) or if ≥1 other SLE-associated gene in the same immunological pathway had a “high impact” mutation. Results were integrated with demographic, clinical and treatment-related datasets.

**Results:**

Variants predicted to be damaging were identified in approximately 3.5% of jSLE patients. Affected genes and associated pathways are summarised in the table.

Compared to the rest of the cohort, patients with damaging gene variants (“genetic” SLE”) were younger and more frequently of African/Caribbean ancestry. Patients with “genetic” SLE had less overall organ involvement and associated damage, but neuropsychiatric involvement developed over time. Less aggressive first-line treatment was chosen in patients with “genetic” SLE, but more second- and third-line agents were used. “Genetic” SLE is associated with anti-dsDNA antibody positivity at diagnosis, and reduced ANA, anti-LA and anti-Sm antibody positivity at the last visit.

**Conclusion:** “Genetic” jSLE associates with younger age at disease-onset, reduced persistent antibody positivity, less organ involvement, fewer disease flares and less damage, but the development of neuropsychiatric disease over time. Routine sequencing may allow patient stratification, risk assessment and targeted treatment, thereby increasing efficacy and reducing toxicity.

**Patient Consent:** Yes, I received consent

**Disclosure of Interest**: None declared

**Table 1 (abstract O16).** See text for description

Pathway	Gene	Disease associated
**IC clearance**	*C1S*	SLE-like disease, aHUS, primary angioedema
	*C3*	SLE-like disease
	*PEPD*	SLE-like disease
**Immune cell signalling**	*BANK1*	AITD, CLL, SSc, RA, SLE
	*PTPN22*	RA, T1D, CD, JIA, HT, SLE
	*TNFSF4*	SSc, SLE
**NFκB signalling**	*TNFAIP3*	SS, RA, SSc, SLE, A20 haploinsufficiency
**Nucleic acid sensing and processing**	*DNASE1, RNASEH2A, RNASEH2B, RNASEH2C, SAMHD1, TREX1*	AGS, ChLE
**TLR/IFN signalling**	*IRF7*	SLE

### O17. Genetical and phenotypical findings of childhood-onset systemic lupus erythematosus

#### P. Morán Álvarez^1^, C. Passarelli^2^, V. Messia^1^, M. Pardeo^1^, E. Marasco^1^, A. Insalaco^1^, F. De Benedetti^1^, C. Bracaglia^1^

##### ^1^Ospedale Pediatrico Bambino Gesù, Rome, Italy, ^2^Pediatric Rheumatology, Ospedale Pediatrico Bambino Gesù, Rome, Italy

###### **Correspondence:** P. Morán Álvarez

**Introduction:** Systemic lupus erythematosus (SLE) is a chronic systemic autoimmune disease which leads to inflammation and organ damage caused by immune complex deposition. Classically, childhood SLE (cSLE) has been considered as a polygenic autoimmune disease; however, a pediatric monogenic lupus-like phenotype is emerging due to the recent recognition of several related novel high-penetrance gene variants in the last years. This fact associated to the high degree of concordance among monozygotic twins, supports the importance of genetic background in the cSLE pathogenesis.

**Objectives:** To identify the presence of variants in gene related to monogenic lupus and their relationship with clinical manifestations in cSLE or lupus-like phenotype.

**Methods:** A descriptive, observational, cross-sectional study was carried out in children with a diagnosis of cSLE or with lupus-like. The genetic analysis (Sanger/Clinical Exome Sequencing) was performed from isolated DNA obtained from blood sample.

**Results:** Forty-two children were included in the study. The genetic analysis detected at least one variant in 11 (26.1%) children, 5 (45.4%) with cSLE (*ADAR, TNFAIP3, RNASEH2B, SHOC2, IFIH1)* and 6 (54.5%) with lupus-like phenotype (*TREX, DNASE1,RNASEH2B, C1S, TLR7, STAT5A, TNFRSF13B*). Of those who carry a genetic variant, the median age at disease onset was 11 years (range: 2-16) and 72.7% were female. Most of them were Caucasians (72.7%). Four (36.3%) and 3 (27.2%) out of 11 patients had a positive family history and/or a personal history for autoimmune diseases, respectively.

Regarding the clinical manifestations at onset, musculoskeletal was the most frequent (8 patients, 72.7%), followed by hematological (6 patients, 54.5%), cutaneous (6 patients, 54.5%), constitutional with fever (5 patients, 45.45%), neurological (4 patients, 36.3%), renal (3 patients, 27.2%), cardiac (3 patients, 27.2%) and pulmonary (2 patients, 18.1%) manifestations.

Related to immunological parameters, 10 (90.9%) were ANA positive, 5 (45.4%) anti-dsDNA, 4 (36.3%) ENA and 2 (18.1%) were antiphospholipid antibodies and lupus anticoagulant positive. Both C3 and C4 were low in 5 (45.4%) children and isolated C3 levels were low in 4 (36.3%) patients.

Among the variants, we found that only two patients who carry a *TREX* variant showed normal C3 and C4 levels; one of them presented with lupus pernio as reported in literature. The same *RNASEH2B (c.868G>A)* variant was identified in two siblings with similar phenotype. The patient who carried the *SHOC2* variant presented polyarthritis and serositis, while the patient with the *TNFRSF13B* variant onset with a glomerulonephritis. Those manifestations have already been described related to these gene variants.

**Conclusion:** Around 25% pediatric patients with cSLE or lupus-like phenotype in our cohort showed at least one variant in gene related to monogenic-lupus and some of them had a phenotype similar to those already described. The evidence of these variants may suggest the genetics potential contribution to the cSLE pathogenesis. Further studies in larger cohorts are necessary to confirm these data.

**Patient Consent:** Yes, I received consent

**Disclosure of Interest**: None declared

### O18. Patient-specific and disease-related determinants for cardiovascular disease (CVD) risk stratification in the apple (atherosclerosis prevention in paediatric lupus erythematosus) clinical trial cohort

#### J. Peng^1,2^, G. Robinson^1,2^, S. Ardoin^3^, L. Schanberg^4^, E. Jury^1^, C. Ciurtin^2^

##### ^1^Centre for Rheumatology Research, Division of Medicine, ^2^Centre for Adolescent Rheumatology Versus Arthritis, Division of Medicine, University College London, London, United Kingdom, ^3^Department of Medicine, Nationwide Children’s Hospital, Ohio State University, Columbus, ^4^Duke Clinical Research Institute, Duke University School of Medicine, Durham, United States

###### **Correspondence:** J. Peng

**Introduction:** The risk of developing CVD through atherosclerosis in juvenile-onset systemic lupus erythematosus (JSLE) patients is significantly increased.

**Objectives:** This study aimed to stratify and characterize JSLE patients at elevated CVD-risk using patient/disease-related factors and metabolomic data from patients recruited to the APPLE (Atherosclerosis Prevention in Paediatric Lupus Erythematosus) clinical trial, designed to assess atherosclerosis development.

**Methods:** Unsupervised hierarchical clustering was performed to stratify patients by arterial intima-media thickness (IMT) measurements at baseline (N=151) and carotid (c)IMT progression over 36 months (placebo arm only, N=60). Baseline metabolomic profiles (~250 serum metabolites) were compared between clusters using conventional statistics, univariate logistic regression, sparse Partial Least-Squares Discriminant Analysis (sPLS-DA) and random forest classifier. An independent cohort (UCL-JSLE cohort, N=89) with matching metabolomics, immunophenotyping and proteomics, was used to validate the discovered CVD risk-related signatures from the APPLE cohort.

**Results:** Baseline IMT stratification identified 3 clusters with high, intermediate, and low baseline IMT measurements and progression trajectories over 36 months, each having distinct racial/BMI/household education/income characteristics. Analysis of cIMT progression over 36 months identified 2 patient groups with high and low IMT progression. Unique metabolomic profiles differentiated high and low cIMT progression groups, with good discriminatory ability (0.81 AUC in ROC analysis) using the top 6 metabolites (Total cholesterol esters, Total cholesterol, Phospholipids in small LDL particles, Total cholesterol in small LDL particles, Free cholesterol in medium LDL particles and Total lipids in small LDL particles) selected from the analysis. cIMT progression over 36 months in the placebo group correlated positively with baseline disease activity (SLEDAI), damage score (SLICC), white blood cell count, serum complement C3, blood pressure (both systolic and diastolic) and BMI. Metabolomics signatures discovered from the APPLE cohort were applied to stratify JSLE patients in the validation cohort (UCL-JSLE), where 3 groups were identified with distinct metabolomics profiles indicating JSLE patients with high risk (N= 20), intermediate risk (N= 43) and low risk (N= 26) CVD-risk. Significant differences were observed in the frequency of classical monocytes (p=0.015) and nonclassical monocytes (p=0.005) when comparing high and low CVD risk group in the UCL-JSLE cohort.

**Conclusion:** Complex analysis of IMT patterns and progression in the APPLE trial cohort identified novel key determinants that could guide further research for CVD-risk stratification in JSLE.

**Disclosure of Interest**: None declared

## Oral Communications Session 3 - JIA

### O19. Outcomes of patients with juvenile idiopathic arthritis following biologic switching in the childhood arthritis and rheumatology research alliance registry

#### M. Mannion^1^, S. Amin^2^, S. Balevic^3^, C. Correll^4^, T. Beukelman^1^, for the CARRA Registry Investigators^2^

##### ^1^Pediatric Rheumatology, University of Alabama at Birmingham, Birmingham, ^2^Childhood Arthritis and Rheumatology Research Alliance, Washington, DC, ^3^Duke Clinical Research Institute, Duke University, Durham, NC, ^4^Division of Rheumatology, Department of Pediatrics, University of Minnesota, Minneapolis, MN, United States

###### **Correspondence:** M. Mannion

**Introduction:** Tumor necrosis factor inhibitors (TNFi) are the most commonly used first biologics to treat juvenile idiopathic arthritis (JIA), but it is unknown what subsequent biologic medications are most effective after failure of an initial TNFi.

**Objectives:** We compared the effectiveness of using a 2^nd^ TNFi versus a non-TNFi following failure of a 1^st^ TNFi in routine clinical practice.

**Methods:** We included individuals with a primary diagnosis of non-systemic polyarticular course JIA who received a TNFi as their 1^st^ biologic medication and started a 2^nd^ biologic (index date) within 90 days of stopping the 1^st^ TNFi on or after enrollment in the Childhood Arthritis and Rheumatology Research Alliance Registry. Included patients had a 6 month follow up visit (+/- 3 months) after the index date and prior to February 29, 2020. Exclusion criteria included active uveitis on the index date and no Registry visit within 4 weeks of the index date. The primary outcome was inactive disease (ID) and minimal disease activity (MiDA) based upon the clinical juvenile arthritis disease activity score (cJADAS; cJADAS ID<2.5, cJADAS MiDA<5) at the 6 month visit. We used fully conditional specification multiple imputation to account for missing data. Propensity scores (PS) for likelihood of being in the 2^nd^ TNFi group were calculated by logistic regression (LR) with covariates from index date including active and limited joint count, physician and parent global assessments, childhood health assessment questionnaire, pain, erythrocyte sedimentation rate, time from diagnosis to index date, time from diagnosis to start of 1^st^ TNFi, sex, JIA category, methotrexate (MTX) use, glucocorticoid use, cJADAS, 1^st^ TNFi, index date calendar year, uveitis history. We used LR to compare outcomes following switch to a 2^nd^ TNFi versus non-TNFi between index date and 6 month follow up unadjusted and adjusted for PS quintile.

**Results:** 216 individuals were included in the study, 84% receiving etanercept initially and most patients stopping for ineffectiveness (74%). 183 (85%) started a 2^nd^ TNFi and 33 (15%) started a non-TNFi. Adalimumab was the most common 2^nd^ biologic (71% overall, 84% of 2^nd^ TNFi) and tocilizumab was the most common non-TNFi 2^nd^ biologic (9% overall, 58% of non-TNFi). On the index date, 56% of patients had used MTX (55% 2^nd^ TNFi and 58% non-TNFi) and 12% of patients used glucocorticoids (11% 2^nd^ TNFi and 15% non-TNFi). There was no difference in meeting cJADAS ID or MiDA criteria by treatment group even after adjusting for PS quintile.

**Conclusion:** Among polyarticular course JIA patients in a large North American registry following failure of a 1^st^ TNFi, switch to a 2^nd^ TNFi was more common than switch to a non-TNFi. There were no differences between those starting a 2^nd^ TNFi or non-TNFi in achieving cJADAS inactive disease or MiDA after 6 months. More research is necessary to determine which patients would benefit from change in treatment mechanism.

**Patient Consent:** Not applicable (there are no patient data)

**Disclosure of Interest**: M. Mannion Grant / Research Support with: Rheumatology Research Foundation Norman B Gaylis, MD Clinical Investigator Award, S. Amin: None declared, S. Balevic Grant / Research Support with: National Institutes of Health, US Food and Drug Administration, Patient-Centered Outcomes Research Institute, Rheumatology Research Foundation Scientist Development Award, Childhood Arthritis and Rheumatology Research Alliance, Purdue Pharma, Consultant with: UCB, C. Correll: None declared, T. Beukelman Consultant with: Novartis, UCB, ,. for the CARRA Registry Investigators: None declared

**Table 1 (abstract O19).** Logistic regression estimates for meeting cJADAS ID or cJADAS MiDA at 6-months for patients with polyarticular course JIA following start of a 2^nd^ biologic, referent group are those who started a non-TNFi. Models are adjusted for PS quintiles

	Second TNFi (n=183) Odds ratios (95% confidence intervals)
cJADAS MiDA	
Unadjusted	1.22 (0.54, 2.74)
Adjusted	1.11 (0.47, 2.62)
cJADAS ID	
Unadjusted	1.23 (0.49, 3.04)
Adjusted	1.23 (0.47, 3.20)

### O20. Orofacial manifestations of juvenile idiopathic arthritis from diagnosis to adult care transition: a population-based, cohort study

#### M. Glerup^1^, A. Tagkli^2^, A. Küseler^2^, A. Estmann^3^, C. Verna^4^, A. E. Bilgrau^5^, S. E. Nørholt^6,7^, T. Herlin^8^, T. K. Pedersen^2,6^, P. Stoustrup^2^

##### ^1^MG and AT Contributed Equally to the Study and Shares the First Authorship. Department of Paediatrics and Adolescent Medicine, Aarhus University Hospital, ^2^Section of Orthodontics, Aarhus University , Aarhus, ^3^Department of Pediatrics, Odense University Hospital, Odense, Denmark, ^4^Department of Pediatric Oral Health and Orthodontics and UZB University Center for Dental Medicine Basel, University of Basel, Basel, Switzerland, ^5^Department of Mathematical Sciences, Aalborg University, Aalborg, ^6^Department of Oral and Maxillofacial Surgery , Aarhus University Hospital , ^7^Department of Dentistry and Oral Health, Aarhus University, ^8^Department of Paediatrics and Adolescent Medicine, Aarhus University Hospital, Aarhus, Denmark

###### **Correspondence:** M. Glerup

**Introduction:** Juvenile idiopathic arthritis (JIA) involvement of the temporomandibular joint (TMJ) may cause orofacial symptoms and dysfunction that can persist into adulthood. Contemporary, population-based estimates for the prevalence of JIA-related orofacial manifestations are unavailable. Such estimates are important for patient counseling, critical for clinical decision-making, and planning of the transition to adult rheumatology care. Furthermore, much attention has been devoted to the identification of risk factors of poor outcome in JIA but knowledge about risk factors exclusive of TMJ involvement is limited.

**Objectives:** 1) To estimate the cumulative incidences of orofacial conditions followed by temporomandibular joint (TMJ) arthritis in juvenile idiopathic arthritis (JIA) from diagnosis in childhood until transition to adult care. 2) To identify features in JIA associated with involvement of the temporomandibular joint (TMJ).

**Methods:** A population-based cohort analysis was conducted, involving longitudinal data on orofacial health from 2000 to 2018. Regardless of TMJ status, the patients were referred to the Regional Craniofacial Clinic of Western Denmark for routine orofacial examinations according to a standardized protocol. All patients received tax-funded health care. Data collection included information about disease-specific background information, TMJ involvement, JIA-induced dentofacial deformity, and orofacial symptoms and dysfunction. The association between JIA disease parameters and the diagnosis of TMJ involvement at transition to adult care (16-18 years of age) was assessed using multivariate regression statistics.

**Results:** 613 patients were followed with a mean clinical TMJ observation time of 4.0 years (range: 0-15.3 years; 0.25/0.75 percentiles: 0/6.7 years). From JIA onset to transition to adult care, the cumulative incidence of patients with JIA involvement of the TMJ was 30.0% during the 18 years of observation. Furthermore, 20.6% of the cohort had developed arthritis-induced dentofacial deformity. A substantial proportion of the cohort experienced several events of orofacial symptoms (23.5%) and dentofacial dysfunction (52%). In a multivariate analysis, the following baseline variables were significantly adversely associated with TMJ involvement: young age at diagnosis (<9 years), female gender, and ANA positivity. However, HLA-B27 positivity was associated with a lower risk of TMJ involvement. Of the orofacial symptoms and dysfunctions during the disease course TMJ pain during function, reduced translation, asymmetric mandibular mouth opening, and crepitation were significantly associated with TMJ involvement.

**Conclusion:** Orofacial signs and symptoms were frequent findings in children and adolescence with JIA. Involvement of the TMJ was seen in 30% of the cohort. 20.6% of the total cohort developed JIA-related dentofacial deformity before transition into adult care. TMJ involvement and dentofacial deformity were associated with JIA diagnosis at age younger than 9 years, ANA positivity, and several events of orofacial symptoms and TMJ dysfunction. This is the first population-based study in the biologic era to document these frequent orofacial complications in children with JIA.

**Patient Consent:** Yes, I received consent

**Disclosure of Interest**: None declared

### O21. Joint-specific responses to tofacitinib in JIA: a post HOC analysis of the open-label period of a phase 3 clinical trial

#### R. Micheroli^1^, D. J. Lovell^2^, A. Martini^3^, L. Stockert^4^, H. Jo^5^, K. Kwok^5^, A. Diehl^4^, T. Killeen^6^, C. Ospelt^1^, H. I. Brunner^2^, N. Ruperto^3^ on behalf of on behalf of PRINTO/PRCSG

##### ^1^University Hospital Zürich, Zürich, Switzerland, ^2^Cincinnati Children’s Hospital Medical Center, Cincinnati, OH, United States, ^3^IRCCS Istituto Giannina Gaslini, Genova, Italy, ^4^Pfizer Inc, Collegeville, PA, ^5^Pfizer Inc, New York, NY, United States, ^6^Pfizer Ltd, Tadworth, United Kingdom

###### **Correspondence:** T. Killeen

**Introduction:** Joint involvement in juvenile idiopathic arthritis (JIA) varies substantially.^1^ Tofacitinib is an oral Janus kinase inhibitor for the treatment of active polyarticular-course JIA and active juvenile psoriatic arthritis. The effect of tofacitinib on specific joints has not been well studied.

**Objectives:** Explore joint-specific responses to tofacitinib in the open-label phase of a clinical trial in patients with JIA.

**Methods:** This was a post hoc analysis of week (W)18 data from the open-label treatment phase of a Phase 3 clinical trial^2^ (N=225) in children with JIA aged 2–<18 years, where patients received oral tofacitinib (weight-based doses; ≤5 mg twice daily); 65% received concomitant methotrexate. Tenderness was defined as pain on motion and/or tenderness. Paired joint pathology scores (PJPS; range: 0 [neither side swollen and/or tender] to 4 [both sides swollen and tender] for each pair of joints) and PJPS % change from baseline (%Δ) were calculated; data were available for 34 joint pairs. Small joints of the hands and feet were grouped; for each grouping, PJPS was calculated as the mean of the included paired joints’ PJPS, and %Δ was calculated using the assigned % value (-100–100% by 25% increments) based on a combination of baseline and post-baseline scoring, which was calculated as mean of the individual paired joints’ PJPS.

**Results:** Baseline joint involvement and PJPS were greatest in the ankle, knee and wrist, followed by the metacarpophalangeals and proximal interphalangeals (Table). PJPS decreased in all joints by W4, continuing up to W18. Greatest %Δ PJPS responses were observed in the ankle, knee and wrist; with metatarsophalangeals, toes and distal interphalangeals responding least (Table).

**Conclusion:** Joint-specific responses to tofacitinib in JIA were strongest in the frequently involved ankle, knee and wrist, improving over time up to W18. This analysis was limited by its post hoc nature and lack of a placebo group.

**Trial registration identifying number:** ClinicalTrials.gov (NCT02592434)

**References**

[1] Eng SWM, et al. PLOS Med 2019; 16: e1002750

[2] Ruperto N, et al. Lancet 2021; 10315: 1984-96

**Acknowledgements:** Study sponsored by Pfizer Inc. Medical writing support was provided by Karen Thompson, PhD, CMC Connect, and funded by Pfizer Inc.

**Patient Consent:** Not applicable (there are no patient data)

**Disclosure of Interest**: R. Micheroli Speaker Bureau with: AbbVie, Eli Lilly, Gilead Sciences and Pfizer Inc, D. J. Lovell Grant / Research Support with: Bristol-Myers Squibb, Janssen, Novartis, Pfizer Inc, Roche and UBC, Consultant with: AstraZeneca, Boehringer Ingelheim, GSK, Novartis, Pfizer Inc, Roche, Takeda and UBC, and DSMB chairperson for NIH, A. Martini: None declared, L. Stockert Shareholder with: Pfizer Inc, Employee with: Pfizer Inc, H. Jo Shareholder with: Pfizer Inc, Employee with: Syneos Health, K. Kwok Shareholder with: Pfizer Inc, Employee with: Pfizer Inc, A. Diehl Shareholder with: Pfizer Inc, Employee with: Pfizer Inc, T. Killeen Shareholder with: Pfizer Inc, Employee with: Pfizer Ltd, C. Ospelt Grant / Research Support with: Novartis Foundation for Biomedical Research, H. I. Brunner Grant / Research Support with: Bristol-Myers Squibb, MedImmune, Novartis and Pfizer Inc, Consultant with: AbbVie, AstraZeneca/MedImmune, Bayer, Biocon, Boehringer Ingelheim, Bristol-Myers Squibb, Eli Lilly, Janssen, Novartis, Pfizer Inc, Roche and R Pharm, Employee with: Cincinnati Children’s Hospital Medical Center, Speaker Bureau with: GSK, Novartis and Roche, N. Ruperto Grant / Research Support with: Bristol-Myers Squibb, Eli Lilly, F. Hoffmann-La Roche, GSK, Janssen, Novartis, Pfizer Inc and Sobi, Consultant with: Ablynx, AstraZeneca/MedImmune, Biogen, Boehringer Ingelheim, Bristol-Myers Squibb, Eli Lilly, EMD Serono, F. Hoffmann-La Roche, GSK, Janssen, Merck Sharp & Dohme, Novartis, Pfizer Inc, R-Pharm, Sanofi, Servier, Sinergie and Sobi, Speaker Bureau with: Ablynx, AstraZeneca/MedImmune, Biogen, Boehringer Ingelheim, Bristol-Myers Squibb, Eli Lilly, EMD Serono, F. Hoffmann-La Roche, GSK, Janssen, Merck Sharp & Dohme, Novartis, Pfizer Inc, R-Pharm, Sanofi, Servier, Sinergie and Sobi

**Table 1 (abstract O21).** PJPS at baseline, joint grouping/joint grouping

Single joints					Joint groupings				
Joint	Baseline involvement frequency,^**a**^ n (%)	Baseline inflammation (mean PJPS)^**b**^	PJPS W4 (mean %Δ)	PJPS W18 (mean %Δ)	Joint grouping	Baseline involvement frequency,^**a,c**^ n (%)	Baseline inflammation (mean PJPS)^**b,c**^	PJPS W4 (mean %Δ)	PJPS W18 (mean %Δ)
Ankle	165 (73.3)	2.84	-38.60	-60.46	MCPs	48–98 (21.3–43.5)	2.08–2.65	-25.38	-35.75
Knee	176 (78.2)	2.76	-36.87	-55.82	PIPs	67–109 (29.8–48.4)	2.24–2.57	-21.68	-34.91
Wrist	156 (69.3)	2.76	-35.38	-55.04	Proximal foot^d^	43–48 (19.1–21.3)	2.10–2.26	-22.24	-34.69
Elbow	94 (41.8)	2.29	-34.11	-41.88	MTPs	27–44 (12.0–19.6)	2.00–2.29	-16.88	-30.00
Shoulder	50 (22.2)	1.92	-32.41	-41.67	Toes	10–27 (4.4–12.0)	1.86–2.30	-12.50	-25.71
TMJ	53 (23.6)	1.77	-17.05	-31.38	DIPs	16–31 (7.1–13.8)	1.87–2.10	-16.83	-22.92

PJPS range: 0–4

^a^Joint involvement defined as PJPS >0; ^b^calculated among patients with joint involvement at baseline; ^c^baseline values for joint groupings shown as ranges of included individual joints; ^d^proximal foot comprised ankle, subtalar and intertarsal joints

Only joints that could be assessed for swelling, pain and/or tenderness were analysed. Acromioclavicular and sternoclavicular joints not shown due to low n for baseline involvement

DIP, distal interphalangeal; MCP, metacarpophalangeal; MTP, metatarsophalangeal; n, number of patients; PIP, proximal interphalangeal; PJPS, paired joint pathology score; TMJ, temporomandibular joint; W, week

### O22. Distinguishing Kawasaki disease from other febrile conditions in a US cohort with the Kawasaki disease gene expression profiling (KIDS-GEP) classifier

#### D. Van Keulen^1^, C. Shimizu^2^, V. J. Wright^3^, D. Habgood-Coote^3^, D. Huigh^1^, A. H. Tremoulet^2^, R. Kuiper^1^, C. J. Hoggart^3,4^, J. Rodriguez-Manzano^3^, J. A. Herberg^3^, M. Kaforou^3^, D. Tempel^1^, M. Levin^3^, J. C. Burns^2^

##### ^1^SkylineDx, Rotterdam, Netherlands, ^2^Department of Pediatrics, Rady Children’s Hospital and University of California San Diego, La Jolla, California, United States, ^3^Department of Infectious Disease, Imperial College London, London, United Kingdom, ^4^Department of Genetics and Genomic Sciences, Icahn School of Medicine at Mount Sinai, New York City, New York, United States

###### **Correspondence:** D. Van Keulen

**Introduction:** Timely diagnosis of Kawasaki disease (KD) is challenging but may become more straightforward with the Kawasaki Disease Gene Expression Profiling (KiDs-GEP).

**Objectives:** To compare the KiDs-GEP classifier score between KD patients and febrile controls in a US cohort.

**Methods:** Biobanked whole blood RNA samples from 100 KD patients and 400 febrile controls who were diagnosed at Rady Children’s Hospital in San Diego between 2010 and 2019 were retrospectively collected. All patients were under 18 years of age and blood samples were obtained within the first 12 days of illness and prior to treatment with IVIG. RNA expression of the collected blood samples was measured with qRT-PCR and analyzed with the KiDs-GEP classifier.

**Results:** The KD patients had a median age of 3.05 years and 56.0% were male, which was comparable to the febrile control group (median age of 3.30 months and 56.3% male). KD patients had a significantly higher KiDs-GEP classifier score (mean 25.6 [IQR 24.0-27.1] than the febrile control patients (mean 21.0 [IQR 19.4-23.0], p<1x10^-10^). The lowest classifier score was observed for patients with viral infections (mean 20.4 [IQR 19.1-22.1]), making them easiest to distinguish from KD patients with the KiDs-GEP classifier.

**Conclusion:** The KiDs-GEP classifier score was significantly higher in KD patients than in febrile control patients. These results are consistent with our previous study and indicate that the KiDs-GEP classifier may be a useful tool to discriminate KD patients from febrile control patients.

**Patient Consent:** Yes, I received consent

**Disclosure of Interest**: D. Van Keulen Shareholder with: Option holder of SkylineDx, Employee with: Employee of SkylineDx, C. Shimizu: None declared, V. Wright: None declared, D. Habgood-Coote: None declared, D. Huigh Shareholder with: Option holder of SkylineDx, Employee with: Employee of SkylineDx, A. Tremoulet: None declared, R. Kuiper Shareholder with: Option holder of SkylineDx, Employee with: Employee of SkylineDx, C. Hoggart: None declared, J. Rodriguez-Manzano: None declared, J. Herberg: None declared, M. Kaforou: None declared, D. Tempel Shareholder with: Option holder of SkylineDx, Employee with: Employee of SkylineDx, M. Levin: None declared, J. Burns: None declared

### O23. Challenges for Ukrainian patients with rheumatic diseases: how to survive

#### Y. Vyzhga

##### National PIROGOV Memorial Medical University, Vinnytsya, Vinnitsya, Ukraine

###### **Correspondence:** Y. Vyzhga

**Introduction:** We used to know that patients with rheumatic diseases – is potentially vulnerable group not just due to the aggressive diseases course, but as well due to irregularity of the medical care, access to drugs in dependence to country of origin.

**Objectives:** The aim of my study was to analyse the impact of war on access and quality of medical care for children with rheumatic diseases in Ukraine.

**Methods:** The social networks started to be the separate platform for direct communication between patients and doctors, as well that’s a way to share correct and updated professional information with them. I supervise my medical blog for more than 1 year, and by now my audience is more than 13 thousand of subscribers, among them more than 1.5 thousand – patients with rheumatic diseases, both adults and children, their parents. At the end of March, in 1 month after the war started, I proposed my subscribers to complete questionnaire in goggle-form, that counted 15 questions highlighted current medical and social situation. Results of the questionaries were analysed with description statistical methods.

**Results:** Had been analysed 278 unique completed questionaries from the patients with rheumatic diseases and their parents. Among the patients mainly were children diagnosed with JIA (79.5 %), adults with RA (15.8 %), patients with SLE (1.4 %), dermatomyositis (0.7 %) and autoinflammatory syndromes (2.6 %). Geography of the patients before the war counted 15 regions from 24 available in Ukraine. 52,5 % of the responders were forced to leave their usual place of residence due to the war, among them – 42.7 % went abroad. Among the countries that mainly accepted our patients were: Poland, Germany, Czech Republic, Slovak, Italy, Belgium, Great Britain, France, Ireland, Portugal. 67.7 % of the patients that had gone abroad, recieved medical care from paediatric rheumatologists, 17.7 % were able to get medical support by general practitioners at the country of temporary residence. Among the patients that stayed in Ukraine, 74 % were able to receive consultation of general practitioner, without access to rheumatologist, 11 % were consulted by rheumatologist online. 47.8 % of the responders admitted worsening of the disease status or flare that occurred within the last month. Majority of the patients – 81 % were able to continue their DMARD therapy at least partially, 15 % of the patients temporarily interrupted their therapy due to the lack of the access to treatment. 33 % of the responders received immune-biologic therapy of rheumatic disease before the war, that represents general trend through the country. 65 % of the patients were able to continue it, among them 38% continued abroad. Among the patients settled abroad, 34 % received medical care thanks to PRINTO contacts, 22 % with patient’s organizations and societies support, 15 % - were assisted by volunteers and others – by general practitioners’ admissions. The separate aspect of the questionnaire counted psychological condition of the patients with its evaluation in 10 points score. According to parental evaluation, just 8 % of responders were not influenced by the war, all of them are infants. Absolut result was 7±2 points, that confirms absolute negative impact.

**Conclusion:** War makes unreversible influence on humanitarian life aspects and when we discuss problems of the patients with rheumatic disease, its difficult to predict final outcomes. But its possible to improve cooperation and networking between European centres, that will help our patient to win in every life causality.

I want to thank our association, every doctor and nurse, every member of our society, patients’ organization, European citizens, that didn’t allow us and our patient to feel themselves alone.

**Patient Consent:** Yes, I received consent

**Disclosure of Interest**: None declared

### O24. Disease activity in juvenile idiopathic arthritis from childhood to adulthood in the nordic JIA cohort

#### V. Rypdal^1,2^ on behalf of Nordic Study Group of Pediatric Rheumatology (NoSPeR), M. Glerup^3^, M. Rypdal^4^, E. D. Arnstad^5,6^, K. Aalto^7^, L. Berntson^8^, A. Fasth^9^, T. Herlin^10^, S. Nielsen^11^, M. Rygg^12,13^, E. Nordal^1,2^

##### ^1^Dep.of Pediatrics, University Hospital of North Norway, ^2^Dep.of Clinical Medicine, UIT the Arctic University of Norway, Tromsø, Norway, ^3^Dep.of Pediatrics, Aarhus University Hospital, Aarhus, Denmark, ^4^ Dep.of Mathematics and Statistics, UIT the Arctic University of Norway, Tromsø, ^5^Dep.of Pediatrics, Levanger Hospital, Nord-Trøndelag Hospital Trust, Levanger, ^6^Dep.of Clinical and Molecular Medicine, NTNU, Trondheim, Norway, ^7^Dep.of Pediatrics, New Children’s Hospital, Helsinki University Hospital, Helsinki, Finland, ^8^Dep.of Women’s and Children’s Health, Uppsala University , Uppsala, ^9^Dep.of Pediatrics, Institute of Clinical Sciences, Sahlgrenska Academy, University of Gothenburg, Gothenburg, Sweden, ^10^Dep. of Pediatrics, Aarhus University Hospital, Aarhus, ^11^Dep.of Pediatrics, Rigshospitalet Copenhagen University Hospital , Copenhagen, Denmark, ^12^Dep.of Clinical and Molecular Medicine, NTNU , ^13^Dep.of Pediatrics, St. Olavs Hospital, Trondheim, Norway

###### **Correspondence:** V. Rypdal

**Introduction:** Inactive disease (ID) is the primary goal of the treat-to-target (T2T) strategy in juvenile idiopathic arthritis (JIA). The Juvenile Arthritis Disease Activity Score (JADAS) has cut-off values for ID, low disease activity (LDA), moderate disease activity (MDA), and high disease activity (HDA) for oligo- and polyarticular disease courses. Few studies have assessed the disease activity trajectories in a population-based setting from childhood into adulthood.

**Objectives:** This study aimed to evaluate JADAS10 through a disease course of 18 years, and to investigate if there are identifiable clusters of JADAS10 development over time among the Nordic patients with JIA.

**Methods:** Patients with JIA (n=510) from centers participating in the Nordic JIA study were included at disease onset and followed prospectively. There were three major study visits: the baseline, the 8-year, and 18-year visit. The patients were divided into poly- and oligoarthritis groups if >4 joints or ≤4 joints were affected, respectively. Descriptive statistics and K-means clustering were used to present differences in disease activity states throughout the follow-up period. McNemar’s test was used to determine if there were statistically significant changes in disease activity states from baseline to the 18-year visit.

**Results:** At baseline, JADAS10 was available in 219 patients with a median score of 5.0 (IQR 2.0-11.0). At the 8-year visit, JADAS10 was available in 240 patients with a median score of 1.2 (IQR 0-4.2), and at the 18-year visit 268 patients had a JADAS10 score of median 2.2 (IQR 0.8-6.0). From baseline to the 8-year visit the proportion of ID increased (*p*<10^-4^, table), however, ID significantly decreased from the 8-year to the 18-year follow-up both for the oligo- and the polyarthritis groups (*p*=0.004 and 0.03, respectively). In addition, there was a significant increase of HDA in the oligoarthritis group (*p*=0.02). We identified three distinct JADAS10 patterns over time among 105 patients with complete JADAS10 information in the 3 major visits. Cluster 1: patients with high disease activity early in the course and improvement during the period. Cluster 2: patients with remaining low disease activity, and Cluster 3: patients with early low disease activity with worsening during the disease course.

**Conclusion:** We found increasing disease activity on long term follow-up after initial improvement the first years after onset of JIA. We identified a subset of children with increasing disease activity over time that may need tighter follow-up with a T2T-strategy. The impact of different JADAS cut-offs for oligo- and polyarticular JIA should further be investigated.

**Patient Consent:** Yes, I received consent

**Disclosure of Interest**: None declared

**Table 1 (abstract O24).** See text for description

JADAS10		N	Baseline visit	N	8-year visit	N	18-year visit
Olig JIA	ID	124	29(23.4)	120	70(58.4)	153	59(38.6)
	LDA		12(9.7)		12(10.0)		18(11.7)
	MDA		31(25.0)		16(13.3)		28(18.3)
	HDA		52(41.9)		22(18.3)		48(31.4)
Poly JIA	ID	95	6(6.3)	120	46(38.3)	115	33(28.7)
	LDA		15(15.8)		34(28.3)		39(33.9)
	MDA		28(29.5)		25(20.8)		30(26.1)
	HDA		46(48.4)		15(12.5)		13(11.3)

Values are n (%). JADAS10 cut-off: Inactive disease (ID); ≤1.0. Oligoarthritis group: low disease activity (LDA): 1.1-2.0. Moderate disease activity (MDA): 2.1-4.2. High disease activity (HDA): >4.2. Polyarthritis group: LDA: 1.1-3.8. MDA: 3.9-10.5. HDA: >10.5

### O25. Anti-TNF agents impair seroprotection in paediatric patients with juvenile idiopathic arthritis and inflammatory bowel disease vaccinated against meningococcal ACWY: the 24 months post vaccination follow-up data

#### M. Ohm^1^, J. W. van Straalen^2^, M. Zijlstra^3^, A. J. Sellies^2^, G. C. De Joode-Smink^2^, J. F. Swart^2^, S. J. Vastert^2^, J. M. van Montfrans^2^, M. Bartels^4^, A. van Royen-Kerkhof ^2^, J. G. Wildenbeest^5^, C. A. Lindemans^2,6^, V. M. Wolters^3^, R. A. Wennink^7^, J. H. de Boer^7^, M. W. Heijstek^8^, F. M. Verduyn Lunel^9^, G. Berbers^1^, N. M. Wulffraat^2^, M. H. Jansen^2^

##### ^1^Centre for Infectious Disease Control, National Institute for Public Health and the Environment, Bilthoven, ^2^Department of Pediatric Immunology and Rheumatology, ^3^Department of Pediatric Gastroenterology, ^4^Department of Pediatric Hematology, ^5^Department of Pediatric Infectiology and Immunology, Wilhelmina Children’s Hospital, University Medical Center Utrecht, ^6^Stem cell transplantation, Princess Máxima Center for Pediatric Oncology, ^7^Department of Ophthalmology, ^8^Department of Rheumatology and Immunology, ^9^Microbiology and Virology, University Medical Center Utrecht, Utrecht, Netherlands

###### **Correspondence:** M. H. Jansen

**Introduction:** In 2018, the meningococcal C vaccination was replaced by the meningococcal ACWY (MenACWY) vaccination in the Dutch Immunisation Programme. Therefore, we investigated the immunogenicity and safety of the MenACWY vaccine in paediatric patients with (auto)immune disease, here focussing on Juvenile Idiopathic Arthritis (JIA) and Inflammatory Bowel Disease (IBD). We now present the 24 month follow-up data and the results of the serum bactericidal antibody (SBA) assay.

**Objectives:** To assess immunogenicity and safety of the MenACWY vaccine in paediatric patients with JIA or IBD and study the effect of biological DMARDs on seroprotection.

**Methods:** In this prospective study, patients with immune disorders from the Wilhelmina Children’s Hospital Utrecht were sampled at baseline and 3, 12 and 24 months post vaccination. Serology was performed using the Fluorescent bead-based Multiplex ImmunoAssay (MIA) and the SBA assay. We assessed immunogenicity by determining geometric mean concentrations (GMCs) for polysaccharide-specific IgG concentrations. We assessed the proportion protected at 12 months post vaccination in a subset of the patients with the internationally-accepted correlate of protection (SBA titre ≥8). Safety was measured by questioning adverse events 3 months post vaccination. Alterations in disease activity were measured by the Clinical Juvenile Arthritis Disease Activity Score-27 (cJADAS-27) in JIA patients and the Paediatric Crohn’s Disease Activity Index (PCDAI) and Paediatric Ulcerative Colitis Activity Index (PUCAI) in IBD patients.

**Results:** 229 patients were included (33% IBD and 67% JIA). 40% were male and median age was 16 years (IQR: 14 – 17). At baseline, 72% of the patients were using DMARDs, of which 48% biologicals (39% anti-TNF agents). Except for MenC at 3 months, GMCs were significantly lower for each serogroup at all post vaccination time-points in patients who were treated with anti-TNF agents. The proportions protected (SBA titre ≥8) for MenA, C, W, Y 12 months post vaccination were 94%, 96%, 85% and 96%, respectively. This was significantly lower for anti-TNF users for serogroup W but not for A, C and Y. The MenACWY vaccine did not increase disease activity and no severe adverse events were reported.

**Conclusion:** The MenACWY vaccine is well tolerated in JIA and IBD patients but less immunogenic compared to healthy controls. GMC’s are significantly lower for anti-TNF users at 3, 12 and 24 months post vaccination and the proportion protected at 12 months post vaccination was significantly lower in anti-TNF users for serogroup W (but not A, C and Y). We therefore advice to measure antibodies in patients on anti-TNF 12 months post vaccination and to consider a booster vaccination accordingly.

**Patient Consent:** Yes, I received consent

**Disclosure of Interest**: None declared

### O26. Factors affecting the scoring of physician global assessment in JIA – survey results from PR-COIN and PRINTO

#### M. Tarkiainen^1,2^, M. Backström^3,4^, B. Gottlieb^5^, C. Trincianti^6^, T. Qiu^7^, E. Morgan^8^, D. Lovell^9^, N. Ruperto^10^, A. Consolaro^6,10^, P. Vähäsalo^11,12,13^

##### ^1^University of Helsinki, ^2^New Children’s hospital, Helsinki University Central Hospital, Helsinki, ^3^Department of Pediatrics, The Wellbeing Services County of Ostrobothnia, Vaasa, Finland, Vaasa, ^4^Pedego Research Unit, University of Oulu, Oulu, Finland, ^5^Cohen Children’s Medical Center, New York, United States, ^6^University of Genova, Genova, Italy, ^7^Department of Biostatistics and Epidemiology, USA., Cincinnati Children’s Hospital Medical Center, Cincinnati, ^8^Seattle Children’s Hospital, Seattle, ^9^Cincinnati Children’s Hospital, Cincinnati, United States, ^10^IRCCS Istituto G. Gaslini, Genova, Italy, ^11^PEDEGO Research Unit, University of Oulu, ^12^Department of Children and Adolescents, Oulu University Hospital, ^13^Medical Research Center Oulu, University of Oulu and Oulu University Hospital, Oulu, Finland

###### **Correspondence:** M. Tarkiainen

**Introduction:** Evaluating disease activity in juvenile idiopathic arthritis (JIA) is mainly done by rating the juvenile arthritis disease activity score in which the physician global assessment (PhGA) of disease activity has an important role. However, it is not known what affects physicians scoring the PhGA.

**Objectives:** To assess the heterogeneity of factors affecting PhGA scoring through a global web-based survey.

**Methods:** An electronic questionnaire regarding factors affecting PhGA was sent to all PRINTO and PR-COIN members. The responders were asked to rate from 0 to 100 the relevance of 17 factors possibly affecting PhGA scoring. These factors were chosen based on consensus of the study panel. Results were analyzed also in groups based on the responders’ level of experience in the field of pediatric rheumatology (<5 years, 5-10 years, or >10 years). Coefficient of variation (CV) was used to measure the heterogeneity in the 17 factors contributing to PhGA soring.

**Results:** 491 (431 from PRINTO and 60 from PR-COIN) responders completed the questionnaire. A large individual variation was observed in the impact of different factors on PhGA assessing JIA (Table). For systemic JIA, the smallest variations (mean/SD/CV) were seen in fever (89.2/16.4/18.4) and serositis (81.0/20.2/25.0), and the largest in the presence of erosions (48.2/33.8/70.1). To the question, “If a patient with oligoarticular non-systemic JIA and a polyarticular patient with non-systemic JIA had the same clinical picture would your VAS be different?” 244 (49,7%) physicians replied “NO” and 244 (49,7%) “YES”.

**Conclusion:** In a global perspective, scoring of PhGA is divergent. Especially the roles of the patient’s clinical history (i.e. oligoarticular or polyarticular disease), presence of extra-articular manifestations, or patient-reported outcome measures should be discussed. To obtain consistent patient assessment in clinical trials and routine practice, a consensus on guidelines for scoring the PhGA is required.

**Trial registration identifying number:** N/A

**Patient Consent:** Not applicable (there are no patient data)

**Disclosure of Interest**: None declared

**Table 1 (abstract O26).** Factors affecting physician´s global assessment in non-systemic JIA. N = 491

Factors	Mean (SD)	CV	Factors	Mean (SD)	CV
Number of swollen joints	86.8 (18.6)	21.0	Functional ability	53.2 (31.7)	59.6
Number of tender joints	74.4 (24.6)	33.0	Parent/patient pain VAS	48.9 (31.3)	64.2
Duration of morning stiffness	64.0(28.4)	44.7	Psoriatic skin manifestations	48.7 (31.9)	65.5
Presence of active uveitis	67.5 (31.1)	46.1	Parent/patient global VAS	46.9 (31.6)	67.5
Number of restricted joints	63.6 (30.0)	47.1	Presence of erosions	52.8 (35.6)	67.5
Dactylitis	63.9 (29.4)	47.2	Proportion of large vs small active joints	46.1 (31.2)	67.7
Laboratory findings	59.5 (29.3)	49.8	Presence of fever	46.5 (37.2)	80.0
Degree of inflammation in the active joints valued by US	59.9 (32.7)	54.6	Other factors	13.3 (29.2)	218.7
Degree of inflammation in the active joints valued by MRI	60.8 (33.4)	55.0			

### O27. Investigation the effects of Tai Chi in children with juvenil idiopathic arthritis: a randomized controlled trial

#### S. Y. Cetin^1^, E. Comak^2^, S. Akman^2^

##### ^1^Department of Physiotherapy and Rehabilitation, Faculty of Health Sciences, ^2^Department of Child Health and Diseases, Faculty of Medicine, Akdeniz University, Antalya, Turkey

###### **Correspondence:** S. Y. Cetin

**Introduction:** Juvenile idiopathic arthritis (JIA) is the most common childhood rheumatic disease characterized by pain, stiffness and joint swelling. JIA is associated with muscle weakness, impaired mobility, balance, and exercise capacity, resulting in decreased aerobic capacity and functional ability in children. It is stated that exercise programs such as strengthening, stretching and balance are beneficial in terms of increasing functionality and improving the quality of life in these children. Tai Chi is a mind-body Chinese exercise method that consists of a series of gentle movements characterized by balance. As a form of physical exercise, Tai Chi appears to improve cardiovascular fitness, muscle strength, balance, coordination and physical function in rheumatic diseases, and is also associated with improvements in psychological well-being. To the best ourknowledge, there is no study in literature that has investigated the effects of Tai Chi in children with JIA. Consequently, in this study, it was thought that due to the positive effect of Tai Chi in rheumatic diseases, it would also improve balance, functionality and increase the quality of life in children with JIA.

**Objectives:** The aim of this study was to investigate the effect of Tai Chi exercise program on balance, functional mobility, muscle strentgh of lower extremity, exercise capacity, fatigue and quality of life and compare with home exercises group in children with JIA.

**Methods:** 20 children with JIA (11 girls, 9 boys) with an average age of 11.85±3,16 years were included in the study. Children were divided into two groups by block randomization method. Group 1 received 1-hour Tai Chi exercise program twice a week for 10 weeks. Group 2 received a 1-hour strengthening, stretching and balance exercise program twice a week for 10 weeks as a home exercises. Functional Reach Test (FRT) was used to evaluate the balance, Timed Up and Go Test (TUG) for functional mobility, 30-sec Sit to Stand Test for muscle strentgh of lower extremity, 6-Min Walk Test (6MWT) for exercise capacity, PedsQL Multidimensional Fatigue Scale for fatigue, PedsQL Arthritis Modul for quality of life and Childhood Health Assessment Questionnarie (CHAQ) for health assessment. All evaluations were performed at baseline and at the end of the 10th week.

**Results:** When the pre-training values of the groups were compared, there was no statistically significant difference in parameters (p > 0.05). After training, there was a statistically significant difference within groups for the Tai Chi and the home exercise groups for all parameters (p: 0.00-0.04) except for CHAQ scores. When the groups compared after training, FRT, TUG, 6MWT scores and fatigue scores of PedsQL were found to be significantly in favor of the Tai Chi group (p:0.00- 0.04).

**Conclusion:** Tai Chi should be considered for inclusion in rehabilitation programs as a safe mind-body type of exercise to improve balance, functional mobility, muscle strentgh of lower extremity, exercise capacity, fatigue and quality of life in patients with JIA.

References

1-Kuntze G, Nesbitt C, Whittaker JL, Nettel-Aguirre A, Toomey C, et al. Exercise Therapy in Juvenile Idiopathic Arthritis: A Systematic Review and Meta-Analysis Arc Phys Med Rehabil 2018;99(1):178-193

2-Wang C. Role of Tai Chi in the treatment of rheumatologic diseases. Curr Rheumatol Rep. 2012;14(6):598-603

3-Cetin SY, Erel S, Bas Aslan U. The effect of Tai Chi on balance and functional mobility in children with congenital sensorineural hearing loss, Dis Rehabil, 2020; 42:12, 1736-1743

**Patient Consent:** Yes, I received consent

**Disclosure of Interest**: None declared

## Oral Communications Session 4 - Basic&Translational Science

### O28. Sex differences in regulatory T-cells may contribute to autoimmune disease susceptibility

#### G. A. Robinson^1^, J. Peng^1^, H. Peckham^1^, G. Butler^2^, I. Pineda-Torra^3^, C. Ciurtin^1^, E. C. Jury^4^

##### ^1^Centre for Adolescent Rheumatology Versus Arthritis, ^2^Department of Paediatric and Adolescent Endocrinology, ^3^Centre for Cardiometabolic and Vascular Science, ^4^Centre for Rheumatology, University College London, London, United Kingdom

###### **Correspondence:** G. A. Robinson

**Introduction:** Men and women have differential immune responses resulting in variation in response to infection, vaccination and autoimmune disease risk. An example is in juvenile-onset systemic lupus erythematosus (JSLE), which predominately affects young women with a common disease onset at puberty. Sexual dimorphisms have been described across both the innate and adaptive immune system which vary depending on age group and pubertal status. We hypothesised that sex-hormones could influence inflammation and autoimmune disease susceptibility following puberty.

**Objectives:** The aim of this study was to investigate the role of sex hormones and chromosomes in driving inflammatory profiles by sex and/or gender using unique cohorts of young individuals.

**Methods:** Flow cytometry was used to measure the frequency of 28 immune-cell subsets from young post-pubertal healthy and JSLE cis-men/women (n=17/22, mean age 18/17.5 and n=12/23, mean age 18/20) recruited from University College London Hospital, UK. Immune phenotype data was analysed by logistic regression and balanced random forest machine learning (BRF-ML). RNA-sequencing was used to assess the phenotype of regulatory T-cells (Tregs) isolated from matched healthy and JSLE individuals (n=5 per group), and from age matched transgender individuals under cross-sex-hormone treatment (trans-men/women, n=5/5, mean age 18.2/18.7, puberty blocked for 12-months followed by testosterone/oestradiol treatment). Differentially expressed gene (DEG) data was analysed by cluster, extended-network, pathway and open-target disease analysis. Suppression assays assessed the anti-inflammatory function of Tregs *in vitro*.

**Results:** BRF-ML identified increased circulating anti-inflammatory Tregs and decreased pro-inflammatory responder T-cells in healthy young cis-men compared to cis-women (p=0.0097 and p<0.0001, ranked highest in the BRF-ML-model). Tregs from healthy young cis-men were also more suppressive of activated T-cells *in vitro*. From RNA sequencing analysis, 82 Treg DEGs were identified between healthy young cis-men and cis-women, which could confidently cluster individuals by sex and significantly enriched the PI3K/AKT signalling gene ontology pathway. Importantly, 58.5% of these genes were not located on the X/Y chromosomes, suggesting a role of sex-hormones in Treg function. Despite having no influence on Treg frequency, cross-sex-hormone treatment altered many Treg transcriptomic pathways, including increased cytokine production and decreased immune activation in trans-men and trans-women, respectively, supporting a role of sex-hormones in Treg function. Many of the sex-hormone-induced Treg functional DEGs overlapped with the 82 DEGs identified between cis-men and cis-women and were significantly associated with PI3K/AKT signalling and with SLE by open-target disease analysis (p=0.02). Strikingly, sex differences in Tregs were lost or reversed in young JSLE patients, including Treg frequency, suppressive capacity and gene expression, suggesting that sex-hormone signalling could be dysregulated in autoimmune pathogenesis.

**Conclusion:** Sex-chromosomes and hormones may drive specific changes in circulating Treg frequency and function, respectively. Healthy young cis-men have a more anti-inflammatory Treg profile which could explain autoimmune susceptibilities by sex and inform sex-tailored therapeutic strategies.

**Patient Consent:** Yes, I received consent

**Disclosure of Interest**: None declared

### O29. FOXP3-specific deletion of CREB generates ST-2 positive regulatory T-cells with shifts towards type 2 immune responses

#### K. Ohl^1^, S. H. Subramanyam^1^, E. Verjans^1^, T. Clarner^2^, S. Böll^3^, I. G. Costa Filho^4^, L. Gan^5^, H. Huehn^6^, T. Bopp^7^, R. Beyaert^8^, B. Lambrecht^8^, M. Scheld^2^, T. Goodarzi^3^, N. Wagner^1^, M. Zenke^1^, K. Tenbrock^3^

##### ^1^RWTH AACHEN University, Aachen, Germany, ^2^Anatomy, ^3^Pediatrics, ^4^Computational Genomics, ^5^IZKF, RWTH AACHEN University, Aachen, ^6^HZI, Braunschweig, ^7^Institute of Immunology, Mainz, Germany, ^8^VIB, Ghent, Belgium

###### **Correspondence:** K. Tenbrock

**Introduction:** Regulatory T cells (T_regs_) are gatekeepers of immune homeostasis and characterized by expression of Foxp3, which maintains T_reg_ identity. The transcriptional activator CREB critically stabilizes Foxp3 expression *in vitro.*

**Objectives:** To analyze the effect of CREB on regulatory T cells, we generated mice with a deletion of CREB in all hematopietic cells (VAV-CRE) as well as a specific deletion in regulatory T cells (Foxp3-CRE)

**Methods:** Foxp+ CD4 T cells of mice were analyzed virgorously with flow cytometry, whole transcriptome analysis, ATACseq, TSDR methylation in vitro and in animal models of experimental colitis, ovalbumin induced asthma and TLR7 induced lupus in vivo

**Results:** Here we demonstrate that in mice with a Foxp3-specific knockout of CREB, T_regs_ show a reduced Foxp3 expression *in vivo*, but surprisingly enhanced expression of IL-13, IL-10, ST-2 and CREM. This rendered such T_regs_ highly suppressive *in vitro*. *In vivo*, Foxp3-specific knockout of CREB prevents colitis in a T cell mediated transfer colitis model in an IL-10 dependent way however aggravates disease activity in a murine lupus and asthma model. Mechanistically, in cooperation with CREM, CREB expression in T_regs_ alters chromatin accessibility to different loci like the ST-2 region and thereby influences T cell specific immune responses by regulation of IL-10.

**Conclusion:** Our data suggest that CREB expression in T_regs_ is important for the balance between Th1 and Th2 responses by regulating ST-2 and IL-10.

**Disclosure of Interest**: None declared

### O30. A gene signature for regulatory T cell fitness as a measure of disease activity in juvenile idiopathic arthritis

#### M. H. Attrill^1^, D. Shinko^1^, T. Martins Viveiros^1^, L. R. Wedderburn^2,3,4^, S. G. CHARMS^2,3,4^, S. G. JIAP^2,3,4^, A. M. Pesenacker^1^

##### ^1^Infection and Immunity, UCL Institute of Immunity and Transplantation, ^2^UCL Great Ormond Street Institute of Child Health, ^3^Centre for Adolescent Rheumatology Versus Arthritis at UCL UCLH and GOSH, ^4^NIHR Biomedical Research Centre at GOSH, London, United Kingdom

###### **Correspondence:** A. M. Pesenacker

**Introduction:** Juvenile Idiopathic Arthritis (JIA) is a disease of flare-ups, which do occur without warning or known trigger on or off treatment, yet there is no reliable biomarker to predict disease course. Better predictability of inflammation flare-ups could lead to more personalised disease management with preventative treatment if a flare is likely, or withdrawing medication safely when sustained remission is indicated.

**Objectives:** A regulatory T cell (Treg) defect has previously been suggested in JIA. Using our Treg gene signature as a measure of Treg fitness, we aim to identify the nature of Treg fitness changes in active JIA, which may be used as biomarker to predict disease trajectory and those at more at risk of flare-ups. Here, we focus on peripheral blood (PB) samples from clinically active and inactive JIA as well as synovial fluid (SF) from acutely inflamed joints.

**Methods:** SF mononuclear cells (SFMC, n=30), PBMCs from healthy adults (n=20) and healthy children (n=5) and from active (n= 30) and inactive (n= 17) JIA individuals were thawed, PB/SFMC lysates stored, and remaining cells sorted into CD4+CD25hiCD127low Tregs and CD4+CD25lowCD127hi T-conventional cells (Tconvs) and lysed in RLT buffer. Cell lysates were hybridised to a custom nanoString gene set (48 gene Treg signature plus) and analysed on nanoString nCounter Pro Analysis System. Normalisation by positive control and total sum normalisation, gene expression analysis and biomarker discovery pipeline, via machine learning, were performed using R software. Biomarker discovery analysis used combinations of feature select and classifier-building with logistic or elastic net regression and leave-one-out cross-validation (LOOCV) was used to obtain performance estimates, with the best algorithm chosen.

**Results:** As expected, the 48 gene signature successfully distinguished between Tregs and Tconvs, regardless of activation or disease state. PCA analysis clustered cells from SF and PB separately, with Tregs also distinguished from unfractionated PBMC and SFMC lysates. Genes usually associated with Treg functionality, such as FoxP3, CTLA-4 and TIGIT, were upregulated in SF Tregs. Interestingly, genes connected to the TGF-beta pathway, although not TGF-beta itself, were reduced in SF Tregs compared to blood Tregs. Crucially, using machine learning we could identify differences between blood Tregs from active and inactive JIA samples.

**Conclusion:** Our Treg gene signature serves as a measure of Treg fitness and has biomarker potential for immune-mediated conditions, such as JIA. We identified possible mechanisms behind loss of Treg fitness in the inflamed joint with changes in genes stabilising and responding to TGF-beta signalling. Moreover, our biomarker discovery pipeline could be used to establish an algorithm distinguishing active from inactive JIA blood Treg, indicating biomarker potential that could be ultimately used to track and possibly predict disease flares versus sustained remission.

**Patient Consent:** Yes, I received consent

**Disclosure of Interest**: None declared

### O31. Evidence-based occupational and physical therapy for children and adolescents with inflammatory pediatric rheumatic diseases suffering from fatigue

#### U. Nilsson, I. H. Bolstad, K. Krosby, B. W. Njølstad, S. Hansbø, H. Sanner, K. Risum

##### Oslo University Hospital, Oslo, Norway

###### **Correspondence:** U. Nilsson

**Introduction:** Fatigue is common in children and adolescents with inflammatory pediatric rheumatic diseases (PRD) and there is a need to develop an evidence-based guideline for physiotherapy (PT) and occupational therapy (OT) interventions to standardize and improve the quality of care offered to patients with PRD.

**Objectives:** To develop an evidenced-based guideline for PT and OT interventions, including identifying measurement tools to assess fatigue and interventions to treat fatigue.

**Methods:** The guideline is developed according to The Appraisal of Guidelines for Research and Evaluation (AGREE) Instrument, which includes scientific evidence, clinical experience and patient preferences. A systematic literature search of various medical databases was performed, and identified studies were critically evaluated. Occupational therapists and physiotherapists across Norway, working in both hospitals and primary care, contributed with clinical expertise. Patient experiences and preferences were obtained by interviewing representatives from the Norwegian Patient Association for Children with Rheumatic Diseases. Relevant representatives across Norway reviewed the guideline, and then final adjustments were performed.

**Results:** In total, 159 studies were identified and 17 included. In patients with Juvenile Idiopathic Arthritis, the Visual Analogue Scale is the most used unidimensional measurement tool to assess fatigue and the Pediatric Quality of Life Inventory Multi Fatigue Scale is the most used multidimensional questionnaire to assess fatigue. There is insufficient scientific evidence of the efficacy in interventions to reduce fatigue. However, a few studies showed that exercise significantly could reduce fatigue. The therapists often used a biopsychosocial approach in assessment and treatment of fatigue in PRD. They mainly used self-constructed questions to assess how fatigue affects body functions and structures, activities and participation. The therapists often used a time-schedule to get an overview of patient’s activity pattern and any need for adjustments. Since fatigue is a complex symptom, it is necessary to take several factors into consideration, such as sleep, pain and physical activity. The intervention is adapted by individual factors affecting the patient. The patient representatives highlighted a need for focus on fatigue and support by health care professionals to manage their fatigue. Patients experienced that management to reduce pain also could reduce fatigue.

The guideline is based on a biopsychosocial understanding of fatigue. It is divided into two parts; assessment and intervention. The assessment part contains patient interview, uni- and multidimensional measurements, activity registration, and assessment of different factors that can influence fatigue. The intervention part consists of patient education, understanding, awareness and regulation of activity level, enhancing self-efficacy, intervention of contributing factors to fatigue, and collaboration with other professionals involved in the care of the patients.

A brochure with patient information was developed as a part of the guideline. The guideline will be published and available at the Norwegian Electronic Health Library after final approval by Oslo University Hospital.

**Conclusion:** Scientific evidence is limited for assessment and interventions of fatigue in children and adolescents with PRD. Thus, the evidenced-based guideline is mainly based on clinical experience and patient preferences. Fatigue is a complex symptom and the guideline provides a framework for PT and OT in clinical care of children and adolescents with PRD suffering from fatigue. When applying the guideline, individual adjustments are necessary.

**Patient Consent:** Not applicable (there are no patient data)

**Disclosure of Interest**: None declared

### O32. Ultrasound versus MRI in the evaluation of ankle/midfoot joints in juvenile idiopathic arthritis

#### M. Mazzoni^1^, S. Lanni^2^, F. Magnaguagno^3^, M. Valle^3^, A. Pistorio^4^, C. Lavarello^5^, M. Gattorno^5^, A. Ravelli^4^, C. Malattia^5,6^

##### ^1^Struttura Complessa Pediatria, Ospedale di Lavagna, Lavagna, ^2^UOC Pediatria a Media Intensità di Cura Clinica de Marchi, Fondazione IRCCS Ca’ Granda Ospedale Maggiore Policlinico, Milano, ^3^Radiologia, ^4^Direzione Scientifica, ^5^Clinica Pediatrica e Reumatologia, IRCCS Istituto G. Gaslini, ^6^Dipartimento di Neuroscienze, Riabilitazione, Oftalmologia, Genetica e Scienze Materno-Infantili, Università degli Studi di Genova, Genova, Italy

###### **Correspondence:** M. Mazzoni

**Introduction:** Arthritis of the ankle/midfoot occurs commonly in all subtypes of JIA and might cause considerable functional impairment. Clinical assessment of this region is often challenging due to the multiplicity of joint recesses and surrounding tendons**.**

**Objectives:** To set-up an ultrasound (US) scoring system to assess synovial inflammation in ankle/midfoot joints and provide preliminary evidence of its validity by comparing US and MRI findings in JIA patients with this region involvement.

**Methods:** JIA patients who underwent contrast-enhanced (CE) MRI and US at the study Centre on the same day were included. The tibio-talar (TT) (anterior recess), the subtalar (ST) (lateral aspect) and the talonavicular (TN) (dorsal aspect) joints were examined by B-mode (synovial hypertrophy and/or joint effusion) and power Doppler (PD) mode. The anterior, lateral and posterior ankle tendons were assessed for tenosynovitis. Synovitis was graded using a specifically devised 0-3 semiquantitative joint-specific scoring system for B-mode and PD signal. MRIs were scored by using the Outcome Measure in Arthritis Clinical Trials Rheumatoid Arthritis MRI Scoring System. The agreement between MRI and US scores in each recess was evaluated by calculating Cohen’s kappa coefficient (k). The interpretation of the k coefficient values was as follows: 0–0.20 poor, 0.20–0.40 fair, 0.40–0.60 moderate, 0.60–0.80 good, and 0.80–1 very good.

**Results:** 67 patients (89.5% F; median age 12.7 y; median disease duration 7.3 y) were included. The agreement between MRI and US B-mode synovitis scores was moderate for the TT (Cohen’s kappa = 0.58; 95% CI = 0.35 - 0.81) and TN joints (Cohen’s kappa = 0.55; 95% CI = 0.31 - 0.79); it was good for the ST joint (Cohen’s kappa = 0.62; 95% CI = 0.39 – 0.85). The agreement between contrast-enhanced MRI and PD score was fair for the TT (k=0.24; 95% CI = 0.07 - 0.41) and ST joints (K= 0.38; 95% CI = 0.19 - 0.57) and moderate for TN joint (K= 0.5; 95% CI = 0.27 - 0.73). Concordance between MRI and MSUS for tenosynovitis was good (k = 0.79; 95% CI = 0.55 -1.00).

**Conclusion:** The joint specific US B-mode scoring system was accurate to assess and quantify synovitis in the ankle/midfoot joints and tendons. The discordances between US and MRI might be explained by MRI ability to visualize the entire joint, while the US can visualize only the superficial joint recesses. US scanning protocol and equipment settings might explain the lower sensitivity of PD mode compared to CE MRI in detecting hypervascularity in JIA synovial tissue that is an expression of active inflammation. Further studies are required to assess the reproducibility and sensitivity to change of the joint specific ankle/midfoot US score.

**Disclosure of Interest**: None declared

### O33. High-dimensional immunophenotyping reveals altered regulatory T cell fitness between inactive and active disease in juvenile idiopathic arthritis

#### M. H. Attrill^1^, D. Shinko^1^, L. R. Wedderburn ^2,3,4^, S. G. Charms^2,3,4^, S. G. JIAP^2,3,4^, A. M. Pesenacker^1^

##### ^1^Infection and Immunity, UCL Institute of Immunity and Transplantation, ^2^UCL Great Ormond Street Institute of Child Health, ^3^Centre for Adolescent Rheumatology Versus Arthritis at UCL UCLH and GOSH, ^4^NIHR Biomedical Research Centre at GOSH, London, United Kingdom

###### **Correspondence:** M. H. Attrill

**Introduction:** Juvenile Idiopathic Arthritis (JIA) is an autoimmune condition in children characterised by unpredictable, T-cell rich inflammatory flares of the joints. Despite increased number of CD4+FoxP3+ regulatory T cells (Tregs) within the synovial fluid (SF) of active joints, these fail to suppress autoimmunity.

**Objectives:** Here, we take a closer look at the CD4+FoxP3+ Treg populations in JIA. We proposed that through in-depth high-dimensional phenotyping we will be able to identify and distinguish unfit Treg sub-populations present in JIA SF and clinically active peripheral blood (PB) which may be disrupting the immunoregulatory balance leading to ongoing disease.

**Methods:** Using 5-laser full spectrum flow cytometry, we designed and verified a 33-parameter panel to assess differences in the cellular composition and Treg phenotype between JIA SF mononuclear cells (SFMCs, n=18), JIA PBMCs from clinically active (n=29) and inactive disease (n=17), and healthy adult control PBMCs (n=18). The panel was designed to identify monocyte, B, NK and dendritic cell subsets in addition to T cell phenotype. Incorporating markers and receptors associated with Treg functionality, activation, memory, co-stimulation, and co-inhibition, we were able to characterise Tregs in terms of overall ‘fitness’. In addition to traditional gating analysis via Flowjo, unbiased high dimensional analysis was achieved using the R package and computational tool Spectre, allowing raw data integration, clustering through FlowSOM, dimensionality reduction via UMAP and quantitative statistical analysis and visualisation.

**Results:** 12/18 clusters in overall cell composition, with most changes among T lymphocytes, and 8/10 Treg clusters had altered frequencies in SF. We found an emergence of more active subtypes in both CD4- and CD4+ T cells, with increased expression of GITR, HLA-DR, CD71 and CD69. This highly activated phenotype was also mimicked in SF Tregs, which additionally adopted a classically “suppressive” phenotype, with high CTLA-4 and PD-1 levels compared to PB. However, despite a shift to a more active state, SF Tregs were not any more proliferative than blood Tregs, with no change in %Ki67+ in Tregs across SF and PB from JIA or healthy adults. Analysis of co-receptors found the upregulation of the co-stimulatory receptor CD226 on SF Tregs, mostly co-expressed with TIGIT, while blood Tregs rarely co-expressed both co-receptors. Moreover, we identified a sub-population of CD137low ID2intermediate Tregs in PB of inactive individuals, with reversed expression pattern in PB Tregs from active disease, highlighting differences in Treg maintenance with disease activity.

**Conclusion:** High dimensional flow cytometry revealed subsets of Tregs within the inflammatory joint of JIA with a highly active and suppressive phenotype, yet we have identified changes to Treg overall fitness. A shift to a more co-stimulatory receptor presence on SF Tregs could be backing the persistence of inflammation within the joint. Alterations to Treg stability and maintenance in the blood during active disease may also hint towards immunoregulatory disruption in the periphery. These may have biomarker potential for predicting flare-ups and offer new potential mechanistic targets for restoring the immunological balance in JIA.

**Patient Consent:** Yes, I received consent

**Disclosure of Interest**: None declared

### O34. New biomarkers from plasma and synovial fluid of oligoarticular juvenile idiopathic arthritis patients

#### S. Pelassa^1^, C. Rossi^1^, F. Raggi^1^, D. Cangelosi^2^, M. Bartolucci^3^, A. Petretto^3^, F. Antonini^3^, P. Bocca^4^, F. Penco^4^, M. Rossano^5^, F. Baldo^5^, G. Filocamo^5^, C. Trincianti^6^, A. Eva^1^, A. Ravelli^4^, A. Consolaro^4^, M. C. Bosco^1^

##### ^1^Laboratory of Molecular Biology, ^2^Clinical Bioinformatic Unit, ^3^Core Facilities, ^4^Pediatric Rheumatology Clinic, IRCCS Istituto G. Gaslini, Genova, ^5^Fondazione IRCCS Ca’ Granda, Ospedale Maggiore Policlinico, Milano, ^6^University of Genova, Genova, Italy

###### **Correspondence:** S. Pelassa

**Introduction:** Extracellular vesicles (EVs) from plasma (PL) and synovial fluid (SF) could represent a valuable source of early predictive biomarkers for Oligoarticular Juvenile Idiopathic Arthritis (OJIA), the most common pediatric rheumatic disease in Western countries

**Objectives:** To identify new low-invasive biomarkers for early diagnosis of OJIA and prediction of disease course through the characterization of the EV miRNome (EVs-miR) and proteome (EV-prot) combined with the study of mononuclear cells in specimens from children with new-onset OJIA

**Methods:** EVs were isolated using a membrane affinity spin column from PL and SF of 50 OJIA patients at disease onset. Patients were followed up for 12 months, and clinical data were collected. PL samples were also obtained from 24 age/gender-matched control children (CTR-PL). EV-miR and EV-Prot expression profiling was carried out using the TaqMan Array RT-PCR and mass spectrometry, respectively. EVs surface protein expression was assessed by the MACSPlex Exosomes Kit. Macrophages and T cells subsets were isolated from the SF aspirates of 14 patients by MACS magnetic beads and characterized by cytofluorimetry**Results:** EVs-miR expression profiles were compared between OJIA-SF, OJIA-PL, and CTR-PL samples. We identified 46 EV-miR differentially expressed in SF vs both paired and control PL targeting genes involved in processes related to inflammation, cartilage/bone homeostasis, and hypoxia, suggesting EV-miR role in disease pathogenesis and potential as biomarkers of OJIA development.

Comparative analysis of the EV-miR Nome in OJIA-PL vs CTR-PL samples identified 57 EV-miRs able to differentiate OJIA patients from CTR children, of which 55 were significantly upregulated and 2 significantly downregulated. 15 EV-miR were consensually overexpressed in OJIA-SF and OJIA-PL compared to CTR samples, potentially representing a disease-specific EV-miR signature at both local and systemic levels. Correlation with clinical data demonstrated that a subset of 3 EV-miR was able to stratify OJIA patients in distinct subgroups, suggesting their potential as predictive biomarkers. Comparative analyses were also carried out among EV-Prot expression profiles of SF- and PL from 30 OJIA patients and 24 CTR children. Significant up- and down-regulation of 268 and 263 EV-Prot were demonstrated in SF vs both paired and CTR PL, respectively, several of which are involved in inflammatory and immunological processes. A subset of EV-Prot able to discriminate subgroups of patients was also identified within the OJIA cohort. We also evaluated the expression of surface proteins on EVs from SF and PL patients by flow cytometry, showing modulation of specific immunologic markers. Finally, we phenotypically characterized macrophages and T cell subsets from OJIA-SF samples. We demonstrated that the ratio between T cells (CD3+):macrophages (CD14+) and between cytotoxic T cells (CD8+):helper T cells (CD4+) were higher in patients who developed polyarticular course respect to patients with oligoarticular course .

**Conclusion:** We provide the first database integrating EV-miR, EV-Prot, and mononuclear cell phenotypic data from PL and SF of new-onset OJIA patients. Results may lead to a better understanding of the molecular mechanisms underlying OJIA development and the identification of new biomarkers for the disease

**Patient Consent:** Not applicable (there are no patient data)

**Disclosure of Interest**: None declared

### O35. Expansion of autoreactive CD27-IGD- double negative b cells in the joints of antinuclear antibody positive JIA patients

#### C. Strenzel^1^, J. Dirks^1^, G. Haase^1^, U. Fischer^1^, J. Fischer^1^, A. Holl-Wieden^2^, C. Hofmann^2^, H. Morbach^1^

##### ^1^Pediatric Immunology, ^2^Pediatric Rheumatology and Osteology, University Children’s Hospital, Wuerzburg, Germany

###### **Correspondence:** C. Strenzel

**Introduction:** Juvenile idiopathic arthritis (JIA) is a chronic inflammatory disease of unknown origin which is assumed to be mediated by an autoimmune response. The appearance of antinuclear antibodies (ANAs) in almost half of the patients suggests B cell dysregulation as a distinct pathomechanism of JIA. Furthermore, ANA positive JIA patients seem to constitute a clinically homogenous group of patients. However, the site of dysregulated activation of autoreactive B cells as well as the B cell subsets involved are still unknown.

**Objectives:** To dissect the distribution of different synovial fluid (SF) B cell subsets in JIA patients and assess their B cell receptor (BCR) specificities on a clonal level.

**Methods:** Distribution of different B cell subsets within SF were assessed by flow cytometry in a large cohort of JIA patients and correlated to disease subgroup (oligo-JIA, poly-JIA) and ANA status. Single cell sorting was used for generation of monoclonal antibodies (mAbs) from individually sorted B cells by expression cloning of the respective immunoglobulin genes. Generated mAbs from single B cells were assessed for antinuclear autoreactivity using HEp-2 based immunofluorescence tests. The phenotype of each B cell corresponding to the HEp-2 reactive mAbs was retraced by an index sorting algorithm.

**Results:** SF B cells in JIA patients mainly displayed a phenotype of activated CD21lo/-CD11c+ B cells. We used single B-cell based cloning technology to isolate and express immunoglobulin genes from SF B cells of 5 active JIA patients. By this, we generated 140 mAbs from SF CD21lo/-CD11c+ B cells from 3 ANA positive and 2 ANA negative JIA patients. Of these mABs 17-33% per patient were HEp-2 reactive and thus could be considered as autoreactive. The binding pattern of these mAbs in HEp-2 immunofluorescence tests revealed reactivity against cytoplasmic as well as nuclear antigens. Further flow cytometric analysis of SF B cells in a larger cohort of JIA patients (n=46) revealed a significant expansion of CD21^lo/-^CD11c^+^ CD27-IgD- “double-negative” (DN) B cells in ANA positive JIA patients. Retracing by index sort analysis showed that these DN B cells in ANA positive JIA patients were also enriched in autoreactive clones. Interestingly, HEp-2 reactive (“autoreactive”) B cell clones within the DN B cell and CD27+IgD- switched memory B cell compartment differed significantly in the frequency of somatic hypermutation (SHM) events with lower SHM frequencies in the first subset.

**Conclusion:** Accumulation of autoreactive B cell clones in the inflamed joints is a common phenomenon in JIA patients. However, CD21^lo/-^CD11c^+^ DN B cells are particularly expanded in the SF of ANA+ JIA patients and enriched in autoreactive B cell clones. The specific pattern of SHM within autoreactive DN B cell clones particularly expanded in ANA positive JIA patients suggests that different activation pathways may drive this B cell subset.

**Disclosure of Interest**: None declared

### O36. Traditional laboratory parameters and new biomarkers in Macrophage Activation Syndrome (MAS) and Secondary Hemophagocytic Lymphohistiocytosis (SHLH)

#### A. De Matteis^1^, D. Pires Marafon^1^, I. Caiello^1^, M. Pardeo^1^, G. Marucci^1^, E. Sacco^1^, F. Minoia^2^, F. Licciardi^3^, A. Miniaci^4^, I. Maccora^5^, M. C. Maggio^6^, G. Prencipe^7^, F. De Benedetti^8^, C. Bracaglia^8^

##### ^1^Division of Rheumatology, IRCCS Ospedale Pediatrico Bambino Gesù, Roma, Italy, Roma, ^2^Pediatric Rheumatology, Fondazione IRCCS Ca’ Grande Ospedale Maggiore Policlinico, Milano, ^3^ Department of Pediatrics and Infectious Diseases, School of Medicine, University of Turin, Regina Margherita Children’s Hospital, Torino, ^4^Department of Pediatrics, University of Bologna, S. Orsola-Malpighi Hospital, Bologna, ^5^Pediatric Rheumatology Unit, Meyer Children’s University Hospital, Firenze, ^6^University Department Pro.Sa.M.I. “G. D’Alessandro”, University of Palermo, Palermo, ^7^Division of Rheumatology, ERN-RITA Cent, IRCCS Ospedale Pediatrico Bambino Gesù, ^8^Division of Rheumatology, ERN-RITA center, IRCCS Ospedale Pediatrico Bambino Gesù, Roma, Italy

###### **Correspondence:** C. Bracaglia

**Introduction:** MAS and sHLH are hyperinflammatory conditions, in which IFNγ plays a pivotal role. Early recognition and treatment improve the outcome and the mortality rate.

**Objectives:** This is a retrospective multicenter study. We correlate traditional laboratory parameters of hyperinflammation with IL-18 and IFNγ related biomarkers. We have also evaluated the diagnostic and prognostic role of IL-18, CXCL9, CXCL10 and neopterin in patients with MAS and sHLH.

**Methods:** One hundred-six patients from 6 Italian centers were enrolled: 41 with sHLH, 41 with MAS in the context of sJIA, and 24 with sJIA without MAS. The samples were collected at three different time points: active disease (T0), 7-10 days from starting therapy (T1) and in clinical inactive disease on medication (from 1 to 3 months from onset) (T2).

**Results:** A total of 378 samples were collected. Laboratory features at T0 are detailed in table 1. Using the 2016 classification criteria for MAS, we can confirm that platelet count is a specific parameter, whilw ferritin is a sensitive parameter. Lactate dehydrogenase (LDH) values were statistically higher in MAS and sHLH compared to sJIA. ROC curve of LDH values in MAS showed a statistically significant area under the curve (AUC= 078.2%, p-value <0.0001). A cut-off of 683 U/L had a sensitivity of 73.6% and a specificity of 70.3%. IL-18, CXCL9, CXCL10 and neopterin levels in T0 were significantly higher in MAS and sHLH. In MAS, IL-18 levels were significantly higher compared to sHLH (p<0.0001). The ROC curves performed for each biomarker showed a statistically significant AUCs (p<0.01), except for IL-18 in sHLH. We have identified a cut off value for each biomarker in MAS (CXCL9 900 pg/ml, CXCL10 260 pg/ml, neopterin 5.0 ng/ml, IL-18 82996 pg/ml) and sHLH (CXCL9 2145 pg/ml, CXCL10 270 pg/ml, neopterin 7.1 ng/ml). In T0 neopterin correlates significantly with IL-18, CXCL9 and CXCL10 in MAS group but not in sHLH. We found also strong correlation between CXCL9 and CXCL10 only in MAS group.

**Conclusion:** Platelet count and ferritin have high specificity and sensitivity, respectively, to diagnose MAS in the context of sJIA. Even if LDH is not included in 2016 classification criteria for MAS in sJIA, we have found that this parameter could help to discriminate MAS in sJIA, in addition to the others. Our results confirm that IL-18 and the IFN-γ related biomarkers are significantly higher in patients with MAS and sHLH and might be useful to diagnose MAS/sHLH in addition to the traditional laboratory parameters. Moreover, IL-18 could help to distinguish sHLH from MAS and MAS from active sJIA.

**Patient Consent:** Yes, I received consent

**Disclosure of Interest**: A. De Matteis: None declared, D. Pires Marafon: None declared, I. Caiello: None declared, M. Pardeo: None declared, G. Marucci: None declared, E. Sacco: None declared, F. Minoia: None declared, F. Licciardi: None declared, A. Miniaci: None declared, I. Maccora: None declared, M. C. Maggio: None declared, G. Prencipe: None declared, F. De Benedetti Consultant with: Abbvie, SOBI, Novimmune, Novartis, Roche, Pfizer, Employee with: SOBI, C. Bracaglia Consultant with: SOBI and Novartis

**Table 1 (abstract O36).** Laboratory parameters in T0. Values are shown as median (IQR); p-value: Mann-Whitney U test

	MAS (***N***=54)	sHLH (***N***=48)	sJIA (***N***=4)	MAS vs sHLH	MAS vs sJIA	sHLH vs sJIA
Ferritin (ng/ml)	4755 (1816-10988)	4098 (2075-16867)	717 (335-2313)	0.77	**<0.0001**	**<0.0001**
Platelet count (x10^9/l)	199 (107-314)	97 (47-184)	450 (343-570)	**0.0003**	**<0.0001**	**<0.0001**
AST (U/L)	85 (51-146)	150 (51-340)	30 (22-44)	0.06	**<0.0001**	**<0.0001**
Triglycerides (mg/dl)	188 (148-263)	226 (166-381)	105 (75-139)	0.11	**<0.0001**	**<0.0001**
Fibrinogen (mg/dl)	337 (222-433)	228 (137-331)	572 (482-692)	**0.003**	**<0.0001**	**<0.0001**
LDH (U/L)	965 (679-1369)	1255 (701-2748)	587 (365-724)	0.10	**<0.0001**	**<0.0001**
CXCL9 (pg/ml)	2236 (675-9670)	4159 (1880-10016)	300 (300-1979)	0.14	**<0.0001**	**<0.0001**
Neopterin (ng/ml)	9.4 (5.0-16.2)	20.3 (9.6-35.0)	4.2 (3.0-7.2)	**0.0018**	**<0.0001**	**<0.0001**
IL-18 (pg/ml)	170338 (83277-287152)	11787 (21881120)	36764 (8958-82714)	**<0.0001**	**<0.0001**	0.19

## Poster session: JIA (oligo, poly, psoriatic)

### P001. A retrospective study of subcutaneous golimumab in Juvenile Idiopathic Arthritis (JIA), an alternative biologic in refractory disease

#### A. R. Al Mheiri, O. Killeen

##### Rheumatology, National Centre for Paediatric Rheumatology, CHI@Crumlin, Ireland, Dublin, Ireland

###### **Correspondence:** A. R. Al Mheiri

**Introduction:** The introduction of biologics at the turn of this century has greatly improved the clinical outcome of JIA. Golimumab, a monoclonal tumour necrosis factor (TNF)-antagonist antibody was approved for paediatric patients with Polyarticular JIA in 2020.

**Objectives:** The purpose of this study was to assess the effectiveness of Golimumab and to document our experience of this biologic agent to date in our National Centre.

**Methods:** This retrospective, observational chart review included all patients followed in our service with JIA, who were treated with golimumab, between November 1, 2017, and November 30, 2020. Data were collected for a minimum of twelve weeks after the start date, or until discontinuation of anti-TNF therapy, whichever occurred last. The primary outcomes were clinical effectiveness at 4-6 months, defined as 1. Active clinical response (decrease in total number of active joints), 2. Clinical inactive disease (no active arthritis; no fever, no rash, serositis, splenomegaly, or generalized lymph- adenopathy attributable to JIA; no active uveitis; normal erythrocyte sedimentation rate (ESR) and C-reactive protein (CRP) levels), 3. Clinical no response (same total active disease or worsening of active joint cunt). A total of 17 patients met the inclusion criteria and included in the final analysis.

**Results:** Five patients (29.4%) experienced a reduction in the total number of joints with active disease, six patients (35.3%) were in clinical inactive disease, four patients (23.5%) had same number of joints with active disease and two patients (11.8%) were worse at follow-up. No significant change was noticed in the inflammatory markers or CHAQ score. There were no reported significant side effects or any flare of uveitis or psoriasis in any of the patients. Golimumab therapy was discontinued in eight patients (47%) for lack of efficacy or loss of effectiveness.

**Conclusion:** Data from this analysis demonstrate that Golimumab is an effective therapy in treating patients with refractory JIA. It was well tolerated with no major adverse reactions or flare of extra-articular co morbidities of psoriasis or uveitis.

**Disclosure of Interest**: None declared

### P002. The potential of CD161+ T cells as a surrogate measure of IL-17A expressing T cells in the synovial fluid of JIA patients

#### V. Alexiou^1,2^, B. Jebson^1,2^, E. Ralph^1,2,3^, M. Kartawinata^1,2^, E. Vigorito ^4^, L. R. Wedderburn^1,2,3^ on behalf of the CLUSTER Consortium

##### ^1^Centre for Adolescent Rheumatology Versus Arthritis at UCL University College London Hospital (UCLH) and Great Ormond Street Hospital (GOSH), ^2^Infection, Immunity and Inflammation Programme, UCL Great Ormond Street Institute of Child Health, ^3^National Institute for Health Research (NIHR) GOSH Biomedical Research Centre, London, ^4^MRC Biostatistics Unit, University of Cambridge, Cambridge, United Kingdom

###### **Correspondence:** V. Alexiou

**Introduction:** CD161+ T cells are highly enriched in the synovial fluid (SF) of juvenile idiopathic arthritis (JIA) patients. The group has previously shown that CD161 (a C-type lectin-like receptor encoded by *KLRB1*) is expressed on Th17 cells undergoing transition to an intermediate Th17/Th1 fate in response to inflammation^1^. Therapeutic agents targeting the Th17 cytokine, IL-17A, such as Ixekizumab and Secukinumab are currently used primarily for psoriatic arthritis and ankylosing spondylitis. Their use in JIA could be informed by assessing the levels of IL-17A production in JIA patients. Identifying a reliable surrogate marker for IL-17A which can be easily clinically tested, such as cell surface marker CD161, could be valuable for patient stratification for the administration of IL-17A targeting agents.

**Objectives:** To explore the relationship between the CD161+ CD4 T cell population and the pro-inflammatory environment of the joint in JIA, and to test the hypothesis that the CD161 molecule is a surrogate marker of IL-17A-expressing T cells in the joints of JIA patients.

**Methods:** Multi-parameter flow cytometry analysis was performed on paired (collected on the same day or timepoint) samples of peripheral blood mononuclear cells (PBMC) and synovial fluid mononuclear cells (SFMC) of JIA patients (n=47, male:16, female:31). The JIA patients were grouped by ILAR subtype: oligo-articular JIA (n=21), poly-articular JIA (n=15), systemic JIA (n=6), enthesitis-related JIA (n=3) and psoriatic JIA (n=2). T cell populations were analysed based on the expression of CD3, CD4, CD8 and CD161. Following PMA/Ionomycin stimulation in the presence of Brefeldin A, intracellular production of GM-CSF, IFNγ, IL-17A. TNFα and IL-10 was analysed.

**Results:** Analysis confirmed that the proportion of CD161+ CD4 T cells was increased in SFMC relative to peripheral blood PBMC of JIA patients (p <0.0001). Correlation analysis showed that CD161+ CD4 T cells strongly, positively correlate with IL-17A-expressing CD4 T cells in both PB (r =0.935, P<0.0001) and SF (r =0.823, P=0.0003). The analysis also showed that CD161+ positively correlated with CD4 T cells expressing other pro-inflammatory cytokines, TNFα (r = 0.626, P=0.0165) and GM-CSF (r =0.563, P=0.045), but not those expressing IFNg (r = −0.100, P=0.733). This concurs well with previous studies which have shown that IFNγ is also expressed by the CD161− CD4 T cell population in the joint^1,2^. Additionally, IL-17A expressing CD4 T cells correlate between PB and SF of JIA patients (r = 0.414, P=0.013).

**Conclusion:** These data show that the proportions of IL-17A+ CD4 T cells correlate between the PB and SF of JIA patients and that the CD161+ CD4 T cell population may serve as a surrogate marker of IL-17A expressing CD4 T cells in both compartments. CD161 may represent a reliable indicator of the Th17/Th1 type of inflammation in the joint and extent of inflammation. This could have translational potential, as the detection of a cell surface marker provides a readily measurable biomarker and could be valuable for the provision of treatments targeting IL-17A in JIA.

**References:**

1. Nistala K, Adams S, Cambrook H, et al. Th17 plasticity in human autoimmune arthritis is driven by the inflammatory environment. *Proc Natl Acad Sci U S A*. 2010;107(33):14751-14756. doi:10.1073/pnas.1003852107

2. Piper C, Pesenacker AM, Bending D, et al. T cell expression of granulocyte-macrophage colony-stimulating factor in juvenile arthritis is contingent upon Th17 plasticity. *Arthritis Rheumatol*. 2014;66(7):1955-1960. doi:10.1002/art.38647

**Patient Consent:** Yes, I received consent

**Disclosure of Interest**: V. Alexiou Grant / Research Support with: NIHR BRC at GOSH, B. Jebson Grant / Research Support with: NIHR BRC at GOSH, E. Ralph Grant / Research Support with: NIHR BRC at GOSH, M. Kartawinata Grant / Research Support with: NIHR BRC at GOSH, E. Vigorito : None declared, L. Wedderburn Grant / Research Support with: NIHR BRC at GOSH. The CLUSTER Consortium is supported by grants from MRC, Versus Arthritis and GOSCC and has partnerships with AbbVie, GSK, UCB, Sobi and Pfizer inc. LWR declares in kind contributions to CLUSTER by AbbVie, GSK, UCB, Sobi and Pfizer inc and non-renumerated collaborations with Lilly and Novartis.

### P003. Inflammatory arthritis as the presentation finding of autoimmune polyendocrinopathy with candidiasis and ectodermal dystrophy syndrome

#### G. Aytac^1^, B. Guven ^2^, I. Aydin^2^, E. Topyildiz^3^, A. Aykut^4^, G. Aksu^1^, N. E. K. Edeer Karaca^3^, N. Kutukculer^1^

##### ^1^Pediatric rheumatology, Ege University, ^2^Pediatrik romatoloji, Ege Üniversitesi, ^3^Pediatrik immunolgy, ^4^Ege University, Izmir, Turkey

###### **Correspondence:** G. Aytac

**Introduction:** Introduction: Autoimmune polyendocrinopathy with candidiasis and ectodermal dystrophy (APECED) is an autosomal recessive disorder of immune regulation caused by mutations in the autoimmune regulatory (*AIRE*) gene. AIRE is a protein involved in the development of central immunological tolerance. APACED is characterized by multiorgan autoimmunity leading to chronic mucocutaneous candidiasis (CMC), hypoparathyroidism and adrenocortical failure.

**Objectives:** Case: A 3-year-old boy was admitted with complaints of fever, pain and swelling on ankles and nail dystrophy on thumbs for 2 months. He was born to third-degree consanguineous healthy parents. On physical examination, the patient had splenomegaly, onychomycosis on both thumbs with oral candidiasis. Musculoskeletal examination demonstrated joint effusion with a decreased range of motion of wrist and ankles bilaterally and swelling of the knee. Laboratory investigations showed elevated acute phase reactants, hypergammaglobulinemia, and antinuclear antibody positivity. Biochemical parameters, including calcium, were normal. Rheumatic factor, antineutrophil cytoplasmic antibody, and antithyroid antibodies were negative. The patient responded well to nonsteroid antiinflammatory medications. Due to the coexistence of onychomycosis, arthritis, splenomegaly, consanguinity, and hypergammaglobulinemia, targeted next-generation sequencing of a Primary Immune Deficiency Research Panel was performed and a homozygous mutation (c.769C>T, p. Arg257Ter) in the AIRE gene was detected.

**Methods:** There is no method section as it is a case report.

**Results:** None

**Conclusion:** Conclusion: The classical triad of APACED syndrome is CMC, hypoparathyroidism and adrenal failure. Inflammatory arthritis is rarely described in association with APECED. The clinical presentation of our patient reinforces the idea that non-classical manifestations can occur before classic symptoms develop. It is, therefore, useful to consider the diagnosis in patients with CMC and arthritis beyond the first year of life.

**Patient Consent:** Yes, I received consent

**Disclosure of Interest**: None declared

### P004. The predictors of etanercept efficacy and treatment outcomes in non-systemic juvenile idiopathic arthritis: the data of 375 patients

#### K. Belozerov^1,1^, A. Lobacheva^1^, E. Gaidar^1^, A. Yakovlev^1^, N. Gordeeva^2^, A. Khabirova^1^, A. Kupreeva^1^, V. Masalova^1^, T. Kornishina^1^, E. Isupova^1^, L. Snegireva^1^, O. Kalashnikova^1^, L. Sorokina^1^, M. Kaneva^1^, I. Chikova^1^, T. Likhacheva^1^, M. Dubko^1^, V. Chasnyk^1^, M. Kostik^1^

##### ^1^Department of Hospital Pediatrics, Federal State budgetary Educational Institution of Higher Education «St. Petersburg State Pediatric Medical University» of the Ministry of Healthcare of the Russian Federation, ^2^Cardiorheumatology, Children’s City Hospital #2 of Holy Mary Magdalene, Saint-Petersburg, Russian Federation

###### **Correspondence:** M. Kostik

**Introduction:** TNF-⍺ is a one of the predominating cytokines in the pathogenesis of juvenile idiopathic arthritis (JIA). There are more than 20 years experience of using TNF-ibhinitors for treatment of JIA.

**Objectives:** to report about our 10 years experience of treatment JIA with etanercept (ETN).

**Methods:** We retrospectively studied cases of 375 patients, who were diagnosed as JIA with ILAR 2001 criteria and who used ETN. The treatment compliance, remission achievement, flare, as well as the switching to next biologic and the appearance of uveitis (de-novo) were taken into account.

**Results:** according to our data, 90.4% of children were receiving DMARDs (mostly methotrexate) at the time of ETN initiation. Other biological agents were used before ETN in 6.9% of cases. Initial JIA remission was observed in 254/322 (78.9%) cases after 6 months (0,3-1,6 years) after starting therapy. Patients who hadn’t previously received biological therapy achieved remission faster (LogRank test, p=0.001), as well as those who used therapy regularly (LogRank test, p=0.004). Cumulative likelihood of flare was higher in ANA-positive patients (LogRank test, p=0.060) and RF-positive polyarthritis. The presence of concomitant non-biological treatment didn’t affect the likelihood of flare. The switch from etanercept to another biological drag occurred in 47/270 (17.4%) children, which in a quarter of cases is associated with the development of de-novo uveitis.

**Conclusion:** ETN is effective, especially in distinct JIA categories.

**Patient Consent:** Not applicable (there are no patient data)

**Disclosure of Interest**: None declared

**Table 1 (abstract P004).** Outcomes of etanercept therapy for different JIA subtypes

Point	Oligoartritis	Polyartritis RF-negative	Polyartritis RF-positive	Enthesitis	Psoriatc arthritis	***р***
Time before ETN, years	2,2 (1,3; 4,9)	2,4 (0,8; 5,1)	1,6 (0,8; 3,3)	2,1 (0,9; 4,0)	2,6 (1,3; 4,8)	0,767
Remission, n (%)	55/69 (79,7)	119/149 (79,9)	14/18 (77,8)	54/71 (76,1)	12/15 (80,0)	0,976
Time to remission, years	0,5 (0,3; 1,3)	0,7 (0,4; 1,4)	0,8 (0,4; 1,3)	0,6 (0,3; 1,2)	0,7 (0,3; 1,6)	0,899
Flare, n (%)	13/50 (26,0)	32/112 (28,6)	5/16 (31,3)	16/48 (33,3)	4/11 (36,4)	0,917
Time to flare, mounths.	26,3 (7,0; 41,5)	18,2 (6,0; 25,4)	4,4 (4,0; 9,1)	7,6 (4,7; 14,2)	3,6 (3,0; 10,5)	0,050
Change of biological therapy	7/59 (11,9)	21/128 (16,4)	3/18 (16,7)	13/61 (21,3)	3/14 (21,4)	0,709

### P005. Feasibility study of taking minimally invasive, ultrasound-guided tissue biopsies of synovial tissue in children with juvenile idiopathic arthritis for research

#### C. Bolton^1^, C. G. Smith^2^, A. McNeece^1^, S. Sultan^3^, V. Alexiou^1^, A. Hackland^2^, J. Crook^4^, H. D. Nguyen^1^, C. C. -^5^, M. Thyagarajan^3^, Z. Shiekh^6^, C. Cotter^3^, P. Reis Nisa^2^, E. Al-Abadi^3^, S. Chippington^7^, S. Compeyrot-Lacassagne^7^, A. Filer^3^, L. Wedderburn^1^, A. Croft^2^

##### ^1^UCL, London, ^2^University of Birmingham, ^3^Birmingham Women’s and Children’s NHS Foundation Trust, Birmingham, ^4^Guy’s and St Thomas’ Hospital, London, ^5^CLUSTER Consortium, UK wide, ^6^Birmingham Women’s and Children’s NHS Foundation Trust, ^7^Great Ormond Street Hospital, London, United Kingdom

###### **Correspondence:** H D Nguyen

**Introduction:** When investigating disease mechanisms, site-specific differences in immune cell phenotype and function have highlighted the need to analyse cellular and molecular mechanisms at the tissue site directly^1^. In adults, the ability to obtain synovial tissue biopsies using ultrasound-guided techniques, combined with advanced tissue analytics, has revolutionised our understanding of the cellular ecosystem that operates within the joint and how it contributes to disease^2^. However, a similar approach in paediatric disease is lacking.

**Objectives:** 1) To describe the protocol for undertaking minimally-invasive ultrasound-guided synovial tissue biopsies in children and young people with arthritis, for the purpose of research, alongside routine clinical care. 2) To investigate whether high-quality synovial tissue can be obtained that is suitable for downstream applications including single cell profiling technologies, histology and digital spatial profiling.

**Methods:** Following ethical approval, treatment-naïve children with a diagnosis of Juvenile Idiopathic Arthritis were recruited from two large UK Paediatric Rheumatology centres. Participating families completed questionnaires prior to and following synovial biopsy. We established a workflow pipeline for performing synovial tissue biopsies in child and young people with arthritis, using standardised procedures for biopsy and sample processing. Procedures were performed by experienced paediatric interventional radiologists with experience of joint biopsy for diagnostic purposes. In brief, children who were referred for aspiration and corticosteroid joint injection were recruited. Following a general anaesthesic, required as part of routine clinical care and the establishment of sterility, synovial fluid was aspirated. Needle-biopsies were undertaken from the same needle insertion site and subsequently corticosteroid was injected into the joint. Thickened synovium was graded via ultrasonography.

**Results:** 10 participants were recruited to the study over a nine month period, with a median age of 7 years (range 1-16 years); 90% were female. Samples obtained included core synovial biopsies, paired synovial fluid and peripheral blood. Synovial tissue fragments were processed for histology by formalin fixation and cryopreserved for downstream applications, including RNA sequencing and cell culture. Quality control indices included histological analysis to ensure the biopsied material was characteristically synovium and to grade the severity of inflammation. No significant complications were reported, however one child had a mild hemarthrosis controlled with cold saline wash out and cold compresses.

**Conclusion:** Obtaining biopsies of synovial tissue in children with Juvenile Idiopathic Arthritis for the purpose of research, alongside clinical care is feasible. Analysis of tissue direct from the site of inflammation with single-cell RNA sequencing in children is achievable.

1 Veale DJ, Fearon U. Next-generation analysis of synovial tissue architecture. Nat. Rev. Rheumatol. 2020; **16**: 67–68.

2 Croft AP, Campos J, Jansen K, Turner JD, Marshall J, Attar M *et al.* Distinct fibroblast subsets drive inflammation and damage in arthritis. *Nature* 2019; **570**: 246–251.

**Patient Consent:** Yes, I received consent

**Disclosure of Interest**: None declared

### P006. IL-4 and S100A9 can differentiate acute lymphoblastic leukemia from juvenile idiopathic arthritis better than existing laboratory values

#### N. Brix^1,2^, M. Glerup ^1^, D. Foell^3^, C. Kessel^3^, H. Wittkowski^3^, L. Berntson^4^, A. Fasth^5^, S. Nielsen^6^, E. Nordal ^7^, M. Rygg^8,9^, H. Hasle^1^, S. Hagstrøm^2^, T. Herlin^1^ on behalf of the Nordic Study Group of Pediatric Rheumatology (NoSPeR) group

##### ^1^Department of Pediatrics and Adolescent Medicine, Aarhus University Hospital, Aarhus, ^2^Department of Pediatrics and Adolescent Medicine, Aalborg University Hospital , Aalborg , Denmark, ^3^Department of Pediatric Rheumatology and Immunology, Medical Center (UKM), Muenster, Germany, ^4^Department of Women’s and Children’s Health, Uppsala University, Uppsala, ^5^Department of Pediatrics, Institute of Clinical Sciences, Sahlgrenska Academy, University of Gothenburg, Gothenburg, Sweden, ^6^Department of Pediatrics, Rigshospitalet, Copenhagen University Hospital, Copenhagen , Denmark, ^7^Department of Pediatrics, University Hospital of North Norway, and Department of Clinical Medicine, UiT The Arctic University of Norway, Tromsø, ^8^Department of Clinical and Molecular Medicine, NTNU - Norwegian University of Science and Technology, ^9^Department of Pediatrics, St. Olavs Hospital, Trondheim, Norway

###### **Correspondence:** N. Brix

**Introduction:** Acute lymphoblastic leukemia (ALL) may be misdiagnosed as juvenile idiopathic arthritis (JIA) with the risk of mistreatment and prolonged diagnostic interval. In demand are sensitive and specific biomarkers in order to optimize the diagnostic process.

**Objectives:** To evaluate the predictive value of biomarkers of inflammation like phagocyte-related S100 proteins and a panel of cytokines in order to differentiate the child with ALL and arthropathy from the child with JIA.

**Methods:** In this cross-sectional study, we measured S100A9, S100A12 and 14 cytokines in serum from children with ALL (n=150, including 27 with arthropathy) and JIA (n=238) by multiplexed bead array assay on a MAGPIX instrument using Luminex software. We constructed predictive models using logistic regression, including ‘ten-fold cross-validation and recalibration. Thereby ﻿computing the area under the curve (AUC) as well as predicted probabilities in order to differentiate ALL from JIA.

**Results:** The proinflammatory S100A12 was three-fold higher and the anti-inflammatory IL-10 two-fold lower in the patients with ALL and arthropathy (n = 27) compared to the ALL patients without any arthropathy (p = 0.002 and p = 0.03, respectively). The median levels of IL-4 was 12 pg/mL (IQR 10-14) in ALL with arthropathy versus 89 pg/mL (IQR 55-133) in the JIA patient, p < 0.001. The median levels of S100A9 was 47 pg/mL (IQR 30-113) in ALL with arthropathy versus 511 pg/mL (IQR 315-1281) in JIA, p < 0.001. In predictive models, we found IL-4 and S100A9 to be valuable markers to separate the child with ALL from JIA, with AUCs of 98% (95% CI: 98-100%) and 95% (95% CI: 91-97%), respectively, exceeding both hemoglobin, CRP, ESR and for IL-4 also platelets.

**Conclusion:** The IL-4 and S100A9 serum concentration may be used as biological serum markers to distinguish ALL with arthropathy from JIA.

**Disclosure of Interest**: None declared

### P007. Economic impact of juvenile idiopathic arthritis in Mexico

#### R. C. Calderón Zamora^1^, A. K. Leos-Leija^1^, N. Rubio-Perez^1^, A. V. Villarreal-Treviño^1^, F. García-Rodríguez^1^, I. Peláez-Ballestas^2^, E. Faugier-Fuentes^3^

##### ^1^Department of Pediatrics, Universidad Autónoma de Nuevo León, Hospital Universitario “Dr. José E. González”, Monterrey, ^2^Rheumatology Unit, Hospital General de México “Dr. Eduardo Liceaga”, ^3^Rheumatology Department, Hospital Infantil de México “Federico Gómez” , Cd de México, Mexico

###### **Correspondence:** R. C. Calderón Zamora

**Introduction:** Juvenile Idiopathic Arthritis (JIA) is the most common chronic pediatric rheumatic disease. Due to the multidisciplinary treatment and the wide range of therapeutic options, this disease is considered to have a high economic cost (1), however, it depends on factors as family income, coverage for medical services, the cost of medications, the frequency of exams and interventions, and disease activity and progression (2, 3). There are few reports about economic cost of JIA in the world (4), especially in those low and middle-low-income countries.

**Objectives:** The aim of this study is to estimate the economic costs for the family of patients with Juvenile Idiopathic Arthritis in Mexico.

**Methods:** Observational and descriptive study including caregivers of pediatric patients with JIA (according to the ILAR Classification) from January to May 2022. Data related to health care, medication, complementary exams, indirect costs, and total family income were collected by the adaptation of the questionnaire “Determination of the Economic Impact on Rheumatic Diseases, Section B. Interview regarding demographic and socioeconomic aspects”. A descriptive analysis of the data was carried out, reporting measures of central tendency and proportions.

**Results:** Twenty-nine caregivers of patients with JIA were included, with an average age of 37 years (IQR 23-53), 25 were female, 15/29 caregivers had 12 or more years of formal education, and 48% had a remunerated job. The patients had a mean age of 10 years (IQR 5-19), 69% female, the predominant subtype is polyarticular arthritis (18, 62%), followed by oligoarticular and systemic arthritis (4, 13.7% each), no patient had psoriatic or undifferentiated JIA. One third patients had history of hospitalization due to disease activity, only 3 patients reported disability. The most prescribed treatment is DMARDs, 75% synthetics and 31% biologicals. The proportion of total family income used for JIA related costs was 34%, 14.5% associated with treatment and 19.5% from other direct and indirect health care (Table). Half of the caregivers responded that after the diagnosis of JIA in their patient, their economic situation worsened a little, and a third of the relatives considered that the situation worsened significantly.

**Conclusion:** The results of this study demonstrate the impact of JIA on families. The direct costs of the disease are influenced by the partial or total coverage of public health institutions, however, indirect costs reflect the difficulties to specialized health care for the patients. The impact that JIA causes in the economy of the patient and his family, can significantly affect adherence to treatment and multidisciplinary care, worsen their prognosis.

**Trial registration identifying number:** 1. Angelis A, BURQOL-RD Research Network, Kanavos P, López-Bastida J, Linertová R, Serrano-Aguilar P. Socioeconomic costs and health-related quality of life in juvenile idiopathic arthritis: a cost-of-illness study in the United Kingdom. BMC Musculoskelet Disord [Internet]. 2016;17(1).

2. Lapsley HM, March LM, Tribe KL, Cross MJ, Courtenay BG, Brooks PM, et al. Living with rheumatoid arthritis: expenditures, health status, and social impact on patients. Ann Rheum Dis [Internet]. 2002;61(9):818–21.

3. Mould-Quevedo J, Peláez-Ballestas I, Vázquez-Mellado J, et al. El costo de las principales enfermedades reumáticas inflamatorias desde la perspectiva del paciente en México. Gac Med Mex. 2008;144(3):225-231.

4. García-Rodríguez F, Gamboa-Alonso A, Jiménez-Hernández S, Ochoa-Alderete L, Barrientos-Martínez VA, Alvarez-Villalobos NA, et al. Economic impact of Juvenile Idiopathic Arthritis: a systematic review. Pediatr Rheumatol Online J [Internet]. 2021;19(1)

**Patient Consent:** Yes, I received consent

**Disclosure of Interest**: None declared

**Table 1 (abstract P007).** Characteristics of the patients and caregivers participating in the study (n = 29)

Public medical access (%)	10 (34.3)
Medium socioeconomic status or above (%)	15 (51.7)
Cost of monthly medications, median per month (IQR)*	54.3 (16.7-118.5)
Cost of laboratories, each visit, median (IQR)*	34 (14.8-148.2)
Cost of image exams, each, median (IQR)*	28.1 (17.2-98.7)
Cost of health care, median (IQR)”*	46.9 (24.6-642)
Indirect costs, median (IQR)>*	64.2 (4.9-518.5)
Time to the center in hours, median (IQR)	3.5 (0.5-14)
Percentage of spending on medicines over net income, median (IQR)	14.5 (0.4-36.3)
Percentage of spending per health visit over net income, median (IQR)	19.5 (1.6-70)

IQR: Interquartile range. Including hospitalization & surgeries, > including transportation, food, lodging, exercise *dollar USA

### P008. Predictors of 2 and 5 years sustained disease remission among children and young adults with extended oligoarticular, enthesitis-related, or psoriatic juvenile idiopathic arthritis: results from clipper studies

#### V. Chasnyk^1^, I. Nikishina^2^, P. Dolezalova^3^, I. Rumba-Rozenfelde^4^, N. Wulffraat^5^, R. Burgos-Vargas^6^, J. Chaitow^7^, A. Martini^8^, V. Tsekouras^9^, D. Graham^9^, C. Borlenghi^9^, B. Vlahos^9^, C. Zang^9^, N. Ruperto^10^ on behalf of Paediatric Rheumatology International Trials Organisation (PRINTO)

##### ^1^Saint-Petersburg State Pediatric Medical University, Saint-Petersburg, ^2^Pediatric Department, V.A. Nasonova Research Institute of Rheumatology, Moscow, Russian Federation, ^3^General University Hospital, Faculty of Medicine, Charles University in Prague, Prague, Czech Republic, ^4^University Children Hospital, Riga, Latvia, ^5^Department of Pediatric Immunology and Rheumatology, Wilhelmina Children’s Hospital, Utrecht, Netherlands, ^6^Department of Rheumatology, Hospital General de Mexico, Mexico City, Mexico, ^7^Sydney Adventist Hospital, Wahroonga, NSW, Australia, ^8^Università degli Studi di Genova, Genoa, Italy, ^9^Pfizer, New York, NY, United States, ^10^Istituto Gianina Gaslini, Genoa, Italy

###### **Correspondence:** N. Ruperto

**Introduction:** CLIPPER2 (NCT01421069) was an 8-year, open-label extension of the phase 3b, 2-year CLIPPER study (NCT00962741) investigating the long-term safety/efficacy of etanercept (ETN) in patients with 3 subtypes of juvenile idiopathic arthritis (JIA): extended oligoarticular JIA (eoJIA), enthesitis-related arthritis (ERA), or psoriatic arthritis (PsA).

**Objectives:** To identify predictors of sustained clinical remission over 2 and 5 years.

**Methods:** Patients with eoJIA (2–17 years), ERA, or PsA (each 12–17 years) who received ≥1 ETN dose in CLIPPER could enter CLIPPER2. Inactive disease was defined according to the American College of Rheumatology (ACR)-approved Juvenile Arthritis Disease Activity Score (JADAS, Consolaro et al. 2009) and Wallace cut-off criteria (Wallace et al. 2004). Sustained clinical remission was defined as consecutive ≥2 years or ≥5 years of inactive disease using either set of criteria. Predictors of sustained disease remission were identified using a stepwise logistic regression model.

**Results:** Of 127 patients enrolled in CLIPPER, 109 enrolled into CLIPPER2. 84 patients completed CLIPPER2; 27 still received active treatment. 26 patients were in clinical remission ≥2 years after starting CLIPPER according to JADAS criteria and 13 according to Wallace criteria. 6 patients were in JADAS remission and 2 in Wallace remission after ≥5 years. Predictors of response are shown in the Table.

**Conclusion:** JADAS low disease activity (LDA) at 3 months and lower prorated number of active joints were significant predictors of 2-year sustained JADAS remission; Wallace remission at 3 months, higher Childhood Health Assessment Questionnaire score at baseline, JADAS LDA at 3 months, and baseline methotrexate use were significant predictors of 2-year Wallace remission.

The low number of patients remaining in remission over 5 years limited the predictive power of these data.

**Trial registration identifying number:** NCT01421069, NCT00962741

**Patient Consent:** Not applicable (there are no patient data)

**Disclosure of Interest**: V. Chasnyk Consultant with: Amgen, Bristol Myers Squibb, Eli Lilly, Pfizer, GlaxoSmithKline, UCB, Novartis, I. Nikishina Consultant with: Pfizer, Novartis, MSD, Roche, Sobi, UCB, Eli Lilly, Ipsen, P. Dolezalova Consultant with: Sobi, Novartis, Pfizer, I. Rumba-Rozenfelde Consultant with: Pfizer, N. Wulffraat Consultant with: Sobi, Novartis, UBC, R. Burgos-Vargas: None declared, J. Chaitow: None declared, A. Martini Consultant with: Aurinia, Bristol Myers Squibb, Eli Lilly, EMD Serono, Janssen, Pfizer, Roche, V. Tsekouras Employee with: Pfizer, D. Graham Employee with: Pfizer, C. Borlenghi Employee with: Pfizer, B. Vlahos Employee with: Pfizer, C. Zang Employee with: Pfizer, N. Ruperto Consultant with: Ablynx, Amgen, AstraZeneca-MedImmune, Aurinia, Bayer, Cambridge Healthcare Research, Celgene, Domain Therapeutic, EMD Serono, GlaxoSmithKline, Idorsia, Janssen, UCB, Bristol Myers Squibb, Eli Lilly, Novartis, Pfizer, Sobi, F. Hoffmann-La Roche.

**Table 1 (abstract P008).** Predictors of sustained clinical remission

Predictor, OR (95% CI)	JADAS criteria	Wallace criteria
**≥2 years sustained remission**		
JADAS LDA at 3 months*	4.0 (1.5; 10.6); p=0.005	6.1 (1.0; 36.4); p=0.047
**Prorated number of active joints ≤1**	4.8 (1.1; 21.4); p=0.038	–
Wallace remission at 3 months*	–	51.3 (3.0; 884.2); p=0.007
**CHAQ score >0.75**	–	12.8 (1.2; 137.4); p=0.035
**Baseline MTX use**	–	23.1 (1.0; 510.9); p=0.047
**≥5 years sustained remission**		
**Age at disease onset ≤0.41 years**	48.5 (2.2; 1082.4); p=0.014	110.0 (3.7; 3295.1); p=0.007
Wallace remission at 3 months*	9.7 (1.2; 76.5); p=0.031	–

*after starting treatment during CLIPPER

*CHAQ* Childhood Health Assessment Questionnaire; *LDA* low disease activity; *MTX* methotrexate

### P009. Understanding the role of ultrasound in detecting temporomandibular joint involvement in juvenile idiopathic arthritis: a pilot study

#### C. Eboli^1^, E. A. Conti^1^, F. Chironi^1^, S. Testa^1^, F. Lucioni^1^, G. B. Beretta^1^, A. Marino^2^, P. Cressoni^3^, S. Lanni^1^, M. Rossano^1^, G. Filocamo^1^

##### ^1^Clinica Pediatrica De Marchi, IRCCS Ca’ Granda Ospedale Maggiore Policlinico, ^2^ASST Gaetano Pini-CTO, ^3^IRCCS Ca’ Granda Ospedale Maggiore Policlinico, Milan, Italy

###### **Correspondence:** A. Conti

**Introduction:** Involvement of the temporomandibular joint (TMJ) is frequent in juvenile idiopathic arthritis (JIA). Early recognition and treatment of TMJ arthritis may potentially reduce long-term damage, such as facial asymmetry, micro and retro-gnathia, dental malocclusion, and joint ankylosis. A TMJ clinical examination protocol has been recently developed to screen TMJ involvement in JIA, and showed acceptable construct validity and reliability [1]. Ultrasound (US) has been suggested as a useful tool to evaluate inflammation and damage of TMJ in JIA [2].

**Objectives:** The aim of the study was to establish the validity of US in detecting signs of TMJ arthritis in JIA irrespective of the presence of clinical signs of involvement.

**Methods:** A sample of consecutive JIA patients, either in remission or with active disease, seen at our Centre underwent clinical evaluation of TMJs. Patients with TMJ disorders (dental problems, pre-existing cranio-maxillofacial disorder, history of facial trauma) unrelated to JIA were excluded. History of TMJ pain or dysfunction was assessed by a TMJ screening questionnaire for parents together with the Italian version of the Juvenile Arthritis Multidimensional Assessment Report (JAMAR). Clinical assessment was performed according the published TMJ screening protocol. Clinical and US examinations were carried out on the same consultation and blindly each other. TMJ was considered clinically involved in case of presence of either signs of active TMJ arthritis or evidence of TMJ deformity. Clinical data were then compared to US assessment.

**Results:** : A sample of 23 consecutive patients were recruited in the study. Overall clinical and US examinations resulted well accepted by patients and parents.

The table shows the preliminary results on clinical and US assessment.

**Conclusion:** The results observed in this preliminary cohort of patients do not allow to evidence a strong agreement between US and clinical data in detecting signs of previous or ongoing involvement of TMJ in JIA. Further studies with a larger cohorts of patients and involving the use of MRI will help to clarify the role of US in the assessment of TMJ arthritis in JIA.

**Patient Consent:** Yes, I received consent

**Disclosure of Interest**: None declared

**Table 1 (abstract P009).** See text for description

	Patients with history of TMJ disfunction (N 4)	Patients with clinical signs of TMJ disfunction (N 5)	Absence of history or clinical findings of TMJ disfunction (N 16)
Functionality score ^1^	2.5 (1.5-3.8)	0 (0-2)	0 (0-0)
Quality of life score ^1^	3.5 (2-5)	2 (2-3)	1 (0-4)
VAS pain ^1^	5.5 (3.8-6)	0.5 (0-2)	0 (0-0)
VAS MD global ^1^	0.5 (0.3-0.8)	1 (0-1.5)	0 (0-0)
Patients with US abnormalities ^2^	3	4	9

1: median value (interquartile range); 2: number

### P010. Study of diagnostic delay in patients with juvenile idiopathic arthritis

#### M. Corbeto, E. M. Ruzafa, L. M. Mitjana

##### Pediatric Rheumatology, Hospital Universitari Vall d’Hebron, Barcelona, Spain

###### **Correspondence:** M. Corbeto

**Introduction:** Juvenile idiopathic arthritis (JIA) is the most common cause of inflammatory arthritis in children under 16 years of age. The only epidemiological project carried out to date in southern Europe estimated a prevalence rate of 39.2/100,000 children. However, we do not have studies evaluating the patient’s journey towards accurate diagnosis and care in our area. The time to diagnosis (TD) is a fundamental quality measure since increases in this can lead to a higher comorbidity of patients with JIA.

**Objectives:** To describe and analyze the time to diagnosis from the onset of symptoms in patients with JIA and to identify factors associated with the patient related to a longer time to diagnosis.

**Methods:** We conducted a retrospective cohort study by reviewing the medical records of patients diagnosed with JIA according to the ILAR criteria and treated at the Pediatric Rheumatology Unit (PRU) of the Vall d’Hebron University Hospital from 2009 to the present. Epidemiological variables were collected. Time to diagnosis (TD) was defined as the time elapsed between the onset of symptoms and the diagnosis established in the PRU. Delay in TD has been categorized as times greater than two months. Time to referral (TTR), defined as the time elapsed between the onset of symptoms and the date of referral to the PRU, was also evaluated. Qualitative variables are expressed in absolute values ​​and percentages. Quantitative variables are expressed as medians and IQR as they present a non-normal distribution. In the bivariate analysis, the qualitative variables have been compared with the χ2 test and the quantitative variables with logistic regression. The level of statistical significance has been set at 0.05. Statistical analyzes have been performed with STATA/IC 15.1

**Results:** 60 patients have been included, 40 girls (66.67%) with a measured age of 8.01 years (SD 4.43). All patients come from an urban environment. The most frequent JIA subtype is oligoarticular with 28 patients (46.67%). 98% of the patients presented joint symptoms at debut followed by systemic manifestations in 21.67% of the patients. 61.67% of the patients were referred by primary care pediatrics. The TD was a median of 3.68 months (IQR 2.34 – 10.10), and the TTR was a median of 2.76 months (IQR 1.25 – 8.32). 11 patients (18.33%) did not present a diagnostic delay compared to 49 (81.66%) who were diagnosed with a delay of more than 2 months. The JIA subtype with the highest TD was polyarticular RF negative 9.26 months, but without finding significant differences between the different subtypes (p=0.523). No significant associations were observed between the delay in diagnosis and the epidemiological, clinical or analytical variables.

**Conclusion:** The TD in patients with JIA in our cohort is a median of 3.68 months, a time similar to other cohorts previously described at a European level, with RF polyarticular JIA being the one with the highest TD, probably due to the wide differential diagnosis that characterizes this subtype. No significant differences have been found that relate the diagnostic delay and the referral professionals or previous consultations to the emergency room or previous admissions. The retrospective design of the study and the non-inclusion of socioeconomic variables or rural population constitute important limitations of the study to be considered in future research.

**Patient Consent:** Yes, I received consent

**Disclosure of Interest**: None declared

### P011. Increasing incidence of juvenile idiopathic arthritis? a trend over 31 years in Southern Sweden

#### E. Berthold^1,2,3^, A. Dahlberg^2,4,5^, H. Tydén^1,3^, B. Månsson^1,3^, R. Kahn^1,2,5^

##### ^1^Skåne University Hospital, Lund and Malmö, ^2^Wallenberg Center for Molecular Medicine, ^3^Department of Rheumatology, Clinical Sciences Lund, Lund University, Lund, ^4^Helsingborg Hospital, Helsingborg, ^5^Department of Pediatrics, Clinical Sciences Lund, Lund University, Lund, Sweden

###### **Correspondence:** A. Dahlberg

**Introduction:** The incidences of chronic immune diseases are proposedly increasing(1), but whether this is applicable to juvenile idiopathic arthritis (JIA) is unestablished. Previous studies present conflicting results and reported incidence differs profoundly based on different study design and geographic regions(1-5).

**Objectives:** To investigate the incidence of JIA over a period of 31 years, using two population-based JIA cohorts.

**Methods:** Incident cases of juvenile arthritis 1980-2001 in the Swedish region Skåne (population 1 023 479 by 1980) were identified with registered International Classification of Diseases (ICD)-code of juvenile arthritis from both in- and outpatient care, through an ICD-code search at the local database at the regional center for pediatric rheumatology, Lund, and at the diagnosis register at the National Board for Health and Welfare (NBHW). Included ICD-codes were: 696.00, 712, 713.10-19, and 714.93 (ICD-8); 696A, 713B and 714 (ICD-9); and M08-M09 (ICD-10). To validate the diagnoses, medical records were reviewed. In total, 400 cases were confirmed 1980-2001, and to compare the incidences of JIA over time with rate ratios (RR), this new cohort of 400 cases was combined with the previously published population-based cohort of 251 validated JIA cases in Skåne 2002-10(6).

**Results:** The annual incidence of juvenile arthritis 1980-2010 was 9.9 (95% CI 9.2-10.7) per 100 000 children <16 years, increasing from 5.9 (4.5-7.5) in 1980-84 to 14.0 (12.1-16.2) in 2005-10 with a significant RR of 2.4 (95% CI 1.8-3.2) (Table 1). When comparing the rates from the 90’s to the rates after the millennium, they were significantly higher when comparing the rate in 2005-10 to the rate in 1990-94 and 1995-99 (RR 1.6 (1.2-2.1) and 1.4 (1.1-1.7) respectively). The incidence of JIA in the cohort of 2002-2010 was increasing from 12.1 in 2002-05 to 13.9 in 2006-10, however this increase was not significant (RR 1.2 (0.9-1.5)).

**Conclusion:** We show a significantly increasing incidence of JIA over a period of 31 years in two population-based cohorts, supporting the theory of increasing occurrence of chronic immune diseases. Although possibly missing a few mild outpatient cases in the 80’s and 90’s due to non-compulsory outpatient registration, most cases of JIA in the region are believed to be included since most cases during this period required inpatient care for treatment. To evaluate if an increasing incidence of JIA is a continuous trend, further studies are necessary.

**References:**

1. Sevelsted A, et al. Eur J Epidemiol. 2021;36(11):1179-85.

2. Krause ML, et al. Arthritis Rheumatol. 2016;68(1):247-54.

3. Shiff NJ, et al. Arthritis Care Res (Hoboken). 2019;71(3):413-8

4. Cardoso I, et al. Int J Environ Res Public Health. 2021;18(16).

5. Danner S, et al. J Rheumatol. 2006;33(7):1377-81.

6. Berthold E, et al. Arthritis Res Ther. 2019;21(1):218.

**Patient Consent:** Not applicable (there are no patient data)

**Disclosure of Interest**: None declared

**Table 1 (abstract P011).** Incidences of juvenile arthritis divided by lustrum

	Incident cases	Incidence (95% CI)	Rate ratio (95% CI)
1980-1984	60	5.9 (4.5-7.5)	1.0
1985-1989	76	7.7 (6.1-9.6)	1.3 (0.9-1.8)
1990-1994	92	8.8 (7.1-10.7)	1.5 (1.1-2.1)
1995-1999	114	10.5 (8.7-12.5)	1.8 (1.3-2.5)
2000-2004	121	11.1 (9.3-13.2)	1.9 (1.4-2.6)
2005-2010	183	14.0 (12.1-16.2)	2.4 (1.8-3.2)

Incidence rates are presented as the incidence of juvenile arthritis per 100 000 children. Rate ratios calculated with the cases in 1980-1984 as comparator

### P012. Interferon signature as possible new marker for the stratification of patients with JIA

#### L. De Nardi^1^, F. Rispoli^1^, S. Pastore^2^, A. Taddio^1,2^, A. Tommasini^1,2^

##### ^1^University of Trieste, ^2^Pediatrics, IRCCS “Burlo Garofolo” - Trieste, Trieste, Italy

###### **Correspondence:** L. De Nardi

**Introduction:** Interferon signature (IS) is a valuable instrument to quantify the expression of interferon-stimulated genes in the bloodstream, providing an indirect estimate of cells’ exposition to type I Interferon-mediated inflammation. Apart from interferonopathies, where the pivotal role of type I interferon (IFN) is recognized, dysregulation in IFN-I production and function has been observed also in some autoimmune diseases. An over-reactive IFN-I signaling pathway has been first described in Systemic Lupus Erythematosus (SLE) and Juvenile Dermatomyositis (JDM), leading to high levels of IS in such conditions. Unfortunately, there is no agreement in literature on the role of IS in other polygenic autoimmune diseases such as Juvenile Idiopathic Arthritis (JIA), whose inflammation can be dominated by other types of cytokines. Furthermore, the growing evidence of JIA heterogeneity encourages physicians to research new markers for integrating clinical and biological data, to identify subgroups of patients which could benefit from a specific therapeutical approach.

**Objectives:** This study aims to explore the clinical significance of IS among a cohort of patients with JIA, especially concerning its association with disease stratification and prognosis.

**Methods:** This is a consecutive case series conducted at the Institute for Maternal and Child Health “Burlo Garofolo” of Trieste, Italy. Pediatric patients between 6 months and 18 years old who accessed the Rheumatology Department of the Institute between May 2017 and December 2021 with a diagnosis of JIA (according to 2001 ILAR criteria) were recruited; patients with systemic JIA were excluded. For each patient the following variables were collected: age, sex, age at onset, family history, arthritis type, involved joints, JADAS-27 score, presence of enthesitis, tenosynovitis, uveitis, IS, erythrocyte sedimentation rate (ESR), C-reactive protein, immunoglobulins, anti-nucleus antibody and rheumatoid factor levels. Also information about previous and ongoing therapies were collected.

**Results:** A total number of 44 patients were recruited (35 F, 9 M). Subtypes of arthritis were distributed as follows: 19 polyarticular, 19 oligoarticular (of which 6 extended), 5 psoriatic (2 oligo- and 3 polyarticular). Twenty-seven patients had a disease which required 2^nd^ line therapy with any biological drug, while 17 showed disease remission on 1^st^ line medication. Out of 44 patients, 15 had a positive IS (considered if ≥ 3). This subgroup of patients did not show statistically significant higher frequency of tenosynovitis (p=0.31), enthesitis (p=0.52), uveitis (p=0.43) or cervical spine involvement (p=0.57). IS positivity was not associated with higher levels of JADAS score (p=0.17). However, an association between IS positivity and the number of involved joints was found (p=0.019). Notably, a correlation between IS positivity and polyarticular type was detected (including extended oligoarticular and polyarticular psoriatic type, p=0.013), along with a correlation between ESR values and IS frequency (p=0.010). Patients who required biological drugs, did not have significantly higher IS levels (p= 0.33). Finally, no correlation between IS and age at onset was found.

**Conclusion:** This study shows an association between the presence of IS positivity and the polyarticular involvement, along with a correlation between IS frequency and ESR levels. Even though these evidences are too limited to consider IS a novel biological marker for the stratification of JIA, we could speculate on the possible role of JAK inhibitors therapy for some refractory cases of JIA with elevated IS levels, in which they could be beneficial and safer. Further studies are needed to explore such clinical associations in a larger cohort of patients.

**Patient Consent:** Not applicable (there are no patient data)

**Disclosure of Interest**: None declared

### P013. The factors affecting the duration of remission of polyarticular JIA

#### E. Esen^1^, A. P. Kısaarslan^1^, S. Ö. Çiçek^2^, M. H. Poyrazoğlu^1^

##### ^1^Pediatric Rheumatology, Erciyes University Faculty of Medicine, ^2^Pediatric Rheumatology, Kayseri City Hospital, Kayseri, Turkey

###### **Correspondence:** A. P. Kısaarslan

**Introduction:** Polyarticular Juvenile idiopathic arthritis(polyJIA) is divided into seronegative (RF-) and seropositive (RF+) polyarticular JIA.

**Objectives:** In this study, we aimed to determine the factors affecting the duration of remission with or without treatment in polyarticular JIA patients.

**Methods:** The data of 88 patients diagnosed with polyarticular JIA according to the ILAR diagnostic criteria in the Erciyes University pediatric rheumatology were evaluated retrospectively. The factors affecting the duration of remission were analysed by using univariate cox regression analysis.

**Results:** Seventy(79.5%) patients were female, median age was 175.5(28-270) months,and median follow-up time was 45(4-238) months. Median joint involvement was 10(5-36). Rheumatoid factor positivity was in 11(12.5%), and anti-CCP positivity in 10(11.4%) patients. In our study, 82 (93.2%) patients had at least one large joint involvement with small joints, 8 (9.1%) patients had temporamandibular joint, and 11 (12.5%) patients had cervical vertebral involvements. 65 (73.9%) patients had acute phase reactant elevations at their first admission. Fifteen (17%) patients had accompanying diseases. Ten(11.4%) patients had uveitis. Sixty-three (71.6%) patients received systemic steroids, 22 (25%) patients received intra-articular steroid injection. All of our patientsterated with the disease-modifying drug, which was methotrexate. Sixty-five of them (73.9%) treated with at least one of the biological agents. At the last visit, 68 patients were in remission. JADI-A median score was 0(0-22) and JADI-E median score was 0(0-2). Fifty-one(58%) patients did not have articular sequelae,68 (77.3%) patients did not have extra-articular sequelae. The median time to remission with DMARD was 2(0-47) months. The median time to remission with biological agents was 14(0-50) months, and the median time to remission without medication was 0(0-96) months. The median total remission time (with treatment and without treatment) was 18 (0-114) months. The factors affecting the duration of remission analysed by using cox regression analysis are shown in table 1.

**Conclusion:** Our results show that steroid dose and duration did not affect the duration of remission periods. Because the total remission time without treatment was very short, we think that especially biological therapy should not be interrupted.

**Patient Consent:** Yes, I received consent

**Disclosure of Interest**: None declared

**Table 1 (abstract P013).** The factors affecting the duration of remission

Variable	HR	95.0 % CI	*p*
Gender	0.973	0.545-1.738	0.927
Age of diagnosis	1.003	0.998-1.008	0.205
RF positivity	1.182	0.580-2.405	0.646
Anti-CCP positivity	1.028	0.868-1.219	0.748
High AFR value in first admission	0.774	0.441-1.357	0.375
Number of involved joints	1.014	0.974-1.056	0.489
Large joint involvement	0.584	0.210-1.623	0.302
Temporamandibular joint involvement	1.336	0.567-3.146	0.508
Cervical involvement	1.444	0.652-3.198	0.365
Accompanying diseases	0.751	0.399-1.412	0.374
Uveitis	0.624	0.268-1.451	0.273
İntra-articular injection	0.627	0.354-1.111	0.110
Use of Biyolojical drug	0.067	0.007-0.594	**0.015**
Systemic Steroid treatment	0.828	0.493-1.390	0.475
Duration of systemic steroid treatment	1.003	0.970-1.036	0.875
Amount of systemic steroid (mg/m^2^ )	1.000	1.000-1.000	0.865
Duration of MTX	0.976	0.964-0.988	**0.000**
Duration of Biyolojic drug treatment	0.971	0.956-0.986	**0.000**

### P014. Improving sleep reduces pain in childhood arthritis; a crossover randomized controlled trial

#### S. Dover^1^, H. Clairman^1^, D. Beebe^2^, B. Cameron^1^, R. Laxer^1,3^, D. Levy^1,3^, I. Narang^1,3^, R. Schneider^1,3^, L. Spiegel^1^, S. Stephens^1^, J. Stinson^1,3^, G. Tomlinson^3^, S. M. Tse^1,3^, S. Weiss^1,3^, K. Whitney^1^, B. M. Feldman^1,3^

##### ^1^The Hospital for Sick Children, Toronto, Canada, ^2^Cincinnati Children’s Hospital, Cincinnati, United States, ^3^University of Toronto, Toronto, Canada

###### **Correspondence:** B. M. Feldman

**Introduction:** Childhood arthritis is a chronic childhood disease that is very prevalent and negatively impacts quality of life. It can be a source of discomfort and in refractory cases, despite modern biologic therapies, can lead to persistent pain and stiffness. Adolescents have poor sleep practice, and their poor sleep has been associated with negative outcomes, including pain. Poor sleep, combined with childhood arthritis, may lead to even worse health outcomes. Previous studies have shown a strong link between sleep duration and pain in children with childhood arthritis.

**Objectives:** The aim of the present study was to determine if longer sleep duration leads to reductions in self-reported pain when compared to restricted sleep duration in adolescents with childhood arthritis.

**Methods:** Adolescents between 12-18 years of age, with a minimum pain score of 1 on a 10cm visual analog scale, completed a 3-week sleep manipulation protocol involving a baseline week, followed by a restricted sleep (RS) condition (6.5 hrs in bed per night) and a health sleep (HS) condition (9.5 hrs in bed per night). We used a randomized crossover experimental design. Participants’ sleep was monitored at home via self-report and actigraphy. Pain was assessed up to 3 times daily using the iCanCope with Pain app. At baseline and the end of each sleep condition, participants completed validated questionnaires about pain interference, pain behaviour, daytime fatigue, and had their disease activity assessed by a trained clinician. We used Bayesian hierarchical models to estimate the effect of sleep duration on pain, as measured by iCanCope, as well as the effects on pain interference, pain behaviour, and disease activity.

**Results:** Participants (n=31) had a mean age of 15.0 ± 1.8 years (see Table 1 for basic demographic information). Participants averaged 1.4 (95% credible interval, CrI 1.2-1.6) more hours of sleep per night during the HS condition relative to the RS condition. Compared to the RS condition, participants were 40% (odds ratio 0.61, 95% CrI 0.39-0.95) less likely to report pain in the HS condition. There were no differences between the RS and HS conditions on pain interference, pain behaviour, daytime fatigue, or disease activity.

**Conclusion:** Adolescents with childhood arthritis improved their sleep duration, and this longer sleep duration resulted in a moderate reduction in pain. These findings complement prior correlational studies and confirm a causal relationship between reduced sleep duration and increased pain.

**Trial registration identifying number:** Clinicaltrials.gov registration: NCT04133662

**Disclosure of Interest**: None declared

**Table 1 (abstract P014).** Participant Demographic Information

Characteristic		
Female sex, n (%)	24 (77.4)	
Age at diagnosis of childhood arthritis (years), mean (SD)	7.3 (5.2)	
Arthritis Type, n (%)	Oligoarticular	5 (16.1)
	Polyarticular RF negative	13 (41.9)
	Polyarticular RF positive	3 (9.7)
	Enthesitis related arthritis	4 (12.9)
	Systemic arthritis	1 (3.2)
	Other	5 (16.1)

### P015. Acceptability of preliminary Arabic PGALS in patients with juvenile idiopathic arthritis

#### H. Ferjani^1^, E. Rabhi^1^, S. al mayouf^2^, D. Hadef^3^, L. Loucif^3^, W. Hamdi^1^, H. Foster^4^

##### ^1^Rheumatology, Kassab orthopedics institute, Ksar Said, Tunisia, ^2^pediatrics, King Faisal specialist hospital and research center, Riyadh, Saudi Arabia, ^3^pediatrics, Batna 2 University, Batna, Algeria, ^4^pediatrics, Population Health Sciences Institute, Newcastle upon Tyne, United Kingdom

###### **Correspondence:** H. Ferjani

**Introduction:** The paediatric Gait, Arms, Legs, and Spine (pGALS) is a quick, easy tool for evaluating musculo-skeletal problems in school-age children. It facilitates early recognition of joint problems and prompts referral to specialist center to optimize clinical outcomes. pGALS hasbeen shown to be practical and useful, with excellent acceptability. Its use was limited in Arabic-speaking countries because of the lack of an Arabic version.

**Objectives:** Under the umbrella of the musculoskeletal matter, we aimed to translate the English pGALS into Arabic form using the Delphi approach and to evaluate the acceptability of the preliminary version in children and their parents.

**Methods:** The original pGALS was translated by three native-speaker translators. The different propositions were mixed in a consensual way by a children’s musculoskeletal specialist. The version was validated according to the Delphi method. Delphi method is the consensus-building method, providing the consensual opinion of the experts.

For each translated item of the pGALS, the experts assessed the relevance using a scale ranging from 1 to 9 (not relevant-completely relevant). Then the median was calculated giving for each item the position of the group: disagree (if the median < 3), equivocal (median between 4-6) and agreement (median >7). The degree of the convergence with the group was assessed to clarify this result: the group’s opinion is consensual if 70% of the responses were within the range of the median. For the non consensual and no relevant item, the experts propose a comment to reformulate the sentence. The acceptability of the first form was evaluated using the visual analog scale in patients with juvenile idiopathic arthritis (JIA).

**Results:** Six experts from different Arabic-speaking countries (pediatricians and rheumatologists) were interviewed during 3 rounds by electronic survey individually and anonymously to validate the Arabic form. After each round: the median, consensus, and comments of every item are collected and a meeting with experts was held to analyze the results. During the first meeting, we were consensual and we had an agreement on 83 % of the items using the Arabic version (30 items were validated, and 6 items were reformulated. Then the forms were reformulated using the results of the preliminary rounds: opinions of the experts and their proposals during the last meeting). We were in agreement and we validated the remaining item during the second meeting. In the last round, we obtained a consensual preliminary version of the Arabic pGALS. After translation–back-translation, the Arabic pGALS was used in five pairs (child and parent) with JIA. The acceptability varied between 8 to 9, and all the questions and maneuvers were understood by the pairs.

**Conclusion:** After the consensual translation of the Arabic pGALS and evaluation of its acceptability in the small sample, the second step is to validate this form in a large paediatric population and to assess its sensibility and specificity of this form in screening musculoskeletal disorders in Arabic countries.

**Patient Consent:** Yes, I received consent

**Disclosure of Interest**: None declared

### P016. Quantifying cost impact of withdrawing biologic dmards in children with JIA

#### A. A. Florax^1^, M. J. Doeleman^2,3^, S. de Roock^2,3^, N. van der Linden^1^, E. Schatorjé^4,5^, G. Currie^6^, D. A. Marshall^6^, M. J. IJzerman^1^, R. S. Yeung^7^, S. M. Benseler^6^, S. J. Vastert^2,3^, N. Wulffraat^2,3^, J. F. Swart^2,3^, M. M. Kip^1,2^ on behalf of UCAN CAN-DU and UCAN CURE consortia

##### ^1^University of Twente, Enschede, ^2^University Medical Center Utrecht, ^3^Utrecht University, Utrecht, ^4^St. Maartenskliniek, ^5^Radboud University Medical Center, Nijmegen, Netherlands, ^6^University of Calgary, Calgary, ^7^ University of Toronto, Toronto, Canada

###### **Correspondence:** M. M. Kip

**Introduction:** The cost impact of withdrawing biologic DMARDs (bDMARDs) in JIA patients in clinically inactive disease is currently unknown.

**Objectives:** To quantify the difference in costs of hospital-associated care before and after withdrawing bDMARDs (discontinuing or tapering) in JIA patients <18 years old, after they achieved clinically inactive disease on bDMARDs.

**Methods:** Retrospective analysis of prospective data from electronic medical records of JIA patients at the Wilhelmina Children’s Hospital (Utrecht, the Netherlands), aged <18 years between 8 April 2011 and 8 April 2022, and treated with TNF-α bDMARDs, which were discontinued or tapered during this period. The hospital-associated resource use and associated costs during clinically inactive disease (i.e. pre-withdrawal) were compared to the costs within the first year after initiating withdrawal (i.e. post-withdrawal), grouped by discontinuing and tapering. The paired t-test was used to evaluate the significance of the cost difference between the pre- and post-withdrawal period. Unit prices were obtained from Dutch reimbursement lists, pharmaceutical price lists, and hospital price lists.

**Results:** Of the 55 JIA patients, 25 discontinued and 30 tapered bDMARDs. The mean annual costs of hospital-associated care per patient were €9,906 in the pre-withdrawal period (mean follow-up of 448 days) and decreased significantly to €5,633 in the post-withdrawal period (fixed follow-up of 365 days, p<0.05). The mean absolute difference in the entire study population is €-4,273 per patient per year (i.e. -43%), Table 1. The medication costs represented 80% and 60% of total mean annual costs in the pre- and post-withdrawal period, respectively. When distinguishing between withdrawal strategies, mean annual costs per patient reduced significantly from €9,670 to €4,338 in the discontinuation group (i.e. -55%, p<0.05), and from €10,103 to €6,712 in the taper group (i.e. -34%, p<0.05).

**Conclusion:** The mean annual costs of bDMARDs within the first year post-withdrawal period are significantly lower than the annual costs pre-withdrawal. In addition, abruptly discontinuing bDMARDs results in greater cost reductions, although there is substantial patient-level variation.

**Patient Consent:** Yes, I received consent

**Disclosure of Interest**: None declared

**Table 1 (abstract P016).** Mean annual hospital-associated costs of JIA (and 95% CIs) per patient during clinically inactive disease on bDMARDs (pre-withdrawal) and during the first year following withdrawal of bDMARDs (post-withdrawal, subdivided into discontinuing and tapering)

*Group and period*	*Total (n=55)* *pre-withdrawal*	*Total (n=55)* *post-withdrawal*	*Discontinuation (n=25)* *pre- withdrawal*	*Discontinuation (n=25)* *post-withdrawal*	*Taper (n=30)* *pre-withdrawal*	*Taper (n=30)* *post-withdrawal*
Total	**€9,906** **(€8,983 - €10,845)**	**€5,633** **(€4,711 - €6,568)**	**€9,670** **(€8078 - €11,270)**	**€4,338** **(€3,225 - €5,426)**	**€10,103** **(€9,032 - €11,185)**	**€6,712** **(€5,386 - €8,058)**
Medication	€7,940 (€7,041 - €8,840)	€3,381 (€2,682 - €4,070)	€7,537 (€5,857 - €9,217)	€2,128 (€1,246 - €2,999)	€8,275 (€7,453 - €9,090)	€4,426 (€3,560 - €5,296)
Consultation	€1,429 (€1,309 - €1,549)	€1,585 (€1,389 - €1,778)	€1,671 (€1,502 - €1,840)	€1,674 (€1,414 - €1,932)	€1,227 (€1,100 - €1,355)	€1,511 (€1,236 - €1,785)
Diagnostics*	€294 (€201 - €386)	€300 (€212 - €388)	€366 (€187 - €542)	€358 (€203 - €513)	€234 (€157 - €311)	€253 (€158 - €347)
Other**	€244 (€0 - €637)	€367 (€0 - €786)	€96 (€0 - €199)	€179 (€6 - €3,501)	€367 (€0 - €1,074)	€523 (€0 - €1,274)

* including laboratory and radiology tests

** including emergency department visit, hospitalisation (including daycare) and surgeries

### P017. Juvenile psoriatic arthritis - clinical characterization and differences from adult-onset disease

#### V. Fraga, A. Ana Catarina, S. Sousa, M. J. Santos

##### Rheumatology, Hospital Garcia de Orta, Almada, Portugal

###### **Correspondence:** V. Fraga

**Introduction:** Juvenile psoriatic arthritis (jPsA) is a category of juvenile idiopathic arthritis (JIA), clinically heterogenous.

**Objectives:** Characterize clinical features of jPsA and compare with adult-onset psoriatic arthritis (aPsA).

**Methods:** Children fulfilling the ILAR criteria for jPsA and adults fulfilling CASPAR criteria for PsA were included. Demographic and clinical data was retrieved from the Portuguese registry Reuma.pt. A cross-sectional descriptive analysis of jPsA followed by a comparison with aPsA was performed.

**Results:** A total of 11 jPsA and 132 aPsA were included. jPsA represented 8.9% of JIA patients: 8 girls (72.7%), mean age at disease onset 10.4 ± 3.7 years and at diagnosis 11.9 ± 3.9 years, all Caucasian. Five (45.5%) developed psoriasis before arthritis and in 2 patients, psoriasis followed the onset of arthritis. Family history of psoriasis in 1^st^ degree relatives was positive in 63.6%. The most frequent articular pattern was oligoarthritis (63.6%, n=7) and the most common musculoskeletal features were dactylitis (27.3%, n=3) and enthesitis (27.3%, n=3). Axial involvement was present in only one case. All jPsA patients were rheumatoid factor negative, six (54.5%) were ANA positive and none was HLA-B27 positive. Seven were treated with methotrexate, in combination with a biologic in four of them.

The mean age of aPsA onset was 40.5 ± 18.9 years, 51.5% (n=68) were males, and the most common joint pattern was polyarthritis (59.1%, n=78). Presence of psoriasis at diagnosis was significantly more frequent in aPsA than in jPsA (84,8% vs 45.5%; p-value= 0.01). A total of 77 patients were treated with a biologic, most frequently adalimumab (n=27), etanercept (n=21) and infliximab (n=15). Seventy-two patients were treated with csDMARDs alone or in combination with a biologic.

**Conclusion:** At disease onset, less than half of the jPsA had psoriasis, and the most common joint pattern was oligoarthritis. The presence of dactylitis, enthesitis, and a positive family history of psoriasis are helpful in establishing the diagnosis. Conversely, aPsA patients were more often male, with psoriasis at the time of diagnosis and with polyarticular disease. No differences were found concerning extra-articular manifestations.

**Patient Consent:** Yes, I received consent

**Disclosure of Interest**: None declared

**Table 1 (abstract P017).** Extra-articular manifestations in jPsA and aPsA

	jPsA %(n)	aPsA % (n)	p-value
**Psoriasis at diagnosis**	45.5 (5)	84.8 (112)	p=0.01
**Dactylitis**	27.3 (3)	30.2 (38)	p=0.572
**Enthesitis**	27.3 (3)	35.3 (44)	p=0.748
**Nail involvement**	18.2 (2)	42.9 (48)	p=0.197
**Axial involvement**	9.1 (1)	17.4 (23)	p=0.418
**Inflammatory bowel disease**	9.1 (1)	0 (0)	p=0.080
**Uveitis**	0 (0)	1.6 (2)	p=0.846

### P018. Validation of a lateral flow rapid test for adalimumab drug monitoring in patients with juvenile idiopathic arthritis

#### S. Fuehner^1^, A. Mureseanu^1^, S. de Roock^2^, J. F. Swart^2^, G. Simonini^3^, E. Marrani^3^, P. Rovero^4^, F. Real-Fernandez^4^, J. Weber^5^, R. Cotti^5^, K. Minden^6^, D. Foell^1^, H. Wittkowski^1^

##### ^1^Department of Paediatric Rheumatology and Immunology, University Hospital Münster, Münster, Germany, ^2^Department of Paediatric Immunology and Rheumatology, University Medical Centre Utrecht, Utrecht, Netherlands, ^3^Rheumatology Unit, Meyer Children Hospital, University of Florence, Florence, ^4^Interdepartmental Laboratory of Peptide and Protein Chemistry and Biology, Department of NeuroFarBa, University of Florence, Sesto Fiorentino, Italy, ^5^BÜHLMANN Laboratories AG, Schönenbuch, Switzerland, ^6^Charité University Medicine and Epidemiology Unit, German Rheumatism Research Centre, Berlin, Germany

###### **Correspondence:** H. Wittkowski

**Introduction:** Therapeutic drug monitoring in patients with juvenile idiopathic arthritis (JIA) could guide dose adjustments and treatment intervals and thereby further improve patient outcomes during treatment with biologic agents. Point-of-care tests become increasingly available for routine clinical care and provide the possibility of immediate therapy decisions at the outpatient clinic.

**Objectives:** We have tested Lateral Flow Immunoassays (LFIA) with a Point-Of-Care-Device (POC, Quantum Blue® Reader) to investigate whether they can be helpful in the context of therapy decisions and drug monitoring in patients with JIA.

**Methods:** We included 91 JIA patients from the biobank of the German multicenter inception cohort (ICON), 71 of whom received the anti-TNF biologic adalimumab and a control group of 20 patients treated with etanercept. From these patients, 148 serum samples (stored at -80°C) were identified to measure adalimumab levels with the BÜHLMANN Quantum Blue® Adalimumab rapid test (measuring range 1.3 - 35.0μg/ml) in at least one sample per patient.

Anti-drug antibodies (ADAs) were determined with the BÜHLMANN Quantum Blue® Anti-Adalimumab rapid test (measuring range 0.2 - 12μg_eq_/ml). Since this assay is described as drug sensitive, only samples with adalimumab levels below the detection limit (n = 41) were analyzed.

For external validation 10 selected samples were analyzed in a routine laboratory with an enzyme immunoassay (EIA). An additional cross-check was performed with 21 samples from two external cohorts. In these, adalimumab was measured by liquid chromatography-tandem mass spectrometry (LC-MS/MS) assay and assayed for ADAs by radioimmunoassay (RIA) or ELISA (LisaTracker).

**Results:** In our cohort, adalimumab levels above the detection limit were measured in 87 of 128 samples, from 57 out of 71 JIA patients treated with adalimumab. In 14 patients, although adalimumab treatment was documented, no adalimumab could be measured at any time point. The samples selected for external validation were measured in a routine laboratory with a correlation (Spearman r) of 0.93 (*p = 0.0012*), thus confirming the rapid test as highly reliable. Furthermore, the comparison of rapid tests with LC-MS/MS measured adalimumab in the external partners’ samples showed a correlation of 0.86 (*p = 0.0278*). In patients treated with Etanercept no interference occurred and no false positive result was measured using the LFIA.

All samples with adalimumab levels below the detection limit were then tested for ADAs and in only one of these samples, antibodies could be detected with the used rapid test. The external laboratory was able to measure ADAs positively in two out of 10 selected samples. This may indicate that the rapid test is less sensitive than the EIA method.

This trend was also observed in the cross-check measurements using the samples of the external partners. Here, the BÜHLMANN ADA test showed no false-positive result (positive predictive value of 100%) compared to the results measured with RIA and ELISA, but a negative predictive value of only 40% and 85%, respectively.

**Conclusion:** Adalimumab LFIA rapid test with a POC-Device can be a useful tool for drug monitoring in patients with JIA. The handling is simple and tests are time saving and yet reliable. In patients with undetectable adalimumab levels, a rapid ADA test may be helpful in interpreting the absence of adalimumab. None-detectable ADA levels according to POC-Device should be validated and/or confirmed with conventional laboratory testing.

**Patient Consent:** Yes, I received consent

**Disclosure of Interest**: None declared

### P019. Cutaneous disorders and systemic manifestations, the importance of the multidisciplinary approach

#### L. Gago^1,2^, M. H. Lourenço^1,2^, R. Pinheiro Torres^1,2^, M. Guedes^3^, C. Amaro^4^, M. Costa^1^, J. C. Branco^1,2^, A. F. Mourão^1,2^

##### ^1^Rheumatology, Hospital Egas Moniz, ^2^Centro de Estudos de Doenças Crónicas (CEDOC), Universidade Nova de Lisboa, ^3^Ophthalmology, ^4^Dermatology, Hospital Egas Moniz, Lisboa, Portugal

###### **Correspondence:** L. Gago

**Introduction:** Wooly hair nevus is a hair change, where there is a curly, hypopigmented patch of hair in a restricted area of the scalp. It may be associated with ophthalmologic, auditory, renal, cutaneos, cardiac and skeletal manifestations.

**Objectives:** The authors describe a case of a 4-year-old girl with woolly hair nevus and psoriatic arthritis associated with chronic anterior uveitis.

**Methods:** A 4-year-old girl presented to the dermathology department with confluent erythematous papules on plaques on her thighs, elbows, and knees, and a curly patch of hair that contrasted with her straight hair. No relevant family history. In addition, the patient had a history of frequent falls. She denied arthralgias, myalgias, dactylitis. She also denied visual acuity changes. After careful objective examination she was diagnosed with psoriasis and wooly hair nevus.

An ophthalmological screening was performed which detected the presence of bilateral chronic anterior uveitis, and treatment with topical corticotherapy was initiated.

Due to her history of frequent falls and bilateral uveitis she was referred to our department. On physical examination she presented with arthritis of the right knee, with no associated pain, no enthesitis, dactylitis or other swollen or painful joints. She also had no strength deficits.

Blood tests showed no elevation of inflammatory parameters. She tested positive for the antinuclear-antibodies, and negative for extractable nuclear antigens. She also tested positive for the human leukocyte antigen B27 (HLA-B27). An ultrasound of the right knee was performed with evidence of synovitis of this joint. An echocardiogram was also performed and did not show any alterations.

**Results:** Considering the presence of right knee arthritis, bilateral uveitis and psoriasis associated with positive HLA-B27, the diagnosis of Psoriatic Juvenile Idiopathic Arthritis was made and the patient was medicated with Methotrexate 7.5 mg/week and prednisolone 7.5 mg, with significant improvement of symptoms. Three weeks after starting therapy the patient had no arthritis or skin lesions, however, bilateral uveitis persisted and for this reason topical ocular corticosteroid therapy was maintained.

**Conclusion:** Woolly hair nevus may be associated with ophthalmological, auditory, renal, cutaneous, and musculoskeletal manifestations, which makes referral to different specialties extremely important. The ophthalmologic examination is always indicated in these children.

Psoriatic arthritis is rare in children , representing the adult form of spondyloarthritis, but axial involvement is uncommon. Joint involvement may be mono- or polyarticular, symmetrical or asymmetrical. About 30% of patients are HLA-B27 positive.

In this particular case, the ophthalmologic involvement may be related to both wooly hair nevus and psoriatic arthritis, since both conditions are associated with ocular pathology. Thus, early ophthalmologic screening is of utmost importance, for diagnosis and treatment at early stages of the disease, in order to avoid irreversible changes.

**Patient Consent:** Yes, I received consent

**Disclosure of Interest**: None declared

### P020. Biomarkers related to myeloid derived cells obtained at baseline and at 18-year follow-up in juvenile idiopathic arthritis. relation to remission status and inactive disease

#### M. Glerup^1^, C. Kessel^2^, D. Foell^2^, M. Høllsberg^1^, L. Berntson^3^, A. Fasth^4^, S. Nielsen^5^, E. Nordal^6^, V. Rypdal^6^, M. Rygg^7^, E. Arnstad^8^, S. Peltoniemi^9^, K. Aalto^10^, T. Herlin^1^ on behalf of the Nordic Study Group of Pediatric Rheumatology (NoSPeR)

##### ^1^Department of Paediatrics and Adolescent Medicine, Aarhus University Hospital, Aarhus , Denmark, ^2^Department of Pediatric Rheumatology and Immunology, University Hospital Münster, Münster, Germany, ^3^Department of Women’s and Children’s Health, Uppsala University, Uppsala, Sweden, ^4^Department of Pediatrics, Institute of Clinical Sciences, Sahlgrenska Academy, University of Gothenburg, Gothenburg, Sweden, ^5^Department of Pediatrics, Rigshospitalet, Copenhagen University Hospital, Copenhagen, Denmark, ^6^Department of Pediatrics, University Hospital of North Norway, Department of Clinical Medicine, UiT The Arctic University of Norway, Tromsø, Norway, ^7^Department of Clinical and Molecular Medicine, NTNU - Norwegian University of Science and Technology, Department of Pediatrics, St. Olavs Hospital, Trondheim, Norway, ^8^Department of Clinical and Molecular Medicine, NTNU - Norwegian University of Science and Technology, Department of Pediatrics, Levanger Hospital, Nord-Trøndelag Hospital Trust, Levanger, Norway, ^9^Clinic of Rheumatology, Helsinki University Hospital, Helsinki, Finland, ^10^Department of Pediatrics, New Children’s Hospital, Helsinki University Hospital, Pediatric Research Center, University of Helsinki, Helsinki, Finland

###### **Correspondence:** M. Glerup

**Introduction:** Several blood biomarkers have been suggested to track with disease activity in juvenile idiopathic arthritis (JIA) and facilitate prediction of clinical outcome.

**Objectives:** To evaluate whether levels of biomarkers of inflammation in serum from patients with JIA close to disease onset was related to disease activity at baseline and at long-term follow-up (FU).

**Methods:** Patients from the population-based Nordic JIA cohort study were recruited at disease onset from defined regions of Denmark, Sweden, Norway, and Finland between 1997-2000. Serum was obtained at baseline (within 6 months from disease onset) and at 18-yr FU. S100 proteins, and 14 other inflammatory markers (cytokines, chemokines) were determined ﻿by multiplexed bead array assay. Data acquisition and analysis were performed on a MAGPIX instrument using xPONENT v4.2 software (Luminex). The analyzing laboratory in Muenster was blinded for the patients’ clinical data.

**Results:** Of the 510 patients from the Nordic cohort, serum samples from 236 patients at baseline and 284 patients at 18-yr FU were analyzed. Of these, 150 patients had paired samples taken at both time-points. Median age at onset was 6.0 yrs (IQR 2.9-10.4) and 23.6 yrs (IQR 20.5-27.6) at last FU. Median JADAS10 at baseline was 5.0 (IQR 2.0-11.0) compared to 2.0 (IQR 0.0-6.4) at FU. Inactive disease at 18-yr FU was observed in 58% and remission off medication in 39.4% of the patients.

At baseline levels of IL-1b, IL-6, IL-10, IL-17, IL-18, and sCD25 ranged 1.4-5.5 times higher than at 18-Y FU (all p<0.001) whereas baseline levels for IL-12 and MPO was half the values obtained at FU (both p<0.001). Sampled at baseline IL-6, IL-12p70, GM-CSF and S100A12 correlated significantly with baseline JADAS71 (p<0.01), but IL-1b, IL-4, IL-13 and MMP-3 correlated only weakly (0.01

Patients with active disease at 18-yr FU had significantly higher levels of S100A9, S100A12, IL-1β, IL-6, IL-12p70, IL-13, MMP-3, and GM-CSF at baseline than patients with inactive disease at FU, but levels of TNFα, IL-4, IL-10, IL-17, IL-18, CCL-2, sCD25, and MPO were not significantly different. Patients who achieved remission off medication at 18-y FU had significantly lower levels of IL-1β, IL-12p70, IL-13, MMP-3 measured at baseline compared to the rest of the cohort.

**Conclusion:** Biomarkers of inflammation obtained within the first 6 months after JIA onset may complement the characterization of disease activity and may even contribute to future prediction models of long-term outcome.

**Patient Consent:** Yes, I received consent

**Disclosure of Interest**: None declared

### P021. Clinical patterns in psoriatic juvenile idiopathic arthritis in a pediatric rheumatology unit from a tertiary hospital

#### M. I. Gonzalez Fernandez^1^, B. Lopez Montesinos^2^, M. Marti Masanet^1^, L. Lacruz Perez^2^, I. Burgos Berjillos^2^, I. Calvo Penades^2^

##### ^1^Pediatric Rheumatology Unit, Medical Research Institute Hospital La Fe, ^2^Pediatric Rheumatology Unit, Hospital Universitario y Politécnico La Fe, Valencia, Spain

###### **Correspondence:** M. I. Gonzalez Fernandez

**Introduction:** Psoriatic Juvenile Idiopathic Arthritis (PsJIA) is reported to account for around 5% of Juvenile Idiopathic Arthritis (JIA). It is considered a heterogeneous entity, with observation of a bimodal distribution of age at the disease onset (as in JIA globally) and description of an early-onset and a late-onset form.

**Objectives:** To describe a cohort of patients with PsJIA from a paediatric rheumatology unit in a tertiary hospital.

**Methods:** Patients seen at our paediatric rheumatology unit between January 2014 and April 2022 who met PsJIA ILAR criteria and with at least 18 months of follow-up were included. Medical records were reviewed to collect demographic, clinical, laboratory, treatment and evolution data.

**Results:** 39 patients met Vancouver criteria for probable or definite Juvenile Psoriatic Arthritis, of which 34 (87%) fulfilled ILAR criteria for PsJIA. The other 5 patients were all female, ANA-positive, early-onset JIA patients with dactylitis who met probable Vancouver criteria (four of them with psoriasis in a second-degree relative and one with nail pitting).

Median (IQR) age at JIA onset was 2,78 (1,58-6,81) years. 71% were female. Median (IQR) follow-up was 11,45 (8,01-13,74) years. As expected, distribution of the age at JIA onset was not consistent with normality, resembling a bimodal distribution. Patients were divided into 2 subgroups according to the age at disease onset, group of early onset (<=6 years) with 25 patients and group of late onset (>7 years) with 9 patients. Female gender predominance was found in both groups: 18/25 (72%) in early onset and 6/9 (67%) in late onset.

With respect to arthritis, 12/34 (35%) patients had oligoarticular involvement at the beginning of the disease. 26/34 (76%) had polyarticular course: 21/25 (84%) in the early-onset group and 5/9 (56%) in the late-onset group. Only one patient presented sacroilitis, in the late-onset group. 19/34 (56%) had dactylitis and 5/34 (15%) enthesitis.

ANA were positive in 20/34 (59%) patients (68% early-onset group vs 33% late-onset group). HLA-B27 was positive in 5/34 (15%) patients (8% early-onset group vs 33% late-onset group). 9/34 (26%) patients had anterior uveitis, all in the early-onset group.

Nail pitting was observed in 15/34 (44%) patients. Psoriasis appeared in 23/34 (68%) patients over the course of follow-up: 16/25 (64%) in the early-onset group and 7/9 (78%) in the late-onset group. Psoriasis was diagnosed after the arthritis in all patients in the early-onset group and in 3/7 (43%) patients in the late-onset group (p<0,05 Fisher test). Median (IQR) time from JIA diagnose to psoriasis diagnose 6,04 (3,27-9,16) years.

Regarding treatment, all patients received MTX and 24/34 (71%) biological DMARDS. 12/34 (35%) patients switched to a second biologic drug and 2/34 (6%) patients switched to a third biologic agent (early-onset patients).

Median (IQR) time until clinical remission with treatment was 1,62 (1,32-2,46) years. At last visit, 23/34 (68%) patients where in clinical remission (5/34 (15%) off therapy).

**Conclusion:** In our cohort of PsJIA, the form of early onset is more represented, with higher tendency to ANA positivity and chronic anterior uveitis. Female gender predominance was observed in both groups. High percentage of patients were treated with biological agents, in relation with uveitis and high frequency of polyarticular course. Psoriasis diagnosis was made a median (IQR) of 6,95 (4,24-9,46) years after arthritis diagnosis in early-onset patients.

**Disclosure of Interest**: M. I. Gonzalez Fernandez Consultant with: Novartis, B. Lopez Montesinos: None declared, M. Marti Masanet: None declared, L. Lacruz Perez: None declared, I. Burgos Berjillos: None declared, I. Calvo Penades Consultant with: Novartis, Speaker Bureau with: Novartis, Sobi, Abbvie

### P022. The effect of nutritional status on disease activity and disease course in juvenile idiopathic arthritis and use of malnutrition screening tests

#### A. Günay^1^, Ö. F. Beşer^2^, A. Adroviç Yıldız^3^, S. Şahin^3^, M. Yıldız^3^, F. Haşlak^3^, A. Günalp^3^, Ö. Kasapçopur^3^, K. Barut^3^

##### ^1^Department of Pediatrics, ^2^Department of Pediatric Gastroenterology, Hepatology & Nutrition, ^3^Department of Pediatric Rheumatology, Istanbul University, Cerrahpaşa - Cerrahpaşa Medical Faculty, Istanbul, Turkey

###### **Correspondence:** K. Barut

**Introduction:** Juvenile idiopathic arthritis is the most common chronic, systemic, autoimmune connective tissue disease of unknown etiology in children. Malnutrition can be seen frequently during the course of chronic diseases. Depending on the severity of malnutrition, the course of chronic diseases may be adversely affected. For these reasons, it is emphasized that malnutrition screening tests should be routinely applied in chronic patients in order to detect nutritional status disorders that may occur during the course of chronic diseases. During the course of JIA, nutritional status disorders can often be encountered as a result of various complications or related to the severity of the disease and this may adversely affect the course of the disease. There are screening tests used to determine the risk of malnutrition. Pediatric Yorkhill Malnutrition Score (PYMS) and Screening Tool for Risk of Impaired Nutritional Status and Growth (STRONGKids) are two accepted tests.

**Objectives:** We aimed to determine the frequency of malnutrition in JIA and the possible malnutrition status that may develop in JIA patients with screening tests. Also we aimed to reveal the factors in patients with existing malnutrition and/or patients who will develop malnutrition.

**Methods:** JIA (n=150) and FMF (n=156) patients were included in the study. All physical examinations of the patients were performed, height and weight measurements were recorded with the scale and meter in the outpatient clinic, BMI was calculated, laboratory values ​​at the time of admission were examined, disease activities of JIA patients were determined according to JADAS and Wallace criteria. All patients’ malnutrition risk scores were determined by applying PYMS and STRONGKids malnutrition screening tests. Afterwards, the files of all patients were analyzed retrospectively. All patients were made an appointment again at least 3 months after the examination. Anthropometric measurements of each patient were repeated in the outpatient clinic, and their BMIs were calculated. Physical examinations of all JIA patients were performed, laboratory parameters were requested and the results were recorded, and the activity level of the disease was determined again.

**Results:** According to PYMS values, malnutrition risk is seen in 11.3% (n=17) of JIA group cases. According to STRONGKids values, malnutrition risk is observed in 9.3% (n=14) of JIA group cases. According to the two screening tests, there is no statistically significant difference in the subtypes, ages, age at onset of disease, diagnosis age, drugs used, duration of drugs used and total doses of prednisolone used according to malnutrition risk. Although it is not statistically significant, it is noteworthy that the probability of malnutrition is higher in polyarticular JIA and enthesitis-related arthritis subgroups compared to other subgroups. According to PYMS and STRONGKids; JADAS applied at the time of admission and at least 3 months later and Wallace applied at the time of admission, the rate of being active was found to be statistically significantly higher than those without the risk of malnutrition.

**Conclusion:** Our study indicates the importance of determining the risk of malnutrition in this patient group by showing that the frequency of disease activity increases in patients at risk for malnutrition in JIA cases. It is emphasized that patients in polyarticular and enthesitis-related arthritis subgroups, whose prognosis is worse than other subgroups, should be followed more closely in this regard.

**Disclosure of Interest**: None declared

### P023. A10-year inception cohort of mexican-mestizo patients with juvenile idiopathic arthritis (JIA) from 3 tertiary centers in Mexico City. A Real-World Evidence (RWE), based on real world data from routine clinical assessment

#### R. Gutiérrez Suárez

##### Pediatric Rheumatology, Shriners Hospital of Mexico City, Mexico, Mexico

###### **Correspondence:** R. Gutiérrez Suárez

**Introduction:** Cohort studies can provide useful access to RWE which reflects the reality of daily clinical practice. RWE is becoming highly valuable and complementary to randomized clinical trials (RCT) in the generation of clinical evidence due to their high external validity, better generalisability, time and resource-efficiency and the possibility of long term surveillance.

**Objectives:** To evaluate propectively several disease outcome measures, in a 10-year period inception cohort of Mexican-Mestizo patients JIA, with the aim of generating real world diagnostic, therapeutic and prognostic evidence of this patients.

**Methods:** A10-year inception cohort was designed and conducted in three tertiary referal centers in Mexico City. Incident and prevalent JIA Mexican-Mestizo patients according to ILAR criteria were included in the cohort since its inception on January, 2012; and evaluated clinically and with laboratories at 12, 26 and 54-weeks periods and thereafter at year: 2, 3, 5, 7.5 and 10. Different demographical (referal time, time to adequate diagnosis and treatment, family income, parents education and social security); clinical (MD-centered measures: MD-VAS for disease activity and joint count; and parent/patient-centered measures: VAS of well-being and pain; disease activity (JADAS-71), functional capacity (CHAQ), articular and extra-articular damage (JADI-A and JADI-E); criteria for minimal disease activity (MDA), remission with (RWT) and without treatment (RWOT)); laboratory (RF, ESR CRP, antinuclear antibodies and HLA-B27) and therapeutic (NSAID’s, Steroids, Non biological and biological drugs: time and availability) variables were collected. Descrptive and some inferential statistics were performed for the 7-JIA subtypes according ILAR

**Results:** A total of 265 (60 % female) Mexican-Mestizo JIA patients according with ILAR criteria were included in the inception cohort. Age at onset (median ± IR); 6.5 (3-16) years; referal time (years): 2.5 (0.1-7.2); time to adequate diagnosis (years): 2.8 (0.1-7.5) time to adequate treatment; (years): 2.8 (0.2-7); family income per month; 300 USD (50-1100 USD). There were several differences between different subtypes in terms of disease activity (JADAS-71) functional capacity (CHAQ), articular and extra-articular damage (JADI-A and JADI-E). Also a high frequency of oligoarhritis patients complete criteria for minimal disease activity (MDA) and remission with (RWT) and without treatment (RWOT)): Patients with enytehsitis related arthritis and polyarthritis RF (+) were unable in a high proportion of patients to complete MDA or RWT and without treatment RWOT. Biological availabilty for treatment (4% of patientes in the inception cohort) Principally TNF inhibitors were used.. Time for Biological treatment (years): 1.5 (0.3-10). a large proportion of patients in RF (+) polyarthritis required combination or 2 or 3 non biological drugs related to the biological avaliability.

**Conclusion:** RWE reflects the reality of daily clinical practice and is a complement for RCT in the generation of clinical evidence. This studies enables research into the general quality of pediatric Rheumatogy care and provide insights into a broader optimization of pediatric Rheumatogy care, refined therapeutic strategies for patient subgroups as well as avenues for further research in pediatric Rheumatology.

**Patient Consent:** No, I have not receive consent

**Disclosure of Interest**: None declared

### P024. Validation of the parent global assessment as a quality of life measure in juvenile idiopathic arthritis: results from reacch-out

#### K. Oen^1^, K. Toupin-April^2^, B. M. Feldman^3^, R. A. Berard^4^, C. M. Duffy^5^, L. B. Tucker^6^, J. Tian^7^, D. G. Rumsey^8^, J. Guzman^6^ on behalf of ReACCh-Out Investigators

##### ^1^Department of Pediatrics and Child Health, University of Manitoba, Winnipeg, ^2^School of Rehabilitation Sciences and Department of Pediatrics, University of Ottawa, Ottawa, ^3^Pediatrics and Medicine, University of Toronto, Toronto, ^4^Pediatrics, London Health Sciences Centre, London, ^5^Pediatrics, Children’s Hospital of Eastern Ontario and University of Ottawa, Ottawa, ^6^Pediatrics, BC Children’s Hospital and UBC, Vancouver, ^7^Statistics and Actuarial Science, Simon Fraser University, Burnaby, ^8^Pediatrics, Stollery Children’s Hospital and University of Alberta, Edmonton, Canada

###### **Correspondence:** J. Guzman

**Introduction:** The Juvenile Idiopathic Arthritis (JIA) parent global assessment (parent global) is a visual analogue scale anchored by the words 0 very well and 10 very poor, and headed by the instruction: “Considering all the ways that arthritis affects your child, rate how your child is doing by placing a mark on the line.” Despite its extensive use there has been no formal conceptualization or validation of the parent global, resulting in uncertainty as to what it measures. We hypothesized that the parent global is an assessment of a child’s health as affected by arthritis, and should be considered a disease-specific health-related quality of life (HRQoL) measure.

**Objectives:** To 1) validate the parent global as a HRQoL measure, 2) evaluate measurement properties of accepted HRQoL measures relative to those of the parent global, and 3) assess causal pathways determining parent global scores.

**Methods:** Data from the Research in Arthritis in Canadian Children emphasizing outcomes (ReACCh-Out) cohort were used. Measurement properties were assessed in 344 patients at enrolment and 6 months later. Causal pathways were tested by structural equation modelling to understand root causes and mediators leading to parent global scores.

**Results:** Construct validity was supported by moderate to high Spearman correlations (0.53-0.70) of the parent global with the Juvenile Arthritis Quality of Life Questionnaire (JAQQ), Quality of My Life health scale (HRQoML), Pediatric Quality of Life Inventory (PedsQL)-Parent, and Child Health Questionnaire (CHQ)-Physical; and lower correlations (0.14-0.49) with disease activity measures (physician global assessment of disease activity (PGADA), active joint count, erythrocyte sedimentation rate(ESR)). Responsiveness of the parent global to improvement according to parent ratings (0.51) was acceptable and within the range (0.32-0.71) of that of other measures. Reliability estimates and measurement errors for all measures were unsatisfactory, likely due to the prolonged time between assessments. Causal pathways for the parent global matched those previously reported for HRQoML [1].

**Conclusion:** Our results support the parent global as a valid measure of HRQoL. If confirmed, the findings of previous studies using this measure and its role in JIA core sets and composite measures should be re-interpreted in this light.

[1] K Oen et al, Causal Pathways to Health-Related Quality of Life in Children with Juvenile Idiopathic Arthritis: Results from the ReACCh-Out Cohort. Rheumatology (Oxford) 2021;60(10):4691-4702

**Disclosure of Interest**: None declared

**Table 1 (abstract P024).** Spearman correlations of parent global with recognized HRQoL measures and other JIA measures at study enrolment

	Active joints	PGADA	ESR	JAQQ	HR-QoML	CHQ-Physical	CHQ-Psychosocial	PedsQL-Parent
**Parent global**	0.34	0.41	0.23	0.63	0.53	0.64	0.31	0.58
**JAQQ**	0.43	0.43	0.24	1	0.51	0.70	0.49	0.69
**HRQoML**	0.27	0.29	0.17		1	0.49	0.31	0.48
**CHQ-Physical**	0.36	0.40	0.29			1	0.35	0.63
**CHQ-Psychosocial**	0.18	0.22	-0.09				1	0.53
**PedsQL-Parent**	0.23	0.28	0.17					1

### P025. Juvenile psoriatic arthritis: a case report

#### A. Harbi, N. Balhoudi, B. Ibrahim, H. Mejaouel

##### Pediatric department, Medical School, Kairawan , Tunisia

###### **Correspondence:** A. Harbi

**Introduction:** Psoriatic arthritis is a heterogeneous entity defined by the association of psoriasis and arthritis with two forms: one more frequent in girls with an age of onset of about 6 years, close to oligoarthritis with a risk of uveitis, the other later, around 11-12 years, more frequent in boys, close to spondylarthropathies. It is an autoimmune inflammatory disease with hyperproduction of pro-inflammatory cytokines, particularly TNF alpha.

**Objectives:** The objective of our case study is to describe a rare and interesting case of Juvenile psoriatic arthritis .

**Methods:** We describe a clinical case of a child aged 13 years, complete vaccination according to age with a history of myositis post viral infection 5 years ago. Currently he presents walking disorder for 3 weeks, spinal pain, diffuse polyarthralgia with functional repercussions and interruption of his education.

The muscular testing globally at 3+ but it is generated by the pain, X-ray of the painful joints: without abnormalities with an ENMG having shown no myogenic attack nor motor or sensory deficit with a cerebral-medullary MRI without abnormalities. Ultrasound of the joints: discrete effusion of the knees bilaterally .Biology: antinuclear antibodies, antiAMAM2 rheumatoid factor, anti LKM, anti SLA LC1: negative, thyroid check-up correct with the fundus showing no uveitis.

On skin examination, he has erythematous scaly patches on 2 knees with post-inflammatory hypopigmentation on the elbows, nails punctuated with thimbles, distal interphalangeal attack, oral mucosa: no abnormalities: it is psoriasis, hence the need for local treatment. It is a case of juvenile psoriatic arthritis and is treated with NSAIDs, with a good outcome.

**Results:** Juvenile idiopathic arthritis is considered an autoimmune disease, which may result from an abnormal immunological response triggered by environmental factors such as infection or trauma in a genetically susceptible individual. Psoriatic arthritis is less than 10% of JIA.

In more than 60% of cases, arthritis precedes the skin manifestations of psoriasis, sometimes by several years, and usually presents as an asymmetric oligoarthritis. Monoarthritis is relatively common initially, with isolated involvement of the knee and small joints of the hands and feet.

The Edmonton diagnostic criteria are used. Psoriatic arthritis is characterized by the presence of arthritis and psoriasis, or failing that, by arthritis accompanied by at least two of the following signs: - Dactylitis - Nail staining or onycholysis - Family history of psoriasis in a first degree relative.

Management should be done in a specialized setting, in the context of a pediatric rheumatology consultation. As with other JIAs, treatment is based on non-steroidal anti-inflammatory drugs (NSAIDs). NSAIDs are indicated as the first-line treatment; they are prescribed in sufficient and continuous doses. They may be combined with intra-articular injections of delayed corticoids. Background treatment involves methotrexate and biotherapies.

**Conclusion:** Psoriatic arthritis is characterised by the presence of arthritis associated with psoriasis. Non-steroidal anti-inflammatory drugs have traditionally been the main treatment for all forms of juvenile idiopathic arthritis (JIA) and other paediatric rheumatic diseases.

**Trial registration identifying number:** .

**Patient Consent:** Yes, I received consent

**Disclosure of Interest**: None declared

### P026. Evaluation of comorbidities in patients with juvenile idiopathic arthritis: a cross-sectional single center study

#### F. Haslak, V. Guliyeva, B. Hotaman, C. Duman, M. Yildiz, A. Gunalp, A. Aliyeva, A. Adrovic, S. Sahin, K. Barut, O. Kasapcopur

##### Pediatric Rheumatology, Istanbul University-Cerrahpasa, Cerrahpasa Medical Faculty, Istanbul, Turkey

###### **Correspondence:** M. Yildiz

**Introduction:** Juvenile idiopathic arthritis (JIA) is the most common rheumatic disease in childhood. The most common subtype is the oligoarticular one. While autoimmunity is generally responsible for the pathogenesis of subtypes other than systemic, evidence of autoinflammation is usually pointed out in the pathogenesis of the systemic subtype. Although there are previous studies evaluating JIA patients in terms of other accompanying autoimmune diseases, psychiatric diseases, and cardiovascular diseases, there is a lack of data in the literature to evaluate all comorbidities together.

**Objectives:** We aimed to evaluate all the comorbidities of our JIA patients and compare the results between those with systemic JIA and those with other subtypes.

**Methods:** Among the patients with JIA under 21, who were admitted for the routine control between September 2020 and November 2020 were included in the study. Those with additional rheumatic diseases other than JIA and those with less than six months of follow-up duration were excluded from the study. Data were obtained from face-to-face interviews and their medical records cross-sectionally during these three months.

**Results:** The study included 459 participants (Female: 62.1%) (Systemic JIA group: 57, Non-systemic JIA group: 402). The median age was 12.87 (1.53-20.95) years, and 36.8% (n=169) of the patients were under biologic therapy. About one-third of the patients (n=155) had at least one non-rheumatic disease. The most common comorbidities were allergic rhinitis (n=38, 8.27%), attention deficit-hyperactivity disorder (n=35, 7.62%), atopic dermatitis (n=28, 6.1%), allergic asthma (n=14, 3.05%) and migraine (n=10, 2.17%). While atopic diseases were seen in 16.5% (n=76) of the patients, autoimmune disease frequency was 3.26% (n=15). Although the incidence of autoimmune disease and atopic disease was not significantly different in systemic and non-systemic JIA groups, no autoimmune disease was detected in any patient in the systemic JIA group.

**Conclusion:** The increased comorbidity burden in JIA patients brings problems of care, follow-up, treatment adherence, and decreased quality of life. This study showed that JIA patients need to be followed up with a multidisciplinary approach. Although autoimmunity was held responsible for the pathogenesis of most subtypes of JIA, it was remarkable that the most common comorbidities were atopic diseases, not autoimmune. On the other hand, none of the detected autoimmune diseases were in the systemic JIA group, and this finding was compatible with the idea of autoinflammation rather than autoimmunity is responsible for the pathogenesis of systemic JIA.

**Patient Consent:** Yes, I received consent

**Disclosure of Interest**: None declared

### P027. Neutrophils extracellular traps formation may serve as a biomarker for disease activity in oligoarticular juvenile idiopathic arthritis

#### M. Heshin-Bekenstein^1,2^, S. Baron^2,3^, G. Schulert^4^, A. Shusterman^3^, R. Shukrun^2,3^, Y. Binenbaum^2,3^, R. Elhasid^2,3,5^

##### ^1^Pediatric Rheumatology, Tel Aviv Medical Center, ^2^Sackler School of Medicine, Tel Aviv University, ^3^Pediatric Hemato-Oncology Research Laboratory, Tel Aviv Medical Center, Tel Aviv, Israel, ^4^Division of Rheumatology, Cincinnati Children’s Hospital Medical Center, Cincinnati, OH, United States, ^5^Department of Pediatric Hemato-Oncology, Tel Aviv Medical Center, Tel Aviv, Israel

###### **Correspondence:** M. Heshin-Bekenstein

**Introduction:** Juvenile idiopathic arthritis (JIA) is the most common chronic rheumatic disease in children, causing significant morbidity. Despite the dramatic improvement in treatment, many patients do not achieve complete remission, and biomarkers for subclinical disease, flares and response to treatment are lacking. Neutrophils and neutrophil extracellular traps (NETs) were revealed to partake in pathogenesis of autoimmune and inflammatory conditions, but their role in JIA pathogenesis and as biomarkers for JIA disease activity are largely unexplored.

**Objectives:** In this study, we aimed to characterize the neutrophil enzymatic activity and NETs in oligoarticular and polyarticular JIA and explored its association with disease activity.

**Methods:** Neutrophils from 7 patients with oligoarticular and RF negative polyarticular JIA were isolated at time of diagnosis and after glucocorticoid intraarticular injection with or without DMARD treatment. Enzymatic activity of neutrophil granular enzymes were monitored by colorimentry, while NET formation was assessed using confocal fluorescence microscope.

**Results:** In oligoarticular JIA patients (n=4), all disease activity parameters and neutrophil function-related parameters decreased following intraarticular steroid injection. However, only three parameters exhibited statistically significant decline, including Physician Global Assessement (PhGA), Juvenile Activity Disease Activity Score-10 (JADAS-10) and clinical JADAS-10 (cJADAS-10). Importantly, NET formation was strongly positivily correlated with the cJADAS-10 at all time points (R=0.93, p=0.007). In polyarticular JIA patients (n=3), neither clinical disease activity parameters, the CRP, or the neutrophil function-related parameters showed consistent and significant decreases after steroid injections. Similarly, there was no significant correlation was found between NETs formation and cJADAS-10 in polyarticular JIA patients.

**Conclusion:** This is the first study exploring the link between NETs formation and JIA activity. In this pilot study we demonstrated a statistically significant linear correlation between cJADAS-10 and NETs formation in oligoarticular but not in polyarticular JIA patients. Hence, we suggest that NETs might play a role in JIA activity and may serve as a putative biomarker for treatment response. Further work is needed to validate these initial results and determine dynamics of NETosis in JIA.

**Patient Consent:** Yes, I received consent

**Disclosure of Interest**: None declared

### P028. Functional state of the right ventricle of the heart in adolescents with juvenile idiopathic arthritis

#### T. Holovko^1,2^, N. S. Shevchenko^1,2^, L. Bogmat^1,2^, V. Nikonova^1^

##### ^1^Department of rheumatology and comorbid states, SI “Institute for Children and Adolescents Health Care of the NAMS of Ukraine”, ^2^Department of pediatrics № 2, V.N.Karazin Kharkiv national university, Kharkiv, Ukraine

###### **Correspondence:** T. Holovko

**Introduction:** The relevance of cardiovascular complications in patients with rheumatoid arthritis remains. This is due to the high frequency of vascular accidents (myocardial infarction and stroke) against the background of atherosclerotic vascular lesions, as well as the development of cardiopathy with the formation of heart failure.

The development of chronic heart failure is manifested by a violation of the left ventricle of the heart and, above all, a decrease in its systolic function. The state of the right ventricle is not characterized. However, it is known that the functioning of the left and right ventricles of the heart is closely interconnected due to their common blood supply, the anatomical structure of muscle fibers, the common interventricular septum, pericardium, and innervation.

**Objectives:** To study the systolic and diastolic function of the right ventricle (RV) of the heart in adolescents with juvenile idiopathic arthritis (JIA).

**Methods:** 53 patients with polyarticular JIA, mostly girls (69.82%), mean age 13.36±0.39 years, disease duration 68.29±5.67 months were examined. All patients were on basic therapy with methotrexate at a dose of 10-15 mg/m2 of body surface, the average dose was 11.74 ± 0.37 mg/m2, the duration of treatment was 44.64 ± 5.17 months. The obtained results were compared with the indicators of healthy peers comparable in sex and age (54 adolescents, mean age 14.63 ± 0.29 years), who were in the control group.

The study program included an ultrasound study of the morpho-functional state of the heart with Doppler echocardiography using a LOGIO V2 General Electric device (USA), a 3Sc-RS sensor. The systolic function of the right ventricle was characterized by indicators: ejection fraction (EFF), stroke volume (SV), right ventricular minute volume (RV). To assess the diastolic function of the right ventricle, the maximum flow rate in the early diastolic filling phase (E), the flow rate in the late diastolic flow phase (A), their ratio (E/A), the flow rate deceleration time in the early diastolic filling phase (DT) and isovolumetric relaxation time (IVRT).

**Results:**

**Conclusion:** The study of RV systolic function in patients with JIA showed a significant decrease in RVF compared with adolescents in the control group, and RV stroke volume and RV minute volume were significantly higher than in healthy children.

Thus, in adolescents with JIA, there is a decrease in the systolic function of the right ventricle, which is accompanied by an increase in the strength of its contraction. There is also a significant increase in the rate of blood flow during the period of late diastolic flow, which indicates the inclusion of cardiac compensatory mechanisms to ensure proper intracardiac hemodynamics.

**Disclosure of Interest**: None declared

**Table 1 (abstract P028).** Morphological and functional parameters of the right ventricle of the heart

Index	Main group, n = 53	Control group, n = 54	significance of differences
EFrv %	42,18 ± 1,53	58,51 ± 1,77	p < 0,001
SVrv, ml	14,33 ± 1,47	7,80 ± 0,51	p < 0,001
MV, l/min	1,12 ± 0,12	0,51 ± 0,03	p < 0,001
DTrv, с	683,46 ± 33,86	733,66 ± 50,37	p ≥ 0,05
IVRTrv, с	210,99 ± 10,67	212,75 ± 14,59	p ≥ 0,05
peak Еtv, m/s	0,67 ± 0,05	0,62 ± 0,03	p ≥ 0,05
Peak Atv, m/s	0,40 ± 0,06	0,34 ± 0,02	p < 0,001
Е/А, c.u.	1,89 ± 0,0	1,87 ± 0,02	p ≥ 0,05

### P029. Α2-fraction and haptoglobin as biomarkers for disease activity in oligo- and polyarticular juvenile idiopathic arthritis

#### L. Zeller^1^, P. Tyrrell^2^, S. Wang^2^, N. Fischer^1^, J.-P. Haas^1^, B. Hügle^1^

##### ^1^German Center for Pediatric and Adolescent Rheumatology, Garmisch-Partenkirchen, Germany, ^2^Department of Statistical Sciences, Institute of Medical Science, Toronto, Canada

###### **Correspondence:** B. Hügle

**Introduction:** Unlike in adult rheumatology, in most forms of juvenile idiopathic arthritis (JIA) no reliable biomarkers currently exist to assess joint and disease activity. However, electrophoresis is frequently changed in active juvenile arthritis.

**Objectives:** The objective of this study was to evaluate the α2-fraction of serum electrophoresis and its main components as biomarkers for JIA, categories extended/persistent oligoarthritis and seronegative polyarthritis, in comparison with the conventionally used erythrocyte sedimentation rate and C-reactive protein.

**Methods:** Serum samples and clinical data from 181 patients with JIA were collected. Serum electrophoresis and α2-fraction and its components were determined using standard methods. Relationship between calculated α2-fraction of serum electrophoresis (CA2F) and its components, acute-phase parameters and cJADAS27 was assessed using Pearson’s correlation coefficient and linear regression modelling, adjusting for confounding effects. Results were confirmed in a second cohort with 223 serum samples from 37 patients, using a mixed model to account for repeated measures.

**Results:** Compared to ESR and CRP, CA2F showed higher correlation to cJADAS27, in particular for persistent oligoarthritis. Of the three components of the α2-fraction, haptoglobin showed the highest correlation to cJADAS27. Regression analysis demonstrated higher ability to predict cJADAS27 for CA2F, and especially for haptoglobin as a component thereof, than for CRP and ESR.

**Conclusion:** Compared to conventional methods, α2-fraction of serum electrophoresis and specifically, haptoglobin show higher correlations with disease activity in common subtypes of JIA, representing excellent candidates as biomarkers for disease activity. Further studies are necessary to determine diagnostic value and correlations in other subtypes.

**Patient Consent:** Yes, I received consent

**Disclosure of Interest**: None declared

### P030. Liver stiffness in low-dose methotrexate use in JIA patients

#### V. Iacomi, N. Revenco, R. Eremciuc

##### Pediatrics, SUMPh “Nicolae Testemitanu”, Chisinau, Moldova, Republic of

###### **Correspondence:** V. Iacomi

**Introduction:** Evaluation of methotrexate treatment results in JIA patients is challenging in the of absence of validated predicting models or screening methods. The disease activity score is the main reference value in monitoring the therapeutic effects. Low-dose treatment may involve the liver damage that expresses the outcomes.

**Objectives:** Assessment of the interdependence of JIA disease activity score and the degree of liver fibrosis in children that use low-dose methotrexate.

**Methods:** Children with JIA who received low-dose methotrexate at least 6 months underwent evaluation by transient unidimensional liver elastography with the M probe. The data results were adjusted according to the EFSUMB guidelines.

**Results:** Out of 68 JIA who were examined in our clinic, only 50 eligible patients have performed liver elastography. A sample of 38 (76%) were confirmed with the liver stiffness median greater than 4,8 kPa after 24 weeks of methotrexate use (p < 0,005). We noted a substantial decrease in DAS28, up to 1,09 compared to 2,87 (p <0,0005), in children with high liver stiffness. We also noted a decrease in liver stiffness after 144 weeks of methotrexate administration and a remaining low disease activity score for this sample.

**Conclusion:** Low-dose methotrexate treatment has an impact on the liver stiffness in JIA. This is the toxicity outcome. The use of liver elastography as a method of its evaluation, allows to monitor it in compliance with the disease activity.

**Patient Consent:** Not applicable (there are no patient data)

**Disclosure of Interest**: None declared

### P031. A clinical case of a patient with down syndrome and juvenile idiopathic arthritis: difficult to diagnose, difficult to treat, difficult to manage severe comorbid problems

#### K. Zarina, V. Matkava, N. Irina, B. Leonid, A. Svetlana

##### Pediatric Department, V. A. Nasonova Research Institute of Rheumatology, Moscow, Russian Federation

###### **Correspondence:** N. Irina

**Introduction:** There is a lot of difficulties in the medical management of patient with Down syndrome and joints involvement on the stage of diagnosis, choice of therapy and resolving numerous problems associated with comorbid conditions, including COVID19 infection.

**Objectives:** To describe the difficulties of caring for a patient with Down’s syndrome and multiple comorbidities who has had a COVID-19 infection.

**Methods:*Case report***: Girl N., 9 years old was admitted to our observation in November, 2021. Diagnosis: Down syndrome; congenital heart defect, high pulmonary hypertension. Grade 2A circulatory insufficiency. Dislocation of left patella, subluxation of right patella Since 2018 she had pain in the wrist, knee, ankle joints and the diagnosis of Down’s arthropathy was suspected. Juvenile idiopathic arthritis (JIA) was diagnosed in March 2020, methotrexate (MTX) was started with insufficient effect. Persistent tendency to leukopenia limited the regular therapy.

**Results:** The patient had swelling and hypermobility of all joints, inadequate assessment of the patient’s subjective pain perception. There were no laboratory activity, ANA, RF were negative. A Whole-body MRI (WB-MRI) scan detected polyarthritis with synovitis and areas of osteitis, which confirmed the inflammatory origin of the disease. Due to inefficacy of MTX therapy, leukopenia, severe multimorbidity, planned orthopedic surgery and a psychological barrier for use of injections, oral JAK inhibitor Tofacitinib (TOFA) 5 mg BID was chosen. Good results were achieved rapidly. Open reduction of the left patella was performed in January 2022. The early post-operative period was uneventful. Before discharge from the surgical hospital on 1 February a PCR revealed COVID-19 infection, up to 15% lung involvement according to CT, persistent hypercoagulability (D-dimer >10000). Girl was hospitalized in infectious department. She was treated by monoclonal antibodies, Heparin with further switched to Warfarin 5mg/d under INR control (target 2-3). This choice was apparently made due to a previously performed cardiac surgery. Warfarin was continued but adequate INR monitoring was not performed. The spokes were removed at the outpatient orthopedic clinic on 30 March as was planned. The surgeon was not informed about the warfarin treatment. Bleeding from the post-operative wound and haemarthrosis of the left knee were developed a day later. The girl admitted to the ICU in a severe condition with haemorrhagic syndrome, great anaemia, no coagulation. Warfarin an TOFA were withdrawn, blood components transfusion and aspiration of blood from the left knee were done. The condition was improved. In May 2022 the girl was admitted to our center with the clinical picture of JIA exacerbation. Control WB-MRI showed inflammatory lesions with polyarthritis, increased areas of osteitis. Due to the positive effect of earlier therapy with TOFA and the negative dynamics of interrupted treatment, the decision was made to continue treatment with TOFA. In addition, for the preventing of infectious complications intravenous immunoglobulin at the dose of 0.2 mg/kg was added to the treatment.

**Conclusion:** In this case we met a lot of problems and obstacles regarding to diagnosis verification and choice of medicine. WB-MRI is a useful tool for identifying the inflammatory origin of arthritis in a child with Down syndrome. Peroral TOFA is preferred for patients with multimorbidity and intolerance to injections. It extremally important to keep good coordination between doctors and parents, especially in case of life-threatening condition during COVID-19 pandemic. Because of the high risk of hospital-acquired COVID-19, it is recommended to postpone any planned surgery, as possible.

**Patient Consent:** Yes, I received consent

**Disclosure of Interest**: None declared

### P032. Ana status is not associated with altered b cell subset distribution in the peripheral blood and synovial fluid of JIA patients

#### B. Jebson^1,2^, N. de Gruijter^1,3^, M. Kartawinata^1,2^, V. Alexiou^1,2^, L. Wedderburn^1,2,4,5^, E. Rosser^1,3^

##### ^1^Centre for Adolescent Rheumatology Versus Arthritis at UCL, UCL, UCLH, GOSH, ^2^UCL GOS Institute of Child Health, ^3^Centre for Rheumatology, Division of Medicine, UCL, ^4^Great Ormond Street Hospital for Children NHS Trust, NHS, ^5^NIHR Biomedical Research Centre at GOSH, NIHR, London, United Kingdom

###### **Correspondence:** B. Jebson

**Introduction:** In Juvenile Idiopathic Arthritis (JIA), many patients are positive for autoantibodies known as anti-nuclear antibodies (ANA), which are secreted by B cells. Production of ANA is a hallmark of a breakdown in B cell tolerance and cellular dysregulation. Despite this, B cell biology remains understudied in JIA and the mechanisms controlling ANA production, and their contribution to pathogenicity, remain unclear.

**Objectives:** To assess whether B cell phenotype is altered in the peripheral blood mononuclear cells (PBMC) and synovial fluid mononuclear cells (SFMC) of JIA patients compared to PBMC from age-matched healthy children and whether altered B cell subset distribution is associated with ANA status.

**Methods:** B cell phenotype was analysed based on CD19, CD24 and CD38 expression using multiparameter flow cytometry analysis and was performed on PBMC from JIA patients (n=185), age-matched healthy controls (n=37) as well as SFMC from JIA patients (n=52, n=47 of which were paired with blood samples from the same patient). JIA patients were further stratified by ANA titre with levels of ≥1:160 being positive and <1:80 negative tested at Great Ormond Street Hospital (PBMC n=117 ANA+, n=56 ANA-), (SFMC n=24 ANA+, n=16 ANA-). Where ANA status was not available, patients were excluded from later analysis.

**Results:** In our large cohort, the frequency of total B cells (CD19+) was significantly higher within PBMC of JIA patients compared to healthy control samples (p=0.0004). There were also higher levels of immature B cells (CD19^pos^CD24^hi^CD38^hi^, p=0.0037) and lower levels of memory B cells (CD19^pos^CD24^hi^CD38^lo^, p=0.0104) within PBMC of JIA patients compared to healthy controls. In agreement with previously published data^1^, when comparing B cell phenotype between JIA PBMC and SFMC, we observed that there were significantly higher levels of ‘atypical’ (CD19^pos^CD24^lo^CD38^lo^, p=<0.0001) memory B cells, memory (CD19^pos^CD24^hi^CD38^lo^, p=<0.0001) B cells and plasmablast (CD19^pos^CD24^lo^CD38^hi^, p=<0.0001) within JIA SFMC. Interestingly, despite a recently published study describing B cell subset differences in the SFMC of ANA+ JIA patients compared to ANA- patients^2^, using our B cell phenotyping strategy we found no differences in the frequency or phenotype of PBMC and SFMC B cells in this cohort of ANA+ and ANA- JIA patients.

**Conclusion:** In this JIA cohort, we found that there were B cell subset abnormalities in patients versus controls, but that these differences were independent of ANA. These data suggest that mechanisms other than altered B cell subset distribution are driving a breakdown of B cell tolerance and ANA production in JIA. Uncovering these mechanisms will form the basis of our future work.

**REFERENCES**

1. Corcione, A. *et al.* Phenotypic and functional characterization of switch memory B cells from patients with oligoarticular juvenile idiopathic arthritis. *Arthritis Research & Therapy***11**, R150 (2009).

2. Dirks, J. *et al.* CD21lo/−CD27−IgM− Double-Negative B Cells Accumulate in the Joints of Patients With Antinuclear Antibody-Positive Juvenile Idiopathic Arthritis. *Frontiers in Pediatrics***9**, 143 (2021).

**Patient Consent:** Yes, I received consent

**Disclosure of Interest**: B. Jebson: None declared, N. de Gruijter: None declared, M. Kartawinata: None declared, V. Alexiou: None declared, L. Wedderburn Grant / Research Support with: MRC, Versus Arthritis, GOSCC, AbbVie, GSK, UCB, Sobi, Pfizer, Novartis, Lilly, Sobi, E. Rosser Grant / Research Support with: NIHR BRC

### P033. Juvenile psoriatic arthritis: data from the pera psoriasis study group

#### S. G. Karadağ^1^, T. Coşkuner^2^, F. G. Demirkan^3^, H. E. Sönmez^4^, S. Özdel^5^, M. Çakan^2^, G. Otar Yener^6^, K. Öztürk^7^, F. Demir^8^, B. Sözeri^2^, N. Aktay Ayaz^3^

##### ^1^Pediatric Rheumatology, University of Health Sciences, Basaksehir Cam and Sakura City Hospital, ^2^Pediatric Rheumatology, University of Health Sciences, Ümraniye Research and Training Hospital, ^3^Pediatric Rheumatology, Istanbul University, Faculty of Medicine, İstanbul, ^4^Pediatric Rheumatology, Kocaeli University, Faculty of Medicine, Kocaeli, ^5^Pediatric Rheumatology, University of Health Sciences, Sami Ulus Maternity and Children’s Diseases Training and Research Hospital, Ankara, ^6^Pediatric Rheumatology, Şanlıurfa Training and Research Hospital, Şanlıurfa, ^7^Pediatric Rheumatology, Istanbul Medeniyet University, Göztepe Prof. Dr. Süleyman Yalçın City Hospital, ^8^Pediatric Rheumatology, Acıbadem Healthcare Group, İstanbul, Turkey

###### **Correspondence:** S. G. Karadağ

**Introduction:** Juvenile psoriatic arthritis (JPsA) is one of the 7 subtypes of juvenile idiopathic arthritis (JIA) according to the International League of Associations for Rheumatology (ILAR) classification criteria. JPsA is the rarest subgroup of JIA, so the corresponding literature describing the clinical characteristics, long-term outcomes, or treatment status of JPsA is limited

**Objectives:** To describe demographic and clinical features of children with JPsA and to compare distinct patterns of the disease between early onset and late onset groups.

**Methods:** Patients classified as JPsA according to ILAR criteria in 7 different pediatric rheumatology centers and followed regularly for at least 6 months between 2010 and 2020 were included in the study. The files of the patients were reviewed retrospectively, and their demographic, clinical and treatment characteristics were evaluated.

**Results:** A total of 87 (46 male/41 female) patients were included in the study. The mean age at diagnosis of JPsA was 11.9 ± 4.5. While 57 (65.5%) of the patients had psoriasis at the time of diagnosis, arthritis preceded psoriasis in 10 (11.5%) patients. There were 32 (36.8%) patients with a history of psoriasis in the first-degree relatives. Thirty (34.5%) patients had dactylitis, 28 (32.2%) had nail pitting, 36 (41.4%) had small joint involvement, 20 (23%) had enthesitis, and 14 (16.1%) had axial involvement. Sacroiliitis was detected in 11 (12.6%) patients on magnetic resonance imaging. Uveitis developed in 4 (4.5%) patients during the follow-up period. Anti- nuclear antibodies (ANA) were positive in 35 (40.2%) patients. Twelve children were in the early-onset (<5 years) group. Uveitis and ANA positivity were more common in the early early-onset group.

**Conclusion:** About one-third of patients with JPsA do not have psoriasis at the time of diagnosis. In some patients, no skin lesion is seen during the course of the disease. Children with psoriatic arthritis seem to displaying two different phenotypes. Younger children tend to have female predominance, ANA positivity and uveitis, while older children have more axial involvement .

**Patient Consent:** Yes, I received consent

**Disclosure of Interest**: None declared

### P034. Investigation of physical activity levels of patients with juvenil idiopatic arthritis era COVID 19

#### E. P. Kısa^1^, E. Tarakcı^2^, G. Leblebici^3^, Ö. Kasapçopur^4^

##### ^1^Department of Physiotheraphy and Rehabilitation,Health Science Faculty, Biruni University, ^2^Physiotheraphy and Rehabilitation,Health Science Faculty, İstanbul University- Cerrahpasa, ^3^Department of Physiotheraphy and Rehabilitation,Health Science Faculty, Medeniyet University, ^4^Department of Pediatric Rheumatology, Cerrahpaşa Faculty of Medicine, İstanbul University- Cerrahpasa, İstanbul, Turkey

###### **Correspondence:** E. P. Kısa

**Introduction:** Childhood rheumatic diseases are a group of diseases that can affect many organs and systems with problems such as pain, joint stiffness, atrophy and weakness. The presence of physical inactivity is one of the most frequently reported conditions. Exercise and physical activity are recognized as an important part of the treatment of children with rheumatic disease. Physical inactivity and the accompanying sedentary lifestyle aggravate problems common in pediatric rheumatic diseases such as weakness, atrophy and muscle dysfunction, chronic pain, fatigue, bone loss, insulin resistance, and decreased health-related quality of life. Conversely, some of these symptoms create a fear of the thought that physical activity may cause an exacerbation of the disease and may be significant barriers to increasing physical activity. In addition to all these problems we have already encountered in the course of the disease, the increase in the time spent at home due to the Covid-19 pandemic has made these problems even more evident.

**Objectives:** The aim of the study was to examine the physical activity levels of children with Juvenile Idiopathic Arthritis in the COVID 19 era and to compare them with their healthy peers.

**Methods:** Fifty-five patients with a diagnosis of oligoarticular JIA, aged 7-18 years, who applied to the Istanbul University-Cerrahpaşa Pediatric rheumatology clinic, and 52 healthy controls in the same age group were included. Participants were invited to the study by making an announcement. All participants were informed about the purpose and procedure of the study before the evaluation. The participant statement was read to the families of all participants included in the study.

The sociodemographic characteristics of the participants (age, gender, height, body weight, disease duration, joint involvement, exercise habits) were evaluated with a sociodemographic questionnaire. Pain status and general well-being level in activity status were questioned with the Visual Analogue Scale (VAS). Physical activity levels and energy expenditure levels were evaluated with the 1-day physical activity scale (1-day activity diary), and functional levels were evaluated with the Childhood Health Assessment Questionnaire (CHAQ). Statistical analysis was performed using SPSS for Windows (Statistical Package ort he Social Sciences-24.0, Inc., Chicago, IL). P≤0.05 was considered statistically significant in all analyzes.

**Results:** Fifty-five patients and 52 controls with a mean age of 12.43±6.33 and 13.24±3.17 years, respectively, were included. The mean disease duration was 4.5 years. Compared to the control group, the JIA group had significantly less time in physical activity (p=0.001), decreased energy expenditure (p=0.042), and higher CHAQ scores (p=0.001) (Table 1).

**Conclusion:** This study demonstrated that children with JIA had significantly lower levels of physical activity, energy expenditure, and functional ability during the COVID-19 pandemic than healthy controls. It has been revealed that patients with JIA who have low physical activity levels due to pain, fatigue and fear of avoiding movement are more inactive duege to inevitable reasons during the pandemic process. This result shows us that children need correct guidance to use the time they stay at home more efficiently in order to increase their physical activity level.

**Patient Consent:** Yes, I received consent

**Disclosure of Interest**: None declared

**Table 1 (abstract P034).** See text for description

Parameters	Group of JIA (n=55)	Healthy Cotrol (n=52)	p
**Physical Activity (MET/day)**	31,23 ± 11,48	34,43 ± 10.08	0,001
**Energy Expendature (kcal/day)**	1124,39 ±358,12	1419,58 ±534,61	0,024
**CHAQ**	0,28 ± 0,24	0,05 ± 0,20	0,001
**VAS pain**	34,14 ± 14,42	-	-
**VAS general feeling of well-being**	53,41 ± 32.18	-	-

### P035. Serum calprotectin (S100A8/A9): a promising biomarker of activity and erosive changes in different subtypes of juvenile arthritis

#### A. Kozhevnikov^1,2^, N. Pozdeeva^1^, S. Vissarionov^1^, S. Bogdanova^1^, G. Novik^2^

##### ^1^H.Turner National Medical Research Center for Сhildren’s Orthopedics and Trauma Surgery, ^2^Saint-Petersburg State Pediatric Medical University, Saint-Petersburg, Russian Federation

###### **Correspondence:** A. Kozhevnikov

**Introduction:** Juvenile arthritis (JA) is an autoimmune inflammatory joint disease. Some laboratory tests neither rule in nor rule out juvenile arthritis. An elevated ANF titer is not a diagnostic criteria for JA. C-reactive peptide and erythrocyte sedimentation rate can be normal despite marked involvement of arthritis. Calprotectin is a heterodimeric complex of S100A8/9 (MRP8/14) has been proposed as serum biomarker that reflects disease activity (J.Hurnakova et al., 2015; T. Marushko et al., 2019; Y. Boyko et al., 2020; M. Romano et al., 2021).

**Objectives:** The **aim of this study** was to test the hypothesis that calprotectin is associated with structural joint damage and reflects disease activity in children with juvenile arthritis.

**Methods:** We evaluated the level of serum calprotectin in 70 children with JIA and 20 adolescents with non-rheumatic joint pain. All children fulfilled the ILAR criteria for JIA. 50 children had active JIA, 17 of them had oligoarticular disease subtype (oJA, mean age 7,2±2,2 years), 15 – polyarthritis (pJA, mean age 9±2,3 years), 18 – enthesitis-related arthritis (erJA, mean age 13±2,7 years). 20 children were with inactive disease by DMARDs. Quantitative indicators distribution is given as a median [5th; 95th percentile]. Clinical data, radiology and laboratory results (serum Calprotectin level/sCal, Vimentin, serum IL6/sIL6, serum TNF-alpha/sTNF) were collected and evaluated in all 90 children.

**Results:** Level of sCal in the active oJA was 2,61 μg/ml [1,015; 3,935], inactive oJA was 1,258 μg/ml [0,772; 2,254], active pJA was 5,845 μg/ml [3,408; 8,005], inactive pJA was 1,36 μg/ml [0,678; 2342], active erJA was 2,98 μg/ml [0,897; 6,876], inactive erJA was 0,94 μg/ml [0,429; 1,92]; sCal level in children with non-rheumatic joint pain was 1,288 μg/ml [0,513; 2,364]. All children with active JIA were divided into three groups depending on their treatment and aggressive disease. 1^st^ gr – consist of 12 children with non-erosive erJA (JADAS10-ESR 3.2 – 4.6), 2^nd^ gr – 16 children with erosive JA which start to therapy of methotrexate (4 erJA /10 oJA /2 pJA, JADAS10-ESR 4.8 – 8.2), 3^rd^ gr - 22 children with erosive JA which switch to anti-TNF drugs (2 erJA /7 oJA /13 pJA, JADAS10-ESR > 13). In the 1^st^ group sCal level were 1,0175 μg/ml [0,45; 2,378], sIL6 - 2,94 pg/ml [1,549; 5,617], Vimentin - 9,872 U/ml [3,87; 18,81], sTNF - 1,144 pg/ml [0,397; 3,757]. In the 2^nd^ sCal level were 3,81 μg/ml [2,48; 5,992], sIL6 16,15 pg/ml [1,769; 48,85], Vimentin - 13,632 U/ml [2,319; 44,492], TNF - 1,18 pg/ml [0,204; 3,54]. In the 3^rd^ sCal level were 4,828 μg/ml [2,93; 7,954], sIL6 - 11,048 pg/ml [1,5; 33,7], Vimentin - 17,22 U/ml [4,212; 52,1], TNF - 10,5 pg/ml [0,5; 50,43]. Statistic analysis were revealed a correlation between sCalc and active erosive JA (R^2^ = 0.4159, T = 4.336, OR erosive JA = 3.3193, 95%CI 1,7006-6.4789, p=0.0079). Serum Level of vimentin, IL6 and TNF-alpha were not correlated with active stage JA and erosive joint damage. The ROC analysis of the sCalc showed that a cut-off point more of 2,9 μg/ml may be high prognostic factor for related erosive JA (AUC 0,837±0,0553, 95%CI 0,711-0.923).

**Conclusion:** The serum levels of calprotectin are significantly associated with oligo and polyarticular JA disease activity. These results suggest that calprotectin might be superior to serum IL6 and TNF-alpha for erosive joint damage in children with JA.

**Patient Consent:** Yes, I received consent

**Disclosure of Interest**: None declared

### P036. Disease activity assessment for juvenile idiopathic artritis in transitional care

#### F. La Torre^1^, F. Cardinale^1^, M. G. Anelli^2^, F. Cacciapaglia^2^, G. Lopalco^2^, F. Iannone^2^

##### ^1^Department of Pediatrics, Pediatric Rheumatology Center, Giovanni XXIII Pediatric Hospital, University of Bari, ^2^Department of Emergency and Organs Transplantation (DETO), Rheumatology Unit, University of Bari, Bari, Italy

###### **Correspondence:** F. La Torre

**Introduction:** Although Juvenile Idiopathic Arthritis and Rheumatoid Arthritis have well-defined disease activity scores, there is no validated score during transition. In clinical practice a patient with JIA who transfer to the adult rheumatologists is evaluated with the disease activity scores validated for RA (DAS28, SDAI and CDAI) and not for JIA.

**Objectives:** Given the differences between the “scores” used in adulthood and pediatric age, the primary objective of our study was to identify which of those scores was the best one in assessing disease activity for JIA during transitional care.

**Methods:** Data from all cases of Juvenile Idiopathic Arthritis (JIA) with almost one year of transition follow-up, older than 16 years at time of baseline visit, were collected. In each visit (at baseline and after 12 months of follow-up) we calculated the disease activity scores used in childhood (JADAS and cJADAS) and in adulthood (DAS28, SDAI and CDAI). Because JADAS10 and JADAS27 may have a joint count lower than the active joints in a subject with polyarticular JIA, and since the need to identify a single score to correlate to the adult ones, JADAS71 was chosen. The linear regression model has been applied to continuous variables. For the discrete variables, instead, the Kendall’s tau test was used. For both methods the concordance is poor between 0-0.4; it’s fair between 0.4-0.6; it’s good between 0.6-0.8 and finally if it assumes values between 0.8-1 the agreement is excellent.

**Results:** We recruited 26 patients with JIA, 11 patients were Polyarticular (42.3%) and 15 patients were Oligoarticular (53.1%). No patient was lost during the follow-up. All the disease activity scores were calculated at baseline and 12-month evaluation. The linear regression analysis was used to correlate continuous variables (applied to the values expressed in mean ± SD) and the results were expressed as R-square. The R-square value was 0.690 for the correlation between JADAS71 and DAS28; 0.865 for the correlation between JADAS71 and SDAI; 0.809 for the correlation between JADAS71and CDAI (Table). To correlate the discrete variables (expressed as a percentage) the Kendall’s tau test was used; the cutoffs for correlation are the same seen for R-square in the linear regression analysis. The Kendall’s tau value was 0.797 for the correlation of JADAS71 with DAS28 and 0.788 between cJADAS71 and DAS28; 0.831 for the correlation with SDAI both for JADAS71 and cJADAS71; and 0.831 for the correlation of JADAS71 with CDAI and 0,813 between cJADAS71 and CDAI (Table).

**Table** shows the correlation between JADAS71 and cJADAS71 to the other scores used in adulthood (DAS28, SDAI, CDAI).

**Conclusion:** Our study confirms that the Simplified disease activity score(SDAI)is the adult score that correlates better with JADAS71. This best agreement is expressed by excellent correlation evaluated by linear regression with R-square value for continue variables (0.865) and with of k (Kendall’s) value for discrete variables (0.831). New prospective studies for disease activity score in transitional care with a large number of patients will be needed to confirm these preliminary results.

**Patient Consent:** Yes, I received consent

**Disclosure of Interest**: None declared

**Table 1 (abstract P036).** See text for description

R-Square	DAS28	SDAI	CDAI
JADAS71	R2= 0,690	R2= 0,865	R= 0,809
Kendall’s tau	DAS28	SDAI	CDAI
JADAS71	0,797	0,831	0,831
cJADAS71	0,788	0,831	0,813

### P037. Patterns of biological switching among children with non-systemic juvenile idiopathic arthritis

#### M. Lindegaard Pedersen^1^, A. Neve-Græsbøll^2^, M. Bonde Glerup^2^, T. Herlin^2^

##### ^1^MP and AG contributed equally to the study and shares the first authorship. Department of Paediatrics and Adolescent Medicin, ^2^Department of Paediatrics and Adolescent Medicin, Aarhus University Hospital, Aarhus N, Denmark

###### **Correspondence:** M. Lindegaard Pedersen

**Introduction:** With the advent of biological agents (BA) for the treatment of patients with juvenile idiopathic arthritis (JIA) achievement of remission has become a realistic goal. However, clinical remission is unattainable in some patients despite BA therapy and switching to another BA is required. The best choice of second-line BA remains unclear.

**Objectives:** This retrospective observational study aims to describe the pattern, timing, frequency, and reasons for BA switching among children diagnosed with non-systemic JIA treated at Aarhus University Hospital, Denmark.

**Methods:** Patients were identified by combining unique personal identification numbers, the International Code of Diagnosis (ICD10) for JIA and biologic therapy: Etanercept, adalimumab, golimumab, infliximab, anakinra, canakinumab, tocilizumab, abatacept, rituximab, tofacitinib and baricitinib. Clinical characteristics were collected retrospectively from the electronic medical records. 200 children diagnosed with non-systemic JIA who started their first biologic drug between January 1st, 2012, and March 1st, 2021, were included in the study. We compared characteristics of non-switchers vs switchers and early switchers (≤6 months) vs late switchers (>6 months).

**Results:** We found that 37% switched to a different BA after median 6.3 (3.8-9.4) years after diagnosis and 17.5% of patients switched at least twice. In total, 6% of patients switched three or more times. The most common reason for switching was inefficacy (57%) followed by injection/infusion reactions (15%) and uveitis (13%). 77% were late switchers, and switched primarily due to inefficacy, and 23% were early switchers who switched more often due to other reasons (61%). All patients started a tumor necrosis factor inhibitor (TNFi) as initial BA (etanercept (ETN): 49.5%, other TNFi: 50.5%). The patients who started ETN as first-line BA were more likely to be switchers compared to those who started another TNFi.

**Conclusion:** During a 6-year observation period biologic switching was observed in more than one third, primarily due to inefficacy.

**Patient Consent:** Yes, I received consent

**Disclosure of Interest**: None declared

**Table 1 (abstract P037).** Clinical characteristics among biologic switchers and non-switchers in juvenile idiopathic arthritis (JIA)

	Switchers	Non- switchers	p-value
**N**	74	126	
Male	17 (23)	40 (32)	0.185
Female	57 (77)	86 (68)	
Subcategory, n (%)			
- Persistent Oligo	10 (13.5)	16 (13)	0.549
- Extended Oligo	27 (36.5)	34 (27)	
- RF+ Polyarticular JIA	2 (3)	6 (5)	
- RF- Polyarticular JIA	23 (31)	36 (29)	
- Psoriatic	4 (5)	9 (7)	
- ERA	5 (7)	19 (15)	ERA: 0.08
- Undifferentiated	3 (4)	6 (5)	
1st biologic, n (%)			
ETN	49 (66)	50 (40)	Enbrel vs Benepali:
Enbrel	36 (73)	35 (70)	0.911
Benepali	13 (27)	12 (24)	
Erelzi	0	3 (6)	ETN vs ADA: <0.001
ADA	15 (20)	61 (48)	
GOL	4 (6)	14 (11)	
IFX	6 (8)	1 (1)	
Disease duration (years)	7.3 (4.7-10.6)	5.4 (3.5-9.0)	0.017
Active joints at start of 1^st^ biologic	2 (1-4)	2 (1-4)	0.987
Cumulative joints at start of 1^st^ biologic	3 (1-8)	6 (4.25-10)	<0.001
DMARD before 1^st^ biologic	69 (93)	107 (85)	0.042
DMARD plus 1^st^ biologic	55 (74)	102 (81)	0.128

Data are expressed as medians with interquartile range (IQR) or as number of patients with % in parentheses. RF: rheumatoid factor; ERA: enthesitis related arthritis; ETN: etanercept; ADA: adalimumab; GOL: golimumab; IFX: infliximab

### P038. Normal neonatal TREC and KREC levels in early onset juvenile idiopathic arthritis

#### J. A. Gudmundsdóttir^1^, S. Thorgeirsdóttir^2^, V. Lundbäck^3^, C. Göngrich^3^, J. Lingman Framme^2^, K. Rydenman^2^, E. Kindgren^4^, B. R. Ludviksson^5^, S. Nilsson^6^, R. Zetterström^3^, O. Ekwall^7^, S. Lindgren^2,7^

##### ^1^Children’s Medical Cente, Landspitali, Reykjavik, Iceland, ^2^Department of Pediatrics, Institute of Clinical sciences, Göteborg, ^3^Centre for Inherited Metabolic Diseases, Karolinska University Hospital, Stockholm, ^4^Division of Pediatrics, Biomedical and Clinical Sciences, Linköping, Sweden, ^5^Department of Immunology, Landspital, Reykjavik, Iceland, ^6^Department of Mathematical Sciences, Chalmers University of Technology, ^7^Department of Rheumatology and inflammation research, Institute of Medicin, Göteborg, Sweden

###### **Correspondence:** S. Lindgren

**Introduction:** Disturbed central tolerance mechanisms predispose to autoimmune diseases. Reduced thymic output as well as compromised central B cell tolerance checkpoints have been proposed in the pathogenesis in juvenile idiopathic arthritis (JIA).

**Objectives:** The aim of this study was to investigate neonatal levels of T cell receptor excision circles (TRECs) and kappa-deleting element excision circles (KRECs), as markers of T- and B-cell output at birth, in patients with early onset JIA.

**Methods:** TRECs and KRECs levels were quantitated by multiplex qPCR from dried blood spots (DBS), collected 2-5 days after birth, in 156 children with early onset JIA and 312 matched controls.

**Results:** When analysed from neonatal dried blood spots, the median TREC level was 78 (IQR 55-113) in JIA cases and 88 (IQR 57-117) copies/well in controls. The median KREC level was 51 (IQR 35-69) and 53 (IQR 35-74) copies/well, in JIA cases and controls, respectively. Stratification by gender and age at onset of disease did not reveal any difference in the levels of TRECs and KRECs.

**Conclusion:** T- and B-cell output at birth, as measured by TRECs and KRECs levels in neonatal dried blood spots, does not differ in children with early onset JIA compared to controls.

**Patient Consent:** Not applicable (there are no patient data)

**Disclosure of Interest**: None declared

### P039. Temporomandibular Joint (TMJ) involvement in Juvenile Idiopathic Arthritis (JIA) at disease onset: a rheumatologist’s, orthodontist’s and radiologist’s joint venture

#### S. Giancaspro^1,2^, G. Tarantino^3^, P. Festa^2,4^, G. Vallogini^2^, L. M. Gregori^5^, D. Pires Marafon^3^, A. Aquilani^3^, R. Nicolai^3^, E. Marasco^3^, F. De Benedetti^3^, V. Quinzi^1^, A. Galeotti^2^, S. Magni-Manzoni^3^

##### ^1^Dep.of Health, Life and Environmental Science, University of L’Aquila, L’Aquila, ^2^Dentistry Unit, IRCCS Bambino Gesù Children’s Hospital, ^3^Rheumatology, IRCCS Bambino Gesù Children’s Hospital, Rome, ^4^AORN Santobono-Pausilipon, Naples, ^5^Imaging Dep., IRCCS Bambino Gesù Children’s Hospital, Rome, Italy

###### **Correspondence:** S. Magni-Manzoni

**Introduction:** TMJ arthritis in JIA often occurs asymptomatic in the initial stages. The frequency of TMJ involvement at JIA onset has been scarcely investigated.

**Objectives:** To assess in a multidisciplinary approach the frequency of TMJ involvement in a prospective cohort of patients at JIA onset; to describe demographic and clinical features of JIA patients with TMJ involvement at JIA onset and follow up.

**Methods:** Consecutive patients at JIA onset in a single tertiary care centre, who provided consent, underwent rheumatologic and orthodontic assessments. Demographic, clinical features and joint counts were registered by rheumatologists. Diagnostic Criteria for Temporomandibular Disorders (DC/TMD) Axis I and Axis II assessment protocol was runned in each patient in separate orthodontic examination. The following features were registered by consensus of at least two orthodontists: presence and type of myalgia of the masticatory muscles, myofascial pain, TMJ arthralgia, TMJ associated headache, disc displacement, intermitted locking, limited opening, clinically detectable osseous changes, subluxation. The overall orthodontic assessment required 50-60 minutes. Results of the rheumatologic and orthodontic evaluations in each patient were regularly discussed in periodic multidisciplinary meetings for the definition of the subsequent diagnostic and therapeutic program. Only patients with time frame of less than 8 weeks between rheumatologic and orthodontic assessment were included in the analysis. Descriptive statistics was used.

**Results:** One hundred and fifteen patients at JIA onset seen at the study centre from 2018 to 2021 were invited to participate. Twenty-three (22%, 78% female) performed complete rheumatologic and orthodontic assessment maximum 2 months apart; 66% and 30% were respectively affected by oligoarticular and polyarticular RF-negative JIA subtype, and presented a median age at disease onset of 5.2 years (IQR 2.8-8.5). Five patients (22%) showed at least one pathologic feature at the DC/TMD assessment; 3 of them presented any TMJ sign/symptom also at the rheumatologic examination. TMJ MRI investigation was required in one patient (female, oligo JIA, of 9 years of age), with confirmation of TMJ active involvement. MRI follow up after 11 months, with meaningwhile subcutaneous methotrexate and TNF-inhibitor treatment, showed structural improvement and no disease progression. At the last follow up, after a median of 24 months (IQR 7-27), only 2 of the 5 JIA patients with baseline pathological signs/symptoms still presented clinically detectable TMJ involvement, with no progression; 1 patient negative for TMJ involvement at JIA onset (6%) reported mild TMJ symptoms at the last follow up visit, with no indication to imaging exam or medical treatment.

**Conclusion:** Though the relatively low rate of combined rheumatologist’s and orthodontist’s assessment in the study cohort, TMJ involvement was detected in a relatively high proportion of patients at JIA onset. Joined efforts are required for increasing the performance of the clinical and imaging tools for quick identification and treatment of this potential invalidating feature of JIA. Improvement in the feasibility of the diagnostic investigations are also urgently required.

**Patient Consent:** Yes, I received consent

**Disclosure of Interest**: None declared

### P040. Factors increasing the risk of flare in JIA with inactive disease under MTX monotherapy

#### R. Isguder, Z. Kizildag, R. Torun, T. Aydın, B. Makay, S. E. Unsal

##### Pediatric Rheumatology, Dokuz Eylül University Faculty of Medicine, IZMIR, Turkey

###### **Correspondence:** R. Isguder

**Introduction:** Juvenile idiopathic arthritis (JIA) is a chronic and heterogeneous disease causing structural changes in the joints. Treatment aims to stop inflammation, relieve pain, maintain joint function, prevent damage, and suppress disease activity. As treatment options increase, maintenance of drug-free inactive disease (ID) has gained more importance. Methotrexate (MTX) is the most commonly used agent among synthetic DMARDs in JIA. Nevertheless, there is no consensus on how and when to stop MTX to maintain drug-free remission after ID. Factors that increase the risk of flare, apart from the drug withdrawal strategy are also unclear.

**Objectives:** To determine the factors that increase the risk of disease flare in patients with JIA who stopped MTX monotherapy following inactive disease.

**Methods:** Files of all JIA cases between 1992-2022 were examined retrospectively. Patients who stopped MTX monotherapy following inactive disease were evaluated. Patients with disease flare and ongoing ID were compared.

Demographic data, JIA subgroup, age of disease onset, autoantibodies, acute phase reactants, MTX dose and administration route, MTX discontinuation method, and time interval were recorded.

**Results:** Among the files of 1,036 patients with JIA, a total of 333 patients were identified as MTX monotherapy, and ID was achieved in 138 (41.5%). Among 138 patients, 105 (76%) were female and median age was 5.8 years. Oligoarticular JIA was the most frequent type. Other subtypes included polyarticular JIA, systemic-onset JIA (SoJIA), and juvenile psoriatic arthritis (JpsA) (Table-1). Patients reached ID in median 7.9 months after starting MTX, and MTX treatment was discontinued median 1.02 year after ID.

The disease flare developed in 67 (48.6%) of the cases. The ID continued in 71 patients (51.4%) (Table-1). The flare developed median 1.6 years after ID and median 8.8 months after discontinuation of MTX. The follow-up period of cases with persistent inactive disease was 3.06 years.

The age at disease onset and at diagnosis were lower in the group with flare, and SoJIA cases were less frequent in this group. The anti-nuclear antibody (ANA) positivity was more frequent in cases with flare. At MTX initiation visit, the median C-reactive protein (CRP) value in the group with the flare was found to be higher compared to the patients with ongoing ID (Table-1).

The risk of flare increased in cases with a CRP value above 6.7 mg/L at the onset of MTX, age at disease onset below 5.1 years, and age at diagnosis below 5.9 years.

There was no difference between the two groups in terms of MTX dose and administration route, the duration to reach inactive disease, MTX discontinuation time, and method.

**Conclusion:** In this study, risk of flare was associated with early disease onset, ANA positivity and high CRP values at the beginning, rather than the administration and discontinuation method of MTX.

**Patient Consent:** Yes, I received consent

**Disclosure of Interest**: None declared

**Table 1 (abstract P040).** Comparison of “flare” and “persistent ID” groups

	Flare n=67 (48,6%)	Persistent ID n=71 (51,4%)	All patients n=138 (100%)	p
**Median age at disease onset, yrs** median (min-max), IQR*	3,8 (0,4-16,3) 5,2	6,9 (0,4-15) 8,6	5,1 (0,4-16) 6,4	**0,02**
**Median age at diagnosis, yrs** median (min-max), IQR*	4,2 (0,5-16) 5,3	7,8 (0,8-15,7) 8,8	5,8 (0,5-16) 6,8	**0,004**
**JIA subgroup** n (%)				
Oligoarticular JIA	34 (61)	22 (39)	56 (40,6)	**0,007**
Polyarticular JIA	21 (46,7)	24 (53,3)	45 (32,6)	
So JIA	5 (20)	20 (80)	25 (18,1)	
JPsA	7 (58,3)	5 (41,7)	12 (8,7)	
**ANA positivity** n (%)	41 (61,2)	26 (36,6)	67 (48,5)	**0,004**
**CRP value at MTX initiation (mg/L)** median (min-max), IQR*	8 (0,2-222) 23,8	4,2 (0,02-193) 15	6,8 (0,02-222) 21	**0,01**

### P041. Charcot’s neuropathic arthropathy – when no pain is not pleasure!

#### N. Maldar, A. Khan, A. Prabhudesai, R. Khubchandani

##### Pediatric Rheumatology, NH SRCC Children’s Hospital, Mumbai, India

###### **Correspondence:** N. Maldar

**Introduction:** Charcot’s neuropathic osteoarthropathy (CNA) is a rare eponymous condition associated with autonomic sensory neuropathy, first described in 1868. Warm, painless boggy joints and instability, erosive arthropathies, chronic osteomyelitis and deformities are the predominant manifestations. Differentials are congenital insensitivity to pain and anhidrosis (CIPA), familial dysautonomia(Riley-Day syndrome), hereditary sensory motor neuropathy (Charcot-Marie-Tooth disease), traumatic nerve injuries, syringomyelia and meningomyelocele. CIPA is caused by mutations in NTRK1 gene and ﻿is classified as type IV hereditary sensory and autonomic neuropathy according to the Dyck’s classification.

**Objectives:** We present a case series of 3 CNA misdiagnosed as inflammatory/infective conditions.

**Methods: Case 1**: 18 months-old girl born of third-degree consanguinity, presented with low grade fever, tender hard swelling around the right shoulder extending to the distal forearm with palmoplantar skin rash. Suspecting hereditary autoinflammatory bone disease with chronic non-bacterial osteomyelitis as a feature, a clinical exome was performed. On reverse phenotype corelation there was history of self-mutilation, absence of pain during immunization with anhidrosis.**Case 2**: An 8-year-old boy was referred for fever and right ankle swelling, misdiagnosed as JIA, was on steroids and disease modifying drugs. There were hyperkeratotic lesions on both heels and large warm, boggy left ankle swelling albeit with a full and *painless* range of motion. Neurological evaluation suggested gait disturbance, normal muscle power, intact temperature and deep tendon reflexes with insensitivity to pain. Suspecting a syndromic arthropathy, a whole exome was sent.

**Case 3**: 7-year-old boy had trauma induced fracture in distal shaft of left tibia. In a few months, this was followed by painful swelling that progressed into chronic osteomyelitis of tibia. He had a limp, boggy left ankle, high steppage gait with foot drop and reduced sensations in the affected foot. He was treated by debridement with saucerization of left distal tibia with common peroneal nerve release.

**Results:**

**Conclusion:** While traditional teaching includes Blau syndrome and pigmented villonodular synovitis as differentials of a large boggy warm joint, addition of the adjective *painless,* should bring to mind CNA. This entity may remain undiagnosed for long and be mistaken for commoner rheumatological entities. Management consists of counselling regarding the disease, need for orthotics and prevention of accidental or thermal injuries. Simple questions pertaining to pain perception, sweating, local hair loss and bedside neurological evaluation easily provide the diagnosis.

**Patient Consent:** Yes, I received consent

**Disclosure of Interest**: None declared

**Table 1 (abstract P041).** See text for description

Case	1	2	3
**Age /Sex**	18m/ F	8y/M	7y/M
**Height/Weigh**	84.5 cm/ 10.3kg	117cm/19kg	115cm/18.3kg
**Duration of complaints (months)**	6	12	36
**Diagnosis**	CIPA	CIPA	Traumatic peroneal neuropathy
**ESR(mm/hr)**	30	98	68
**CRP(mg/dL)**	12.5	26.8	336
**X ray**	Fluid distension- subacromial sub deltoid bursa Osseous fragmentation Calcinosis	Sclerosis Bone fragmentation	Chronic osteomyelitis in left tibia Valgus deformity
**MRI**	Bilateral humeral head fractures Metaphyseal marrow edema Joint effusion.	Intramedullary collection- tibial distal metaphysis Moderate marrow edema Ankle joint effusion Tenosynovitis	Osteomyelitis tibia. Marrow edema tarsal bones Left tibiotalar chronic synovitis
**EMG/NCV**	Not done	Asymmetric, axonal peripheral sensory neuropathy- Bilateral. Peripheral motor neuropathy- left lower limb. Absent sympathetic skin response	Severe axonal left deep, superficial peroneal motor neuropathy
**Genetic Evaluation**	Pathogenic homozygous mutation NTRK1 gene Exon 1 c.97delG (p.Ala33ProfsTer36)	Double heterozygous missense mutation NTRKI gene (NTRK1):c.1787G>A (p.Arg596Gln)Exon 16 Chromosome 1 (pathogenic) (NTRK1):c.2263C>T (p.Arg755Trp) Exon 13 (VOUS)	Not done

### P042. Predicting drug-free inactive disease two years after diagnosis of non-systemic juvenile idiopathic arthritis

#### M. Mannion^1^, C. Chen^2^, O. Halyabar^3^, S. Paetkau^4^, T. Qui^2^, B. Huang^2^ on behalf of for the PR-COIN Investigators

##### ^1^University of Alabama at Birmingham, Birmingham, ^2^Division of Biostatistics, Cincinnati Children’s Hospital Medical Center, Cincinnati, OH, ^3^Boston Children’s Hospital, Boston, MA, United States, ^4^parent, Hospital for Sick Children, Toronto, Canada

###### **Correspondence:** M. Mannion

**Introduction:** The goal of treatment for juvenile idiopathic arthritis (JIA) is inactive disease (ID). The best treatment for each patient to maximize ID and minimize overtreatment is unknown.

**Objectives:** The objective of this study was to assess if clinical measures could predict the onset of ID or drug-free inactive disease (DFID) within 2 years of diagnosis of non-systemic JIA.

**Methods:** Using an inception cohort from a large pediatric rheumatology clinic in the US from 2009 to 2020, we identified patients with non-systemic JIA in the electronic health record (EHR) with ≥ 2 clinical visits and ≥ 2 years of follow up following diagnosis. JIA category at baseline is reported; oligoarticular (oligo), rheumatoid factor (RF) - polyarticular (poly), RF+ poly, and all other non-systemic. Medications were classified into systemic corticosteroids, non-biologic (nb) disease modifying anti-rheumatic drug (DMARD), biologic (b) DMARD, and intra-articular corticosteroid injections. ID was defined as no active joint count (AJC), no enthesitis, no active uveitis, and physician global (PGA) <1. Descriptive statistics and Kaplan-Meier curves for time to ID and DFID were calculated based on baseline characteristics. Cox Proportional hazard (CoxPH) modeling was used to evaluate the effect of baseline characteristics and 1^st^ year medication use on onset of ID and DFID.

**Results:** 605 patients with JIA were included (Table). By 1(2) years post diagnosis, 52(73)% and 28(42)% achieved ID and DFID respectively. Time to 1^st^ ID or DFID is significantly different (Log-rank test P=0.001 for ID; P<.0001 for DFID) by category; median (95% CI) time to ID and DFID in oligo 0.76 years (0.63, 0.90), 1.65 (1.08, 2.24), RF- poly 1.23 (1.01, 1.49), 5.05 (3.21, 7.83), RF+ poly 1.36 (0.63, 2.79), >2.22, and other types 0.90 (0.73, 1.18), 2.87 (1.99, 4.67). Of 30 RF+ poly patients only 10 achieved DFID, the median time to DFID could not be estimated. JIA category was not significant in multivariable CoxPH analyses, but lower clinical juvenile disease activity score (cJADAS), shorter time from symptoms to diagnosis, and private insurance were significantly associated with sooner time to ID and DFID. Less DMARD use (b and nb) was associated with sooner DFID, but more bDMARD use was associated with sooner ID.

**Conclusion:** In the two years after diagnosis, 73% of JIA patients achieved ID and 42% achieved DFID. There are disease characteristics associated with sooner time to ID and DFID, but further research is needed to predict medication needs for patients with JIA.

**Patient Consent:** Not applicable (there are no patient data)

**Disclosure of Interest**: M. Mannion Grant / Research Support with: Rheumatology Research Foundation Norman B Gaylis, MD Clinical Investigator Award, C. Chen: None declared, O. Halyabar: None declared, S. Paetkau: None declared, T. Qui: None declared, B. Huang: None declared

**Table 1 (abstract P042).** Disease and treatment characteristics stratified by ILAR classification. Values are median (IQR) or n (%). Medication use is in the 12 months after diagnosis. All p-values <0.0001

	RF- poly JIA (n=171)	RF+ poly JIA (n=30)	Oligo JIA (n=243)	Other JIA (n=161)
Time (year) from diagnosis to 1st DMARD	0.01 (0, 0.09)	0.01 (0, 0.05)	0.31 (0.07, 1.21)	0.04 (0, 0.13)
cJADAS	16 (10.5, 20.5)	19 (15.5, 25)	7 (5, 10)	11 (7.5, 15)
AJC	8 (4, 10)	10 (7, 10)	1 (1, 2)	2 (1, 6)
Patient global	4 (2, 6)	5.5 (3, 7)	3 (1, 5)	4 (2, 6)
PGA	4 (2.5, 7)	5.5 (3, 8)	3 (1, 4)	3 (1.5, 5)
Joint injection	37 (22%)	9 (30%)	106 (44%)	41 (25%)
Glucocorticoids	80 (47%)	20 (67%)	68 (28%)	48 (30%)
nbDMARD >6 months	133 (78%)	23 (77%)	89 (37%)	79 (49%)
bDMARD >6 months	91 (53%)	20 (67%)	46 (19%)	82 (51%)

### P043. Short term outcomes following tolerated disease activity level for individuals with juvenile idiopathic arthritis in the childhood arthritis and rheumatology research alliance registry

#### M. L. Mannion, F. Xie, T. Beukelman, J. R. Curtis on behalf of for the CARRA Registry Investigators

##### University of Alabama at Birmingham, Birmingham, United States

###### **Correspondence:** M. L. Mannion

**Introduction:** Current recommendations suggest treatment escalation for juvenile idiopathic arthritis (JIA) until the disease activity target is reached.

**Objectives:** We compared patient reported outcomes (PRO) 6 months after maximally tolerated disease activity level.

**Methods:** We included all individuals enrolled with non-systemic JIA in the Childhood Arthritis and Rheumatology Research Alliance (CARRA) Registry. We defined the maximally tolerated disease activity at the registry visits with no medication change for >180 days prior. Individuals could contribute more than one observation but were excluded for a medication change between the index and 6 month follow up visit. Individuals were considered to have polyarticular (poly) course if total joint count was >4 and oligoarticular (oligo) course if total joint count was ≤ 4. Disease activity was classified based on clinical Juvenile Arthritis Disease Activity Score (cJADAS) inactive disease (ID; oligo ≤1.1, poly ≤2.5), cJADAS low disease activity (LDA; oligo ≤2, poly ≤3.8), American College of Rheumatology (ACR) provisional criteria for clinical inactive disease (ACRCID) and ACR preliminary criteria for clinical inactive disease (Wallace). Change in outcome variables (Patient Reported Outcomes Measurement Information System [PROMIS] pain interference, mobility, and pediatric global health) from index to 6 month visit was stratified by disease activity status at the index visit and presented as relative change determined by measure specific minimal clinical important difference. The association with tolerated disease activity state was compared by descriptive statistics.

**Results:** 6,235 individuals with JIA and 10,653 observations were included. The tolerated disease activity state was cJADAS ID in 36%, cJADAS LDA in 41%, ACRCID in 32%, and Wallace criteria in 33%. At the index visit the median (interquartile range; IQR) pain interference was 49 (40.6, 56.6, n=6359), mobility was 56 (43, 58.5, n=6670), and global health was 42.1 (37.9, 45.7, n=8445). cJADAS ID, cJADAS LDA, ACRCID, and Wallace were all associated with differences in relative change of mobility, but only cJADAS ID and cJADAS LDA were associated with relative change in pain interference or global health.

**Conclusion:** Individuals with JIA often have higher than ID or LDA at the time of no medication change and have a lower global health than population norms. There were similar frequency of worsening for all measures and tolerated disease activity states; these results do not suggest preference of one criteria set over another.

**Patient Consent:** Not applicable (there are no patient data)

**Disclosure of Interest**: M. Mannion Grant / Research Support with: Rheumatology Research Foundation Norman B Gaylis, MD Clinical Investigator Award, F. Xie: None declared, T. Beukelman Consultant with: Novartis, UCB, J. Curtis: None declared

**Table 1 (abstract P043).** Relative change in variables from index to 6 month follow up visit stratified by disease activity state at index visit. Minimal clinical important difference for each variable were determined to be: pain interference improvement -3 and worsening +3, mobility and global health improvement +3 and worsening -3

	Change from index to 6 month visit	cJADAS ID	cJADAS LDA	ACRCID	Wallace	Not cJADAS ID	Not cJADAS LDA	Not ACRCID	Not Wallace
Pain inter-ference	improved	83 (32%)	108 (33%)	78 (33%)	92 (36%)	176 (45%)	151 (47%)	265 (42%)	251 (41%)
	unchanged	89 (35%)	109 (33%)	82 (35%)	87 (34%)	117 (30%)	97 (30%)	189 (30%)	184 (30%)
	worsen	86 (33%)	111 (34%)	74 (32%)	80 (31%)	100 (25%)	75 (23%)	184 (29%)	178 (29%)
Mobility	improved	90 (19%)	108 (19%)	80 (21%)	90 (22%)	164 (43%)	146 (48%)	247 (36%)	237 (36%)
	unchanged	311 (64%)	350 (63%)	242 (63%)	248 (61%)	149 (39%)	110 (36%)	316 (46%)	310 (46%)
	worsen	85 (17%)	101 (18%)	65 (17%)	68 (17%)	67 (18%)	51 (17%)	123 (18%)	120 (18%)
Global Health	improved	171 (26%)	197 (26%)	156 (29%)	164 (29%)	193 (37%)	167 (39%)	339 (33%)	331 (33%)
	unchanged	311 (47%)	353 (46%)	246 (45%)	261 (45%)	191 (36%)	149 (35%)	410 (40%)	395 (39%)
	worsen	181 (27%)	211 (28%)	144 (26%)	149 (26%)	143 (27%)	113 (26%)	286 (28%)	281 (28%)

### P044. Impact of weekdays versus weekend days on accelerometer measured physical behavior in adolescents with Juvenile Idiopathic Arthritis (JIA): results from the ActiMON study

#### F. Milatz^1^, R. Trauzeddel^2^, T. Kallinich^3,4^, M. Klaas^5^, H. Girschick^5^, S. Hansmann^6^, G. Horneff^7,8^, D. Windschall^9,10^, J.-P. Haas^11^, N. Baumeister^12^, M. Niewerth^1^, K. Minden^1,13^

##### ^1^Epidemiology and Health Services Research, German Rheumatism Research Centre, ^2^Department of Paediatrics, Helios Klinik Berlin-Buch, ^3^Pathophysiology of Rheumatic Inflammation, German Rheumatism Research Centre , ^4^Department of Pediatric Respiratory Medicine, Immunology and Critical Care Medicine, University Medicine Charité Berlin, ^5^Children’s Hospital, Vivantes Hospital im Friedrichshain, Berlin, ^6^Center for Pediatric Rheumatology, autoinflammation reference centre Tuebingen (arcT), University Children’s Hospital Tuebingen, Tuebingen, ^7^Department of Paediatrics, Asklepios Clinic Sankt Augustin, Sankt Augustin, ^8^Department of Pediatric and Adolescents Medicine, University of Cologne, Cologne, ^9^Clinic of Paediatric and Adolescent Rheumatology, Northwest German Center for Rheumatology, St. Josef-Stift Sendenhorst, Sendenhorst, ^10^Medical Faculty, Martin Luther University of Halle-Wittenberg, Halle, ^11^Center for Pain Treatment in Young People, German Center for Paediatric and Adolescent Rheumatology, Garmisch-Partenkirchen, ^12^Department of Sport and Health Sciences, Technical University of Munich, Munich, ^13^Department of Rheumatology and Clinical Immunology, University Medicine Charité Berlin, Berlin, Germany

###### **Correspondence:** F. Milatz

**Introduction:** JIA have been linked to reduced physical activity (PA) and increased time spent sedentary [1]. While structured activities in which young patients participate (e.g. at school) are consistent and limited in scope, after-school or weekend activities, in contrast, encompass a broader range of behaviors. Rrecording a wide range of intensity under daily life conditions is an important prerequisite for deriving measures to promote PA.

**Objectives:** Since it is assumed that adolescents’ PA is lower on weekends compared to weekdays or school days, this study aimed i) to objectively assess PA in detailed intensity range over the course of a week and ii) to determine the proportion of patients achieving the WHO recommended minimum level of at least 60 minutes moderate-to-vigorous physical activity (MVPA) daily.

**Methods:** Within the framework of the ActiMON study as part of the TARISMA research network, data were collected at several German paediatric rheumatology centres. In the period from June 2021 to April 2022, measurements were collected exclusively outside lockdowns and during compulsory attendance at schools. The study sample included 12- to 18-year-olds with JIA who wore an accelerometer (ActiGraph wGT3X-BT) on an elastic waist band during the waking hours of a 7-day period for measuring PA in categories, including sedentary behavior, as well as light, moderate and vigorous PA. Absolute and percentage intensity distributions were evaluated daily. Clinical parameters of participating patients were used from the National Paediatric Rheumatologic Database (NPRD).

**Results:** Data of 54 patients (mean age 14.9 ± 1.8 years, female 71%, patients’ disease duration 7.9 ± 4.6 years, polyarthritis 41%, cJADAS-10 2.3 ± 2.4) were available for evaluation. Almost 70% of participating adolescents met the WHO-recommended minimum level of MVPA, while the average daily step count achieved was 7107. The average wear time was 857 min daily from Monday–Thursday with significant deviations from the mean on Friday (+46 min), Saturday (–40 min), and Sunday (–117 min). While the number of steps was slightly higher during the week than at the weekend, absolute MVPA times were lower. However, the percentage distribution remained constant over all days both for girls and boys. The average daily time spent sedentary was 10.8 ± 1.9 hours (no gender differences), with no relevant differences between weekdays and weekend.

**Conclusion:** While a large proportion of patients achieve the WHO recommended minimum level of PA, they exhibit very pronounced sedentary behavior. The percentage distributions of the different physical behavior intensity categories are similar over all weekdays and weekend days. Interventions should generally try to shift activity away from sedentary behavior towards a more active lifestyle. Further results are expected with ongoing recruitment.

**References:**

[1] Gualano B et al. Physical activity for paediatric rheumatic diseases: standing up against old paradigms. Nat Rev Rheumatol 2017;13:368-379.

**Acknowledgement:** ActiMON is funded by the Federal Ministry of Education and Research (01EC1902F)

**Trial registration identifying number:** German Clinical Trials Register DRKS00022258

**Patient Consent:** Yes, I received consent

**Disclosure of Interest**: None declared

### P045. Parents´ experiences of a one-year support program when their child was diagnosed with juvenile idiopathic arthritis – a qualitative interview study

#### K. Mördrup^1^, J. G. Jungner^2^, E. Broström^3^, C. Bartholdson^3^

##### ^1^Women’s and Children’s Health, Karolinska University Hospital, ^2^Women’s and Children’s Health, Karolinska Institutet, ^3^Women’s and Children’s health, Karolinska University Hospital, Karolinska Institutet, Stockholm, Sweden

###### **Correspondence:** K. Mördrup

**Introduction:** Juvenile Idiopathic Arthritis (JIA) is one of the most common acquired diseases during childhood in Sweden with approximately 200 children diagnosed every year^1^. Being diagnosed with JIA can be shocking and overwhelming for the child and the parents^2^, in addition all information given directly after the diagnose can be difficult to assimilate^3,4^. They therefore need supportive care by repeated information and communication between the scheduled visits^5^. A one-year support program, including scheduled visits and the possibility of direct contact with a nurse was therefore developed and offered to all newly diagnosed children and their parents to improve quality of care.

**Objectives:** To explore parents experiences of participating in the support-program.

**Methods:** When the child was diagnosed with JIA, the child and the parents were offered to participate in the support program from the time of diagnose to one year ahead. The program included seven structured person-centered visits. The nurse was coordinating and participating in all the visits (including visits with physicians) from the start. After completing the program, parents were invited to participate in semi-structured interviews. Fieldnotes were taken by the researcher who conducted the interviews and qualitative analysis was performed.

**Results:** The interviewed parents were ten mothers and six fathers, and their children were between 1 and 16 years old when they were diagnosed with JIA. Data from the field notes show that the families greatly appreciated the possibility of direct contact by phone with the coordinating nurse i.e. good accessibility. They also found continuity as important at the unit and that they expressed great trust for the healthcare professionals. The parents described a professional and holistic care where their child have been listened to and treated from a child’s perspective. The family felt safe about the disease and medication. The coordination of the visits were described as good and the advantage to have a patient responsible nurse was mentioned. Some parents experienced that they felt they were included in a new familly.

**Conclusion:** The 16 Swedish parents who had participated in the one-year support program after their child was diagnosed with JIA were very satisfied with the care they had received. We thus conclude that the new one-year program is a suitable way to improve quality of pediatric rheumatology care and supports the families during their first year.

**Patient Consent:** Not applicable (there are no patient data)

**Disclosure of Interest**: None declared

### P046. The parent/child juvenile idiopathic arthritis disease activity score: responsiveness to change and factor analysis

#### R. Naddei^1^, F. Bovis^2^, F. Ridella^2^, C. Trincianti^2^, S. Pastore^3^, K. Minden^4^, M. Ekelund^5^, P. Barone^6^, S. Scala^1^, E. Patrone^1^, N. Ruperto^1^, A. Ravelli^1^, A. Consolaro^1^ on behalf of The Paediatric Rheumatology International Trials Organisation (PRINTO)

##### ^1^Istituto Giannina Gaslini, ^2^Università degli Studi di Genova, Genoa, ^3^IRCCS Burlo Garofolo, Trieste, Italy, ^4^German Rheumatism Research Centre, Berlin, Germany, ^5^Ryhov County Hospital, Jonkoping, Sweden, ^6^Catania University Hospital, Catania, Italy

###### **Correspondence:** R. Naddei

**Introduction:** Assessment of disease activity is a crucial component of the clinical management of children with juvenile idiopathic arthritis (JIA). According to the most recent requirements, both parent’s and children’s perception should be considered when evaluating the disease course and assessing effectiveness of therapy. Therefore, a new disease activity evaluation tool, based on parent/patient-centered outcome measures, is under development and named parent/child Juvenile Arthritis Disease Activity Score (par/childJADAS).

**Objectives:** Aim of this study was to evaluate responsiveness to change of the parJADAS and to conduct an explanatory factor analysis (EFA) on the scores’ items.

**Methods:** The parJADAS and childJADAS include 4 measures: 1) parent/child assessment of disease activity, rated on a 0-10 visual analogue scale (VAS); 2) assessment of pain intensity, rated on a 0-10 VAS; 3) self/proxy count of any swollen or painful joint up to a maximum of 10 joints; 4) assessment of morning stiffness (MS) on a Likert scale, ranging from no MS (0 points) to > 2 hours of MS (10 points). Responsiveness to change was assessed by computing the standardized response mean (SRM) in a subgroup of JIA patients enrolled in the PharmaChild registry, including subjects having a study visit at the time of biologic treatment initiation and a subsequent study visit no more than 6 months later, with a subjective rating of improvement by the attending physician. An EFA on the 4 items of the par/childJADAS was performed on a multinational dataset of JIA children enrolled in the study of Epidemiology, treatment and Outcome of Childhood Arthritis (EPOCA). The factors were extracted according to the principal factors method and the optimal number of factor extraction was based upon eigenvalues ≥ 1. The factors were rotated by the varimax method.

**Results:** Since the amount of children’ observations in the corresponding dataset was not sufficient, responsiveness to change was assessed only for the parJADAS. Sixty patients met the requirements for SRM analysis. Second visit was at a median of 37 days after biologic treatment initiation. The SRM value obtained was 0.71. The EFA was conducted on data from 8,431 parents and 5,873 children of EPOCA dataset, who had the 4 items of the scores available. The Kaiser–Meyer–Olkin measure was 0.79 and 0.78 for the parents’ and the children’s samples, respectively, indicating that both samples were adequate, and the Bartlett’s test of sphericity resulted significant (p<0.0001) for both scores, indicating that a EFA may be useful. The EFA showed that one factor explained 59.0% of the variance in the parent sample and 61.0% in the child one. The factor loadings were high for both the samples, ranging from 0.60 (joint count and MS) to 0.90 (parent/patient assessment of disease activity, pain intensity level).

**Conclusion:** The parJADAS SRM was in moderate range in patients judged as improved by the caring physician after starting a biological medication, suggesting that the responsiveness to change of the score is good. The EFA showed that the 4 items of both parJADAS and childJADAS work well together, indicating a good internal consistency.

**Patient Consent:** Yes, I received consent

**Disclosure of Interest**: None declared

### P047. A decade of progress in juvenile idiopathic arthritis treatments and outcomes in Canada: results from reacch-out and the CAPRI registry

#### K. Nguyen^1^, J. Barsalou^2^, S. Benseler^3^, R. Berard^4^, G. Chédeville^5^, P. Dancey^6^, E. Dermikaya^4^, C. Duffy^7^, K. Houghton^8^, A. Huber^9^, N. Johnson^3^, D. Levy^10^, L. Lim^11^, K. Oen^12^, J.-P. Proulx-Gauthier^13^, A. Rosenberg^14^, D. Rumsey^11^, H. Schmeling^3^, L. Tucker^8^, R. Yeung^10^, J. Guzman^8^

##### ^1^University of British Columbia, Vancouver, ^2^Pediatrics, University of Montreal, Montreal, ^3^Pediatrics, University of Calgary, Calgary, ^4^Pediatrics, Western University, London, ^5^Pediatrics, McGill University, Montreal, ^6^Pediatrics, Memorial University, St. John’s, ^7^Pediatrics, U of Ottawa, Ottawa, ^8^Pediatrics, University of British Columbia, Vancouver, ^9^Pediatrics, Dalhousie University, Halifax, ^10^Pediatrics, University of Toronto, Toronto, ^11^Pediatrics, University of Alberta, Edmonton, ^12^Pediatrics, U of Manitoba, Winnipeg, ^13^Pediatrics, Laval University, Quebec City, ^14^Pediatrics, U of Saskatchewan, Saskatoon, Canada

###### **Correspondence:** K. Nguyen

**Introduction:** Treatments for juvenile idiopathic arthritis (JIA) have evolved at an accelerated pace over the last decade. It is important to document these changes and whether there has been concurrent improvements in patient outcomes.

**Objectives:** To compare JIA treatments and outcomes in the first year after diagnosis from two cohorts in Canada, the 2005-10 Research in Arthritis in Canadian Children emphasizing Outcomes (ReACCh-Out) cohort and the 2017-21 Canadian Association of Pediatric Rheumatology Investigators (CAPRI) Registry cohort.

**Methods:** To enhance the validity of the comparison, we included only patients recruited within three months of JIA diagnosis, used the same outcome criteria in all subjects, and compared Kaplan-Meier estimates at 70 weeks in both cohorts (1 year plus window for data capture). Inactive disease was defined as per Wallace criteria and inactive and minimally active disease were defined according to clinical Juvenile Arthritis Disease Activity Score 10 (cJADAS10) using the recently revised cut-offs [1].

**Results:** The 2005-10 cohort included 1128 subjects. The 2017-21 cohort 721. The cohorts were comparable in proportion of JIA categories and median cJADAS10 at subject enrollment, 8.2 (IQR 4.4, 14.6) and 8.0 (4.5, 13), respectively. Median age at disease onset was 8.5 years (3.2-12.2) and 8 years (4.1-13.2). The use of disease-modifying antirheumatic drugs (DMARDs) and biologics increased substantially (Table 1). By 70 weeks after diagnosis, 6% of subjects had started a biologic in the 2005-10 cohort compared to 26% in the 2017-21 cohort. The proportion of patients attaining inactive disease increased from 63% to 84% by Wallace Criteria.

**Conclusion:** The main changes observed in JIA treatments in Canada between 2005-2010 and 2017-2021 were increased use of DMARDs and biologics. There was a concomitant increase in attainment of inactive and minimally active disease within 70 weeks of diagnosis, suggesting more aggressive treatment was associated with improved outcomes.

[1] Trincianti C, et al. Arthritis Rheumatol 2021; 73: 1966-75.

**Patient Consent:** Yes, I received consent

**Disclosure of Interest**: None declared

**Table 1 (abstract P047).** Kaplan-Meier estimates, % (95% CI), for use of selected medications and attainment of inactive or minimally active disease within 70 weeks of JIA diagnosis

Group	Cohort	DMARD	Biologic	Prednisone	Joint Injection	Inactive Wallace	Inactive ^1^ cJADAS10	Minimal ^2^ cJADAS10
All categories	2005-10	43 (40,47)	6 (5,7)	16 (14,18)	43 (40,46)	63 (60,66)	50 (47,53)	69 (66,72)
	2017-21	60 (56,64)	26 (23,30)	16 (13,19)	36 (32,40)	84 (80, 87)	69 (65,73)	84 (81,87)
Oligoarthritis	2005-10	21 (18,26)	2 (1,4)	5 (3,7)	56 (51,61)	78 (74,82)	56 (51,61)	75 (71,79)
	2017-21	46 (39,52)	10 (6,15)	6 (3,10)	55 (49,62)	90 (85,93)	73 (67,79)	89 (85,93)
Polyarthritis RF negative	2005-10	78 (72,84)	6 (4,10)	18 (13,24)	38 (32,45)	53 (47,60)	46 (39,56)	64 (58,71)
	2017-21	84 (76,90)	41 (32,52)	22 (16,31)	29 (21,39)	86 (78,92)	80 (72,87)	88 (81,93)
Systemic	2005-10	63 (51,75)	18 (10,30)	77 (66,87)	8 (3,18)	68 (56,79)	62 (50,74)	80 (69,89)
	2017-21	47 (29,69)	66 (49,83)	83 (65,95)	--	91 (76,98)	78 (59,92)	85 (67,96)

^1^cJADAS scores of ≤1.1 and ≤ 2.5, ^2^≤ 4 and ≤ 5, for subjects with oligo- and polyarthritis, respectively

### P048. Tofacitinib in the therapy of different kind of pediatric rheumatic deseases: real clinical practice experience of a single center

#### I. Nikishina^1^, S. Arsenyeva^1^, V. Matkava^1^, M. Kaleda^1^, S. Salugina^1^, E. Fedorov^1^, A. Shapovalenko^1^, T. Pachkoria^1^

##### ^1^Paediatric, V.A. Nasonova Scientific Research Institute of Rheumatology, Moscow, Russian Federation

###### **Correspondence:** I. Nikishina

**Introduction:** Tofacitinib (TOFA) is new therapy approach in pediatric rheumatology for management children with different rheumatic diseases (RD). In Russia TOFA was approved from September 2021 for the treatment of polyarticular juvenile idiopathic arthritis (JIA). The unique mechanism of action of Janus-kinases suggests that TOFA can be a useful option in the treatment of various RD in children.

**Objectives:** To evaluate efficacy and safety of TOFA in children with different kind of RD.

**Methods:** We analyzed data base of 52 patients (pts) who were treated by TOFA since 2018 to 2022. The data includes JIA, juvenile ankylosing spondylitis (JAS), chronic recurrent multifocal osteomyelitis (CRMO), juvenile dermatomyositis (JDM), and rare genetic syndromes such as fibrodisplasia ossificans progressive (FOP), STING-Associated Vasculopathy with onset in Infancy (SAVI), Chronic Atypical Neutrophilic Dermatosis with Lipodystrophy and Elevated Temperature (CANDLE), Blau syndrome, camptodactyly-arthropathy-coxa vara-pericarditis syndrome (CACP). All cases of «off-label» TOFA use were approved by Local Ethic Committee.

**Results:** The prospective analysis includes data of 52 pts (18/35% boys; 34/65% girls). The largest number of pts had JIA: 26/63% RF-negative polyarticular subtype of JIA (7 was combined with psoriasis, 3-with uveitis, 2-with Blau syndrome, 1 – with Chron’s disease, 1 – with congenital insensitive to pain and CRMO, 1 – with Down’s syndrome, 1 – with CACP syndrome), RF-positive – 2/3%. 4/8% were diagnosed as systemic JIA (2 pts were in clinical trials, 2 – without current systemic features). JAS was presented in 3 pts, 1 of them was associated with CRMO. 1 pt has JDM with severe course of the disease. All other pts (16/31%) had rare genetic disorders: 2 pts with SAVI syndrome and 1 pt with CANDLE were treated by TOFA as first line with excellent effects (relief of fever, reduction of skin manifestations and pulmonary lesions); 13 pts with extremely rare genetic disease FOP (TOFA was administered due to strong uncontrolled progression of heterotopic ossification). Presence of synovitis, sacroiliitis and axial damage confirmed by X-ray/MRI/CT allowed us to establish alternative diagnosis of JAS in 6 pts and JIA in 7 cases for legal prescription. The dosage of TOFA was up to 5 mg twice a day. All JIA pts received methotrexate. The mean duration of TOFA therapy is 10.8 months (from 1.5 to 43,9). The majority of TOFA administration was as a first-line treatment (27/52%), in 2-nd line – 12/23%, 3-d line – 4/7%, 4-th line – 3/6%, 5-th line – 5/10%, 6-th line – 1/2%. Approximately all patient had a good response on TOFA therapy with relief of active arthritis and uveitis, significant reduction of psoriasis skin lesions. In most FOP pts new nodes formation immediately stopped and improvement of motions was registered. Also, we noticed regression of sacroiliitis and coxitis activity by MRI under TOFA therapy Drug tolerance was good in 51/98% pts. In 1 pt TOFA therapy was withdrawn due to adverse event presented by extended maculopapular skin rash which appeared 3 days after TOFA initiation. 3 more withdrawals were due to primary inefficacy (2 pts with sJIA from clinical trial, 1 received TOFA as 5-th line therapy).

**Conclusion:** We found that TOFA is highly efficient, well-tolerated medicine which may be a promising option for the difficult to treat variants of JIA (including psoriasis and CRMO- associated) and rare autoinflammatory diseases, such as FOP, Blau syndrome, CANDLE, SAVI, CACP. The anti-inflammatory action of TOFA seems to be preferable to the direct anti-cytokine effect of most biologics. The further study of the therapeutic potential of JAK-kinase inhibitors in pediatric RD is needed.

**Patient Consent:** Yes, I received consent

**Disclosure of Interest**: None declared

### P049. Drug survival of abatacept therapy in children with rheumatic diseases: 10 years experience in a single center

#### S. Arsenyeva, I. Nikishina, V. Matkava, M. Kaleda, A. Shapovalenko

##### Paediatric, V.A. Nasonova Scientific Research Institute of Rheumatology, Moscow, Russian Federation

###### **Correspondence:** S. Arsenyeva, I. Nikishina

**Introduction:** Among the wide spectrum of Biologics used in pediatric rheumatology, abatacept (ABA) has a special place, which differs from other Biologics with direct anti-cytokine action. In addition to therapy with ABA in patients (pts) with juvenile idiopathic arthritis (JIA) in real clinical practice there is experience of its use “off-label” in such systemic rheumatic diseases (RD) as juvenile systemic scleroderma (jSS), juvenile dermatomyositis (JDM), systemic lupus erythematosus with juvenile onset (jSLE) and its overlaps.

**Objectives:** To evaluate the drug survival of ABA therapy in children with different RD with focus on adverse events (AE).

**Methods:** The study based on cohort of pts who were treated by ABA from 2010 to 2021 in our clinic. These pts are fulfilled the criteria for JIA and the criteria of above-mentioned RD. Data of the disease course were used to estimate drug survival with Kaplan-Meier and calculate AE rates.

**Results:** The retrospective analysis includes data of all pts, who received ABA in our center, 205 pts in total. The largest number of patients were presented by RF-negative polyarticular subtype of JIA - 131/64%, RF-positive – 40/20%, persistent oligoarthritis – 8/4% (6 of them with uveitis). 15/7% were diagnosed as systemic JIA without current systemic features. 36/18% pts suffered from active uveitis. All other pts (11/5%) had systemic RD (jSS – 5, JDM – 3, jSLE – 3). In group of JIA pts we identified overlap-syndrome with JDM, jSLE, jSS features in 31/15% cases. 28/14% had Sjogren’s syndrome. The average age of disease onset was 5.9 years (from 1.25 to 17.2). The average age of ABA initiation (first-line) was 10,7 years (from 2 to 17.8 years). The mean duration of disease was 4.8 years. The mean duration of ABA treatment was 4,4 years. The majority of pts received ABA as first-line therapy (164), 2-nd line – 29, 3-rd line – 8, 4-th line – 4. There were 71 cases of ABA withdrawals. The most often reasons for drug discontinuation were inefficacy – 48/ 68%, secondary mostly (in 1^st^ line -30 from 164 (18%), 2^nd^ line- 10/29 (34%), 3^rd^ line- 4/8 (50%), 4^th^ line-4/4 (100%). Non-medical «organizational» problems led to the cancellation of therapy in 12/17%. Just in 1 pt ABA was stopped because of remission. AE were as reason ABA withdrawn in 10/15% (3 pts due to post-infusion reactions, 2 pts (the both with early oligoarticular onset) developed uveitis de-novo, one had verruca vulgaris, 2 - psoriasis de novo). In 1 girl we observed the development of cryptogenic epilepsy after 3-year ABA treatment, but therapy was continued. In 2 cases ABA was cancelled due to multiple sclerosis development. The first pt had 70-90% ACR response, however in 2 years of ABA-treatment the psoriasis was developed, ABA was continued. After 4 years of the therapy (at the age of 13) neurological symptoms appeared (headache, loss of sensitivity, ataxia, visual field defect) and multiple sclerosis was diagnosed. In 2-nd pt CNS demyelination was verified after 2 years of ABA therapy with appearance of dizziness, unilateral numbness. MRI-evidence was observed in both cases. ABA treatment survival at 1 year for the 1^st^ line was 88% and 2-4^th^ lines 84%, at 5 years 60% and 45% respectively. There were not significant differences in drug survival according to kind of RD.

**Conclusion:** Our data showed great experience of ABA use in wide spectrum of pediatric RD. ABA often is the front-line therapy for certain subtype of JIA, especially in overlap-syndrome. It may be good option for the some pts with jSS, JDM, jSLE despite of indication «off label». Drug survival of ABA was higher in biologics-naïve patients. ABA had satisfactory safety profile in the long-term period, but rare adverse events, such as CNS demyelination and uveitis or psoriasis de novo were found.

**Patient Consent:** Yes, I received consent

**Disclosure of Interest**: None declared

### P050. Assessment of liver stiffness and steatosis in juvenile idiopathic arthritis patients treated with methotrexate

#### C. Niyasom^1^, C. Lertudomphonwanit^1^, S. Getsuwan^1^, S. Vilaiyuk^1^, A. Sobhonslidsuk ^2^, P. Kaewduang^2^, S. Soponkanaporn^1^

##### ^1^Pediatric, ^2^Medicine, Faculty of Medicine Ramathibodi Hospital, Mahidol University, Bangkok, Thailand

###### **Correspondence:** C. Niyasom

**Introduction:** Monitoring serum transaminases is unreliable to detect liver stiffness and steatosis in patients with juvenile idiopathic arthritis (JIA) who have been treated with methotrexate. Transient elastography (TE) with controlled attenuation parameter (CAP) (FibroScan®) has emerged as a non-invasive tool to assess liver stiffness and steatosis.

**Objectives:** To evaluate the prevalence and predictors of liver stiffness and steatosis in JIA patients treated with MTX by using TE with CAP.

**Methods:** A cross-sectional study was conducted at Ramathibodi Hospital. Included subjects were JIA patients aged 1-18 years who received methotrexate (MTX) for at least 1 year, while patients with known chronic liver disease were excluded. Clinical features, medication information, and laboratory data were collected. TE measured liver stiffness and reported as liver stiffness measurement (LSM) with a cut-off >7 kilopascal (kPa) indicating significant liver stiffness. Steatosis was diagnosed if CAP >225 decibels/meter (dB/m).

**Results:** Sixty JIA patients were enrolled with a median age (IQR) of 12.8 (10.6–15.0) years and a median disease duration (IQR) of 4.2 (2.1–7.8) years. The most common JIA subtype was systemic JIA (28.3%), followed by rheumatoid factor-positive polyarthritis (25%), and enthesitis-related arthritis (18.3%). A cumulative median (IQR) dose of MTX and steroids was 3,768 (1,806–6,466) mg, and 1,563 (0–7,694) mg, respectively. A median (IQR) duration of MTX and steroid usage was 45 (22–85) months, and 5.3 (0–29) months, respectively. None of them had significant liver stiffness with the median LSM (IQR) was 4.10 (3.43–4.58) kPa. Thirteen patients (21.7%) were diagnosed with steatosis. There was a significant higher median body mass index (BMI) Z-score in patients with steatosis (0.86, IQR 0.23–1.44) when compared to non-steatosis (-0.32, IQR -0.95–0.38) (p=0.007). Median cumulative dose of MTX in steatosis patients was 5,405 mg (IQR 2,760–9,761) which were significantly higher than in non-steatosis patients (3,370 mg, IQR 1,438–5,614) (p=0.017). Similarly, there were significantly higher median cumulative dose of steroids in steatosis patients 1,732 mg (IQR 0–26,657) VS 1,410 mg (IQR 0–6,545) than in non-steatosis patients (p=0.069). From multivariable logistic regression analysis, the only predictor of steatosis was BMI Z-score (OR 2.78 [95%CI 1.23– 6.13], p=0.013).

**Conclusion:** Long-term low-dose MTX usage with median duration of 45 months does not increase the risk of liver stiffness in JIA patients. Since levels of liver enzymes can be normal in patients with steatosis, caution and monitoring for steatosis using TE in JIA patients with high BMI Z-score might be useful during MTX treatment.

**Patient Consent:** Yes, I received consent

**Disclosure of Interest**: None declared

### P051. Cognitive impairment associated with higher C-reactive protein values in patients with juvenile idiopathic arthritis: a prospective cohort study

#### F. Ortiz-Márquez^1^, R. Redondo-Rodríguez^1^, L. Martín-Pedraz^2^, C. Padilla-Leiva^3^, G. Díaz-Cordovés^1^, R. Galindo-Zavala^2^, E. Núñez-Cuadros^2^

##### ^1^Instituto de Investigación Biomédica de Málaga (IBIMA). UCG de Reumatología , Hospital Regional Universitario de Málaga, ^2^UCG Pediatría, Hospital Materno-Infantil de Málaga, ^3^Universidad de Málaga, Málaga, Spain

###### **Correspondence:** F. Ortiz-Márquez^1^

**Introduction:** Cognitive impairment is a comorbidity that affects rheumatological patients. Despite this, it hasn’t been studied in Juvenile Idiopathic Arthritis patients yet.

**Objectives:** Prospectively evaluate changes in the cognitive function of patients with juvenile idiopathic arthritis (JIA) and associated factors.

**Methods:** Design and protocol: We performed a prospective cohort study with JIA patients that participated in a previous cross-sectional study (2019) to evaluate cognitive function. After 24 months, the patients were administered the same test battery previously used through an established protocol, and data was collected from their clinical histories. The neuropsychological tests were corrected by a neurologist and neuropsychologist.

Study population: Inclusion criteria: Patients aged ≥16 years with JIA classified according to the criteria of ILAR 2001. Patients with inflammatory or rheumatic diseases other than JIA, previous neurological disease not associated with the course of JIA, and patients with scores lower than the normal in the manual skill test were excluded. Outcomes: The main variable was cognitive impairment, defined as worsening of ≥2 scaled points after 24 months (V24) in any of the subtests used to evaluate each cognitive area in the Wechsler Adult Intelligence Scale (WAIS). The evaluated cognitive domains and their respective subtests were: Attention/concentration (Digit Span); verbal function (Vocabulary); visuospatial organization (Block Design); working memory (Letter-Number Sequencing); problem-solving (Similarities). Depression was evaluated by The Beck Depression Inventory-II (BDI-II): minimal (0-13), mild (14-19), moderate (20-28), and severe (29-63). Other variables: Clinical-epidemiological characteristics; treatments; and inflammatory activity evaluated as the C-reactive protein average (CRP) and JADAS-27 along the 2 years of follow-up.

Statistical analysis: Descriptive analysis, followed by χ2 and paired T-test. Multivariate analysis to identify independent variables associated with impairment of cognitive function in JIA.

**Results:** Fifty two patients with JIA were included. The clinical characteristics are shown in Table 1. Fifteen patients (28.8%) showed impairment in one or more cognitive functions. The most frequent impaired cognitive functions were working memory (17.3%) and attention/concentration (9.6%); followed by verbal function (7.7%), visuospatial organization (7.7%) and problem solving (3.8%). The variables independently associated with cognitive impairment were the mean CRP along the follow-up (OR [IC 95%], 1.291 [1.002-1.663]; p=0.047), depression (OR [IC 95%], 1.178 [1.001-386]; p=0.049) and biological treatment (OR [IC 95%], 0.196 [0.039-0.978]; p=0.049). This model would explain the 45% of the cognitive impairment in JIA (R2=0.45).

**Conclusion:** Thirty percent of the patients with JIA showed cognitive impairment after 24 months of follow-up. Cognitive impairment was associated with higher inflammatory activity and depression. Biological therapy improves cognitive function.

**Patient Consent:** Not applicable (there are no patient data)

**Disclosure of Interest**: None declared

**Table 1 (abstract P051).** Clinical, laboratory, and treatment characteristics

Variable	JIA basal n=52	JIA V24 n=52	p-value
Age in years, mean (±SD)	22.8 (5.1)	25.0 (6.1)	<0.001
Sex, woman, n (%)	35 (67.3)	35 (67.3)	1.000
Evolution time JIA, months, median (IQR)	134.1 (95.6-214.2)	161.4 (103.6-220.4)	<0.001
Oligoarticular JIA, n (%)	30 (57.7)	30 (57.7)	1.000
Depression with BDI-II, n (%)	8 (15,4)	13 (25)	0.046
CRP average, median (IQR)	-	3.4 (2.9-4.2)	-
JADAS27 average, median (IQR)	-	4.0 (2.8-9.2)	-
Synthetic DMARDs, n (%)	24 (46.2)	22 (42.3)	0.785
Biological DMARDs, n (%)	28 (53.8)	31 (59.6)	0.182

### P052. Cluster analysis of patients with oligoarticular, RF negative polyarticular and undifferentiated juvenile idiopathic arthritis

#### S. Ozdemir Cicek^1^, N. Şahin^2^, A. Paç Kısaarslan^3^, M. H. Poyrazoğlu^3^

##### ^1^Pediatric Rheumatology, University of Health Sciences, Kayseri City Research and Training Hospital, Kayseri/Melikgazi, ^2^Pediatric Rheumatology, Kocaeli Derince Training and Research Hospital, Kocaeli, ^3^Pediatric Rheumatology, Erciyes University, Kayseri/Melikgazi, Turkey

###### **Correspondence:** S. Ozdemir Cicek

**Introduction:** In recent years, attempts have been made to classify JIA into more homogeneous clinical groups.

**Objectives:** We planned to classify the patients with oligoarticular, RF- polyarticular and undifferentiated groups according to ILAR criteria, according to their clinical and laboratory findings.

**Methods:** Two hundred three patients with oligoarticular, RF- polyarticular JIA and undifferentiated arthritis included to the study. RF+ polyarticular JIA, enthesitis-related arthritis, psoriatic arthritis and systemic JIA patients were not included. Eighteen clinical and laboratory variables evaluated with TwoStep Cluster analysis. Categorical variables were sex, affected joints(hip, knee, ankle, shoulder, elbow, wrist, small joints of the hand and foot, cervical vertebra and temporomandibular joint), laboratory findings which determines the JIA subtype(ANA, RF and HLA B27 positivity), continuous variables were the age of disease onset, the number of affected joints, ESR and CRP values at disease onset. Clinical and laboratory features of the resulted clusters were then compared with each other.

**Results:** Two hundred three JIA patients included to the study. The median age of patients was 13 (2.5-21.07) years, the age of diagnosis was 7 (1-16) years and 133 (65.5%) of the patients were female. Two clusters were generated as the result of cluster analysis. Cluster 1 had 94 (46.3%) and cluster 2 had 109 (53.7%) patients. The most important indicators in differentiating of the two clusters were small joint involvement, the number of involved joints, wrist, knee and elbow arthritis.

In the first cluster, small joint involvement was observed, the number of affected joints was higher, TMJ, shoulder, wrist, elbow and ankle arthritis were higher, and initial acute phase reactants were found to be higher than the second cluster. Corticosteroids, DMARDs and biological treatments were at higher rates in the first cluster, and the remission rates at twelfth months and last visit were lower according to the second cluster. All of the patients in the second cluster had knee involvement and IAS was used more frequently compared to first cluster.

**Conclusion:** Our results classified current oligoarticular, RF negative polyarticular and undifferentiated arthritis subgroups patients into two clusters. Small joint, wrist, elbow involvement and the number of involved arthritis were the most important factors for differentiating two groups.

**Patient Consent:** Yes, I received consent

**Disclosure of Interest**: None declared

**Table 1 (abstract P052).** Characteristics of all JIA patients and comparisons of cluster 1 and cluster 2

Variables	All patients (n=203)	Cluster 1 (n=94)	Cluster 2 (n=109)	P value
Number of the patients	203 (100.0)	94 (46.3)	109 (53.7)	-
Sex/Female	133 (65.5)	68 (72.3)	65 (59.6)	0.058
Age at disease diagnosis, years	7 (1-16)	7 (1-16)	7 (1.5-15)	0.353
ESR at diagnosis, mm/h	17 (1-100)	23.5 (2-100)	10 (1-97)	**0.002**
CRP at diagnosis, mg/L	3.7 (0.03-233)	6.8 (0.04-125)	3.3 (0.03-233)	**0.022**
The number of affected joints	2 (1-26)	5 (1-26)	2 (1-6)	**<0.001**
**Affected joints**				
Hip	24 (11.8)	14 (14.9)	10 (9.2)	0.208
Knee	168 (82.8)	59 (62.8)	109 (100.0)	**<0.001**
Ankle	70 (34.5)	49 (52.1)	21 (19.3)	**<0.001**
Shoulder	6 (3.0)	6 (6.4)	0 (0.0)	0.009*
Elbow	33 (16.3)	33 (35.1)	0 (0.0)	**<0.001**
Wrist	47 (23.2)	47 (50.0)	0 (0.0)	**<0.001**
Small joints	52 (25.6)	52 (55.3)	0 (0.0)	**<0.001**
Cervical vertebrae	3 (1.5)	3 (3.2)	0 (0.0)	0.098*
TMJ	5 (2.5)	5 (5.3)	0 (0.0)	**0.020**
**Treatments**				
İAE	106 (52.2)	34 (36.2)	72 (66.1)	**<0.001**
Steroids	98 (48.3)	57 (60.6)	41 (37.6)	**0.001**
DMARD	188 (92.6)	92 (97.9)	96 (88.1)	**0.008**
Biologics	88 (43.3)	58 (61.7)	30 (27.5)	**<0.001**
Remission at 12 months	100 (49.3)	36 (38.5)	64 (58.7)	**0.004**
Relapse of arthritis	124 (61.1)	62 (66.0)	62 (56.9)	0.186
Remission at last visit	154 (75.9)	63 (67.0)	91 (83.5)	**0.006**

*Fisher’s Exact Test

### P053. How to diagnose juvenile psoriatic arthritis? Multicenter prospective observational study in children with suspected diagnosis

#### N. Palmou-Fontana^1^, A. Garcia-Rogero^2^, C. Redondo-Figuero^1^, A. Lopez-Sundh^1^, P. Mesa-del-Castillo^3^, G. Diaz-Cordoves^4^, B. Magallares-Lopez^5^, M. J. Cabero-Perez^6^, M. A. Gonzalez-Gay^1^, P. Collado-Ramos^7^ on behalf of Sociedad Española de Reumatología Pediátrica (SERPE)

##### ^1^Servicio de Reumatologia, Hospital Universitario Marques de Valdecilla, Santander, ^2^Gerencia de Atencion Primaria, Servicio Cantabro de Salud, Castro Urdiales, ^3^Servicio de Reumatologia, Hospital Universitario Virgen de la Arrixaca, Murcia, ^4^Servicio de Reumatologia, Hospital Regional Universitario, Malaga, ^5^Servicio de Reumatologia, Hospital de la Santa Creu i Sant Pau, Barcelona, ^6^Servicio de Pediatría, Hospital Universitario Marques de Valdecilla, Santander, ^7^Servicio de Reumatologia, Hospital Universitario Severo Ochoa, Madrid, Spain

###### **Correspondence:** N. Palmou-Fontana

**Introduction:** Juvenile idiopathic arthritis (JIA) constitutes a heterogeneous group of inflammatory joint diseases. The International League of Associations for Rheumatology (ILAR) classifies and defines seven subtypes of JIA by specific and non-specific clinical features with exclusions. Among these diseases is Juvenile Psoriatic Arthritis (JPA).

There are two pediatric diagnostic classifications based on clinical criteria: ILAR classification criteria and Vancouver classification criteria. Classification of JIA has been a great debate since this entity disappeared. (1) The CASPAR criteria are standard in adults. These criteria have a specificity of 91.4% and a sensitivity of 98.7%, making diagnostic classification easier. However, the peculiarities of childhood cause some differences to consider.

**Objectives:** To compare the performance of the three different PsJIA classification criteria (ILAR, Vancouver and CASPAR criteria) in children identified as such in the routine rheumatology Clinic.

**Methods:** Multicenter prospective observational study started in 2020. Patients seen in the pediatric rheumatology outpatient clinic with suspected PsJIA diagnosis were consecutively included. Sociodemographic, clinical characteristics and family history of Psoriasis were collected to assess compliance with diagnostic classification criteria (ILAR, Vancouver, and CASPAR).

**Results:** Thirty-two children were included, predominantly female (24 girls). The median age at diagnosis was 13.5 (IQR: 4.5). Clinical onset was predominantly oligoarticular (19, 59.5%), followed by polyarticular (n:5, 18.6%), monoarticular (n:5, 18.6%), and onychopathy (n:2, 6.2%). Cutaneous Psoriasis was present in 20 children (62.5%). Family history of Psoriasis was recorded in 24 patients, 1st degree in 19 patients (54%), and a second degree in 5 (15.6%). Among the extra-articular manifestations, dactylitis stood out (n:14, 3.8%). Twelve patients (37,5%) had nail involvement. Rheumatoid factor (RF) was negative in 100% of the children, ANAS were positive in 11 children (34.4%), whereas HLAB27 was positive in 3 children (9.4%). Twenty-seven children met the VANCOUVER criteria: twenty children, defined dco (62.5%) and 7 (21.9%) probable diagnosis. Twenty children (62.5%) met the ILAR criteria. Twenty-seven (84.4%) children were diagnosed according to the CASPAR criteria. The diagnostic agreement between the ILAR and CASPAR criteria on the one hand, and between the ILAR and Vancouver criteria on the other hand, was weak (K=0.17 and K= 0.41). In contrast, the agreement was total (K=100) between the CASPAR and Vancouver criteria.

**Conclusion:** Despite minimal changes between the Vancouver and ILAR criteria, the ILAR exclusion criteria limit the diagnosis of PsA in childhood. Given that CASPAR and Vancouver criteria were able to detect a more significant number of patients with PsA in our series (predominantly adolescents), the application of the CASPAR Criteria in the subgroup of children with late onset could be considered. (1)

(1) Martini A, Ravelli A, Avcin T, et al, for the Pediatric Rheumatology International Trials Organization (PRINTO). Towards New Classification

**Patient Consent:** Yes, I received consent

**Disclosure of Interest**: N. Palmou-Fontana Consultant with: ABBVIE, AMGEN, PFIZER, Speaker Bureau with: ABBVIE, AMGEN, PFIZER, A. Garcia-Rogero Speaker Bureau with: GSK, C. Redondo-Figuero: None declared, A. Lopez-Sundh: None declared, P. Mesa-del-Castillo: None declared, G. Diaz-Cordoves: None declared, B. Magallares-Lopez: None declared, M. J. Cabero-Perez: None declared, M. A. Gonzalez-Gay: None declared, P. Collado-Ramos: None declared

### P054. Early cervical involvement in children with juvenile idiopathic arthritis and its variants: a frequently neglected domain in paediatric pathology

#### E. Pardo Campo^1^, S. Burguer^1^, M. Pino Martinez^1^, I. Braña Abascal ^1^, S. Murias Loza^2^, J. Rodriguez^2^, S. Alonso Castro ^1^, S. Fernandez Aguado^1^, M. Alperi Lopez^1^, R. Queiro Silva^1^

##### ^1^Rheumatology, ^2^Pediatrics, Hospital Univ Central De Asturias, Oviedo, Spain

###### **Correspondence:** E. Pardo Campo

**Introduction:**

Cervical spine involvement is often unrecognized in the early stages of juvenile idiopathic arthritis

(JIA), as it is a rare manifestation. We know that JIA is the most common chronic rheumatic disease

in childhood.

However, cervical involvement is a rare manifestation in the early stages of the disease, and is

extremely infrequent as the only manifestation.

It is for this reason that the exact frequency, clinical features and evolution of these patients are not

yet known.

It has been described as a late complication and is considered to have a poor prognosis.

**Objectives:** Therefore, our aim is to describe the clinical characteristics of patients with JIA who have experienced cervical involvement as a manifestation at the onset of the disease.

**Methods:**

An observational study was performed on patients with JIA who presented cervical involvement as

the form of onset of the disease. Data was obtained from a total of 75 patients with JIA being

followed by the Pediatric Rheumatology Unit of the Hospital Central de Asturias. Hospital Central de Asturias.

We have reviewed the clinical histories and selected those with cervical involvement as a form of onset.

**Results:**

Three cases of JIA were detected in which cervical involvement was the form of onset of the

disease in three different types of JIA. (Table 1)

**Conclusion:**

JIA in its different forms should be considered as a differential diagnosis in children presenting with

cervical pain, cervical limitation or stiffness.

This manifestation can go unnoticed in our patients and delay diagnosis. We should consider its

occurrence in different categories within JIA.

MRI can be of great help in the diagnosis of these patients. Early targeted therapy with anti-TNF

inhibitors can help in the remission of cervical symptomatology.

**Patient Consent:** Yes, I received consent

**Disclosure of Interest**: None declared

**Table 1 (abstract P054).** Number of Patients (3)

**Median Age (years)**	3.5	**Lumbar involvement**	1/3
**Sex**	Female	**Diagnostic arthrocentesis inflammatory**	3/3
**Onset of cervical involvement**	Cervical arthralgia 2/3 Limitation and stiffness 1/3	**Cervical X-ray pathological**	0/3
**Diagnosis**	Early onset Psoriatic arthritis type JIA Monophasic systemic JIA Oligoarticular JIA ANA positive.	**Pathological cervical MRI**	1/3
**Family history**	1/3 Grandfather with psoriasis.	**Pathological lumbar MRI**	1/3
**Elevated ESR and/or CRP**	3/3	**Corticotherapy**	2/3
**ANA positive**	2/3	**Methotrexate**	2/3
**HLAB27 positive**	0/3	**antiTNF**	2/3
**Acute anterior uveitis**	1/3		

### P055. Juvenile psoriatic arthritis: a case series

#### S. Pastore^1^, F. Corona^2^, A. Taddio^3^, A. Tommasini^3^

##### ^1^Institute of Maternal and Child Health IRCCS “Burlo Garofolo, ^2^Department of Medicine, Surgery, and Health Sciences, ^3^Institute of Maternal and Child Health IRCCS “Burlo Garofolo”, University of Trieste, Trieste, Italy

###### **Correspondence:** S. Pastore

**Introduction:** Juvenile psoriatic arthritis (JPsA) has been a recognized entity since the 1970s. In 1989 the Vancouver criteria were proposed to identify cases of probable or definite diagnosis. Subsequently, the Vancouver criteria were replaced with ILAR criteria, revised for the last time in 2004 and still used in clinical practice. With these criteria JpsA is permitted also in the absence of frank psoriasis in children with arthritis accompanied by two of the following: dactylitis; nails pitting or oncycholysis, or psoriasis in a first-degree relative. Recently, the Pediatric Rheumatology International Trials Organization suggested new criteria for the diagnosis of Juvenile Idiopathic Arthritis (JIA), but psoriatic arthritis subgroup is missing.

**Objectives:** To describe clinical and lab features of patients with JPsA, and therapeutic strategies.

**Methods:** We retrospectively enrolled all patients cared at the Rheumatology Service of the Institute for Maternal and Child Health IRCCS “Burlo Garofolo” (Trieste) with JPsA diagnosis according 2001 ILAR criteria. For each patient we collected: age, sex, age at onset, familiar history, number of involved joints, inflammatory indexes at onset disease, anti-nuclear antibodies (ANA), human leukocyte antigen (HLA) B27 expression, presence of enthesitis, tenosynovitis and uveitis, concomitant autoimmune diseases. We collected also treatments and treatment response assessed by Wallace remission criteria.

**Results:** 21 patients (16 F, 5 M) with juvenile psoriatic arthritis are enrolled. Seven out of 21 had a oligoarticular pattern, 14/21 had a polyarticular pattern (2/14 oligoextended). Out of 21, 3 patients had onset disease before age six. Thirteen out of 21 (62%) had high inflammatory indexes at onset disease: 7/13 had only high Erythrocyte Sedimentation Rate (ESR > 25 mm/h), 6/13 also had elevated C-Reactive Protein (CRP > 1 mg/L). Out of 21, 10 had ANA positivity and 2 had HLAB27 positivity. Seventeen out of 21 (81%) had fingers or toes dactylitis or both. Ten out of 21 patients (48%) had psoriasis, which in 9/10 appeared before arthritis. Six out of 21 (28%) had tenosynovitis of ankle. Two patients developed anterior uveitis. One of these is a girl with disease onset before the age of 6, positive ANA and HLAB27, with recurrent and symptomatic iridocyclitis unresponsive to first and second line therapies. Five patients had a concomitant autoimmune disease (3 celiac disease and 2 hypothyroidism). Seven out of 21 (33%) achieved disease remission with a modifying antirheumatic drug (DMARD): 6 with methotrexate and one with sulfazalazine. Nine out of 21 patients (43%) achieved disease remission with adalimumab; then one of these had loss of response. Three out of 21 (14%) achieved disease remission with etanercept. Two patients had a severe disease non responsive to different drugs, non-biologic and biologic DMARDs.

**Conclusion:** In our patients with psoriatic arthritis tenosynovitis is the predominant clinical feature; most patients have dactylitis. More than half of the patients have high ESR at onset disease. In most patients disease remission was achieved with biologic therapy.

**Disclosure of Interest**: None declared

### P056. The current state of global research on juvenile idiopathic arthritis

#### M. Pavlenko^1^, D. Pavlenko^2^

##### ^1^Universum Clinic, ^2^Department of Ophthalmology, Bogomolets National Medical University, Kyiv, Ukraine

###### **Correspondence:** M. Pavlenko

**Introduction:** Juvenile idiopathic arthritis is the most common chronic rheumatic disease in children.

**Objectives:** To describe the current state of research articles on juvenile idiopathic arthritis using bibliometric analysis.

**Methods:** An advanced search was performed in May 2022 within all titles in the Core Collection of Clarivate Web of Science to identify all articles on juvenile idiopathic/rheumatoid arthritis until 2021. The predominant language, h-index of the research topic, and the number of citations were identified according to the built-in Analyze Result and Citation Report. Biblioshiny application of Bibliometrix (Aria, M. & Cuccurullo, C., 2017) R-package (RStudio, PBC, Boston, MA, USA) was used for scientific production, author, authors collaboration, and citation analyses.

**Results:** Only articles, which comprised 46.7% (n=4,271) of all 9,144 publications between 1970 and 2021, were analysed. They were written by 13,018 authors, and published in 810 sources. Almost all articles were written in the English language (n=4,049; 94.8%). Singled-authored documents were rare (n=194; 4.5%) and were written by 1.1% (n=146) of all authors. The average years from publication was 14.8. The collaboration index was 3.2. There were 0.3 documents per author and 3.1 authors per document and 6.8 co-authors per document. The annual growth rate was 2.1%. The most productive year was 2021 (n=259; 6.1%). Source clustering through Bradford’s law revealed that *Journal of Rheumatology* (n=454; 10.6%; h-index=58), *Arthritis and Rheumatism* (n=253; 5.9%; h-index=83), *Pediatric Rheumatology* (n=239; 5.6%; h-index=26), *Rheumatology* (n=157; 3.7%; h-index=43), *Clinical and Experimental Rheumatology* (n=149; 3.5%; h-index=29), *Annals of the Rheumatic Diseases* (n=145; 3.4%; h-index=49), and *Clinical Rheumatology* (n=114; 2.7%; h-index=21) formed the core sources. Martini A (n=177; 4.1%**;** h-index=57), Ravelli A (n=128; 3.0%**;** h-index=49), and Ruperto N (n=112; 2.6%; h-index=48) contributed most and had the highest local impact. The most relevant affiliations included University of Toronto (n=244), University Genoa (n=199), and University Cincinnati (n=170). The h-index of the research field was 123. Totally, there were 110,668 citations with average citations per document of 25.9 and average citations per year per document of 2.0. Each publication had an average of 12.8 references. Top-5 cited papers are depicted in Table 1. The corresponding authors were most likely from the United States (n=871; 22.5%), Italy (n=328; 8.5%), and Germany (n=285; 7.4%). The United States (n=3426; 20.6%), Canada (n=1515; 9.1%), and Italy (n=1397; 8.4%) were the most productive countries. The United States, Italy, and the United Kingdom were cited mostly (30,558, 12,883, and 8,274 citations, respectively). When average article citations were considered, Italy, Canada, and Switzerland were in the top (39.28, 38.34, and 35.51 citations/article, respectively).

**Conclusion:** In this study, the current state of research related to juvenile idiopathic arthritis was quantitatively characterized. The United States was the most prolific country and its research had the highest number of total citations. University of Toronto was the most common affiliation. Italy had the highest average article citations.

**Patient Consent:** Not applicable (there are no patient data)

**Disclosure of Interest**: None declared

**Table 1 (abstract P056).** Top-5 cited publications on juvenile idiopathic arthritis

Paper	Total Citations, n	Total Citations per Year, n/year	Normalized Total Citations, n
PETTY RE, 2004, J RHEUMATOL	2691	141.6	35.2
LOVELL DJ, 2000, NEW ENGL J MED	858	37.3	16.6
SINGH G, 1994, ARTHRITIS RHEUM	676	23.3	10.3
PASCUAL V, 2005, J EXP MED	630	35.0	12.8
YOKOTA S, 2008, LANCET	537	35.8	12.6

### P057. The adulthood transition of patients with polyarticular juvenile idiopathic arthritis in the era of biologic agents

#### E. Pelechas, E. Kaltsonoudis, P. Karagianni, P. V. Voulgari, A. A. Drosos

##### Rheumatology, University of Ioannina, Ioannina, Greece

###### **Correspondence:** E. Pelechas

**Introduction:** Polyarticular JIA (rheumatoid factor positive and rheumatoid factor negative), years after the first diagnosis, can affect significantly the patients’ quality of life (1). In the pre-biological era, conventional synthetic disease-modifying anti-rheumatic drugs (csDMARDs) and glucocorticoids (GCs) were the mainstay treatments affecting patients with the development of new comorbidities and complications at a very sensitive age (2).

**Objectives:** Evaluation of the comorbidities and complications from csDMARDs and GCs in 23 patients with transition to adulthood in a tertiary university hospital in Greece.

**Methods:** 23 patients (16 women) with a mean age 32 years and a mean follow-up of 5±2 years were evaluated. Average disease duration 12±3 years. Nineteen received biologic DMARDs (17 anti-TNFα, 1 Tocilizumab and 1 abatacept), and 4 csDMARDs with or without low dose GCs. Nine of the patients due to disease duration (pre-biological) received large doses of GCs for long periods of time (>10mg for >3 years) and the rest received short-term GCs. Twelve, had uncontrolled disease (DAS28 >3,2) for at least 3 years. Due to the nature of the data the JADAS score could not be used and the DAS28 has been used instead. In addition, the SF-36 (Short Form 36 Health Survey) has been used in order to evaluate health status of the patients.

**Results:** osteoporosis resulted in 15 patients (9 due to high and prolonged GC intake and significant disease activity, and 6 only due to prolonged GC intake). Underdevelopment in 8 patients (height <1,55m), and 8 with emotional instability (all had received large doses of GCs and had significant disease activity). Two patients required total knee arthroplasty due to severe disease and one of them had to operate both knees. One patient had total hip arthroplasty due to osteonecrosis of the right hip. The SF-36 score was lower than 50% in most patients, and more specifically the components of the test with the worst scores were a) role limitations due to emotional problems (approx. 10%), b) general health (approx. 25%), c) energy/fatigue (approx. 30%). Nevertheless, the transition to the adulthood was without significant problems and the therapeutic interventions required were minimal.

**Conclusion:** the advent of biological agents has significantly improved the quality of life and comorbidities of patients with polyarticular JIA. The increasing number of biological treatment options in recent years is expected to improve further the final outcomes of the disease.

**Patient Consent:** Not applicable (there are no patient data)

**Disclosure of Interest**: None declared

### P058. Calcium intake and bone mineral density in juvenile idiopathic arthritis

#### E. Rabhi^1^, K. Maatallah^2^, H. Ferjani^2^, W. Triki^2^, D. Ben Nessib^2^, D. Kaffel^2^, W. Hamdi^2^

##### ^1^Rheumatology, ^2^Kassab orthopedics institute, Ksar Said, Tunisia

###### **Correspondence:** E. Rabhi

**Introduction:** Juvenile idiopathic arthritis (JIA) is the most common chronic rheumatic disease in childhood. It’s an inflammatory condition that can lead to bone metabolism disturbance and osteoporosis (OP). We have increasing data on the negative effect of disease activity on bone mineral density (BMD) [1]. It is therefore essential to ensure optimal management of inflammation and a balanced diet to avoid adverse consequences on the bone.

**Objectives:** Our study aimed to assess bone mineral density (BMD) and calcium intake in children with JIA.

**Methods:** We conducted a retrospective study including children fulfilling the international league against rheumatism (ILAR) 2010 criteria. We collected epidemiological, clinical, juvenile arthritis disease activity score (JADAS), daily calcium intake, and therapeutic data.

**Results:**

Among 43 patients, thirteen underwent a bone mineral density measurement and were thus enrolled. There were 9 males and 4 females. The mean age was 13.1 ± 2.8 [10-20] years. The mean duration of the disease was 4.3 ± 3.2 years [1-11]. The mean body mass index (BMI) was 19.9 ± 5.9 [14-35.25]. The mean daily calcium intake was 574 ± 5.97 mg [252-725]. The mean JADAS was 2.7 ± 2.48 [0-6].

The main bone density was 0.975 ± 0.124 [0.73 -1,14] g/cm2 and the Z score ranged from -2.5 to 1.5 standard deviations (SD). Seven children had a low bone density (Z score<-2SD). Four patients had a history of bone fractures.

Calcium intake was significantly correlated with BMD (p<0.05). However, there was no association between BMD and BMI (p=0.23) or JADAS (p=0.4).

Among 43 patients, thirteen underwent a bone mineral density measurement and were thus enrolled. There were 9 males and 4 females. The mean age was 13.1 ± 2.8 [10-20] years. The mean duration of the disease was 4.3 ± 3.2 years [1-11]. The mean body mass index (BMI) was 19.9 ± 5.9 [14-35.25]. The mean daily calcium intake was 574 ± 5.97 mg [252-725]. The mean JADAS was 2.7 ± 2.48 [0-6]. The main bone density was 0.975 ± 0.124 [0.73 -1,14] g/cm2 and the Z score ranged from -2.5 to 1.5 standard deviations (SD). Seven children had a low bone density (Z score<-2SD). Four patients had a history of bone fractures. Calcium intake was significantly correlated with BMD (p<0.05). However, there was no association between BMD and BMI (p=0.23) or JADAS (p=0.4).

**Conclusion:**

Our study showed that more than half of the patients had a low bone density and all of them had, according to the recommendation of the international osteoporosis foundation, a low calcium intake. These results emphasize the need to screen for calcium deficiency intake in order to avoid the aggravation of bone fragility.

Our study showed that more than half of the patients had a low bone density and all of them had, according to the recommendation of the international osteoporosis foundation, a low calcium intake. These results emphasize the need to screen for calcium deficiency intake in order to avoid the aggravation of bone fragility.

References

1. John Burnham, Justine Shults, Sarah E Dubner, Harjeet Sembhi, Babette S Zemel, Mary B Leonardi. Bone density, structure, and strength in juvenile idiopathic arthritis: Importance of disease severity and muscle deficits. 2008 Aug;58(8):2518-27

**Disclosure of Interest**: None declared

### P059. Defining KLRB1/CD161 expression in synovial fluid immune cells in patients with juvenile idiopathic arthritis using single-cell RNA sequencing

#### E. Ralph^1,2,3^, Y. Sanchez-Corrales^3,4^, T. Xenakis^3,4^, V. Alexiou^1,2,3^, C. Bolton^1,2,3^, S. Castellano^3,4^, L. Wedderburn^1,2,3^

##### ^1^Infection, Immunity and Inflammation Department, UCL Great Ormond Street Institute of Child Health, University College London, ^2^Centre for Adolescent Rheumatology Versus Arthritis at UCL University College London Hospital (UCLH) and Great Ormond Street Hospital (GOSH), ^3^NIHR Great Ormond Street Hospital Biomedical Research Centre, ^4^Genetics and Genomic Medicine Department, UCL Great Ormond Street Institute of Child Health, University College London, London, United Kingdom

###### **Correspondence:** E. Ralph

**Introduction:** CD161 is a C-type lectin receptor encoded by the gene *KLRB1* and is expressed on a variety of immune cells. It has been shown that CD161+ cells are highly enriched in the synovial fluid of Juvenile Idiopathic Arthritis (JIA) patients, with CD161 expressed on several cell populations including Th17, Th17/1, and some Th1 cells (‘ex-Th17’ cells)^1^, regulatory CD4+ T cells^2^, some CD8 cells (e.g. MAIT cells), NK cells and innate lymphoid cells (ILC)^3^. Previous studies have generally relied on pre-selected populations and/or bulk studies, often using flow cytometry. Thus, there has not been a comprehensive survey of *KLRB1*/CD161 expression in the different cell types present in synovial fluid with single-cell resolution.

**Objectives:** To map the diversity of synovial inflammatory cells expressing CD161 using single-cell RNA sequencing to define expression of CD161 in immune populations in the joints of patients with JIA.

**Methods:** Single-cell RNASeq analysis was carried out on synovial fluid mononuclear cell (SFMC) samples of JIA patients (n=6), targeting 50,000 cells per patient. Gene expression and TCR libraries were prepared using the Chromium Single Cell 5' Reagent Kit (v2, Dual Index) (10X Genomics) and sequenced on a NovaSeq 6000 (Illumina). Sequencing reads were pre-processed and aligned to the GrCh38 reference genome using Cell Ranger v6 (10x Genomics). Quality control, downstream analysis and average gene expression was performed using Seurat v4. Cell type annotation was performed using a single-cell multimodal PBMC reference^4^. Normalised data counts were used to calculate average gene expression per cell type. Units are transcripts per million (TPM) defined as TPM = (number of counts mapped to a gene x 10^6) / Total number of counts per cell).

**Results:***KLRB1* expression was detected in 32% of the total SFMCs and was enriched in 7 of 30 clusters. Expression of *KLRB1* was highest in MAIT cells, with 98.9% of these cells expressing *KLRB1*, with an average expression level of 1903 TPM. The cluster identified as cytotoxic CD4+ T cells also expressed high levels of KLRB1, (89.8%, 1099 TPM), as did ILCs (80.3%, 1138 TPM), gamma delta T cells (70.4%, 911 TPM), natural killer (NK) cells (76.9%, 833 TPM), NK CD56^bright^ cells (70.1%, 697 TPM), and proliferating NK cells (58.8%, 314 TPM ). Central memory, effector memory and proliferating CD4+ T cells, as well as effector memory, naïve and proliferating CD8+ T cells, and regulatory T cells also expressed *KLRB1*, but at lower levels.

**Conclusion:** We have generated a single cell map of CD161-expressing populations in JIA synovial fluid cells. We have shown that *KLRB1* is expressed in a variety of immune cells and quantify cell expression heterogeneity that has not been previously described. Future work will investigate the expression of CD161 on CD4+ T cell subsets, its correlation with inflammatory markers and clinical outcomes.

**REFERENCES**

1. Nistala K. *et al.* Th17 plasticity in human autoimmune arthritis is driven by the inflammatory environment. Proc Natl Acad Sci U S A. 2010 Aug 17;107(33):14751-6.

2. Pesenacker A.M. *et al.*, CD161 defines the subset of FoxP3+ T cells capable of producing proinflammatory cytokines. Blood. 2013 Apr 4;121(14):2647-58.

3. Del Castillo Velasco-Herrera, M. *et al.* A novel innate lymphoid cell delineates childhood autoimmune arthritis. bioRxiv 416784.

4. Hao Y. *et al.*, Integrated analysis of multimodal single-cell data. Cell. 2021 Jun 24;184(13):3573-3587.e29.

**Disclosure of Interest**: E. Ralph Grant / Research Support with: ER holds a personal Fellowship from NIHR Great Ormond Street Hospital Biomedical Research Centre, Y. Sanchez-Corrales: None declared, T. Xenakis: None declared, V. Alexiou: None declared, C. Bolton: None declared, S. Castellano: None declared, L. Wedderburn Grant / Research Support with: LW is a PI for the CLUSTER Consortium. The CLUSTER Consortium is supported by grants from MRC, Versus Arthritis and GOSCC and has partnerships with AbbVie, GSK, UCB, Sobi and Pfizer inc. LW declares in-kind contributions to CLUSTER by AbbVie, GSK, UCB, Sobi and Pfizer inc and non-renumerated collaborations, unrelated to this work, with Lilly and Novartis.

### P060. Juvenile arthritis or recessive multiple epiphyseal dysplasia – differential diagnosis

#### A. Kozhevnikov, V. Kenis, E. Melchenko, A. Ramazanova

##### National Medical Children’s Orthopedics and Trauma Surgery Research Center Named after H. Turner, Saint Petersburg, Pushkin, Russian Federation

###### **Correspondence:** A. Ramazanova

**Introduction:** Chronic undifferentiated progressive arthropathy (CUPA) of children is a large group of musculoskeletal diseases. A form of juvenile arthritis (JIA), the clinical finding of which includes not only chronic synovitis, but also progressive contractures of several joints, requires differential diagnosis with the genetic disorders e.g., skeletal dysplasia. Frequent misinterpretations of the results of examinations of children with recessive multiple epiphyseal dysplasia (rMED/MED4, gene SLC26A2 Mut, OMIM 229600) in favor of rheumatic pathology demonstrate the relevance of this problem.

**Objectives:** The aim of the study is to determine the diagnostic criteria of skeletal dysplasia for differential diagnosis with JIA.

**Methods:** We retrospectively analyzed clinical unit, laboratory findings, ultrasound, x-ray, MRI in our series of patients with CUPA (low laboratory activity, enthesopathy or synovitis with progressive idiopathic contracture). The study included 74 children who were treated at the National Medical children’s orthopedics and trauma surgery Research Center, Saint-Petersburg between 2005 and 2020.

**Results:** We divided children into two groups: patients with rMED (52) and patients with JIA (22). Children with other monogenic mutations similar to arthritis and other forms of dysplasia were excluded from the study.

Distinctive features in the clinical picture of the rMED were disproportionate constitution, absence of morning stiffness, symmetrical contracture formation, axial deformities of the lower extremities, and absence of physiological hypermobility at an early age with impaired motor activity. Laboratory data in children with rMED were normal or indicated moderate inflammatory response without any specific changes. Radiographic data demonstrated bilateral and symmetric flattening of epiphyse with epimetaphyseal widenings, double-layered patella, dystrophic spondylolisthesis at the L5-S1 vertebrae to 10-12 years of life. X-ray of the hip joints in young children with rMED showed symmetrical delay in ossification of the femoral heads, and some patients developed bilateral extrusion subluxation of the epiphyses and avascular necrosis. In JIA, we could see a staging of radiological phases: accelerated ossification of cartilaginous epiphyses, erosive-dystrophic changes, arthrosis-arthritis.

Ultrasound and MRI are not helpful in making the diagnosis because signs of synovitis exist in both groups. Sensitivity to anti-inflammatory therapy was also not a pathognomonic criterion due to its effect on synovitis.

**Conclusion:** Early diagnosis and non-surgical treatment rMED are important for recovery. Because in the opposite case, progressive arthritis leads to significant disability. A comprehensive approach in the evaluation of medical data and their dynamics, which takes into account the anatomical and physiological features of children, allows us to identify the true cause of arthropathy.

**Patient Consent:** Not applicable (there are no patient data)

**Disclosure of Interest**: None declared

### P061. Phenotypes of presentation of psoriatic arthritis in pediatric patient

#### P. P. Ramos, H. F. Menchaca-Aguayo, E. R. Mercedes-Pérez, N. De la Rosa-Encarnación, M. I. De la Cera-Rodríguez, A. Guzmán-Revilla, A. Barba-Aguilar, K. Primero-Nieto, H. Bermudez-Canales, S. Rodríguez-Aguayo, E. Faugier-Fuentes

##### Reumatología Pediátrica., Hospital Infantil De México Federico Gomez, Mexico, Mexico

###### **Correspondence:** P. P. Ramos

**Introduction:** Juvenile idiopathic arthritis (JIA) unifies all forms of chronic arthritis, affecting not only the joints but also other systems. Psoriatic JIA (JPsA) is an uncommon category in childhood and there are few reports describing the characteristics and long-term outcome of patients with psoriatic JIA than other subtypes of JIA.

**Objectives:** To describe the case and evolution in a patient with a diagnosis of JPsA.

**Methods:** Description of a clinical case and review of the literature.

**Results:** A 12-year-old female with 2 months of evolution with constitutional syndrome and arthralgias in the right hip and proximal interphalangeal joints associated with morning stiffness. Polyarticular JIA was diagnosed FR negative and methotrexate was started, and sulfasalazine was added due to persistent activity. He lost follow-up for four years. Subsequently, she presented with disease activity and clinical signs of sacroiliitis. MRI of the sacroiliacs was normal. She persists with arthritis of more than 10 joints, the change from methotrexate DMARD to leflunomide was performed, and the dose of sulfasalazine was increased. Approach for biologic therapy was performed. She lost follow-up for two years. She came again with disease activity, with arthritis and joint limitation, dermatosis in elbows and nail pitting; new laboratory studies, imaging and skin biopsy were performed. Due to the clinical features, a diagnosis of psoriasis was integrated and management with calcineurin inhibitor was started. Six years after the onset of symptoms, bilateral sacroiliitis was documented clinically and in sacroiliac MRI. Skin biopsy: psoriasis. Management with infliximab and double DMARDs was started. She does not present uveitis. Current treatment: methotrexate, sulfasalazine, infliximab, and topical tacrolimus.

**Conclusion:** There are few reports describing the evolution of AIJps and it can be confusing when classifying it. In the case of the patient, regardless of the irregularity of adherence to treatment, the clinical expression presented different phenotypes: polyarticular JIA, spondyloarthropathy and psoriatic JIA; being a complex diagnosis at the time of classification.

**Patient Consent:** Yes, I received consent

**Disclosure of Interest**: None declared

### P063. Efficacy of secukinumab in enthesitis-related arthritis and juvenile psoriatic arthritis: results from a phase 3 study (JUNIPERA)

#### N. Ruperto^1^, E. Chertok^2^, J. Dehoorne^3^, G. Horneff^4^, T. Kallinich^5^, I. Louw^6^, M. Alessio^7^, S. Compeyrot-Lacassagne^8^, B. Lauwerys^9^, N. Martin^10^, K. Marzan^11^, W. Knibbe^12^, R. Martin^13^, X. Zhu^14^, S. Whelan^15^, L. Pricop^14^, D. J. Lovell^16^, A. Martini^17^, H. I. Brunner^16^ on behalf of PRINTO/PRCSG

##### ^1^IRCCS Istituto Giannina Gaslini, Genova, Italy, ^2^Voronezh State Medical University, Voronezh, Russian Federation, ^3^University Hospital Gent, Gent, Belgium, ^4^Asklepios Klinik Sankt Augustin GmbH, Sankt Augustin, ^5^Charité-Universitätsmedizin Berlin, Berlin, Germany, ^6^Panaroma Medical Centre, Cape Town, South Africa, ^7^Università degli Studi della Campania Luigi Vanvitelli , Napoli, Italy, ^8^Great Ormond Street Hospital for Children, London, United Kingdom, ^9^Cliniques Universitaires Saint-Luc, Bruxelles, Belgium, ^10^Yorkhill Hospital, Glasgow, United Kingdom, ^11^Children’s Hospital Los Angeles, Los Angeles, ^12^St Lukes Intermountain Research Center , Boise, ^13^Novartis Pharmaceutical Corporation, ^14^Novartis Pharmaceuticals Corporation, East Hanover, United States, ^15^Novartis Ireland Ltd., Dublin, Ireland, ^16^University of Cincinnati, Cincinnati, United States, ^17^Università di Genova, Genova, Italy

###### **Correspondence:** N. Ruperto

**Introduction:** Juvenile idiopathic arthritis (JIA) categories of enthesitis-related arthritis (ERA) and juvenile psoriatic arthritis (JPsA) represent paediatric counterparts of adult non-radiographic axial spondyloarthritis and psoriatic arthritis, respectively.^1,2^ JUNIPERA, a 2-year, randomised, double-blind, placebo (PBO)-controlled trial demonstrated significantly longer time to flare with secukinumab (SEC) vs PBO with sustained improvements up to Week (Wk) 104.^3^

**Objectives:** To evaluate the effect of SEC on axial and peripheral manifestations in active ERA and JPsA patients (pts).

**Methods:** Pts (2–<18 years age) with active disease (both ≥3 active joints and ≥1 active enthesitis site) were treated with open-label (OL) s.c. SEC (75/150 mg in pts <50/≥50 kg) in treatment period (TP)1. SEC was administered at baseline (BL) and Wks 1–4, 8 and 12. Responders at Wk 12 (JIA-ACR30 response) were randomised into the double-blind, withdrawal TP2 to continue SEC or PBO every 4 wks until a disease flare or up to Wk 100. The time to flare in ERA and JPsA pts in TP2 is reported here. JIA-ACR responses, resolution of enthesitis and dactylitis, JADAS-27, and axial, peripheral and skin manifestations were also assessed.

**Results:** A total of 52/86 (60.5%) pts with ERA (mean age, 13.7 years) and 34/86 (39.5%) pts with JPsA (mean age, 12.2 years) were enrolled in the OL TP1. Flare risk reduction in TP2 was 55% (HR 0.45, 95% CI: 0.16–1.28, *P*=0.075) in ERA pts and 85% (HR 0.15, 95% CI: 0.04–0.57, *P*<0.001) in JPsA pts. Improvements in JIA-ACR responses, inactive disease and JADAS-27 were observed with SEC treatment at Wk 12 and at the end of TP2 (Table). Axial symptoms in ERA with SEC and PBO at the end of TP2 were: modified Schober’s test, 100% and 100%; inflammatory back pain, 100% and 50%; Flexion, ABduction and External Rotation (FABER) test, 100% and 83.3%; and clinical sacroiliitis, 100% and 50%. Pts with CHAQ score of 0 at the end of TP2 with SEC and PBO treatment were 9 each in ERA and 8 each in JPsA. Sustained improvements were also observed in enthesitis, dactylitis and other psoriatic skin manifestations (data not shown).

**Conclusion:** In pts with active ERA and JPsA, SEC demonstrated a longer time to disease flare with flare risk reduction compared to PBO up to Wk 104 and exhibited rapid and sustained improvement of axial, peripheral and skin manifestations.

**References**

1. Pagnini I, et al. *Front Med* 2021;8:6673052

2. Zisman D, et al. *J Rheumatol* 2018;94:11–16

3. Brunner H, et al. *Arthritis Rheumatol* 2021;73(10)

**Trial registration identifying number:** NCT03031782

**Patient Consent:** Not applicable (there are no patient data)

**Disclosure of Interest**: N. Ruperto Grant / Research Support with: Bristol Myers and Squibb, Eli Lilly, F Hoffmann-La Roche, Novartis, Pfizer and SOBI, Consultant with: Ablynx, Amgen, Astrazeneca-Medimmune, Aurinia, Bayer, Bristol Myers and Squibb, Cambridge Healthcare Research (CHR), Celegene, Domain therapeutic, Eli Lilly, EMD Serono, GlaxoSmith and Kline, Idorsia, Janssen, Novartis, Pfizer, SOBI and UCB, Paid Instructor with: Eli Lilly, GlaxoSmith and Kline, Pfizer, SOBI and UCB, Speaker Bureau with: Eli Lilly, GlaxoSmith and Kline, Pfizer, SOBI and UCB, E. Chertok: None declared, J. Dehoorne Grant / Research Support with: Abbvie, Roche, Consultant with: Abbvie, Roche, Pfizer, Speaker Bureau with: Abbvie, Roche, G. Horneff Grant / Research Support with: Pfizer, Novartis, Roche, MSD, Speaker Bureau with: Novartis, Pfizer, Janssen, T. Kallinich Speaker Bureau with: Roche, I. Louw Consultant with: Pfizer, Abbvie, Janssen, Amgen, Cipla, Speaker Bureau with: Pfizer, Abbvie, BMS, M. Alessio: None declared, S. Compeyrot-Lacassagne: None declared, B. Lauwerys Employee with: UCB Pharma, N. Martin: None declared, K. Marzan Grant / Research Support with: Novartis, Sanofi, W. Knibbe Speaker Bureau with: Novartis, Amgen, UCB, Abbvie, R. Martin Shareholder with: Novartis, Employee with: Novartis, X. Zhu Shareholder with: Novartis, Employee with: Novartis, S. Whelan Shareholder with: Novartis, Employee with: Novartis, L. Pricop Shareholder with: Novartis, Employee with: Novartis, D. J. Lovell Grant / Research Support with: Astra Zeneca, Boehringer Ingelheim, GSK, Hoffman LaRoche, Novartis, UBC, Consultant with: Astra Zeneca, Boehringer Ingelheim, GSK, Hoffman LaRoche, Novartis, UBC, A. Martini Consultant with: Eli-Lilly, EMD Serono, Janssen, Novartis, Pfizer, AbbVie, Idorsia, H. I. Brunner Grant / Research Support with: Novartis, Consultant with: Novartis

**Table 1 (abstract P063).** Efficacy of SEC in ERA and JPsA pts

Variable	ERA			JPsA		
	TP1-Wk 12	End of TP2*		TP1-Wk 12	End of TP2*	
	SEC (N=52)	SEC (N=22)	PBO (N=22)	SEC (N=34)	SEC (N=15)	PBO (N=16)
**JIA ACR30, %**	84.6	90.9	68.2	91.2	86.7	62.5
**JIA ACR50, %**	78.8	81.8	68.2	91.2	73.3	56.3
**JIA ACR70, %**	65.4	68.2	54.5	70.6	66.7	31.3
**JIA ACR90, %**	32.7	45.5	50.0	47.1	60.0	25.0
**JIA ACR100, %**	26.9	36.4	45.5	20.6	53.3	25.0
**Inactive disease, %**	38.5	50.0	50.0	29.4	46.7	18.8
**JADAS-27, mean (SD) change from BL**	–9.6 (7.5)	–11.0 (8.9)	–7.6 (8.9)	–11.9 (6.7)	–11.5 (9.0)	–7.0 (7.2)

*End of TP2 is based on individual pts’ last visit at TP2

N, total number of patients

### P064. Exploratory methods identify novel autoantigens in juvenile idiopathic arthritis

#### S. Arve-Butler^1,2,3^, E. Rydén^1,2^, A. Mossberg^1,2^, R. Kahn^1,2^

##### ^1^Wallenberg Center for Molecular Medicine, Lund University, ^2^Department of Pediatrics, ^3^Department of Rheumatology, Clinical Sciences, Lund University, Lund, Sweden

###### **Correspondence:** E. Rydén

**Introduction:** Specific autoantibodies in Juvenile idiopathic arthritis (JIA) remain mostly unidentified, although they may serve as potential biomarkers for disease outcome and uveitis.

**Objectives:** To investigate different strategies for autoantibody discovery of JIA-specific autoantigens.

**Methods:** Serum (n =57) and plasma (n=6) were collected from JIA patients with oligoarticular or seronegative polyarticular JIA at the Department of Pediatrics at Lund University Hospital with informed consent and assent. Anonymized serum samples from pediatric controls (n=22) were available. Ethical permission was granted by the Regional Ethical Review board for southern Sweden (LU 2016/128). Plasma from six patients with ANA positive persistent oligoarticular JIA positive were included in two exploratory analyses to identify novel autoantigens. Two pools with three patients in each (with or without uveitis) was used. First, an assay covering 42 100 unique recombinant peptides of 50-150 amino acid each, from 18 000 different proteins was used. This explored approximately 94% of the human proteome by printing the peptides on glass slides which then were incubated with the two plasma pools. Second, immunoprecipitation (IP) by plasma incubated with mixed with cell lysates from the human epithelial cell line followed by liquid chromatography mass spectrometry at BioMS (Lund University) of captured proteins was used. Peptides or proteins identified in either of the two assays in combination with autoantigens found in literature made up a selection of peptides being further investigated in a targeted array (SciLifeLab, Stockholm). In this array, patient serum (n=57) was tested individually, as well as serum from controls (n=22). The array used color coded magnetic beads, individually coated with one of the peptides and was analyzed by flow cytometry (Luminex). The data was given as raw MFI and autoantigens were defined as reactive in <10% of controls and >10% of the patients after adjustment to background beads.

**Results:** In the autoimmune profiling planar array, reactivity was detected to 332 peptides in at least one of the two plasma pools. Thirty-four peptides showed reactivity in both pools, 75 in the non-uveitis pool and 223 in the uveitis pool. Immunprecipitation followed by mass spectrometry showed reactivity for 131 full length proteins. Ninety-five of the precipitated proteins were overlapping in the two patient pools, 14 showed reactivity only in the non-uveitis pool and 22 in the uveitis pool. Unexpectedly, only two proteins showed reactivity in both the planar array and the IP. For targeted bead array, we selected 335 peptides for validation with an additional method and patient cohort. 174 from the planar array, 97 from IP and 102 from the literature. There was an overlap between peptides found in literature and in the IP or planar array. Of the 335 peptides, 4 were excluded due to weak coupling to the beads and 73 peptides due to high reactivity to control serum. Twenty antigens showed high reactivity in JIA patients. Fifteen of these were chosen from the planar array, three found in the literature as well as by IP and one each found exclusively in IP or in literature.

**Conclusion:** Autoantibody discovery is highly dependent on the choice of method, as there was almost no overlap of autoantigens found by planar array and immunoprecipitation followed by mass spectrometry. The planar array identified 15 novel potential autoantigens and the IP identified one that was not previously mentioned in literature. Our findings show that established methods can be used to discover novel autoantigens in JIA.

**Patient Consent:** Yes, I received consent

**Disclosure of Interest**: None declared

### P065. Polyarticular JIA has a distinct co-inhibitor receptor profile among other JIA subtypes

#### E. Sag^1,2^, Z. Balik^3^, S. Sener^3^, U. Akca Kaya^3^, S. Demir^3^, M. Kasap Cuceoglu^3^, E. Atalay^3^, S. Bocutcu^3^, T. Vural^1^, Y. Bilginer^3^, B. Deleuran^4^, S. Ozen^1,3^

##### ^1^Pediatric Rheumatology Unit, Translational Medicine Laboratories, Hacettepe University, ^2^Pediatric Rheumatology Clinic, Ankara Training and Research Hospital, ^3^Pediatric Rheumatology, Hacettepe University, Ankara, Turkey, ^4^Institution of Biomedicine, Aarhus University, Aarhus, Denmark

###### **Correspondence:** E. Sag

**Introduction:** Juvenile idiopathic arthritis (JIA) is the most common inflammatory joint disease in children, driven by continuous T-cell activation. T cell activation is counter-balanced by signals generated by co-inhibitory receptors (co-IRs) such CTLA-4, PD-1, LAG-3, and TIM-3.

**Objectives:** We aimed to identify the role of co-IRs in the pathogenesis of different subtypes of JIA.

**Methods:** In total, we included patients with oligoarticular JIA (n=67), polyarticular JIA (n=12), enthesitis related arthritis (n=17), systemic JIA (n=11) and healthy controls (HC, n=10). We collected plasma samples from the patients during the active phase of their disease. We measured the soluble plasma levels of co-IRs by commercial pre-defined cytometric bead array kits and their cellular expression by flow cytometry in blood mononuclear cells. We compared the plasma levels and cellular expressions of different coIRs within different JIA subgroups.

**Results:** IL-2 levels were lower than HC in all JIA subgroups. The polyarticular JIA group distinguished from the four different JIA subgroups, by having different co-IR pattern. In this specific subgroup, CTLA4, PD-1 and 4-1BB levels were higher than other groups. Polyarticular JIA is the more chronic and severe form of JIA, especially when compared to oligoarticular JIA.

We investigated the correlations between disease activity markers and plasma co-IRs. Plasma TIM3 levels correlated with erythrocyte sedimentation rate, C-reactive proteins and JADAS in the polyarticular JIA group. In oligoarticular JIA group, JADASs correlated with plasma PD-1 levels, C-reactive protein with PD-L1 plasma levels. Erythrocyte sedimentation rates correlated with IL-2, CD86, PD-L1 and PD-1 plasma levels. There was no correlation between disease activity markers and co-IRs levels in the systemic JIA group and enthesitis related arthritis group.

Finally, we analysed the cellular surface expression of different co-IRs on the PBMCs of different JIA subtypes. Similar to plasma levels, both the percentage and the MFI (mean fluorescence intensity) of CTLA4 expression was higher in polyJIA subgroup.

**Conclusion:** This is the first report studying the effects of different co-IRs in in different subtypes of JIA. Polyarticular JIA patients had a different co-IR profile, having more CTLA-4, PD-1 and 4-1BB in their plasma than the other subtypes of JIA, which may due to both increased degree of cellular activation and exhaustion of cells in this more resistent form of JIA.

**Acknowledgement**

This work was supported by a research grant from FOREUM Foundation for Research in Rheumatology

**Patient Consent:** Not applicable (there are no patient data)

**Disclosure of Interest**: None declared

### P066. Evaluation of comorbidities in patients with juvenile idiopathic arthritis

#### N. Sahin, S. Asadova^2^, S. N. Taşkın^3^, S. Doğantan^3^, S. Özdemir Çiçek^4^, E. Esen^3^, A. Paç Kısaarslan^3^, M. H. Poyrazoğlu^3^

##### ^1^Pediatric Rheumatology, Derince Training and Research Hospital, Kocaeli, ^2^Pediatrics, ^3^Pediatric Rheumatology, Erciyes University, Faculty of Medicine, ^4^Pediatric Rheumatology, Kayseri City Hospital, Kayseri, Turkey

###### **Correspondence:** N. Sahin

**Introduction:** Juvenile idiopathic arthritis (JIA) is a common chronic rheumatologic disease in children. In chronic diseases, comorbidities can adversely affect the course of the disease.

**Objectives:** In this study, we aimed that investigated comorbidities in patients with juvenile idiopathic arthritis and their effects on the disease course.

**Methods:** We retrospectively analyzed 204 patients under 18 years and with juvenile idiopathic arthritis. Patients were stratified into two groups with comorbidities and without comorbidities. Clinical findings, JIA subtypes, JADAS27, JSPADAS, JADI-A and E, and CHAQ scores were investigated in two groups.

**Results:** The number of patients with comorbidity was 99 (48.5%). The most common comorbidity was familial Mediterranean fever (FMF) in 31 (31.3%) and uveitis in 23 (23.2%). The clinical and laboratory characteristics of the patients are summarized, and the comorbidities in table 1. There was no difference between patients with and without comorbidity in terms of age, age at diagnosis, duration of JIA, and sex. The most common JIA subtype in both groups was oligoarticular JIA, followed by enthesitis-related arthritis and polyarticular JIA. There was no significant difference in the number of comorbidities by JIA subtype (p=0.79). In addition, no correlation was found between the duration of JIA disease and the number of comorbidities (Rho=-0.69, p=0.49). According to the presence of comorbidity, there was no difference in the scores of JADI-A, JADI-E, CHAQ disability, CHAQ discomfort, and CHAQ pain (respectively, p= 0.52, p=0.33, p=0.77, p=0.44, p= 0.19). There was no significant difference in JSPADAS or JADAS 27 scores between the two groups (p=0.63, p=0.55, respectively).

**Conclusion:** There was comorbidity in approximately half of the patients with juvenile idiopathic arthritis. Although the frequency was low, comorbidities involving all systems were observed. However, the presence of comorbidity did not affect the course of JIA.

**Patient Consent:** Yes, I received consent

**Disclosure of Interest**: None declared

**Table 1 (abstract P066).** See text for description

Clinical findings	With comorbidity n=99 (100%)	Without comorbidity n=105 (100%)	p
Female	64 (64.6%)	65 (61.9%)	0.69
Age (year)	13 (5-17)	13 (4-17.5)	0.57
Age of diagnosis (year)	7 (1-15)	8 (1-15)	0.18
Duration of JIA (year)	6 (2-14)	5 (2-16)	0.11
JIA subtypes			0.43
Oligoarticular	44 (45.3%)	38 (36.2%)	
ERA	28 (28.3%)	27 (25.7%)	
Polyarticular	16 (16.2%)	26 (24.8%)	
Juvenile psoriatic arthritis	1 (1%)	4 (3.8%)	
Systemic	8 (8.1%)	9 (8.6%)	
Undifferatial	2 (2%)	1 (1%)	
The number of comorbidities	1 (1-3)		
The presence of additional autoimmune disease	34 (34.3%)		
The presence of disease or treatment-related complications	39 (39.4%)	30 (28.6%)	0.10
Amyloidosis	2 (2%)	1 (1%)	0.61
MAS	2 (2%)	5 (4.8%)	0.34
ANA (+)	42 (42.4%)	48 (45.7%)	0.51
HLA B27 (+)	14 (14.1%)	18 (17.1%)	0.17
RF (+)	2 (2%)	2 (1.9%)	1.00
JADI-A	0 (0-22)	0 (0-24)	0.52
JADI-E	0 (0-3)	0 (0-4)	0.33
CHAQ			
Disability	0 (0-1.25)	0 (0-1.3)	0.77
Discomfort	0 (0-2.4)	0 (0-2.7)	0.44
Pain	0 (0-1.5)	0 (0-2.7)	0.19
JADAS 27	0 (0-29)	0 (0-21)	0.55
JSPADAS	0 (0-8.5)	0 (0-9)	0.63
Comorbidities			
Rheumatological			
FMF	31 (31.3%)		
Uveitis	23 (23.2%)		
Recurrent aphthous stomatitis	4 (4%)		
Primary raynaud’s syndrome	1 (1%)		
Neurological			
Epilepsy	5 (5%)		
Infarct	2 (2%)		
Migraine	2 (2%)		
Syringomyelia	1 (1%)		
Spina bifida	1 (1%)		
Facial paralysis	1 (1%)		
Neurocutaneous disease	1 (1%)		
Renal			
Proteinuria	3 (3%)		
Essential hypertension	2 (2%)		
Enuresis nocturna	2 (2%)		
Vesicoureteral reflux disease	2 (2%)		
Nephrolithiasis	1 (1%)		
Simple kidney cyst	1 (1%)		
Endocrinological			
Congenital hypothyroidism	2 (2%)		
Autoimmune thyroiditis	2 (2%)		
Precocious puberty	2 (2%)		
Congenital adrenal hyperplasia	1 (1%)		
Gastrointestinal			
Inflammatory bowel disease	4 (4%)		
Esophagitis	2 (2%)		
Allergic	6 (6%)		
Immunological	5 (5%)		
Psychiatric	5 (5%)		

### P067. Conventional dendritic cells type 1 (CDC1) are strongly enriched, quiescent and relatively tolerogenic in local inflammatory arthritis

#### A. Boltjes^1^, A. Samat^1^, M. Plantinga^1^, M. Mokry^1^, B. Castelijns^2^, J. F. Swart^3^, B. J. Vastert^1,3^, M. Creyghton^2,4^, S. Nierkens^1,5^, J. V. Loosdregt^1,3^, F. V. Wijk^1,3^

##### ^1^Center of Translational Immunology, University Medical Center Utrecht, ^2^Hubrecht Institute, ^3^Pediatric Rheumatology & Immunology, Wilhelmina Children’s Hospital, Utrecht, ^4^Erasmus University Medical Center, Rotterdam, ^5^Princess Maxima Center for Paediatric Oncology, Utrecht , Netherlands

###### **Correspondence:** A. Samat

**Introduction:** Dendritic cells (DC) are crucial for initiating and shaping immune responses. So far, little is known about the functional specialization of human DC subsets in (local) inflammatory conditions.

**Objectives:** We profiled conventional (c)DC1, cDC2 and monocytes based on phenotype, transcriptome and function from the synovial fluid (SF) from Juvenile Idiopathic Arthritis patients (JIA).

**Methods:** Paired peripheral blood (PB) and synovial fluid (SF) samples from 32 JIA and 4 RA patients were collected for mononuclear cell isolation. Flow cytometry was done for definition of antigen presenting cell (APC) subsets. Cell sorting was done on the FACSAria II or III. RNA sequencing was done on SF APC subsets. Proliferation assays were done on co-cultures after CD3 magnetic activated cell sorting (MACS). APC Toll-like receptor (TLR) stimulation was done using Pam3CSK4, Polyinosinic:polycytidylic acid (Poly(I:C)), Lipopolysaccharide (LPS), CpG-A and R848. Cytokine production was measured by Luminex.

**Results:** cDC1, a relatively small DC subset in blood, were found to be strongly enriched in SF, and showed a quiescent immune signature without a clear inflammatory profile, low expression of pattern recognition receptors (PRR), chemokine and cytokine receptors, and poor induction of T cell proliferation and cytokine production. In stark contrast, cDC2 and monocytes from the same environment, showed a pro-inflammatory transcriptional profile, high levels of (spontaneous) pro-inflammatory cytokine production, and strong induction of T cell proliferation and cytokine production, including interleukin-17 (IL-17).

**Conclusion:** At the site of inflammation, there is specific functional programming of human DCs, especially cDC2. Monocytes in particular seem to be the most pro-inflammatory, producing high levels of IL-6 and tumor necrosis factor alpha (TNF-a) and cDC2 also show a strong pro-inflammatory profile with high T cell activation capacity. In contrast, the enriched cDC1 remain relatively quiescent and seemingly unchanged under inflammatory conditions, pointing to a potentially more regulatory role.

**Patient Consent:** Yes, I received consent

**Disclosure of Interest**: A. Boltjes: None declared, A. Samat: None declared, M. Plantinga: None declared, M. Mokry: None declared, B. Castelijns: None declared, J. Swart Consultant with: Personal fees from Amgen outside of the relevant work, B. Vastert Consultant with: Personal fees from SOBI & Novartis outside of the relevant work , M. Creyghton: None declared, S. Nierkens: None declared, J. Loosdregt: None declared, F. Wijk Grant / Research Support with: ReumaFonds Nederland; grants from Sanofi, Regeneron and Leo Pharma outside of the relevant work, Consultant with: Johnson&Johnson and Takeda outside of relevant work

### P068. Effect of drug therapy for juvenile idiopathic arthritis on the level of cystatin c as a marker of renal dysfunction

#### S. Samsonenko, T. Borysova

##### Pediatric, “Dnipro State Medical University”, Dnipro, Ukraine

###### **Correspondence:** S. Samsonenko

**Introduction:** Juvenile idiopathic arthritis (JIA) is a chronic disease requiring years of therapy with non-steroidal anti-inflammatory drugs (NSAIDs), immunosuppressant’s, cytostatics, immunobiological agents. The aforementioned drugs, namely NSAIDs and cytostatics are potentially nephrotoxic. The above drugs, namely NSAIDs and cytostatics, are potentially nephrotoxic. About 8% of children with JIA have kidney damage, which develops on average 5 years after the onset of the disease. It has been established that the main risk factor for the development of kidney damage is the long-term exposure to NSAIDs and methotrexate in children with active forms of JIA. Early diagnosis of kidney damage will allow timely correction in the dosage of drugs and avoid their nephrotoxic effects.

**Objectives:** To determine the effect of drug therapy in children with JIA on eGFR by using the Cystatin C-based equation and the Hoek formula based on the serum cystatin C study.

**Methods:** 80 children with JIA participated in the study. The age of subjects was 10.4±4.41 (10.6-15.0) years. All children received methotrexate as a base drug. At the moment of examination 22 children received NSAIDs, 25 children received immunobiological preparations. Serum cystatin C content was determined by enzyme immunoassay. The Cystatin C-based equation 2012 and Hoek formulas were used to set the GFR by serum cystatin C levels.

**Results:** Non-steroidal anti-inflammatory drugs led to a decrease in GFR as found by both the Cystatin C-based equation 2012 and the Hoek formula. The incidence of GFR reduction in patients treated with NSAIDs using the Cystatin C-based equation 2012 was 100%, and using the Hoek formula was 81.8%.The use of NSAIDs in children with JIA is a risk factor for the development of reduced GFR calculated by the Hoek formula. The incidence of reduced GFR in children with NSAID use was 54.5%, 6.7 times greater than in those without NSAIDs (OR = 12.9; CI: 3.76-44.25; p<0.001). There was a low chance of a Hoek formula decrease in GFR in children with JIA who received immunobiological therapy 9.1% vs 46.8% (OR = 0.11; CI: 0.03-0.42; p<0.001).

**Conclusion:** Use of NSAIDs in children with JIA was more often associated with a reduction in GFR: by Cystatin C - based equation 2012 in 100% of cases p<0.01, by Hoek in 81.8%, p<0.001. The average of GFR was significantly lower in children treated with NSAIDs than in children without NSAIDs. Immunobiological therapy had a positive effect on the GFR value. The frequency of a decrease in GFR was significantly lower in the children treated with immunobiological therapy compared with those without immunobiological therapy 9.1% vs 46.8% (OR = 0.11; CI: 0.03-0.42; p<0.001).

**Patient Consent:** Yes, I received consent

**Disclosure of Interest**: None declared

### P069. A key role of IL-6/STAT signaling and cell-cell interactions in monocyte function in oligoarticular juvenile idiopathic arthritis

#### T. Schmidt^1,2^, E. Rydén^1,2^, A. Dahlberg^1,2^, S. Arve-Butler^1,2,3^, A. Mossberg^1,2^, R. Kahn^1,2^

##### ^1^Wallenberg Center for Molecular Medicine, Lund University, ^2^Department of Pediatrics, ^3^Department of Rheumatology, Clinical Sciences, Lund University, Lund, Sweden

###### **Correspondence:** T. Schmidt

**Introduction:** Synovial monocytes in oligoarticular juvenile idiopathic arthritis (oJIA) are polarized, displaying markers of both pro- and anti-inflammation.

**Objectives:** To study the function of synovial monocytes, and to unravel how they obtain their phenotype.

**Methods:** Synovial fluid (SF) and blood (which served as control) were collected from untreated oJIA patients upon therapeutic joint aspiration (total n=26). Surface markers (CD16, MerTK, HLA and CD86), cytokine production (IL-1ß, IL-6, IL-8 and TNF) and STAT phosphorylation (STAT1, STAT3 and STAT6) were analyzed in synovial- and circulating monocytes using flow cytometry. Isolated monocytes were used for efferocytosis assays using apoptotic neutrophils and co-stimulation of CD3 activated T cells from healthy donors. The influence of SF on healthy monocytes was analyzed by liquid-chromatography mass spectrometry and broad-spectrum phosphorylation assays. The mechanisms of how synovial monocytes obtain their functional phenotype was studied *in vitro* through stimulation of healthy monocytes using SF, migration using a transwell system or co-culture with fibroblast-like synoviocytes.

**Results:** Monocytes from the joint of oJIA patients display functional alterations with pro- and anti-inflammatory features. Synovial monocytes, as compared to circulating monocytes, express markers of antigen presentation (HLA, CD86), induce proliferation and activation markers (CD25, HLA and CTLA-4) in healthy T cells and are primed for STAT1 phosphorylation. In contrast, synovial monocytes also express markers of clearance (MerTK, CD16), display less production of the pro-inflammatory cytokines (IL-1ß, IL-6, IL-8 and TNF) upon activation, and have increased efferocytosis. In healthy monocytes, SF from oJIA patients, as compared to serum, induces upregulation of biological processes involved in immune responses and regulation of lymphocyte proliferation, cell-cell adhesion, and endocytosis. At the phosphorylation level, synovial fluid induces mainly STAT3, which correlates with IL-6 levels in the synovial fluid (r=0.78, p<0.0011) and is fully blocked by pre-incubation with the anti-IL-6R antibody tocilizumab (p<0.0001) or the JAK inhibitor tofacitinib (p<0.0001). At the functional level, synovial fluid, mainly through an IL-6/STAT mechanism, induces the anti-inflammatory aspects observed in the patients’ synovial monocytes, such as surface markers (CD16, MerTK (p<0.0001), increased efferocytosis (p<0.0001) and resistance to production of IL-1ß, IL-6, IL-8 and TNF (p=0.0002) upon activation. The pro-inflammatory aspects are driven through cell-cell interactions *in vitro*, either through migration or co-culture, which result in increased expression of CD86, HLA and T cell activation (p<0.0001).

**Conclusion:** Synovial monocytes from patients with oJIA display both pro- and anti-inflammatory functional alterations. This phenotype can be replicated *in vitro*, where SF induces the anti-inflammatory aspects mainly through IL-6/STAT signaling, whilst the pro-inflammatory aspects is replicated by cell-cell interactions through migration or co-culture with fibroblast-like synoviocytes. These data support a role of monocytes in the pathogenesis of oJIA and highlight potential impact of current and future drugs for the treatment of oJIA.

**Patient Consent:** Yes, I received consent

**Disclosure of Interest**: None declared

### P070. Rare association of juvenile polyarthritis and steroid-resistant nephrotic syndrom – case report

#### V. Selmanovic^1^, A. Cengic^2^, A. Mustajbegovic^3^, D. Pokrajac^4^, T. Avcin^5^, M. Debeljak^6^, I. Sefic-Pasic^7^, M. Bukvic^7^, A. Dzananovic^7^

##### ^1^Department for Allergology, Rheumatology and Clinical Immunology, Children’s Hospital University Clinical Center Sarajevo, ^2^Department for Allergology, Rheumatology and Clinical Immunology, Children’s Hospital University Clinical Center Sarajevo, ^3^Department for Nephrology, ^4^Department fo Nephrology, , Children’s Hospital University Clinical Center Sarajevo, Sarajevo, Bosnia and Herzegovina, ^5^Departmentfor Allergology, rhjeumatology and Clinical Immunology, Childen’s Hospital University Clinical Center Ljubljana, ^6^Department for Allergology, rheumatology and Clinical Immunology, Children’s Hospital University Clinical Center Ljubljana, Ljubljana, Slovenia, ^7^Institut for Radiology, institut for Radiology, Sarajevo, Bosnia and Herzegovina

###### **Correspondence:** V. Selmanovic

**Introduction:** Renal disease is rare in children with juvenile idiopathic arthritis. The majority of children with nephrotic syndrome have minimal change disease, generally responsive to steroids. Only 10-20% are steroid-resistant.

**Objectives:** to describe a case of rare association of early-onset polyarthritis, steroid-resistant nephrotic syndrom and MEFBgene mutation

**Methods:** case report

**Results:** boy,4,5y, first child of healthy non-consanguineous Caucasian parents, unremarkable family history. Disease onset: age 18mo with nephrotic sy. Treated accordingly with steroids; experienced 3 relapses by age 3y. At age 2y2mo, while od steroids, developed symmetrical arthritis of hips, knees, ankles. ANA, RF, HLAB27 were negative. There were positive Scl-70 antibody, repeatedly negative. Treatment was quickly escalated and cyclosporin A was succesfully added. Kidney biopsy showed minimal change disease, without signs of amiloidosis. No signs of other organ involvement. On follow up, at age 4,5y, child has preserved renal function, normotensive, mildly active left knee arthritis. Bilateral pes equinovarus was surgically corrected in infancy. Gene panels for nephrotic syndrome and arthritis revealed MAFBgene mutation NM_005461.5:c[125C>A];[=] in patient and mother, associated with hereditary osteolysis of carpal bones with and without nephropathy. Both are not fullfilling diagnostic criteria, so there is highly unlikely that the variant is disease causing.

**Conclusion:** Pediatric rheumatologist treat early onset polyarthritis on everyday basis as well as nephrologist children with nephrotic syndrom of which 10-20% are steroid-resistant. Concomitant association of these two entities is rarely described, raising question whether are they part od the same still undefined disease and question of disease evolution. In this case, genetics were not diagnostic.

**Disclosure of Interest**: None declared

### P071. Analysis the survival of genetically engineered biological therapy in children with juvenile idiopathic arthritis

#### V. Sevostyanov^1^, P. Lototskaya^2^, N. Babich^1^, D. Rassoha^1^, E. Zholobova^3^

##### ^1^Department of Propaedeutics of Children’s Diseases, ^2^Department of Rheumatology No. 1 of the University Children’s Clinical Hospital, ^3^Department of pediatrics, I.M. Sechenov First Moscow State Medical University Ministry of Health of Russia (Sechenov University), Moscow, Russian Federation

###### **Correspondence:** V. Sevostyanov

**Introduction:** At present, the issue of studying effectiveness and safety of various genetically engineered biological drugs is of great interest. A clear analysis of the reasons for drug withdrawal and an algorithm for biology switching will allow a more balanced attitude towards the initiation of this type of therapy and development of individual plans for monitoring, including management of patients receiving genetically engineered therapy.

**Objectives:** To analyze the survival of genetically engineered biological therapy in children with juvenile idiopathic arthritis.

**Methods:** An observational analytical cross-sectional study was conducted and included patients with juvenile idiopathic arthritis aged 0 to 17 years old, living in Moscow, who had history of switching and/or discontinuation of a biology in their anamnesis. This study assessed the following indicators: the period of biology therapy in months; the reason for changing or canceling therapy; the drug to which the switch was made.

**Results:** The switching frequency was 9.9%. The main reasons for switching/cancellation were secondary failure - 57.8%, primary failure - 12.5%, therapy intolerance - 10.9%, and the development of “de novo” uveitis in 9.4% of cases. Cancellation of therapy due to the achievement of remission was noted in 6.2% of cases. There is a trend that more often patients were switched to adalimumab (29.3%); and when adalimumab was canceled, switching was carried out to tocilizumab and golimumab. When tocilizumab has been discontinued, the majority of patients were switched to canakinumab therapy.

**Conclusion:** This analysis, being one of the first presenting the data from Russian Federation on the topic, shows that further accumulation and analysis of data, including cases of discontinuation of therapy, switching between drugs, as well as the duration of therapy before the event that caused the change or discontinuation of therapy are required.

**Patient Consent:** Yes, I received consent

**Disclosure of Interest**: None declared

### P072. Clusters of JIA at methotrexate initiation identified using topological data analysis

#### S. J. W. Shoop-Worrall^1,2^, K. L. Hyrich^1,3^, L. R. Wedderburn^4,5,6^, N. Geifman^7^ on behalf of CLUSTER, BSPAR-ETN Study, BCRD Study, CAPS, CHARMS

##### ^1^Centre for Epidemiology Versus Arthritis, ^2^Centre for Health Informatics, The University of Manchester, ^3^NIHR Manchester BRC, Manchester University NHS Foundation Trust, Manchester Academic Health Science Centre, Manchester, ^4^Centre for Adolescent Rheumatology Versus Arthritis, GOS Institute of Child Health, University College London, ^5^Paediatric Rheumatology, Great Ormond Street Hospital NHS Foundation Trust, ^6^NIHR Great Ormond Street Hospital Biomedical Research Centre, NIHR Great Ormond Street Hospital Biomedical Research Centre, London, ^7^School of Health Sciences, Faculty of Health and Medical Sciences, The University of Surrey, Guildford, United Kingdom

###### **Correspondence:** S. J. W. Shoop-Worrall

**Introduction:** Stratified medicine requires the identification of unique strata of a disease within which to base prognostic and treatment decisions. Juvenile idiopathic arthritis (JIA) offers a unique challenge in its inherent heterogeneity. The current ILAR classification, whilst useful for clinical categorisation, does not correlate with treatment outcomes. Therefore, further refinement, clustering and correlation of patient characteristics with treatment response are urgently required.

**Objectives:** To identify novel, phenotypically consistent subgroups of children and young people (CYP) with JIA at the point of starting methotrexate, across 19 patient and disease characteristics.

**Methods:** MTX-naïve CYP with JIA were selected if enrolled prior to April 2021 in one of four national JIA studies contributing to the UK CLUSTER consortium. Data from 19 harmonised study variables were extracted at point of starting MTX. Topological data analysis using a Gower similarity metric was used to identify clusters with distinct characteristics. Intervals and percent overlap between clusters were varied until an optimal model identified stable, potentially clinically plausible clusters. Significant differences in characteristics between identified clusters were tested using Kruskall-Wallis and Chi-Squared statistics.

**Results:** Of 2915 CYP included, the majority were female (68%), of white ethnicity (90%); with the most common ILAR categories being oligoarthritis (35%) and RF-negative JIA (34%).

The optimal TDA model identified six clusters which significantly differed across 16 of the 19 clinical variables at MTX initiation: Adolescents with low-moderate disease (Cluster 1, 41%), adolescents with predominantly sJIA and moderate-high disease (Cluster 2, 4%), children with predominantly sJIA and high disease (Cluster 3, <1%), children with oligo/RF-polyarthritis and low-moderate disease (Cluster 4, 43%) and two ANA-positive groups of largely females with moderate (Cluster 5, 11%) and high (Cluster 6, 1%) disease. Clustered groups also significantly differed in gender proportions (p<0.001), ethnicities (p<0.001), history of uveitis (p<0.001) and disease duration to both diagnosis (p<0.001) and MTX initiation (p<0.001), but did not differ in limited joint count (p=0.117), height (p=0.245) or BMI (p=0.394) z-scores.

**Conclusion:** This study shows substantial heterogeneity in JIA at the point of MTX initiation, with six clusters identified across 19 demographic and clinical variables. ILAR categories across clusters were not always indicators of disease activity or symptom burden. Future analyses will correlate MTX treatment response within each cluster to understand what role these combined factors may have on initial treatment response.

**Patient Consent:** Yes, I received consent

**Disclosure of Interest**: None declared

### P073. What are the outcomes of persistent oligoarthritis? A systematic review plan

#### A. Smith^1,2^, C. Swindells-Macleod ^1,2^, L. Kearsley-Fleet ^1^, K. Hyrich ^1,2^, S. Shoop-Worrall ^1,3^

##### ^1^Centre for Epidemiology Versus Arthritis, University of Manchester, ^2^NIHR Manchester Biomedical Research Centre, Manchester University NHS Foundation Trust, ^3^Centre for Health Informatics, University of Manchester, Manchester, United Kingdom

###### **Correspondence:** L. Kearsley-Fleet

**Introduction:** Oligoarthritis, a sub-type of JIA, can be categorised as persistent or extended. Children with persistent oligoarthritis, involving <5 joints, are often excluded from clinical trials as it is considered a milder form of JIA and managed less aggressively. Therefore, less is known about the longer term outcomes of those with persistent oligoarthritis. This knowledge could lead to better targeting of existing therapies for individuals and higher quality patient information.

**Objectives:** To conduct a systematic literature review on the clinical and patient-reported outcomes of persistent oligoarthritis. This abstract presents the plans and current status of the review.

**Methods:** Inclusion criteria for this systematic review are 1) inception cohorts with at least partial prospective data collection, 2) available in English language, 3) includes at least 50 patients with persistent oligoarthritis using ILAR criteria, 4) investigates clinical or patient-reported outcome. Exclusion criteria are 1) not specifically presenting or analysing outcomes in persistent oligoarthritis and 2) not reporting disease duration or follow-up time points from diagnosis at the point of outcome measurements.

Search terms related to ‘oligoarthritis’, (variants of juvenile oligoarthritis plus outcomes of interest) were used in MEDLINE and Embase from 01/01/1997 to 30/05/2022 to cover the advent of current ILAR categories to present. These terms were also entered into PubMed from 30/05/2020 to 30/05/2022 to include publications published online ahead of print.

Quality assessment, including common biases, of selected articles will be assessed using relevant questions from the Pasma et al. quality assessment (QA) tool.

Primary outcomes to be extracted are the core JIA outcome variables (active joint counts, limited joint count, physician’s global assessment, parent global evaluation, erythrocyte sedimentation rate and additional functional status (CHAQ/HAQ). Additional outcomes are 1) remission status, 2) uveitis status, 3) pain, 4) Health-related Quality of Life and 5) treatment response.

One reviewer will initially screen titles and abstracts. Two reviewers will then screen the full text of selected articles independently and agree the master list. The first reviewer will then extract data regarding study populations and outcome and another reviewer will check a selection of the extracted data. If there is disagreement or uncertainty between the two reviewers at any stage, a third reviewer will adjudicate. The review has been registered with Prospero (ID: CRD42022291943).

**Results:** To date 1051 publications have been highlighted for initial review (title and abstract) by the first reviewer. The second review is now underway and preliminary results will be ready to present.

**Conclusion:** There is an unmet need for improved understanding of outcome in persistent oligoarthritis. This systematic review will help inform treatment targets and future research in this common ILAR JIA category.

**Patient Consent:** Not applicable (there are no patient data)

**Disclosure of Interest**: None declared

### P074. Circulating mirnas as non-invasive biomarkers for juvenile idiopathic arthritis disease activity monitoring

#### A. Snipaitiene^1^, K. Snipaitiene^2^, I. Juskeviciute^2^, B. Skerbaite^2^, A. Baranauskaite^3^, S. Jarmalaite^2^

##### ^1^Academy of Medicine, Pediatric Department, Lithuanian University of Health Scienses, Kaunas, ^2^Human Genome Research Group, Life Sciences Center, Vilnius University, Vilnius, ^3^Academy of Medicine, Rheumatology Department, Lithuanian University of Health Scienses, Kaunas, Lithuania

###### **Correspondence:** A. Snipaitiene

**Introduction:** New biomarkers for defining the disease activity in the juvenile idiopathic arthritis (JIA) patients are needed to predict disease course and tailor the individual treatment strategy. Several previous studies have shown that epigenetic regulation of inflammation through microRNAs (miRNAs) has significant impact on cytokine production and disease course in adult rheumatoid arthritis.

**Objectives:** The aim of this study was to analyze if urine- and serum-derived inflammatory miR-16, -146a, and -155 could represent the disease activity in JIA patients.

**Methods:** Twenty-three children diagnosed with JIA were included into the study (oligoarthritis and polyarthritis, excluding systemic JIA). For comparison, 5 healthy controls (HC) without any signs of inflammation were enrolled. Serum and urine samples were prospectively collected from Methotrexate- or anti-TNF-treated JIA patients and HC. JIA disease activity was evaluated using JADAS27, and JIA patients were divided into remission (R1, N=13) and active disease group (R0, N=10). The urinary and serum levels of selected miRNAs were evaluated by using quantitative reverse transcription PCR (RT-qPCR) method.

**Results:** Eighty-seven % (20/23) of included JIA patients were female, and the mean age of JIA patients was 12.8 yrs (range 3-17 yrs). Significantly lower miR-16 serum levels were detected in JIA patients compared to HC (p=0.021). miR-16 levels were also significantly decreased in separate R0 and R1 groups vs. HC (p=0.023 and p=0.039, respectively). None of analyzed miRNAs were able to dichotomize JIA from HC in urine, however, significantly higher miR-16 levels were detected in R1 and R0 group patients *vs.* HC (p=0.013 and p=0.028, respectively). Furthermore, urinary and serum miR-16 levels were higher in those JIA patients who had the lowest patient global assessment (PGA) evaluation in compare to higher scores (PGA≤2 *vs.* ≥6, p=0.022 and *vs.* 3-5, p=0.041, respectively). Regarding the medications, serum miR-16 level was decreased in Methotrexate- *vs.* anti-TNF-treated group (mostly R0 *vs.* R1, p=0.067), resembling the lowest miR-16 abundancy in active disease. Moreover, male patients had lower urinary levels of miR-146a than females (p=0.011), and serum miR-155 was less abundant in younger patients (p>0.05). Bodily fluid comparison showed significantly higher levels of miR-146a in serum samples than in urine (p=0.005), whereas higher levels of miR-155 were detected in urine (p=0.013). Serum miR-16 demonstrated high diagnostic potential (AUC=0.85; p=0.017) with 85% sensitivity and 80% specificity.

**Conclusion:** Findings of the study suggest that inflammatory miR-16 analysis in bodily fluids of JIA patients can be considered as potential diagnostic tool for disease activity and could be applied for non-invasive monitoring of children with JIA.

**Patient Consent:** Yes, I received consent

**Disclosure of Interest**: None declared

### P075. Cardiovascular risk assessment in children with juvenile idiopathic arthritis based on aerobic capacity, echocardiographic and laboratory parameters

#### A. Stasiak^1^, A. Ryk^2^, J. Stańczyk^1^, E. Smolewska^1^

##### ^1^Department of Pediatric Cardiology and Rheumatology, ^2^Department of Biostatistics and Translational Medicine, Medical University of Lodz, Lodz, Poland

###### **Correspondence:** A. Stasiak

**Introduction:** Juvenile idiopathic arthritis (JIA) is the most common chronic rheumatic disease in children. It is believed that children with JIA have lower cardiopulmonary capacity and worse exercise tolerance. The gold standard for assessing physical fitness is aerobic fitness, commonly referred to as the maximum or peak oxygen uptake volume (VO2 peak) measured during a maximum load exercise test. Reduced aerobic fitness may play a key role in predicting the health of JIA patients as it has been associated with cardiovascular diseases and increased adult mortality. The likely cause of this significant impairment is multifactorial and should be investigated to improve treatment strategy. Exercise programs should be applied and individualized or at least modified according to different types of the disease to improve aerobic fitness.

**Objectives:** The aim of this study was to assess the oxygen capacity of adolescents with JIA along with echocardiographic and laboratory parameters in order to determine a group of patients with increased risk of developing cardiovascular diseases in comparison with healthy individuals.

**Methods:** Determining cardiopulmonary parameters such as peak oxygen consumption, tidal volume, minute ventilation, exercise time, heart rate or oxygen pulse in combination with echocardiographic parameters and early markers of heart failure, such as NT-proBNP allowed to identify a group of patients with potential risk of developing cardiovascular disease, including heart failure. Patients were assessed based on parameters such as age, sex, BMI, type of JIA, disease activity, laboratory parameters, treatment.

**Results:** Study consisted of 50 patients with JIA and 50 healthy patients who served as a control group. Patients with JIA had lower median values of peak VO2 (29.05 vs 38.02 ml/min/kg, p<0.001), O2 pulse (7.00 vs 11.40 ml/beat, p<0.001), minute ventilation (55.5 vs 84.5 l/min, p<0.001), oxygen uptake efficiency slope (1.62 vs 2.17, p<0.001), and cardiac output (8.25 vs 12.75 l/min, p<0.001) than in the control group. The ventilatory anaerobic threshold (VAT) was achieved earlier and at lower VO2 values ​​in children with JIA (p=0.0001). Children with JIA also had lowered respiratory parameters such as maximum voluntary ventilation (p= 0.0031) and tidal volume (p=0.0002). Echocardiography revealed a significantly lower shortening fraction (p=0.0389); the ejection fraction was also lower in the JIA group, but it was not statistically significant. Fourteen patients (28%) had enlarged right ventricular dimensions in relation to BSA. Two patients had E/A on the mitral valve greater than 2, indicating a higher risk of left ventricular diastolic dysfunction**.** NT-proBNP levels of all but four patients were within norm range. Twenty-two patients attended any form of physical activity and they had significantly higher peak VO2 (p=0.0099) and ΔVO_2_/ΔWR relationship (p=0.0041) values than JIA patients who were not physically active. There were no statistically significant correlations between peak VO2 and disease duration and treatment.

**Conclusion:** Children with JIA show moderate to severe physical impairment compared to healthy peers. Some of the patients had abnormalities in laboratory tests and echocardiographic examination, which may suggest an increased risk of cardiovascular complications. Patients who were physically active had significantly better aerobic capacity than those who were not physically active. Exercise programs tailored to the patient’s abilities should be implemented to reduce the risk of developing cardiovascular diseases.

**Patient Consent:** Yes, I received consent

**Disclosure of Interest**: None declared

### P076. The relationship between pain, kinesiophobia and proprioception in children with juvenile idiopathic arthritis

#### E. Tarakcı^1^, E. P. Kısa^2^, G. Leblebici^3^, O. Kasapcopur^4^

##### ^1^Health Science Faculty, Department of Physiotheraphy and Rehabilitation, Istanbul University Cerrahpasa, ^2^Health Science Faculty, Department of Physiotheraphy and Rehabilitation, Biruni University, Istanbul, ^3^Health Science Faculty, Department of Physiotheraphy and Rehabilitation, Medeniyet University, İstanbul, ^4^Department of Pediatric Rheumatology, Cerrahpaşa Faculty of Medicine, Istanbul University Cerrahpasa, Istanbul, Turkey

###### **Correspondence:** E. Tarakcı

**Introduction:** Juvenile idiopathic arthritis (JIA) is a group of diseases that can affect many joints and systems with problems such as pain, joint stiffness, muscle atrophy and weakness. Pain is one of the most frequently reported conditions. The thought that pain may cause an exacerbation of the disease creates a fear and there may be significant barriers to increasing the level of activity. In the case of pain caused by the disease, kinesiophobia may develop in children.

**Objectives:** The aim of the study is to evaluate the perception of kinesiophobia and proprioception due to pain status in children with knee involvement.

**Methods:** Fifteen patients aged between 4 and 16 who were followed up with rheumatism diagnosis and treatment in Istanbul University Cerrahpaşa Health Sciences Physiotherapy and Rehabilitation Unit were included in the study. Sociodemographic information, involved joint location and disease duration of all cases who voluntarily accepted to participate in the study were questioned with a case report form. “Visual analog scale (VAS)” was used for pain, “Tampa Kinesiophobia scale (TKS)” for kinesiophobia, and “universal goniometer” for proprioception assessment. The target angle to be reached was determined as 60 degrees of knee flexion. The difference between the targeted angle and the achieved angle was recorded as the “achieved proprioception angle”. Statistical analysis of the data was performed using the SPSS 24.0 (Statistical Package for Social Sciences) program. P≤0.05 was considered statistically significant in all analyzes.

**Results:** The age of 15 children (4 boys, 11 girls) included in the study was calculated as 11.16±2.44, while the disease duration was calculated as 42.20±23.30 months. Pain status were determined as 4±2.39 (min:0- max:7). While there was a positional correlation between reaching the “achieved proprioception angle” by disease duration and TKS (p≤0.05), a positive correlation was found between reaching the proprioception angle determined by TKS and pain status (p≤001). No significant correlation was found between disease duration and pain status.

**Conclusion:** It showed that the sensation of pain due to the disease in children with JIA may cause fear and avoidance behavior towards movement in children. Considering the duration of the disease, the importance of directing the children to movement with methods that will relieve pain in the early period and adding proprioception exercises to the treatment made us think. Although the small number of patients seems to be a limitation in the results of our study, it is not easy to use the TKS in children. The sample group of our study continues to be expanded.

**Patient Consent:** Yes, I received consent

**Disclosure of Interest**: None declared

**Table 1 (abstract P076).** See text for description

Parameters	rho	p
TKS-Pain status	**0,90**	≤0,001*
TKS-Achieved proprioception angle	**0,69**	≤0,001*
TKS-Duration	**0,62**	≤0,001*
Pain status-TKS	**0,90**	≤0,001*
Pain status-Achieved proprioception angle	**0,59**	≤0,001*
Pain status-Duration	0,43	0,10
Achieved proprioception angle-Pain status	**0,59**	≤0,001*
Achieved proprioception angle-Duration	**0,34**	≤0,001*
Achieved proprioception angle-TKS	**0,69**	≤0,001*

### P077. Adalimumab and anti-drug antibodies in a cohort of children with juvenile idiopathic arthritis: a single-center experience

#### G. Tarantino^1^, D. Pires Marafon^1^, F. Comitini^2^, R. Simeoli^3^, A. Aquilani^1^, R. Nicolai^1^, E. Marasco^1^, F. De Benedetti^1^, S. Magni Manzoni^1^

##### ^1^Rheumatology, Ospedale Pediatrico Bambino Gesù, Rome, ^2^Paediatrics, Ospedale Di Cagliari, Cagliari, ^3^Metabolic Disease, Ospedale Pediatrico Bambino Gesù, Rome, Italy

###### **Correspondence:** G. Tarantino

**Introduction:** Adalimumab (ADA), a fully humanized antibody against tumor necrosis factor (TNF)-α, has revolutionized treatment of patients with juvenile idiopathic arthritis (JIA). Although most of these respond within the first weeks, a minority may show loss of response (LOR) after continued exposure. Many studies demonstrate the influence of anti-adalimumab antibodies (AAA) on serum drug concentrations and clinical outcome in adults. However, little information about AAA and LOR is available for children with JIA.

**Objectives:** To describe demographic and clinical features in a single-center cohort of JIA patients treated with ADA, grouped according to frequency of drug administration (1W vs 2W); to assess ADA levels versus AAA titer and, finally, to investigate possible correlation between LOR to ADA and AAA.

**Methods:** Records of JIA patients on ADA treatment were retrospectively reviewed with focus on medical history and ELISA (enzyme-linked immunosorbent assay) ADA/AAA levels in a 6-month-period (Feb 2021-Jul 2021). Children with idiopathic uveitis were excluded. Samples furthest from last drug administration was defined as “trough level”. Data were analyzed via descriptive statistics (STATA 15.1).

**Results:** Of 51 JIA patients treated with ADA (39% females), 28 had ANA positive oligoarthritis with almost one large joint involvement at disease onset (T0). None of them presented RF-positive polyarthritis, nor systemic JIA (s-JIA). The median age at T0 was about 3 years. Half of study cohort was b-DMARDS naive at ADA start (T1), while all children were on c-DMARDs. Chronic recurrent uveitis was the main reason for ADA starting, followed-by tenosynovitis and spine or hip active arthritis. At the first ADA/AAA sampling (T2), approximately 10% of patients had active disease, with elbows or wrists as the most frequently involved. Extreme variability was observed between AAA titers (median 8.4 AU/ml) and ADA levels (median 16.4 micrograms/ml), regardless from 1 or 2-weekly administration. ADA levels, compared to the days from last drug administration, reached plateau values corresponding to a pharmacokinetic steady state. Among both study groups, a possible inverse correlation between ADA and AAA was found. We identified 20 JIA patients with clinical (presence of arthritis or uveitis) or laboratory (ESR ≥15 mm/h; CRP> 0.5 mg/dL) disease activity. Among these in only 3 we found very high AAA titers (350.0 - 113.4 AU/mL). In the subgroup of 27 patients with “trough-level” sampling heterogeneous data about AAA titers and drug level distribution were observed.

**Conclusion:** Our preliminary data showed a possible association between occurrence of AAA and lower ADA levels. However, a targeted risk analysis about high AAA titers and LOR incidence was not available. Monitoring of drug immunogenicity should be implemented in daily practice and become subject of future studies JIA.

**Patient Consent:** Yes, I received consent

**Disclosure of Interest**: None declared

### P078. Intra-Articular Glucocorticoid Injections (IAGI) in Juvenile Idiopathic Arthritis (JIA): a single center experience straddling COVID-19 pandemic

#### G. Tarantino, D. Pires Marafon, A. Aquilani, R. Nicolai, E. Marasco, F. De Benedetti, S. Magni Manzoni

##### Rheumatology, Ospedale Pediatrico Bambino Gesù, Rome, Italy

###### **Correspondence:** G. Tarantino

**Introduction:** IAGI allow precise release of steroids in inflamed sites, with proven efficacy in JIA. In younger children and in multiple injections procedures IAGI usually require general sedation, whereas local anesthesia in older patients and in single/few injection(s) procedures is well tolerated. It is not known whether COVID-19 pandemic conditioned the access of JIA children to IAGI and the procedure setting.

**Objectives:** To observe the frequency and the features of IAGI procedures in JIA patients straddling COVID-19 pandemic.

**Methods:** Records of JIA patients, who underwent IAGI from January 2019 to May 2021, with available information in the medical reports were retrospectively reviewed. Demographic and clinical features, including type, number and setting of intra/peri-articular injections, were registered. Data were analyzed through descriptive statistics.

**Results:** Of a total of 297 Caucasian patients included in the study (78.8% females), 152 (51.2%) had persistent oligoarthritis, 46 (15.5%) extended oligoarthritis, 89 (30.0%) polyarthritis (only 4 RF-positive), 6 (2.0%) systemic arthritis, 2 (0.7%) psoriatic arthritis and 2 (0.7%) enthesitis-related arthritis (ERA). The median age at IAGI was of 9.4 years in patients undergone general sedation, and of 16.2 years in those in local anesthesia. At GCs injection, 167 patients (56.2%) were off therapy, 77 (25.9%) on treatment only with Methotrexate, 30 (10.1%) with TNF-inhibitors association. During the study period, 434 IAGI were practiced , without significant differences in frequency of procedures between the time interval before and after the COVID-19 pandemic outbreak (Nr.16/month and 14/month, respectively) and the setting (general sedation in 73% and 70%, respectively) . The median age of children at IAGI was of 9.4 yrs in those who underwent general sedation, whereas was of 16.2 yrs in those treated in local anesthesia. Most of patients (73%) underwent only one procedure, 19% two and 9% three or more. Of the 149 procedures with single injection, 69% were performed in local anesthesia. Of note, in 117 procedures with multiple injections (27%) 5 or more sites were injected. Of a total of 396 sites injected, 80% were large joints, 47% tendons/bursae, 40% small joints. As expected, the knee, the tibiotalar, the elbow and the wrist were the most frequently injected joints (55.3%, 36.9%, 16.4%, 14.8% respectively). Notably, all of hip injections (n=6) were performed after the COVID-19 outbreak.

**Conclusion:** The frequency of IAGI procedures in JIA patients did not change with the COVID-19 outbreak at the study center. The knee, the tibiotalar, the elbow and the wrist were the most frequently injected joints. Hip injection were performed only during COVID-19 pandemia. Our results suggest that JIA patients and their caregivers were not limited by the pandemic in accessing the procedures and/or rely on the benefits of this treatment strategy despite potential logistic difficulties

**Patient Consent:** No, I have not receive consent

**Disclosure of Interest**: None declared

**Table 1 (abstract P078).** See text for description

		Pre COVID-19 outbreak	Post COVID-19 outbreak	Pre COVID-19 outbreak	Post COVID-19 outbreak
**N° JOINTs (%)**	**396**	**157**	**140**	**49**	**50**
**Temporo-mandibular**	**21 (4.8)**	**6 (3.7)**	**13 (8.9)**	**2 (3.2)**	**0 (0.0)**
**Elbow**	**71 (16.4)**	**31 (18.9)**	**32 (21.9)**	**2 (3.2)**	**6 (9.7)**
**Wrist**	**64 (14.8)**	**34 (20.7)**	**25 (17.1)**	**1 (1.6)**	**4 (6.5)**
**Small joints of hand**	**81 (18.7)**	**40 (24.4)**	**29 (19.9)**	**10 (16.1)**	**2 (3.2)**
**Hip**	**6 (1.4)**	**0 (0.0)**	**6 (4.1)**	**0 (0.0)**	**0 (0.0)**
**Knee**	**240 (55.3)**	**96 (58.5)**	**89 (61.0)**	**25 (40.3)**	**30 (48.4)**
**Tibiotalar**	**160 (36.9)**	**78 (47.6)**	**69 (47.3)**	**7 (11.3)**	**6 (9.7)**
**Small joints of foot**	**35 (8.1)**	**17 (10.4)**	**17 (11.6)**	**0 (0.0)**	**1 (1.6)**

### P079. Children with extended oligoarticular and polyarticular juvenile idiopathic arthritis have a cytokine profile in peripheral blood sustaining b cell activation

#### C. Tomé^1^, F. Oliveira-Ramos^1,2^, R. Campanilho-Marques^1,2^, A. F. Mourão^3,4^, S. Sousa^5^, A. T. Melo^1,6^, R. L. Teixeira^1,6^, A. P. Martins^7^, S. Moeda^8^, P. Costa-Reis^1,8^, R. P. Torres^3,4^, H. Fonseca^8^, M. Gonçalves^7^, M. J. Santos^1,5^, L. Graca^1,9^, J. E. Fonseca^1,6^, R. A. Moura^1^

##### ^1^Instituto de Medicina Molecular João Lobo Antunes, Faculdade de Medicina, Universidade de Lisboa, Lisbon Academic Medical Center, ^2^Pediatric Rheumatology Unit, Centro Hospitalar Universitário Lisboa Norte, EPE, Hospital de Santa Maria, Lisbon Academic Medical Center, ^3^Rheumatology Department, Centro Hospitalar Lisboa Ocidental, EPE, Hospital de São Francisco Xavier, ^4^NOVA Medical School, Faculdade de Ciências Médicas, Universidade Nova de Lisboa, Lisbon, ^5^Reumatology Department, Hospital Garcia de Orta, Almada, ^6^Rheumatology Department, Centro Hospitalar Universitário Lisboa Norte, EPE, Hospital de Santa Maria, Lisbon Academic Medical Center, ^7^Pediatric Surgery Department, Centro Hospitalar Universitário Lisboa Norte, EPE, Hospital de Santa Maria, Lisbon Academic Medical Center, ^8^Pediatric Department, Centro Hospitalar Universitário Lisboa Norte, EPE, Hospital de Santa Maria, Lisbon Academic Medical Center, Lisbon, ^9^Instituto Gulbenkian de Ciência, Oeiras, Portugal

###### **Correspondence:** F. Oliveira-Ramos

**Introduction:** Juvenile idiopathic arthritis (JIA) is the most common pediatric rheumatic disease and encompasses several different categories. Recently, our group has described that most patients diagnosed with the categories of extended oligoarticular JIA (eoJIA) and polyarticular JIA (pJIA) fulfil classification criteria for rheumatoid arthritis (RA) in adulthood. Moreover, we have also shown that B cell alterations and a B-cell related cytokine pattern are present since the first weeks of RA development. Therefore, we hypothesized that eoJIA and pJIA could also exhibit a cytokine profile in peripheral blood sustaining B cell activation.

**Objectives:** The main goal of this study was to evaluate a panel of proinflammatory and anti-inflammatory cytokines and chemokines relevant for B cell triggering and function in serum samples of eoJIA and pJIA when compared to healthy controls and persistent oligoarticular JIA (poJIA).

**Methods:** Blood samples were collected from eoJIA (n=15), pJIA (n=19) and poJIA (n=28) patients treated with disease modifying anti-rheumatic drugs. A group of age-matched healthy individuals (n=16) was used as control. Serum levels of APRIL, BAFF, IL-1β, IL-2, IL-4, IL-6, IL-10, IL-17A, IL-21, IL-22, IFN-γ, PD-1, PD-L1, sCD40L, CXCL13 and TNF were measured by LEGENDplex^TM^ multiplex bead-based immunoassay and/ or ELISA in all groups included.

**Results:** Children with eoJIA and pJIA, but not poJIA, had significantly increased serum levels of APRIL and BAFF when compared to controls. Furthermore, APRIL and BAFF serum levels were significantly correlated in JIA patients. No significant differences were detected in serum levels of other cytokines between all groups analyzed.

**Conclusion:** Patients diagnosed with eoJIA and pJIA, but not poJIA, have elevated serum levels of APRIL and BAFF in comparison to controls. These results indicate an involvement of these cytokines in the pathogenesis of these JIA categories, suggesting a B cell activation profile. Nevertheless, influence of treatment on the pattern of cytokine environment cannot be excluded. Further studies on B cell immune responses should be explored in JIA.

**Patient Consent:** Yes, I received consent

**Disclosure of Interest**: None declared

### P080. Do we all score the physician global assessment in the same way? Results from a large international survey with 17 case scenarios

#### C. Trincianti^1^, M. Backström^2^, M. Tarkiainen ^3^, F. Bovis ^1^, T. Qiu^4^, M. Esi^5^, D. J. Lovell^6^, N. Ruperto^7^, B. Gottlieb^8^, P. Vähäsalo ^9,10,11^, A. Consolaro^1,7^

##### ^1^University of Genova, Genova, Italy, ^2^Department of Pediatrics, The Wellbeing Services County of Ostrobothnia, Vaasa, , ^3^New Children’s Hospital, Helsinki University Central Hospital and University of Helsinki, Helsinki, Finland, ^4^Department of Biostatistics and Epidemiology, Cincinnati Children’s Hospital Medical Center, Cincinnati, ^5^University of Washington and Seattle Children’s Hospital, Seattle, Washington, ^6^Cincinnati Children’s Hospital Medical Center, Cincinnati, Ohio, United States, ^7^Pediatric Rheumatology Unit, IRCCS Istituto G. Gaslini, Genova, Italy, ^8^Cohen Children’s Medical Center, Lake Success, New York, United States, ^9^Pedego Research Unit, University of Oulu, Oulu, ^10^Department of Children and Adolescents, Oulu University Hospital, Oulu, ^11^Medical Research Center Oulu, University of Oulu and Oulu University Hospital, Oulu, Finland

###### **Correspondence:** C. Trincianti

**Introduction:** The physician’s global assessment of disease activity (PhGA) is a key outcome measure of juvenile idiopathic arthritis (JIA). It consists of the rating of the overall level of child’s disease activity on a 100-mm or 21-numbered circle visual analogue scale (VAS), with anchors of ‘0 = no activity’ and ‘100 = maximum activity’. The PhGA is a complex construct that captures the examiner’s subjective appraisal of patient’s disease activity at the time of the visit. However, physicians do not always seem to use the VAS in a uniform way.

**Objectives:** Aim of the study is to show the heterogeneity in PhGA scoring by comparing the assessment of a large international cohort of pediatric rheuamtologists evaluating multiple JIA patient’s case scenarios proposed through a web-based survey

**Methods:** A questionnaire including 17 detailed JIA patient cases was sent electronically to all PRINTO and PR-COIN members. The cases were structured to represent a wide spectrum of clinical situations and the PhGA was assessed by the responders on a scale from 0 to 100. To demonstrate the consistency among the VAS scores provided by multiple physicians we assessed the inter-rater reliability using the intra-class correlation (ICC). ICC estimates and their 95% confidence intervals (CI) were calculated using the “psych” package available in R (version 3.5) based on a single-rating, 2-way random-effects model. ICC values less than 0.5 indicate poor reliability, values between 0.5 and 0.75 indicate moderate reliability, values between 0.75 and 0.9 indicate good reliability, and values greater than 0.90 indicate excellent reliability. Moreover, the variability of scoring in each case was compared by the interquartile range (IQR) of the PhGA scoring.

**Results:** Out of 491 physicians who completed the survey, 418 (85.1%) completed the VAS scoring for all the 17 patients. The ICC of this group was 0.53 (95%CI: 0.38-0.72) indicating moderate to poor reliability. Considering the IQR of the single cases, those with the highest variability were: Case 1 (a polyarticular JIA patient with no joint involvement but with active uveitis, IQR= 37); Case 17 (an enthesitis related arthritis subject with 1 swollen joint, several restricted joints, complaining of important pain and morning stiffness, IQR= 30.5); Case 13: a RF positive polyarticular arthritis child with 10 active and 21 limited joints with 0.5 as pain VAS and no morning stiffness (IQR=28).

**Conclusion:** The scoring of the PhGA seems to have a poor to moderate reliability throughout the world and, in some clinical pictures, the variability expressed as IQR of this measure result higher than the expected. Shared guidelines for scoring the PGA are therefore needed to obtain consistent patient assessment in clinical trials and routine practice.

**Patient Consent:** Not applicable (there are no patient data)

**Disclosure of Interest**: None declared

### P081. Natural antibodies levels depend on the activity of juvenile idiopathic oligoarthritis

#### E. Tsitsami^1^, I. Sarrigeorgiou^2^, M. Tsinti^1^, P. Lymberi^2^

##### ^1^First Department of Pediatrics, University of Athens Medical School, Children’s Hospital Aghia Sofia, ^2^Laboratory of Immunology, Department of Immunology, Hellenic Pasteur Institute, Athens, Greece

###### **Correspondence:** E. Tsitsami

**Introduction:** Natural (auto)antibodies (NAAs) are primarily polyreactive antibodies encoded by germline V-gene segments. They detect autoantigens and new antigenic determinants with low affinity, and present prior to the body encountering cognate antigens. Therefore, NAAs provide a first line of defense allowing time for a specific antibody response to be elicited. Moreover, they play several roles in the immune system, including regulation of B-cell responses, control of B-cell development, selection of the B-cell repertoire, protecting thus the organism against autoimmune diseases. The IgM-NAAs are the most abundant in the body and the best studied while IgA-NAAs against lipopolysaccharides (LPS) have been considered as an endogenous homeostatic mechanism with innate specificity to microbiota.

**Objectives:** Given the autoimmune nature of juvenile idiopathic oligoarthritis (JIO), this study was scheduled to examine the possible involvement of NAAs in its pathogenesis.

**Methods:** Seventy JIO patients (aged 8,13±4,45 years, 14 males) were enrolled. The IgM antibody activity against the hapten trinitrophenyl (TNP) (proposed as a surrogate measure for polyreactivity because it is a synthetic molecule not present in the environment, to which individuals are not normally exposed), actin (a highly conserved cytoskeletal protein) and F(ab’)_2_ IgG fragments (proposed as an index of immunoregulatory function) as well as concentrations of IgM ([IgM]) were measured by in-house ELISAs. Similarly, the IgA antibody activity against LPS and the concentration of IgA ([IgA]) were measured. Multivariate analysis was performed with ratios of specific IgM and IgA activities to the total IgM and IgA concentration as dependent variables and the clinical parameters of patients as independent variables.

**Results:** Patients with active JIO presented with ratios of IgM activities against TNP, actin and F(ab’)_2_ IgG fragment to the [IgM] as well as the ratio of IgA anti-LPS activity to [IgA] significantly lower than those with inactive disease. These findings were independent of patients’ gender and age, the age at disease onset, the duration of disease, the antinuclear antibodies (ANA) positivity and the presence of uveitis.

**Conclusion:** Our results provide evidence supporting the autoimmune etiology of JIO. They also postulate a contribution of IgM-NAAs in the emergence of oligoarthritis flares. It is well known that the lower activity of IgM NAAs induces disturbances in tissue homeostasis, in the modulation of the immunological response and in the apoptotic clearance of cells that could drive disease flares. Finally, the observed correlation between IgA anti-LPS NAAs and disease activity might be related to the recently uncovered involvement of microbiota in the disease pathogenesis.

**Patient Consent:** Not applicable (there are no patient data)

**Disclosure of Interest**: None declared

### P082. Body mass index is related to disease variables in young adults with juvenile idiopathic arthritis

#### A.-K. Tuomi^1^, K. Rebane^2^, E. D. Arnstad^3,4^, L. Berntson^5^, A. Fasth^6^, M. Glerup^7^, T. Herlin^7^, H. Kautiainen^8,9^, E. Nordal^10^, S. Peltoniemi^11^, M. Rygg^12,13^, V. Rypdal^14^, M. Zak^15^, K. Aalto^2^

##### ^1^Department of Pediatrics , New Children’s Hospital, Helsinki University Hospital, ^2^Department of Pediatrics, New Children’s Hospital, Helsinki University Hospital, Helsinki, Finland, ^3^Department of Pediatrics, Levanger Hospital, Nord-Trøndelag Hospital Trust, Levanger, ^4^Department of Clinical and Molecular Medicine, NTNU, Trondheim, Norway, ^5^Department of Women’s and Children’s Health, Uppsala University, Uppsala, ^6^Department of Pediatrics, Sahlgrenska Academy, University of Gothenburg, Gothenburg, Sweden, ^7^Department of Pediatrics, Aarhus University Hospital, Aarhus, Denmark, ^8^Primary Health Care Unit Kuopio, Kuopio University Hospital, Kuopio, ^9^Folkhälsan Research Center, Helsinki, Finland, ^10^Department of Pediatrics, University Hospital of North Norway and UIT, Tromso, Norway, ^11^Department of Pediatrics, Helsinki University Hospital, University of Helsinki, Helsinki, Finland, ^12^Department of Clinical and Molecular Medicine, NTNU, Norway, ^13^Department of Pediatrics, St. Olavs University Hospital , Trondheim, ^14^Department of Pediatrics, University Hospital of North Norway and UiT, Tromso, Norway, ^15^Department of Pediatrics, Rigshospitalet Copenhagen University Hospital, Copenhagen, Denmark

###### **Correspondence:** A.-K. Tuomi

**Introduction:** Obesity is a worldwide problem^1^ but relatively little is known about its influence on the disease activity, functional ability, and quality of life (QoL) of patients with juvenile idiopathic arthritis (JIA).

**Objectives:** To investigate the disease variables and QoL of patients with JIA divided into different body mass index (BMI) categories.

**Methods:** This study is a part of the population-based Nordic JIA cohort study. All newly diagnosed JIA patients were recruited from defined regions of Finland, Sweden, Norway, and Denmark between 1997-2000^2^. Altogether 434 patients attended a follow-up visit (mean disease duration 17.5 ± 1.7 years) and 355 were included. Patients were categorized according to their (BMI): BMI<25.0, BMI 25.0-29.9, and BMI≥30.0. Disease characteristics were collected, and QoL (Fatigue Severity Scale FSS, Pittsburg Sleep Quality Index PSQI, SF36) was measured.

**Results:** In total, 355 (81.7 %) patients were included (mean age 24,7 years, females 72 %). Fifty-five percent of patients in the group with BMI<25.0 had oligoarthritis, whereas in the group with BMI≥30.0 (obesity) 32 % belonged to either psoriatic or enthesitis-related JIA. BMI was also found to be related to higher disease activity score (DAS28).

Higher BMI was related to lower functional ability, measured by Health Assessment Questionnaire (HAQ) (p<0.001). Patients with higher BMI reported significantly more pain (p=0.005) and had higher number of tender joints (p=0.006). Likewise, they had significantly higher CRP and ESR (p=0.002) and global VAS (p<0.001). They reported lower functional ability, experienced more fatigue, and their sleep quality was assessed to be inferior. Significant relationship was also found in the two domains of QoL: Bodily pain and general health. BMI was not found to be correlated with physical activity.

**Conclusion:** BMI was related to disease activity, disability, and quality of life. The role of obesity in inflammatory conditions, like JIA, needs more attention in future research.

**References:** 1) www.who.int/health-topics/obesity 2) Glerup M, Rypdal V, Arnstad ED, Ekelund M, Peltoniemi S, Aalto K, et al. Long-Term Outcomes in Juvenile Idiopathic Arthritis: Eighteen Years of Follow-Up in the Population-Based Nordic Juvenile Idiopathic Arthritis Cohort. Arthritis Care and Research. 2020;72(4)

**Patient Consent:** Yes, I received consent

**Disclosure of Interest**: None declared

**Table 1 (abstract P082).** Disease variables and QoL in young adults with JIA in different BMI groups

Characteristics N=355	BMI <25.0 N=258	BMI 25.0-29.9 N=59	BMI ≥30.0 N=38	-*linearity
Disease duration	16.7(1.8)	16.6(1.4)	17.1(1.0)	0.29
DAS28	1.84(0.87)	1.91(0.97)	2.26(1.01)	0.013
HAQ	0.13(0.32)	0.33(0.59)	0.38(0.65)	<0.001
Pain, VAS	16.6(22.6)	24.5(27.7)	28.1(29.9)	0.005
Global, VAS	15.2(22.8)	26.2(29.9)	29.3(33.8)	<0.001
FSS, mean (SD)	3.1(1.5)	3.5(1.7)	3.6(1.6)	0.032
PSQI, mean (SD)	5.5(3.3)	6.0(4.1)	6.8(4.1)	0.039
SF36 - Physical	52.2(9.5)	49.9(10.8)	47.7(10.7)	0.004

### P083. Prevalence of autoimmune diseases in parents of children with juvenile idiopathic arthritis: results from the international pharmachild register

#### J. W. van Straalen^1^, S. de Roock^1^, V. Stanevicha^2^, L. Lamot^3^, A. Estmann Christensen^4^, I. Rumba-Rozenfelde^5^, C. Lazar^6^, Y. Uziel^7^, T. Arkachaisri^8^, N. M. Wulffraat^1^, N. Ruperto^9^, J. F. Swart^1^ on behalf of Paediatric Rheumatology INternational Trials Organisation (PRINTO)

##### ^1^Department of Pediatric Immunology and Rheumatology, Wilhelmina Children’s Hospital, Utrecht, Netherlands, ^2^Riga Stradins University, Children University Hospital, Riga, Latvia, ^3^University Hospital Center Zagreb, University of Zagreb School of Medicine, Zagreb, Croatia, ^4^Department of Pediatrics, Odense University Hospital, Odense, Denmark, ^5^Pediatric Rheumatology, University of Latvia, Riga, Latvia, ^6^Spitalul Clinic de Urgenta pentru Copii, Cluj-Napoca, Romania, ^7^Pediatric Rheumatology Unit, Department of Pediatrics, Meir Medical Centre, Kfar Saba, Israel, ^8^Translational Immunology Institute, DukeNUS Medical School, SingHealth Academic Medical Center, Singapore, Singapore, ^9^Clinica Pediatrica e Reumatologia, IRCCS Istituto Giannina Gaslini, Genoa, Italy

###### **Correspondence:** J. W. van Straalen

**Introduction:** Autoimmune diseases (ADs) tend to cluster within families. However, little is known about the disposition to AD among children diagnosed with juvenile idiopathic arthritis (JIA).

**Objectives:** To determine the prevalence of and factors associated with ADs in parents of children with JIA.

**Methods:** Data were collected from the international Pharmachild register. Characteristics were compared between patients with and without a family history of AD. Prevalence rates of familial ADs were calculated and compared with general population prevalence rates as reported in the literature.

**Results:** The most common ADs in parents of the included JIA patients (n = 8,673) are listed in Table 1. For several ADs, prevalence was higher compared to reported numbers in the general population. Clinical Juvenile Arthritis Disease Activity Scores (cJADAS) at study entry and last follow-up were not significantly different between patients with (n = 1,231) and without a family history of AD (n = 7,442) (*P* = 0.61 and *P* = 0.82, respectively). Factors associated with a family history of AD were older age at JIA onset (*P* < 0.01), Scandinavian residence (*P* < 0.01), enthesitis-related arthritis, psoriatic arthritis and undifferentiated arthritis (*P* < 0.01), ANA positivity (*P* = 0.03) and HLA-B27 positivity (*P* < 0.01).

**Conclusion:** Several ADs have an increased prevalence in parents of children with JIA. Familial AD therefore proves to be a risk factor for JIA development and certain diseases should not be overlooked during family health history at the diagnosis stage. A family history of AD is associated with the JIA subtype but does not influence the severity or disease course.

**References**1. Parisi R, Iskandar IYK, Kontopantelis E, Augustin M, Griffiths CEM, Ashcroft DM. National, regional, and worldwide epidemiology of psoriasis: systematic analysis and modelling study. *BMJ*. 2020;369. 2. Vanderpump MPJ. The epidemiology of thyroid disease. *Br Med Bull*. 2011;99(1):39-51. 3. Almutairi K, Nossent J, Preen D, Keen H, Inderjeeth C. The global prevalence of rheumatoid arthritis: a meta-analysis based on a systematic review. *Rheumatol Int 2020 415*. 2020;41(5):863-877. 4. Stolwijk C, Onna M van, Boonen A, Tubergen A van. Global Prevalence of Spondyloarthritis: A Systematic Review and Meta-Regression Analysis. *Arthritis Care Res (Hoboken)*. 2016;68(9):1320-1331. 5. Ng SC, Shi HY, Hamidi N, et al. Worldwide incidence and prevalence of inflammatory bowel disease in the 21st century: a systematic review of population-based studies. *Lancet*. 2017;390(10114):2769-2778. 6. Thierry S, Fautrel B, Lemelle I, Guillemin F. Prevalence and incidence of juvenile idiopathic arthritis: A systematic review. *Jt Bone Spine*. 2014;81(2):112-117. doi:10.1016/j.jbspin.2013.09.003

**Patient Consent:** Not applicable (there are no patient data)

**Disclosure of Interest**: None declared

**Table 1 (abstract P083).** Most common ADs in parents of children with JIA (n = 17,346) from the Pharmachild register

Autoimmune disease	Frequency	Prevalence per 100,000 (95% CI)	General population prevalence per 100,000
Psoriasis	369	2,127 (1,916 – 2,356)	140 – 1,990^1^
Autoimmune thyroid disease	275	1,585 (1,404 – 1,784)	Graves’ disease: 0 – 2,000^2^ Hashimoto’s thyroiditis: 0 – 7,000^2^
Rheumatoid arthritis	141	813 (684 – 959)	300 – 700^3^
Ankylosing spondylitis	136	784 (658 – 927)	20 – 350^4^
Inflammatory bowel disease	68	392 (304 – 497)	Ulcerative colitis: 2 – 505^5^ Crohn’s disease: 1 – 322^5^
Juvenile idiopathic arthritis	51	294 (219 – 387)	21^6^

### P084. How ready are adolescents with JIA for transfer to adult care?- parental perspective

#### A. Vermé, J. Granhagen Ljugner, C. Bartholdson

##### Karolinska Institutet, Stockholm, Sweden

###### **Correspondence:** A. Vermé

**Introduction:** In Sweden, approximately 1500-2000 children suffers from Juvenile Idiopathic Arthritis (JIA). The transfer from pediatric rheumatology care to adult rheumatology care is often perceived as difficult and creates a great deal of concern for the adolescents and their parents. Releasing control and allowing the adolescents to take greater responsibility is, for example, one if the difficulties in the transition process. It´s important that parents feel confident and ready to handover the responsibility to facilitate and give support to the adolescent during the transition process.

**Objectives:** The purpose of the study is to investigate parent’s perspective of how ready adolescents with Juvenile Idiopathic Arthritis (JIA) are to transfer to adult care and to take responsibility for their own health.

**Methods:** Parents to members of The Swedish National Organization for Young Rheumatics and parents to patients at the pediatric rheumatology clinic at Astrid Lindgren´s Children´s hospital in Sweden, aged 14-18, received an invitation to participate in the study by responding to questions included in the *Readiness for Transition questionnaire*.

**Results: Key results:**

- Most of the parents felt that their adolescents took responsibility for talking to the healthcare professionals when they visit the clinic but few took talked with healthcare professionals via phone.

- Only a few percent (3%) of the parents reported that the felt that their adolescents where fully ready to transfer to adult care.

The data consist of 96 parents who answered the questionnaire (women n= 67, men n= 28). The results show that parents perceive that adolescent’s responsibility varies greatly depending on the area of responsibility. When it came to areas concerning their own care parents reported that the adolescents took greater responsibility than when it came to for example taking contact whit the clinic in different ways.

Furthermore, the results show that a little more than a quarter of the parents felt that their adolescent didn’t take any responsibility for regularly blood test, but it was also little more than a quarter of the parents who felt that their adolescents almost always took responsibility for regularly blood test. The parents additionally reported that the adolescents often took responsibility for their medications, showing up at booked visits at the clinic and talking to the healthcare professionals. The areas that the parents reported that the adolescents took little responsibility for were booking visits to the clinic, talking on the phone with healthcare professionals and renewing prescriptions. When the parents were asked to tell how ready they felt their adolescent where to take full responsibility for their health only 4 % reported that the adolescent where fully ready and 43% reported that their adolescent was not ready at all. Similar results were reported when the parents where asked how ready the adolescents where to transfer to adult care. Only 3% reported that their adolescent was fully ready for transfer and 47% was not ready at all to transfer to adult care.

**Conclusion:** The result demonstrates that parents to Swedish adolescents with juvenile arthritis didn´t perceive that their adolescents where ready to transfer to adult care. Parents need support to be able to find strategies to help the adolescents to increase their participation and knowledge in their own care. One possible way to meet those needs is to introduce a structed transition program in pediatric rheumatology care in Sweden.

**Patient Consent:** Yes, I received consent

**Disclosure of Interest**: None declared

### P085. Adaptation and validation of the methotrexate intolerance scale (Miss - Methotrexate Intolerance Severity Score) in children with juvenile idiopathic arthritis

#### S. Zhukov, V. A. Malievsky

##### Pediactric, BSMU, Ufa, Russian Federation

###### **Correspondence:** S. Zhukov

**Introduction:** Juvenile idiopathic arthritis (JIA) is the most common rheumatic disease in children. According to modern criteria, JIA is arthritis of unknown etiology, which begins before the age of 16 years, with a duration of more than 6 weeks. International and domestic protocols for the management of children with this pathology provide for the early prescription of basic antirheumatic drugs, mainly methotrexate (MT). Methotrexate is widely used for the treatment of JIA as a starting, basic drug with proven efficacy, but at the same time, the possibility of long-term therapy with methotrexate may be limited by a number of undesirable effects, the risk of which increases in proportion to the duration of the drug. A group of Dutch scientists developed the Methotrexate Intolerance Inventory (MISS). which have proven to be highly effective in clinical practice. In Russia, in the Republic of Bashkortostan, the necessary questionnaire was validated, which made it possible to successfully introduce the methotrexate intolerance questionnaire into the work of the department.

**Objectives:** Adaptation and validation of the Russian version of the MISS questionnaire (assessment of the severity of methotrexate intolerance).

**Methods:** The text of the questionnaire was translated and tested at the departments of hospital pediatrics (head of the department - Doctor of Medical Sciences, Professor Malievsky V.A.) and foreign languages ​​​​with a Latin language course of the Bashkir State Medical University (head of the department - candidate of philological sciences, associate professor Mayorova O.A.). The validation procedure for the MISS questionnaire included language and cultural adaptation, which, according to international recommendations for the validation of questionnaires, took place in several stages. Under our supervision there were 100 children whose age at the onset of the disease ranged from 3 to 17 years inclusive (32 boys, 32.0% and 68 girls, 68.0%). The most common variant of JIA was polyarthritis (58 children, 58.0%). 36 children (36.0%) had persistent oligoarthritis. 3 children (3.0%) had juvenile arthritis with a systemic onset. Spreading oligoarthritis was diagnosed in 3 children (3.0%).

**Results:** The value of the ɑ-Kronbach coefficient according to the scales of the MISS questionnaire

All scales of the questionnaire have a ɑ-coefficient >0.7, which corresponds to the optimal level of internal constancy of the questionnaire adopted for group studies. In addition, these data are comparable with the results obtained by the authors of the original questionnaire.

**Conclusion:** The MISS questionnaire has been adapted and validated in accordance with generally accepted requirements. An analysis of the reliability of the Russian version of the questionnaire based on the calculation of the Cronbach’s alpha coefficient indicates its sufficient reliability and the possibility of use in clinical practice.

**Patient Consent:** Not applicable (there are no patient data)

**Disclosure of Interest**: None declared

**Table 1 (abstract P085).** See text for description

Questionnaire scales	ɑ-Kronbach coefficient
Stomach pain	0,76
Nausea	0,85
Vomiting	0,78
Behavioral complaints	0,89

## Poster session: Systemic JIA

### P086. Aortic stiffness comparison between healthy and juvenile rheumatoid arthritis children

#### P. Avramovski^1^, S. Stoilova^2^, M. Lazarevski^3^, S. Talev^4^, M. P. M. P. Avramovska^5^

##### ^1^Internal medicine, Clinical hospital D-r T. Panovski - Bitola, ^2^High medical scholl, St Klemet of Ohrid - Bitola, Bitola, ^3^Internal medicine, GOB 8th september, Skopje, ^4^Chirurgie, ^5^Gynecology and obstetric, Clinical hospital D-r T. Panovski - Bitola, Bitola, North Macedonia

###### **Correspondence:** P. Avramovski

**Introduction:** Aortic pulse wave velocity (PWV) is indicator of arterial stiffness. It is advances with age and seems accelerated in certain disease conditions like rheumatoid arthritis (RA), diabetes and chronic kidney disease.

**Objectives:** The aim of this study was to find the difference between PWVs between healthy and RA patients.

**Methods:** arotid-femoral PWV was assessed by pulsed-Doppler ultrasound synchronized with electrocardiography (ECG) in 65 healthy and 87 juvenile rheumatoid arthritis children aged 10.3 ± 4.1 years. Demographic data: age, height, weight, heart rate (HR) and high-sensitive C-reactive protein (hs-CRP) were measured or taken from a health record.

**Results:** The median PWV were: 4.52 (3.24 to 5.21) vs. 4.71 (3.47 to 5.56) in healthy and RA patients. PWV is significantly higher in RA patients (P = 0.0007). PWV significantly correlates with age (r = 0.245, P = 0.022), height (r = 0.312, P = 0.003), but not with HR (r = 0.176, P = 0.102) and weight (r = 0.187, P = 0.082) in RA patients group. PWV significantly correlated with CRP in juvenile RA patients (r = 0.241, P = 0.024), but not in healthy control (r = 0.179, P = 0.154).

**Conclusion:** Arterial stiffness or precisely PWV was more pronounced in the child with juvenile RA than in the healthy control. Hs-CRP as a marker of acute and low-grade inflammation is correlated with artery elasticity in juvenile RA patients

**Patient Consent:** No, I have not receive consent

**Disclosure of Interest**: None declared

### P087. Polyserositis as the initial presentation of systemic JIA

#### J. N. Bathia^1^, P. Pal^1^, I. R. Chaudhury^2^, P. P. Giri^3^, R. Kapoor^3^

##### ^1^Department of Pediatric Rheumatology, ^2^Department of Pediatric Medicine, ^3^Department of Pediatric Intensive Care, Institute of Child Health, Kolkata, India

###### **Correspondence:** J. N. Bathia

**Introduction:** In systemic onset juvenile idiopathic arthtitis (sJIA), polyserositis may precede the development of arthritis but they constitute rare and incidental findings

**Objectives:** to describe a three years old who presented with polyserositis to be later diagnosed as sJIA on development of arthritis.

**Methods:** Following a trauma to the left ankle this 3 year old boy presented with high grade fever for 15 days. This was associated with shortness of breath for 5 days. there was history of a transient erythematous rash over the trunk extremities 2 days prior to admission. On examination, the child was febrile, there was pallor, air entry was decreased in the right chest, abdominal examination revealed hepatosplenomegaly, ascites, and there a tender swelling over the left groin. Initial investigations revealed: Hb-7.9 gm/dL, total leucocyte count was 43. 6 x 10^3^/μL neutrophils 79%, lymphocytes 18%, Platelet-120 x 10^3^/μL ,serum albumin 2.3 g/dL, serum globulin 5.8gm/dL, serum ferritin 534 ng/ml, CRP- 168 mg/L. Initial Chest Xray was apparently normal however a ultrasonography of the chest done a few days later revealed right sided pleural effusion. USG guided pleural tap was done which revealed fibrinopurulent collection. Ascitic tap was also done. Blood and pleural fluid culture revealed no growth

**Results:** Considering sepsis the child was started on broad spectrum antibiotics. The fever was persistent despite adequate antibiotic coverage of 2 weeks. Bone marrow and echo-cardiogram were normal. Tuberculosis ruled out. However, the fever still persisted. With a suspicion of an autoinflammatory disease child given pulse methyl prednisolone followed by oral prednisolone following which the fever subsided and the child’s condition improved. The child was then discharged on oral oral steroids. On follow up, as the steroid were tapered the child developed arthritis of bilateral ankle joints. At this juncture the diagnosis of systemic onset juvenile idiopathic arthritis (sJIA) was made. Subcutaneous methotrexate was added and the child currently remains in remission

**Conclusion:** Systemic onset juvenile idiopathic arthritis may present with persistent fever and polyserositis that may resemble a disseminated sepsis without any arthritis or specific sign to establish diagnosis.

**Patient Consent:** Yes, I received consent

**Disclosure of Interest**: None declared

### P088. Vaccination with SRP as a trigger of systemic juvenile idiopathic arthritis

#### H. Bermúdez Canales, E. Faugier, S. Rodríguez Aguayo, K. Primero Nieto, A. Barba Aguilar, A. Guzmán Revilla, N. De la Rosa Encarnación, P. Ramos Tiñini

##### Reumatología, Hospital Infantil de México Federico Gómez, Ciudad de México, Mexico

###### **Correspondence:** H. Bermúdez Canales

**Introduction:** Vaccines have been considered triggers of autoimmune diseases in genetically predisposed individuals through various mechanisms, such as molecular mimicry, the formation of immune complexes and the activation of polyclonal lymphocytes. However, for most patients, viral vaccines don’t carry a risk of systemic autoimmune disease and should be administered according to current recommendations because of their remarkable efficacy in reducing morbidity and mortality in all age groups.

**Objectives:** Describe the case of a patient who triggered Systemic Juvenile Idiopathic Arthritis after vaccination with SRP.

**Methods:** Caso clínico

**Results:** One year old female. Her condition began 5 days after administration of the vaccine (SRP). Clinical manifestations: evening fever (39°C) associated with salmon-colored and evanescent macular exanthema. She was diagnosed with Kawasaki disease and was treated with intravenous immunoglobulin (2grkgdo), acetylsalicylic acid (50mgkgday) and methylprednisolone (2mgkgday). Referred for treatment due to persistent symptoms and arthritis of the knees, carpals and ankles of 4 months of evolution. At evaluation, febrile, arthritis in shoulders, elbows, carpus, knees and ankles, lymphadenopathies in right anterior cervical region of 2 x 2 cm and bilateral inguinal region of 1 x 1 cm, in addition to erythematous macules predominantly in trunk, upper extremities and pelvic limbs, 2 to 5 mm in size were evident. Laboratory: leukocytosis with neutrophilia; anemia and thrombocytosis. Elevation of acute phase reactants (ESR and CRP). Renal and hepatic function preserved. Chest X-ray and echocardiogram without alteration. Negative infectious approach (blood cultures, urine culture, procalcitonin and viral serology). Oncological approach, bone marrow aspirate negative for malignancy. Diagnosis of Systemic Juvenile Idiopathic Juvenile Arthritis was integrated and treatment with prednisone (2mgkgday) and Tocilizumab (162mg subcutaneous every 2 weeks) was started, achieving clinically inactive disease.

**Conclusion:** There is limited data on the relationship of immunization and systemic autoimmunity. We report the exceptional case of Systemic Juvenile Idiopathic Juvenile Arthritis following vaccination with SRP. The comprehensive and thorough evaluation of the patient with systemic manifestations allows a timely diagnosis and initiation of appropriate treatment to control the disease without complications or sequelae.

**Disclosure of Interest**: None declared

### P089. Prolonged fever, rash, arthritis and hyperferritinemia in a child: a case of systemic idiopathic juvenile arthritis with suspicion of occult MAS

#### M. E. Caseiro Alves^1^, A. Sampaio Mesquita^2^, H. Sousa^2^, M. Paula Ramos^3^

##### ^1^Departamento de Pediatria Médica, Hospital De Dona Estefania - Centro Hospitalar e Universitário de Lisboa Central, ^2^Serviço de Pediatria, Hospital de Vila Franca de Xira, ^3^Unidade de Reumatologia Pediátrica, Hospital De Dona Estefania - Centro Hospitalar Universitário de Lisboa Central, Lisboa, Portugal

###### **Correspondence:** M. E. Caseiro Alves

**Introduction:** Systemic Juvenile Idiopathic Arthritis (sJIA), mediated by autoinflammatory cytokines, is an unique form and the most severe one of Juvenile Idiopathic Arthritis. Its diagnosis is usually challenging and a high index of suspicion is needed. Macrophage activation syndrome (MAS) is a known potentially fatal complication of sJIA which needs prompt treatment.

**Objectives:** To describe a case of severe SJIA complicated with MAS and to explore therapeutic options

**Methods:** An 11 year-old african male child, brought to the emergency room of a second level portuguese hospital, for a 1 month daily fever, anorexia, fatigue and joint pain with 8-Kg weight loss. The patient also reported feeling illness, with intermittent low-grade fever and an evanescent erythematous exanthem in the previous six months, already evaluated in a medical appointment. Upon observation, the patient appeared fatigued, febrile with an evanescent exanthema (face and trunk), hepatomegaly and bilateral arthritis of small joints of the hands and feet, wrists and ankles. Blood tests revealed microcytic hypochromic anemia (Hb 9.4 g/dL) with leukocytosis, neutrophilia, thrombocytosis, hypoalbuminemia, with elevated C-Reactive protein (15 mg/dL), erythrocyte sedimentation rate (VS 113 mm/h), fibrinogen and ferritin (1703 ng/mL). Cardiac evaluation was normal. Infectious, neoplasic and others auto-immune diseases were excluded.

**Results:** Ceftriaxone and ibuprofen were started. Progressive worsening in the next 24 hours with unremitting fever and prostration. Blood tests revealed a slight decrease of hemoglobin and increase in ferritin levels (15.637 ng/mL) and soluble CD 25. Concerning of installation of MAS, bolus of methylprednisolone was initiated. Treatment was maintained with prednisolone (PRD) and methotrexate (anti-IL1 was not authorized for the hospital administration) with improvement of systemic manifestations but refractory arthritis. Six months later, still in PRD, he was referred to a central hospital and interleukin-6 (IL-6) inhibitor was started with good disease control, allowing complete weaning of PRD 6 months later.

**Conclusion:** Diagnosis of sJIA may be rather difficult with unspecific manifestations, as reported in this case with several months of disease evolution and a severe presentation on admission.

Even though not fulfilling the 2016 criteria of MAS in sJIA, the clinical deterioration with extreme hyperferritinemia (>10,000ng/mL), strongly suggested rapidly evolving MAS. Prompt and life-saving treatment prevented the child of a high morbi-mortality risk.

Nonethless, in sJIA the precocious treatment with anti-IL1/6 is usually related to better prognosis.

**Patient Consent:** Yes, I received consent

**Disclosure of Interest**: None declared

### P090. Comparative analysis of serum biomarkers and peripheral blood gene expression in systemic juvenile idiopathic arthritis and macrophage activation syndrome

#### C. Hinze^1^, M. Saers^1^, C. Kessel^1^, G. Prencipe^2^, F. de Benedetti^2^, D. Föll^1^, C. Bracaglia^2^

##### ^1^Paediatric Rheumatology and Immunology, University Hospital Münster, Münster, Germany, ^2^Paediatric Rheumatology, Ospedale Pediatrico Bambino Gesù, Rome, Italy

###### **Correspondence:** C. Hinze

**Introduction:** Systemic juvenile idiopathic arthritis (SJIA) is characterized by severe inflammation and may be complicated by life-threatening macrophage activation syndrome (MAS) which is driven by activation of the interleukin (IL)-18-interferon (IFN)γ-axis. How peripheral blood gene expression and respective serum biomarkers are related in SJIA and MAS is incompletely understood.

**Objectives:** To evaluate the relationship of peripheral blood innate immunity-driven gene expression and related serum biomarkers in a cohort of patients with SJIA in different disease states.

**Methods:** Seventy-five whole-blood-derived RNA and parallel serum samples from 45 patients (median age at sample 6.2 years) from 2 centres were analyzed using a 24 gene custom NanoString panel including IL-1/NFkB, type 1 interferon (IFN), and type 2 IFN-related genes, and a 23-plex Luminex panel. The panels included 9 related targets, i.e., both gene in the NanoString panel and related protein in the Luminex panel (CXCL9/CXCL9, CXCL10/CXCL10, IL1A/IL-1a, IL1B/IL-1b, IL1RN/IL-1RA, IL6/IL-6, IL18/IL-18, S100A9/S100A9, and S100A12/S100A12. Four different disease states were considered (18 clinically inactive disease [CID], 37 active disease [AD], 9 MAS [as defined by ACR/EULAR], and 11 pre-MAS, i.e., prior to the development of full-blown MAS). Normalized NanoString counts and composite IL-1/NFkB-related, type 1 interferon (IFN) and type 2 IFN scores, and Luminex MFI values were used for comparisons. Correlation analyses were performed. Groups were compared using non-parametric statistics.

**Results:** There was mild to moderate correlation between the respective gene expression and serum protein levels as measured by Spearman rank correlation across all samples for the following genes/proteins: CXCL10 (r=0.40), IL6/IL-6 (r=0.40), IL1A/IL-1a (r=0.59), IL1B/IL-1b (r=0.45), IL18/IL-18 (r=0.40), S100A9/S100A9 (r=0.50) but not between CXCL9/CXCL9 and IL1RN/IL-1RA. When comparing the individual parameters across the different disease states, significant differences across disease states were seen for most of the serum proteins, and especially prominent for CXCL9, CXCL10, and IL-18. Strong correlations were seen, for example, between the following composite gene expression scores and serum proteins: IL-1/NFkB-related score with CXCL9 (r=0.60), IL-1a (r=0.64), IL-6 (r=0.72), S100A9 (r=0.55), type 1 IFN score with CXCL10 (r=0.58), and type 2 IFN score with FasL (r=0.85), CXCL10 (r=0.78), TNF (r=0.71) but not with CXCL9 (r=0.08).

**Conclusion:** In patients with SJIA in different disease states, a marked dysregulation of inflammation-related serum proteins is observed, and especially IL-18 and CXCL9 in pts with MAS or pre-MAS. There was a strong correlation between IL-1/NFkB-related, type 1 IFN, and type 2 IFN gene expression signatures with several of the serum proteins assessed. IL-1RA serum levels were strongly confounded by anakinra (recombinant IL-1RA) therapy, explaining a lack of correlation between IL1RN expression and IL-1RA levels. In summary, both assessment of serum biomarkers and peripheral blood gene expression signatures may be instructive when assessing different disease states in SJIA.

**Patient Consent:** Yes, I received consent

**Disclosure of Interest**: None declared

### P091. Speaking the same language: international cross-validation of emerging biomarkers for juvenile idiopathic arthritis - the 2022 CARRA-PRES collaborative research award

#### C. Kessel^1^, R. Marsh^2^, C. Wouters^3^, P. Proost^4^, P. Matthys^4^, D. Foell^1^, S. Canna^5^, G. Prencipe^6^, C. Bracaglia^6^, F. De Benedetti^6^, D. Dissanayake^7^, R. Laxer^7^, S. Vastert^8^, F. Minoia^9^, K. L. Brown^10^, D. A. Cabral^11^, G. Schulert^12^ on behalf of PReS sJIA/MAS working party

##### ^1^Department of Pediatric Rheumatology & Immunology, University Children’s Hospital Muenster, Muenster, Germany, ^2^Division of Bone Marrow Transplantation and Immune Deficiency, Cincinnati Children’s Hospital, Cincinnati, United States, ^3^ Department of Pediatrics, University Hospitals Leuven, ^4^Department of Microbiology, Immunology and Transplantation, Rega Institute KU Leuven, Leuven, Belgium, ^5^Children’s Hospital of Philadelphia and University of Pennsylvania Perelman School of Medicine, Philadelphia, United States, ^6^Bambino Gesù Children’s Hospital, Rome, Italy, ^7^Department of Pediatrics, Hospital for Sick Children, Toronto, Canada, ^8^Department of Pediatric Rheumatology and Immunology, University Medical Center Utrecht, Utrecht, Netherlands, ^9^Pediatric Rheumatology, Fondazione IRCCS Ca’ Granda Ospedale Maggiore Policlinico, Milan, Italy, ^10^Department of Pediatrics, British Columbia Children’s Hospital Research Institute, ^11^BC Children’s Hospital University of British Columbia, Vancouver, Canada, ^12^Cincinnati Children’s Hospital, Cincinnati, United States

###### **Correspondence:** C. Kessel

**Introduction: :** Over the past decade, several studies have validated the use of key biomarkers such as IL-18, CXCL9 and S100 proteins in diagnosis and follow-up of children with polyarticular course and systemic juvenile idiopathic arthritis (JIA). Despite the promise of these biomarkers, their utility is limited by their availability in only some specialized labs and medical centers. In addition, as opposed to CRP the appropriate reference ranges for these biomarkers is not standardized. Finally, even though good correlation of biomarker levels acquired in different centers using different assays may occur, absolute values can vary substantially. Collectively, this hampers a wider introduction of these markers into routine clinical care.

**Objectives:** In this project we set out to cross-validate emerging JIA, systemic JIA and MAS biomarkers across different measurement platforms and different international centers involved in CARRA and PReS.

**Methods:** We have assembled an international consortium of four CARRA Registry sites (Cincinnati, Philadelphia, Toronto, Vancouver) and four PReS (Leuven, Muenster, Rome, Utrecht) clinical research centers. In a first step we will distribute healthy donor serum samples spiked with defined concentrations of recombinant S100 proteins, CXCL9 and IL-18 to all participating centers. IL-18, S100 proteins, and CXCL9 will be determined using locally used platforms including specific ELISA, luminex, mesoscale and Ella assay. In a second step patients’ samples enrolled in the FROST study will be shipped to co-investigators labs for respective biomarker analyses. Following this all data will be analyzed for correlation coefficients and spike recovery to determine agreement across identical platforms in different labs. Linear fit models will be used to determine conversion of results across different platforms.

**Results:** Data collection is still in progress and ongoing.

**Conclusion:** The findings of this project will be highly significant because broad standardization of biomarker levels and interpretation across centers is essential for international collaborative research. Our efforts can strongly impact any future (clinical) research study in the field and beyond, that involves IL-18/CXCL9/S100 protein-driven differential diagnosis, disease activity assessment or guided treatment. The results from our study will enable wide interpretation and translation of respective biomarker data and pave the way towards their wider use in routine clinical practice.

**Disclosure of Interest**: C. Kessel Grant / Research Support with: Novartis, Consultant with: Novartis and SOBI, R. Marsh: None declared, C. Wouters: None declared, P. Proost: None declared, P. Matthys: None declared, D. Foell Grant / Research Support with: Novartis, SOBI, Pfizer, Consultant with: Novartis, SOBI, Chugai-Roche, S. Canna Consultant with: Novartis, SOBI, AB2 Bio, Johnson & Johnson, Simcha Therapeutics, G. Prencipe: None declared, C. Bracaglia Consultant with: Novartis, SOBI, F. De Benedetti Consultant with: Hoffmann-La Roche, Novartis, AbbVie, Novimmune, and SOBI, D. Dissanayake Grant / Research Support with: Gilead Sciences, Consultant with: Novartis, R. Laxer Consultant with: SOBI, Novartis, Eli Lilly Canada, Sanofi, S. Vastert Grant / Research Support with: Novartis and SOBI, Consultant with: Novartis and SOBI, F. Minoia: None declared, K. Brown: None declared, D. Cabral: None declared, G. Schulert Consultant with: Novartis

### P092. Predictors of remission in children with systemic juvenile idiopathic arthritis receiving tocilizumab: a retrospective cohort study

#### E. Krekhova^1,2^, E. Alexeeva^1^, T. Dvoryakovskaya ^1^, K. Isaeva^2^, R. Denisova ^2^, A. Chomakhidze ^2^, O. Lomakina ^2^, A. Mamutova ^2^, A. Fetisova ^2^, M. Gautier ^2^, C. Chibisova ^2^, I. Kriulin ^1^, I. Tsulukia ^2^

##### ^1^Pediatric, Sechenov First Moscow State Medical University, ^2^Rheumatology, National Medical Research Center of Children’s Health, Moscow, Russian Federation

###### **Correspondence:** E. Krekhova

**Introduction:** Tocilizumab (TOC) is a highly effective biological drug for therapy of systemic juvenile idiopathic arthritis (sJIA). The identification of early prognostic factors of response to TOC is necessary to increase treatment efficacy and predict long-term outcomes in children with sJIA.

**Objectives:** This study aimed to identify possible predictors of response to the TOC treatment in patients with sJIA.

**Methods:** We analyzed retrospectively the 258 prescribing of TOC to the patients with sJIA treated at the National Medical Research Center of Children’s health, Moscow, Russia (initiation between July 2009 and February 2021). Predicted outcomes included inactive disease after 12 months of therapy with TOC (according to C. Wallace criteria and JADAS71). Probable prognostic factors (n=115) included baseline demographic, clinical, laboratory variables, previous or concomitant therapies and changes some of them on the 1^st^ and 3^rd^ month of the therapy. We determined predictors of response using multivariate logistic regression analysis.

**Results:** After 12 months of TOC therapy, in 179 cases (76%) was achieved inactive disease according to C. Wallace criteria and in 170 cases (72%) was achieved JADAS remission.

The predictors of achieving the stage of inactive disease/remission according to C. Wallace criteria by the 12 month of TOC therapy were: duration of morning stiffness at sJIA onset, its dynamics after 1 and 3 months of treatment, the physician global assessment of disease activity after 1 month of therapy, and the patient/parent global assessment of overall well-being after 3 months of therapy.

The duration of morning stiffness at sJIA onset and upon treatment initiation, the physician global assessment of disease activity after 1 and 3 months, and number of painful joints after 3 months were associated with remission according to JADAS71 index by the 12 month of TOC therapy.

**Conclusion:** Our results showed that the characteristics of joint syndrome before and 1–3 months following treatment initiation, dynamics of objective and subjective assessment of disease activity after 1-3 months of therapy, could predict the effectiveness of TOC by 1 year of therapy in sJIA patients.

**Patient Consent:** Yes, I received consent

**Disclosure of Interest**: None declared

### P093. SARS-COV-2 in systemic juvenile idiopathic arthritis: can ANTI-IL-1 drugs prevent MIS-C? A monocentric data collection

#### M. C. Maggio, G. Corsello

##### PROMISE “G. D’Alessandro”, University of Palermo, Palermo, Italy

###### **Correspondence:** M. C. Maggio

**Introduction:** The worldwide pandemic of SARS-CoV-2 continues to have a serious impact on global health care across the world. The risk of severe COVID-19 is of concern for patients with rheumatic diseases and especially systemic Juvenile Idiopathic Arthritis (sJIA) using disease-modifying anti-rheumatic drugs (DMARDs) including biologics. Even if COVID-19 shows to be milder in children than in adults, multisystem inflammatory syndrome in children (MIS-C) is a fearful and a life-threatening complication.

**Objectives:** The international literature showed that COVID-19 underwent without serious complications in sJIA patients controlled by biologics, but not by steroid therapy. Many studies sustain the hypothesis that uncontrolled sJIA and its underlying inflammatory state are a risk factor for severe COVID-19 in children, and that immunosuppression associated with prolonged corticosteroid treatment represents another risk factor for severe disease.

**Methods:** We describe the case series of 5 patients affected by sJIA, acquiring SARS-CoV-2 infection. 4/5 was treated for sJIA with anti-IL-1 beta biologic drug, at the dosage of 4 mg/kg/4 weeks and showed a good control of the sJIA. One patient, with a concomitant mutation of NLRP-3, was treated with anakinra and prednisone for the difficult control of the disease. 60% of the patients previously received 2 doses of the SARS-CoV-2 vaccine.

**Results:** All the patients showed persistent fever (100%), arthralgia and or arthritis (80%), respiratory symptoms (100%), with a good saturation in air, abdominal pain (40%). They were treated with ibuprofen, and they continued anti-IL-1 treatment. The acute phase resolved in a variable period of 5-10 days and the haematological parameters returned to the normal range.

The patient with the worse control of the disease and the NLRP3 mutation, 4 weeks later, showed fever, rash, cheilitis, conjunctivitis, significant increase of AST, ALT, gamma-GT, pro-BNP, troponin. The diagnosis of MIS-C was defined, and the treatment choice was with IVIG (2 gr/kg), methylprednisolone (2 mg/kg/day i.v.) and the increase of anakinra dose to 4 mg/kg/day. The acute phase of the disease was controlled, with the complete recovery of the child.

**Conclusion:** Our case series confirm a good outcome of the disease in children with SARS-CoV-2 infection, when the rheumatic disease is well controlled. The NLRP3 gene mutations increase IL-1 beta synthesis, with and underlying amplification of the risk of MIS-C. furthermore, the undergoing treatment with steroids in a patient with a bad control of sJIA is a further risk factor for a poor outcome. The vaccination with the SARS-CoV-2 vaccine can be a further protective factor for patients with sJIA.

**Disclosure of Interest**: None declared

### P094. The emerging role of cardiovascular magnetic resonance in the assessment of cardiac involvement in systemic juvenile idiopathic arthritis: early treatment for an early resolution

#### M. C. Maggio^1^, A. Alaimo^2^, F. Finazzo^3^, G. Corsello^4^

##### ^1^University Department PROMISE “G. D’Alessandro”, University of Palermo, ^2^U.O.C. of Paediatric Cardiologic Division, Children Hospital “G. Di Cristina”, ^3^U.O.C. of Paediatric Radiology, Children Hospital “G. Di Cristina”, ARNAS, ^4^University Department PROMISE “G. D’Alessandro”, University of Palermo, Palermo, Italy

###### **Correspondence:** M. C. Maggio

**Introduction:** Systemic Juvenile Idiopathic Arthritis (sJIA) may seriously damage various structures of the cardiovascular system. However, cardiovascular disease (CVD) is understudied and, than, underestimated in patients with sJIA, but chronic systemic inflammation associated with cardiovascular risk factors are causes of CVD. The initial evaluation of sJIA patients is based on non-invasive modalities, however frequently they fail to detect subclinical forms of CVD.

**Objectives:** Cardiovascular magnetic resonance (CMRI) can offer early and non-invasive information about CVD in sJIA, allowing timely starting of cardiologic and anti-rheumatic drugs.

**Methods:** A 11-year-old girl presented with flu-like symptoms, systemic inflammation with myocarditis, and cardiomyopathy. She was treated with paracetamol and FANS, however, for the worsening of clinical conditions, she was transferred to our paediatric rheumatic unit. She showed fever, with 2-3 spikes over the day > 39°C, associated with evanishing rash, and with spontaneous defervescence during the day. She showed arthralgia at the legs, back pain, with functional limitation, with a polyarticular localization, without swelling. ECG and echocardiography were normal in the first days of the disease. During the first weeks of the follow-up, she presented infero-lateral negative T wave. She showed enhanced troponin levels, and for the suspicion of myocarditis, CMR was performed. The delayed myocardial enhancement exhibited focal myocardial fibrosis.

**Results:** She was treated with steroids and after a gradual tapering, with canakinumab, a biological drug against IL-1 beta, at the dosage of 4 mg/Kg/every 4 weeks. Her condition improved dramatically, with a complete recovery of ventricular function. ECG documented the complete resolution of negative T wave and CMR showed the resolution of myocardial fibrosis. She is still followed in our unit. She reached complete remission, and canakinumab is now administered every 10 weeks.

**Conclusion:** Myocarditis is a well-documented complication of sJIA. This unusual case emphasizes the important role of canakinumab in the treatment of sJIA and the efficacy of a early treatment with biological drugs in these cases. Furthermore, we can speculate that the early treatment with canakinumab contributed to the prompt remission of the patient, to the complete recovery of the myocarditis and to the sJIA remission of the disease.

**Disclosure of Interest**: None declared

### P095. Fever with rash - the path leads us here

#### S. Maity

##### Department of Pediatrics, P D Hinduja National Hospital and Medical Rsearch Centre, Mumbai, India

###### **Correspondence:** S. Maity

**Introduction:** 8 year old male child, 1^st^ by birth order , born out of non-consanguineous union presented with complaints of high grade fever upto 102-103 degree F , 2-3 spikes/day , since last 2.5 months with intact activity in inter-febrile period. History of rash over face , usually at the time of fever and subsiding with fever . Weight loss 3-4kgs. No history of joint pain/ swelling/ redness.

**Objectives:** 1) To determine the etiology of the illness

2) To start necessary treatment /intervention at the earliest .

**Methods:** On examination , the child had Left upper cervical and bilateral inguinal lymph nodes palpable. No systemic localizing sign ,No hepatosplenomegaly.

**Laboratory Evaluation :**

CBC-Hemoglobin 11.3 gm%, Total Leucocyte count 27900, N92L6, PLATELET 2.66 LAKHS, PCV 32, ESR 37. Given antibiotics- azithromycin and amoxiclav-clavulinic acid , without resolution. Blood and urine cultures were negative ,sputum for Tuberculosis work-up was negative. CSF- Protein-112, sugar- 64, WBC- 10/hpf, CSF for TB work-up- Negative.

Shifted to tertiary care center .CT chest done, no significant abnormality found. Broncho alveolar lavage fluid sent for evaluation , negative for TB workup and malignant cells . 2D Echo showed no vegetations. ANA, ANCA, C3 -normal. Ferritin was high- 1354 (<140). Bone marrow study -normal. MRI brain with spine s/o no brain demyelination or meningeal enhancement , bilateral **Atlantoaxial joint effusion** was noted.

**Results:** In view of

1. Quotidian fever for more than 6 weeks associated with evanescent rash
2. lymphadenopathy and
3. **Radiological evidence** of joint involvement ,

Diagnosis of **SYSTEMIC JUVENILE IDIOPATHIC ARTHRITIS (SJIA)** was considered. Started on steroid and weekly methotrexate, responded well to the treatment.

**Conclusion:** High grade fever with evanescent rash for prolonged duration in an otherwise well child should raise the suspicion of **SJIA** and the child should be worked-up accordingly. The take home message is , subtle radiological evidences of joint involvement can help in the diagnosis of the same.

Statement : I tried to include the image in this submission but unable to do so, kindly note that i can communicate the necessary image via amail or any appropriate portal.

**Patient Consent:** Yes, I received consent

**Disclosure of Interest**: None declared

### P096. Atypical skin manifestations at onset of systemic juvenile idiopathic arthritis are associated with failure of first-line corticosteroid treatment and difficult-to-treat disease

#### L.-A. Eveillard^1^, P. Quartier^2^, N. Ouldali^3^, B. Badder-Meunier^2^, F. Aeschlimann^2^, C. Abasq-Thomas^4^, C. Ballot^5^, P. Bouric^6^, A. Desdoits^7^, C. Dumaine^3^, C. Galeotti^4^, V. Hentgen^8^, A. Lefèvre-Utile^9^, A. Chausset^10^, T. Hubiche^11^, I. Kupfer-Bessaguet^12^, S. Leclerq-Mercier^13^, S. Mallet^14^, I. Melki^3^, E. Merlin^15^, J. Miquel^16^, M. Piram^17^, D. Talmud^18^, N. Garcelon^19^, C. Vinit^3^, A. Welfringer^20^, E. Bourrat^21^, U. Meinzer^3^

##### ^1^Department of Infectious Disease and Internal Medicine, Reference centre for Rheumatic, AutoImmune and Systemic diseases in children (RAISE), Robert Debré University Hospital, Assistance Publique Hôpitaux de Paris, ^2^Pediatric Immunology-Hematology and Rheumatology Unit, Rare Disease Reference Centre (RAISE), IMAGINE Institute, Necker-Enfants Malades Hospital, Assistance Publique Hôpitaux de Paris, Université de Paris, ^3^General Paediatrics, Department of Infectious Disease and Internal Medicine, Reference centre for Rheumatic, AutoImmune and Systemic diseases in children (RAISE), Robert Debré University Hospital, Assistance Publique Hôpitaux de Paris, Université de Paris, Paris, ^4^Department of Pediatric Rheumatology Reference Centre for Autoinflammatory diseases and amyloidosis (CEREMAIA), Hôpital Bicêtre, Assistance Publique Hôpitaux de Paris, Le Kremlin-Bicêtre, ^5^Paediatric haematology, Jean-Minjoz Hospital, Besançon, ^6^Department of Biochemistry and Molecular Biology, CNRS, Recombinaisons genetiques, Rennes, Rennes, ^7^Department of pediatric surgery, AutoImmune and Systemic diseases in children (RAISE), Reference Centre for Autoinflammatory Disorders (CEREMAI), CHU de Caen, Caen, ^8^Reference Centre for Autoinflammatory Disorders (CEREMAI), Centre Hospitalier de Versailles, Le Chesnay Cedex, ^9^General Pediatrics and Pediatric Emergency Department, Jean Verdier Hospital, Université de Paris, INSERM, U976 HIPI Unit, Institut de Recherche Saint-Louis, F-75006,Assistance Puplique-Hôpitaux de Paris (APHP), Paris, ^10^Inserm CIC 1405, service de pédiatrie, CHU Estaing, Clermont-Ferrand, ^11^Department of Dermatology, Université Côte d’Azur, Nice, ^12^Department of Dermatology, Centre Hospitalier de Quimper, Quimper, ^13^Pathology Department, Necker-Enfants Malades Hospital, Assistance Publique-Hôpitaux de Paris, Paris, ^14^Department of Dermatology, Timone Hospital, Marseille, ^15^Department of Pediatrics, Clermont-Ferrand University Hospital, Clermont-Ferrand, France, ^16^Department of Pediatrics, CHU Reunion Saint Pierre, Saint-Pierre, Réunion, ^17^Pediatric dermatology unit, Department of Pediatrics, CHU Sainte Justine, CHU Sainte Justine Research Centre, University of Montreal, Montréal, Canada, ^18^Pediatric Department, American Memorial Hospital, CHU de Reims, Reims, ^19^Institut Imagine, Centre de Recherche des Cordeliers, UMR 1138, INSERM, ^20^Department of Dermatology, Reference Centre for Genodermatoses and Rare Skin Disease (MAGEC), Necker-Enfants Malades hospital, Assistance Publique Hôpitaux de Paris, Université de Paris, ^21^Department of Dermatology, Robert Debré University Hospital, Assistance Publique Hôpitaux de Paris, Université de Paris, Paris, France

###### **Correspondence:** L.-A. Eveillard

**Introduction:** The rash in systemic juvenile idiopathic arthritis (SJIA) is usually transient, but cases of atypical skin lesions because of their fixed nature have been reported.

**Objectives:** To describe the spectrum of typical and atypical skin lesions present at onset of SJIA in children and examine whether their presence may be an early clinical marker associated with difficult-to-treat patients.

**Methods:** A retrospective French multicenter study from 2002 to 2020, including pediatric patients with SJIA meeting the PRINTO classification criteria with documented skin lesion.

**Results:** 80/112 (71%) patients presented typical and 32/112 (29%) atypical skin lesions. Patients with atypical skin lesions had a higher CRP level (p=0.002) and higher ferritin (p=0.0015). The presence of atypical skin disease was associated with a risk of first-line treatment failure at 1 year (OR 10.6 CI[3.4; 46.8]), failure of glucocorticoids as first-line treatment at 1 year (OR 5.6 CI[2.2; 16.4]), need for biotherapy treatment at one year (OR 5.5 CI[1.9; 19.7]) and at two years (OR 8.2 CI[2.2; 53.3]).

**Conclusion:** Initial atypical skin lesions are associated with a poorer disease evolution, and may require the use of first-line biotherapies. Initial diagnostic evaluations for SJIA should include an examination by a dermatologist.

**Trial registration identifying number:** CNIL 1980120

**Patient Consent:** Yes, I received consent

**Disclosure of Interest**: None declared

### P097. Clinical features of systemic juvenile idiopathic arthritis associated with interstitial lung disease: single center’s experience

#### M. Kaleda, I. Nikishina, E. Nikolaeva, S. Salugina, E. Fedorov

##### Pediatrics, V.A. Nasonova Research Institute of Rheumatology, Moscow, Russian Federation

###### **Correspondence:** M. Kaleda, I. Nikishina

**Introduction:** It’s now well known, that systemic juvenile idiopathic arthritis (sJIA) may be associated with lung disease (LD), which albeit insufficiently defined and characterized.

**Objectives:** to characterize the clinical features all cases of LD associated with sJIA (sJIA-LD) based on long-term experience of the single center.

**Methods:** Retrospective study of all consequently pts with sJIA-LD in pediatric department of single center for the last 10 years.

**Results:** sJIA-LD is rare pulmonary complications which found in 5.0% of our cases of sJIA - 6 pts in total (4 were girls). The median (Me) age of sJIA at onset was 2.7 y.o. [IQR 1.8; 5.9]. The Me of the number of systemic manifestations at onset was 4.5 [3;6], 83.3% of pts had typical rash. The Me of the number of active joints at the time of sJIA verification was 8.0 [6; 18]. The Me of CRP level at onset was 72.5 mg/l [IQR 45.2; 112.4]), the Me of ferritin level - 892.5 ng/ml [IQR 349.0; 7256.0]). 5 pts had the history of macrophage activation syndrome (MAS) – 13.5% from all pts with MAS in our cohort of sJIA. All of them had MAS at onset, 4 – recurring MAS. There was a known family history of lung abnormalities in 1 patient – ​​2 of her older sisters died in the neonatal period from unidentified pulmonary pathology. 1 patient had the history of repeated episodes of eosinophilia (max 16%) and atypical urticarial rash that occur before LD detection. The therapy in all pts included steroids (100%), DMARDs (methotrexate (MTX) alone – 2 pts, MTX and cyclosporine consecutive – 4 pts), biologics (B) – all pts. The Me disease duration prior to start of treatment with B was 11.0 months [IQR 3.0; 18.0]. All pts were treated by tocilizumab (TCZ) as the 1^st^ line of B. 1 patient received TCZ only. 5 pts had anaphylactic reactions on TCZ so they have got from 2 to 5 B consecutively (in 3 pts TCZ switched to canakinumab (CAN), in 1 – to rituximab-anakinra-CAN, in 1 – to adalimumab-abatacept-CAN-sarilumab). The reasons for substitution therapy for the other B were loss of efficacy. The median of disease duration at the time of sJIA-LD verification was 4.0 years [IQR 2.8; 6.25]. At LD diagnosis, respiratory signs and symptoms were subtle in 4 pts, absent in 2 pts, although hypoxia was in 2 pts. The clinical signs of pulmonary hypertension were absent. All pts had bulbous deformity with erythematous clubbing of fingers. All pts had pulmonary involvement on chest CT scans (4 - septal thickening involving the periphery of multiple lobes, most marked in the lower lung zones, with ground-glass opacities, 1 – predominance of peribronchovascular consolidation, 1 - crazy-paving). 2 pts died, the duration of the disease at the time of death was 4.0 and 4.5 years. They had progressive LD with multiple organ failure, but without new episodes of MAS, 1 patient received TCZ, 1 - CAN. Other pts continued to receive B in stable mode with steroids in a low dose without evidence of LD progression and without of recurrent MAS during the last year of follow-up.

**Conclusion:** According to our data, patients with sJIA-LD mostly had an early onset of sJIA, the history of MAS, and an anaphylactic reactions on TCZ. The vast majority of pts had difficulties with the choice of therapy of sJIA. Usually lung involvement had hidden course for the years just with small subclinical signs. The establishment of predictors for early recognition of sJIA-LD, prevention strategies and special approaches to the choice of therapy are very important to ensure a better prognosis.

**Patient Consent:** No, I have not receive consent

**Disclosure of Interest**: None declared

### P098. Dynamics of vascular condition and endothely functions in patients with systemic form of juvenile idiopathic arthritis on the treatment

#### T. Marushko^1^, O. Onufreiv^1^, Y. Marushko^2^

##### ^1^Pediatrics-2, Shupyk National Healthcare University of Ukraine, ^2^Pediatrics of postgraduate education, Bogomolets National Medical University, Kyiv, Ukraine

###### **Correspondence:** O. Onufreiv

**Introduction:** The course of systemic juvenile idiopathic arthritis (JIA) is characterized by damage to small vessels. Diagnosis of vascular changes in people with risk factors for their early development, including the systemic form of JIA, in childhood in the subclinical stage is important for the prevention of cardiovascular disease in these patients in adulthood.

**Objectives:** To analyze the condition of blood vessels and endothelial function in patients with the systemic form of JIA on the background of basic therapy and treatment with immunobiological drugs.

**Methods:** We observed 24 patients with the systemic form of JIA, 15 received methotrexate, 9 patients were prescribed tocilizumab due to insufficient effectiveness of basic therapy. The mean patient age was 9.2±0.5 years. The average duration of the disease was 2.8±0.9 years. At the time of tocilizumab administration systemic glucocorticoid therapy (GC) (methylprednisolone) was administered for 15 (62.5%) over an average of 2.3 years, with a mean dose of 0.2 mg/kg for prednisolone. To assess the effect of immunobiological drugs on the condition of blood vessels, patients underwent duplex sonography to determine the thickness of the intima-media complex of the common carotid arteries (CCA), the IMT of the abdominal aortic, the stiffness index of CCA, differences in diastolic velocities and diameters of the brachial artery using the test for endothelium-dependent dilatation of the vessel and the index of left ventricular myocardial mass (LVM). The difference between the values of these indicators before investigation and after 6 months of therapy was also calculated.

**Results:** The inclusion of tocilizumab in the complex therapy of patients with systemic JIA, who had insufficient effect from treatment with methotrexate and GC, led to the elimination of systemic manifestations of the disease within six months, reducing the severity of joint syndrome, average number of joints with synovitis per patient and normalization of laboratory parameters. In patients with JIA who received tocilizumab in combination therapy, the magnitude of the change in the IMT of CCA, the IMT of the abdominal aortic, the stiffness index of CCA and the index of LVM mass were significantly smaller than in children receiving methotrexate. In the group of children receiving methotrexate, in all patients the value of these indicators increased over 6 months of follow-up, which indicated the progression of subclinical vascular damage despite a decrease in JIA activity. The mean differences in the difference in diastolic velocities and brachial artery diameter in children receiving tocilizumab before to immunobiological administration were significantly lower than those in the methotrexate group. After six months of therapy with immunobiological drug, the value of these indicators increased significantly compared to their initial values. These data indicate that before starting treatment with immunobiological drugs in patients with JIA there was endothelial dysfunction, which after 6 months of therapy was not detected again.

**Conclusion:** In children with JIA on immunobiological therapy, the rate of progression of subclinical vascular wall lesions and increase in LVM mass was significantly lower than in patients receiving basic treatment. In patients with systemic JIA on complex therapy with tocilizumab for six months the rate of increase in the IMT of CCA, the IMT of the abdominal aortic, the stiffness index of CCA and the index of LVM mass was significantly lower than in patients with JIA who received standard base therapy. There was observed normalization of endothelial function during 6 months of using tocilizumab.

**Patient Consent:** Yes, I received consent

**Disclosure of Interest**: None declared

### P099. Fever of unknown origin: an unexpected outcome

#### M. Rodriguez Ortiz^1^, S. Murias Loza^1^, L. Calle Miguel^1^, M. A. Alonso Álvarez^1^, J. Rodríguez Suárez^1^, E. Pardo Campo^2^, J. Zanabili Al-Sibai^3^, S. Rodríguez Ovalle^1^

##### ^1^Pediatrics Department, ^2^Rheumatology Department, ^3^Hematology Department, Hospital Universitario Central de Asturias, Oviedo, Spain

###### **Correspondence:** M. Rodriguez Ortiz

**Introduction:** Most of fever of unknown origin (FUO) cases are caused by infectious diseases, but autoinflammatory entities, such as Systemic onset Juvenile Idiopathic Arthritis (SoJIA) must be included in the differential diagnosis of FUO, since early therapeutic approach is key to improve prognosis.

**Objectives:** We herein present a 15-month-old male patient admitted for FUO. We describe the initial stages of the disease, and the unexpected outcome after diagnosis.

**Methods:** Medical chart was reviewed.

**Results:** A 15-month-old male patient presented with fever >39,5°C for 11 days; evanescent maculopapular erythematous rash which intensifies with fever; generalized adenopathies; carpus and right knee arthritis; increased acute phase reactants (APR), and anemia. Infectious and neoplastic aetiology were ruled out by complementary examinations (including PET-CT and bone marrow aspirate). SoJIA was then diagnosed, and corticosteroids were started on 19th day of fever with rapidly good response, which allowed hospital discharge with oral prednisolone (2 mg/kg/day dosage with subsequent tapering) and prophylactic trimethoprim-sulfamethoxazole. After 12 days, while receiving prednisolone 0.6 mg/kg/day, inflammatory symptoms reappeared. Given SoJIA relapse he was hospitalized and anti-IL1 biological agent (Anakinra) was started. However, no response was observed and the child showed clear clinical worsening which led to Anakinra withdrawal after 3 days. Analytical criteria for Macrophage Activation Syndrome (MAS) secondary to SoJIA were then met. Megaboluses of methylprednisolone and cyclosporine were started, followed by frank progressive clinical and analytical improvement. Unexpectedly, he developed severe neutropenia (up to 10 neutrophils/μL), being the only altered analytical parameter. Filgrastim was thus started but no increase of neutrophils count was reached after 9 days. Review of all medications was then performed, leading to trimethoprim-sulfamethoxazole discontinuation due to its known myelotoxicity. Three days later, laboratory examinations showed recovering of neutrophils count up to 13,000/μL, with good parallel outcome of MAS and SoJIA.

**Conclusion:** When suspecting SoJIA, early treatment must be started, and close clinical and analytical monitoring should be carried out to assess response and identify the possibility of MAS as a potentially serious complication. In the case of neutropenia with no clear origin after conventional studies, it is key to collect information on treatments, suspending those that are potentially myelotoxic. Trimethoprim-sulfamethoxazole is a widely used antibiotic as prophylactic agent, thus its potential adverse events should be always taken into account.

**Patient Consent:** Yes, I received consent

**Disclosure of Interest**: None declared

### P100. Establishing the CARRA Registry research network for systemic juvenile idiopathic arthritis-associated lung disease (CARE-NETS)

#### G. Schulert^1,2^, E. Verweyen^1^, S. Thornton^1,2^, C. S. Lages^1^, E. Eloseily^1^, M.-L. Chang^3^, M. E. Riordan^4^, A. Russell^5^, M. Natter^3^, Y. Kimura^4^ on behalf of CARRA Registry SJIA-LD Cohort Investigators

##### ^1^Cincinnati Children’s Hospital, ^2^University of Cincinnati College of Medicine, Cincinnati, ^3^Boston Children’s Hospital, Boston, ^4^Hackensack Meridian Health, Hackensack, ^5^Duke Clinical Research Institute, Durham, United States

###### **Correspondence:** G. Schulert

**Introduction:** Systemic juvenile idiopathic arthritis (SJIA) associated lung disease (SJIA-LD) is an emerging and life-threatening clinical problem, and currently affects as many as 1 in 20 SJIA patients. Despite recent advances, there remain key unanswered questions regarding disease prevalence, influence of biologic treatments, pathogenesis, and outcomes.

**Objectives:** The central objective of this project is to establish a cohort of patients with SJIA-LD within the CARRA Registry to accelerate clinical and translational research to understanding SJIA-LD.

**Methods:** Existing or newly enrolled CARRA Registry patients with SJIA and suspected, probable, or definite SJIA-LD were enrolled in the cohort. In addition to standard registry data, lung disease specific clinical data was obtained at baseline and at 6 month follow-up. In addition, CARRA standard biosamples were collected at baseline and follow-up. Multiparameter flow cytometry panel was developed and validated using peripheral blood mononuclear cells (PBMC) previously collected locally using CARRA standard biosamples kits and standard operative procedures (SOP). Flow cytometry data was collected using the Aurora spectral cytometer (Cytek) and analyzed using FlowJo (v10.7.1).

**Results:** As of May 1, 2022, 24 patients were enrolled in the SJIA-LD cohort from 12 CARRA Registry sites. Patients were 58% female and median age at enrollment was 4.5 (IQR 6.5). 55% of patients had definite (biopsy-proven) SJIA-LD, 36% probable SJIA-LD, and 9% suspected SJIA-LD. Median SJIA duration at time of lung disease diagnosis was 1.8 years. Most common clinical features were clubbing (60%), tachypnea (60%) and cough (40%). Most common radiographic findings were bronchial wall or peribronchovascular thickening (63%), ground-glass opacities (60%), and septal thickening 56%). Patients had a median Physician Global Assessment of Lung Disease (PGALD) of 3.5 (range 0-8/10) at time of cohort enrollment. Baseline biosamples have been obtained from 19 patients collected at 10 sites. To allow for immunophenotyping of PBMC from patients with SJIA-LD, we have developed a 27-color flow cytometry panel including lineage markers, surface activation markers, and intracellular cytokines. Validation experiments showed this panel could reliably identify monocyte (classical, non-classical, intermediate), dendritic cells, T cell (CD4 vs CD8, Th1/2/17, resting, memory, effector), B cells (naïve, regulatory, plasma), and NK cell subsets, as well as differences in expression of activation markers and cytokines in cellular subpopulations.

**Conclusion:** The CARRA SJIA-LD cohort is actively enrolling patients and collecting biosamples across the network. We have developed a multiparameter flow cytometry panel suitable for analysis of longitudinal biosamples. Ongoing goals include characterizing clinical features of the SJIA-LD cohort, disease progression over time, and defining cellular populations associated with disease trajectory. The SJIA-LD cohort will serve as an ongoing prospective cohort study for future clinical and translational research in this emerging clinical entity.

**Patient Consent:** Yes, I received consent

**Disclosure of Interest**: G. Schulert Consultant with: Novartis, E. Verweyen: None declared, S. Thornton: None declared, C. Lages: None declared, E. Eloseily: None declared, M.-L. Chang: None declared, M. E. Riordan: None declared, A. Russell: None declared, M. Natter: None declared, Y. Kimura: None declared

### P101. Patterns of disease course in systemic juvenile idiopathic arthritis – preliminary data from the CAPS cohort

#### P. Sinnappurajar^1^, S. Shoop-Worrall^2^, A. Ramanan^1^, K. Hyrich^2^, G. Cleary^3^, C. Ciurtin^4^, F. McErlane^2^, L. R. Wedderburn^4^, A. Chieng^5^

##### ^1^Paediatric Rheumatology, Bristol Royal Hospital for Children, Bristol, ^2^Arthritis Research UK Centre for Epidemiology, University of Manchester, Manchester, ^3^Paediatric Rheumatology, Alder Hey Children’s Hospital, Liverpool, ^4^Centre for Adolescent Rheumatology Versus Arthritis, UCL UCLH GOSH, London, ^5^Paediatric Rheumatology, Royal Manchester Children’s Hospital, Manchester, United Kingdom

###### **Correspondence:** P. Sinnappurajar

**Introduction:** Systemic juvenile idiopathic arthritis (sJIA) is thought to follow a monocyclic, polycyclic or persistent course. The reported proportions of children following each course varies in the literature, with the percentages ranging from 15% to 83% for a persistent course in different cohorts. This study examined the clinical course of children with sJIA in the Childhood Arthritis Prospective Study (CAPS), a UK multi-centre cohort.

**Objectives:** To determine the patterns of disease course in sJIA patients in the CAPS cohort.

**Methods:** Data from children with sJIA were extracted from the CAPS database, which captures clinical outcome data at presentation (baseline), 6 months, 1 year then annually to 5 years. Cases were included from the cohort inception in 2001 until 2016, ensuring all cases had opportunity for 5 years of follow up. Clinically inactive disease (CID) was defined at each time point using the 2004 Wallace criteria (no active joints, no fever/rash/serositis/splenomegaly/lymphadenopathy, no active uveitis, normal ESR/CRP, physician’s global assessment (PGA) indicating no disease activity), with modifications that reflected the core clinical outcomes seen in systemic JIA. Wallace criteria 1 excluded uveitis outcomes, whilst Wallace criteria 2 excluded both uveitis outcomes and PGA. CRP data was also excluded from both criteria as the results are not standardised between different laboratory assays. If CID status could not be generated due to missing data at individual time points, it was replaced using last-observation carried forward.

Children were classed as having a persistent course if they never attained CID by the above definitions, a monocyclic course if they attained CID then maintained it throughout the 5-year follow up period, and a polycyclic course if they attained CID and subsequently flared during the follow up. Children who only first attained CID at the last follow up visit were excluded, due to the inability to further define their clinical course.

**Results:** There were 96 cases of systemic JIA identified in the CAPS database between 2001 and 2016. 59% were female, and the mean age at onset was 7.5 years (SD 4.8).

Based on Wallace criteria 1 (excluding uveitis and CRP), two cases that first reached CID on the last visit (5^th^ year), were excluded. Of the remaining 94 patients, 82% (77/94) were defined as having a persistent course (never achieved CID), 12% (11/94) had a monocyclic course (reached CID and subsequently maintained remission throughout the follow up period) and the remaining 6% (6/94) had a polycyclic course (achieved CID and subsequently flared).

Using Wallace criteria 2 (excluding uveitis, CRP and PGA), three cases that attained CID on the last visit were excluded, leaving 93 patients for analysis. By this definition of CID, the proportions of children were similar across the 3 clinical patterns with 39% (36/93) having a persistent course, 34% (32/93) having a monocyclic course and 27% (25/93) exhibiting a polycyclic course.

**Conclusion:** Following this preliminary analysis of the CAPS database, we found that similar percentages of children with sJIA will follow either monocyclic, polycyclic or persistent disease patterns over a 5-year time period, which is broadly in keeping with the literature. A higher percentage of children attain CID when PGA is excluded. This cohort will be further examined to identify whether the proportions following the three disease patterns have changed over time, and whether any variables at disease onset correlate to the disease patterns, which could ultimately help guide early treatment decisions.

**Patient Consent:** Yes, I received consent

**Disclosure of Interest**: None declared

## Poster session: Spondyloarthritis (SpA) and enthesitis related arthritis (ERA)

### P102. Childhood-onset sacroiliitis: rheumatic causes and the correlation between clinical findings and magnetic resonance imaging

#### P. O. Avar-Aydin^1^, Z. B. Ozcakar^1^, S. Kaynak-Sahap^2^, F. Aydin^1^, C. Arslanoglu^1^, N. Cakar^1^, O. S. Fitoz^2^, F. Yalcinkaya^1^

##### ^1^Department of Pediatric Rheumatology, ^2^Department of Pediatric Radiology, Ankara University Faculty of Medicine, Ankara, Turkey

###### **Correspondence:** P. O. Avar-Aydin

**Introduction:** Childhood-onset sacroiliitis is a challenging condition that requires to consider several causes in the differential diagnosis including infections, malignancies, medications, and rheumatic diseases.

**Objectives:** To evaluate the etiology and the features of MRI-proven sacroiliitis in pediatric patients with rheumatic diseases in a referral center from an Eastern Mediterranean country.

**Methods:** Demographic and clinical data were extracted from the electronic medical records of the patients with sacroiliitis followed in the last 5 years. Active inflammatory changes of the sacroiliac joint (SIJ) were scored by the inflammation score according to the degree, intensity, and depth of the bone marrow edema (BME) and the presence of enthesitis, capsulitis, and structural damage lesions was evaluated by the modified SPARCC scoring system.

**Results:** A total of 46 patients were found to have MRI-proven sacroiliitis with a mean follow-up of 3.13±2.15 years. There were three groups of patients diagnosed with sacroiliitis when classified according to etiology: JIA (n:17), FMF (n:14), and CNO (n:8). Seven patients, FMF and JIA (n:6) and FMF and CNO (n:1) had a co-diagnosis that might cause sacroiliitis and they were excluded from the group analyses. Thirty patients (65.2%) were male. The mean age at disease onset was 10.97±3.93 years and six patients diagnosed with FMF or CNO were younger than 6 years. The prevalence of sacroiliitis in the relevant cohorts was 11.9% for JIA, 3.8% for FMF, and 34.6% for CNO groups. On the MRI of the SIJ, capsulitis was present in 17 patients (37.0%) and enthesitis in 14 patients (30.4%) in addition to BME with a mean total inflammation score of 22.50±13.80. Besides, structural damage lesions were present in 73.9% of the patients at the onset of sacroiliitis. Nonsteroidal anti-inflammatory drugs were used in all patients as the first-line treatment. Disease-modifying anti-inflammatory drugs were given to 67.4% and TNFi to 54.3% of the patients.

In the comparative analyses of three groups of patients, the weight and height percentiles were significantly lower in FMF group and HLA-B27 was more frequent in JIA group. Other findings of demographics, family history, differential blood counts, acute phase reactants, and the usage of TNFi were similar. BME intensity score on MRI and the frequency of capsulitis and enthesitis were significantly higher in CNO group. There was a significantly strong or moderate correlation between total inflammation score and inflammatory back pain, JSpADA score, ISSF score, clinical CNO score, and CRP; BME intensity score and inflammatory back pain, JSpADA score, and CRP; BME depth score and ISSF score, clinical CNO score, CRP, and ESR; and the presence of sclerosis/erosion and clinical CNO score.

**Conclusion:** This is the first study to evaluate the etiology of sacroiliitis in children with various pediatric rheumatic diseases and the relationship between clinical and MRI findings of newly-diagnosed sacroiliitis. We demonstrated that JIA, FMF, and CNO were the causes of childhood-onset sacroiliitis in our region. Characteristic findings on MRI might help the differential diagnosis of patients with sacroiliitis. Moreover, inflammatory and structural damage lesions found on MRI correlated with several clinical and laboratory features.

**Patient Consent:** Yes, I received consent

**Disclosure of Interest**: None declared

### P103. Inflammatory marker profiles in pediatric familial Mediterranean fever and accompanying spondyloarthritis: siblings or distant cousins?

#### S. Bocutcu Cetin^1^, E. Sag^2^, K. Cankirili^3^, M. Kasap Cuceoglu^1^, E. D. Batu^1^, O. Basaran^1^, Y. Bilginer^1^, S. Ozen^1^

##### ^1^Pediatric Rheumatology, Hacettepe University, ^2^Pediatric Rheumatology, Ankara Training and Research Hospital , ^3^Translational Medicine Laboratory, Hacettepe University, Ankara, Turkey

###### **Correspondence:** S. Bocutcu Cetin

**Introduction:** Familial Mediterranean fever (FMF) is a hereditary disease characterized by recurring, self-limited inflammatory episodes. The disease is caused by a gain-of-function mutation in MEFV (Mediterranean FeVer) gene, causing an excessive activation of pyrin inflammasome and thus overproduction of proinflammatory cytokines.^1^ Spondyloarthropathies accompanying FMF include inflammatory arthritides, sacroiliitis, spondylitis, enthesitis, and extraskeletal manifestations, and in children, enthesitis-related arthritis (ERA).^2^ Although the evidence supporting the role of innate immunity is overwhelming, the exact pathogenesis of the aforementioned diseases is yet to be determined.

**Objectives:** In this study, chosen inflammatory marker levels are evaluated in order to gain further knowledge on the pathogenetic mechanisms of FMF and accompanying spondyloarthritis (SpA).

**Methods:** Between December 2020 and June 2021, we collected four groups of patients with FMF-SpA with high disease activity, ERA with high disease activity, FMF during the attack period, and FMF remission period. Having collected 20 samples from 16 patients, the inflammatory marker levels of these groups were compared with each other and the healthy donors.

**Results:** TNF-α levels are significantly increased in all the patient groups compared to the healthy controls. No statistically significant difference in IL-1β levels was found among the patient groups or the healthy controls. However, transcriptomic studies found a significant difference between the patient groups and the healthy controls. IL-6 levels showed a significant decrement in remission period in FMF patients compared to the attack period. IL-18 levels were significantly increased in the FMF-SpA group, suggesting the possibility of a potential inflammatory biomarker for ERA/spondyloarthritides in the FMF group.

**Conclusion:** This study is the first to investigate the inflammatory pathogenesis of FMF and accompanying spondyloarthropathy. Corroboratory studies are needed to further evaluate the role of these cytokines and their incorporation into personalized medicine.

**References**

1. Chae JJ, Cho Y-H, Lee G-S, Cheng J, Liu PP, Feigenbaum L, et al. Gain-of-function Pyrin mutations induce NLRP3 protein-independent interleukin-1β activation and severe autoinflammation in mice. Immunity. 2011;34(5):755-68.

2. Sönmez, H. E., Batu, E. D., Demir, S., Bilginer, Y., & Özen, S. (2017). Comparison of patients with familial Mediterranean fever accompanied with sacroiliitis and patients with juvenile spondyloarthropathy. Clinical and experimental rheumatology, 35(6), 124-127.

**Patient Consent:** Yes, I received consent

**Disclosure of Interest**: None declared

### P105. MRI evaluation of active sacroiliitis in juvenile spondyloarthritis treated with TNF inhibitors

#### K. Yamazaki^1^, A. Fujikawa^2^, T. Nozaki^3^, Y. Motonaga^4^, M. Mori^4^

##### ^1^Division of Rheumatology and Allergology, Department of Internal Medicine, ^2^Department of Radiology, St. Marianna University School of Medicine, Kawasaki, ^3^Department of Radiology, St. Luke’s International Hospital, Tokyo, ^4^St. Marianna University School of Medicine, Division of Rheumatology and Allergology, Kawasaki, Japan

###### **Correspondence:** K. Yamazaki

**Introduction:** Even though magnetic resonance imaging (MRI) assessment of the sacroiliac joints (SJ) is useful in the early diagnosis of spondyloarthritis (SpA), the evaluation of MRI features in juvenile SpA has not been well established.

**Objectives:** We aimed to evaluate JSpA-specific MRI features of SJ for use in the early diagnosis of sacroiliitis.

**Methods:** We collected seven cases clinically diagnosed with JSpA and successfully treated with TNF inhibitors. Two pediatric musculoskeletal radiologists retrospectively evaluated the MRI features of sacroiliac joints in the patients before and after the treatment.

**Results:** Seven JSpA patients (5 males, 2 females; Median age 14 (12 to 20)) are included in this study. The median time from onset to diagnosis is three years. Before TNF-inhibitors treatment, inflammatory back pain, SJ tenderness, and peripheral enthesitis were present in all patients. One of the patients has HLA-B27. MRI features at diagnosis showed active inflammatory lesions of sacroiliitis such as bone mallow edema (3/7), capsulitis (0), enthesitis outside of sacroiliac joint (1/7), and structural lesions such as erosion (3/7), sclerosis (3/7), backfill (0), fat metaplasia (0) and ankylosis (0). The clinical severity of inflammatory back pain and peripheral enthesitis did not correlate with the MRI findings. MRI findings of bone marrow edema improved in all three patients 6-12 months after initiation of TNF inhibitors treatment.

**Conclusion:** The assessment of SpA International Society (ASAS) definition for adult has previously been reported to be less sensitive in children. In the early stages of sacroiliitis, inflammatory changes in the SJ are so subtle that they may not be detected on MRI. Since the early diagnosis of active sacroiliitis is important in terms of treatment options, a practical definition of active sacroiliitis specific to children and adolescents is required for pediatric rheumatologists.

**Disclosure of Interest**: None declared

## Poster session: Autoinflammatory diseases

### P106. First case with a possible diagnosis of haploinsufficiency of A20 in Libya

#### A. A. Abushhaiwia^1^, Y. Elfawires^2^

##### ^1^Paediatric Rheumatology , Faculty of Medicine,Tripoli university, Tripoli Children’s Hospital , ^2^Paediatric Rheumatology , Faculty of Medicine,Tripoli university, Tripoli Children’s Hospital , Tripoli, Libya

###### **Correspondence:** Y. Elfawires

**Introduction:** Monogenic autoinflammatory disorders (AID) and primary immunodeficiencies can present early in life with features that may be mistaken for Behçet’s disease (BD). These disorders involve a growing multisystem inflammatory diseases associated with defects in the innate immune system. The symptoms appears to range through unprovoked inflammation (fever, rash, ulcers, and elevation of acute phase reactants ).We herein report a case under workup for Haploinsufficiency of A20 in a young Libyan female that is suffering from recurrent mouth and genital ulcers.

**Objectives:** to highlight the importance of considering the monogenic AID in children presenting with signs and symptoms mimicking BD

**Methods:** a case report

**Results:** A 7 yrs old Libyan female born to consanguineous parents, presented to our clinic as a referral from the GI clinic,with the complaint of having recurrent mouth and perianal ulcerations. The problem started at age of five, six months before presentation. With the appearance of the ulcerations about three/month, lasting for about a week and resolved spontaneously with no recognized trigger, the ulcers then started to involve other areas including the genitalia, behind ears, around umbilicus with discharging fluid/pus. Systemic review showed that these ulcers are not associated with fever, good general wellbeing, good appetite, no significant wt loss ( although her wt fall below 3rd centhile for age and sex) , arthralgia ( both knees/ no arthritis PGALS was normal), skin rash follicular/postular involving pressure areas initially then progressed to involve the face, no nail nor hair changes, she had H/O recurrent URTI and skin infections that were managed with local Rx as they appear, also H/O constipation initially that then progressed to altered bowel habit. F/H her grandmother know case of IDDM & RA. The girl was approached with the DD of BD, IBD, SLE, FMF, hyper IgD and Haploinsufficiency of A20. Her blood tests showed: CBC WBC 10.2 ( 5.11 neutro, 4.39 lympho, 0.58 mono, EO 0.10), Hgb 11.9 g/dl, PLT 477+. ESR 107, 70, 78 ( always high). CRP never been +ve, LDH 369, Ferretine 16.37 ( low ), urine for Pr:Cr ratio N, ALT 10, AST 23, Bil 0.3, serology : viral screen negative, Immunoglobuline assay IgA, IgE, IgD, IgG, IgM all within normal levels, ENA screen 7.3 ( > 1.2 +ve), ANA, ds DNA, p-ANCA and c-ANCA all negative, EBV serology negative, TTG negative. Vitamin B12 normal level, Zinc serum level normal, S Amyloid normal. Molecular genetic analysis of the MEFV and MVK gene came with no significant pathologic variant detected, HLA B51 positive. She had an upper and lower GI endoscopy done for her with an ilial biopsy taken all came normal with no abnormality. U/S abdomen also normal. Ophthalmological examination Normal no uveitis. Furthermore complement level ( C1-C9 &C50 levels), T cell function assessment ( CD4,CD8 level & ratio, CD 18, CD 45 & CD56 ), immunoglobuline response for vaccination ( anti tetanus and diphtheria IgG levels) neutrophils function ( DHR flow cytometry ), genetic testing ( TNF AIP3 gene mutation +/- WES), biopsy from the ulcers for histopathology but unfortunately due to lack of resources and lack of financial support these test are not done yet.she is on colchicine tab 1mg once daily, local steroids for mouth and genital ulcers and systemic steroids has been used for short courses with no much improvement, Azathioprine has been added waiting for the response, she is also on oral hygiene care and septrine as prophylactic Abx.

**Conclusion:** Due to the wide variety of disorders that present with symptoms that mimic BD, especially when presenting early in life, consideration of monogenic AID and immune deficiencies with early implementation of gene testing is crucial to shorten the duration of patients suffering and to come up with appropriate management plan.

**Patient Consent:** Yes, I received consent

**Disclosure of Interest**: None declared

### P107. Successful treatment with infliximab in a child with deficiency of interleukin-36 receptor antagonist (DITRA)

#### A. A. Abushhaiwia^1^, Y. Elfawires^2^

##### ^1^Paediatric Rheumatology , Faculty of Medicine,Tripoli university, Tripoli chlidren’s hospital , ^2^Paediatric Rheumatology, Faculty of Medicine, Tripoli university, Tripoli Children’s hospital , Tripoli , Libya

###### **Correspondence:** A. A. Abushhaiwia

**Introduction:** General pustular psoriasis (GPP) is a rare form of psoriasis and is clinically characterized by widespread eruptions of sterile pustules and bright erythematous skin accompanied by periods of fever, chills, neutrophilia, and elevated serum C-reactive protein. In 2011, Marrakchi et al3 reported a subgroup of GPP patients with a specific genetic defect: a deficiency of interleukin-36 receptor antagonist (DITRA). We report for the first time DITRA in a Libyan child successfully treated with infliximab.

**Objectives:** To report the efficacy of treatment with 5 mg/kg infliximab intravenous infusion at Weeks 0, 2, and 6, followed by a monthly regimen, was effective and in inducing complete or almost complete responses in a child with

**Methods:** case report is described

**Results:** A 4 year-old Arab Libyan boy, born from non-consanguineous parents came to our attention due to a suspicion of a systemic autoinflammatory condition. Of note, the index patient had two healthy brothers and an uncle diagnosed with psoriatic arthritis. At the age of 2 years recurrent and severe episodes of generalized pustular psoriases requiring many hospital admissions started. Initially, he was managed as of pustular psoriasis after a 6-month history of appearance. Diffuse pustules involving cheeks, perioral area associated with mouth ulcers, characterized skin lesions. A rapidly progression of his condition could be observed characterized by worsening of general condition and generalization of the rash over different body parts. Other clinical pictures frequently observed was fever; psoriatic nail changes (e.g. Pitting and onychomadesis) and ichthyosis altogether with joint involvement limiting his activity. Rash became severe with discharging pustules and failure to thrive could be observed. At his admission, widespread presence of scaly erythematous plaques with pinhead pustules involving the scalp, face, extremities, torso, and buttocks. The palms and soles were also affected. Nail pitting and onychomadesis. At his admission a complete blood count demonstrated anaemia HGB 8.1 g\dl and leukocytosis. However, during febrile episodes the patient’s white blood cell count increased to 17,000/ll with neutrophilia of 92%, ESR 50mm\h, and elevated CRP 20mg\dl level. No other condition, such as infection, could be observed over the time. A skin biopsy demonstrated parakeratosis, spongiosis, and psoriasiform acanthosis; we could also observe an elevated level of vitamin B12 757.90 pg\ml (reference range). Due to a suspicion of a systemic autoinflammatory condition, a genetic sequencing was requested. A homozygous already described mutation in the IL36RN was found (c.80T>C;. pLeu27Pro), thus confirming the diagnosis of DITRA. Once neither anti-IL1 (anakinra) neither anti-Il12/23 (secukinumab) is available in Libya , high doses of systemic steroids was initiated and could achieve clinical control. In order to avoid prolonged use of steroids, the patient’s dose was gradually tapered. However, cutaneous exacerbation could be observed and a steroid spared agent, methotrexate, at a dose of 7.5 mg once per week was therefore added. Under this treatment no improvement was seen .Treating him by anti-TNF (inflixmab) 5mg\kg IV, 2 weeks after starting infliximab IV reveals near complete resolution of psoriatic skin lesions. He is in total remission, “completely cleared skin”. Since October 2021

**Conclusion:** DITRA is a rare disease. We demonstrate in this a child with DITRA that TNF-alpha inhibition with infliximab dramatically improved the dermal changes and could normalize the skin within 2 weeks and his quality of life clearly improved after the use of infliximab treatment.

**Patient Consent:** Yes, I received consent

**Disclosure of Interest**: None declared

### P108. A two-year-old libyan boy with recurrent abdominal pain and fever: familial mediterranean fever

#### A. A. Abushhaiwia^1^, Y. Elfawires^1^, A. Ateeq^2^, S. Elazragh ^3^

##### ^1^Paediatric Rheumatology , Faculty of Medicine,Tripoli university, Tripoli Children’s Hospital , ^2^Paediatric Rheumatology , ^3^General Paediatrics , Tripoli Children’s Hospital , Tripoli , Libya

###### **Correspondence:** A. A. Abushhaiwia

**Introduction:** Familial Mediterranean fever (FMF) is the most common monogenic autoinflammatory disease, characterized by recurrent self-limited attacks of fever accompanied with peritonitis, pleuritis, or arthritis. Inherited in an autosomal recessive fashion. Occurs as a result of pathogenic variants in Mediterranean fever (MEFV) gene. Rarity of these diseases exposure during medical practice and lack of genetic testing for confirmation makes diagnosis challenging in Libya and in other middle-income countries

**Objectives:** To report for the first time the clinical and genetic findings of FMF in a two-year -old boy from Libya.

**Methods:** is a target gene panel commercially available (ISU university Germany) was requested. The results: clinical presentation was and we found a heterozygous mutation on MEFV gene (c.2177T>C p.Val726Ala

**Results:** A 2 year-old Libyan boy from the North Africa, born of non-consanguineous healthy parents, came to our attention because of recurrent episodes of fever since at age 4 months. That’s required him many hospitalizations and several and no specific diagnosis being made and could be achieved, recurrent attacks of fever that reached as high as 39c daily, lasting from 5-8 days, every 2-8 weeks sometimes accompanied by abdominal pain, vomiting, joint pain and diarrhea. The initial attacks were occurring as frequently as every 3 weeks. He had neither history of previous surgeries except he had undescended testes on May 2020, ultrasound scrotum&inguinal regions that showed retractile right testicle and left testicle seen within medial third of left inguinal canal and LH, testosterone both were within normal range, nor a family history of same illness or immunodeficiency. His physical examinations were unremarkable only he is under weight 10.5kg (failure to thrive). Laboratory tests during attacks revealed mild anaemia. (Haemoglobin as low as 9 -10.5g/l) during the attacks with platelets were within normal limits, white blood cells count were as high as 24,400.Remarkable leukocytosis (15,000 mm3-24, 000 mm3) mainly neutrophil 66.3%, blood film revealed mild microcytic hypochromic anemia, always with fluctuant levels of acute reactant C-reactive protein (values between 50 and 283mg/L) markers as erythrocyte sedimentation rate (ranged from 40 to 80 mm/h), all cultures included blood, urine and CSF were negative, vitamine B12 was elevated 1230pg\ml ,immune profile tests all of them were within normal range included immunoglobulin assay was normal .Evaluation of lymphocyte immunophenotyping test ( quantitation of total B cell numbers (CD19) and total T cell numbers (CD3), and quantitation of T cell subsets (CD4, CD8) and natural killer (NK) cells (CD16), total CH50,C3,C4 all were within normal range, serum amyloid was normal <3mg\dl , With normal laboratory work-up during wellbeing was a constant finding. Chest radiography, echocardiography and abdominal ultrasound were always normal. Based on his clinical background, physical examinations and his above some lab tests he is most likely the diagnosis of FMF or MVK. Molecular analysis for FMF (MEFV) revealed heterozygous c.2177T> p. V726A. Therefore the clinical and diagnosis of FMF was established for the child. The patient responded well to colchicine therapy started at a dose of 0.5 mg/day

**Conclusion:** FMF is frequent in many Mediterranean countries, Libya is one of the Mediterranean countries but rarely reported in Libya or delay in diagnosis due to we don’t have those particular tests in public hospitals, it is one of the greatest challenges that these diseases require so many investigations that people can’t afford it. Furthermore; the prevalence of hereditary autoinflammatory diseases particular FMF remains unknown in Libya.

**Patient Consent:** Yes, I received consent

**Disclosure of Interest**: None declared

### P109. Plasma MIR-520B overexpression in patients with rheumatoid arthritis

#### Y. Achour, S. Chaabane, L. Keskes

##### Laboratory of Human Molecular Genetics, Faculty of Medecine of Sfax, Sfax, Tunisia

###### **Correspondence:** Y. Achour

**Introduction:** Rheumatoid arthritis (RA) is a chronic inflammatory autoimmune disorder, affecting 0.5 to 1% of the population worldwide. It is characterized by unknown etiology in which genetics, environmental, and epigenetics aspects are considered as the main risk factors that contribute to the development of this chronic disease. MicroRNAs are endogenous small noncoding sequences of single-stranded RNA, which cause alteration of gene expression by post-transcriptional modifications, destabilize target mRNAs to inhibit protein synthesis and regulate crucial pathways and cellular processes, such as cell growth, differentiation, proliferation, and cell death. Some microRNAs have been associated with a higher risk and progressionto RA, as well as with clinical variables of RA (tender joint and disease activity score (DAS28) -erythrocyte sedimentation rate (ESR)). Moreover, miRs such miR-520b is recognized as a critical regulator by suppressing endothelial cell inflammation and might serve as a potential therapeutic target for atherosclerosis.

**Objectives:** The current study aimed to investigate the expression levels of circulating plasma miR-520b in Tunisian RA patients and to determine possible correlations between expression levels and clinical and immunological features.

**Methods:** Total RNA was isolated from plasma of 40 RA patients and 10 age-gender-matched healthy controls. Expression of miR-520b was measured using quantitative real-time PCR methods by TaqMan qRT-PCR. Correlation analyses was performed using Mann-Whitney U test and Spearman’s coefficient test.

**Results:** The results shows that the expression level of miR-520b was considerably increased in RA patients compared to the control group (p-value = 0.0005). A positive correlation was observed with high activity disease (DAS28 > 5,1) (p-value = 0.008) and overall C-reactive Protein (p-value = 0.031). Moreover, a significant association of the expression of miR-520b with high CRP levels ( CRP > 10mg/L) was revealed. There was no significant correlation of miR-520b overexpression and seropositivity to anti-CCP and RF patients.

**Conclusion:** To the best of our knowledge, this is the first report of miR-520b expression in RA. Our study demonstrates that plasma miR-520b is associated with the pathogenesis of RA and it may act as a novel diagnostic marker for disease.

**Disclosure of Interest**: None declared

### P110. DADA2 in Malaysian children - novel mutations and “heterozygous presentation”

#### S. A. Affendi^1^, S. P. Tang^1^, S. C. Lim^2^, B. S. Koay^1^, S. K. Ng^1^, P. Nadarajah^1^, S. Y. Lee^1^, L. R. Sangaran^1^

##### ^1^Paediatric Rheumatology, Selayang Hospital, ^2^Paediatric Rheumatology, MARA University of Technology Malaysia (UITM), Selangor, Malaysia

###### **Correspondence:** S. A. Affendi

**Introduction:** Deficiency of adenosine deaminase 2 is a recently described monogenic autoinflammatory condition characterised by multi-system vasculopathy that often presents in early childhood. It has a wide heterogenous presentation which poses a diagnostic challenge to treating clinician. The aim of this case series is to highlight novel mutation associated with DADA2, the clinical spectrum and treatment outcome at our centre.

**Objectives:** We describe here a case series of children with proven DADA2.

**Methods:** Five children with DADA2 presenting with various forms of vasculopathy and immunodeficiency were identified in a single cetre tertiary hospital over 6 years follow-up duration. Their clinical data and treatment outcomes were analysed.

**Results:** this case series we share our experience in managing children with DADA2 in the only paediatric rheumatology centre in Malaysia. 5 DADA2 patients were reported in 6 years follow-up duration period since 2016. Their age range from 2 months to 9 years old. There are 3 males and 2 females (with a pair of siblings) within this case series and all of them are of Malay ethnicity. 3 children were diagnosed within less than a year, whilst 2 other children took more than a year highlighting the diagnostic challenge posed by this condition. Neurological symptoms namely stroke was the dominant clinical picture (n=4) and 1 patient presented with right retinal artery occlusive vasculitis at 2 months of age. 2 children had skin manifestations (1 with livedo reticularis whilst 1 had morphea-like skin condition). Recurrent fever (n=3) and orchitis (n=1) with abdominal pain (n=1) are other clinical symptoms that were reported. 1 child went on to developed low IgM with recurrent CMV infection. Of note, haematological involvement were not observed in our case series The diagnosis of DADA2 were made via genetic panel for autoinflammatory (Invitae panel) . Via this genetic panel, the pair of siblings showed homozygous novel mutation in ADA2 gene leading to their clinical symptoms. The novel mutation c.369G>T (p.Trp123Cys) has not been reported in the literature, highlighting the potential genomic heterogeneity of DADA2 . All of the children in this case series has ADA2 plasma level activity of 0 mU/g protein and 1 child had level of 5.6 mU/g protein. All 4 children were treated with anti-TNF except for 1 child who is on MMF whilst awaiting for funding approval for anti-TNF. 3 of these children have shown good inflammatory control with anti-TNF recovery whilst 1 patient has non-sustainable respond to anti-TNF. 4 of these children has had significant morbidities due to uncontrolled inflammation, steroid toxicity and sequelae from the recurrent stroke.

**Conclusion:** The spectrum of clinical presentation in DADA2 continues to expand in tandem with discoveries of novel mutation in ADA2 gene. More studies are needed in the future to highlight genotype-phenotype correlation and this will perhaps help to tailor a more target therapy for these children, High index of suspicion amongst clinician is vital in order to clinch the diagnosis and to prevent accrued organ damage.

**Patient Consent:** Yes, I received consent

**Disclosure of Interest**: None declared

### P111. Chronic non-bacterial osteomyelitis in children: a multi-center cohort from Saudi Arabia

#### L. Alahmadi^1,2,3^, J. Almasoud^1,2,3^, W. Alsuwairi^1,2,3^, A. Alrasheed^1,2,3^, S. Al-Mayouf^4^, F. Alkhars^4^, A. Asiri^5^, D. Alwakeel^5^, A. Alenazi^6^, S. Alenazi^6^, R. Bakry^7^, K. Alsufyani^8^, O. Aldibasi^2^, J. Alqanatish^1,2,3^

##### ^1^Pediatric Rheumatology, King Saud bin Abdulaziz University for Health Sciences, ^2^King Abdullah International Medical Research Center (KAIMRC), ^3^King Abdullah Specialist Children’s Hospital,National Guard Health Affairs, ^4^Pediatric Rheumatology, King Faisal Specialist Hospital and Research Center, ^5^Pediatric Rheumatology, Prince Sultan Military Medical City, ^6^Pediatric Rheumatology, King Fahad Medical City, Riyadh, ^7^Pediatric Rheumatology, East Jeddah Hospital, Jeddah, ^8^Pediatric Rheumatology, Maternity and Children’s Hospital, Mecca, Saudi Arabia

###### **Correspondence:** L. Alahmadi

**Introduction:** Chronic non-bacterial osteomyelitis (CNO) is an auto-inflammatory disorder that mainly affects the bone and occurs in children and adolescents. It is a chronic condition with a spectrum of presentation ranging from a mild self-limiting mono-focal disease to a sever chronic recurrent multifocal or so called Chronic Recurrent Multifocal Osteomyelitis (CRMO).

**Objectives:** The objective of the study was to evaluate demographic, clinical, laboratory, imaging,histopathology characteristics, and treatment responses of pediatric CNO patients seen in Saudi children.

**Methods:** The clinical records of 58 patients diagnosed with CNO between 2015 and 2021 at 6 centers in 3 major cities in Saudi Arabia were reviewed.

**Results:** We reviewed a total of 58 children (n=36, 62% females), with median age of 8 years at diagnosis of CNO. The most common clinical manifestation was bone pain (n=51, 88%) followed by bone swelling (n=32, 55%). Fever was the most frequent systemic manifestation (n=8, 13%), followed by skin rash (n=6, 10%) and GI symptoms (n=5, 9%). Eighty five percent (n=49) of patients had elevated ESR while CRP was positive in (n=24, 41%). X-ray was the most frequent imaging modality (n=49, 85%) followed by Regional-MRI (n=41, 71%), bone scan (n=33, 57%), CT-scan (n=24, 41%), whilst a whole body MRI was the least used modality. The most common sites of bony lesions were lower limb (n=37, 64%), and bone biopsy was done in 34/58 (59%) patients. The pelvis and clavicle were involved in 18/58 (31%) in our cohort. NSAIDs were the most used medications (n=51, 86%), followed by Bisphosphonate and Anti-TNF in 55% (n=32), and Methotrexate and systemic steroids in 36% (n=21) of patients. Clinical remission was achieved in 44/58 (83%) of CNO patients.

**Conclusion:** This study describes the phenotype of a large-scale sample of CNO in our pediatric population and the practice of our pediatric rheumatologist in treating these patients. It is a step forward towards developing a local consensus in treating this rare entity in our Saudi patients.

**Patient Consent:** Not applicable (there are no patient data)

**Disclosure of Interest**: None declared

### P112. Colchicine response in TRAPS – a molecular enigma

#### S. Balan, S. Raj, M. CB

##### Rheumatology & Clinical Immunology, Amrita Vishwavidyapeetham, Kochi, India

###### **Correspondence:** S. Balan

**Introduction:** TNF receptor associated Periodic Fever Syndrome (TRAPS) is an autosomal dominant, monogenic, autoinflammatory disease, characterized by mutations in TNFRSF1A gene coding for TNFR1 , which results in aberrant downstream signalling, resulting in widespread systemic inflammation and increased risk of reactive amyloidosis. Lack of response to colchicine is one of the defining features of TRAPS.

**Objectives:** To present a child and a family with a TRAPS mutation who are responsive to Colchicine, an unusual observation in patients with this illness

**Methods:** We present here a case of 9 year old female child, who presented with recurrent episodes of fever lasting for 7 to 12 days, associated with abdominal pain, maculo-papular rash, arthralgia, conjunctivitis every 3-4 months once since the age of 9 months, with strong family history. She was noted to have neutrophilic leucocytosis, grossly elevated inflammatory markers during febrile episodes. She was previously treated elsewhere with NSAIDs and glucocorticoids during flares with some response. As genetic testing was not a viable option when she first presented ,she was subsequently started on colchicine at a dose of 1mg/day with excellent clinical response. Genetic testing finally performed 4 years after the initial presentation ,revealed pathogenic TNFRSF1A variant. Her aunt ,also in the pediatric age group as well as her father, uncle and grandfather have the same mutation and all of them are currently responsive to Colchicine. She continues to be under our follow up since 3 years and is doing well on colchicine

**Results:** Following commencement of Colchicine, she went down from 3-4 episodes/year to none and from 40-60 days of symptomatology a year to about 6-8 days of minor symptoms/year

Inflammatory markers have stayed in the normal range.

Her 17 year old aunt, father and great uncle with the same gene have shown similar clincial response with Colchicine

The mutation this family carries-TNFRS1A Exon3 c.236C>T, Heterozygous and pathogenic .

**Conclusion:** In resource limited settings, Colchicine can be considered as a therapeutic option in patients with TRAPS in those patients who show good clincial response

**Trial registration identifying number:** none

**Patient Consent:** Yes, I received consent

**Disclosure of Interest**: None declared

### P113. DADA2 with 2 different phenotypes - our first experience

#### B. Balažiová^1^, P. Čižnár^1^, D. Moravčíková^2^, I. Hulínková^1^, T. Freiberger^3^, E. Froňková^4^, T. Dallos^1^

##### ^1^Department of Paediatrics, Comenius University Medical School in Bratislava, Bratislava, ^2^2nd Department of Paediatrics, Slovak Medical University, Banská Bystrica, Slovakia, ^3^Genetic laboratory, Centre for Cardiovascular and Transplant Surgery, Brno, ^4^Department of Paediatric Haematology and Oncology, 2nd Faculty of Medicine, Charles University, Prague, Czech Republic

###### **Correspondence:** B. Balažiová

**Introduction:** Deficiency of ADA2 (DADA2) is a rare multisystem monogenic autoinflammatory disease with a constantly increasing spectrum of clinical phenotypes and disease-causing mutations in the ADA2 gene (formerly CECR1).

**Objectives:** To analyse the phenotypes of our first two Slovak patients with DADA2.

**Methods:** Demographics and clinical phenotype of 2 patients with DADA2 were analysed in retrospect by review of their medical records.

**Results:** Patient 1 is a Caucasian boy of unrelated parents, who presented acutely with diplopia and ataxia due to a mesencephalic brain lesion at the age of 4.5 years. Soon after neurological restoration, he developed intermittent fever, livedo racemosa, bilateral hand thrombophlebitis after cannulation, splenomegaly with multiple focal lesions considered to be due to embolism and generalized lymphadenopathy. Persistently increased inflammatory markers (CRP 50-60 mg/l), B-lymphopenia, hypogammaglobulinemia with low post-vaccination antibodies were noted. Inflammation responded promptly to corticosteroids (CS); he was substituted with immunoglobulins regularly. Two heterozygous mutations in the ADA2 gene were confirmed at the age of 5.3 years (pathogenic missense mutation c.506G>A;p.Arg169Gln(1) and a missense variant of uncertain significance c.505C>T;p.Arg169Trp(2)). Biologic therapy with etanercept resulted in control of inflammation, the immunological disorder persists.

Patient 2 is a Caucasian girl of unrelated parents, treated for autoimmune haemolytic anaemia (AIHA) at 8 months of age, who developed persistent firm maculopapular lesions after CS discontinuation (11 months). She developed arthralgias without apparent arthritis and intermittent fever (up to 38.5°C) at 21 and 23 months, respectively, splenomegaly and permanently increased inflammatory parameters (CRP 50-110 mg/l) were noted and the patient was treated for suspected systemic JIA with pulse CS and methotrexate and later cyclosporine A. With CS tapering intermittent fever and joint pain recurred, livedo racemosa was noted and a defect in B-cell differentiation without hypogammaglobulinaemia was identified (28 months). NGS analysis revealed two heterozygous mutations in the ADA2 gene (pathogenic missense mutation c.140G>C;p.Gly47Ala(3) and a new variant c.881+1G>C predicted to considerably reduce the efficacy of gene splicing), each inherited from one parent. Treatment with etanercept was recommended.

**Conclusion:** Even in our limited experience, our first two Slovak patients with DADA2 presented with highly variable phenotypes and with two novel, likely pathogenic mutations identified in each patient. A high degree of suspicion is required to identify DADA2 among patients with persistent inflammation, immunological and/or haematological disorders, as well as neurologic manifestations.

**References:**

1. National Center for Biotechnology Information. ClinVar; [VCV000120303.32] [Internet]. [cited 2022 May 24]. Available from: https://www.ncbi.nlm.nih.gov/clinvar/variation/120303/

2. National Center for Biotechnology Information. ClinVar; [VCV000956376.3] [Internet]. [cited 2022 May 23]. Available from: https://www.ncbi.nlm.nih.gov/clinvar/variation/956376/

3. National Center for Biotechnology Information. ClinVar; [VCV000120301.14] [Internet]. [cited 2022 May 24]. Available from: https://www.ncbi.nlm.nih.gov/clinvar/variation/120301/

**Patient Consent:** Yes, I received consent

**Disclosure of Interest**: None declared

### P114. Efficacy and safety of anti-interleukin-1 treatment in large colchicine resistant familial Mediterranean fever cohort: single center experience

#### K. Barut, I. Ulkersoy, N. Gucuyener, F. Haslak, M. Yildiz, A. Gunalp, G. Yalcin, S. Sahin, A. Adrovic, O. Kasapcopur

##### Department of Pediatric Rheumatology, Istanbul University Cerrahpasa, Istanbul, Turkey

###### **Correspondence:** K. Barut

**Introduction:** Familial Mediterranean fever (FMF) is the most common hereditary autoinflammatory disease, characterized recurrent episodes of fever peritonitis, pleuritis, arthritis and rash, Colchicine is the mainstay of FMF treatment, although colchicine is the first line treatment in FMF, 5-10% of patients do not respond, another 2–5% do not tolerate the drug well. Interleukin (IL)-1 antagonists are the treatment of choice in resistant or intolerant cases. The FMF50 score is used in evaluation of disease activity, defined as 50% improvement in all of 6 criteria. Complete clinical and laboratory response is characterized with absence of clinical features and normal laboratory findings

**Objectives:** We aimed to evaluate anti IL-1 effect (anakinra and canakinumab) on colchicine resistant FMF cases by comparing the FMF50 score and complete clinic and laboratory response.

**Methods:** In this study all FMF patients followed up at tertiary pediatric rheumatology clinic assessed by examining patients’ files and network information, among these cases who met definition of resistant FMF were included in the study. in the first step colchicine dosage increased to maximum tolerated dose, after maximum dosage colchicine, if still had considered Cr FMF, then started other colchicine form (opacalcium colchicine OC) anti-IL1 treatment was started in the patients who were resistant despite receiving the OC. The FMF50 response of the resistant FMF patients under colchicine or other treatment modalities for at least 6 months has been determinates retrospectively by using data from the patients’ records. Complete clinical and laboratory remission has been evaluated for all resistant FMF patients.

**Results:** A total 74 FMF patients has been considered colchicine resistant FMF(Cr FMF).FMF 50 response has been obtained in only 5/74(6.7%) patients under colchicine treatment. Among patients under other treatment options, FMF 50 response has been obtained in 23/48(48%) patients under OC, in 13/18(72.2%) patients treated with anakinra and in 26/28(93%) patients under canakinumab treatment. There was a statistically significant difference in FMF50 response according to treatment modality (p<0,05). Clinical remission has been achieved in 18/48(37,7%) patients under Opacalcium colchicine, 12/18(66,7%) patients under anakinra and in 25/28(89,3%) patients under Canakinumab. There was a statistically significant difference between patients treated with OC and those with anti-IL-1 according to complete clinical remission (p<0,05).

Laboratory remission has been obtained in 19/48(39.6%), 11/18(61,1%) and 23/28(82,1%) patients treated with OC, anakinra and canakinumab, respectively. The laboratory remission was significantly different between patients treated with OC and anti-IL-1 agents (p<0,001). In patients treated with maximum dosage of colchicine (1,7±0,4 mg/day, max 2 mg/day), the most seen adverse effect was diarrhea in 18/74(24,3%), transaminase elevation in 4/74(5.4%) patients and leukopenia only in 2 patient. Diarrhea was seen in 10/48(20.8%) patients under opacalcium colchicine treatment, which is lower comparing to previously mentioned colchicine form. There was no serious adverse event with Anti- IL 1 treated patients.

**Conclusion:** Despite the regular and maximum dose of colchicine use, Cr FMF cases are seen at a substantial rate. In these CrFMF cases, FMF50 response was higher in both canakinumab and anakinra, and clinical and laboratory remission found more than other colchicine form. In this study found that, the both of anti IL-1 agents were safe and effective in colchicine resistant FMF patients.

**Patient Consent:** Yes, I received consent

**Disclosure of Interest**: None declared

### P115. A score for predicting colchicine resistance at the time of diagnosis in Familial Mediterranean Fever (FMF): data from the TURPAID registry

#### E. D. Batu^1^, S. Şener^1^, E. Arslanoglu Aydin^2^, E. Aliyev^1^, I. Bagrul^2^, S. Türkmen^3^, O. Akgün^4^, Z. Balık^1^, A. Tanatar^4^, Y. Bayındır^1^, Z. Kızıldağ^5^, R. Torun^5^, A. Günalp^6^, T. Coskuner^3^, R. İşgüder^5^, T. Aydın^5^, F. Haşlak^6^, M. Kasap Cuceoglu^1^, E. Esen^7^, U. Akçay^3^, Ö. Başaran^1^, A. Pac Kisaarslan^7^, F. Akal^8^, S. Özdel^2^, M. Bülbül^2^, Y. Bilginer^1^, N. Aktay Ayaz^4^, B. Sözeri^3^, O. Kasapcopur^6^, E. Unsal^5^, S. Ozen^1^

##### ^1^Hacettepe University Faculty of Medicine, ^2^Dr. Sami Ulus Maternity and Child Health and Diseases Research and Training Hospital, Ankara, ^3^Umraniye Research and Training Hospital, ^4^Istanbul University Faculty of Medicine, Istanbul, ^5^Dokuz Eylül University Faculty of Medicine, Izmir, ^6^Istanbul University Cerrahpasa Faculty of Medicine, Istanbul, ^7^Erciyes University Faculty of Medicine, Kayseri, ^8^Department of Computer Engineering, Hacettepe University, Ankara, Turkey

###### Correspondence: E. D. Batu

**Introduction:** Familial Mediterranean fever (FMF) is the most common monogenic systemic autoinflammatory disease. Colchicine forms the mainstay of FMF treatment. Around 5-10% of FMF patients are colchicine resistant and require anti-interleukin 1 (IL-1) drugs.

**Objectives:** To compare the characteristics of colchicine resistant and colchicine responsive patients and to develop a score for predicting colchicine resistance at the time of FMF diagnosis.

**Methods:** FMF patients enrolled in the TURPAID (Turkish Pediatric Autoinflammatory Diseases) registry were included. All patients met the Eurofever FMF criteria and had confirmatory *MEFV* genotype. Colchicine resistance was defined as having one or more attacks per month or presence of subclinical inflammation despite receiving maximum tolerated dose of colchicine. The predictive score for colchicine resistance in FMF was developed by using univariate/multivariate regression and ROC analyses.

**Results:** A total of 2944 FMF patients (297 colchicine resistant and 2687 colchicine responsive) were included (F/M=1.02, mean age at diagnosis 78.3±47.7 months). In colchicine resistant patients, the age of symptom onset and diagnosis was younger (39.1±33.9 vs. 55.4±43.8; p<0.001 and 64.2±43.1 vs. 79.7±48.0 months; p<0.001, respectively) the mean duration of attacks (2.8±1.0 vs. 2.6±1.0 days, p=0.009) and the number of attacks in the one year before diagnosis (16.7±12.9 vs. 11.3±10.4, p<0.001) were higher than colchicine responsive patients. Considering the clinical findings, fever (91.4% vs. 82%), malaise (5.4% vs. 2.8%), erysipelas-like erythema (12.5% vs. 5.7%), arthralgia (50.8% vs. 44.3%), arthritis (34.9% vs. 18.1%), myalgia (16.1% vs. 10.1%), abdominal pain (91.4% vs. 84.2%), diarrhea (10.1% vs. 5.6%), and chest pain (31.9% vs. 16.3%) were significantly more prevalent among colchicine resistant patients compared to colchicine responsive patients (p<0.05). Also, comorbidity (28.4% vs. 14.5%), parental consanguinity (24.9% vs. 15.4%), and homozygosity or compound heterozygosity for exon 10 MEFV mutations (90.3% vs. 62.3%) were more frequent in colchicine resistant than responsive patients (p<0.05). With univariate and multivariate regression and ROC analyses, a score for predicting colchicine resistance in FMF was developed (Table 1). The cut-off value that discriminated best between colchicine resistant and colchicine responsive FMF patients was ≥6. Its sensitivity was 78% while its specificity was 60%.

**Conclusion:** The strongest predictors of colchicine resistance at the time of FMF diagnosis were the presence of arthritis, chest pain, homozygosity/compound heterozygosity for exon 10 mutations, and ≥1 attack/month. This predictive score could help us to identify FMF patients with a higher risk of severe disease.

**Funding:** This study was supported by the Science Academy’s Young Scientist Awards Program (BAGEP) of Turkey.

**Patient Consent:** Yes, I received consent

**Disclosure of Interest**: None declared

**Table 1 (abstract P115).** Score for predicting colchicine resistant disease course in FMF patients

Feature	Points per item
Arthritis	2 points
Chest pain	2 points
Homozygosity/compound heterozygosity for exon 10 mutations	4.5 points
≥1 attack/month	2 points

### P116. Menstruation-triggered attacks in adolescent girls with Familial Mediterranean Fever (FMF)

#### E. D. Batu, Y. Bayındır, Z. Balık, S. Sener, E. Aliyev, M. Kasap Cuceoglu, U. Kaya Akca, O. Basaran, Y. Bilginer, S. Ozen

##### Hacettepe University Faculty of Medicine, Ankara, Turkey

###### **Correspondence:** E. D. Batu

**Introduction:** Familial Mediterranean fever is a monogenic autoinflammatory disease characterized by febrile episodes of serositis. In some FMF patients, attacks are triggered by menstruation. There are no studies analyzing peri-menstrual FMF attacks focused on adolescent girls with FMF.

**Objectives:** To analyze the characteristics and treatment in adolescent FMF patients with menstruation-triggered attacks compared to FMF patients who did not have any menstruation-triggered FMF attacks.

**Methods:** Post-pubertal girls (12-18 years of age) with FMF were included. All patients met the Eurofever criteria for FMF and had confirmatory MEFV genotypes.

**Results:** A total of 153 adolescent girls with FMF were included. FMF attacks were triggered by menstruation in 37 (24.2%). In these patients, the median number of attacks within one year increased from 4 to 10 after menarche (p=0.005). The median age at disease onset and diagnosis were younger and both pre-menarche and post-menarche attack frequency were higher in FMF patients with menstruation-triggered attacks compared to the rest of the group (Table). Dismenorrhea was more common among these patients, as well (Table). Regarding the characteristics of non-menstruation associated FMF attacks, the features were similar between two groups (Table). Also, the frequency of patients with exon 10/exon 10 *MEFV* mutations was similar among two groups. 17 out of 37 patients did not receive any treatment addressing their menstruation-triggered attacks. Colchicine dose was increased or a different preparation of colchicine was initiated in 14 patients and 11 of them benefit from this change. On demand anakinra or corticosteroid use was prescribed to two and one patients. Two patients were receiving NSAIDs during the first 3 days of their menstruation which prevented menstruation-triggered attacks.

**Conclusion:** Menstruation-triggered FMF attacks are probably more common among adolescents. These patients often have earlier disease onset and higher attack frequency in the pre-menarche period, as well. Raising awareness about these patients would help us to improve effective management strategies.

**Patient Consent:** Yes, I received consent

**Disclosure of Interest**: None declared

**Table 1 (abstract P116).** See text for description

Median (min-max) OR n (%)	FMF patients with menstruation-triggered attacks (n=37)	FMF patients without menstruation-triggered attacks (n=116)	P value
Age at disease onset, years	3 (0.15-15)	5 (0.5-15.5)	0.005
Age at diagnosis, years	5 (1.5-15.7)	7 (0.75-17)	0.01
Pre-menarche attack frequency*	4 (0-20)	2 (0-20)	<0.001
Post-menarche attack frequency*	10 (2-24)	0 (0-12)	<0.001
Dysmenorrhea	28 (75.7)	45 (38.8)	<0.001
Abdominal pain during attacks**	31 (83.8)	88 (75.9)	0.31
Arthritis during attacks**	3 (8.1)	22 (19)	0.12
Fever during attacks**	30 (81.1)	84 (72.4)	0.29
Chest pain during attacks**	12 (32.4)	22 (19)	0.086

### P117. The performance of a gene panel analysis among patients with systemic autoinflammatory diseases

#### Y. Bayındır^1^, E. D. Batu^1^, O. Bölük^2^, C. Koşukcu^3^, E. Sağ^2^, A. Özcan^2^, M. Kasap Cüceoğlu^1^, Z. Balık^1^, S. Şener^1^, E. Aliyev^1^, H. Ö. Başaran^1^, Y. Bilginer^1^, S. Özen^1^

##### ^1^Hacettepe University Faculty of Medicine, ^2^Ankara Research and Training Hospital, ^3^Hacettepe University, Department of Bioinformatics, Institute of Health Sciences, Ankara, Turkey

###### **Correspondence:** Y. Bayındır

**Introduction:** Systemic autoinflammatory diseases (AIDs) are usually associated with disturbances of innate immune system. Although clinical phenotypes may suggest a specific SAID, most of these diseases are diagnosed with genetic tests.

**Objectives:** To determine the diagnostic yield of our autoinflammatory gene panel (mainly covering inflammasomopathies) in a cohort of AID patients and analyze and compare the characteristics of gene panel positive versus negative patients.

**Methods:** Patients with features of AIDs were included in this study followed in the Department of Pediatric Rheumatology at Hacettepe University, all initially screened for the common MEFV mutations. They were screened with a gene panel including IL1RN, ADA2, IL10RA, IL10RB, NOD2, PSMB8, LPIN2, MEFV, MVK, NLRP3, PSTPIP1, TNFRSF1A, NLRP12, NLRP7, CARD14, TNFRSF11A, ELANE genes. Patients with a definite or probable disease-causing variant were classified as being ‘panel positive’. ROC analysis was used to determine best performing cut-off value for C-reactive protein (CRP).

**Results:** In our cohort, we enrolled 122 (40.2% female) patients suspected of AID. The median age at symptom onset was 23 months (range 0.1-180). Gene panel provided a definite or probable disease-causing variant in 26 of 122 patients (21.3%). These patients were diagnosed with: FMF (n=12), HIDS (n=8), CAPS (n=1), TRAPS (n=1), Blau syndrome (n=1), PAPA (n=1), DADA-2 (n=1), NLRC3-related disease (n=1). Of the panel negative patients, 14 were fulfilling Eurofever criteria for autoinflammatory recurrent fevers. Male gender (76.9% vs. 55.2%; p=0.046) and diarrhea (38.5% vs. 19.8%; p=0.048) were more prevalent and the median CRP levels during disease flares were higher (12.8 vs. 6.39 mg/dl; p=0.009) among panel positive patients than panel negative patients (Table 1). The cut-off CRP value that discriminated best between panel positive and panel negative patients was >6 mg/dl (sensitivity 72%; specificity 50%).

**Conclusion:** In our cohort, the aforementioned panel offered diagnosis in 21.3% of the patients. Urticarial rash and arthralgia/arthritis were more frequent among panel positive patients and a higher CRP predicted gene panel positivity. The reason for this could be due to the fact that the gene panel used was mainly directed to genes associated with IL-1.

**Patient Consent:** Yes, I received consent

**Disclosure of Interest**: None declared

**Table 1 (abstract P117).** The characteristics of gene panel positive and negative patients who had features of systemic autoinflammatory diseases

Characteristics	Panel positive (n=26)	Panel negative (n=96)	p-value
Female (n, %)	6 (23.1)	43 (44.8)	0.046
Age at symptom onset, months, (median, range)	18 (1.5-150)	24 (0.1-180)	0.943
Abdominal pain (n, %)	13 (50)	45 (46.9)	0.778
Arthritis (n, %)	2 (7.7)	15 (15.6)	0.302
Aphthous stomatitis (n, %)	5 (19.2)	24 (25)	0.542
Tonsillitis (n, %)	8 (30.8)	36 (37.5)	0.528
Urticarial rash (n, %)	5 (19.2)	7 (7.3)	0.071
Diarrhea (n, %)	10 (38.5)	19 (19.8)	0.048
C-reactive protein (mg/dL) (median, range)	12.8 (0.64-26.6)	6.39 (0.13-30.9)	0.009

### P118. A novel missense mutation in NLRP3 causing inflammasome hyperactivation and subsequent sensorineural hearing loss as a part of atypical CAPS

#### M. Birk-Bachar^1^, H. Cohen^2^, E. Sofrin-Dr^1,2^, N. Kropach-Gilad^2,3^, N. Orenstein^1^, L. Kornreich^2,3^, R. Tal^1,2^, G. Amarilyo^2,3^, Y. Levinsky^2,3^, M. Sokolov^2,3^, E. Raveh^1,2^, M. Gerlic^2^, L. Harel^1,2^

##### ^1^Schneider Children’s Medical Center of Israel, Petach Tikvah, ^2^Sackler Faculty of Medicine, Tel Aviv University, Tel Aviv, ^3^Schneider Children’s Medical Center of Israel, Petach Tikva, Israel

###### **Correspondence:** M. Birk-Bachar

**Introduction:** Gain-of-function mutations in the NLRP3 gene, lead to hyperactivation of the NLRP3 inflammasome, resulting in the inappropriate release of inflammatory cytokines including IL-1β, and cause a spectrum of autosomal-dominant systemic autoinflammatory diseases called cryopyrin-associated periodic syndromes (CAPS). Many of these patients also develop progressive sensorineural hearing loss (SNL) due cochlear autoinflammation, which in rare cases may be the primary finding. ^(1-4)^ A Jewish Ashkenazi family, presented at our clinic with: autosomal dominant progressive sensorineural hearing loss, without clinical features consistent with typical CAPS, and a novel missense variant in the NLRP3 gene (NM 001079821:c.1790G>A, p.Ser597Asn).

**Objectives:** The combination of this unique clinical presentation spanning 3 generations, alongside the novel variant in NLRP3, led us to explore whether carriers of the novel variant indeed show evidence of NLRP3 inflammasome hyper-activity.

**Methods:** We conducted a prospective study in 15 family members (10 known carriers, 5 age-group matched non-carriers), which included clinical and hearing assessment along with functional measurement of the NLRP3 inflammasome activity. Peripheral blood mononuclear cells (PBMCs) were isolated from peripheral blood. IL-1 levels secreted by PBMCS were measured under 3 main conditions: basal state, exposure to IL-1 stimulant (lipopolysaccharide, LPS) and exposure to LPS + MCC950 (specific inflammasome inhibitor). Quantitative in-vitro measurement of secreted IL-1 in supernatants of carriers and controls was conducted using ELISA (Enzyme-Linked Immunosorbent Assay).

**Results:** Of 10 known carriers, 6 family members from 3 generations, suffered from progressive SNL, varying from isolated high frequency impairment to severe hearing loss and cochlear implant. All family members with hearing impairment were variant carriers, and carriers who had normal hearing function were all under the age of 13. 6 carriers reported occasional episodes of fever/arthralgia, 3 of which reported past episodes of monoarthritis. Functional assessment of the inflammasome in carriers vs. non carriers (controls) revealed that although basal levels of secreted IL-1 were not significantly different in both groups, the fold of change (FC) of secreted IL-1 in response to LPS stimulation was at least 2 times higher among carrier vs. control group (2 way Anova, P-value < 0.05, sidak multiple comparison). Addition of MCC950 inhibited IL-1β secretion after stimulation with LPS+CaCl2, in both groups.

**Conclusion:** The suggestive clinical presentation, genotype-phenotype correlation, and evidence of inflammasome hyperactivation, as seen in functional inflammasome stimulation tests, are proof that the novel variant 001079821:c.1790G>A p.Ser597Asn is a pathogenic gain of function mutation. This hyperactivation state leads to atypical CAPS phenotype, with predominant non-syndromic and syndromic bilateral progressive SNL that initially affects ultra-high frequency ranges.

**Trial registration identifying number:** Study was approved by the local Helsinki Comittee (RMC-0941-20)

**Patient Consent:** Yes, I received consent

**Disclosure of Interest**: None declared

### P119. A family with pulmonary deficiency, polyarthritis, glomerulonephritis and vasculopathy: diagnosis in 3rd generation

#### L. Malz^1^, M. A. Lee-Kirsch^1,2^, C. Wolf^1^, B. Mayer^1^, C. Günther^3^, K. Stamos^1^, C. Schütz^1,2^, R. Berner^1,2^, N. Brück^1^

##### ^1^Department of Pediatrics, ^2^UniversitätsCentrum für Seltene Erkrankungen, ^3^Department of Dermatology, Medizinische Fakultät Carl Gustav Carus, Technische Universität Dresden, Dresden, Germany

###### **Correspondence:** N. Brück

**Introduction: History and main symptoms**

We report on a family with an unusual clustering of interstitial lung disease as well as acral vasculopathy and polyarthritis. The index patient – a 10-year-old boy - was referred to us with progressive pulmonary deterioration, chilblain lesions and anemia. He presented with severe growth retardation, arterial hypertension and glomerulonephritis. Laboratory investigations showed increased inflammation, strongly positive values for RF, ANA and cANCA. The child’s father had bluish-discolored acral skin lesions and suffered from fibrosing interstitial lung disease since the age of 11 years. Additionally he reported night sweats, pulmonary hypertension, and right ventricular failure. Due to respiratory failure, he had been treated with cyclophosphamide, azathioprine and corticosteroids in adolescence.

The family history revealed similar clinical abnormalities in the paternal grandmother and aunt. The grandmother who had suffered from severe rheumatoid arthritis died at the age of 35 years from lung disease. The paternal aunt presented with recurrent pneumonia at the age of 1 year, followed by diagnosis of interstitial pulmonary fibrosis at 11 years, and onset of severe polyarthritis, anemia and accompanying glomerulonephritis at the age of 12. Her pulmonary deterioration was only temporarily controlled with cyclophosphamide and corticosteroids. She died at the age of 20 due to acute respiratory failure.

**Objectives: Diagnosis**

In view of the suggestive family history, and high clinical suspicion of STING-associated vasculopathy with onset in infancy (SAVI), genetic testing was initiated revealing the following pathogenic variant in the TMEM173 gene (c.463G>A [p.Val155Met; V155M] heterozygous) within a few days. The child’s father is also carrier of the mutation.

SAVI is a rare autoinflammatory disease with autosomal dominant inheritance, caused by gain-of-function variants in the TMEM173 gene (prevalence 1:1 M births). The V155M variant is located in the dimer interface of the STING protein (stimulator of interferon genes), and causes hyperactivation of the type 1 interferon axis. Systemic inflammation and small-vessel-vasculopathy start in infancy. Patients later present with interstitial lung disease and failure to thrive, acral dermatoses, and elevated acute-phase proteins and interferon signature. More rarely, anemia, polyarthritis and chronic glomerulonephritis with nephrotic syndrome are the presenting features.

**Methods:** Case report

**Results: Therapy and prognosis**

After confirming the diagnosis, therapy with the Januskinase (JAK) inhibitor Ruxolitinib was initiated, which interrupts the positive interferon feedback loop. Treatment led to normalization of the initially pronounced type 1 interferon activation (score: 8907.36) within 28 days. Clinically, there was a striking improvement in respiratory (FVC 59% to 79%) and renal functions.

The index patient’s father is being treated with the JAK inhibitors Baricitinib and Nintedanib

**Conclusion: Discussion**

Chilblain lesions and interstitial lung disease in combination with other autoimmune phenomena are suggestive for rare monogenic diseases such as SAVI. Genetic testing is essential for rapid initiation of targeted therapies.

**Patient Consent:** Yes, I received consent

**Disclosure of Interest**: None declared

### P120. Experience with IL-1 inhibitors in patients with monogenic autoinflammatory diseases in a tertiary hospital’s pediatric rheumatology unit

#### I. Burgos Berjillos^1^, M. Gonzalez Fernandez^2^, B. Lopez Montesinos^1^, L. Lacruz Pérez^1^, M. Martí Masanet^1,2^, I. Calvo Penadés^1^

##### ^1^Pediatric Rheumatology Unit, Hospital Universitario y Politécnico La Fe, Valencia, ^2^Pediatric Rheumatology Unit, Medical Research Institute Hospital La Fe, Valencia, Spain

###### **Correspondence:** I. Burgos Berjillos

**Introduction:** IL-1 plays a pivotal role in the pathogenesis of some monogenic autoinflammatory diseases (AID) in which IL-1 inhibitors have been shown to be effective.

**Objectives:** To describe the experience with IL-1 inhibitors in patients with a monogenic AID in a pediatric rheumatology unit in a tertiary hospital.

**Methods:** Clinical and demographical data, efficacy, dose and adverse events were collected from patients with a monogenic AID who received treatment with IL-1 inhibitors (anakinra and/or canakinumab), followed in our pediatric rheumatology unit from January 2007 until December 2021.

**Results:** 31 patients were identified with a monogenic AID diagnosis and treatment with IL-1 blockers (table 1), 16 anakinra treatment courses (3 FMF, 7 HIDS, 5 TRAPS, 1 APLAID) and 26 canakinumab treatment courses (9 FMF, 9 HIDS, 4 TRAPS, 2 CAPS, 2 PAPA). Median (Q1;Q3) treatment time with anakinra and canakinumab was: 1,98 (1,04 ; 3,70) and 5,61 (2,30 ; 7,16) years, respectively.

We assessed treatment response: 81,3% of patients treated with anakinra achieved disease control, 25% complete response (3 FMF, 1 HIDS) and 56,3% partial response (6 HIDS, 2 TRAPS, 1 APLAID); for canakinumab courses, 92,3% of patients responded, 73,1% with complete response (6 FMF, 6 HIDS, 3 TRAPS, 2 CAPS, 2 PAPA) and 19,2% with partial response (3 FMF, 2 HIDS).

Related to dose management, 4/7 HIDS patients and 2/5 TRAPS patients needed anakinra dose increase (>2mg/kg/day or >100mg/day). 8/9 HIDS patients, 2/4 TRAPS and 1/2 CAPS patients needed canakinumab dose increase (4mg/kg/4 week or 300mg/4 week).

During the follow-up, it was possible to decrease the dose (interval increase) in 12/26 (46,2%) patients treated with canakinumab (5 FMF, 3 TRAPS, 2 HIDS and 2 PAPA).

Reported severe or of interest adverse events were 2 pneumonia in 2 patients and 2 generalized skin reaction in 2 patients while on anakinra and 5 pneumonia in 4 patients, 2 appendicitis, soft tissue infections in 2 patients and 2 varicella-zoster virus primoinfection while on canakinumab. One patient with HIDS developed hidradenitis during the follow-up. No death was reported.

**Conclusion:** We describe our experience with IL-1 inhibitors in a cohort of patients with monogenic AID. About one third (39%) of our patients with a monogenic AID were treated with IL-1 blockers, leading them to a better disease control. Globally, 67,7% of patients achieved remission. In our cohort, 89% of HIDS patients, and around 50% of TRAPS and CAPS patients needed higher dose of IL-1 inhibitors to achieve disease control. These findings highlight the importance of IL-1 blockade, with an accurate dose adjustment, in monogenic AID.

**Disclosure of Interest**: I. Burgos Berjillos: None declared, M. Gonzalez Fernandez: None declared, B. Lopez Montesinos: None declared, L. Lacruz Pérez: None declared, M. Martí Masanet: None declared, I. Calvo Penadés Consultant with: Novartis, Speaker Bureau with: Novartis, Sobi

**Table 1 (abstract P120).** See text for description

	n	Female (%)	Age at first IL-1 inhibitor started (years), median (Q1-Q3)	Age at onset (years), median (Q1-Q3)	Age at diagnosis (years), median (Q1-Q3)
FMF	11	2 (18,18)	15,06 (11,16-17,60)	4,00 (1,75-6,77)	6,70 (4,35-11,61)
HIDS	9	4 (44,44)	7,85 (6,17-13,16)	4 (1,75-6,77)	6,38 (5-12)
TRAPS	6	1 (16,66)	10,46 (4,60-13,47)	4,50 (2,44-6,63)	8,01 (2,57-9,00)
CAPS	2	0	4,92	2,08	4,60
PAPA	2	0	9,60	5	9,47
APLAID	1	0	4,17	2,5	3,74
All	31	7 (22,58)	11,01 (6,71-14,45)	3,00 (1,33-5,50)	6,97 (4,13-11,23)

### P121. Acute febrile neutrophilic dermatosis -sweet syndrome- case presentation

#### A. Calin, C. Scurtu, D. Sfrijan, R. Vidlescu

##### Pediatrics, M.S. Curie Hospital, Bucuresti, Romania

###### **Correspondence:** A. Calin

**Introduction:** In most cases, the cause of Sweet’s syndrome isn’t known. Acute febrile neutrophilic dermatosis is an uncommon skin condition characterised by fever and inflamed or blistered skin and mucosal lesions.

**Objectives:** Sweet’s syndrome can present in several clinical settings: classical (or idiopathic) Sweet’s syndrome, malignancy-associated Sweet’s syndrome (leukemia or solid tumors, such as breast or colon cancer) and drug-induced Sweet’s syndrome (most commonly a type of drug that boosts production of white blood cells). Classical Sweet’s syndrome usually presents in women between the age of 30 to 50 years, it is often preceded by an upper respiratory tract infection. We present ca case of a 9 years old boy with malignancy-associated SS.

**Methods:** The authors present a case of a 9 years old boy that was admitted to our oncology departement for acute leukemia. He developed a painful skin rash (plaques and nodules), fever resistent to antibiotics, arthralgia, elevated ESR and CRP. Clinical, laboratory and histopatological (oedema of the dermis and a diffuse infiltrate of numerous neutrophils with leukocytoclasis without vasculitis in the superficial and the deep dermis) findings established the diagnostis.

Diagnostic criteria for classic acute febrile neutrophilic dermatosis have been proposed.

**Results:** Sweet syndrome, although rare, it can be present in children. This is why pediatricians with rheumatology concerns and beyond, need to know about it and to take it into consideration when needed.

**Conclusion:** Early recognition and treatment of malignancy-associated Sweet syndrome is imperative to limit patient morbidity and provide anti-cancer therapy.

**Patient Consent:** Yes, I received consent

**Disclosure of Interest**: None declared

### P122. Effects of unopposed IL-18 in mixed inflammatory environments

#### V. Dang^1^, E. Landy^2^, J. Varghese^1^, L. Van Der Kraak^2^, L. Huang^1^, A. Frank-Kamenetskii^1^, S. Canna^1^

##### ^1^The Children’s Hospital of Philadelphia, Philadelphia, ^2^University of Pittsburgh, Pittsburgh, United States

###### **Correspondence:** S. Canna

**Introduction:** Interleukin 18 (IL-18) is an inflammasome-activated, IL-1 family cytokine that canonically induces interferon-gamma (IFNg). IL-18 activity is potently inhibited by a soluble, IFNg-inducible antagonist, IL-18 Binding Protein (IL-18BP). IFNg appears central to the pathogenesis of rare, hyperinflammatory states like Hemophagocytic Lymphohistiocytosis (HLH) and Macrophage Activation Syndrome (MAS), and IL-18 is both a biomarker of MAS and may be central to its pathogenesis. In some patients, MAS may compete with IFNg-independent features like arthritis, and IL-18 may contribute to both. More recently, patients with PSTPIP1 mutations causing neutrophilic skin rashes and arthritis were found to have chronic elevation of IL-18. These observations reinforce abundant evidence from model systems that IL-18 is also capable of amplifying Type 2 and 17 inflammatory responses.

**Objectives:** We hypothesized that excess IL-18 will amplify the dominant inflammatory T-cell paradigm and exaggerate models of mixed inflammation regardless of their prevailing type of inflammation (e.g. Type 1, Type 2, Type 17, etc.).

**Methods:** C57BL/6 WT and transgenic mice underwent experimental autoimmune encephalomyelitis (EAE) and inhaled house dust mite (HDM), which are models Type 1/17 and Type 1/2 competition, respectively.

**Results:** Without stimulation, *Il18bp*^*-/-*^ mice show no baseline skew towards Type1 responses except in intestinal epithelium. By contrast, *Il18tg* mice show a mild increase in serum IFNg and IFNg-producing cells in multiple tissues, most notably liver and spleen. Upon induction of EAE, *Il18bp*^*-/-*^ mice were profoundly protected from weight loss, clinical score, and CNS infiltration of T-cells. This protection was lost upon systemic neutralization of IFNg, suggesting unopposed IL-18 was protective via induction of IFNg. Intestinal immune responses have been shown to affect EAE. However, mice with an *Nlrc4* inflammasome mutation causing Th1-expansion and excess IL-18 restricted to the intestines were not protected from EAE. Notably, CD4 T-cell infiltration into spinal cords was not significantly different during EAE in WT and *Il18bp*^*-/-*^ mice, but only *Il18bp*^*-/-*^ mice showed significant infiltration of CD8 T-cells.

Excess IL-18 protected against airway eosinophilia and Th2 differentiation in HDM in *Il18tg* but not *Il18bp*^*-/-*^ mice, possibly due to minimal induction of IL-18 in the latter.

**Conclusion:** Despite IL-18’s ability to amplify Type 2 and 17 responses in some circumstances, in models where T-cell differentiation states actively compete with each other, excess IL-18 prevents against non Type 1 immunopathology. This likely occurs indirectly, via the products of preferential-enhancement of type 1 responses. This may be relevant to therapeutics aimed at blocking pathogenic Th2 or 17 responses or in predicting the consequences of IL-18 blockade in complex inflammatory states.

**Patient Consent:** Not applicable (there are no patient data)

**Disclosure of Interest**: V. Dang: None declared, E. Landy: None declared, J. Varghese: None declared, L. Van Der Kraak Employee with: Bluesphere Bio, L. Huang: None declared, A. Frank-Kamenetskii: None declared, S. Canna Grant / Research Support with: AB2Bio, Novartis, SOBI, IMMvention Therapeutix, Consultant with: Simcha Therapeutics, Paid Instructor with: Clinical Viewpoints

### P123. DNA methylation and gene expression signatures in CD14+ monocytes separate cno patients from healthy controls

#### E. Carlsson^1^, A. Charras^1^, G. Duffy^1^, M. W. Beresford^1^, H. J. Girschick^2^, H. Morbach^3^, C. M. Hedrich^1^

##### ^1^Department of Women’s and Children’s Health, University of Liverpool, Liverpool, United Kingdom, ^2^Department of Paediatrics, Vivantes Klinikum im Friedrichshain, Berlin, ^3^Pediatric Immunology and Rheumatology, Children’s Hospital, University of Würzburg, Würzburg, Germany

###### **Correspondence:** E. Carlsson

**Introduction:** Chronic non-bacterial osteomyelitis (CNO), and its severe form chronic recurrent multifocal osteomyelitis (CRMO), is an autoinflammatory bone disease typically affecting children and adolescents. While the underlying pathophysiological molecular mechanisms are not well understood, dysregulation of monocyte-derived pro- and anti-inflammatory cytokines has been reported in previous studies.

**Objectives:** This study aimed at identifying CNO/CRMO disease-specific gene expression and DNA methylation signatures in monocytes that may be used as biomarkers or therapeutic targets.

**Methods:** Peripheral blood mononuclear cells (PBMCs) from healthy controls (n=12) and CNO/CRMO patients (n=12) were collected at diagnosis and 6-12 months after treatment initiation with naproxen. Cells were immunostained against CD14 and CD16 to identify and characterise monocytes, which were isolated via FACS. Monocyte RNA and DNA were then extracted using a Qiagen AllPrep DNA/RNA Kit. Isolated RNA was subjected to rRNA depletion and RNAseq analysis to identify differentially expressed genes, while isolated DNA was analysed by Illumina Infinium Methylation EPIC Arrays to identify DNA methylation signatures. Gene Ontology (GO) and KEGG pathway analyses was performed for differentially expressed genes and genes presenting at least one promoter differentially methylated position (TSS1500, TSS200, 5’UTR).

**Results:** CNO/CRMO patients exhibited an increased proportion of classical monocytes compared to age- and sex-matched healthy controls (98% vs 93%, p<0.05), which did not significantly change following treatment with naproxen (98% vs 98%, ns). This was associated with altered gene expression and DNA methylation profiles. A total of 54 differentially expressed genes were identified when comparing CNO/CRMO patients at baseline with healthy controls, including IL17RC. Furthermore, 6 genes were found to be differentially expressed when comparing CNO/CRMO patients at baseline with patients at 6-12 months after treatment with naproxen, including PTGES and CXCR5. Differentially methylated positions (n=1176) were identified in promoter regions comparing monocytes from CNO/CRMO patients with healthy controls. These included genomic regions associated with genes encoding pro- or anti-inflammatory cytokines, or their receptors, including IL10RA and IL10RB, and enrichment of genes associated with TGF-beta activated receptor activity. No DMPs were identified comparing CNO/CRMO patients at baseline with follow-up.

**Conclusion:** Gene expression and DNA methylation signatures in CD14+ monocytes differentiate CNO/CRMO patients from matched healthy controls. As molecular signatures largely do not change in response to treatment with naproxen, they may represent early events in the pathophysiology rather than effects secondary to ongoing inflammation. The exact clinical relevance, and whether these may be exploited for therapeutic purposes need to be validated in further studies.

**Disclosure of Interest**: None declared

### P124. Evaluation of activity and performance using Canada occupational performance measure in children with Familial Mediterrenian Fever

#### S. Y. Cetin^1^, O. Kaya Kara^1^, D. S. Kara^2^, S. Yardım^2^, E. Comak^3^, S. Akman^3^, M. Koyun^3^, G. Kaya Aksoy^3^

##### ^1^Faculty of Health Sciences, Department of Physiotherapy and Rehabilitation, ^2^Institue of Health Sciences, Department of Physiotherapy and Rehabilitation, ^3^Faculty of Medicine, Department of Child Health and Diseases, Akdeniz University, Antalya, Turkey

###### **Correspondence:** S. Y. Cetin

**Introduction:** Familial Mediterranean Fever (FMF) is an autoinflammatory disease that typically begins in childhood and more common among Mediterranean populations. The primary symptoms experienced by the patients are usually fever and pain with inflammatory attacks, which may cause obstacles in the individual’s role and functions in daily living activities. The Canadian Occupational Performance Scale (COPM) is an individualized measure designed to detect a person’s self-reported activity performance in daily life and can be used among children at least aged 8 years. Practicing COPM helps children to identify their own ideas about the supports required in their daily living activities. The activity and performance of children with FMF has not been questioned in the literature.

**Objectives:** The aim of this study was to determine the activity and performance of children with FMF by using COPM.

**Methods:** The study was included 42 children (22 girls, 20 boys between the ages of 8-17 years) with a mean age of 11.14±3.62 years. COPM was used to determine children’s activity and performance. The assessment was carried out face-to-face with the children by a physiotherapist. During this interview, the children identified up to five key activities they would like to tackle first. Then, the children determine the activities they want to do, should do or are expected to do; however, they determined the activities that they could not do or had difficulty and were not satisfied with because of their rheumatic diseases. Children identified activities for 3 different areas of COPM: self-care, productivity, leisure. For each of these activities, they gave performance and satisfaction scores between 1-10 (1 point indicates that he/she cannot do/’not at all satisfied; 10 points indicates that he/she is able/extremely satisfied).

**Results:** Children with FMF determined 20 different activities that they have difficulty in daily living activities. Children with FMF had most difficulties in three activities including running (64.1%), writing (41%), and climbing stairs (33.3%).

**Conclusion:** According to the results of our study, it was observed that children with FMF had difficulties with a wide variety of activities. It can be thought that COPM may be a useful tool to identify activity performance problems, can be used by clinicians in routine evaluation, and may also be useful in determining goals of rehabilitation programs for children with FMF.

References

1- Verkerk GJQ, van der Molen-Meulmeester L, Alsem MW. How children and their parents value using the Canadian Occupational Performance Measure (COPM) with children themselves. J Pediatr Rehabil Med 2021;14 (1):7-17

2- Ben-Chetrit E, Touitou I. Familial Mediterranean fever in the world*.* Arthritis Care Res 2009;61(10):1447–1453

**Patient Consent:** Yes, I received consent

**Disclosure of Interest**: None declared

### P125. Determination of participation in children with Familial Mediterrenian Fever

#### O. Kaya Kara^1^, S. Y. Cetin^1^, S. Karademir^2^, E. Comak^3^, S. Akman^3^, M. Koyun^3^, G. Kaya Aksoy^3^

##### ^1^Faculty of Health Sciences, Department of Physiotherapy and Rehabilitation, ^2^Instıtue of Health Sciences, Department of Physiotherapy and Rehabilitation, ^3^Faculty of Medicine, Department of Child Health and Diseases, Akdeniz University, Antalya, Turkey

###### **Correspondence:** S. Y. Cetin

**Introduction:** Familial Mediterranean Fever (FMF) is a genetic disease characterized by recurrent episodes of fever accompanied by pain in the abdomen or joints. It is most common in individuals of Mediterranean and Middle Eastern descent, and the first attacks typically begin in childhood. In addition, musculoskeletal findings such as arthritis, arthralgia or myalgia can often be seen in patients younger than 18 years of age in children with FMF. The quality of life and psychosocial status of these children may also be adversely affected. These symptoms and negative experiences by children can adversely affect children’s participation of activities in home, school and community settings. The participation of these children has not been evaluated so far and there is no relevant evidence in the literature. Participation and Environment Measure - Children and Youth (PEM-CY) is the most comprehensive measurement based on International Classification of Functioning (ICF) components to evaluate participation of children and adolescents with or without disabilities at aged of 5 to 17 years-old, in home, school, and community settings, alongside environmental factors.

**Objectives:** This aim of this study was determine the participation of children with FMF and compared them with aged matched healthy peers.

**Methods:** The study was included 94 children (57 children with FMF and 37 healthy children between the ages of 7-17) with a mean age of 10.76 ±2.82 years. PEMC-Y was used to assess children’s participation according to ICF. The questions in the scale were answered by the families of children in face-to-face interviews. Student t test and chi-square test was used to compare the groups.

**Results:** When the groups compared in terms of participation at home, a significant difference was found in favor of the healthy group in the involvement, barriers, helpfullness and overall support scores (p:0.00). At school, a significant difference was found in favor of the healthy group in the participation frequency, involvement, barriers, helpfullness and overall support scores (p:0.00). In the community, a significant difference was found in favor of the healthy group in the all of subdomain scores (p:0.00-0.03).

**Conclusion:** According to our study, the participation of children with FMF in home, school and community settings was found to be lower than healthy children. Especially, it was observed that the participation of activities in community setting was lower in every field. The participation of these children should also be taken into account when planning rehabilitation programs and goals, and it should be target to ensure maximum participation at home, school and community settings.

References

1-Kaya Kara O, Turker D, Kara K, Yardimci-Lokmanoglu B. Psychometric properties of the Turkish version of Participation and Environment Measure for Children and Youth. Child Care Health Dev. 2020;46:711–722.

**Trial registration identifying number:**

**Patient Consent:** Yes, I received consent

**Disclosure of Interest**: None declared

### P126. Damaging variants in P2X7R associate with chronic nonbacterial osteomyelitis (CNO)

#### A. Charras^1^, S. R. Hofmann^2^, A. Cox^3^, F. Schulze^2^, S. Russ^2^, H. Hartmann^4^, S. Haldenby^5^, X. Liu^5^, H. Morbach^6^, H. Wittkowski^7^, P. J. Ferguson^3^, C. Hedrich^1,8^

##### ^1^Department of Women’s and Children’s Health, Institute of Life Course and Medical Sciences, University of Liverpool, Liverpool, United Kingdom, ^2^Klinik und Poliklinik für Kinder- und Jugendmedizin, Universitätsklinikum Carl Gustav Carus, TU Dresden, Dresden, Germany, ^3^Stead Family Department of Pediatrics, Carver College of Medicine, University of Iowa, Iowa City, United States, ^4^Centre for Molecular and Cellular Bioengineering, TU Dresden, Dresden, Germany, ^5^Centre for Genomic Research, Institute of Infection, Veterinary & Ecological Sciences, University of Liverpool, Liverpool, United Kingdom, ^6^Pediatric Immunology and Rheumatology, Children’s Hospital, University of Würzburg, Würzburg, ^7^Klinik für Pädiatrische Rheumatologie und Immunologie, Universitätsklinikum Münster, Münster, Germany, ^8^Pediatric Immunology and Rheumatology, Children’s Hospital, University of Würzburg, Liverpool, United Kingdom

###### **Correspondence:** A. Charras

**Introduction:** Chronic Nonbacterial Osteomyelitis (CNO) is an autoinflammatory bone disease primarily affecting children. It can cause pain, hyperostosis and fractures, thereby affecting quality-of-life and psychomotor development. While increased inflammasome activation has been reported, the exact molecular pathophysiology of CNO is unknown.

**Objectives:** To identify disease mechanisms in CNO to allow patient stratification and individualized treatment.

**Methods:** Whole exome sequencing in families with CNO, and target sequencing of *P2X7R* in a large German CNO cohort. Findings were integrated with demographic and clinical datasets. Genetically modified THP-1 cells were used to investigate altered potassium flux, inflammasome assembly (ASC specks), cytokine release and pyroptosis.

**Results:** In 8 families with a history of CNO in 2 generations, damaging mutations in *P2X7R*, a regulator of inflammasome assembly, were identified. Targeted sequencing in 196 unrelated patients identified rare damaging heterozygous variants in 5. In the remaining cohort, common variants were over-represented when compared to the healthy population. Patients with rare variants were younger, more frequently exhibited multifocal disease, and required more aggressive treatment with 2^nd^-line agents when compared to the remaining cohort. THP-1 cells expressing variant P2X7 exhibited altered potassium flux, inflammasome assembly, IL-1 and IL-18 release, and pyroptosis.

**Conclusion:** Rare damaging variants in *P2X7R* account for approximately 2.5% of CNO cases. Common *P2X7R* variants were present in all remaining (97.5%) patients and may represent a risk allele underscoring the key role of inflammasome dysregulation in CNO. Observations will aid in future patient stratification and individualized care.

**Patient Consent:** Yes, I received consent

**Disclosure of Interest**: None declared

### P127. Type 1 interferon pathway and Aicardi-Goutieres syndrome: a case report

#### A. Cilona, R. Asaro, C. Martorana, C. Alizzi, M. C. Maggio, G. Corsello

##### A.O.U.P. Paolo Giaccone- Università degli studi di Palermo, Palermo, Italy

###### **Correspondence:** A. Cilona

**Introduction:** Interferonopathies are a new class of Mendelian inherited disorders, belonging to the group of systemic autoinflammatory diseases(SADs); they are characterized by a constitutive yet anomalous activation of the type I interferon pathway (IFN I). They are clinically heterogeneous; the first identified interferonopathy, the Aicardi-Goutieres syndrome (AGS) , mainly determines neurological and cutaneous involvement. At the beginning, AGS was originally defined as pseudo-TORCH syndrome, identifying a group of serologically negative disorders that mimic congenital TORCH infections, suggesting a similar pathogenetic mechanism. The underlying genetic mutations induce an accumulation of DNA and RNA fragments, generated during the genomic repair process, which act as a trigger for the production of IFN I. Other pathologies, such as systemic lupus erythematosus (SLE) have in common with the interferonopathies the overproduction of IFN and skin involvement. It has been suggested that the accumulation of nucleic acids may lead to the same pathways involved in interferonopathies, with over-production of IFN, suggesting an overlap between interferonopathies and SLE. Recent evidence suggests that interferonopathies should be considered in differential diagnosis in cases of early onset or atypical presentation of rheumatological diseases in the pediatric setting, especially if lipodystrophy, lupus pernio, vascular disease and arthralgia are found among the signs of the disease.

**Objectives:** Our objective is to report a case which shows a possible correlation between a specific mutation and a well known autoimmune pathology such as the Aicardi-Goutieres.

**Methods:** F., 16 years old, comes to our observation complaining of arthralgia, nodular dermatitis affecting lower limbs and Her laboratory evaluation showed positivity of ANA, hypocomplementemia and proteinuria. Adaptive SLE was initially suspected, and the patient was treated accordingly with hydroxychloroquine and corticosteroids, and a rheumatological follow-up was initiated. Despite therapy, there was no regression of the vasculitic lesions, nor normalization of the values ​​of complementemia and proteinuria. The patient was therefore subjected genetic investigation with NGS technique for Interferonopathies.

**Results:** Despite therapy, there was no regression of the vasculitic lesions, nor normalization of the values ​​of complementemia and proteinuria. The patient was therefore subjected genetic investigation with NGS technique for Interferonopathies.

**Conclusion:** The variant c.2893G> A, found in the patient and in her father, is currently classified as a variant of uncertain significance (VUS). However, considering that literature reports cases of interferonopathies with vasculitis as only clinical presentation, and that genetic correlation is subject to periodic review, to this day an univocal diagnosis has not been made yet the patient. We hope that further observations may help us to define her diagnosis more precisely.

**Patient Consent:** Yes, I received consent

**Disclosure of Interest**: None declared

### P128. Effect of the janus kinase inhibitor baricitinib in the treatment of COPA syndrome

#### F. Corona^1^, C. Matucci-Cerinic^2^, S. Pastore^3^, P. Bocca^4^, A. Ravelli^4^, R. Caorsi^4,5^, M. Gattorno^4,5^, S. Volpi^2,4,5^, A. Tommasini^1,3^

##### ^1^Università degli Studi di Trieste, Trieste, ^2^DINOGMI, Università degli Studi di Genova, Genova, ^3^IRCCS Institute Burlo Garofolo, Trieste, ^4^Center for Autoinflammatory diseases, IRCCS Institute Gaslini, ^5^Clinica pediatrica e Reumatologia, IRCCS Istituto Gaslini, Genova, Italy

###### **Correspondence:** C. Matucci-Cerinic

**Introduction:** COPA syndrome is a rare primary immunodeficiency, characterized by features of autoinflammation, immune dysregulation and autoimmunity caused by heterozygous mutations with incomplete penetrance of the *COPA* gene. Clinically, the disease is characterized by lung, kidney and joint involvement. No therapeutic guidelines exist for the treatment of COPA syndrome, even if the use of JAK-inhibitors has been reported in some patients.

**Objectives:** to evaluate the efficacy of the JAK 1/2 inhibitor baricitinib in controlling and preventing disease progression in paediatric COPA patients.

**Methods:** the data of 3 COPA patients treated with Baricitinib were retrospectively reviewed. Clinical, serological, immunological and radiological data were collected.

**Results:** all patients were females and had a disease onset before 3 years of age. One patient (P1) carried the p.Arg233His mutation, while the 2 other unrelated patients (P2 and P3) carried the p.Arg281Trp mutation. P1 and P2 mother’s were asymptomatic carriers. P2 has a brother and a sister affected: the brother has an articular and pulmonary involvement, the sister died at 23 years of heart failure. P3’s mother is actually diagnosed with Still disease but no COPA mutation was found in the family. Clinically, all patients presented with arthritis, which was deforming in 2 cases (P1 and P2), and with pulmonary involvement, characterized by interstitial lung disease and by multiple pulmonary cysts on the CT scan. P1 and P3 presented also ground glass opacities and bronchiectasis. No pulmonary haemorrhages were reported. All patients have a normal renal function. P3 has congenital bilateral renal dysplasia. At diagnosis, spirometry in P1 showed a severe reduction of the FVC and of the DLCO, the 6minute walking test (6MWT) was pathologic with a minimal SaO2 of 85%. Spirometry and 6MWT were not performed in the other 2 patients because of the young age. All the patients presented a positive IFN signature at disease onset and were ANA and rheumatoid factor positive. P1 had also positive anti-citrullinated peptide antibodies. P1 had a disease onset 7 years before the first description of COPA disease and underwent several immunosuppressive and biologic therapies before the diagnosis (oral and intraarticular steroids, abatacept, methotrexate, mofetil mycophenolate MMF, rituximab). P2 was diagnosed one year after the disease onset and was previously treated with steroids, MMF and hydroxychloroquine. P3 was started on baricitinib at disease onset. After baricitinib was started, in all the patients a reduction of the inflammatory biomarkers was evident and steroids could be stopped in P1 and P2 (while never used in P3). In P1 a dramatic improvement of the 6MWT was achieved (minimal SaO2 during the test at last f-up 96%), CT scan and spirometry showed no disease progression over the years, and no articular injections were needed in the last 36 months (1-2 intra-articular injections/year before baricitinib start). Both P1 and P2 have a current follow-up of 36 months, P3 of 3 months on baricitinib and didn’t experience any disease relapse since the start of the therapy.

**Conclusion:** baricitinib seems to block lung disease progression in medium term follow-up, controlling arthritis and reducing systemic inflammation in COPA disease. Larger cohorts of patients are needed to confirm these results.

**Disclosure of Interest**: None declared

### P129. Characterization of AR-CGD female patient with novel homozygous deletion in CYBC1 gene presenting with unusual clinical phenotype

#### A. De Matteis^1^, M. F. Natale^1^, M. Chiriaco^2^, C. Cifaldi^3^, S. Di Cesare^2,3^, C. Passarelli^4^, A. Novelli^4^, G. Di Matteo^2,3^, A. Finocchi^2,3^, F. De Benedetti^5^, A. Insalaco^5^

##### ^1^Division of Rheumatology, ERN-RITA center, IRCCS Ospedale Pediatrico Bambino Gesù, Roma, Italy, ^2^Department of Systems Medicine, University of Rome Tor Vergata, ^3^Academic Department of Paediatrics, Immune- Infectivology Unit, ^4^Translational Cytogenomics Research Unit, ^5^Division of Rheumatology, ERN-RITA center, IRCCS Ospedale Pediatrico Bambino Gesù, Roma, Italy

###### **Correspondence:** M. F. Natale

**Introduction:** Mutations in EROS/CYBC1 gene cause a rare form of autosomal recessive (AR) chronic granulomatous disease (CGD), characterized by severe life-threatening infections, hyperinflammation and immune-dysregulation.

**Objectives:** We described an AR-CGD patient with a novel homozygous deletion in CYBC1 gene leading to absent EROS/CYBC1 protein and presenting with a clinical phenotype almost exclusively characterized by inflammatory manifestations.

**Methods:** Whole Exome Sequencing (WES), Flow Cytometry Studies (phenotype, DHR assay, protein expression), Western Blot (protein expression

**Results:** The clinical history of a previously well-being two years old female child was predominantly characterized by autoinflammatory phenotype. At onset she presented with recurrent episodes of fever of unknown origin responsive to glucocorticoids. Subsequently she developed inflammatory bowel disease treated with mesalazine. NBT test was negative. At the age of 9 years old she presented with pancytopenia, hepatosplenomegaly, bowel inflammation and granulomatous lung disease. Laboratory tests showedincreased levels of chitotriosidase (> 10 times normal value) and angiotensin converting enzyme (> 2 times normal value). In the suspicion of sarcoidosis immunosuppressive treatment with high dose of glucocorticoids, methotrexate and adalimumab was started. WES identified a novel homozygous deletion c. 8_7del CTCTCGGGATGTACC in the CYBC1 gene leading to absent CYBC1/EROS protein in PBMC and EBVB cells. Patient’s parents, heterozygous for the mutation, expressed about half of the protein. The gp91phox protein expression/function was impaired in patient’s neutrophils and monocytes (about 50%), but severely compromised in B cells (gp91phox<15%; DHR+ < 4%).

**Conclusion:** This patient’s case emphasize the importance to consider a diagnosis of CGD even in absence of classical clinical presentation and negative NBT test.

**Patient Consent:** Yes, I received consent

**Disclosure of Interest**: None declared

### P130. The preferential use of anti-interleukin 1 therapies in various pediatric rheumatic disorders: outcomes of treat-to-target approach

#### F. G. Demİrkan^1^, Ö. Akgün^2^, V. Guliyeva^2^, N. Aktay Ayaz^1^

##### ^1^Pediatric Rheumatology, ^2^İstanbul School of Medicine, İstanbul, Turkey

###### **Correspondence:** F. G. Demİrkan

**Introduction:** Interleukin-1 inhibitors are proven treatment options for autoinflammatory disorders. Anakinra and canakinumab are the most commonly used anti-interleukin 1 agents in the field of pediatric rheumatology.

**Objectives:** The aim of this study was to evaluate the broader effectiveness and safety of anakinra and canakinumab in a ‘real world’ pediatric population.

**Methods:** Patients with colchicine-resistant familial Mediterranean fever (crFMF), systemic juvenile idiopathic arthritis (sJIA), or polyarticular JIA treated with anakinra and canakinumab in any order were identified using the database of İstanbul Faculty of Medicine between January 2020-May 2022.Background characteristics of the patients, reason for switching to IL-1 inhibitor, and the side effects observed during the treatment were extracted form the patient files recorded at every 3 month visits.

**Results:** Forty-eight pediatric patients(65%female) were enrolled in this study. The median age was 11 years (4-17 years), and the median disease duration was 37 months (14-59 months). The median (IQR) duration of treatment with anti-IL-1 agents was 16 (12-36) months. Forty-three patients were treated with canakinumab (150 mg/4 week) and 48 patients with anakinra (100 mg/day).Six (12.5%) patients receiving anakinra were diagnosed with sJIA, 1(2%) with polyarticular JIA and 41(85.4%) with crFMF in, and . In the canakinumab group diagnoses were sJIA in 2 (4.6%) and crFMF in 41 (95.3%) patients. The patients with crFMF were first treated with colchicine alone for a median duration of 12.5(8-38) months. Canakinumab was prescribed for crFMF after previous anakinra treatment, whereas no patients who switched treatment from canakinumab to anakinra were identified. According to the current guidelines for the treatment of FMF, colchicine was continued along with the IL-1 inhibitors. The autoinflammatory diseases activity and attacks decreased with both anakinra and canakinumab. However, 18/41(43.9%) patients with crFMF discontinued anakinra due to inadequate response (4 of them with secondary failure after a good initial response) and switched to canakinumab. In 9(21.9%) children anakinra was switched to canakinumab due to patient preference regarding refuse of daily injection. Frequency of flares and the median duration of flares were significantly decreased following switching to canakinumab from anakinra treatment (p<0.01). Anakinra was effective in decreasing proteinuria and canakinumab was also successful in decreasing proteinuria in anakinra unresponsive patients. The modified FMF score was achieved in 76.2% of anakinra and 88.9% of canakinumab group. Anakinra could be stopped due to complete remission in 4 children with sJIA. However, in 2/6 patients, anakinra was switched to canakinumab due to resistant disease course. Injection site reactions (ISRs, n:13) was the most common reason for the discontinuation of anakinra and most of ISRs developed in the first month of treatment. Two severe skin rashes and one significant elevation of transaminases were observed with anakinra. No severe side effects or side effect-related discontinuation of canakinumab was observed. No serious infections were detected in both anakinra and canakinumab group.

**Conclusion:** While canakinumab, a human monoclonal anti-IL-1 beta antibody, for the convenience of its use, became the preferred IL-1 blocker in FMF, anakinra has its own benefits in certain circumstances of FMF. Canakinumab had a favorable safety/tolerability profile. Anakinra is also generally safe with side effects that may be observed in the short and long-term use should be taken into account. Large series and extended follow-up studies are required for establishing the long-term effects of these treatments.

**Patient Consent:** Yes, I received consent

**Disclosure of Interest**: None declared

### P131. Predict-CRFMF score: a novel model for predicting colchicine resistance in children with Familial Mediterranean Fever

#### N. Aktay Ayaz^1^, F. G. Demİrkan^1^, T. Coşkuner^2^, F. Demir^3^, A. Tanatar^1^, M. Çakan^2^, S. G. Karadağ^4^, G. Otar Yener^5^, K. Öztürk^6^, E. Bağlan^7^, F. Çakmak^1^, S. Çağlayan^2^, S. Özdel^7^, K. Ulu^2^, B. Sözeri^2^, H. E. Sönmez^8^

##### ^1^Pediatric Rheumatology, İstanbul School of Medicine, ^2^Pediatric Rheumatology, Umraniye Research and Training Hospital, ^3^Pediatric Rheumatology, Acıbadem Healthcare Group, ^4^Pediatric Rheumatology, Çam ve Sakura City Hospital, İstanbul, ^5^Pediatric Rheumatology, Sanliurfa Mehmet Akif Inan Training and Research Hospital, Sanlıurfa, ^6^Pediatric Rheumatology, Istanbul Medeniyet University, School of Medicine, Goztepe Research and Training Hospital, İstanbul, ^7^Pediatric Rheumatology, Dr. Sami Ulus Obstetrics and Gynecology, Pediatric Health and Disease Training and Research Hospital, Ankara, ^8^Pediatric Rheumatology, Kocaeli School of Medicine, Kocaeli, Turkey

###### **Correspondence:** F. G. Demİrkan

**Introduction:** As none of the previous scoring systems have a power of estimating the refractory disease course at the initiation of the colchicine treatment in familial Mediterranean fever (FMF) cases, a predictive model appear to be beneficial for achieving early control of disease in colchicine resistant FMF (cr-FMF) patients.

**Objectives:** We intended to develop a novel scoring system based on the initial clinical features and laboratory findings for predicting colchicine resistance in FMF, thus providing a reliable and easy tool for pediatric rheumatologists while evaluating patients at diagnosis.

**Methods:** The medical records including baseline clinical and laboratory findings of patients prior to initiation of colchicine were analyzed. After generating a predictive score in the initial cohort, it was applied to an independent cohort for external validation of effectiveness and reliability.

**Results:** Between June 2020 and December 2021, 1464 patients were admitted to the pediatric rheumatology outpatient clinic with the diagnosis of FMF. Forty-six patients were excluded from the analysis due to short follow-up period (less than 6 months). Among 1418 patients with FMF, 56 (3.9%) were colchicine resistant (cr) and 1312 (96.1%) were colchicine responsive. According to the logistic regression analysis, *recurrent arthritis (4-points), protracted febrile myalgia (8-points), presence of ELE (2-points), exertional leg pain (2-points), and carrying M694V homozygous mutation (4-points)* were determined as the scoring parameters for predicting patients with cr-FMF. Scores were assigned according to β coefficients in the final model. (Table 1)

**Conclusion:** Intolerance/resistance in a definite portion of patients with FMF is posing a dilemma for pediatric rheumatologists and uncontrolled inflammation resulting in amyloid deposition in organ systems is detrimental for the patients as well. By constructing this novel reliable predictor tool, we enunciate that predicting colchicine resistance in children with FMF at the initiation of the disease and interfering timely before the emergence of complications during the disease course will be possible.

**Patient Consent:** Yes, I received consent

**Disclosure of Interest**: None declared

**Table 1 (abstract P131).** See text for description

Predictors	Odds Ratio (95% CI)	P value	Score assigned
Recurrent arthritis	3.6 (1.77-7.61)	<0.001	4
Protracted febrile myalgia	8.4 (3.41-31.85)	<0.001	8
Erysipelas-like erythema	1.74 (0.86-3.51)	0.02	2
Exertional leg pain	2.1 (1.16-3.62)	0.01	2
Carrying M694V homozygous mutation	4.2 (2.2-8.06)	<0.001	4

The cut-off value (9) was 87% sensitive and 82% specific to foresee the risk of colchicine resistance in the ROC

Validation of the scoring system with an independent group (cr-FMF=107, colchicine responsive= 1935) revealed that the cut-off value was 82% sensitive and 79% specific to identify the risk of colchicine resistance. Seventy-nine (73.8%) cr-FMF patients and 137 (7.1%) colchicine responsive patients had a total score of more than 9

### P132. Can the diagnosis of PFAPA syndrome be confirmed? data from the long-term follow-up of a single-centre cohort

#### A. Doležalová, P. Šeferna, A. Zahornadský, Š. Fingerhutová, P. Doležalová

##### Department of Paediatrics and Inherited Metabolic Disorders, General University Hospital in Prague, Prague, Czech Republic

###### **Correspondence:** A. Doležalová

**Introduction:** Diagnosis of Periodic Fever, Aphtae, Pharyngitis and Adenitis syndrome (PFAPA) is based on its typical clinical picture and exclusion of other conditions. It has been postulated that it is a self-limited disease with spontaneous remission occurring by puberty in majority of patients.

**Objectives:** We aimed at supporting the presumption that diagnosis of PFAPA based on the combination of systematic clinical evaluation using modified diagnostic criteria (1) and standardized follow-up is ultimately confirmed only by its complete resolution.

**Methods:** A structured phone call interview was carried out during May 2022 with patients born between 1999-2008 from our PFAPA cohort described in 2013 (2) that have not been seen at our fever clinic for more than 5 years. Questions covered current health status including existence of any unexplained periodic symptoms, date of the last typical PFAPA episode, tonsillectomy (TE) date. In combination with the hospital electronic record the disease characteristics were derived.

**Results:** During the period of 2004-2011 diagnosis of PFAPA syndrome was confirmed in 125 children (all Czech origin Caucasians) with the median age at onset of 23 months (2). In 60 patients who answered the call the current age was 16.1 (SD 2.3) years (response rate 48%). Mean follow-up (F/U) duration (from the 1^st^ visit until the call) was 12.7 (SD 1.7) years. In 3/60 (5%) patients recurrence of typical PFAPA was reported after the prolonged (>1year) asymptomatic interval before they entered sustained remission. Only 4/60 (6.7%) patients reported ongoing periodic problems different from the original PFAPA episodes. In 56 patients in remission (93.3%) the total disease duration (from the first to the last reported typical PFAPA episode) was 4.1(SD 2.8) years with the mean age of the last PFAPA episode of 5.9(SD 3) years. Mean asymptomatic interval (from the last reported episode until the interview) was 9.5 years (SD 2.9). 16/60(26.7%) patients reported TE at mean age of 4.8 (SD 2.1) years that was followed by full resolution of PFAPA in 14 cases (87.5%). In one patient there was a short recurrence of typical episodes followed by remission, in the other one typical PFAPA ended post-TE but short febrile episodes with no other symptoms have been recuring until now in 3-monthly intervals.

**Conclusion:** Using a diagnostic approach described previously (2) the original diagnosis of PFAPA was confirmed by its longstanding remission (>9 years) in the majority (93.3%) of cases with symptom resolution occurring during the early school years. TE was a potent remission inductor in almost 90% of cases. Recurrence of symptoms after prolonged afebrile interval was uncommon. Persistence of periodic complaints in 4 patients discovered by the interview will lead to the clinical and genetic re-evaluation of their diagnosis.

References:

1. Hofer M, Cochard M, Anton J *et al*.: PFAPA (periodic fever, oral aphtae, pharyngitis and cervical adenitis) syndrome: a new consensus on diagnostic criteria. *Ann Rheum Dis* 2009; 68 (Suppl.3): 705

2. Król P, Böhm M, Sula V, *et al*.: PFAPA syndrome: clinical characteristics and treatment outcomes in a large single-centre cohort. *Clin Exp Rheumatol* 2013;31(6):980-987

**Trial registration identifying number:**

**Patient Consent:** Yes, I received consent

**Disclosure of Interest**: None declared

### P133. Long-term efficacy and safety of canakinumab in patients with Familial Mediterranean Fever (FMF) - interim analysis of the reliance registry

#### F. Dressler^1^, J. Henes^2^, N. Blank^3^, T. Krickau^4^, T. Kallinich^5^, G. Horneff^6^, F. M. Meier^7,8^, I. Foeldvari^9^, F. Weller-Heinemann^10^, B. Kortus-Goetze^11^, M. Hufnagel^12^, J. Rech^13^, P. T. Oommen^14^, J. Weber-Arden^15^, J. B. Kuemmerle-Deschner^16^

##### ^1^Division of Pediatric Pneumology, Allergology and Neonatology, Hannover Medical School, Hannover, ^2^Center of Interdisciplinary Rheumatology, Immunology and autoimmune diseases (INDIRA), University Hospital Tuebingen, Tuebingen, ^3^Rheumatology, University Hospital Heidelberg, Heidelberg, ^4^Pediatrics, Friedrich-Alexander Universität Erlangen-Nürnberg (FAU), Erlangen, ^5^Department of Pediatrics, Division of Pulmonology, Immunology and Critical Care Medicine, Charité University Medicine Berlin, Berlin, ^6^Asklepios Clinic Sankt Augustin, Sankt Augustin, ^7^Project Group Translational Medicine and Pharmacology TMP, Fraunhofer Institute for Molecular Biology and Applied Ecology IME, ^8^Division of Rheumatology, University Hospital Frankfurt, Goethe University, Frankfurt am Main, ^9^Centre for Pediatric and Adolescence Rheumatology Hamburg, Hamburg, ^10^Prof. Hess Kinderklinik, Klinikum Bremen-Mitte, Bremen, ^11^Division of Nephrology, University of Marburg, Marburg, ^12^Division of Pediatric Infectious Diseases and Rheumatology, Department of Pediatrics and Adolescent Medicine, University Hospital Medical Center Freiburg, Medical Faculty, University of Freiburg, Freiburg, ^13^Department of Internal Medicine 3 - Rheumatology and Immunology, Friedrich-Alexander University (FAU) Erlangen-Nuernberg and Universitaetsklinikum Erlangen, Erlangen, ^14^Clinic of Pediatric Hematology, Oncology and Clinical Immunology, Heinrich-Heine-University Duesseldorf, Duesseldorf, ^15^NOVARTIS, Nuernberg, ^16^Department of Pediatrics, Division of Pediatric Rheumatology, University Hospital Tuebingen, Tuebingen, Germany

###### **Correspondence:** F. Dressler

**Introduction:** Familial Mediterranean fever (FMF) is a chronic disease characterized by recurrent episodes of fever and serositis, with a risk of severe complications (e. g. amyloidosis). Treatment of FMF according to EULAR recommendations aims to control acute relapses and subclinical inflammation and improve patients’ quality of life.

**Objectives:** The present study investigates the long-term efficacy and safety of CAN in routine clinical practice in pediatric (age ≥2 years) and adult FMF patients.

**Methods:** RELIANCE is a prospective, non-interventional, multicenter observational study in Germany with a follow-up period of up to seven years. Patients with a clinically confirmed diagnosis of FMF who routinely receive CAN will be enrolled in the study. Disease parameters will be recorded at the time of study inclusion and six-month intervals.

[1]AIDAI: *Auto-Inflammatory Diseases Activity Index*

**Results:**

This interim analysis of FMF patients (N=74) enrolled through December 2021 includes data from baseline through the 24-month visit. The mean age in this cohort was 25 years (2-61 years). The proportion of female patients was 51% (N=38). At baseline, the median duration of prior CAN treatment was 1.0 years (0-6 years). At month 24, approximately 63% of patients were in disease remission by physician assessment, and 67% of patients documented inactive disease in the AIDAI[1] score (Table 1).

Mutations were documented in a total of N=57 patients, including M694V (9 homozygous, 10 compound heterozygous, 12 heterozygous), V726A (1 hom., 6 comp. het., 1 het.), and M680I (1 hom., 6 comp. het., 1 het.).

At baseline, N=16 FMF patients were CAN-naive and received a starting dose of median 150 mg CAN (40 mg/kg - 150 mg every 4 weeks). In this subgroup, a total of 12 dose adjustments were made over time (9 dose increases, 3 dose reductions).

A total of 18 SAE[2] were reported, of which 2 (tonsillectomy and tachycardia) were classified as drug-related.

[1]AIDAI: *Auto-Inflammatory Diseases Activity Index*

[2]SAE: Serious Adverse Event

[1]SAE: Serious Adverse Event

**Conclusion:** Interim data from FMF patients in the RELIANCE study, the longest running canakinumab registry, confirm the efficacy and safety of long-term treatment with canakinumab.

**Disclosure of Interest**: F. Dressler Grant / Research Support with: Novartis, Consultant with: Abbvie, Mylan, Novartis, Pfizer, J. Henes Grant / Research Support with: Novartis, Roche, Consultant with: Novartis, AbbVie, Sobi, Roche, Janssen, Boehringer-Ingelheim, N. Blank Grant / Research Support with: Novartis, Sobi, Consultant with: Novartis, Sobi, Lilly, Pfizer, Abbvie, BMS, MSD, Actelion, UCB, Boehringer-Ingelheim, Roche, T. Krickau Grant / Research Support with: Novartis, Consultant with: Novartis, Speaker Bureau with: Novartis, T. Kallinich Speaker Bureau with: Roche, G. Horneff Grant / Research Support with: AbbVie, Chugai, Merck Sharp & Dohme, Novartis, Pfizer, Roche, Speaker Bureau with: AbbVie, Bayer, Chugai, Merck Sharp & Dohme, Novartis, Pfizer, Roche, F. M. Meier Speaker Bureau with: Novartis, I. Foeldvari Consultant with: Novartis, F. Weller-Heinemann: None declared, B. Kortus-Goetze Consultant with: Novartis, M. Hufnagel Grant / Research Support with: Novartis, J. Rech Grant / Research Support with: Novartis, Sobi, Consultant with: AbbVie, Biogen, BMS, Chugai, GSK, Janssen, Lilly, MSD; Mylan, Novartis, Roche, Sanofi, Sobi, UCB, Speaker Bureau with: AbbVie, Biogen, BMS, Chugai, GSK, Janssen, Lilly, MSD; Mylan, Novartis, Roche, Sanofi, Sobi, UCB, P. T. Oommen Grant / Research Support with: Novartis, J. Weber-Arden Employee with: Novartis, J. B. Kuemmerle-Deschner Grant / Research Support with: Novartis, AbbVie, Sobi, Consultant with: Novartis, AbbVie, Sobi

**Table 1 (abstract P133).** Evaluation of clinical disease activity and safety aspects of patients with FMF

Efficacy parameters (total FMF cohort)	Baseline	12 months	24 months
Number of patients, N	74	46	24
Number (%) of patients in disease remission (physician rating)	22 (45)	23 (72)	12 (63)
Patient assessment of current disease activity; 0–10, median (min; max)	2.0 (0; 10)	2.0 (0; 7)	2.0 (0; 10)
Patient assessment of current fatigue; 0–10, median (min; max)	5.0 (0; 10)	2.0 (0; 10)	4.0 (0; 10)
AIDAI score <9 ≙ inactive disease, N (%)	12 (66.7)	14 (58.3)	8 (66.7)
CRP / SAA, median (mg/dl)	0.2 / 0.7	0.2 / 0.5	0.2 / 0.7
**SAE (total FMF cohort)**	**Number of events**	**Incidence rate per 100 patient years**	
All types of SAE	18	14.03	
SADR	2	1.56	

CRP, c-reactive protein; SAA, serum amyloid A; SADR, serious adverse drug reaction; SAE, serious adverse event

### P134. Impact of coronavirus outbreak restrictions on behaviors of patients with Familial Mediterranean Fever

#### S. Ertem, Z. B. Ozcakar, F. Aydin, N. Cakar, F. F. Yalcinkaya

##### Pediatric Rheumatology, Ankara University, School of Medicine, Ankara, Turkey

###### **Correspondence:** S. Ertem

**Introduction:** Familial Mediterranean fever (FMF) is the most common systemic autoinflammatory disease characterised by recurrent, self-limiting attacks of fever and serositis. The pandemic caused by novel coronavirus-2 (SARS-CoV-2), known as coronavirus disease 2019 (COVID-19), is a global public health problem leading to significant mortality and morbidity worldwide. To prevent the spread of COVID-19, state and local governments enacted numerous restrictions on human movement and physical interactions. The effect of COVID-19 pandemic and the implemented restrictions on the frequency of attacks and the course of pediatric FMF remains unknown.

**Objectives:** The aim of this study was to investigate the impact of COVID-19 restrictions on clinical status of FMF patients.

**Methods:** We have enrolled patients with FMF, diagnosed according to Turkish pediatric FMF criteria, who were admitted to outpatient clinic of pediatric rheumatology department between June 2021 and December 2021.

Medical records of the patients were evaluated retrospectively. Demographic data, family history, genetic results, clinical findings before and after FMF diagnosis, and the treatment modalities were recorded. Patients were also questioned about the pandemic days, information about school attendance, clinical findings of FMF, frequency of respiratory infections, colchicine dosages were noted. The clinical and laboratory findings of the patients before and during the pandemic were compared. The schools were closed for three semesters and children received online education during this period.

Parameters were expressed as mean ± standard deviation (SD), median [interquartile range (IQR)], and number (percentage). The Cochran Q test was used to compare categorical variables between dependent groups. McNemar test was used to determine which of the categorical variables caused the difference between the three groups (post-hoc analysis), and Bonferroni corrected alpha values were presented. A p<0.05 was considered to be significant.

**Results:** A total of 201 patients ( 97 male, 48% ) with a median age of 13 years ( 2-19 years ) and median follow up of 6 years (3-8.5 years) were included. Major clinical findings of FMF including abdominal pain, fever, arthritis, artralgia, leg pain, heel pain, and frequency of FMF attacks were significantly reduced in pandemic era compared with prepandemic era (Table 1). Moreover, the frequency of upper respiratory tract infections were also reduced during pandemic period. Attack free white blood cell (WBC) count and sedimentation rate levels were significantly decreased in pandemic era, compared to prepandemic era (p<0.05).

**Conclusion:** Attacks of FMF can be triggered by various factors including infections, lack of sleep or stress. During COVID-19 restrictions, patients with FMF had reduced attack frequency in childhood period. Together with online education; the rate of respiratory tract infections decreased, possibly resulting in decreased disease activity. This showed us once again the role of environmental factors in autoinflammatory disease activity.

**Patient Consent:** Yes, I received consent

**Disclosure of Interest**: None declared

**Table 1 (abstract P134).** Comparison of clinical findings before FMF diagnosis, after FMF diagnosis before pandemic (after treatment), during pandemic

Characteristics	Before FMF diagnosis (n=201)	After FMF diagnosis before pandemic (after treatment), (n=201)	During pandemic (n=201)	P value
Abdominal pain	169 (%84)	136 (%68)	80 (%40)	<.001
Fever	168 (%84)	117 (%58)	65 (%32)	<.001
Arthritis	51 (%25)	44 (%22)	29 (%14)	.001
Arthralgia	112 (%56)	96 (%48)	57 (%28)	<.001
Leg pain	93 (%46)	91 (%45)	56 (%28)	<.001
Heel pain	40 (%20)	40 (%20)	21 (%10)	<.001
Number of attacks / year	15 (6-27)	2 (0-2)	0 (0-2)	<.001
Frequency of RID (n,min-max)	2 (2-3)	2(2-3)	0 (0-1)	<.001

Abbreviations: FMF, Familial Mediterranean Fever; RID,Respiratory infectious disease

### P135. Blood levels of interleukin-18 (IL-18) in patients with systemic juvenile arthritis and monogenic autoinflammatory diseases (FMF, CAPS, TRAPS)

#### E. S. Fedorov^1^, S. S. Salugina^1^, M. Kaleda^1^, M. Cherkasova^2^, Z. Kolkhidova^1^

##### ^1^Pediatrics, ^2^1 Immunology and Molecular Biology of Rheumatic Diseases, V.A. Nasonova Research Institute of Rheumatology, Moscow, Russian Federation

###### **Correspondence:** E. S. Fedorov

**Introduction:** Interleukin1 plays a leading role in the pathogenesis of autoinflammatory diseases (AIDs), to which systemic juvenile arthritis (sJA) also belongs.

**Objectives:** IL 18 is a member of the interleukin-1 superfamily with a range of special properties; that is why it is of interest to estimate its level in patients with monogenic and polygenic AIDs.

**Methods:** 147 patients with sJA and monogenic AIDs (FMF, CAPS, TRAPS) participated in the study. The diagnosis in all patients with monogenic AIDs was confirmed basing on detection of pathogenic alleles of corresponding genes. IL18 was detected in blood serum through ELISA method using Invitrogen kits (Bender MedSystems GmbH, Austria): reference parameter 0-732.7 ng/ml. Statistical processing was performed using the program “Jamovi”. The reliability of differences between the FMF, CAPS, TRAPS and JA groups was assessed under the Mann-Whitney criterion.

**Results:** The study including 61 patients with sJA: male/female 23/38; age of inclusion into the study 3-19 years; of them ≥ 18 years - 2 (3%), FMF 40 patients; male/female 22/18; age 3-37 years, of them ≥ 18 years - 8 (20%), CAPS 30 patients; male/female 19/11; age 1-51 years, of which ≥ 18 years - 13 (43%), TRAPS 16 patients; age 4-38 years, of which ≥ 18 years - 4 (25%). In the group of patients with sJA, the Median (Me) of IL18 concentration amounted to 4 245.51 ng/ml, 1st quartile 1 136.94 ng/ml, 3rd quartile 4 324.6 ng/ml, interquartile range (IQR) 3,187.6 ng/ml. In patients with FMF Me 657.08 ng/ml, 1st quartile 180.96 ng/ml, 3rd quartile 1 815.51 ng/ml, IQR 1 634.55 ng/ml. In patients with CAPS Me 207.6 ng/ml, 1st quartile 89.0 ng/ml, 3rd quartile 291.0 ng/ml, IQR 202.0 ng/ml. In patients with TRAPS Me 338.6 ng/ml, 1st quartile 159.04 ng/ml, 3rd quartile 1 924.08 ng/ml, IQR 1 765.4 ng/ml. Differences between patients with sJA and patients with FMF, CAPS and TRAPS were reliable: sJA - FMF, sJA - CAPS p<0.001; sJA – TRAPS p<0.009.

**Conclusion:** The maximum concentrations of IL18 are observed in patients with multifactorial AID - sJA, which exceed the concentrations in patients with monogenic AIDs (FMF, CAPS, TRAPS). This fact may explain the greater predisposition to development of macrophage activation syndrome in patients with sJA compared to the above-stated monogenic AIDs. Among patients with monogenic AIDs, the maximum concentrations of IL18 are in patients with FMF, and the minimum concentrations - in patients with CAPS.

**Patient Consent:** Not applicable (there are no patient data)

**Disclosure of Interest**: None declared

### P136. Renal involvement and amyloidosis in autoinflammatory diseases: data from the Eurofever registry

#### Š. Fingerhutová^1^, H. Lachmann^2^, E. Papadopoulou-Alataki^3^, J. Frenkel^4^, L. Cantarini^5^, L. Obici^6^, G. Fabio^7^, G. Fabio^7^, I. Koné-Paut^8^, G. Amaryan^9^, W. Armbrust^10^, E. Hoppenreijs^11^, J. Kuemmerle-Deschner^12^, E. Moreno^13^, M. Alessio^14^, N. Ruperto^15^, M. Gattorno^16^, P. Dolezalova^1^ on behalf of for the Paediatric Rheumatology International Trials Organisation (PRINTO) and the Eurofever Registry

##### ^1^General University Hospital in Prague, Centre for Paediatric Rheumatology and Autoinflammatory Diseases, Prague, Czech Republic, ^2^Royal Free Campus, National Amyloidosis Centre, London, United Kingdom, ^3^Papageorgiou General Hospital, Fourth Department of Pediatrics, Aristotle University of Thessaloniki, School of Medicine, Faculty of Health Sciences , Thessaloniki, Greece, ^4^Wilhelmina Kinderziekenhuis, Department of Pediatric Immunology and Rheumatology, Utrecht, Netherlands, ^5^University of Siena, Department of Medical Sciences, Surgery and Neurosciences, Rheumatology Unit, Siena, ^6^Fondazione IRCCS Policlinico San Matteo, Centro per lo Studio e la Cura delle Amiloidosi Sistemiche, Pavia, ^7^Fondazione IRCCS Ca’ Granda Ospedale Maggiore Policlinico, Dipartimento di Medicina Interna, UOS Malattie Rare, Milano, Italy, ^8^National Referral Centre of Auto-Inflammatory Diseases and inflammatory amyloidosis, CEREMAIA, CHU de Biĉetre, APHP, University of Paris Sud, Department of Pediatric Rheumatology, Le Kremlin Biĉetre, France, ^9^Arabkir Medical Complex - Institute of Child and Adolescent Health, National Pediatric Centre for Familial Mediterranean Fever; Yerevan State Medical University, Yerevan, Armenia, ^10^Beatrix Kinderkliniek, University Medical Center, Department of Pediatric Rheumatology, Groningen, ^11^Radboud UMC, Paediatric Rheumatology , CUKZ 435, Nijmegen, Netherlands, ^12^University Hospital Tuebingen, Department of Pediatrics, Division of Pediatric Rheumatology and Autoinflammation Reference Center, Tuebingen, Germany, ^13^University Hospital Valle de Hebron, Rheumatology Unit, Barcelona, Spain, ^14^Universita’ di Napoli Federico II, Dipartimento di Pediatria, Napoli, ^15^IRCCS Istituto Giannina Gaslini, UOSID Centro Trial, PRINTO, ^16^IRCCS Istituto Giannina Gaslini, UOSD Centro Malattie Autoinfiammatorie e Immunodeficienze, Genoa, Italy

###### **Correspondence:** Š. Fingerhutová

**Introduction:** Despite estimates of the rate of AA amyloidosis complicating autoinflammatory diseases (AID), its true incidence especially in children is not known. It is anticipated that kidney involvement manifesting as proteinuria can be the first detectable marker of organ amyloid A deposition.

**Objectives:** To assess the frequency of renal pathology in a large cohort of patients with AID reported to the Eurofever registry.

**Methods:** Clinical and genetic data were extracted from the Eurofever registry based on optional responses. Data from the registration form submitted until July 2021 were analysed.

**Results:** From the total of 6628 patients enrolled in the Eurofever registry to date, in 229 (3.46%; 114 females) abnormal value of proteinuria (PU) and/or microalbuminuria (miALBU) was reported. Mean age at the time of registration was 23.1 years (SD 17.1), 123 (53.7%, 62 females) were children. Disease duration from symptom onset was 14.9 (SD 14.1) years. The most common diagnosis was Familial Mediterranean Fever (FMF) (n=89, 38.9%), undefined AID (uAID) (n=35, 15.3%), Syndrome of Undifferentiated Recurrent Fever (SURF) (n=26, 11.4%), cryopyrinopathy (CAPS) (n=26, 11.4%), Mevalonate Kinase Deficiency (MKD) (n=14, 6.1%) and Tumor Necrosis Factor Receptor-Associated Periodic Syndrome (TRAPS) (n=13, 5.7%). Chronic nonbacterial osteomyelitis (CNO), DADA2, PFAPA, Behcet disease, PAPA syndrome, Blau syndrome, CANDLE and TNFAIP3-associated autoinflammatory syndrome were less common (2.6%, 2.2%, 2.2%, 1.8%, 0.9%, 0.9%, 0.4% and 0.4%) respectively. Presence of amyloidosis was confirmed in 47/6628 patients (0.7%; 25 females, mean age 39.8 years, SD 17.2), 6 of them were children (12.8%, 4 girls, mean age 11 years, SD 4.1). Majority of patients had TRAPS (n=19, 40.4%) and FMF (n=16, 34%) followed by CAPS (n=6, 12.8%), MKD (n=4, 8.5%), PAPA and Blau syndrome (n=2, 4.3%). Amyloidosis in paediatric patients was associated with FMF (4 cases), TRAPS and MKD (1 case each).

**Conclusion:** The data suggest that despite low numbers children are at risk of developing amyloidosis. High proportion of children with proteinuria warrants further analysis of follow-up data. As these have been available for minority of patients only this should alert physicians to keep submitting these data into the registry.

This study was supported by the Czech Health Research Council (AZV CR) grant NU21-05-00522

**Disclosure of Interest**: None declared

### P137. Bisphosphonates in chronic recurrent multifocal osteomyelitis: pamidronate vs zoledronate

#### R. Galindo Zavala^1^, L. G. Martín-Pedraz^1^, G. Díaz-Cordovés Rego^2^, E. Núñez-Cuadros^1^

##### ^1^Pediatrics, ^2^Rheumatology, Hospital Regional Universitario de Málaga, Malaga, Spain

###### **Correspondence:** R. Galindo Zavala

**Introduction:** Pamidronate showed to be an effective and secure treatment for chronic recurrent multifocal osteomyelitis (CMRO). Nevertheless, there are very few reports about other bisphosphonates.

**Objectives:** To compare pamidronate vs zoledronate’s effectivity and safety in children suffering from CMRO.

**Methods:** Prospective, descriptive, and analytical research on children younger than 16 suffering from CMRO (diagnosed according to Jansson criteria) and treated with bisphosphonates between January 2013 and December 2020. Flares treated with pamidronate (0,5 mg/kg day 1; 1 mg/kg day 2; 1 mg/kg day 3 followed by 1 mg/kg/month) were compared to those treated with zoledronate (0,025 mg/kg/3 months).

**Results:** We identified 16 bisphosphonates treated flares, 6 with pamidronate and 10 with zoledronate, in 12 patients. Average length of pamidronate and zoledronate courses were 3,67 and 12,3 months (p < 0,01), respectively. Epidemiological and clinical data are shown in table 1. There were no significative differences in sex, age or clinical features in patients treated with pamidronate vs zoledronate.

50% of patients treated with pamidronate received corticosteroids simultaneously vs 40% of patients treated with zoledronate (p =0,696). There were no differences in the highest corticosteroid dose (0,33 *vs* 0,72mg/kg/day; p=0,095) or the corticosteroids’ treatment length (1,67 *vs* 1,85 months; p=0,879)

66,7% of pamidronate treated patients reached full response vs 80% of patients who were treated with zoledronate (p=0,474). There were no differences in the clinical response time (2 months vs 0 months; p =0,524), the remission time after discontinuing bisphosphonate (9,25 *vs* 10 months; p=0,877) or the recurrences rate while on treatment (1 *vs* 0; p=0,298). 33,3% of patients on pamidronate and 40% of patients on zoledronate needed switching to anti-TNFa (p=0,790).

Flu-like syndrome was the only reported adverse event. It was recorded in 16,7% of patients treated with pamidronate, and 40% of patients who received zoledronate (p=0,588).

**Conclusion:** Pamidronate’s and zoledronate’s effectivity and security in children suffering from CMRO seem to be similar. Zoledronate permits a more comfortable dosage schedule and reduces hospital stay, improving the quality of life of these patients. Therefore, it may be selected as treatment of choice in children suffering from CMRO resistant to NSAIDs.

**Patient Consent:** Yes, I received consent

**Disclosure of Interest**: None declared

**Table 1 (abstract P137).** Clinical and analytical data comparative study

	Pamidronate	Zoledronate	p-value
**Clinical onset**			
Fever, n (%)	2 (33,3%)	0	0,051
Pain, n (%)	6 (100%)	10 (100%)	-
Swelling, n (%)	2 (33,3%)	6 (60,0%)	0,302
Number of inflammatory foci, average±SD	2,67±1,75	1,90±0,99	0,278
Clinical evolution length, median (IQR) (months)	3,00 (1,12-9,75)	0,50 (0,29-7,00)	0,280
**Laboratory data**			
CRP, median (IQR)	10,00 (7,20-24,55)	5,40 (2,90-17,10)	0,354
ESR, average±SD	36,20 ± 19,01	26,37 ± 21,06	0,415

### P138. Use of canakinumab in children with cryopyrin-associated periodic syndrome: a single center study

#### A. Gunalp, M. Deveci, F. Haslak, M. Yildiz, A. Aliyeva, O. Koker Turan, S. Sahin, A. Adrovic, K. Barut, O. Kasapcopur

##### Pediatric Rheumatology, Istanbul University-Cerrahpasa, Cerrahpasa Medical Faculty, Istanbul, Turkey

###### **Correspondence:** A. Gunalp

**Introduction:** Cryopyrin-Associated Periodic Syndrome (CAPS) is a rare inherited autoinflammatory disease with uncontrolled inflammatory symptoms due to excessive secretion of IL-1β caused by a mutation in the NLRP3 gene.

**Objectives:** The aim of this study is to evaluate the efficacy and safety of canakunimab in a large patient cohort.

**Methods:** 24 patients who were diagnosed with CAPS according to the Eurofever/PReS diagnostic criteria were included. The frequency of attacks and acute phase reactants before and after canakinumab treatment in 2 year follow up were evaluated.

**Results:** A total of 24 (%54 female, %46 male) patients with 5.18 mean age (11-13) were included. 19 of patients were diagnosed with FCAS, 1 Muckle Wells Syndrome and 1 was CINCA. Pathogenic mutation was found in NLRP3 gene in 21 of the patients. 3 of patients had no mutation. The clinical symptoms of our patients were; all of them had urticeria-like skin rash, 21 had fever, 13 had arthritis, 9 had abdominal pain, same 6 patients had oculer findings and convulsions, 4 patients had hearing loss, 1 had bone deformity and 1 had myalgia. 4 of the patients had central nervous system involvement with pathological MRI findings. The main reason to iniate canakinumab treatment was unresponsiveness and non-compliance to the current treatment (anakinra). The frequency of the attacks in the prior year of canakinumab treatment was median 17. 3 (5-30) and it decreased to median 1.17 (0-5) following 1 year use of canakinumab (p<0.01). Statistically significant difference was found between repeated measures of ESR levels when compared prior and following 1, 6, 12, 24 months of canakinumab treatment (p:0.01, p:0.013, p: 0.21, respectively). Mean canakinumab treatment duration was 4.2 (2-8,7) years. In the follow up one of our patients had MAS attacks and SLE-like autoimmune disease developed in the 5th year of canakinumab treatment in the same patient. One of the patient with CINCA syndrome died in the 15th month of treatment at the age of 39 months. No significant side effects were observed in our patients.

**Conclusion:** Cryopryrinoyrin-Associated Periodic Syndrome is a rare childhood disease with urticaria-like rash, fever and arthritis. Canakinumab is a safe and effective agent to control the inflammation of the disease.

**Patient Consent:** Yes, I received consent

**Disclosure of Interest**: None declared

### P139. A20 haploinsufficiency identified in a girl initially diagnosed with systemic juvenile idiopathic arthritis

#### W. J. Jang^1^, J. W. Rhim^2^, S. Y. Lee^3^, H. Y. Choi^4^, S. J. Lee^5^, M. Kim^6^, D.-C. Jeong^1^

##### ^1^Pediatrics, Seoul St. Mary’s Hospital, College of Medicine, The Catholic University of Korea, Seoul, ^2^Pediatrics, Daejeon St. Mary;s Hospital, College of Medicine, The Catholic University of Korea, Daejeon, ^3^Pediatrics, Bucheon St. Mary’s Hospital, College of Medicine, The Catholic University of Korea, Bucehon, ^4^Pediatrics, Seoul St. Marys’s Hospital, College of Medicine, The Catholic University of Korea, Seoul, ^5^Pediatrics, International St. Mary’s Hospital, Catholic Kwandong University , Incheon, ^6^Laboratory Medicine, Catholic Genetic Center, Seoul St. Mary’s Hospital, College of Medicine, The Catholic University of Korea, Seoul, Korea, Republic Of

###### **Correspondence:** W. J. Jang

**Introduction:** Haploinsufficiency of A20 (HA20) is a newly described autoinflammatory disease caused by mutations in tumor necrosis factor-α-induced protein 3 (TNFAIP3) gene. Patients present wide spectrum of manifestations with early-onset systemic inflammation like Behcet’s disease or other autoimmune features.

**Objectives:** To investigate the clinical course and immunologic characterization in HA20 patient.

**Methods:** We report the case of HA20 initially diagnosed with systemic juvenile idiopathic arthritis (sJIA).

An 11-year-old girl has presented recurrent episodes of fever with vomiting, diarrhea since 11-month of age. She was diagnosed with sJIA presenting fever with skin rash, lymphadenopathy and arthritis with pericardial effusion at 27-month-old. She improved after high dose steroid, but developed fever with gastrointestinal symptoms including abdominal pain and vomiting about five times per year in spite of disease-modifying anti-rheumatic drugs therapy. Acute phase reactants were markedly increased during the period of her symptoms and decreased by conservative treatment with hydration, but still showed elevated levels when she was well-being state. She was shown as growth retardation, and microcytic hypochromic anemia in spite of iron supplement.

**Results:** At the age of 11, she admitted due to the recurrent gastrointestinal symptoms with high acute phase reactants. Colonoscopy showed multiple ulcers on intestinal mucosa compatible with Crohn’s disease. The next generation sequencing identified the pathogenic heterozygous mutation (c.427C>T) at *TNFAIP3* gene, leading to HA20. This genetic mutation presents to fail to the negative feedback mechanism for nuclear factor kappa B (NF-κB) activation, developing autoinflammatory features such as fever and systemic inflammation. Immunologic study showed high expression of IL-17 and IFN-γ in CD4 and CD8 T cells independent on zinc, and elevated level of regulatory T cells. Also, IL-1β and TNF-α in plasma were shown high level than healthy control.

**Conclusion:** HA20 should be considered in the patient with recurrent inflammatory disease that presented with fever and gastrointestinal symptoms which might be regarded as respective episodes of acute infections.

**Patient Consent:** Yes, I received consent

**Disclosure of Interest**: None declared

### P140. Evaluation of gastrointestinal system complaints in pediatric Familial Mediterranean Fever patients

#### H. D. Karakas, Z. B. Ozcakar, F. Aydin, N. Cakar, F. Yalcinkaya

##### Pediatric Rheumatology, Ankara University, Ankara, Turkey

###### **Correspondence:** H. D. Karakas

**Introduction:** Familial Mediterranean fever (FMF) is the most prevelant hereditary autoinflammatory disease among children, manifesting with recurrent attacks of serositis accompanied by fever, usually lasting 12-72 hours. Abdominal pain and various gastrointestinal system (GIS) manifestations may arise directly from FMF or from other causes independent of primary disease.

**Objectives:** The aim of this study was to evaluate gastrointestinal complaints other than classical peritonitis attacks in patients with FMF and to interpret laboratory, endoscopic and histopathological findings of GIS manifestations.

**Methods:** The medical records of the cases with FMF, who attended to the Ankara University Pediatric Rheumatology outpatient clinic, were evaluated retrospectively from December 2011 to December 2021. Demographic data, anthropometric measures, main clinical symptoms of the episodes, treatment modalities, genetic mutations, family history, gastrointestinal system complaints, endoscopic and histologic data, serum aminotransferase levels (alanine aminotransferase [ALT], aspartate aminotransferase [AST]) were recorded. Descriptive statistics were used. All statistical analyses were carried out using SPSS 25.0.

**Results:** A total of 576 pediatric patients (female 301, 52.3%), diagnosed with FMF since 2011 to 2021, were included. Majority of the patients (n=416, 72.2%) were found to have at least one exon 10 mutations. All of the patients were treated with colchicine, and almost 90.3% uses colchicine regularly. Functional gastrointestinal symptoms were reported by 106 patients (18.4%). Among these, abdominal pain (65.1%) and diarrhea (42.5%) were the most common complaints, followed by dyspepsia (33%), nausea and vomiting (27.4%). Malnutrition was observed in 42 patients (7.3%). Appendectomy was notified in 40 patients (6.9%). High serum aminotransferase levels were detected in 105 FMF patients (18.2%) at any visits, only 38 patient had hypertransaminasemia (AST x 3 unv; ALT x 3 unv). The most common cause of hypertransaminasemia was viral infections (31.6%). Only three patients’ elevated enzymes were associated with colchicine usage and stopped only the longest period of a month. Gastroenterology referral was done in 144 patients (25%) during follow-up. A total of 174 patients was monitored by abdominal ultrasonography for any reason. Upper GIS endoscopy was performed in 61 patients (10.6%), and colonoscopy in 30 (5.2%) patients. Accompanying GIS diseases were detected in 78 patients (13.5%). Upper gastrointestinal tract diseases (gastroesophageal reflux disease, gastritis, duodenitis, peptic ulcer) (6.1%), inflammatory bowel disease (2.6%), and irritable bowel syndrome (1%) were the most common coexisting GIS diseases.

**Conclusion:** Patients with FMF could have frequent GIS complaints other than classical abdominal attacks. Taking a good medical history, clinical evaluation and gastroenterology consultation will result in early diagnosis and treatment of coexisting or associated diseases in these patients.

**Patient Consent:** Yes, I received consent

**Disclosure of Interest**: None declared

### P141. Chronic nonbacterial osteomyelitis and immune checkpoint molecules

#### U. Kaya Akca^1^, B. Aydın^2^, E. Sag^2,3^, Y. Bayındır^1^, Y. Bilginer^1^, S. Ozen^1,2^

##### ^1^Pediatric Rheumatology, ^2^Pediatric Rheumatology Unit, Translational Medicine Laboratories, Hacettepe University, ^3^Pediatric Rheumatology Clinic, Ankara Training and Research Hospital, Ankara, Turkey

###### **Correspondence:** U. Kaya Akca

**Introduction:** Varying levels of immune checkpoint molecules have been reported in inflammatory and autoimmune diseases. Chronic nonbacterial osteomyelitis (CNO) is a rare autoinflammatory bone disease characterized by sterile bone inflammation.

**Objectives:** This study aimed to investigate the levels of checkpoint molecules in pediatric patients with CNO.

**Methods:** Plasma samples were collected from CNO patients at diagnosis or during treatment with a biologic agent. Plasma levels of PD-1 (programmed cell death protein 1) and TIM3 (T cell immunoglobulin and mucin domain-containing protein 3), which are immune checkpoint molecules, were measured using the sandwich enzyme-linked immunosorbent assay (ELISA) method. Plasma samples of healthy controls were used as the control group.

**Results:** Plasma samples were obtained from 18 CNO patients at the time of diagnosis and from nine patients after receiving biologic treatment. A total of 27 CNO patients (51.8% male) and six healthy controls (50.0% male) were included in the study. The median age of the patient and control groups was 14.5 years and 13.5 years, respectively (p=0.762). Plasma TIM3 levels were within the normal range in both the patient and control groups, and no significant difference was found between the two groups (p=0.981). Median plasma PD-1 levels were significantly lower in the total CNO group (treated and at diagnosis) compared to healthy controls (998.7 vs 8263.1, p=0.011). Plasma PD-1 levels of CNO patients at diagnosis were also lower compared to the healthy controls (p=0.023).

There was no difference in plasma TIM3 and PD-1 levels between diagnosis and after treatment with a biologic agent (p=0.136 and p=0.735, respectively). Also, the plasma TIM3 and PD-1 levels of CNO patients were not different between those with and without spinal lesions (p=0.072 and p=0.621, respectively).

**Conclusion:** Plasma PD-1 levels were significantly lower in CNO patients compared to healthy controls. This may reflect the low contribution of adaptive immunity in the pathogenesis of the disease since CNO is an autoinflammatory disease.

**Patient Consent:** Yes, I received consent

**Disclosure of Interest**: None declared

### P142. Familial case of PSTPIP1-associated myeloid-related proteinemia inflammatory (PAMI) syndrome

#### Y. Y. Choi^1^, S. H. Kim^2^

##### ^1^Pediatrics, ^2^Department of Pediatrics, Seoul National University Children’s Hospital, Seoul, Korea, Republic Of

###### **Correspondence:** S. H. Kim

**Introduction:** In recent years, the phenotype of PSTPIP1 associated autoinflammatory syndrome has expanded. Classic phenotype is PAPA syndrome (Pyogenic Arthritis, Pyoderma gangrenosum, and severe nodulocytic Acne) and a newly identified PSTPIP1-associated myeloid-related proteinemia inflammatory (PAMI) syndrome has some unique features including very early onset severe chronic systemic inflammation, lymphadenopathy, hepatosplenomegaly and pancytopenia.

**Objectives:** I would like to share our experience on familial PAMI syndrome, one of the familial autoinflammatory syndromes.

**Methods:** We described our first 1^st^ case of familial PAMI syndrome in Korea.

**Results:** A 14-month-old male patient presented with fever for 11 days, diarrhea, cervical lymphadenitis, and cyclic rash. Despite prolonged antibiotics treatment, fever continued for 12 days, and subsided spontaneously from 13th day. Fever usually spiked at night, but sometimes presented during daytime. CSF study confirmed sterile. Abdominal CT-scan showed mild hepatomegaly and several mesenteric lymph node enlargement. Peripheral blood smear showed mild leukopenia with a few plasmacytoid lymphocytes (2%). Laboratory work up showed LDH(Lactate dehydrogenase), CRP(C-reactive protein), ESR(erythrocyte sediment rate) elevation. At his 15-month of age, he experienced prolonged fever for 10 days again and subsided spontaneously. At his 16th month of age, he visited again with rhinorrhea, voice change, cervical lymphadenitis, erythematous rash, and fever. Laboratory work up showed elevated LDH, CRP, ESR and mild leukopenia. Immunoglobulin profile (IgG,A,M,D,E) showed mild IgG elevation, and ANA appeared positive (1:80). Echocardiogram revealed no cardiac anomaly. From medical history of unexplained recurrent fever with persistently increased inflammatory markers, we suspected autoinflammatory syndrome and performed targeted exome sequencing, which revealed pathogenic known mutation on his PSTPIP1 gene, which caused protein change (E275K). The same point mutation was found in his father as well and detailed history taking revealed his father had similar symptom throughout his life. The patient’s father was treated for lymphadenitis when he was four years of age, and he has been treated for periodic fever and JIA from his high school period since now. Recently, he is experiencing recurrent abdominal pain, diarrhea and oral ulcers which is thought to be symptoms of this disease.

**Conclusion:** Here, we present the 1^st^ case of familial PAMI syndrome in Korea.

**Patient Consent:** Yes, I received consent

**Disclosure of Interest**: None declared

### P143. Withdrawn

### P144. Long-term safety and efficacy of canakinumab in cryopyrin-associated periodic syndromes (CAPS) – 36-month data from the reliance registry

#### J. B. Kuemmerle-Deschner^1^, B. Kortus-Goetze^2^, P. T. Oommen^3^, A. Janda^4^, J. Rech^5^, C. Schuetz^6^, T. Kallinich^7^, F. Weller-Heinemann^8^, G. Horneff^9^, I. Foeldvari^10^, F. M. Meier^11,12^, M. Borte^13^, T. Krickau^14^, J. Weber-Arden^15^, N. Blank^16^

##### ^1^Department of Pediatrics, Division of Pediatric Rheumatology, University Hospital Tuebingen, Tuebingen, ^2^Division of Nephrology, University of Marburg, Marburg, ^3^Clinic of Pediatric Hematology, Oncology and Clinical Immunology, Heinrich-Heine-University Duesseldorf, Duesseldorf, ^4^Department of Pediatrics, University Hospital Ulm, Ulm, ^5^Department of Internal Medicine 3 - Rheumatology and Immunology, Friedrich-Alexander University (FAU) Erlangen-Nuernberg and Universitaetsklinikum Erlangen, Erlangen, ^6^Pediatrics, Medizinische Fakultaet Carl Gustav Carus, Technische Universitaet Dresden, Dresden, ^7^Department of Pediatrics, Division of Pulmonology, Immunology and Critical Care Medicine, Charité University Medicine Berlin, Berlin, ^8^Prof. Hess Kinderklinik, Klinikum Bremen-Mitte, Bremen, ^9^Asklepios Clinic Sankt Augustin, Sankt Augustin, ^10^Centre for Pediatric and Adolescence Rheumatology Hamburg, Hamburg, ^11^Division of Rheumatology, University Hospital Frankfurt, Goethe University, Frankfurt am Main, ^12^Project Group Translational Medicine and Pharmacology TMP, Fraunhofer Institute for Molecular Biology and Applied Ecology IME, Frankfurt am Main, ^13^ImmunoDeficiencyCenter Leipzig (IDCL), Hospital St. Georg gGmbH Leipzig, Leipzig, ^14^Pediatrics, Friedrich-Alexander Universität Erlangen-Nürnberg (FAU), Erlangen, ^15^Novartis, Nuernberg, ^16^Rheumatology, University Hospital Heidelberg, Heidelberg, Germany

###### **Correspondence:** A. Janda

**Introduction:** Cryopyrin-associated periodic syndromes (CAPS) are monogenic autoinflammatory diseases with severe systemic inflammation. The IL-1β inhibitor canakinumab (CAN) leads to a rapid remission of CAPS symptoms in clinical trials as well as in practice.

**Objectives:** The RELIANCE registry investigates the long-term safety and efficacy of CAN under routine clinical conditions in pediatric (≥2 years) and adult patients with CAPS, including MWS, FCAS, and NOMID/CINCA[1].

[1]CINCA: chronic infantile neurologic cutaneous articular syndrome, FCAS: familial cold-induced autoinflammatory syndrome, MWS: muckle-wells syndrome, NOMID: neonatal multisystem inflammatory syndrome

**Methods:** This prospective, non-interventional, observational study enrolls patients with a clinically confirmed diagnosis of CAPS who routinely receive CAN. Clinical data, physician assessments, and patient-reported outcomes will be collected at baseline and at 6-monthly visits.

**Results:**

98 CAPS patients (52% female; median age 20 years) were enrolled through December 2021. At the 36-month visit, both physicians and patients of all ages rated current disease activity as absent or mild/moderate (Table 1). The proportion of patients without disease activity was highest in <12-year-old patients (78%). More pediatric compared to adult CAPS patients received higher than standard CAN (<12 years: 76%, 12-17 years: 43%, ≥18 years: 35%). Proportionate, more AE, SAE, and presumably drug-related SAE occurred in the pediatric cohort.

Pathogenic mutations were documented for a total of N=38 patients, including R260W: N=15, A439V: N=9, T348M: N=9, D303N: N=3, and E627G, G755R, and G569R: N=1 each. N=27 patients with pathogenic mutations received standard dose CAN and N=9 patients received higher dose.

**Conclusion:** The 36-month interim analysis of the RELIANCE study shows that long-term treatment with CAN is safe and effective in patients with CAPS regardless of the underlying mutation. Pediatric patients tend to have a higher infection rate with a better response rate.

**Patient Consent:** Yes, I received consent

**Disclosure of Interest**: J. B. Kuemmerle-Deschner: None declared, B. Kortus-Goetze Consultant with: Novartis, P. T. Oommen Grant / Research Support with: Novartis, A. Janda: None declared, J. Rech Grant / Research Support with: Novartis, Sobi, Consultant with: AbbVie, Biogen, BMS, Chugai, GSK, Janssen, Lilly, MSD, Mylan, Novartis, Roche, Sanofi, Sobi, UCB, Speaker Bureau with: AbbVie, Biogen, BMS, Chugai, GSK, Janssen, Lilly, MSD; Mylan, Novartis, Roche, Sanofi, Sobi, UCB, C. Schuetz: None declared, T. Kallinich Speaker Bureau with: Roche, F. Weller-Heinemann: None declared, G. Horneff Grant / Research Support with: AbbVie, Chugai, Merck Sharp & Dohme, Novartis, Pfizer, Roche, Speaker Bureau with: AbbVie, Bayer, Chugai, Merck Sharp & Dohme, Novartis, Pfizer, Roche, I. Foeldvari Consultant with: Novartis, F. M. Meier Speaker Bureau with: Novartis, M. Borte Grant / Research Support with: Pfizer, Shire, T. Krickau Grant / Research Support with: Novartis, Consultant with: Novartis, Speaker Bureau with: Novartis, J. Weber-Arden Grant / Research Support with: Novartis, AbbVie, Sobi, Consultant with: Novartis, AbbVie, Sobi, N. Blank Grant / Research Support with: Novartis, Sobi, Consultant with: Novartis, Sobi, Lilly, Pfizer, Abbvie, BMS, MSD, Actelion, UCB, Boehringer-Ingelheim, Roche

**Table 1 (abstract P144).** Age-dependent assessment of clinical disease activity and incidence of infection

	<12 years		12-17 years		≥18 years	
Disease activity by age group	N=33		N=7		N=55	
	Baseline	36 Months	Baseline	36 Months	Baseline	36 Months
Patient assessment of current disease activity; 0-10, median (min; max).	1.0 (0; 6)	0 (0; 6)	2.0 (0; 5)	0 (0; 4)	2.0 (0; 7)	2.0 (0; 6)
Physician assessment of current disease activity, % of patients absent / mild/moderate	39/52	78/22	43/57	60/40	43/50	57/43
**Infection rate by age group** N patients (%) [number of events / incidence rate per 100 patient-years]	**N=33**	**N=7**	**N=55**			
AE	19 (58) [59 / 69.4]	5 (71) [7 / 37.8]	21 (38) [46 / 35.4]			
SAE	4 (12) [5 / 5.9]	2 (29) [4 / 21.6]	3 (6) [3 / 2.2]			
SADR	3 (9) [3 / 3.5]	1 (14) [1 / 5.4]	1 (2) [1 / 0.7]			

AE, adverse event; SADR, serious adverse drug reaction; SAE, serious adverse event

### P145. Gene expression analysis of pro-inflammatory cytokines and associated transcription factors in patients with blau syndrome from India

#### R. Kumrah, R. Rikhi, D. Suri, A. Rawat, S. Singh

##### Advanced Pediatrics Centre, PGIMER, Chandigarh, India

###### **Correspondence:** R. Kumrah

**Introduction:** Blau syndrome (BS) is a rare monogenic form of autoinflammatory disease caused by gain-offunction mutation in NOD2 gene and is characterized by granulomatous arthritis, dermatitis, and uveitis since early childhood.

**Objectives:** To perform gene expression analysis of pro-inflammatory cytokines and associated transcription factors in patients with Blau syndrome

**Methods:** Confirmation of genetic diagnosis in patients suspected with Blau syndrome was carried out. Complementary DNA (cDNA) converted from extracted whole blood RNA was used to perform real-time PCR analysis. Genes for pro-inflammatory cytokines and associated transcription factors were selected. Comparison of fold change [2^(-ΔΔCT) method] between patients and controls were performed.

**Results:** BS was genetically confirmed in 6 patients (3 males, 3 females from 5 families). Missense heterozygous mutation involving amino acid change from arginine to tryptophan at position 334 (hotspot) was identified in the nucleotide-binding domain of NOD2 gene in all. All the patients were on treatment in clinic with methotrexate, corticosteroids and adalimumab. Real-time PCR analysis revealed reduced NOD2 gene expression in patients as compared to control. Expression of Eomes, IL-1B, FOXP3 was also found to be less in patients with Blau syndrome. Reduced expression of transcription regulators of inflammation (NFĸβ1 and NFĸβ2) was noted in patients as compared to healthy control. Elevated expression of pro-inflammatory (TNF- ) and master regulator for T cells (T-bet) was noted in patients. Other genes (ROR-γT, GATA3, IL-6, IL18) were comparable in patients and healthy controls.

**Conclusion:** R334W variant was identified in all patients with clinically suspected BS. We found altered expression of various cytokines and transcription factors in patients with Blau syndrome.

**Patient Consent:** Yes, I received consent

**Disclosure of Interest**: None declared

### P146. Multiple autoinflammatory and autoimmune features in families with haploinsufficiency of A20

#### L. Levantino^1^, S. Pastore^2^, F. Biscaro^3^, M. Girardelli^2^, A. Tesser^2^, S. Martelossi^3^, A. Zabotti^4^, A. Tommasini^1,2^

##### ^1^Department of Medical, Surgical and Health Sciences, University of Trieste, ^2^Department of Pediatrics, IRCCS Burlo Garofolo, Trieste, ^3^Department of Pediatrics, Santa Maria di Ca’ Foncello Hospital, Treviso, ^4^Department of Rheumatology, AOU Santa Maria della Misericordia, Udine, Italy

###### **Correspondence:** L. Levantino

**Introduction:** Haploinsufficiency of A20 (HA20) is an immune dysregulation disease due to heterozygous loss-of-function mutations in *TNFAIP3*, encoding A20 protein, a crucial negative regulator of the NF-kB/TRAF6 pathway. HA20 leads to an imbalance in both innate and adaptive immunity, with high penetrance but variable expressivity. As a result, the phenotype of HA20 may feature both autoinflammatory and autoimmunity disorders.

**Objectives:** To describe phenotypic features, response to treatments, and natural history of HA20 in children and adults.

**Methods:** We performed a retrospective analysis of a case series with HA20, currently cared for at three rheumatologic centres in North-eastern Italy.

**Results:** Overall seven patients from three unrelated Italian families with a genetic diagnosis of HA20 were included in the study. Four were female (57%) and the median age was 10 years (range 1-62). All patients were symptomatic, showing clinical manifestations with an early onset and a relapsing-remitting course. Previous diagnoses included PFAPA, Behçet’s disease, Hashimoto’s disease, celiac disease, autoimmune hepatitis, IBD, psoriatic arthritis, rheumatoid arthritis, Sjogren syndrome, and SLE. In all cases, a history of recurrent aphthous was present since infancy. Other clinical features were recurrent fever (4/7), recurrent upper respiratory infections (4/7), gastrointestinal symptoms (3/7), arthritis/arthralgia (4/7), skin involvement (2/7), psychiatric complaints (4/7), and autoimmune disorders (3/7, including celiac disease, thyroiditis, hepatitis). About laboratory findings, acute-phase reactants were elevated in 100% of patients and ANA/ENA autoantibodies were positive in one; IFN-score was high in 85% of cases and in two cases it increased during anti-TNF treatment. All patients had been received corticosteroids; six of them needed other immunosuppressive treatments.

**Conclusion:** HA20 is an heterogenous immune disorder characterized by both autoinflammation, tending to develop in early childhood, and autoimmunity, usually appearing from late childhood/adolescence. The “amplifier” role of A20 deficiency on distinct pathways in the lifetime may challenge therapeutic and preventive approaches.

**Patient Consent:** Yes, I received consent

**Disclosure of Interest**: None declared

**Table 1 (abstract P146).** See text for description

FAMILY	PATIENT	GENDER	AGE	ONSET	COURSE	CLINICAL MANIFESTATIONS	LABORATORY FINDINGS	PREVIOUS DIAGNOSES	TREATMENTS
1	1	F	62y	early childhood	relapsing-remitting	rA, HD, AA, PS	CRP/ESR ↑, ANA/ENA +, IFN-score -	HD, PA, RA, SS, SLE	steroids > adalimumab > tofacitinib
	2	F	32y	early childhood	relapsing-remitting	rA, rF, rI, CD, HD, AA, SK, PS, AH	CRP/ESR ↑, ANA/ENA -, IFN-score +	CD, AH, HD	steroids > azathioprine
	3	M	10y	<1y	relapsing-remitting	rA, CD	CRP/ESR ↑, IFN-score +	none	steroids > colchicine
	4	M	8y	<1y	relapsing-remitting	rA, rF, rI, GI, PS	CRP/ESR ↑, IFN-score +	PFAPA, IBD, BD	steroids > adalimumab
	5	F	1y	<1y	relapsing-remitting	rA, rF, rI	CRP/ESR ↑, IFN-score +	none	steroids
2	6	M	22y	<1y	relapsing-remitting	rA, rF, rI, GI, AA, PS	CRP/ESR ↑, ANA/ENA -, IFN-score +	PFAPA, IBD, BD	steroids > adalimumab > apremilast
3	7	F	2y	<1y	relapsing-remitting	rA, GI, AA, SK	CRP/ESR ↑, IFN-score +	IBD	steroids > anakinra > infliximab + methotrexate

F, female; M, male; y, years; rA, recurrent aphthous; rF, recurrent fever; rI, recurrent infections; GI, gastrointestinal symptoms; CD, celiac disease; HD, Hashimoto’s disease; AA, arthritis/arthralgia; SK, skin involvement; PS, psychiatric complaints; AH, autoimmune hepatitis; PA, psoriatic arthritis; RA, rheumatoid arthritis; SS, Sjogren syndrome; BD, Behçet’s disease

### P147. Do Familial Mediterranean Fever (FMF) patients treated with interleukin-1 inhibitors adhere to colchicine treatment? A population-based study

#### Y. Levinsky, L. Harel, R. Tal, G. Amarylio

##### Schneider children medical center of Israel, Petah Tikva, Israel

###### **Correspondence:** Y. Levinsky

**Introduction:** Familial Mediterranean fever (FMF) is the most common monogenic autoinflammatory disease. Despite the progress research since the discovery of the MEFV gene, colchicine treatment is still considered a main treatment for FMF patients. It reduces the frequency of attacks and effectively prevents the complication of secondary amyloidosis. Therefore, it is currently recommended to continue colchicine prophylaxis during the treatment with IL-1 inhibitors, despite clinical remission.

**Objectives:** Our aims where to evaluate the rate of adherence to colchicine prophylaxis among patients with FMF under IL-1 Inhibitors treatment and to examine different risk factors for low adherence among that group.

**Methods:** The databases of Maccabi Health Services (MHS), a 2.6 million member state-mandated health provider in Israel was searched for patients with FMF diagnosis. Patients under treatment with IL-1 inhibitors were matched to themselves – before and after starting the treatment. A sub-analysis was done among patients without amyloidosis or chronic renal failure. In the second phase, patients treated with IL-1 inhibitors were matched in a ratio of 1:4 to patients on colchicine only. Medication possession ratio (MPR) was used as main outcome measure.

**Results:** The final cohort included 4526 patients. Among them, 108 patients were treated with IL-1 inhibitors. The MPR was higher after starting IL-inhibitors among the total population (19.7±13.1 before versus 32.2±14.5 after, p<0.01), and among patients without amyloidosis or chronic renal failure (21.6±15.07 before versus 32.8±15.3 after, p<0.01). In the second phase, patients treated with IL-1 inhibitors were matched in 1:4 ratio to 432 “colchicine only” patients. The total MPR in each groups was similar (78.9 ±41.4 versus 82.5 ± 80.6, P=0.5). In the sub-analyses, females treated with IL-1 inhibitors had less MPR than in the matched group (81.4 ± 33.2 versus 85.1 ± 91, P=0.03).

**Conclusion:** Contrary to initial concerns, the adherence to colchicine increases among the same patients after starting IL-1 inhibitors, which may reflect the improvement in disease perception following the need to start the biological treatment. Moreover, the adherence is similar to the general FMF population after matching. Physicians who take care of FMF patients can consider treatment with IL-1 inhibitors when indicated without fear of decline in adherence to colchicine. Education regarding the importance of colchicine treatment is important among all FMF patients.

**Disclosure of Interest**: None declared

**Table 1 (abstract P147).** Comparison of medication possession ratio (MPR) of colchicine between FMF patients treated with IL-1 inhibitors Versus patients without IL-1 inhibitors treatment

Parameter	With IL-1 inhibitors – “study group” (n=108)	Without IL-1 inhibitors – “matched group” (N=432)	P-value
Age category, years			
≤6	74.9±24.9 (N=32)	75.8±33.7 (N=23)	0.9
7-14	90.2±53.6 (N=24)	75.6±70.3 (N=93)	0.09
15-29	76.5±47.9 (N=29)	91.1±123.3 (N=101)	0.3
≥30	75.8±33.7 (N=23)	77±49.9 (N=112)	0.6
Sex			
Male	75.7±49.9 (N=48)	79.2± 65.5 (N=192)	0.9
Female	81.4±33.2 (N=60)	85.1±91.0 (N=240)	0.03
Time of colchicine use			
< 8 years	102.8±46.2 (N=42)	100.9±99.5 (N=245)	0.2
8-15 years	66.6±35.8 (N=26)	56.7±36.5 (N=103)	0.2
> 15 years	61.9±24.8 (N=40)	60.4±25.8 (N=84)	0.7

### P148. IL-1antagonist treatment in recurrent aseptic meningitis related to Familial Mediterranean Fever

#### R. Livny^1^, Y. Bitterman^2^, Y. Butbul Aviel^2^

##### ^1^Pediatric Rheumatology, Schneider’s childrens hospital, Petach Tikva, ^2^Rambam medical center, Haifa, Israel

###### **Correspondence:** R. Livny

**Introduction:** Familial Mediterranean fever (FMF) is an autosomal recessive, auto-inflammatory disease, presenting with recurrent bouts of fever and polyserositis. FMF has been associated with central nervous system (CNS) manifestations and recurrent aseptic meningitis (RAM) is a rare described one. There are only few case reports of aseptic meningitis due to FMF.

**Objectives:** In this case-based review, we present a pediatric patient with FMF, who suffered from proven episodes of RAM while on colchicine and responded dramatically to treatment with anakinra. We compare this patient to previously described patients with FMF who contracted aseptic meningitis.

**Methods:** A systematic search of the literature was performed retrieving English-language original case reports, case series, case-based reviews, and review articles on aseptic meningitis in FMF patients, up to may 2022. Articles involving patients with FMF suffering from aspetic meningitis, recurrent or single episode – were included.

**Results:** In addition to our case, we identified seven cases describing aseptic meningitis in patients with underlying proven FMF; 6 of 7 cases were described in the adult population, with patient age ranging from 32 to 64 years of age, two (including our case) were in the pediatric population (13 and 14 years old). All cases in the literature responded well to colchicine treatment and aseptic meningitis episodes were diminished. Our case report is the first to document a resistance to colchicine and complete response to anti IL-1 treatment of RAM due to FMF.

**Conclusion:** Central nervous system (CNS) manifestations of FMF are rare and under debate. Primary headaches are common among FMF patients, and may develop due to the autoinflammation. There are several reports of headaches due to recurrent episodes of RAM presumed to be related to FMF, mainly in adults. In most cases, events subsided or resolved following colchicine treatment. however, reports are scarce and vary significantly in criteria for FMF diagnosis (with or without genetic proof).

According to Capron et al. Current diagnostic criteria for RAM related to FMF are: (a) RAM episodes due to FMF should be accompanied by other clinical or biological features of FMF attacks; (b) Colchicine prevents or lessens episodes; and (c) other classical causes of RAM (drugs, HSV2 infection, systemic diseases and cystic brain lesions) should be excluded. Our patient fulfilled both criteria for FMF and at least two of the criteria for RAM related to FMF. Only seven confirmed cases investigating association between FMF and RAM were found in the systemic review.

Colchicine is the main therapeutic modality for reducing inflammatory attacks and preventing amyloidosis due to FMF. Nevertheless, 30-40 percent of FMF patients continue to suffer from recurrent attacks despite therapy, and 5-10 percent are considered resistant to colchicine. Until recently there was no other known effective treatment for FMF.

Based on the role of pyrin in the regulation of interleukin (IL)-1β activation, the efficacy of IL-1 inhibitors has been assessed and well established in FMF patients who were resistant or intolerant of colchicine.

Our study is the first report of RAM due to FMF that is responsive to anakinra. The immediate response strongly suggests there was a relationship between FMF and RAM in this patient. Our experience also offers a novel therapeutic option for colchicine resistant RAM and maybe even other CNS manifestations of FMF.

**Patient Consent:** Not applicable (there are no patient data)

**Disclosure of Interest**: None declared

### P149. Music for CAPS: the experience of the centre for paediatric rheumatic diseases of Palermo

#### M. C. Maggio^1^, C. Scalisi^1^, F. Benfratello^2^, S. Amato^3^, G. Corsello^1^

##### ^1^University Department PROMISE “G. D’Alessandro”, ^2^University of Palermo, University of Palermo, ^3^Children Hospital “G. Di Cristina”, Arnas Palermo, Palermo, Italy

###### **Correspondence:** M. C. Maggio

**Introduction:** Noise is an environmental factor that can influence human health status. Any sound that causes stress, irritability or disorder on the normal course life can be defined as noise. The WHO, in 2011 defined noise pollution as the most important environmental public health risk factor after air pollution. On the contrary, music is universally recognized as an anti-stressor method, and can be used as a therapeutic strategy. Children with autoinflammatory diseases (AIDs), especially with CAPS, need to wait in the hospital during the days of day hospital for blood sampling and biological drugs parenteral administration, sometimes for many hours, waiting the results of blood sampling. Many patients refer malaise and fatigue the afternoon and the evening of the day hospital, as possible effect of tiredness and stress.

**Objectives:** To evaluate the music impact on wellness of patients with AIDs during the days of day hospital for blood sampling and biological drugs parenteral administration.

To empower the nurse’s role in the global care of patients with AIDs, as main actor of alternative strategies to improve the treatment compliance.

**Methods:** We engaged a musician nurse for the care of our patients with AIDs, especially with CAPS. The nurse played piano, involving patients to contribute with other instruments. In fact, in the following meetings, some patients chose to play music with the nurse in the waiting room in front of other patients.

**Results:** We collected the records of the effects of music therapy on their wellness perception, both during the hours of day hospital and after coming back home. Most of the patients referred a positive impact of music on the personal patient journey.

**Conclusion:** Patients with CAPS and generally with AIDs experience stress as a trigger for further attacks. Programs that improve care and support children and their families, through the complex way of AIDs, significantly improve the quality of life of patients with AIDs and their families. Healthcare providers should be aware of the impact of the long diagnostic journey on families and must work to create an environment of trust and collaboration facing a prolonged and difficult diagnostic process. Music can be considered as a winning strategy for supportive care, especially in children with CAPS or other AIDs, who experience stress as a trigger of disease attacks.

**Disclosure of Interest**: None declared

### P150. FMF or SURF: a diagnostic dilemma in children with recurrent febrile episodes

#### M. C. Maggio, M. Liuzzo Scorpo, R. Panzeca, G. Corsello

##### University Department PROMISE “G. D’Alessandro”, University of Palermo, Palermo, Italy

###### **Correspondence:** M. C. Maggio

**Introduction:** Familial Mediterranean Fever (FMF) is an inherited auto-inflammatory disorder and is still extremely underdiagnosed in the Mediterranean area.

**Objectives:** We collected a retrospective case series of children with recurrent febrile or inflammatory episodes and referred to the Children Hospital of Palermo.

**Methods:** We divided all the patients with a diagnosis of FMF of our centre in 2 groups.

Group A: 16 families with 24 children (15 M; 9 F) with the polymorphism R202Q (heterozygous in 14), (homozygous in 2), that show elevated inflammatory markers during the attacks, do not fulfil the PFAPA criteria, but respond to colchicine. They show the classical symptoms of FMF, fulfilling the Eurofever/PRINTO FMF classification criteria (fever, arthralgia, abdominal pain, rash, aphthous stomatitis, thoracic pain, with attacks induced by triggers as stress, fatigue, menses, etc). The children were treated with colchicine, 4 stopped colchicine after 4 years or more for the remission of clinical presentation and are still in remission. 2 children (8%) showed a partial response to colchicine, with the reduction but not the complete resolution of recurrent attacks. Parents were frequently symptomatic in childhood and in the 13% of the families, symptoms persisted in adulthood.

Group B: 16 families with 36 children (19 M; 17 F) carrying MEFV gene mutations (M694V: heterozygous in 2, heterozygous associated with R202Q in 2, homozygous in 1; A289V: heterozygous in 3; A744S: heterozygous in 2, heterozygous associated with P369S, R408Q in 1; V796T: heterozygous in 2; I591T, P369S, R408Q: heterozygous in 1; F479D, E167D, R202Q: heterozygous in 3; R348H, R202Q: heterozygous in 3; E148Q: heterozygous in 10; R408Q, P369S: heterozygous in 2; R716H: heterozygous in 1; P369S, R408Q, R202Q: heterozygous in 1; R716H: heterozygous in 2) were included.

**Results:** In group A, the children were treated with colchicine, 4 stopped colchicine after 4 years or more for the remission of clinical presentation and are still in remission. 2 children (8%) showed a partial response to colchicine, with the reduction but not the complete resolution of recurrent attacks. Parents were frequently symptomatic in childhood and in the 13% of the families, symptoms persisted in adulthood.

In group B, 7 children were asymptomatic; 29 were symptomatic before treatment, and treated with colchicine (27) or colchicine plus anakinra (1), or colchicine plus canakinumab (2). 4 patients stopped colchicine for the resolution of symptoms and a good control of the disease without treatment. They are still in follow-up. Their parents showed a different clinical presentation, and, in all the cases, the diagnosis was done after the genetic study of their children. In the 16 families, all the parents, except 1, did not show fever. Most of them, however, had anamnestic records of recurrent fever in childhood. Most of the parents had recurrent abdominal pain, arthralgia at the limbs, headache, fatigue. In children, the clinical presentation was typically characterized by recurrent attacks of fever, arthralgia, abdominal pain, rash, aphthous stomatitis, thoracic pain, with attacks induced by triggers as stress, fatigue, menses, etc, with no significant differences with the children of group A.

**Conclusion:** We can speculate that children in group A, carrying R202Q mutation, are patients with SURF (Syndrome of undifferentiated recurrent fever, as proposed by M. Gattorno and coll.). 92% responded to colchicine. The clinical presentation of the parents diverged from A to group B: most of the patients of group A do not refer a family history of recurrent fever and/or symptoms of SURF. Most of the patients of group B have a family history of recurrent fever in siblings and parents, and actual records of abdominal pain, arthralgia at the limbs, headache, fatigue.

**Disclosure of Interest**: None declared

### P151. Pediatric SAPHO syndrome: single entity or different diseases?

#### C. Matucci Cerinic^1,2^, G. Viglizzo^3^, R. Caorsi^2,4^, S. Volpi^1,2^, C. Malattia^1,4^, M. Gattorno^2,4^

##### ^1^DINOGMI, Università degli studi di Genova, ^2^Centro malattie autoinfiammatorie e immunodeficienze, ^3^UO Dermatologia, ^4^Clinica pediatrica e reumatologia, Istituto Giannina Gaslini, Genova, Italy

###### **Correspondence:** C. Matucci Cerinic

**Introduction:** SAPHO is a heterogeneous autoinflammatory disease, characterized by bone and joint involvement and by a wide variety of dermatologic manifestations, including palmo-plantar pustulosis (PPP), acne, hidradenitis suppurativa (HS), pyoderma gangrenosum (PG), psoriasis, Snedd-Wilkinson disease and Sweet syndrome. The SAPHO treatment still today is a challenge, and no therapeutic guidelines are available in children: NSAIDs are frequently used as first line treatment for CRMO lesions, followed by bisphosphonates, methotrexate and biologic therapies. In the literature there are many case reports but only a few case series, of which the present one is the largest in Europe (29 cases reported in China).

**Objectives:** to report the clinical and radiological features and the response to therapy of a cohort of SAPHO children

**Methods:** the clinical data (serological, imaging and therapy) of 13 SAPHO patients, followed between 2001 and 2021 at the Unit for Autoinflammatory diseases at the Gaslini Hospital were reviewed. Patients were divided into 2 groups according to their cutaneous manifestations in an acne-HS and in a PPP group.

**Results:** 8 patients presented an acne-HS while 5 patients had a PPP. In the former group, 7/8 patients were male, while in the latter group 5/5 patients were females. At disease onset, in the acne-HS group, skin manifestations were the first symptom, while in the PPP group were concomitant or subsequent to bone manifestations. In the PPP group, the cutaneous involvement was mild and responded well to topical treatments, while the bone involvement required a therapy with methotrexate or salazopirin, that induced remission in 4/5 patients. 1 PPP patient showed a refractory bone disease and underwent several biological therapies. The acne-HS skin involvement was characterized as follows: 7 patients presented with severe acneiform eruptions (pustular, nodular, cystic, comedonic and ulcerative lesions), of these, the 3 patients with comedonic acne presented also HS, and 1 PG. In the acne-HS group, the osteoarticular involvement responded well to conventional DMARDS, but the skin manifestations required a therapeutic upgrade on biologics. Etanercept obtained only a partial result in 5 patients, Adalimumab had an optimal response in 6 patients, in 1 obtained a partial response even if requiring an increased dose (80 mg per week) and 1 was swapped to dapsone. Anakinra (3), ustekinumab and secukinumab (2) were not efficacious. The 3 patients with comedonic acne were refractory to all treatments and needed therapy cycling, which achieved a partial remission only.

When comparing the 2 groups, sternal and axial involvement were more frequent in the acne-HS group (75% and 87,5% versus 40 and 60% respectively) while clavicular and mandibular involvement were more frequent in the PPP group (60% and 40% vs 25% and 0% respectively). An involvement of the metaphysis of the long bones was present in both groups, however in PPP a major involvement of the tibia and fibula was present (100 and 80% vs 62,5 and 50% respectively)

**Conclusion:** All our patients were characterized by well-known SAPHO clinical features. For what concerns skin involvement, all patients with comedonic acne were refractory to treatments while PPP and nodulo-cystic acne achieved remission. Interestingly, a difference in the bone manifestations was found in the 2 groups. Our observation may suggest that in SAPHO two different phenotypes exist. In fact, skin involvement may have a different response to the treatment, and comedonic acne seems to belong to a separate cluster of patients that poorly respond to treatments.

**Patient Consent:** Not applicable (there are no patient data)

**Disclosure of Interest**: None declared

### P152. Validation of the pediatric Behçet disease criteria (PEDBD): a consensus-based approach

#### C. Matucci-Cerinic^1,2^, H. Palluy^3^, S. Al-Mayouf^4^, P. Brogan^5^, L. Cantarini^6^, A. Gul^7^, O. Kasapcopur^8^, J. Kuemmerle-Deschner ^9^, S. Ozen^10^, D. Saadoun^11^, F. Shahram^12^, N. Ruperto^13^, M. Gattorno^14,15^, I. Kone-Paut^16^

##### ^1^Center for Autoinflammatory Diseases, IRCCS Istituto Giannina Gaslini, ^2^DINOGMI, Università degli studi di Genova, Genova, Italy, ^3^Pediatric rheumatology and CEREMAIA, Bicêtre hospital, APHP, Paris, France, ^4^Department of Pediatrics, King Faisal Specialist Hospital and Research Center; College of Medicine, Alfaisal University, Riyadh, Saudi Arabia, ^5^University College London Great Ormond Inst of Child Health, and Great Ormond Street Hospital NHS Foundation Trust, London, United Kingdom, ^6^Rheumatology Unit, Department of Medical Sciences, Surgery and Neurosciences, University of Siena, Siena, Italy, ^7^Division of Rheumatology, Department of Internal Medicine, Istanbul Faculty of Medicine, Istanbul University, ^8^Cerrahpasa Medical School, Istanbul University-Cerrahpasa Turkey, Istanbul, Turkey, ^9^Division of Pediatric Rheumatology, Department of Pediatrics and autoinflammation reference center Tuebingen, University Hospital Tuebingen, Tuebingen, Germany, ^10^Department of Pediatric Rheumatology, Hacettepe University, Ankara, Turkey, ^11^Sorbonne Universités, Department of Internal Medicine and Clinical Immunology, CEREMAIA, AP-HP, Groupe Hospitalier Pitié-Salpêtrière., Paris, France, ^12^Rheumatology Research Center, Shariati Hospital, Tehran University of Medical Sciences, Tehran, Iran, Islamic Republic Of, ^13^UOSID Centro Trial, IRCCS Istituto Giannina Gaslini , ^14^Center for Autoinflammatory Diseases, IRCCS Istituto Giannina Gaslini, ^15^Pediatrics and Rheumatology Clinic, IRCCS Istituto Giannina Gaslini, Genova, Italy, ^16^Pediatric rheumatology and CEREMAIA, Bicêtre hospital, APHP; University of Paris Saclay, Paris, France

###### **Correspondence:** C. Matucci-Cerinic

**Introduction:** Behçet disease (BD) is an autoinflammatory disease characterized by a variable vessel vasculitis. In children, the first BD symptoms may start early in life, mimicking other autoinflammatory diseases and making the diagnosis a real challenge in the paediatric population. While many criteria exist for the diagnosis or classification of BD in adults in children only classification criteria exist, the PEDBD criteria (2015), created by international Expert consensus.

**Objectives:** to perform an external validation of the PEDBD criteria and attempt to improve their sensitivity and specificity with respect to other autoinflammatory diseases.

**Methods:** 210 patients were randomly selected from the Eurofever Registry: patients were included if enrolled after 2010 with a diagnosis of BD, PFAPA, FMF, MKD, TRAPS, SURF and undefined autoinflammatory diseases (UND); patients were excluded if they participated in the first PEDBD study. A set of 11 Experts blinded to the original diagnosis, were chosen to evaluate the patients, and reach a consensus defined as > 80% for the following diagnosis: BD, PFAPA, FMF, MKD, TRAPS, SURF, UND. In the 1^st^ round only clinical and serological data were shown; in the 2^nd^ round genetic data were added; and in the 3^rd^ round the other Experts’ votes and comments were shared with all experts. Using the expert consensus as gold standard, the PEDBD, the ISG and the ICBD criteria were applied to BD patients and to the confounding diseases in order to define the sensitivity and specificity.

**Results:** In the 1^st^ round a consensus was reached in 45/210 (21%) of patients (22/70 BD, 5/35 FMF, 2/26 MKD, 11/40 PFAPA, 2/22 TRAPS, 2/12 SURF, 1/5 UND). While in the 2^nd^ round a consensus was reached in another 58/163 (35%) of patients, for a total consensus on 103/210 patients (49%): 31/70 BD, 17/35 FMF, 21/26 MKD, 15/40 PFAPA, 14/22 TRAPS, 3/12 SURF, 2/5 UND. The 3^rd^ round is now ongoing, and the data are not yet available. The next step will consist on the application of the PEDBD, the ISG and the ICBD criteria to the cohort of classified BD, and to the other diseases in order to see their performance on BD and confounding diseases.

**Conclusion:** the classification of Behçet disease showed a wide range of opinions among Experts. The heterogeneity of the disease is a challenge but a more robust classification can at least allow homogeneous groups of patients for research purpose.

**Patient Consent:** Not applicable (there are no patient data)

**Disclosure of Interest**: None declared

### P153. Pericarditis in children: systemic and isolated recurrent pericarditis

#### A. Mauro^1^, E. Bizzi^2^, A. M. Pisacreta^2^, V. Ansuini^1^, C. Carollo^2^, A. Pedroli^2^, F. Casini^3^, F. Savoia^4^, A. Brucato^5^, L. Bernardo^6^

##### ^1^Pediatric Rheumatology Unit, ^2^Department of Medicin, Rheumatology Unit, ASST Fatebenefratelli-Sacco, ^3^Department of pediatrics, Vittore Buzzi Children’s Hospital, Milan, ^4^Childhood Cancer registra of campania, Santobono-Pausilipon Children’s Hospital, Naples, ^5^Department of Medicin, ^6^Department of Pediatrics, ASST Fatebenefratelli Sacco, Milan, Italy

###### **Correspondence:** A. Mauro

**Introduction:** Pericarditis is a clinical syndrome characterized by inflammation of the pericardium with or without pericardial effusion. The course can be acute, recurrent or chronic. Recurrent pericarditis (PR) is defined as the recurrence of pericarditis after the first attack, with a symptom-free interval greater than 4-6 weeks. Etiopathogenesis of RP is idiopathic in 70% of pediatric cases, more rarely, it can be secondary to autoinflammatory, autoimmune diseases, tumors, metabolic diseases or infections. PR can occur isolated or in the context of a polyserositis with characteristics of systemic inflammation (fever, leukocytosis, pleural effusion and more rarely peritoneal).

**Objectives:** The aim of the study is to assess the differences in laboratory parameters between two groups of patient affected respectively by systemic recurrent pericarditis (PRs) and isolated recurrent pericarditis (PRi).

**Methods:** Patients diagnosed with PRs and PRi aged <18 years were included. Inclusion criterion was the presence of fever and pleural effusion, in patients with PRs. Demographics, clinical characteristics, and blood tests were collected and compared.

**Results:** We prospectively enrolled 42 patients (14 RPs and 28 RPi). The distribution by gender and age was similar in the two groups (M / F 1: 1; age of onset: 15 years (IQR 13-17). The PRs compared to the PRi had significantly higher levels of leukocytes, neutrophils and CRP (p < 0.01). In addition, patients with PRs performed pericardiocentesis more frequently (p <0.01)

**Conclusion:** Our study showed that in patients with PRs mechanisms of autoinflammation, probably mediated by IL1, which manifest themselves with elevated levels of CRP and neutrophilia, seem to play a fundamental pathogenetic role, compared to the PRi forms.

**Patient Consent:** Yes, I received consent

**Disclosure of Interest**: None declared

### P154. Idiopathic chronic pericardial effusion in pediatric age: the occasional finding in an asymptomatic patient

#### A. Mauro^1^, E. Bizzi^2^, L. Trotta^2^, M. Pancrazi^2^, F. Casarin^2^, V. Ansuini^2^, S. lassainato^3^, A. Brucato^4^, L. Bernardo^5^

##### ^1^Departments of Pediatrics, Rheumatology Unit, ^2^Departments of Medicin, Rheumatology Unit, ASST Fatebenefratelli-Sacco, ^3^Departments of Pediatrics, Vittore Buzzi Children’s Hospital, ^4^Department of Medicin, ^5^Department of Pediatrics, ASST Fatebenefratelli sacco, Milan, Italy

###### **Correspondence:** A. Mauro

**Introduction:** Chronic pericardial effusion of unknown etiology is a very rare event in pediatric age. The autoinflammatory etiology of acute and recurrent pericarditis often causes acute pericarditis and simultaneous pericardial effusion as an inflammatory epiphenomenon. Idiopathic pericardial effusions not associated with acute pericarditis in children is not reported in literature.

**Objectives:** We describe a case of pediatric patients with an Idiopathic, chronic and large pericardial effusion

**Methods:** Male patient, 9 years old, hospitalized for persistent fever and pharyngotonsillitis. On chest x-ray he presented cardiomegaly then confirmed by echocardiography which showed abundant circumferential pericardial effusion, with heart oscillating in the pericardial cavity, without signs of cardiac tamponade and normal inferior vena cava (VCI). He had no symptoms due to pericardial effusion.

**Results:** Pericardiocentesis was carried out with the withdrawal of 650 ml of citrine liquid. In suspicion of acute pericarditis, prednisone therapy (25 mg / day) was initiated without any benefit. Blood tests showed: PCR 147 mg / L, GB 20,000 / mm3, anti-Mycoplasma IgM positive.

Throat swab for SBEGA, autoimmunity, thyroid functions, troponin and Mantoux intradermal reaction were negative.

Discharged in good general conditions, he began therapy with indomethacin (50 mg / day).

After 7 days, for the finding of pericardial effusion of 1 cm, he combined therapy with Prednisone (37.5 mg / day) with little benefit on the extent of the effusion. For this reason he added Colchicine (1 mg / day), and made a slow decalage of the steroid.

Over the years he performed numerous echocardiographic exam that showed a slow increase in effusion of about 0.5-1 cm per year with non-dilated and normocollassing VCI, well tolerated.

At the last visit, about 7 years after onset, the patient reported full well-being. On physical examination, PA 100/70 mmHg, HR 60 bpm. Non turgor of the jugular, cardio-thoracic and abdominal examination were negative. Not organomegaly.

Echocardiography showed slightly dilated, normocollassing VCI; circumferential massive pericardial effusion, with the heart oscillating in the pericardial cavity. At the moment he does not perform any therapy.

**Conclusion:** It was not possible to clarify whether patient presented an effusion secondary to acute pericarditis, possibly autoinflammatory and / or due to mycoplasma infection, or an occasional finding of idiopathic pericardial effusion in the course of acute pharyngotonsillitis. Generally, acute pericarditis and related effusion respond to anti-inflammatory therapy, while occasional pericardial effusion does not respond to anti-inflammatory therapy and is often well tolerated. To our knowledge, this is the first case of idiopathic, asymptomatic, pericardial effusions not associated with acute pericarditis in pediatric age.

**Patient Consent:** Yes, I received consent

**Disclosure of Interest**: None declared

### P155. A case of recurrent pericarditis in a patient with Bardet-Biedl syndrome

#### A. Mauro^1^, S. Lassainato^2^, F. Casini^2^, E. Chittano Congedo^2^, V. Ansuini^1^, C. Carollo^3^, A. Pedroli^3^, F. Casarin^3^, A. Brucato^3^, L. Bernardo^4^

##### ^1^Department of Pediatrics, Rheumatology Unit, ASST Fatebenefratelli-Sacco, ^2^Department of Pediatrics, , Vittore Buzzi Childrem Hospital, ^3^Department of Medicin, ^4^Department of Pediatrics, ASST Fatebenefratelli-Sacco, Milan, Italy

###### **Correspondence:** A. Mauro

**Introduction:** Recurrent pericarditis (RP) is defined as the recurrence of pericarditis after the first attack, with a symptom-free interval greater than 4-6 weeks. Very often RP is idiopathic in pediatric age and more rarely, it can be secondary to autoinflammatory, autoimmune diseases, tumors, metabolic diseases or infection.

**Objectives:** We describe a case of Bardet-Biedl syndrome associate to RP

**Methods:** A. is a 9-year-old boy with Bardet-Biedl syndrome characterized by retinitis pigmentosa, polydactyly of the left foot, kidney cysts and learning difficulties.

He presented the first episode of pericarditis at the age of 12, successfully treated with colchicine. He also presented three further recurrences of pericarditis at a distance of 1 year each other.

During the last episode, due to the presence of large pericardial effusion, he performed pericardiocentesis with evacuation of 900 ml of serum-hematic liquid. Culture and cytological examinations of the pericardial fluid were negative.

The patient came to our attention at age 16 for retrosternal pain. At the visit he showed BP 130/90 mmHg, cardio-thoracic and abdominal physical examination negative. He performed blood tests (complete blood count, ESR, PCR, troponin, liver and kidney function, electrolytes) which were in normal range. Echocardiography showed the presence of a minimal effusion of the free pericardium wall (5 mm) with the presence of fibrin. Colchicine therapy (1 mg / day) was started.

**Results:** After two months he returned to our department with chest pain which worsened with movement. On physical examination the patient was in fairly good general conditions, BP 150/90 mmHg, HR 110 beats/minute. Negative cardio-thoracic and abdominal physical examination.

Blood tests were carried out and showed an increase of inflammation index (CRP 170 mg / L, ESR 80 mmHg) and white blood cells count (WBC) 10750 / mm3, Neutrophils 7920 / mm3. Anti-nucleus antibodies, ENA antibodies, anti-phospholipid antibodies and Mantoux test were performed and all were negative.

Chest X-ray and electrocardiogram were negative and Echocardiography revealed the presence of pericardial effusion (20 mm).

He started therapy with Indomethacin (50 mg / day), Prednisone (25 mg / day), Anakinra (100 mg / day).

Progressively, the patient showed an improvement of the clinical, laboratory and instrumental conditions and, gradually reduced steroid treatment.

**Conclusion:** To our Knowledge, this is the first case reported the association between Bardet-Biedl Syndrome and recurrent pericarditis. Given the brilliant response to therapy with Anakinra, an autoinflammatory etiology has been hypothesized for pericarditis.

Are Pericarditis there a new manifestation of Bardet-Biedl syndrome or a form of autoinflammatory pericarditis associated with it?

**Patient Consent:** Yes, I received consent

**Disclosure of Interest**: None declared

### P156. Current practice and research priorities in chronic non-bacterial osteomyelitis – an international survey directed to patients, families, and clinicians

#### M. Mohanna^1^, E. Roberts ^2^, L. Whitty^1^, J. Gritzfeld^1^, A. Theos^3^, P. Ferguson^4^, J. Preston^1^, C. Hedrich^1,2^

##### ^1^University of Liverpool, ^2^Alder Hey Children’s Hospital, Liverpool, United Kingdom, ^3^Georgetown University, Washington, DC, ^4^University of Iowa Stead Family Children’s Hospital, Iowa City, United States

###### **Correspondence:** M. Mohanna

**Introduction:** Chronic non-bacterial osteomyelitis (CNO) is a rare autoinflammatory bone disease that mainly affects children and young people. It is characterised by bone pain and reduced function (1). The pathophysiology of CNO is incompletely understood and there are no licensed treatments, diagnostic criteria, or outcome measures (2).

**Objectives:** The aim of this study was to explore and compare the experience of patients, their families, and clinicians with CNO, its diagnosis and treatment. Furthermore, opinions on research priorities were explored.

**Methods:** Questionnaires were designed in 2020 to capture patient (20 items) and clinician (24 items) experience with the diagnosis and treatment of CNO and to explore research priorities. Questionnaires were circulated internationally in 2021 (Google Form) among patients and families through the CNO Facebook group, shared by other patient groups on twitter including fight_CRMO, and mailing list from the CRMO Foundation, and among clinicians historically involved in CNO research and participating in international working groups on diagnostic and therapeutic approaches.

**Results:** We received 93 responses to the patient questionnaire*;* 23% were from young people affected by CNO, and 77% were from parents or carers on behalf of the young person they care for. There were 21 responses (27 contacted) from clinicians. The median time taken for the patient group to receive a diagnosis was 6 months (mean: 14 months). This matched the clinician perspective that estimated a diagnosis after 6 months. 56% of patients/families reported to have received alternative diagnoses before they received a CNO or CRMO diagnosis, most commonly infection (29%). The most common medications taken for CNO amongst the patient group were NSAIDs (34%), followed by bisphosphonates (28%). The most frequently prescribed by clinicians were NSAIDs, followed by bisphosphonates. Research priorities showed strong alignment between patients and clinicians. Pathophysiology was the most commonly mentioned research priority in both patient (45%) and clinician (34%) groups, followed by medication trials (18%, 33% respectively). Based on this survey, four topics were identified for further discussion between clinicians and patients/families at the inaugural “*International conference on CNO and autoinflammatory bone disease*”, held in Liverpool, UK in May 2022: pathophysiology, nomenclature, clinical trials, and psychosocial aspects and psychological support.

**Conclusion:** This survey compared clinician and patient/family experience with CNO to identify unmet need and research priorities. Results will inform roundtable discussions to guide future research and provide ongoing support and information for patients and their families.

*References:*

1. Zhao DY, McCann L, Hahn G, Hedrich CM. Chronic nonbacterial osteomyelitis (CNO) and chronic recurrent multifocal osteomyelitis (CRMO). Vol. 4, Journal of Translational Autoimmunity. 2021.

2. Hedrich CM, Morbach H, Reiser C, Girschick HJ. New Insights into Adult and Paediatric Chronic Non-bacterial Osteomyelitis CNO. Curr Rheumatol Rep. 2020.

**Patient Consent:** Yes, I received consent

**Disclosure of Interest**: None declared

### P157. Sacroiliitis as a presenting symptoms in a young patient with FMF

#### M. Nashawi^1^, A. Sabo^2^, F. Blankenburg^2^, A. Heinkele^3^, P. Müller-Abt^4^, T. Hospach^3^

##### ^1^Pediatric Rheumatology, King Abdulaziz University, Jeddah, Saudi Arabia, ^2^Klinikum Stuttgart, Stuttgart, Germany, ^3^Pediatric Rheumatology, ^4^Pediatric Radiology, Klinikum Stuttgart, Stuttgart, Germany

###### **Correspondence:** M. Nashawi

**Introduction:** Growing pains (GP) are frequent in children. They usually encompass mostly bilateral, nocturnal pains, with changing localization, especially in the lower extremities, which respond well to symptomatic therapy and resolve until the next day. It is however a diagnosis of exclusion, and selected cases warrant further investigation.

**Objectives:** Our objective is to point out that growing pain is a diagnosis of exclusion by presenting a case that had been falsely diagnosed as a growing pain in the beginning.

**Methods:** we present a case report.

**Results:** We present a 3-yo girl with recurrent pain of the lower extremities which was initially diagnosed with growing pains. Upon further investigation, we found severe bilateral sacroiliitis. Considering the mediterranean origin of the family we sought to investigate the MEFV Gene and found a homozygous single nucleotide mutation strongly associated with familial mediterranean fever (FMF).

**Conclusion:** Especially in the pediatric population, sacroiliitis (SI) is a rare and uncommon localization for FMF-associated arthritis. In this case, the patient was very young and nocturnal pains were the only manifestation of this disorder. In the population with a susceptible genetic background, we recommend further investigation of limb and lower back pains, to rule out FMF-associated arthritis.

**Patient Consent:** Yes, I received consent

**Disclosure of Interest**: None declared

### P158. A case of adenosine deaminase 2 deficiency (DADA2) with an uncommon complex phenotype: a diagnostic challenge

#### M. F. Natale^1^, C. Celani^2^, S. Federici^2^, F. De Benedetti^2^, A. Insalaco^2^

##### ^1^Rheumatology, Bambino Gesu Children’s Hospital, ^2^Rheumatology, Bambino Gesù Children’s Hospital, Rome, Italy

###### **Correspondence:** M. F. Natale

**Introduction:** Deficiency of adenosine deaminase type 2 (DADA2) is an autosomal recessive disease caused by loss-of-function mutations of the ADA2/CECR1 gene which encodes adenosine deaminase type 2 (ADA2). The disease is associated with a highly variable clinical presentation, such as vasculitis of small- and medium-sized arteries, systemic inflammation, skin involvement, hematologic manifestations, immunodysregulation and neurological ischemic or hemorrhagic stroke. It is important to arrive at an early diagnosis in order to start an effective therapy to control clinical manifestations and reduce risk of complications.

**Objectives:** We described a 16 years old male patient with a genetically confirmed DADA2 characterized by a complex clinical phenotype.

**Methods:** Next generation sequencing

**Results:** The patient was first seen at our center at age 16 with a clinical history, started about 1 year earlier, characterized by intermittent fever (mainly serotin), persistent increase of inflammatory markers, splenomegaly (14cm) and diffuse lymph-node enlargement. At home, empiric antibiotic and glucocorticoid therapy was attempted without benefit. In family history, an older brother died when he was 12 years old with an undefined hyperinflammatory syndrome.

In our unit we performed extended microbiological tests resulted negative. Circulating autoantibodies were absent. Radiological exams confirmed splenomegaly and enlarged lymph-nodes in supra and subdiaphragmatic areas with 18F-FDG enhancement at PET-TC. In the suspicion of lymphoproliferative disease a lymphonode biopsy was performed (resulting in reactive lymphadenopathy) and bone marrow aspiration did not reveal atypic cells. Immunological exams revealed an immunodisregulation with a low level of circulating immunoglobulins (IgG 861 mg / dl; IgA 54 mg / dl, IgM 30 mg / dl) and a reduction of total count of memory B cells.

Given the complex clinical picture and the family history, a large NGS diagnostic panel for autoinflammatory diseases and immunodeficiencies was performed revealing the homozygous c.1358A>G mutation in ADA2 (CECR1) gene described in literature as pathogenetic.

We performed a brain MRI to exclude neurological involvement and then started biological therapy with Anti-TNF alfa (Etanercept) with a rapid and brilliant clinical and instrumental response.

**Conclusion:** This case emphasize the importance to consider a diagnosis of DADA2 in complex clinical cases characterized by systemic inflammation and/or immunedysregulation

**Patient Consent:** Yes, I received consent

**Disclosure of Interest**: None declared

### P159. Treatment of hematologic manifestations in a group of patients with monogenic autoinflammatory diseases (AID): single center experience

#### Z. Nesterenko^1^, A. Kozlova^1^, V. Burlakov^1^, A. Laberko^1^, Y. Rodina^1^, E. Viktorova^1^, E. Deordieva^1^, A. Moiseeva^1^, A. Mukhina^1^, N. Kuzmenko^1^, D. Bogdanova^1^, A. Khoreva^1^, D. Yukhacheva^1^, V. Bludova^1^, N. Kan^1^, D. Balashov^2^, A. Shcherbina^1^

##### ^1^Immunology, ^2^Hematopoietic Stem Cell Transplantation , Dmitry Rogachev National Medical Research Center Of Pediatric Hematology, Oncology and Immunology, Moscow, Russian Federation

###### **Correspondence:** Z. Nesterenko

**Introduction:** AIDs are a group of diseases caused by dysregulation of mechanisms controlling innate immune response and characterized by systemic inflammation and a plethora of manifestations, including hematological symptoms.

**Objectives:** To analyze hematological manifestations and their response to treatment in a group of monogenic AIDs.

**Methods:** We retrospectively analyzed data of 31 patients (12- females, 19- males) in whom persistent hematological symptoms were part of their AID phenotype.

**Results:** The forms of AIDs with hematological symptoms included: PSTPIP1-associated syndrome (PAID) - 8, adenosine deaminase 2 deficiency (DADA2) - 8, type I Interferonopathies - 8, mevalonate kinase deficiency (MKD) - 4, A20 haploinsufficiency (HA20) - 1, POMP-related autoinflammation and immune dysregulation (PRAID) – 1, NLRC4-associated autoinflammatory disorder – 1 (AIFEC).

Median age of onset of any disease symptoms was 2 months (range 0–16 years). Median age of onset of hematologic manifestations was 2.5 years (range 0–33 years). Hematological manifestations as the first symptom of AID were seen in 23/31 patients. Median age at diagnosis was 8 years (range 0.5-33), with the mean diagnostic delay of 4 years (range 0.5-32).

9/31 patients had three-lineage cytopenia, 12/31 – two-lineage cytopenia and 5/31 - single lineage cytopenia. Also myelodysplastic syndrome was seen in 1/31 and hemophagocytic lymphohistiocytosis in 4/31.

Jak-inhibitors monotherapy were effective in 3/11 cases with interferonopathies, in 1/11- with DADA2, and in 1/11 - with HA20. Combination of an IL-6 inhibitor and JAK-inhibitor had effect in 1 patient with DADA2 (Tabl.1).

Anti-IL1 therapy was successful in 3 patients with MKD and 1 patient with AIFEC.

Immunosuppressive therapy (steroids, high-dose IVIG, mycophenolate mofetil, cyclosporine A, methotrexat) were not effective in any of the patients treated.

Hematopoietic stem cell transplantation (HSCT) was performed in 5 patients (3 – with PAID, 2- with MKD), the median follow-up time after HSCT is 2.16 years (1,08–4,58).

Four patients are alive, with predominantly donor chimerism, good immune function and complete absence of the disease symptoms.

**Conclusion:** The hematological manifestation of AIDs represent a therapeutic challenge. HSCT might be a viable treatment option.

**Disclosure of Interest**: None declared

**Table 1 (abstract P159).** Treatment of patients with monogenic AIDs with hematological symptoms

Therapy	Patient (n)	Hematological response		
		full	partial	no effect
Jak-inhibitors	11	5	2	4
Anti-TNFα	5	0	2	3
Anti-IL1/ Anti-IL6	11/4	4/0	2/0	5/4
Jak-inhibitors+Anti-TNFα	3	1	1	1
Jak-inhibitors+Anti-IL6	3	0	1	2
Anti-IL6+ Sirolimus	2	0	1	1
Jak-inhibitors+rituximab+ Anti-IL6	3	0	2	1
Jak-inhibitors+rituximab+ Steroides	4	1	3	0

### P160. Long-term efficacy and safety of canakinumab in patients with hids (Hyper-IGD Syndrome) - interim analysis of the reliance registry

#### P. T. Oommen^1^, T. Kallinich^2^, J. Rech^3^, N. Blank^4^, J. Weber-Arden^5^, J. B. Kuemmerle-Deschner^6^

##### ^1^Clinic of Pediatric Hematology, Oncology and Clinical Immunology, Heinrich-Heine-University Duesseldorf, Duesseldorf, ^2^Department of Pediatrics, Division of Pulmonology, Immunology and Critical Care Medicine, Charité University Medicine Berlin, Berlin, ^3^Department of Internal Medicine 3 - Rheumatology and Immunology, Friedrich-Alexander University (FAU) Erlangen-Nuernberg and Universitaetsklinikum Erlangen, Erlangen, ^4^Rheumatology, University Hospital Heidelberg, Heidelberg, ^5^NOVARTIS, Nuernberg, ^6^Department of Pediatrics, Division of Pediatric Rheumatology, University Hospital Tuebingen, Tuebingen, Germany

###### **Correspondence:** J. Weber-Arden

**Introduction:** Hyper-IgD Syndrome/Mevalonate Kinase Deficiency (HIDS/MKD) is a rare autoinflammatory disease caused by a defect in the gene encoding mevalonate kinase. Treatment with the interleukin-1β inhibitor canakinumab (CAN), which since 2017 has been approved for use in HIDS/MKD patients, resulted in rapid remission in most patients, both in clinical trials and in practice.

**Objectives:** The present study investigates the long-term efficacy and safety of CAN under routine clinical conditions in pediatric (age ≥2 years) and adult HIDS/MKD patients.

**Methods:** RELIANCE is a prospective, non-interventional, multicenter observational study based in Germany with a follow-up period of up to 7 years. Patients with clinically confirmed HIDS/MKD diagnosis who routinely receive CAN will be enrolled in the study and followed at 6-month intervals.

**Results:** The present interim analysis includes baseline data from 8 HIDS/MKD patients enrolled in the study through December 2021 and preliminary 18-month data. Of these patients, N=5 (63%) were female and the median age at study entry was 8 years (2-39 years). The median duration of prior CAN treatment at study entry was 1.5 years (0-5 years). Standard, low-dose, and high-dose CAN treatment was evenly distributed at each interval.

Preliminary results indicate stable remission and disease control (as assessed by physicians and patients as well as laboratory parameters; Table 1). Overall, adverse drug reactions occurred in N=4 patients, but none were considered serious. During the study, N=4 patients underwent vaccinations, with one vaccination reaction classified as non-therapeutic occurring after a DTP (diphtheria, tetanus, pertussis) vaccination.

According to mutational analysis, pathogenic or likely pathogenic mutations (some homozygous) were present in N=7 patients. In no patient disease activity was considered to be severe.

**Conclusion:** Baseline characteristics and preliminary data from HIDS/MKD patients in the RELIANCE study suggest good disease control. In addition, interim analysis at 18 months revealed no unexpected safety concerns. In relation to vaccinations, vaccination reactions unrelated to CAN therapy occurred in only one case.

**Disclosure of Interest**: P. T. Oommen Grant / Research Support with: Novartis, T. Kallinich Speaker Bureau with: Roche, J. Rech Grant / Research Support with: Novartis, Sobi, Consultant with: AbbVie, Biogen, BMS, Chugai, GSK, Janssen, Lilly, MSD; Mylan, Novartis, Roche, Sanofi, Sobi, UCB, Speaker Bureau with: AbbVie, Biogen, BMS, Chugai, GSK, Janssen, Lilly, MSD; Mylan, Novartis, Roche, Sanofi, Sobi, UCB, N. Blank Grant / Research Support with: Novartis, Sobi, Consultant with: Novartis, Sobi, Lilly, Pfizer, Abbvie, BMS, MSD, Actelion, UCB, Boehringer-Ingelheim, Roche, J. Weber-Arden Employee with: Novartis, J. B. Kuemmerle-Deschner Grant / Research Support with: Novartis, AbbVie, Sobi, Consultant with: Novartis, AbbVie, Sobi

**Table 1 (abstract P160).** Baseline characteristics and interim analysis data of patients with HIDS

	Baseline (N=8)	12 months (N=6)	18 months (N=4)	
Number of patients (consecutive patient number #1-8) in disease remission (physician’s assessment)	4 (#1,3,5,6)	4 (#1,2,5,6)	3 (#2,5,7)	
Patient assessment of current disease activity (d) and fatigue (f); 0–10, median (min; max)	d: 0 (0; 7) f: 2.5 (0; 7)	d: 0,0 (0; 8) f: 1.0 (0; 4)	d: 0,0 (0; 0) f: 1.0 (0; 2)	
Number (%) of patients with days absent from work/school within the last 6 months	2 (25)	0 (0)	1 (25)	
CRP / SAA, median (mg/dl)	0.2 / 0.6	0.3 / 0.8	2.1 / 0.5	
**Mutation(s), disease activity, CRP/SAA value and dosage per patient**	**Mutation**	**Disease activity** (absent, mild/moderate, severe)	**CRP / SAA** (mg/dl)	**Dosing** mg (mg/kg)*, per 4 weeks
Patient #1	n.a.	Absent	0.1 / 0.5	300
Patient #2	V203A / I268T	mild / moderate	89.0 / 0.7	150
Patient #3 (MKD)	V377I / E284Kfs*17	mild / moderate	0.0 / 0.3	102 *(6.2 mg/kg)*
Patient #4	V377I / E93fs*38	mild / moderate	0.4 / 2.6	65 *(5.6 mg/kg)*
Patient #5	G212R / G212R	Absent	1.1 / -	150 *(7.7 mg/kg)*
Patient #6	V377I / V377I	absent	0.1 / 0.3	60 *(1.6 mg/kg)*
Patient #7	V357I	Absent	0.1 / 3.6	100 *(4.4 mg/kg)*
Patient #8	V377I / I268T	mild / moderate	0.5 / 0.7	150

CRP, c-reactive protein; n.a., not annotated; SAA, serum amyloid A

*In parentheses: Dosage in mg/kg for patients <40 kg

### P161. The usefulness of the genetic study in the clinical practice of monogenic autoinflammatory diseases: from diagnosis to the application of precision medicine

#### J. M. Garcia Aznar ^1^, N. Palmou Fontana^2^, N. P. Gandeaga^3^, J. G. Ocejo-Viñals^4^, M. A. Gonzalez-gay^2^

##### ^1^Inmunology, Health in code, A Coruña, ^2^Rheumatology, ^3^IGENETISTA, ^4^Inmunology, Universitary Marques De Valdecilla Hospital, Santander, Spain

###### **Correspondence:** N. Palmou Fontana

**Introduction:** The monogenic autoinflammatory disease accounts for at least 10% of cases with the suspected autoinflammatory syndrome. inflammasome complex, overproduction of IL-1β and IL-18. Different inflammasomes have been described in which genetic defects have been identified in their protein components, resulting in the development of Pyrin-associated inflammasopathy (MEFV), Cryopyrin (NLRP3), NLRP1, NLRC4, PSTPIP1, WDR1, LPIN2, IL1RN, and IL36RN. Another group is the interferonopathies, which include three types depending on the class of interferon involved in the dysregulation: IFNα, IFNβ, IFNγ, and IFNλ. The genes involved have JAK1/2, TYK2, nucleic acid degradation-associated genes such as TREX1, IFIH1, DNASE1/2/L3, and proteasome genes (POMP, PSMA3, PSMB8/9/10, PSMG2), among others. Genes associated with deregulation of the NF-kB/TNF pathway (NF-kBopathies) also result in autoinflammatory disorders characterized by aberrant activation of NF-kB (NFkbopathies), including TNFAIP3, TNFRSF1A, OTULIN, LUBAC complex genes, NOD2, ADA2, or RIPK1 among others. Additionally, other genetic factors not included in any of the above categories can affect both intrinsic and adaptive immunity genes with an autoinflammatory component (COPA, PLCG2, LRBA, C2).

**Objectives:** Defining and identifying the phenotypic spectrum associated with these diseases is challenging for clinical practice. The clinical manifestations, although heterogeneous, allow defining criteria for the categorization of autoinflammatory disease.

**Methods:** Ninety-nine patients with suspected autoinflammatory diseases were included in the cohort, with a panel containing more than 270 genes. According to the genetic result, the subjects were categorized into five groups: inflammasome disorders, interferon disorders, NFkB/TNF alteration, unfavorable genetics, and other pathologies. A group of patients phenotype divided into eight categories.

**Results:** The study results revealed that 48% of the studies performed with suspected autoinflammatory disease presented relevant genetic findings. The genetic variants identified were distributed in the following genes from higher to lower frequency: MEFV (21%), NOD2 (9%), PSTPIP1 (9%), NLRP12 (9%), TNFRSF1A (6%), TNFAIP3 (6% ), NLRC4 (4%), JAK1 (4%), NLRP3 (4%), TNFRSF1A (2%), ADA2 (2%), OTULIN (2%), TNFRSF11A (2%), BANK1 (2%), C2 (2%), PLCG2 (2%), PSMB8 (2%), LRBA (2%), NLRP1 (2%), POMP (2%), RNF31 (2%). 23% contained genes associated with inflammasome disorders, 4% interferon disorders, 16% NF-kb disorders, and 3% genes from other metabolic pathways. The mean age of disease onset was lower in patients with NF-kb-pathway disorder. Overall was lower in all categories than the studies that did not have relevant genetic variants.

Among patients with the autoinflammatory genetic disease, febrile and gastrointestinal manifestations were more frequent in 0-11 years. In contrast, rash and joint manifestations appeared more frequently in patients with 36 years old

**Conclusion:** The genetic study turned out to be a helpful tool for identifying the altered inflammatory pathway, which is an essential element for selecting treatment. Knowledge of the clinical presentation of each age and group of diseases adds value to selecting patients with the suspected autoinflammatory disease.

**Disclosure of Interest**: None declared

### P162. Adenosine deaminase 2(DADA2) deficiency in five indian children : a single center experience

#### D. B. Pandya, M. Agarwal, S. Sawhney

##### Pediatric Rheumatology Department, Sir Gangaram Hospital, New Delhi , India

###### **Correspondence:** D. B. Pandya

**Introduction:**

Deficiency of ADA2(DADA2) is the first molecularly described monogenic vasculitis syndrome which is caused by autosommal recessive loss of function mutations in the ADA2 gene(CERC1). Deficiency of ADA2 has been linked to an imbalance in differentiation of monocytes towards proinflammatory M1 macrophages, neutrophil extracellular traps(NETosis) dysregulation and endothelial dysfunction. DADA2 can present with Vasculitis or Vasculopathy , Bone marrow failure , Lymphoproliferation , Immunodeficiency and Type 1 interferonopathy.^1^

**Objectives:**

We aim to unveal Characteristics of DADA2 deficiency in five children at Our Center.

**Methods:**

This is a retrospective analysis of five children who were treated at our unit between August 2013 & August 2021 for severe systemic polyarteritis nodosa. In view of resistent disease in these children , ADA2 gene analysis was ordered and returned Positive.

**Results:**

**Conclusion:**

Early age of onset, female sex, severe disease activity mainly affecting gastrointestinal system , recurrent and resistant course in polyarteritis nodosa may suggest monogenic vasculitis syndrome - DADA2 deficiency.

**Trial registration identifying number:**

1. J Clin Immunol. 2018, Published online 2018 Jun 27. doi: 10.1007/s10875-018-0525-8

Deficiency of Adenosine Deaminase 2 (DADA2): Updates on the Phenotype, Genetics, Pathogenesis, and Treatment

Isabelle Meyts and Ivona Aksentijevich

**Patient Consent:** Yes, I received consent

**Disclosure of Interest**: None declared

**Table 1 (abstract P162).** See text for description

Mean age at Onset	5.2 years
Sex Ratio (F:M)	1.5:1
Livedo Reticularis	5/5(100%)
Nodular Skin lesions	3/5(60%)
Myalgia	2/5 (40%)
Hypertension	5/5 (100%)
Severe Abdominal Pain	5/5(100%)
Gastrointestinal Ulcers	3/5(60%)
Gastrointestinal Perforation	3/5(60%)
Splenic or Renal Infarcts	3/5(60%)
Central Nervous System Infarcts	1/5(20%)
Peripheral Neuropathy	3/5 (60%)
Angiographic Evidence of Medium Vessels Vasculitis	5/5(100%)
Hematological involvement , Evidence of Immunodeficiency & Type 1 Interferonopathy	0/5
Average delay between diagnosis of PAN and DADA2 deficiency	7 years
Intravenous Cyclophospahmide	5/5(100%)
Oral Azathioprine	5/5(100%)
Intravenous Immunoglobulin	3/5(60%)
Subcutaneous Adalimumab	1/5 (20%)
Intravenous Infliximab	1/5(20%)
Inactive disease	3/5(60%)
Resistent Disease	1/5(20%)
Death	1/5(20%)

### P163. Chronic non-bacterial osteomyelitis (CNO): a study from a single center in India

#### D. Patro^1^, A. Abraham^1^, J. Raghuram^2,3^, A. P. Rao^1,3^

##### ^1^Pediatric Rheumatology, Manipal Hospital, ^2^Pediatric Rheumatology, Aster Whitefield Hospital, ^3^Pediatric Rheumatology, Indira Gandhi Institute of Child Health, Bengaluru, India

###### **Correspondence:** D. Patro

**Introduction:** First described in 1972 by Giedion et al, Chronic nonbacterial osteomyelitis (CNO) is a rare autoinflammatory bone disorder that presents as recurrent bone pain in one or multiple sites.

**Objectives:** 1. To understand the clinical profile of CNO patients from Indian subcontinent

2. To elucidate the clinical outcomes of the children affected with CNO.

**Methods:** We retrospective reviewed 38 cases diagnosed as CNO as per Bristol Diagnostic criteria in the Pediatric rheumatology clinic in Manipal hospital, Bengaluru, India after obtaining ethical approval.

**Results:** The findings are summarized in the table below. Of the 164 multifocal lesions detected by WB- MRI in 33 cases, tibial lesions (26%) were the commonest. LPIN2 deficiency was also reported in two cases. All cases were initiated with naproxen. 27 patients were started on methotrexate of which 2 cases each responded to Bisphosphonates and TNF inhibitor respectively. 4 out of 25 cases required additional TNF inhibitor after pamidronate and methotrexate therapy. 2 cases responded well to pamidronate infusion alone. 8 children had frequent flares. 14 children are in remission on drugs and 12 children are in remission off drugs. 2 cases are lost to follow up.

**Conclusion:** With increasing knowledge and timely referral to a rheumatology center may bridge the gap to delay diagnosis. WB- MRI can detect bone lesions and prevents unnecessary invasive procedures. In a resource-limited setting, methotrexate and pamidronate have helped in achieving remission. TNF inhibitors are helpful in resistant cases with good response.

**Patient Consent:** Yes, I received consent

**Disclosure of Interest**: None declared

**Table 1 (abstract P163).** See text for description

Parameter	Our study (n=38) 2022	Surendran et al (n=20) 2018	Roderick et al (n=41) 2016	Chandrika et al (n= 131) 2018	German NPRD (n=774) 2020
Median age of onset of symptom in years	7.3	10.25	9	9.5	11.1
Median age of Diagnosis in years.	8.86	11.75	11	10.7	NA
Time to diagnosis in months	12	17.75	15	12	8.4
Female	26.4%	25%	75.6%	71.8%	62.8%
Median ESR:	44.5	43	27	NA	18.7
Multifocal	86.84%	NA	76%	83.2%	63%
Total no of Bone lesions by WB- MRI	169	100	162	405	NA
Tibial/ Fibula lesions	26+ 14.8 %	10 %	25+ 5 %	NA	120 + 29 cases
Mean duration of follow up in months	27.63	11.5	NA	NA	12

### P164. Pyoderma gangrenosum- mirror of active autoimmune disease

#### A.-M. Ramazan^1^, A. Apostol^2^, S. Mehdi^3^

##### ^1^Rheumatology Department, ^2^Paediatric Onco-Hematology , ^3^Dermatology Department, Emergency County Clinical Hospital “Sf Apostol Andrei”, Constanta, Romania

###### **Correspondence:** A.-M. Ramazan

**Introduction:** Pyoderma gangrenosum (PG) is a rare neutrophilic dermatosis that presents with rapidly developing, painful skin ulcers hallmarked by undermined borders and peripheral erythema. The cause of PG is not well understood, but PG is generally considered an autoinflammatory disorder. Children hospitalized in US for PG were found to have higher odds of thyroid disease, inflammatory bowel disease, hematologic malignancy, and other autoimmune disorders.

**Objectives:** To report a rare autoinflammatory and autoimmune condition with therapeutical difficulties

**Methods:** A 13-year-old girl without any past medical history was sent to our clinic for consultation and treatment of refractory pyoderma gangrenosum (PG) of bilateral lower extremities.

**Results:** Our patient had incomplete therapeutical response with high doses of prednisone indicated by our dermatologist. We tried to add Cyclosporine (CsA), accordingly to the first line of therapy in PG, followed by a significant flare of dermatological and biliary involvements (only hepatic cytolysis), as our best option remained Azathioprine with progressive increasing doses. The abnormal liver tests were obviously under CsA when they had a peak, a clear sign of worsening and an autoimmune involvement.

**Conclusion:** PG could be more than a dermatological disease and the rheumatologist must look for the underlying autoimmune disease, a required diagnosis for the adjustment of therapy.

**Patient Consent:** Yes, I received consent

**Disclosure of Interest**: None declared

**Table 1 (abstract P164).** See text for description

Date	ESR (mm/h)	ASMA (N<20 ui/ml)	ALT (u/l)	ANA	Prednisone	DMARD
Nov 2019	90	-	45	1/640	0.7 mg/kgb/day	-
Feb 2020	89	-	30	-	0.35 mg/kgb/day	-
May 2020	103	-	63	1/640	0.35mg/kgb/day	AZA 0.8mg/kgb/day
Sept 2020	64	-	65	-	5mg/day	AZA 1.25mg/kgb/day
Nov 2020	75	-	72	1/320	2.5mg/day	AZA 1.5mg/kgb/day
Feb 2021	58	-	38	1/320	2.5mg/day	CsA 2.5mg/kgb/day
Jul 2021	65	82.32	150	1/320	0.5mg/kgb/day	AZA 2.5 mg/kgb/day
Nov 2021	47	65.21	44	1/160	10mg/day	AZA 2.6mg/kgb/day
Feb 2022	26	-	14	1/160	5mg/day	AZA 2.7mg/kgb/day

### P165. Criteria of remission and risk factors for disease severity in long-term follow-up of children with chronic non-bacterial osteomyelitis

#### C. Reiser^1^, J. Klotsche^2,3^, T. Hospach^4^, G. Heubner^5^, D. Windschall^6,7^, R. Trauzeddel^8^, N. Grösch^2^, M. Niewerth^2^, K. Minden^2,3^, H. Girschick^9^

##### ^1^Department of Pediatrics, LKH Bregenz, Bregenz, Austria, ^2^Deutsches Rheuma-Forschungszentrum Berlin, ein Institut der Leibniz-Gemeinschaft, ^3^Charité – Universitätsmedizin Berlin, corporate member of Freie Universität Berlin, Humboldt-Universität zu Berlin, and Berlin Institute of Health, Berlin, ^4^Department of Pediatrics, Olgahospital, Klinikum Stuttgart, Stuttgart, ^5^Städtisches Klinikum Dresden-Neustadt, Klinik für Kinder- und Jugendmedizin, Dresden, ^6^Clinic for Pediatric and adolescent Rheumatology, St. Josef-Stift, Sendenhorst, ^7^University of Halle –Wittenberg, Halle, ^8^Fachambulanz Kinderrheumatologie, Helios Klinikum Berlin-Buch, Klinik für Kinder- und Jugendmedizin, ^9^Vivantes Klinikum Friedrichshain, Children’s Hospital, Berlin, Germany

###### **Correspondence:** C. Reiser

**Introduction:** Chronic non-bacterial osteomyelitis (CNO) is an autoinflammatory bone disease of unknown origin. A 5 year longitudinal analysis of patients in the German National Pediatric Rheumatologic Database (NPRD) with CNO was performed.

**Objectives:** To document the long term course and to assess risk factors for severe disease and items defining remission of patients with CNO.

**Methods:** From 2015-2020 all patients with a confirmed diagnosis of CNO, who were registered in the NPRD during their first year of disease course and at least one follow-up visit, were included in this analysis.

**Results:** Additional to the previously reported cross-sectional analysis of almost 800 CNO patients, a data set of 314 eligible patients including up to 5 years of follow-up visits was analysed. Selected patients’ characteristics are as follows: 64% of the patients were female; the medium age was 11.2 years. 10.8% of patients were HLA-B27 positive. The mean number of clinical lesions was 2.2; using whole-body MRI imaging, the mean number was 2.9 lesions per patient at diagnosis. Co-manifestation of the skin was present in 17%. MRI-scans (whole-body) were performed in 255 patients, and most of them (86%) showed a positive TIRM/STIR signal.

The analysis of the location of the lesions showed that the distribution changed over the years. Whereas in the first year of disease course sites like vertebrae or mandibula were inflamed (8% and 2% of all affected sites), these sites completely resolved over time, while other sites like pelvis or tibia (18%/20%) remained affected after 5 years. Initially, patients were mainly treated with non-steroidal anti-rheumatic drugs (NSAIDs). During disease course the proportion of patients treated with conventional or biological disease modifying anti-rheumatic drugs (DMARDs) raised during these five years from 37% to 54%, or 5% to 12.5% respectively. Inflammation of pelvis or femur at baseline posed an elevated risk for long term severe disease (OR 1.51/1.61, each p=0.0001). Other risk factors for severe disease (defined as physician reported disease activity (PRDA) ≥4 (Visual Analogue Scale 0-10)) were raising numbers of lesions, elevated ESR (erythrocyte sedimentation rate) and multifocal disease (defined as more than one lesion). The risk for arthritis increased with the initially higher number of lesions. Items defining remission best were PRDA <1, patient reported pain score and number of radiologically defined inflammatory bone lesions. The composite Childhood health assessment score (C-HAQ) and patient reported overall well-being did not correlate with inactive disease.

**Conclusion:** The NPRD long-term cohort documents a large number of children and adolescents with CNO. Most of the patients were treated effectively with non-steroidal anti-inflammatory drugs, second-line treatment are disease-modifying agents, steroids or bisphosphonates. An improvement of patient-, physician- and imaging-defined disease activity measures was documented, suggesting CNO generally as benigne disease with a modest number of complicated disease course. Remission defining items and risk factors for severe disease course were defined.

**Patient Consent:** Yes, I received consent

**Disclosure of Interest**: C. Reiser Grant / Research Support with: Pfizer, Speaker Bureau with: Novartis, J. Klotsche: None declared, T. Hospach: None declared, G. Heubner: None declared, D. Windschall: None declared, R. Trauzeddel: None declared, N. Grösch: None declared, M. Niewerth: None declared, K. Minden Speaker Bureau with: Pfizer, Novartis, and Medac, H. Girschick: None declared

### P166. Alone we can diagnose so little; together we can diagnose so much! when multispecialty collaboration spotlights PFIT and autoinflammation

#### A. Remy^1^, W. Abou Chahla ^2^, G. Boursier^3^, M. Le Coniac ^4^, B. Catteau^5^, H. Reumaux^6^

##### ^1^General pediatrics, ^2^Hematology, CHU Lille, Lille, ^3^Rare and Autoinflammatory diseases laboratory, Molecular Genetics and cytogenomics , CHU de Montpellier, Montpellier, ^4^General pediatrics, CH Valenciennes, Valenciennes, ^5^Dermatology, ^6^Rheumatology, CHU Lille, Lille, France

###### **Correspondence:** A. Remy

**Introduction:**

Auto-inflammatory diseases have a broad range of symptoms and thus are difficult to diagnose.

**Objectives:**

To underline the significance of multispecialty collaboration in the diagnosis of rare inflammatory disease. To describe the phenotype of a patient with undescribed mutations of *WDR1* gene.

**Methods:**

We report a case of a child where multispecialty collaboration led to a diagnosis of Periodic Fever Immunodeficiency and Thrombocytopenia (PFIT).

**Results:**

The girl was born to unrelated Caucasian parents. From infancy, she presented with recurrent purulent conjunctivitis and unexplained severe mouth ulcers. The case was found atypical to her general pediatrician that sought the help of a dermatologist. Indeed, on top of mucous involvement, the patient had recurrent pustulosis on different parts of her body. Rheumatologist help was also sought because of recurrent stereotyped attacks, with or without fever, associated to high blood inflammation in and in between outbreaks (Orosomucoide: 5 g/L). Joints have never been involved and the patient had no hepatomegaly.

Immunohematologist joined the discussion when the patient presented with mild diarrhea. The hypothesis of an underlying primary immunodeficiency was confirmed with lymphocyte immunophenotyping revealing B cell lymphopenia (40/mm^3^ CD19+ cells) and inadequate postvaccination antitetanus immune response, along with history of recurrent infections. The hematologist point of view also gave a significant breakthrough: the patient had relative thrombopenia with lower platelets than expected regarding inflammation (157-222 × 10^9^/L).

When pediatrician, dermatologist, hematologist, rheumatologist met around this case, all agree to say that the atypical symptoms were suggestive of an autoinflammatory disease. Behcet disease was unlikely due to uncommon skin lesions. PFIT was hypothesized and confirmed by molecular analysis revealing compound heterozygosity of *WDR1* gene for 2 likely pathogenic variants: NM_017491.5:c.1264G>A p.(Val422Met) and c.1790C>T p.(Ala597Val).

**Conclusion:** PFIT is caused by mutations in the *WDR1* gene leading to actin accumulation, pyrin activation and IL-18 release.(1) Impaired cytosqueleton homeostasis impacts hematopoietic cells especially leukocytes (resulting in immunodeficiency) and platelets (resulting in microthrombocytopenia).

PFIT is a new entity first described by Standing et al in 2017.(2) Our patient has a milder phenotype than the previously described patients with no pathological scarring or microstomia to date. She had a good clinical response to colchicine, a cytosqueleton targeted therapy that has shown partial efficacy in PFIT. Should the treatment be unsuccessful, IL-18 targeted blockade might be effective, if available.

To conclude, routine blood tests and patient history as seen by a multidisciplinary team prevail extended genetics. Diagnosis and understanding of rare auto-inflammatory diseases such as PFIT require a collaboration between specialists. With respect of precision medicine, the functional consequences of those newly described *WDR1* mutations need to be clarified to optimize targeted therapy.

**REFERENCES:**

(1) Pfajfer L, Mair NK, Jiménez-Heredia R, et al. Mutations affecting the actin regulator WD repeat-containing protein 1 lead to aberrant lymphoid immunity. *J Allergy Clin Immunol*. 2018;142(5):1589-1604.e11.

(2) Standing AS, Malinova D, Hong Y, et al. Autoinflammatory periodic fever, immunodeficiency, and thrombocytopenia (PFIT) caused by mutation in actin-regulatory gene WDR1. *J Exp Med*. 2017;214(1):59-71.

**Patient Consent:** Yes, I received consent

**Disclosure of Interest**: None declared

### P167. Human type I interferonopathy with mutations in CTNNA3

#### J. Riedl^1^, C. Eide^2^, J. Tolar^1^, A. Kwiatkowski^3^, J. Heier^3^, A. Almeida de Jesus^4^, R. Goldbach-Mansky^4^, B. Binstadt^1^

##### ^1^Medical School, Pediatrics, ^2^Medical School, University of Minnesota, Minneapolis, ^3^Cell Biology, University of Pittsburgh, Pittsburgh, ^4^Translational Autoinflammatory Diseases Section, National Institutes of Health (NIH), Bethesda, United States

###### **Correspondence:** J. Riedl

**Introduction:** The interferonopathies are a group of monogenic disorders defined by persistent type 1 interferon (IFNα,β,k) signaling and upregulation of interferon-stimulated gene (ISG) expression. They are highly heterogeneous, and approximately 20 genes have been implicated in driving ISG expression and pathogenesis in these autoinflammatory disorders. The full spectrum of clinical presentation and mechanisms of disease are not fully described, and treatment remains challenging.

**Objectives:** We identified an 11-year-old male patient with a high IFN score in skin (1000x normal) and peripheral blood (200x normal). The patient has severe inflammatory skin lesions, conjunctivitis, mucositis and joint contractures: symptoms akin to described monogenic interferonopathies

**Methods:** Whole exome sequencing revealed compound heterozygous mutations in CTNNA3 encoding the protein αT-catenin, which localizes to adherens junctions of a subset of cells, where it links the cadherin complex to filamentous actin, providing cell-cell attachment.

**Results:** The CTNNA3 mutations encoded a premature stop codon (R208*) and an amino acid substitution (R378C). ClinVar suggests that these are pathogenic mutations. The R378C mutation is located in an intramolecular salt bridge that regulates protein conformation. Mechanical force breaks the salt bridge to induce conformational change and reveal a cryptic binding site for the protein vinculin (important for both cadherin- and integrin-mediated adhesion to the actin cytoskeleton).

The patient also had mutations in *NOD2*, a gene linked to Blau and Yao syndromes, This patient’s *NOD2* variants included IVS8^+158^ and R702W in linkage disequilibrium; both parents have the same *NOD2* mutations and are unaffected. We therefore propose a possible link between *CTNNA3* and an inflammatory disease with an interferon signature.

**Conclusion:** There are no prior reported associations of *CTNNA3* mutations in inflammatory disorders. It is possible that the combination of the *CTNNA3* and *NOD2* gene mutations together drives patient’s phenotype. Further study is needed to investigate the potential role of *CTNNA3* in in interferon-associated autoinflammatory disease.

**Patient Consent:** Yes, I received consent

**Disclosure of Interest**: None declared

### P168. Chronic Recurrent Multifocal Osteomyelitis (CRMO): new insights into extra-osseous manifestations

#### M. Robert, C. Guillemin, L. Rossi-Semerano, C. Galeotti, I. Koné-Paut, P. Dusser

##### Rhumatologie pédiatrique, Hôpital Bicêtre, Le Kremlin Bicêtre, France

###### **Correspondence:** M. Robert

**Introduction:** Chronic recurrent multifocal osteomyelitis (CRMO) is a rare auto-inflammatory disease. Clinical manifestations include skeletal symptoms that are well described. Whole body magnetic resonance imaging (MRI) is the gold standard for the diagnosis. Sometimes, skin or digestive manifestations occur. Treatment relies on non-steroidal anti-inflammatory drugs (NSAIDs), bisphosphonates are used as second-line treatments. To date, the focus has been set on bone involvement and few data are available on extra-osseous manifestations in CRMO.

**Objectives:** This study aims to further describe these extra-osseous manifestations in CRMO.

**Methods:** A historical cohort was designed with patients followed at the Pediatric Rheumatology Department in a tertiary university hospital. All patients fulfilled Jansson’s criteria. Skeletal and extra-osseous manifestations were characterized. Treatments used were recorded.

**Results:** Forty-one patients were included, with 31 females (75.6%). The mean age at onset was 79.1 months, with a delay at diagnosis of 6.71 months. Twenty-one patients had a familial history of inflammatory diseases (n=21/41, 51.2%). At diagnosis, pain level was 5.71/10. Eleven patients (42.3%) had blood inflammation. The mean number of bone lesions, assessed thanks to MRI, was 6.65. Twenty-seven patients had extra-osseous symptoms (65.9%), with 7 patients with fever (17.1%), 24 patients had skin manifestations (58.5%) including palmoplantar lesions (n=3, 12.5%), acne (n=6, 25.0%), psoriasis (n=5, 20.8%) and aphthous (n=10, 41.7%). Four patients (9.76%) had gastro-intestinal symptoms and 7 (17.1%) had enthesitis. One patient had uveitis. A patient had a vasculitis which mimicked Behçet’s disease. Almost all patients received NSAIDs (n=39/41, 95.1%), 51.2% (n=21/41) of the cohort was treated with bisphosphonates. Nine patients (22.0%) received biologics. Subgroup analysis between CRMO with and without extra-osseous manifestations revealed significant differences for age at diagnosis (126 vs 20.5 months, p=0.03), blood inflammation (87% vs 46.2%, p=0.018) and the use of biologics (33.3% vs 0%, p=0.017).

**Conclusion:** Extra-osseous manifestations have to be carefully searched in CRMO and integrated in the therapeutic strategy.

**Patient Consent:** Yes, I received consent

**Disclosure of Interest**: None declared

### P170. The Russian cohort of patients with TRAPS according to the federal rheumatology center

#### S. Salugina^1^, E. Fedorov^1^, M. Kaleda^1^, E. Kamenets^2^, E. Zakharova^2^

##### ^1^Pediatrics, V.A. Nasonova Research Institute of Rheumatology, ^2^Selective Screening, Research Center of Medical Genetics, Moscow, Russian Federation

###### **Correspondence:** S. Salugina

**Introduction:** TRAPS (TNF-receptor-associated periodic syndrome) is a rare autosomal dominant disease associated with a mutation of the TNFRSF1A gene. It belongs to the group of monogenic autoinflammatory diseases (mAIDs) characterized by repeated prolonged episodes of fever, skin rashes, musculoskeletal, ophthalmological symptoms, and an increase in the level of acute phase markers. The main targeted therapy is IL1 inhibitors (iIL–1).

**Objectives:** to present the Russian cohort of patients (pts) with TRAPS according to the Federal Rheumatology Center

**Methods:** For the period from 2013 to 2021, 28 pts with TRAPS were included in the study, 17 of them female, aged 2.5 to 65 years, Me 9.0 [IQR 7.9;14.5] y.o., 7 adults, 21 children. All pts underwent rheumatological examination. Molecular genetic analysis for mutations in the TNFRSF1A gene was made to all pts.

**Results:** Among mAIDs (162 pts), TRAPS was diagnosed in 28 (17.3%). The age of the onset ranged from 1 month of life to 28 years, Me 4.0 [IQR 1.0;7.1] y.o. The majority of pts started to hurt before the age of 10 (89.3%), of them up to a year - 8 (28.6%), from 1 to 5 years – 25%, older than 18 years – 2 (7.1%), at the age of 21 and 28. At the time of the study the duration of the disease and the delay in diagnosis ranged from 3 months to 59 years. The diagnosis was made within 1 year of the disease in 4 (14.3%), ranging from 1 to 10 years in 18 (64.3%), a delay in diagnosis of more than 10 years was in 8 (28.6%), more than 20 years in 4 (14.3%). The duration of attacks ranged from 10 to 30 days, the intervals between seizures ranged from 2 weeks to 6 months. Clinical manifestations included most often: fever – 96.4% of pts, skin rashes - 71.4% (annular in 9, erythema in 6, urticaria in 2), musculoskeletal symptoms - 64.3% (among them oligoarthritis in 9, polyarthritis in 2), gastrointestinal symptoms - 64.3% (more often abdominal pain), lymphadenopathy of various groups (cervical, intra-thoracic, mesenteric lymph nodes) - 35.7%, ophthalmological manifestations - 28.6% (more often periorbital edema-5 and eye redness-5). Less frequently observed: manifestations from the nervous system (headaches, dizziness, fatigue, convulsions) in 6 pts, pharyngitis - in 4, stomatitis - in 2. An increase in ESR was noted in 82.1% pts, CRP-71.4%, leukocytosis - 39.3%. The diagnosis in all pts was confirmed genetically. 10 pts had a low-penetrant mutation R92Q (p. Arg121Gln). 5 family cases were identified, the total number of sick family members was 12, from 2 to 4 in each family, 3 cases of amyloidosis, all adults. Treatment included: GC-19 (67.9%), cyclosporine A - 1, colchicine-5. IL-1 inhibitor (canakinumab) was prescribed to 10 pts (35.7%), duration of administration was from 1 to 9 years; etanercept-1, adalimumab-1, tocilizumab-3.

**Conclusion:** A cohort of pts with TRAPS in the practice of a rheumatologist is presented. The majority of pts had an early onset of the disease (before the age of 10), the delay in diagnosis was 10 years or more in a third of pts. Most pts had a typical inflammatory phenotype, the diagnosis in all was confirmed genetically. About a third of the pts needed the appointment of biological drugs, mainly iIL-1. There was a complete response to the therapy in most pts and good tolerability of the treatment.

**Patient Consent:** Yes, I received consent

**Disclosure of Interest**: None declared

### P171. Long-term efficacy and safety of canakinumab in patients with tumor necrosis factor receptor-associated periodic syndrome (TRAPS) - interim analysis of the reliance registry

#### C. Schuetz^1^, N. Blank^2^, J. Henes^3^, T. Kallinich^4^, P. T. Oommen^5^, M. Borte^6^, M. Hufnagel^7^, A. Janda^8^, J. Weber-Arden^9^, J. B. Kuemmerle-Deschner^10^

##### ^1^Catharina.Schuetz@uniklinikum-dresden.de, Medizinische Fakultaet Carl Gustav Carus, Technische Universitaet Dresden, Dresden, ^2^Rheumatology, University Hospital Heidelberg, Heidelberg, ^3^Center of Interdisciplinary Rheumatology, Immunology and autoimmune diseases (INDIRA), University Hospital Tuebingen, Tuebingen, ^4^Department of Pediatrics, Division of Pulmonology, Immunology and Critical Care Medicine, Charité University Medicine Berlin, Berlin, ^5^Clinic of Pediatric Hematology, Oncology and Clinical Immunology, Heinrich-Heine-University Duesseldorf, Duesseldorf, ^6^ImmunoDeficiencyCenter Leipzig (IDCL), Hospital St. Georg gGmbH Leipzig, Leipzig, ^7^Division of Pediatric Infectious Diseases and Rheumatology, Department of Pediatrics and Adolescent Medicine, University Hospital Medical Center Freiburg, Medical Faculty, University of Freiburg, Freiburg, ^8^Department of Pediatrics, University Hospital Ulm, Ulm, ^9^NOVARTIS, Nuernberg, ^10^Department of Pediatrics, Division of Pediatric Rheumatology, University Hospital Tuebingen, Tuebingen, Germany

###### **Correspondence:** C. Schuetz

**Introduction:** TRAPS[1] is a rare hereditary autoinflammatory disease characterized by periodic fever and severe systemic and organ inflammation. Successful treatment was achieved with the interleukin-1β inhibitor canakinumab (CAN) in a pivotal phase 3 study, in which 45% of patients achieved clinical remission. CAN has been approved for the treatment of TRAPS patients since 2017.

1TRAPS: Tumor necrosis factor receptor-associated periodic syndrome

**Objectives:** The present study investigates the long-term efficacy and safety of CAN under routine clinical practice conditions in pediatric (age ≥2 years) and adult TRAPS patients.

**Methods:** RELIANCE is a prospective, non-interventional, multicenter observational study in Germany. Patients with a clinically confirmed diagnosis of TRAPS who routinely receive CAN will be enrolled in the study to evaluate the efficacy and safety of CAN under standard clinical conditions at baseline and at six-month intervals.

**Results:**

The interim analysis of TRAPS patients enrolled through December 2021 included baseline data (N=19) and preliminary 24-month data. Of these patients, N=12 (63%) were female and the median age at baseline was 16 years (3-43 years).

Mutation information was provided for a total of N=13 patients, all of whom were pathogenic or likely pathogenic (Table 1).

N=4 patients had dose adjustments (↑ dose increase, ↓ dose reduction): patient 1: ↑, patient 2: ↑↓, patient 3: ↑↑, patient 4: ↑↑↓↑.

Preliminary results at baseline, 12 months and 24 months indicate stable disease control by remission according to physician assessment (47, 73 and 50% of patients), laboratory parameters (CRP 0.2, 0.1, 0.2 mg/dl and Serum Amyloid A (0.5, 0.4, 0.4 mg/dl median) and disease control according to patient assessment (1.5, 1.0, 0.0). Of N=9 serious adverse events, none occurred in association with therapy. A total of N=7 adverse drug events were observed, none of which were considered serious.

**Conclusion:** Baseline and interim data from the RELIANCE trial for TRAPS patients indicate continued good efficacy and safety of various CAN doses.

**Disclosure of Interest**: C. Schuetz: None declared, N. Blank Grant / Research Support with: Novartis, Sobi, Consultant with: Novartis, Sobi, Lilly, Pfizer, Abbvie, BMS, MSD, Actelion, UCB, Boehringer-Ingelheim, Roche, J. Henes Grant / Research Support with: Novartis, Roche, Consultant with: Novartis, AbbVie, Sobi, Roche, Janssen, Boehringer-Ingelheim, T. Kallinich Speaker Bureau with: Roche, P. T. Oommen Grant / Research Support with: Novartis, M. Borte Grant / Research Support with: Pfizer, Shire, M. Hufnagel Grant / Research Support with: Novartis, A. Janda: None declared, J. Weber-Arden Employee with: Novartis, J. B. Kuemmerle-Deschner Grant / Research Support with: Novartis, AbbVie, Sobi, Consultant with: Novartis, AbbVie, Sobi

**Table 1 (abstract P171).** Mutation in relation to disease activity and dosage

Patients with pathogenic or likely pathogenic mutation in relation to disease activity and dosage, current age in years.	Mutation	Disease activity (absent, mild/moderate, severe)	Dosing (normalized to mg every 4 weeks)
**Patient 1**, 16 years	T50M	absent	150
**Patient 2**, 46 years	T50M	absent	150
**Patient 3**, 5 years	C70R	Absent	75 (4 mg/kg)
**Patient 4**, 10 years	R92Q	mild/moderate	150 (4 mg/kg)
**Patient 5**, 36 years	R92Q	mild/moderate	150
**Patient 6**, 16 years	R92Q	n.s.*	n.s.
**Patient 7**, 41 years	R92Q	n.s.	150
**Patient 8**, 32 years	C55R	severe	150
**Patient 9**, 33 years	I170N	absent	150
**Patient 10**, 39 years	I170N	absent	150
**Patient 11**, 45 years	N116S	absent	150
**Patient 12**, 35 years	Y20C+Y20C	absent	150
**Patient 13**, 4 years	S321N	mild/moderate	105 (7.2 mg/kg)

n.s. not specified

### P172. Outcomes and post-acute sequelae of COVID-19 in patients with systemic autoinflammatory diseases

#### S. Nazzar^1^, L. Moorthy^2^, S. Lapidus^3^, G. Schulert^4^, J. Tousseau^5^, M. Correia Marques^6,7^, L. Mansfield^8,9^, M. Twilt^10^, M. Gutierrez^11^, S. Angevare^12^, F. Dedeoglu^13^, K. Durrant^5^

##### ^1^UCLA, Los Angeles, ^2^Rutgers-RWJMS, New Brunswick, ^3^The Joseph M. Sanzari Children’s Hosp., Hackensack, NJ, ^4^Cincinnati Children’s Hosp., Cincinnati, ^5^Autoinflammatory Alliance, San Francisco, ^6^NIAMS, NIH, Bethesda, ^7^Children’s Nat’l Hosp., Washington DC, ^8^Hosp. for Special Surgery, ^9^Weill Cornell, NY, United States, ^10^Alberta Children’s Hospital, Calgary, Canada, ^11^Johns Hopkins Hosp., Baltimore, United States, ^12^KAISZ, Amsterdam, Netherlands, ^13^Boston Children’s Hosp., Boston, United States

###### **Correspondence:** G. Schulert

**Introduction:** The exaggerated inflammatory responses to SARS-CoV-2 infection and the paucity of data on the risks for patients with Systemic Autoinflammatory Disease (SAID) patients is concerning.

**Objectives:** To assess the outcomes of SAID patients with COVID-19.

**Methods:** The CARRA Autoinflammatory Working Group and the Autoinflammatory Alliance conducted an anonymous online survey that was distributed to SAID patients through social media between January and March of 2021 about their experiences with COVID-19 in 2020.

**Results:** We analyzed data from 593 individuals with SAID including Cryopirin-associated Periodic Syndromes (CAPS) (18%, n=108), Undifferentiated Systemic Autoinflammatory Disease (USAID) (12%, n=70), Periodic Fever Aphthous Stomatitis Pharyngitis and Cervical Adenitis (PFAPA) (11%, n=64) and others. Seventy four percent of the patients were from the United States, US territories and Canada and 26% from other countries. Fifty-two subjects had COVID-19. Of these, 12% (n=6) were asymptomatic, 81% (n=42) had mild or moderate symptoms managed at home and four were hospitalized. Thirty-three percent (n=17) had a flare of their underlying SAID concurrently (n=10) or after recovering from COVID-19 (n=7). Half of the patients who were hospitalized (n=2/4) noted increased frequency of flares during the pandemic, whereas the ones who had asymptomatic infection had decreased or unchanged flare frequency. Twenty-one (40%) of patients reported post-COVID manifestations (Table 1), which were seen more often after moderate illness and hospitalized patients (60%, n=15/25), compared to mild and asymptomatic cases (22%, n=6/27). Thirty three percent (n=7) of the SAID patients who had post-COVID symptoms did not have a specific SAID diagnosis listed, 14% (n=3) had USAID, and 9.5% (n=2) for each diagnosis had CAPS, PFAPA or Familial Mediterranean Fever.

**Conclusion:** These data reflect the largest known global cohort of SAID patients’ experiences with COVID-19. Most of the SAID patients who had COVID-19 reported mild to moderate symptoms managed at home with a third presenting an exacerbation of their baseline SAID and lingering symptoms. The severity of acute COVID-19 was associated with reported changes in the frequency of flares during the pandemic and with the likelihood of post-COVID symptoms.

**Patient Consent:** Not applicable (there are no patient data)

**Disclosure of Interest**: S. Nazzar: None declared, L. Moorthy Grant / Research Support with: Brystol Myers Squibb, S. Lapidus: None declared, G. Schulert Consultant with: Novartis, J. Tousseau: None declared, M. Correia Marques: None declared, L. Mansfield: None declared, M. Twilt: None declared, M. Gutierrez: None declared, S. Angevare: None declared, F. Dedeoglu: None declared, K. Durrant: None declared

**Table 1 (abstract P172).** Post-COVID Symptoms in SAID Patients

**Immunologic 33%**	New PFAPA, recurrence of PFAPA, worsened flares, generalized lymphadenopathies and hepatosplenomegaly, Multisystem Inflammatory Syndrome in children (MIS-C)*, Multisystem Inflammatory Syndrome in adults (MIS-A) and Macrophage Activation Syndrome (MAS)^
**Nervous system 29%**	Headaches, hemiplegic migraine, increased seizures, weakness, cognitive issues, loss of taste, memory
**Respiratory 29%**	Cough, shortness of breath, worse asthma, chest pain
**Constitutional 19%**	Fatigue, inability to function
**Musculoskeletal 10%**	Arthralgias, myalgias
**Cardiovascular 10%**	Bradycardia, hypertension
**Integumentary 10%**	Rash, COVID toes
**Miscellaneous 25%**	Physician diagnosed Post-Acute Sequelae of SARS CoV-2 infection (PASC), prolonged recovery, chronic pharyngitis, chronic conjunctivitis, irritable bowel syndrome, kidney disease

*MIS-C was reported in one patient whose SAID diagnosis was not specified and in one patient with a CAPS variant

^MIS-A and MAS were reported once by the same patient who had presumed COVID-19 infection and USAID at baseline

### P173. Genotypes and phenotypes patterns in patients with NLRP3 gene variants

#### E. Krekhova^1^, E. Alexeeva^1^, M. Shingarova^1^, T. Dvoryakovskaya^2^, K. Isaeva^2^, R. Denisova^2^, A. Chomakhidze^2^, O. Lomakina^2^, A. Mamutova^2^, A. Fetisova ^2^, M. Gautier ^2^, C. Chibisova ^2^, I. Kriulin ^2^, I. Tsulukia ^2^, A. Pushkov^3^, K. Savostyanov^3^

##### ^1^Pediatric, Sechenov First Moscow State Medical University, Pediatric, Moscow, Russian Federation, ^2^Rheumatology, ^3^Molecular genetic, National Medical Research Center of Children’s Health, Moscow, Russian Federation

###### **Correspondence:** M. Shingarova

**Introduction:** Cryopyrine-associated periodic syndromes (CAPS) are a group of rare congenital auto-inflammatory diseases (AID) in an autosomal dominate manner and caused by variants in *NLRP3* gene. The main difficulties in diagnosing CAPS are the similarity of their clinical manifestations with other rheumatic diseases.

**Objectives:** To reveal genotype and phenotype characteristics in patients with *NLRP3* gene variants at the National Medical Research Center of Children’s health, Moscow, Russia.

**Methods:** Retrospective study included 60 patients (37 females, 62%) with *NLRP3* gene variants revealed by molecular genetic analysis and classified according ACMG criteria. Median age of debut was 6,3 (interquartile range (IQR) 0,1:13,7) years. The diagnosis of CAPS was founded according Eurofever/PRINTO criteria.

**Results:** 5/60 (8,3%) patients had *NLRP3* pathogenic variants: two had *c.943A>G* variant, and one each with *c.2173C>A, c.1991T>C* and *c.214G>A* respectively. Likely pathogenic variants had 5/60 patients (8,3%)/ Among them two had *c.1085T>A*, two - *c.410G>A*, and one - *c.796C>T*; all heterozygous. Clinical manifestations 10/60 (16,7%): rash in 7 (70%), fever in 7 (70%) patients, hepatomegaly in 9 (90%), splenomegaly in 6 (60%) patients, lymphadenopathy in 8 (80%), and serositis in 4 (40%) patients. Аrthritis was observed in 10/10 (100%) patients, affection eyes in 4/10 (40%), сentral nervous impairment observed in 6(60%), sensorineural hearing loss was in 2 (20%) patients. The criteria of CAPS accorded 9/10 (90%) children. One patients with pathogenic variants was sibling child with CAPS (asymptomatic carrier). Variant with variant with conflicting pathogenicity *c.598G>A* was observed in 10/60 (16,7%). Clinical manifestations 10/60 (16,7%): rash in 3 (30%), fever in 7 (70%) patients, hepatomegaly in 5 (50%), splenomegaly in 3 (30%) patients, lymphadenopathy in 4 (40%), serositis in 1 (10%) patients, arthritis was observed in 9/10 (90%) patients, affection eyes in 4/10 (40%), сentral nervous impairment observed in 2(20%), and sensorineural hearing loss was in 1 (10%) patients. The criteria of CAPS accorded 4/10 (40%) children, juvenile polyarthritis (RF-) - 4/10 (40%), persistent oligoarthritis – 1/10(10%) and 1/10 with DADA2 . Among the remaining patients, 30/60 (50%) had polymorphism *c.2113C>A*, 8/60 had VUS *c.2861C>T*, *c.584C>T*, *c.585G>A, c.459C>G* and 2/60 patients - probably benign variants : *c.2664-26G>C* and *c.1050G>A*, respectively.

**Conclusion:** The criteria of CAPS accorded 14 patients. The most frequent clinical manifestations were in musculoskeletal involvement 93%, fever 86 % and rash 79 % of children. Central nervous impairment observed in 7/14(60%), sensorineural hearing loss was in 3/14(21,4%) and affection eyes in 4/14(29%). The number of identified causal variants of the *NLRP3* gene in these 14 patients showed high diversity, while the high frequency of polymorphism *c.2113C>A* in the studied sample was consistent with data obtained in other populations.

**Patient Consent:** Yes, I received consent

**Disclosure of Interest**: None declared

### P174. Netosis dysregulation in adenosine deaminase 2 deficiency

#### S. Signa^1^, A. Bertoni^1^, G. Del Zotto^2^, M. Bonacini ^3^, A. Corcione^1^, M. Bartolucci^4^, M. Bruschi^5^, A. Petretto^4^, R. Bertelli^6^, S. Croci^3^, S. Della Bella^7^, M. Gattorno^1^, F. Schena^1^

##### ^1^Centro per le malattie autoinfiammatorie e immunodeficienze, ^2^Core Facilities Flow Cytometry and Cell imaging Lab, IRCCS, Giannina Gaslini Institute, Genova, ^3^Clinical Immunology, Allergy and Advanced Biotechnologies Unit, Azienda Unità Sanitaria Locale-IRCCS di Reggio Emilia, Reggio Emilia, ^4^Core Facilities-Clinical Proteomics and Metabolomics, ^5^Laboratory of Molecular Nephrology, ^6^Laboratory of Human Genetics, IRCCS, Giannina Gaslini Institute, Genova, ^7^Lab of Clinical and Experimental Immunology, IRCCS Humanitas Research Hospital, Milano, Italy

###### **Correspondence:** S. Signa

**Introduction:** Deficiency of Adenosine deaminase 2 (DADA2) is a monogenic autoinflammatory disorder presenting a broad spectrum of clinical manifestations, including vasculitis, immunodeficiency and hematologic disease. Biallelic mutations in ADA2 gene have been associated to an insufficient ADA2 activity and a consequent accumulation of extracellular adenosine. Recently a chronic neutrophil activation and a dysregulation of NETosis triggered by extracellular Adenosine and inducing TNF-α secretion from macrophages, has been implicated in the pathogenesis of DADA2.

**Objectives:** The aim of the project is to analyze NETosis in DADA2 and healthy neutrophils, quantifying suicidal and vital NETosis induced by several stimuli and by analyzing NETs remnants. Moreover, we investigated the mechanisms of NETs removal, evaluating DNAse activity. To determine if NET epitopes can change depending from the inflammatory microenvironment and if protein composition of NETs is disease specific, we used quantitative proteomics approach to characterize DADA2 patients’ NET proteins. To investigate the cross-talk between neutrophils and Dendritic cells (mDCs), we performed DCs immune-phenotype analysis in DADA2 patients and analyzed in vitro moDC maturation and cytokine production in presence of NETs.

**Methods:** We analyzed NETosis by Imaging Flow Citometry. We evaluated NETs remnants and DNAse in the plasma samples by ELISA assay whereas DNAse activity by DNA digestion. We used quantitative proteomics approach to characterize NET proteins. We isolated monocytes from peripheral blood with microbeads and we generated moDC after culture stimulation with LPS/NETs. On the 7th day we analyzed by flow cytometry the phenotype and the markers of differentiation. Quantification of cytokines was performed by flow cytometry bead array.

**Results:** Neutrophils from DADA2 patients show a significant increased suicidal NETosis, and we found an increased vital NETosis. Accordingly, plasmatic levels of circulating nucleosomes were elevated in patients; DNAse levels were normal but its activity was reduced, possibly due to presence of inhibitors. We set up experimental conditions for proteomic analysis of NETs, induced by PMA, Adenosine and TNF-α, testing two patients, two HDs and two patients with non-genetic vasculitis: in total we identified 1770 proteins among which a hundred of proteins were significantly up or down-modulated in DADA2 NETs compared to controls NETs. DC phenotype in DADA2 patients result normal, as well as moDCs cytokine production after LPS stimulation. We observed a stimulatory effect of patients’ NETs towards induction of TNF-α , IL-6 production and IP-10 from moDCs in both HD and DADA2.

**Conclusion:** Our findings confirm a dysregulation in NETosis process in DADA2 patients. An increase of vital NETosis was also identified. Proteomic profile of NETs isolated from DADA2 is different from HD and PAN patients: NETs are qualitatively different between HD and DADA2. NETs in DADA2 may therefore interact differently with innate immunity compartment, stimulating DCs to produce cytokines, contributing to the typical inflammatory phenotype.

**Patient Consent:** Yes, I received consent

**Disclosure of Interest**: None declared

### P175. Type I interferon signature: a standardized method for clinical application

#### P. Bocca^1^, A. Tesser^2^, D. Cangelosi^3^, M. Ulivi^4^, F. Candotti^5^, A. Tommasini^2,6^, M. Gattorno^1^, S. Volpi

##### ^1^Clinica Pediatrica e Reumatologia, IRCCS Istituto G. Gaslini, Genova, ^2^Pediatric Department, Institute for Maternal and Child Health - IRCCS “Burlo Garofolo”, Trieste, ^3^Unità di Bioinformatica Clinica, IRCCS Istituto G. Gaslini, ^4^Centro Biotecnologie Avanzate, TIB Molbiol, Genova, Italy, ^5^Laboratory of Inherited Immune Disorders, Division of Immunology and Allergy, Lausanne University Hospital and University of Lausanne, Lausanne, Switzerland, ^6^University of Trieste, Trieste, Italy

###### **Correspondence:** M. Gattorno

**Introduction:** Type I interferon (IFN) signature analysis is extensively used to identify pathological conditions characterized by a type I IFN dysregulation (i.e. monogenic interferonopathies, dermatomyositis, systemic lupus erythematosus), and to direct therapy approaches. IFN signature is used to discriminate patients with IFN-related inflammation from conditions predominantly mediated by other cytokines (i.e. periodic fevers), through the calculation of an IFN score (IS). However, no standardized method is yet available for the clinical practice, and comparing values at different time points or between different centers remains a challenge.

**Objectives:** To implement a standardized method for IFN signature detection with the use of a synthetic control, and to evaluate the concordance of IFN signature results obtained with this method among the laboratories of two Italian Hospitals.

**Methods:** Peripheral blood expression analysis of the six IFN Stimulated Genes (ISGs) *IFI27, IFI44L, IFIT1, ISG15, RSAD2* and *SIGLEC1* was detected by Real Time PCR. A synthetic control was used to determine copy numbers of the selected genes.

The IS for each subject was calculated as the geometric mean of the genes’ copy numbers normalized using an endogenous reference gene. IS cut-off value was obtained as the mean + 2SD of 20 healthy donors’ IS.

The method was validated by analyzing the IS of 89 patients with different inflammatory, autoimmune and infectious disorders driven by type I IFN or other inflammatory pathways.

To assess inter-laboratory variability, 12 patients with different degrees of type I IFN inflammation were run in parallel in two different centers.

**Results:** As expected, our test confirmed a positive IFN score for inflammatory diseases such as systemic lupus erythematosus, type I interferonopathies, dermatomyositis and Sjögren syndrome.

The test demonstrated a high reproducibility as assessed by multiple analyses of the same sample. Furthermore, IS obtained from the samples shared by two different laboratories resulted in comparable values.

**Conclusion:** The proposed method represents a quantitative, standardized and easy to perform assay for the evaluation of type I IFN signature in IFN-driven inflammatory conditions.

The reproducibility of the synthetic control minimized the inter-assay and inter-laboratory variability. The application of a standardized method for type I IFN signature detection greatly improves the test interpretation, patient follow up and clinical discussion among centers promoting the use of type I IFN signature in clinical practice.

**Disclosure of Interest**: None declared

### P176. Recurrent fever without molecular diagnosis: lessons from autoinflammatory disease registries

#### Y. Vyzhga^1^, V. Hentgen^2^, R. Papa^3^, H. Wittkowski^4^, N. Ruperto^3^, E. Lainka^5^, K. Theodoropoulou^6^, R. Caorsi^3^, S. Fuehner^4^, D. Foell^4^, M. Gattorno^3^, M. Hofer^6^ on behalf of AID-NET, Eurofever and JIRcohort registries

##### ^1^National Pirogov Memorial Medical University, Vinnytsya, Vinnitsya, Ukraine, ^2^Versailles Hospital, Le Chesnay, France, ^3^IRCCS Instituto G. Gaslini, Genova, Italy, ^4^University Hospital Childreńs Muenster, Muenster, ^5^University Hospital Essen, Essen, Germany, ^6^Centre Hospitalier Universitaire Vaudois CHUV, Lausanne, Switzerland

###### **Correspondence:** Y. Vyzhga

**Introduction:** Patients with recurrent fever without molecular diagnosis can often be diagnosed as Periodic Fever, Aphthous Stomatitis, Pharyngitis, Adenitis (PFAPA) syndrome, the most common pediatric autoinflammatory fever syndrome, with a highest incidence in children up to 5 years of age. Nevertheless, a number of patients do not fulfil current diagnostic criteria and have to be classified as undefined autoinflammatory diseases (uAID). In recent years the syndrome of undifferentiated recurrent fever (SURF) was proposed as a heterogeneous group of autoinflammatory diseases characterized by the presence of self-limiting episodes of systemic inflammation with multiple clinical presentations, but without confirmed molecular diagnosis. Presence of the similar clinical features makes these 2 conditions to be in a scope of interest from the perspective of the potential overlap and misdiagnosing.

**Objectives:** In a project endorsed by PReS, supported by the EMERGE fellowship program, and performed in line with the Metadata registry for the ERN RITA (MeRITA) project, our objective was to analyse recurrent fevers without molecular diagnosis, the evolution of diagnosis over time and the clinical characteristics in PFAPA, SURF and uAID.

**Methods:** Currently there are two international AID-registries active (Eurofever and JIRcohort) and one German AID-registry, that was active from 2009 to 2018. Patients in Germany were recruited to the BMBF-funded AID-Net Registry and the AID-Net Biobank. Patients with PFAPA, uAID or SURF were identified from the registries. Diagnostic (modified Marshall’s criteria) and classification criteria (Eurofever) for PFAPA and the preliminary indications for the suspicion of SURF (Papa et al) were applied to all patients.

**Results:** The overall number of patients with a diagnosis of PFAPA in all three registries was 1230 (Eurofever - 634, AIDnet - 140, JIR cohort - 456). Complete epidemiological and clinical dataset was available in 929 patients (Eurofever - 476, AIDnet - 136, JIR cohort - 317). Marshall’s modified criteria were satisfied in 51.5 to 67.2 % of the PFAPA patients, while 59 to 69.4 % patients fulfilled the Eurofever classification criteria. At the same time, 29.4 to 47.5 % of the patients met both – modified Marshall and Eurofever criteria. Up to 20 % of the patients didn’t fulfil any of the proposed criteria.

The overall number of patients with a diagnosis of SURF was n=809 (Eurofever - 429, AID net - 83, JIR cohort - 297). Among them, 561 of them displayed a negative genetic test for hereditary recurrent fevers (Eurofever - 307, AIDnet - 79, JIR cohort - 175). 417 patients satisfied the preliminary indications for the suspicion of SURF (Eurofever - 230, AIDnet - 51, JIR cohort - 136). From 22 to 35 % of SURF patients didn’t fulfil the proposed preliminary indications .

From 8 to 17% of PFAPA patients in the three registries fulfilled the preliminary indications for suspicion of SURF. Vice versa, 26 to 37 % SURF patients satisfied at least one PFAPA criteria.

**Conclusion:** Patients with recurrent fevers but without molecular diagnosis are still an heterogenous group of patients regarding clinical manifestations. More accurate classification criteria are needed for both conditions. A better definition of the clinical manifestations associated with the 2 conditions would allow correct grouping of these patients improving clinical management and outcome.

This project was supported by PRES-EMERGE and Novartis AG funding for IIR.

**Disclosure of Interest**: None declared

### P177. Real-life data on autoinflammatory diseases: what do we learn from international patient registries?

#### Y. Vyzhga^1^, V. Hentgen^2^, R. Caorsi^3^, H. Wittkowski^4^, N. Ruperto^3^, E. Lainka^5^, S. Fuehner^4^, K. Theodoropoulou^6^, M. Hofer^6^, D. Foell^4^, M. Gattorno^3^ on behalf of AID-NET, Eurofever and JIRcohort registries

##### ^1^National Pirogov Memorial Medical University, Vinnytsya, Ukraine, ^2^Versailles Hospital , Le Chesnay, France, ^3^IRCCS Instituto G. Gaslini, Genova, Italy, ^4^University Hospital Childrens Muenster, Muenster, ^5^University Hospital Essen, Essen, Germany, ^6^Centre Hospitalier Universitaire Vaudois CHUV, Lausanne, Switzerland

###### **Correspondence:** Y. Vyzhga

**Introduction:** Knowledge about autoinflammatory diseases (AID) has changed the general view of innate immune activation over the recent decades. Based on common pathophysiological mechanisms, autoinflammatory diseases can be divided into groups (IL-1-, interferon type I- and NF-kB-mediated diseases, diseases caused by macrophage activation, and other diseases). Also due to modern sequencing techniques, the number of known entities has grown strongly in recent years, but still their peculiarities in different countries. Access of the patients to diagnostic and treatment options are variable, may affecting disease control and the risk of long term complications.

**Objectives:** In a project endorsed by PReS, supported by the EMERGE fellowship program, and performed in line with the Metadata registry for the ERN RITA (MeRITA) project, our objective was to perform data harmonization and comparative analyses of selected most relevant research questions, e.g. changes in the diagnostic work-up of AID-patients over time, and how newly described disease entities or the emergence of new drugs have changed patient care. This is also meant to improve further work in the registries.

**Methods:** Currently there are two European AID-registries active (Eurofever and JIRcohort) and one German AID-registry, that was active from 2009 to 2018. Cohort design, duration of the observance and patient’s follow-up, main epidemiological, clinical and laboratory information, data collection methods, process of IT-support, etc. were analysed and compared between registries for harmonization of data granularity and further analysis.

**Results:** Together, the 3 registries cover 7825 patients with different AID from participating centers all over the world. The German AID-Net presents a national type of registry involving 36 pediatric rheumatology centers in Germany. JIRcohort and Eurofever are international cohorts, covering about 40 countries in total. The most widely presented AID in all registries were FMF, PFAPA, URF (Undefined Recurrent Fever), CRMO, SoJIA, CAPS, TRAPS, and MKD. Eurofever and JIRcohort additionally covered other exclusively rare AID as PAPA, DADA2, Blau syndrome, HA20, PASH, DIRA, etc.

One of the important scopes of the study was evaluation of the diagnostic delay. There was a marked delay between disease onset and diagnosis in all registers, with an obvious trend of its shortening between children. The highest diagnostic delay is more common for classical monogenic AID, as TRAPS, MKD and CAPS, in which the diagnostic delay in adult was mainly related to the time of the identification of the molecular defect in respect to the disease onset. Moreover, the diagnostic delay was also strongly associated with the availability of the genetic testing in different time periods. In all registers capacity of the tested patients exceed 50 % over the whole period of registry work. Detailed data of treatment options shows availability of novel biologic therapy for approximately 20 – 25% of the patients with AID and depends on its availability in the country of patient’s residence.

**Conclusion:** Several important questions arise around clinical features, diagnostic strategy and optimal management of the AID. Analysis of 3 large registries shows still relevant diagnostic delays especially in adult patients and a varying availability of access to diagnostic work up and treatment. Further efforts should be made in data harmonization for future registry studies.

This project was supported by PRES-EMERGE and Novartis AG funding for IIR.

**Disclosure of Interest**: None declared

### P178. Flow cytometric analysis of ASC specks as a novel biomarker for monitoring autoinflammatory diseases

#### S. Wieland^1^, T. Welzel^2^, S. Fühner^3^, A. Gabrielyan^1^, E. Lainka^4^, C. Kastl^1^, L. Pietzsch^1^, N.-C. Knopf^1^, C. Michler^2^, A. J. Ritter^2^, A. Rösen-Wolff^1^, D. Foell^3^, H. Wittkowski^3^, J. Kuemmerle-Deschner^2^, C. Schuetz^1^

##### ^1^Department of Paediatrics, Medizinische Fakultät Carl Gustav Carus, Technische Universität Dresden, Dresden, ^2^Division of Paediatric Rheumatology, University Hospital Tuebingen, Tübingen, ^3^Department of Paediatric Rheumatology and Immunology, Universitätsklinikum Münster, Münster, ^4^Division of Pediatric Rheumatology, University Children’s Hospital Essen, Essen, Germany

###### **Correspondence:** S. Wieland

**Introduction:** Autoinflammatory diseases (AI) like FMF, CAPS or TRAPS lead to a constitutive activation of the inflammasome and secretion of IL-1b with severe long-term consequences like amyloidosis when undiagnosed or insufficiently treated. Currently, clinicians routinely use nonspecific acute phase proteins – most of them synthetized in the liver – to monitor inflammation and treatment efficacy.

**Objectives:** Apoptosis-associated speck-like protein containing a CARD (ASC) is an adaptor protein in different inflammasomes released during pyroptosis. It assembles following activation to a speck-like oligomer in the cytoplasm. Previous studies would detect the ASC- agglomerates intracellularly after time-consuming sample preparation. We have established a rapid and simple method for routine detection of ASC agglomerates in patients’ serum.

**Methods:** We collected 68 serum samples from 52 patients (aged 1 to 30 years) with various AI and at different stages of disease activity. After centrifugation for 10-15 min at 2000g within 2 hours after sampling, the supernatant was removed and cryopreserved at -80°C. For analysis thawed sera were incubated with a PE-linked anti-ASC antibody (Biolegend) diluted 1:4. To detect the ASC agglomerates of different sizes between 1- 3mm, PE-linked microbeads (Polysciences) were used to define our gate. Flow cytometry was performed on a BD LSR II. Clinical data collected via a simplified questionnaire were available for most samples collected at 3 different pediatric centers.

**Results:** In the serum of healthy donors, null to minor amounts of ASC agglomerates were present. In contrast, we measured relevant amounts of ASC agglomerates in serum of patients with FMF, CAPS, TRAPS, sJIA and SURF. During disease flares, the amount of ASC specks was significantly elevated in samples from FMF and CAPS patients constituting over half of the collected specimens.

**Conclusion:** Measurement of ASC agglomerates may be taken as a surrogate for inflammasome activity. The methodology presented allows detection in serum without laborious preanalytics. In clinical practice, monitoring this novel biomarker may be of relevance for diagnosis, monitoring and treatment decisions. We will extend our study by measuring more specimens of patients with TRAPS, SURF/PFAPA and sJIA comparing disease flares with periods of remission.

**Disclosure of Interest**: S. Wieland: None declared, T. Welzel: None declared, S. Fühner: None declared, A. Gabrielyan: None declared, E. Lainka: None declared, C. Kastl: None declared, L. Pietzsch: None declared, N.-C. Knopf: None declared, C. Michler: None declared, A. Ritter: None declared, A. Rösen-Wolff: None declared, D. Foell: None declared, H. Wittkowski: None declared, J. Kuemmerle-Deschner: None declared, C. Schuetz Grant / Research Support with: Novartis

### P179. Significant reduction in time to diagnosis of chronic non-bacterial (CNO) and Chronic Recurrent Multifocal Osteomyelitis (CRMO) over the past 13 years in 45 children from one clinical center

#### J. Wojtowicz, A. Gazda, B. Kolodziejczyk, I. Szczygielska, M. Szwarc-Bronikowska, I. Witkowska, A. Adamczuk, E. Hernik, P. Gietka

##### Department of Pediatric Rheumatology, National Institute of Geriatrics, Rheumatology and Rehabilitation, Warszawa, Poland

###### **Correspondence:** J. Wojtowicz

**Introduction:** Chronic non-bacterial osteomyelitis (CNO) or chronic recurrent multifocal osteomyelitis (CRMO) are rare autoinflammatory bone diseases that occur in children. There are diagnosed on the basis of a pooled analysis of clinical data, results of imaging studies and histopathological examinations, if needed.

**Objectives:** The diseases are not widely known. First symptoms, auch as pain, swelling and tenderness can be therefore misinterpreted. Moreover absence of deviations in laboratory assays and initialy correct imaging results (especially x-ray) can extend time from the first symptoms to diagnosis.

**Methods:** The study presents a retrospective analysis of clinical data of 45 patients, diagnosed and treated because of CNO/CRMO at our Department over the past 13 years (2009-2022) with regard to the time from first symptoms to diagnosis.

**Results:** The median time from first symptoms to CNO/CRMO diagnosis in the group was 13 months (minimum 1,5 months; maximum 107 months). The difference in time to diagnosis between the sexes was not statistically significant (girls median 22 months, boys 11 months). There was a weak positive correlation between the age of the patient and the time to diagnosis (r 0,34, p=0,03). The crucial result of the analysis is a strong negative correlation between the year of firts symptoms of CNO/CRMO (2003-2022) and a time to diagnosis (r -0,63, p <0,001).

**Conclusion:** The analysis of the clinical data from our center shows the significant reduction in time to diagnosis of CNO/CRMO in children over the last 13 years. It depends mainly on better and better ability to distinguish symptoms reported by children with joints and bone complaints and ordering targeted diagnostics, including the whole body magnetic resonanse imaging, at our department. This may depend also on greater awareness of the diseases among medical doctors (especjally surgeons, orthopedic surgeons and oncologist) who referre children to the paediatric rheumatology department faster than in the past. However none active educational campain has been organised yet.

**Trial registration identifying number:**

**Patient Consent:** Yes, I received consent

**Disclosure of Interest**: None declared

### P180. Encephalitis due to Anti-N-Methyl-D-Aspartate receptor antibodies in a pediatric patient first case report andliterature? Review

#### F. L. Zaldumbide-Alcocer, Y. R. Loyola on behalf of Hospital de especialidades del niño y la mujer, Querétaro, México

##### Pediatría, Hospital de especialidades del niño y la mujer, Querétaro, Mexico

###### **Correspondence:** F. L. Zaldumbide-Alcocer

**Introduction:** Autoinmune encephalitis (AD) due to anti-NMDA receptor antibodies (anti-NMDAr) isa subacute encephalopathy that can progress to severe encephalopathy. In Mexico, the incidence is unknown, however the world literature? reports cases associated with paraneoplastic syndrome with a predominance in females. It is the most common AD in pediatric patients, although the paraneoplastic association is less frequent.

**Objectives:** The objective of this research is to present the first case of autoinmune encephalitis due to anti NMDA antibodies in our Hospital, as well as to realize a review of the pathology.

**Methods:** We present the case of a 10 year old male patient who began suffering from infection in the upper airways (sinusitis) characterized by fever spikes and general malaise, receiving antibiotic therapy with second-generation cephalosporins with remission of the condition. Ten days later, he begins with disorganized thoughts, bradilalia, bradypsychia, psychomotor agitation, delusional ideas, visual hallucinations, insomnia, anxiety, facial dyskinesias, behavioral and conductive alteration, inattention, rapid and incongruous speech, bilateral dysmetries. Physical examination Roomberg doubtful positive, stiff neck and hiperreflexia.

**Results:** Lumbar? puncture was realized and showed 63% NM, 37% PMN, glycorrhachia 59, proteinorachia <2, DHL 23. Negative results for BAAR, Gram stain, india ink, cerebrospina fluid culture, respiratory viral panel and hepatitis. Positive anti -NMDA antibodies were demonstrated.

Electroencephalogram with abnormal activity in the right fronto-temporal region frequently, sometimes bilateral, slowing of the base activity. Magnetic resonance T2 FLAIR cortical restriction in both temporo-occipital lobes, predominantly left, with inflammatory changes of edema. Encephalic and extrapyramidal syndrome was integrated. During hospitalization, the patient presented symtoms of neurological deterioration with status epilepticus requiring intensive care management and phase III ventilation. Extensive? studies were performed with the intentional search for associated tumors that weren´t demonstrated. Testicular ultrasound did not report seminoma.

Phatology of endocrine origin was ruled out. The management with antipsychotics, benzodiazepines, anticonvulsants and steroids with pulses 30mgkgdose for 5 days associated with gammaglobulin 2grkgdo. Given with favorable response to first line management, he was discharged 31 days after the onset of symptoms with reduced steroid management, anticonvulsant, antipsychotics and maintenance inmunossupressants.

**Conclusion:** In patients with encephalic syndromes, it is important to consider autoimmune encephalitis due to anti -NMDA receptor antibodies as a differential diagnosis, since this pathology may be under-diangosed in our enviroment, timely diagnosis and treatment can considerably reduce morbidity and mortality.

**Patient Consent:** Yes, I received consent

**Disclosure of Interest**: None declared

## Poster session: Uveitis

### P181. Prevalence, demographic and clinical patterns of uveitis in juvenile idiopathic arthritis at a tertiary care pediatric hospital in Libya

#### A. A. Abushhaiwia^1^, S. T. Ashur^2^, M. Zletni^3^, A. Ganbour^4^

##### ^1^Pediatric Rheumatology, Tripoli Children’s Hospital; Faculty of medicine; University of Tripoli, ^2^Family and Community Medicine, Faculty of Medicine, University of Tripoli, Libya, ^3^Paediatric Rheumatology , Faculty of Medicine,Tripoli university, Tripoli Children’s Hospital , ^4^Paediatric Rheumatology , Tripoli Children’s Hospital , Tripoli , Libya

###### **Correspondence:** A. A. Abushhaiwia

**Introduction:** Juvenile idiopathic arthritis (JIA) is the most common childhood rheumatic disease. The development of associated uveitis represents a significant risk for serious complications, including permanent loss of vision. Initiation of early treatment is important for controlling JIA-uveitis, but the disease can appear asymptomatically, making frequent screening procedures necessary for patients at risk. It is difficult to assess which JIA patients are at risk of developing uveitis. In the present study, we described the prevalence and clinical profile of JIA-associated uveitis and out come among children in the Libyan clinical settings.

**Objectives:** The present study described the prevalence, demographic and clinical profiles of uveitis in children diagnosed with JIA in a referral pediatric hospital in Libya. The study also compared the demographic and clinical characteristics of children who developed uveitis with those who did not develop uveitis, and examined the association between these characteristics and having uveitis

**Methods:** A total of 90 JIA patients who fulfilled International League of Associations for Rheumatology (ILAR) diagnostic criteria were included in this retrospective study. The data collected were age, gender, age at disease onset and at diagnosis, and follow-up duration. Duration from JIA diagnosis to uveitis diagnosis. Antinuclear antibody (ANA), RF, and human leukocyte antigen B-27 were evaluated for each patient

**Results:** A total of eight uveitis cases were identified among the 90 JIA cases, which gives a prevalence of 8.9%. All cases were females (100.0%), the majority were Libyans (87.5%), and their mean age was 12.3 (SD=4.3) years old. The mean age at JIA onset for this group was 5.3(SD=2.3) years old and that for JIA diagnosis 6.2 (SD=2.6) years old, with an average duration of JIA of 6.8 (SD=3.7) years. The most common JIA subtype in this group was oligoarthritis (50%), followed by poly arthritis (37.5%). The majority of the cases had a negative rheumatoid factor test (75%), and ANA was positive only in 1 of 7 valid cases (14.3%).The mean age at uveitis diagnosis was 8.2 (SD=3.7) years old. The median duration from time at diagnosis of JIA till uveitis diagnosis was 1 (IQR=1-1.7) year. Only 1 out of 7 valid uveitis cases (14.3%) was symptomatic, and it was presented with eye redness. The course of uveitis was acute in 3 out of 6 cases (50.0%). Anterior uveitis constituted 62.5% of all uveitis types, and 66.6% of the cases had unilateral eye involvement. Over half of the patients (62.5%) were on follow-up for uveitis for duration of at least 3 months, while the remaining cases (37.5%) had a follow-up duration shorter than one month. Half of the cases had impaired visual acuity. Concerning complications, cataract and synechiae were the commonest (37.5%), followed by cataract, synechiae and band keratopathy in a quarter of cases. All cases were on a combination of systemic steroids and methotrexate (87.5%).The types of the biological medications varied considerably across those who received biological therapy, however, Etanercept was the most popular one, as it was used either as a single biological treatment (25%), then Infliximab (12.5%), and Adalimumab (12.5%).

**Conclusion:** early recognition of uveitis in JIA is required to improve outcome,the reasons for our lower complication rates and better visual outcome may be the more frequent use of systemic immunosuppressive agents (particularly methotrexate and anti- TNF agents), and close collaboration between our pediatric ophthalmologists and pediatric rheumatologists

**Patient Consent:** Yes, I received consent

**Disclosure of Interest**: None declared

### P182. Methotrexate therapy associated with lower risk for uveitis-onset in juvenile idiopathic arthritis

#### G. Akay^1^, J. W. Straalen^1^, C. V. Kouwenberg^2^, N. M. Wulffraat^1^, S. de Roock^1^, J. H. de Boer^2^, J. F. Swart^1^

##### ^1^Department of Pediatric Immunology and Rheumatology, Wilhelmina Children’s Hospital, ^2^Department of Ophthalmology, University Medical Center Utrecht, Utrecht, Netherlands

###### **Correspondence:** G. Akay

**Introduction:** Juvenile idiopathic arthritis associated uveitis (JIA-U) is a common complication of juvenile idiopathic arthritis (JIA), with a cumulative incidence of 11.4% (1). It can result in significant visual impairment and blindness if not treated in a timely manner. Risk factors for uveitis-onset include oligoarticular JIA, ANA-positivity, young age at JIA onset and short JIA disease duration (2,3).

**Objectives:** To investigate if the risk for new-onset uveitis in children with JIA is lower when treated with (different doses of) methotrexate (MTX) compared to treatment with other first- and second-line therapy. A second objective is to assess if this risk increases after MTX-discontinuation.

**Methods:** For this case-control study, JIA-U cases from the University Medical Centre Utrecht (UMCU) were matched 1:1 to JIA control patients based on known risk factors for new-onset uveitis. Patient characteristics and medication use during the observation period were compared between JIA-U cases and JIA controls. Multivariable Cox regression analysis adjusted for JIA subtype, ANA-positivity and age at JIA onset was used to evaluate the effect of (different doses of) MTX on new-onset uveitis. MTX therapy was entered into the model as a time-varying covariate.

**Results:** 54 out of 160 identified JIA-U cases were eligible, yielding a total of 108 matched JIA patients with similar uveitis-risk at baseline for cases and controls. The ever-use of drugs did not differ significantly between JIA-U cases and controls, although the ever-use of MTX was higher in JIA controls (n = 36, 66.7%) compared to JIA-U cases (n = 26, 48.1%) (p = 0.05). The number of exposure years for MTX was also higher for JIA controls (median 0.56 years, IQR 0 – 1.62) compared to JIA-U cases (median 0 years, IQR 0 – 1.53) but was not statistically significant (p = 0.18). However, the adjusted hazard ratio (HR) for new-onset uveitis during the observation period as obtained from the survival analysis was significantly decreased for MTX therapy compared to no MTX therapy with a HR of 0.28 (95% CI 0.13-0.58). We observed no MTX dose differences between low (<10 mg/m^2^/wk) and normal dose (≥10 mg/m^2^/wk). Of JIA-U cases, 26/54 (48.1%) were treated with MTX prior to uveitis-onset. Upon occurrence of new-onset uveitis, 34.6% of MTX-users even had ongoing MTX-therapy at that time, while 65% discontinued MTX prior to uveitis-onset. New-onset uveitis occurred most often (82.4%) within the first two years after discontinuation and half of them were during the first 6 months.

**Conclusion:** Our results demonstrate a significantly reduced risk for new-onset uveitis in patients on MTX therapy after adjusting for known risk factors for JIA-U. We therefore suggest earlier initiation of MTX in JIA patients at high risk for new-onset uveitis. Furthermore, we advise more frequent ophthalmologic screenings after MTX-discontinuation since this is in itself a risk factor for new-onset uveitis.

**References:**

1. Carvounis PE, Herman DC, Cha S, Burke JP. Incidence and outcomes of uveitis in juvenile rheumatoid arthritis, a synthesis of the literature. Graefe’s Arch Clin Exp Ophthalmol. 2006 Mar;244(3):281–90.

2. van Straalen JW, Giancane G, Amazrhar Y, Tzaribachev N, Lazar C, Uziel Y, *et al*. A clinical prediction model for estimating the risk of developing uveitis in patients with juvenile idiopathic arthritis. Rheumatology (Oxford). 2021 Jun 1;60(6):2896–905.

3. Angeles-Han ST, Yeh S, Vogler LB. Updates on the risk markers and outcomes of severe juvenile idiopathic arthritis-associated uveitis. Int J Clin Rheumatol. 2013 Feb 1;8(1):109–21.

**Patient Consent:** Not applicable (there are no patient data)

**Disclosure of Interest**: None declared

### P183. Non-infectious isolated uveitis in children: clinical features and multimodal imaging of a challenging condition

#### G. B. Beretta^1^, G. Leone^2^, M. Rossano^1^, E. A. Conti^1^, G. Rogani^1^, F. Baldo^1^, S. Testa^1^, F. Viola^2^, G. Filocamo^1^, F. Minoia^1^, C. Mapelli^2^

##### ^1^Pediatric Rheumatology, ^2^Ophtalmology Unit, FONDAZIONE IRCCS CA’ Granda Ospedale Maggiore Policlinico, Milan, Italy

###### **Correspondence:** G. B. Beretta

**Introduction:** Although rare, uveitis is the main cause of visual morbidity in children. Juvenile idiopathic arthritis (JIA) is the most frequently associated disease in pediatric uveitis; however, 28–51% of non-infectious uveitis remains idiopathic and still represents a major challenge. So far, there is a lack of evidence about pediatric non-infectious isolated uveitis (NII-U), especially regarding its characterization with multimodal imaging.

**Objectives:** To describe clinical course, ophthalmological features, multimodal imaging and management of a monocentric cohort of pediatric NII-U.

**Methods:** Medical records of pediatric-onset NII-U were retrospectively reviewed regarding systemic and ocular features, treatment and outcome. Ophthalmological evaluation included best corrected visual acuity (BCVA), slit lamp biomicroscopy, intraocular pressure measurement and fundus examination, and it was integrated with fluorescein angiography (FA), indocyanine green angiography (ICGA) and optical coherence tomography (OCT) when needed. NII-U data were compared with the cohort of JIA-associated uveitis (JIA-U) followed up at the same service. Quantitative and qualitative variables were analysed by means of Mann-Whitney U test or chi-square/Fisher exact test, as appropriate.

**Results:** Data from 32 children (53% males) with NII-U were collected. Median age at uveitis diagnosis was 11.3 years (IQR 5.25) and median duration of follow-up was 1.9 years (IQR 3.0). Panuveitis, anterior and intermediate uveitis were diagnosed in 41%, 38% and 22% of cases, respectively; in 12 patients a granulomatous aspect was observed. In 67% of patients (82% of panuveitis) symptoms were present at onset, primarily ocular redness (48%), photophobia (22%), visual loss (26%) and eye pain (15%). Antinuclear antibodies (ANA) were positive only in 10 patients (6 anterior uveitis). In more than half of NII-U ocular damage was observed at diagnosis. FA, ICGA and OCT showed abnormalities in 88% of cases (100% of panuveitis) and major findings are described in Table 1. Methotrexate or cyclosporine were used in 59% and 13% of cases, respectively, with a median interval from diagnosis of 3.7 (IQR 5,6) months. A biological medication was required in 14 children after a median of 13.4 (IQR 21,0) months from diagnosis. At last visit, more than a third of NII-U patients still present active uveitis, with a BCVA < 4/10 reported in 10% of cases. Compared to JIA-U, NII-U was less common in females (47% vs 86%, p 0.0003) with a higher median age at onset (11.3 vs 5.0 years, p <.00001). NII-U patients were more frequently symptomatic (67% vs 0%, p < .00001), with a higher incidence of ocular damage at onset (40% vs 7%, p 0.0007) and a higher frequency of active uveitis at last visit (31% vs 7%, p 0.006).

**Conclusion:** Pediatric NII-U is a challenging condition with a potential impact on visual outcome. Multimodal imaging is crucial in the proper definition of uveitis and should always be included in the diagnostic work-up of NII-U, especially when posterior segment’s involvement is suspected.

**Patient Consent:** Yes, I received consent

**Disclosure of Interest**: None declared

**Table 1 (abstract P183).** See text for description

	NII-U N=32	Anterior NII-U N=12	Intermediate NII-U N=7	Panuveitis NII-U N=13
Optic disc involvement	21 (66)	3 (25)	5 (71)	13 (100)
Macular edema	5 (16)	1 (8)	3 (43)	1 (8)
Epiretinal membrane	2 (6)	0	0	2 (15)
Retinal vasculitis	21 (66)	2 (17)	6 (86)	13 (100)
Retinal Ischemia	5 (16)	0	2 (29)	3 (23)
Retinal neovascularization	2 (6)	0	1 (14)	1 (8)
Retinoschisis	2 (6)	0	2 (29)	0
Chorioretinal scar	6 (19)	0	1 (14)	5 (38)
Choroidal alterations in ICGA	10 (32)	0	0	10 (77)

### P184. Treatment and prognosis of of chronic non infectious uveitis in rheumatology unit in tripoli children hospital

#### S. Hashad^1^, H. M. Atyari^2^, M. N. Altofeel^3^

##### ^1^Rheumatology unit, Tripoli children hospital, Tripoli, Libya, ^2^Pediatric rheumatology , Tripoli Children Hospital , Tripoli , Lebanon, ^3^Pediatric, Rheumatology , Tripoli , Libya

###### **Correspondence:** S. Hashad

**Introduction:** Noninfectious uveitis is an autoimmune mediated inflammation of the uveal tract, and it is a potentially sight threatening condition with associated consequences on the quality of life of these patients and high costs for the health system. It represents 12% of cases in pediatric rheumatology clinic which is the only clinic caring for non-JIA autoimmune uveitis in all Libya. Pediatric uveitis is a topic of special interest because of its diagnostic and therapeutic challenges. In the last decade, many biomarkers have been identified to help stratify the risk of uveitis and many new treatment modalities were introduced

**Objectives:** • To study the demographic and clinical characteristics of JIA and non-JIA associated autoimmune uveitis.

• To Study and compare response to treatment in both categories.

**Methods:** The medical records of the patients with uveitis presented to rheumatology clinic from January 2000 to September 2021 were reviewed and data collected about the demographics (Gender, Date of birth, address) clinical data: date at presentation to ophthalmology, date at presentation to rheumatology, diagnosis, clinical findings at presentation, complications at presentation, treatment and number of flares before presentation, treatment during follow-up, slitlamp examination findings at last visit, complication of systemic treatment

**Results:** A total of 75 cases of uveitis were presented in rheumatology OPD in the research period. Mean age at uveitis onset was 8.3±3.4 the patients years and mean follow-up period of 2.8±2.5 years. JIA associated uveitis represent 32% of cases (62% of them are with various JIA subclasses and 37.5% JIA froste forme) and 68% of them are nonJIA associated. Table 1 summarizes the diagnosis of all cases. Of the JIA cases 54.2% were symptomatic while 100% of the non-JIA uveitis were symptomatic. Anterior uveitis was the most common location in JIA associated uveitis (58.3%) while panuveitis was the most common location in nonJIAassociated uveitis (74.5%). of the cases of JIA cases 62.5% were bilateral and nonJIA associated uveitis 88.2% were bilateral. Methotrexate was the drug used in most cases of JIA and nonJIA associated uveitis (91% of total cases). Oral prednisolone was used in 66.7% of JIA associated cases while it was used in 80.4% of nonJIA cases. Biologics were needed in the treatment of 3 (12.5%) cases of JIA associated uveitis while they were neededin 9 (17.6%) of non-JIA cases. Intraocular injections were not used in JIA associated uveitis while it was used in 8 (15.7%) of the nonJIA associated uveitis. Of the JIA cases 2 (8.3%) underwent operations and of nonJIA cases 10 (19.6%) underwent operations. Of the JIA cases 2 (8.3%) underwent operations and of nonJIA cases 10 (19.6%) underwent operations. In JIA cases remission was achieved in 75% of the cases with MTX and 95% Of cases with biologics. In nonJIA associated uveitis remission was achieved in 70% of cases with MTX and in 90% of cases with first biologic and in 95% of cases with second biologic. Of JIA cases 58.3% had complications at last visit while 72.5% of nonJIA cases had complications in JIA and nonJIA associated uveitis.

**Conclusion:**

Biological and nonbiological DEMARDs are effective in introducing maintaining remission in nonJIA associated uveitis with various diagnosis.

**Disclosure of Interest**: None declared

**Table 1 (abstract P184).** See text for description

Diagnosis	Frequency	Percent
JIA associated	24	32.0%
Behcet disease	5	6.7%
Sarcoidosis	3	4%
Chronic idiopathic	40	53.3%
Vogt-koyanagi-haradadisease	2	2.7%
Orbitalpseudotumor	1	1.3%
Total	75	100.0%

### P185. Paediatric uveitis at the royal Victoria infirmary: looking back and forwards

#### S. Jandial, S. Cairns

##### Paediatric Rheumatology, Great North Children’s Hospital, Newcastle, United Kingdom

###### **Correspondence:** S. Jandial

**Introduction:** Uveitis is an inflammatory eye disorder that if untreated can lead to irreversible damage such as cataract and glaucoma. In children, uveitis is a common association with Juvenile Idiopathic Arthritis (JIA) with a well-established screening guideline for all newly diagnosed JIA patients. Uveitis in non-JIA patients requires careful scrutiny for an underlying diagnosis but many will be labelled as ‘idiopathic uveitis’. Regardless of the underlying diagnosis, management of chronic uveitis requires immunosuppression to prevent persistent inflammation and structural damage to the eye. The immunosuppressive approach is similar regardless of the underlying diagnosis and needs multidisciplinary (MDT) care and careful management.

**Objectives:** In Newcastle (UK), children with uveitis are managed in the Newcastle Eye Centre with rheumatology care provided in the Great North Children’s Hospital. A monthly paediatric uveitis clinic exists but is under considerable strain and is without a uveitis clinical nurse specialist (CNS); we therefore undertook a structured evaluation of the current service to support service development led by a paediatric rheumatology CNS on temporary secondment to support the uveitis service.

**Methods:** Retrospective review of patient data currently attending the paediatric uveitis service at the Newcastle Eye Centre and Great North Children’s Hospital in 2021 with focus on patient number, diagnosis and treatments. Additional information about safeguarding was collected. Patients and carer feedback in a snapshot time period in 2021 was collected via a QR code link to an online survey.

**Results:** A total of 202 patients were known to the paediatric rheumatology team and coming to the uveitis service; 58/202 (29%) for uveitis screening, 5/202 (2%) were shared with renal, 62/202 (31%) had idiopathic uveitis and 77/202 (38%) with JIA-associated uveitis.

Of patients on treatment, 56/202 (27%) were not on treatment, 104/202 (51%) were on a single immunosuppressive agent (either adalimumab or methotrexate) and 42/202 (21%) were on multiple immunosuppressive agents (methotrexate, azathioprine, mycophenolate mofetil, adalimumab, infliximab, tozilizumab, abatacept).

67/202 (33%) were ready for transitional care (13yrs and older) which was not routinely planned for.

Patient feedback was collected from 16/50 patients seen in June 2021. Satisfaction was high (12/16, 76% very satisfied) and particularly praised the clinical nurse specialist ‘The nursing team have always been very reassuring with myself and my son”.

A patient and parent information leaflet ‘All about uveitis’ was developed with patient and clinician input.

**Conclusion:** A significant number of patients attend the Newcastle Eye Centre for care related to paediatric uveitis. Three quarters of these patients are on immunosuppressive treatment, many on complex regimes, requiring multidisciplinary care including counselling, prescribing, blood monitoring and disease progression assessment. This can only be provided in a cross-specialty multidisciplinary service supported by both paediatric rheumatology and ophthalmology, with adult ophthalmology providing input for transitional care. Paediatric uveitis services need to be appropriately funded and must take into account the complex needs of multi-system inflammatory disease.

**Patient Consent:** Not applicable (there are no patient data)

**Disclosure of Interest**: None declared

### P186. Pediatric uveitis: single-center experience for six years

#### M. Kasap Cuceoglu^1^, Y. Kapucu^2^, A. Keskin^3^, S. L. Sadigh^2^, S. Sener^1^, Z. Balik^1^, Y. Bayindir^1^, E. Aliyev^1^, O. Basaran^1^, E. D. Batu^1^, S. Kadayıfcilar^2^, S. Ozen^1^, Y. Bilginer^1^

##### ^1^Pediatric Rheumatology, ^2^Ophtalmology, ^3^Pediatrics, Hacettepe University, Ankara, Turkey

###### **Correspondence:** M. Kasap Cuceoglu

**Introduction:** The inflammation of uveal structures (iris, choroid, retina) is called uveitis which is anatomically classified into anterior, intermediate, posterior and panuveitis.^1^ At the time of diagnosis, it may present acute or chronic course. Chronic anterior uveitis, particularly in juvenile idiopathic arthritis, might lead to visual impairment and blindness due to its asymptomatic and latent course.^2^

**Objectives:** This study aimed to evaluate the data of pediatric patients with uveitis in our outpatient clinic and statistically exhibit our findings.

**Methods:** 131 patients (<18yo) with uveitis from 553 patients were retrospectively screened using the electronic medical records between January 2015 and January 2021 at Hacettepe University. Demographic and clinical characteristics of patients, drug treatments, frequency of recurrence, and uveitis-related ocular complications were indicated.

**Results:** A total of 190 eyes (68 bilateral, 23 right eyes, 31 left eyes) of 131 patients were assessed, including 70 female (53.4%). The median age at diagnosed for uveitis was 7 (1-17) years. The association of uveitis was recorded in patients of 58 (44.2%) isolated uveitis, 55 (41.9%) juvenile idiopathic arthritis, 11 (8.3%), Behçet’s disease, 2 (1.5%) sarcoidosis, 1 (0.7%) Cogan syndrome. Anterior uveitis was the most prevalent anatomic type (n=101; 77.1%) followed by 14 (10.7%) panuveitis, 10 (7.6%) intermediate uveitis, 6 (4.6%) posterior uveitis. Eighty-seven of the patients were treated with corticosteroid drops, intraocular corticosteroid injection in 12 patients, systemic corticosteroids in 13 patients, synthetic DMARD in 55 patients, and biological DMARD in 43 patients were used. Of biological agents, 34 (75.6%) patients received adalimumab, 7 (15.6%) infliximab, 2(4.4%) tocilizumab, 2 (4.4%) alpha-2a interferons. The ocular complications associated with uveitis were cataracts in 23 patients, posterior synechiae in 12 patients, band keratopathy in 7 patients, glaucoma in 6 patients, macular edema, retinal vasculitis/scar in 3 patients, and vitriol in 2 patients. Intraocular surgery was performed on nine patients who developed uveitis-related ocular complications. Finally, 74 patients had a recurrence of uveitis.

**Conclusion:** Juvenile idiopathic arthritis is the most common rheumatological disease that causes non-infectious chronic uveitis in the childhood. Isolated uveitis is also widely seen. Patients should be closely monitored to prevent possible eye complications, and appropriate treatment strategies should be carefully applied.

**References**

1. Sen. ES, Ramanan A V. Juvenile idiopathic arthritis-associated uveitis. *Clin Immunol*. 2020;211:108322. doi:10.1016/j.clim.2019.108322

2. Gautam Seth N, Kaur S, Yangzes S, et al. Ophthalmic Complications in Pediatric Uveitis. *Ocul Immunol* Inflammation. Published online July 2020:1-6. doi:10.1080/09273948.2020.1762897

**Patient Consent:** Yes, I received consent

**Disclosure of Interest**: None declared

### P187. Ocular involvement in paediatric Behçet disease: a monocentric experience

#### I. Maccora, S. I. Orsini, I. Pagnini, M. V. Mastrolia, E. Marrani, G. Simonini

##### Rheumatology Unit, Meyer Children’s University Hospital, NeuroFARBA Department, University of Florence, Florence, Italy

###### **Correspondence:** I. Maccora

**Introduction:** Behçet syndrome (BS) is a rare disease in childhood and ocular involvement may lead to several complication including blindness if not properly recognized and treated.

**Objectives:** To describe a cohort of paediatric Behçet patients with ocular involvement and report the performed treatment.

**Methods:** This is a monocentric retrospective study based on chart review conducted at the Rheumatology Unit of Meyer Children’s Hospital, Florence. The study involved patients with a diagnosis of paediatric BS who fulfilled at least one of the following criteria: International Criteria for Behçet’s Disease Criteria, the International Study Group Criteria for BS, Paediatric BD classification criteria. Demographic, laboratory, and clinical data were collected at onset and during disease course. Ocular characteristics were evaluated, and the different treatment performed were assessed evaluating the outcome according to SUN criteria. Statistical Analysis were performed using SPSS v27.

**Results:** Among 33 paediatric patients with a diagnosis of BS, 9 children (27.3%) showed an ocular involvement. Among these 9 patients, 7 were male (77.8%), 5 had a family history for autoimmunity (55.6%), 3 had ANA positivity (33.3%) and 5 HLA B51 positivity (55.6%). The diagnosis of BS was performed at median age of 12.5 years old (IQR 10.1-15.1) while the diagnosis of uveitis at 12.5 years old (IQR 9-14.2). The median of ESR and CRP at disease onset were respectively 7 mm/h (IQR 2-29) and 0 mg/dl (IQR 0-0). Five patients showed a bilateral ocular involvement (55.6%), 2 showed a panuveitis (22.2%), 4 a posterior involvement (44.4%), 2 an intermediate involvement (22.2%), and 1 an anterior involvement (11.1%). Of these 9 patients, Five patients showed ocular signs and symptoms: 4 hyperaemia, 3 photophobia, 2 ocular pain, 4 blurred vision. One patient showed recurrent fever (11.1%), 8 recurrent oral ulcers (88.9%), 4 recurrent genital ulcers (44.4%), 2 gastrointestinal symptoms (22.2%), 3 cutaneous involvement (33.3%). All the patients received at least one treatment, 4 patients received 2 treatment and one patient 4 treatment to achieve disease control (1 methotrexate, 3 colchicine, 3 Azathioprine, 2 adalimumab and 1 canakinumab). Ocular control was achieved in all patients with the following drugs: 3 Adalimumab, 1 tocilizumab, 1 infliximab, 2 azathioprine, 1 colchicine and 1 systemic corticosteroid. The drug able to achieve ocular control was administered after a median time of disease onset of 2 months (IQR 1-30 months), with a median follow-up of 20 months (IQR 3.5-31.5months) and a median duration of therapy of 16 months (IQR 3-29 months). The median time to achieve a response to therapy was 3 months (IQR 2-3 months), and median time to achieve remission on therapy of 10 months (IQR 6-18 months). One patient treated with adalimumab, and one with azathioprine relapsed respectively after 11 months and 33 months from achievement of remission. The treatment was stopped in 2 patients (1 infliximab and 1 systemic corticosteroid) without relapse.

**Conclusion:** Ocular involvement in paediatric BS is one of the most common sight threatening complications. In our case series, a biologic was required in 5 children in order to achieve the ocular control in BS. Further studies in large cohort of paediatric BS are necessary in order to evaluate the most efficacious treatment of this sight-threatening complication.

**Patient Consent:** Not applicable (there are no patient data)

**Disclosure of Interest**: None declared

### P188. Subcutaneous tocilizumab in JIA related uveitis: a monocentric experience

#### A. Marino^1^, L. Marelli^2^, R. Caporali^1,3^, E. Miserocchi^4^

##### ^1^Pediatric Rheumatology, ASST Pini-CTO, ^2^University of Milan, Milan , Italy, ^3^Department of Clinical Sciences and Community Health and Research Center for Pediatric and Adult Rheumatic Diseases (RECAP.RD), University of Milan, ^4^Ophtalmology Department, Ospedale San Raffaele, University Vita-Salute, Milan, Italy

###### **Correspondence:** A. Marino

**Introduction:** Uveitis associated with juvenile idiopathic arthritis (JIA-U) is a cause of ocular morbidity. JIA-U might be difficult to treat since a substantial proportion of children are refractory to methotrexate (MTX) and TNF inhibitors (TNFi). Tocilizumab (TCZ) is a humanized monoclonal antibody to the IL-6 receptor preventing IL-6 from binding to its soluble and membrane-bound receptors; TCZ is approved for the treatment of systemic and polyarticular JIA. Few studies, mainly case series, explored the efficacy of intravenous TCZ in JIA-U, whereas little is known about subcutaneous TCZ (SC-TCZ) for uveitis in patients with JIA.

**Objectives:** To describe the efficacy and safety of SC-TCZ in a cohort of JIA patients with refractory uveitis.

**Methods:** Retrospective observational monocentric study. Patients with JIA-U treated with SC-TCZ were enrolled from the Pediatric Rheumatology Unit of Gaetano Pini Hospital, Milan, Italy from January 2011 to December 2021.

**Results:** Thirteen patients (9 female) were enrolled: 8 patients with oligoarticular JIA and 4 with polyarticular JIA. All patients were ANA positive. The age at articular disease onset was 2.2 ± 2.1 (0.8-6.5) years; the age at uveitis onset was 4.5 ± 2.8 (1.8-10.6) years. The mean follow-up time was 19.3 ± 9.2 (6.8-32.3) years. Ten patients had bilateral uveitis. Uveitis location included anterior uveitis (8 patients and 15 eyes), anterior and posterior (1 patient and 1 eye), intermediate uveitis (1 patient and 2 eyes), posterior uveitis (1 patient and 1 eye), and panuveitis (2 patients and 4 eyes).

All patients have been treated with synthetic disease modifying antirheumatic drugs (sDMARDs): all received methotrexate (MTX), 8 of them received cyclosporine too. However, just three patients received MTX in combination with SC-TCZ. The mean number of biological DMARDs (bDMARDs) was 2.4 ± 1.3 (1-5). The bDMARDs which had been used before starting SC-TCZ included adalimumab in 12 patients, infliximab in 7 patients, other TNFi in 10 patients (4 etanercept, 3 certolizumab, 3 golimumab), and 2 subjects received rituximab. Descriptors of subcutaneous tocilizumab treatment in the cohort are reported in Table 1. Two patients started SC-TCZ for articular flare and do not experienced uveitis flare during the anti-IL6 treatment. Overall, SC-TCZ was safe, and no side effects were observed during the treatment period. Three patients discontinued SC-TCZ: 2 patients due to secondary inefficacy (switched to golimumab and rituximab, respectively), 1 subject due to loss of compliance (switched to oral tofacitinib). One patient withdrawn the drug because of pregnancy.

**Conclusion:** Subcutaneous tocilizumab can be effective and safe in patients with JIA and uveitis recalcitrant to several bDMARDs. Further studies, with larger cohorts and prospectively designed, are advisable to verify this opportunity.

**Patient Consent:** Yes, I received consent

**Disclosure of Interest**: None declared

**Table 1 (abstract P188).** Descriptors of subcutaneous tocilizumab treatment in the cohort

Age at SC-TCZ onset	21.5 ± 7.5 (6.3-33.2) years
Duration of uveitis at SC-TCZ onset	17.4 ± 7.1 (4-26.6) years
Duration of SC-TCZ therapy	2.6 ± 1.9 (0.6-5.5) years
Flare/year rate on SC-TCZ	0.4 ± 0.7
Flare/year rate on bDMARDs before SC-TCZ	1.6 ± 2.0

SC-TCZ: subcutaneous tocilizumab; bDMARDs: biologic disease modifying antirheumatic drugs

### P189. Juvenile idiopathic arthritis-associated uveitis: a bibliometric and network analyses

#### M. Pavlenko^1^, D. Pavlenko^2^

##### ^1^Universum Clinic, ^2^Department of Ophthalmology, Bogomolets National Medical University, Kyiv, Ukraine

###### **Correspondence:** M. Pavlenko

**Introduction:** Uveitis is the most common extra-articular manifestation of juvenile idiopathic arthritis (JIA) and may result in sight-threatening complications.

**Objectives:** To explore the current state of publications on JIA-associated uveitis and to characterize it using a bibliometric approach.

**Methods:** An advanced search using the query [(“juvenile rheumatoid arthrit*” AND (uveit* OR iridocyclit*)) OR (“juvenile idiopathic arthrit*” AND (uveit* OR iridocyclit*))] was performed in May 2022 within all titles in the Core Collection of the Clarivate Web of Science database. Only publications until 2021 were included. The predominant language, h-index of the research topic, and the number of citations were identified according to the built-in Analyze Result and Citation Report. Biblioshiny application of Bibliometrix (Aria, M. & Cuccurullo, C., 2017) R-package (RStudio, PBC, Boston, MA, USA) was utilized for scientific production, author, authors collaboration, and citation analyses. Network visualization cluster analysis was conducted in VosViewer (Leiden University, Leiden, The Netherlands).

**Results:**

The search yielded 496 items. After 5 items that were published in 2022 and 7 irrelevant items were excluded, 484 papers were analysed. They were published starting in 1970 by 1660 authors in 98 sources, and 97.7% (473 of 484) of them were written in English. Articles (n=225; 46.5%) and meeting abstracts (n=148; 30.6%) comprised the majority of the publications. Singled-authored document were rare (n=22; 4.5%) and were written by 1.0% (n=17) of all authors. The average years from publication was 11.1. The collaboration index was 3.6. There were 0.3 documents per author and 3.4 authors per document and 6.8 co-authors per document. The annual growth rate was 1.6%. The most productive year was 2019 (n=42; 8.7%). Bradford’s law showed that only the following journals formed the core sources: *Annals of the Rheumatic Diseases* (n=50; 10.3%), *Arthritis & Rheumatology* (n=35; 7.2%), *Journal of Rheumatology* (n=33; 6.8%), *Ocular Immunology and Inflammation* (n=33; 6.8%), *Arthritis and Rheumatism* (n=29; 6.0%). Top-contributing authors included Heiligenhaus A (n=61; 12.6%), Ramanan AV (n=27; 5.6%), and Minden K (n=26; 5.4%). Heiligenhaus A (h-index=23), Heinz C (h-index=15), and de Boer JH (h-index=14) had the highest local impact. The most prolific institutions included St. Franziskus-Hospital (n=48; 9.9%), Emory University (n=33; 6.8%), Johns Hopkins University (n=31; 6.4%), and the University of Duisburg-Essen (n=31; 6.4%). The h-index of the research field was 54. Totally, there were 9,191 citations with average citations per document of 19.0 and average citations per year per document of 1.8. Each publication had an average of 7.4 references. Shown in Table 1, the United States was the most common corresponding author’s country (106 of 341; 31.1%) and had the highest number of total citations (2,213 of 8,093; 27.3%). Switzerland had the highest average article citations (52.75 citations/article). Network co-authorship analysis showed that 21 countries, each with at least 5 documents, formed 6 clusters; Germany, Switzerland, and the United States had the highest link strength.

**Conclusion:** The current study of publications related to juvenile idiopathic arthritis-associated uveitis identified the major contributors, co-authorship relationship, and article citations. The United States was the most prolific country. Switzerland produced the most influential papers. Germany showed the highest co-author collaboration. St. Franziskus-Hospital was the most productive institution.

**Patient Consent:** Not applicable (there are no patient data)

**Disclosure of Interest**: None declared

**Table 1 (abstract P189).** Top-7 countries ranked by productivity

Country	Scientific Production Frequency, n	Total Citations, n	Average Article Citations, citations/article
USA	403	2213	20.88
GERMANY	300	1747	36.40
UK	228	899	29.97
ITALY	204	723	23.32
SPAIN	175	294	22.62
CANADA	102	93	15.50
NETHERLANDS	91	501	26.37

## Poster session: Immunodeficiency and infection related arthritis

### P190. A case of 15-years-old girl with parvovirus-arthritis

#### A. Margaryan

##### WIGMORE CLINIC, Yerevan, Armenia

###### **Correspondence:** A. Margaryan

**Introduction:** Acute arthritis is one of the presentations of Parvovirus B19 beside erythema infectiosum and aplastic crisis.

**Objectives:** This is a case report of 15-years-old girl with acute polyarthritis due to parvovirus B19.

**Methods:** This is a case report based on medical chart of the patient.

**Results:** A 15-years-old girl presented to pediatrician on 7^th^ day of the following complains: pain in upper extremities, difficulty raising arms, fatigue, painful and swollen wrists, rash on palms.

On 2^nd^ day of illness CBC was performed: PLT 91*10^3^/L. Objective examination: general condition was stable, mild hyperemia of cheeks, red-purple papulose rash on palms, swollen wrists, painful and swollen small joints of hands with limited movements. Palpation of the upper extremities from the shoulders to the wrists were painful. No other changes: no changes in gait, no squatting difficulties, no changes in other joints, normal muscle strength, no hepatosplenomegaly. Laboratory findings: CBC’ normal (no anemia, no reticulocytosis, PLT’ 160*10^3^/L), ANA, ALT, AST, LDH, CK’ Normal, Covid-19 total Ab’ positive, Parvovirus B19 IgM’ positive, EBV IgM’ negative. Treatment with NSAID initiated. After two weeks the diagnosis of the parvovirus B19 was proven by seroconversion. Follow-up: after 4 weeks from treatment minor improvement in complains. NSAID had been continued and on 6^th^ week there were no any complains and examination was normal.

**Conclusion:** Parvovirus B19 associated acute arthritis is mostly self-limited and resolves with symptomatic treatment.

**Patient Consent:** Yes, I received consent

**Disclosure of Interest**: None declared

### P191. Synovial inflammation in antibiotic-refractory Lyme arthritis is characterized by clonally expanded peripheral T helper cells and TCR convergence

#### J. Dirks^1^, J. Klaussner^1^, A. Almamy^1^, G. Haase^1^, U. Fischer^1^, A. Holl-Wieden^1^, C. Hofmann^1^, H. Girschick^2^, H. Morbach^1^

##### ^1^Children’s University Hospital, Würzburg, ^2^Vivantes Klinikum Friedrichshain, Berlin, Germany

###### **Correspondence:** H. Morbach

**Introduction:** Antibiotic-refractory Lyme arthritis (ARLA) is defined by persistent arthritis despite adequate antibiotic treatment and may develop in up to 10 % of patients with Lyme arthritis. Disease pathogenesis of ARLA is still inadequately understood. In detail, whether chronic inflammation in ARLA is maintained by chronic antigen stimulation (e.g. by persistent borrelial antigens or autoantigens) is not elucidated yet.

**Objectives:** To dissect the cellular and molecular landscape of T helper cell responses in the inflamed joints of patients with ARLA.

**Methods:** Flow cytometric analysis of synovial fluid CD4+ T cells from children with ARLA and juvenile idiopathic arthritis (JIA). High-throughput sequencing of the T cell receptor Vβ (TCRVB) repertoire as well as single-cell RNA sequencing (scRNAseq) of sorted CD4+ T cell populations.

**Results:** Multidimensional flow-cytometric analysis revealed a striking expansion of an IL-21 and IFN-γ co-expressing PD-1hiCXCR5-HLA-DR+ CD4+ T cell population resembling peripheral T helper (TPH) cells in the joints of pediatric ARLA patients compared to JIA patients. Indeed, ARLA patients displayed the highest frequencies of TPH cells, which could separate this group of patients from JIA. Accumulating TPH cells exhibited signs of clonal expansion with restricted TCR clonotypes. Those clonotypes showed an overlap between different ARLA patients but not to JIA patients. SF TPH cells were enriched for distinct and overlapping TCRVB motives that showed signs of a convergent immune response and could not be identified in SF CD4+ T_PH_ cells of JIA patients.

**Conclusion:** The inflamed joints of children with ARLA are characterized by a striking expansion of oligoclonal TPH cells with signs of TCRVB convergence that is distinct from other forms of chronic arthritis (e.g. JIA). This peculiar pattern suggests that disease specific immune response may sustain chronic inflammation in ARLA. Current experiments are ongoing to dissect whether this maladaptive immune response targets persisting borrelial antigens or rather autoantigens.

**Disclosure of Interest**: None declared

### P192. Characterization of an ataxia-teleangectasia patient with inflammatory phenotype mimicking a rheumatologic disease

#### M. F. Natale^1^, L. De Nardi^2^, C. Celani^3^, F. De Benedetti^3^, A. Insalaco^3^

##### ^1^Rheumatology, Bambino Gesu Children’s Hospital, Rome, ^2^Rheumatology, IRCCS materno infantile Burlo Garofolo, Trieste, ^3^Rheumatology, Bambino Gesù Children’s Hospital, Rome, Italy

###### **Correspondence:** M. F. Natale

**Introduction:** It is well known that some inflammatory manifestations such as arthritis and lung diseases could be part of both rheumatological and immunological disease, representing a real diagnostic challenge. Ataxia telangiectasia (A-T) is a rare autosomal recessive condition characterized by skin telangiectasia, ataxia and immunodeficiency in which lungs and joints are frequently involved.

**Objectives:** We described an autosomal dominant A-T patient with a novel heterozygous mutation in ATM gene presenting with a clinical phenotype characterized by inflammatory manifestations, articular and lung involvement.

**Methods:** Whole-exome sequencing

**Results:** When the patient was first seen at our center at age 7 years , presented with a clinical picture characterized by poor growth, chronic liver granulomatous inflammation, interstitial lung disease with bilateral bronchiectasis and recurrent episodes of large joint ahrtritis (wrists, knees and ankles). The diagnosis of pediatric sarcoidosis had been done in another center by where a treatment with high dose systemic glucocorticoids and intraarticularar glucocorticoids injection were started with no significant improvement.

We performed the combined dosage of angiotensin converting enzyme (ACE) and chitotriosidase, resulted in the normal range excluding the diagnosis of pediatric sarcoidosis. In the suspicion of interferonopathy with pulmonary and joint involvement (COPA syndrome) an interferon signature was performed with a normal value (n.v. <2). Immunological blood exams showed a reduction in total immunoglobulin levels and in the absolute count of T and B lymphocytes, a marked reduction in thymic output (CD4Ra 1.4%), a population B with phenotypic characteristics of “ atypical B Memory“, abnormal NK cells function and very high levels of serum alpha-protein (273ng /ml)

Whole exome sequencing identified a novel homozygous mutation in ATM gene causing the ataxia-telangiectasia syndrome

**Conclusion:** This case emphasize the importance to consider a diagnosis of primary immunodeficiency (PID) even in patients with inflammatory lungs and joints involvement mimicking a rheumatologic condition.

**Patient Consent:** Yes, I received consent

**Disclosure of Interest**: None declared

### P193. Juvenile idiopathic arthritis associated to primary immunodeficiency in pediatric age: a case series

#### I. Pagnini, A. rossi, I. maccora, M. V. mastrolia, E. marrani, G. simonini

##### Aou Meyer, Firenze, Italy

###### **Correspondence:** I. Pagnini

**Introduction:** Primary Immunodeficiency (PID) are a group of more than 400 monogenic diseases with an estimated overall incidence of 1:10,000. The related immunoregulatory defects may predispose to development of autoimmune diseases, such as inflammatory arthropathies. Juvenile Idiopathic Arthritis (JIA) is the most common chronic rheumatic disease of unknown etiology in childhood. It occurs 50 times more common in DiGeorge syndrome (DGS) than in normal population, and has a prevalence of about 10% in Bruton agammaglobulinemia (BA).

**Objectives:** We describe clinical features, laboratory, imaging data and therapeutic outcomes in children with JIA and PID.

**Methods:** Among 450 JIA children followed at our unit, we enrolled 3 patients, followed in Pediatric Rheumatology Unit at Meyer Children’s Hospital in Florence, affected by JIA, according to ILAR criteria, and fulfilling the diagnosis of PID: 2 patients with DGS and 1 with BA.

**Results:** Case 1: male of 5 years old affected by DGS from 2 years old, who developed oligoarticular JIA. He was treated with NSAID and methotrexate, without benefit. He received two consecutive ankle injections, 6 months apart, in addition to 6 courses of oral glucocorticoids also. Etanercept was then added to methotrexate 8 months after JIA diagnosis. He then achieved remission over 2 months later.

Case 2: female of 5 years old affected by DGS, by 3 years and 11 months old of age, who developed oligoarticular JIA. She was treated with NSAID, joint injection and methotrexate, without clinical response after 8 months. Due to the increase of liver tests, methotrexate was then stopped, and Adalimumab started. At 6-month follow-up, she was in clinical remission.

Case 3: male of 10 years and 10 moths old affected by BA, since he was 9 years old, later developing polyarticular JIA. He was treated with NSAID, methotrexate, joint injections and three course of oral glucocorticoid with no benefits, plus IVIg at 400 mg/kg/monthly. Due to persistent clinical activity after 5 months, Adalimumab was then added. However, over the following 6 months new multiple joint injections, two course of oral glucocorticoid and increased of IVIg at immunomodulation dose was performed without achieving disease control. Adalimumab was switched to Etanercept and after 4 months he was in clinical remission on medications.

**Conclusion:** The association between autoimmune diseases, including JIA, and immunodeficiency is well described in literature, but scanty evidence is referred to the clinical outcomes according to the standard treatment. In our experience these patients showed severe disease, with recurrent arthritis, although several joint injections, oral glucocorticoids courses and methotrexate treatment. In our cohort, children exhibited no response to the conventional first step treatment with methotrexate, whilst achieved disease remission on medication using anti-TNF agents. Larger, multicenter cohort will be necessary to identify the optimal treatment for these specific patients.

**Patient Consent:** Yes, I received consent

**Disclosure of Interest**: None declared

## Poster session: Macrophage activation syndrome

### P194. CD8 T-cell expansion may compensate for natural killer cell dysfunction in mice with chronic excess IL-18

#### J. Varghese^1^, E. Landy^2^, V. Dang^1^, E. Peauroi^3^, L. Eisenlohr^3^, S. Canna^1^

##### ^1^The Children’s Hospital of Philadelphia, Philadelphia, ^2^University of Pittsburgh, Pittsburgh, ^3^University of Pennsylvania, Philadelphia, United States

###### **Correspondence:** S. Canna

**Introduction:** Systemic Juvenile Idiopathic Arthritis (SJIA) and Macrophage Activation Syndrome (MAS) are associated with both low peripheral Natural Killer (NK) cell numbers and NK dysfunction. They are also associated with chronically elevated and unopposed peripheral blood levels of the inflammasome-activated cytokine IL-18. NK cells constitutively express the IL-18 receptor and canonically their response to IL-18 is to augment the effects of other cytokines (like IL-12) on Interferon gamma (IFNg) production and cellular cytotoxicity.

**Objectives:** To determine the effects of chronic IL-18 excesson NK cell phenotype, distribution and function.

**Methods:** We assessed NK cells by flow cytometry and RNAseq in mice with transgenic expression of mature, excretable IL-18 (*Il18tg* mice).

**Results:** Similarly to observations in SJIA/MAS, *Il18tg* mice demonstrated decreased numbers of NK cells in peripheral blood, spleen, and liver. Such mice also demonstrated a concomitant increase in activated CD8 T-cells in these same organs. Transcriptionally, NK cells from *Il18tg* mouse spleens showed increases in a few innate and NF-kB-related pathways, with effects on specific cytokines like Csf2 (encoding GM-CSF). These findings were paralleled by in vitro stimulation of WT NK cells with various cytokine combinations, including IL-18. However, the main transcriptional program was dominated by increases in cell cycle and gene expression programs, suggesting rapid cellular turnover. Additionally, *Il18tg* NK cells show a likely compensatory downregulation of *Il18r1* at the mRNA and protein levels. This effect was diminished in *Il18tg* mice with selective deletion of Il18r1 on T cells (*Il18tg;Il18r1*^*delT*^), suggesting T-cell responses to IL-18 affect NK cell homeostasis. Type 1 Innate Lymphoid cells showed similar transcriptional changes but no appreciable diminution of numbers.

In vitro, NK cells from *Il18tg* mice died quickly after isolation, clouding assessment of in vitro killing or cytokine production. In vivo, *Il18tg;Il18r1*^*delT*^ were not protected from immunopathology induced by repeated stimulation through Toll-like Receptor 9 (TLR9-MAS) but rather showed more severe disease by some parameters, suggesting non-T cell response to IL-18 contribute to the more severe TLR9-MAS observed in *Il18tg* mice. The DNA poxvirus ectromelia causes an abortive infection in WT mice, but severe viral immunopathology in perforin-deficient or NK-depleted mice. *Il18tg* mice infected with ectromelia failed to expand NK cells or upregulate activation markers like CD49b or NKG2D, but nevertheless clear virus similarly to WT mice and did not develop immunopathology at early timepoints.

**Conclusion:***Il18tg* mice may be a valid model to examine the effects of chronic IL-18 on NK cell homeostasis and the inter-relationship of NK and CD8 T-cells. Preliminary results suggest CD8 T-cell expansion may overcome NK cell deficiency/dysfunction in the early fight against viral infection, but may nevertheless predispose to MAS-like immunopathology.

**Patient Consent:** Not applicable (there are no patient data)

**Disclosure of Interest**: J. Varghese: None declared, E. Landy: None declared, V. Dang: None declared, E. Peauroi: None declared, L. Eisenlohr: None declared, S. Canna Grant / Research Support with: AB2Bio, Novartis, SOBI, IMMvention Therapeutix, Consultant with: Simcha Therapeutics, Paid Instructor with: Clinical Viewpoints

### P195. Liver involvement in systemic juvenile idiopathic arthritis, a multi faced problem

#### R. Erkens^1,2^, M. Jansen^2^, J. Calis^1^, J. van Loosdregt^1^, S. Vastert^1,2^

##### ^1^Center for Translational Immunology, UMC Utrecht, ^2^Pediatric Rheumatology, Wilhelmina Children’s Hospital, Utrecht, Netherlands

###### **Correspondence:** R. Erkens

**Introduction:**

Recently several cases of Systemic Juvenile Idiopathic Arthritis (SJIA) with hepatic inflammation have been described and linked to Macrophage Activation Syndrome (MAS) or delayed drug reactions (DDR) to biologicals.

**Objectives:**

To identify characteristics that aided in the differential diagnostic process between two forms liver involvement in SJIA.

**Methods:**

Here we present two recent cases of liver involvement in SJIA and describe their clinical presentation, disease course, and response to installed treatment.

**Results:**

**Patient 1**

A girl of 2 years and 10 months, at the start of 2020 diagnosed with SJIA complicated by severe MAS. DNA HLH-panel, functional and cytolytic cell analysis showed no signs of underlying HLH associated abnormalities. Clinical improvement was induced by the combination of corticosteroids (GC), cyclosporine (CSA) and anakinra. Medication was tapered without problems. Early 2021 there was a flare of SJIA with a re-emergence of MAS, methylprednisolone (MP) pulses were given and anakinra was switched to canakinumab. Initially the patient seemed to improve clinically and fever disappeared, but the patient subsequently developed signs of hepatitis with conserved liver function and increasing transaminases, Galectin 9 and CLXL10 levels. Viral and autoantibody causes of hepatitis were excluded. Liver biopsy showed a mononuclear infiltrate with focal erytrophagocytosis. The combination of an activated INFy pathway and infiltration of activated monocytes and T cells on histology together with the history of MAS pointed to hepatic MAS. Canakinumab was continued, MP pulses initiated and CSA was added which resulted in clinical improvement. She is now in remission on canakinumab and CSA in tapering.

**Patient 2**

A previously healthy girl of 1 year and 8 months was diagnosed with SJIA in 2021. First line treatment with Anakinra was started with a good initial response. 10 days after start of anakinra there was a reemergence of fever. The anakinra dose was increased with a short lasting improvement. The patient developed a pruritic rash, hepatomegaly, increasing transaminases and eosinophilia without appearing clinically ill. Liver function remained intact. Infectious and autoantibody causes of hepatitis were excluded. The HLH/MAS criteria were carefully monitored, DNA and functional HLH tests were initiated. A PET-CT scan was performed that showed lymphadenopathy with increased metabolic uptake, suspicious for lymphoma. The pathology report of the lymph extirpation showed no signs of lymphoma but a striking eosinophilia. This together with a distinctly elevated CCL17/TARC and no clinical picture of MAS prompted the suspicion of a DDR. Anakinra was stopped and GC were added. Hepatitis improved after this but the SJIA flared as the patient developed a spiking fever. Canakinumab was started and clinical outcome improved. Patient is currently in remission and GC are being tapered.

**Conclusion:**

These two cases show that patients with liver involvement in the setting of SJIA can have a similar presentation with different implications for treatment. Differentiation between DDR and hepatic MAS is important, but can be challenging as a clear definition of hepatic MAS is lacking. Interestingly, both patients are carriers of the DRB1*15:01 allele, that has been associated with DDR to biologicals. Key factors that aided in the differential diagnosis were the extent of eosinophilia in blood and biopsy, the pruritic rash, and relatively low HLH parameters for a DDR. The history of recurrent MAS, IFNγ pathway activation and monocytic infiltration in the liver for the diagnosis of hepatic MAS. A broadly supported and well defined definition of Hepatic MAS is needed to boost collaborative research into this rare complication of SJIA.

**Patient Consent:** Yes, I received consent

**Disclosure of Interest**: R. Erkens: None declared, M. Jansen: None declared, J. Calis: None declared, J. van Loosdregt: None declared, S. Vastert Consultant with: Sobi and Novartis

### P196. Predictors of relapse of macrophage activation syndrome in patients with systemic juvenile idiopathic arthritis: cohort study

#### E. Krekhova^1^, E. Alexeeva^1^, I. Kriulin ^1^, T. Dvoryakovskaya ^2^, K. Isaeva^2^, R. Denisova ^2^, A. Chomakhidze^2^, O. Lomakina^2^, A. Mamutova^2^, A. Fetisova^2^, M. Gautier^2^, C. Chibisova^2^, I. Tsulukia^2^

##### ^1^Pediatric, Sechenov First Moscow State Medical University, ^2^Rheumatology, National Medical Research Center of Children’s Health, Moscow, Russian Federation

###### **Correspondence:** I. Kriulin

**Introduction:** Macrophage activation syndrome (MAS) is a life-threatening complication of systemic juvenile idiopathic arthritis (sJIA), self-diagnosis of which can significantly improve the outcomes of this complication. MAC, like sJIA, can have a continuously recurrent character of disease. Knowledge of predictors of relapse in patients with MAS in anamnesis will allow to suspect MAS in time and start therapy.

**Objectives:** To develop a model for predicting MAS recurrence in patients with systemic juvenile idiopathic arthritis.

**Methods:** Predictors of clinical and laboratory features of 100 patients with sJIA-associated MAS were collected in our study. In the analyzed cohort of 100 patients 114 cases of MAS were registered, 100 primary and 14 repeated. The method of multiple logistic regression analysis was used to predict the occurrence of MAS relapses in patients with sJIA. All clinical and laboratory manifestations of MAS were collected as prospective predictors. As a model of logistic regression, the dependence of the logarithm of the chance of occurrence of the predicted event on a linear combination of predictor variables was used. The interpretation of the logistic regression parameters was performed on the basis of the odds ratio (OR) with a 95% confidence interval. The sensitivity and specificity of the predictors were evaluated using the analysis of ROC curves.

**Results:** For the development of MAS relapse, a statistically significant value was found among the following predictors: lymphadenopathy (OR =0.038; CI 95% 0.003-0.572), decrease in the number of red blood cells (OR =18.915; CI 95% 3.472-103.056), platelets (OR =0.988; CI 95% 0.980-0.995), chloride levels (OR =4,401; 95% CI 1,401-1,823) and increase lactate dehydrogenase activity (OR =0.996; 95% CI 0.992-1,000). The coefficient of determination (R2) for this model was 0.636. The specificity of the model is 98.0%. The total percentage of correct predictions was 95.6%. According to the results of constructing the ROC curve, the AUC index was 0.954±0.027 (95% CI 0.902 – 1,000; p <0.001).

**Conclusion:** According to the results of our study, the parameters for predicting the recurrence of MAS in patients with sJIA can be: lymphadenopathy, decrease in number of red blood cells, platelets, chloride level and increase LDH activity.

**Patient Consent:** Yes, I received consent

**Disclosure of Interest**: None declared

### P197. CD38HIGH/HLA-DR+ CD8+ T cells as diagnostic marker for hlh secondary to visceral leishmaniasis

#### M. V. Mastrolia^1^, S. Boscia^2^, L. Galli^3^, L. Lodi^2^, L. Pisano^2^, I. Maccora^1^, S. Ricci^2^, I. Pagnini^1^, E. Marrani^1^, C. Azzari^2^, G. Simonini^1^

##### ^1^Rheumatology Unit, Meyer Children’s University Hospital, ^2^Immunology and Molecular Microbiology Unit, Meyer Children’s University Hospital , ^3^Pediatric Infectious Diseases Unit, Meyer Children’s University Hospital, Department of Health Sciences, University of Florence, Florence, Italy

###### **Correspondence:** M. V. Mastrolia

**Introduction:** The measurement of the CD8^+^ CD38^high^/HLA-DR^+^ population in children with acute onset of shock and multisystem organ failure represents an important diagnostically useful parameter, to readily distinguish Hemophagocytic lymphohistiocytosis (HLH) from sepsis or healthy controls. A cut-off value > 7% of CD38^high^/HLA-DR^+^ cells among CD8^+^ T cells differentiates HLH from sepsis with a positive predictive value of 96%, and a negative predictive value of 100%. Therefore, this novel biomarker may be helpful in the differential diagnosis of critical patients, for which a timely treatment appears crucial.

**Objectives:** To confirm the role of CD38^high^/HLA-DR^+^ CD8^+^ T cells as precocious HLH diagnostic biomarker

**Methods:** We measured the percentage of CD38^high^/HLA-DR^+^ among CD8^+^ T cells by flow cytometry assay in 4 Italian children suffering from HLH secondary to visceral Leishmaniasis

**Results:** Four patients, all coming from Tuscany, 3 males and one female, mean age of 20.25 months (IQR 24.25), were included. No one reported a recent stay in endemic areas, recent insectbites, contact with wild animals. All of them met the HLH 2004 diagnostic criteria^2^ at hospital admission. Signs of hemophagocytosis were detected on the bone marrow (biopsy performed in 3/4) in all cases. The Leishmaniosis diagnosis was performed by the polymerase chain reaction positivity of the protozoan genome in all subjects: on bone marrow blood in 1 subject; on peripheral blood in the other 3, in 1 out of them subsequently confirmed even on the bone marrow. Serological tests resulted positive IgG and IgM in all patients. Meanwhile infectious tests were ongoing, due to life-threating clinical conditions, immunosuppressive therapy was started to control of the fast-evolving HLH. Steroids were administered in all patients, and immunoglobulin (2g/kg over 12 hours) and anakinra (5 mg/kg/daily) were added in 3.After positive results for Leishmania, liposomal amphotericin B was started according to standard protocol in 3 children with 7 infusions (3 mg/kg/dose) on days 1-5, and after at 14 and 21 days. In one patient,10 doses were required due to massive immunosuppression. All patients promptly recovered and immunomodulatory therapy was weaned and completely stopped in 4 weeks (range 2-6).

CD38^high^/HLA-DR^+^ cells were measured at the disease onset before starting treatment, reporting a mean percentage value of 36.95% (IQR 32%). CD38^high^/HLA-DR^+^ levels were also assessed over the disease course in 3 out 4 children. A decreasing trend has been documented with a complete normalization at 4 weeks (range 3-5) from the HLH onset. However, the reduction up to the normalization resulted slower if compared to the clinical and biochemical HLH parameters normalization, including ferritin.

**Conclusion:** Our results confirm the pivotal function of CD38^high^/HLA-DR^+^ cells as precocious HLH biomarker in the clinical setting of children presenting fever and multisystem organ failure, thus allowing an early initiation of the appropriate immunomodulatory treatment. The curve of the CD38^high^/HLA-DR^+^ cells over the HLH course seems to suggest that this specific CD8+ T cell subset may be a valuable parameter for monitoring the response to treatment. Further prospective studies will be needed to better define the expansion of CD38^high^/HLA-DR^+^ T cells in this group of patients.

**Patient Consent:** Yes, I received consent

**Disclosure of Interest**: None declared

### P198. COVID-19 in pediatric patients with rheumatic diseases and the history of macrophage activation syndrome: retrospective single center study

#### M. Kaleda, I. Nikishina, S. Salugina, E. Fedorov, Z. Kolkhidova

##### Pediatrics, V.A. Nasonova Research Institute of Rheumatology, Moscow, Russian Federation

###### **Correspondence:** I. Nikishina

**Introduction:** It is well known that viral infections may be associated with an increased risk of flare in children with rheumatic diseases (RD), including probability of developing macrophage activation syndrome (MAS) in predisposed patients (pts). The pandemic of novel coronavirus disease 2019 (COVID-19) represents a source of concern for the management of pts with RD and the history of MAS.

**Objectives:** To analyze in a retrospective study the course of COVID-19 in pts with RD and the history of MAS.

**Methods:** The study included all pts with RD and the history of MAS who had COVID-19. The diagnosis of COVID-19 was confirmed either by positive polymerase chain reaction (PCR) or positive of serologic tests for SARS-CoV-2 (immunoglobulin (Ig) G, IgM or both).

**Results:** During the period from March 2020 to April 2022 COVID-19 was verified in 12 pts with RD and the history of MAS (3 males/9 females). 8 pts had systemic juvenile idiopathic arthritis (sJIA), 2 – systemic lupus erythemathosus, 1 – juvenile dermatomyositis, 1 – JIA, polyarthritis. The median age of RD onset was 6.75 y.o. [IQR 5.5; 9.0]. 8 pts had 1 episode of MAS in history, 3 – 2 episodes, 1 – 3 episodes. Total 17 episodes of MAS was verified (11 – at onset, 4 – associating with flare of RD, 1 – after surgery, 1 – accompanied with viral infection H1N1). The median of disease duration at the time of COVID-19 was 6.0 y [5.75; 9.25]. Prior to COVID-19 8 pts received glucocorticoids per os (6 pts - 0-10 mg/day, 2 pts > 10 mg/day), 10 pts received DMARDs (methotrexate - 6, cyclosporine A - 2, mycophenolate mofetil - 1, hydroxychloroquine - 2), 10 pts - biologics (B) (tocilizumab – 6 pts, canakinumab – 2 pts, abatacept – 1 patient, infliximab – 1 patient). All pts received stabile therapy and had an inactive status of RD before COVID-19. 3 pts were asymptomatic COVID-19 (only PCR+, planned before hospitalization or after contact). 7 pts had a mild course of COVID-19: 7 pts had rhinorrhea, fatigue, 4 - anosmia, 3 - abdominal pain, 2 - diarrhea, 1 - headache. They received symptomatic treatment. 2 pts required hospital admission: 16 year-old boy with sJIA (duration of disease – 9 years) and interstitial lung disease (ILD) (initial manifestations verified 18 months before COVID-19) and 12 year-old girl with sJIA (duration of disease – 5 years). Earlier both pts had on 2 episodes of MAS, they received canakinumab in current mode (the boy as a 4th B after secondary inefficiency of tocilizumab, rituximab and anakinra, the girl as a 2nd B after infusion reaction on tocilizumab). Symptoms of COVID-19 in these pts included fever, headache, fatigue, arthralgia. The boy had COVID-associated pneumonia (ground-glass opacities of 45% of lung on CT scan images) with cough, chest pain and dyspnea. The girl had flare of sJIA and Epstein-Barr virus superinfection with persistence of elevated TA (ALT- 577-335 U/l, AST-268-154 U/l) during 2 months after COVID-19. Both pts received COVID-19–related treatments: glucocorticoids IV, azithromycin, canakinumab with reduced interval; acyclovir and intravenous immunoglobulin were also prescribed to the girl. No pts had critical course of disease.

**Conclusion:** In our study the majority of pts had a mild course of COVID-19 infection despite a history of MAS. This may be due to the development of COVID-19 against the background of an inactive status of RD and stable therapy. The small number of pts in our study, however, does not allow us to reliably judge the impact of the history of MAS on the outcomes of COVID-19 and the further course of RD. As understanding of the COVID-19 pandemic appears to change, prospective follow-ups will provide additional data on the long-term impact of COVID-19 on the course of RD in these pts.

**Patient Consent:** Yes, I received consent

**Disclosure of Interest**: None declared

### P199. CD4 DIM CD8 + T cell frequency distinguishes patients with MAS/SHLH from patients with active SJIA and is associated with MAS/SHLH severity

#### G. Prencipe^1^, A. De Matteis^2^, M. Colucci^3^, M. N. Rossi^1^, I. Caiello^1^, P. Merli^4^, N. Tumino^5^, V. Bertaina^4^, M. Pardeo^2^, C. Bracaglia^2^, F. Locatelli^6^, F. De Benedetti^2^

##### ^1^Laboratory of Rheumatology, ^2^Division of Rheumatology, ^3^Renal Diseases Research Unit, ^4^Department of Onco-Haematology and Cell and Gene Therapy, ^5^Department of Immunology, Ospedale Pediatrico Bambino Gesù, ^6^Department of Onco-Haematology and Cell and Gene Therapy, , Ospedale Pediatrico Bambino Gesù; Sapienza, University of Rome, Rome, Italy

###### **Correspondence:** G. Prencipe

**Introduction:** CD8^+^ T-cell activation has been demonstrated to distinguish patients with primary and infection associated hemophagocytic lymphohistiocytosis (pHLH and iaHLH) from patients with early sepsis.

**Objectives:** In this study, we evaluated the activation profile of CD8^+^ T cells in patients with various forms of secondary HLH (sHLH), including macrophage activation syndrome (MAS) complicating systemic juvenile idiopathic arthritis (sJIA).

**Methods:** Flow-cytometry analysis was performed on peripheral blood mononuclear cells (PBMCs) isolated from children with inactive sJIA (n=17), active sJIA (n=27), MAS in sJIA (n=14), iaHLH (n=7) and with other forms of sHLH (n=9). Analyses were also performed in a prospectively enrolled validation cohort including 9 patients with active sJIA and 16 patients with sHLH.

**Results:** PBMCs immunophenotyping revealed the presence of a significantly higher percentage of CD8^+^ T cells co-expressing the CD4 marker (CD4^dim^CD8^+^ T cells) in patients with MAS or with other forms of sHLH, compared to patients with inactive or active sJIA. ROC curve analysis showed that the percentage of CD4^dim^CD8^+^ T cells reliably discriminated patients with all forms of sHLH (AUC=0.92, p<0.001) as well as MAS patients (AUC=0.90, p<0.001) from those with active sJIA. We found that, compared to CD8^+^ T cells not expressing CD4 (CD4^-^CD8^+^ T cells), a markedly higher percentage of CD4^dim^CD8^+^ T cells expressed high levels of CD38 and HLA-DR activation markers. In addition, a significantly higher frequency of CD4^dim^CD8^+^ T cells expressed the activation/exhaustion markers CD25, PD1, and CD95. We also found that the frequency of IFNγ positive cells was significantly higher among CD4^dim^CD8^+^ T than among CD4^-^ CD8^+^ T cells from peripheral blood of MAS patients. Accordingly, peripheral CD4^dim^CD8^+^ T cells cultured *ex vivo* tended to spontaneously release higher amount of IFNγ compared to CD4^-^CD8^+^ T cells. Supporting the pathogenic role of these cells in sHLH, the frequency of CD4^dim^CD8^+^ T cells significantly correlated with most of the laboratory parameters of disease severity, including ferritin, and with circulating levels of IL-18 and CXCL9. We confirmed our results in the validation cohort, by showing that the percentage of CD4^dim^CD8^+^ T cells was significantly higher in patients with MAS/sHLH than in patients with active sJIA (p<0.001) and was able to distinguish sHLH patients from patients with active sJIA (AUC=0.93, p<0.001). In the validation cohort, we also analyzed the TCR Vβ repertoire by using a flow cytometric approach. We did not find expansion of any particular TCR Vβ family in CD3^+^ T cells of patients with MAS/sHLH compared to those of patients with active sJIA, suggesting that in sHLH CD8^+^ T cell activation is antigen-independent. Finally, we found that the frequency of CD4^dim^CD8^+^ T cells was significantly associated with the clinical severity score (r=0.56, p<0.0001), based on endpoints including death, length of hospitalization, stay in intensive care unit and dose of glucocorticoids.

**Conclusion:** Altogether, our data showing that CD4^dim^CD8^+^ T cells are increased in patients with MAS/sHLH and associated with disease severity strongly support their involvement in MAS/sHLH pathogenesis. They also suggest a potential prognostic relevance of the assessment of CD4^dim^CD8^+^ T cells in HLH/MAS syndrome and support the rationale for novel therapeutic strategies targeting activated CD8+ T cells.

**Disclosure of Interest**: None declared

### P200. A strange case of hyperferritin: from management to treatment

#### M. T. Riccio^1^, A. Catzola^1^, M. Alessio^1^, R. naddei^2^

##### ^1^Department of Translational Medicine, Section of Pediatrics, University of Naples Federico II, Naples, ^2^Department of Translational Medicine, Section of Pediatrics, University of Naples Federico II, napoli, Italy

###### **Correspondence:** M. T. Riccio

**Introduction:** Macrophage Activation Syndrome (MAS) is a severe hyperinflammatory condition, belonging to the spectrum of Haemophagocytic Lymphohistiocytosis, whose secondary forms occur in the context of pre-existing disorders such as infectious, rheumatic and oncological diseases. It may clinically manifest as fever, cytopenia, hypertriglyceridemia, hyperferritinemia, reduced fibrinogen, coagulopathy due to self-activation of T-cells, macrophages and overproduction of pro-inflammatory cytokines. MAS can have a fatal course, therefore an early diagnosis is necessary for rapid therapeutic intervention, including immunosuppressive and anti-inflammatory drugs such as corticosteroids, cyclosporine and biological drugs.

**Objectives:** the aim is to demonstrate how HLH can be secondary to lymphomas and how anakinra is crucial in treatment.

**Methods:** Child, 12 years old, severely obese (BMI 41), hospitalized in another structure for fever, joint pain, lymphadenopathy, where was suspected MAS secondary to Systemic Juvenile Idiopathic Arthritis (sAIG) and started therapy with methylprednisolone EV and cyclosporine. He is therefore transferred to our department for treatment and follow-up. The presence of fever, laterocervical lymphadenomegaly, skin rash in association with thrombocytopenia, hyperferritinemia, hypertriglyceridemia, increased LDH and hypofibrinogenemia confirmed the suspicion of MAS, but the presence of lymphadenomegaly and pruritus that did not respond to antihistamine treatment put the diagnosis in doubt. So it was decided to perform echography of the lymph nodes, histological examination if necessary, and serial checks of ferritin values and other laboratory indices.

**Results:** Ultrasonography of the lymph nodes and abdomen revealed hepatosplenomegaly and diffuse lymphadenomegaly (dmax 43x21 mm), and consequently the right supraclavicular lymph node was biopsied. The clinical and laboratory profile showed a rapidly worsening evolution despite the early start of treatment: ferritin values progressively increased (>80,000 ng/ml), plurilinear cytopenia, hypofibrinogenemia (103mg/dl) and increased LDH (7720U/L), transaminases and bilirubin. Subcutaneous therapy with Anakinra 4 mg/Kg was therefore started first, then, due to lack of clinical-laboratory improvement, intravenous at a dose of 8 mg/kg. The shift in the treatment method led to an amelioration of the clinical and laboratory parameters. The histological examination, arrived during the phase of clinical and laboratory improvement, established the diagnosis of anaplastic T-cell lymphoma.

**Conclusion:** The case report highlights aspects related to the management and treatment of MAS and its aetiology. Although MAS is frequently a complication of sAIG, it can be associated with other diseases, including malignancies, which should always be considered in the differential diagnosis. Anaplastic T-cell lymphoma, although rare, should be suspected as it may begin with MAS. Another consideration arises from the lack of response to therapy with Anakinra subcutaneously: the brilliant response to the intravenous shift may be due to severe obesity, which has reduced the efficacy of the drug by reducing its bioavailability.

**Disclosure of Interest**: None declared

### P201. Idiopathic systemic hyperinflammation, a formidable diagnostic challenge

#### R. Stander, C. Scott, T. De Wit

##### Paeditaric rheumatology, university of cape town, cape town, South Africa

###### **Correspondence:** R. Stander

**Introduction:** Here we describe a hyperinflammatory syndrome presenting as recurrent episodes of inflammation with a homozygous PRF1 mutation suggestive of a familial hemophagocytic lymphohistiocytosis (HLH).

**Objectives:** A 5 year old previously well female presented with a history of intractable fever, rash, abdominal pain and arthralgia. Clinically she had lymphadenopathy and liver enlargement but no other supporting clinical features of systemic JIA, MIS-C or acute rheumatic fever. Laboratory features were suggestive of inflammation with striking hyperferritinemia, persistently raised CRP and raised d-dimers. She had mild anaemia and a bone marrow that was normocellular and showed no features of MAS.

**Methods:** Sepsis and disseminated TB were excluded, her COVID PCR and COVID-Ab were negative. Initial therapy included intravenous immunoglobulins (IVIG), 10mg/kg of IV methylprednisolone for 3 days and IV antibiotics. Her temperature spikes persisted and cyclosporin was commenced to which she responded and was discharged home with a working diagnosis of idiopathic hyperinflammation. Over the next 3 months she had patchy recurrent episodes of inflammation which responded to non-specific immune suppression (a second pulse of IVMP with maintenance prednisone and cyclosporin). She was initiated on an IL-6 inhibitor Tocilizumab for ‘presumed’ systemic JIA. She followed up in rheumatology clinic one month later with severe back pain; and MRI showed a wedge collapse of T5 vertebra and a subligamentous collection; Staph Aureas and TB treatment were commenced in view of the immunosuppression and radiological findings.

**Results:** During this admission she deteriorated; experiencing another hyperinflammatory episode with refractory shock and cardiac arrests; which responded to Anakinra. She developed a dense vitritis of the left eye and purpura fulminans with auto-amputation of her finger digits and toes. She remains symptom free on Tocilizumab and cyclosporin.

**Conclusion:** The genetic panel was unable to identify pathogenic variants in known HLH genes. But here we describe a patient with an immune dysregulation syndrome and uncontrolled inflammation resembling familial HLH, but which may form part of an extended syndrome with a subacute onset.

**Patient Consent:** Yes, I received consent

**Disclosure of Interest**: None declared

### P202. A case of macrophage activation syndrome associated with juvenile systemic lupus erythematosus in a 7-year old girl

#### T. Vasilev, M. Ganeva, K. Temelkova, A. Telcharova-Mihaylovska, V. Kostova, A. Dasheva, D. Hristova, S. Stefanov

##### Department of Pediatric Rheumatology and Cardiology, Medical University of Sofia, Sofia, Bulgaria

###### **Correspondence:** T. Vasilev

**Introduction:** Macrophage activation syndrome (MAS) is a potentially life-threatening complication of rheumatic diseases, including juvenile systemic lupus erythematosus (jSLE). The incidence of MAS in jSLE ranges from 5.5 to 9 %.(1)

**Objectives:** The aim of this clinical case report is to describe the clinical and laboratory features of MAS which occurred at the time of diagnosis of jSLE.

**Methods:** We report the case of a child who presented with MAS at the onset of highly active SLE and who developed steroid-induced diabetes, which required adjustment in therapy.

**Results:** We present a 7-year old girl with unremarkable history, who was admitted due to photosensitive skin lesions over the body. In the last two months the child had lost weight. Fever and painful ulcerative lesions over the oral cavity and hard palate appeared, as well. On admission the child was ill appearing, with erythematous-annular and papular lesions over the face, body and limbs. Erosions of varying depth over the hard palate and crusts on the lower lips were observed. Generalized lymphadenopathy and enlarged liver of 4 cm below the rib margin were detected. Laboratory tests revealed cytopenia - anemia Hgb - 89.0 g/L with positive direct Coombs test and thrombocytopenia Plt - 72.0 x10^9/L; impaired renal function - GFR 79.9 ml/min/1.73; low complement levels - C3 0.25 g/L and C4 < 0.020 g/L; positive ANA 1:640, anti-ds DNA 191 U/ml and anti SS-A > 200 antibodies. The diagnosis of jSLE was made based on fulfilling 5 clinical (acute cutaneous lupus, oral ulcers, renal involvement, hemolytic anemia, thrombocytopenia) and 4 immunological Systemic Lupus International Collaborating Clinics (SLICC)-12 criteria.(2)

Furthermore, fever and elevated liver enzymes were noticed - AST - 676 U/L and ALT - 91 U/L with elevated triglycerides 170.15 mg/dL, hypofibrinogenemia 162 mg/dL and hyperferritinemia 4113.1 ng/mL. The child fulfilled 2016 Classification criteria for MAS complicating sJIA applicable to jSLE.(3) No infectious triggers for MAS were detected - negative serology for EBV; CMV; HSV; SARS-CoV-2. Pulse therapy with methylprednisolone (15 mg /kg /day) for 3 consecutive days was initiated, followed by 2 mg /kg /day. Moreover, seizures were observed with blood pressure up to 200/120 mmHg. Brain MRI revealed normal image. Anticonvulsant and antihypertensive therapy was started. The child was transferred to ICU. During follow up steroid-induced diabetes was diagnosed. Insulin therapy was started and discontinued on steroid tapering. Cyclosporine A was added to the treatment. After a 40-day stay at the Hospital, the girl was discharged.

**Conclusion:** The presented clinical case shows the difficulties faced by the clinicians when it comes to managing life-threatening complications of a serious disease such as jSLE.

**References:**

1. Sato S, Uejima Y, Arakawa Y, et al. Rheumatol Adv Pract. 2019 May 14;3(1).

2. Petri M, Orbai AM, Alarcуn GS, et al. Arthritis Rheum. 2012;64:2677–86.

3. Ravelli A, Minoia F, Davı S, et al. Arthritis Rheumatol. 2016 Mar;68(3):566-76.

**Patient Consent:** Yes, I received consent

**Disclosure of Interest**: None declared

## Poster session: Imaging

### P203. Musculoskeletal ultrasound in juvenile dermatomyositis – a proposal for a stratified evaluation method

#### M. Krumrey-Langkammerer^1^, S. Bechthold^2^, T. Fröhlich^3^, B. Hügle^1^, A. Jansson^2^, R. Jones^4^, M. J. Laaths^5^, T. Lutz^6^, C. Schütz^7^, J.-P. Haas^8^

##### ^1^German Centre for Pediatric and Adolenscent Rheumatology, Garmisch-Partenkirchen, ^2^Dr. von Hauner´sches Kinderspital, Ludwig-Maximillians University, Munich, ^3^Practice for Pediatric Rheumatology, Forchheim, Germany, ^4^Pediatric rheumatology, Landeskrankenhaus, Salzburg, Austria, ^5^Pediatric rheumatology, Children´s hospital, Amberg, ^6^Practice for Pediatric Rheumatology, Heidelberg, ^7^Pediatric Immunology and Rheumatology, Gustav-Carus University , Dresden, ^8^German Centre for Pediatric and Adolescents Rheumatology , Garmisch-Partenkirchen, Germany

###### **Correspondence:** M. Krumrey-Langkammerer

**Introduction:** Juvenile dermatomyositis (JDM) is a chronic autoimmune disease in childhood that presents with vasculitis and inflammation of the muscles causing significant muscular damage if untreated. So far, the gold-standard for assessing disease activity and damage is magnetic resonance imaging. However, muskuloskeletal ultrasound (MSUS) is inceasingly used to assess inflammation in JDM.

**Objectives:** To develop a scoring system to standardize evaluation of the observed changes in muscle patterns seen in MSUS.

**Methods:** A single-center cross-sectional study included a cohort of 15 JDM patients examined between November 2021 and February 2022. We used a standardized procedure for MSUS (Canon Aplio i800®, linear probe 14MHz) in six different muscles. All MSUS and the interpretation of data were performed by two pediatric rheumatologist experienced in muskuloskeletal ultrasound, using a proprietary evaluation scheme designed for the interpretation of inflammatory myositis (see table 1). Pearson’s correlation was used to correlate MSUS scores with different markers of activity and damage in JDM including CMAS, MMT-8, DM-DAS (dermatomyositis disease activity score) and MDI (myositis damage index).

**Results:** 15 JDM patients (9f/6m) have been included. Patients were 10,95 years (4,69-18,52) old at the time of examination with a mean diseaseduration of 6,11 yrs. (0,82-13,45). Mean values were: CMAS: 42,4; MMT-8: 68,91; CHAQ: 1,1; Phy-Global: 2,27; Pat/Par-Global 2,4; CK 531 U/l. Five exhibited a monocyclic, 7 a chronic-active and 3 a polycyclic course of JDM. Myositis specific antibodies had been detected in 10 patients (8xNXP-2, 1xMDA5, 1xTIFF-1g). The proposed evaluation score was found to have the highest correlation with the DAS (p=0,64). Increasing inhomogenity, thickening of fascia and hypervascularisation of muscle and fascia were found to indicate inflammatory disease activity. Chronic perimysial inflammation and necrotic myofibrils were represented by spotted or focally altered echogenity.

**Conclusion:** This proposal of a scoring-system represents a first attempt at interpretation of MSUS in JDM. It should help to define disease activity and assess damage in JDM patients. Further prospective studies are required in order to adjust this score.

**Patient Consent:** Yes, I received consent

**Disclosure of Interest**: None declared

**Table 1 (abstract P203).** Evaluation scheme for the interpretation and scoring of MSUS in JDM and data from the cohort

	remarkable findings (0-1) 0 = no 1 = yes	Texture (0-2) 0 = homogeneous 1 = inhomogeneous 2 = polymorph	Echogenity (0-2) 0 = black >> white 1 = increased 2 = white > black	Structure (0-2) 0 = feathery intact muscle fibers 1 = reticular speckled, spotted 2 = lamellary, focal streaky	Perfusion Fascia and intramuscular (0-1) 0 = not increased 1 = increased	Fascia (0-2) 0 = unremarkable 1 = thickend/stapled 2 = destroyed structure	Calcinosis (0-1) 0 = no 1 = intramuscular/epifascial	Total (max. 66)
Mean	5,0	6,0	5,7	7,3	1,3	3,2	1,2	**29,7**
Max	6,0	12,0	12,0	12,0	6,0	6,0	6,0	**58,0**
Min	2,0	2,0	0,0	0,0	0,0	0,0	0,0	**4,0**
Median	6,0	6,0	5,0	8,0	0,0	4,0	0,0	**32,0**
Stan of mean	1,3	2,9	3,0	3,1	2,0	2,0	2,0	**13,1**

### P204. Internal consistency and interrater reliability in musculoskeletal ultrasound in children

#### D. S. Lazarevic^1,2^, J. Vojinovic^1,2^, C. Malattia^3^, L. Rossi-Semerano^4^, B. Sozeri^5^, M. Tsinti^6^, S. Lanni^7^, C. Host^8^, D. Windschall^9^, A. Snipaitiene^10^

##### ^1^Pediatric Rheumatology and Immunology, Clinic of Pediatrics, University Clinical Center , ^2^Pediatrics, Faculty of Medicine, University of Nis, Nis, Serbia, Nis, Serbia, ^3^IRCCS Instituto Giannina Gaslini, Genoa, Italy, ^4^Department of Pediatric Rheumatology, CEREMAIA, Bicêtre Hospital, AP-HP, Le Kremlin Bicêtre, France, ^5^Health Sciences University, Umraniye Education and Research Hospital, Istanbul, Turkey, ^6^Aghia Sophia Children’s Hospital, Athens, Greece, ^7^Fondazione IRCCS Cà Granda Ospedale Maggiore Policlinico, Milan, Italy, ^8^Aarhus University, Aarhus, Denmark, ^9^University of Halle-Wittenberg and St. Josef-Stift Sendenhorst, Sendenhorst, Germany, ^10^Hospital of Lithuanian University of Health Sciences Kauno Klinikos, Kaunas, Lithuania

###### **Correspondence:** D. S. Lazarevic

**Introduction:** Numerous publications provided rational for involvement of musculoskeletal ultrasound (MSUS) and several inflammatory biomarkers as the clinically meaningful prediction panel for juvenile idiopathic arthritis (JIA). This could help to achieve personalized treatment and define new sensitive outcome tool important for evaluation of disease activity and treatment response.

**Objectives:** To examine internal consistency and interrater reliability of pediatric MSUS in JIA patients evaluated by experts in this field.

**Methods:** DAISY study is a multicentric longitudinal study that will recruit 150 JIA patients (ILAR classification criteria) with active disease (JADAS 10 and 27) prior starting recommended treatment. At enrollment and during predefined follow up visits (3, 6, 12 months) patients are evaluated by JADAS 10 and 27, examined by gray-scale (GS) and power-doppler (PD) in 44 joints using OMERACT synovitis scoring by an expert in pediatric MSUS, while blood samples are collected for evaluation of inflammatory markers (cytokines, chemokines, S100A8, S100A9 and S100A12). To be eligible pediatric ultrasound experts must have at least intermediate EULAR certified MSUS course and at least 5 years of experience in MSUS in children.

Study is conducted in 9 participating centers in 8 different countries after Ethics Committee Approvals and Informed Consents were obtained. Prior to the project start up all ultrasonographers accessed the study educational material on DAISY web-portal (US normal and pathological images with standardized scanning from 4 different age categories in children). Once reviewed the educational material, 9 ultrasonographers performed “test for ultrasonographers” which represents calibration web-based reliability exercise on 56 MSUS still images of different aged categories with different GS and PD grades of synovitis using OMERACT synovitis scoring (to optimize interrater reliability). To pass the test, ultrasonographers must have more than 80 percent’s of right answers.

We assessed the internal consistency reliability with Cronbach’s alpha. The inter-observer interclass correlation coefficient (ICCs) was calculated to estimate agreement between nine raters. ICCs were calculated and tested with a significance level of 5%. Cronbach’s alpha> 0.9 and ICC ≥0.8 were considered excellent.

**Results:** Cronbach alpha was very high for all joints and reflects good internal consistency (α>0.9). The interrater ICCs for all joints (elbow, wrist, MCP II, hip, knee, tibiotalar, talonavicular, subtalar, MTP) was high with confidence interval 95%. The observed values for inter-observer analysis showed an excellent (ICC ≥ 0.80) agreement, with the strongest one on the hip (ICC =1), and a little bit lower, but still excellent on tibiotalar (ICC=0.828) and subtalar joint (ICC=0.861).

**Conclusion:** The internal consistency reliability and interobserver reliability were excellent, pointing out that all ultrasonographers were highly reliable in grading paediatric musculoskeletal ultrasound.

**Trial registration identifying number: Funding:** This study is supported by the Foundation for Research in Rheumatology (FOREUM).

**Disclosure of Interest**: None declared

### P205. Qualitative and semiquantitative characterization of videocapillaroscopy evaluation of pediatric patients with perniosis

#### A. K. Leos Leija, R. C. Calderón Zamora, A. V. Villarreal Treviño, F. García Rodríguez, N. Rubio Pérez

##### Paediatric Rheumatology , Hospital Universitario “Dr. José Eleuterio González”, Monterrey, Mexico

###### **Correspondence:** A. V. Villarreal Treviño

**Introduction:** Perniosis is a disease caused by exposure to damp cold, which particularly affects unprotected areas of the skin. Although the exact cause of the disease is unknown, it may be idiopathic or secondary to other systemic diseases such as systemic lupus erythematosus (SLE). Nailfold videocapillaroscopy (NVC) is an important tool for patients with rheumatic diseases, evaluating morphological changes of the nailfold capillaries, such as capillary density, avascularity and abnormal capillaries. During the fourth wave of COVID19 pandemic we observed an increase of cases of perniosis in previously healthy pediatric patients, so capillaroscopic evaluation was performed.

**Objectives:** To describe qualitative and semiquantitative capillaroscopic findings in pediatric patients with perniosis.

**Methods:** Eight fingers, except the thumb, of 10 patients from a pediatric rheumatology department of a tertiary center in Monterrey, Nuevo León, México from December 2021 to March 2022 were included. There were analyzed the most representative 1 mm images of each one with x200 videocapillaroscope (Optilia), analyzing a total of 80 images by the same observer. Demographic data such as sex and age were included and capillaroscopic qualitative data such as pattern, density, abnormal shapes, microbleeding and edema, and semiquantitative characteristics such as length and width were obtained. Autoantibodies for the evaluation of connective tissue diseases were obtained for each patient.

**Results:** Ten patients were included of which 8 (80%) were female, the mean age was 13 years. Perniosis was present in 100% of patients. Antinuclear antibodies (ANA) were present in 3 patients (30%) of which 2 (20%) had DFS70 pattern and 1 (10%) had nuclear fine speckled pattern. The rest of autoantibodies were negative in all patients. Among the semiquantitative characteristics, we found no capillaroscopic changes.

Right hand finger No. 2 to No. 5 =R2, R3, R4, R5. Left hand finger No.2 to No. 5 = L2, L3, L4, L5.

**Conclusion:** This study shows the semiquantitative data of the NVC analysis, and therefore it is proposed as a possible tool for the diagnosis and monitoring of perniosis, not only in patients with rheumatic diseases, but also in those previously healthy individuals with no clinical signs or symptoms of an autoimmune disease. Despite being a study with a small sample of patients, there is currently a lack of literature in pediatric patients where these characteristics are evaluated in individuals with perniosis, so it is recommended to carry out studies with a greater sample to obtain more data.

**Patient Consent:** Not applicable (there are no patient data)

**Disclosure of Interest**: None declared

**Table 1 (abstract P205).** See text for description

	R2	R3	R4	R5	L2	L3	L4	L5
**Length (μm)**	342.85	278.45	291.28	310.66	307.79	298.2	290.09	221.73
**Width (μm)**	18.27	17.53	18.23	18.01	20.35	15.55	16.52	17.76

### P206. Comparison of coronary artery diameters on CT coronary angiography with 2D-Echocardiography in children with Kawasaki disease: a single center experience from Chandigarh, North India

#### R. K. Pilania^1^, M. Singhal^2^, H. Chaudhary ^1^, A. Sil^1^, A. K. Jindal^1^, P. Vignesh ^1^, S. Singh^1^

##### ^1^Pediatric Allergy Immunology Unit , ^2^Department of Radiodiagnosis and Imaging , PGIMER, Chandigarh, India

###### **Correspondence:** R. K. Pilania

**Introduction:** 2D echocardiography (2DE) has hitherto been standard of care for evaluation of coronary artery abnormalities (CAAs) in children with Kawasaki disease (KD). CT coronary angiography (CTCA) is an emerging imaging modality in this field.

**Objectives:** To compare coronary artery dimensions on CTCA with 2DE in children with KD.

**Methods:** Records of children with KD were retrieved wherein both CTCA and 2DE had been carried out within 48 hours of 2DE. Comparison of proximal coronary artery dimensions on both modalities was made. 2DE was carried by clinical fellows experienced in coronary artery assessment, while CTCA were performed by an experienced cardiac radiologist blinded to findings of 2DE. Statistical analysis was done on SPSS software version 23 and paired t-test was used to compare mean diameters recorded on two imaging modalities.

**Results:** There were 61 children with KD who fulfilled the inclusion criteria. Twenty-four children were imaged at presentation, while 37 were imaged during convalescence/ follow-up. There was a significant difference in left circumflex artery (LCx) measurements between CTCA and 2DE (t=2.23, p=0.04). On an average, LCx measurements on CTCA were 1.06 mm higher than on ECHO (95% CI [0.04, 2.09]). There was no significant difference between left main coronary artery (LMCA) dimensions measured through 2DE and CTCA (p=0.534). Similarly, no significant difference was noted between left anterior descending (LAD) and right coronary artery (RCA) dimensions measured through 2DE and CTCA (p=0.675 and p=0.198 respectively).

**Conclusion:** Dimensions of coronary arteries derived from CTCA in proximal segments of LMCA, RCA and LAD were comparable with 2DE, while LCx dimensions were significantly higher on CTCA. This may be due to difficulties in assessment of LCx on 2DE. CTCA thus has potential to become standard of care for diagnostic evaluation children with KD.

**Trial registration identifying number:** Not applicable

**Patient Consent:** Yes, I received consent

**Disclosure of Interest**: None declared

### P207. High-frequency ultrasound and clinical correlation of disease activity in juvenile localized scleroderma: preliminary data

#### D. G. P. Piotto, R. L. F. D. Andrade, G. Clemente, M. M. Fraga, C. A. Len, M. T. R. Terreri

##### Pediatrics, Universidade Federal de São Paulo, São Paulo, Brazil

###### **Correspondence:** D. G. P. Piotto

**Introduction:** Juvenile localized scleroderma (JLS) is a rare pediatric disease characterized by inflammation and skin thickening. High-frequency ultrasound (HF-US) shows great promise in helping clinical assessment of the disease activity and fibrosis in JLS.

**Objectives:** The aim of this study was to evaluate HF-US findings in JLS patients and to correlate clinical and ultrasound activity.

**Methods:** The data collected included presence of symptoms, disease features, and the Localized Scleroderma Cutaneous Assessment Tool (LoSCAT), the modified Localized Scleroderma Skin Severity Index (mLoSSI), the Localized Scleroderma Skin Damage Index (LoSDI), activity and damage Patient Global Assessment of Disease (PGAD). All US images were obtained using a 22 MHz HF-US (GE machine). The clinical and US data were blindly evaluated by 2 examiners specialized in Paediatric Rheumatology. The dermal thickness, echogenicity score and the presence of Power Doppler of the JLS lesional dermis were measured in each lesion with the highest score for erythema, skin thickness, and dermal atrophy. Measurements were compared to contralateral side of unaffected skin.

**Results:** A total of 22 JLS were included, 68.2% female with a mean age of 13.6 years (SD ± 4.6 years). Mean age at symptoms onset was 6.6 years (SD ± 3.3 years), mean age at diagnosis 7.5 years (SD ± 3.2 years) and disease follow up time of 7.1 years (SD ± 4.7 years). The linear form was the most prevalent (68.2%), followed by the linear + morphea association (18.2%) and isolated morphea plaques (13.6%). Regarding the activity and damage PGAD of lesions, the means were 1.8 (SD ± 1.7) and 1.1 (SD ± 1.5) respectively. The mean Rodnan score was 1.5 (SD ± 2.1) and LoSCAT 14.6 (SD ± 9.7). We studied 77 images of HF-US and we could observe a reduction in the thickness of the dermal layer and subcutaneous tissue, with greater echogenicity of the dermis compared to the skin of the contralateral unaffected skin in those patients with longer duration of the disease. Inflammatory (erythematous) lesions presented increase in the dermal thickness and decrease in the echogenicity, while fibrosis was characterized by hyperechogenicity and increased (sclerotic lesions) or decreased (atrophic lesions) dermal thickness, compared with healthy skin. Doppler US did not reveal increased vascularization of the lesion in these patients. As the study has not yet been completed, a statistical analysis was not possible.

**Conclusion:** Our findings suggest that 22 MHz HF-US allows quantitative evaluation of the JLS lesions in different stages. HF-US morphological evaluation provides a reliable and more comprehensive measurement of the JLS lesions.

**Patient Consent:** Yes, I received consent

**Disclosure of Interest**: None declared

### P208. Ultrasound and muscle elastography could be a promising tool to assess disease activity in juvenile dermatomyositis patients?

#### R. L. F. de Andrade^1^, J. A. Mendonça^2^, D. G. P. Piotto^1^, P. P. Aires^1^, A. M. D. O. Rocha^1^, M. T. R. Terreri^1^

##### ^1^Pediatric, Universidade Federal de São Paulo, São Paulo, ^2^Rheumatology, UNICAMP, Campinas, Brazil

###### **Correspondence:** D. G. P. Piotto

**Introduction:** Juvenile Dermatomyositis (JDM) is the most common idiopathic inflammatory myopathy (IIM) in the pediatric age group. For diagnosis, we often need invasive tests that are difficult to be accepted by children. In addition, it is hard to define whether the disease is active or only sequelae lesions are present, which is important in determining the therapy. The introduction of new imaging exams has allowed a better assessment of muscle involvement in JDM, with ultrasound (US) being a promising method in the pediatric age group. US proved to be useful in helping to define the degree of disease activity and also in patient follow-up, demonstrating the severity and tissue damage associated with the myopathy. Regarding muscle elastography, that is a technique more recently incorporated into US, studies are scarce and have controversial results. In IIM, elastography seems to show an increase in muscle stiffness compatible with that found in MRI. More particularly in the pediatric age group, elastography was not satisfactory for detecting active myositis, although it showed a correlation between the elastographic abnormalities with the increase in muscle echogenicity found in MRI.

**Objectives:** This study aimed to establish an association of disease activity through clinical muscle assessment (through validated instruments), laboratory and nailfold capillaroscopy (NFC) with quantitative and semiquantitative ultrasound and elastography through the Strain Elastography (SE) technique.

**Methods:** Twenty-two JDM-patients and fourteen healthy controls, aged between 5 and 21 years, matched for age and sex were enrolled. Patients underwent clinical evaluation through the application of muscle assessment questionnaires (CMAS and MMT), global assessment of the disease through the application of a questionnaire (DAS) and visual analogue scale (VAS) scores by the physician and parents and patients and assessment of functional capacity through the CHAQ questionnaire. Patients were also submitted to NFC and measurement of muscle enzymes, as recommended in follow-up. All subjects in the study underwent US assessment using gray scale, application of Power Doppler and application of SE.

**Results:** In the categorical and semi-quantitative gray scale evaluation of patients and controls, we observed a higher frequency of alterations in the patients’ exams, with the categorical gray scale being more altered in the quadriceps femoris (p=0.029) and the semi-quantitative gray scale presenting higher frequency of alterations in all evaluated muscle groups (p<0.001). When comparing the US assessment of patients with disease activity parameters (CK, MMT, CMAS and VAS) no robust correlations were found. From the gray scale, we proposed a cut-off point for elastography, from which we can consider the muscle with pathological stiffness. This cut-off point was 54.36, with 45.3% of sensitivity, 64.4% of specitivity, 18% of positive predictive value, 87.2% of negative predictive value and 61.6% of accuracy.

**Conclusion:** USG was able, through semiquantitative grayscale evaluation, to differentiate patients with JDM from healthy patients, however there was no correlation between ultrasound, grayscale, PD and elastography findings and disease activity markers.

**Patient Consent:** Yes, I received consent

**Disclosure of Interest**: None declared

### P210. Phalangeal microgeodic syndrome. A case report

#### O. Vougiouka^1^, I. Nikas^2^

##### ^1^2nd dep. of Paediatrics Athens University School of Medicine, P A Kyriakou Hospital, ^2^Pediatr Diagnostic Center, Bioiatriki Healthcare Group, ATHENS, Greece

###### **Correspondence:** O. Vougiouka

**Introduction:** Phalangeal Microgeodic Syndrome (PMS) is a rare syndrome affecting finger phalanges, mostly accounted in children, first described in 1970 by Maroteaux P.

**Objectives:** The presentation of an 11year old girl with finger oedema, purple skin discoloration, mild stiffness and pruritus manifested in early March this year.

**Methods:** The girl had already visited her pediatrician and an orthopedic surgeon who recommended a right hand MRI. Immunology profile was prescribed.

**Results:** There were multiple areas of bone marrow oedema on the whole finger phalanges on presented MRI compatible to PMS. Anti-dsDNA, ENA, RNP were all negative. Inflammatory indexes, blood cultures, quantiferon test, complement, TSH and PTH were normal. The girl, a rhythmic gymnastics’ athlete, was double COVID-19 vaccinated in January this year. COVID-19 or known contact were not reported. A frostbite condition diagnosed and conservative measures suggested.

**Conclusion:** Radiographic features of PMS, as first described, show mild osteosclerosis with cortical irregularity and multiple small radiolucent spots of osteolysis in the diaphysis. Recently, MRI is helpful for the diagnosis. In PMS, the bone marrow of the phalanges shows diffuse low signal intensity on T1-weighted images and high signal intensity on STIR images which indicates bone oedema. Frostbites attributed to cold exposure, chilblains because of COVID-19 and Raynaud’s phenomenon are conditions with microgeodic findings on imaging, which we should be aware of in order to avoid extented investigations and reassure parents and patients.

**Trial registration identifying number:** References: Maroteaux P. A microgeodic disease of unknown etiology affecting the finger phalanges in infants: report of five cases. Ann Radiol (Paris) 1970;13:229–36.

Nishino et al. Two rare cases of adult-onset phalangeal microgeodic syndrome with magnetic resonance imaging-proven bone edema transiently occurring in winter. Joint Bone Spine 80 (2013) 523–524.

Ramani S L et al. Musculoskeletal involvement of COVID-19: review of imaging. Skeletal Radiology 2021 Sep;50(9):1763-1773.

**Patient Consent:** Yes, I received consent

**Disclosure of Interest**: None declared

## Poster session: Disease outcome and transition

### P211. Rheumatology transition of young people in Switzerland – the heroes study

#### L. Berben^1^, M.-L. Daly^1^, T. Daikeler^2^, A. Wörner^1^

##### ^1^University Children’s Hospital Basel, ^2^University Hospital Basel, Basel, Switzerland

###### **Correspondence:** L. Berben

**Introduction:** About half of all children with rheumatic diseases need continuous medical care during adolescence and adulthood. An effective and well-planned transition built on individual, structured management plans is crucial for successful transition into adult services. Although transition principles have been described and implemented, outcome data are still scarce.

**Objectives:** The overall objective of the HEROES study is to develop, implement and evaluate a transitional care (TC) program for adolescents and young adults (AYA) with a rheumatic disease moving from pediatric to adult settings in Switzerland.

Specific aims are:

1. assess AYAs’ and parents’ experiences and unmet needs related to current TC practices
2. assess stakeholder, healthcare professional, setting and system barriers and facilitators to implementing a TC program
3. develop and implement a TC program that includes a nurse TC coordinator
4. evaluate the effectiveness of the TC program in relation to disease-related outcomes
5. evaluate AYA-, parent- and healthcare professional-reported outcomes and care experiences in relation to the TC program
6. evaluate the implementation outcomes of the TC program in relation to adoption, implementation and sustainability
7. evaluate economic outcomes of the TC program

**Methods:** This study which includes all 10 Swiss pediatric rheumatology centers and their adult counterpart, will use a hybrid effectiveness-implementation type 2 design. For the different parts of the study, specific designs will vary.

For the qualitative data (i.e. AYA’ and parents’ experiences and unmet needs, acceptance and appropriateness of the intervention and assessment of contextual factors), an *explanatory sequential mixed method* design will be used.

The quantitative data, i.e. the intervention-effectiveness outcomes, in this study will be assessed using a *multi-center quasi experimental pre-post design*. To maximize the acceptability and sustainability, a participatory partnership approach will be used, i.e. people that the intervention aims to help and those who will implement it are involved throughout the process. Intervention outcomes will be measured at the AYA (e.g. quality-of-life, treatment adherence), parent (e.g. care satisfaction, giving responsibility for care to the AYA), healthcare professional (e.g. time for AYA, work satisfaction), setting (e.g. billing of consultations) and system (e.g. costs) levels.

**Results:** The (poster) presentation will describe the study methodology.

**Conclusion:** This innovation has a potential to improve TC for children with rheumatic diseases as well as serving as a model for TC in other chronic diseases.

**Patient Consent:** Not applicable (there are no patient data)

**Disclosure of Interest**: None declared

### P212. Functional state of the pulmonary system in children with juvenile idiopathic arthritis according to dynamic monitoring

#### N. S. Shevchenko^1,2^, T. Holovko^1,2^, L. Bohmat^1,2^, V. Nikonova^1^

##### ^1^Department of rheumatology and comorbid states, SI “Institute for Children and Adolescents Health Care of the NAMS of Ukraine”, ^2^Department of pediatrics № 2, V.N.Karazin Kharkiv national university, Kharkiv, Ukraine

###### **Correspondence:** T. Holovko

**Introduction:** Pulmonary manifestations of rheumatic diseases in children are uncommon. At the same time, there is a high risk of involvement of the respiratory organs in the pathological process, which manifests itself mainly as interstitial lung disease and pulmonary arterial hypertension. These pathological conditions can occur both as a result of the underlying disease and as a result of drug toxicity of basic therapy. In the future, they may be the cause of worsening the prognosis and increasing disability and mortality.

**Objectives:** For timely diagnosis of pulmonary complications, lung ventilation function was assessed in children with juvenile idiopathic arthritis.

**Methods:** The study included 38 children aged 7 to 18 years, 12 boys and 26 girls with oligoarticular and polyarticular variants of juvenile idiopathic arthritis. Spirometry was performed to determine the indicators of external respiration twice, with an interval of 12 months.

**Results:** It was found that in a quarter of patients (26.31 ± 2.80%) there was a decrease in the function of external respiration. In all cases, the violations were restrictive in nature, mild. The frequency of disorders did not have a significant relationship with gender and subtypes of the disease. All patients with respiratory disorders had the disease for more than 3 years, 80.0% of them had ANA-positivity, which persisted for a long time and testified to the immunological activity of the process. Patients received methotrexate (15-20 mg / week), the duration of therapy was at least 2.5 ± 0.30 years. Re-examination after 12 months revealed a decrease in reduced pulmonary ventilation function in 23.74 ± 2.62%. In this group, ANA-positivity occurred in 66.7% of patients, which indicated the preservation of the activity of the pathological process and the need for further basic therapy.

**Conclusion:** In children with juvenile idiopathic arthritis under the age of 18 years, there are signs of restricted external respiration, which persists for a long time, indicating involvement in the pathological process of the lung. The detected changes persist and occur after one year of illness and are associated with the duration and activity of JIA.

**Disclosure of Interest**: None declared

### P213. The current evidence for transitional care in young people with chronic pain: a systematic review

#### L. Huckerby^1^, J. E. McDonagh^1,2,3^, R. R. Lee^4,5^

##### ^1^Royal Manchester Children’s Hospital, ^2^National Institue of Health Research Biomedical Research Centre, Manchester University Hospital NHS Trust, ^3^Centre for Epidemiology Versus Arthritis, Centre for Musculoskeletal Research, University of Manchester, ^4^National Institute of Health Research Biomedical Research Centre, Manchester University Hospital NHS Trust, ^5^Centre for Epidemiology Versus Arthritis, Centre for Musculoskeletal Research, University of Manchester, Manchester, United Kingdom

###### **Correspondence:** L. Huckerby

**Introduction:**

Paediatric chronic musculoskeletal pain presents a significant individual and societal burden, with an estimated prevalence of 11-38% (1,2,3). A large proportion of adolescents with chronic pain are likely to have unresolved pain which continues into adulthood (4,5). Transitional care is a lengthy process, starting in early adolescence and includes preparation and support for the move from paediatric to adult centred services. To date, there is limited evidence identifying the extent to which transitional care for adolescents with chronic pain has been developed or investigated in research (6).

**Objectives:**

To review the current evidence for transitional care in young people with chronic pain.

**Methods:**

Studies were identified by searching 4 databases: EMBASE, Medline, CINAHL and PsycINFO. The PEO tool was used to develop a search strategy, and terms such as “Adolescent”, “Persistent long-term pain”, and “Transition* (or variations of such words) were implemented.

Inclusion criteria were: sample population age between 10-24 years, a confirmed diagnosis of a condition characterised by chronic pain, any health care setting, any service provider, published peer reviewed, English language. Excluded were case reports, editorials, abstracts, meta-analyses, books or book chapters. Searches took place between September and December 2021.

**Results:**

98 papers were identified by the search, 14 were selected after abstract screening. Two independent reviewers then screened papers for inclusion, extracted data, and assessed the quality of the studies followed by a senior reviewer. Of the 14 papers, full text review found that none of the papers looked specifically at the evidence with respect to transitional care for young people with chronic pain. Of those which did not meet the inclusion criteria, there were 4 papers which informed our discussion.

**Conclusion:**

We found a lack of research considering chronic pain in the context of transitional care. Chronic pain is a feature of many long-term health conditions. It remains unknown as to whether there are any pain-specific aspects of transitional care. How pain management is addressed in existing transitional care provision and the relationship of pain to outcomes of transitional care needs further research. If effective interventions can be provided during these crucial years, the trajectory of these young people as adults can potentially be improved (5).

**References**

1. King S, Chambers CT, Huguet A, et al. The epidemiology of chronic pain in children and adolescents revisited: a systematic review. Pain; 2011;152:272938.

2. Szer IS. Musculoskeletal Pain Syndromes That Affect Adolescents. Arch Pediatr Adolesc Med; 1996;150:740–7.

3. Weiss JE, Stinson JN. Pediatric Pain Syndromes and Noninflammatory Musculoskeletal Pain. Pediatr Clin North Am. 2018 ;65:801–826.

4. Walker LS, Dengler-Crish CM, Rippel S, Bruehl S. Functional abdominal pain in childhood and adolescence increases risk for chronic pain in adulthood. Pain; 2010; 150: 568–72.

5. Kashikar-Zuck S, Cunningham N, Peugh J, et al. Long-term outcomes of adolescents with juvenile-onset fibromyalgia into adulthood, and impact of depressive symptoms on functioning over time. Pain; 2019; 160: 433–41.

6. Dolezalova P, et al. The European network for care of children with paediatric rheumatic diseases: care across borders. Rheumatology (Oxford); 2019; Jul 1;58(7):1188-1195.

**Patient Consent:** Not applicable (there are no patient data)

**Disclosure of Interest**: None declared

### P214. Cardiopulmonary screening in connective tissue disease: UK multi-centre audit

#### H. Lythgoe^1^, K. Mageean^2^, P. Lawrence^1^, S. Mayell^1^, D. Luciano^1^, P. Duong^1^, J. Walsh^3^, E. Twynam-Perkins^3^, M. Ahmid^3^, H. Sansby^3^, C. Anderson^4^, F. Ritchie^4^, L. Crosby^2^, C. Longthorpe^2^, L. McCann^1^, C. Pain^1^

##### ^1^Alder Hey Children’s NHS Foundation Trust, Liverpool, ^2^Bristol Royal Hospital for Children, Bristol, ^3^Royal Hospital for Children, Glasgow, ^4^Royal Hospital for Children and Young People, Edinburgh, United Kingdom

###### **Correspondence:** H. Lythgoe

**Introduction:** Cardiopulmonary involvement in connective tissue disease (CTD) manifests in many ways, may be asymptomatic and can have a significant impact on morbidity and mortality. Investigation of cardiopulmonary symptoms and signs in these patients is therefore important and screening asymptomatic patients may facilitate early recognition and treatment.

**Objectives:** To describe cardiopulmonary involvement in patients with four CTDs (juvenile systemic lupus erythematosus (JSLE), juvenile dermatomyositis (JDM), juvenile systemic sclerosis (JSSc) and juvenile mixed connective tissue disease (JMCTD), define audit standards and measure performance against them.

**Methods:** Current patients seen within the last twelve months from four tertiary paediatric rheumatology centres were included. Available guidance was reviewed and both generic and disease-specific audit standards were defined.

**Results:** 82 patients were included with a median follow-up of 2.8 years. Most patients had JSLE (33) and JDM (32) with smaller numbers of JSSc and JMCTD patients (6 and 11 respectively). Guidance on cardiopulmonary involvement was identified for JSLE (Single Hub and Access Point for Paediatric Rheumatology in Europe (SHARE) and British Society for Rheumatology (BSR) guidance), JDM (SHARE) and JSSc (SHARE). No guidelines were identified for JMCTD.

Screening for cardiopulmonary involvement was inconsistent and standards were largely not met (table 1). Abnormal findings did not always lead to further/repeat investigation; HRCT was not performed for all patients with persistently abnormal lung function. Pulmonary involvement was identified in 24.4% and was most frequent in JSSc (83%). Two patients had ILD. Cardiac involvement was less frequent (9.8%) with pericardial effusion the most common manifestation and one patient with pulmonary arterial hypertension.

**Conclusion:** Guidance for cardiopulmonary screening is limited but cardiopulmonary screening is inconsistently performed for both symptomatic and asymptomatic patients and standards are not met. Service evaluation and development with multi-disciplinary team engagement is required to increase screening and improve long-term outcomes for patients.

**Patient Consent:** Not applicable (there are no patient data)

**Disclosure of Interest**: None declared

**Table 1 (abstract P214).** Performance against audit standards

Audit Standard	JSLE	JDM	JSSc	MCTD
Evaluated for respiratory symptoms and signs at diagnosis and every 6 months	18/30 (60)	13/32 (41)	1/5 (20)	4/11 (36)
Patients with respiratory symptoms and signs should have CXR and PFT including DLCO	4/10 (40)	2/5 (40)	0/1 (0)	3/5 (60)
Patients with concerns re ILD on initial investigation should have HRCT chest	4/5 (80)	1/3 (33)	2/2 (100)	2/3 (67)
Evaluated for cardiac symptoms and signs at diagnosis and every 6 months	20/30 (67)	14/32 (44)	2/5 (40)	4/11 (36)
Patients with cardiac symptoms and/or signs should have echo and ECG	5/8 (63)	2/4 (50)	-	3/6 (50)
JSLE: Patients should have a CXR at diagnosis	24/33 (73)	-	-	-
JSLE: Patients with exertional intolerance should have CXR, PFT (with DLCO), ECG and echo	2/7 (29)	-	-	-
JDM/JSSc: All patients should have PFT including CO diffusion at diagnosis (JSSc: Patients should have PFT including CO diffusion every 6 months)	-	6/32 (19)	5/6 (83) 0/5 (0)	-
JSLE/JDM: All patients should have an ECG at diagnosis	20/33 (61)	16/32 (50)	-	-
JSLE/JDM: Patients should have an echo at diagnosis	23/33 (70)	11/32 (34)	-	-
JSSc: Patients should have an echo every 6 months	-	-	0/5 (0)	-

DLCO=diffusing capacity for carbon monoxide; ILD=interstitial lung disease; HRCT=high resolution computerised tomography

### P215. Educating and empowering young patients with JIA using a board game in group sessions

#### A. Pieler, H. K. Ipsen, A. Estmann Christensen, P. Toftedal

##### H.C. Andersen Childrens Hospital, Odense , Denmark

###### **Correspondence:** A. Pieler

**Introduction:** During the process of improving transition to adult rheumatology it became apparent that some of the adolescents had little insight and understanding of their disease or of disease management.

**Objectives:** To evaluate group sessions in adolescents using a board game designed to facilitate discussions on JIA. To optimize the young patients knowledge about their diagnosis and treatment.

**Methods:** The nurses interviewed a random sample of the adolescents during their ordinary visit to the outpatient clinic, in order to get an understanding of what would make them attend and take an active part in a group session.

The most optimal format seemed to be one-time-only sessions of maximum duration of two hours in the evening as to not interfere with school.

The young persons needed a unifying element in the sessions that made it feel safe for them to initiate talking and sharing with others while in an un-known setting. We created a board game about JIA as a way to begin the sessions and facilitate a conversation about JIA.

Nurses from the pediatric rheumatology team organized and hosted group sessions with 4-8 adolescents. All the participants knew the nurses in advance. No parents or doctors were present in these group sessions.

The adolescents were invited by mail to participate in a group session on a day of an ordinary visit.

An anonymous evaluation format was used at the end of the session.

**Results:** Since the first group session was held in 2019, there has been six totally. Between two and five adolescents attended each session.

Getting the adolescents to attend has proven to be a challenge. Many are skeptical about being in a setting with strangers and without their parents. The younger the adolescents are the easier it is to get them to attend as they do not have the same pressure of not missing school and are still very influenced by their parents, who often want them to attend.

Feedback shows that they all gain something from attending and find it has meaning for them.

The board game works very well as a means to convey information about disease, complications and treatment and to facilitate discussion and thoughts about the future. Sometimes it was used throughout the session, and other times just as a preliminary means to get the conversation started.

**Conclusion:** The most appropriate age for group session seemed to be at the age of 15-16 years. The patients older than this were more obliged regarding school.

Feedback from those attending shows that the group sessions give meaning for the adolescents. They gain knowledge that they did not have before.

For most of the attending adolescents, the session was their first meeting with other young persons with JIA. Meeting and talking with others in a similar situation was of high value for them.

Though the groups have been smaller than intended, it may have had a positive effect on the level of engagement. Almost all of the adolescents have participated actively during the sessions.

**Disclosure of Interest**: None declared

**Table 1 (abstract P215).** See text for description

N = 14 (9 female, 5 male)	Yes	No	Maybe
Did you learn something you did not know before?	13	1	
Has it been meaningful to participate in the session?	14		
Would you attend another session if asked	8		2

### P216. Clinical evolution of adults with childhood onset rheumatic diseases: analysis of a 10-year experience from a transitional unit in Spain

#### C. Udaondo^1^, L. Nuño Nuño^2^, C. Millan - Longo^1^, C. Muñoz - Gomez^1^, B. Diaz - Delgado^1^, S. Murias^1^, R. Alcobendas^1^, A. Balsa^2^, A. Remesal^1^

##### ^1^Paediatric Rheumatology, ^2^Rheumatology, Hospital La Paz, Madrid, Spain

###### **Correspondence:** C. Udaondo

**Introduction:** Most childhood-onset rheumatic diseases continue into adulthood and in some cases they have sequelae of their disease, with clinical features that differ from those of paediatric age. Transitional units as well as specific training for rheumatologists who will follow up these patients are needed to ensure the successful control of the disease.

**Objectives:** To describe the characteristics, complications and clinical evolution of patients followed up in transitional consultation and identify characteristics associated with a better prognosis of the disease.

**Methods:** We performed a retrospective, longitudinal observational study of patients followed up in a transitional clinic in a reference hospital in Madrid from 2012 to 2022. Patients were seen together by paediatric rheumatologists and an adult rheumatologist in the paediatric unit for 2-4 visits, and were subsequently transferred to an adult rheumatology clinic with the same adult rheumatologist, who did long-term follow-up.

Medical charts were reviewed. Clinical and treatment data were collected and patients with loss to follow-up were contacted. A descriptive analysis was carried out and differences were analysed using contingency tables for qualitative variables and t-tests for independent samples for quantitative variables.

**Results:** 87 patients were included in follow-up at the transitional unit in the study period (72.4% female), with an age at diagnosis of 9±5 years, and a mean time of disease evolution of 10±5 years. The most frequent diagnosis was oligoarticular JIA. At the time of transfer, patients had a mean age of 19±1 years (16-24 years), 85.1% receivied immunomodulatory treatment and 62.1% biologic therapy (etanercept 23%, adalimumab 18.4%, tocilizimab 8%, golimumab and anakinra and infliximab 2.3% each, abatacept and certolizumab 1.1% each). Two patients were being treated with 2 biologics simultaneously due to high disease activity.

15 (17.2%) were in prolonged clinical remission without treatment, showing a longer time of disease progression (13±4 years vs. 8±5 years; p=0.07). No differences were found between prolonged remission off medication and sex or age at onset. There was only a loss to follow-up in 5 cases (5.7%). As important complications, there was an unwanted pregnancy in patient with active SLE and a Felty’s syndrome that required intensive care unit admission and was related to poor adherence to treatment.

**Conclusion:** The combined paediatricians-rheumatologists model seems to be well accepted by patients and relatives, with a low number of losses to follow-up. There was a high number of patients on biologic therapy. There is a need for additional resources to avoid major complications.

**Disclosure of Interest**: None declared

## Poster session: Treat-to-target

### P217. Treat-to-target strategies for the management of Familial Mediterranean Fever in children

#### L. Ehlers^1^, E. Rolfes^1^, M. Lieber^1^, D. Müller^1^, E. Lainka^2^, F. Gohar^3^, G. Klaus^4^, H. Girschick^5^, J. Hörstermann^6^, J. Kümmerle-Deschner^7^, J. Brunner^8^, K. Palm-Beden^3^, K. Tenbrock^9^, L. von Wrangel^10^, M. Faßhauer^11^, N. Blank^12^, R. Trauzeddel^13^, S. L. von Stuckrad^1^, S. Higgins^14^, T. Welzel^7^, T. Lutz^12^, V. Hentgen^15^, D. Föll^16^, H. Wittkowski^16^, T. Kallinich^1^

##### ^1^Charité University Hospital, Berlin, ^2^University Hospital Essen, Essen, ^3^St. Josef-Stift Sendenhorst, Northwest German Center for Rheumatology, Sendenhorst, ^4^KfH Center of Paediatric Nephrology, Marburg, ^5^Vivantes Klinikum Friedrichshain, Children’s Hospital, ^6^Deutsches Rheuma-Forschungszentrum (DRFZ), An Institute of the Leibniz Association, Berlin, ^7^University Hospital Tübingen, Tübingen, Germany, ^8^Medical University Innsbruck, Innsbruck, Austria, ^9^RWTH Aachen, Aachen, ^10^Patient representative, Berlin, ^11^Hospital St. Georg GmbH Leipzig, Academic Teaching Hospital of the University of Leipzig, Leipzig, Leipzig, ^12^University Hospital of Heidelberg, Heidelberg, ^13^Helios Klinikum Berlin-Buch, Berlin, ^14^Paediatric medical practice Hürthpark, Hürth, Germany, ^15^Versailles Hospital, Versailles, France, ^16^University Hospital Münster, Münster, Germany

###### **Correspondence:** L. Ehlers

**Introduction:** Familial Mediterranean fever (FMF) is a monogenic autoinflammatory disease presenting with recurrent fever episodes accompanied by serositis and elevated inflammatory parameters. Amyloidosis as a sequela of persistent inflammation represents the main cause of mortality. 5-10% of patients do not achieve satisfactory disease control on colchicine monotherapy. Increasing therapeutic options with the recent approval of IL-1 antagonists underline the need for disease activity-guided treatment recommendations.

**Objectives:** The objective of this PRO-KIND initiative of the German Society for Paediatric Rheumatology (GKJR) was to develop consensus-based treat-to-target (T2T) strategies for FMF to improve patient care and long-term outcome.

**Methods:** An initial set of statements was developed in accordance with existing management recommendations. Following a systematic literature review, these preliminary statements were adapted in line with the current state of knowledge and evaluated by means of a Delphi survey. Applicability of the approach in daily practice was ensured via a survey conducted among paediatric rheumatologists in Germany. Data from the national AID-Net registry were analysed with respect to therapeutic response.

**Results:** This T2T initiative yielded a total of 26 statements supported by evidence and validated by expert agreement that cover the following categories: i) diagnosis, ii) differential diagnosis, iii) treatment targets, iv) colchicine treatment, v) monitoring, vi) colchicine intolerance / inadequate colchicine response, and vii) colchicine reduction / termination. Multidimensional treatment targets incorporating objective and subjective reported outcome measures were developed. They comprise attack frequency, severity of the single attack, number of school days missed due to FMF, level of inflammatory markers in the attack-free intervals, occurrence of chronic sequelae, as well as the subjective patient and physician reported outcome measure of satisfaction with the current disease status. A composite score of these items categorises disease severity as follows: remission or minimal disease activity, mild/moderate/severe disease activity. According to this evaluation, management recommendations are provided in the form of a flow chart to guide individual treatment decisions following the respective paths: continue colchicine, persisting attacks / inflammation, colchicine intolerance, persisting arthritis, colchicine reduction and adjustment/reduction of biologics. Data from the national AID-Net showed that colchicine therapy was successfully terminated in 63% of patients (27/44) with heterozygous *MEFV* mutations.

**Conclusion:** The proposed consensus treatment plan for the management of FMF incorporates multidimensional targets allowing transparent treatment decisions, which will promote personalised disease management and increase adherence to therapy.

**Patient Consent:** Not applicable (there are no patient data)

**Disclosure of Interest**: L. Ehlers: None declared, E. Rolfes: None declared, M. Lieber: None declared, D. Müller: None declared, E. Lainka Consultant with: Mirum, Albireo, F. Gohar: None declared, G. Klaus: None declared, H. Girschick: None declared, J. Hörstermann: None declared, J. Kümmerle-Deschner Grant / Research Support with: Novartis, SOBI, Consultant with: Novartis, SOBI, J. Brunner: None declared, K. Palm-Beden: None declared, K. Tenbrock Grant / Research Support with: Pfizer, Consultant with: Pfizer, Novartis, L. von Wrangel: None declared, M. Faßhauer: None declared, N. Blank Grant / Research Support with: Novartis, SOBI, Consultant with: Novartis, SOBI, R. Trauzeddel Consultant with: Novartis, S. L. von Stuckrad: None declared, S. Higgins: None declared, T. Welzel: None declared, T. Lutz: None declared, V. Hentgen Grant / Research Support with: Novartis, SOBI, D. Föll: None declared, H. Wittkowski Grant / Research Support with: Takeda, Consultant with: Novartis, Takeda, T. Kallinich Consultant with: Roche

### P218. Implementing a treat to target strategy for pediatric lupus: design of an intervention

#### E. A. Smitherman^1^, J. G. Harris^2^, A. O. Hersh^3^, A. Chapson^4^, S. Oscar^4^, K. Wiegand^5^, E. M. Morgan^6^, J. M. Burnham^7^

##### ^1^University of Alabama at Birmingham, Birmingham, ^2^University of Missouri Kansas City, Kansas City, ^3^University of Utah, Salt Lake City, ^4^parent co-author, n/a, n/a, ^5^Cincinnati Children’s Hospital, Cincinnati, ^6^University of Washington, Seattle, ^7^University of Pennsylvania, Philadelphia, United States

###### **Correspondence:** E. A. Smitherman

**Introduction:** Achievement of a low disease activity state, including remission, has been associated with less organ damage, fewer disease flares, and improved health-related quality of life in systemic lupus erythematosus (SLE). No prior studies have operationalized disease activity measures for clinical use.

**Objectives:** Our objective was to design an intervention to standardize measurement of lupus disease activity and implement a Treat to Target strategy for lupus in the pediatric rheumatology clinic.

**Methods:** Our team of 4 pediatric rheumatologists from 4 centers and 2 parents of children with lupus participated in bimonthly coaching sessions with a quality improvement specialist and advisor from May to November 2021. A scoping literature review was conducted to determine disease activity definitions with the best evidence for clinical use. We utilized the Model for Improvement and implementation mapping to design toolkit materials. Finally, we completed a baseline assessment using the National Implementation Research Network’s Hexagon Tool, Acceptability of Intervention Measure, Intervention Appropriateness Measure, and Feasibility of Intervention Measure. Each domain was assessed using a Likert scale 1-5 with 5 as the most positive response, and the mean response was calculated for each item.

**Results:** We selected the Lupus Low Disease Activity State (LLDAS) and Definition of Remission in SLE (DORIS) definitions to operationalize for clinical use. We identified 4 key drivers for improving the proportion of clinic visits with disease activity and glucocorticoid targets assessed: 1) timely and complete data collection and documentation, 2) usable tools for data interpretation, 3) collaborative, efficient, and effective care teams, and 4) informed, activated, and engaged patients and families. We developed process maps and failure modes effect analysis around expected barriers to collecting disease activity measures in clinic. We defined process and outcome measures for a Lupus Treat to Target intervention and designed a phased implementation approach (Table 1). We co-produced a handout with information on lupus disease activity for patients and parents with our parent partners, as well as clinic staff educational material. On the Hexagon Tool, the highest ranking domain was Fit (4.3), followed by Capacity (4.2), Supports (4.0), Usability (4.0), Need (3.5), and Evidence (3.3). We found the intervention to be acceptable (4.9), appropriate (4.8), and feasible (4.4) among our team members.

**Conclusion:** We designed and evaluated a Lupus Treat to Target Toolkit. Our next step is to pilot test implementation. Our long-term goal is to form a multi-center Pediatric Lupus Quality Collaborative with potential for significant impact on health outcomes in pediatric lupus.

**Disclosure of Interest**: None declared

**Table 1 (abstract P218).** Process and outcome measure definitions for a Lupus Treat to Target Intervention

Implementation Phase	Estimated Length of Time	Measure Title
Pre-Work, Phase 0	3 months	Implementation science measures (appropriateness, acceptability, feasibility)
Process Measures, Phase 1	6 months	SLE Disease Activity Index (SLEDAI) completion New disease activity attestation Physician global assessment (0-3) completion Prednisone dose documented
Process Measures, Phase 2	6 months	Disease activity target set Steroid dose target set Provider attestation of disease activity status Provider attestation of steroid dose status
Outcome Measures, Phase 3	3 months	LLDAS criteria met SLEDAI-2K ≤ 4 Prednisone dose (continuous, dichotomous)
Balancing Measures, Phase 3	3 months	New disease activity Steroid dose escalation
Sustain Phase, Phase 4	ongoing	All of the above

## Poster session: Treatment

### P219. Multitarget therapy in lupus podocytopathy: case and literature review

#### M. A. V. Barba Aguilar, S. Rodriguez Aguayo, E. Faugier Fuentes, A. K. Primero Nieto, H. W. Bermudez Canales, A. Guzman Revilla

##### Rheumatology, HOSPITAL INFANTIL DE MÉXICO, FÉDERICO GÓMEZ, Ciudad de México, Mexico

###### **Correspondence:** M. A. V. Barba Aguilar

**Introduction:** Lupus Nephritis is a major cause of morbidity and mortality in adult and pediatric patients with systemic lupus erythematosus. Approximately 20% to 75% of children with will develop nephritis. While, the pathogenesis of LN indicates the formation of immunocomplexes by complement activation and Fcy receptors and relates to the classification of Lupus Nephritis according to the 2003 International Society of Nephrology/Renal Pathology Society criteria. Additional histologic subtypes have now been described. Lupus Podocytopathy has been described in 1-1.6% of renal biopsies compatible with lupus nephritis as an additional histologic subtype of this disease, characterized by effacement of the podocyte footprint observed on electron microscopy and characterized by histopathologic lack of subendothelial and subepithelial immunocomplex deposits, occasional presence of mesangial deposits on immunofluorescence; clinically it is characterized by severe nephrotic range proteinuria. Currently, comparative studies have been carried out with the use of multitarget therapy with mycophenolate and tacrolimus as therapy for this entity, observing remission and reduction of relapse.

**Objectives:** To present a case of Lupus Podocytopathy treated with multitarget therapy with mycophenolate and tacrolimus in a patient with lupus nephritis.

**Methods:** Clinical case description and literature review.

**Results:** A 14 year old boy who started with acute renal failure and Nephrotic Syndrome, was treated with hemodialysis due to dialysis urgency. Systemic Lupus Erythematosus was diagnosed, so methylprednisolone and steroid were administered orally and multitarget therapy with mycophenolate 2 gr/day and cyclophosphamide 750 mg/m2/dose, during hospitalization he presented cytomegalovirus infection and due to persistent activity gamma globulin and plasmapheresis was administered for 5 sessions. He presented Thrombotic Microangiopathy and 5 sessions of plasmapheresis were indicated again. A renal biopsy was performed which reported class III lupus nephropathy, activity index 4/24 and chronicity 2/23, immunofluorescence with mesangio IgG+, IgM+, scarce deposits. Proteinuria persisted in nephrotic range after 6 boluses of cyclophosphamide, so rituximab 375 mg/m2 (4 weeks) was started with refractoriness. Electron microscopy was performed with a report of effacement of filtration diaphragms, consistent with lupus podocytopathy. Multitarget therapy was started with tacrolimus 0.07 mg/kg and mycophenolate with remission of proteinuria, negative anti DNA, normal C3 and C4, inactive sediment.

**Conclusion:** Podocytopathy should be suspected in those patients with proteinuria persisting in the nephrotic range and electron microscopy should be performed. In addition, it should be considered that in spite of not being included in the classification there are cases in the literature in adults and children with this entity. Multitarget therapy in the case of this patient achieved complete remission of the disease and SLEDAI of 0 points, which could suggest a therapy option in those refractory cases, even those treated with rituximab.

**Patient Consent:** Yes, I received consent

**Disclosure of Interest**: None declared

### P220. Oral midazolam before intra-articular corticosteroid injections in juvenile idiopathic arthritis

#### M. Burrone^1^, M. Guazzi^1^, R. Naddei^1^, M. Spelta^1^, C. Malattia^1^, N. M. Disma^2^, A. Ravelli^3^, A. Consolaro^1^

##### ^1^UOC Clinica Pediatrica e Reumatologia, ^2^UOC Anestesia e Terapia del dolore Acuto e Procedurale, ^3^Direzione Scientifica, Istituto Giannina Gaslini, Genova, Italy

###### **Correspondence:** M. Burrone

**Introduction:** Intraarticular corticosteroid injections (IACI) are widely used in the management of juvenile idiopathic arthritis (JIA) to induce rapid relief of symptoms of active synovitis and to obtain resolution of functional impairment. Midazolam, a short acting benzodiazepine, has been found to be an effective premedication in children by reducing anxiety and mitigating uncooperative behavior with a large margin of safety. Midazolam is primarily administered intravenously; however, recently a new oral solution designated for procedural sedation in children has been introduced. Several studies in this field have shown that a child’s level of anxiety before a painful medical procedure is directly correlated with the amount of pain experienced.

**Objectives:** We aimed to determine the efficacy of oral midazolam as premedication to improve tolerance of IACI in children with JIA

**Methods:** In this one month pilot study, all consecutive patients who were prescribed one or more IACIs by the caring physician at the study Unit were proposed to participate in the study. Premedication with midazolam was offered to an unselected group of children based on the availability of the procedural anesthesia team of the Gaslini Institute. All children received local anesthesia. Patients in Group 1 received 0.25 mg/kg (maximum 15 mg) of oral midazolam solution and local anesthesia with lidocaine/prilocaine 5% cream, 30 to 45 minutes before IACI; patients in Group 2 received only local anesthesia. Patients’ pain experienced before and at the end of the procedure were assessed using a 0-10 21-circle visual analog scale (VAS). Subjective rating of the ease of performing the procedure by the operator was recorded on a 4 points Likert scale (“Very easy”, “Easy”, “Quite difficult”, “Very difficult”). The level of anterograde amnesia was recorded on a 3 points Likert scale (“No amnesia”, “Partial amnesia”, “Complete amnesia”). During procedure and until two hour after, vital signs and oximetry were monitored and recorded in children receiving midazolam.

**Results:** Seven patients were enrolled in Group 1 and seven patients in Group 2. Patients receiving only local anesthesia were older (mean age at procedure 16 years for Group 2, 12 years for Group 1, p = 0.04); 28% of patients received multiple IACIs in both groups. Pain level prior the procedure was similar in the two groups (p = 0.7). Patients received midazolam reported less pain (mean score 2.1) compared those receiving local anesthesia alone (mean score 3.4) at the end of the procedure, although the difference was not significant (p = 0.25). In 28% of cases physician described the procedure as difficult in Group 2, compared to 14% in Group 1. All patients receiving midazolam reported some degree of anterograde amnesia (28% reported complete amnesia, 72% partial amnesia) when recalling the procedure. No adverse events were recorded in both groups.

**Conclusion:** In this small cohort of children, although not significantly, oral midazolam reduced pain during IACIs and increased the ease of the procedure. Oral midazolam is a safe and effective premedication before IACI in patients who require or prefer sedation and improves the child’s perception of the procedure, by inducing anterograde amnesia. This pilot study opens to further investigation, aiming to contribute to improvement in patient care and outcomes.

**Patient Consent:** Yes, I received consent

**Disclosure of Interest**: None declared

### P221. JAK inhibitors for the treatment of rheumatologic diseases in children: a retrospective case series

#### M. D’Agostin^1^, A. Pin^2^, A. Tesser^2^, S. Pastore^2^, A. Taddio^2^, A. Tommasini^1,2^

##### ^1^University of Trieste, ^2^Institute of Maternal and Child Health – IRCCS Burlo Garofolo, Trieste, Italy

###### **Correspondence:** M. D’Agostin

**Introduction:** Based on their mechanism of action and clinical experiences in small series, JAK inhibitors (JAKinibs) have been proposed to treat paediatric diseases characterized by an interferon-mediated inflammation, when conventional anti-inflammatory therapies have failed (1,2).

**Objectives:** We aim to describe our clinical experience with JAKinibs in the treatment of rheumatologic diseases in children.

**Methods:** We enrolled all the paediatric patients who underwent a treatment with JAKinibs for a rheumatologic condition at the Institute for Maternal and Child Health IRCCS Burlo Garofolo (Trieste), between December 2016 and April 2022. The benefit was evaluated considering the reduction of clinical symptoms, the need of corticosteroids treatment and the number of concomitant medications. Changes in IFN score and inflammation biomarkers were assessed. Finally, adverse effects were reported and evaluated.

**Results:** Overall, we treated thirteen patients (10 females; mean age 13.6 ± 5.9 years) for a mean duration of 2.1 ± 1.5 years. Nine patients were treated with baricitinib (mean dose 0.17 ± 0.11 mg/kg/day), the remaining four patients with tofacitinib (mean dose 0.30 ± 0.05 mg/kg/day). The treatment indications were heterogeneous. Half patients had monogenic disorders associated with increased interferon signalling: two COPA syndrome, one monogenic lupus due to DNAse2 deficiency, one STAT1 gain-of-function disease with systemic lupus erythematosus (SLE)-like phenotype, one histiocytosis-lymphadenopathy plus syndrome, one CANDLE (chronic atypical neutrophilic dermatosis with lipodystrophy and elevated temperature) syndrome. The others had multifactorial disorders with high interferon score and poor response to first-line treatment: two polyarticular juvenile idiopathic arthritis (JIA), one SLE, one Weber-Christian panniculitis, one juvenile systemic sclerosis, one localized scleroderma and one scleroderma-like chronic cutaneous graft versus host disease. The mean age at disease onset and at the start of treatment were respectively 5.6 ± 4.3 years and 11.4 ± 5.3 years. Almost all the patients, except one, had a refractory disease that had not responded to conventional antirheumatic drugs and biologics. Eleven patients had tried several medications before JAKinibs (mean 3.6, range 0–9 medications). Moreover, ten patients out of 13 (76.9%) were receiving corticosteroids before the start of JAKinibs treatment. Daily prednisone dose decreased significantly after the start of JAKinibs from 0.52 mg/kg/day (range 0.2 – 1 mg/kg/day) to 0.07 mg/kg/day (range 0 - 0.2 mg/kg/day, p-value <0.001). Interesting, for six patients out of 10, the treatment with corticosteroids was not necessary once they started JAKinibs therapy. Moreover, the number of concomitant medications used after the start of JAKinibs tended to be lower than before (mean 2.07, range 1-3) (p=0.56). We obtained a good control of the disease in ten patients (76.9%), with clinical improvements. In three cases we attended a progression of disease. A reduction of interferon signalling was recorded in eight out of ten subjects at last follow-up, irrespectively from clinical improvements. Three patients developed drug-related adverse events, which were considered serious just in one case: two of them had a worsening of lymphopenia and one a transient increase of gammaglutamyl transferase and dyslipidaemia.

**Conclusion:** Our experience confirms how JAKinibs may be a valuable treatment for severe interferon-mediated inflammatory disorders in children and it is confirmed by an improvement of symptoms, a reduction of daily prednisone dose and of the number of concomitant medications.

**Patient Consent:** Yes, I received consent

**Disclosure of Interest**: None declared

### P222. Impact of medications’ side effects on jia patients’ health related quality of life and well-being

#### M. Dellepiane^1^, E. Pescio^1^, M. Spelta^1^, R. Naddei^1^, M. Burrone^2^, C. Trincianti^2^, F. Ridella^2^, R. Cuttica^3^, A. Estmann^3^, S. Kamphuis^3^, A. Ravelli^2,4^, N. Ruperto^3^, A. Consolaro^1,2^

##### ^1^Clinica Pediatrica e Reumatologia, IRCCS G. Gaslini, ^2^Dipartimento di Neuroscienze, Riabilitazione, Oftalmologia, Genetica e Scienze Materno-Infantili, Università degli Studi di Genova, ^3^UOSID Centro Trial Printo, ^4^Direzione Scientifica, IRCCS G. Gaslini, Genoa, Italy

###### **Correspondence:** M. Dellepiane

**Introduction:** In recent years, there have been remarkable advances in the management of JIA, which comprise the advent of medications that are capable of inducing extended periods of complete disease quiescence. However, it is well known that prolonged treatment with immune-modulatory medications may be associated with the presence of side effects (SEs). It was not yet explored the impact of SEs on patients’ perception of the disease status.

**Objectives:** Aim of the study is to explore the impact of the presence and number of medications’ SEs on the health related quality of life of JIA children.

**Methods:** We included subjects in the EPidemiology, treatment and Outcome of Childhood Arthritis (EPOCA) study, receiving medications to treat JIA. The EPOCA dataset is made of more than 9.000 patients with JIA from 118 pediatric rheumatology centres in 49 countries. The presence and classification of SEs was collected by the juvenile arthritis multidimensional assessment report (JAMAR). When available, patient-reported data were considered; differently, parent proxy-reported data were retained. Disease features and parent/patient reported outcomes (PROs) were compared between subjects reporting and those not reporting medications SEs. The frequency of each side effect was compared as well: school related problems; compliance to prescribed therapy; satisfaction with current disease state. Finally, we compared the level of well-being measured with a 0-10 VAS and the level of health related quality of life measured with the Pediatric Rheumatology Quality of Life (PRQL) scale according to the number of SEs reported by the patient or parent.

**Results:** We included in the analysis 6,911 JIA patients receiving treatment for JIA; 2,041 (29.5%) reported at least one side effect of medications. “Nausea” (12%), “Mood swings (excitement, depression, anxiety)” (7%), “Pain or burning feeling in the stomach” (7%), and “Headache” (7%) were the SEs more frequently reported. Children with SEs had older age at onset and higher JADAS, and were receiving more frequently synthetic DMARDs and corticosteroids (p < 0.001). Table shows the comparison of main PROs based on the presence of SEs. Patients reporting school related problems had more frequently SEs (43% vs 24%) and subjects satisfied with disease outcome and reporting compliance to therapy had less frequently SEs of medication (27% vs 39% and 30% vs 48%, respectively). The Well-Being VAS and the PRQL score progressively worsened when subjects reported no SE, 1 SE, 2 SEs, and >2 SEs (p < 0.001 for both comparisons).

**Conclusion:** The presence of medications SEs had a significant impact on patients’ health related quality of life, and in particular on school-related problems, compliance to therapy, and satisfaction with disease state. Parents and patients give weight to the presence and the number of SEs when scoring the well-being VAS.

**Patient Consent:** Yes, I received consent

**Disclosure of Interest**: M. Dellepiane: None declared, E. Pescio: None declared, M. Spelta: None declared, R. Naddei: None declared, M. Burrone: None declared, C. Trincianti: None declared, F. Ridella: None declared, R. Cuttica: None declared, A. Estmann: None declared, S. Kamphuis: None declared, A. Ravelli Speaker Bureau with: AbbVie, Angelini, BMS, Pfizer, Hoffman LaRoche, Novartis, Pfizer and Reckitt Benckiser, N. Ruperto Grant / Research Support with: Bristol Myers and Squibb, Eli-Lilly, F Hoffmann-La Roche, Novartis, Pfizer, Sobi, Consultant with: 2 Bridge, Amgen, AstraZeneca, Aurinia, Bayer, Brystol Myers and Squibb, Celgene, inMed, Cambridge Healthcare Research, Domain Therapeutic, EMD Serono, Glaxo Smith Kline, Idorsia, Janssen, Eli Lilly, Novartis, Pfizer, Sobi, UCB, A. Consolaro Grant / Research Support with: Pfizer

**Table 1 (abstract P222).** See text for description

	Reporting no SEs N = 4562	Reporting SEs N = 2041	p
Well-being VAS, 0-10 (median [IQR])	1.0 [0.0, 3.0]	2.0 [0.5, 5.0]	<0.001
Pain VAS, 0-10 (median [IQR])	0.5 [0.0, 3.0]	2.0 [0.0, 5.0]	<0.001
Disease activity VAS, 0-10 (median [IQR])	1.0 [0.0, 3.5]	2.0 [0.5, 5.0]	<0.001
Presence of morning stiffness (%)	1422 (31.3)	935 (46.1)	<0.001
Functional ability score, 0-45 (median [IQR])	1.0 [0.0, 4.0]	2.0 [0.0, 6.0]	<0.001
PRQL physical, 0-15 (median [IQR])	1.0 [0.0, 4.0]	3.0 [1.0, 6.0]	<0.001
PRQL psychosocial, 0-15 (median [IQR])	1.0 [0.0, 3.0]	3.0 [1.0, 5.0]	<0.001
PRQL total, 0-30 (median [IQR])	3.0 [0.0, 6.0]	6.0 [2.0, 10.0]	<0.001

### P223. Use and outcome of intra-articular steroid injections in juvenile idiopathic arthritis

#### A. Dghaies, K. Maatallah, H. Ferjani, L. Kharrat, S. Miladi, W. Triki, D. Ben Nessib, D. Kaffel, W. Hamdi

##### Rheumatology, Mohamed Kassab Institute, Manouba, Tunisia

###### **Correspondence:** W. Hamdi

**Introduction:** The intra-articular steroid injections are a commonly used tool in the management of juvenile idiopathic arthritis (JIA).

**Objectives:** This study aimed to assess the efficacy of intra-articular steroid injections in JIA patients.

**Methods:** It was a retrospective study including children with JIA (according to the International League of Associations for Rheumatology (ILAR)) followed in the rheumatology department of Kassab institute Tunisia. General demographic and clinical information were collected; family history of chronic inflammatory diseases, child’s current age, age at disease onset, duration of disease progression, JIA subset, visual analog scale (VAS), inflammatory biomarkers (sedimentation rate (SR), c reactive protein (CRP), and concomitant treatments. Intra-articular steroid injections and their efficiency were noted.

**Results:** Among 43 patients with JIA, 11 children (8 boys) needed intra-articular steroid injections and one of them needed two injections. The mean age was 12.2 ±4.2 years old [4-19]. The mean duration of the disease was 2.5 years, with extremes varying from one to 4 years. The mean age at disease onset was 9.7 ±4.3 years [2-15]. Family history of a chronic inflammatory disease was noted in 3 cases and psoriasis in 2 cases. The frequency of each JIA subset was as follows: polyarticular with rheumatoid factor (n=1), psoriatic arthritis (n= 1), enthesitis-related arthritis (n=6), and oligoarthritis (n= 3). One patient had a positive antinuclear antibody. As for the treatment course, 9 patients received non-steroidal anti-inflammatory drugs (naproxen in 3 patients, diclofenac in 5 patients, and naproxen then diclofenac in 3 patients). Seven patients received methotrexate with a mean dose of 10mg per week and 3 patients received biological treatment (etanercept in 2 cases, adalimumab in 1 case). The mean patient VAS was 4.7±2.4, the mean parent VAS was 2± 1.42, and the mean doctor VAS was 5±1.4. The mean SR was 26.5 ±29 mm/h, and the mean CRP was 12.1 ±13.2. Among the eleven intra-articular steroid injections performed, 2 injections were in the sacroiliac joints, 6 in hip joints, and 4 in knee joints. The corticosteroid used was triamcinolone hexacetonide in 3 cases for the hip joint and betamethasone in the other cases. There was a negative significant association between the use of the injection and the duration of the disease (p=0,04).

Patients were evaluated during the next visit after a mean duration of 2.6± 1.2 weeks.

Six patients reported improvement of the pain (mean patient VAS dropped to 3.2 ±1.3) with a mean improvement rate in joint pain of 53.3% [40-80]. There was no improvement and no worsening of the pain for the rest of the patients.

**Conclusion:** The result of our study showed that Intra-articular steroid injections helped to reduce pain in JIA patients. Those injections may be useful, especially as a bridge therapy, in non-yet controlled disease.

**Disclosure of Interest**: None declared

### P224. Neridronate treatment in a pediatric cohort

#### M. Ricci^1^, A. Marino^2^, M. Gattinara^2^, C. Chighinzola^1^, I. Pontikaki^2^, R. Caporali^1^, R. Cimaz^1^, T. Giani^1^

##### ^1^University of Milan, ^2^Gaetano Pini Hospital, Milan, Italy

###### **Correspondence:** T. Giani

**Introduction:** Bisphosphonates (BPs) have been increasingly used in pediatrics for the treatment of different disorders characterized by bone fragility, even if long-term efficacy and safety studies are still lacking. Currently, the use of Neridronate in Italy is licensed for adult patients affected by Paget’s disease and complex regional pain syndrome type 1 (CRP1), and under 18 years of age, for the treatment of Osteogenesis Imperfecta (OI).

**Objectives:** We analyzed the safety profile of Neridronate in a pediatric cohort, and secondary we evaluated its efficacy in reducing bone lesions observed at Magnetic Resonance Imaging (MRI).

**Methods:** In this retrospective observational study, we included 25 patients (17 females, 8 males), with a median age of 11 years and 6 months (range 1 year and 2 months- 17 years and 4 months) who received Neridronate for one of the following conditions: CRP1 (13), chronic recurrent multifocal osteomyelitis, CRMO (6), OI (2), osteochondritis (1), sacroiliac bone edema (1), avascular necrosis (AVN) of the femoral head (1), reduced bone mineral density associated with Neurofibromatosis type I (1).

Neridronate was intravenously administered at a standard dose of 2mg/kg. The treatment regimen included a single infusion every 3 months or a cycle of 4 infusions over 14 days, eventually followed by a 3-month booster dose.

**Results:** Fifteen out of 25 children (60%) had some side effect; flu-like symptoms were the most frequent one, observed in 14/15 cases. These patients complained of arthralgia and low-grade fever, that developed within the first 24-48 hours from the infusion and lasted 1-2 days. One patient referred itching during the first minutes of the first infusion in the absence of cutaneous signs, one presented bilateral conjunctivitis that resolved in one day, one referred significant nausea, which spontaneously resolved in a few hours, and one presented transient neutropenia (lowest value 700/mmc), normalized within 2 weeks. The majority of the patients who presented some symptoms (14/15, 93.3%) developed them at the first infusion.

In 21/25 patients a post-Neridronate treatment MRI was available. A clear improvement or a complete recovery of the radiological findings detected at the pre-Neridronate MRI was observed in 9/13 patients with CRP1 , 3/6 with CRMO, 2/2 with OI, and 1/1 with bone edema at the sacroiliac joint.

**Conclusion:** In this pediatric cohort side effects linked to intravenous use of Neridronate, although not unusual, resulted to be mild, rapidly self-limiting, and commonly associated with the first infusion.

Despite our limited and heterogeneous data, Neridronate seems to be effective.

**Patient Consent:** Yes, I received consent

**Disclosure of Interest**: None declared

### P225. Safety, and disease activity after live attenuated measles-mumps and rubella booster vaccination in children with juvenile idiopathic arthritis treated with immunosuppressive therapy: a retrospective long term follow up study

#### M. Hamad Saied, J. W. van Straalen, M. Jansen, G. de Joode-Smink, N. M. Wulffraat

##### Department of Paediatric Immunology and Rheumatology, Wilhelmina Children’s Hospital, University Medical Center Utrecht, Utrecht, The Netherlands, Utrecht, Netherlands

###### **Correspondence:** M. Hamad Saied

**Introduction:** Vaccines, especially live-attenuated vaccines, in immunocompromised patients pose a great challenge, due to the hypothetical risk of infection with the live-attenuated pathogen, lower immunogenicity due to treatment and the fear that vaccination itself may lead to disease flare[1].

In the Netherlands, JIA patients receive a measles, mumps and rubella (MMR) booster vaccine around age nine years as part of the Dutch National Immunization Program. This practice has been supported by a randomized control trials and EULAR recommendations [2].

**Objectives:** To study long-term disease activity, vaccine-related adverse events in a large cohort of JIA patients who received the MMR booster vaccine while being treated with immune modulatory drug therapy**.**

**Methods:** Clinical and drug therapy data were collected for two visits prior and five visits after the 9^th^ birthday from electronic medical records of JIA patients treated with corticosteroids, synthetic disease-modifying antirheumatic drugs and biologicals since 2007 at the Wilhelmina Children’s Hospital. Frequency of patient-reported adverse events following MMR booster vaccination was determined. The adjusted effect of MMR booster vaccination on MMR antibody levels, the active joint count, physician global assessment of disease activity, patient-reported visual analogue scale (VAS) for disease activity and clinical Juvenile Arthritis Disease Activity Score (cJADAS) was analyzed using linear mixed effects models with a random intercept per patient and a random slope for MMR booster vaccination. Missing values were handled by multiple imputation.

**Results:** Data were collected for 202 patients and 1254 visits. Following MMR booster vaccination, 5 patients (2.5%) reported fever and/or flu like symptoms, 1 patient (0.5%) reported joint pain and 6 patients (3.0%) reported injection site reactions. Disease activity after MMR booster vaccination was not higher than before vaccination (Table 1). Analysis of MMR antibody levels is still in progress.

**Conclusion:** MMR booster vaccination was safe and did not result in worse disease activity in a large cohort of patients with JIA under immune modulatory drug therapy during long-term follow-up. Currently we are performing MMR serology to test the long term immunogenicity of the MMR booster

**References**

1. Heijstek MW, Kamphuis S, Armbrust W, Swart J, Gorter S, de Vries LD, et al. Effects of the Live Attenuated Measles-Mumps-Rubella Booster Vaccination on Disease Activity in Patients With Juvenile Idiopathic Arthritis. JAMA. 2013 Jun 19;309(23):2449.

2. Heijstek M, Ott de Bruin L, Borrow R, van der Klis F, Koné-Paut I, Fasth A, et al. Vaccination in paediatric patients with auto-immune rheumatic diseases: A systemic literature review for the European League against Rheumatism evidence-based recommendations. Autoimmunity Reviews. 2011 Dec;11(2):112-22.

**Disclosure of Interest**: None declared

**Table 1 (abstract P225).** See text for description

Analysis^1^	Mean difference after vs. before MMR booster	95% CI
Active joint count	-0.25	-0.47 – -0.04*
Physician global assessment	-0.17	-0.32 – -0.01*
VAS well-being	-0.19	-0.57 – 0.20
clinical JADAS score	-0.58	-1.14 – -0.03*

^1^adjusted for JIA disease duration, drug therapy (time-varying variables), age at JIA onset and JIA subtype (constant variables)

*statistically significant

### P226. Female susceptibility to elevated ALT-levels and high methotrexate intolerance severity scores in juvenile idiopathic arthritis – a cross-sectional study

#### C. Wibrand^1^, N. Kyvsgaard^2^, A. E. Christensen^3^, T. Herlin^1,*^

##### ^1^Department of Pediatrics, Aarhus University Hospital, Aarhus N, ^2^Department of Pediatrics, Goedstrup Hospital, Herning, ^3^Department of Pediatrics, Odense University Hospital, Odense, Denmark

###### **Correspondence:** T. Herlin

**Introduction:** Methotrexate (MTX) plays a key role in the treatment of juvenile idiopathic arthritis (JIA), but MTX-intolerance (including nausea) is a significant clinical challenge, affecting up to 50% of the treated children. It is well established that MTX-treatment might affect the liver in JIA, illustrated by elevated levels of alanine aminotransferase (ALT), and that nausea is a common but unspecific symptom of liver affection.

**Objectives:** Our study aimed to investigate the association between ALT-levels during MTX-treatment and MTX-intolerance in children with JIA.

**Methods:** Children aged 9 years or above, diagnosed with JIA and treated with MTX for at least 6 weeks, were eligible for enrollment. Enrollment period was December 2013 - July 2016. MTX-intolerance was assessed on the date of enrollment using the Methotrexate Intolerance Severity Score (MISS), completed by the children’s parents. The MISS ranges from 0-36 points, and a child was categorized as MTX-intolerant if the MISS total was 6 or above with at least 1 point for a behavioral, anticipatory and/or associative symptom. ALT-levels were determined at the date of enrollment. ALT-levels were categorized as elevated if above the upper normal limit.

**Results:** A total of 121 children were enrolled (82 girls;39 boys). The MISS-questionnaire was not completed for one child, and for two children no ALT-value in proximity to the enrollment date was available, leaving 118 patients for analysis (Table 1). MTX-intolerance was registered in 61%. There was no statistically significant difference between the MTX-intolerant group and the MTX-tolerant group on any of the demographic parameters. Additionally, ALT-levels did not differ between the MTX-intolerant group (mean = 29.8 U/L [95%CI: 21.4-38.1]) and the MTX-tolerant group (mean = 29.4 U/L [95%CI: 19.7-39.2]; t-test p= 0.96). MTX-intolerance was prevalent in around 60% of both boys and girls, and the MTX-intolerant girls and the MTX-intolerant boys did not differ statistically significantly on any of the demographic parameters. Nine (18%) of the MTX-intolerant girls had elevated ALT-levels, compared to none of the MTX-intolerant boys. The MTX-intolerant girls also had higher numerical ALT-values (mean = 33.9 U/L [95%CI: 19.9-47.8]) than the MTX-intolerant boys (mean = 19.4 U/L [95%CI: 15.6-23.1]; t-test p=0.05). Furthermore, the MTX-intolerant girls had a higher MISS (median = 14.0 [IQR: 9.3-17]) than the MTX-intolerant boys (median = 10.0 [IQR: 7.3-12]; Mann-Whitney-U-test; p = 0.009).

**Conclusion:** No overall association between MTX-intolerance and ALT-levels was found. However, our results suggest a female susceptibility to MTX treatment recorded as high MISS and elevated transaminase levels.

**Patient Consent:** Yes, I received consent

**Disclosure of Interest**: None declared

**Table 1 (abstract P226).** See text for description

	All patients	MTX-tolerant	MTX-intolerant	p-value	MTX-intolerant girls	MTX-intolerant boys	p-value
Number of patients	118	46	72	-	50 (62.5%)	22 (57.9%)	-
Girls:boys, n, (% females)	80;38 (68%)	30;16(65%)	50;22 (69%)	0.78			-
Age at enrollment, years	13.4(11.3-15.2)	13.8(11.5-15.4)	13.3(11.1-14.6)	0.34	12.8(11.1-15.3)	13.7(12.2-14.3)	0.76
MTX_o_:MTX_sc_, n	44:74	21:25	23:49	0.19	15:35	8:14	0.80
MTX-dose (mg/m^2^ pr. week)	9.75(9-10.98)	10.1(9.4-10.9)	9.6 (8.6-11)	0.12	9.7(8.4-11.1)	9.6(9.2-10.6)	1.00
MTX treatment duration, days	334 (141.2-756.8)	261.5(141.2-611.8)	335 (142.5-778.5)	0.40	399.5(146.8-938.5)	284 (130.8-602.8)	0.41

Data are expressed as median (interquartile range) unless otherwise specified

### P227. Worldwide evaluation of Clinical Practice Strategies (CLIPS) in Juvenile Inflammatory Rheumatisms (JIR) through the JIR-CLIPS network

#### M. Hofer^1,2^, D. Gorczyca^3^, R. Cimaz^4^, S. Georgin-Lavialle^5^, V. Hentgen^6^, F. Hofer^7^, T. Kallinich^3^, K. Theodoropoulou^1,2^, H. Wittkowski^8^, S. Kamphuis^9^

##### ^1^Pediatric Rheumatology Unit of Western Switzerland, Lausanne University Hospital (CHUV), Lausanne, ^2^Geneva University Hospital, Geneva, Switzerland, ^3^Pediatric Pneumology and Immunology, Charité University Medicine, Berlin, Germany, ^4^†, †, Italy, ^5^Department of Internal Medicine, French reference center for autoinflammatory diseases (CEREMAIA), Tenon Hospital, Paris, ^6^Department of General Pediatrics, French reference center for autoinflammatory diseases (CEREMAIA), Versailles Hospital, Versailles, France, ^7^JIRcohorte, Foundation RES, Etoy, Switzerland, ^8^Pediatric Rheumatology and Immunology, University Hospital Muenster, Muenster, Germany, ^9^Pediatric Rheumatology, Erasmus MC, Sophia’s Children Hospital Rotterdam, Rotterdam, Netherlands

###### **Correspondence:** M. Hofer

**Introduction:** Juvenile Inflammatory Rheumatism (JIR) is a family of rare and chronic diseases that carry the risk of significant morbidity and early mortality. Many affected patients will need lifelong medication. Although evidence or consensus-based recommendations for diagnosis and treatment exist, they are difficult to implement in a real-life setting due to the wide array of (country-specific) medical systems and financial capabilities. Additionally, physicians in many countries need support in decision-making processes for these rare diseases in childhood

**Objectives:** The objectives of this proposal are: 1) to build a network of pediatric and adult rheumatology to foster exchange of clinical experiences, training, and clinical research between European and non-European countries; 2) to assess, based on this network, the diagnostic procedures, treatment algorithms, and outcome measures applied among patients with five medical conditions representative of the large spectrum of JIR-diseases (lupus nephritis, vasculitis, Still’s disease, biological treatments in monogenic periodic fever syndromes, PFAPA); 3) to analyze and compare these clinical practice strategies (CliPS) within and between the different countries.

**Methods:** The JIR-CliPS project was launched in October 2021. Working groups for each of the five medical conditions cited above were formed and online questionnaires for the surveys developed. These include questions on participants’ demographics and local/national constraints as well as specific questions on the diagnosis, therapy, and general care for these five medical conditions. We are now starting a pilot data collection in four countries (Brazil, Ghana, New-Zealand, and Switzerland) to test the questionnaires and the methodology.

**Results:** This exchange of expert experiences will help to better harmonize the CliPS used for the five studied JIR medical conditions and should help to improve the outcome of children with Juvenile Inflammatory Rheumatism worldwide.

**Conclusion:** -

**Patient Consent:** Not applicable (there are no patient data)

**Disclosure of Interest**: None declared

### P228. Persistent hypogammaglobulinemia after rituximab therapy in children

#### S. Höppener^1^, S. Veldkamp^2^, M. De Groot^3^, S. Haitjema^3^, J. Drylewicz^2^, J. J. Boelens^1,4^, C. Lindemans^5^, J. Montfrans^1^, A. Van Royen-Kerkhof^1^, M. Jansen^1^

##### ^1^Pediatric Rheumatology and Immunology, Wilhelmina Children’s Hospital, University Medical Center Utrecht, ^2^Center for Translational Immunology, ^3^Central Diagnostic Laboratory, University Medical Center Utrecht, Utrecht, ^4^Department of Pediatrics, Memorial Sloan Kettering Cancer Center, New York, ^5^Blood and Bone Marrow Transplantation, Princess Máxima Center for Pediatric Oncology, Utrecht, Netherlands

###### **Correspondence:** S. Höppener

**Introduction:** Hypogammaglobulinemia (HG) occurs regularly as a result of rituximab therapy (RTX). Although post-RTX Hypogammaglobulinemia and its associated risk factors have been well defined in adults, research on this complication in children is still scarce.

**Objectives:** This study aims to determine the prevalence, risk factors and clinical consequences of HG in children after RTX therapy.

**Methods:** A retrospective cohort study was conducted at the University Medical Center Utrecht including all patients aged ≤ 18 years treated with RTX between 2000 and 2020. Data including patient characteristics, treatment characteristics, recurrent infections, need for IVIG supplementation, and Immunoglobuline (Ig) levels were collected from the Utrecht Patient Oriented Database and evaluated. Patients were classified as having HG when (1) IgG levels were less then 1.96 standard deviations below the mean of age matched healthy controls, or (2) during IVIG therapy for any indication.

**Results:** 145/171 eligible patients met the inclusion criteria. RTX indications included post-HSCT complications (n=67), autoimmune disease (n=43), haematological malignancies (n=13), kidney disease (n=11), post-solid organ transplantation complication (n=6) and immunodeficiencies with immune dysregulation (n=5). HG post-RTX was observed in 84/116 patients of whom IgG follow-up data was available (72%). Median follow-up after last RTX dose was 2.0 years (IQR 0.7-4.6 years), with 23 patients not having recovered from HG at this time (39%). Persistent HG (>6 months after last RTX dose) was observed in 61/95 patients (64%) and occurred more frequently in children treated due to post-HSCT complications compared to autoimmune disease (OR=8.34, p<0.001). Low baseline IgG, IgA and IgM levels were associated with persistent HG (p=0.002, p=0.005, p=0.024, respectively), while multiple logistic regression identified low baseline IgG levels (p=0.024) and post-HSCT complication as RTX indication (p<0.001) as significant risk factors for persistent HG. 89/145 patients received IVIG supplementation during follow-up. Recurrent infections were observed in 18 patients, which could be directly attributed to RTX-related HG in 7 patients. Twenty-three patients died during follow-up, with 2 deaths due to, or complicated by, proven bacterial infections during persistent HG. Secondary Ig class switch impairment was observed in 2 patients, one of whom died of bacterial meningitis after discontinuing IVIG supplementation against medical advice.

**Conclusion:** In this study, 72% of children developed HG following RTX therapy. This rate is higher than reported rates in adults, and in line with recent paediatric studies. Furthermore, a majority developed persistent HG, for which children post-HSCT and/or with low pre-RTX IgG levels were especially at risk. Children with HG regularly need IVIG supplementation, can suffer from (lethal) recurrent infections and, rarely but importantly, can develop secondary Ig class switch impairment after RTX. Evaluation of pre-RTX Ig levels and close monitoring of Ig levels after treatment are therefore highly recommended.

**Trial registration identifying number:** In this study, 72% of children developed HG following RTX therapy. This rate is higher than reported rates in adults, and in line with recent paediatric studies. Furthermore, a majority developed persistent HG, for which children post-HSCT and/or with low pre-RTX IgG levels were especially at risk. Children with HG regularly need IVIG supplementation, can suffer from (lethal) recurrent infections and, rarely but importantly, can develop secondary Ig class switch impairment after RTX. Evaluation of pre-RTX Ig levels and close monitoring of Ig levels after treatment are therefore highly recommended.

**Disclosure of Interest**: None declared

### P229. Rapid response to pulse methylprednisolone in an adolescent girl with IGG4 disease related orbital mass

#### B. Kasap-Demir

##### Pediatric Nephrology and Rheumatology, İzmir Katip Çelebi University, İzmir, Turkey

###### **Correspondence:** B. Kasap-Demir

**Introduction:** Immunoglobulin G4-related disease (IgG4-RD) is a rare systemic, immune mediated, fibroinflammatory disease caused by monoclonal proliferation of IgG4(+) plasma cells. Orbital involvement is frequent in children. We have reported a case with an orbital mass related to IgG4-RD.

**Objectives: Case:** A 12-year-old girl presented with a swelling in the right eye for the last five months, which was intensified in the last two weeks. In the orbital magnetic resonance, there was a heterogeneous and intensely enhancing mass lesion located in the right orbital superior extraconal area, extending to the medial and lateral extraconal areas, measuring 45x43x20 mm in the widest part. The lesion surrounds the superior rectus muscle and is in contact with the lateral and medial rectus muscles. She was referred to neurosurgery for biopsy, which revealed IgG4 related disease. In her physical examination, she had a huge mass in her right eye. All other systemic findings were normal. Laboratory test revealed normal complete blood count test and urinalysis. Biochemical markers were within normal limits. C3 and C4 were normal; ANA, p-ANCA and c-ANCA were negative. IgG4 level was normal. Abdominal, thyroid and parotid ultrasonography were negative. We treated her with three pulse metylprednisonone (PMP) and the mass retarded dramatically in a few days, but she had ptosis in the . Concomittantly, we have prescribed mycophenolate mofetil and used corticoteroids with tapering doses. However, residual orbital tumor was defined in the control orbital MRI and debulking surgery was performed. As the latest current status, she was on MMF and the lowest dose of corticosteroids.

**Methods:** -

**Results:** -

**Conclusion: Conclusion:** PMP is may be an effective treatment of choice for huge orbital masses associated with IgG4-RD as the first step. Surgery may be another option for cases resistant to medical treatment.

**Trial registration identifying number:** -

**Patient Consent:** Yes, I received consent

**Disclosure of Interest**: None declared

### P230. Preferences of pediatric rheumatologists for tapering biologic dmards in children with Juvenile Idiopathic Arthritis (JIA) – results of a clinical vignette study

#### J. A. van Til^1^, M. M. Kip^1,2^, M. Twilt^3^, E. Schatorjé^4,5^, K. Groothuis-Oudshoorn^1^, S. Warta^1^, G. Currie^3^, D. A. Marshall^3^, J. F. Swart^2,6^, R. S. Yeung^7^, S. M. Benseler^3^, S. J. Vastert^2,6^, N. Wulffraat^2,6^, M. J. IJzerman^1^ on behalf of on behalf of UCAN CAN-DU and UCAN CURE Consortium

##### ^1^University of Twente, Enschede, ^2^University Medical Center Utrecht, Utrecht, Netherlands, ^3^University of Calgary, Calgary, Canada, ^4^Radboud University Medical Center, ^5^St. Maartenskliniek, Nijmegen, ^6^Utrecht University, Utrecht, Netherlands, ^7^University of Toronto, Toronto, Canada

###### **Correspondence:** M. M. Kip

**Introduction:** Conclusive evidence on clinical and biologic determinants for successfully withdrawing (i.e. discontinuing or tapering) biologic DMARDs (bDMARDs) in patients with JIA in remission is lacking. In clinical practice, each decision to withdraw bDMARDs is based on the clinical judgement of individual pediatric rheumatologists.

**Objectives:** To determine the influence of specific disease and clinical characteristics on the timing of the decision of pediatric rheumatologists to withdraw bDMARDs in children with JIA.

**Methods:** A clinical vignette design was used to study predictors of clinician decision making regarding the timing of bDMARD withdrawal. We included the following nine disease and clinical characteristics: time to treatment response with the current bDMARD, rheumatoid factor (RF), flare history, bDMARD treatment failure, joint damage in the current treatment period, uveitis in the current treatment period, spine involvement, temporomandibular joint (TMJ) involvement, and patient/parent preference. An experimental design was used to systematically vary levels of the characteristics over 16 clinical vignettes. These clinical vignettes were distributed in an online survey among pediatric rheumatologists in the Netherlands and Canada. Respondents were first asked about their shortest treatment time after reaching clinical remission (6-18 months in 3 month intervals) in a child without complications. For each vignette, they were asked whether they would taper/stop bDMARDs at that time, or for how long they would continue. Statistical analysis included descriptive statistics, logistic and interval regression analysis.

**Results:** Twenty-nine pediatric rheumatologists responded (response rate 35%) to the survey. Pediatric rheumatologists in Canada continue bDMARDs in an uncomplicated child in remission longer compared to pediatric rheumatologists in the Netherlands (median of 13.5 vs. 9.0 months, p=0.009). Mean treatment time before withdrawing bDMARDs for the clinical vignettes is 11.6 months. Logistic regression analysis shows that the strongest predictors of continuing bDMARDs are: patient/parent preference for continued treatment (OR 4.8; p<0.001), uveitis in the current treatment period (OR 3.7; p<0.001), bDMARD treatment failure (OR 2.2; p=0.017), and RF positive JIA (OR 1.8; p=0.033). Pediatric rheumatologists who indicate a longer treatment time in a child without complications in the clinical vignettes are significantly less likely to continue bDMARDs (OR 0.19; p=0.005). Interval regression analysis showed that, on average, pediatric rheumatologists wait 11.6 months before withdrawing bDMARDs. In a child with RF positive JIA, bDMARDs are withdrawn 4.9 months later than in a child with RF negative JIA. In a child with uveitis in the current treatment period, bDMARDs are withdrawn 3.7 months later than in a child with no history of uveitis.

**Conclusion:** Of the characteristics included in the clinical vignettes, only TMJ involvement had no influence. Pediatric rheumatologists with shorter treatment times in a child without complications are more likely to continue treatment in a child with complications. We aim to integrate these results in a decision support tool for pediatric rheumatologists, and thereby reduce unwanted treatment variation among children with JIA on bDMARDs.

**Patient Consent:** Not applicable (there are no patient data)

**Disclosure of Interest**: None declared

### P231. Comparison of the effectiveness of the structured 3d exercise and the conventional exercise program for scoliosis in children with rheumatic disease

#### E. P. Kısa^1^, E. Tarakcı^2^, G. Leblebici^3^, O. Kasapcopur^4^

##### ^1^Health Science Faculty, Department of Physiotheraphy and Rehabilitation, Biruni University, İstanbul, ^2^Health Science Faculty, Department of Physiotheraphy and Rehabilitation, Istanbul University Cerrahpasa, ^3^Health Science Faculty, Department of Physiotheraphy and Rehabilitation, Medeniyet University, ^4^Cerrahpasa Faculty of Medicine, Department of Pediatric Health and Diseases, Department of Pediatric Rheumatology, Istanbul University Cerrahpasa, Istanbul, Turkey

###### **Correspondence:** E. P. Kısa

**Introduction:** Arthritis is one of the most common chronic diseases in childhood. It is one of the important causes of short and long-term sequelae. Problems such as weakness or pain may occur, especially in the muscles around the trunk. These conditions may lead to abnormal biomechanics in children with rheumatism. Delayed neuromuscular development, muscle weakness, foot problems, ligament laxity and growth disorders are other factors that contribute to changes in the musculoskeletal system (MS). Problems in the MS in children with rheumatism may cause displacement of the center of gravity, deterioration of biomechanics, and muscle imbalance. All these situations can lead to scoliosis, which we often encounter in children with rheumatism. Scoliosis is an important health problem, cosmetic, social and psychological problems related to the deformity that is mostly seen in adolescents. When we look at the literature, there are many recent studies describing various 3 dimension (3D) exercise methods (SEAS, Schroth, Dobomed, BSPTS, Side-shift, Lyon, etc.) effective on scoliosis. However, no study was found in the literature that applied these exercise methods in children with rheumatism.

**Objectives:** In this study, it was aimed to compare the effectiveness of the conventional exercise program against the personalized structured 3D exercises in children with scoliosis with rheumatic disease.

**Methods:** Children randomly divided into 2 groups. Structured 3D scoliosis exercises were taught to the first group (n=25), and conventional physiotherapy exercises (posture exercises and core stabilization exercises) were taught to the second group (n=25). Demographic characteristics were questioned with the “Sociodemographic data form”. Pain were evaluated using the visual analog scale (VAS), and trunk rotation angles (ATR) were evaluated using a scoliometer. Cobb angle was measured using the SubrotoAngleAid application on x-ray, and risser degrees were measured by the physician. Body awaraness was evaluated with Walter Reed Scale (WRS). Quality of life was questioned with Scoliosis Research Society Scale-23 (SRS-23). Statistical analysis of the data was performed using the SPSS 24.0 (Statistical Package for Social Sciences) program. P≤0.05 was considered statistically significant in all analyzes.

**Results:** Of the children participating in the study, 47 were diagnosed with JIA, 2 with scleroderma, and 1 with dermatomyositis. The mean age of the first group was 11.76±2.26, and the second group was 11.40±2.73. There was no significant difference between the groups between age, height, BMI and VAS. Risser was measured as 2.56±1.38 in Group 1 and 2.38±1.20 in Group 2, and there was no difference between the groups. While Cobb angle and ATR improved in both groups, Group 1 showed a more significant improvement compared to Group 2. A positive correlation was found between disease duration, Cobb angle and ATR. Cobb angle and ATR increased as the disease duration increased (r: 0.55, 0.45, p≤0.001, respectively). A significant correlation was found between the decrease in Cobb angle and ATR and WRS (r:0.57, r:0.44, p≤0.001).

**Conclusion:** Cobb angle, rotation angle, and body image components have an important place in determining the severity, progression or treatment method of scoliosis. The results of this study, which we planned to investigate the effectiveness of structured 3D scoliosis exercises, showed that this method is effective in reducing Cobb angle and rotation angle in scoliosis in children with rheumatism, increasing body image and quality of life. Being the first study in this field makes our study valuable.

**Patient Consent:** Yes, I received consent

**Disclosure of Interest**: None declared

### P232. Treat-to-target approach in JIA, SLE and JDM: parent-reported outcomes and treatment satisfaction, first data of the ProKind-rheuma project

#### J. Klotsche^1^, K. Vollbach^2^, S. Eulert^1^, K. Tenbrock^2^, D. Foell^3^, J.-P. Haas^4^, F. Weller-Heinemann^5^, P. Oommen^6^, D. Windschall^7,8^, K. Moenkemoeller^9^, T. Kallinich^10^, M. Hufnagel^11^, I. Foeldvari^12^, T. Hospach^13^, M. Klaas^14^, M. Rühlmann^15^, R. Trauzeddel^16^, C. Schütz^17^, J. Kuemmerle-Deschner^18^, A. Klein^19,20^, K. Minden^1^, G. Horneff^19,20^

##### ^1^Epidemiology, German Rheumatism Research Center Berlin, a Leibniz Institute, Berlin, ^2^Klinik für Kinder- und Jugendmedizin, Universitätsklinikum Aachen, Aachen, ^3^Klinik für Pädiatrische Rheumatologie und Immunologie, Universitätsklinik Münster, Münster, ^4^Zentrum für Schmerztherapie junger Menschen, Deutsches Zentrum für Kinder- und Jugendrheumatologie, Garmisch-Partenkirchen, ^5^Prof-Hess- Kinderklinik, Klinikum Bremen-Mitte, Bremen, ^6^Klinik für Kinder-Onkologie, -Hämatologie und Klin. Immunologie, Bereich Pädiatrische Rheumatologie, Heinrich-Heine-Universität, Düsseldorf, ^7^Clinic of Paediatric and Adolescent Rheumatology, St.-Josef-Stift Sendenhorst, Sendenhorst, ^8^Medical Faculty, Martin- Luther-University Halle-Wittenberg, Halle, ^9^Klinik für Kinder- und Jugendmedizin, Kinderkrankenhaus der Stadt Köln, Köln, ^10^Department of Pediatric Respiratory Medicine, Immunology and Critical Care Medicine, Charité - Universitätsmedizin Berlin, Berlin, ^11^Zentrum für Kinder- und Jugendmed, Universitätsklinik Freiburg, Freiburg, ^12^Kompetenz-Zentrum für Uveiits und Sklerodermie im Kindes- und Jugendalter, Hamburger Zentrum für Kinder- und Jugendrheumatologie, Hamburg, ^13^Zentrum für pädiatrische Rheumatologie, Olgahospital Stuttgart, Stuttgart, ^14^Klinik für Kinderund Jugendmedizin, Vivantes Klinkum Friedrichshain, Berlin, ^15^Kinderarztpraxis, Göttingen, ^16^Fachambulanz Kinderrheumatologie, Helios Klinikum Berlin-Buch, Berlin, ^17^Klinik und Poliklinik für Kinder- und Jugendmedizin, Universitätsklinikum Carl Gustav Carus, Dresden, ^18^Klinik für Universitätsklinik für Kinder- und Jugendmedizin, autoinflammation reference center Tübingen und Abteilung für Pädiatrische Rheumatologie, Universitätsklinikum Tübingen, Tübingen, ^19^Department of Pediatrics, Asklepios Kinderklinik St. Augustin, St. Augustin, ^20^Department of Pediatric and Adolescents Medicine, University Hospital of Cologne, Cologne, Germany

###### **Correspondence:** J. Klotsche

**Introduction:** There is growing evidence that a treat-to-target (t2t) approach can improve the prognosis of juvenile chronic inflammatory rheumatic diseases (IRD). Therefore, this therapeutic strategy is recommended and increasingly implemented for the most common IRD [1,2]. The acceptance and outcomes of a t2t-approach in clinical practice are currently being investigated in the ProKind-Rheuma project of the GKJR.

**Objectives:** To assess outcomes and parent-reported treatment satisfaction in a prospective cohort of children and adolescents with recent-onset IRD.

**Methods:** ProKind-Rheuma is a multicenter prospective non-interventional observational study. Patients with recently diagnosed juvenile idiopathic arthritis (JIA), systemic lupus erythematosus (SLE), and juvenile dermatomyositis (JDM) have been enrolled in 18 centres in Germany since January 2020 and prospectively followed during the first year of treatment. Physician- and parent-reported data are collected up to five times in a standardized way; data from patients with at least one follow-up visit through May 2, 2022, were included.

**Results:** A total of 420 patients included so far, 173 from 18 rheumatology sites with follow-up (FU) were considered for this analysis. Patients with JIA (n=147), SLE (n=16) and JDM (n=10) were enrolled 0.7±1.1 months after diagnosis and followed for 7.5±3.9 months until their last documented FU. Patients showed substantial health improvement over time (cJADAS-10, SLEDAI, Myositis-DAS). Parent-reported outcomes improved significantly (see table).

Almost half of patients were treated with glucocorticoids and 66% with DMARDs.

Most families (83%) were satisfied with the drug treatment (80% of JIA, 93% of SLE, and 100% of JDM patients), with 47% being very satisfied and 15% being extremely satisfied. 82% reported taking medications regularly or as recommended, with higher adherence in patients with JDM (100%) and SLE (93%) than in those with JIA (79%).

57 families (36%) reported medication-related adverse events at last follow-up, of which nausea (43%), stomach pain (41%), and sleep disturbances (21%) were the most common. The most frequently reported adverse events in SLE and JDM were different from those in JIA. In patients with SLE and JDM, weight gain and headache or increased body hair dominated in addition to gastrointestinal complaints.

**Conclusion:** Patients with IRD improve rapidly in their health status during the first months under targeted therapy. Parents are largely satisfied with drug treatment, although adverse events are not uncommon. Satisfaction with therapy and treatment adherence are higher in patients with SLE and JDM.

**Patient Consent:** Yes, I received consent

**Disclosure of Interest**: None declared

**Table 1 (abstract P232).** See text for description

	Baseline			Follow-up		
	JIA	SLE	JDM	JIA	SLE	JDM
Glucocorticoids systemically, %	17	63	60	6	69	60
DMARD, %	33	63	50	64	88	70
Overall well-being (NRS 0-10)	4.0	4.4	6.1	1.9	3.1	2.5
Pain (NRS 0-10)	3.5	3.7	4.8	1.6	2.8	2.2
Fatigue (NRS 0-10)	3.0	4.9	5.4	1.6	3.4	2.5
Coping with illness (NRS 0-10, 0 = very good)	3.5	4.2	5.6	1.3	2.9	1.5
PedsQL 4.0 (total score 0-100)	70.2	62.9	48.7	82.3	76.0	74.1

### P233. Safety of vaccination under canakinumab in patients with autoinflammatory periodic syndromes - interim analysis of the reliance registry

#### J. B. Kuemmerle-Deschner^1^, J. Henes^2^, B. Kortus-Goetze^3^, T. Kallinich^4^, P. T. Oommen^5^, J. Rech^6^, T. Krickau^7^, F. Weller-Heinemann^8^, G. Horneff^9^, A. Janda^10^, I. Foeldvari^11^, F. M. Meier^12,13^, C. Schuetz^14^, F. Dressler^15^, M. Borte^16^, M. Hufnagel^17^, M. Fiene^18^, J. Weber-Arden^19^, N. Blank^20^

##### ^1^Department of Pediatrics, Division of Pediatric Rheumatology, ^2^Joerg.Henes@med.uni-tuebingen.de, University Hospital Tuebingen, Tuebingen, ^3^Division of Nephrology, University of Marburg, Marburg, ^4^Department of Pediatrics, Division of Pulmonology, Immunology and Critical Care Medicine, Charité University Medicine Berlin, Berlin, ^5^Clinic of Pediatric Hematology, Oncology and Clinical Immunology, Heinrich-Heine-University Duesseldorf, Duesseldorf, ^6^Department of Internal Medicine 3 - Rheumatology and Immunology, Friedrich-Alexander University (FAU) Erlangen-Nuernberg and Universitaetsklinikum Erlangen, ^7^Pediatrics, Friedrich-Alexander Universität Erlangen-Nürnberg (FAU), Erlangen, ^8^Prof. Hess Kinderklinik, Klinikum Bremen-Mitte, Bremen, ^9^Asklepios Clinic Sankt Augustin, Sankt Augustin, ^10^Department of Pediatrics, University Hospital Ulm, Ulm, ^11^Centre for Pediatric and Adolescence Rheumatology Hamburg, Hamburg, ^12^Division of Rheumatology, University Hospital Frankfurt, Goethe University, Frankfurt am Main, ^13^Project Group Translational Medicine and Pharmacology TMP, Fraunhofer Institute for Molecular Biology and Applied Ecology IME, Frankfurt am Main, ^14^Pediatrics, Medizinische Fakultaet Carl Gustav Carus, Technische Universitaet Dresden, Dresden, ^15^Division of Pediatric Pneumology, Allergology and Neonatology, Hannover Medical School, Hannover, ^16^ImmunoDeficiencyCenter Leipzig (IDCL), Hospital St. Georg gGmbH Leipzig, Leipzig, ^17^Division of Pediatric Infectious Diseases and Rheumatology, Department of Pediatrics and Adolescent Medicine, University Hospital Medical Center Freiburg, Medical Faculty, University of Freiburg, Freiburg, ^18^Rheumatology Center Greifswald, Greifswald, ^19^NOVARTIS, Nuernberg, ^20^Rheumatology, University Hospital Heidelberg, Heidelberg, Germany

###### **Correspondence:** G. Horneff

**Introduction:** Treatment of autoinflammatory periodic syndromes with the interleukin-1β inhibitor canakinumab (CAN) has been shown to be safe and effective in clinical trials and in practice. Patients are recommended to be vaccinated against common infections (including influenza, covid-19, pneumococcus) while on therapy, as is the general population. It is known from the literature that severe local and systemic inflammatory reactions can occur frequently in patients with immunosuppressive therapy, especially after pneumococcal vaccination [1].

**Objectives:** In this study, in addition to general safety parameters, the safety of the recommended vaccinations in patients with CAPS, FMF, HIDS/MKD and TRAPS under CAN therapy was investigated in clinical practice.

**Methods:** RELIANCE is a prospective, non-interventional observational study in Germany enrolling pediatric (age ≥2 years) and adult patients with a clinically confirmed diagnosis of autoinflammatory periodic syndrome who routinely receive CAN. Efficacy and safety parameters were recorded at baseline and assessed at 6-month intervals.

**Results:** The interim analysis includes data from N=199 patients with autoinflammatory diseases enrolled in the RELIANCE registry between October 2017 and December 2021. The mean age of the overall cohort is 24.4 years (2-79 years; N=104 female patients [53%]) and the median duration of CAN treatment before study entry was 2 years (0-12 years).

During the study, N=87 patients received a total of 130 vaccinations. Vaccination reactions were reported for N=16 patients, and N=8 patients were classified as suspected adverse drug reactions (Table 1). In no case was the vaccination reaction classified as severe.

Covid-19 vaccination was given to N=42 patients (N=6 Comirnaty, N=1 Spikevax, N=36 not reported; 1 patient received 2 different vaccines). Of these, a vaccination reaction was reported for N=6 patients, which were not considered drug-related or classified as severe.

**Conclusion:** The interim data from the RELIANCE study confirm the safety of long-term treatment with canakinumab in the entire study population. Vaccination with CAN therapy also did not reveal any new safety signals beyond known vaccine side effects.

**Disclosure of Interest**: J. B. Kuemmerle-Deschner Grant / Research Support with: Novartis, AbbVie, Sobi, Consultant with: Novartis, AbbVie, Sobi, J. Henes Grant / Research Support with: Novartis, Roche, Consultant with: Novartis, AbbVie, Sobi, Roche, Janssen, Boehringer-Ingelheim, B. Kortus-Goetze Consultant with: Novartis, T. Kallinich Speaker Bureau with: Roche, P. T. Oommen Grant / Research Support with: Novartis, J. Rech Grant / Research Support with: Novartis, Sobi, Consultant with: AbbVie, Biogen, BMS, Chugai, GSK, Janssen, Lilly, MSD; Mylan, Novartis, Roche, Sanofi, Sobi, UCB, Speaker Bureau with: AbbVie, Biogen, BMS, Chugai, GSK, Janssen, Lilly, MSD; Mylan, Novartis, Roche, Sanofi, Sobi, UCB, T. Krickau Grant / Research Support with: Novartis, Consultant with: Novartis, Speaker Bureau with: Novartis, F. Weller-Heinemann: None declared, G. Horneff Grant / Research Support with: AbbVie, Chugai, Merck Sharp & Dohme, Novartis, Pfizer, Roche, Speaker Bureau with: AbbVie, Bayer, Chugai, Merck Sharp & Dohme, Novartis, Pfizer, Roche, A. Janda: None declared, I. Foeldvari Consultant with: Novartis, F. M. Meier Speaker Bureau with: Novartis, C. Schuetz: None declared, F. Dressler Grant / Research Support with: Novartis, Consultant with: Abbvie, Mylan, Novartis, Pfizer, M. Borte Grant / Research Support with: Pfizer, Shire, M. Hufnagel Grant / Research Support with: Novartis, M. Fiene: None declared, J. Weber-Arden Employee with: Novartis, N. Blank Grant / Research Support with: Novartis, Sobi, Consultant with: Novartis, Sobi, Lilly, Pfizer, Abbvie, BMS, MSD, Actelion, UCB, Boehringer-Ingelheim, Roche

**Table 1 (abstract P233).** Overview of vaccinations and vaccination side effects in the RELIANCE study across all study indications (N=199 patients)

Vaccinations during the study	Number of patients	No vaccination reactions	Vaccination reaction occurred	Probably drug- related	Vaccination reaction unknown
**Covid-19**	42*	27	6	0	10
**Tetanus, diphtheria, pertussis**	8	4	2	0	2
**Influenza**	46*	38	5	5	15
**Pneumococcus**	3	2	0	0	1
**Meningococcus**	5	2	1	1	2
**Other**	9	7	2	2	0

*For patients under recurrent vaccination who were experiencing different types of vaccination reaction, each type of reaction was counted separately

### P234. Uveitis as predictive factors of relapse after TNF-inhibitor withdrawal in a cohort of JIA

#### I. Maccora, V. Accardo, E. Marrani, M. V. Mastrolia, I. Pagnini, G. Simonini

##### ^1^Rheumatology Unit, Meyer Children’s University Hospital, NeuroFARBA Department, University of FLorence, Florence, Italy

###### **Correspondence:** I. Maccora

**Introduction:** TNF-inhibitors (TNFi) have radically modified the prognosis of Juvenile Idiopathic arthritis (JIA). However, when, and how discontinuing the treatment is still a challenge.

**Objectives:** The purpose of this study is to describe a cohort of patients with JIA treated with TNFi (Adalimumab (ADA) and Etanercept (ETA)) in whom these drugs were discontinued due to persistent remission on medications.

**Methods:** This is a monocentric retrospective study based on chart review conducted at the Rheumatology Unit of Meyer Hospital, Florence. The study involved patients with a diagnosis of polyarticular and oligoarticular JIA according to ILAR criteria treated with a first course of ADA or ETA, that were then discontinued for persistent remission. We collected demographic, clinical and laboratory data at onset and at biologic starting. Relapse was entered when a JIA children flared after stopping medication due to arthritis, due to uveitis or both. We recorded the time between the onset of symptoms and diagnosis, diagnosis and start of biologics, start of biologics and the achievement of inactivity, presence of flare, duration of treatment, of disease inactivity, presence of relapse after withdrawal, time intercourse between withdrawal and relapse, and discontinuation modality (distinguishing increase of interval of administration, reduction of dose and abrupt discontinuation). Statistical Analysis were performed using SPSS v27

**Results:** 62 patients were enrolled (54 female), the 51.6% had an oligoarticular JIA and 48.4% polyarticular, 41.9% had uveitis, and 85.5% had ANA positivity. The median age at onset was 3 years old (R 1-11y), with a median value of ESR of 39 mm/h (R 2-100), of CRP 1.34 mg/dl (0.23-9.1) and of JADAS10 16.5 (R 6.4-34). The first course of the TNFi inhibitors was started after a median time of disease of 15 months (R 2-93m) and at a median age of 6 years (R 1-13 y). The 61.3% of patients (38) were treated with ADA, while the 38.7% (24) with ETA, of whom the 79% (49) were treated with a concomitant therapy of Methotrexate and the 14.5% (9) with corticosteroids. All the patients achieved the disease remission after median time of 4 months (R 1-32). The last item that achieved remission was arthritis in 79% (49) of patients, uveitis in 14.5% (9) and both in 6.5% (4). The therapy was discontinued after a median time of treatment of 30 months (R 7-62) and a median time of inactivity of 22 months (R 9-60). TNFi were stopped in the 56.5% increasing the interval of administration, in the 38.7% reducing the dose, and in the 19.4% abrupt discontinuation. The 80.6% (50) of patients relapsed after a median time of 5.5 months (R 0.5-96), because of arthritis in 64% (32) of patients, uveitis in 22% (11), both in 14% (7). We observed that patients who relapsed had significantly more frequently a positive anamnesis for uveitis (χ^2^ 3.9, p 0.04) and were younger when the TNFi were started (5.84 vs 7.67, p 0.021), while no difference were evaluated in the modality of suspension. The chance to relapse after TNF withdrawal resulted significant higher in JIA children with a history of uveitis (Log Rank χ^2^ = 5.54 p = 0.019).

**Conclusion:** The presence of uveitis seems to be significantly more frequent in subjects who relapsed, children with an history of uveitis had a shorter period of remission out of medication.

**Patient Consent:** Not applicable (there are no patient data)

**Disclosure of Interest**: None declared

### P235. What matters most to pediatric rheumatologists in deciding whether to withdraw biologics in a child with juvenile idiopathic arthritis: a best-worst scaling study

#### G. R. Currie^1^, K. Groothuis-Oudshoorn^2^, M. Twilt^1^, M. M. Kip^3^, M. J. IJzerman^3^, S. M. Benseler^1^, J. F. Swart^4^, S. J. Vastert^4^, N. M. Wulffraat^4^, R. S. Yeung^5^, D. A. Marshall^1^ on behalf of on behalf of UCAN CAN-DU and UCAN CURE Consortium

##### ^1^University of Calgary, Calgary, ^2^University of Twente, Enschede, Canada, ^3^University of Twente, Enschede, ^4^University Medical Centre Utrecht, Utrecht, Netherlands, ^5^University of Toronto, Toronto, Canada

###### **Correspondence:** M. M. Kip

**Introduction:** The care of children with JIA has significantly been transformed in the biologics era, however, these medications carry important (although rare) risks and are costly. There is little clinical guidance for physicians to identify which patients in clinical remission can safely have their biologic withdrawn (by stopping or tapering). Therefore we examined what characteristics of the child or their context are important to pediatric rheumatologists when making the decision to discuss the possibility of withdrawal of biologicals in JIA.

**Objectives:** To identify what characteristics of the child, or their context, are important to pediatric rheumatologists when making the decision to discuss the possibility of withdrawal of biologics in JIA.

**Methods:** We conducted a survey including a best-worst scaling (BWS) exercise in pediatric rheumatologists in Canada and the Netherlands to assess the relative importance of 14 previously identified characteristics of children or their health care context. A balanced incomplete block design was used to generate choice sets, and respondents were asked to evaluate 14 sets of 5 characteristics of a child with JIA, and identify which among each set was most and least important in their decision to offer withdrawal. Results were analysed using conditional logit estimation and expressed as odd-ratios.

**Results:** Fifty-one pediatric rheumatologists participated, with an overall response rate of 65%. The most important three characteristics were how challenging it had been to achieve remission, a history of established joint damage and the time spent in disease remission. The least important three characteristics in the opinion of pediatric rheumatologists for this issue were history of temporomandibular joint involvement, accessibility of biologics and the patient’s age.

**Conclusion:** These findings give quantitative insight into the factors that are important to the decision making of pediatric rheumatologists about biologic withdrawal, an area where there is currently little clinical guidance. In addition to high quality clinical evidence, further research is needed to understand the perspective of patients and families to help inform shared decision-making about withdrawal of biologics in JIA patients with clinically inactive disease.

**Patient Consent:** Not applicable (there are no patient data)

**Disclosure of Interest**: None declared

### P236. Strategic use of levofolinic acid for methotrexate-induced side effects in juvenile idiopathic arthritis: a prospective observational study

#### G. Martini, A. Meneghel, M. Fastiggi, F. Dell’Apa, F. Tirelli, F. Vittadello, F. Zulian

##### Department of Pediatrics, University Of Padova, Padova, Italy

###### **Correspondence:** G. Martini

**Introduction:** Low-dose methotrexate (MTX) is a pivotal therapy in Juvenile Idiopathic Arthritis (JIA) and in other autoimmune diseases. Around 50% of treated patients report side effects, mainly gastrointestinal symptoms, such as nausea and vomiting. These symptoms can occur after drug administration or even be anticipatory thus suggesting a psychological origin in these cases. Different methods have been suggested to reduce side effects such as use of anti-emetics, folic and levofolinic acid (LVF) but strategies are highly variable from centre to centre.

**Objectives:** In our centre all patients receiving MTX take a single dose of LVF 48 hours after MTX administration but, despite this, some of them still report gastrointestinal discomfort. Our aim was to evaluate the efficacy of a supplemental dose of LVF, administered 48 hours before MTX, in reducing side effects without interference with drug efficacy

**Methods:** A prospective observational study was performed including consecutive JIA patients reporting significant gastrointestinal side effects or discomfort post-MTX and excluding patients with anticipatory symptoms. At study entry (T0) patients were suggested to take a supplemental dose of LVF 48 hours before MTX administration and were followed every 3-4 months (T1 to T5). At each visit the following data were collected: efficacy of LVF on gastrointestinal symptoms, disease activity (JADAS, ESR, CRP values) and treatment (MTX reduction/increase/stop and biological agents start/reduction/increase)

**Results:** 21 patients were recruited: 15 oligoarticular (12 ANA+), 5 polyarticular (1 RF+) and 1 enthesitis-related arthritis (ERA). At study entry (T0) mean MTX treatment duration was 40 months (median 22, range 2-185 months) and 7 patients also received a biological agent (5 adalimumab, 2 tocilizumab). In all patients, MTX was administered subcutaneously (mean dose 9.54 mg/m2) and was associated with LVF 48 hours later (mean 6.5 mg, range 2.5-7.5 mg). Side effects were nausea in 14 patients, vomiting in 5, nausea and diarrhoea (1) and vomiting and diarrhoea (1); 7 patients used anti-emetics (ondansetron). All patients were followed for at least 1 year (range 12- 29 months). Complete remission of the side effects was reported in 13/21 at T1, in 18/21 patients at T2, in 20/21 at T3, in 12/14 at T4, in 7/7 at T5).

Friedman test for repeated measures from study start to T4 demonstrated statistically significant differences for JADAS and CRP values (p=0.006 and 0.008 respectively). Over the observation period, two patients started adalimumab (1 for disease relapse after SARS-CoV2 infection, 1 for persistent disease activity), in all remaining patients MTX dose was tapered and in 8 of them it was withdrawn for remission.

**Conclusion:** Our study suggests that LVF administered 48 hours before MTX is effective in reducing MTX-induced gastrointestinal side effects. Furthermore, although MTX mechanism of action is to target folate pathway, increasing doses of LVF were not associated with a decreased efficacy of the drug. Taken together our results suggest that this strategic use of LVF may improve patients’ compliance and quality of life.

**Disclosure of Interest**: None declared

**Table 1 (abstract P236).** See text for description

	T0	T1	T2	T3	T4	T5
**JADAS**	3.3	2.3	1.5	1.0	1.6	2.0
**ESR** (mm/h)	7.6	7.4	4.3	6.7	5.9	8.0
**CRP** (mg/L)	2.5	2.9	1.5	2.0	1.5	2.1

### P237. Use of intravenous iloprost for the treatment of digital ulcers in juvenile systemic sclerosis: a pediatric clinical case

#### G. Nardini^1^, C. Borzacchiello^1^, E. Coppola^1^, L. Martemucci^2^, F. Orlando^2^, M. Tardi^2^

##### ^1^Department of Translational Medical Sciences, Pediatrics, Federico II University, ^2^Department of Pediatrics, AORN Santobono Pausilipon, Naples, Italy

###### **Correspondence:** G. Nardini

**Introduction:** Juvenile Systemic Sclerosis (JSSc) is a rare connective tissue disease which affects skin, blood vessels, heart, lungs, kidneys, gastrointestinal tract and musculoskeletal system. Skin involvement has different phases: edematous, fibrotic, and eventually atrophic. In all phases there are: reduction of peripheral perfusion and risk of Digital Ulcers (DU). The management of this complication in adult population consists of different pharmacological options such as Calcium antagonists, Phosphodiesterase 5 inhibitors, Prostanoids and Endothelin 1 receptor antagonists, until even surgery.

**Objectives:** The objective of this case report is to present a case of pediatric JSSc with DU successfully treated with intravenous Iloprost.

**Methods:** We present the case of M., 4 years old, female, in apparent good health, who came to our attention for bilateral acute necrosis of foot fifth finger. At clinical examination on admission, DU with advanced necrotic evolution of both fifth fingers of feet, livedo reticularis, hardening and xerosis of the skin (especially on the face and extremities of inferior limbs), rhinolalia, thin hair, several necrotic areas (earcup, scalp) and right knee arthritis were observed. Blood tests showed a positive C Reactive Protein (22,43 mg/L, normal value <5), severe hypernatremia and hyperchloremia (Na + 165 mMol/L, Cl- 133 mMol/L) and hypoalbuminemia. At abdominal ultrasound hepatomegaly and mild left kidney hypotrophy were shown. Chest CT scan was performed and showed atelectasis areas in superior and medium right lobe. Inferior limbs angio CT scan showed normal vascularization. An onset of JSSc was hypothesized, so M., after performing a bone marrow examination, started pulse intravenous methylprednisolone therapy (30 mg/kg/die), followed by daily intravenous dose of 2 mg/kg and subcutaneous methotrexate (15 mg/mq/wk).

**Results:** For DU M. performed a cycle of five infusions of intravenous Iloprost (2 ng/kg/min), subsequently shifted to oral Nifedipine. Clinical response was excellent with progressive improvement of most ulcerative lesions until almost complete healing and no new lesions. Nevertheless, necrosis of fifth finger of the left foot persisted, so surgical curettage was performed with good clinical and esthetic result.

**Conclusion:** Iloprost is an analogous of Prostacyclin (PGI2), which inhibits platelet aggregation and adhesion, dilates arterioles and venules, actives fibrinolysis and reduces the release of oxygen free radicals. There is evidence of effectiveness of intravenous Iloprost in treatment of DU in adult patients with Systemic Sclerosis. So far, poor data about its use in pediatric population are available. We present a pediatric case of a patient with DU in JSSc successfully treated with intravenous Iloprost.

**Patient Consent:** Yes, I received consent

**Disclosure of Interest**: None declared

### P238. Strategy for stopping tnf inhibitor in juvenile idiopathic arthritis does not affect effectiveness after re-treatment

#### S. S. Pelk, M. J. Doeleman, J. F. Swart, S. de Roock

##### Department of Pediatric Immunology and Rheumatology, Wilhelmina Children’s Hospital, Utrecht, Netherlands

###### **Correspondence:** S. S. Pelk

**Introduction:** Discontinuation and re-treatment with tumor necrosis factor inhibitors (TNFi) is common during the treatment course of children diagnosed with JIA. These medications are either gradually tapered over a longer period by increasing treatment intervals (tapering) or immediately discontinued [1]. Although there is some evidence that re-treatment with TNFi after withdrawal remains effective [2], there is no scientific consensus on the association of withdrawal strategy (taper or immediate discontinuation) with response to re-treatment with TNFi.

**Objectives:** To determine if tapering or immediate discontinuation of TNFi affects drug efficacy after re-treatment.

**Methods:** This is a monocentric retrospective cohort study of patients diagnosed with JIA, re-treated with adalimumab, etanercept, or golimumab during or after withdrawal. Data was collected from the Wilhelmina Children’s Hospital. Patient characteristics were compared between patients where TNFi treatment was tapered or immediately discontinued. Adequate response to re-treatment was defined as an active joint count (AJC) equal to 0 after approximately 6 months. Nonresponse was defined as a switch to another biological agent or the inability to achieve an AJC equal to zero at the 6 month visit. Association of withdrawal strategy prior to re-treatment and response to re-treatment with TNFi was analyzed using multiple logistic regression, adjusted for sex, age, AJC at re-treatment, and length of TNFi-free period prior to re-treatment.

**Results:** 55 patients were included in this study, 28 patients tapered before re-treatment and 27 patients discontinued immediately. Patient characteristics of the two groups are presented in Table 1. Multiple logistic regression demonstrated no significant association of withdrawal strategy with response to re-treatment (taper vs. immediate discontinuation OR 1.62, 95% CI 0.35-7.57). The length of TNFi-free period prior to re-treatment (in months) was significantly associated with the probability of response to re-treatment (OR 0.91 95% CI 0.84-1.00).

**Conclusion:** Our data suggest that tapering or immediate discontinuation was not significantly associated with the probability of response to re-treatment with TNFi. The length of TNFi-free period prior to re-treatment was negatively associated with the probability of response. Nevertheless, our results are limited by a small sample size. Future efforts will focus on increasing our sample size and investigation of other patient and treatment characteristics that could influence the efficacy of re-treatment, including the development of anti-drug antibodies and TNFi dosage.

**References:**

1. Halyabar O, Mehta ·J, Ringold S, et al. Treatment withdrawal following remission in juvenile idiopathic arthritis: A systematic review of the literature. doi: 10.1007/s40272-019-00362-6.

2. Klotsche, J., Klein, A., Niewerth, M. et al. Re-treatment with etanercept is as effective as the initial firstline treatment in patients with juvenile idiopathic arthritis. Arthritis Res Ther 23, 118 (2021). doi: 10.1186/s13075-021-02492-0

**Patient Consent:** Yes, I received consent

**Disclosure of Interest**: None declared

**Table 1 (abstract P238).** Characteristics and results of the included JIA patients

	Immediate stop, N =27^**1**^	Taper, N = 28^**1**^	p-value^**2**^
Response	20 (74%)	23 (82%)	0.5
Sex, Female	17 (63%)	14 (50%)	0.3
Age (years)	10.0 (7.9, 13.9)	14.0 (10.7, 16.7)	0.5
TNFi-free period before re-treatment (months)	6.2 (4.0, 8.9)	5.1 (2.4, 12.4)	0.020
Active Joint Count	3.0 (1.0, 5.50)	1.0 (0.8, 2.2)	0.012

^1^ n (%); Median (IQR). TNFi = trumor necrosis factor inhibitor

^2^ Pearson’s Chi-squared test for categorical data; Wilcoxon rank sum test for numeric data

### P239. What after the first biologic agent in JIA: preliminary data

#### M. R. Pellico^1^, E. A. Conti^2^, F. Minoia^3^, G. Filocamo^3^, M. V. Gattinara^4^, C. B. Chighizola^4^, R. Caporali^4^, A. Marino^4^ on behalf of on behalf of the Pediatric Rheumatology Associated Group of the Milan Area (PRAGMA)

##### ^1^Reumatolgy Department, ^2^Pediatrics Department, University of Milan, ^3^Pediatric Rheumatology, Fondazione IRCCS Ca’ Granda Ospedale Maggiore Policlinico, ^4^Unit of Pediatric Rheumatology, ASST Pini-CTO, Milan, Italy

###### **Correspondence:** M. R. Pellico

**Introduction:** Biologic agents (bDMARDs) have dramatically changed the disease course of juvenile idiopathic arthritis (JIA). Nowadays, TNF-α inhibitors (TNFi) are frequently used as first-line treatment, while there are no clear recommendations on the choice of the second bDMARDs. Indeed, little is known about the prescription pattern and efficacy of second bDMARDs in JIA patients.

**Objectives:** To describe the prescription pattern of the second bDMARDs in a cohort of non-systemic JIA patients and to report data on the efficacy and safety of TNFi vs. non-TNFi biologic agents as second-line bDMARDs.

**Methods:** This retrospective cohort study included non-systemic JIA patients treated with at least two bDMARDs followed in two Rheumatology Pediatric tertiary centers in Milan, Italy. Demographic and clinical data were collected from medical charts. A descriptive analysis was then undertaken.

**Results:** The study cohort included 30 patients (3 males); the mean age at disease onset and at diagnosis were 5 ± 4.9 (1-15) years and 5.3 ± 4,96 (1-15) years, respectively. Of the 30 patients, 6 (20%) had a diagnosis of polyarticular JIA, 19 (63%) of oligoarticular JIA, 3 (10%) of enthesitis-related arthritis and 2 (6%) of psoriatic arthritis. Fourteen patients (47%) had a history of uveitis (all with oligoarticular JIA). 87% of the patients received methotrexate (MTX) before the introduction of bDMARDs and 90% received TNFi as the first bDMARDs, etanercept and adalimumab being the most prescribed (13 and 12 patients, respectively).

The mean age at the first bDMARDs was 7.3 ± 5.1 (1-17.3) years. Most of the patients (87%) received the first bDMARDs in combination with MTX. The causes of the first bDMARDs discontinuation were the following: articular flare (16 patients), uveitis flare (7 patients), both articular and uveitis flare (1 patient), and adverse events (6 patients). Three out of 13 patients treated with etanercept as the first bDMARDs withdrawn it because of uveitis occurrence.

Adalimumab was the most prescribed second bDMARDs (13; 43.3%), followed by infliximab (7; 23.3%), abatacept (5; 16.7 %), etanercept (3; 10%) and tocilizumab (2; 6.7%).

The most frequent switch was from etanercept to adalimumab (11 times). The mean age at the second bDMARDs start was 10.8 ± 5 (3-19) years. 63.3% of the patients received a second bDMARDs in combination with MTX. A non-TNFi was prescribed as second bDMARDs in 7 (23.3%) patients: 3 oligoarticular JIA and 4 polyarticular JIA. Comparison between TNFi vs. non-TNFi groups is shown in Table 1. Three patients of the non-TNFi group withdrawn the second bDMARDs due to articular disease. Among the TNFi group, 6 patients discontinued the second bDMARDs due to articular disease, 4 for uveitis, 1 for both articular and ocular disease, and 2 because of adverse events.

**Conclusion:** Most of the patients in our cohort received a TNFi as second bDMARDs, with adalimumab being the most prescribed. No clear differences were seen between the non-TNFi and TNFi as for achievement of clinical inactive disease, rate of discontinuation, and duration of the second bDMARDs. Further studies are needed to explore the choice of the second bDMARDs in JIA.

**Disclosure of Interest**: None declared

**Table 1 (abstract P239).** See text for description

	Non-TNFi (n=7)	TNFi (n=23)
Age at II bDMARDs, years	10.7 ± 4.9 (5.3-19.4)	10.9 ± 5.2 (0.2-19)
Clinical inactive disease	4 (57%)	14 (61%)
II bDMARDs discontinuations	3 (43%)	13 (56%)
Duration of II bDMARDs, years	2.7 ± 3.1 (0.15-8.8)	2.6 ± 2.3 (0.2-9)

### P240. Sirolimus in a case of generalized lymphatic anomaly

#### A. Prabhudesai^1^, A. Khan^1^, H. Panwala^2^, N. P. Maldar^1^, R. Khubchandani^1^

##### ^1^Pediatric Rheumatology, ^2^Pediatric Radiology, NH SRCC Children’s Hospital, Mumbai, India

###### **Correspondence:** A. Prabhudesai

**Introduction:** Generalized Lymphatic anomaly (GLA) is a rare, congenital, non-neoplastic condition characterized by abnormal proliferation of lymphatics. It is multisystemic with lungs and bones being most commonly involved. Thoracic involvement in children is associated with higher morbidity and mortality. With no evidence-based treatment protocol, emerging data suggests mammalian target of rapamycin (mTOR) inhibitor, Sirolimus, as a viable treatment option. mTOR,a serine/threonine kinase in the PI3K/AKT pathway governs cellular growth and angiogenesis. By inhibiting mTOR, Sirolimus reduces disease burden by causing partial or complete regression in bony disease, pericardial and pleural effusions.

**Objectives:** Due to recurrent/ persistent symptomatology, it mimics rheumatological illnesses with polyserositis. Also, as sirolimus and other immunomodulators are common drugs in the rheumatologist/immunologist’s repertoire, rheumatologists are likely to be referred such patients for management. We describe a child with GLA with predominant thoracic disease referred as a diagnostic dilemma. She was diagnosed due to classic radiological features and showed improvement after 1 year of treatment with Sirolimus.

**Methods:** An 11-year-old girl of average built with recurrent pleuropericardial effusions in the last 4 years, presented with severe respiratory distress, O2 saturation of 88% in room air and an intercostal tube in situ draining a massive right sided pleural effusion. The fluid was milky, exudative, with high triglycerides. Laboratory results showed hemoglobin 11g/L, total leucocyte count 910^9^/L, absolute lymphocyte count 2.210^9^/L and platelet count 2210^9^/L. The serum creatinine was 0.3 mg/dl with normal electrolytes and serum albumin of 3.7g/l. Spirometry showed moderate restriction. Whole body MRI showed diffuse confluent sheet like lesions in the anterior and middle mediastinum, extending superiorly up to the neck with bilateral peribronchovascular and interlobular septal thickening. Multiple hyperintense lesions were seen in the spleen and vertebral bodies, pointing towards a multisystem involvement. The characteristic radiology coupled with the clinical features led to a diagnosis of GLA. After parental counselling and informed consent, treatment with Sirolimus was initiated at 0.8mg/m^2^and doses adjusted to maintain trough levels between 10-15ng/ml. Lipid levels, blood counts and liver function were tested at baseline and monitored monthly. After 1 year of treatment with Sirolimus, the patient reported no exertional dyspnoea, no hospitalizations and improved lung function reflecting in the spirometry by a significant increase in the FEV1 and FVC. MRI showed partial reduction in the diffuse mediastinal infiltration and peribronchovascular and interlobular septal thickening. There was significant regression in the splenic and vertebral lesions. There was no major side effect of sirolimus except for hyperlipidemia with triglycerides 201mg/dl and cholesterol 249mg/dl, which was managed with statins. Sirolimus thus helped provide symptomatic relief and stabilized the disease in our patient without any major adverse effects.

**Results:**

Pre and post treatment radiological images shall be demonstrated in the poster

**Conclusion:** GLA is a rare multisystemic condition which may present as a mimic to a rheumatologist. Alternately the rheumatologist may be called upon to opine on its treatment with sirolimus. Although Sirolimus is emerging as an effective and well tolerated option helping to stabilize and reduce the disease burden, hyperlipidemia may be encountered.

**Patient Consent:** Yes, I received consent

**Disclosure of Interest**: None declared

**Table 1 (abstract P240).** See text for description

Spirometry	Pre treatment	1 Year of treatment
FEV1 (L)	0.92 (46%)	1.49 (65%)
FVC (L)	0.98 (46%)	1.50 (61%)
FEV1/FVC (%)	93.88	99.33
SpO2 in room air	88%	99%
6minute walk test	Unable to perform	Distance- 486.7m

### P241. Overview of anakinra therapy in multisystemic inflammatory syndrome in children associated with COVID-19 (MIS-C) from tertiary pediatric rheumatology clinics perspective

#### S. caglayan^1^, H. E. Sonmez^2^, G. Otar Yener^3^, E. Baglan^4^, K. Ozturk^5^, K. Ulu^1^, V. Guliyeva^6^, S. Ozdel^4^, H. Bukulmez^7^, N. Aktay Ayaz^6^, B. Sozeri^1^

##### ^1^Pediatric Rheumatology, University of Health sciences, umraniye education and research hospital, Istanbul, ^2^Kocaeli University, Kocaeli, ^3^Şanlıurfa Research and Training Hospital, Sanlıurfa, ^4^Sami Ulus Research and Training Hospital, Ankara, ^5^Istanbul Medeniyet University, Göztepe Research and Training Hospital, ^6^Istanbul University, Faculty of Medicine, Istanbul, Turkey, ^7^Case Western Reserve University, Cleveland, United States

###### **Correspondence:** B. Sozeri

**Introduction:** Multisystemic inflammatory syndrome in children (MIS-C) is a hyperinflammatory condition that has recently entered our lives after severe acute respiratory syndrome coronavirus 2 (SARS-CoV-2) infection and presents with Kawasaki disease (KD) and/or shock-like findings. MIS-C is a rare complication of COVID-19 with an incidence of 0.4% . A total of 6851 MIS-C cases have been reported and 59 deaths have been observed since May 2020. The underlying pathogenesis has not yet been fully elucidated and an abnormal immune response is blamed as the main factor in the pathogenesis of MIS-C

**Objectives:** The study aimed to report the efficacy and safety of anakinra treatment in patients with refractory multisystemic inflammatory syndrome in children (MIS-C).

**Methods:** This is a cross-sectional study consisting of pediatric patients diagnosed with MIS-C who were treated with anakinra.

**Results:** Eighty-two (48 male/34 female) patients with MIS-C were evaluated. The median age of patients was 115 (6-214) months. The median duration of hospitalization was 15 (6-42) days. Sixty patients (73.1%) were admitted to pediatric intensive care unit. Patients were treated with a median dose of 2.7 mg/kg/day anakinra concomitant with IVIG and steroids. Intravenous anakinra was applied to 12 patients while 70 patients received it subcutaneously. Twenty-eight patients required high dose (4-10 mg/kg/day) anakinra. Anakinra was ceased within a median of 7 days. No injection site reactions were observed while elevated transaminase levels were detected in 13 patients. Seventy-three patients (89.1%) were discharged without any sequela or morbidity. Seven patients (8.5%) died. Abnormal echocardiographic findings continued in two patients (2.4%) (coronary artery dilatation in one, low ejection fraction in one) at discharge and became normal on the 2^nd^ month.

**Conclusion:** MIS-C patients should be evaluated and treated dynamically due to its severe and potentially fatal course. In refractory MIS-C cases, anakinra provides significant improvement in both clinical and laboratory findings. The use of anakinra in refractory MIS-C cases is safe even at high doses.

**Disclosure of Interest**: None declared

### P242. A single center experience in biological therapy for juvenile idiopathic arthritis in Bulgaria

#### K. Temelkova, M. Ganeva, A. Teltcharova, V. Kostova, T. Vasilev, S. Stefanov

##### ^1^Department of Pediatric Rheumatology and Cardiology, University Children’s Hospital, Sofia, Bulgaria

###### **Correspondence:** M. Ganeva

**Introduction:** Biological therapy is indicated in the treatment of Juvenile Idiopathic arthritis (JIA) after failure of disease-modifying anti-rheumatic drugs (DMARDS) by the ACR/EULAR recommendations. Despite the availability of wide range of EMA approved biological agents (BA) for JIA, only three of them are allowed to be used in Bulgaria -Adalimumab, Etanercept and Tocilizumab.

**Objectives:** The objective of the study is to describe the characteristics of Bulgarian patients with JIA at the initiation of biological therapy.

**Methods:** This is a retrospective study of JIA patients treated with biological agents in University Children’s Hospital in Sofia, for a period of 12 years(2009 – 2021). Data was collected from the medical records of all JIA patients on biological therapy including: demographics, disease characteristics and concomitant therapy.

**Results:** Тhree hundred and eighty-four patients were included in the study, of whom 237 (61%) were females .The average age of diagnosis of the patients was 6,5 years and the average age of initiation of biological therapy was 9,9 years. In one hundred and sixty-nine (44%) children polyarthritis was identified, making it the most common subtype of JIA. Oligoarthritis was found in 150 (39%) patients, predominantly in girls with Male:Female ratio 1:2. Thirty-eight (10%) children had systemic JIA (sJIA) and 27 (7%) had enthesitis related arthritis (ERA), with M:F ratios being 1,3:1 and 3,3:1 respectively. The average number of affected joints was 3.1, with maximum count of 13 joints and the most commonly involved were knee joints, followed by ankles and wrists. Rheumatoid factor (RF) was positive in 14 (3.6 %) patients and HLA B27 positivity was found in 41 (10.6 %) children. Antinuclear antibodies were detected in 101 (26 %) children with four times higher frequency in girls. Eye involvement was observed in 37 (9, 6 %) JIA patients with female predominance (21 girls to 16 boys) and 67 % of them were treated with Adalimumab. All of the children were treated with DMARDS - Methotrexate or Sulfasalazine, and all of them were also on concomitant corticosteroid (CS) therapy . Intraarticular CS injections were performed in 41 % of the patients during the disease course. The use of BA made possible tapering and discontinuation of the systemic CS therapy in 44 % of the children. TNF- inhibition was performed in 280 (73 %) of the patients and IL- 6 inhibition in 104 (27 %) patients. Most commonly used BA was Adalimumab (38 %) followed by Etanercept in 35 % and Tocilizumab in 27 % of JIA patients. As some of the JIA patients could not achieve remission despite using the prescribed biologicals, BA switching was required in 59 (15 %)children. The most common reason for BA switching was inadequate response (69 %).In 16 ( 28 %) patients eye involvement was observed during the disease course and was the reason for switching between different BA. Allergic reactions in 2 patients led to change in medication.BA were switched 2 times in 11 patients.

**Conclusion:** Our data demonstrate real-world treatment practice in patients with JIA in tertiary care center. Availability of biological therapy was increased during the last years in Bulgaria, which led to better outcomes for our young patients.

**Patient Consent:** Yes, I received consent

**Disclosure of Interest**: None declared

## Poster session: Bone in rheumatic diseases

### P243. The characteristics of 71 patients with chronic non-bacterial osteomyelitis (CNO): a single-centre cohort study

#### E. D. Batu^1^, S. Sener^1^, A. E. Yıldız^2^, U. Kaya Akca^1^, M. Kasap Cuceoglu^1^, Z. Balık^1^, Y. Bayındır^1^, E. Aliyev^1^, O. Basaran^1^, Y. Bilginer^1^, Ü. Aydıngöz^2^, S. Ozen^1^

##### ^1^Department of Pediatrics, Division of Rheumatology, ^2^Division of Musculoskeletal Radiology, Department of Radiology, Hacettepe University Faculty of Medicine, Ankara, Turkey

###### **Correspondence:** E. D. Batu

**Introduction:** Chronic non-bacterial osteomyelitis (CNO) is an autoinflammatory bone disease.

**Objectives:** We aimed to analyse the characteristics of CNO patients in our centre.

**Methods:** Patients with CNO followed at Hacettepe University Paediatric Rheumatology Department in Ankara, Turkey, were included in this retrospective study.

**Results:** 71 CNO patients (M/F:1.2/1) were included (Table). The median (min-max) ages at disease onset and diagnosis were 9 (0.2-17) and 10 (2-18) years, respectively. The median time from onset to diagnosis was 12 months. The median follow-up period was 26 months. Arthritis and enthesitis were present in 16 (22.5%) and 3 (4.2%) patients, respectively. HLA-B27 was positive in 9 (29%) out of 31 tested patients. Seven patients fulfilled the ILAR criteria for enthesitis-related arthritis. Skin lesions were present in 11 (15.5%) patients. 63 (88.7%) patients had whole-body MRI. There was long bone involvement in 65 (91.5%), sacroiliitis in 44(62%), and vertebral involvement in 23 (32.4%) patients. Mandible, clavicle, or sternum was involved in 2 (2.8%), 10 (14.1%), or 10 (14.1%) patients, respectively. Bone biopsy was performed in 23 (32.4%) patients, revealing mostly a fibroinflammatory reaction. The most frequently administered drug was NSAID (n=58; 81.7%), followed by methotrexate (n=47; 66.2%), etanercept (n=42; 59.2%), and pamidronate (n=20; 28.2%). The response rate to treatment was highest among patients who received etanercept (33/41; 80.4%). The response rate was 50% for pamidronate. Five patients were lost-to-follow-up; there was not enough time after the treatment to evaluate the outcome in seven patients. Complete and partial clinical remission was achieved in 52 (73.2%) and 6 (8.5%) out of 59 patients, respectively. One patient died from tuberculous meningitis.

**Conclusion:** The frequent sacroiliac involvement and low frequency of skin lesions were the striking features of our cohort. The most effective treatment was anti-TNF drugs and the outcome was good in the majority of patients.

**References:**

Nuruzzaman F, Zhao Y, Ferguson PJ. Chronic nonbacterial osteomyelitis: insights into pathogenesis, assessment and treatment. Rheum Dis Clin North Am 2021;47:691-705

Aydıngöz U, Yildiz AE. MRI in the diagnosis and treatment response assessment of chronic nonbacterial osteomyelitis in children and adolescents. Curr Rheumatol Rep 2022;24:27-39.

**Patient Consent:** Yes, I received consent

**Disclosure of Interest**: None declared

### P244. Lung involvement in children with Chronic Non-bacterial Osteomyelitis (CNO )

#### C. Celani^1^, V. Messia^1^, M. Pardeo^1^, P. Toma’^2^, F. De Benedetti^1^, P. Moràn Alvarez^1^, A. Insalaco^1^

##### ^1^Division of Rheumatology IRCSS Ospedale Pediatrico Bambino Gesù, ^2^Department of Diagnostic Imaging, Bambino Gesù Children’s Hospital, IRCCS, Rome Italy , Rome , Italy

###### **Correspondence:** C. Celani

**Introduction:** Chronic non-bacterial osteomyelitis (CNO) is an idiopathic autoinflammatory disease characterized by osteolytic as well hyperostotic/osteosclerotic lesions. It is characterized by multifocal and often symmetrical pattern of osseous involvement. Other organ systems may be involved as well. Up to 20% of CNO patients develop skin manifestations (psoriasis, cystic acne, pustulosis), up to 10% of patients bowel disease and occasionally (up to 3% of patients) hepatospenomegaly and lymph node enlargement. Rarely pulmonary involvement may occur. Nowadays three cases of pulmonary involvement were reported: two children and one adult with CNO and pulmonary lesions described as parenchymal consolidations

**Objectives:** To describe four cases of CNO with pulmonary involvement

**Methods:** We describes four cases of children with CNO and pulmonary involvement discovered by WBMRI. In 3 out of 4 patients, pulmonary lesions were discovered accidentally by WBMRI, just one patient presented with respiratory symptoms

**Results:** We report four cases of children affected by CNO associated with pulmonary lesions. Median age at the time of diagnosis was 12.5 years (range: 10-17 years). The median time to develop pulmonary lesions from the CNO diagnosis was 21 months (0-72 months). The suspicion of CNO was risen by a clinical examination and the diagnosis was confirmed by Whole body MRI. All patients underwent a bone biopsy to exclude infections and bone malignancies .All of them presented with a multifocal pattern and 2 with vertebral involvement. At the onset of the disease, all patients presented with bone pain. Only one patient presented fever and increased inflammatory markers CRP (4 mg/dl) and ESR (43 mm/h). In 3 out of 4 patients, pulmonary lesions were discovered accidentally by WBMRI, just one patient presented with respiratory symptoms .The patients showed multiple pulmonary lesions with radiological characteristics of bronchiolitis obliterans organizing pneumonia (BOOP).

**Conclusion:** We presented four cases of CNO with a BOOP pattern. This radiological finding was previously described in others rheumatic diseases in adults and in one case was reported in CNO adult patient. Our case reports suggest a possible link between these two inflammatory conditions that should be confirmed in a larger study population.

**Patient Consent:** Yes, I received consent

**Disclosure of Interest**: None declared

### P245. Incidence of Chronic Recurrent Multifocal Osteomyelitis (CRMO) in the UK and republic of Ireland: intial results from 13 months of surveillance study

#### C. Suo^1^, D. Chia^1^, A. Toms^2^, A. P. Sanghrajka^3^, A. V. Ramanan^4^, O. Killeen^5^, B. Jacobs^6^, C. Ilea^6^, K. Mahmood^7^, S. Compeyrot-Lacassagne^8^, K. Armon^1,9^

##### ^1^Department of Paediatrics, University of Cambridge, ^2^Department of Radiology, Cambridge University Hospitals, Cambridge, ^3^Department of Truama and Orthopaedics, Norfolk and Norwich University Hospitals, Norwich, ^4^Department of Paediatric Rheumatology, Bristol Royal Hospital for Children, Bristol, United Kingdom, ^5^National Centre for Paediatric Rheumatology, Children’s Health Ireland, Dublin, Ireland, ^6^Royal National Orthopaedic Hospital, London, ^7^Department of Rheumatology, Alder Hey Children’s Hospital, Liverpool, ^8^Great Ormond Street Hospital, London, ^9^Department of Paediatric Rheumatology, Cambridge University Hospitals, Cambridge, United Kingdom

###### **Correspondence:** D. Chia

**Introduction:** Chronic Recurrent Multifocal Osteomyelitis (CRMO), also known as chronic nonbacterial osteomyelitis (CNO), is a rare autoinflammatory condition affecting the bones. It occurs primarily in children and teenagers and is characterised by bone pain and swelling in the absence of infection or tumour. The incidence of CRMO remains uncertain, with estimates ranging from 0.4-1 per 100,000 person years (1,2).

**Objectives:** The primary aim of the study was to identify the incidence of CRMO in patients under the age of 16 in the United Kingdom (UK) and Republic of Ireland (ROI). Additional aims include describing the demographics, clinical features, treatment, and healthcare needs of patients with CRMO.

**Methods:** A prospective surveillance study was undertaken via the British Paediatric Surveillance Unit. A monthly e-reporting card was sent to all registered paediatric consultants in the UK and ROI. A parallel surveillance study was sent via the British Society for Children’s Orthopaedics to identify patients managed solely by orthopaedics. A standardised questionnaire was sent to the reporting clinicians to collect further information.

**Results:** During initial 13 months of surveillance, 168 cases were reported. The 23 questionnaires were not returned (13.7% of reported cases). After de-duplication, a preliminary analysis was performed to exclude cases outside the reporting time period and age-group. 82 confirmed and 8 probable cases were included in our interim results. The estimated incidence of CRMO is 0.605 cases /100,000 children per year.

Median age at time of diagnosis was 10 years (range 3-16). 53 (58.9%) of cases were female. Median delay from symptom onset to diagnosis was 5 months and 16 patients (17.78%) had a delay of greater than 12 months. Most (48.9%) of the cases were diagnosed by paediatric rheumatology specialists. Other cases were diagnosed by orthopaedics (16.7%), general paediatricians (15.6%) or by a multidisciplinary team. 34 cases (37.8%) reported requiring admission related to CRMO.

The most common presenting feature was bone pain (96.67%). 34 patients (37.8%) presented with clavicular pain, and thirty-one (34.4%) had unifocal bone pain. Patients also presented with bone swelling (52.2%), joint swelling (20.0%), fever (12.2%) and general malaise (13.3%). A median of 3 investigations were reported for each case, of which 61 cases (67.7%) had whole body MRI performed and 33 cases (36.67%) had bone biopsy. The most common treatment was NSAIDs (90.0%) and bisphosphonates (33.3%).

**Conclusion:** Our results estimate the incidence of CRMO as 0.605 cases per 100,000 person years. The study will continue to capture new CRMO cases for a further 12 months. Reported cases will be followed up for 24 months. This will provide greater insight into the medium-term outcomes for patients diagnosed with CRMO and provide a snapshot into treatment strategies used by clinicians in the UK and ROI. These results will provide a valuable baseline for further research and improvement in care for patients with CRMO.

1. Jansson, A.F., V. Grote, and E.S. Group, Nonbacterial osteitis in children: data of a German Incidence Surveillance Study. Acta Paediatr, 2011. 100(8): p. 1150-7.

2. Walsh, P., et al., Chronic recurrent multifocal osteomyelitis in children: nine years’ experience at a statewide tertiary paediatric rheumatology referral centre. Rheumatology (Oxford), 2015. 54(9): p. 1688-91.

**Patient Consent:** Not applicable (there are no patient data)

**Disclosure of Interest**: C. Suo: None declared, D. Chia: None declared, A. Toms: None declared, A. Sanghrajka: None declared, A. Ramanan Consultant with: Abbvie, Eli Lilly, SOBI, Roche, UCB, Novartis, O. Killeen: None declared, B. Jacobs: None declared, C. Ilea: None declared, K. Mahmood: None declared, S. Compeyrot-Lacassagne: None declared, K. Armon: None declared

### P246. An unusual onset of chronic recurrent multifocal osteomyelitis in a 10- year-old boy

#### M. F. Gicchino^1^, G. Lodato^2^, A. N. Olivieri^1^

##### ^1^Department of Woman, Child and General and Specialistic Surgery, University of the study of Campania Luigi Vanvitelli, ^2^University of Naples Federico II, Naples, Italy

###### **Correspondence:** M. F. Gicchino

**Introduction:** Chronic recurrent multifocal osteomyelitis (CRMO), also known as chronic nonbacterial osteomyelitis, is a rare, noninfectious inflammatory disorder that causes multifocal lytic bone lesions with swelling and pain characterized by periodic exacerbations and remissions of unclear/unknown pathogenesis.

**Objectives:** To describe an unusual CRMO onset in a 10 years old boy.

**Methods:** A 10- year-old boy suffered from recurrent episodes of ocular pain associated with marked hyperaemia and periorbital oedema. Suspecting periorbital cellulitis these episodes were treated with antibiotics and steroids. Before came to our Department patient was evaluated by an ENT specialist who prescribed a computed tomography (CT) and a magnetic resonance imaging (MRI) of the orbits. The CT revealed an osteolytic lesion on the orbital process of the zygomatic bone. The MRI showed a lesion on the orbital process of the zygomatic bone, this lesion appeared hypointense in T1 sequence and hyperintense in T2 and STIR sequences, suggesting an inflammatory process. To perform a correct diagnosis a bone biopsy of this lesion was planned. Meanwhile the child developed back pain and since his brother was in follow up to our Department because of juvenile idiopathic arthritis the patient was evaluated by us for the first time. Clinical examination revealed pain on the pressure of dorsal column and sacroiliac joints. Laboratory examinations revealed increase of inflammatory parameters: erytrocyte sedimentation rate (ERS) 30mm/h (normal value <15mm/h), C-reactive protein (CRP) 2.5 mg/dL (normal value < 1mg/dL). Liver and kidney function, iron levels, C3/C4 levels, LDH, blood coagulation profile were in the norm. Both Antinuclear antibodies and ENA were negative. Virological screening (including Sars-Cov2) and QuantiFERON test were negative. Thyroid profile was in the norm and coeliac disease screening was negative. Faecal calprotectin was in the normal range. Both heart and abdomen ultrasound were in the norm. X-ray of the spine was in the norm. Spine MRI revealed abnormal marrow signal (low signal on T1, high signal on T2 and STIR) in T6, T11 vertebral bodies and in S2. A bone biopsy of T6 was preformed.

**Results:** Bone biopsy revealed: infiltration by lymphocytes and neutrophils. No neoplastic cells were identified. All cultures were negative. Langerhans cells histiocytosis (LCH) was excluded since immunohistochemical evaluation of bone marrow for CD1a and S100 expression was negative. Clinical history, physical signs, instrumental investigations and histopathological pattern were highly suggestive of CRMO. In consideration of vertebral column involvement was started therapy with bisphosphonates.

**Conclusion:** In a patient with recurrent bone pain CRMO should be considered. The diagnosis is of exclusion and it is based on the clinical and radiological data. Biopsy is needed to exclude infectious osteomyelitis, malignancy, LCH. Skeletal manifestations are unifocal or multifocal, and the involvement of clavicle, sternum or mandible suggest a CRMO diagnosis. Extra- articular manifestation are gastrointestinal and skin involvement (acne, pustolsis, psoriasis). The peculiarity of our case concerns the initial site of disease, the periorbital region. This primary localization caused difficulties in the differential diagnosis with other condition, in particular infections. The appearance of a second lesion on the vertebral column and the investigations performed, allowed the diagnosis of CRMO. The treatment of CRMO has been mostly empiric, NSAIDs are the first choice for CRMO treatment. When disease activity is high or there are complications therapy with Methotrexato, bisphosphonates or biologic drugs such as TNF antagonists (etanercept) should be considered.

**Patient Consent:** Yes, I received consent

**Disclosure of Interest**: None declared

### P247. Effect of adjusting dxa scan results for body size on the diagnosis and management of low bone mineral density and osteoporosis in children

#### F. Hamblin^1^, K. Armon^2^, N. Crabtree^3^, A. Thankamony^4^, S. Walton-Betancourth^4^, E. Hendriks^1^

##### ^1^Department of Paediatrics, University of Cambridge, ^2^Department of Paediatric Rheumatology, Cambridge University Hospitals NHS Foundation Trust, Cambridge, ^3^Department of Endocrinology and Diabetes, Birmingham Children’s Hospital, Birmingham, ^4^The Weston Centre, Department of Paediatric & Adolescent Diabetes and Endocrinology, Cambridge University Hospitals NHS Foundation Trust, Cambridge, United Kingdom

###### **Correspondence:** F. Hamblin

**Introduction:** Bone mineral density (BMD) from Paediatric DXA scans are often reported as Z-scores based on an areal calculation of BMC and normative data for age. It does not take into account effect of stature on the areal bone density. Bone mineral adjusted density (BMAD) of lumbar spine adjusts for volume of the vertebra and therefore body size. We studied the effect of making this adjustment in a clinical population.

**Objectives:** We aimed to re-evaluate DXA scan results of paediatric patients in Addenbrooke’s Hospital, Cambridge, UK by applying BMAD adjustments and up-to-date guidelines, and to assess whether this affected the diagnosis and management of reduced bone density and osteoporosis in these children. Additionally, we aimed to investigate whether clinical decisions made based on the standard BMD DXA scan results were appropriate given these adjustments.

**Methods:** All children under 16 years who had a DXA scan in 2016-2018 at Addenbrooke’s Hospital were included. Data were retrospectively collected and included fracture history and treatment decisions concerning bone health. Lumbar spine DXA results were adjusted for vertebral size to derive age adjusted BMAD Z-scores and were compared with lumbar spine BMD Z-scores. Patients classified as having normal and low bone density by each method were also compared.

**Results:** 153 patients with mean age 11.72 years (SD = 3.01) were included in the study. The Z-scores of lumbar spine BMD were significantly lower than lumbar spine BMAD Z-scores (-0.85 (SD = 1.23) vs -0.47 (SD = 1.16), p<0.001). In total 11 patients were classified as having low bone density by both measures. Fewer patients were categorised as having low bone density using BMAD compared to BMD (7.2% vs 17.0%) and only 42.3% found to have low BMD had this confirmed with BMAD (P<0.001). 16 patients had treatment changes based on low BMD. 8 of them did not have low BMAD and therefore change may not have been implemented had adjustment been used. Only 10 (33%) of patients categorised with low bone density had any form of vertebral imaging for crush fractures.

**Conclusion:** The use of lumbar spine BMD alone in reporting DXA scans in paediatric patients is more likely to result in an inaccurate diagnosis of low bone density. It is essential to use adjustment methods, such as lumbar spine BMAD, in paediatric patients to derive bone density and evaluate along with fracture history (including potentially silent vertebral fractures) when making a diagnosis of osteoporosis and therefore any treatment decisions.

**Disclosure of Interest**: None declared

### P248. Chronic Non-bacterial Osteomyelitis (CNO) and bone mineral density

#### M. Kasap Cuceoglu^1^, M. Canturk^2^, S. Sener^1^, Z. Balik^1^, Y. Bayindir^1^, E. Aliyev^1^, O. Basaran^1^, A. E. Yildiz^3^, E. D. Batu^1^, Y. Bilginer^1^, U. Aydingoz^3^, A. Ozon^2^, S. Ozen^1^

##### ^1^Pediatric Rheumatology, ^2^Pediatric Endocriology, ^3^Radiology, Hacettepe University, Ankara, Turkey

###### **Correspondence:** M. Kasap Cuceoglu

**Introduction:** Chronic nonbacterial osteomyelitis (CNO) is an inflammatory bone disorder that affects children.^1^ In the absence of accepted diagnostic criteria or clinical/radiological biomarkers, CNO remains a diagnosis of exclusion.^2^

**Objectives:** We aim to show the relationship between CNO and bone mineral density and investigate whether osteoporosis with/without vitamin D deficiency can cause challenges in the treatment of CNO.

**Methods:** In the study, 70 patients (<18yo) with CNO were reviewed using electronic files at Hacettepe University retrospectively between January 2015 and April 2022. Demographic and clinical characteristics of patients, 25 OH vitamin D level status (N:>20 ng/ml), radiological findings as well as medical treatments were evaluated. A score between -1.1 and -2.4 is classified as osteopenia (low bone mass). A score of -2.5 and below is defined as osteoporosis. In the pediatric population, osteoporosis is a clinical diagnosis and is reserved for those patients with a BMD Z- score less than or equal to -2.0 in combination with a clinically significant fracture.

**Results:** A total of 70 patients (females 53%) were included in this study. The median age at diagnosis of CNO was 7 (1-17) years. Overall, vitamin D level was available before diagnosis in 46/70 patients, 28 of which were normal, while 18 patients had severe deficiency (25 OH vitamin D <10 ng/ml). Bone mineral density was studied in 24 patients, normal in 14, osteoporotic in 4, and osteopenic in 6 patients. 19 patients received pamidronate treatment, 13 of whom received methotrexate and 9 received anti-TNF before pamidronate. Among the patients receiving pamidronate, 7 had 25 OH vitamin D <10 ng/ml, 3 had osteopenia, 3 had osteoporosis in DEXA. Thirty-five patients received anti-TNF therapy (29 of them used ETA, 5 ADA, 2 INF). Although it was not statistically significant, there was no patient with osteoporosis in the group receiving synthetic DMARD/NSAID (pamidronate/anti TNF naive group, n=20). The median lumbar BMD z score was -0.90 (min-3.8, max 3.2) in the pamidronate/anti TNF group and 0.4 (min-1.8, max1.02) in the DMARD/NSAID group. Overall history of fracture was present on admission in ten patients (3 osteoporosis, 3 osteopenia, 3 normal bone mineral density, 1 NA). All patients with fracture received pamidronate as first option and they benefited from this treatment. (p=0.013). In the pamidronate/ anti TNF group, 25 OH vitamin D level was lower than synthetic DMARD/NSAID (pamidronate naïve) group, and the difference was nearly significant. (p=0.073)

**Conclusion:** Patients with normal bone mineral density responded well to DMARD/NSAID, better than that of remaining patients, and they did not need alternative treatment. Vitamin D deficiency and/or osteopenia may lead to a more refractory course, thus it should be monitored effectively in patients with CNO.

**References**

1. Zhao Y, Ferguson PJ. Chronic Nonbacterial Osteomyelitis and Chronic Recurrent Multifocal Osteomyelitis in Children. *Pediatr Clin North Am*. 2018;65(4):783-800. doi:10.1016/j.pcl.2018.04.003

2. Oliver M, Jayatilleke A, Wu E, et al. Establishing core domain sets for Chronic Nonbacterial Osteomyelitis (CNO) and Synovitis, Acne, Pustulosis, Hyperostosis, Osteitis (SAPHO): A report from the OMERACT 2020 special interest group. *Semin Arthritis Rheum*. 2021;51(4):957-961. doi:10.1016/j.semarthrit.2021.05.015

**Patient Consent:** Not applicable (there are no patient data)

**Disclosure of Interest**: None declared

### P249. Osteoid osteoma presenting with chronic limb pain- our experience with noninvasive therapy

#### K. Rubelj^1^, I. Radoš^1^, L. Novosel^2^, R. Vukojević^2^, M. Vidovic^1^

##### ^1^Department of pediatric, Devison of rheumatology and clinical immunology, ^2^Department of radiology, University Hospital Center Sestre milosrnice, Zagreb, Croatia

###### **Correspondence:** K. Rubelj

**Introduction:** Limb pain is common childhood complaint. Chronic and recurrent pain raises different diagnostic options osteoid osteoma (OO) being one of them. OO is the most common benign bone tumor of young adults and it’s relatively rare in children. It mostly affects long bones with chronic nighttime pain as predominant symptom. Current method of treatment is complete surgical excision of the nidus. This is the first time that the new method - percutaneous thermal radiofrequency ablation (RFA) with temperature of 90°C under control of conventional computerized tomography scan (CT-scan) or cone-beam CT-scan is used in pediatric population in Croatia.

**Objectives:** In this paper we present pediatric patients in whom OO is successfully removed by percutaneous RFA.

**Methods:** Nine patients hospitalized with chronic limb pain from 05/2020 to 10/2021 in the Department of rheumatology and clinical immunology, Clinic of Pediatrics Sestre milosrdnice University Hospital Center were diagnosed with OO and treated with percutaneous RFA in corroboration with Clinical department for diagnostic and interventional radiology. All procedures were performed under general anesthesia. The average age of the patient was 11.8 year with overhang of female gender (6/9) over male (3/9). Classical symptom in all patients was with sleep disturbance. Average time from first symptom to the diagnosis was 7.8 months. Patients underwent different diagnostic procedures including x-ray, ultrasound, multisided computed tomography (MSCT) or scintigraphy of the bones. Almost everyone (8/9) had the diagnosis confirmed with magnetic resonance imaging (MRI) with pathognomonic sclerotic lesion with nidus and perifocal bone and soft tissue subperiosteal edema.

**Results:** Procedure was successful in all patients, with only occasional oral analgesia during 24-hour observation and no delayed complications. In all cases the diagnosis was confirmed by pathophysiological evaluation. Two patients had recurrence of pain in 2 month period following the ablation due to recurrence of the lesion. Complete pain relief was however achieved after a second ablation in both cases. Thus, our primary and secondary clinical success rates were 78 and 100%, respectively.

**Conclusion:** One of the differential diagnostic options in children with chronic nighttime limb pain is OO. Complete surgical excision is preferred treatment option, providing pain cessation. However, surgery has some disadvantages, including the difficulties in locating the lesion intraoperatively, need for prolonged hospitalization, and the possibility of postoperative complications such as infection, fracture and unsatisfactory cosmetic result.. Percutaneous RFA as new method is safe minimally invasive, and extremely effective method for the management of OO in children and should be considered as primary option.

**Patient Consent:** No, I have not receive consent

**Disclosure of Interest**: None declared

## Poster session: Genetics, genomics and environment

### P250. Fibrodysplasia ossificans progressiva : first case report in Libya

#### A. A. Abushhaiwia^1^, Y. Elfawires^2^

##### ^1^Paediatric Rheumatology , Faculty of Medicine,Tripoli university , Tripoli Children’s Hospital , ^2^Paediatric Rheumatology , faculty of Medicine, Tripoli university, Tripoli children’s hospital, Tripoli , Libya

###### **Correspondence:** Y. Elfawires^2^

**Introduction:** Fibrodysplasia ossificans progressiva (FOP) is a very rare genetic connective tissue disorder characterized by abnormal development of bone. Patients with FOP have characteristically malformed big toes that are present at birth (congenital). Other skeletal malformations may occur. Most cases of FOP is caused by the mutation of a gene (ACVR1) in the bone morphogenetic protein (BMP) pathway. To our knowledge so far there is no reported cases with FOP in Libya, and this case has been worked up initially as infectious or malignant causes behind the bone swelling. Here we describe the clinical features and management.

**Objectives:** to highlight the importance of early diagnosis and recognition of FOP, and avoid unnecessary blood test, injections, biopsies and dental work minimizing the disease progression and early disability as much as possible

**Methods:** the diagnosis was established mainly on typical clinical picture ( appearance of malformed big toes and the new bone formation at site of biopsy and trauma), the case was discussed with an international FOP awareness campaign and due to lack of facilities and the high cost of the gene test clinical diagnosis was found to be sufficient.

**Results:** a two years and four months old Moroccan girl born of non-consanguineous healthy parents, presented to P/Rheumatology department Oct/2021 with history of neck lumps at level of lower cervical spine, associated with neck stiffness and limitation of movement. The neck lumps where first noted three to four months before presentation discovered accidentally and not preceded by a recognized trauma nor a special event. The lumps where about 2x2 cm, firm to hard in consistency, not tender, no redness, and no hotness, associated with cervical lymphadenopathy evident by examination and neck U/S, and associated with limitation of neck movement especially neck extension and decreased range of movement in both shoulders. The case was initially addressed as lyphadenopathy. Her initial lab results showed, WBC 14.28 x 103 (49.9% neutro: 39.9% lympho), Hgb 12.9g/dl, PLT 360 x103, blood film showed no significant abnormality. ESR 5 mm/hr, CRP negative, LDH 366 normal, Alkp 142 normal, serum Ferritin 61.7 ng/ml, Ca+2 9.8, CK 92 IU/L, Renal function and Liver function tests came within normal range. TB also was excluded, immunoglobulin level came within normal. Regarding the imaging U/S neck = normal study apart from non specific deep cervical lymphadenopathy. U/S chest wall/ abdomen/ pelvis = ill defined fusiform thickening of subcutaneous fat layers at Lt Lateral aspect of the chest wall, measuring 30x 14 mm suggesting post inflammatory residual edema. CT scan of chest/ abdomen/ pelvis = no significant abnormality. CT neck with IV contrast = bilateral multiple deep cervical enlarged lymph nodes. Her case was discussed with orthopedists, endocrine and infectious specialties and unfortunately a true cut Biopsy from the lesion was taken showing evidence of infantile myofibromatosis. She received IV and IM Abx during workup of the case. The illness progressed involving the elbow joints and both wrists, with pain disturbing her sleep but not her activity, also the site of injections and bone biopsy started to harden. Depending on the above mentioned scenario and the typically malformed big toes noticed on examination the diagnosis of FOP was made and the case was discussed regarding the need of gene testing putting in consideration the very limited resources. She was started on an induction course of steroids 1 mg/kg and NSAIDs ( Ibuprofene ) showing much improvement in her symptoms.

**Conclusion:** FOP should be considered in any child with malformed big toes presenting at birth, to protect the child from any un needed interventional tests and procedures minimizing the early complications of disease.

**Patient Consent:** Yes, I received consent

**Disclosure of Interest**: None declared

### P251. Livedo racemosa a cutaneous finding that could be an early presentation for Sneddon’s syndrome

#### A. A. Abushhaiwia^1^, Y. AElfawires^2^

##### ^1^Paediatric Rheumatology , Faculty of Medicine,Tripoli university, Tripoli Children’s Hospital , ^2^Paediatric Rheumatology , Faculty of Medicine, Tripoli university, Tripoli Children’s Hospital , Tripoli , Libya

###### **Correspondence:** Y. AElfawires

**Introduction:** Sneddon’s syndrome is a rare disorder that most commonly affects young females, characterized by livedo racemosa and central nervous system disease. Livedo racemosa is differentiated from livedo reticularis by its shape, pattern in addition to its persistence on warming. Livedo racemosa in Sneddon syndrome is widespread and almost always involves the trunk and/or buttocks.

**Objectives:** to highlight the importance of considering sneddon’s syndrome in young females with levido racemosa due to life threatening Ischemic events such as stroke and transient ischemic attack. Headache and hypertension are also known associated problem

**Methods:** two case reports are described

**Results: case one**, eight year-old Libyan female for non-consanguineous parents and no significant past nor family histories. Presented to Pediatric rheumatology clinic as referral from Dermatology clinic with livedo racemosa appeared one year ago on lower limbs, upper limbs and trunk later. Among the presenting symptoms she mouth ulceration and mild arthralgia rest of the systemic review was neg. Her peripheral pulses and BP was normal, ECG & ECHO also normal, U/S abdomen normal, MRI brain normal (changes are expected to be seen in 2^nd^ decade of life). Blood tests: WBC 9.1, HgB 11.9 g/dl, plt 436. ESR 60 mm/hr, CRP 23.4 mildly raised, LFT normal, U/E/C normal, LDH 187,ENA ( Ro/SS-A 52, Ro/SS-A 60 and La/SS reacted +ve), D-dimer normal, PT APTT INR normal, ANA +ve, antids- DNA neg,anticardiolipin IgG& IgM neg, anti phospholipid IgG&IgM neg, lupus anticoagulant weak +ve, C3&C4 normal range, coomb’s test normal. slit lamp eye examination normal. Skin biopsy and MRA brain has been ordered waiting for results. Currently she is on asprin tab 75mg. she received short course steroid 1mg/kg and hydroxychloroqin 4mg/kg earlier. **Case two,** 14 year- old Libyan female for non-consanguineous parents also neither significant family history nor past medical history. Referred to our clinic from dermatology clinic as a case of livedo racemosa involving both LL (since 2011 till 2017 when diagnosed), with progression and involvement of both UL and trunk. Initially she had only arthralgia affecting both ankles but started to have headache and despite normal BP and normal peripheral pulses she feels tingling, hotness and dull aching pain in LL. She had a Doppler scan for LL showed it post tibial vein heamangioma/venous angioma of both calf with no evidence of thrombosis. MRI & MRA brain both normal, ECHO & ECG normal, x-ray of both ankles and wrists showed soft tissue swelling only, MRI Rt ankle showed acute non destructive arthritis. Slit lamp eye examination normal. Blood tests: WBC 10, Hgb 10.7 g/dl, PLT 459, blood film normocytic hypochromic anemia with normal iron study. ESR 52mm/hr, CRP 25mg (weak +ve), LFT normal, U/E/C normal, CPK & LDH normal, PT APTT INR normal, D-dimer 607 ng/ml raised, fibrinogen 4.5 g/l just above normal range. Serology: ANA, anti ds DNA, ENA screen, C3&C4, anticardiolipin, lupus anticoagulants and anti beta2 glycoproteine1 Abs all came negative. Skin biopsy was requested many times but family was reluctant to do it, with poor compliance to follow up visits. She received short course steroid and Naproxen for arthritis and Aspirin 75 mg daily. Re-evaluation workup including new MRA brain and new serology was requested in last follow up considering she now is in her 2^nd^ decade of life.

**Conclusion:** Ischemic events such as stroke and transient ischemic attack are a hallmark of Sneddon’s syndrome and usually appear later in adulthood. Early screening for patients with livedo racemosa with brain MRA & MRI, control of BP and starting anticoagulants can minimize cerebrovascular events and their impact on patients life. Also importance of skin biopsy for diagnosis in such cases.

**Patient Consent:** Yes, I received consent

**Disclosure of Interest**: None declared

### P252. Glutathione S-transferase gene polymorphisms and methotrexate effectiveness in patients with juvenile idiopathic arthritis

#### S. Huljev Frkovic^1^, M. Frkovic^1^, K. Crkvenac Gornik^2^, D. Rogic^2^, M. Jelusic^1^

##### ^1^Department of pediatrics, UHC Zagreb / School of medicine Zagreb, ^2^Department of laboratory diagnostics, UHC Zagreb, Zagreb, Croatia

###### **Correspondence:** M. Frkovic^1^

**Introduction:** Early control of the inflammatory process in juvenile idiopathic arthritis (JIA) correlates with more favourable treatment outcomes. In case of methotrexate, combination of heterogeneous treatment response and 3–6-month period of continuous administration required for clinical assessment of the drug effectiveness, result in closing the “window of opportunity” in more than 30 % of JIA patients. Deleting polymorphisms of glutathione S-transferase T1 (GSTT1) and M1 (GSTM1) genes influence the effectiveness of different drug combinations in a variety of rheumatic diseases.

**Objectives:** To investigate the impact of GSTM1 and GSTT1 gene deletion polymorphisms on MTX effectiveness in JIA patients.

**Methods:** The study included 109 JIA patients: 46 patients in stable clinical remission for a minimum 6-month period during MTX therapy and 63 patients who did not achieve stable clinical remission during MTX therapy. In all patients participating in the study, GSTM1 and GSTT1 deletion polymorphisms were determined.

**Results:** Deletion polymorphism of the GSTM1 gene was detected in 40/63 (63.5%) of patients who did not achieve remission during MTX therapy and 27/46 (58.7%) of patients who achieved remission during MTX therapy. Deletion polymorphism of the GSTT1 gene was detected in 14/63 (22.2%) of patients who did not achieve remission during MTX therapy in 8/46 (17.4%) of patients who achieved remission during MTX therapy. The combination of deletion polymorphisms for the GSTM1 and GSTT1 genes was detected in 8/63 (12.7%) of patients who did not achieve remission during MTX therapy and in 6/46 (13.0%) of patients who achieved remission during MTX therapy. No statistically significant differences were observed in the distribution of deletion polymorphisms or their combinations among the study groups. In a subgroup of JIA patients who did not achieve remission during MTX therapy, statistically significant difference in frequency of GSTM1 deletion polymorphism was detected between subgroup with one or more changes of biologic disease modifying drugs (bDMARD) compared to the subgroup with one bDMARD in terms of achieving remission: 64.3% to 28.6%; P=0.026.

**Conclusion:** According to this study, the determination of GSTM1 and GSTT1 gene deletion polymorphisms is not useful in predicting the efficacy of MTX in JIA patients. However, the possible influence of GSTM1 gene deletion polymorphism on bDMARD efficacy in JIA patients opens new horizons in investigations of GST gene polymorphisms and their influence on treatment outcomes in JIA and other rheumatic diseases.

**Patient Consent:** Not applicable (there are no patient data)

**Disclosure of Interest**: None declared

### P253. Lack of association of SAA1 RS12218 and IL1B RS1143634 gene polymorphisms with FMF and CAPS, but the presence of a correlation of IL1B RS1143634 gene polymorphism with the level of serum IL-18 in patients with FMF

#### I. Guseva^1^, E. Fedorov^2^, S. Salugina^2^, M. Krylov^1^, E. Samarkina^1^, M. Kaleda^2^

##### ^1^Immunology and Molecular Biology of Rheumatic Diseases, ^2^Pediatrics, V.A. Nasonova Research Institute of Rheumatology, Moscow, Russian Federation

###### **Correspondence:** S. Salugina

**Introduction:** Autoinflammatory diseases (AIDs) are a group of rare, genetically determined diseases characterized by periodic events of inflammation, fever, and clinical symptoms that mimic rheumatic pathology. Laboratory serological markers of AIDs are C reactive protein (CRP) and serum amyloid A (SAA) protein. Serum IL-18 level is also elevated in AIDs.

**Objectives:** We investigated whether *SAA1* -13T/C (rs12218) and *IL1B* C3954T (rs1143634) gene polymorphisms may affect the susceptibility to pediatric patients (pts) with Familial Mediterranean Fever (FMF) and Cryopyrin associated periodic syndromes (CAPS). We also evaluated whether these polymorphisms can affect CRP, SAA and IL-18 serum levels.

**Methods:** 44 FMF pts – 22 boys, 22 girls; age at onset – M (SD) 7.85 (5.64) years and 27 CAPS pts - 15 girls, 12 boys; age at onset – 4.68 (5.83) years, and 95 healthy individuals (controls) were included in this study. The diagnosis of FMF was based on Turkish paediatric criteria for the diagnosis of FMF and was confirmed by the detection of pathogenic mutations of the *MEFV* gene in the homozygous or compound-heterozygous states. The diagnose of CAPS was made on the basis of characteristic clinical signs and was confirmed by the detection of a pathogenic mutation in the *NLRP3* gene. *SAA1* (rs12218) and *IL1B* (rs1143634) gene polymorphisms were genotyped using allele-specific RT-PCR assay. CRP, SAA and IL-18 serum concentrations were measured in FMF and CAPS pts.

**Results:** Table shows the genotypic frequencies of *SSA1* and *IL1B* genes in FMF, CAPS and controls. There were no significant differences between FMF, CAPS pts and controls in the genotypic *SSA1* and *IL1B* gene polymorphisms (p>0,05). The *SAA1* (rs12218) gene polymorphism did not correlate with CRP, SAA and IL-18 levels in FMF and CAPS pts. The *IL1B* gene polymorphism also did not correlate with the levels of CRP and SAA in FMF and CAPS pts, but was significantly associated with the level of IL-18 in FMF pts. In carriers of at least one mutant allele T (CT+TT) the level of IL-18 was significantly higher compared to carriers of the CC genotype (2256.74 ± 1532.28 and 231.19 ± 218.80 respectively, p=0.003).

**Conclusion:** Our preliminary study in small groups of pediatric FMF and CAPS pts revealed that *SAA1* (rs12218) and *IL1B* (rs1143634) gene polymorphisms are not associated with susceptibility to FMF and CAPS. The influence of *IL1B* (rs1143634) gene polymorphism on proinflammatory cytokine IL-18 serum levels in FMF pts is also shown.

**Patient Consent:** Yes, I received consent

**Disclosure of Interest**: None declared

**Table 1 (abstract P253).** See text for description

Genotypes		FMF, n=44 (%)	CAPS, n=27 (%)	Controls, n=95 (%)
*SAA1* (rs12218)	TT	17 (38.6)	6 (22.2)	39 (41.1)
	TC	24 (54.6)	15 (55.6)	42 (44.2)
	CC	3 (6.8)	6 (22.2)	14 (14.7)
p (pts vs controls)		0.32	0.19	
*IL1B* (rs1143634)	TT	25 (56.8)	18 (64.3)	66 (69.5)
	TC	16 (36.4)	9 (32.1)	25 (26.3)
	CC	3 (6.8)	1 (3.6)	4 (4.2)
p (pts vs controls)		0.34	0.83	

### P254. Genetic analysis of whole exome sequencing in a cohort of children with refractory JIA reveals rare genetic risk factors for JIA at loci of known inflammatory diseases

#### M. Tordoff^1^, S. L. Smith^1^, G. Rice^2^, L. R. Wedderburn^3,4,5^, K. L. Hyrich^6,7^, A. Morris^1^, T. A. Briggs^8^, W. Thomson^1,7^, S. Eyre^1,7^, J. Bowes^1,7^ on behalf of The CLUSTER Consortium

##### ^1^Centre for musculoskeletal research, Centre for genetics and Genomics versus arthritis, ^2^Division of Evolution Infection and Genomics, University of Manchester, Manchester, ^3^Infection, immunity and inflammation research and teaching department, UCL, great ormond street institute of child health, ^4^Centre for Adolescent Rheumatology Versus Arthritis at UCL, UCL Hospital and Great Ormond Street hospitall, ^5^NIHR Biomedical Research Centre at Great Ormond Street Hospital, Great Ormond Street Hospital , London, ^6^Centre for Epidemiology Versus Arthritis, The university of manchester, ^7^NIHR Manchester Biomedical Research Centre, Manchester Academic Health Science Centre, ^8^Division of Evolution Infection and Genomics, The university of manchester, Manchester, United Kingdom

###### **Correspondence:** M. Tordoff

**Introduction:** Juvenile idiopathic arthritis (JIA) is a childhood rheumatic disease that can result in long term disability. The goal of treatment is remission. The absence of a response to multiple standard disease therapies is referred to as refractory JIA.

**Objectives:** Investigate the role of rare genetic risk factors in a cohort of children with refractory JIA using whole exome sequencing (WES).

**Methods:** WES of 99 children with JIA was performed with the Agilent SureSelect Human All ExonV6 kit. Individuals were defined as refractory if they had failed treatment to methotrexate and at least one further biologic drug. All quality control (QC), variant filtering and annotation was performed in Varseq (version 2.2.1). Variants with a read depth <30 and genotype quality <80 were removed. Rarity and pathogenicity filters were applied to remove variants with an allele frequency >1% (based on ExAC, gnomAD, gnomAD exome, NHLBI and 1KGp phase 3), classified as benign or likely benign on ClinVar, with a CADD score <15 and a REVEL score >0.7. Variants were annotated if they appeared in a gene from the primary immunodeficiency PanelApp (Martin et al., 2019), in a gene associated with an arthritis phenotype or in a gene that appeared on a paediatric monogenic gene list. The variants were then classified using American College of Medical Genetics (ACMG) guidelines (Richards et al., 2015). Benign, or likely benign, classified variants were removed.

**Results:** A total of 470 variants passed VarSeq analysis. Of these variants, 24 were within genes of interest to JIA or paediatric inflammatory disease. Three gene regions will be discussed as examples. Within *KIR2DL3*, the heterozygous variant p.Gly12Ala was detected in six individuals. This variant has not been reported on gnomAD. KIR2DL3 is as an inhibitory receptor to HLA-C molecules. This region has been linked to psoriatic arthritis previously. Two further variants, p.Trp20Gly and p.Arg162Thr, were also detected in this region. Two heterozygous variants, p.Asp127Asn and p.Ala265Val, were detected in *NOD2*, which is a susceptibility locus for Blau syndrome and has a role in immune homeostasis. Blau syndrome is characterised by arthritis, uveitis and dermatitis. Variants p.Asp127Asn and p.Ala265Val had a gnomAD Exomes frequency of 6.6x10^-4^ and 1.3x10^-4^. One final example is the region *UNC13D*. Three heterozygous variants were detected in this region. Variant p.Ille848Leu was detected in two individuals in this cohort and has recently been detected in a compound heterozygous state in two patients in a study of JIA. Variant p.Ille848Leu had a gnomAD Exomes frequency of 1.0x10^-4^.

**Conclusion:** WES analysis of 99 children with refractory JIA revealed that individuals carry rare genetic variants within gene regions of interest to JIA and that have been implicated in inflammatory paediatric monogenic diseases. Further analysis is needed to determine if these variants are present in a cohort of JIA patients who do not have refractory diseases. These results highlight the need for further research into the genetic risk factors of refractory disease and that genetic analysis can provide information to improve treatment outcome in the future.

**Patient Consent:** Yes, I received consent

**Disclosure of Interest**: None declared

## Poster session: Immunoregulation and basic science

### P255. Cyclic amp response element modulator α governs PD-1 expression on CD4+ T cells in psoriasis across age groups

#### E. Carlsson^1^, A. L. Carvalho^1^, S. Hofmann^2^, L. J. McCann^3^, P. J. Ferguson^4^, B. Thompson^5^, T. Liloglou^6^, S. Abraham^7^, A. E. Surace^1^, S. Northey^1^, S. Russ^2^, M. W. Beresford^1^, C. M. Hedrich^1^

##### ^1^Department of Women’s and Children’s Health, University of Liverpool, Liverpool, United Kingdom, ^2^Department of Pediatrics, University Hospital Carl Gustav Carus, Technische Universität Dresden, Dresden, Germany, ^3^Alder Hey Children’s NHS Foundation Trust, Liverpool, United Kingdom, ^4^Pediatrics Department, University of Iowa Carver College of Medicine, Iowa City, Iowa, United States, ^5^Department of Dermatology, Alder Hey Children’s NHS Foundation Trust, ^6^University of Liverpool, Liverpool, United Kingdom, ^7^Department of Dermatology, University Hospital Carl Gustav Carus, Technische Universität Dresden, Dresden, Germany

###### **Correspondence:** E. Carlsson

**Introduction:** The pathophysiology of psoriasis, psoriatic arthritis (PsA) and psoriatic juvenile idiopathic arthritis (PsJIA) are poorly understood. In psoriasis, effector CD4^+^ T lymphocytes contribute to inflammation and tissue damage. The inhibitory surface coreceptor Programmed Death (PD)-1 plays a key role in the control of effector T cell function, and its therapeutic inhibition in cancer associates with the development of psoriasis and other autoimmune phenomena. Increased expression of the transcription factor cAMP response element modulator (CREM)α is a hallmark of T cell mediated autoimmune diseases.

**Objectives:** This study investigated PD-1 regulation and surface expression in CD4^+^ T cells from psoriasis, PsA and PsJIA patients to identify molecular pathomechanisms that may be used as biomarkers and/or treatment targets.

**Methods:** Peripheral blood naïve and effector T cell populations (CD45RA, CCR7 surface expression) were quantified in controls, psoriasis, PsA and PsJIA. CD4^+^ T cells were isolated using FACS and characterised based on PD-1 expression. Cells were stimulated (anti-CD3/CD28) and cytokine expression and release was measured (LUMINEX, Meso Scale Discovery). Jurkat CD4^+^ T cells deficient (CRISPR/Cas9) or over-expressing (lentiviral) CREMα were generated to investigate underlying molecular mechanisms. Luciferase reporter assays were used to test *trans*-regulatory events mediated by CREMα. Chromatin immunoprecipitation was performed using antibody against CREMα and DNA methylation in the *PDCD1* (encoding PD-1) promoter region was assessed by bisulfite pyrosequencing using primary cells and genetically modified model Jurkat cell lines deficient in CREMα.

**Results:** CD4^+^ T cells from psoriasis, PsA and PsJIA patients exhibit reduced PD-1 surface expression when compared with matched controls (p<0.05). This associates with increased expression of CREMα and imbalanced secretion of pro-inflammatory IL-17A and immune-regulatory IL-2 in psoriasis and PsA. CREMα *trans*-represses the *PDCD1* promoter and mediates DNA methylation through co-recruitment of the *de novo* DNA methyltransferase DNMT3a.

**Conclusion:** This study for the first time links increased expression of CREMα in CD4^+^ T cells from psoriasis and PsA patients with altered PD-1 expression and effector cytokine expression. The CREMα:PD-1 network promises potential as disease biomarker and/or treatment target.

**Disclosure of Interest**: None declared

### P256. Exploratory immunophenotype of the rare disease juvenile sjögren’s syndrome reveals a dysregulation of B and T memory cell frequencies

#### L. Martin-Gutierrez^1^, H. Peckham^2^, A. Radziszewska^2^, J. Peng^2^, O. Nettey^2^, E. Jury^1^, C. Ciurtin^2^

##### ^1^Centre for Rheumatology Research, ^2^Centre for Adolescent Rheumatology Versus Arthritis, University College of London, London, United Kingdom

###### **Correspondence:** L. Martin-Gutierrez

**Introduction:** Sjögren’s syndrome (SS) is an autoimmune rheumatic disease characterised by dryness resulting from chronic lymphocytic infiltration of the exocrine glands. Patients also present with other extraglandular manifestations such as arthritis, anemia and fatigue or various organ and systems involvement. The disease is more frequent in women aged 30-50. However, in rare cases, the disease starts in childhood and is known as juvenile SS (JSS) or childhood SS. Children have different clinical manifestations compared to adults, with dryness being less common, making the diagnosis very challenging.

**Objectives:** To investigate in depth the immune cell profile of patients with JSS for better understanding of disease pathogenesis.

**Methods:** Peripheral blood was collected from a cohort of patients with JSS while attending appointments at UCLH clinics. None had received B-cell depletion therapy. Immune-phenotyping of 29 immune-cell subsets, including B and T cells, in peripheral blood from patients with JSS (n=10) and age and sex-matched healthy controls (n=10) was performed using flow cytometry as we have performed previously for patients with adult onset SS. Data were analysed using multiple t-tests and compared with the adult SS immune phenotype.

**Results:** Patients with JSS had an average age of 18 years (range 16-21) with an average age of disease onset at 14 years (range 12-18). Up to 60% of patients presented Anti-Ro autoantibodies while 50% presented Anti-La autoantibodies.

Patients with JSS had an altered immune profile compared to age matched healthy controls (average of 18 years, range 15-25). In the B cell compartment, JSS patients had higher frequencies of Total CD19+ B cells (p=0.0044), Naïve B cells (CD19+IgD+CD27-) (p=0.0183) and bm2 (CD19+IgD+CD38+) (p=0.0490) whereas memory B cell subsets such as early bm5 (CD19+IgD-CD38+) and late bm5 (CD19+IgD-CD38-) were significantly reduced (p=0.0249, and p=0.0117 respectively), similar to the profile seen in patients with adult-SS. Interestingly, in the CD4+ T cell compartment, central memory (CD4+CD27+CD45RA-) T cells were significantly reduced (p=<0,0001) but effector memory (CD4+CD27-CD45-) and effector memory-re-expressing-CD45RA (EMRA, CD4+CD27-CD45RA+) T-cell subsets were significantly elevated (p=0.0171 and p=0.0002 respectively). These changes were not identified in adult-SS patients. Finally, unlike our observations in patients with adult-onset SS there was no widespread deregulation of CD8+ T cell subsets in JSS patients; only a significant increase in CD8+CD25-CD127+ responders T cells (p=0.0392) was observed in JSS patients versus healthy.

**Conclusion:** This is the first pilot study investigating the immunophenotype profile of patients with JSS. Our preliminary findings suggest altered immune phenotypes in both B-cell and T cell compartments and for B cells are in concordance with previous immunophenotyping studies in adult SS (predominance of naïve and lower frequencies of memory B cells), suggesting an immunological rationale for the use of similar therapies. Further studies, comparing the adult with the juvenile phenotype could help stratify patients for targetted therapies and improve treatment in this rare disease in children for which no evidence-based recommnedations exist.

**Patient Consent:** Not applicable (there are no patient data)

**Disclosure of Interest**: None declared

### P257. Preliminary results of the inflammatory biomarkers measurement in patients with fibrodysplasia ossificans progressiva in compare to systemic juvenile idiopathic arthritis

#### V. Matkava^1^, I. Nikishina^1^, S. Arsenyeva^1^, M. Kaleda^1^, E. Samarkina^2^, M. Cherkasova^3^, E. Fedorov^1^, A. Arefieva^1^

##### ^1^Pediatric Department, ^2^Laboratory of Immunology and Molecular Biology of Rheumatic Diseases, ^3^Laboratory of thromboinflammation, V. A. NASONOVA RESEARCH INSTITUTE OF RHEUMATOLOGY, Moscow, Russian Federation

###### **Correspondence:** V. Matkava

**Introduction:** Fibrodysplasia ossificans progressiva (FOP) is an autosomal-dominant rare genetic disease which provokes many inflammatory flare-ups with formation of heterotopic ossifications due to uncontrolled osteogenesis. It is one of the most disability condition which caused by mutation de novo in ACVR1 gene. Some data suggests that FOP may belong to the spectrum of autoinflammatory rheumatic disease.

**Objectives:** To describe the first experience of measurement some serum biomarkers levels received from patients (pts) with FOP compared with Systemic juvenile idiopathic arthritis (sJIA) pts.

**Methods:** Since 2018 we collected biobank of blood samples from 5 pts with FOP, 52 pts with sJIA and 5 samples from healthy children (as a control group). Serum levels of IL-18, IL1RA, IL1b, IL-6, TNFR1, TNFR2, ferritin were measured using standard commercial enzyme-linked immuno-sorbent assay (ELISA). CRP was determined by commercial nephelometric method.

**Results:** The pts were divided into 2 groups: group I - with sJIA (52 pts), group II – with FOP (5 pts), control group – 5 pts. The ratio of boys and girls was 1:1,5 in pts with FOP and 1:1,7 in pts with sJIA. Median age of pts with FOP at the examination time was 12.5 years [5; 17], with sJIA – 11.4 years [7; 13], control group – 12.8 [7; 17]. CRP and ferritin levels were in normal range in all pts with FOP and control group and were significant higher in sJIA pts. Among of spectrum of investigated biomarkers levels of the IL-6, TNFR1 were in normal range. Level of the IL1b in pts with FOP was the same (0.001 pg/ml) with control group but significantly higher in sJIA pts (median 0,9 [0,002; 2,4]). However high levels for IL-1RA (reference range 78-970 pg/ml) were detected in both groups: sJIA (Me 1267,43 pg/ml [interquartile range (IQR) 936,75; 2292,75]) and FOP (Me 1342,36 pg/ml [IQR 927,0; 1811,6]) and values of FOP pts were higher than in 44 sJIA pts without macrophage activation syndrome (MAS) (Me 1165,2 pg/ml [IQR 868,0; 1743,8]) and lower than in 8 sJIA pts with MAS (Me 2654,2 pg/ml [IQR 1432,2; 6252,2]). In control group IL-1RA was in normal range (Me 404,77 pg/ml [IQR 255,3; 506,08]). Also high levels of TNFR2 (ref. range 3.4-10,8 ng/ml) were presented only in pts with FOP (Me 14,99 ng/ml [IQR 5,49; 23,12]) compared to sJIA (Me 9,47 ng/ml [IQR 4,46; 29,49]) and control group (Me 0,35 ng/ml [IQR 0,243; 3,496]). It was expected, that the level of IL-18 (ref. range 0-732,7 pg/ml) was significantly higher in pts with sJIA (Me 690,14 pg/ml [IQR 361,18; 3607]) than in FOP (Me 0,01 pg/ml [IQR 0,008; 0,05]) pts and control group (Me 0,0194 pg/ml [IQR 0,0032; 14,926]).

**Conclusion:** Our preliminary data confirmed, that immunopathogenesis of FOP can be considered as autoinflammatory disease. High level of IL-1RA and TNFR2 was detected in pts with FOP. It seems important that of IL-1RA was higher than sJIA without MAS, but lower than in sJIA with MAS. This evidence confirmed our decision to treat FOP pts with targeted therapy (JAK-kinase inhibitors), which was successfully used in 15 pts. We are obtaining more blood samples for further investigations and identification more correlations between biomarkers and FOP.

**Patient Consent:** Yes, I received consent

**Disclosure of Interest**: None declared

**Table 1 (abstract P257).** See text for description

Cytokine	Reference range	sJIA Me [IQR]	FOP Me [IQR]	Control group Me [IQR]
**IL-18**	0-732,7 pg/ml	1690,14 [361,18;3607]	0,01 [0,008; 0,05]	0,0194 [0,0032; 14,926]
**IL-1RA**	78-970 pg/ml	1267,43 [936,75;2292,75]	1342,36 [927,0; 1811,6]	404,77 [255,3; 506,08]
**TNFR2**	3,4-10,8 ng/ml	9,47 [4,46;24,68]	14,99 [5,49; 23,12]	0,35 [0,243; 3,496]

### P258. Partial usp18 deficiency leads to early onset childhood inflammation

#### A. Munk

##### Pediatrics, Icahn School of Medicine at Mount Sinai, New York, United States

###### **Correspondence:** A. Munk

**Introduction:** Type I interferonopathies are characterized by an overabundance of IFN-I, which cause a broad spectrum of clinical presentation. Ubiquitin Specific Peptidase 18 (USP18) plays an important role in regulating the IFN-I response by dampening the IFN-I signaling pathway. Autosomal recessive USP18 deficiency results in severe systemic inflammation and neurological anomalies, which is fatal in the perinatal period. In our study, we sought to understand the etiology of disease in three different patients with genetic variations in USP18 who exhibited early onset autoinflammatory clinical features.

**Objectives:** Assess USP18 variants expressed by patients, and characterize the function of individual USP18 proteins.

**Methods:** HEK293T cells were transiently transfected with USP18 variants, and expression of USP18 mRNA and protein were measured using qPCR and western blot, respectively. Cells were transiently transfected and stimulated with IFN-I and levels of pSTAT1 and pSTAT2 were assessed using western blotting and flow cytometry. USP18 -/- dermal fibroblasts were transduced with USP18 variants, and IFN-related genes were measured using qPCR.

**Results:** USP18 variants did not impair USP18 protein or mRNA expression. In contrast, we found that unlike WT USP18, the three distinct USP18 variants failed to prevent phosphorylation of STAT1 and STAT2 when stimulated with IFN-I.

**Conclusion:** Assessed genetic variations in USP18 variants are hypomorphic in its ability to prevent type I interferon signaling, and are likely causative of patient’s autoinflammatory clinical features.

**Patient Consent:** No, I have not receive consent

**Disclosure of Interest**: None declared

### P259. New safest approach to study fibrodisplasya ossificans progressiva by using urinary stem cells

#### I. Nikishina^1^, A. Borovikov^2^, V. Matkava^1^, S. Arsenyeva^1^, V. Tabakov^2^, M. Sharova^2^, I. Sermyagina^2^

##### ^1^Pediatric Department, V. A. Nasonova Research Institute of Rheumatology, ^2^Research Centre for Medical Genetics, Moscow, Russian Federation

###### **Correspondence:** I. Nikishina

**Introduction:** Fibrodysplasia ossificans progressiva (FOP) is an ultra-rare monogenic disease caused by a de novo pathogenic mutation in the *ACVR1* gene with population frequency 1:2000000. FOP characterized by catastrophic progression a number of flare-up episodes that resolved to heterotopic ossification. Non-invasive methods for obtaining culture from these patients could help in understanding of «new bone formation» mechanism.

**Objectives:** To analyze available approaches for future research to explore osteogenesis obtaining urinary stem cells (USC) from patients with FOP.

**Methods:** From 24-year period (1998 – 2022) we observed 41 patients (pts) with FOP. DNA sequencing coding regions of the ACVR1 gene were performed by the Sanger method in most of patients. Fresh urine samples were taken from 9 pts. USC were cultivated by protocol described in Falzarano and Ferlini work [1].

**Results:** Fresh urine samples were taken from 9 patients with FOP (age 3 to 19; 4 female, 5 male). Among them 7 pts have «typical» heterozygous missense substitution c.617G>A (p.Arg206His), ultra-rare heterozygous missense substitution in the ACVR1 gene were presented in 2 pts (1 –p.Gly356Asp, 1 - p.Gly328Glu). We obtain and depone USC lines from 3 (33%) patients for future investigations. In other cases, we couldn’t grow USC lines due to low cell’s number and/or poor adhesion (4, 44%), bacterial contamination (2, 22%). In the group where it failed, we can repeat obtaining USC lines in a next hospitalization without additional risk of complications.

**Conclusion:** The obtained USC lines from patients with FOP-specific mutations in the gene *ACVR1* are a good model for studying the role of BMP in heterotopic ossification, which is important not only for understanding the nature of FOP, but also the universal processes of neo-osteogenesis in other rheumatic and non-rheumatic diseases. Obtaining primary cultures from patients with FOP by the usage invasive manipulations (puncture or tissue biopsy) always come with additional life-threatening risks. We suggest that USC can be a good non-invasive approach to learning and understanding a mechanism of abnormal ossification «in vitro».

References.

1. Falzarano M. S., Ferlini A. Urinary stem cells as tools to study genetic disease: overview of the literature. Journal of clinical medicine. 2019. Т. 8. № 5. С. 627.

**Patient Consent:** Yes, I received consent

**Disclosure of Interest**: None declared

## Poster session: Juvenile dermatomyositis

### P260. Juvenile dermatomyositis with subclinical cardiac manifestations in a 10 year-old Libyan boy: a rare case

#### A. A. Abushhaiwia^1^, H. S.Alrabte^2^, Y. Elfawires^3^

##### ^1^paediatric Rheumatology , Faculty of medicine, University of Tripoli, Tripoli Children’s Hospital, ^2^Paediatric cardiology, faculty of Medicine, Tripoli University Tripoli Children Hospital, Libya, ^3^Paediatric Rheumatology, Faculty of Medicine,Tripoli university , Tripoli Children’s Hospital, Tripoli , Libya

###### **Correspondence:** A. A. Abushhaiwia

**Introduction:** Juvenile dermatomyositis (JDM) is a rare autoimmune disease in the paediatric population, that typically presents with symmetric proximal muscular weakness and classic dermatologic findings. Cardiac abnormalities are known to occur in JDM but are less common than other clinical features. Cardiac involvement is usually subclinical, including conduction abnormalities. A previously healthy 10-year-old boy is described who presented with SVT by holter ECG secondary to newly diagnosed JDM and all symptoms resolved following treatment with oral corticosteroids, IVIG and MTX SC

**Objectives:** To highlight the importance of early diagnosis and recognition of the index of suspicion of subclinical cardiac manifestations in patients with JDM

**Methods:** Based on the clinical, biochemical and imaging findings, a definite diagnosis of JDM was made by using the 2017 European League Against Rheumatism/American College of Rheumatology classification criteria for adult and juvenile idiopathic inflammatory myopathies.

**Results:** A previously healthy 10-year-old boy born to a non- consanguineous marriage and family history was not significant. He presented to P\ Rheumatology clinic with a 2-month history of gradual tiredness and weakness. Later on he had palpitation, breathlessness. Initially was diagnosed by cardiologist as a case of SVT by holter ECG. He not being able to sit, stand or ascend stairs or take turns in bed and severe myalgia since 2 months. After 4 weeks he developed a faint red rash on the upper half of his face, along with skin-colored papules on his hands and just below his right elbow. During symptom progression, there was dysphasia but no fever, weight loss, vision change, vomiting, or diarrhea. **On physical examination** his weight was 36kg (50th-75thpercentile) and his height 142cm (50th -75th percentile). His vital signs were normal included BP 100/65mmHg, HR 80 beats\min and RR 20 breaths\min. He was profoundly weak, with head lag. A faintly erythematous rash over the malar prominences, and hyperpigmented papules over the dorsal metacarpophalangeal and proximal interphalangeal joints he had (Gorton’s papules). Bilateral Flexion contractures involving almost every joint of upper and lower extremities were present; therefore, power in the extremities 4\5. Power of neck flexor and extensor was 2/5. Superficial reflexes, plantar response and sensory examination when performed were found to be normal. Other systemic examinations were normal. **CMAS SCORING SHEET; WAS 8\52.Investigations** performed showed WBC 8.3X103, HGB 11.5g\dl and platelets375X103. ESR was 14mm\hr, and CRP level was 3.4mg/dl was negative. Antinuclear Antibodies (ANA) was Positive high titer 1:640, dsDNA-abs was negative,u1RNP, Sm, SSA, SSB, SCL70, jo1 all were negative and RF was negative. CPK 3036u\l, LDH was 719 U/l ALT=144U/L, AST=268U/L.Chest X-ray, ECG and ECHO were normal, but holter ECG showed SVT, NCV&EMG were consistent with active myopathy and polymyositis. Based on clinical features, laboratory investigation and EMG the diagnosis of JDM was made. Patient was given oral steroids (1mg/kg/day) accompanied by methotrexate 15mg\m2\week sc, IVIG 2g\k for 6 months, supplemental vitamin D, and calcium. Following this treatment, improvement continued with normalisation of the holter ECG, disappearance of the skin rash and normalisation of CPK and LDH within 2 months no any symptoms of weakness, or rash, and had 5/5 strength in the muscle groups of both upper and lower extremities.

**Conclusion:** cardiovascular complications should be considered in any child with JDM. Undergo a routine cardiovascular risk assessment at the onset of diagnosis and the potential value of corticosteroids and IVIG for its treatment.

**Patient Consent:** Yes, I received consent

**Disclosure of Interest**: None declared

### P261. Juvenile dermatomyosirtis as a paraneoplastic phenomenon in a preadolescent boy ( case report)

#### M. A. Alessi^1^, N. Alquorain ^2^, J. M. Alqahtani^3^, A. A. Alrahim ^4^, R. A. Alrumaih^5^, N. S. Alsaif^5^

##### ^1^Saudi Arabia , Dammam , Department of Pediatric , King Fahad Hospital of the University, ^2^Dermatology, King Fahad Hospital of the University, ^3^Dermatology, King Fahad Hospital of University , College of Medicine, Imam Abdulrahman bin Faisal University, Dammam, Saudi Arabia., ^4^Dermatology, King Fahad Hospital of the University , College of Medicine, Imam Abdulrahman bin Faisal University, Dammam, Saudi Arabia., ^5^Dermatology, King Fahad Hospital of the University, College of Medicine, Imam Abdulrahman bin Faisal University, Dammam, Saudi Arabia., Dammam, Saudi Arabia

###### **Correspondence:** M. A. Alessi

**Introduction:** We report a rare case of Juvenile Dermatomyositis (JDM ),to highlight ultimate need for multidisciplinary team approach to make an early diagnosis of occult malignancy in a preadolescence . Also, to report the success of initiation of intravenous (IV) pulse steroids and IVIG in a parallel with Chemo radiotherapy as a treatment modality of our case as malignancy-associated JDM.

**Objectives:** A13 y/o Saudi boy was admitted due to skin rash , fatigue and Arthralgia for few weeks. He had progressing fatigue to difficulties on doing his usual activities . He reported difficulty of swallowing started 1 month ago. In addition he had progressive sense of nasal obstruction over 2 months time associated with mild bilateral epistaxis. There is weight loss around 4 kg over 1 month ,No fever.

He had Gottron’s papules , heliotrope rash, itchy extensive rash over Arms , genitalia , back. Nail capillaroscopy showed dilated and capillaries with areas of dropouts. He had positive Gower’s signs and proximal muscle weakness . Investigations : normal CBC, Ferritin 336 ng/ml , ESR 43 mm/h, CRP 1.5 mg/dl , (CPK) 4195 U/L, (LDH) 810, SGOT 186 U/L , SGPT 92 U/L , (ANA) was highly positive 1280, (SSA) was highly positive 841 , Myositis Specific Antibodies panel revealed high titer of NXP-2AB >100 , and other Autoimmune serology were negative .Pelvic girdle MRI revealed inflammatory myositis.

Based on the clinical and laboratory findings we made a diagnosis of JDM, his initial Childhood Myositis Assessment Scale was 31/52.Videofluoroscopic showed no abnormality. So our patient fulfilled the Bohan and Peter criteria1 for JDM.

Due to dysphagia ENT evaluation done including nasopharyngoscopy that showed nasopharyngeal mass . CT head and neck showed right sided nasopharyngeal mass with foci of calcification and heterogenous no-vascular enhancement.

Biopsy was taken and histopathology reports undifferentiated nasopharyngeal carcinoma.

Multidisplaniray team meeting done between (Haemato-Oncology , Pediatric and adult Rheumatology and Dermatology services )

**Methods:** The presence of progressive nasal obstruction and epistaxis over short period in male pre-adolescent warrants need for detailed ENT assessment that showed the presence of nasopharyngeal mass.

The presence of cutaneous necrosis is highly associated with malignancy in adult dermatomyositis which was noticed in our patient clinically and histologically.(1). Many studies in adult population reported that cutaneous vasculitis is suggestive of associated malignancy which is in general a distinctive cutaneous features of JDM.(2,3). Therefore, cutaneous vasculitis might have no predicting value for malignancy in JDM, however some dermatological findings in DM patients are highly suggestive of underlying malignancy, including all signs of severe vasculitis(4).

Early pick up of these patients is crucial before steroid therapy as it can suppress the tumor growth and delay establishing the right diagnosis. Our patient went in complete remission after radiochemotherapy and JDM managment.

**Results:** Malignancy and DM have been identified as paraneoplastic phenomenon in adult population . But this phenomenon is rare in children , Hence paediatric patients are not routinely assessed for occult malignancy, so there is need of study aimed to analyze the clinical features of patients with JDM in order to identify predictors of malignancies in these patients.

**Conclusion:** Paediatric Rheumatologists should look for occult malignancy in adolescent age group who presented with atypical rash and JDM features.

**Patient Consent:** Not applicable (there are no patient data)

**Disclosure of Interest**: None declared

### P263. Withdrawn

### P264. Monocentric study of juvenile dermatomyositis

#### K. Bouayed, Y. Mahdar, A. Sakhi

##### Pediatric Rheumatology, CHU Ibn Rochd, casablanca, Morocco

###### **Correspondence:** K. Bouayed

**Introduction:** Although rare, juvenile dermatomyositis is the most common pediatric inflammatory myopathy. Studies concerning this condition in our country are almost non-existent.

**Objectives:** The objective was to describe the epidemiological, clinical, paraclinical, therapeutic and evolutionary profile of juvenile dermatomyositis.

**Methods:** This monocentric retrospective study, conducted between 2010 and 2020, enrolled 16 patients followed for juvenile dermatomyositis in the Department of Pediatric Rheumatology of the Ibn Rochd University Hospital in Casablanca.

**Results:** The mean age at diagnosis was 8 years (1-14 years); the sex ratio M/F was 0.7. The average diagnostic delay was 228 days; the disease was revealed by a typical skin involvement (75%); amyopathic forms were described (25%); joint involvement was found (93.75%); calcinosis was the most frequent complication (43.75%), followed by diffuse interstitial lung disease (37.5%). Muscle and liver function tests were disturbed in 81.25% and 62.5% respectively. Dermatomyositis DOT was performed systematically, showing the presence of anti-MDA5 antibodies in 50% of cases. Juvenile dermatomyositis was confirmed according to the criteria of Bohan and Peter (56.3%). Corticosteroid therapy was systematically used for muscle damage (87.5%). Methotrexate was the main background treatment 87.5% followed by cyclosporine 43.8%; hydroxychloroquine and/or mycophenolate mofetil were prescribed for severe skin involvement 18.75% respectively; cyclophosphamide for visceral complications 25%; immunoglobulins 37.5% and Rituximab 18.75% for refractory forms. Complete clinical and paraclinical remission was reported in 43.75% of cases. An osteoarticular tuberculosis occured in a patient with ciclosporin treatment.

**Conclusion:** Our series illustrates the phenotypic diversity of juvenile dermatomyositis as well as its severity. Joint involvement is predominant. Methotrexate seems to be ineffective in severe muscular forms. Early therapeutic intensification in refractory visceral and muscular forms improves the prognosis. Immunosuppressive drugs expose patients to infections, particularly tuberculosis in endemic areas. The diagnosis delay is long due to the lack of knowledge of this condition, which deserves the awareness of practitioners

**Patient Consent:** Yes, I received consent

**Disclosure of Interest**: None declared

### P265. The evaluation of nailfold capillaroscopy findings and its association with disease activity in juvenile dermatomyositis patients

#### S. Caglayan^1^, F. çakmak^2^, R. İşgüder^3^, K. Ulu^1^, E. Ünsal^3^, N. A. Ayaz^2^, B. Sözeri^1^

##### ^1^Pediatric Rheumatology, Umraniye Training and Research HospitaL, ^2^Pediatric Rheumatology, Istanbul University, Faculty of Medicine, Istanbul, ^3^pediatric rheumatology, Dokuz Eylül University, Faculty of Medicine, Izmir, Turkey

###### **Correspondence:** S. Caglayan

**Introduction:** Juvenile dermatomyositis (JDM) is a rare autoimmune vasculopathy that primarily affects the skin and muscle. It is characterized by skin findings such as heliotrope rash, Gottron papules, and proximal muscle weakness. Nailfold capillaroscopy (NFC) to evaluate microvascular involvement is a frequently used method in JDM patients

**Objectives:** This study aims to evaluate the NFC findings in patients with JDM and analyze its relationship with disease activity

**Methods:** This is a cross-sectional study involving pediatric patients who were diagnosed with JDM between 2014-2021, from three pediatric rheumatology centers in Turkey. Simultaneously at the date of NFC, patients were evaluated for disease activity with the physician visual analog scale (VAS), patient VAS, and childhood myositis assessment scale (CMAS). NFC was performed on eight fingers and at least four images were obtained from one finger. Capillary density, arterial width, venous width, apical loop, capillary morphology, presence of meandering capillary, microhemorrhage, avascular area, neoangiogenesis, and capillary ramification were evaluated from the images.

**Results:** 16 JDM and 25 control patients were included in the study. There were 11 females and 5 males in the patient group, and 13 females and 12 males in the control group and there was no statistically significant difference. The mean age of the patients was 10.4 ± 3.4 years in the patient group and 12.6 ± 3.6 years in the control group, and there was no statistically significant difference. The mean age at diagnosis was 7.1±4 years, and the mean follow-up period was 40.8±29 months. Initially, 6 (37.5%) of the patients had severe disease activity, 6 (37,5%) had moderate, and 4 (25%) had mild disease activity. During the follow-up, 6 patients (37.5%) showed monocyclic, 6 patients (37.5%) chronic persistent and 4 patients (25%) polycyclic course. The most common clinical findings were proximal muscle weakness (100%), myalgia (93.3%), heliotropic rash (93%), malar rash (93%), fatigue (87%), Gottron papule (81%), Gowers sign (80%), periorbital edema (60%) arthralgia (53.3%), calcinosis (25%), and arthritis (20%). respectively.

In patient group had an abnormality in at least one finding on NFC. Capillaryscopy findings of the patient and control groups are given in Table 1. Tortuosity and crossing capillary were non-specific changes that can be seen in the healthy control group, and there was no statistical difference between the two groups. There was no correlation between the CMAS and apical loop width, the presence of bizarre capillary, bushy capillary, microhemorrhage, avascular area, and neovascularization. Between the long time to diagnosis and the presence of apical loop width and bizarre capillary statistically significant difference was found (p=0,004, p=0,003 respectively). Avascular area and neoangiogenesis scores were higher in patients with high initial disease activity. There were nine patients in remission at the final follow-up. Even though they were in remission, 90% of the patients had dilated capillaries.

**Conclusion:** Performing NFC findings have critical importance at any stage of the disease in the evaluation of JDM patients.

**Patient Consent:** Yes, I received consent

**Disclosure of Interest**: None declared

**Table 1 (abstract P265).** Capillaryscopy findings of patient and control group

Capillaryscopy findings	Patient Group n(%)	Control Group n(%)	p
Capillary density (<6 capillary)	7 (43,7%)	0	0,000
Venous width (>20 mikron)	8 (53,3%)	1 (4%)	0,001
Dilated capillary	14 (87,5%)	4 (16%)	0,000
Giant capillary	4 (25%)	0	0,018
Meandering capillary	4 (25%)	0	0,000
Bushy capillary	9 (56,2%)	1 (4%)	0,000
Neoangiogenesis	14 (87,5%)	1 (4%)	0,000
Avascular area	10 (62,5%)	0	0,000
Microhemorrhage	7 (43,7%)	0	0,000

### P266. Clinical presentation and laboratory findings in patients with anti-mda 5 positive juvenile dermatomyositis-a single- center case series

#### A. Čengić^1^, V. Selmanović^1^, F. Hadzagić Ćatibušić^2^, S. Užičanin^2^, E. Vukas^2^, Z. Huseinbegović^2^, D. Pokrajac^3^, S. Hasanbegović^4^, V. Mišanović^5^, D. Anić^5^, Z. Begić^6^, A. Kadić^6^, M. Halimić^6^, S. Dizdar^6^, S. Kapić^6^, A. Džananović^7^, I. Pašić^7^, M. Bukvić^7^, A. Džinović^8^, A. Mustajbegović^3^

##### ^1^Allergology, Rheumatology and Clinical Immunology, ^2^Pedijatric Neurology, ^3^Pediatric nephrology, ^4^Endocrinology, ^5^intensive care unit, ^6^Cardiology, Pediatric Clinic, Clinical Centre University of Sarajevo, ^7^Pediatric radiology, Radiology clinic, Clinical university centre Sarajevo , ^8^Pulmology, Pediatric Clinic, Clinical Centre University OF Sarajevo, Sarajevo, Bosnia and Herzegovina

###### **Correspondence:** A. Čengić

**Introduction:** Juvenile dermatomyositis ( JDM) with positive anti-melanoma differentiation-associated gene 5 (anti-MDA5) antibody have been associated with distinct clinical phenotype which includes characteristic mucocutaneous features, small hand joint arthritis, mucus membrane and skin ulcers, palmar papules, less muscle involvement and severe interstitial lung disease (ILD). The clinical presentation of anti-MDA5 JDM differs substantially from the other forms of JDM.

**Objectives:** To describe the main presenting clinical and laboratory features of children with anti-MDA5 positive JDM followed in our Pediatric Rheumatology Unit**.**

**Methods:** Retrospective review of the clinical records of all patients with anti-MDA5 positive JDM who were treated in our center since January 2018 until May 2022. We followed fever, skin rashes that is characteristic for JDM, oral aphthae, palmar and plantar pustules, arthralgias, arthritis, weight loss, muscle weakness, CT verified ILD, C reactive protein, Sedimentation rate, aspartate aminotransferase(AST), alanine aminotransferase (ALT), lactate dehydrogenase (LDH), creatine kinase (CK), ANA, ANTI- Ro52

**Results:** Three patients were included in case series two of them were males. The median age at diagnosis was 9 years (range 2– 9 years). The median diagnostic delay was 2.5 months (range 1 month – 4 months). All patients had JDM characteristic skin changes, aphthous stomatitis, arthralgia, arthritis and weight loss at presentation. Only 1/3 patient had palmo-plantar pustules and 2/3 CT verified ILD. None of the patients presented with cutaneous calcinosis nor dysphagia and dysphonia. All patients had slightly elevated serum AST ( median value 145, normal range 15-59 U/L ), ALT ( median value 151, normal range 9-72 U/l ) LDH ( median value 453, normal range 100-190 U/l) and all patient had normal levels of creatine kinase and CRP. Sedimentation rate was slightly elevated in all children (median 35). Only one child had positivity for antinuclear antibodies and 2/3 for antiRo52. Magnetic resonance imaging was performed in all three patients and the results showed muscle changes compatible with dermatomyositis.

**Conclusion:** Anti-MDA5 JDM is a distinct subset of inflammatory myositis, with frequent skeletal and constitutional features such as intermittent fever, arthritis, weight loss, and less severe myositis, with strong association to ILD**.** Anti-MDA5 JDM is characterized by lower serum CK and slightly elevated other muscle enzymes.

**Patient Consent:** Not applicable (there are no patient data)

**Disclosure of Interest**: None declared

### P267. Severe vasculopathy of a case of juvenile dermatomyositis associated with anti-MDA5 antibody without pulmonary involvement

#### N. Choon Seong

##### Rheumatology, Hospital Selayang, Selayang, Malaysia

###### **Correspondence:** N. Choon Seong

**Introduction:** Anti-MDA5 antibody-positive juvenile dermatomyositis can have various subset of phenotype at presentation which could sometimes mimic other forms of autoimmune rheumatic disease.

**Objectives:** Here, we report a rare manifestation of a patient with juvenile dermatomyositis.

**Methods:** Case report.

**Results:** A 15 years old girl, previously well, presented initially as articular symptom (oligoarthritis of knees) following upper respiratory tract symptoms. The symptoms resolved with nonsteroidal antiinflammatory drug and antibiotic. Two months later, she was noted incidentally to have multiple cutaneous rashes typical of dermatomyositis but no other systemic manifestations as yet. However, three months later, she had altered sensorium followed by similar oligoarthritis of knees. It rapidly progressed to involve digital gangrenes and spontaneous thigh hematoma and mononeuritis multiplex. She also had polyarthralgia, fever and hyperkeratosis. ANA and dsDNA were negative with normal CK. Myositis panel showed positive anti-MDA5 associated with antiRo52. No interstitial lung disease was detected. No evidence of thrombotic microangiopathy. Cerebrospinal fluid was clear of infection. Brain MRI revealed posterior reversible leukoencephalopathy. CTA both upper and lower limbs did not reveal any aneurysm or thrombosis. She was given pulse steroid followed by pulse cyclophosphamide. Her symptoms improved thereafter.

**Conclusion:** It is important to recognize such severe systemic phenotype of anti-MDA5 patients to allow early intervention and better survival of patients.

**Patient Consent:** Yes, I received consent

**Disclosure of Interest**: None declared

### P268. Assessing the impact of a family day for young people and their families living with juvenile dermatomyositis (JDM) in the Northwest of England, UK

#### V. Cuthbert^1^, N. Woodend^2^

##### ^1^Paediatric Rheumatology, Royal Manchester Children’s Hospital, ^2^Paediatric Rheumatology, Royal Manchester Children;s Hosital & Versus Arthritis, Manchester, United Kingdom

###### **Correspondence:** V. Cuthbert

**Introduction:** JDM is a rare, autoimmune disease affecting primarily the muscles and skin and there are approximately 2-4 cases per million children and young people. JDM can have a huge impact on the lives of the young people, their siblings and families and many young people and families have never met others with the condition.

**Objectives:** To organise the first family day for young people and families living with JDM in the Northwest of England, UK and to assess the impact of the day.

For Young people and their families to feel less isolated living with JDM

For Young people and siblings get to take part in an activity to build their confidence

For Young people and their families receive information about JDM and living with the condition

**Methods:** Invitations were sent out to young people and families with JDM who attended Alder hey Children’s Hospital, Manchester Royal Children’s Hospital, Sheffield Children’s Hospital and Leeds children’s Hospital in the UK. The family day was organised outside of a hospital setting at the Chill Factor in Manchester. The young people were invited to enjoy snow activities, a science workshop, arts and crafts and soft play, as well as enjoy a fabulous lunch! A programme of speakers was organised and included a specialist nurse from Great Ormond Street Children’s hospital, discussing her current JDM research; a Manchester patient talked about the lived experience of having JDM and entering adulthood and the transition experience into adult healthcare. A Question and answer session was organised for young people and families to ask questions to a multi-professional panel.

**Results:** Feedback from questionnaires filled out on the day and email responses suggest the young people and families found the day beneficial and benefited from meeting up with other families and sharing experiences. The feedback suggested they found the education sessions and tips gained helpful and would aid management of the condition. Feedback suggested that the young people and their siblings had fun! Meeting others with the same condition was frequently mentioned in the feedback as very beneficial and helped to reduce feelings of isolation. Feedback suggests that the majority of young people with JDM and families would like to keep in touch with other JDM families they met.

**Conclusion:** There is evidence from the feedback gained from the family day for JDM patients and families that the families found the day beneficial: Meeting others with the same condition in the same region of the UK helped to reduce feelings of isolation and there was enthusiasm for keeping in touch and for further similar events.

**Patient Consent:** Not applicable (there are no patient data)

**Disclosure of Interest**: None declared

### P269. Myopathy with anti-3-Hydroxy-3-Methylgltaryl-coenzime a redutase antibodies in an adolescent

#### J. Lima^1^, S. Alves^1,2^, M. Santos^3^, C. Zilhão^1,2^

##### ^1^Pediatric Department, ^2^Pediatric Rheumatology Unit, ^3^Pediatric Neurology Unit, Centro Materno Infantil do Norte, Centro Hospitalar Universitário do Porto, Porto, Portugal

###### **Correspondence:** J. Lima

**Introduction:** Idiopathic Inflammatory Myopathies (IIM) may present with different clinical phenotypes, although the finding of myositis specific antibodies often leads to similar clinical manifestations, response to therapy and prognosis. Antibodies against 3-hydroxy-3-mehylglutaryl-coenzime A reductase (HMGCR) have been recently associated with Immune-mediated Necrotizing Myopathy (IMNM) in pediatric patients, accounting for 1% of all pediatric IIM. It is classically characterized by progressive proximal weakness, highly elevated serum creatine kinase (CK) levels, typical histopathological features of an IMNM, and poor response to therapy. Although cutaneous involvement is exceptional in adults, juvenile dermatomyositis (JDM)-like manifestations can develop in children.

**Objectives:** To highlight IMNM as a rare but relevant differential diagnosis to JDM, through the report of an adolescent with anti-HMGCR myopathy.

**Methods:** Case report.

**Results:** We report a 16-year-old male patient with a 5-month history of progressive symmetrical proximal limb weakness, more noticeable in the morning, hindering dressing, climbing stairs, and getting out of bed. He denied any other symptoms including joint swelling, fever, dysphagia, respiratory or gastrointestinal manifestations. Physical exam was remarkable for Gottron sign on the knees and pronounced symmetrical proximal weakness (Childhood Myositis Assessment Scale (CMAS) of 11/52 points) with severe limb muscle atrophy. Cervical involvement was less pronounced. Laboratory evaluation revealed a markedly elevated CK (13469 U/L), AST (319 U/L), ALT (416 U/L) with normal inflammatory markers. Myositis autoantibody panel revealed positive anti-Ku. Thigh MRI was compatible with myositis and electromyography consistent with a myopathy. Electrocardiogram and echocardiogram were normal. Muscle biopsy showed predominant perifascicular atrophy with scattered fiber necrosis and upregulation of MHC-1, findings consistent with JDM. Treatment with oral prednisolone (1mg/Kg/day) and subcutaneous methotrexate (MTX) (20mg/wk) was started, in parallel with physical rehabilitation. One month later, only slight clinical improvement was achieved (CMAS 18 points; CK 5246 U/L), and intravenous immunoglobulins (0,8g/Kg/month) was started. Throughout the subsequent 6 months, clinical and MRI improvement ensued, reaching a CMAS score of 52/52. Though asymptomatic, muscular enzymes persisted elevated (lower CK level of 685 U/L), without the possibility of weaning prednisolone under 12,5mg/day, whereby it was decided to switch from MTX to azathioprine. As clinical and analytical worsening followed, extended antibodies panel was performed revealing anti-HMGCR (>200U/mL; reference value <20). Attending to the previous clinical response and expert recommendations, treatment with MTX was restarted, and we ponder treatment with rituximab in case of clinical worsening following new corticosteroid weaning.

**Conclusion:** Anti-HMGCR antibodies are not always included in the commercial myositis panel, and this possibility should be considered in pediatric IIM patients with an indolent course, severe presentation or treatment refractoriness, even when cutaneous findings are present. We report a challenging case of a progressive myopathy with extremely high CK levels, that persisted beyond clinical improvement, with therapeutic implications. Recent studies have shown that increased CK levels do not always correspond to clinical aggravation in anti-HMGCR myopathy, making the decision of whenever to change or escalate therapy challenging.

**Disclosure of Interest**: None declared

### P270. Mandibuloacral dysplasia -a genetic mimic of juvenile dermatomyositis

#### N. Maldar, A. Khan, A. Prabhudesai, R. Khubchandani

##### Pediatric Rheumatology, NH SRCC Children’s Hospital, Mumbai, India

###### **Correspondence:** N. Maldar

**Introduction:** Mandibuloacral dysplasia Type A (MAD A; OMIM 248370), is a form of progeroid laminopathy syndrome. It is characterized by growth retardation, craniofacial anomalies, lipodystrophy with progressive osteoporosis and localised osteolysis. Pigmentary skin changes in addition to metabolic dysfunction are the other complications noted. A homozygous or compound heterozygous mutation in the gene encoding lamin A/C (LMNA) or ﻿ ZMPSTE24 (FACE1) gene, causes type A or type B MAD, respectively. Juvenile dermatomyositis (JDM) is an autoimmune condition characterized by muscle weakness and skin changes. At least 10% of these patients have acquired lipodystrophy.

**Objectives:** Laminopathies like MADA with a phenotype of generalised lipodystrophy with pigmentary changes can mimic JDM. We report a case with a novel mutation in the LMNA gene mimicking JDM.

**Methods:** A 3-year-old Indian girl, born of 3^rd^ degree consanguinity, was referred to our rheumatology clinic as a suspected JDM. She had failure to thrive [weight: 8.6kg (<1^st^ centile); height 82.5cm (3^rd^ - 50^th^ centile)], pigmentary skin changes and proximal muscle weakness. From two years of age, there was history of progressive generalised skin hyperpigmentation with multiple hyperkeratotic flat maculopapular lesions over of the bony prominences in both upper and lower limbs. Hypopigmented scaly lesions on the buttocks, subtle heliotrope rash around the eyes and sparing of the oral mucosa and the arm pits with no signs of erythema or pruritus were noted. Craniofacial dysmorphism in the form of mandibular dysplasia with facies looking advanced for age were present. She had prominent eyes, a thin nose, crowded teeth, sparse hair on the scalp, with hypotrichosis over the extremities. The nails appeared dystrophic and the terminal phalanges were small with bulbous ends. On musculoskeletal examination, the muscle bulk and tone appeared normal. There was diffuse muscle weakness in the lower limbs with difficulty in rising from sitting position and generalised loss of subcutaneous adipose tissue of the with acral lipodystrophy. Nervous system examination was normal and development milestones were appropriate for age.

**Results:** Lab investigations revealed normal hemogram and sedimentation rate (10mm/hr), and deranged muscle enzymes with creatinine phosphokinase 867U/L, lactate dehydrogenase 405U/L, aspartate aminotransferase 62U/L and alanine aminotransferase 64U/L. Antinuclear antibody (by immunofluorescence) and myositis antibody profile were negative. Lipid profile and HbA1C were deranged (Cholesterol 170mg/dL, HDL 51 mg/dL, LDL 85 mg/dL, VLDL 33.6 mg/dL, Triglycerides 168 mg/dL, HbA1C 6.4). MRI quadriceps was normal while roentgenogram showed acro-osteolysis of distal phalynges with wormian bones in the lambdoid sutures and mandibular hypoplasia. There was no cardiomyopathy on echocardiography. A provisional diagnosis of atypical JDM was made. But while initiating steroids and methotrexate we were mindful of the atypical features. Suspecting a JDM mimic such as chronic atypical neutrophilic dermatosis with lipodystrophy and elevated temperature (CANDLE) syndrome, whole exome sequencing was done. It showed a homozygous missense mutation in exon 9 of the LMNA gene (c.1583C>T) (p.Thr528Met) on chromosome 1q22 with sanger sequencing confirming both parents to be carriers. Immunomodulatory therapy was withdrawn, and the family counselled.

**Conclusion:** On reverse phenotype match, our case had the classic features of MAD. However, the raised muscle enzymes with proximal muscle weakness, and skin lesions could mimic JDM leading to an unwarranted initiation of immunomodulatory therapy. Therefore genetic testing and counselling acquires relevance in establishing accurate diagnosis and preventing mishaps in subsequent pregnancies.

**Patient Consent:** Yes, I received consent

**Disclosure of Interest**: None declared

### P271. Pres juvenile dermatomyositis working group (JDM WG) achievements, September 2021-September 2022

#### M. G. L. Wilkinson^1^, C. Papadopoulou^1^, S. R. Veldkamp^2^, R. Campanilho-Marques^3^, S. Röstlund^4^, M. Nørgaard^5^, J. H. K. Swan ^6^, O. Kul Cinar^7^, H. Sanner^8^, L. J. McCann^9^

##### ^1^UCL Great Ormond Street Institute of Child Health, London, United Kingdom, ^2^ Center for Translational Immunology, University Medical Center Utrecht, Utrecht, Netherlands, ^3^Pediatric Rheumatology Unit - Rheumatology Department, Hospital de Santa Maria, Centro Hospitalar Universitário Lisboa Norte, Centro Académico de Medicina de Lisboa, Lisbon, Portugal, ^4^ Women´s Health and Allied Health Professionals Theme, Medical Unit Occupational therapy and Physiotherapy, Karolinska University Hospital, Solna, Sweden, ^5^Department of Physiotherapy (Pediatrics), Aarhus University Hospital, Aarhus, Denmark, ^6^Parent and patient representatives, Dundee, ^7^ UCL Great Ormond Street Institute of Child Health, London, United Kingdom, ^8^Department of Rheumatology, Oslo University Hospital , Oslo, Norway, ^9^Alderhey Children’s NHS Foundation Trust, Liverpool, United Kingdom

###### **Correspondence:** L. J. McCann

**Introduction:** The PReS Juvenile Dermatomyositis (JDM) working group (WG) brings together clinicians and researchers to improve knowledge, promote good practice and facilitate research in JDM.

**Objectives:** To describe the workings of the group from September 2021-September 2022.

**Methods:** The PReS JDM working party has 241 active members. A core group of elected / co-opted representatives meet 6x year. Meetings for all members are held 3x year; including one during the PReS annual conference.

**Results:**

Since the PReS conference 2021, work has been on-going in line with PReS pillars (Table 1).

**ACROSS ALL PILLARS: INPUT FROM ALLIED HEALTH PROFESSIONAL (AHP) & PARENT/PATIENT REPRESENTATIVES.**

**Conclusion:** The PReS JDM WP has been active in collaborative projects to enhance clinical care, translational research and education / training on an international platform. The working group benefits from inclusion of co-opted patient/parent and Allied Health Professional (AHP) representatives to ensure a holistic ethos to improve outcomes for children and young people with JDM.

**Patient Consent:** Not applicable (there are no patient data)

**Disclosure of Interest**: None declared

**Table 1 (abstract P271).** See text for description

Clinical care: Lead – C. Papadopoulou	Science & research: Lead – S. Veldkamp	Education & training: Lead – R. Campanilho-Marques	Collaborative working: Lead – L.McCann / H. Sanner
Study of sleep / fatigue in JDM. Work in progress for revised grant submission (CP).	Collaborative opportunities in basic science /translational research.	Podcast - Myositis Specific Antibodies available on PReS website JDM WP tab (as well as narrated PowerPoint on MSAs & training tools for MMT8 / CMAS).	CARRA / PReS JDM WP SIG: Study on physical activity in JDM - Research proposal circulated for comments April 2022 (HS).
Survey of practice in JDM / SHARE survey – write up in progress (OKC).	Updates given during meetings.	Preparing MCQ questions for PReS Knowledge Based Exam.	CARRA / PReS JDM WP SIG: Telemedicine in JDM – survey in progress.
Publication: Rational for a position statement on Developmentally appropriate Transitional Care during the Covid pandemic: McDonagh J et al, Pediatric Rheumatology (2021) 19: 136. https://doi.org/10.1186/s12969-021-00609-y	Open educational session Nov 2021: What’s wrong with tools in JDM (Brian Feldman).	Planning JDM course 2023.	IMACS / PReS JDM WP SIG: Extension of the Single Hub & Access point for paediatric Rheumatology in Europe (SHARE) consensus guidance; survey prepared, to be distributed after IRB approval.

Abbreviations: CARRA - Childhood Arthritis & Rheumatology Research Alliance. SIG – Special Interest Group. IMACS - International Myositis Assessment & Clinical Studies Group

### P272. Clinical presentation, risk factors and prognosis of MDA5-positive JDM; clinical diversity and red flags

#### K. Mclellan, M. Al-Obaidi, S. Compeyrot-Lacassagne, C. Pilkington, E. Moraitis, C. Papadopoulou

##### ^1^Paediatric Rheumatology, Great Ormond Street Hospital, London, London, United Kingdom

###### **Correspondence:** K. Mclellan

**Introduction:** MDA5-positive juvenile dermatomyositis (JDM) represents a distinct clinical phenotype associated with skin and oral ulceration, milder muscle involvement and a higher incidence of interstitial lung disease (ILD) and severe rapidly-progressive ILD (RP-ILD).

**Objectives:** To describe the presenting clinical characteristics of patients with MDA5-positive JDM, the incidence of ILD and risk factors associated with development of RP-ILD.

**Methods:** Retrospective clinical notes review of patients with MDA5-positive JDM managed at Great Ormond Street Hospital (GOSH) over a 24-year period.

**Results:**

Demographics:

Twenty-five patients were managed at our centre with MDA5-positive JDM. One additional patient was excluded as no notes were available. Sixty-four % were females. In terms of ethnicity, 60% White British, 20% Black African, 8% Mixed, 8% Asian, 8% European and 4% South Asian. Median age of presentation was 10.0 years (IQR 6.9, 12.3). Diagnostic delay was significant amongst this cohort with a median 14 weeks (8, 26) between initial symptoms and diagnosis. Twenty-four% of this cohort were also Ro-52 positive.

Presentation:

All patients had skin involvement; 52% had ulcerating skin disease, 64% had heliotrope rash and 100% had Gottrons papules on initial presentation. Forty-four % had peri-ungal changes, 35% had digital pitting or infarcts and 64% had nailfold changes. Forty % had peri-orbital oedema. Muscle involvement was present in all, with median CMAS 40/52 (36,44) and MMT8 57/80 (51,62). Eighty-eight % had arthritis, of whom an initial active joint count was available for 16 patients with a median of 5.5 joints (3,16). Forty % had gastrointestinal symptoms; fever, weight loss and mouth ulcers were present in 52%, 52% and 60% respectively. Twenty-four % had respiratory symptoms at diagnosis.

Interstitial lung disease:

Fifty-two % were diagnosed with ILD and a further 20% had abnormal pulmonary CT scans not thought to be diagnostic of ILD. 13% (2 patients) had RP-ILD; both of whom died despite immunosuppression and extracorporeal membrane oxygenation. There were no other deaths amongst this cohort. No respiratory involvement was identified in 28%. Of 19 patients who had a CT chest at diagnosis, 84% were abnormal. Only 1 patient developed respiratory involvement during the disease course whilst on treatment; the other 93% with ILD had evidence of ILD at diagnosis. Four risk factors were found to be associated with development of RP-ILD which reached statistical significance, a further 3 factors were significantly protective, as is shown in Table 1, out of 58 putative variables. 2 patients had pneumocystis pneumonia (PCP) , both of whom required admission to PICU and one associated with death.

Table 1 shows risk factors at presentation which were found to be significantly associated with RP-ILD

**Conclusion:** Skin and muscle involvement were identified in all patients, with the majority also presenting with ulcerating skin disease, oral ulceration and arthritis. The majority of this cohort had lung involvement and 2 patients died of RP-ILD. Four risk factors were found to be predictive of RP-ILD and a further 3 protective factors were identified. Rapid deterioration of respiratory symptoms was associated with PCP, while ILD was unlikely to develop whilst on treatment if not present at diagnosis. These findings need to be validated in a larger cohort of patients.

**Disclosure of Interest**: None declared

**Table 1 (abstract P272).** See text for description

	Factor	p value
**Risk factor**	Periungal erythema	0.024
	Heliotrope rash	0.06
	Respiratory symptoms	0.017
	Haemoglobin	0.0129
**Protective factor**	Normal CT	0.01
	Normal lung function	0.008
	Normal CXR	0.005

### P273. Clinical spectrum of anti MDA-5 autoantibody associated juvenile dermatomyositis from a tertiary-care centre in Northern India

#### P. L. Nadig, P. Vignesh, S. Basu, R. Tyagi, M. Sudhakar, S. Sharma, M. Dhaliwal, A. Rawat, S. Singh

##### Pediatrics, Post Graduate Institute of Medical Education and Research, Chandigarh, Chandigarh, India

###### **Correspondence:** P. L. Nadig

**Introduction:** With the recognition of myositis specific autoantibodies, distinct clinical phenotypes of juvenile dermatomyositis (JDM) now been identified. Anti–MDA-5 autoantibody associated juvenile dermatomyositis is associated with clinically amyopathic form of disease and rapidly progressive interstitial lung disease (ILD). However, ethnic variations in disease manifestations have been noted.

**Objectives:** We describe a pediatric cohort of anti-MDA5 associated JDM from a tertiary care centre in North India.

**Methods:** We retrieved the medical records of Pediatric Rheumatology Clinic from January 1992 to April 2022 and did a retrospective analysis of 8 children with anti-MDA-5 antibody positive JDM. Clinical features including age at presentation, cutaneous manifestations, muscle weakness, presence of ILD, and other manifestations were noted. Myositis associated autoantibodies were assayed using a 16-antigen kit immunodot assay (Euroline Autoimmune Inflammatory Myopathies 16 Ag, Euroimmun, Lübeck, Germany).

**Results:** Of 53 children with JDMS who underwent testing for MSA, 8 had positivity for anti-MDA5 (15%). The latter were analyzed in detail. Mean age at onset of clinical manifestations was 8.4 years and mean age at diagnosis was 9.6 years. F:M ratio was 1:3. Mean follow-up period was 16.5 months. Cutaneous manifestations included photosensitivity (n=8), malar rash (n=8), shawl sign (n=2), heliotrope rash (n=7), and Gottron papules (n=8), inverse Gottron papules (n= 5) and skin ulceration ( n=5).

Muscle weakness was observed in 5 patients - 3 had severe truncal weakness and 2 had pharyngeal involvement. Arthralgia and arthritis were present in 4 and 5 patients respectively with involvement of both large and small joints. Two had Raynaud’s phenomena, and 1 had generalized adenopathy. ANA positivity was noted in 3 patients, 1 amongst these had anti-dsDNA positivity. Anti-Ro 52 positivity was noted in 2 patients. Of 6 patients who underwent HRCT chest, 3 patients had ILD; however, none amongst the latter had progressive disease on follow up. Treatment included oral corticosteroids (n=8), methotrexate (n=8), cyclophosphamide (n=2) and mycofenolate mofetil (n=2). Remission was seen in 5 out of 8 patients within a mean duration of 6 months after initiation of treatment. Three patients had relapses.

**Conclusion:** We report 3 significant findings from our cohort of anti-MDA5 JDM:

1. Rapidly progressive ILD as commonly noted with adult-onset anti-MDA5 associated JDM in South East Asian population, was not seen.
2. Involvement of large joints (arthralgia/ arthritis) was a common finding
3. Severe truncal weakness and pharyngeal involvement was noted

**Patient Consent:** Yes, I received consent

**Disclosure of Interest**: None declared

### P274. A pediatric case of intractable immune-mediated necrotizing myopathy treated with multi-targeted therapy

#### N. Okamoto^1,2^, Y. Sugita^1^, Y. Ozeki^1^, K. Shabana^1^, A. Ashida^1^

##### ^1^Department of Pediatrics, School of Medicine, Osaka Medical and Pharmaceutical University, Takatsuki-City, ^2^Department of Pediatrics, Osaka Rosai Hospital, Sakai-City, Japan

###### **Correspondence:** N. Okamoto

**Introduction:** We report the case of intractable Immune-mediated necrotizing myopathy treated with multi-targeted therapy.

**Objectives:** Immune-mediated necrotizing myopathy (IMNM) is first defined in 2003 by European Neuromuscular Center classification and is characterized with significant muscle weakness, muscle atrophy and hyper serum creatine kinase (CPK). Invasion of inflammatory cell in muscle tissue is modest and patients are often misdiagnosed as muscular dystrophy. The specific autoantibodies, anti-single recognition particle antibody and anti-3-hydroxy-3-methylglutaryl coenzyme A reductase (HMGCR) antibody, are identified. The continuous treatment including glucocorticoid, immunosuppressant, immunoglobulin and rituximab are necessary to control disease activity and prevent the deterioration of muscle atrophy.

**Methods:** A-5-year-old girl visited former hospital complaining acute muscle weakness and significant hyper serum CPK (35000 U/L) was revealed. She was transferred to our hospital and was diagnosed as IMNM by muscle biopsy and positivity of anti-HMGCR antibody (2.7 IU/ml). Blood test results; WBC 6210/μl, CPK 24635 U/L, aldorase 362U/L, AST 770 U/L, ALT 1199 U/L, LD 3491 U/L, ferritin 57.9ng/mL. Induction therapy was performed with methyl-prednisolone pulse therapy (30mg/kg/day, 3days in a week) followed by oral prednisolone and oral methotrexate 5mg/W (8mg/m^2^). CPK decreased to 3000-4000 U/L and muscle weakness improved, however the Gowers’s phenomenon was positive and she needed help for daily activity. Additional single intravenous immunoglobulin therapy (IVIG, 500mg/kg/day, 5 days) was effective for activity improvement and CPK decrease that was still high up to 900 U/L. Monthly intravenous cyclophosphamide (IVCYC 500mg/m^2^/day, 1 day/month) and mycophenolate mofetil (MMF 600mg/m^2^) were started due to disease activity and intolerance to methotrexate, however she complaint muscle weakness and CPK increased to 5000 U/L again. Instead of IVCYC, tacrolimus (TAC) 0.5mg/day was added and IVIG every 3 months was started, then CPK decreased to 1500-3000 U/L. Because muscle weakness appeared 1 month before each IVIG, rituximab (RTX) was performed. Since then, her disease is well controlled.

**Results:**

**Conclusion:** Multi-targeted therapy including MMF, TAC, IVIG and RTX was effective for intractable pediatric case of IMNM with anti-HMGCR antibody positivity. The written informed consent for the publication was obtained from her mother.

**Patient Consent:** Yes, I received consent

**Disclosure of Interest**: None declared

**Table 1 (abstract P274).** See text for description

	onset	after induction	before single IVIG	After single IVIG	Before MMF switch	Before TAC and regular IVIG add	Before RTX	6month after RTX
CKP (U/L)	24635	3516	2979	883	1473	5781	2507	1835
ALD (U/L)	362	83.4	64.0	16.1	26.7	101.4	44.6	25.9

### P275. Myositis specific autoantibodies in juvenile idiopathic inflammatory myositis: our experience from a tertiary care Centre in North India

#### V. Pandiarajan^1^, A. Rawat^1^, P. Nadig^1^, R. Kumrah^1^, A. Dod^1^, R. Garg^1^, R. Pilania^1^, A. Jindal^1^, D. Suri^1^, S. Singh^1^

##### ^1^Allergy Immunology Unit, Department of Pediatrics, Postgraduate Institute of Medical Education and Research, Chandigarh, India

###### **Correspondence:** V. Pandiarajan

**Introduction:** Distinct clinical phenotypes of juvenile idiopathic inflammatory myositis (JIIM) have been identified based on the myositis specific autoantibodies (MSAs). However, the spectrum of MSAs and clinical phenotypes within the particular category of MSA can vary amongst different population groups.

**Objectives:** To analyse the spectrum of MSAs in children with JIIM and compare the clinical phenotypes in a cohort of JIIM from North India.

**Methods:** We retrieved the records of 53 children with JIIM who underwent testing for MSA and had been followed up in the Pediatric Rheumatology Clinic, Advanced Pediatric Centre, Post Graduate Institute of Medical Education and Research, Chandigarh, India during the period January 2019- April 2022. Laboratory assay for MSAs was performed using a 16-antigen kit immunodot assay (Euroline Autoimmune Inflammatory Myopathies 16 Ag, Euroimmun, Lübeck, Germany). Results that were 2+ or more were considered positivity for MSA.

**Results:** Of the 53 children with JIIM who underwent testing for MSA, 41 (77.3%) had positivity for MSA. Positivity for anti-TIF-gamma, anti-NXP2, anti-MDA5, and anti-SAE were noted in 5 (9.4%), 13 (24.5%), 8 (15%), and 4 (7.5%) children, respectively. Anti-synthetase syndrome with anti-Jo positivity was noted in 1 child. Positivity for anti-PM-Scl was noted in 5 children. Anti-SRP positivity was noted in a child with polymyositis. Of the anti-NXP2 positivity group, 2 had calcinosis-predominant presentation of juvenile dermatomyositis (JDM) with minimal muscle weakness and complete paucity of other cutaneous features of JDM. Amongst the MDA5 positivity group, clinically amyopathic form of JDM and rapidly progressive interstitial lung disease (ILD) was not seen. However, involvement of large joints, severe truncal weakness and pharyngeal involvement were noted in majority of them. Cutaneous relapses were commonly noted in the anti-TIF-gamma positivity group. Of the 4 children with anti-SAE positivity, only 1 had clinically amyopathic form of disease.

**Conclusion:** Anti-NXP2 is the most common MSA noted in our cohort of JIIM from North India, followed by anti-MDA5, anti-PM-Scl, and anti-TIF-gamma. Anti-MDA5 positivity in our cohort is not associated with clinically amyopathic JDM or rapidly progressive ILD. Anti-SAE positivity is noted at a higher proportion (7.5%) in our cohort compared to studies reported from other regions.

**Patient Consent:** Yes, I received consent

**Disclosure of Interest**: None declared

### P276. Defining criteria for disease activity States in Juvenile dermatomyositis based on the juvenile dermatomyositis activity index

#### S. Rosina^1^, A. Consolaro^1,2^, A. Pistorio^3^, A. Rebollo-Giménez^1^, N. Ruperto ^1^, A. Ravelli^3^

##### ^1^UOC Clinica Pediatrica e Reumatologia, IRCCS Istituto Giannina Gaslini, ^2^Dipartimento di Neuroscienze Riabilitazione Oftalmologia Genetica e Scienze Materno-Infantili, Università degli studi di Genova, ^3^Direzione Scientifica, IRCCS Istituto Giannina Gaslini, Genova, Italy

###### **Correspondence:** S. Rosina

**Introduction:** The Juvenile DermatoMyositis Activity Index (JDMAI) is the first composite disease activity score validated for the measurement of muscle and skin involvement in juvenile dermatomyositis (JDM). Six preliminary versions (JDMAI1 to 6) have been proposed. Both JDMAI1 and JDMAI2 include the physician’s global assessment of overall disease activity (PhGA) on a visual analogue scale (VAS) (where 0 = no activity and 10 = maximum activity), the parent’s global assessment of child’s overall wellbeing (PaGA) on a VAS (where 0 = best and 10 = worst), and the hybrid MMT8/CMAS (hMC) expressed in deciles (0 = best to 10 = worst). To estimate the activity of skin disease, the JDMAI1 includes the skin activity VAS (where 0 = no activity and 10 = maximum activity), whereas the JDMAI2 includes the skin component of the Disease Activity Score (DAS) (score range 0 = no activity to 9 = maximum activity). The theoretical range is, therefore, 0 to 40 for the JDMAI1 and 0 to 39 for the JDMAI2.

**Objectives:** Cutoffs for the states of inactive disease (ID), low disease activity (LDA), moderate disease activity (MDA) and high disease activity (HDA) are necessary to interpret the JDMAI scores. The aim of the study was to develop and validate these cutoffs for JDMAI1 and JDMAI2.

**Methods:** The cutoffs definition cohort was composed of 129 patients included in the PRINTO JDM trial and evaluated at 6 months from baseline. Optimal cutoff values were determined against external criteria by calculating the 10^th^ and 25^th^ percentile (for ID), the 30^th^ and 40^th^ percentile (for LDA), and the 75^th^ and 90^th^ percentile (for HDA) of cumulative score distribution and through receiver operating characteristic curve analysis. External criteria included the modified PRINTO criteria for clinically inactive disease (for ID) and the PRINTO/ACR/EULAR level of improvement (for LDA and HDA). MDA cutoffs were defined as the score interval between LDA and HDA cutoffs. The choice of final cutoffs was based on clinical and statistical grounds. The validation cohort included 213 patients followed up in standard clinical care at 13 international paediatric rheumatology centres, and 275 patients enrolled in a study aimed to validate prospectively the provisional PRINTO/ACR/EULAR disease activity core set for the evaluation of response to therapy in JDM. Cutoff validation was conducted by assessing discriminative ability.

**Results:** The selected JDMAI1 cutoffs were <=2.4 for ID, <=6.6 for LDA, 6.7-11 for MDA, and >11 for HDA. The selected JDMAI2 cutoffs were <=5.2 for ID, <=8.5 for LDA, 8.6-11.3 for MDA, and >11.3 for HDA. In cross-validation analyses, the cutoffs showed strong ability to discriminate among disease activity states defined subjectively by physicians and parents, parents’ satisfaction/dissatisfaction with illness outcome, levels of child’s pain and fatigue, and presence/absence of functional impairment and disease damage.

**Conclusion:** Cutoff values for classifying various disease activity states in JDM using the JDMAI1 and JDMAI2 were developed. The cutoffs revealed good metrologic properties in both definition and validation samples, and are therefore suitable for application in clinical practice and research.

**Patient Consent:** Not applicable (there are no patient data)

**Disclosure of Interest**: None declared

### P277. Comparison between electromyography and whole-body mri in the assessment of disease activity in juvenile dermatomyositis

#### M. Rossano, F. Baldo, F. Chironi, G. B. Beretta, G. Rogani, I. Borzani, F. S. Minoia, A. M. Cappellari, G. Filocamo

##### Fondazione IRCCS Ca’ Granda Ospedale Maggiore Policlinico, Milan, Italy

###### **Correspondence:** M. Rossano

**Introduction:** Juvenile dermatomyositis (JDM) is a multisystem inflammatory disease involving primarily the skin and proximal muscles. One of the main challenges in the clinical management of JDM is accurate assessing of disease activity to optimize treatment and reduce disease morbidity.

**Objectives:** The aim of this preliminary study is to understand the role in assessing disease activity of a validated electromyography (EMG) scoring protocol and a whole body MRI score (WB-MRI), by comparison with the clinical standardized evaluation as gold standard.

**Methods:** A total of 17 patients with JDM seen at our hospital between Oct. 2018 and Dec. 2021 were enrolled. Each patient underwent concomitantly clinical evaluation based on hybrid MMT/CMAS (hMC) score, laboratory exams including creatinine kinase (CK), aspartate aminotransferase (AST) and lactic dehydrogenase (LDH) levels, EMG and WB-MRI. For the purpose of the analysis the EMG score was calculated as sum of two subsets: acute denervation signs (fibrillation potentials) with score ranging from 0 (maximal inflammation) to 8 (no inflammation) and reinnervation signs (motor unit remodeling) with a score ranging from 0 (no reinnervation) to 8 (maximal reinnervation). WB-MRI was scored in 42 muscular groups using a 0-2 point scale for each group; myofascial and subcutaneous tissue inflammation were assessed on the upper and lower extremities using a 0-1 point scale. A muscular visual analog scale (VAS) was defined by an expert rheumatologist: the disease was considered clinically inactive in case of VAS ≤ 0,5. Due to the choice to focus our study mainly on muscular involvement, our definition of inactive disease did not take into account PRINTO definition which includes also skin involvement. Correlations were assessed by Spearman’s rank order correlation coefficient (rs). Comparison of quantitative variables in the analysis of discriminant validity was made by Mann–Whitney U Test. Cohen’s kappa was used to evaluate the agreement between physician muscular VAS and EMG and MRI scores.

**Results:** Seventeen patients (82.3% female) were enrolled for a total of 27 examinations considered in the study (7 at disease onset, and 20 follow-up visits). The median age at JDM diagnosis was 6.59 years (IQR 3.49-10.1), and the median duration of follow-up was 3.56 years (IQR 1.43-6.89). In 15 patients JDM satisfied Bohan and Peter’s criteria, while 2 patients were classified as amyopathic JDM. The hMC revealed a high correlation with the EMG score (rs=0.63), compared to the moderate correlation observed with MRI muscle score (rs =-0.47) and MRI myofascial score (rs =-0.43), and to the poor correlation with MRI subcutaneous score (rs =-0.11). The EMG score, and its single subsets (fibrillar potentials and remodeling), as well as the MRI muscle score differed significantly between patients with active or inactive disease according to the muscular VAS (p= 0.015, p=0.001, p=0.015, p = 0.004), proving a good discriminative ability. Overall agreement was substantial between EMG fibrillar score and muscular VAS (k= 0.701), and moderate between MRI muscle score and muscular VAS (k= 0.482).

**Conclusion:** This preliminary data suggest a good association between the EMG score and clinical evaluation of muscular disease activity, endorsing the role of EMG in the management of JDM. Although at present standardized clinical evaluation remains the gold standard, EMG and WB-MRI were proved to have a fair discriminative power compared to clinical assessment of disease activity. Further prospective analysis on larger samples may contribute to better define the role of both EMG and WB-MRI in evaluating muscular disease activity.

**Patient Consent:** Yes, I received consent

**Disclosure of Interest**: None declared

### P278. Early-onset juvenile dermatomyositis: a tertiary referral center experience and review of the literature

#### S. Sener^1^, O. Basaran^1^, E. D. Batu^1^, E. Sag^1^, S. Oz^2^, S. Sari^2^, Y. Bilginer^1^, G. Haliloglu^2^, S. Ozen^1^

##### ^1^Pediatric Rheumatology, ^2^Pediatric Neurology, Hacettepe University Faculty of Medicine, Ankara, Turkey

###### **Correspondence:** S. Sener

**Introduction:** Juvenile dermatomyositis (JDM), the most common idiopathic inflammatory myopathy of childhood, is quite heterogeneous in terms of disease course.

**Objectives:** The aim of our study was twofold: To evaluate the presentation, diagnosis, clinical course and management of JDM in children less than three years of age and to compare with the late-onset patients, and to review the literature for early-onset JDM.

**Methods:** Nine patients with the early-onset disease and 63 patients with the late-onset disease with JDM followed between December 2010 and April 2022 were included in the study. We also reviewed the literature in terms of early-onset JDM from the inceptions of the PubMed/MEDLINE and Scopus databases up to April 1^st^, 2022.

**Results:** The median delay time between the symptom onset and diagnosis of the early- and late-onset JDM patients was 5.5 (2-31) months and 2.1 (0.2-25), respectively (p=0.05). While calcinosis was seen at a higher rate in the early-onset group (p=0.060), skin ulcers were also more common in this group, although not statistically significant (22.2% versus 3.1%, p=0.074). There was no difference in median serum creatinine kinase (CK) levels between the two groups (p=0.940). Anti-NXP2 was more common in the early-onset group (p=0.049). Muscle biopsy was frequently preferred in the diagnosis of early-onset JDM patients (77.7% versus 50.8%). In the treatment, corticosteroid and methotrexate (MTX) were frequently used in patients in both groups (p=1.000). In the early-onset group, intravenous immunoglobulin (p=0.001), cyclophosphamide (p=0.011) and biological agents (p=0.016) were used more frequently as a second and third-line therapy. Although there was no difference between the partial and complete remission rates, the relapse rate was significantly higher in the early-onset group (p=0.001). There was no mortality in either group during the follow-up. Of note, our early-onset JDM patients visited approximately 3 centers before the definite diagnosis of JDM. More than half of these patients had further investigations to rule out genetic, metabolic, and infectious myopathies.

Our literature search revealed 32 articles reporting 75 patients with JDM who were younger than 3 years of age. The median diagnostic time window was 5 (1-30) months. Skin ulcers were seen in 12.6% of patients, while calcinosis was present in 29.5%. Twenty-three of the 44 patients (52.3%) had a muscle biopsy for diagnostic purposes. Corticosteroids (98.7%) and MTX (91.1%) were main agents used in the treatment. Forty-one patients (64.1%) received treatment other than MTX/corticosteroids. Complete remission was achieved in almost half of these patients (48.9%), but relapse was observed in 75%. The mortality rate was 10.2%.

**Conclusion:** Diagnosis can be challenging and delayed in early-onset JDM patients. Furthermore, compared to later-onset JDM patients, this group had a higher relapse rate with a considerable requirement for intensive immunosuppressive treatment. Increasing the knowledge about clinical phenotype of early-onset JDM is crucial for timely introduction of disease-modifying treatments.

**Trial registration identifying number:** not applicable

**Patient Consent:** Yes, I received consent

**Disclosure of Interest**: None declared

### P279. Features of myositis-specific and myositis-associated antibodies and antinuclear antibody patterns in patients with juvenile dermatomyositis

#### S. Sener^1^, E. D. Batu^1^, M. Kasap Cuceoglu^1^, Z. Balik^1^, E. Aliyev^1^, Y. Bayindir^1^, O. Basaran^1^, Z. Saribas^2^, Y. Bilginer^1^, S. Ozen^1^, B. Sener^2^

##### ^1^Pediatric Rheumatology, ^2^Medical Microbiology, Hacettepe University, ANKARA, Turkey

###### **Correspondence:** M. Kasap Cuceoglu

**Introduction:** Myositis-specific and myositis-associated antibodies (MSA and MAA) are highly specific for classifying patients with juvenile dermatomyositis (JDM) according to their different features.

**Objectives:** We investigated the reflection of MSA and MAA on clinical features and their relationship with antinuclear antibody (ANA) patterns in JDM patients.

**Methods:** We examined the presence of MSA/MAA and ANA by EUROLINE Autoimmune Inflammatory Myopathies Profile and EUROPLUS ANA Mosaics (EUROIMMUN, Germany) in 70 JDM patients, followed between January 2014 and March 2022.

**Results:** MSA and MAA were positive in 41 JDM patients (58.5%) (Table 1). Muscle involvement and elevated CK levels were more frequent among patients with anti-NXP2, anti-TIF-1γ, anti-Mi-2 and anti-Ku antibodies (85.9%). Although skin involvement was prevalent in all patients, the frequency of calcinosis was highest in patients with anti-TIF-1γ antibodies (50%), and lung involvement was more common in patients with anti-MDA5 antibodies (40%). Calcinosis was more frequent among patients with positive myositis autoantibodies compared to the ones negative for MSA/MSA (p=0.016). ANA was positive in 34 JDM patients (48.5%). The most common ANA pattern was anti-cell (AC) 4 and 5. While AC4 pattern was present in all patients who were positive for anti-Mi-2, anti-Ku, anti-Pm-Scl75, or anti-Pm-Scl100 antibodies; AC5 pattern was detected in patients with anti-Mi-2, anti-Ku, or anti-Pm-Scl75 antibodies.

**Conclusion:** In our JDM cohort, anti-MDA5 autoantibody was associated with pulmonary involvement, while calcinosis was most frequent among patients with anti-TIF-1γ autoantibody. Analyzing the association of MSA/MAA with JDM clinical phenotype could pave the way for using personalized medicine strategies in the management of these patients.

**Trial registration identifying number:** not applicable

**Patient Consent:** Yes, I received consent

**Disclosure of Interest**: None declared

**Table 1 (abstract P279).** Characteristic features and antinuclear antibody patterns of 41 patients

	Anti-NXP2 (n=13)	Anti-TIF-1γ (n=10)	Anti-Mi-2 (n=7)	Anti-MDA5 (n=5)	Anti-PmScl75 (n=5)	Anti-PmScl100 (n=6)	Anti-Ku (n=4)	Others (n=6)
Age, years, median (min-max)	7.5 (3.5-11.7)	8.5 (1.4-12.8)	9.7 (2.4-16.9)	13.1 (6.4-16.8)	6.5 (2.5-12.1)	9.4 (6.8-14.5)	11.3 (2.5-13.9)	10.2 (4.3-14.7)
Gender, female, n (%)	8 (61.5)	6 (60)	4 (57.1)	3 (60)	4 (80)	4 (66.6)	4 (100)	4 (66.6)
Elevated CK levels, n (%)	11 (84.6)	6 (60)	4 (57.1)	2 (40)	1 (20)	2 (33.3)	3 (75)	2 (33.3)
Muscle weakness, n (%)	11 (84.6)	8 (80)	7 (100)	2 (40)	2 (40)	2 (33.3)	3 (75)	3 (50)
Calcinosis, n (%)	5 (35.7)	5 (50)	1 (14.3)	2 (40)	1 (20)	0	1 (25)	0
GIS involvement, n (%)	2 (14.3)	0	0	1 (20)	0	1 (16.6)	0	0
Pulmonary involvement, n (%)	2 (14.3)	0	0	2 (40)	0	1 (16.6)	0	0
Cardiac involvement, n (%)	0	1 (10)	0	0	0	0	0	0
ANA patterns, n (%)								
AC4	4 (80)	5 (83.3)	5 (100)	4 (80)	4 (100)	6 (100)	3 (100)	1 (16.6)
AC5	2 (40)	4 (66.6)	5 (100)	4 (80)	4 (100)	4 (66.6)	3 (100)	1 (16.6)
AC1	1 (20)	0	0	1 (20)	0	1 (16.6)	0	0
Others	2 (40)	1 (16.6)	1 (20)	1 (20)	0	0	0	0

### P280. Interstitial lung disease in juvenile dermatomyositis– our experience from Northern India

#### A. Thangaraj^1^, P. Vignesh^2^, M. Arora^2^, S. K. Loganathan^2^, M. Dhaliwal^2^, S. Sharma^2^, A. Rawat^1^, S. Singh^1^

##### ^1^Pediatric, PGIMER, ^2^pediatrics, Post Graduate Institute of Medical Education and Research, Chandigarh-160012, India

###### **Correspondence:** A. Thangaraj

**Introduction:** Interstitial lung disease (ILD) is one of the important complications of Juvenile dermatomyositis (JDMS) and is seen in ~20% of patients with (JDMS). ILD is predominantly associated with specific myositis antibodies like anti-aminoacyl-tRNA synthetase antibody, anti-MDA5, anti-Ku, anti-PM-Scl, anti-SRP, and anti-Mi2. We herein describe our experience of patients with JDMS with ILD from North India.

**Objectives:** To analyse the clinical, immunological profile, treatment protocols and outcomes of children with JDMS and ILD.

**Methods:** We analysed case records of all children diagnosed to have JDMS in the Pediatric Rheumatology Clinic during the period January 1992 to April 2022. The diagnosis of JDMS was based on modified Bohan and Peter criteria.

**Results:** Of the 140 patients with JDMS, 16 (11.4%) had features of ILD. Male to female ratio was 1:1.2. Mean age at disease onset was 9.6 years (range 4- 14 years). Clinical features included fever in 8 (50%), heliotrope rash in 8 (50%), Gottron papules in 13 (81.2%), muscle weakness in 15 (93%) and arthritis in 7 (43.75%) patients. One patient had a clinically amyopathic form and this was associated with anti-Ku, anti-PM-Scl, and anti-Ro 52 antibodies. Additional features of scleroderma overlap were seen in 4 patients. Nail fold capillaroscopy was carried out in 7 patients and all showed abnormalities (i.e., capillary loss, dilated tortuous loops, micro bleeding). Of the 16 patients with ILD, myositis associated antibodies were tested in 8 patients , 3 amongst those had anti- MDA5 antibodies. One patient had anti-Jo 1 and anti-Ro 52. Anti-PM-Scl was found in 1 patient who was also positive for anti-Ku and anti-Ro 52. Anti-SAE was positive in 1 patient. Nine patients had ANA positivity (56%). High-resolution computed tomography (HRCT) was carried out in 11 patients. Predominant findings included ground-glass opacities (n=6), septal and intra-lobar thickening (n=3), reticular pattern (n=4), atelectasis (n=2), consolidations (n=2), and honeycombing (n=2). Lower lobe involvement was seen in 7 patients. Pulmonary function tests were carried out in 7 patients and all of them showed restrictive pattern. Pulmonary artery hypertension on 2D Echocardiography was noted in 4 patients. All patients received oral steroids and methotrexate. In addition, 12 patients received intravenous pulse methyl prednisolone (followed by tapering); 5 patients received monthly intravenous pulse cyclophosphamide for 6 months followed by maintenance azathioprine in 4; one patient required rituximab but continued to have progressive respiratory distress syndrome and succumbed. On follow-up, 12 patients were stable with immunosuppressants and had no worsening in ILD; 4 patients were lost to follow up.

**Conclusion:** In our cohort, we noted that ILD was present in 11.4% of patients with JDMS. Children with JDMS-ILD need long term follow-up, however, the prognosis remains guarded.

**Patient Consent:** Yes, I received consent

**Disclosure of Interest**: None declared

**Table 1 (abstract P280).** See text for description

	JDMS without ILD	JDMS with ILD	Chi square	P Value
MDA5	5	3	5.69	0.016
Cyclophosphamide	22	5	1.94	0.164
Mortality	11	1	0.124	0.724

### P281. There are significant delays in diagnosis of juvenile dermatomyositis- 30 years of clinical experience at a tertiary care centre in north india

#### R. Tyagi, S. Basu, P. Vignesh, R. K. Pilania, D. Suri, A. Rawat, S. Singh

##### Pediatrics, Postgraduate Institute of Medical Education and Research, Chandigarh, India

###### **Correspondence:** R. Tyagi

**Introduction:** Juvenile dermatomyositis (JDM) is the most common type of inflammatory myopathy seen in children and comprises 1.7 % of all patients registered in Pediatric Rheumatological Clinic (PRC) at our institute. We report herein our clinical experience on the initial presentation of JDM at our centre.

**Objectives:** To assess the association of delay in diagnosis of juvenile dermatomyositis with various clinical parameters

**Methods:** We analysed patients registered in PRC at Advanced Pediatrics Centre, PGIMER, Chandigarh during the period of January 1992 to April 2022. Of the 8200 patients registered during this period, 140 had been diagnosed to have JDM. Diagnosis was based on Modified Bohan and Peter criteria. Time interval between onset of symptoms and presentation to our unit was noted and patients were classified as follows: i) Group 1- Time interval between 0-3 months ii) Group 2- Time interval between 3-6 months, iii) Group 3- Time interval between 6 -12 months, iv) Group 4- Time interval >12 months. Additional clinical parameters that were noted were age at onset, calcinosis, interstitial lung disease (ILD), mortality, lipodystrophy, gastrointestinal (GI) vasculitis, respiratory weakness. Statistical analysis was done using the SPSS 22 software

**Results:** Of the 140 patients with JDM, 43 (30.7%) patients had a delay in diagnosis of more than 12 months (group iv). Of the latter, calcinosis was seen in 18 (41.8%) patients, ILD was seen in 6 (11.6%) patients and lipodystrophy was seen in 10 (23.2%) patients.

**Conclusion:** Our data show that 30.7% of patients with JDM at our centre have significant delays (>12 months) in diagnosis. We noted that calcinosis, ILD and lipodystrophy were more common in this subgroup. Our findings would impact therapeutic decision making in this condition.

**Trial registration identifying number:** Our data show that 30.7% of patients with JDM at our centre have significant delays (>12 months) in diagnosis. We noted that calcinosis, ILD and lipodystrophy were more common in this subgroup. Our findings would impact therapeutic decision making in this condition.

**Disclosure of Interest**: None declared

**Table 1 (abstract P281).** See text for description

Time interval between onset of symptoms and presentation (months)	Total number (n)	Age at onset (years)	Calcinosis	ILD	Lipodystrophy	GI vasculitis	Respiratory muscle weakness	Mortality
0-3	38	73.4	7 (18.4%)	2 (5.2%)	4 (10.5%)	5 (13.1%)	3 (7.8%)	5 (13.1%)
3-6	28	63.9	6 (21.4%)	5 (14.2%)	1 (3.5%)	1 (3.5%)	4 (14.2%)	3 (10.7%)
6-12	31	6.53.4	4 (12.9%)	3 (9.6%)	2 (6.4%)	1 (3.2%)	3 (9.6%)	2 (6.4%)
≥12	43	6.43.1	18 (41.8%)	6 (11.6%)	10 (23.2%)	0 (0%)	3 (6.9%)	2 (4.6%)

## Poster session: Systemic lupus erythematosus and antiphospholipid syndrome

### P282. The first case of Systemic Lupus Erythematosus (SLE) with Chronic Inflammatory Demyelinating Polyneuropathy (CIDP): in a libyan female child

#### A. A. Abushhaiwia^1^, Y. Elfawires^2^, A. Ravelli^3^

##### ^1^Paediatric Rheumatology , Faculty of Medicine, Tripoli university, Tripoli Children’s Hospital , ^2^Paediatric Rheumatology, Faculty of Medicine, Tripoli university , Tripoli Children’s hospital , Tripoli , Libya, ^3^ Head division of Paediatric Rheumatology, Giannina Gaslini Institute, Genova , Italy

###### **Correspondence:** A. A. Abushhaiwia

**Introduction:** chronic inflammatory demyelinating polyneuropathy (CIDP) is an uncommon manifestation of systemic lupus erythematosus (SLE). CIDP is characterized by muscular weakness with or without sensory loss in the extremities and can have a chronic progressive course with remission and repeated relapses. To our knowledge, few cases of CIDP and SLE have been previously reported .We herein report first Libyan female a child with JSLE complicated withwith Chronic demyelinating sensori-motor polyradioculoneuropathy (CIDP).

**Objectives:** We report a case of SLE presenting as CIDP and discuss the diagnosis, management, and prognosis of CIDP.

**Methods:** A case report

**Results:** A 15th year-old Arab Libyan female child born from non-consanguineous healthy parents. There was no family history of neither autoimmune nor metabolic diseases. She developed bilateral LL muscular weakness with decreased deep tendon reflexes. At that point, the patient experienced paresthesia on both feet and sever epigastric pain since 2015 that requires her multiple admissions to hospital and required to use wheel chair. Electroneuromyography was compatible with chronic demyelinating peripheral polyneuropathy, in both lower limbs, brain MRI: showed acute pansinusitis with moderate left orbital proptosis. MRI spine:was normal. Diagnosis made by Neurologists as a case of chronic demyelinating peripheral polyneuropathy, she was treated with IVIG and with IV steroids and oral steroids but the patient stopped the follow-up appointments and treatment since May 2020. She was referred to paediatric rheumatology clinic last August 2021 with of a 7 year history of progressive bilateral leg weakness, Paraparesis and lost ability to walk. Accompanied by intermittent high grade fever reached 39oC fatigue, joint pain,severe epigastric pain associated with vomiting sometimes poor appetite , lost weight, mouth ulcer and Malar rash. Physical examination showed a grading for motor strength of LL 2\5, decreased tone, absent deep reflexes of LL, intact both superficial & deep sensation. She was under weight 24 kgm (<2nd percentile) with sever muscle wasting, contractures of both elbow and knee joints. Laboratorial exams showed anemia (Hb 9.7 g/dL), leukopenia1.42X10^3^, thrombocytopenia 132X10^3^_,_ normal complete metabolic and panel thyroid profile, ESR 85mm\h, Protein\Cr ratio 0.01%, panel of autoimmune serology showed ANA was positive1: 800,Anti-ds DNA antibodies 76 IU, Anti SM antibodies was positive >300 U\mL, anti-RNP was positive >200 U\mL , Anti-SSA-52, SSA-60 (Ro) was positive 14 U\ml and C4 was low 0.13 g/L.Antiphospholipid Ab were negative .The diagnosis of JSLE was made at this point and treatment with CIDP, Pulse therapy with methylprednisolone, and oral prednisone 40 mg/day, IV cyclophosphamide (750 mg/m^2^/month) for 6th cycles, azathioprine, hydroxychloroquine sulfate 200 mg/day and attended weekly sessions of physical therapy. Following treatment with cyclophosphamide IV and prednisone, the patient showed marked clinical improvement and regained her ability to walk with minor assistance. Case was discussed regarding case was discussed regarding the need further cycle of cyclophosphamide during in PReS sister initiative hospital with prof. Raville who’s suggested that steroids, IVIG, plasmapheresis therapy should be considered if there is relapse or disease flare up.

**Conclusion:** Peripheral polyneuropathy has been rarely reported in association with JSLE,Also the treatment of JSLE can be challenging .As this case report has shown, late clinical diagnosis of CIDP but it demonstrates efficacy and safety of cyclophosphamide in the JSLE with Chronic demyelinating sensorimotor polyradiculoneuropathy (CIDP).

**Patient Consent:** Yes, I received consent

**Disclosure of Interest**: None declared

### P283. A different perspective on the interpretation of neurocognitive functions in childhood-onset systemic lupus erythematosus (psle) patients without neuropsychiatric involvement: Functional Magnetic Resonance Imaging (FMRI)

#### E. Aliyev^1^, E. S. Akbaş Aliyev^2^, H. Daşgın^3^, K. Karlı Oğuz^3,4^, B. Anlar^5^, M. Kasap Cüceoğlu^1^, S. Demir^6^, H. T. Çak Esen^2^, E. Çengel Kültür^2^, S. Özen^1^, Y. Bilginer^1^

##### ^1^Pediatric Rheumatology, ^2^Child and Adolescent Psychiatry, Hacettepe University, ^3^Aysel Sabuncu Brain Research Center (UMRAM), Bilkent University National Magnetic Resonance Research Center, ^4^Radiology, ^5^Pediatric Neurology , Hacettepe University, Ankara, ^6^Pediatric Rheumatology, Erzurum Training and Research Hospital, Erzurum, Turkey

###### **Correspondence:** E. Aliyev

**Introduction:** SLE is an autoimmune disease characterized by multi-organ involvement, including neuropsychiatric (NP) involvement. NPSLE patients are easily overlooked, so early identification of risky cases for NPSLE is essential in practice.

**Objectives:** We aimed to show neurocognitive effects with neuroimaging methods, especially fMRI before NP findings develop in SLE.

**Methods:** Wechsler Intelligence Scale for Children-IV (WISC-IV) and resting-state fMRI (TR=2000ms; slice thickness=3mm, 180 volume) were performed on pSLE patients included in the study and on healthy controls (HCs). Data were analyzed with the general linear model. The t-test for the global BOLD signal variation within the group was compared with the two-sample t-test for the difference and the Spearman correlation test was between the groups. Spearman correlation test was used to evaluate the correlation. The cluster size p-FDR threshold correction is p<0.05 between SLE and HCs. Neurological and psychiatric clinical evaluation was performed on all participants, and patients with clinical neurological or psychiatric symptoms were excluded.

**Results:** The mean age of 17 SLE patients (11 girls) included in the study was 16.4±2.4, and the mean age of 8 HCs (4 girls) was 17.4±1.7. There was no significant difference between age, gender, depression, anxiety, and other psychiatric symptoms. When the two groups were compared neurocognitively, significant differences were found in the WISC-IV total score, verbal comprehension, perceptual reasoning scores, patterns with cubes, number sequence, and vocabulary subtests. BOLD signal activation on fMRI was decreased in the left pallidum, putamen, and thalamus in the SLE group compared to healthy controls (voxel p<0.001, cluster (k) p< 0.05-FDR corrected). In addition, precisely the same brain regions showed significantly higher functional signal activation in HCs than in pSLE. The right and left insular and frontal operculum cortex and putamen positively correlated with ‘WISC-IV Total Score’ in SLE patients compared to the HCs (the p-FDR corrected for right and left brain regions according are 0.0075 and 0.0496). Brain regions (right insular and frontal operculum cortex and putamen) were a positive correlation with ‘WISC-IV Working Memory Score’ in between groups (p-FDR corrected are 0.0069, Stats (t) are 6.83). Therewithal left part of the brain, particularly the frontal operculum cortex, insular cortex, central opercular cortex, and putamen showed a positive correlation with ‘WISC-IV Working Memory Score’ in SLE patients compared to the HCs (p-FDR corrected are 0.0069, Stats (t) are 6,36). Finally right side of the brain regions: temporal pole, temporal fusiform cortex, anterior division, parahippocampal gyrus, and anterior division showed a negative correlation with ‘WISC-IV Working Memory Score’ between groups (p-FDR corrected are 0.0188, Stats (t) are -5,44).

**Conclusion:** We showed that lupus could cause neurocognitive dysfunction even before neuropsychiatric involvement in children. With the prospective follow-up of SLE patients with impaired neurocognitive functions, whether they will develop neuropsychiatric SLE can be evaluated. Those may be useful in identifying the patient population at risk for neuropsychiatric involvement of autoimmune diseases if fMRI is used routinely in the future.

**Patient Consent:** Yes, I received consent

**Disclosure of Interest**: None declared

### P285. Intima-media thickness and disease activity over time in childhood systemic lupus erythematosus: preliminary results from a perspective study

#### F. Baldo, M. Rossano, E. A. Conti, F. Chironi, S. Testa, F. Lucioni, A. Petaccia, G. Filocamo

##### Fondazione IRCCS Ca’ Granda Ospedale Maggiore Policlinico, Milano, Italy

###### **Correspondence:** F. Baldo

**Introduction:** Childhood systemic lupus erythematosus has been studied as a possible cause of early atherosclerosis, since the relationship between chronic systemic inflammation, atherosclerosis and, subsequently, cardiovascular disease has been clearly demonstrated in adults. However, due to the rarity of cardiovascular events in youngster, surrogate markers of atherosclerosis are needed. Carotid intima-media thickness is a feasible, noninvasive method to assess the risk of early subclinical atherosclerosis in children with cSLE.

**Objectives:** To assess cIMT at baseline in a cohort of patients, to investigate its possible relationship with disease-related variables, as well as to register cIMT variations with over time.

**Methods:** Outpatient with cSLE in a single pediatric rheumatology tertiary center center were included in the study. A clinical evaluation was performed the same day of the IMT measurement. IMT of the distal 10mm of the far wall of common carotid artery was determined by ultrasonography with qIMT technology, a semi-automated method, by a highly experienced single sonographer. A second measurement was done 36 months after the first visit. Clinical information at baseline was collected from clinical records, other information was collected prospectively. Correlation between variable was assessed with Spearman’s Rho, association between variables was studied with Mann-Whitney U-test.

**Results:** To date, 21 patients have been enrolled in the study. Clinical data are shown in table 1.

At baseline, mean age was 15,8 years (SD 3,5), and mean disease duration was 3,7 years (SD 3,4) 14 patients (67%) were on mycophenolate, 16 patients (76%) on hydroxychloroquine, all were on oral steroid. Eighteen patients (86%) showed renal involvement, 3 patients (14%) CNS involvement and 8 patients (38%) were positive to antiphospholipid antibodies, 5 patients experienced macrophage activation syndrome. We found a negative, statistically significant correlation (rs = -0.49392, p (2-tailed) = 0.019) between disease duration and IMT at baseline, while no statistically significant correlation was found between IMT at baseline and SLEDAI2k, BMI or current corticosteroid dose. No clear association was found between antiphospholipid antibodies, anti ds-DNA positivity, renal involvement, hypertension or Macrophage Activation Syndrome (MAS) and higher IMT values. AT follow up, mean IMT variation was +28,9 micrometers (SD 44,9). A statistically significant correlation was observed between the variation of SLEDAI2K between baseline and follow up visit (rs = -0.62945, p (2-tailed) = 0.038.

**Conclusion:** Our study shows that cIMT in lupus can be increased. Unexpectedly, shorter disease seems to be linked to higher IMT values. This could be due to the burden of uncontrolled inflammation at disease onset, to high-dose induction steroid therapy or both. The negative correlation between variation of SLEDAI2K at a 36 months follow up is likely due to age bias, since IMT increases with age, while higher SLEDAI2k score at onset decreased over time. Further observations in a larger cohort of patients may better explore the relevance of IMT as an early marker of cardiovascular risk in cSLE affected children and show correlation with disease related parameters.

**Disclosure of Interest**: None declared

**Table 1 (abstract P285).** See text for description

	At baseline N=21 M:F=4:17	At follow-up N=11 M:F=4:7
*SLEDAI2k-Median (IQR)*	6(2-8)	2(0-2)
*BMI-Mean (SD)*	22,7 (4,1)	23,7(3,6)
*IMT>90th° percentile -Number (%)*	5(24)	4(36)
*prednisone current dose (mg)-Mean (SD)*	8,3(5,3)	5,3(2,5)
*IMT (μm)-Median (IQR)*	389,5 (361-403)	420(390-448)
*IMT percentile-Median (IQR)*	25(25-68,8)	75(50-90)

### P286. Pediatric neuropsychiatric systemic lupus erythematosus: case series from a tertiary care center in Eastern India

#### J. N. Bathia, P. Pal

##### Department of Pediatric Rheumatology, Institute of Child Health, Kolkata, India

###### **Correspondence:** J. N. Bathia

**Introduction:** Neuropsychiatric systemic lupus erythematosus (NPSLE) is a serious manifestation of childhood onset SLE. In majority of the cases the central nervous system (CNS) involvement occurs during the first year of disease onset. We present a case series of NPSLE patients encountered during last 7 years in a tertiary pediatric care center in eastern India.

**Objectives:** To evaluate the demographic, clinical, laboratory, radiological features of patients diagnosed as NPSLE as per the ACR 1999 classification and to evaluate the treatment given and response to treatment.

**Methods:** It is a retrospective observational study of data of patients diagnosed as having NPSLE, admitted between January 2015 to April 2022 in the Department of Pediatrics, at Institute of Child Health, Kolkata. NP-SLE was defines as per the ACR 1999 classification. CNS manifestations due to secondary causes were ruled out.

**Results:** Fifteen (n=15) patients were found to have NPSLE. 14 were females and 1 was male. The median age at onset of disease was 10 years (quartile 1: 9.5 yrs quartile 3: 12 yrs and IQR 2.5). 12 patients had NP manifestations at diagnosis of SLE, the rest manifested at 6, 12 and 14 months from diagnosis. Neuropsychiatric manifestatons was were variable. The most common manifestation, with 9 patients each,was cognitive dysfunction, psychosis and acute confusional state, anxiety and mood disorders followed by seizures (n=7), headache (n=3), cerebro vascular disease (n=3), aseptic meningitis (n=2), limb weakness (n=2), vertigo (n=1) and bulbar palsy with ptosis (n=1). amongst three patients has headche one had isolated headache as the only manifestation. Other system involvement were renal (n=6), musculoskeletol (=5), hematological (n=4), hepatitis (n=1) and pancreatitis (n=1). Anti-nuclear antibody (ANA) positivity was seen in all patients, anti double stranded DNA (anti dsDNA) was positive in seen in 10 and 2 patients were positive for antiphospholipid antibodies. MRI brain was done in all. 4 had normal MRI brain, 6 had diffuse brain atrophy, 2 had diffuse meningeal enhancement of which 1 also had cerebral venous sinus thrombosis, 1 had acute non hemorrhagic infract with beading in bilateral middle cerebral artery, 1 had ischaemic lesions and 1 had multifocal oedema. Depending on severity immunomodulator therapy was initiated with either pulse methyl prednisolone alone (n=2), cyclophosphamide alone (n=5), rituximab alone (n3) or a combination of Rituximab and cyclophosphamide (n=5). Maintenance was done with mycophenolate mofetil or azathioprine. Two APLA positive patients also received aspirin. One APLA negative patient received aspirin due to thrombotic lesion in brain. Steroids and hydroxychloroquine sulphate was given all. 4 patients had severe disease and were comatosed. All four recovered after 6 to 8 months of therapy with no residue. No patient had any further neuropsychiatric flare and there was no mortality.

**Conclusion:** NPSLE is a serious manifestation of childhood onset SLE. Secondary causes of CNS manifestations should be ruled out especially infection. Prompt diagnosis and aggressive immunomodulator therapy results in good response.

**Patient Consent:** Yes, I received consent

**Disclosure of Interest**: None declared

### P287. A child with chronic headache, fever and diffuse meningeal enhancement

#### J. N. Bathia^1^, P. Pal^1^, P. Khatua^2^, M. Ganguly^2^

##### ^1^Department of Pediatric Rheumatology, ^2^Department of Pediatric Medicine, Institute of Child Health, Kolkata, India

###### **Correspondence:** J. N. Bathia

**Introduction:** In juvenile onset systemic lupus erythematosus (SLE) neuropsychiatric manifestations are most commonly seen. Isolated lupus headache is a known clinical feature of Neuro Psychiatric - SLE (NP-SLE) however it is more common in adults.

**Objectives:** To present a 10 years old girl who presented with chronic headache as the initial manifestation of NP-SLE.

**Methods:** 10 years old female presented to us with recurrent headache for last 6 months. Initially she was treated as migraine. However, the headache persisted. Thereafter, she started having intermittent episodes of fever and there was left cervical lymphadenopathy. Lymph node biopsy was done outside which was suggestive of reactive lymphocytes with epitheloid granuloma. The tuberculin sensitivity test, chest X ray were non-contributory. Antitubercular therapy was started. She then started complaining of weakness of both lower limbs and had an episode of seizure and hence admitted

**Results:** After admission the child was started on anti convulsants. On examination ther was pallor. Other systemic examination was normal. Laboratory findings were hemoglobin 8.5 g/dL, total leukocyte count 10.0 x 10^3^/μL, neutrophils 78%, lymphocyte 18%, platelets 90 x 10^3^/μL. C reactive protein 57 mg/L,albumin 3.7 g/dL. MRI brain with contrast showed diffuse meningeal enhancement, cerebral venous sinus thrombosis in superior sagittal and right transverse sinuses with mild to moderate dilatation of the ventricles. T2W sagittal screening of dorsal spine showed ill-defined signal abnormalities. Considering an infectious etiology, CSF analysis was done which was normal. Ophthalmoscopy showed retinal haemorrhages. Following reasonable exclusion of infectious causes, detailed evaluation of an underlying autoimmune pathology was undertaken. Antinuclear antibody (ANA) was positive (1:1000, homogenous pattern) with positive anti-dsDNA. Complement levels were, C3 49.7 mg/dL, C4 6.9mg/dL. Final diagnosis of neuro psychiatric systemic lupus erythematosus (NP-SLE) was made. APLA studies were negative. The anti-tubercular therapy was stopped and patient was initiated on systemic steroids, monthly cycles of cyclophosphamide, hydroxychloroquine sulphate, and aspirin . Patient responded well to the management protocol. At present she is asymptomatic on oral corticosteroids and monthly pulse cyclophosphamide.

**Conclusion:** Isolated Lupus Headache in not uncommon however a high index of suspicion is required for appropriate diagnosis. Our patient also had diffuse meningeal enhancement in MRI brain. This case shows that not all diffuse meningeal enhancements have an infectious etiology.

**Disclosure of Interest**: None declared

### P288. Clinico pathological profile of pediatric lupus nephritis : ICH Kolkata experience

#### J. N. Bathia^1^, D. Biswas^2^, P. Pal^1^, R. Sinha^3^, D. Dasgupta^3^

##### ^1^Department of Pediatric Rheumatology, ^2^Department of Pediatric Medicine, ^3^Department of Pediatric Nephrology, Institute of Child Health, Kolkata, India

###### **Correspondence:** J. N. Bathia

**Introduction:** Lupus nephritis (LN) is one the most serious complications of systemic lupus erythematosus (SLE). With an overall incidence of about 70% in children, it is the most important determinant of long term prognosis

**Objectives:** To determine the proportion of the various initial clinical features, the various patterns of biochemical parameters including immunological markers and to determine the proportion of children with the various histopathological staging and to determine the outcome of various immunosuppressive agents in induction and remission

**Methods:** It is a single centre study of patients with LN attending the Lupus Clinic at the Institute of Child Health, Kolkata from January 2020 to October 2021.

**Results:** A total of 60 patients were enrolled; the female:male ratio was 4:1. The median age at diagnosis was 10.6 +/- 2.4 years. The most common extra-renal manifestation was mucocutaneous (n=54) followed by musculoskeletal (n=26), hematological (n=29), cardiovascular (n=14), neurological (n=11), pulmonary (n=5) and endocrine (n=1). The various clinical features were fever (n=55), oedema (n=50), hypertension (n=31), oliguria (n=27) and gross hematuria (n=16). Most common biochemical parameter detected was proteinuria (95%); median value was 1.4 gm (IQR of 0.6 to 2.3), GFR less than 90 was seen in 72%. Mean eGFR was 72.2 +/- 26.9 ml/min/1.72 m^2^. 58.3% had microscopic hematuria. At presentation anti-Nuclear Antibody was positive in all; C3 was low in 92% and C4 in 85%. Anti-phospholipid antibody was positive in 18%, anti-double stranded DNA (anti ds DNA) was seen in 63%. On biopsy Class IV Lupus Nephritis was the most common (53%), Class III in 20%, class II in 5%, Class III/IV + V in 8%, class VI in 2% and class V in 12%. All children received steroids (pulse/oral). Mycophenolate Mofetil (MMF) was given as induction agent in 28 patients, cyclophosphamide in 19 and MMF in combination with cyclophosphamide was given in 2. Rituximab was used in 10 patients as a rescue agent and Tacrolimus was used in 1. At one year follow up, complete response was seen in 24 patients and partial response was seen in 28. The mean time to remission was 5.5 +/- 1.6 months. The incidence of flares was 0.18 per person year and the mean time to first flare was 19 +/- 6 months. Mortality rate was 10% (n=6); 2 had ESRD and 4 succumbed to serious infectons. The baseline eGFR in those who received cyclophosphamide was 61.3 +/- 19.9 ml/min/1.72 m^2^ and in those who received MMF was 84.0 +/- 19.7 ml/min/1.72 m^2^. Binary logistic regression analysis was done to assess prediction of complete or partial response at 1 year. Considering eGFR and proteinuria there was marginal improvement in prediction compared to baseline (88.3% vs 86.7%, p value – 0.024) which accounts for 21% variability in outcome. The coefficients for eGFR was 0.023 (p = 0.268) and that for proteinuria was 0.001 (p = 0.04)

**Conclusion:** Although a difficult disease to treat, early diagnosis and timely aggressive management leads to better response rates as seen in 86% of our patients. The efficacy of the use of Cyclophosphamide and Mycophenolate Mofetil needs a further elaborative study with a larger sample size. However, to summarize the findings of our study, we found that the use of MMF was more efficacious in inducing remission as compared to cyclophosphamide although not statistically significant

**Patient Consent:** Yes, I received consent

**Disclosure of Interest**: None declared

### P289. Monogenic systemic lupus erythematosus (SLE) in Northern Israel

#### Y. Butbul Aviel^1^, T. Hershkovitz^2^, R. Zaid^3^, K. Weiss^3^, I. Spivak^1^

##### ^1^Pediatric Rheumatology Service, ^2^Rambam Medical Center Haifa Israel, Haifa, Israel, ^3^Genetic Institute, Rambam Health Care Campus, Haifa, Israel., Rambam Medical Center Haifa Israel, Haifa, Israel

###### **Correspondence:** Y. Butbul Aviel

**Introduction:** Systemic Lupus erythematous (SLE) is a heterogenic clinical syndrome with a multifactorial etiology including diverse environmental, immunological and genetic causes and modifiers. Increasingly, utilizing next generation sequencing tooIs, monogenic forms of SLE have been identified.

**Objectives:** The aim of our study was to identify monogenic causes of SLE in the unique pediatric population of Northern Israel.

**Methods:** A retrospective and prospective study was carried out between 2010-2021 in a single tertiary pediatric medical center. Genetic testing including Whole exome sequencing (WES) was performed for select patients including family history of SLE, consanguinity and\or clinical findings suggestive of specific disorder.

**Results:** 75 children were diagnosed with SLE. 18/75 (24%) had one or more relatives with SLE or suspected monogenic disorder, including a pedigree with 4 affected members. Mean age at presentation was 10.1±4.7years and 16/18 (89%) were females. A monogenic disorder was identified in total of 7/75 of pedigrees. Four patients were diagnosed with Prolidase deficiency, one patient with *ADAR1* mutation related to Aicardi–Goutières syndrome and one pedigree with APC5 mutations. Candidate variants in genes related to immune system were identified in one proband and her father requiring further study. Additional WES results are pending.

**Conclusion:** We detected monogenic causes of SLE in a select cohort of patient in Northern Israel. Identification of a genetic basis for disease has direct clinical implication for patients and families and can also enhance our understanding of the pathogenesis and disease mechanisms involved in the more common sporadic forms of SLE.

**Disclosure of Interest**: None declared

### P290. Diet, gut microbiota, and intestinal permeability in systemic lupus erythematosus

#### M. Castro^1,2^, I. Almada-Correia^1,2^, C. M. Motta^3^, C. M. Sousa Guerreiro^4^, R. A. Moura^1^, N. Khmelinskii^1,5^, F. Oliveira-Ramos^1,5,6^, C. I. Mendes^7^, M. Ramirez^7^, J. E. Fonseca^1,5^, P. Costa-Reis^1,6^

##### ^1^Rheumatology Research Lab, Instituto Medicina Molecular (iMM), ^2^Faculdade de Ciências , ^3^Instituto Nacional de Saúde Doutor Ricardo Jorge, ^4^Laboratório de Nutrição, Faculdade de Medicina, Universidade de Lisboa, Centro Académico de Medicina de Lisboa, ^5^Serviço de Reumatologia e Doenças Ósseas Metabólicas, ^6^Unidade de Reumatologia Pediátrica, Hospital de Santa Maria, Centro Hospitalar Universitário Lisboa Norte, Centro Académico de Medicina de Lisboa, ^7^Instituto Medicina Molecular (iMM), Lisboa, Portugal

###### **Correspondence:** F. Oliveira-Ramos

**Introduction:** The gut microbiota modulates mucosal immunity, gut permeability, and endotoxemia. In a systemic lupus erythematosus (SLE) murine model it was shown that the translocation of a gut pathobiont induces an autoimmune response and death, which is prevented by antibiotics and a vaccine. We hypothesize, therefore, that dysbiosis, impaired intestinal barrier integrity, and endotoxemia are crucial to the chronic activation of the immune system seen in SLE.

**Objectives:** To study diet, physical activity, body composition, gut microbiota, and intestinal permeability in SLE patients.

**Methods:** Evaluation of healthy controls (HC) and SLE patients (children and adults) who fulfil the 2019 EULAR/ACR SLE classification criteria. Individuals with inflammatory bowel disease, celiac disease, irritable bowel syndrome, diabetes, malignancy, or other immune-mediated diseases were excluded. Three-day diet recalls, PREDIMED, KIDMED and the International Physical Activity questionnaires were used to evaluate diet and physical activity. Body composition was analysed by whole-body air-displacement plethysmography. Gut microbiota was studied by Next Generation Sequencing, with amplicon sequencing-based 16S rRNA analysis. The intestinal permeability was assessed directly by the lactulose/mannitol test. Serum markers of gut permeability and inflammation (zonulin, claudin-3, sCD14) were measured by ELISA. The SLEDAI-2K score was used to evaluate disease activity.

**Results:** We studied 16 HC (median age 35.5Y; 14-50Y; 88% females) and 36 SLE patients (11 children and 25 adults; median age 33.5 Y; 11-57Y; 89% females; median age at diagnosis 23.17Y; median disease duration 5.2 years (0.25-28.67Y); 66% had lupus nephritis; median SLEDAI-2k at time of sample collection 4). SLE patients had lower physical activity and higher sitting time, lower adherence to Mediterranean diet and higher fat mass and central obesity than HC (p<0.05). In addition, SLE patients had a lower intake of alpha-linolenic acid and manganese (p<0.05). A decreased a-diversity of gut microbiota (p=0.02) was identified in SLE patients, reflecting dysbiosis. Mediterranean diet, zonulin levels and SLE disease duration significantly impacted gut diversity in our cohort (p<0.05). Zonulin levels and the lactulose/mannitol ratio were elevated in SLE patients compared to HC (p<0.05), reflecting higher gut permeability. We also found significantly increased levels of sCD14 in SLE patients (p<0.05). There were no significant differences in claudin-3. No significant correlation was observed between any evaluated biomarker and SLEDAI-2k.

**Conclusion:** Our data support the hypothesis that gut dysbiosis and higher intestinal permeability contribute to SLE pathogenesis, being two promising therapeutic targets in this disease.

**Disclosure of Interest**: None declared

### P291. Primary antiphospholipid syndrome – clinical review of three pediatric patients

#### M. Rebelo^1^, A. I. Carvalho^1^, R. Maia^2^, C. Henriques^3^, S. Batalha^2^, P. Kjöllerström^2^, M. Conde^3^

##### ^1^Pediatrics, ^2^Pediatric Hematology, ^3^Pediatric Rheumatology, Hospital Dona Estefânia - Centro Hospitalar Universitário Lisboa Central, Lisbon, Portugal, Lisbon, Portugal

###### **Correspondence:** M. Conde

**Introduction:** Primary antiphospholipid syndrome (Primary APS) is an acquired autoimmune thrombotic disease in which patients with persistently positive antiphospholipid antibodies (aPLs)- Lupus anticoagulant (LA), anti-Cardiolipin (aCL) and B2-glicoprotein I (B2GP1) antibodies) have a thrombotic event in the absence of another autoimmune disorder.

**Objectives:** To describe cases of Primary APS diagnosed in a level III pediatric hospital over a 5-year period (2017 to 2022).

**Methods:** Review of the medical records of patients < 18 years old diagnosed with APS, as per the revised Sapporo classification criteria. Patients with another rheumatic disease were excluded. Demographic, clinical, laboratory, treatment and follow-up data were reviewed.

**Results:** Three cases of Primary APS were identified. All were diagnosed by a thrombotic event accompanied by persistent triple positive aPLs. None had a combination of clinical and/or serological work-up consistent with another autoimmune disease. No other clinically relevant pro-thrombotic risk factors were identified beyond the ones mentioned.

Case 1: 15-year-old, previously healthy adolescent, maternal SLE and positive aPLs, presented with unilateral deep venous thrombosis (DVT) in the right lower limb. Blood tests showed mild thrombocytopenia and increased liver enzymes all of which resolved spontaneously. A prolonged aPTT (106s) led to the identification of persistent triple positive aPLs at high titres which have persisted to date. He had ANA 1/160 and transiently low C3. He was treated with enoxaparin and then switched to warfarin with complete resolution and no recurrence of thrombotic events.

Case 2: 15-year-old obese adolescent boy, on oral isotretinoin for acne, presents with right popliteal DVT. Thrombophilia studies revealed persistent high titre triple positive aPL; ANA 1/80 and an isolated weak positive direct antiglobulin test (DAT) without anaemia or haemolysis. He was treated with enoxaparin. Three months later, he had another extensive DVT of his right lower limb. Enoxaparin dose was adjusted to weight and later switched to tinzaparin. Nine months later, he suffered a bilateral pulmonary thromboembolism (PTE) with a large thrombus in the inferior vena cava. The patient received enoxaparin, later switched to warfarin plus hydroxychloroquine (HCQ) and acetylsalicylic acid with satisfactory recovery. He presented mild thrombocytopenia since diagnosis that resolved after his last thrombotic event.

Case 3: 12-year-old obese girl evaluated due to thrombocytopenia (platelets 28x10^9^/L), weak positive DAT (without anaemia or haemolysis) and prolonged aPTT (80,4s). She had persistent triple positive aPLs; a positive ANA (1/320) with otherwise repeatedly negative autoimmune work-up; heterozygous Factor V Leiden mutation in the thrombophilic work-up; and no other clinical or laboratorial findings. 6 months later she had a PTE with a small ipsilateral pleural effusion and worsening of thrombocytopenia. She was treated with enoxaparin. Immunoglobulin and high-dose steroid pulses were given to control thrombocytopenia and allow for anticoagulation. Prednisolone, azathioprine, HCQ and fluvastatin (dyslipidemia) were also initiated. Enoxaparin was maintained and prednisolone progressively reduced with no thrombotic recurrence.

**Conclusion:** Pediatric Primary APS is rare and both primary and secondary prophylaxis are not well defined. Non-thrombotic APS manifestations overlap with other autoimmune diseases. Higher-risk patients presenting triple-positive aPLs require strict follow-up and may need combined anticoagulant therapy. Minimizing vascular risk factors is also essential along with anticoagulation while aPLs are present.

**Patient Consent:** Yes, I received consent

**Disclosure of Interest**: None declared

### P292. High expression of spata5l1 protein in lupus nephritis

#### P. P. Kahwage^1^, R. R. Moura^2^, G. R. Sousa^1^, K. B. Salomão^1^, F. P. Saggioro^3^, I. Facincani^1^, D. Q. Nascimento^4^, R. S. Costa^1^, R. G. P. Queiroz^1^, E. T. Valera^1^, L. Rodrigues^1^, V. P. L. Ferriani^1^, S. Crovella^5^, P. Sandrin-Garcia^4^, L. M. de Carvalho^1^

##### ^1^Ribeirão Preto Medical School, University of São Paulo, Ribeirão Preto, Brazil, ^2^Advanced Diagnostics, Institute for Maternal and Child Health-IRCCS “Burlo Garofolo”, Trieste, Italy, ^3^Pathology, Rede D’Or , São Paulo, ^4^Genetics, Federal University of Pernambuco, Recife, Brazil, ^5^Biological and Environmental Sciences, College of Arts and Sciences, Qatar University, Doha, Qatar

###### **Correspondence:** L. M. de Carvalho

**Introduction:** Systemic lupus erythematosus is a complex autoimmune disease with genetic and immunological factors involved in its triggering. The Spermatogenesis-associated protein 5-like protein 1 - SPATA5L1 is ubiquitously expressed in kidneys and other tissues and previously had been associated with indices of renal function and chronic kidney disease (CKD)^(1)^. In a previous study we reported a *SPATA5L1* gene pathogenic variant associated with a high expression of the protein in full-house nephropathy^(2)^.

**Objectives:** The aim of this study was to explore the expression of this protein in renal biopsies of lupus nephritis and normal kidney controls.

**Methods:** This cross-sectional study was performed with 29 childhood-onset systemic lupus erythematosus patients – cSLE, followed in the Pediatric Rheumatology Clinic of the Clinical Hospital of Ribeirão Preto Medical School – São Paulo University, Brazil. All cSLE patients fulfilled the American College of Rheumatology classification criteria for SLE, onset of the disease occurred under 18 years and recruited up to 21 years of age. As controls, four healthy kidneys were obtained from patients submitted to an autopsy from our Pathology and Forensic Medicine Service. Sanger sequencing analysis was performed in the cSLE patients to identify a previously reported pathogenic variant (rs143453038) associated with high protein expression in full-house nephropathy. The evaluation of SPATA5L1 protein expression was performed in biopsy samples by immunohistochemistry of all patients and controls. This study was approved by the Research Ethics Committee.

**Results:** The sequencing analysis did not find the *SPATA5L1* gene variant previously reported (rs143453038) in cSLE patients. However, we observed strong positive immunostaining for SPATA5L1 in nephritis lupus renal biopsies (class IV and V), while no or weak SPATA5L1 immunostaining was observed in non-inflammatory renal tissues.

**Conclusion:** The expression of SPATA5L1 protein is higher in lupus nephritis biopsies as compared to health kidneys. Our results suggested that the SPATA5L1 protein may be involved in lupus pathogenesis or may function as a renal biomarker of inflammatory activity in these patients. More studies are needed to clarify the role of SPATA5L1 protein in cSLE.

**References:** 1.Köttgen A, Glazer NL, Dehghan A, Hwang SJ, Katz R, Li M, et al. Multiple loci associated with indices of renal function and chronic kidney disease. Nat Genet. 2009;41(6):712-7. 2.de Carvalho LM, de Sousa GR, Moura R, Saggioro F, Facincani I, Costa R, et al. Full-house nephropathy associated with high expression of SPATA5L1 due to a genetic pathogenic variant. Rheumatology (Oxford). 2022;61(4):e84-e6.

**Patient Consent:** Yes, I received consent

**Disclosure of Interest**: None declared

### P293. Potential role for oxysterol receptor gpr183 in b cell-driven pathology in juvenile-onset systemic lupus erythematosus

#### N. M. De Gruijter^1,2^, B. Jebson^1^, A. Radziszewska^1,2^, H. Peckham^1,2^, O. Nettey^1,2^, M. Butt^1,2^, C. Ciurtin^1,2^, E. Rosser^1,2^

##### ^1^Centre for Adolescent Rheumatology Versus Arthritis, ^2^Centre for Rheumatology Research, Division of Medicine, University College London, London, United Kingdom

###### **Correspondence:** N. M. De Gruijter

**Introduction:** Juvenile-onset systemic lupus erythematosus (JSLE) is a rare severe inflammatory disease that starts before the age of 18 and can affect any part of the body. JSLE is often accompanied by disruptions in the cholesterol metabolism and is associated with increased risk of developing cardiovascular disease. JSLE is also characterised by the production of autoantibodies, secreted by B cells, against nuclear components. To become antibody secreting cells or plasma cells, B cells must be activated. This occurs in B cell follicles of secondary lymphoid organs in a germinal centre (GC) reaction. B cells can also be activated outside the follicle into extrafollicular plasma cells. Heightened extrafollicular B cell activation is associated with the production of autoantibodies in multiple disorders, and the frequency of extrafollicularly activated B cells is increased in the peripheral blood (PB) of patients with adult SLE. In mice, oxidised cholesterol, or ‘oxysterol’ receptor GPR183 is required for appropriate B cell positioning in the lymphoid follicle.

**Objectives:** To investigate whether pathological changes in GPR183-oxysterol interactions (due to altered cholesterol metabolism) contribute to heightened extrafollicular B cell responses and pathogenicity in (J)SLE.

**Methods:** Multi-parameter flow cytometry was used for phenotyping PB B cells of 28 JSLE patients and 33 age-matched controls (HC). The parent into F1 mouse model of lupus was used; only female F1 animals were included due to known sex bias. To assess the effect of blocking GPR183-ligand interactions, following injection of parent lymphocytes, F1 mice were injected with small molecule GPR183-antagonist NIBR189 or vehicle as a control daily for two weeks, then twice weekly. Proteinuria was scored between 0-4 using urine dipsticks over 10 weeks after disease induction (NIBR189 n=4; control n=5). The frequency of GC (CD19^+^GL7^+^CD95^+^) B cells was assessed by flow cytometry at two weeks post-injection of parent lymphocytes (NIBR189 n=3; control n=5). Unpaired t-test was used to compare significance of differences in marker expression between groups. Two-way ANOVA was used to compare significance of differences in urine score between groups. All numbers are portrayed as mean, unless otherwise specified.

**Results:** We found that GPR183 is co-expressed with B cell activation marker CD27 and identifies memory B cells. GPR183^+^CD27^+^ B cells are reduced in JSLE compared to HC: 17.2% of PB B cells are GPR183^+^CD27^+^ in JSLE, compared to 26.5% in HC (*p*=0.0003). GPR183^+^CD27^+^ B cells of JSLE patients have reduced expression of lymph node-homing and follicle-positioning receptor CXCR5 and gut-homing receptor CCR9: 56.6% and 47.87% in JSLE (n=12), vs. 80.0% and 62.8% in HC (n=14), respectively (CXCR5: *p*=0.0034; CCR9: *p*=0.0464).

Treatment with GPR183 antagonist NIBR189 significantly suppressed experimental lupus severity, as measured by lower proteinuria score (3.2 in NIBR189-treated mice vs. 1.1 in control lupus mice, *p*=0.0006). The percentage of GC B cells was significantly increased in NIBR189-treated mice (2.63%) vs. control lupus mice (1.34%) (*p*=0.0154) during disease initiation phase (2 weeks post-induction), suggesting reduced extrafollicular B cell differentiation.

**Conclusion:** Our data suggest a possible role for GPR183-oxysterol interactions in driving aberrant B cell differentiation in (J)SLE and identify this pathway as potential therapeutic target in this disease.

**Patient Consent:** Yes, I received consent

**Disclosure of Interest**: None declared

### P294. Pulmonary involvement in juvenile-onset systemic lupus erythematosus: data from the Uk JSLE cohort study

#### A. Faleye^1^, K. Mahmood^2^, M. Beresford^2,3^, C. Hedrich^2,3^, E. Smith^2,3^ on behalf of UK JSLE cohort study

##### ^1^Paediatrics, Lagos State University Teaching Hospital, Lagos, Nigeria, ^2^Paediatric Rheumatology, Alder Hey Children’s NHS Foundation Trust, ^3^Institute of Life Course & Medical Sciences (Child Health) , University of Liverpool, Liverpool, United Kingdom

###### **Correspondence:** A. Faleye

**Introduction:** Pulmonary involvement in Juvenile Systemic Lupus Erythematosus (JSLE) has not been comprehensively described in the literature, particularly as compared to adult SLE.

**Objectives:** To report the incidence, prevalence, demographic and clinical characteristics of pulmonary involvement in JSLE patients, assessing clinical and/or laboratory features associated with pulmonary involvement.

**Methods:** Participants of the UK JSLE Cohort Study (2006-2021), <18 years at diagnosis, with ^3^4 ACR-1997 classification criteria for SLE, were eligible for inclusion. Patients were grouped according to the presence or absence of pulmonary involvement (defined as a pBILAG pulmonary domain score A or B). Patients were assessed at diagnosis, 10-14, 22-26 and 58-62 months of follow-up, with demographic/clinical characteristics compared between those with/without pulmonary involvement.

**Results:** 480 JSLE patients were included, median age was 12.93 years (interquartile range, IQR 10.5-14.7). 119/480 (24.8%) had pulmonary involvement, 109/480 (22.7%) at diagnosis. 30/157 (19.1%) displayed pulmonary involvement at 10-14 months, 22/128 (17.2%) at 22-26 months, and 11/49 (22.4%) at 58-62 months. Sub-types of pulmonary involvement are displayed in the Table. Pulmonary involvement was associated with higher ACR-1997 (median 6.0 [IQR 4-6] vs 5 [IQR 5-6], p<0.002) and pBILAG numerical disease activity scores (median 22 [IQR 11-33] vs 9 [IQR 3-16], p<0.001) at diagnosis, with no differences at subsequent time points. There were no differences in SLICC-SDI scores between those with/without pulmonary involvement (all p>0.05). PBILAG defined constitutional (48.3 vs 26.1%), musculoskeletal (49.1 vs 26.1%), gastrointestinal (10.3 vs 3.8%) and haematological (37.9 vs 20.6%) involvement was more common in patients with pulmonary involvement (all p<0.05). Differences were seen in haemoglobin levels, neutrophil counts, erythrocyte sedimentation rate, and C4 levels in those with/without pulmonary involvement (all p<0.05).

**Conclusion:** Pulmonary disease is common in JSLE, with serositis the main form of involvement. It associates with wider organ involvement which suggests a need for close monitoring and aggressive treatment.

**Patient Consent:** Yes, I received consent

**Disclosure of Interest**: None declared

**Table 1 (abstract P294).** Subtypes of pulmonary involvement at diagnosis and during follow-up

Pulmonary manifestations collected by the UK JSLE Cohort Study	Diagnosis (n=109/480)	10-14 m (n=30/157 )	22-26 m (n=22/128)	58-62 m (n=11/49)
**Serositis (ACR classification criteria defined)**	44 (41%)	22 (73%)	18 (82%)	9 (82%)
**Pleuropericardial pain**	29 (27%)	2 (7%)	3 (14%)	1 (9%)
**Dyspnea**	38 (35%)	2 (7%)	-	2 (18%)
**Effusion (pericardial / pleural)**	35 (32%)	-	-	-
**Mild or intermittent chest pain**	29 (27%)	-	-	-
**Pulmonary function fall by >20%**	7 (7%)	2 (7%)	-	-
**Pulmonary hemorrhage/vasculitis**	1 (1%)	1 (4%)	-	-
**Interstitial alveolitis/pneumonitis**	5 (5%)	-	-	-
**Pleural fibrosis**	1 (1%)	1 (4%)	1 (5%)	-

### P295. Pediatric systemic lupus: the experience of the french overseas departments of America

#### A. Felix^1^, F. Delion^2^, B. Suzon^3^, A. Ogrizek^4^, N. Elenga^5^, M. Dramé^6^, C. Hospice^7^, M. Mohamed Sahnoun^8^, B. Bader-Meunier^9^, C. Deligny^10^, Y. Hatchuel^7^

##### ^1^Department of pediatrics, CHU de la Martinique, Fort de France, ^2^Department of Pediatrics, CHU Guadeloupe, Pointe à pitre, ^3^Department of internal medicine, ^4^Department of pediatrics, CHU de la Martinique, Fort-De-France, France, ^5^department of pediatrics, CH Andrée Rosemond , Cayenne, French Guiana, ^6^CIC Antilles-Guyane, ^7^Department of pediatrics, CHU de la Martinique, Fort de France, ^8^Department of pediatrics, CH de l’ouest Guyanais, Saint Laurent du Maroni, ^9^Department of Pediatric Rheumatology, CHU Necker enfants malades, Paris, ^10^Department of Internal Medicine, CHU de la Martinique, Fort de France, France

###### **Correspondence:** A. Felix

**Introduction:** Systemic diseases of pediatric onset are more frequent in Afro-Caribbean population, especially Pediatric systemic lupus (pSLE).

**Objectives:** Our work is a retrospective study of patients followed in French overseas departments of America for systemic lupus of pediatric onset. It describes their clinical and biological specificities at diagnosis, during childhood and early adulthood.

**Methods:** A retrospective study was conducted between January 2000 and September 2021. Listings of patients with pediatric onset were obtained through multiple sources: computerized hospital archives, registry of referent pediatricians and adult specialists in internal medicine and French National Registry for rare diseases. The spectrum of diseases studied was systemic lupus according to international criteria with onset before the age of 18 years old.

**Results:** Overall, 2148 patients were identified and 54 patients were included. Average follow-up was 8.3 years (range: 0.3 - 25 years). We found an increase of new diagnoses throughout the years. At onset, pSLE patients had median 10 SLICC criteria (range: 4-12) and median EULAR/ACR 2019 score was 38 (12 - 54). At onset, a third of patients had renal involvement, 15% neurolupus and 41% cardiac involvement. During childhood, 54% had renal involvement and 26% suffered from neurolupus. Patients suffered in median from 3 flares during childhood and 26% from more than 5 flares. Younger patients at onset had worst outcomes, they had more flares (median at 5 p = 0,02) and needed an average of 4 background therapies (p = 0,04).

**Conclusion:** This is a large cohort of patients of Afro-Caribbean origin with a higher frequency of pSLE. The outcomes of Afro-Caribbean patients, where pSLE is more frequent, were similar to western countries with worse disease activity at onset. Further studies should be performed to identify the genetic and environmental factors in this population.

**Patient Consent:** Yes, I received consent

**Disclosure of Interest**: None declared

### P296. Instrument for nursing consultation for patients with juvenile systemic lupus erythematosus

#### J. C. O. A. Ferreira^1^, A. A. Costa^2^, H. C. S. Robba^2^, A. C. S. Monteiro^2^, J. R. Campana^2^

##### ^1^Clinical Research Center, ^2^Nursing Division, Instituto Da Criança Do HCFMUSP, São Paulo, Brazil

###### **Correspondence:** J. C. O. A. Ferreira

**Introduction:** The chronic nature of Juvenile systemic lupus erythematosus and the possible complications or limitations demands special nursing knowledge and attention to treat the adolescent patient and their family. The structural and functional maturation of the brain, besides disease activity, continuous use of medication, presence of comorbidities and pain can change body image and cause social isolation.

**Objectives:** To develop an instrument for nursing care for patients with JSLE.

**Methods:** It was developed an instrument for nursing consultation to patients with JSLE from a published study carried out on the care provided by Brazilian nurses to JSLE patients, approved by the local Ethics Committee number 2.089.884.

**Results:** The instrument is composed by information from vital signs and anthropometric data, physical examination, clinical signs and manifestations of JSLE, aspects of care such as adherence, pain, sexuality, drug use, physical violence, suicidal ideation, bullying, vaccination, acceptance of disease.

**Conclusion:** The instrument has already started to be used and allowed the diagnosis of problems such as pain, suicidal ideation and bullying. A multicenter study will be conducted to verify its reliability and reproducibility.

**Patient Consent:** Not applicable (there are no patient data)

**Disclosure of Interest**: None declared

### P297. Lupus manifestations in first degree consanguineous relatives of a single family in Nicaragua

#### K. V. Jirón Mendiola, M. Jarquín Jaime

##### Reumatología Pediátrica, Hospital Infantil de Nicaragua Manuel de Jesús Rivera, Managua, Nicaragua

###### **Correspondence:** K. V. Jirón Mendiola

**Introduction:** Family studies have shown the genetic contribution to developing systemic lupus erythematosus, and a 20-fold increased risk of developing lupus has been found compared to the general population, in addition to the fact that 10% of first-degree relatives can also develop lupus.

There are previous studies that described the aggregation of connective tissue diseases in families; thus, following a clinical observation, we studied an unusual case of Lupus with different manifestations in 4 siblings born to mothers with SLE, in addition, 5 cases of relatives in second and third degree of consanguinity were studied.

**Objectives:** To describe an unusual case of lupus manifestations in first degree consanguinity members of a single family

**Methods:** An observational retrospective study of a 45-year-old woman diagnosed with Lupus, mother of 4 children diagnosed with SLE, of which the clinical manifestations presented were described.

**Results:** 9 cases were identified; all alive at the time of the study, of which clinical records and studies that conditioned the diagnostic criteria were available. A genealogical mapping was carried out with antecedents, finding 5 patients of first degree of consanguinity, 3 in second degree and 2 in third degree of consanguinity with a diagnosis of SLE, inhabitants of the same area of ​​the country.

Of the 9 cases reviewed, 5 were male and 4 female. The mean age at diagnosis was 22 years. The most dominant clinical presentation was photosensitive maculopapular rash, malar rash, aphthous ulcer in 8/9 (88.8%), arthritis in 7 of them, neurolupus with demyelinating syndrome in 4/9 (44%), migraine headache in 3/9. Cutaneous vasculitis was present in 2/9 (22.2%) Cardiopulmonary involvement with pleurisy and pericarditis was noted in 2/9 (22.2%), myelopathy in one patient, anemia in 2/9 (22.2%), and thrombocytopenia and leukopenia in one each . Autoantibodies were mainly ANA in all patients (100%) and anti-dsDNA in 9/9 (100%).

The nine patients had received therapy without a diagnosis of SLE prior to its detection and integration of criteria, finally once the diagnosis was made, management with bolus glucocorticoids was indicated, two of them received rituximab and cyclophosphamide.

**Conclusion:** The genetic predisposition to SLE is already described, however the extensive grouping of SLE in the same family is less described, especially when they present with similar manifestations and classification criteria for SLE, with a predominance of neurological manifestations in members of a single family.

Our observational study is the first to describe the family grouping of SLE in Nicaragua, finding a considerable number of patients, which would provide a follow-up guideline to investigate genetic factors and/or racial risk factors in our population.

**Disclosure of Interest**: None declared

### P298. Safety and efficacy of rituximab in pediatric lupus nephritis: retrospective cohort data of 25 patients

#### E. Kalashnikova^1^, R. Rinat^1^, N. Lubimova^2^, K. Ekaterina^2^, E. Isupova^3^, E. Gaidar^3^, V. Masalova^3^, L. Snegireva^3^, T. Kornishina^3^, L. Sorokina^3^, M. Kaneva^3^, O. Kalashnikova^3^, V. Chasnyk^3^, M. Kostik ^3^

##### ^1^Pediatrics, Saint-Petersburg State Pediatric Medical University, Saint Petersburg, ^2^Laboratory of autoimmune and autoinflammatory diseases, Almazov National Medical Research Centre, ^3^Pediatrics, Saint-Petersburg State Pediatric Medical University, Saint-Petersburg, Russian Federation

###### **Correspondence:** M. Kostik

**Introduction:** Lupus nephritis (LN) is one of the severest manifestations of systemic lupus erythematosus (SLE), determining the outcomes of the disease.

**Objectives:** to analyze the safety and efficacy of rituximab (RTX) in pediatric patients with lupus nephritis.

**Methods:** in the retrospective cohort study were included 25 patients with LN (10 boys and 25 girls) with onset age 13 (12; 15 years). Diagnosis was made using Systemic Lupus International Collaborating Clinics (SLICC) classification criteria. RTX was prescribed in dosage 375 mg/m^2^ every week (2-4 infusions) with repeated courses every 6-12 months (2-4 infusions) according disease activity, the degree of B-cell depletion and hypoIgG-emia. The indications for rituximab were severe LN, resistant to conventional non-biologic treatment or corticosteroid dependence/toxicity. The dynamics of clinical, laboratory data, activity of the disease by SLEDAI, corticosteroid doses were assessed in the onset, and during RTX trial.

**Results:** The main patient’s characteristics were: Pre-RTX non-biologic conventional treatment includes: cyclophosphamide 15 (60%), MMF 8 (32%), azathyoprine 3 (12%), hydroxychlorquine (HCQ)12 (48%), pulse therapy of methylprednisolon with following oral methylprednisolon 25 (100%). Time before RTX was – 7.0 (3.0; 24.0) months, whole observation period was 7.0 (0; 24) months. Initial pre-RTX treatment (corticosteroids, HCQ, non-biologic DMARDS) slightly reduced SLEDAI levels and the proportion of the patients with LN, but following administration of the RTX realized in prominent reducing of SLEDAI, anti-dsDNA level, proteinuria, hematuria, C4 complement, ESR, and median corticosteroid dose by 80% from the initial, as well as the proportion of patients without corticosteroids. Two deaths were observed due to catastrophic SLE with MAS, accompanied severe infection (invasive aspergillosis, n=2). Three patients realized SAE: pneumonia (n=2), transient agranulocytosis (n=1) after 3rd RTX infusion and meningitis, caused by Lysteria monocytogenis, after 1st RTX infusion (further RTX treatment continued without adverse events), patella osteomyelitis (n=1). Eight patients received antibacterial treatment for different respiratory infections not required hospital admissions.

**Conclusion:** RTX showed effectiveness in LN, where previous non-biologic treatment was insufficiently effective. Randomized controlled trials are required to evaluate the efficacy and safety of RTX and set the benefits compare to conventional SLE treatment.

**Patient Consent:** Yes, I received consent

**Disclosure of Interest**: None declared

**Table 1 (abstract P298).** Dynamics of SLE features pre-rituximab and during rituximab treatment

Parameter	SLE onset	RTX (baseline)	p_1_	Last visit (LV)	p_2_	p_3_
SLEDAI	23 (16.0; 26)	18,0 (8,0; 26,0)	0.003	4 (2,0; 8.0)	0.0015	0.000001
ANA, titer	5120 (1280;10240)	820 (240; 3200)	0.345	640 (160; 1280)	0.515	0.0014
Anti-dsDNA, U/ml (n.v.<25)	150 (48; 237)	36 (7,7;148)	0.263	12 (0; 65)	0.05	0.001
Proteinuria, g/l	1.0 (0.5; 3,5)	0.6 (0.3; 3.7)	1.0	0,17 (0; 0.9)	0.05	0.0008
Hematuria, # cells	15 (0; 42)	15 (5; 50)	0.170	0 (0; 2,5)	0.088	0.003
C4, g/l	0.11 (0.06; 0.4)	0.13 (0.06; 0.24)	0.715	0.16 (0.1; 0.2)	0.042	0.0066
Active LN n, (%)	25 (100)	15 (60.0)	0.001	8 (32.0)	0.048	0.001
Patients with GCS therapy n, (%)	23 (92)	24 (96)	0.522	16 (64)	0.005	0.004
Corticosteroids, mg/kg	1.0 (0.7; 1.0)	0.8 (0.23; 1.0)	0.148	0.2 (0.1; 0.9)	0.001	0.0002

p_1_ comparison between onset and RTX baseline, p_2_ – comparison between RTX baseline and LV, p_3_ – comparison throughout the study

### P299. Turkish translation and adaptation of the pediatric automated neuropsychological assessment metrics (pedanam) for childhood-onset systemic lupus erythematosus: initial findings

#### M. Kasap Cuceoglu^1^, S. Demir^2^, E. S. Akbas Aliyev^3^, P. Avar Aydin^4^, E. Aliyev^1^, E. Cengel Kultur^3^, S. Ozen^1^, T. Cak^3^, Y. Bilginer^1^

##### ^1^Pediatric Rheumatology, Hacettepe University, Ankara, ^2^Pediatric Rheumatology, Erzurum City Hospital, Erzurum, ^3^Child and Adolescent Psychiatry, Hacettepe University, ^4^Pediatric Rheumatology, Ankara University, Ankara, Turkey

###### **Correspondence:** M. Kasap Cuceoglu

**Introduction:** Childhood-onset SLE is associated with major organ involvement, including neuropsychiatric disease (NPSLE) is easy to overlook unless it’s obvious. The Pediatric Automated Neuropsychological Assessment Metrics (Ped-ANAM) is a computerized battery that measures cognitive ability, mental processing speed, and memory.

**Objectives:** We aimed to study the psychometric properties of the Turkish version of the the Ped-ANAM and compare to a formal cognitive testing tool in SLE patients.

**Methods:** We performed Ped-ANAM, and the Wechsler Intelligence Scale for Children-IV (WISC-IV) as a formal neuropsychological testing tool on 30 cSLE, synchronously at Hacettepe University Ankara. All participants were assessed by a child and adolescent psychiatrist with Schedule for Affective Disorders and Schizophrenia for School-Age Children Present and Lifetime Version, DSM-5- (K- SADS-PL-DSM-5-T) to rule out current psychiatric disorders. PedANAM is an automatic battery that only requires a computer without the need for other specialized equipment; a psychometric specialist consists of 10 subtests and four performance parameters. The WISC-IV measures intellectual performance as a multidimensional construct. The WISC-IV contains 10 core subtests and 4 indexes namely, Verbal Comprehension Index, Perceptual Reasoning Index, Working Memory Index, and Processing Speed Index. SLE patients were divided into two groups as present/absent neurocognitive dysfunction (NCD). NCD definition: At least one of the WISC-IV was below -2 standard deviations, or at least two were between -1 and -2 standard deviations.

**Results:** Thirty patients with cSLE (male, 30%) were recruited for the study. The median age of cSLE patients was 16.6 (11-20) years, and the median follow-up period for cSLE was 59 months (6-180). When the SLEDAI-K activity indexes of the patients were evaluated, there was no severe disease activity in both groups. 12 (40%) of 30 cSLE patients in the study met the criteria for NCD. There was no significant difference according to age, gender, SLEDAI-K disease activity index, organ involvement, depression, anxiety, and other psychiatric symptoms between NCD and without NCD groups. When we compared the median (min_max) of PedANAM-CPS indices of the two groups; NCD SLE patients had lower CPSpca than without NCD SLE [-1.75 (min:-13.30, max:1.47) vs. 0.19 (min:-13.30 max:2.69), p = 0.087] and CPSmultiscore was higher in NCD SLE than without NCD SLE [8.50 (2.93 – 17.30) vs. 5.64 (1.65 – 15.22), p = 0.043]. There were statistically significant differences in simple reaction time (p=0.014), spatial processing (p=0.036), attention/processing speed (p=0.059), spatial processing/matching grids (p=0.053) in PED-ANAM subtests. Six Ped-ANAM subtests (simple reaction time, attention/proc. speed, learning, logical relations-reasoning, spatial processing, delayed memory) significantly correlated with WIS-C-IV subsets (vocabulary, coding measures, picture concepts, similarities, block design, perceptual reasoning, verbal comprehension, coding, symbol search) in overall two groups (Spearman’s correlation coefficient >0.4, P < 0.05; n=30).

**Conclusion:** The findings of this study represent the results that the Turkish version of Ped-ANAM can also be used in the clinic for this purpose. The significant difference between groups in subtest scores indicates that Ped-ANAM supports this view.

**Patient Consent:** Yes, I received consent

**Disclosure of Interest**: None declared

### P300. Vitamin D levels in patients with juvenile systemic lupus erythematosus

#### D. Klepárník, Z. Pytelová, K. Bouchalová

##### Paediatric Rheumatology, Department of Paediatrics, Faculty of Medicine and Dentistry, Palacky University Olomouc and University Hospital, Olomouc, Czech Republic

###### **Correspondence:** D. Klepárník

**Introduction:** Juvenile Systemic lupus erythematosus (jSLE) is a heterogenous disease. The prevalence of hypovitaminosis D in patients with jSLE was determined to be between 36 and 96%(Rezaei E. 2020). According to the available literature, no study has yet looked at vitamin D levels in jSLE patients at the time of diagnosis. Double-blind randomized trial proved the effect of vitamin D supplementation in patients suffering from jSLE (Lima GL 2016).

**Objectives:** Comparison of 25-OH vitamin D levels in patients with jSLE at the time of diagnosis and levels measured at the last follow-up.

**Methods:** Twenty-five patients were included in this study. Data were collected from September 2021 to February 2022. Patients who suffered from jSLE, most met SLICC or ACR classification criteria. All patients were treated with standard jSLE therapy and substituted with vitamin D based on their baseline levels. The normal range for serum 25-hydroxyvitamin D is 75–250 nmol/L (Charoenngam N 2020). A comparison of vitamin D levels at the time of diagnosis and last follow-up with a reference value of 75 nmol/L was performed by a nonparametric Wilcoxon single-sample test. A comparison of concertation at the time of diagnosis level and last follow-up was performed using the Wilcoxon paired test.

**Results:** The patients mean age was 15.71, SD ± 2.964. The mean age at the time of diagnosis was 14.49 years, SD ± 3.12 years. Vitamin D levels at the time of diagnosis were statistically significantly lower than 75 nmol/L (**p = 0.020**). The level of vitamin D at the last control was statistically significantly higher than 75 nmol/L (**p = 0.023**). Vitamin D levels at the time of jSLE diagnosis were significantly lower (**p <0.0001**) than at the last follow-up.

**Conclusion:** Initially, 18 patients had suboptimal levels of vitamin D. At the last follow-up, only 3 patients had suboptimal levels. Our results suggest a good effect of vitamin D supplementation. We demonstrated that low vitamin D levels were present in patients at the time of diagnosis of jSLE. To date, no published study has investigated the status of vitamin D levels at the time of jSLE diagnosis.

**Patient Consent:** Not applicable (there are no patient data)

**Disclosure of Interest**: None declared

### P301. Creation of a core dataset for international registry-based research on childhood-onset systemic lupus erythematosus

#### L. B. Lewandowski^1^, R. E. Sadun^2^, J. C. Cooper^3^, A. Belot^4^, E. M. Smith^5^

##### ^1^Pediatric Rheumatology, National Institutes of Health, Rockville, ^2^Rheumatology, Duke University, Durham, ^3^Pediatric Rheumatology, University of Colorado, Denver, United States, ^4^Pediatric Rheumatology, Centre Hospitalier Universitaire de Lyon, Lyon, France, ^5^Pediatric Rheumatology, University of Liverpool, Liverpool, United Kingdom

###### **Correspondence:** R. Sadun

**Introduction:** Childhood-onset systemic lupus erythematosus (cSLE) occurs in approximately 20% of all SLE cases. cSLE is associated with more severe disease, often requires more aggressive treatment than adult-onset SLE, and has been under-researched compared to its adult counterpart. Regional cSLE registries exist in some countries to capture demographics, disease features, and outcomes. International, iter-registry collaborations could allow pooling of data across registries, with the potential to greatly advance cSLE outcomes research by improving patient numbers. However, differences in collection methodology and data fields have been major barriers.

**Objectives:** To obtain international consensus around standardized data collection that enables high-quality research on patients with cSLE.

**Methods:** To identify essential, “core” data elements for registry-based research in studies of children and adolescents with cSLE, 21 SLE experts (15 peds rheum, 4 med/peds or adult rheum, 2 peds nephrology) with international representation (10 countries; 5 continents) completed two sequential Delphi surveys to indentify areas of consensus and disagreement prior to a virtual consensus meeting. The virtual consensus meeting was conducted over two days in May 2022 and used adapted nominal group technique to discuss and vote on data elements to be included in the core dataset. Consensus required ≥ 80% agreement for inclusion.

**Results:** Twenty (95%) experts responded to the first Delphi questionnaire and 21 (100%) to the second questionnaire. Twenty-one physicians and 4 patients/caregivers attended the consensus meeting, all with equal votes. The group achieved consensus for core data collection for cSLE registries in the following areas: demographics, SLE classification criteria, visit data, laboratory testing, kidney biopsy data, disease activity measurements, damage index, medications, and severe adverse events (Table 1). In addition, consensus was achieved regarding frequency of data collection (every 3 months + flare visits). Experts agreed that an additional follow-up meeting will be needed to clarify data elements pertaining to patient reported outcome measures.

**Conclusion:** A core dataset for registry-based international childhood-onset SLE research was defined by global expert consensus. The core dataset considers unique aspects of children with childhood-onset SLE in a range of geographic and resourced settings. The core dataset will facilitate international collaborative research for children and adolescents with childhood-onset SLE worldwide.

**Trial registration identifying number:** Not applicable

**Patient Consent:** Not applicable (there are no patient data)

**Disclosure of Interest**: L. Lewandowski Grant / Research Support with: CARRA-PReS Collaborative Award, R. Sadun Grant / Research Support with: CARRA-PReS Collaborative Award, J. Cooper Grant / Research Support with: CARRA-PReS Collaborative Award, A. Belot Grant / Research Support with: CARRA-PReS Collaborative Award, E. Smith Grant / Research Support with: CARRA-PReS Collaborative Award

**Table 1 (abstract P301).** Data elements voted into the “Core cSLE Dataset” by consensus meeting participants (≥ 80% agreement)

Data Categories	Data Elements
Demographics	Date of birth, country of residence, sex, gender, ancestry, date of SLE symptom onset, date of SLE diagnosis, family history of SLE in a 1st degree relative
SLE Classification Criteria	2012 SLICC, 2019 EULAR/ACR
Visit Data	Blood pressure, height, weight, menarche status, menstrual cycle status
Disease Activity	SLEDAI-2K, physician global assessment, patient/parent global assessment
SLE-related Damage Index	SLICC Damage Index
Laboratory Tests	Complete blood count, c-reactive protein, erythrocyte sedimentation rate, ANA, ENA, dsDNA, antiphospholipid antibodies, C3/C4, IgG, creatinine, albumin, urine protein:creatinine, urine microscopy
Kidney Biopsy Data	ISN/RPS class, biopsy date (for each biopsy)
Medications	Steroids, immunomodulators, anti-hypertensives, lipid-lowering agents, anticoagulants, contraception, anti-depressants, anti-epileptics
Serious Adverse Events	Hospitalization, ESRD, dialysis, kidney transplant, pregnancy, malignancy, death

### P302. Myocarditis as an initial manifestation of pediatric systemic lupus erythematosus and associated with SARS COV-2

#### H. F. F. Menchaca Aguayo^1^, P. P. Ramos Tiñini^2^, E. R. Mercedes-Perez^1^, E. Faugier^3^, A. Barba^1^, A. Guzman^4^, K. Primero^4^, H. Bermudez^1^

##### ^1^Pediatric Rheumatology, Hospital Infantil de Mexico Federico Gomez, ^2^Pediatric Rheumatology, Hospital Infantil de México Federico Gómez, ^3^Pediatric Rheumatology, Hospital Infantil de Mexico Federico Federico, Ciudad de Mexico, ^4^Reumatología Pediátrica, Hospital Infantil de México Federico Gomez, Ciudad de México, Mexico

###### **Correspondence:** H. F. F. Menchaca Aguayo

**Introduction:** Currently, after the onset of the COVID 19 pandemic, there has been an increase in cases of autoimmune diseases, this in relation to the viral pathophysiology that triggers an autoimmune disruption in a genetically predisposed individual (1). The incidence of myocarditis as a cardiovascular manifestation in pediatric Systemic Lupus Erythematosus Erythematosus (pSLE) is approximately 2% to 19% (2), however, myocarditis triggered by SARS-CoV-2 has been described 2 to 3 weeks after infection (3).

**Objectives:** To describe the case of a patient with debut of pSLE with carditis and its association with SARS-CoV-2.

**Methods:** Description of a clinical case and review of the literature.

**Results:** A 16-year-old female with 6 months of evolution with constitutional syndrome, myalgias, arthralgias and hair loss; 3 days before, medium effort dyspnea was added. On admission she was tachycardic, polypneic, oximetry 80%, supplemental oxygen was administered. Physical examination: pale, frontal alopecia, bilateral cramps and left basal hypoventilation. Laboratory tests: normal chromic anemia, normal renal and hepatic function tests, normal renal and hepatic function tests. Proteinuria 12 hours 24.3 mg/m2/h, PCR SARS-CoV-2 negative, Ig G SARS-CoV-2 positive. Chest X-ray: left pleural effusion and cardiomegaly (CI 0.68). TTE: structurally healthy heart, trivial pericardial effusion, moderate LV systolic dysfunction, moderate TR and mild MI. SLE was diagnosed (ACR 1997 criteria) due to: serositis, proteinuria, positive lupus anticoagulant (1.61) and homogeneous ANA pattern 1: 640. One day after admission he presented respiratory and hemodynamic deterioration, requiring aminergic support and non-invasive mechanical ventilation and was admitted to the PICU. Pulses of methylprednisolone 30 mg/kg/day for 5 days and human immunoglobulin 2 g/kg/day were administered, as well as treatment for heart failure: carvedilol, lisinopril, spironolactone and furosemide. She presented clinical improvement and after 5 days she was discharged from PICU.

**Conclusion:** Our patient presented carditis as the initial manifestation of SLE and positive Ig G serology for SARS CoV-2, so this infection could have been the trigger for the clinical manifestation and decompensation of the disease.

In the context of COVID-19 pandemic and in the presence of patients with systemic clinical symptoms, autoimmune diseases should be considered and in the presence of cardiovascular manifestations it is necessary to perform specific inflammatory markers and cardiological screening evaluations.

**Patient Consent:** Yes, I received consent

**Disclosure of Interest**: None declared

### P303. Mofetil mycophenolate versus cyclophosphamide in juvenile systemic lupus erythematous: a long-term follow-up study

#### A. Meneghel^1^, A. Carriero^2^, G. Martini^2^, F. Tirelli^2^, L. Iaccarino^3^, F. Zulian^2^

##### ^1^Pediatric Rheumatology Unit, Department for Women and Child Health, University Hospita of Padova, ^2^Pediatric Rheumatology Unit, Department for Women and Child Health, ^3^Rheumatology Unit, University Hospital of Padova, Padova, Italy

###### **Correspondence:** A. Meneghel

**Introduction:** In the last decades, the mortality rate of Juvenile Systemic Lupus Erythematosus (jSLE) has significantly decreased but the morbidity has increased due to both the severity of the disease and side effects of immunosuppressive therapy. Thus, while there is a growing interest towards the identification of treatment protocols that would allow a better control of the disease, data regarding the long-term prognosis of jSLE are limited so far.

**Objectives:** To compare the long-term prognoses of two groups of jSLE patients treated with different therapeutic protocols: Cyclophosphamide (CYC) and Mofetil Mycophenolate (MMF).

**Methods:** We performed a retrospective cohort study including jSLE patients, diagnosed according to ACR 1997 and/or EULAR/ACR 2019 criteria, followed in our Unit between 2003 and 2021. Patients were divided into two treatment groups: CYC and MMF. Clinical, demographic, laboratory variables, therapies, disease activity index (SLEDAI-2K), damage index (SLICC/SDI) and disease flares (SFI index) were collected at the time of diagnosis (T0), at treatment initiation (Tstart), at 6 months (T6) and annually after treatment initiation (T12, T24, T36, T48, T60). Patients were defined as “active disease” (AD), “disease in clinical remission on medication” (CRM) and “disease in clinical remission off medication” (CR) according to the most recent definitions. The groups were compared for clinical variables (by Fisher exact test and Mann–Whitney U test) and treatment efficacy over time (by mixed-design analysis of variance model) considering statistically significant a p-value <0.05.

**Results:** 20 patients (15 F) were included: CYC=12 (9 F), MMF=8 (6 F). No statistically significant differences between the two groups were found for clinical and demographic characteristics at T0 (Table). During the follow-up, mean SLEDAI-2K showed a significant reduction (p<0.001) from Tstart (CYC=19.9±5.8; MMF=22.9±5.5) in both groups at T12 (CYC=6.9±5.0; MMF=6.3±5.3), T24 (CYC=9.1±10.0; MMF=4.3±2.7), T60 (CYC=11.7±5.0; MMF=7.7±7.6), showing no statistically significant difference between the 2 treatments. As for the doses of corticosteoids and SFI, no statistically significant differences were detected between the 2 groups at any timepoints. We found a statistically significant difference in SDI between the 2 groups at T6 (p=0.028), T 12 (p=0.015), T24 (p=0.04), T36 (CYC=5, MMF=0; p=0.029). Of interest, 1 patient in the MMF group achieved full clinical remission at both T48 and T60.

**Conclusion:** CYC and MMF present comparable efficacy both in the short- and long-term follow up, independently from the use of corticosteroids. Patients treated with MMF showed a significantly lower cumulative damage as defined by SDI over time. Although with the limit of its retrospective nature and small size of the cohort, our study, confirms the role of MMF as first-line treatment for jSLE, also considering the higher risk of side effects on reproductive system reported with CYC. Future prospective studies on larger samples are needed to confirm these results.

**Disclosure of Interest**: None declared

**Table 1 (abstract P303).** See text for description

Variable	CYC N=12	MMF N=8	TOT N=20	P value
**Age at Tstart (years)–m±DS**	14,50±1,98	14,00±2,56	14,30±2,18	0,969
**Untreated disease duration (days)–m±DS**	456,08±661,50	145,88±146,08	153,00±534,33	0,374
**SLEDAI-2K at T0–m± DS**	18,08±7,59	18,87±7,99	18,40±7,55	0,846
**WBC < 4000/mm3 – n (%)**	7 (58,3)	4 (50,0)	11 (55,0)	>0,999
**Hb < 11 g/l – n (%)**	9 (75,0)	4 (50,0)	13 (65,0)	0,356
**PTL < 150000/mm3 – n (%)**	4 (33,3)	2 (25,0)	6 (30,0)	>0,999
**ESR > 28 mm/h – n (%)**	5 (41,7)	5 (62,5)	10 (50,0)	0,650
**C3 <0 ,90 g/L – n (%)**	11 (91,7)	7 (87,5)	18 (90,0)	0,999
**Proteinuria > 0,5 g/die – n (%)**	7 (58,3)	4 (50,0)	11 (55,0)	0,999

### P304. Hematological manifestations in the presence of antiphospholipid antibodies in a pediatric cohort

#### P. Morán Álvarez^1,2^, Á. Andreu ^1^, L. Caballero^3^, S. Gassiot Riu^4^, R. Berrueco Moreno^4^, M. Vazquez Diaz^1^, J. Calzada Hernandez^4^, L. Giovannelli^5^, A. Boteanu^1^, J. Antón^4^, F. De Benedetti^2^, C. Bracaglia^2^

##### ^1^Hospital Universitario Ramón y Cajal, Madrid, Spain, ^2^Ospedale Pediatrico Bambino Gesù, Rome, Italy, ^3^Hospital Universitario Gregorio Marañón, Madrid, ^4^Hospital Sant Joan de Deu, Barcelona, ^5^Ospedale Pediatrico Bambino Gesù, Rome, Spain

###### **Correspondence:** P. Morán Álvarez

**Introduction:** Antiphospholipid syndrome (APS) is an autoimmune disease characterized by thrombotic events (TEs) and/or pregnancy morbidity, in association with two consecutive positive determinations (at least 12 weeks apart) of antiphospholipid antibodies (aPLs). Several manifestations which are not considered clinical criteria of APS have been identified, including hematological disorders.

**Objectives:** To identify the different variables associated with the development of hematological manifestations in the presence of aPLs in a pediatric cohort.

**Methods:** Multicentric historical cohort of children £ 18 years from three Spanish and one Italian tertiary referral hospitals with at least 2 positive determinations (^3^12 weeks apart) of IgG/IgM anticardiolipin (aCL), IgG/IgM anti β2-glycoprotein I (aβ2GP) and/or LA. Demographic, clinical and analytical data were collected. A bivariate and multivariate analysis were carried out. Multicollinearity was also explored to build the model.

**Results:** One hundred and thirty-one children were included in the study. The median age was 10.9 5.1 years and 64.9% were female. Most of them were Caucasians (81.4.7%). Thirty-three (25.2%) children had a positive family history for autoimmune diseases and 34 (26%) a personal history for systemic lupus erythematosus (SLE). Fifty-five (42%) patients developed at least one hematological manifestation. Of them, we registered: 22.9% thrombocytopenia; 8.4% leukopenia; 5.3% hemolytic anemia; and 1.5% Evans syndrome. Related to thrombotic events (TE), 16 (12.2%) children had at least one TE. Significant differences between the groups (patients with hematological manifestations and patients without hematological manifestations) in terms of age, gender, race, TE and family history for autoimmune diseases were not identified. However, a higher prevalence of SLE was found among the children with hematological manifestations (36.4% vs 18.4%; p=0.021).

Other clinical manifestations, immunological parameters and results of bivariant analysis are shown in Table 1.

The multivariate analysis identified as independent risk factors to develop a hematological manifestation, children with: SLE diagnosis [OR 1.2, 95 CI (0.5-5.8), p= 0.015], cutaneous manifestations [OR 1.01, 95 CI (0.6 -2.8), p= 0.091], LA + [OR 0.8, 95 CI (0.4 -3.6), p= 0.058], and IgG aβ2GP + [OR 0.9, 95 CI (0.4 - 3.9), p= 0.048]. Sex, age, familiar history of AIDs or IgM B2GP+ did not show a higher risk.

**Conclusion:** Non-criteria manifestations, especially hematological disorders, are the most frequent events in the presence of aPLs in our cohort. Cutaneous manifestations, a positive personal history of SLE, LA and IgG aβ2GP positivity were associated with a higher risk of developing hematological manifestations. Therefore, their inclusion as APS classification criteria should be considered.

**Patient Consent:** Yes, I received consent

**Disclosure of Interest**: None declared

**Table 1 (abstract P304).** See text for description

Variables	Total n= 131	Hematological manifestations n= 55	Absence of hematological manifestations n= 76	P-value
**Neurological, n (%)**	26 (20)	10 (18.2)	16 (21.3)	0.657
Chorea	8 (6.1)			
Epilepsy	5 (3.8)			
Migraine	4 (3)			
**Cutaneous, n (%)**	23 (17.6)	6 (10.9)	17 (22.4)	0.089
Raynaud	17 (13)			
Livedo	5 (3.1)			
**Cardiac, n (%)**	22 (16.8)	12 (21.8)	10 (13.2)	0.191
**Renal, n (%)**	2 (1.5)	0 (0)	2 (2.6)	0.225
**aCL, n (%)**				
IgG	54 (41.2)	24 (43.6)	30 (39.5)	0.633
IgM	31 (23.7)	20 (36.4)	11 (14.5)	0.004*
**aB2GP, n (%)**				
IgG	59 (45)	29 (52.7)	30 (39.5)	0.132
IgM	24 (18.3)	16 (29.1)	8 (10.5)	0.007*
**LA, n (%)**	76 (58.5)	37 (67.3)	39 (52)	0.081
**aPL profile, n (%)**				
Simple	76 (58)	25 (45.5)	51 (67.1)	
Double	30 (22.9)	13 (23.6)	17 (22.4)	0.008*
Triple	25 (19.1)	17 (30.9)	8 (10.5)	
**ANA +, n (%)**	72 (59.5)	33 (66)	39 (54.9)	0.222

### P305. Different patterns of longitudinal changes in antinuclear antibodies titres in children with systemic lupus erythematosus

#### P. Morán Álvarez, C. Bracaglia, V. Messia, I. Caiello, L. Giovannelli, F. De Benedetti, E. Marasco

##### Ospedale Pediatrico Bambino Gesù, Rome, Italy

###### **Correspondence:** P. Morán Álvarez

**Introduction:** Systemic lupus erythematosus (SLE) is an autoimmune disease characterized by the presence of antinuclear antibodies (ANA). Monitoring of anti-DNA antibody levels can reflect disease activity, by contrast a single anti-RBP antibody determination is thought to suffice for clinical purposes. Recently, evidence suggests that ANA levels may decrease over time secondary to the natural history of lupus or to treatments.

**Objectives:** To investigate the trend of ANA and anti-dsDNA autoantibodies titers over time in children with a diagnosis of SLE.

**Methods:** We enrolled 15 children with a diagnosis of SLE, fulfilling the SLICC criteria. For all patients included in the study ANA and anti-dsDNA antibodies testing was carried out from diagnosis every 3-4 months for 2 years. ANA were defined as negative for titers lower than 1:80. Laboratory parameters, clinical and demographic data was retrieved and analyzed.

**Results:** Following two years of follow-up, all patients had ANA titers significantly lower than at time of SLE diagnosis (Mann Whitney test, p=0.0002). After two years of follow-up, 11 patients (73%) still had positive ANA (group 1), while 4 patients (26%) had negative ANA (group 0). At time of diagnosis no significant differences in ANA titers (Mann Whitney test, p=0.74) nor in disease activity, as measured by SLEDAI, (Mann Whitney test, p=0.88) were observed (table 1). No significant differences in organ involvement were observed (table 1). Assessing the change over time in ANA titers, the 2 groups of patients showed two different patterns: in group 0, ANA titers quickly declined and disappeared in the first 6 months after diagnosis; in group 1, ANA titers declined more slowly, remaining positive at 2-year follow-up. Both C3 and C4 increased in the follow-up period, with no different patterns between the 2 groups. Similarly, anti-dsDNA antibodies titers declined over time with no clear different patterns between the 2 groups.

**Conclusion:** Our analysis showed two different patterns in the reduction of ANA titers over time in patients with childhood onset SLE, with 26% of patients becoming ANA negative after 6 months from diagnosis and remaining persistently negative during follow-up. Our data have important implications, specifically for the recruitment of patients into clinical trials, where the latest classification criteria of SLE require ANA positivity as entry criterion. Moreover, a seronegative state may represent a different subcategory of patients with SLE with specific pathogenetic pathways involved, possibly independently from autoantibodies. Therefore, further studies are needed to confirm and expand our data.

**Patient Consent:** Yes, I received consent

**Disclosure of Interest**: None declared

**Table 1 (abstract P305).** See text for description

	ANA- at 2y	ANA+ at 2y
Patients (n)	4	11
Female n(%)	3 (25)	10 (9)
Age (mean (SD))	13.80 (1.91)	13.45 (2.72)
Disease duration (mean (SD))	6.47 (4.22)	6.17 (2.71)
SLICC criteria:		
Acute cutaneous, n (%)	4 (100.0)	6 (54.5)
Chronic cutaneous, n (%)	0 (0.0)	0 (0.0)
Alopecia, n (%)	0 (0.0)	1 (9.1)
Oral or nasal ulcers, n (%)	2 (50.0)	7 (63.6)
Arthritis, n (%)	3 (75.0)	4 (36.4)
Serositis, n (%)	0 (0.0)	2 (18.2)
Renal, n (%)	1 (25.0)	2 (18.2)
Neurological, n (%)	0 (0.0)	0 (0.0)
Hemolytic anemia, n (%)	1 (25.0)	4 (36.4)
Leukopenia, n (%)	4 (100.0)	9 (81.8)
Thrombocytopenia, n (%)	3 (75.0)	5 (45.5)
ANA+, n (%)	4 (100.0)	11 (100.0)
Anti-dsDNA+, n (%)	4 (100.0)	11 (100.0)
Anti-Sm+, n (%)	1 (25.0)	2 (18.2)
LAC, n (%)	0 (0.0)	1 (9.1)
anti-cardiolipin, n (%)	2 (50.0)	2 (18.2)
anti-beta2GPI+, n (%)	2 (50.0)	2 (18.2)
Low complement, n (%)	4 (100.0)	11 (100.0)
C3, mean (SD) mg/dL	40.25 (5.51)	52.73 (20.81)
C4, mean (SD) mg/dL	3.67 (2.89)	4.60 (2.72)
SLEDAI at diagnosis, mean (SD)	12.75 (4.50)	12.18 (7.14)
SLEDAI at last follow-up, mean (SD)	0.00 (0.00)	1.36 (1.43)
Treatment:		
PDN, n (%)	3 (75.0)	11 (100.0)
HCQ, n (%)	4 (100.0)	11 (100.0)
MMF, n (%)	4 (100.0)	11 (100.0)
RTX, n (%)	0 (0.0)	2 (18.2)

### P306. Multi-parametric interrogation of the Systemic Lupus Erythematosus (SLE) immunome reveals multiple derangements

#### K. Nay Yaung^1,2^, J. G. Yeo^1,2,3^, M. Wasser^1^, P. Kumar^1^, S. P. Tang^4^, S. K. Ng^4^, S. L. Poh^1^, T. Arkachaisri^2,5^, S. Albani^1^

##### ^1^Translational Immunology Institute, Singhealth/Duke-NUS Academic Medical Institute, ^2^Duke-NUS Medical School, ^3^KK Women’s and Children’s Hospital, Singapore, Singapore, ^4^Paediatric Rheumatology Unit, Department of Paediatrics, Selayang Hospital, Kuala Lumpur, Malaysia, ^5^Paediatric Rheumatology & Immunology, KK Women’s and Children’s Hospital, Singapore, Singapore

###### **Correspondence:** K. Nay Yaung

**Introduction:** Systemic lupus erythematosus (SLE) is a complex, systemic autoimmune disease that interferes with the balance between regulation and immunity, resulting in immune system dysfunction. Disease course is unpredictable due to alternating remissions and flares, impeding reliable patient assessments. Thus, mechanistic insights are required for better assessment. Current studies are mainly descriptive, and SLE is best interrogated with a multi-parametric, holistic approach such as mass cytometry (CyTOF). We hypothesize that significant differences exist between the immunomes of newly diagnosed SLE patients and healthy subjects.

**Objectives:** The objectives of this study are as follows: (1) To characterise immune signatures of newly diagnosed SLE patients and in the process: Study the roles of B and T cells in SLE to gain a holistic understanding of the adaptive immune response, (2) To compare immunological profiles of newly diagnosed SLE patients with age-matched healthy controls and (3) Build a database of CyTOF data for paediatric SLE patients so that comparisons can be made with adult SLE data in the future.

**Methods:** Peripheral blood mononuclear cells (PBMCs) of 5 SLE subjects (median age 125 months) were tested with CyTOF. Data was uploaded to an online analytical database, the Extended Polydimensional Immunome Characterization (EPIC) discovery tool, for comparison with 51 age-matched controls. Normalization and FlowSOM (Flow cytometry analysis by Self-Organising Maps) clustering to 50 nodes were performed with 37 functionally and phenotypically important immune markers. Mann-Whitney U test identified significantly different cluster frequencies.

**Results:** Correspondence analysis comparing global differences in cluster frequencies showed segregation of SLE subjects away from healthy controls. Multiple significant differences were identified (p < 0.05). Notably, a memory CD4+CD152+PD1+ T cell subset (CD4+CD152+PD1+CD45RO+CD25-FoxP3-) was enriched in SLE (median: 2.17%, interquartile range: 1.66 to 7.74% of CD45+ PBMCs) versus control (1.34%, 1.06-1.58%; p = 0.00267). Expression of known checkpoint inhibitors (PD1, CD152) could be important contributors to SLE immunopathogenesis. Secondly, the innate lymphoid cell 2 (ILC2) subset (Lin-CD7+CD25+CD127+GATA3+) was markedly depressed in SLE (0.11%, 0.1-0.255%) versus control (0.41%, 0.25 - 0.55%; p = 0.0293). ILC2s protect epithelial integrity; a reduction suggests impaired protective roles in SLE. It would be interesting to study in further detail the changes in other protective and regulatory compartments. Supervised cell frequencies from bivariate analysis correlate strongly with unsupervised cell frequencies, validating these results (Pearson’s correlation coefficient r = 0.9926, p < 0.001 (CD4+CD152+PD1+CD45RO+CD25-FoxP3-); r = 0.8863, p < 0.05 (ILC2)).

**Conclusion:** With a multi-parametric unbiased approach comparing SLE subjects to a database of age-matched healthy controls, we identified two immune subsets of potential immunopathogenic importance for further studies. This would guide the redesign of the CyTOF panel to include more functionally important organ-specific markers such as skin-homing or kidney-homing receptors. Data obtained from this study will also form the basis of the paediatric SLE database for comparison with adult SLE CyTOF data, which we have commenced work on.

**Patient Consent:** Yes, I received consent

**Disclosure of Interest**: None declared

### P307. Eosinophilic fasciitis associated with inaugural juvenile systemic lupus erythematosus

#### I. Pizarro Madureira Salgado Da Costa^1^, L. Fernandes^2^, M. P. Ramos^3^

##### ^1^Unidade de Reumatologia Pediátrica, Centro Hospitalar Universitário Lisboa Central, ^2^Unidade de Radiologia, Centro Hospitalar UniversitárioLisboa Central, ^3^Unidade de Reumatologia Pediátrica, Centro Hospitalar Universitário Lisboa Central, EPE, Lisboa, Portugal

###### **Correspondence:** I. Pizarro Madureira Salgado Da Costa

**Introduction:** Eosinophilic fasciitis is a rare disorder presenting with skin edema and erythema, followed by collagenous thickening of the subcutaneous fascia. Its pathogenesis is poorly understood and been associated with multiple conditions, such as strenuous exercise, exposure to certain medications as well as hematological and autoimmune diseases.

**Objectives:** Increase awareness for the diagnosis of eosinophilic fasciitis

**Methods:** Report of a patient with eosinophilic fasciitis and juvenile systemic lupus erythematosus

**Results:** A 17-year-old girl was referred to a tertiary hospital with a 9-month history of fatigue and migratory arthralgias. In the previous month, she also reported limb edema and a generalized photosensitive rash. Her medical history was unremarkable, with no regular medication. Her sister had been diagnosed with juvenile systemic lupus erythematosus. Physical examination revealed a disseminated maculopapular lupus rash with scaly appearance, as well as a malar rash. There was also bilateral erythema, edema and thickening of the skin of the four limbs and trunk, sparing the fingers and toes. Laboratory investigations showed non regenerative anemia, eosinophilia, elevated erythrocyte sedimentation rate, elevated transaminases and LDH; positive ANA, anti-double-stranded DNA, anti-nucleosome, antihistone, RF and CCP; low levels of C3 and functional CH50 complement. Direct Coombs test, creatine-kinase, creatinine, urea, electrolytes, urine analysis and thyroid hormones were within normal limits. Systemic sclerosis antibody panel and antiphospholipid antibodies were negative. Diffusing capacity for carbon monoxide, cardiac ultrasound and ECG were normal. Nailfold capillaroscopy revealed a normal pattern. Skin biopsy revealed an infiltrate of lymphocytes and plasma cells surrounding the superficial and deep vessels, the adipocytes and the skin derivatives in the dermis. In immunofluorescence, a dense linear pattern of IgM was found in the dermoepidermal junction. Whole-body MRI showed edema and thickening of the fascia and subcutaneous tissue, affecting the trunk and the limbs. The diagnosis of juvenile erythematous lupus and eosinophilic fasciitis was established and treatment with high dose steroids and hydroxychloroquine was started. During the following months, clinical stability was achieved and steroids were gradually tapered. However, skin edema recurred and she developed an irregular, *peau d'orange* texture and a “groove sign” of the arms. Methotrexate was added after nine months of the diagnosis. The patient responded favorably and steroids were suspended. During the last 2 years of follow-up there was no recurrence of the eosinophilic fasciitis.

**Conclusion:** Eosinophilic fasciitis is a scleroderma-like syndrome predominantly affecting the extremities, sporadically seen in children. In contrast to systemic sclerosis, sclerodactyly, Raynaud’s phenomenon, internal organ involvement and nailfold capillaries abnormalities are uncommon. Early recognition and treatment are essential in order to prevent long-term disabling outcomes in eosinophilic fasciitis. Myalgias, fatigue and, less frequently, arthritis are commonly associated symptoms. Eosinophilia is a prominent laboratory finding in the early phase of this disease. MRI is an important tool for the diagnosis, particularly when the skin biopsy is inconclusive. Eosinophilic fasciitis has been described in association with collagen tissue diseases, such as Sjögren’s syndrome and systemic lupus erythematosus. To the best of our knowledge, this is the first reported case of juvenile eosinophilic fasciitis associated with inaugural systemic lupus erythematosus.

**Patient Consent:** Yes, I received consent

**Disclosure of Interest**: None declared

### P308. Levamisole and autoimmunity- wondering about wonder drugs

#### A. Prabhudesai, A. Khan, N. P. Maldar, R. Khubchandani

##### Pediatric Rheumatology, NH SRCC Children’s Hospital, Mumbai, India

###### **Correspondence:** A. Prabhudesai

**Introduction:** Drug induced lupus (DIL) has a clinical phenotype resembling that of idiopathic systemic lupus erythematosus (SLE) but usually without any major organ complications and which resolves after drug withdrawal. The drugs commonly implicated in DIL include hydralazine, procainamide, anti-tuberculous drugs like isoniazid and more recently reported tumor necrosis factor α (TNFα) inhibitors. Levamisole is a synthetic Imidathiazole derivative, originally used as an anthelminthic with potent immunomodulatory properties. This low-cost drug has been widely used in dermatology especially in developing countries for the management of various dermatoses including Alopecia areata. Although not a commonly recognized cause of DIL, Levamisole has been associated with widespread autoimmunity mainly in the form of cutaneous vasculitis and Antineutrophil cytoplasmic antibodies (ANCA) associated vasculitis with a few reports on lupus-like side effects.

**Objectives:** To alert practitioners about Levamisole induced autoimmunity so as to enable early diagnosis and avoid an extensive diagnostic work up.

**Methods:** A 7-year-old girl had developed patchy alopecia on the scalp for which she had been taking Levamisole 3mg/kg twice a week for 2 years. Results were satisfactory and she did well for about 1.5 years while continuing the drug. However, at this point in time she was referred to the rheumatology department for fever, arthralgia, mouth ulcers, increased hair fall and a positive Antinuclear antibody (ANA) (titre 1+). On examination, besides the patchy alopecia, she also had short stature (121cm; between 3^rd^ -10^th^ percentile), pallor and bilateral hallux valgus. Her spine Xray showed bullet shaped vertebrae and the labs suggested a Coombs positive hemolytic anemia, with normal complements. In light of these findings and the presence of consanguinity, albeit distant, we suspected a monogenic cause of lupus and a whole exome sequencing was advised. The genetic report showed a homozygous nonsense variation in exon 12 of the PAPSS2 gene (c.1666C>T) (Brachyolmia Type 4 with Mild Epiphyseal and Metaphyseal Changes (OMIM 612847)). This explained the skeletal findings, but it failed to explain the autoimmunity. Since hemolytic anemia and disseminated autoimmunity have been reported with Levamisole, she was advised to omit Levamisole and was kept under follow up.

**Results:** Her symptoms improved rapidly without the need for steroids or immunomodulators. After 4 months her general condition improved. Fever and arthralgias subsided and hemoglobin improved while Coombs turned negative and the acute phase reactants normalised. We thus concluded it to be Levamisole induced autoimmune haemolytic anemia. Only the alopecia was persistent for which she was subsequently started on Methotrexate 12mg/m^2^ subcutaneously. She showed dramatic improvement and now has full hair growth and no systemic complaints.

**Conclusion:** Levamisole is a widely used immunomodulatory drug. Knowledge about its potential to induce autoantibodies should caution practitioners against its long-term use and enable early diagnosis of levamisole induced autoimmunity with less aggressive therapeutic strategies.

**Patient Consent:** Yes, I received consent

**Disclosure of Interest**: None declared

### P309. Reduced CD8+ T Cell and CD56+ natural killer cell cytotoxic capacity in patients with juvenile systemic lupus erythematosus

#### A. Radziszewska^1,2^, H. Peckham^1,2^, N. M. de Gruijter^1,2^, O. Nettey^1,2^, M. Butt^1,2^, E. C. Jury^2^, E. C. Rosser^1,2^, C. Ciurtin^1,2^

##### ^1^Centre for Adolescent Rheumatology Versus Arthritis at UCL, UCLH, GOSH, ^2^Centre for Rheumatology Research, Division of Medicine, University College London, London, United Kingdom

###### **Correspondence:** A. Radziszewska

**Introduction:** CD8^+^ T cells and NK (natural killer) cells are cytotoxic lymphocytes which play a role in adaptive and innate immune responses to infection. They exert their effector functions by releasing toxic proteins such as perforin and granzymes to kill target cells. Recent evidence suggests that aberrations in cytotoxic cell function can contribute to tissue damage in autoimmunity. SLE (Systemic Lupus Erythematosus) is a multisystem autoimmune disease characterized by production of autoantibodies against nuclear antigens. While B cells and CD4^+^ T cells have been studied extensively in SLE, there is limited and at times contradictory evidence of how cytotoxic cells contribute to disease pathology, in particular, in the more severe, juvenile form of the disease (JSLE).

**Objectives:** The aim of this study is to investigate the phenotype and function of cytotoxic lymphocytes in patients with JSLE compared to age/sex-matched healthy controls (HCs).

**Methods:** Peripheral blood mononuclear cells (PBMCs) and serum were collected from JSLE patients (n=40, 15.6-29.8 years), and age/sex-matched healthy controls (n=51, 15.2-31.3 years). *Ex vivo* PBMCs and PBMCs cultured for 72hr with anti-CD3 were stained with anti-CD3, -CD8, -CD56, -CD45RO, -CD132, -perforin, -granzyme A, -granzyme B, -granulysin, -CD107a, -IFN-γ, -TNF-α, and -IL-2 antibodies and analysed by flow cytometry. Serum perforin levels were measured using a multiplex bead-based assay in 17 patients and 18 HCs. NK function was assessed by incubating isolated NK cells from JSLE patients or HCs together with CFSE labelled K562 target cells for 4hrs. After co-incubation cells were stained with DRAQ7 dye to identify non-viable cells via flow cytometry. Statistical significance was calculated using Mann-Whitney U tests or t-tests as appropriate.

**Results:** Patients with JSLE had diminished frequencies of CD8^+^ T cells expressing perforin (p=0.028) and effector cytokines IFN-γ (p=0.029) and TNF-α (p=0.01) compared to HCs. These cytotoxic and pro-inflammatory molecules were in large part produced by memory CD8^+^ T cells and frequencies of effector memory and central memory CD8^+^ T cell subsets were reduced in peripheral blood in JSLE (p=0.0048 and p=0.0017, respectively). In addition, the proportions of NK cells as a whole (p=0.000012), as well as perforin (p=0.000006) and granzyme A (p=0.00013) expressing NK cells were markedly diminished in JSLE patients versus HCs. This was accompanied by a reduction in serum perforin concentration in JSLE (p=0.025). Reductions in cytotoxic capacity in JSLE were observed regardless of treatment regimen or patient disease activity. The expression of CD8^+^ T cell cytotoxic markers and intracellular cytokines was rescued by stimulation of the T cell receptor in vitro with anti-CD3, indicating that a mechanistic defect in cytotoxic mediator/cytokine production is unlikely in JSLE. NK functional killing assays revealed that NK cells from patients with JSLE were as effective at killing labelled targets as NK cells from HCs (mean % specific target death 12.27 vs 11.12, respectively, p=0.71).

**Conclusion:** Cytotoxic capacity of CD8^+^ and NK cells as well as CD8^+^ memory T cell and NK cell frequencies were reduced in patients with JSLE. NK cell cytotoxic function appeared to be preserved in JSLE. Further work is necessary to establish if there is a functional defect in CD8^+^ T cells in JSLE and to ascertain if the reduction in cytotoxic cell frequencies is due to migration of these cells out of peripheral blood into affected tissues.

**Patient Consent:** Not applicable (there are no patient data)

**Disclosure of Interest**: None declared

### P310. Lupus nephritis - study from a single centre in South India

#### S. Ravindran^1^, D. Patro^2^, A. P. Rao^2,3^, J. Raghuram^3,4^

##### ^1^Pediatrics, ^2^Pediatric Rheumatology, Manipal Hospital, Ola Airport Road, Bangalore, ^3^Pediatric Rheumatology, Indira Gandhi Institute of Child Health, ^4^Pediatric Rheumatology, Aster Whitefield, Bangalore, India

###### **Correspondence:** S. Ravindran

**Introduction:** Systemic Lupus Erythematosus (SLE) is a chronic multiorgan autoimmune inflammatory disease and nephritis is a major risk factor for morbidity and mortality in SLE.

**Objectives:** To analyze the clinical and laboratory profile of children with lupus nephritis in a cohort of SLE patients attending a pediatric rheumatology clinic in SouthIndia.

**Methods:** We retrospectively reviewed 60 cases of biopsy proven lupus nephritis from a total of 134 patients with SLE over a period of 10 years (April 2011-March 2021) in the Pediatric Rheumatology Clinic in Manipal hospital, Bangalore, India.

**Results:** The findings are summarized in the table below. Out of 134 children with SLE, 60 (44.7%) had biopsy-proven LN. 60% had renal involvement at the onset, 88.3% within 6 months, and 100% patients had presented within 2 years of onset of the disease. Class IV (41.7%) was most common followed by class III (25%).

**Conclusion:** Majority of the children who develop LN, do so in the initial 2 years of the disease onset. Anti dsDNA antibody elevation was seen in majority of patients. Presence of anti dsDNA might indicate increased risk of developing LN and should be followed more closely.

**Patient Consent:** Yes, I received consent

**Disclosure of Interest**: None declared

**Table 1 (abstract P310).** See text for description

	Our study (10 years)	Hari P et al (10 years)	Srivastava et al (25 years)	S.Singh et al (24 years)	Samanta et al (12 years)
Sample size	60	54	134	72	46
Male: female	1:4.45	1:2.9	1:9.3	1:3.23	1:1.7
Number of biopsy proven cases	60	54	92	53	41
Mean age of diagnosis of LN (years)	11.9± 3.38	10.4±2.6	13.5±3.7	10.5±2.1	10.2±2.43
Duration between diagnosis of SLE and LN	3.53± 10 months	NR	1.87±2.5 years	9.4±12.6 months	8.5±10 months
Lupus nephritis at presentation	60%	NR	82.8%	76.45%	82.6%
**Clinical features**	**n=60**	**n=54**	**n=92**	**n=72**	**n=46**
Fever	88.3%	62.9%	98.9%	87.5%	NR
Arthritis	58.33%	42.6%	78.2%	52.8%	NR
Hypertension	40%	55.6%	32.1%	NR	NR
Headache	33.33%	NR	7.6%	NR	NR
CNS involvement	18.33%	14.8%	31.5%	23.6%	28.2%
Photosensitivity	51.67%	22.2%	NR	38.9%	NR
Oral/palatal ulcer	40%	29.6%	31.5%	NR	NR
**Laboratory features**					
ANA (+)	100%	96.3%	97.7%	94.4%	97.8%
Anti-dsDNA(+)	91.11%	57.4%	85.4%	87.5%	82.6%
Low C3	60.34%	NR	75.8%	81.8%	76.09%
**Renal biopsy**					
Class II	16.67%	18.51%	14.2%	18.9%	26.09%
Class III	25%	11.1%	26%	1.9%	23.91%
Class IV	41.66%	48.1%	46.7%	66.0%	30.43%
Class V	3.33%	12.9%	13.1%	13.2%	8.7%
Class III+V	8.33%	NR	NR	NR	NR
Class IV+V	1.67%	NR	NR	NR	NR
Class II+III	1.67%	NR	NR	NR	NR

NR: Not Reported

### P311. Prevalence and predictors of damage in childhood-onset systemic lupus erythematosus: preliminary data from an international cohort of 1096 patients

#### S. Sahin^1^, E. M. Smith^2,3^ on behalf of the UK JSLE Study Group, A. Corley^2,3^ on behalf of the UK JSLE Study Group, C. Ciurtin^4^, S. Demir^5^, D. Schonenberg-Meinema^6^, N. Kifer^7^, A. L. Rodríguez-Lozano^8,9^, E. Marasco^10^, C. Bracaglia^10^, A. Rodrigues Fonseca^11^, M. F. Rodrigues^11^, G. Kavrul Kayaalp^12^, N. Aktay Ayaz^12^, S. Tharwat^13^, E. Marrani^14^, K. Bouchalova^15^, D. Kleparnik^15^, S. K. Feitosa de Oliveira^16^, B. Kasap-Demir^17,18^, P. Costa-Reis^19^, T. Kallinich^20^, A. S. L. von Stuckrad^20^, K. Tenbrock^21^, K. Vollbach^21^, E. Demirkaya^22^, M. J. Wahadat^23,24^, H. C. Culpan^25^, M. Jelusic^7^, S. Ozen^5^, O. Kasapcopur^1^, M. W. Beresford^3,26^ on behalf of the UK JSLE Study Group, S. Kamphuis^24^

##### ^1^Department of Pediatric Rheumatology, Istanbul University Cerrahpasa, Istanbul, Turkey, ^2^Department of Women’s & Children’s Health, Institute of Life Course and Medical Sciences, University of Liverpool, ^3^Department of Paediatric Rheumatology, Alder Hey Children’s NHS Foundation Trust, Liverpool, ^4^Centre for Adolescent Rheumatology, University College London, London, United Kingdom, ^5^Department of Pediatrics, Division of Rheumatology, Hacettepe University Faculty of Medicine, Ankara, Turkey, ^6^Department of Pediatric Immunology, Rheumatology and Infectious Diseases, Amsterdam UMC, University of Amsterdam, Emma Children’s Hospital, Amsterdam, Netherlands, ^7^Department of Paediatrics, University of Zagreb School of Medicine, University Hospital Centre Zagreb, Zagreb, Croatia, ^8^Servicio de Inmunología, Instituto Nacional de Pediatría, ^9^Programa de Maestría y Doctorado en Ciencias Médicas, Odontológicas y de la Salud, Universidad Nacional Autónoma de México (UNAM), Mexico City, Mexico, ^10^Division of Rheumatology, Bambino Gesù Children’s Hospital, IRCCS, Rome, Italy, ^11^Pediatric Rheumatology Unit , Universidade Federal do Rio de Janeiro, Instituto de Puericultura e Pediatria Martagão Gesteira, Rio de Janeiro, Brazil, ^12^Department of Pediatric Rheumatology, Istanbul Faculty of Medicine, Istanbul University, Istanbul, Turkey, ^13^Rheumatology & Immunology Unit, Department of Internal Medicine, Faculty of Medicine, Mansoura University, Mansoura, Egypt, ^14^Pediatric Rheumatology Unit, Aou Meyer, Firenze, Italy, ^15^Paediatric Rheumatology, Department of Paediatrics, Faculty of Medicine and Dentistry, Palacky University Olomouc and University Hospital, Olomouc, Czech Republic, ^16^Universidade Federal do Rio de Janeiro, Rio de Janeiro, Brazil, ^17^Department of Pediatrics Division of Nephrology & Rheumatology, İzmir Katip Çelebi University, Izmir, ^18^Department of Pediatrics, Health Sciences University İzmir Tepecik Training and Research Hospital, Izmır, Turkey, ^19^Pediatric Rheumatology Unit, Hospital de Santa Maria, Faculdade de Medicina, Universidade de Lisboa, Lisbon, Portugal, ^20^Department of Pediatric Respiratory Medicine, Immunology and Critical Care Medicine, Charité Universitätsmedizin Berlin, Berlin, ^21^Department of Pediatrics, RWTH Aachen University Hospital, Aachen, Germany, ^22^Department of Paediatrics, Division of Paediatric Rheumatology, Schulich School of Medicine & Dentistry, University of Western Ontario, London, ON, Canada, ^23^Department of Immunology, Erasmus Medical Center, University Medical Centre Rotterdam, ^24^Department of Paediatric Rheumatology, Erasmus MC Sophia Children’s Hospital, Rotterdam, Netherlands, ^25^Department of Public Health, Istanbul University Cerrahpasa, Istanbul, Turkey, ^26^Department of Women’s & Children’s Health, Institute of Life Course and Medical Sciences, University of Liverpool, Liverpool, United Kingdom

###### **Correspondence:** S. Sahin

**Introduction:** A higher damage score is a strong predictor of increased mortality in lupus patients.

**Objectives:** To describe the prevalence, predictors and organ-distribution pattern of damage in a large multinational cohort of cSLE patients.

**Methods:** Detailed data on all items of PedSDI, ACR 1997, SLICC 2012 and EULAR/ACR 2019 were collected over the entire disease course for patients from 19 centers across Europe, Africa, Middle East, North and South America

**Results:** Overall, 1096 cSLE patients were included; One-third (32.5%) of the participants were >18 years of age at last visit. Distribution of ethnicity was as follows: 62% White, 18% Asian, 11% Black (African, Afro-Caribbean etc.), 5 % Mixed and 4% Latin American. After a mean disease duration of 6 years, 35% of the patients had accrued damage (PedSDI damage score of ≥1) in at least one PedSDI domain; 20.2% of all study group had a PedSDI score of 1; 7.1% had a score of 2; 4.1% had a score of 3 and the remaining 3.6% had a score of ≥4. The mean PedSDI score at last visit was 0.62 ± 1.1 (range 0–7). Participants with early onset disease (<10 years) were more likely to have damage as compared to those with later onset disease (≥10 years) (p<0.05). Among all items of the 3 classification criteria, the presence of discoid rash (p<0.05), renal disease (p <0.01), neurological disorder/domain (p<0.001), chronic cutaneous lupus (p<0.05), anti-Sm positivity (p<0.05), lupus anticoagulant (p <0.01), anticardiolipin antibodies (p<0.05), or constitutional symptoms/signs (p<0.05) was associated with damage. Other damage predictors (not included in 3 classification criteria) were as follows: systemic hypertension (p<0.001), fulfillment of Sapporo classification criteria for antiphospholipid antibody syndrome (p<0.001), anti-RNP positivity (p<0.05), anti-SSA positivity (p<0.05), anti-SSB positivity (p<0.05), corticosteroid (p<0.001) and cyclophosphamide usage (p<0.001). No correlation was found between PedSDI and SLEDAI-2K scores neither at disease onset nor at last visit.

**Conclusion:** This PReS Lupus Working Party initiative multicenter study, one of the largest multinational cohort of patients with cSLE to date, indicate that one-third of the cSLE patients develop damage after an average disease duration of 6 years and this was not correlated with SLEDAI-2K scores.

**Patient Consent:** Yes, I received consent

**Disclosure of Interest**: None declared

### P312. Results of whole exome sequencing in a large cohort of patients with childhood-onset systemic lupus erythematosus - multiple case study

#### M. Sestan^1^, N. Kifer^1^, M. Frkovic^1^, D. Grguric^1^, S. Barisic^1^, J. Ellyard^2^, M. Cook^2^, T. Arsov^3^, C. G. Vinuesa^4^, M. Jelusic^1^

##### ^1^University of Zagreb School of Medicine, UHC Zagreb, Zagreb, Croatia, ^2^Department of Immunology and Infectious Diseases, The John Curtin School of Medical Research, Australian National University, Canberra, Australia, ^3^Institute of Immunobiology and Human Genetics, Faculty of Medicine, University in Skopje, Skopje, North Macedonia, ^4^The Francis Crick Institute, London, United Kingdom

###### **Correspondence:** M. Sestan

**Introduction:** Childhood-onset systemic lupus erythematosus (cSLE) is a complex disease with not fully understood pathogenesis that may involve multiple interactions between genetic, epigenetic and environmental factors. Genes may play a more important role than environmental and hormonal factors in cSLE, as opposed to adult-onset SLE.

**Objectives:** The purpose of the research was to point out new and rare gene variants using the whole exome sequencing (WES) in patients with cSLE that may be involved in the pathogenesis and to expand existing genetic databases.

**Methods:** This multiple case study included patients with cSLE, based on the revised classification ACR-97 criteria and the 2012 SLICC criteria, reviewed in University Hospital Centre Zagreb in the period 1991 to 2019. We performed WES on selected 17 “trios” containing proband case with cSLE and parents, including other informative family members, with severe, atypical clinical features, syndromic characteristics, early onset of the disease, resistance to conventional therapy and/or family pattern of occurrence.

**Results:** Overall, in 7 patients with cSLE the total number of 17 novel and/or rare variants that may contribute to disease was discovered. One variant was classified as pathogenic according to the American College of Medical Genetics (ACMG) classification guidelines. This was structural, frame-shift variant in exon 34 of *KMT2D* gene (NM_003482.3:c.8626delC; 55 reads C, 56 reads delC), predicted to truncate the protein (p.Gln2876Serfs*34), occurred *de novo* in patient with cSLE complicated with lupus nephritis, neurolupus, immunodeficiency and severe intestitial lung disease and previously unrecognized syndrome features consistent with Kabuki syndrome. In addition, we identified four likely pathogenic variants. Two of them were very rare heterozygous mutations: the first in *ADAR1* gene (NM_001111.3:c.2815A>G; 13 reads A, 15 reads G), predicted to encode the protein (p.Ile939Val), and the second in *SH2B3* gene (NM_005475.2:c.398G>A; 7 reads G, 6 reads A) predicted to encode (p.Cys133Thr). Both of them were present in a patient with syndromic features, skin pigmentation changes and cSLE, complicated with lupus nephritis, CNS involvement, immunodeficiency and coagulopathy. The third one was heterozygous mutation in *BLK* gene (NM_001715.2:c.211G>A) predicted to encode (p.Ala71Thr), with low prevalence in population databases, in a patient with cSLE complicated with myocarditis, nephritis and pancreatitis, inherited from the mother affected with SLE. The same patient and her mother carry rare frame-shift variant in *CR1* gene (NM_000651.4:c.4052dup) predicted to encode the protein (p.Asp1351Glufs*23). We have also identified a number of variants classified as variants of uncertain significance (VUS). These are gene variants that encode proteins involved in various cellular signaling pathways, especially related to tyrosine phosphorylation; differentiation, survival, and proliferation of cells; V(D)J recombination in developing lymphocytes; regulation of apoptosis and Th1 and Th2 polarization.

**Conclusion:** Although application of WES has improved the diagnostics and treatment of patients with autoimmune diseases, there is growing number of variants that we still cannot use in the clinical context. It is necessary to improve methods to properly categorize them and increase the amount of practicable information from WES.

**Patient Consent:** Yes, I received consent

**Disclosure of Interest**: None declared

### P313. Clinical and epidemiological analysis of systemic lupus erytheis according to the moscow register of children with rheumatic diseases

#### V. Sevostyanov^1^, P. Lototskaya^2^, E. Zholobova^3^

##### ^1^Department of Propaedeutics of Children’s Diseases, ^2^Department of Rheumatology No. 1 of the University Children’s Clinical Hospital, ^3^Department of pediatrics, I.M. Sechenov First Moscow State Medical University Ministry of Health of Russia (Sechenov University), Moscow, Russian Federation

###### **Correspondence:** V. Sevostyanov

**Introduction:** Systemic lupus erythematosus is a systemic autoimmune disease of unknown etiology, which is based on a genetically determined violation of immune regulation, which determines the formation of organ-specific antibodies to antigens of cell nuclei with the development of immune inflammation in the tissues of various organs.

**Objectives:** Make a clinical and epidemiological analysis of systemic lupus erythematosus (SLE) according to the Moscow register of children with rheumatic diseases.

**Methods:** An observational descriptive cross-sectional study was conducted, including 65 patients diagnosed with systemic lupus erythematosus, aged 7 to 17 years (inclusive), of which 74% (n=48) were girls and 26% (n=17) were boys. All patients live in Moscow and are included in the city register of children with rheumatic diseases.

**Results:** The median age of patients was 16 years, the median age at the disease onset was 12.5 years. The time interval from the onset of the disease to the verification of the diagnosis averaged 8 months. Half of the patients (n=33) had an initial diagnosis other than SLE. When verifying the diagnosis, from the clinical criteria of SLICC-2012, the most common patients had acute and subacute skin lesions - 48%, thrombocytopenia - 35%, arthritis - 32% and leukopenia - 31%. According to the nature of the course of SLE, an acute course was detected in 27.7% of patients, subacute - in 67.7%, and primary chronic - in 4.6% of patients. When assessing the activity of the disease in most patients, the activity was assessed as minimal (40.0%) or its absence was noted (30.8%). Basic anti-inflammatory therapy was received by 98.5% of patients, 87.5% of them in a combination of several drugs. Genetically engineered biological therapy is received by 23% of patients. In the structure of GIBT, rituximab predominates - 73.3%.

**Conclusion:** SLE is one of the most complex diseases in childhood, to improve the prognosis of which requires timely diagnosis and early initiation of antirheumatic therapy.

**Patient Consent:** Yes, I received consent

**Disclosure of Interest**: None declared

### P314. Clinical profile of pediatric systemic lupus erythematosus from a hilly state in north-india: with an uncommon complication

#### J. Sheekha^1^, A. Sharma^2^, S. Guleria^2^

##### ^1^Pediatrics, ^2^Immunology and Pediatric Rheumatology, Dr Rajendra Prasad Governement Medical college, Tanda at Kangra, HP, Kangra, India

###### **Correspondence:** J. Sheekha

**Introduction:** Systemic Lupus Erythematosus (SLE) is a prototype of autoimmune diseases. It is characterized by multisystem inflammation due to exaggerated B- cell and T-cell responses and loss of immune tolerance against self-antigens. Pediatric SLE (pSLE) in comparison to adult onset SLE presents with more severe symptoms at the onset of disease and has a more aggressive clinical course. Avascular necrosis of the hip can occur as a complication of the disease itself or that of treatment.

**Objectives:** To study the clinical profile and presence of osteonecrosis as a complication of pSLE patients from Dr Rajendra Prasad Government Medical College (DRPGMC), Kangra, Himachal Pradesh, India.

**Methods:** Case records of children with systemic lupus erythematosus registered in Pediatric Rheumatology Clinic (PRC) at DRPGMC from July 2017 to Dec 2022 were reviewed for the demographic and clinical characteristics and associated complications, particularly osteonecrosis (ON). We here report this data and cases of pSLE who developed osteonecrosis of hip joints over course of their illness and treatment.

**Results:** During the above-mentioned period, a total of 17 pSLE patients were registered in PRC. Out of which 15 were females (F:M=7.5:1). 24% patients were in the age group of 6-10 years, 35% between 11-15 years and 16-18 year age group comprised 41% of the total SLE patients. There were no patients below 5 years of age. Fever and arthralgia/arthritis were the most common manifestation at the time of presentation followed by rash and photosensitivity. Other clinical features noted were alopecia, headache, myalgia, Raynaud phenomenon and oral ulcers. 45% patients were positive for dsDNA. Hypocomplementemia was seen in 75% of patients at the time of diagnosis. Lupus nephritis was noted in 35% (6/17) of patients. 2 patients belonged to class III nephritis, one each to class II, IV and V. Renal biopsy was not done in one patient. All the patients were initiated on hydroxychloroquine. Two patients developed macrophage Activation Syndrome (MAS) during the clinical course. Interestingly, 2 female patients developed symptomatic osteonecrosis of bilateral hips diagnosed 7 years and 1 year after diagnosis and initiation of treatment. Both had received pulse methylprednisolone and were on tapering doses of oral gluco-corticoids. They were treated with core decompression and autologous bone marrow infiltration. Both were doing well on follow-up after 2 and 2.5 years of surgery.

**Conclusion:** DISCUSSION

Osteonecrosis (ON) or avascular necrosis (AVN) is, though uncommon, a known complication of pSLE. It can occur in patients with pSLE due to disease or as a complication of treatment (cortico-steroids). It has been reported to occur in these patients even without initiation of steroids and can occur early after initiation of cortico-steroids.

CONCLUSION

SLE is a multisystem disorder and has varied clinical presentations most common being fever, arthritis, and nephritis. Use of corticosteroids in the treatment of SLE makes it challenging to distinguish between the role of disease itself and that of treatment in the pathogenesis of AVN. Regular screening and preventive measures like the use of bisphosphonates along with vitamin D and calcium might help.

**Patient Consent:** Yes, I received consent

**Disclosure of Interest**: None declared

### P315. Defining childhood-onset systemic lupus erythematosus, lupus nephritis, and end-stage kidney disease within a us administrative claims database

#### E. Smitherman^1^, R. A. Chahine^1^, A. O. Hersh^2^, J. R. Curtis^1^

##### ^1^University of Alabama at Birmingham, Birmingham, ^2^University of Utah, Salt Lake City, United States

###### **Correspondence:** E. Smitherman

**Introduction:** Data is lacking regarding modern real-world practices and disease outcomes of individuals with childhood-onset systemic lupus erythematosus (cSLE) and lupus nephritis, especially across multiple centers. However, researchers also lack approaches to identify end-stage kidney disease (ESKD) in patients with cSLE utilizing claims-based algorithms.

**Objectives:** To determine optimal definitions that can be used to identify patients with cSLE, lupus nephritis, and ESKD using a large, real-world administrative claims database.

**Methods:** We utilized administrative claims data from IBM MarketScan Commercial and 8-State Medicaid Databases from 2006-2020. We adapted previously validated definitions to identify patients with cSLE: 1) ≥ 3 ambulatory or inpatient claims with ICD-9 code 710.0 or ICD-10-CM code M32.* (excluding M32.0 for drug-induced lupus) with at least 30 days between each code; 2) codes restricted to provider type pediatric rheumatology, rheumatology, pediatric nephrology, nephrology, dermatology, or acute care hospital; and 3) age ≥ 5 and <18 years at the time of the first SLE code. To define incident diagnoses, we selected patients with: 1) at least 182 days between insurance enrollment and the first SLE code; and 2) no evidence of prior anti-malarial or immunosuppressive use more than 182 days before the first SLE code. We then adapted previously validated definitions to select the subgroup of cSLE patients with evidence of lupus nephritis using ≥ 2 ambulatory or inpatient claims with ICD-9 codes 580.*-586.*, 791.0 or ICD-10-CM codes M32.14, M32.15 with at least 30 days between each code. Finally, we defined presence of ESKD using: 1) procedure and diagnosis codes for dialysis from any encounter; 2) procedure and diagnosis codes for kidney transplant from any encounter; or 3) ≥ 3 claims with diagnosis codes for ESKD from any encounter type. We considered each ESKD criteria separately and as a composite if any of the 3 criteria were met. We calculated the frequency of patients meeting each of these criteria.

**Results:**

From 2006-2020, we identified 2,590 individuals receiving care for cSLE in the IBM MarketScan Commercial and 8-State Medicaid Databases, of which 1,302 met criteria for an incident diagnosis. Definition for lupus nephritis was met in 580 (45%) of incident cSLE cases. Of individuals meeting the lupus nephritis definition, 166 (29%) had evidence of ESKD: 100 (17%) with dialysis, 83 (14%) with kidney transplant, and 69 (12%) with other ESKD diagnosis codes. Only 35 (4%) of 722 individuals with incident cSLE who did not meet lupus nephritis definition had evidence of ESKD: 24 (3%) with dialysis, 9 (1%) with kidney transplant, and 2 (0.3%) with other ESKD diagnosis codes (Table 1).

**Conclusion:** This study of individuals with evidence of cSLE and lupus nephritis within a US administrative claims database revealed a relatively high frequency of meeting ESKD criteria among cSLE patients. We identified few individuals with evidence of ESKD who did not meet previously validated lupus nephritis definitions; nevertheless, ESKD care should be included in the definition of lupus nephritis when using administrative claims algorithms.

**Disclosure of Interest**: None declared

**Table 1 (abstract P315).** Frequency of individuals meeting lupus nephritis and ESKD criteria out of the total number of defined incident cSLE cases within IBM MarketScan Commercial and 8-State Medicaid Databases from 2006-2020 (n=1,302)

	Meets Lupus Nephritis Criteria (n=580)	Does Not Meet Lupus Nephritis Criteria (n=722)
No ESKD criteria, n (%)	414 (71)	687 (95)
Any ESKD criteria, n (%)	166 (29)	35 (5)
Dialysis, n (%)	100 (17)	24 (3)
Kidney transplant, n (%)	83 (14)	9 (1)
ESKD diagnosis codes, n (%)	69 (12)	2 (0.3)

### P316. Association of sociodemographic factors with differential diagnostic coding patterns in childhood-onset lupus nephritis

#### E. Smitherman^1^, R. A. Chahine^1^, A. O. Hersh^2^, J. R. Curtis^1^

##### ^1^University of Alabama at Birmingham, Birmingham, ^2^University of Utah, Salt Lake City, United States

###### **Correspondence:** E. Smitherman

**Introduction:** Disparities in long-term kidney outcomes have been documented in patients with childhood-onset systemic lupus erythematosus (cSLE) complicated by lupus nephritis (LN). However, there remains limited understanding of the impact of health care quality, including access to care, on disease outcomes.

**Objectives:** To evaluate early care utilization patterns as a measure of access in individuals identified with cSLE and LN using a large, real-world database.

**Methods:** We utilized administrative claims data from IBM MarketScan Commercial and 8-State Medicaid Databases from 2006-2020. We identified individuals with cSLE using: 1) ≥3 ambulatory or inpatient claims with ICD-9 710.0 or ICD-10-CM M32.* (excluding M32.0) with ≥30 days between each code; 2) provider type pediatric rheumatology, rheumatology, pediatric nephrology, nephrology, dermatology, or acute care hospital; and 3) age ≥5 and <18 years at the time of the first SLE code. We then selected=“selected” patients with: 1) ≥182 days between insurance enrollment and the first SLE code; and 2) no evidence of anti-malarial or immunosuppressant use >182 days prior to the first SLE code to define incident cases. Finally, we identified cSLE patients with LN by ≥2 ambulatory or inpatient claims with ICD-9 580.*-586.*, 791.0 or ICD-10-CM M32.14, M32.15 with ≥30 days between. We abstracted sex, age at first SLE code, codes for dialysis, codes for kidney transplant, and time between first SLE and first LN code. For patients enrolled in commercial insurance, we abstracted US geographic region and population density. For those enrolled in Medicaid, we abstracted race and ethnicity. We divided patients by whether SLE codes or LN codes were used first. Descriptive statistics and bivariate analyses were conducted with significance levels set to 0.05.

**Results:** We identified 580 individuals who met criteria for both incident cSLE and LN. Patients with LN were 82% female and had a mean(SD) age at first SLE code of 14.5(2.9) years. In patients with commercial insurance, the majority were from the US South (51%), followed by West (18%), Northeast (16%), and Midwest (15%). We noted that while there was a mean(SD) time between first SLE and LN diagnosis codes of 0.5(1.5) years, there was a range from -9.5 to 9.7 years. We grouped patients into those who received SLE codes first (77%) and those with LN codes first (23%). In the LN codes first group, we noted a statistically higher proportion of patients with Medicaid (43% vs 29%, p=0.002), living in a rural metropolitan statistical area (14% vs 9%, p=0.032), of Black or unknown race (Table 1), and of Hispanic ethnicity (28% vs 8%, p<0.001). Patients with LN codes first were also more likely to have evidence of kidney transplant.

**Conclusion:** In this group of individuals identified with cSLE and LN, we noted two distinct patterns of whether patients first received SLE diagnosis codes or LN diagnosis codes. Patients who received LN codes before SLE codes were more likely to be publicly insured, live in a rural area, be Black or Hispanic, and have evidence of kidney transplant. Additional analyses are planned to better understand if these patterns reflect heterogeneous disease course or differential access to subspecialty care.

**Disclosure of Interest**: None declared

**Table 1 (abstract P316).** Characteristics of patients with incident childhood-onset lupus nephritis identified in IBM MarketScan Commercial and 8-State Medicaid Databases from 2006-2020

	Incident cSLE-LN Cohort (n=580)	SLE codes first (n=445)	LN codes first (n=135)	p-value
Race, n (%) (Medicaid only)				0.007
Black	82 (44)	59 (46)	23 (40)	
White	29 (16)	20 (16)	9 (16)	
Other	13 (7)	10 (8)	3 (5)	
Unknown	63 (34)	40 (31)	23 (40)	
Ever dialysis, n (%)	100 (17)	73 (16)	27 (20)	0.33
Ever kidney transplant, n (%)	83 (14)	56 (13)	27 (20)	0.03

### P317. Anti-SARS-COV2 vaccination in juvenile systemic lupus erythematosus: tolerability and impact on disease activity

#### I. Suardi^1,2^, C. B. Chighizola^1,2^, M. Gattinara^3^, G. Carrea^1,2^, L. M. Argolini^1^, R. Caporali^1,2^, M. Gerosa^1,2^

##### ^1^Unit of Clinical Rheumatology, ASST Pini – CTO, Milan, Italy, ^2^University of Milan, ^3^Unit of Pediatric Rheumatology, ASST Pini – CTO, Milan, Italy, Milan, Italy

###### **Correspondence:** I. Suardi

**Introduction:** In approximately 10-20% of cases, systemic lupus erythematosus (SLE) begins in pediatric age; juvenile SLE (jSLE) tends to have a severe presentation.

SLE has a remitting course; infections and vaccinations have been proposed as potential triggers of disease flares. Therefore, the safety of anti-SARS-COV2 vaccination has been questioned in jSLE.

**Objectives:** The aim of this study consists in evaluating side effects and disease flare after anti-SARS-COV2 vaccination in a monocentric cohort of patients with jLES.

**Methods:** We retrospectively reviewed the clinical records of patients with jSLE diagnosed before 18 years of age regularly followed-up at our institution. The following data were collected: demographic details, clinical and serological features, ongoing pharmacological treatment, anti-SARS-COV2 vaccination, side effects and disease flare after vaccination. Low lupus disease activity state (LLDAS) was defined according to SLEDAI; disease flare was defined as a new BILAG A or B score in any system. Continuous data are expressed as median (interquartile range [IQR]) and categorical data as percentages. The association between categorical variables was assessed by chi-squared or Fisher exact tests. Univariate logistic regression analyses were drawn. Statistical analysis was performed using Stata v14.

**Results:** Out of the 70 patients with jLES, 65 subjects (93%) received at least one dose of anti-SARS-COV2 vaccine (75% Pfizer-BioNtech, 25% Moderna, Table 1). A 3-dose immunization schedule was completed in 46 cases (46% Pfizer, 26% Moderna, 28% heterologous booster). Adverse events were registered in 28 cases (43%); 24 of the 46 patients completing the vaccination schedule (52%) had side effects after the third shot. The most common side effects were: fever (26%), fatigue (23%), pain at the injection site (21%) and arthromyalgias (23%). Ongoing treatment with immunosuppressors and belimumab was associated with an increased risk of adverse events (odds 2.88, p=0.04 and odds 5.5, p=0.03, respectively). Patients with previous musculoskeletal involvement had more commonly side effects to anti-SARS-COV2 vaccine (p=0.066).

A disease flare occurred in 7 cases: 5 after the first and second shot (71%) and 2 after the third (29%), at a median time of 41 days and 27 days after vaccination, respectively. Flares consisted in lupus nephritis (71%) and arthritis (28.6%). 5 subjects experiencing a lupus flare had a moderate disease activity before starting the vaccination program, while 2 patients were in remission. Overall, patients with LLDAS before first vaccination dose displayed a lower rate of flare (p=0.059, χ2=5.25).

**Conclusion:** Our data suggest that anti-SARS-COV2 vaccination is well tolerated among patients with jLES, even though patients receiving immunosuppressors and/or belimumab have an increased hazard of side effects. Disease flares after anti-SARS-COV2 vaccination occur rarely in patients with LLDAS.

**Patient Consent:** Not applicable (there are no patient data)

**Disclosure of Interest**: None declared

**Table 1 (abstract P317).** See text for description

	2 doses (n=65)	3 doses (n=46)
**Gender (F, %)**	95%	93%
**Age at onset [median (IQR)]**	15 (12-16)	15 (12-16)
**Disease duration at first dose [months, median (IQR)]**	192 (109-332)	229 (143-345)
**Age at first dose [years, (IQR)]**	32 (25-41)	33 (26-45)
**Ongoing immunosuppressors, n**	25	18
**Ongoing belimumab, n**	9	9

### P318. Epstein–BARR virus encephalitis-associated hemophagocytic lymphohistiocytosis in a patient with childhood-onset systemic lupus erythematosus: a case report

#### K. Cheawcharnprapan, T. Kanjanaphan, O. Lertkovit, O. Sirimongkolchaiyakul, S. Tangcheewinsirikul

##### Paediatrics, Faculty of Medicine Vajira Hospital, Navamindradhiraj University, Bangkok, Thailand

###### **Correspondence:** S. Tangcheewinsirikul

**Introduction:** Epstein–Barr virus (EBV) encephalitis-associated hemophagocytic lymphohistiocytosis (HLH) syndrome causes high morbidity and mortality. Rarely reported in patients with systemic lupus erythematosus (SLE), EBV encephalitis-associated HLH is even rarer in patients with childhood-onset SLE (c-SLE).

**Objectives:** Here, we report the unique case of a 12-year-old female patient with c-SLE who presented with neuropsychiatric (NP) manifestations.

**Methods:** Diagnostic workup included basic and immunological blood tests, cerebrospinal fluid (CSF) analysis, brain imaging, and bone marrow assessment.

**Results:** Two months before admission, the patient was diagnosed with c-SLE based on the presence of malar rash, polyarthritis in bilateral hands, hypocomplementemia, and antinuclear antibody positivity. Despite treatment with prednisolone and hydroxychloroquine for two months, she experienced a generalized tonic seizure and exhibited progressive altered mental status, visual and auditory hallucinations, and low-grade fever for several weeks.

Physical examination at admission revealed fever, BP 118/84 mmHg with an inability to incorporate time/place/person, and hepatosplenomegaly. A neurological exam showed no cranial nerve involvement, normal muscle tone, and normal deep tendon reflexes. Other evaluations were unremarkable. Laboratory tests revealed the following: erythrocyte sedimentation rate, 86 mm/h (normal range, 0–20 mm/h); C-reactive protein, 8.9 mg/L (normal range, 0–5 mg/L); creatinine, 0.42 mg/dL; and blood urea nitrogen, 10 mg/dL. Additional laboratory tests to evaluate for HLH revealed markedly elevated levels of ferritin (3966 [normal range, 13–150] ng/mL) and fasting triglycerides 850 mg/dL. Complete blood count showed pancytopenia with urinalysis showed significant proteinuria based on a urine protein-creatinine ratio of 3.8. CSF analysis revealed 60/mm^3^ WBCs, 110,000/mm^3^ RBCs, 141 mg/dL glucose (blood–CSF glucose ratio of 0.7), and <4 mg/dL protein with negative gram stain and bacterial culture. Magnetic resonance imaging and magnetic resonance angiography of the brain did not reveal evidence of thromboembolic events or bleeding.

Broad-spectrum antibiotics and acyclovir were initiated. Based on the lack of bacterial growth after 48 h in blood culture, pulse intravenous methylprednisolone (IVMP) (1000 mg; 30 mg/kg/day) for three days was initiated for the treatment of MAS. Bone marrow aspiration biopsy revealed the presence of histiocytes with active hemophagocytic activity, which led to the potential diagnosis of HLH or MAS secondary to active SLE. Despite the initial aggressive treatment with pulse IVMP, the patient had persistent altered mental status, pancytopenia, and hyperbilirubinemia. Therefore, other potential causes of secondary HLH, such as infection and thrombotic microangiopathy, were considered. Renal biopsy revealed class II lupus nephritis without evidence of thrombosis. The CSF analysis for viral encephalitides revealed EBV positivity using polymerase chain reaction. After a multidisciplinary team discussion, the HLH treatment protocol, including intravenous immunoglobulin, corticosteroids, and cyclosporin A, was initiated. After the treatment initiation, the patient did not have fever, seizures, or hallucinations. Her level of consciousness and laboratory parameters gradually improved.

**Conclusion:** The current case illustrates the diagnostic approach for a patient with c-SLE who presented with NP symptom and refractory to the treatment of MAS with pulse IVMP. The need to consider other potential etiologies, the multidisciplinary care team’s approach and prompt management are recommended to achieve maximum benefit for immunosuppressive patients.

**Patient Consent:** Yes, I received consent

**Disclosure of Interest**: None declared

### P319. Juvenile systemic lupus erythematosus manifesting as isolated hemiparesis in a seven year old girl

#### M. Tsinti, V. Dermentzoglou, E. Tsitsami

##### First Department of Pediatrics, University of Athens Medical School, Children’s Hospital Aghia Sofia, Athens, Greece

###### **Correspondence:** M. Tsinti

**Introduction:** Neuropsychiatric manifestations are frequent among JSLE patients but most often correlate high polysystemic disease activity.

**Objectives:** To report a case of diffuse NPSLE manifesting as left hemiparesis and diagnosed on the basis of imaging and CSF findings as well as autoantibody profile.

**Methods:** Case presentation

**Results:** A Seven year old girl initially treated by the neurology department for left hemiparesis predominantly of the arm is presented. Clinical findings were otherwise normal. Seizures were absent. Family history was notable for mother and paternal grandfather diagnosed with multiple sclerosis (MS). Brain MRI showed right lobe hemiatrophy, most prominent in the frontal lobe. Subcortical white matter lesions were detected in both hemispheres. MR perfusion images showed decreased cerebral blood flow in the right frontal lobe suggesting hypoperfusion. The imaging features were not consistent with MS or ischemic stroke but more suggestive of ischemia from vasculitis and vasculopathy. ECG was normal. Metabolic screening, renal and hepatic biochemistry and blood markers of inflammation were within normal limits. Evaluation of CSF revealed normal leukocyte count, protein, glucose and lactic levels, raised neopterins, and the presence of oligoclonal bands both in CSF and serum (type 3), findings compatible with brain inflammation in the context of systemic inflammatory disease. Cytokines suggestive of type I interferonopathy IL8=86 pg/ml {nv<5 } and IFNα=7 pg/ml {<5 } were detected. Autoantibodies for autoimmune encephalitis anti-: NMDAR, AMPA1R , AMPA2R, GABAβ1R, LGI1R, DPPX , CASPR TPO as well as serum anti-NMO IgG and anti-AQP4 were absent. Thyroid function was normal. ANA titer 1/1280, fine speckled with mitotic cells, normal c3 and c4 and IgG 693 mg/dl (nv 812-1698) were detected. Repeat evaluation 3 months later revealed unchanged clinical, CBC, inflammatory markers and MRI findings but raising ANA titer=1/2560 and the presence of multiple anti-Extractable Nuclear Antigens autoantibodies: anti-SM, anti-RNP, anti-SSA/Ro, anti-SSB/La and anti-cardiolipin. Whole Exome Sequencing was unrevealing. Mycophenolate mofetil and HCQ were initiated and physiotherapy continued. Three months later movement was significantly improved, ANA title was diminished (1/640) and anti-ENAs were negative.

**Conclusion:** Isolated hemiparesis was the first manifestation of Diffuse NPSLE. The diagnosis was posed on the basis of MRI, CSF findings suggestive of intrathecal Immunoglobulin synthesis in the context of systemic inflammation and autoantibody profile with very high ANA titers and specific autoantibodies.

**Patient Consent:** Yes, I received consent

**Disclosure of Interest**: None declared

### P320. Distinct biological phenotypes stratify patients in groups with similar disease activity states; a multi-omic approach in childhood-onset SLE

#### M. J. Wahadat^1,2^, S. J. van Tilburg^1^, Y. M. Mueller^1^, H. J. de Wit^1^, C. G. van Helden-Meeuwsen^1^, A. W. Langerak^1^, M. Gruijters^2^, A. Mubarak^2^, M. Verkaaik^2^, P. D. Katsikis^1^, S. Kamphuis^2^, M. A. Versnel^1^

##### ^1^Department of Immunology, Erasmus MC, University Medical Center Rotterdam, ^2^Department of Paediatric Rheumatology, Erasmus MC-Sophia Children’s hospital, University Medical Center Rotterdam, Rotterdam, Netherlands

###### **Correspondence:** M. J. Wahadat

**Introduction:** Childhood-onset systemic lupus erythematosus (cSLE) is a devastating relapsing remitting autoimmune disease characterized by significant heterogeneity between patients which hinders diagnosis, management and treatment choices. Patients with distinct clinical disease phenotypes may respond to the same medication and vice versa, underlining that solely using clinical phenotype to decide which treatment to start or taper is not enough. Therefore, defining tools that can stratify patients into groups paralleling disease severity can facilitate personalized treatment strategies.

**Objectives:** To stratify patients into biological phenotypes via unsupervised hierarchical clustering of transcriptomic and proteomic data and study the immunological cellular landscape that characterizes these clusters.

**Methods:** Gene expression and cytokine profiles were determined in childhood-onset SLE patients, pre-selected based on three disease activity states, namely at diagnosis, Low Lupus Disease Activity state (LLDAS) and flare. We used an unsupervised hierarchical clustering method, agnostic to disease severity, to identify clusters with distinct biological phenotypes. Disease activity was scored by the clinical SELENA-SLEDAI. Forty-color flow cytometry was used to identify immune cell subsets associated with the identified clusters.

**Results:** This approach identified three unique clusters, each characterized by a set of differentially expressed genes and cytokines. When specifying the clinical phenotypes, the clusters were remarkably unique: in cluster 1 patients were mainly in LLDAS, cluster 2 contained mainly patients at diagnosis, and cluster 3 contained a mixed group of patients with disease flares, patients at diagnosis and patients in LLDAS. Remarkably, healthy controls clustered together with cSLE patients in cluster 1. Interestingly, the biological phenotypes did not reflect previous organ involvement. Lastly, some patients moved from one cluster to another over time, reflecting change of disease activity states. High-dimensional spectral flow cytometry identified specific immune cell subsets that differed between the clusters, including CD11c+B cells, conventional dendritic cells, plasmablasts and early effector CD4+T cells.

**Conclusion:** This study shows that combining transcriptional and proteomic data is a reliable method to cluster patients into distinct biological phenotypes that are related to disease activity state but not to organ involvement. This may lead to a new concept where treatment choices are not based solely on organ involvement but also on measuring novel biological parameters.

**Patient Consent:** Yes, I received consent

**Disclosure of Interest**: None declared

### P321. DDX58 and MX1 gene expression discriminates jsle from healthy children and children with other inflammatory or infectious diseases

#### K. Webb^1,2^, T. Spracklen^3^, C. Butters^1^, H. Facey Thomas^1^, R. Stander^1^, M. Erasmus^4^, J. Day^1^, T. Scriba^4^, C. Scott^1^, S. Mendelsohn^4^

##### ^1^Paediatric Rheumatology, University of Cape Town, Cape Town, South Africa, ^2^Crick African Network, Francis Crick Institute, London, United Kingdom, ^3^Cape Heart Institute, University of Cape Town, ^4^South African Tuberculosis Vaccine Initiative, Institute of Infectious Disease and Molecular Medicine, Cape Town, South Africa

###### **Correspondence:** K. Webb

**Introduction:** Juvenile onset systemic lupus erythematosus (jSLE) is defined by a type 1 interferon (IFN) gene signature. It is not known if this signature is present in the pre-clinical phase of jSLE or whether it may act as an identifiable risk factor for the development of jSLE. There is no simple type 1 IFN assay validated for use as a simple, affordable research tool in large cohort studies to identify jSLE risk.

**Objectives:** This study sought to identify individual type 1 IFN candidate genes whose expression might discriminate children with jSLE from healthy children and those with other inflammatory disorders.

**Methods:** Children were recruited with consent, these included: children with jSLE, healthy children and children with other inflammatory disorders. RNA was extracted from whole blood and the expression of 10 type 1 IFN inducible genes was determined by multiplex Fluidigm real-time quantitative PCR (*DDX58, IRF7, IRF8, IFI6, IFNAR, MX1, MX2, OAS1, IFIT6, ISG15*). Differentially expressed genes (DEGs) were identified through nonparametric pairwise comparisons between groups, adjusted by Bonferroni correction. Receiver operating characteristic (ROC) analysis was used to investigate the discriminatory capacity of each gene.

**Results:** The cohort included multiple samples from 7 children with jSLE, 53 healthy children and 62 children with inflammatory diseases (33 MIS-C, 19 infections, 10 Kawasaki disease). When jSLE was compared to all controls, after correction for multiple testing, DDX*58* (p=0.01) and *MX1* (p=0.03) were differentially expressed. *DDX58* and *MX1* discriminated children with jSLE from healthy children well: *DDX58* area under the curve (AUC)= 0.846 (p=0.001) and *MX1* AUC=0.774 (p=0.009). Out of all genes, *DDX58* and *MX1* also best discriminated jSLE from the whole mixed group of inflammatory controls: *DDX58* AUC =0.810 (p=0.02) and *MX1* AUC=0.798 (p=0.03). When MIS-C and Kawasaki disease were excluded, both genes still discriminated jSLE from the infective controls: *DDX58* AUC=0.800 (p=0.01) and *MX1* AUC=0.850 (p=0.003).

**Conclusion:** In this small, preliminary cohort, *DDX58* and *MX1* were identified as potential genes which individually discriminate jSLE from healthy children, children with inflammatory diseases and children with infectious diseases. Further research is required to confirm this observation. A single PCR gene expression test might provide an easy, affordable measure of lupus-specific type 1 IFN expression as a tool in future studies to better understand the role of type 1 IFN in the pre-clinical phase of jSLE and identifying lupus risk.

**Patient Consent:** Yes, I received consent

**Disclosure of Interest**: None declared

## Poster session: New diseases

### P322. PIMS through the waves of COVID 19: data from the JIR cohort

#### R. Kechiche^1^, A. Schwartz^1^, F. Bajolle^2^, S. Poignant^3^, R. Basmaci^4^, C. Pajot^5^, U. Meinzer^6^, L. Morin^7^, V. Lambert^8^, P. Dusser^1^, N. Matsa^1^, M. Hofer^9^, I. Kone-Paut^1^, C. Galeotti^1^ on behalf of French Covid-19 Paediatric Inflammation Consortium

##### ^1^Paediatric rheumatology, CHU Bicêtre, Kremlin-Bicêtre, ^2^Paediatric cardiology, CHU Necker, Paris, ^3^Paediatrics, CHU Nantes, Nantes, ^4^Paediatrics, CHU Louis Mourier, Colombes, ^5^Paediatrics, CHU Toulouse, Toulouse, ^6^Paediatrics, CHU Robert Debré, Paris, ^7^Paediatric intensive care unit, ^8^Paediatric radiology, CHU Bicêtre, Kremlin-Bicêtre, France, ^9^Paediatric rheumatology, CHUV, Lausanne, Switzerland

###### **Correspondence:** A. Schvartz

**Introduction:** Paediatric inflammatory multisystem Syndrome (PIMS) is a new systemic inflammatory disease linked to SARS-CoV2 that affects children. It was first reported in may 2020.

**Objectives:** The objectives of this study were to describe patients with PIMS through the international JIR cohort registry and to compare the different profiles and treatments of these patients over the different waves.

**Methods:** Study patients with international PIMS criteria were included from March 2020 to June 2021. Patients were identified in the JIR cohort, an international registry collecting demographic, clinical and paraclinical data on patients with pediatric inflammatory diseases. Two groups were distinguished: from March 2020 to July 2020 for patients in the first wave, from July 2020 to June 2021 for patients in the 2nd and 3rd waves. These two groups were compared using a Fischer test for categorical data and a Mann-Whitney test for quantitative data.

**Results:** 136 patients meeting the PIMS criteria were included (64 patients in the 1st wave, 72 patients after). Patients had less frequent myocarditis (51 patients in wave 1 vs. 36 patients after, p=0,0003) and respiratory distress (34 patients vs 10 patients, p<0,0001). Corticosteroids were used more frequently in the second wave (32 patients in wave 1 vs. 67 patients after July 2020, p<0,0001). Intravenous immunoglobulins were used as much over the waves (58 patients in wave 1 vs 68 patients after, p=0.5). Antibiotics were less used since the second wave (53 patients received antibiotics before July 2020 vs 11 after, p<0,0001). The duration of hospitalization decreased significantly (p<0,0001) with a median duration of 9 days during the first wave (interquartile range, 7-12) and 7 days (interquartile range, 5-10) after the first wave.

**Conclusion:** There was a decrease in the number of complications of PIMS, particularly cardiac and respiratory complications, and a decrease in the length of hospitalization over time. The treatment of PIMS has also evolved, with a clear increase in the use of corticosteroids and a decrease in the use of antibiotics.

**Disclosure of Interest**: None declared

### P323. Pediatric IGG4-related disease in India - an experience of four children from a single pediatric rheumatology center

#### D. B. Pandya, M. Aggarwal, S. Sawhney

##### ^1^Pediatric & Adolescent Rheumatology Department, Sir Gangaram Hospital , New Delhi , India

###### **Correspondence:** D. B. Pandya

**Introduction:** IgG4-related disease (IgG4-RD) was first described in year 2003 as an inflammatory syndrome of unknown etiology characterized by tumorous swelling of the organs such as salivary glands, orbit, pancreas, biliary tree, retroperitoneum, lacrimal gland, kidney and thyroid. ^1^

In terms of ocular involvement, it usually presents with ocular adnexal masses which can involve the orbit, extra ocular muscles, lacrimal system, optic nerve, or sclera. ^2^ Unilateral or bilateral eyelid or periorbital swelling is the most common disease manifestation. ^3^

The three major histopathological features associated with IgG4-RD are, dense lymphoplasmacytic infiltrate, storiform fibrosis and obliterative phlebitis. Other supportive finding includes elevated IgG level more than 135 mg/dl, histopathological evidence of >10 IgG4+ cells/hpf and elevated IgG4-to-IgG ratio of>40 % is commonly accepted. ^4, 5^

Glucocorticoids are the first line of therapy. Azathioprine, methotrexate or mycophenolate mofetil have been used with limited success in patients who are resistant to or dependent on glucocorticoids. ^6^ Patients resistant to glucocorticoids may benefit with the use of rituximab.

Children with IgG4 related diseases are not yet well described in literature.

**Objectives:** We aim to reveal spectrum of IgG4-related diseases in four Indian Children at Our Center.

**Methods:** This is a retrospective analysis of four children who were diagnosed with IgG4-related disease between January 2013 to January 2022 at our center. Our collected data included demographics , clinical presentation , management and follow up details.

**Results:** This Table shows Characteristics of four Indian children with IgG4-related disease at our PRC

All four patients are in clinical remission as per their last follow up.

**Conclusion:** Eye involvement remains the most common manifestation in pediatric IgG-4 related disease in our cohort. MRI Orbit is an investigation of choice in such patients. One patient had renal involvement. All patient had elevated IgG4 level. All patients had transient improvement after steroids. Methotrexate was not effective in controlling inflammation in any of our patients. MMF and azathioprine was found to be effective in our cohort.

**Trial registration identifying number:** 1. Stone JH, Zen Y, Deshpande V. IgG4-related disease. N Engl J Med. 2012; 366:539.

2. Wallace ZS, Khosroshahi A, Jakobiec FA, Deshpande V, Hatton MP, Ritter J, et al. IgG4-related systemic disease as a cause of “idiopathic” orbital inflammation, including orbital myositis, and trigeminal nerve involvement. Surd Ophthalmic. 2011;57:26.

3. Cheuk W, Chan JK. IgG4-related sclerosing disease: a critical appraisal of an evolving clinicopathologic entity. Adv Anat Pathol. 2010; 17:303.

4. Plaza JA, Garrity JA, Dogan A, Ananthamurthy A, Witzig TE, Salomão DR. Orbital inflammation with IgG4-positive plasma cells: manifestation of IgG4 systemic disease. Arch Ophthalmic. 2011; 129:421–8.

5. Umehara H, Okazaki K, Masaki Y, Kawano M, Yamamoto M, SaekiT, et al. Comprehensive diagnostic criteriafor IgG4-related disease (IgG4-RD), 2011. Mod Rheumatol. 2012; 22:21–30.

6. Kawano M, Yamada K. Treatment of IgG4-related disease. Curr Immunol Rev. 2011; 7:

**Patient Consent:** Yes, I received consent

**Disclosure of Interest**: None declared

**Table 1 (abstract P323).** See text for description

Mean Age at Onset	8.5 years (2-17years)
Sex Ratio M:F	1:1
Eye Involvement	3 / 4 (75%)
Recurrent scleritis & lacrimal gland involvement	1
B/l orbital inflammation (MRI evidence)	2
Tumour-like mass – mediastinal mass	1 / 4 (25%)
Renal (tubulointerstitial nephritis)	1 / 4 ( 25%)
Respiratory System ( Chronic Cough)	1 / 4 (25%)
Elevated IgG4 level > 200 mg / dl	4 / 4 (100%)
Supportive Histopathological evidence	3 / 4 (75%)
Response to steroids	4 /4 (100%)
Flare on stopping steroids	4 / 4 (100%)
Response to Methotrexate	0 / 2 (0%)
Response to MMF	2 / 3 (75%)
Response to Azathioprine	1 / 2 (50%)

## Poster session: Scleroderma and related syndromes

### P324. Factors affecting survival in juvenile versus adult onset systemic sclerosis

#### A. Adrovic^1^, G. Karatemiz^2^, S. Sahin^3^, M. Yildiz^3^, F. Haslak^3^, A. Gunalp^3^, K. Barut^3^, G. Hatemi^4^, V. Hamuryudan^4^, O. Kasapcopur^3^, E. Seyahi^4^

##### ^1^Pediatric Rheumatology, Koç University Hospital, ^2^Rheumatology, Istanbul Sisli Hamidiye Etfal Training and Research Hospital, ^3^Pediatric Rheumatology, ^4^Rheumatology, Istanbul University-Cerrahpasa, Cerrahpasa Medical School, Istanbul, Turkey

###### **Correspondence:** A. Adrovic

**Introduction:** Systemic sclerosis (SSc) is a rare autoimmune disease characterized by progressive fibrosis of the skin, internal organs involvement and vasculopathy, which could lead to significant morbidity and mortality. Previous studies comparing adult (aSSc) and juvenile (jSSc) patients reported the lower frequency of vital organ involvement and better disease outcome in jSSc.

**Objectives:** We aimed to compare survival and factors affecting mortality in jSSc and aSSc patients.

**Methods:** Demographic characteristics, clinical features, autoantibody profiles, and treatment data were retrieved from the databases. The data presented were the cumulative clinical and serological manifestations throughout the follow up period. The chi-square test was used to compare categorical variables between groups. Univariate and multivariate logistic regression analyses were performed to the independent factors of mortality. The Kaplan-Meier method was used for survival analysis.

**Results:** A total of 214 patients were included: 156 (72.8%) adults and 58 (27.1%) juvenile. The mean age at study was 52.3±15.7 vs. 16.3±4.4 years, at the disease onset 37±14.7 vs. 8.8±4.1 years and at diagnosis 42±15.2 vs. 10.4±3.8 years for adult and juvenile onset patients, respectively. The mean follow-up duration was 6.3±4.9 years for adult vs. 6.6±4.9 years for juvenile patients (p>0.05). The mortality was higher in adults with death seen in 24 (15.9%) adults comparing to 1 (3.33%) of juvenile patients (p=0.005).

The frequency of interstitial lung disease and systemic hypertension was significantly higher among aSSc patients: 77 (50.9%) vs. 9 (30%) (p<0.001) and 27 (17.9%) vs. 0(0%) (p=0.009), respectively. The cardiac and renal involvement was more common among aSSc while musculoskeletal features were more frequent in jSSc, but with insignificant difference. The interstitial lung involvement (p=0.03) and cardiac insufficiency (p=0.05), represent independent risk factors of mortality in patients with SSc.

**Conclusion:** The survival rate is better in jSSc patients comparing to adults. Vital organ involvement (lung, heart) represent main factors of mortality. Prospective studies with longer follow-up and higher patients’ number are required to confirm our findings.

**Patient Consent:** Yes, I received consent

**Disclosure of Interest**: None declared

### P325. Profound hypocalcaemia following pamidronate administration in a child with Systemic Sclerosis (SSC) background

#### E. Sefi^1^, S. Lacassagne^2^, M. Al Obaidi^3^

##### ^1^General Paediatric Department, The Whittington Hospital, ^2^Paediatric Rheumatology, Great ormond Street Hospital, ^3^Paediatric Rheumatology, Great Ormond Street Hospital For Children, London, United Kingdom

###### **Correspondence:** M. Al Obaidi

**Introduction:** We present the case of a 15 year old boy with severe hypocalcaemia following pamidronate administration for treatment of SSC related calcinosis.

**Objectives:** To highlight the potential challenges facing the general paediatricians in managing children and young people with rare diseases and the side effect of medications they are not familiar with.

**Methods:** A 15 year old boy of Afro-Caribbean origin with SSC was diagnosed at the age of eleven. He presented with rash, myositis, arthritis, digital ulcers with tissue loss. At the time of diagnosis he was vitamin D deficient and was treated with vitamin D.

He initially had a good partial response to Methotrexate then Tocilizumab was added. On both medication, he remained in remission for months but went on to develop rapidly progressive calcinosis involving his wrists and fingers with local superinfection. At that time he was being treated with tocilizumab infusions, Methotrexate, amlodipine, hydroxycholoroquine and prednisolone.

**Results:** He was treated acutely with IV antibiotics and a decision was made to give intravenous pamidronate. Prior to treatment his calcium level was normal at 2.23 mmol/L, phosphate 1.65mmol/L and alkaline phosphatase was normal at 301 iu/L and his vitamin D level was not available. One week following the infusion a routine blood test at the local district hospital where he receives shared care revealed a critically low calcium level of 1.44 mmol/L. He was called back from home for emergency treatment of this. He described feeling tired but was otherwise asymptomatic. He was admitted and monitored. Despite intravenous calcium infusions, his calcium level remained low. Subsequently his vitamin D level was found to be low at 18 nmol/L (normal >50 nmol/L) with parathyroid hormone level of 41.6 omol/L. Following vitamin D administration alongside calcium supplementation his calcium levels normalised over the course of a week.

**Conclusion:** Discussion

Pamidronate is a useful treatment of calcinosis but as we have described it can cause profound hypocalcaemia especially in the presence of pre-existing vitamin D deficiency. Pamidronate is not a common medication used in district general hospitals but it is important that paediatricians are aware of its potential side effects as these children are likely to present to their local hospitals for emergency treatment.

**Patient Consent:** Yes, I received consent

**Disclosure of Interest**: None declared

### P326. Pediatric mixed connective tissue disease versus other overlap syndromes: a multicenter study

#### E. D. Batu^1^, S. Sahin^2^, A. Gunalp^2^, S. Ozdel^3^, Z. Kizildag^4^, A. Pac Kisaarslan^5^, I. Bagrul^3^, M. Kasap Cuceoglu^1^, A. Tanatar^6^, H. E. Sonmez^7^, E. Sag^8^, S. Demir^9^, E. Celikel^10^, S. Caglayan^11^, B. Celikel Acar^10^, B. Sozeri^11^, N. Aktay Ayaz^6^, Y. Bilginer^1^, M. H. Poyrazoglu^5^, E. Unsal^4^, O. Kasapcopur^2^, S. Ozen^1^

##### ^1^Hacettepe University Faculty of Medicine, Ankara, ^2^Istanbul University, Cerrahpasa Faculty of Medicine, Istanbul, ^3^Dr. Sami Ulus Maternity and Child Health and Diseases Research and Training Hospital, Ankara, ^4^Dokuz Eylul University Faculty of Medicine, Izmir, ^5^Erciyes University Faculty of Medicine, Kayseri, ^6^Istanbul University Faculty of Medicine, Istanbul, ^7^Kocaeli University Faculty of Medicine, Kocaeli, ^8^Ankara Research and Training Hospital, Ankara, ^9^Erzurum Regional Research and Training Hospital, Erzurum, ^10^Ankara City Hospital, Ankara, ^11^Umraniye Research and Training Hospital, Istanbul, Turkey

###### **Correspondence:** E. D. Batu

**Introduction:** Overlap autoimmune syndromes are very rare in childhood. Pediatric mixed connective tissue disease (MCTD) is a specific subgroup of overlap syndromes associated with anti-U1-RNP antibodies.

**Objectives:** We aimed to analyze and compare the characteristics of patients with pediatric MCTD and other overlap syndromes.

**Methods:** We included all patients with MCTD and other overlap syndromes who had the disease onset before 18 years of age. All MCTD patients met either Kasukawa or Alarcon-Segovia criteria. The patients with other overlap syndromes had the features of ≥2 different systemic autoimmune diseases and did not meet MCTD diagnostic criteria.

**Results:** A total of 30 MCTD (F/M=14/1) and 30 patients (F/M=29/1) with other overlap syndromes were included from 11 centers. The median ages at disease onset were similar between two groups while the median age at diagnosis was slightly younger in the overlap group than MCTD group (11 vs. 11.4 years and 11.8 vs. 13.3. years, respectively). The duration of follow-up was 40.5 months in the whole group. The most prominent phenotype at disease onset and the last visit was SLE in the MCTD group and JIA in the overlap group. Systemic scleroderma phenotype at the last visit was more frequent among MCTD patients compared to overlap patients (60% vs. 33.3%; p=0.038). Weight loss (36.7% vs. 13.3%), digital ulcers (20% vs. 0), puffy hands (60% vs. 20%), Raynaud phenomenon (86.7% vs. 46.7%), hematologic involvement (79% vs. 26.7%), and anti-Sm (29% vs. 3.3%) were more common among MCTD patients than overlap patients (p<0.05 for all). Gottron papules, on the other hand, were more frequently observed in overlap patients compared to MCTD patients (40% vs. 16.7%; p=0.045). Renal involvement (30% vs. 10%) and elevated erythrocyte sedimentation rate during active disease (80% vs. 56.7) were more frequent in MCTD than overlap patients, but these differences were not statistically significant.

Regarding treatment, around 90% and 85% of patients received hydroxychloroquine and corticosteroids, respectively in both groups. The duration of corticosteroid treatment was longer in MCTD patients than overlap patients (52.5 vs. 13.5 months; p=0.059). NSAID, mycophenolate mofetil, rituximab, IVIG, ACE inhibitors, calcium channel blockers, and aspirin were more frequently prescribed to MCTD patients while methotrexate was more commonly used by overlap patients. None of the patients died. And none achieved complete remission off drugs. A higher percentage of overlap patients achieved complete remission on drugs than MCTD patients (51.7% vs. 23.3.%; p=0.047).

**Conclusion:** Although considered in the same group of diseases, both disease phenotype and outcome differs between pediatric MCTD patients and children with other overlap syndromes. Analyzing and addressing the differentiating features of these entities could pave the way for early diagnosis and effective treatment in these group of patients during childhood.

**Patient Consent:** Yes, I received consent

**Disclosure of Interest**: None declared

### P327. pediatric sclerosis in a center of south of Spain

#### A. Capilla Miranda, L. Fernandez Silveira, O. Neth, C. García Malagón, M. Camacho Lovillo

##### ^1^Servicio Inmunología, Reumatología e Infectología pediatrica, Hospital Universitario Virgen del Rocío, Sevilla, Spain

###### **Correspondence:** M. Camacho Lovillo

**Introduction:** Pediatric sclerodermia includes two groups of clinical entities, systemic sclerosis (SS) and localized scleroderma (LS).

**Objectives:** To analyze the pattern of disease expression, laboratory data, treatments used and outcome in pediatric patients diagnosed with SS and LS, followed in a tertiary care Hospital in Spain.

**Methods:** Medical charts of all pediatric patients younger than 16 years old, diagnosed with SS and LS between 1997 and 2021 and followed at the pediatric rheumatology clinic in our hospital were retrospectively reviewed.

**Results:** We describe a cohort of 9 patients, 5 with SS and 4 with LS.

Regarding SS patients, the mean age at disease onset was 11 ± 2.55 years (mean ± 1SD, range 7-13 years), all were female. Sclerodactyly and Raynaud’s phenomenon were the most prevalent clinical feature (5/5). Other sings of skin involvement included edema in 4/5, telangiectasia in 3/5, calcinosis, livedo and digital ulcer in 2 /5 patients each. Nailfold capillary changes were reported in 3/5 patients at the onset of the disease and in 5/5 during the overall disease course. Musculoskeletal symptoms, such as arthralgia (3/5), arthritis (3/5) and reduced muscle strength (1/5) were present in most children. One of our patients presented an overlap syndrome with a muscle biopsy compatible with dermatomyositis and increased serum muscle enzymes. Gastrointestinal or respiratory self-reported symptoms were exceptional, only one patient suffered from dysphagia. However, 2 patients had respiratory system involvement with HRCT or reduced FVC and in 3 patients, although mild, manometry showed esophageal dysmotility sings. None of our patients presented neurologic manifestations, cardiac or renal involvement. Antinuclear antibodies were present in all patients. Anti-topoisomerase I (Scl-70) was found positive in 1 patient, anti-ENA (unable to specify type) was detected in 1 patient and anti-Ro was found in 2 patients. One patient had positive RF. Antiphospholipid antibodies were negative in all patients. None of these patients had abnormal laboratory parameters included blood count, C-reactive protein or erythrocyte sedimentation rate. Metotrexate was selected as first line treatment in most of them (4/5), subsequently changed to mycophenolate in 2 due to intolerance and poor response. Three patients received systemic corticosteroid, cyclophosphamide in 1, tocilizumab in 1. Two patients received oral vasodilators and 4 received topical vasodilators (diltiazem) with good response. At the end of the study, 4/5 patients still required treatment, 2 of them with moderate activity of disease in spite of this.

Regarding LS patients, 3 were linear morphea and 1 was plaque morphea. 3/4 presented proximal skin induration and s2/4 had sclerodactyly, one patient showed arthritis and none of them had Raynaud’s phenomenon. Antinuclear antibodies were present in two patients. Two patients presented mild alterations in esophageal micromanometry, without involvement of other systems. All patients had favorable evolution with methotrexate, but in two patients it was changed to mycophenolate and cyclosporine due to poor tolerance. One patient was treated with oral corticosteroid.

**Conclusion:** Clinical and laboratory characteristics of our patients were similar to previous published series. Respiratory system involvement is common in SS and is usually asymptomatic in the early stages. Abnormalities in esophageal manometry were common even in children without self-reported symptoms. It can be difficult to define the pathological significance and we need more experience.

Nailfold capillaroscopy is an noninvasive and simple test that can help in the diagnosis and follow up of the disease.

**Patient Consent:** Yes, I received consent

**Disclosure of Interest**: None declared

### P328. Diffuse juvenile systemic sclerosis patients show distinct organ involvement and have more severe disease in the largest JSSC cohort of the world. results from the the juvenile scleroderma inception cohort

#### I. Foeldvari^1^, J. Klotsche^2^, O. Kasapcopur^3^, A. Adrovic^3^, K. Torok^3^, M. T. Terreri^3^, A. P. Sakamoto^3^, B. Feldman^3^, F. Sztajnbok^3^, V. Stanevicha^3^, J. Anton^3^, S. Johnson^3^, R. Khubchandani^3^, E. Alexeeva^3^, M. Katsicas ^3^, S. Sawhney^3^, V. Smith^3^, S. Appenzeller^3^, T. Avcin ^3^, M. Kostik^3^, T. Lehman^3^, H. Malcova^3^, E. Marrani^3^, C. Pain^3^, D. Schonenberg-Meinema^3^, W.-A. Sifuentes-Giraldo^3^, N. Vasquez-Canizares^3^, P. Costa Reis^3^, M. Janarthanan ^3^, M. Moll^3^, D. Nemcova ^3^, A. Patwardhan^3^, M. J. Santos^3^, S. Abu Al-Saoud^3^, C. Battagliotti^3^, L. Berntson^3^, B. Bica^3^, J. Brunner^3^, R. Cimaz^3^, D. Eleftheriou^3^, L. Harel^3^, G. Horneff^3^, D. Kaiser^3^, T. Kallinich^3^, D. Lazarevic^3^, K. Minden^3^, S. Nielsen^3^, F. Nuruzzaman^3^, S. Opsahl Hetlevik^3^, Y. Uziel ^3^, N. Helmus^1^

##### ^1^Hamburg Centre for Pediatric and Adolescence Rheumatology, Hamburg, ^2^German Rheumatism Research Center, Berlin, ^3^jSSc collaborative group, Hamburg, Germany

###### **Correspondence:** I. Foeldvari

**Introduction:** Juvenile systemic sclerosis (jSSc) is an orphan disease with a prevalence of 3 in 1 000 000 children. In adult patients there are significant differences between the clinical presentation of diffuse and limited subtypes. We reviewed clinical differences in presentation of subtypes in patients in the juvenile systemic scleroderma inception cohort up to 2021.

**Objectives:** To study the clinical presentation of jSSc patients with diffuse (djSSc) and limited (ljSSc) subtypes.

**Methods:** We reviewed the clinical baseline characteristics of the patients, who were recruited to the juvenile scleroderma inception cohort (jSScC) till 1^st^ of December 2021. jSScC is a prospective cohort of jSSc patients, who developed the first non-Raynaud´s symptom before the age of 16 years and are under the age of 18 years at the time of inclusion.

**Results:** 210 patients with jSSc were included in the cohort, 71% (n=162) had diffuse subtype. The median age at onset of Raynaud phenomenon was 10.4 years (7.3–12.9) and the median age at the first non-Raynaud symptom was 10.9 years (7.4–13.2). Median disease duration was 2.5 years (1–4.4) at the time of inclusion. The female/male ratio was significantly lower in the djSSc subtype (3.7:1 vs 5:1, p<0.001). Antibody profile was quite similar, with the exception of a significantly higher number of anticentromere positive patients in the ljSSc (12% vs 2%, p=0.013). Decreased FVC<80% was found in approximately 30% and decreased DLCO<80% was found in around 40% in both subtypes. Pulmonary hypertension assessed by ultrasound was identified in 5% in both groups. Patients with diffuse subtype had significantly higher modified Rodnan Skin Score (mRSS)(16 vs 4.5, p<0.001), sclerodactyly (84% vs 60%, p<0.001), history of digital ulceration (62% vs 31%, p<0.001), decreased Body Mass Index (BMI) ≤-2 z score (20% vs 4%, p=0.003) and decreased joint range of motion (64% vs 46%, p=0.019). Patients with ljSSc had significantly higher rate of cardiac involvement (13% vs 2%, p=0.001).

Regarding patient related outcomes djSSc patients had more severe disease, looking at patient reported global disease activity (VAS 0–100)(40 vs 25, p=0.039), patient reported global disease damage (VAS 0–100)(40 vs 25, p=0.021) and patient reported assessment of ulceration activity (10 vs 0, p=0.044). Regarding physician related outcomes the physician reported global disease activity (VAS 0–100)(32 vs 20, p<0.001) and physician reported global disease damage (VAS 0–100)(30 vs 15, p=0.014) was significantly higher in djSSc.

**Conclusion:** In this jSSc cohort, the largest in the world, djSSc patients have a significantly more severe disease than ljSSc patients. Interestingly, we found no differences regarding interstitial lung disease and pulmonary hypertension.

This project was supported by an unrestricted grant from “Joachim Herz Stiftung”

**Patient Consent:** Not applicable (there are no patient data)

**Disclosure of Interest**: None declared

### P329. Juvenile systemic sclerosis treatment practices in an international cohort and comparison to recent share consensus guidelines

#### I. Foeldvari^1^, J. Klotsche^2^, O. Kasapcopur^3^, A. Adrovic^3^, K. Torok^3^, M. T. Terreri^3^, A. P. Sakamoto^3^, B. Feldman^3^, J. Anton^3^, F. Sztajnbok^3^, V. Stanevicha^3^, S. Appenzeller^3^, T. Avcin ^3^, S. Johnson^3^, R. Khubchandani^3^, M. Kostik^3^, E. Marrani^3^, W.-A. Sifuentes-Giraldo^3^, D. Nemcova ^3^, M. J. Santos^3^, D. Schonenberg-Meinema^3^, C. Battagliotti^3^, L. Berntson^3^, B. Bica^3^, J. Brunner^3^, R. Cimaz^3^, D. Eleftheriou^3^, L. Harel^3^, G. Horneff^3^, M. Janarthanan ^3^, T. Kallinich^3^, T. Lehman^3^, M. Moll^3^, F. Nuruzzaman^3^, A. Patwardhan^3^, V. Smith^3^, N. Helmus^1^

##### ^1^Hamburg Centre for Pediatric and Adolescence Rheumatology, Hamburg, ^2^German Rheumatism Research Center, Berlin, ^3^jSSc collaborative group, Hamburg, Germany

###### **Correspondence:** I. Foeldvari

**Introduction:** Juvenile systemic scleroderma (jSSc) is an orphan disease with a prevalence of 3 in 1,000,000 children. Currently no medications are licensed for the treatment of jSSc. Due to its rarity, only recently have the first management and treatment guidelines been published, the jSSc SHARE (Single Hub and Access point for paediatric Rheumatology in Europe) recommendations, reflecting consensus opinion upon pediatric rheumatologists.

**Objectives:** To better understand treatment practices internationally for jSSc, both at baseline and over 24 months observation period and to compare if real world therapies are congruent with the recent SHARE recommendations.

**Methods:** The juvenile systemic sclerosis inceptions cohort (jSScC) is a multinational cohort that prospectively collects clinical data, including medications at baseline and subsequent visits. The jSScC enrollment criteria include age of onset of the first non-Raynaud symptom younger than 16 years and age younger than 18 years at cohort entrance. The frequency of medications was calculated across the cohort at timepoint 0, 12 months and 24 months.

**Results:** We extracted data from the jSScC of patients who were followed for 12 or 24 months. 109 patients were followed at time point 0 and 12 months, and data was available for 77 of them up at 24 months. The mean age of the patients was 13.2 years at the timepoint 0. 75% were female and 75% had diffuse subtype. Disease duration at baseline visit was 3.1 years. The medications the patients were on recorded by the physician were captured at T0, T12 and T24 listed in Table 1.

**Conclusion:** At baseline half of the patients were on corticosteroids. This is more frequent than typical adult SSc practice but coincides with jSSc SHARE treatment recommendations (#1). After 12 months in the cohort over 90% of patients received a DMARD therapy. Methotrexate and mycophenolate mofetil were the most commonly DMARDs, which also reflects the SHARE recommendations (#2, #3). At 12 months the use of glucocorticoid decreased and the use of bDMARDs increased. In general, biological DMARDs are typically considered in severe or refractory (#7), reflecting the lower percentage compared to csDMARDs. Autologous stem cell transplantation was observed in one patient at 12 months, reflecting an option in jSSc with progressive and refractory disease (#8). Endothelial receptor antagonists, such as bosentan, were used over time in approximately 20% of the patients, reflecting SHARE recommendation #6 for pulmonary hypertension and/or digital tip ulcers. This is the first evaluation looking at clinical medication practice pattern in jSSc, and its comparison to recently published consensus guidelines.

**Disclosure of Interest**: None declared

**Table 1 (abstract P329).** See text for description

MEDICATIONS	Time point 0 N=109	T12 months N=109	T24 months N=77
**Any Medication**	92%	97%	97%
**Vascular medications**	16%	24%	21%
Endothelial receptor antagonist PDE-5-Blocker	5%	8%	9%
**Corticosteroids**	52%	44%	44%
**All csDMARDs :**	81%	93%	92%
csDMARDs monotherapy	61%	66%	60%
csDMARDs combination therapy	17%	15%	14%
Methotrexate	51%	50%	39%
Mycophenolate Mofetil	26%	44%	47%
**All bDMARDs:**	5%	14%	18%
bDMARDs monotherapy	2%	2%	1%
bDMARDs combined with csDMARDs	3%	12%	17%
Tocilizumab	2%	10%	14%
**Autologous Stem cell transplantation**	0%	1%	0%

### P330. Occurrence of arthritis is in 37% of the patients without overlying skin involvement in juvenile localized scleroderma. summary of the extracutaneous involvement in a monocentric cohort

#### A. Petersen^1^, I. Foeldvari^2^

##### ^1^Asklepios Campus Hamburg of the Semmelweis-Universität, Budapest, Hungary, ^2^Hamburg Centre for Pediatric and Adolescence Rheumatology, Hamburg, Germany

###### **Correspondence:** I. Foeldvari

**Introduction:** Localized scleroderma in childhood(locSSc) occurs with a prevalence of 3.2 to 3.6 per 10 000 children. There are not many publications assessing in detail the extracutaneous manifestations (EM) of locSSc. It is very important to assess the EM too, because the EM can lead to significant damage and morbidity too.

**Objectives:** To assess the occurrence of extracutaneous manifestations in locSSc in our cohort and the correlation of the occurring EM to the subtype of locSSc and the localisation of the skin involvement.

**Methods:** Retrospective chart review of all consecutive patients, who were followed at our centre from January 2000 to July 2020 with the diagnosis of locSSc. The subtype was classified according Laxer et al. The patients were under the age of 18 years at the time point of the first visit. Demographic and clinical data were extracted.

**Results:** 73 patients could be identified, 71% of them were female. Mean age at disease onset was 8 years (4-14 years). The mean time of follow up was 5 years. The subtype distribution was 42 (57%) linear, 24 (33%) mixed, 6 (8%) circumscribed morphea and 1(1%) pansclerotic morphea. 9 (21%) of the 42 patients with linear subtype had coup de sabre and 4 (10%) of them had Parry Romberg. Fifty-six (76%) patients had EM, 40 (53%) of them had 1 form of EM, 10 (13%) of them 2 forms of EM and 6 (8%) patients 3 forms of EM. 53 (73%) of the 76 patients had arthritis. Twenty (37%) of the 53 arthritis involvement occurred on a localisation without overlaying skin involvement. Most frequent localisation of arthritis without overlaying skin involvement was in the hip joints (18%). Of the 53 patients with articular involvement had 31 (58%) linear, 17 (32%) mixed, 4 (7.5) circumscribed morphea and 1 (2%) pansclerotic subtype. 14(19%) of the 73 had length discrepancy of the extremities and 13 (93%) of them had linear subtype. Neurologic symptoms presenting as headache occurred in 8 (11%) patients, 6 (75%) of them had Parry Romberg subtype and 2 (25%) of them coup de sabre. “White” anterior uveitis was screened according to published recommendations and it occurred in 3 patients, only one of them had coup the sabre the other two linear and mixed subtype without involvement of the face.

**Conclusion:** EM is very common and it occurs in 76% of the patients. Thirty seven percent of the articular involvement occurred in joints without overlaying skin involvement, which suggest the importance of the whole body joint count as in juvenile idiopathic arthritis. Only 1 of 3 patients with uveitis had skin involvement in the face, which emphasize the recommended uveitis screening.

**Disclosure of Interest**: None declared

### P331. Clinical characteristics of juvenile onset systemic sclerosis patients from the juvenile scleroderma inception cohort compared to adult age juvenile-onset patients from eustar. are these differences suggesting risk for mortality?

#### I. Foeldvari^1^, J. Klotsche^2^, P. Carreira ^3^, O. Kasapcopur^4^, A. Adrovic^4^, K. Torok^4^, P. Airò^5^, F. Iannone ^6^, Y. Allanore ^7^, A. Balbir-Gurman^8^, T. Schmeiser^9^, F. Sztajnbok^4^, M. T. Terreri^4^, V. Stanevicha^4^, J. Anton^4^, B. Feldman^4^, R. Khubchandani^4^, E. Alexeeva^4^, S. Johnson^4^, M. Katsicas ^4^, S. Sawhney^4^, V. Smith^4^, S. Appenzeller^4^, T. Avcin ^4^, C. Campochiaro^10^, J. de Vries-Bouwstra^11^, M. Kostik^4^, T. Lehman^4^, E. Marrani^4^, D. Schonenberg-Meinema^4^, W.-A. Sifuentes-Giraldo^4^, N. Vasquez-Canizares^4^, M. Janarthanan ^4^, H. Malcova^4^, M. Moll^4^, D. Nemcova ^4^, A. Patwardhan^4^, M. J. Santos^4^, G. Seskute^12^, M.-E. Truchetet^13^, C. Battagliotti^4^, L. Berntson^4^, B. Bica^4^, J. Brunner^4^, R. Cimaz^4^, P. Costa Reis^4^, D. Eleftheriou^4^, L. Harel^4^, G. Horneff^4^, D. Kaiser^4^, T. Kallinich^4^, D. Lazarevic^4^, K. Minden^4^, S. Nielsen^4^, F. Nuruzzaman^4^, S. Opsahl Hetlevik^4^, Y. Uziel ^4^, D. Veale^14^, A.-M. Hoffman-Vold^15^, A. Gabrielli^16^, O. Distler^17^

##### ^1^Hamburg Centre for Pediatric and Adolescence Rheumatology, Hamburg, ^2^German Rheumatism Research Center, Berlin, Germany, ^3^Hospital 12 de Octubre, Madrid, Spain, ^4^jSSc collaborative group, Hamburg, Germany, ^5^Spedali Civili di Brescia, Brescia, ^6^School of Medicine University of Bari, Bari, Italy, ^7^Cochin Hospital, Paris, France, ^8^Rambam Health Care Campus, Haifa, Israel, ^9^Krankenhaus St. Josef, Wuppertal-Elberfeld, Germany, ^10^San Raffaele Hospital, Milan, Italy, ^11^Leiden University Medical Center, Leiden, Netherlands, ^12^State Research Institute for Innovative Medicine, Vilnius, Lithuania, ^13^CHU de Bordeaux, Bordeaux, France, ^14^St. Vincent’s University Hospital, Dublin, Ireland, ^15^Rikshospitalet University Hospital, Oslo, Norway, ^16^Università Politecnica delle Marche & Azienda Ospedali Riuniti, Ancona, Italy, ^17^University Hospital Zürich, Zurich, Switzerland

###### **Correspondence:** I. Foeldvari

**Introduction:** Juvenile systemic sclerosis(jSSc) is an orphan autoimmune disease with a prevalence of 3 in 1 000 000 children. Information on long-term development of organ involvement and clinical characteristics of jSSc patients in adulthood are lacking. It was believed that patients in adult cohorts may represent a survival biased population.

**Objectives:** To assess differences in clinical characteristics of jSSc-onset patients from the pediatric age group, with a mean disease duration of 3 years, compared to the adult age jSSc-onset group, with a mean disease duration of 18.5 years.

**Methods:** We extracted clinical data at time of inclusion into the cohorts from the Juvenile Scleroderma Inception Cohort (jSScC) and data from juvenile-onset adult SSc patients from the European Trials and Research Group (EUSTAR) cohort. We compared the clinical characteristics of the patients by descriptive statistics.

**Results:** We extracted data of 187 jSSc patients from the jSScC and 236 patients from EUSTAR. The mean age at time of assessment was 13.4 years old in the jSScC and 32.4 years old in EUSTAR. The mean disease duration since first non-Raynaud was 3.0 years in jSScC and 18.5 years in the EUSTAR.

We found significant differences between the cohorts. There were more female patients in EUSTAR (88% vs 80%, p=0.04). More patients had diffuse subtype in jSScC (72% vs 40%, p<0.001). The modified Rodnan skin score (mRSS) was significantly higher in jSScC (14.2 vs 12.1, p=0.02). Active digital ulceration occurred more often in EUSTAR (27% vs 18% p=0.01), but history of active ulceration was more frequent in jSScC (54% vs 43%, p<0.001). Mean DLCO was lower in jSScC (75.4 vs 86.3, p<0.001). Intestinal involvement was significantly more common in jSSc (33% vs 24%, p=0.04). Esophageal involvement was more common in EUSTAR (64% vs 34%, p<0.001).

**Conclusion:** Patients with jSSc-onset who are currently adult age (>18 years of age) are less frequently male and from the diffuse subset, have lower mRSS, less digital ulcers and intestinal involvement. This might represent a combination of both survival bias and/or be explained by the longer observation time with less active disease (i.e. natural progression decreased mRSS over time). Further long-term observational studies with jSSc patients are required to address this issue.

**Disclosure of Interest**: None declared

### P332. Patient and physician reported outcomes of juvenile systemic sclerosis patients significantly improve over 12 months observation period in the juvenile systemic scleroderma inception cohort

#### I. Foeldvari^1^, J. Klotsche^2^, O. Kasapcopur^3^, A. Adrovic^3^, K. Torok^3^, M. T. Terreri^3^, B. Feldman^3^, J. Anton^3^, M. Katsicas ^3^, V. Stanevicha^3^, F. Sztajnbok^3^, S. Appenzeller^3^, T. Avcin ^3^, M. Kostik^3^, E. Marrani^3^, W.-A. Sifuentes-Giraldo^3^, S. Johnson^3^, R. Khubchandani^3^, D. Nemcova ^3^, M. J. Santos^3^, C. Battagliotti^3^, L. Berntson^3^, B. Bica^3^, J. Brunner^3^, R. Cimaz^3^, D. Eleftheriou^3^, L. Harel^3^, G. Horneff^3^, M. Janarthanan ^3^, T. Kallinich^3^, K. Minden^3^, M. Moll^3^, S. Nielsen^3^, A. Patwardhan^3^, D. Schonenberg-Meinema^3^, V. Smith^3^, N. Helmus^1^

##### ^1^Hamburg Centre for Pediatric and Adolescence Rheumatology, Hamburg, ^2^German Rheumatism Research Center, Berlin, ^3^jSSc collaborative group, Hamburg, Germany

###### **Correspondence:** I. Foeldvari

**Introduction:** Juvenile systemic sclerosis (jSSc) is an orphan disease with a prevalence of 3 in 1 000 000 children. The Juvenile Systemic Scleroderma Inception cohort (jSScC) is the largest cohort of jSSc patients in the world. The jSScC collects longitudinal data prospectively in jSSc, allowing the evaluation of the development of organ involvement and patients and physician reported outcomes in jSSc over time.

**Objectives:** To review the changes in the clinical characteristics and patient and physician reported outcomes over 12 months observation period from the time of inclusion into the cohort.

**Methods:** The jSScC cohort enrolls jSSc patients who developed the first non-Raynaud´s symptom before the age of 16 years and are under the age of 18 years at the time of inclusion. We reviewed jSScC patient clinical data and patient and physician reported outcomes, who had 12 months follow up from the time of inclusion until 1^st^of December 2021.

**Results:** We could extract data of 113 patients. The female/male ratio was 3.5:1. Median age of onset of Raynaud´s was 10.1 years and the median age of onset of non-Raynaud´s was 10.8 years. Eighty-eight percent of the patients were treated with disease modifying anti-rheumatic drugs (DMARDs) at time of inclusion in the cohort (T0) and 93% after 12 months (T12). Median disease duration was 2.5 years at T0. Antibody profile stayed unchanged. Only 3 clinical parameters changed and improved significantly, the median modified Rodnan skin score improved from 13 to 8 (p=0.002), the number of patients with swollen joints decreased from 17% to 8% (p=0.043) and number of patients with joints with pain on motion decreased from 20% to 12% (p=0.048). All other organ involvement did not show any statistically significant change from T0 to T12.

All collected patient reported outcomes improved significantly from T0 to T12: the patient reported disease activity (VAS 0 – 100) from 40 to 20 (p=0.011), the patient reported disease damage (VAS 0 – 100) from 40 to 20 (p=0.001), patient reported ulceration activity (VAS 0 – 100) from 10 to 0 (p=0.02) and the CHAQ score from 0.3 to 0.1 (p=0.002). Two of the three physician reported outcomes improved significantly, the physician global disease activity (VAS 0 – 100) from 30 to 20 (p=0.011) and physician reported global disease damage (VAS 0 – 100) from 30 to 25 (p=0.028).

**Conclusion:** Skin and musculoskeletal clinical features improved over 12 months, with almost all patients on DMARDs, supporting likely response of these features to therapy. It was promising that internal organ involvement, like cardiac and lung, although potentially stable, did not significantly worsen or increase. The most striking observation in the positive direction is improvement across several patient and physician reported outcome measures over the 12 month time period in this large international cohort.

This project was supported by an unrestricted grant from “Joachim Herz Stiftung”

**Disclosure of Interest**: None declared

### P333. How common is the coexistence of juvenile localized and systemic scleroderma? results of a multination survey

#### I. Foeldvari^1^, N. Helmus^1^, S. Li^2^

##### ^1^Hamburg Centre for Pediatric and Adolescence Rheumatology, Hamburg, Germany, ^2^Hackensack Meridian School of Medicine, Hackensack, United States

###### **Correspondence:** I. Foeldvari

**Introduction:** Pediatric scleroderma consists of two diseases, juvenile localized scleroderma (jLS) and juvenile systemic sclerosis (jSSc). While jLS and jSSc share some disease processes, there are major differences in their clinical features and morbidity patterns. Co-existence of LS and SSc has been reported in adults, with a recent adult systematic review reporting a frequency of 2.4-7.4%, and most patients having SSc prior to development of LS^1^ To explore this issue in pediatric scleroderma, we conducted a survey over the international pediatric rheumatology email board to ask about the co-existence of jLSc and jSSc.

**Objectives:** To explore the coexistence of jLS and JSSc, and the relative timing of their development.

**Methods:** A 12 question was distributed over the worldwide pediatric rheumatology electronic list-serve. Data was collected in an Excel spread sheet and analyzed using Excel software.

**Results:** From approximately 800 survey recipients worldwide, physicians from 28 centers responded (27 pediatric rheumatologists, 1 pediatric dermatologist, half were practicing for over 20 years). Respondents followed a median of 12 jLS and 2 jSSc patients over the past 12 months, and a total of 916 jLS and 193 jSSc patients over the past 60 months. Only 7 of the patients had concurrent jLS and jSSc (0.8% of total jLS, 3.6% of total jSSc patients). Four of the patients developed jSSc following onset of jLS, with interval for 3 patients reported as 6 months, 1 year, or 6 years. Among the other 3 coexisting patients, one simultaneously presented with jLS and jSSc, and the pattern for the other two patients was not reported.

The time between development of jSSc following jLS was 6 months, 1 year, and 6 years, jLS subtype was generalized morphea (2, one as part of mixed morphea), linear of the trunk/limb (2, one as part of mixed morphea), circumscribed superficial morphea, or mixed morphea.

Many of the jLS patients who did not develop jSSc were reported to have features associated with jSSc. Raynaud Phenomenon occurred in 397 patients from 7 centers, for a collective overall frequency of 45%(879 total patients), while nailfoldcapillary changes were reported in 359 patients from 4 centers, for a collective overall frequency of 43% (842 patients). Abnormal pulmonary function or imaging tests were found in 145 from 3 centers, collective overall frequency 24%. Gut involvement including gastroesophageal reflux was found in 534 patients from 9 centers, collective overall frequency 61%.

**Conclusion:** In this international survey cohort of 916 jLS and 193 jSSc patients, the overall frequency of co-existence of these 2 diseases was low, only 0.8% of the jLSc patients, 3.6% of the jSSc patients. However, many jLS patients were reported to jSSc-associated symptoms including Raynaud Phenomenon. More work is needed to evaluate the features associated with co-existence of these two forms of scleroderma in children.

**Disclosure of Interest**: None declared

### P334. Is there a correlation between the distance spanned with six-minute walk test and disease progression in juvenile systemic sclerosis?

#### O. Koker^1,2^, A. Adrovic^2^, M. Yildiz^2^, S. Sahin^2^, A. Gunalp^2^, F. Haslak^2^, K. Barut^2^, O. Kasapcopur^2^

##### ^1^Pediatric Rheumatology, Marmara University-Pendik Training and Research Hospital, ^2^Pediatric Rheumatology, Istanbul University-Cerrahpasa, Cerrahpasa Medical School, Istanbul, Turkey

###### **Correspondence:** O. Koker

**Introduction:** Pulmonary involvement is the most consequential determinant of morbidity and mortality in juvenile systemic sclerosis (jSSC), which is rare but may have a severe progression. The six-minute walking test (6-MWT) has achieved a noteworthy role in the assessment of cardiopulmonary diseases, and improvement in the walking distance is deemed a favourable predictor for the clinical outcome and treatment response. Data concerning the practicality of the test in the management of jSSC is limited.

**Objectives:** The aim of the study is to assess the six-minute walking distance (6MWD), the modification in the distance with disease progression, and oxygen desaturation in patients with jSSC. It is also intended to evaluate the relationship between disease outcome measures and 6MWD.

**Methods:** The study was conducted with patients with a diagnosis of jSSC, who were followed up at Cerrahpasa Medical Faculty, Department of Pediatric Rheumatology at regular intervals. 6MWT was performed twice at 48-month intervals. The test was conducted according to the guidelines recommended by the American Thoracic Society (ATS). The Borg Scale, a well-validated scoring system, was used to determine the patient self-reported fatigue and dyspnea levels.

**Results:** Twelve female patients with a current age of 19,5 (12-23) and diagnosed with jSSC were included in the study. The median age at diagnosis and the disease duration were 12 (8-17) and 112,5 (59-143), respectively. The disease type (limited/ diffuse) was 5/7 (41,7/58,3). Seven patients had Interstitial lung disease (ILD) according to high resolution computed tomography (HRCT). No patients received new cyclophosphamide therapy during this period, but tocilizumab has been added to the treatment of four patients. There were two patients who continued to require steroid treatment. Mycophenolate mofetil treatment has been commenced

**Conclusion:** Although the walking distances of the patients did not show a direct and notable association with disease activity and progression, a significant improvement has been succeeded in the long-term in the follow-up period of the same patients. Factors related to the treatment process, including the use of biologic agents, may influence the test results.

**Patient Consent:** Yes, I received consent

**Disclosure of Interest**: None declared

**Table 1 (abstract P334).** Comparative evaluation of six-minute walk test results in patients with juvenile systemic sclerosis

Tests performed at 48-month intervals	Test results	*p*
6MWD (1st test) (meter)	458 (415-538)	0,04
6MWD (2^nd^ test) (meter)	503 (418-607)	
Borg score of dyspnea (1^st^ test)	2 (1-4)	0,12
Borg score of dyspnea (2^nd^ test)	2 (0,5-4)	
Borg score of fatigue (1^st^ test)	1 (0,5-3)	0,6
Borg score of fatigue (2^nd^ test)	2 (0,5-3)	
Prewalk SpO2 (%)	99 (96-99)	0,18
Postwalk SpO2 (%)	98 (96-99)	
Prewalk heart rate (minute)	86 (69-110)	0,007
Postwalk heart rate (minute)	108 (85-132)	

*jSSC:* juvenile systemic sclerosis, *6MWD:* six-minute walk distance, *SpO2* oxygen saturation by pulse oximetry in four patients. No significant correlation was found between 6MWD and disease activity (J4S) (r=.180 p=0.57) or modified Rodnan skin score (r=.016 p=0.96). Although 6MWD showed a negative correlation with lung involvement, it was significantly not correlated. (r=-.19 p=0.54). The results of 6MWT performed with 48-month intervals are presented in Table 1 comparatively

### P335. Juvenile localized scleroderma: a single center pilot study in the management of children in real clinical practice

#### D. Alekseev, I. Nikishina

##### Pediatric, V. A. Nasonova Research Institute Of Rheumatology, Moskow, Russian Federation

###### **Correspondence:** I. Nikishina

**Introduction:** Juvenile localized scleroderma (JLSc) has a special place among other rheumatic diseases and requires special approaches to assessing the status.

**Objectives:** Development of an approach to stratification of patients (pt) with JLSc based on medical history, clinical manifestations and indices to develop an optimal management approach in real clinical practice.

**Methods:** A study of 100 pt (obtained through a sequential sample from the existing database) was conducted, based on complete anamnesis data, clinical, laboratory and instrumental examinations; the LoSSI and LoSDI indices were additionally calculated.

As a special task, a global assessment of the status was performed, which we use in daily practice, with the empirical selection of the following 4 gradations: (1) remission, (2) chronic course, (3) chronic slowly progressive course, (4) chronic progressive course. Main purpose: determining the prognosis and, accordingly, the necessary therapy. Ctg1 means no obvious signs of disease activity and progression and allows discontinuation of therapy. Ctg2 implies the possibility of maintaining activity / progression, suggests a gradual decrease in the severity of activity / progression, with a slow transition to an inactive non-progressive status / remission, and is the basis for maintenance therapy. Ctg3 requires continuation of therapy, which can be gradually reduced to maintenance as the pt observes. Ctg4 corresponds to the active status, the progression of the manifestations of the disease is assumed, and is the basis for prescribing or intensifying therapy.

**Results:** The age of the pt was 3.8-17.9 years (у) (m±σ 11.06±3.8), with the disease duration at the time of the study being 0.4-12.8 у (m±σ 4.2±3.2).

jLSc forms - morphea 18%, linear with damage to the trunk and extremities 42%, linear with damage in the head area 37%, generalized 1%, mixed 2% (with the presence of forms - morphea and linear with damage to the trunk and extremities) .

Localization of lesions: on the face - 37 pt, on the trunk - 40 pt, on the extremities - 52 pt.

Antinuclear antibodies were detected in 44% of pt. All of them had no manifestations of Raynaud’s phenomenon, significant capillaroscopic changes, visceral lesions.

Therapy at the time of the study: 11 pt were not receiving treatment; 30 - only local drugs; the rest received metipred orally (26% of pt; at a dose of 1-11 mg); methotrexate 53%, mycophenolate mofetil 2%.

The status of the course of the disease was assessed. No one was in remission status. Pt were registered to have a Ctg4 (50%), a Ctg3 (31%), a Ctg2 (19%)

Based on the data obtained, the following decision was made on further pharmacotherapy: pt in a Ctg4 underwent therapy correction aimed at improving the effectiveness of treatment (pulse therapy with methylprednisolone was performed, methylprednisolone, methotrexate or mycophenolate mofetil or abatacept were prescribed). The rest of the pt either reduced the dose of drugs, or recommended drug withdrawal.

When comparing the data of the LoSSI and LoSDI indices with the types of the course of the disease, it was obtained:

— pt classified as Ctg4 had indices LoSSI min 4 - max 34 (m±σ 8.9 ± 6.3), LoSDI 2-21 (m±σ 4.4 ± 3.7)
— pt categorized as a Ctg3 — LoSSI 2-28 (m±σ 6.7 ± 6.1), LoSDI 2-47 (m±σ 7.4 ± 8.9)
— pt classified as Ctg2 — LoSSI 1-14 (m±σ 4.1 ± 3.6) , LoSDI 1-22 (m±σ 5.1 ± 5.2)

**Conclusion:** We present data from our pilot study on the management of children with localized scleroderma, which we consider useful for developing a treatment approach. The specified allocation of 4 subtypes contributes to the formation of a clear verbal judgment about the current status of the patient, which facilitates prediction and makes it possible to make a rational choice of treatment. Requires additional verification and statistical analysis in the future.

**Disclosure of Interest**: None declared

### P336. Clinical and immunological profile in children with positive anti-rnp antibodies: single center experience

#### M. Kaleda, I. Nikishina, S. Salugina, E. Fedorov, N. Yudkina, Z. Verizhnikova

##### Pediatrics, V.A. Nasonova Research Institute of Rheumatology, Moscow, Russian Federation

###### **Correspondence:** I. Nikishina

**Introduction:** Anti-RNP antibodies are considered specific for mixed connective tissue disease (MCTD). Some authors described that anti-RNP antibodies may be present in defined rheumatic diseases (RD), and associated with particular clinical features, for example, like scleroderma-like features in patients (pts) with systemic lupus erythematosus (SLE).

**Objectives:** According to a retrospective study, to describe clinical and immunological characteristics of children with RD with positive anti-RNP antibodies (antiRNP+), which were observed in our pediatric rheumatology center.

**Methods:** The study included all pts with RD, who had antiRNP+ during past 3 years. We tested pts with antiRNP+ on 4 different sets of criteria for MCTD: Kasukawa’s, Alarcon-Segovia, Kahn and Sharp criteria.

**Results:** A total of 23 pts with RD and anti-RNP+ were selected. All pts were girls. The median age at onset of RD was 11.5 years [IQR 8.6; 13.2]. 9 pts had criteria for defined RD: 2 - SLE, 2 - SLE with Sjögren’s syndrome (SS), 2 - rheumatoid factor-positive (RF+) juvenile idiopathic arthritis (RF+ JIA) with SS, 1 - primary SS, 1 - systemic sclerosis, 1 - juvenile dermatomyositis. 14 pts fulfilled of criteria for MCTD (60.9%): Kahn’s criteria - 12 pts, Sharp’s criteria - 9, Kasukawa’s criteria - 6, Alarcon-Segovia criteria – 5. 3 pts met only one set of criteria, 6 pts – 2 sets of criteria, 3 pts – 3 sets, 2 pts – all 4 sets. The most common combination of criteria – Sharp’s and Kahn’s (8 pts). Sjögren’s syndrome was diagnosed in 18 pts (78.3%). The most common symptoms were arthritis (96.6%), followed by Raynaud’s phenomenon and lymphadenopathy (65.2% each). 60.9% of pts had various skin lesions: 6 - sclerodactyly, 2 - telangiectasias, 3 - malar rash, 2 - heliotrope rash and Gottron’s papules, 2 - erythema nodosum, 1 - livedo reticularis. 52.2% of pts had clinically significant fatigue, 17.4% - myopathy, 8.7% – nephritis, and 4.3% - myocarditis. Interstitial lung disease was observed in 26.1% of pts. Among the pts with SS 16 had isolated involvement of salivary glands, 2 – combined with lacrimal glands. Sicca syndrome was occurred in 12 pts, recurrent parotitis – in 1. All pts had anti-RNP+: 87% of pts - in titre of >200 U/l, others - 72.9, 140, 149 U/l, respectively. All pts had positive antinuclear antibodies (ANA) in high titer: 1/1280 – 65.2%, 1/2560 – 34.8%. Isolated speckled (sp) type of ANA was in 60.9% of pts, isolated homogeneous (h) type - 8.7%, mixed type (h+sp+cytoplasmic) – 30.4%. Other autoantibodies included positive anti-Sm in 10 pts, anti-DNA in 7, anti-Ro in 5. RF+ was in 13 pts. Hypergammaglobulinemia was verified in 11 pts, the median level of IgG was 22.5 g/l [19.6; 26.5], max 43 g/l. Capillaroscopic changes in the nailfold were noted in 15 pts (65.2%): 4 pts had non-specific abnormalities, 5 pts had an early scleroderma pattern, 3 had a late scleroderma pattern with a myopathic component and 3 – a changes characteristic of DM. The most common combination of features of MCTD included fatigue, Raynaud’s phenomenon, arthritis, Sjögren's syndrome, lymphadenopathy and hypergammaglobulinemia (7pts, 50%).

**Conclusion:** In our study, only 60.9% of anti-RNP+ pts had MCTD. Most of the pts with MCTD met the criteria of Kahn and Sharp. The combination of fatigue, Raynaud’s phenomenon, arthritis, Sjögren’s syndrome, lymphadenopathy and hypergammaglobulinemia affected a half of pts with MCTD. Taking into account the high frequency of SS, also Raynaud’s phenomenon in combination with the predominance of scleroderma pattern in capillaroscopy in pts with antiRNP+, we can assume a high probability of the evolution of disorders in these pts along the path of the predominance of the SS and/or systemic sclerosis in the future.

**Patient Consent:** Yes, I received consent

**Disclosure of Interest**: None declared

### P337. Clinical and epidemiological analysis of systemic scleroderma according to the moscow register of children with rheumatic diseases

#### V. Sevostyanov^1^, P. Lototskaya^2^, E. Zholobova^3^

##### ^1^Department of Propaedeutics of Children’s Diseases, ^2^Department of Rheumatology No. 1 of the University Children’s Clinical Hospital, ^3^Department of pediatrics, I.M. Sechenov First Moscow State Medical University Ministry of Health of Russia (Sechenov University), Moscow, Russian Federation

###### **Correspondence:** V. Sevostyanov

**Introduction:** Systemic scleroderma is a multisystem progressive disease with characteristic changes in the skin, musculoskeletal system, involvement of internal organs in the pathological process, as well as vascular disorders.

**Objectives:** Make a clinical and epidemiological analysis of systemic scleroderma according to the Moscow register of children with rheumatic diseases.

**Methods:** An observational descriptive cross-sectional study was conducted, including 67 patients diagnosed with systemic scleroderma (SS), aged from 6.8 to 17.5 years, of which 73% (n=49) were girls and 27% (n=18) were boys living in Moscow.

**Results:** The median age of patients was 12.5 years, the median age at the disease onset was 5.4 years. The time interval from the onset of the disease to the verification of the diagnosis averaged 10.5 months. Twenty-eight patients (43%) had an initial diagnosis other than SS. When verifying the diagnosis in accordance with clinical criteria, all patients had skin lesions, 11.9% had involvement in the process of the gastrointestinal tract, Raynaud’s syndrome was registered in 10.5%. By the nature of the course, acute was noted in 6 (9%) patients, subacute - in 59 (88%), chronic - in 2 (3%) patients. When assessing the activity of the disease in most patients, the activity was assessed as low - in 39 (58%), moderate - in 23 (34.5%), high - in 4 (6%), remission - in 1 (1.5%) child . Basic anti-inflammatory therapy was received by 97% of patients, 42% of them in a combination of several drugs. Biological therapy (BT) is received by 6% of patients. In the structure of BT, abatacept predominates - 75%.

**Conclusion:** Systemic scleroderma is one of the rarest, but difficult to diagnose diseases in children, for the improvement of the prognosis of which timely diagnosis and early initiation of therapy are important.

**Patient Consent:** Yes, I received consent

**Disclosure of Interest**: None declared

### P338. Connective tissue nevus misdiagnosed as juvenile localized scleroderma

#### F. Tirelli^1^, M. Soliani^2^, F. Calabrese^3^, C. Giraudo^4^, G. Martini^1^, A. Meneghel^1^, F. Zulian^1^

##### ^1^Pediatric Rheumatology Unit, Department of Woman and Child Health, University Hospital, Padova, ^2^Department of Pediatrics, ASST di Cremona, Cremona, ^3^Department of Cardiac, Thoracic, Vascular Sciences and Public Health, University Hospital of Padova, ^4^Department of Medicine, University Hospital, Padova, Italy

###### **Correspondence:** F. Tirelli

**Introduction:** Connective tissue nevi (CTN) are benign hamartomas of the dermis caused by excessive proliferation of collagen, elastin and proteoglycans that sometimes may mimic juvenile localized scleroderma (JLS).

**Objectives:** To describe a series of patients with CTN misdiagnosed as having JLS and subsequently treated with immunosuppressive drugs. Possible elements of differential diagnosis are discussed.

**Methods:** We included in the study children referred to our Center between 2001 and 2020 for a second opinion in suspected or confirmed JLS with atypical clinical course and/or unresponsive to treatment, who received a final diagnosis of CTN. Clinical, histological and radiological data were retrospectively collected from the patients’ charts.

**Results:** Sixteen children (11 females), with mean age at onset of 4.4 years (range 2 - 9) were included. All patients were referred to our Center for a second opinion with a confirmed diagnosis of JLS (n=12) suspected JLS (n=2) or suspected fasciitis (n=2). Ten patients (62.5%) were already on systemic treatment with methotrexate (MTX) alone (n=3), MTX + corticosteroids (CS) (n=5) or MTX+CS+Tocilizumab (n=2), while 3 (18.8%) were on topical CS; none had shown improvement despite the treatment. Cutaneous lesions were characterized by skin induration involving the lower limbs in 13 (81.2%) patients, with 6 having also involvement of ipsilateral trunk, and 3 of the upper limbs. Erythema or other signs of skin inflammation were not noted. When JLS damage score (LoSCAT) was applied, activity criteria (LoSSI) were grade 1 erythema and 1-2 skin induration, while among damage criteria (LoSDI) only mild pigmentation changes were present (grade 1-2), usually as hyperpigmentation, and no signs of dermal or subcutaneous atrophy were observed; notably, there was a lack of significant changes in the score over time, which is, in contrast, rather typical in JLS. Inflammatory markers and autoantibodies were absent/negative in all patients. Thermography was negative in 15 patients, only one showed mild hyperthermia of the lesion with homogeneous pattern, not typical for JLS. Fifteen patients underwent musculoskeletal MRI that was negative in 10, while showed skin plane thickening without morphological or subcutaneous tissues abnormalities in 5. Each patient underwent a skin biopsy either performed by us (n=3) or at the referring site (n=13) with revision of the specimens at our Center. Pathology examination confirmed the absence of inflammatory infiltrate and allowed a final diagnosis of non-familial collagenoma in 10 (62.5%), mixed CTN in 4 (25%) and familial CTN in 2 (12.5%). Mean diagnostic delay was 5 years (range 1-15).

**Conclusion:** CTN may mimic JLS but some key elements, such as absence of clinical and histological inflammatory features of the skin lesions, negative autoantibody profile, normal thermography and peculiar MRI features are essential to establish a the differential diagnosis between the two conditions and thus avoid unnecessary treatments.

**Disclosure of Interest**: None declared

### P339. Are there predictor variables for progression of interstitial lung disease in juvenile systemic sclerosis?

#### M. G. Villarreal^1^, M. B. Lucero^2^, A. Uriburu^2^, M. C. Bertinotti^1^, J. Manrique^1^, L. Vasconcellos^1^, M. M. Katsicas^1^

##### ^1^Service of Immunology & Rheumatology, ^2^Service of Pulmonology, Hospital de Pediatría J. P. Garrahan, Buenos Aires, Argentina

###### **Correspondence:** M. M. Katsicas

**Introduction:** Juvenile systemic sclerosis (jSSc) is a multisystem connective tissue disease characterized by skin fibrosis and internal organ involvement including the lungs. Pulmonary manifestations in jSSc are associated with morbidity and mortality. The low incidence of jSSc and the late diagnosis of its pulmonary features results in progressive lung damage with the associated mortality. Further knowledge of this rare disease may improve patient treatment and lung survival.

**Objectives:** To describe pulmonary features associated jSSc and to evaluate potential predictor variables for progression of interstitial lung disease.

**Methods:** This is an observational, retrospective study. Clinical charts of children with a definite diagnosis of jSSc who were admitted at a tertiary referral center between 2000 and 2021 were reviewed. Only patients who had lung involvement with follow up at least ≥36 months±6 months were included. Demographics variables were recorded. Lung specific data collection includes clinical, pulmonary functional tests (PFT) and imaging (HRCT: high resolution computed tomography). PFT parameters included: forced vital capacity (FVC) and diffusing capacity of the lungs for carbon monoxide (DLCO). Pulmonary outcome measures were recorded at follow up (36± 6 months). Outcome measures definitions were: a) clinically significant change on PFT :10% difference in FVC with respect to basal/previous value and 15% in DLCO2. A cut off value of <80% (predicted for age, weight, height, sex and race) was used to determine and abnomal FCV and DLCO as defined in healthy children; b) Abnormal findings in HRCT; c) Progression of interstitial lung disease was defined as an irreversible change on imaging showing indirect signs of fibrosis such as ground glass opacification and/or interstitial thickening. Descriptive statistics and stepwise logistic regression were used for data analysis.

**Results:** Overall 34 jSSc patients, 15 (14 female) patients met inclusion criteria. Age at first symptom attributable to JSS of 8.09 (1.2-15.0) years. Age at lung involvement was 10.2 (1.2- 15.0) years. Lung data collection showed: lung symptoms (4 patients: cough (2), dyspnea (2)), abnormal PFTS (10 patients: abnormal FCV (10), abnormal DLCO (8)), abnormal HRCT 11patients (micronodules/nodules 8, ground-glass opacification 5, interstitial thickening 5, cystic changes 1). Pulmonary outcome measures at follow-up showed: a) PFTs: Improvement on FCV: 1patient (7%); improvement on DLCO: 3 (20%). Twelve (80%) patients did not show changes; b) Abnormal HRCT: 11 (73%); c) progression of interstitial lung disease in 7 patients (interstitial thickening and/or ground glass opacification). Only DLCO was found as a significant risk factor for lung damage(p=0.004). DLCO (median, range) was 95% (54-108) and 72.5% (49-75) for patients without or with lung damage, respectively. Other clinical features (non-pulmonary) were skin induration 80%, musculoskeletal 73%, gastrintestinal27%, cardiovascular 7%. Autoantibodies profile: ANA 100% and Scl70 20%. Pharmacologic treatment: corticosteroids, cyclophosphamide, mycophenolate, D-penicillamine, methotrexate, and vasodilatadors.

**Conclusion:** Pulmonary disease is the most frequent visceral involvement for jSSc. Abnormal DLCO showed increased risk for progression of interstitial lung disease. Unmet medical need in jSSc-ILD remains difficult to treat, with limited options showing poor effectiveness or progression of lung disease evidenced in our cohort. Prospective, multicentric studies are needed to validate definitions for progression lung disease, as well as functional tests adapted to pediatric population.

**Disclosure of Interest**: None declared

## Poster session: Vasculitides

### P340. Demographic, time and clinical pattern of Kawasaki disease; the experience in Libya

#### A. A. Abushhaiwia^1^, R. Algaryani^1^, H. Alrabti^2^, S. T. Ashur^3^, M. Zletni^4^

##### ^1^Pediatric Rheumatology, ^2^Pediatric Cardiology, Tripoli Children’s Hospital; Faculty of medicine; University of Tripoli, ^3^Family and Community Medicine, Faculty of Medicine, University of Tripoli, Libya, ^4^Paediatric Rheumatology , Faculty of Medicine,Tripoli university, Tripoli Children’s Hospital , Tripoli , Libya

###### **Correspondence:** A. A. Abushhaiwia

**Introduction:** Kawasaki disease (KD), an acute, febrile, self-limiting vasculitis of unknown etiology, is a disease that predominantly affects medium- and small-sized arteries of infants and preschool children. Kawasaki disease is the leading cause of acquired coronary artery disease in young children. There is a lack of data on Kawasaki disease and its effect on coronary arteries in Libya and other developing countries.

**Objectives:** This study aimed to describe the demographic, time and clinical patterns of Kawasaki disease (KD) in the Libyan settings. It also examined the association between selected demographic and clinical factors with the presence of abnormal echocardiogram findings at the initial presentation of KD cases

**Methods:** This observational, hospital-based retrospective cohort study was conducted at Rheumatology and cardiac department in Tripoli children’s Hospital in Libya; from January 2012 to December 2020. Those with both complete and incomplete KD were considered. The diagnostic criteria for KD were based on the European and American Heart Association recommendations

**Results:** A total of 71 cases were diagnosed as KD in the period from January 2012 to December 2020, the disease was more prevalent among males (76.1%), and in those aged from 1 to 5 years old (67.6%) than in the younger and older age groups. Most of the identified cases were from Tripoli(67.6%), and the majority were presented first at one of the public health care facilities; primary health care, before being referred to the tertiary hospital. Regarding time distribution, KD cases presented throughout all seasons; however, spring reported the highest percentage (40.8%). Total of them 88.7% were typical KD of patients satisfied the diagnostic criteria for complete KD. Among the cases included,cardiac abnormalities confirmed through echocardiography were documented in 12 (16.9%) ,8 cases out of 12 had changes of the coronary arteries where 2 had ectasia without aneurysm, one case had pericardial effusionall. all of them were detected on the initial evaluation and on the subsequent 2-week evaluation. The presence of abnormal findings dropped to 8 (12.9%) out of 62 cases, and to 5 (12.5%) out of 40 cases in the subsequent echocardiogram examinations done at 6 months and 1 year, respectively

**Conclusion:** Majority of patients fulfilled diagnostic criteria of complete KD, and the presence of coronary artery abnormalities consisted with other international published studies. All patients successfully completely recovered during follow-up, and no mortality was documented

**Patient Consent:** Yes, I received consent

**Disclosure of Interest**: None declared

### P341. Peripheral tuberculous lymphadenitis presenting as henoch–schönlein purpur: a first child case report in Libya

#### A. A. Abushhaiwia^1^, N. Abushhiwa^2^, A. Ateeq^3^

##### ^1^Paediatric Rheumatology , Faculty of Medicine,Tripoli university ,Tripoli Children’s Hospital , ^2^Pathology department , Faculty of Medicine ,Tripoli university , ^3^Paediatric Rheumatology , Tripoli Children’s Hospital , Tripoli , Libya

###### **Correspondence:** A. A. Abushhaiwia

**Introduction:** Tuberculosis is a major public health problem worldwide. It is one of the main causes of infectious disease and mortality, especially in developing countries, Peripheral tuberculous lymphadenitis is the second most common presentation of extrapulmonary tuberculosis in children especially in developing countries, tuberculosis was found to be a triggering factor of Henoch–Schönlein purpura in paediatric patients. Traditionally, cervical lymphadenitis has been the most common site (60-80 %).Given the varied differential diagnoses of lymphadenopathies in paediatrics, PTL is an ongoing challenge for a timely diagnosis and management. Here, we report an unusual case of Henoch–Schönlein purpura associated with peripheral tuberculous lymphadenitis in a young Libyan female child

**Objectives:** we describe a case of peripheral tuberculous lymphadenitis, which was seen to be associated with Henoch-schonlein purpura in a young child.

**Methods:** case report is described

**Results:** A 7 year-old Libyan female child for non-consanguineous parents and had significant past medical history during neonatal period, she had BCG vaccine infected and treated her by anti-tuberculous for 2 months but no family histories neither TB nor autoimmune diseases. Presented to Paediatric rheumatology clinic in April 2022 with progressive bilateral pain over the calves, that rapidly extended to the knees and ankles. Accompanied Cutaneous eruption over the lower extremities followed her since March 2022. The patient also complained of fatigue and painless bilateral cervical lymphadenopathy since December 2021. No fever, no pulmonary, urinary, or abdominal symptoms were noted it. On physical examination, both ankles were erythematous and mild swollen. Palpable purpuric or petechial lesions were covering both lower limbs,there were multiple bilateral cervical lymphadenopathy with bulky (the largest measuring 2 cm in diameter) .Heart and lung auscultation were normal; the abdomen was soft and nontender no organomegaly. Blood tests showed an elevated ESR 103ml\hr,CRP(25) mg/dl),WBC (18X10^3^) mainly neutrophilia 85%, HGB 10, and PLT 648X10^3.^The rest of the analysis was normal and included hepatic and renal chemistry profiles, urinalysis, thyroid-stimulating hormone, complement level, as well as immunoglobulin profile IgA,IgM,IgG,IgE, viral screen(HIV,HBSAg,HCV). A complete auto-immune panel , with anti-neutrophil cytoplasmic antibodies ,cryoglobulin and flowcytomter CD3,CD4,CD8,CD19,CD20,CD56 were sent but still not yet result as we evaluated and considered her immunodeficiency. The tuberculin skin test positive for Tb (PPD: 9 mm) neck, abdominal and pelvis ultrasound showed bilateral cervical lymph node up to 33 mm and multiple bulky and necrotic lymph node enlarged Para aortic &aortocaval. Neck, thoracic and abdominal and pelvis contrast computed topographies demonstrated multiple cervical adenomegaly (up to35x27mm in length), and several retroperitoneal lymph nodes (up to 0.9 cm in length), fine- needle aspiration biopsy in the cervical lymph node was performed,histopathological showed findings compatible with tuberculous lymphadenitis. The patient was initiated of anti-TB treatment (Isoniazid, Rifampicin, Pyrazinamide and ethambutol) it will continue for 6-9 months) and Clarithromycin for 2 weeks. Her vasculitic lesions resolved with anti-tuberculous treatment without the addition of CSs.

**Conclusion:** This is the first paediatric case in Libya that describes an assciocation of Peripheral tuberculous lymphadenitis (TB) infection as a predisposing factor in HSP. This clinical scenario highlights the importance of early suspicion and prompt management. Improved with anti-tuberculosis drugs (ATD) alone.

**Patient Consent:** Yes, I received consent

**Disclosure of Interest**: None declared

### P343. Genetic variants and phenotypic features of childhood large vessel vasculitis: case series and literature review

#### S. Alansari, A. AlSaleem, S. Al-Mayouf

##### Pediatric Rheumatology, King Faisal Specialist Hospital and Research Center, Riyadh, Saudi Arabia

###### **Correspondence:** S. Alansari

**Introduction:** Large vessel vasculitis (LVV) rarely affects children. Clinical characteristics, disease progression and outcomes vary based on the size of blood vessel and organ involved. The precise etiology remains incompletely defined. Recently, genetic variants gained more attention and shed more light on the underlying pathomechanism involved in the various systemic vasculitis.

**Objectives:** To report the phenotypic, genetic findings, outcome of children with LVV.

**Methods:** This work is a retrospective case series of LVV associated with genetic variants from a single tertiary medical center. Also, a systematic literature review was conducted.

**Results:** We identified seven patients with LVV, however two patients were excluded after applying the exclusion criteria. The remaining five patients comprised of 3 males and 2 females. All presented with their primary disease before two years of age. Two patients had recurrent chest infection, skin abscesses and eczema proved to have DOCK8 variants. One patient with FOXP3 variant presented with very early onset inflammatory bowel disease, another patient with DiGeorge syndrome; and one patient had skeletal dysplasia and inflammatory bone disease with homozygous ZNF469 variant and de novo variant in KDM5B. The mean age of the aortitis onset was 12 (±3.6) years and the mean time interval between disease onset and aortitis was 10.6 (±3.7) years. All patients had progressive extensive LVV, affecting mainly aorta and its branches confirmed by radiological study. The most common clinical features were hypertension, abdominal pain, fever and vascular bruit. The most common laboratory findings were high inflammatory markers, leukocytosis, anemia and abnormal hepatic profile. All patients received corticosteroids; three patients received DMARDs. Four patients required surgical intervention, and one underwent hematopoietic stem-cell transplantation. One died and three achieved clinical remission. Furthermore, data of 17 patients extracted from the literature; all included patients had inherited disorders, including hyper-IgE syndrome, Blau syndrome, Wiskott–Aldrich syndrome, Hyper-IgM syndrome, Familial Mediterranean fever, X-linked inhibitor of apoptosis deficiency, Marfan syndrome, Noonan syndrome, Noonan-like syndrome, Juvenile Myelomonocytic Leukemia, and Familial hyper-cholesterolaemia. Eleven patients had genetically proved diagnosis while the diagnosis was based on the expert physician’s opinion and fulfilling the diagnostic criteria. Out of 17 patients, nine patients treated with corticosteroids and eight received DMARDs, one patient completed hematopoietic stem-cell transplantation. Three of them underwent surgical intervention. Most of them showed a reasonable therapeutic response. However, two patients had neurological complications. There were four deaths.

**Conclusion:** LVV associated with genetic variants is a rarely described cluster, which might allow to propose monogenic LVV as a distinct entity. Hopefully, these findings will increase the awareness of the association between LVV with genetic variants, and early recognition can lead to earlier effective intervention in order to improve the outcome.

**Patient Consent:** Not applicable (there are no patient data)

**Disclosure of Interest**: None declared

### P344. Withdrawn

### P345. Withdrawn

### P346. Demographic characteristics of childhood vasculitis other than Kawasaki and Henoch-Schoenlein purpura among arab children: a multicenter study demographic characteristics of childhood vasculitis other than Kawasaki disease and Henoch-Schoenlein

#### R. Bakry^1^, S. Alansari^2^, S. Almayouf^2^ on behalf of Soad Hashad, Awatif abushhaiwia, Ayah Altawati, Halah Etayari, Magada Altafyl, Abdullatif Alenazi, Khulood Khawaja, Wafaa AlSuwairi, Abdulrahman Alrasheed, Jubran Alqanatish, Sima Abusaoud, Wafa Madan, Raed Alzyoud, Muatasem AlSuwaiti, Djohra Hadef

##### ^1^Pediatric Rheumatology, East Jeddah Hospital, Jeddah, ^2^Pediatric Rheumatology, king Faisal Specialist Hospital and Research Center, Riyadh, Saudi Arabia

###### **Correspondence:** R. Bakry

**Introduction:** Childhood vasculitis is an umbrella term that describes a group of disorders that are characterized by inflammation of blood vessel walls. It could be primary or due to secondary causes. Clinical manifestations are heterogeneous and depend mainly on the size of the vessels involved.

**Objectives:** To report the spectrum and clinical manifestations of childhood vasculitis other than Kawasaki disease (KD) and Henoch-Schoenlein Purpura (HSP) in Arab children, and to highlight the long-term outcome.

**Methods:** We conducted a multicenter retrospective study recruiting patients with childhood vasculitis other than KD and HSP during the period between January 2000 and May 2022, from ten Pediatric Rheumatology clinics in seven Arab countries that are members of the Pediatric Rheumatology Arab Group (PRAG). The collected data comprised demographic and clinical findings, and long-term outcome using the Pediatric Vasculitis Damage Index (PVDI).

**Results:** A total of 150 (78 female) patients diagnosed with childhood vasculitis other than KD and HSP before 14 years old with a median age at onset was 7.2 (IQR 4.3-11) years were enrolled. The initial diagnosis was inaccurate in 59.3% and the interval time to diagnosis was 0.5 (IQR 0-1) years. Consanguinity among parents was 42% and positive family history of vasculitis was (n=18, 12%). The most frequent vasculitis was Bechet’s disease (n=37, 24.6%) followed by ANCA associated Vasculitis (n=24, 16%), Takayasu arteritis (n=16, 10.6%), Polyarteritis nodosa (PAN) (n=15,10%) and cutaneous PAN (n=14, 9.3%). The most common clinical manifestations were fever (n=90, 60%), abdominal pain (n=74, 50%), arthralgia (n=64, 42.6%), headache (n=53, 35%), hypertension (n=43,28%), and palpable purpura (n=31, 20.5%). Most patients had elevated inflammatory markers (73%). ANA was positive in 22%, C-ANCA and P-ANCA were positive in 12% of patients. Angiography showed abnormalities in 22%, while echocardiography was abnormal in 21%. Tissue biopsy was performed in 65 patients. Genetic analysis was done in 36 patients; 19 patients had genetic variants. Most patients (84.7%) received corticosteroids with good response. One hundred-thirty-five patients received immunosuppressive therapy; cyclophosphamide used in 23 patients with good response. Fifty-seven patients used biologic agents, the most frequently used biologic agents was Rituximab (n= 18) followed by Infliximab (n= 12) and Tocilizumab (n= 11) with overall improvement more than 90%. The PVDI showed renal complication (8%) with ESRD (2.5%), cardiomyopathy (7.3%), osteoporosis (6%), and blindness (2.4%). No reported bone marrow depression or malignancies. However, there were four deaths related to the disease or comorbidities.

**Conclusion:** This study presents the first and largest data on childhood vasculitis other than KD and HSP in Arab children. It shows a heterogenous spectrum of vasculitis with high prevalence of genetically confirmed vasculitis. This report intended to increase awareness of these diseases among health care providers. Hopefully, this work will be the first step for a prospective registry for childhood vasculitis in Arab countries.

**Disclosure of Interest**: None declared

### P347. Epidemiology of Kawasaki disease in covid times: data from a single center from Eastern India

#### J. Bathia^1^, P. Pal^1^, A. Mondal^2^, S. D. Sarkar^2^, H. De^3^

##### ^1^Department of Pediatric Rheumatology, ^2^Department of Pediatric Medicine, ^3^Department of Pediatric Endocrinology, Institute of Child Health, Kolkata, Kolkata, India

###### **Correspondence:** J. Bathia

**Introduction:** Kawasaki disease (KD) is an acute febrile vasculitis in childhood and is the leading cause of acquired heart disease in children. There is a dearth of data on the epidemiology of KD during the COVID times from India.

**Objectives:** The study has been conducted with the objective to estimate the incidence of KD during the two years of the pandemic due to SARS - CoV 2 and to compare it with the pre-pandemic years.

**Methods:** It is an observational, singe center study conducted at the Institute of Child Health, Kolkata. Data of patients admitted with KD during the first and second wave of SARS-CoV2 were compared with the data from the pre-COVID times

**Results:** 1. The first wave (March 2020- December 2020): 33 KD cases, 18 females and 15 males.

2. The second wave (April 2021 - July 2021): 13 KD cases, 4 females and 9 males.

3. In the pre-COVID times, the incidence of KD was:

- 2018: Total cases 36, females 11 and males 25
- 2019: Total cases 39, females 14 and males 25

There was a rising trend of KD every year with cases doubling in number from 18 in 2009 to 39 in 2019

4. First wave had 33.33% cases and the second wave had 61.54% cases with coronary artery dilatations but no giant aneurysms (z score> +10) were seen during either waves. In comparison, in 2018, 16.67% cases had coronary involvement with giant aneurysms in 2.78% and in 2019 30.77% cases had coronary involvement with giant aneurysms in 5.13%

5. The first wave had 75 cases of MISC, with 22 (29.33%) KD phenotypes. The second wave had 48 cases of MISC, with 23 (47.9%) KD phenotype. The second wave of MISC had a greater proportion of younger children (median age 6.6 years) with doubling of the KD phenotype

**Conclusion:** Incidence of KD was similar to that of the preceding years, following the same upward trajectory despite the lockdown. If the KD phenotype of MISC is taken into account, there was a two-fold increase in the incidence of KD-like illnesses. Inspite of wide spread lockdown with restriction in transportation facilities hospital admission of patients with Kawasaki Disease remained the same.

**Patient Consent:** Yes, I received consent

**Disclosure of Interest**: None declared

### P348. A study on the effectiveness of infliximab following ivig on coronary artery aneurysms in patients with Kawasaki disease

#### J. N. Bathia^1^, P. Pal^1^, M. Roy^2^, D. Biswas^2^, N. Ahmed^2^, M. Bhelo^2^

##### ^1^Department of Pediatric Rheumatology, ^2^Department of Pediatric Medicine, Institute Of Child Health, Kolkata, India

###### **Correspondence:** J. N. Bathia

**Introduction:** Kawasaki Disease (KD) is emerging as the commonest childhood vasculitis in India. Cardiac involvement is the major determinant in long term prognosis. Presence of Coronary Artery Abnormalities (CAAs) carries a high risk of complications such as coronary artery aneurysm, ectasia, ischaemic heart disease, arrythmia and sudden death. Both Infliximab (IFX) and corticosteroids have been proposed as add on drugs for patients with CAAs at diagnosis. This study was undertaken to determine effect of IFX on CAAs

**Objectives:** To determine the effect of IFX following IVIG on regression of CAAs.

**Methods:** This single centre, observational **s**tudy was conducted at the Institute of Child Health Kolkata, India, from January 2016 to December 2019. 33 children aged 6 weeks to 7 years with KD received IFX after 1 or 2 doses of IVIG. IFX was given at 5mg/kg within 24 to 48 hours of completion of IVIG infusion. Of these 33 children, 15 received IFX due to the presence of medium to giant CAAs at diagnosis, increasing CAAs or development of new CAAs following IVIG. Patients were analyzed for change in size of CAAs, as determined by z scores proposed by AHA. Serial echocardiography was done weekly, for the next 4 weeks, then monthly for the next 3 months and then every 3 to 6 months.

**Results:** 15 children with CAAs received IFX, 7 had multiple/ giant CAAs at presentation and 8 with new or enlarging CAAs post IVIG. Seven of them were also IVIG resistant. 7 were infants the youngest being 6 weeks old. 4 infants had giant aneurysms. Diminution in z scores was seen in 80 % (12 out of 15) cases on follow up, giant aneurysms decreased to medium or small sized aneurysms over 6 to 18 months. 50% reduction in the aneurysm size was noted in 60% (n=9) within first 6 months of administration of IFX.

**Conclusion:** 80% of children receiving IFX post IVIG showed progressive decrease in size of CAAs by 18 months. Infliximab therapy has shown to improve treatment response and progression of coronary artery abnormalities

**Disclosure of Interest**: None declared

### P349. A challenging case of fever, recurrent oral ulcers and vertigo

#### J. N. Bathia^1^, P. Pal^1^, H. Jagwani^2^, A. Ghosh^2^

##### ^1^Department of Pediatric Rheumatology, ^2^Department of Pediatric Medicine, Institute of Child Health, Kolkata, India

###### **Correspondence:** J. N. Bathia

**Introduction:** Pediatric Behcet’s disease (BD) is a chronic inflammatory vasculitis which can affect any type and size of vessels. It is characterized by recurrent oral ulcerations, recurrent genital ulcerations along with eye and skin manifestations. The global prevalance is reported to be 10/100000 children. Central nervous system manifestations has been reported in 5 to15% of children with BD with variable features such as meningoencephalitis, headache, focal neurological abnormalities, psychosis etc. It is common in adults but rarely seen in adolescent and children.

**Objectives:** To present a six and half yr old female patient with neuro-Behcet’s disease who came with fever, recurrent oral ulcers and vertigo

**Methods:** Six and a half yr old female child presented to us with a history of recurrent oral ulcers along with fever for a duration of 10 days. Initially she was managed as herpetic gingivostomatitis but there was no improvement. Then she developed rashes on face, trunk, palms and soles and limb weakness.

**Results:** On examination we noted the presence of violaceous, nodular rashes, flat topped plaques on knuckles, arthritis of bilateral proximal interphalangeal joints, nodular lesion in bilateral elbows and bilateral hip pain. Initial investigations were haemoglobin 9.6 g/dl, total leukocyte count 7.2 x 10^3^/μL, platelets 193 x 10^3^/μL, serum creatinine kinase 97 units/litre, eye examination was normal and Tzank smear normal. Oral steroids was started but symptoms persisted. HLA B 5 was also negative. With a suspicion of BD oral colchicine was added which decreased the symptoms. After two months the fever reappeared. It was non-responding. Echocardiography was done which was suggestive of left ventricular hypertrophy with myocarditis leading to a diagnostic dilemma. Non remitting fever was worked up. Infection ruled out, ANA profile, ANCA , immunoglobulin profile was normal. Tuberculosis ruled out. Intravenous methyl prednisolone was started. As the fever was persistent the dose was escalated from 2mg/kg/day to 6 and then 30mg/kg/day. In the meantime, she started developing vertigo for which MRI brain with angiography was done. It showed the presence of multiple small white matter signal abnormalities likely ischaemic foci. Itravenous cyclophosphamide for given for 6 cycles during which the fever subsided after the first cycle. After 2 months fever, ulcers and vertigo reappeared and mycophenolate mofetil (MMF) was added. Patient is now on colchicine, tapering dose of steroids and MMF and is in remission

**Conclusion:** Pediatric neuro-Behcet’s disease is a challenging disease and is not well described. Although rare it should be considered even with subtle neurological manifestations in children with recurrent ulcers. Early diagnosis and immunosuppressive therapy is pivotal.

**Patient Consent:** Yes, I received consent

**Disclosure of Interest**: None declared

### P350. Evolution of an immunological disease over a decade

#### J. N. Bathia^1^, P. Pal^1^, G. S. H^2^, M. Bhelo^2^, D. Biswas^2^, S. D. Sarkar^2^

##### ^1^Department of Pediatric Rheumatology, ^2^Department of Pediatric Medicine, Institute of Child Health, Kolkata, India

###### **Correspondence:** J. N. Bathia

**Introduction:** Burkitt’s lymphoma (BL) is a rare and a very aggressive B-cell lymphoma that represents less than 1% of all non-Hodgkin lymphomas (NHL). One of the clinical subtypes of BL is immunodeficiency-related form common among HIV-infected patients, patients with autoimmune diseases and patients with primary immunodeficiency disorders. Autoimmune disorders such as SLE, vasculitis, celiac disease etc have been associated with an increased cancer risk, particularly for certain cancer types. The underlying pathophysiology is yet to be understood.

**Objectives:** We report a case of ANCA- negative small vessel vasculitis who developed Burkitts lymphoma after 10 years of initial diagnosis.

**Methods:** 5 year old girl presented with a history of intermittent painful nodular lesions over her lower limbs for 3 months, two episodes of haemoptysis followed by persistent fever for last 15 days. Investigations showed neutrophilic lymphocytosis, high titres of CRP and ESR and thrombocytosis. Skin Biopsy revealed deep dermis blood vessels with neutrophilic infiltrates and nuclear debris- suggestive of vasculitis. Bronchoscopy showed normal anatomy and congested mucosa. Bronchoalveolar lavage microscopy revealed inflammatory background with many hemosiderin laden macrophages. Antinuclear antibody (ANA), antineutrophil cytoplasmic antibodies (c- ANCA) and perinuclear antineutrophil cytoplasmic antibodies (p-ANCA) were negative. A diagnosis of ANCA negative small vessels systemic vasculitis was made and she was started on steroids and pulse cyclophosphamide. On follow up, she was maintained on mycophenolate but had multiple relapses on attempting to taper steroid. Finally, she was administered Rituximab after two years which had to be repeated after a year for a minor flare. She was in remission but required two hospital admissions at an interval of six months for lobar pneumonia. Investigating for any associated immunodeficiency showed persistent low levels of IgG, which had developed post Rituximab. She was kept on cotrimoxazole prophylaxis and remained asymptomatic.

**Results:** At the age of 15years she presented with pain abdomen with generalized discomfort for 15 days and acute onset right sided ptosis and right medial rectus palsy for 7 days. MRI Brain with contrast revealed irregular enhancing lesion at the confluence of inferior sagittal sinus with the straight sinus- due to venous sinus thrombus/ mass lesion. USG abdomen showed right adnexal mass and biopsy from the mass confirmed the diagnosis of Burkitts lymphoma

**Conclusion:** Several studies have evaluated the association between autoimmunity and cancer. The factors that are speculated to influence the association include medications for autoimmune disease and interaction between treatment and viral exposure. The development of NHL from activated lymphocyte suggests that chronic inflammation might increase the risk of lymphoma in autoimmune diseases

**Patient Consent:** Yes, I received consent

**Disclosure of Interest**: None declared

### P351. The characteristics of Beschet’s disease in Russia: the preliminary data of multicentral retrospective cohort study

#### K. Belozerov^1^, V. Klavdenkova^1^, Z. Shogenova^1^, A. Yakovlev^1^, L. Andaryanova^1^, E. Gaidar^1^, V. Masalova^1^, T. Kornishina^1^, E. Isupova^1^, L. Snegireva^1^, O. Kalashnikova^1^, L. Sorokina^1^, M. Kaneva^1^, I. Chikova^1^, T. Likhacheva^1^, M. Dubko^1^, V. Chasnyk^1^, T. Burtseva^2^, V. Argunova^3^, P. Sleptsova^3^, S. Boeskorova^4^, L. Leonteva^5^, M. Kostik^1^

##### ^1^Department of Hospital Pediatrics, Federal State budgetary Educational Institution of Higher Education «St. Petersburg State Pediatric Medical University» of the Ministry of Healthcare of the Russian Federation, Saint-Petersburg, ^2^ Department of Pediatrics and pediatric surgery, North-Eastern Federal University , ^3^Cardiorheumatology, State Autonomous Institution of the Republic of Sakha (Yakutia) Republican Hospital No. 1 - National Center of Medicine, ^4^Department of Pediatrics and pediatric surgery, North-Eastern Federal University, ^5^Yakut science centre of complex medical problems, Rheumatological, Yakutsk, Russian Federation

###### **Correspondence:** M. Kostik

**Introduction:** Beçet’s disease (BD) is a rare systemic vasculitis, associated with certain nationalities, related to Great Silk Road. BD in Russia is rare and the data about BD in Russia is scarce.

**Objectives:** to describe clinical course of BD in Russia.

**Methods:** in the retrospective cohort study we included data from patient’s case histories. We evaluated demography, family history, clinical and laboratorial features, treatment options and outcomes. The diagnosis was made according criteria of the International Study Group for BD, 1990.

**Results:** from 44 patients with inclusion age 21.5 years (15.7; 38.6) 59.0% (26/44) had pediatric onset (11 females) and 41% (18/44) adult onset (12 females). Asians/Causacians was 9 (20.5%)/ 35 (79.5%) patients. BD positive family history was in 4 patients (9%). The most frequent first symptom of BD was oral ulcers in 31/44 (70.5). Among clinical features patients with BD had the following organ and system involvement: oral ulcers -95%, genital ulcers - 53%, ulcers of both localizations - 52%, eye involvement - 45%, skin - 48.7%, positive pathergy phenomenon - 50%, CNS - 23%, GI - 39%, joints - 59%, thrombotic events/large vessel vasculitis - 7%. Laboratorial features: ESR - 21.0 (12.5; 27.8) mm/h, CRP - 3.9 (0.4; 14.5) mg/l, number of patients with increased ESR - 55%, with increased - CRP 55%, with anemia - 36.4%. The rate of HLAB51 positivity was 50%, HLAB27 positivity - 40%, RF positivity - 18.2%. The main comorbidity included Crohn’s disease 4 (9%)

Treatment options included: corticosteroids – 67%, colchicine – 42%, TNF-a inhibitors 37% (etanercept 6.25%, adalimumab 43.75%, golimumab and infliximab 25% each), azathioprine – 26%, cyclophosphamide and methotrexate – 10 % each. Also less frequent medications used: canakinumab (n=1), tocilizumab (n=2), tofacitinib (n=1), cyclosporine A (n=1), MMF (n=1), hydroxychloroquine (n=2), sulfasalazine (n=3). In 5 patients biologics were switched.

**Conclusion:** the data about BD in Russia is limited, prevalence underestimates, big diagnostic delay is typical and further investigations are required.

**Patient Consent:** Not applicable (there are no patient data)

**Disclosure of Interest**: None declared

### P352. Takayasu’s arteritis revealed by severe hypertension: about 3 pediatric cases

#### K. Bouayed, S. Lotfi, A. Sakhi

##### Department of Pediatric Rheumatology and Internal Medicine, Hôpital Mère Enfant Abderrahim Harouchi, Casablanca, Morocco

###### **Correspondence:** K. Bouayed

**Introduction:** Takayasu’s arteritis (TA) is a chronic panarteritis mainly affecting the aorta and its main branches, which occurs rarely in children.

**Objectives:** We describe the clinical and paraclinical features, the treatment strategies and the evolutive profile of three cases of TA. We draw attention to the association with tuberculosis.

**Methods:** We report three cases of TA, revealed by severe arterial hypertension.

**Results:** Three gilrs with a mean age of 11 years were admitted with severe arterial hypertension, revealed in one case by headaches and tinnitus, and in two cases by a status epilepticus. The clinical examination showed decreased femoral pulses and an audible murmurs at auscultation of abdominal aorta in one case, discrepancy of four limb systolic blood pressure >10 mmHg in another case. Two of our patients had glomerular injury confirmed either by an albuminuria/creatinuria ratio at 539 mg/mmol or by a 24-hour proteinuria at 111mg/kg/day. TA was confirmed by CT angiography which revealed an extensive stenosis of the sub-renal abdominal aorta and of the left renal artery in the first case, a total occlusion of the right renal artery in the second case, and a parietal thickening of the supra- and sub-renal abdominal aorta and the left renal artery in the last case. DMSA renal scintigraphy showed unilateral decline in renal function according to the location of the vascular damage, estimated at 36%, 8% and 5% respectively. All our patients were treated with anti-hypertensives, corticosteroids and azathioprine, with therapeutic failure in both cases leading to nephrectomy, one of them was followed by aorto-aortic bypass surgery. The evolution was favourable for the third patient who presented an associated tuberculosis diagnosed on cervical lymph nodes, an important inflammatory syndrome and a positive Quantiferon and treated with antibacillary drugs with a normalization of the inflammatory markers.

**Conclusion:** Our patients presented a severe clinical features with malignant hypertension very difficult to control. The association with tuberculosis, as described in our case, has already been reported in tuberculosis endemic countries.

**Disclosure of Interest**: None declared

### P353. Double coronary aneurisms in Kawasaki disease successfully treated with anakinra: a case report

#### K. Bouayed^1^, S. Lotfi^1^, A. Sakhi^1^, N. Mikou^1^, A. Driguil^2^

##### ^1^Department of Pediatric Rheumatology and Internal Medicine, Hôpital Mère Enfant Abderrahim Harouchi, ^2^Department of Cardiology, CHU Ibn Rochd, Casablanca, Morocco

###### **Correspondence:** K. Bouayed

**Introduction:** Kawasaki disease is an acute inflammatory vasculitis of the medium and small-caliber arteries, usually occurring in children under 5 years of age. Refractory Kawasaki disease is associated to a major risk of coronary arteries abnormalities and its treatment is not standardized. In this regard, Anakinra, a interleukin-1 receptor antagonist, represents an emerging therapeutic option.

**Objectives:** We report the case of a 9-month-old girl, diagnosed with refractory Kawasaki disease, successfully treated with Anakinra 3 months.

**Methods:** We report the case of a 9-month-old girl, diagnosed with Kawasaki disease, refractory to two doses of intravenous immunoglobulin and acetyl salicylic acid, who developed aneurysms, successfully treated with Anakinra 3 months with 4 years of follow-up

**Results:** A previously healthy 9-month-old girl was admitted with an 8-day persistent fever of 39.7°C, a perineal scaling erythematous rash, bilateral non-purulent conjunctivitis, cheilitis, right cervical lymphadenopathy measuring 2 cm, and asthenia without extremity abnormalities. Blood tests showed normochromic microcytic anemia (Hb:9.9 g/dL, MCV: 69 fl, MCHC: 34 g/dL), hyperleukocytosis at 17.3 G/L with a predominance of neutrophils 11.64 G/L, normal platelets (289 G/L), elevated ESR at 122 mm at the first hour and C-reactive protein at 260 mg/L, hyponatremia (120 mmol/L), hypoalbunemia (28g/L), and transaminases at 69 U/L for AST and 33 U/L for ALT, urine cytobacteriological examination was normal. Kawasaki disease was diagnosed with a Kobayashi score of 4, predicting IVIG resistance. Treatment with IVIG (2 g/kg) and aspirin (100 mg/kg) was initiated on day 9 of fever and an echocardiogram performed on day 11 of fever was normal. The patient received a second dose of IVIG on day 16 because of persistent fever and increased inflammatory markers. Iterative CARDIAC ultrasound revealed an aneurysm exacerbation in the left coronary artery (6 mm with Z-score +11) and in the left anterior descending coronary artery (4 mm with Z-score +11). The blood tests showed a significant increase in platelets (758 G/L) with a decrease in ESR (40 mm/h) and CRP (74 g/L). Regarding the significant coronary lesions, a second-line treatment with Anakinra 5 mg/kg/day was introduced for 3 months associated with a dose of anti-aggregating aspirin 5 mg/kg/day leading to apyrexia on day 6 of Anakinra, complete normalization of inflammatory tests at month 3 and coronary arteries at month 8.

**Conclusion:** Our experience supports the existing data on the efficacy of Anakinra as a second-line treatment in some cases of refractory Kawasaki disease, particularly in cases of severe coronary artery involvement.

**Patient Consent:** Not applicable (there are no patient data)

**Disclosure of Interest**: None declared

### P354. Monogenic mimics of Behçet’s disease

#### A. Burleigh^1,2^, E. Omoyinmi^2^, C. Papadopoulou^3^, E. Al-Abadi^4^, D. Eleftheriou^1,2,3^, P. Brogan^2,3^

##### ^1^Centre for Adolescent Rheumatology Versus Arthritis at UCL, ^2^UCL Great Ormond Street Institute of Child Health, ^3^Great Ormond Street Hospital for Children NHS Foundation Trust, London, ^4^Childhood Arthritis and Rheumatic Diseases Unit, Birmingham Women’s and Children’s Hospital NHS Foundation Trust, Birmingham, United Kingdom

###### **Correspondence:** A. Burleigh

**Introduction:** Behçet’s Disease (BD) is a rare, polygenic autoinflammatory vasculitis. Multiple risk alleles across numerous genes have been associated with BD, the strongest association of which is with HLA-B*51. There is, therefore, no single genetic hallmark of BD, making definitive diagnosis challenging. Many rare monogenic diseases can present with symptoms that are indistinguishable from BD^1^, and it is vital that BD is differentiated from these so-called ‘monogenic mimics’, as the treatments and prognoses can differ.

**Objectives:** The aim of this study was to undertake whole exome sequencing in an unselected cohort of patients from the UK with clinically diagnosed BD to investigate the possibility of a monogenetic pathogenesis. This will enable:

- Informed diagnoses and treatments for the recruited patients.
- Insight into the genetics of BD-like disease.
- Potential for novel autoinflammatory disease and variant discovery.

**Methods:** Patients were recruited from five hospitals in London, Birmingham, and Oxford. The only inclusion criterium was a clinically suspected diagnosis of BD. Patients’ DNA was whole exome sequenced, and analysis performed using three approaches:

1. Rare variants in the ~400 genes of our in-house vasculitis and inflammation panel were manually assessed.
2. Potential disease-causing variants were prioritised based on connection to phenotype using Exomiser^2^.
3. HLA genotype was obtained using OptiType^3^.

**Results:** A total of 33 patients were recruited, 13 female, median age 13 (3-51) and median age of onset 5 (0-20). Using our genetic analysis pipeline, eight patients were found to have suspected monogenic BD mimic diseases:

- Four cases of Haploinsufficiency of A20 with five novel *TNFAIP3* mutations (p. p.G316S, p.S548Dfs, p.M112Tfs, p.C657fs, p.E661Nfs).
- One case of ISG15 deficiency with a novel homozygous *ISG15* mutation (p.Q16X).
- One case of common variable immune deficiency with a pathogenic *TNFRSF13B* mutation (p.A181E).
- Two cases of Tumour necrosis factor receptor associated periodic syndrome (TRAPS) with a pathogenic low penetrance *TNFRSF1A* mutation (p.R121Q).

Of the patients for whom a monogenic cause could not be discerned, seven were HLA-B*51 positive, and classified as having typical BD. The remaining 16 patients were classified as having BD without monogenic cause or HLA-B*51 genotype. Two cases remain under analysis. The functional consequences of the novel mutations discovered in this study are under investigation.

**Conclusion:** This work has expanded the genotypic spectrum of autoinflammatory diseases that can mimic BD. The genetic diagnoses made in this study have enabled clinicians to make informed decisions regarding individuals’ treatments and prognoses, highlighting the importance of next generation genetic sequencing in patients with suspected BD. The analysis workflow designed for this study offers a template for which genetic characterisation of BD-like disease could be implemented more routinely, both for identification of monogenic diseases, and for HLA-typing.

1.Papadopoulou C, Omoyinmi E, Standing A, Pain CE, Booth C, D’Arco F, et al. Monogenic mimics of Behçet’s disease in the young. Rheumatology. 2019

2. Smedley D, Jacobsen JOB, Jäger M, Köhler S, Holtgrewe M, Schubach M, et al. Next-generation diagnostics and disease-gene discovery with the Exomiser. Nature Protocols. 2015;10(12):2004-15

3. Szolek A, Schubert B, Mohr C, Sturm M, Feldhahn M, Kohlbacher O. OptiType: precision HLA typing from next-generation sequencing data. Bioinformatics. 2014;30(23):3310-6

**Patient Consent:** Not applicable (there are no patient data)

**Disclosure of Interest**: None declared

### P355. The nailfold videocapillaroscopy in pediatric behçet’s disease: a multi-center study

#### F. Çakmak^1^, S. Doğantan^2^, R. İşgüder^3^, M. Kasap Cüceoğlu^4^, S. Çağlayan^5^, H. E. Sönmez^6^, Ö. Akgün^1^, B. Sözeri^5^, A. Paç Kısaarslan^2^, E. Ünsal^3^, S. Özen^4^, N. Aktay Ayaz^1^

##### ^1^Pediatric Rheumatology, Department of Pediatric Rheumatology, Istanbul University Medical School, Fatih, Istanbul, Turkey, istanbul, ^2^Pediatric Rheumatology, Kayseri Erciyes University, Kayseri, ^3^Pediatric Rheumatology, Dokuz Eylül University, İzmir, ^4^Pediatric Rheumatology, Hacettepe University, Ankara, ^5^Pediatric Rheumatology, Umraniye Training and Research Hospital, istanbul, ^6^Pediatric Rheumatology, Kocaeli University, Kocaeli, Turkey

###### **Correspondence:** F. Çakmak

**Introduction:** Behçet’s disease (BD) is a chronic inflammatory disease characterized by recurrent oral aphthous and genital ulcers accompanied by eye, joint, skin, gastrointestinal and central nervous system involvement. The vascular involvement may affect both the arterial and venous systems. Nailfold videocapillaroscopy (NVC) is an easy and non-invasive method used in the evaluation of microcirculation.

**Objectives:** With this study, we aimed to find the characteristics and prevalence of nailfold capillary alterations in patients with juvenile BD and to analyze their possible relationship between clinical characteristics and activity of the disease.

**Methods:** Patients aged 5-21 years with a diagnosis of juvenile BD and followed up for at least six months were included in the study. Demographic and clinical characteristics of the patients were recorded. NVC was performed on 8 fingers of both hands, excluding the thumbs, and four consecutive non overlapping fields for each of fingers were evaluated (32 fields per patient). Capillary density, capillary width (arterial width, venous width, apical loop), capillary morphology and the presence of meandering capillary, microhemorrhage, avascular area, neoangiogenesis, capillary ramification were evaluated from the images. Capillary morphology were evaluated by classifying them into four groups as normal, minor abnormalities, major abnormalities and scleroderma pattern. The presence of abnormilities in at least two fingers were recorded as capillary abnormality. The semiquantitative rating score 1-3 was applied for each capillaroscopic alteration.

**Results:** 37 patients from 6 pediatric rheumatology centers were included in the study. The mean age of patients was 17 years (IOR 13-19) and 20 (54.1%) of them were girls. The patients were evaluated in four clusters according to their clinical presentations. Nineteen patients had mucocutaneous involvement, 9 patients had uveitis, 8 patients had vascular and neurological involvement, and 4 patients had gastrointestinal system involvement. During the follow-up period, genital ulcers developed in 22 patients, erythema nodosum in 9 patients, pseudofolliculitis in 18 patients, uveitis in 10 patients, vascular involvement in 8 patients, and neurological involvement in 5 patients. Anterior uveitis was present in five, posterior uveitis in three, panuveitis in one, and retinal vasculitis in three of the patients with ocular involvement. Four patients had lower extremity venous thrombosis, three patients had central nervous system (CNS) thrombosis, and one patient had both lower extremity and CNS thrombosis.

When capillary morphology was evaluated; normal morphology was present in 16 patients, minor abnormality in 13 patients, and major abnormality in 8 patients. Median capillary density was 8, capillary length was 325 μm, arterial width was 12 μm, venous width was 16 μm, apical loop width was 18 μm, capillary width was 39 μm, and intercapillary distance was 107 μm. Neoagiogenesis was seen in 13 patients, enlarged capillaries in 12 patients, capillary meandering in 9 patients, bushy capillaries in 5 patients, bizarre capillaries in 4 patients, and microhemorrhage in 3 patients. Neoangiogenesis was found to be significantly more common in the NVC evaluation of patients with lower hemoglobin values ​​at the time of diagnosis (p=0.014).

**Conclusion:** NVC is an in vivo, non-invasive, and inexpensive imaging technique that allows the direct observation of the capillary network in living tissue throughout the skin and it may be preferred in juvenile BD for evaluating microvascular involvement.

**Patient Consent:** Yes, I received consent

**Disclosure of Interest**: None declared

### P356. Is there an increased future cardiovascular risk in children with vascular behçet’s disease?

#### S. Demir^1^, A. DÜZOVA^2^, O. Cimen^1^, E. Sağ^1^, B. Oguz^3^, T. Karagöz^4^, S. Ozen^1^, Y. Bilginer^1^

##### ^1^Depatrment of Pediatric Rheumatology, ^2^Depatrment of Pediatric Nephrology, ^3^Department of Radiology, ^4^Department of Pediatric Cardiology, Hacettepe University Medical Faculty, Ankara, Turkey

###### **Correspondence:** S. Demir

**Introduction:** Behçet’s disease (BD) is a polygenic multisystemic autoinflammatory disorder and vascular involvement is one of the major causes of morbidity and mortality of the disease. The disease is commonly seen in young adults however can occur in childhood, as well.

**Objectives:** We aimed to define whether vascular involvement of pediatric BD is a risk factor for a future cardiovascular disease.

**Methods:** Thirty-one BD patients who followed at pediatric rheumatology outpatient clinic, were enrolled to the study. The pediatric patients (<16 years of age at disease onset and diagnosis) were classified as having BD according to the Pediatric Behçet’s Disease (PEDBD) classification criteria. Demographic data, clinical manifestations, laboratory and radiological findings and outcomes were documented from patient charts. Patients with incomplete BD, and have other known risk factors for cardiovascular disease as obesity and hypertension were excluded. During the same week, carotid intima-media thickness (cIMT) measurement and echocardiography and 24-hour ambulatory blood pressure monitoring (ABPM) were performed. Physical examination and blood tests were also performed on the same day with cIMT.

**Results:** Thirty-one children with pediatric BD (16 female, 51.6%; F/M: 1.06) were enrolled in the study. The patients were classified as having BD according to the Paediatric BehÇet’s Disease (PEDBD) classification criteria. Based on the cumulative disease characteristics; oral ulcer was the most common clinical finding (100%), followed by skin involvement (78%, n=25), arthritis (56%, n=18), genital ulcers (47%, n=15), ocular involvement (28%, n=9) had and vascular involvement (18%, n=6). We grouped patients into two groups as patients with and without vascular involvement. The mean age at disease onset and at the time of BD diagnosis was 8.79 ± 4.23 years, and 11.62 ± 3.22 years, respectively. The median follow-up duration was not statistically different between two groups (59.63 months (IQR: 58.38-63.57) vs 40.28 months (IQR: 11.30-75.47)). There was no significant difference between the two groups in terms of other organ involvement, except for CNS involvement. In patients with vascular involvement, VLDL, Triglyceride and CRP levels were found to be significantly higher and HDL levels were found to be significantly lower (Table). The mean values of right cIMT, left cIMT were higher in patients with vascular involvement, however it did not reach statistical significance. Similarly, the prevalence of abnormal ABPM, non-dipping, and ambulatory hypertension was higher in patients with vascular involvement, they did not reach to statistical significance, as well. In echocardiography measurements, vascular BD patients had significantly higher rates of aortic outflow and velocity integral of the aorta which points out increased stiffness of the aorta.

**Conclusion:** We suggest that, pediatric-onset vascular Behçet’s disease may increase the risk of advanced cardiovascular disease. Long-term follow-up studies in larger series may clarify the value of ABPM.

**Patient Consent:** Yes, I received consent

**Disclosure of Interest**: None declared

### P357. Longterm kidney prognosis of anca vasculitis in children

#### M. Drozynska-Duklas, I. Zagozdzon, I. Balasz-Chmielewska, I. Zaluska-Lesniewska, A. Zurowska

##### Department of Paediatrics, Nephrology and Hypertension, ERKNet ERN-Rare Diseases Centre, Medical University of Gdansk, Gdansk, Poland

###### **Correspondence:** M. Drozynska-Duklas

**Introduction:** Antineutrophil cytoplasmic antibody (ANCA)-associated vasculitis (AAV) is a necrotizing vasculitis associated with the presence of antibodies specific for myeloperoxidase or proteinase 3.

**Objectives:** Prognosis in children with AAV has improved but is still associated with morbidity and mortality and the long term kidney outcome in these children is not well described. The aim of this study was to analyze the medical data of patients previously treated in our centre for AAV.

**Methods:** Between 2004-2022 a medical record search identified eight children with AAV at a single pediatric nephrology centre serving a population of 2 million inhabitants. The disease was diagnosed in 6 girls and 2 boys at a mean age of 13.1 (range: 8,6-16,7) years. Microscopic polyangiitis (MPA) was recognized in five and granulomatosis with polyangiitis (GPA) in three subjects.

**Results:** In subjects with MPA the prevailing initial symptoms were those of advanced kidney failure frequently with little extrarenal involvement. Upper and lower respiratory tract involvement predominated in GPA subjects who at onset showed less severe kidney symptoms. Initial eGFR in MPA patients was decreased (mean: 28 ml/min/1,73m^2^) in contrast to usually normal values in GPA subjects (mean eGFR 125ml/min/1,73 m^2^). Diurnal proteinuria was more severe in MPA (mean: 3,43 g/day) than in GPA children (mean: 0,83g/day). All patients underwent intensive immunosuppression. After a mean follow-up of 7 years four out of five MPA subjects were on renal replacement therapy but had not experienced further vasculitis relapses. GPA subjects had frequent relapses but after a mean follow-up of 9 years had normal eGFR (mean:105 ml/min/1,73m^2^).

**Conclusion:** Systemic ANCA-associated vasculitis with renal involvement is an ultra-rare disease in children.

MPA carries a poor prognosis with frequent late kidney manifestation of advanced Chronic Kidney Disease.

GPA has less severe kidney manifestations at onset with better kidney prognosis in spite of a relapsing course.

**Patient Consent:** Not applicable (there are no patient data)

**Disclosure of Interest**: None declared

### P358. Association between glutathione s-transferase (gst) m1,t1 and a1 polymorphisms and iga vasculitis: a pilot study

#### A. Juras^1^, M. Held^2^, M. Sestan^2^, M. Batnozic Varga^3^, N. Kifer^2^, S. Srsen^4^, A. Gagro^5^, M. Frkovic^2^, S. Huljev Frkovic^2^, M. Jelusic^2^, K. Crkvenac Gornik^1^

##### ^1^Department of Laboratory Diagnostics, University Hospital Centre Zagreb, ^2^Department of Pediatrics, University Hospital Centre Zagreb, University of Zagreb School of Medicine, Zagreb, ^3^Department of Pediatrics, University Hospital Centre Osijek, Josip Juraj Strossmayer University of Osijek, Faculty of Medicine, Osijek, ^4^Department of Pediatrics, University Hospital Centre Split, University of Split School of Medicine, Split, ^5^Children’s Hospital Zagreb, Josip Juraj Strossmayer University of Osijek, Faculty of Medicine, Zagreb, Croatia

###### **Correspondence:** M. Held

**Introduction:** IgA vasculitis (IgAV) is the most common childhood vasculitis. Considering the clinical heterogeneity, genetic factors might play a role in pathogenesis of IgAV. Glutathione S-transferase (GST) are members of a multigene family of metabolic enzymes divided into four major subfamilies designated as *GST*α (*GSTA1*), *GSTμ (GSTM1), GSTθ* (*GSTT1*) and *GST* π (*GSTP1*), act as cell housekeepers protect cells against oxidative stressors in the environment by detoxifying a wide variety of potentially toxic and carcinogenic electrophiles. Deletions in GSTs lead to reduction in detoxification enzymatic activity. It was identified that detoxification effects modified by *GST*s polymorphism possibly can aggravate the susceptibility to diseases.

**Objectives:** To investigate the *GSTA1, GSTM1 and GSTT1* genes polymorphism and their influence to susceptibility for IgAV.

**Methods:** Clinical data were collected from four Croatian tertiary centers for pediatric rheumatology. GSTA1, GSTM1, and GSTT1 polymorphisms were detected in patients and controls. DNA was isolated from whole blood using the QIAGEN QIAamp kit. GSTA1 (-69C>T) was examined by the polymerase chain reaction-restriction fragment length polymorphism (PCR-RFLP) method whereas the GSTM1 and GSTT1 were determined by the PCR method.

**Results:** Pilot study included 107 patients diagnosed with IgAV, of whom 56 girls and 51 boys, with median age at the time of diagnosis 6.25 (4.5-8.0) years, as well as 75 sex and age-matched controls. All patients had purpuric rash, 75,7% had arthralgia or arthritis, 36,5% had gastrointestinal involvement, while 31,7% patients developed IgA vasculitis nephritis (IgAVN). The frequencies of GSTM1 (−) null allele and GSTT1 null (−) allele in IgAV patients were 56,1% and 26,2% respectively. There was no statistically significant difference in the null genotype distribution of GSTM1 and GSTT1 between groups (CI 0.49-1.62, OR 0.89, p=0.714; CI 0.35-1.44, OR 0.70, p=0.335). The frequency of GSTA1 C/C, GSTA1 C/T and GSTA1 T/T genotypes in IgAV patients were 36,5%, 44,8% and 18,7% respectively. There was no statistically significant differences in genotype frequencies between patients and controls (CI 0.83-3.00, OR 1.38, p=0.167; CI 0.33-1.09, OR 0.60, p=0.09; CI 0.55-2.65, OR 1.20, p=0.639). Patients with gastrointestinal involvement had statistically significant difference in the null genotype distribution of GSTM1 compared with patients without gastrointestinal involvement (CI 0.15-0.81, OR 0.35, p=0.014).

**Conclusion:** Our pilot study provides evidence that the examinated polymorphisms were not associated with the increase individual susceptibility for IgAV, although GSTM1 genotype proved to have effect on gastrointestinal involvement in IgAV. For precise evaluation of results it is necessary to include larger study populations, however this study offers some essential information for further research.

**SUPPORT:** Croatian Science Foundation IP-2019-04-8822.

**Patient Consent:** Yes, I received consent

**Disclosure of Interest**: None declared

### P359. Retropharyngeal abscess as an atypical presentation of Kawasaki disease: a case report and literature review

#### R. Kasem Ali Sliman, M. Hamad Saied

##### Pediatrics, Carmel Hospital, haifa, Israel

###### **Correspondence:** R. Kasem Ali Sliman

**Introduction:** Only 40% of Kawasaki Disease (KD) patients present with adequate clinical criteria for diagnosis, the remainder present with an incomplete or atypical presentation [1][2].

One of the most challenging cases of KD can present with a clinical and radiological manifestation of a retropharyngeal abscess.

**Objectives:** We describe a patient who presented with an atypical retropharyngeal abscess-like lesion, unresponsive to several antibiotics regimens, as well as surgical drainage, but promptly responded to immunoglobulin treatment once a diagnosis of KD was suspected. In addition, we present a literature review of similar cases published over the last 30 years.

**Methods:** A literature search was conducted, the primary database was PubMed (Medline), Embase and Google Scholar. The key words were Kawasaki disease, Pediatrics, Retropharyngeal abscess. Fifteen articles were found.

**Results:** An eight years old girl presented with three days of fever associated with left neck swelling, tenderness and torticollis, CT scan demonstrated left retropharyngeal swelling with a hypodense lesion of 45mm with a suspected abscess. No improvement after five days of different antibiotics regimens and surgical intervention. On the 8^th^ day of fever, symmetrical arthritis of the PIPs, MCPs and MTPs appeared and a non-purulent conjunctivitis as well as a strawberry tongue with swollen dry lips were noted at her examination. A single dose of IVIG led to a rapid clinical improvement and resolution of fever and other signs and symptoms.

Seventeen case reports including our case describing pediatric patients presenting with retropharyngeal abscess, later diagnosed with KD. The patients were predominantly males (82%), age range from 10 months to 9 years with mean age of 5 years, which considered higher than the average of patients with KD. The majority of the cases (94%) initially presented with fever and neck swelling, without additional clinical criteria, leukocytosis was in all patients with average of 18500 WBC/μL. All the cases were initially concerning for possible deep neck bacterial infection, prompting antibiotic therapy and imaging studies. CT\MRI imaging were performed in all the cases, with findings suspicious for retropharyngeal disease, but in the majority without enhancement.

Intravenous antibiotics were administered in 16 patients (94%), surgical drainage was attempted in 7 patients (41%), with two 2 cases (11%) of purulent fluid, only one of them being culture positive for Staphylococcus aureus. The diagnosis was delayed beyond 9 febrile days in 5 patients (30%). 41% of the patients had a cardiac manifestation compared to less than 20% in the general pediatrics population who are diagnosed with KD. All the patients had a dramatic and fast clinical improvement with resolution of fever within 24-48 hours after IVIG administration.

**Conclusion:** KD requires high index of suspicion and awareness of unusual presentations. In our case and literature review Kawasaki disease mimicked a retropharyngeal abscess that was refractory to antibiotics and surgical intervention.

Thus, It should be kept in mind as one of the differential diagnosis of patients with febrile lymphadenitis and/or retropharyngeal abscess who do not respond to antibiotic treatment in the relevant clinical context. This can prevent delay in diagnosis and the detrimental sequelae especially cardiac complications.

**References**

[1] Isidori C., Sebastiani L. and Esposito S. A Case of Incomplete and Atypical Kawasaki Disease Presenting with Retropharyngeal Involvement. Int J Environ Res Public Health. 2019 Sep; 16 (18): 3262.

[2] Kritsaneepaiboon S., Tanaanantarak P., Roymanee S. and Lee E.Y.Atypical presentation of Kawasaki disease in young infants mimicking a retropharyngeal abscess. Emerg Radiol. 2012 Apr; 19 (2): 159-163

**Patient Consent:** Not applicable (there are no patient data)

**Disclosure of Interest**: None declared

### P360. The paediatric vasculitis activity score (pvas) and proteinuria in igav nephritis: is there an association with different histologic findings?

#### N. Kifer^1^, M. Sestan^1^, M. Held^1^, D. Kifer^2^, S. Srsen^3^, A. Gudelj Gracanin^4^, M. Heshin-Bekenstein^5^, T. Giani^6^, R. Cimaz^6^, M. Frkovic^1^, S. Bulimbasic^1^, A. Gagro^7^, M. Coric^1^, M. Jelusic^1^

##### ^1^University of Zagreb, School of Medicine, UHC Zagreb, ^2^University of Zagreb, Faculty of Pharmacy and Biochemistry, Zagreb, ^3^University of Split School of Medicine, UHC Split, Split, ^4^CH Holly Spirit, University of Zagreb School of Medicine, Zagreb, Croatia, ^5^Dana Dwek Children’s Hospital, Tel Aviv Medical Center, Tel Aviv, Israel, ^6^University of Milan, Milan, Italy, ^7^Children’s Hospital Zagreb, University of Osijek, Medical Faculty Osijek, Zagreb, Croatia

###### **Correspondence:** N. Kifer

**Introduction:** IgA vasculitis (IgAV) is usually self-limiting with a favorable prognosis. However, the development of nephritis (IgAVN) can lead to chronic kidney disease and kidney biopsy has been a continued standard in determining the severity of IgAVN. The association between the disease activity, as well as laboratory parameters descibing kidney function, and different histologic classifications used for IgAVN are still left unclear.

**Objectives:** To determine whether there is an association between histologic variables, measures of disease activity (PVAS) and markers of kidney function.

**Methods:** Patients included were diagnosed with IgAV and IgAVN based on EULAR/PRINTO/PRES criteria in the period from 2003 to 2021. Their renal biopsy findings were examined using light microscopy, immunofluorescence, and electron microscopy analyses. Four classifications were used: ISKDC, Haas classification, Oxford classification, and SQC classification. PVAS was determined at the time of diagnosis.

**Results:** The study included 67 patients, with the median (range) age of 10.8 (3.1-28.5) years at the diagnosis. Fifty-eight percent of patients were male, with a male to female ratio of 1.4:1. The median time from IgAV diagnosis to IgAV nephritis was 5 (0-270) days. The median time from the onset of nephritis to kidney biopsy was 30 (2-2555) days. Laboratory parameters were tested for association with all four classifications. Twenty-four hours protein excretion has shown statistically significant correlation with higher grade in Oxford classification (b= 0.58 ± 0.12, p <0.001), SQC classification (b= 0.44 ± 0.11, p <0.001), and ISKDC classification (b= 0.36 ± 0.11, p <0.001), but not with Haas classification. However, it was found that patients with hematuria had a higher grade in Haas classification, in comparison with patients without hematuria (b= 1.93 ± 0.49, p <0.001). The median (range) PVAS of our patients at diagnosis was 15 (2-30). We explored whether there is an association between the activity of the disease and histologic variables. Endocapillary proliferation and tubular atrophy from Oxford classification were found to associate with PVAS. Endocapillary proliferation showed a statistically significant positive correlation (b= 0.70 ± 0.25, p =0.008), while tubular atrophy was negatively associated (b= -1.39 ± 0.55, p =0.015).

**Conclusion:** Our findings suggest that a higher 24h urine protein excretion could be a possible indicator of higher grades in ISKDC, Oxford and SQC classifications. Furthermore, we have found that when the activity index is high, we could expect acute histologic changes such as endocapillary proliferation, while chronic changes, such as tubular atrophy, could occur after the PVAS score decreases.

Support: Research is supported by Croatian Science Foundation project IP-2019-04-8822

**Patient Consent:** Yes, I received consent

**Disclosure of Interest**: None declared

### P361. Clinical profile and medium-term follow up of childhood-onset polyarteritis nodosa in a tertiary care centre in South India

#### A. Kumar^1^, D. Patro^2^, J. Raghuram^1,3^, A. P. Rao^1,2^

##### ^1^Pediatric Rheumatology, Indira Gandhi Institute of Child Health, ^2^Pediatric Rheumatology, Manipal Hospital, ^3^Pediatric Rheumatology, Aster Whitefield, Bengaluru, India

###### **Correspondence:** A. Kumar

**Introduction:** Polyarteritis nodosa (PAN) is a systemic necrotizing vasculitis predominantly affecting medium-sized vessels. Though it is a multisystem disease, it predominantly involves the skin, gastrointestinal tract (GIT), musculoskeletal system (MSK), and kidneys. Cutaneous PAN is a limited form of the disease wherein the disease remains limited to the skin without any visceral organ involvement.

**Objectives:** To study the clinical profile, laboratory findings, and outcome of childhood-onset PAN.

**Methods:** A retrospective study of 20 children fulfilling the European league against rheumatism (EULAR)/ Paediatric rheumatic European Society (PRES)/ Paediatric Rheumatology International Trials Organization (PRINTO) classification criteria of PAN who were seen over 9 years.

**Results:** The findings are summarized and compared in the table below. The median age of onset of symptoms was 9.4 years (Range: 4- 15 years) with M: F ratio being 1:1. The most common presentation is skin involvement (100%) followed by fever (95%) and MSK manifestations (80%). Deficiency of Adenosine Deaminase 2 (DADA2) was detected in two cases. Labs revealed anemia in 50%, leucocytosis in 65%, and thrombocytosis in 30%. In follow-up, three patients were in complete remission whereas two were lost to follow-up. 9 patients had relapsed on medication whereas 6 patients had relapsed off medication. The mean duration of treatment was 3.5 years. One child succumbed due to a complicated varicella infection and another patient died due to an unrelenting PAN.

**Conclusion:** Childhood PAN is chronic pediatric vasculitis characterized by dermatologic and musculoskeletal manifestations. Early diagnosis and treatment may minimize sequelae in patients with PAN. Relapses occurred more frequently in those with systemic involvement and poor compliance with medication.

**Patient Consent:** Yes, I received consent

**Disclosure of Interest**: None declared

**Table 1 (abstract P361).** See text for description

Parameters	Our study, 2022 (n=20)	Lee, JS et al, 2021 (n=9)	Ozen et al, 2019 (n=66 Pediatric +67 Adult=133)	Eleftheriou et al, 2013 (n=69)
Median age at diagnosis in years (Range in years)	10 (4- 15)	7.6 (3-17.5)	16 (2-75)	8.5 (0.9-15.8)
Male: Female	1:1	2:1	0.88:1	1.22:1
Clinical features:				
A. Fever	95%	77.7%	75.7%	87%
B. Skin features:				
Purpura	85%	88.8%	NA	41%
SC nodule	55%	33.3%	NA	23%
Gangrene	55%	22.2%	0	4%
Livedo reticularis	15%	22.2%	54.5%	49%
Ulcer	25%	22.2%	4.5%	4%
RP	10%	44.4%	4.5%	23%
C. MSK features:				
Arthralgia/ Arthritis	65%	77.7%	89.3%	75%
Myalgia	55%	22.2%	72.7%	83%
D. Weight Loss	45%	11.1%	30.3%	64%
E. GIT			25.7%	
Abdomen pain	20%	22.2%	NA	41%
Perforation/ischemia	10%	11.1%	NA	4%
F. Renal			33.3%	
Proteinuria	5%	NA	NA	19%
Haematuria	10%	NA	NA	10%
Impaired function	10%	11.1%	NA	15%
G. Nervous system			21.2%	
Stroke	15%	11.1%	NA	10%
Peripheral neuropathy	35%	11.1%	NA	8.69%
H. Testicular	50%	NA	6%	10%
I. Hypertension	15%	11.1%	39.3%	16%
Investigations:				
Skin biopsy	70%	88.8%	94/133	72.4%
Angiography	20%	11.1%	92/133	95.6%
ASLO	30%			
Immunomodulator				
Corticosteroids	100%	100%	133/133	100%
Cyclophosphamide	60%	55.5%	62/133	86.9%
MMF	40%	NA	39/133	13%
AZA	70%	44.4%	32/133	78%
Diagnosis:				
Systemic PAN	60%	88.8%	63/133	100%
Cutaneous PAN	40%	11.1%	33/133	0
Mortality	10%	0	0/66	4%
Relapses	75%	77.7%	100%	35%
Median duration of follow up in years	5.4	7	13	6

SLO: Anti-streptolysin-O titer; AZA: Azathioprine; MMF: Mycophenolate mofetil; NA: Not available; RP: Raynaud’s phenomenon. SC: Subcutaneous

### P362. Gene expression analysis of inflammation-induced endothelium dysfunction markers in Kawasaki disease patients from North India

#### R. Kumrah, R. Rikhi, A. Rawat, S. Singh, Ankur Kumar Jindal

##### Advanced Pediatrics Centre, PGIMER, Chandigarh, India

###### **Correspondence:** R. Kumrah

**Introduction:** Kawasaki disease (KD) is an acute medium vessel vasculitis, predominantly affecting children <5 years of age. It is the most common childhood vasculitic disorder causing inflammation of the medium sized coronary arteries. The initial inflammatory insult to the endothelium during the acute phase of KD leads to endothelial injury, while the persistent chronic & smouldering inflammation during the chronic phase leads to endothelial dysfunction.

**Objectives: To do gene expression analysis of inflammation-induced endothelium dysfunction markers in Kawasaki disease patients from North India**

**Methods:** KD patients were enrolled at different time intervals in 3 groups (20 each) as per AHA guidelines and 20 age matched healthy controls. **Group 1**: KD diagnosed > 6months-1.5 years; **Group 2**: >1.5 - 3 years ; **Group 3**: > 3 - 4.5 years prior to enrollment. Complementary DNA (cDNA) converted from extracted whole blood RNA was used to perform real-time PCR analysis. Comparison of fold change [2^(-ΔΔCT) method] between patients and controls were performed.

**Results:**

Real-time PCR analysis for intra group 1 revealed elevated CXCL8, pecam-1, osteopontin in KD patients with coronary artery aneurysms (CAA) as compared to KD patients without CAA (p=0.038 ), (p= 0.05) , (non-significant) respectively. Increased levels of endoglin, resistin, VEGF-A and leptin was found in patients without CAA as compared to patients with CAA (non-significant). Intragroup analysis for group 2 showed increased Pecam-1, CXCL8, resistin, osteopontin and decreased levels of leptin, pentraxin-3 in KD with aneurysms as compared to patients without CAA (non-significant). Levels of endoglin, VEGF-A were comparable. Intragroup analysis for group 3 patients revealed elevated VEGF-A, CXCL8, Leptin, Pentraxin-3, resistin in patients with CAA as compared to KD without aneurysms while pecam-1, endoglin and osteopontin were comparable in both. Significant difference was also found for *Leptin* gene in intergroup analysis (1&2) in patients without CAA.

**Conclusion:** Real time analysis revealed altered gene expression in Kawasaki disease patients with aneurysms.

**Patient Consent:** Yes, I received consent

**Disclosure of Interest**: None declared

**Table 1 (abstract P362).** See text for description

S. no.	Gene	Full forms	Function
1.	*CXCL8*	C-X-C motif chemokine ligand 8	Inflammatory cytokine
2.	*Pecam-1*	Platelet endothelial cell adhesion molecule	Migration of leukocytes, formation of vessels, and integrin activation
3.	*Pentraxin -3*		Inflammatory mediator
4.	*ENDOGLIN*		Pro-angiogenic, protects endothelial cells and regulates NO-dependent vasodilatation
5.	*VEGF-A*	Vascular endothelial growth factor	Induces proliferation and migration of vascular endothelial cells
6	*Leptin*		Pro-inflammatory and promotes angiogenesis
7	*Resistin*		Increases expression of endothelin-1, MCP-I, cytokines and upregulates adhesion molecules
8	*Osteopontin*		Pro inflammatory cytokine, controls monocyte adhesion ,migration, survival, differentiation

### P363. Schoenlein-henoch purpura and enterorrhagia: is this a prelude to IBD?

#### M. C. Maggio, S. Accomando, G. Corsello

##### University Department PROMISE “G. D’Alessandro”, University of Palermo, Palermo, Italy

###### **Correspondence:** M. C. Maggio

**Introduction:** Severe gastrointestinal bleeding is rare but potentially life-threatening in patients with Henoch-Schönlein purpura (HSP). The management is not universally codified. Intra venous steroids are a first-line therapy. Nonsteroidal immunomodulatory treatment has been choosed to treat steroid-resistant patients.

**Objectives:** We report the case of a 4-year-old boy who presented acutely with life-threatening gastrointestinal haemorrhage to describe the difficult therapeutic choice to control the clinical presentation.

**Methods:** : a 4-year-old boy with fever, round worsening vasculitic lesions in the legs, gluteal region, auricle, associated with left gonarthritis. Skin lesions were typical of Seydlmayer purpura. The day after, he showed testicular pain, abdominal pain, positive occult blood, proteinuria. The diagnosis of HSP with renal and intestinal involvement was done. Infectious diseases were excluded. A low title p-ANCA antibodies was detected.

**Results:** He was treated with methylprednisolone (2 mg/Kg/day i.v.), parenteral nutrition and high dose of methylprednisolone (30 mg/Kg/day, for 3 days, followed by 4 mg/Kg/day), IVIG (2 gr/Kg) for a massive intestinal bleeding. After a partial improvement, he presented a further intestinal bleeding some days later. Mycophenolate mofetil (600 mg/m2) was started for the persistence of intestinal bleeding. The clinical manifestations resolved, and a polymeric diet was introduced. 4 months later, he stopped treatment and he still is in remission.

**Conclusion:** the control of the clinical manifestations followed the start of Mycophenolate mofetil and polymeric diet. The winning therapeutic choice was focused on Mycophenolate mofetil and polymeric diet. A multidisciplinary follow-up will support the patient, to exclude a bowel inflammatory disease.

**Disclosure of Interest**: None declared

### P364. Behçet syndrome in children and adults: discovering similarities and differences by a comparative study

#### M. V. Mastrolia^1^, A. Bettiol^2^, E. Marrani^1^, I. Maccora^1^, E. Taddei^2^, I. Pagnini^1^, M. Canfora^2^, G. Emmi^2^, E. Silvestri^2^, D. Prisco^2^, G. Simonini^1^

##### ^1^Rheumatology Unit, Meyer Children’s University Hospital, Neurofarba Department, University of Florence, ^2^Unit of Internal Interdisciplinary Medicine, Careggi University Hospital, Department of Experimental and Clinical Medicine, University of Florence, Florence, Italy

###### **Correspondence:** M. V. Mastrolia

**Introduction:** Behçet’s syndrome (BS) is a rare disorder with a relapsing-remitting course. Clinical variance across geographical regions and different age groups has been observed.

**Objectives:** To match demographic, clinical, treatment-related and outcome features of two cohorts of paediatric and adult BS patients.

**Methods:** Two clinical databases of BS patients were retrospectively compared. The paediatric BS database was collected at the Meyer Children’s Hospital, Florence, while the adult one at the Careggi University Hospital, Florence. Medical charts of BS patients were reviewed for age, gender, familiar and genetic predisposition (HLA-B51), clinical symptoms and treatments at onset and over the disease course. The fulfillment of the International Criteria for Behçet’s Disease (ICBD) criteria and/or of the International Study Group Criteria (ISG) for BS was assessed in both adult and paediatric groups, while the paediatric BD (PEDBD) classification criteria were applied only to the paediatric population. All patients met the ICBD and/or ISG and/or PEDBD (where applicable) at BS diagnosis or subsequently, during the whole medical history. In both cohort, disease activity at BS onset and at last available follow-up was assessed by the Behçet’s Disease Current Activity Form (BDCAF).

**Results:** Thirty-three paediatric patients diagnosed with BS, over a median time of 23 months (IQR 11-70 months) were included in the study. They were compared with a cohort of 165 adult BS patients, considered representative of the total BS adult population (n=340), followed over the same median time. In the paediatric cohort, BS onset had occurred at a median age of 12 (IQR 7.7-14.3) years, while in the adult cohort the mean age at symptoms beginning was of 36.6 (IQR 26.5-43.5) years. The female sex was significantly more represented in the adult cohort [105/165 (63.6%) vs 14/33 (42.4%), p=0.032]. A familiar predisposition was significantly more frequent in the paediatric cohort (3/33 vs 1/165, p=0.015). No difference emerged in terms of prevalence of HLA-B51 positivity. The proportion of patients meeting the revised ICBD and/or the ISG criteria at BS diagnosis was comparable. No significant difference emerged in terms of muco-cutaneous, ocular and neurological involvement, and gastrointestinal symptoms. Articular manifestations resulted more common in the paediatric cohort [14/33 (42.4%) vs 35/165 (21.2%), p=0.015], whereas venous vascular events were more frequent in the adult cohort [37/165 (22.4%) vs 2/33 (6.1%), p=0.031]. Regarding treatment strategy, paediatric patients more frequently received no treatment [14/33 (42.4%) vs 61/165 (37%) p<0.001] or corticosteroid monotherapy [17/33 (51.5%) vs 54/165 (32.7%) p<0.001]. Conversely, the use of DMARDs, both traditional and biologics, was significantly higher in the adult cohort [traditional DMARDs: 23/165 (13.9%) vs 1/33 (3%), biologic DMARDs: 86/165 (52.1%) vs 8/33 (24.2%) p<0.001].

**Conclusion:** Remarkable differences between juvenile-onset and adult-onset BS, both in terms of gender, familiar predisposition, and clinical manifestations have been observed and a different therapeutic approach in the real clinical practice of the two settings emerged. Prospective, comparison studies with a longer follow-up are encouraged to provide further data about the disease course for juvenile and adult-onset BS.

**Patient Consent:** Not applicable (there are no patient data)

**Disclosure of Interest**: None declared

### P365. Non-typhoid salmonella and Kawasaki disease: randomness or cause / effect?

#### G. Palamone ^1^, G. Macchini^2^, R. Caiazzo^2^, E. Alterio^1^, V. Tipo^2^, A. Mauro^3^

##### ^1^Pediatrics, Villa Malta Hospital, Sarno, ^2^Pediatrics, Santobono-Pausilipon Children’s Hospital, Naples, ^3^Rheumatology Unit, Fatbenefratelli Hospital, Milan, Italy

###### **Correspondence:** A. Mauro

**Introduction:** Kawasaki disease (MK) is an acute systemic vasculitis of small and medium caliber vessels. The etiology is unknown, probably multifactorial, the major complication of which, if not treated, is represented by aneurysms of the coronary arteries. Non-Typhoid Salmonellae are gram-negative bacteria that cause mild gastroenteritis and, more rarely, invasive forms almost exclusively in defected subjects

**Objectives:** We report the case of S. (age 6 months) hospitalized for persistent fever.

**Methods:** Seven days before entry, S. had presented with mucoematic diarrhea and fever lasting 4 days. Family members also had the same symptoms. After about 48 hours of apyrexia the little S. again manifested high fever for which he was hospitalized, showing a course of continuous fever (T Max 39.6 ° C) which was associated with oropharynx inflammation, tonsillar exudate, and then, to follow, laterocervical lymphadenitis, polymorphic skin rash on the trunk and limbs and finally bulbar conjunctivitis. In Kawasaki’s suspicion, he was practicing therapy with IvIg 2g / kg just over 72 hours after the onset of the fever. After 24 hours of therapy, he presented apyrexia and remission of conjunctivitis and polymorphic rash. In the meantime, the results of the I and II co-cultures that had identified an ESBL + strain of Salmonella Enteritidis, which were no longer detectable at subsequent culture controls, were received. Echocardiographic controls, both acute and follow-up, were all negative for coronary artery disease.

**Results:** Recent evidence in the literature correlates cases of NTS and Kawasaki disease, both as a “complicating”(1) coinfection, and as a potential risk factor or trigger of MK (2). Therefore, the temporal relationship between the episode of salmonellosis from probable domestic infection and the subsequent Kawasaki disease appears to be of considerable interest. It has also been suggested that there is a relationship between intestinal microbiome and MK (3): an alteration of the balance in the intestinal flora in conjunction with external infectious factors, could induce MK in genetically predisposed children.

**Conclusion:** In our patient, enteritis preceded the onset of the clinical picture which in a few days configured the diagnostic criteria of MK, and NTS therefore presented itself as a potential “trigger” agent of MK.

**Patient Consent:** Yes, I received consent

**Disclosure of Interest**: None declared

### P366. Plasma exchange therapeutic effect in refractory Kawasaki disease

#### H. F. Menchaca Aguayo, T. Tamayo Espinosa, E. Faugier Fuentes

##### Centro Médico ABC, Ciudad de Mexico, Mexico

###### **Correspondence:** H. F. Menchaca Aguayo

**Introduction:** -

**Objectives:** To describe the therapeutic effect of plasmapheresis in refractory Kawasaki disease.

**Methods:** Description of a clinical case and review of the literature.

**Results:** A 4-year-old female patient who presented with a 5-day history of persistent fever of 40 oC, abdominal pain, vomiting, diarrhea, maculopapular exanthema on the trunk and non-suppurative conjunctivitis of 5 days of evolution, was taken to the emergency room with signs of shock, vital signs: blood pression 84/40, heart rate: 160 T: 39°C Respiratory: 45. On physical examination, pale, decreased pulses and prolonged capillary filling, cervical lymphadenopathy greater than 2 cm is palpated, crystalloid solutions are administered, aminergic support and supplemental oxygen. Ceftriaxone antibiotic is started. Laboratory tests: normal blood count, renal and hepatic function preserved, hyponatremia, elevated D-dimer, CRP, troponins and proBNP; Ig G serology for SARS CoV-2 positive. Admitted to intensive care unit (ICU). Echocardiogram myocarditis and valvulitis. PIMS Kawasaki phenotype was diagnosed and gamma globulin was administered, methylprednisolone pulses 30 mgkgdo 5 doses, and acetylsalicylic acid 5 mgkg/day, he presented favorable clinical evolution and was discharged from ICU. Control echocardiogram at 7 days with coronary ectasia and increased troponins and proBNP, so a second dose of gamma globulin is administered and full dose continues, persists with elevation of acute phase reactants, new echocardiogram with appearance of aneurysms so infliximab 5 mgkgdo is indicated. Angio tomography at 12 days showed giant aneurysms in anterior descending coronary artery an right coronary artery, not extra coronary. Due to refractoriness, azathioprine 2 mg/kg per day and bolus cyclophosphamide bolus 750 mgm2sc, prophylactic enoxaparin, acetylsalicylic acid and statins were started. Echocardiogram on day 27, there is evidence of increased size of aneurysms so plasma exchange begins 5 sessions with subsequent echocardiogram where there is evidence of decreased size of the same.

**Conclusion:** Patient with a diagnosis of refractory Kawasaki disease, with presence of inflammation and increased size of aneurysms for which therapeutic alternatives were used infliximab, azathioprine, and cyclophosphamide, the latter being a medium vessel vasculitis. Given the refractoriness, plasma exchange was started, a therapeutic alternative used in Kawasaki disease and other autoimmune pathologies to stop inflammatory activity immediately, with which good results were obtained by stopping inflammation. The use of plasma exchange showed efficacy by decreasing the size of aneurysms.

**Disclosure of Interest**: None declared

### P367. Plasmapheresis. therapeutic effect in refractory Kawasaki disease. Case series

#### E. R. Mercedes-Pérez, P. P. Ramos-Tiñini, H. F. Menchaca-Aguayo, M. A. Balba-Aguilar, A. Guzmán-Revilla, K. Primero-Nieto, H. Bermudez-Canales, N. De la Rosa-Encarnación, M. I. De La Cera-Rodriguez, S. Rodriguez-Aguayo, E. Faugier-Fuentes

##### Hospital Infantil de Mexico Federico Gomez, Cuidad de Mexico, Mexico

###### **Correspondence:** E. R. Mercedes-Pérez

**Introduction:** Kawasaki disease (KD) is a vasculitis of unknown etiology and refractory in 10 to 20%. In these cases of refractory, management is controversial. Several therapeutic alternatives that have proven effective in other vasculitides have been used: Glucocorticoids, tumor necrosis factor (TNF) inhibitors, other immunosuppressive agents and plasmapheresis.

We present two cases of KD, refractory to two doses of IVIg, steroids, infliximab and cyclophosphamide, with aneurysm progression and successfully treated with plasmapheresis.

**Objectives:** To describe the therapeutic effect of plasmapheresis in refractory kawasaki disease.

**Methods:** Descrition of clinical case and review of the literature.

**Case 1.**Infant younger than 7 months, previously healthy, with a diagnosis of refractory Kawasaki. Harada 6 points. He did not respond to two doses of intravenous immunoglobulin, three boluses of methylprednisolone, and infliximab. Refractory to cyclophosphamide, azathioprine; in addition to a second regimen of 3 methylprednisolone boluses. Due to persistent inflammatory activity, elevated C-Reactive Protein, thrombocytosis, progressive increase in aneurysm size, treatment was escalated to 3 plasmapheresis sessions. The subsequent evolution was favourable.

**Case 2**.A 2-year-old male patient, previously healthy, with a diagnosis of refractory Kawasaki, did not respond to two doses of intravenous immunoglobulin and three boluses of methylprednisolone. Refractory to cyclophosphamide, azathioprine, and infliximab. However, due to persistence of inflammatory activity, elevated C-reactive protein, thrombocytosis and progressive increase in aneurysm size, treatment was escalated to 5 sessions of plasmapheresis with controls after the last session where negative reactants were reported, as well as a decrease in the Z score size of reported aneurysms.

**Results:** The therapeutic management in both cases was attached to the AHA 2017, SHARE 2019 and Japanese 2020 guidelines for refractory Kawasaki. Given the evidence of persistent inflammatory activity documented by a progressive increase in acute phase reactants and aneurysm size; It was decided to use azathioprine, cyclophosphamide because it is a medium caliber vasculitis and gives long-term therapeutic effect. Affection in other blood vessels was ruled out by CT angiography. To immediately stop the inflammatory effect, the use of plasmapheresis was decided. Obtaining satisfactory result. Individualized therapeutic decision made with the experience of the medical staff and accessibility of hospital resources. Situation referred to in the literature.

The therapeutic management in both cases was attached to the AHA 2017, SHARE 2019 and Japanese 2020 guidelines for refractory Kawasaki. Given the evidence of persistent inflammatory activity documented by a progressive increase in acute phase reactants and aneurysm size; It was decided to use azathioprine, cyclophosphamide because it is a medium caliber vasculitis and gives long-term therapeutic effect. Affection in other blood vessels was ruled out by CT angiography. To immediately stop the inflammatory effect, the use of plasmapheresis was decided. Obtaining satisfactory result. Individualized therapeutic decision made with the experience of the medical staff and accessibility of hospital resources. Situation referred to in the literature.

**Conclusion:** Plasmapheresis is one of the most effective therapies for the treatment of patients refractory to IVIG, when other additional therapies are not effective.

**Patient Consent:** Yes, I received consent

**Disclosure of Interest**: None declared

### P368. Infantile takayasu: clinical features and long-term outcome

#### A. Miller-Barmak^1,2^, F. Sztajnbok^3^, Z. Balik^4^, A. Borzutzky^5^, L. A. Fogel^6^, O. Goldzweig^7^, S. Ozen^4^, Y. Butbul Aviel^1,2,8^

##### ^1^Department of Pediatrics B, ^2^Pediatric Rheumatology Service, Ruth Rappaport Children’s Hospital, Rambam Health Care Campus, Haifa, Israel, ^3^Pediatric Rheumatology Division, Pedro Ernesto University Hospital, State of Rio de Janeiro University, Rio de Janeiro, Brazil, ^4^Department of Pediatric Rheumatology, Hacettepe University Faculty of Medicine, Ankara, Turkey, ^5^Department of Pediatric Infectious Diseases and Immunology, Pontificia Universidad Catolica de Chile, Santigao, Chile, ^6^Department of Pediatrics, Washington University in St. Louis, St. Louis, United States, ^7^Pediatric Rheumatology Service, Kaplan Medical Center, Rehovot, ^8^The Ruth and Bruce Rappaport Faculty of Medicine, Technion - Israel Institute of Technology, Haifa, Israel

###### **Correspondence:** A. Miller-Barmak

**Introduction:** Takayasu arteritis (TA) is a large vessel vasculitis rarely reported in children, with an extremely low incidence in infants. Thus, most articles on pediatric TA have not focused on infants. We present the largest case series of infantile TA.

**Objectives:** Identify demographic and clinical characteristics of infantile TA and compare them with existing data on older children.

**Methods:** We conducted an international multicenter retrospective cohort study. Patients with the diagnosis of TA at age <5 years were included in the study. Epidemiological and clinical data were collected from patients’ charts from six rheumatology centers. All patients met both the EULAR/PReS endorsed Ankara 2008 classification criteria and the 1990 ACR/EULAR criteria for TA.

**Results:** Twelve patients were included (50% female) meeting Median age of symptom onset was 11 months, with a diagnostic delay of 4 months, and median follow-up of 7.5 years. The most common symptoms at presentation were hypertension, Blood pressure differences between upper and lower limbs, and fever. The most commonly involved arteries at diagnosis were the abdominal aorta, renal artery, and superior mesenteric artery. Medications used included steroids, conventional and biological disease-modifying antirheumatic drugs, and other immunosuppressive therapies. Half of the patients received biologic agents of which infliximab had the highest complete remission rate (40%). Other medications resulting in complete remission were cyclophosphamide (40%) and methotrexate (38%). Invasive procedures were required for 58% of patients. The most common complications were cardiac (50%), stroke (42%), and serious infections (33%). No patients died.

**Conclusion:** This study presents the largest series of infantile TA. Compared to other reported series on older children, infants with TA have more severe disease where they were more likely to receive biologic agents, develop complications, and require invasive interventions.

**Disclosure of Interest**: None declared

### P369. infection and Kawasaki disease: an analytical study from North-India

#### S. Mondal, R. K. Pilania, A. K. Jindal, D. Suri, S. Singh

##### Pediatrics, Post Graduate Institute of Medical Education and Research, Chandigarh, India

###### **Correspondence:** S. Mondal

**Introduction:** Kawasaki disease (KD) is the most common vasculitis in children. The etiology of KD remains an enigma despite more than 50 years of extensive research. However, multiple lines of evidence have supported the role of infections as possible triggers for KD. It is believed that KD develops in a genetically susceptible host on exposure to an environmental agent that is most likely an infectious agent.

**Objectives:** Objective of this study is to report various infections identified in a cohort of patients with KD at the Advanced Pediatrics Centre, Postgraduate Institute of Medical Education and Research, Chandigarh, India.

**Methods:** We carried out a review of case records with KD during 1994-2019. Of 950 cases of KD during this period, 102 children had some evidence of infection during the course of their illness. This subgroup of patients was subsequently analyzed in detail.

**Results:** Overall 10.73% children had evident active infection during the course of KD. Of 102 children (67 boys), majority (68/102; 66.6%) was diagnosed as incomplete KD. Mean delay in diagnosis was 11 days (range 4-35 days). Only 1 sibling developed KD at the same time. Fever was present in all and periungual peeling was seen in 98 (96%%) patients. Microorganisms were isolated in 72 children (70.5%) that were bacteria (n=52); virus (n=15); fungus (n=4); protozoa (n=1). Superficial and deep-seated abscess was most common infective features (28/102; 27.45%). Other clinical features were pneumonia (n=27), gastrointestinal manifestations (n=26), shock (n=9), blood stream infection (n=5), urinary tract infection (n=4), splenomegaly (n=8) and arthritis (n=9, septic arthritis 2). Amongst the bacterial etiology, while staphylococcus and streptococcus were frequently encountered, dengue virus was commonest amongst viruses. Neutrophilic leukocytosis (86/102; 86.27%) was most commonly noted amongst supportive laboratory criteria followed by thrombocytosis (76.2%), anemia (62.5%), hypoalbuminemia (59.8%), hyponatremia (50%) and transaminitis (26.4%). Sterile pyuria was noted in 7 (5.83%) children. Ninety-six children were treated with first dose of IVIg (2gm/kg) and 5 required adjunctive therapy (2 received second dose of IVIg and 3 received both infliximab and steroids) along with low dose aspirin. Coronary artery abnormalities (CAAs) were seen in 12 (11.7%) patients during acute phase, which became normalized with in first 6 weeks in all patients. One child had myocarditis and succumbed to his illness.

**Conclusion:** Based on these results it may be concluded that approximately one tenth of all

children with KD may be associated with an infection in our set up and mostly it presents as incomplete KD. This infection may be a possible trigger for the disease. One should not exclude the diagnosis of KD even if there is evidence of infection.

**Patient Consent:** Yes, I received consent

**Disclosure of Interest**: None declared

### P370. Pediatric Behcet’s disease in india - a single center experience of 14 children

#### D. B. Pandya, M. Aggarwal, S. Sawhney

##### Pediatric & Adolescent Rheumatology Department, Sir Gangaram Hospital , New Delhi , India

###### **Correspondence:** D. B. Pandya

**Introduction:** Behçet’s disease is a rare variable vessel vasculitis characterised by recurrent oral ulcers , genital ulcers , arthritis , fever with systemic manifestations including ocular disease, skin lesions, gastrointestinal disease, neurologic disease and vascular disease. The disease is usually seen in young adulthood with a peak age of onset of 25–30 years, but it is also occasionally seen in children before the age of 16 years in 4–26% of cases.^1^ There is limited published data about the profile of Behcet’s disease in children seen at centers across India^.^

**Objectives:** We aim to look at the spectrum of behcet’s disease in Indian children at A tertiary level pediatric rheumatology center.

**Methods:** This is a retrospective analysis of fourteen children who were diagnosed with Behcet’s disease at our pediatric rheumatology center (PRC) , New Delhi , India between January 2013 to August 2021.Our collected data included demographic details , clinical presentation , management and follow up details.

**Results:** This table is showing characteristics of fourteen Indian children with Behcet’s Disease

**Conclusion:** Young age boys found to be affected more commonly. Recurrent oral ulceration remains the most common manifestation at onset . Systemic involvement can be depicted as MSK ~ Skin > Eye ~ GI > CNS > CVS ~ RS. Colchicine and methotrexate alone was not able to control disease in most of the patients. Azathioprine remains the drug of choice in our cohort . Thalidomide was found to be effective in some of our patients as an add on therapy. Adalimumab was effective especially in eye involvement. Most of the patients in our cohort are in remission as per their last follow up data.

**Trial registration identifying number:** 1. Karincaoglu Y, Borlu M, Toker SC *et al.* Demographic and clinical properties of juvenile-onset Behçet’s disease: A controlled multicenter study. *J Am Acad Dermatol* 2008

**Disclosure of Interest**: None declared

**Table 1 (abstract P370).** See text for description

Mean Age at onset	8.5 years (1.5-17)
Sex Ratio (M : F)	1.3 : 1
Average delay between disease onset and diagnosis	9 months
Fever at Onset	7 / 14 (50%)
Recurrent Oral Ulceration	13 / 14 (93%)
Genital ulceration	9 / 14 (64%)
Skin Lesions	7 / 14 (50%)
Musculoskeletal involvement (arthritis)	8 / 14 ( 57% )
Eye involvement	5 / 14 (35%)
Anterior Uveitis	3 / 5 (60%)
Intermediate Uveitis	1 / 5 (20%)
Pan Uveitis	2 / 5 (40%)
GI Involvement	5 / 14 (35%)
CNS	2 / 14 (14%)
CVS (pericardial effusion)	1 / 14 (7%)
RS ( bronchiectasis)	1 / 14 (7%)
Positive HLA B51	8 / 12 (67%)
Favourable response to medications used in our patients	
Colchicine	2 / 8 (25%)
Methotrexate	1 / 8 (12.5%)
Thalidomide	**2 / 5 (40%)**
Azathioprine	**4 / 7 (57%)**
Subcutaneous Adalimumab	**2 /3 (67%)**
Intravenous Tocilizumab	0 / 1 (0%)
Average number of flares on tapering medication	2
Inactive disease on last follow up	13 / 14 (93%)
Lost on follow up during CoVID-19 Pandemic	6 / 14 (43%)
Resistant Chronic Disease	1 / 14 ( 7% )

### P371. Clinical presentation and outcome of rare primary systemic vasculitides in 20 children - a single center experience from India

#### D. B. Pandya, M. Aggarwal, S. Sawhney

##### Pediatric & Adolescent Rheumatology Department, Sir Gangaram Hospital , New Delhi , India

###### **Correspondence:** D. B. Pandya

**Introduction:**

Acute primary vasculitides like IgA Vasculitis & Kawasaki Disease are common in pediatrics while the others described below are rare and their exact frequency is unknown. It includes mainly Polyarteritis nodosa , Takayasu Aortoarteritis , ANCA associated vasculitis and primary CNS angiitis.^1^

**Objectives:**

To study clinical presentation and outcome of rare primary systemic vasculitides in 20 children at our center.

**Methods:**

This is a retropsective analysis of 20 children with rare primary systemic vasculitides who were diagnosed and managed at our center between Aug 2013 to March 2022. Our collected data includes demographics , clinical presentation , management and follow up details.

**Results:**

Table is showing clinical presentation & outcome of 20 children with rare primary systemic vasculitides at our center

**Conclusion:** Females found to be affected more with rare primary vasculitides. Fever , constitutional symptoms , hypertension & elevated inflammatory markers remains the most common initial manifestations. Systemic involvement can be depicted as Gastrointestinal system > Renal > Skin > CNS > MSK > CVS > RS > Eye. Most children with severe systemic vasculitides responded to IV Cyclophosphamide as Induction agent & Oral Azathioprine as Maintenance agent. One patient with ANCA associated vasculitides responded to IV Rituximab as Induction & Maintenance agent. Three patients with Takayasu Aortoarteritis responded to IV Tocilizumab. Four patients with DADA-2 sydrome have been treated with TNF inhibitors but there was no adequate response. In these patients, there was a significant delay between initial presentation and final diagnosis of DADA-2 syndrome. Most patients are in remission off steroids. There are two deaths reported in our cohort and both these cases were diagnosed very late as DADA-2 syndrome with gastrointestinal perforation.

**Trial registration identifying number:** 1. Rheumatology (Oxford). 2019 04 01; 58(4):656-671. European consensus-based recommendations for the diagnosis and treatment of rare paediatric vasculitides - the SHARE initiative.

**Patient Consent:** Yes, I received consent

**Disclosure of Interest**: None declared

**Table 1 (abstract P371).** See text for description

Mean Age at Onset	8.2 years
Female	14
Males	6
Predominantly Large Vessel Vasculitis – Takayasu Aortoarteritis	6/20(30%)
Predominantly Medium Vessel Vasculitis – Polyarteritis Nodosa	8/20(40%)
(Includes Monogenic Vasculitis – DADA 2 syndrome)	6
Predominantly Small Vessel Vasculitis – Granulomatosis with Polyangiitis (GPA)	4/20(20%)
Isolated CNS Angiitis	2/20(10%)
Fever at Diagnosis	16/20(80%)
Constitutional symptoms including weight loss	14/20(70%)
Hypertension	12/20(60%)
Central Nervous System	
Stroke	5/20(25%)
PRES	5/20 (25%)
Peripheral neuropathy	6/20(30%)
Cranial Neuropathy	2/20(10%)
Neuropsychiatric Manifestations	1/20(5%)
Respiratory System	
ENT Disease	3/20(15%)
Diffuse alveolar haemorrhage	1/20(5%)
Pulmonary Hypertension	1/20(5%)
Cardiovascular system	
Left Ventricular Dysfunction	3/20(15%)
Coronary Aneurysms	1/20(5%)
Gastrointestinal system	
Severe abdominal pain	10/20(50%)
GI Ulcers	5/20 (25%)
GI Perforation	3/20(15%)
Renal System	
Perfusion or functional defect	8/20(40%)
Nephritis	3/20(15%)
Musculoskeletal system	
Myalgia or myositis	3/20(15%)
Arthralgia or arthritis	4/20(20%)
Skin involvement	
Livedo reticularis	6/20(30%)
Nodular skin lesions	4/20(20%)
Vasculitic Rash	4/20(20%)
Other Rash	2/20(10%)
Eye involvement	
Hypertensive Retinopathy	2/20(10%)
Scleritis	1/20(5%)
Ptosis	1/20(5%)
Elevated CRP +/- ESR at onset	14/20(70%)
Evidence of Vasculitis by	
MR Angiography	7/20(35%)
CT Angiography	8/20(40%)
PET	2/20(10%)
Biopsy	4/20(20%)
Response to cDMARD	
Intravenous Cyclophosphamide	9/12(75%)
Methotrexate	0/5(0%)
Oral Azathioprine	11/15(73%)
Oral MMF	1/3(33%)
Response to bDMARD	
Intravenous Rituximab	1/1(100%)
Subcutaneous Adalimumab	0/2(0%)
Intravenous Infliximab	0/2(0%)
Intravenous Tocilizumab	3/3(100%)
Follow Up Details	
Inactive disease off steroids	10/20(50%)
Inactive disease on minimum steroids	4/20(20%)
Persistent low disease activity	2/20(10%)
Persistent disease activity and damage	2/20(10%)
Death	2/20(10%)

### P372. Anticoagulation in children with Kawasaki disease and coronary artery aneurysms: our experience at chandigarh, North India

#### R. K. Pilania^1^, M. Singhal ^2^, S. Siniah^1^, S. Basu^1^, A. Thangraj ^1^, J. Ahluwalia^3^, S. Singh^1^

##### ^1^Pediatric Allergy immunology Unit , ^2^Department of Radiodiagnosis and Imaging , ^3^Department of Hematology, PGIMER, Chandigarh, India

###### **Correspondence:** R. K. Pilania

**Introduction:** Kawasaki disease is a common childhood vasculitis with special predilection for coronary arteries. Patients with large coronary aneurysms need long-term antiplatelet and anticoagulation therapy.

**Objectives:** To describe safety and efficacy of antiplatelet and anticoagulation therapy (aspirin and low molecular weight heparin (LMWH)/warfarin) in a cohort of KD patients with large coronary artery aneurysms.

**Methods:** Records of all children diagnosed to have KD during 1994-2021 were analyzed. Of the 1076 patients with KD, clinical details of children who had received aspirin and either LMWH/warfarin were retrieved.

**Results:** Thirty-eight (3.53%) children (28 boys; 10 girls) with KD, were put on aspirin and LMWH/warfarin. Median age of diagnosis was 18 months (range 1.5 months-12 years). Ten children (26.3%) below 1 year at diagnosis. Twenty-seven patients (71%) have received LMWH, while 18 (47.4%) received warfarin. Five patients initially received LMWH for period ranging from 12-31 months, followed by oral warfarin. Giant aneurysms were present in 35 patients while 3 patients had medium sized complex aneurysms. Thromboses developed in acute phase of disease in 4/38 (11.4%) and most common coronary artery affected was left anterior descending (LAD) coronary artery. All patients were continued on oral aspirin (3-5 mg/kg/day) along with anticoagulation therapy and 5 patients also received a second antiplatelet agent (clopidogrel). Median duration of LMWH was 14 months (range: 3-32 months), and median warfarin duration was 42 months (range: 2-126 months). In 18 patients we were able to monitor factor Xa activity and median activity was 0.46 IU/mL (0.32-0.81). Median INR in patients receiving warfarin was 1.55 (0.99-2.73). There were no significant complications related to anticoagulation in any of the patients, although parents frequently complained of local bruising. Serial 2D-echocardiogram during follow-up showed remodeling of coronary arteries. None of the patients developed thrombosis or symptomatic stenosis during follow-up. Duration of follow-up was 1414 patient-months.

**Conclusion:** Although the recommended INR in patients with KD and large aneurysm who are receiving anticoagulation therapy is 2-3, we maintained our patients on lower INR. Our results show that even on a much lower INR, these children have had no significant complication.

**Trial registration identifying number:** NOT APPLICABLE

**Patient Consent:** Yes, I received consent

**Disclosure of Interest**: None declared

### P373. Kawasaki disease: incidence figures at Chandigarh, India (2015-2019)

#### R. K. Pilania, R. Kumrah, S. Loganathan, M. Arora, D. Suri, S. Singh

##### Pediatric Allergy Immunology Unit , PGIMER, Chandigarh, India

###### **Correspondence:** R. K. Pilania

**Introduction:** There is paucity of literature on epidemiological data of Kawasaki disease (KD) from developing world.

**Objectives:** The aim of present study was to establish the current incidence of KD, seasonal variations and to assess incidence of coronary artery abnormalities (CAAs) in patients with KD from Chandigarh, India.

**Methods:** All children diagnosed with KD from Chandigarh UT (below the age of 15 years) during the period January 2015 to December 2019 were analyzed. Annual incidence rates were calculated on the basis of National Census data, 2011. The methodology was similar to previously published studies from our centre pertaining to the period 1994 - 2008 and 2009 - 2014.

**Results:** During the period 2015-2019, 79 patients (63 boys, 16 girls) were identified from Chandigarh, UT. Incidence rates varied during the 5 years period were 5.64, 9.25, 9.11, 9.87 and 9.72 /100,000 children below 5 in year 2015 - 2019 respectively. Maximum number of cases were observed in the year 2018 followed by 2019 while less cases were noted in 2015 below 5. While annual incidence varied between 2.65 in 2015 and 5.07/100,000 children in 2018 below 15. Comparison of yearly data revealed increasing incidence of disease from 11 cases (2009–2014) to 16 cases per 100,000 children below 15 cases/years from 2015-2019. There were clustering of cases during the months of April and September (10 each) with highest peak in these two months followed by August (9 cases) while nadir was seen in January and February. Mean age at diagnosis was 59 ± 43 months (median=48 months) ranging from 12 days - 15 years. There is increase in overall number of KD cases, as 2015-2019 Chandigarh incidence is higher as compared to our previous figures. 50% increase in annual incidence of KD in children below 5 and a 46.2% increase in children below 15 (from 2015-2019) as compared to our previous data from 2009-2014. CAAs during acute phase of illness was as high as 17.7%, while at 6 weeks of illness was reported in 7.6% of patients with KD despite treatment.

**Conclusion:** This study highlights that incidence of KD in Chandigarh has increased over period 2015-2019 compared to previous years. This may reflect true increase in the incidence of KD or may be due to increased ascertainment of disease as a result of increased awareness among pediatricians and physicians. Despite treatment CAAs have been reported in as high as 17.7% of patients with KD during the acute phase of disease.

**Trial registration identifying number:** Not applicable

**Patient Consent:** Not applicable (there are no patient data)

**Disclosure of Interest**: None declared

### P374. Epidemiology and clinical features of pediatric vasculitis: a single-center study

#### C. G. Morais^1^, T. Trindade^2^, A. Maia^1^, M. Rodrigues^3^, I. Brito^3^

##### ^1^Department of Pediatrics, Centro Hospitalar e Universitário São João, ^2^Faculty of Medicine of the University of Porto, ^3^Unidade de Reumatologia Pediátrica e Jovem Adulto, Centro Hospitalar e Universitário São João, Porto, Portugal

###### **Correspondence:** M. Rodrigues^3^

**Introduction:** Vasculitides are rare systemic conditions which may occur in childhood; pediatric studies done so far are few and have small sample sizes which limits the interpretation of results.

**Objectives:** This population-based study aims to develop knowledge regarding the clinical presentation of vasculitides in children.

**Methods:** In this retrospective study we gathered data regarding epidemiology and clinical presentation of all children diagnosed with vasculitides in a tertiary Portuguese center. All patients were assessed for demographics and clinical presenting symptoms.

**Results:** In our study, we included 138 patients. Kawasaki’s disease (KD) had the youngest average patients with a median age of 2.26 years old. In Behçet’s syndrome (BS) group patients were older (13.41). The gender ratio was only higher in females in BS’s patients (1:1.86), as opposed to other vasculitides. Cutaneous involvement was >90% in both IgA Vasculitis (IgAV) and KD. Gastrointestinal symptoms were common in all groups (15-50%); rarer in BS (17%). Arthritis and arthralgia were highly prevalent in IgAV (65%); ophthalmic involvement was frequent in KD (70%). Renal manifestations in IgAV (11%) and ophthalmic involvement in BS (22%) were lower than expected. There was a notable number of children reporting joint involvement in KD (27%). We also noticed a slightly higher prevalence of vascular findings in BS (30%).

**Conclusion:** Clinical symptoms observed in our patients were similar to previous reports in published studies, with some exceptions. The authors suggest that pediatric multicentric population-based studies are needed; such networks will be invaluable to facilitate research and clinical trials.

**Patient Consent:** Not applicable (there are no patient data)

**Disclosure of Interest**: None declared

### P375. Orbital tumor as an initial manifestation of anca-associated vasculitis: a series of three cases

#### S. Rodríguez Aguayo, H. Bermudez Canales, M. A. Barba Aguilar, K. Primero Nieto, A. Guzmán Revilla, E. Faugier Fuentes

##### Paediatric Rheumatology, Hospital Infantil de México Federico Gómez, Ciudad de México, Mexico

###### **Correspondence:** S. Rodríguez Aguayo

**Introduction:** Orbital tumor is a rare manifestation of ANCA-associated vasculitis, occurring in approximately 15% of patients with polyangiitis and 1% of patients with eosinophilic polyangiitis.

**Objectives:** To present 3 clinical cases of pediatric patients diagnosed with granulomatosis with polyangiitis with orbital tumor as initial presentation on the Hospital Infantil de México Federico Gómez.

**Methods:** Description of a clinical cases and review of the literature.

**Results:**

Case 1: an 8-year-old female who was treated for a 2-month-old right orbital tumor. She underwent a biopsy, reporting histopathologically nonspecific chronic inflammation and fibrosis. Within the approach, a renal biopsy was performed with diffuse endocapillary and extracapillary proliferative glomerulonephritis with cellular crescents, high resolution computed tomography of the lung with ground glass areas and pseudonodules, and positive p-ANCA and MPO antibodies.

Case 2: a 7-year-old female with a history of recurrent epistaxis. She was attended to for a 6-month-old right orbital tumor. The results of the lacrimal gland biopsy were consistent with small-vessel vasculitis with inflammatory infiltrate. High resolution computed tomography was reported with a pulmonary infiltrate, and positive p-ANCA and MPO antibodies.

Case 3: a 7-year-old female with a history of recurrent epistaxis. She was attended to for an orbital tumor, left ptosis, pain, and bilateral conjunctival injections for a month of evolution. The orbital computed tomography was reported with the presence of occupied lesions in both orbits. She underwent a biopsy, reporting histopathologically chronic inflammation and granulomatous reaction. In pulmonary high-resolution computed tomography was seen ground glass areas. IgG4 was negative, but p-ANCA and MPO were positive.

**Conclusion:** Orbital tumor is a rare ophthalmologic manifestation of ANCA-associated vasculitis. Its diagnosis and treatment continue to be a challenge. Clinicians should have a high index of suspicion in patients who present these clinical characteristics at the onset of the disease to carry out a systematized and exhaustive approach that allows us to make an early diagnosis and timely treatment.

**Patient Consent:** Yes, I received consent

**Disclosure of Interest**: None declared

### P376. Uveitis in Takayasu arteritis - a case report

#### M. C. Santos, F. D. Kuchiki, R. Tupinamba

##### Pediatric Rheumatology, Irmandade Da Santa Casa De Sao Paulo, São Paulo, Brazil

###### **Correspondence:** M. C. Santos

**Introduction:** Takayasu’s arteritis ( TA) is an arteritis granulomatous that affects the large arteries such as the aorta and its main branches and less frequently the pulmonary arteries; can lead to stenosis, occlusion, dilation and aneurysm formation. It mainly affects women in their 40s, but does not exclude other age groups. In the pediatric age group, 30% of cases were described in adolescents and less than 2% in children under 10 years of age. Its etiopathogenesis is still not well understood, but it is an autoimmune process and an association with infectious processes is observed. There are few reports of ocular involvement in Takayasu’s arteritis, which may be associated with events associated with the disease itself, due to hypoperfusion or to hypertension, may result from its treatment. Uveitis is no longer a frequent manifestation, but it can occur, affecting both the anterior and posterior segments of the eye, either acutely or chronically, transiently or permanently .

**Objectives:** To describe a case of a pediatric patient with Takayasu’s arteritis who presented ocular involvement as the initial manifestation.

**Methods:** Case report with obtaining clinical, laboratory, imaging and treatment data from the review of the patient’s chart followed up at the pediatric rheumatology center.

**Results:** A seven years old boy complaining of hyperemia in the left conjunctiva, was diagnosed with anterior uveiti , associated with the diagnosis of viral myocarditis and hypertension. In addition, due to abdominal pain, a USG of the abdomen was performed, showing lymph nodes in the hepatic hilum and retroperitoneum, without renal changes. Angiotomography of the abdomen initially showed a lymph node mass that determined a reduction in the caliber of the aorta, at the level of the kidneys, which remained patent. Cervical lymph node excision was also performed, with reactive lymphoid hyperplasia . He evolved with worsening of the ocular hyperemia in the left eye, in addition to arthritis of the wrists and ankles and subcutaneous nodules in the lower limbs and upper limbs, and he was referred to our service.

On admission, he had a galloping rhythm on cardiac auscultation with ocular hyperemia, mainly on the left; arthritis in wrists, left elbow and ankles, subcutaneous nodules in the extensor face of the arms and in the tibial extensor region, present and symmetrical pulses. Laboratory tests were performed which showed elevated acute phase tests. It was evaluated by ophthalmology, and biomicroscopy showed uveitis in the left eye (3+/4+ cells), *flare* (2+/4+), PK’s granulomatous . Low visual acuity , and topical treatment with corticosteroids and mydriatics was started , with improvement, allowing for an evaluation of the eye fundus examination, which showed no changes.

A new CT angiography showed significant adenomegaly and a hyperdense halo periaortic artery that suggested aortitis , in addition to aortic stenosis at the level of the renal arteries, not justified by lymph node compression , which associated with evidence of high inflammatory activity, arterial hypertension and difference in blood pressure between the limbs, allowed the diagnosis of Takayasu’s arteritis, treatment being started.

**Conclusion:** Takayasu arteritis can present ocular involvement, with different manifestations, resulting from the disease itself ( hypoperfusive , hypertensive or inflammatory condition) or from the treatment, which can be early or late.

Uveitis is not the most frequent ocular manifestation in TA, but it can occur and its early diagnosis allows for early treatment, avoiding serious complications. Thus, patients with AT should undergo routine ophthalmologic evaluation at the onset of the condition and during the course of the disease.

**Patient Consent:** Yes, I received consent

**Disclosure of Interest**: None declared

### P377. Severe cutaneous manifestations in IGA vasculitis are associated with a more severe clinical course: the experience from the largest international cohort of patients

#### M. Sestan^1^, N. Kifer^1^, B. Sozeri^2^, F. Demir^2^, K. Ulu^2^, C. A. Silva^3^, R. T. Campos^3^, E. D. Batu^4^, O. Koker^5^, M. Sapina^6^, S. Srsen^7^, A. Gagro^8^, A. Rodrigues Fonseca^9^, M. Rodrigues^9^, D. Rigante^10^, G. Filocamo^11^, F. Baldo^11^, M. Heshin-Bekenstein^12^, J. Kataja^13^, M. Held^1^, M. Frkovic^1^, S. Ozen^4^, M. Jelusic^1^ on behalf of PReS Vasculitis Working Party

##### ^1^University of Zagreb School of Medicine, UHC Zagreb, Zagreb, Croatia, ^2^University of Health Sciences, Umraniye Training and Research Hospital, Istanbul, Turkey, ^3^ICr-HC-FMUSP, Faculdade de Medicina da Universidade de São Paulo, São Paulo, Brazil, ^4^Hacettepe University Faculty of Medicine, Ankara, ^5^Marmara University-Pendik Training and Research Hospital, Istanbul, Turkey, ^6^University of Osijek, Medical Faculty Osijek, UHC Osijek, Osijek, ^7^University of Split School of Medicine, UHC Split, Split, ^8^University of Osijek, Medical Faculty Osijek; Children’s Hospital Zagreb, Zagreb, Croatia, ^9^Universidade Federal do Rio de Janeiro, Instituto de Puericultura e Pediatria Martagão Gesteira, Rio de Janeiro, Brazil, ^10^Department of Life Sciences and Public Health, Fondazione Policlinico Universitario A. Gemelli IRCCS; Università Cattolica Sacro Cuore, Rome, ^11^UOC Pediatria a Media Intensità di Cure, Clinica de Marchi, Milan, Italy, ^12^Dana Dwek Children’s Hospital, Tel Aviv Medical Center, Tel Aviv, Israel, ^13^Turku University Hospital, Turku, Finland

###### **Correspondence:** M. Sestan

**Introduction:** Although skin changes in IgA vasculitis (IgAV) are most commonly typical, in about 2% of children, the most severe changes may develop, including hemorrhagic vesicles, bullae, ulcerations or necroses.

**Objectives:** We investigated whether such changes were also associated with a more severe clinical course of the disease and the need for more intensive therapy.

**Methods:** The retrospective multinational study was conducted in 11 tertiary university medical centers. Patients were diagnosed according to the EULAR/PRES/PRINTO criteria. Data were analyzed descriptively and using the Fisher’s exact and χ2 test.

**Results:** A total of 64 patients with the most severe cutaneous manifestations in IgAV were included in the study, of which 41 (64.1%) were male, with a mean (standard deviation, SD) age of 8.49 (4.06) years at the disease onset. They were older than control group of 596 IgAV patients who did not develop bullae or necroses, with a mean (SD) age of 7.17 (3.57) years (p=0.006). The median (25-75p) time from the onset of the first symptom to the first bullous/necrotic change was 5 (2-10.5) days. The total duration of bullous/necrotic changes was 10 (7-16) days. The most important triggers of IgAV were infections, which were present in 68.8% of patients. The distribution of bullae and necroses followed the distribution of the purpuric changes. Scars and pigmentation changes persisted in 48.5% of children. The patients with severe cutaneous manifestations developed nephritis more frequently (40.6% vs. 20.6%, p=0.001), particularly with a combination of hematuria and proteinuria. The renal disease outcome was worse than the control group (p=0.001). They were more likely to have an affected gastrointestinal system (64.1% vs. 45.5%, p=0.007) and to develop the most severe gastrointestinal manifestations (p<0.001). The majority of these patients (90.6%) were treated: 84.4% of them received systemic glucocorticoids and methylprednisolone was the most frequently used with a median (25-75p) cumulative dose of 12 (6-88.7) mg/kg for a median (25-75p) of 12.5 (4-30) days, while 57.8% were treated with nonsteroidal anti-inflammatory drugs for 7 (3.5-18.5) days. Other drugs were administered sporadically. They were significantly more often treated with systemic glucocorticoids (84.4% vs. 37.2%, p < 0.001).

**Conclusion:** This is the largest international cohort study showing that IgAV patients with severe cutaneous manifestations developed nephritis with worse renal outcome more frequently compared to the controls, requiring systemic glucocorticoids. These patients also presented severe gastrointestinal involvement. Support: Croatian Science Foundation, IP-2019-04-8822.

**Patient Consent:** Yes, I received consent

**Disclosure of Interest**: None declared

### P378. Henoch schonlein purpura (HSP)- case series from newly established pediatric rheumatology cell in North-West India

#### A. Sharma

##### Paediatrics, Dr RPGMC Tanda, Dharamshala, India

###### **Correspondence:** A. Sharma

**Introduction:** Immunoglobulin A (IgA) vasculitis, previously known as Henoch Schönlein Purpura (HSP) is a common vasculitic disorder of childhood. Once considered the commonest vasculitis of childhood, may no longer be so. Nephritis is a potentially organ damaging complication that needs regular monitoring for early diagnosis and appropriate management. Relapses are known in IgA vasculitis.

**Objectives:** To study the epidemiological and clinical characteristics of patients diagnosed with IgA vasculitis at Pediatric Rheumatology Clinic (PRC) in Dr. Rajendra Prasad Government Medical College (Dr.RPGMC) Tanda, India, which is a state-funded, tertiary-care centre in the state of Himachal-Pradesh, India.

**Methods:** We analysed records of all patients with IgA vasculitis below 18 years enrolled in PRC at Dr.RPGMC Tanda from Jan 2018 to July 2021. We also compared the number of cases of Kawasaki Disease (KD) seen in PRC during this period.

**Results:** During the above mentioned period, 14 children were diagnosed with IgA vasculitis, out of which 9 were male (M:F= 1.8:1). Majority of cases (6/14, 43%) were aged between 11-15 years, 4 were between 6-10 years. Noe of the patients was below 2 years of age. Skin rash occurred in all 14 patients. Pain abdomen (57%) was the most common initial clinical presentation. Other symptoms being arthritis (3/14), blood in stools (1/14) and scrotal swelling (1/14). Among joint involvement, knee joint (83%) was commonly affected followed by ankle joint (50%), elbow joint (33%), wrist joint(33%) and small joints of hand (1%). Renal involvement occurred in 6 patients (43%). Manifestations of renal involvement were microscopic hematuria and sub-nephrotic albuminuria in all and hypertension (3/6). Mean age of those who had nephritis was 12.2 years as compared to 8.37 years of those without nephritis. Renal biopsy was performed in 2 of them. One, that of a 16 years girl, revealed crescentic glomerulonephritis and the other, that of a 14 years boy revealed mesangioproliferation. Pulse methylprednisolone was given to 4 of these and all were treated with oral gluco-corticoids. Angiotensin converting enzyme inhibitor (enalapril) was used in 3 and other treatment modalities used were immunosuppression with azathioprine and mycophenolate mofetil.

Interestingly during the same time period, 21 cases of Kawasaki Disease were seen in the PRC. Therefore the number of cases of Kawasaki Disease outnumber those with IgA vasculitis.

**Conclusion:** Mild disease resolves spontaneously while systemic steroids are recommended for moderate to severe disease including those with nephritis in IgA vasculitis. Prognosis depend on the extent of renal involvement. Close follow-up is required for early recognition of multiorgan involvement. Recognition of Kawasaki Disease is increasing in many parts of the world. Epidemiological studies are needed to prove that true incidence of Kawasaki Disease is increasing, especially in South-east Asian countries.

**Disclosure of Interest**: None declared

### P379. Withdrawn

### P380. Behcet disease presenting as superior vena cava syndrome

#### J. Sheekha^1^, A. Sharma^2^, D. Dhiman^1^

##### ^1^Pediatrics, ^2^Immunology and Pediatric Rheumatology, Dr Rajendra Prasad Governement Medical college, Tanda at Kangra, HP, Kangra, India

###### **Correspondence:** J. Sheekha

**Introduction:** Behçet’s disease (BD) is a multisystemic inflammatory disease. It is a variable vessel vasculitis, characterized by the involvement of any size and type (arteries, veins) of vessel.

**Objectives:** To report a case of BD who presented without characteristic features and had an unusual manifestation.

**Methods: Case report:** Eleven-year-old male child presented with swelling over the neck and face for 5 days which was insidious in onset and gradually progressive. The child also had 2 episodes of hemoptysis, 2 days back. In past history, 3 months back he had fever and cough with hemoptysis for one month and rash for 5 days. On evaluation at that time, he had severe thinness, hilar lymphadenopathy. Investigations revealed erythrocyte sedimentation rate (ESR) 120 mm in 1st hour, mantoux test and sputum cartridge based nucleic acid amplification test (CBNAAT) for *Mycobacteria* was negative. He was empirically initiated on anti-tubercular therapy (ATT) but according to parents, there was no improvement.

On presentation this time, he had tachypnea with tachycardia, swelling with prominent veins over the face, neck and chest. Fullness in supraclavicular and infraclavicular fossae. A clinical diagnosis of superior vena-cava (SVC) syndrome was made. On respiratory system examination, air entry was decreased in right superior axillary and mammary area. Ophthalmologic evaluation showed panuveitis.

**Results:** Investigations revealed, anemia (haemoglobin 7.6 gm/dl), thrombocytosis (platelets 4,97,000,cmm), elevated ESR (50 mm in 1st hour) and C-Reactive Protein (CRP) (48 mg/L). His coagulation profile and urine examination were normal. Chest X-ray showed homogenous opacity in left upper lobe. Computed tomography thorax showed aneurysm in main pulmonary artery with changes of fibrosing mediastinitis with mediastinal lymphadenopathy with venous thrombosis involving superior vena cava (SVC) and bilateral branchiocephalic trunks on either sides with no involvement of cervical vasculature. His antinuclear antibodies (ANA), anti-phospholipid antibodies (APLA) were negative and angiotensin converting enzyme (ACE) level was normal. With these clinical features, aneurysm in pulmonary artery with thrombosis with SVC syndrome, panuveitis, with elevated inflammatory parameters, possibility of Behcet disease was considered. His pathergy test was negative (on prednisolone). HLA B51 assay was planned. He was given topical betamethasone, pulse methylprednisolone (30 mg/kg/day) for 3 days followed by oral prednisolone (2 mg/kg/day). He was planned for injection infliximab but he had rupture of aneurysm and succumbed to the illness.

**Conclusion:** Classic features of oral and genital ulcers may not always be seen in children with BD. Pulmonary aneurysms are the most severe feature of pediatric BD. SVC syndrome is also a rare manifestation of BD.

**Patient Consent:** Yes, I received consent

**Disclosure of Interest**: None declared

### P383. Bronchoscopy may be a diagnostic tool in microscopic polyangiitis: case report and review of the literature

#### A. T. Suchi^1,2^, Y. Uziel^1,2^, A. Ziv^1,2^, T. Schoenfeld^3^, R. Haviv^1,2^

##### ^1^Sackler School of Medicine, tel aviv university, tel aviv, ^2^Pediatric rheumatology, ^3^Pediatric Pulmonology Unit, Meir Medical Center, Kfar Saba, Israel

###### **Correspondence:** A. T. Suchi

**Introduction:** Microscopic polyangiitis (MPA) is a vasculitis that affects both adult and pediatric populations, mostly involving the lower respiratory and renal systems. MPA is defined as a necrotizing vasculitis, with few or no immune deposits, predominantly affecting small vessels. Epidemiological data on MPA are limited, because of its origins as a subset of polyarteritis nodosa, and because it is now frequently described collectively with the other ANCA-associated vasculitis. MPA is very rare, in general and especially so in pediatric populations. The incidence in the UK was estimated at 3.6 cases per million and the median age of onset in children is 9 to 12 years.

There are no data regarding typical bronchoscopic findings in pediatric patients diagnosed with MPA.

**Objectives:** To report the unique bronchoscopy findings in conjunction with the clinical signs and symptoms, and laboratory test results of a female patient diagnosed with MPA, and to review the literature.

**Methods:** We collected data from the patient’s medical record and pictures taken during a bronchoscopic evaluation prior to the diagnosis. In addition, we searched databases and reviewed relevant articles.

**Results:** A 16-year-old girl was admitted to the Department of Pediatrics after 2 weeks of paroxysmal hemoptysis. She also reported weakness, 5 kg weight loss, arthralgia, night sweats and shivering.

Physical examination upon admission was generally unremarkable, with normal vital signs, and without any significant findings on chest auscultation.

Blood work-up at admission revealed mild leukocytosis and neutrophilia, mild normocytic anemia and mildly elevated inflammatory markers. Serum creatinine was mildly elevated.

Chest radiographs showed bilateral opacities and computed tomography showed ground-glass like opacities in the lower left lobe.

Bronchoscopy was performed and diffuse bronchial mucosal hemorrhage was reported (images will be presented on the poster). The abnormal appearance of the bronchial tree during the procedure prompted the pulmonologist to form an immediate working diagnosis of small-vessel vasculitis, strongly suggestive of MPA. The morning after the bronchoscopy, leukocyturia, erythrocyturia and proteinuria were reported and 24-hour urinary collection further revealed nephrotic range proteinuria. A renal biopsy was performed, and along with results of serologic tests (anti-myeloperoxidase and pANCA positivity), MPA was finally diagnosed. The patient was started on a course of corticosteroids and rituximab, and is now under remission.

Searching medical databases did not reveal any description of diffuse bronchial mucosal hemorrhage in MPA patients. However, diffuse alveolar hemorrhage is reported in patients 16–86 years of age. Limited data are available regarding bronchoscopic findings in MPA and bronchoscopy is not considered a diagnostic tool in this disease.

**Conclusion:** In this case, the diagnosis was based on the unique bronchoscopy findings obtained several days before receiving the renal biopsy and serology results.

As there are no relevant data in the literature, we propose that bronchoscopy might have diagnostic value for vasculitis, in patients presenting with hemoptysis and constitutional symptoms.

We recommend collaborating with other pediatric medical centers, that are willing to perform bronchoscopies and to the gross findings that may lead to another diagnostic tool for this very rare disease.

**Patient Consent:** Yes, I received consent

**Disclosure of Interest**: None declared

### P384. Pan coronary artery involvement in Kawasaki disease: a unique radiological entity on computed tomography coronary angiography

#### A. Thangaraj^1^, H. Chaudary^1^, R. K. Pilania^1^, M. Singhal^2^, A. Sharma^1^, S. Singh^1^

##### ^1^Pediatrics, ^2^Radiology, Post Graduate Institute of Medical Education and Research, Chandigarh-160012, India

###### **Correspondence:** A. Thangaraj

**Introduction:** Kawasaki disease (KD) is one of the most common childhood vasculitides. KD may result in coronary artery abnormalities (CAAs) in ~25% of untreated patients. Transthoracic 2-dimensional echocardiography (2DE) has been used as a standard of care for the diagnosis of CAAs patients with KD. Computed Tomography Coronary Angiography(CTCA) has shown to be superior in picking up a host of coronary artery aneurysms (CAAs) in KD patients that are missed on 2DE. CAAs involving all 3 coronary arteries along with their branches (pan coronary artery involvement-pan-CAAs) represent a severe spectrum of KD and have never been reported. In our observation, we have seen that a minority of patients with KD can have diffuse coronary artery involvement involving all segments of the right and left coronary circulation on CTCA.

**Objectives:** To study the prevalence of pan coronary artery aneurysms in our cohort and to analyze the difference in CTCA and 2DE in identifying pan coronary artery aneurysms

**Methods:** We have reviewed records of children who were diagnosed with KD and underwent CTCA for assessment of coronary artery dimensions between November 2013 to April 2021.CTCA was planned in children who had CAAs on 2DE. Patients with KD who had CAAs in all 3 coronary arteries (left coronary artery, left circumflex coronary artery and right coronary artery), but where the branches of these coronary arteries were spared were not included in this study.

**Results:** Two hundred fifteen patients with KD underwent CTCA between November 2013 to April[2021. Of these, 13 (6.04%) patients were noted to have pan-CAAs on CTCA. The mean age at diagnosis of KD in the patients was 7.4 years (range: 4 months-13 years). Eleven patients satisfied the complete criteria for KD, 2 patients were labelled as incomplete KD. Out of these 13 patients, 3 patients satisfied the clinical criteria for Multisystem inflammatory syndrome associated with coronavirus disease-2019 (MIS-C). All CTCAs were performed in the acute state. CAAs were in the form of aneurysms (saccular in 7, fusiform in 32). Non opacified segments suggesting thrombosis were noted in 4 coronary arteries. 2DE (performed within 48 hours of CTCA) demonstrated left main coronary artery (LMCA) abnormalities in 12(92.3%), proximal segments of left anterior descending (LAD) abnormality in 13(100%), right coronary artery (RCA) abnormalities in 11(84.6%) and left circumflex artery (LCX) abnormalities in 5(38.4%). Distal coronary artery aneurysms (5) and luminal thrombi (2) diagnosed on CTCA were entirely missed by 2DE. All CTCA were performed in the acute stage of KD. All children received an intensified therapy; twelve children received infliximab (5mg/kg) in addition to IVIg(2g/kg), high dose corticosteroids (10mg/kg for 3 doses) were given to intensify therapy in a child with MIS-C along with IVIg(2g/kg). All children received tapering doses of corticosteroids Aspirin (3-5mg/kg/day) is continued in all patients. Anticoagulants were added to patients with giant aneurysms and/or thrombosis.

**Conclusion:** Pan-CAAs in KD are a unique radiological entity and its better diagnosed by CTCA. Although 2DE is excellent in the diagnosis of CAAs in proximal coronary arteries, it is limited in identifying the distal extent of CAAs or distal CAAs and complications like thrombosis. We recommend that CTCA should be considered whenever significant CAAs are detected on 2DE as it provides information on the entire course of coronary arteries along with complications.

**Patient Consent:** Yes, I received consent

**Disclosure of Interest**: None declared

### P385. Interval CT coronary angiography in 11 children with Kawasaki disease: our experience at Chandigarh, North India

#### A. Thangaraj^1^, R. K. Pilania^1^, M. Singhal^2^, S. Mondal^1^, H. Chaudary^1^, A. Sharma^1^, A. Jindal^1^, S. Singh^1^

##### ^1^Pediatrics, ^2^Radiology, Post Graduate Institute of Medical Education and Research, Chandigarh-160012, India

###### **Correspondence:** A. Thangaraj

**Introduction:** Coronary artery abnormalities (CCAs) can occur in ≈25% of children with KD. Transthoracic 2D-Echocardiography (2DE), hitherto the imaging modality of choice, has several limitations as an imaging modality. Computed Tomography Coronary angiography (CTCA) has enabled the comprehensive evaluation of coronary arteries in children with KD. This study pertains to interval CT coronary angiography in 11 children with KD at a tertiary care centre in Chandigarh, India

**Objectives:** To analyse the long term consequences and prognosis of patient with coronary artery aneurysms

**Methods:** CTCA was carried out on 128-slice dual-source CT scanner (Siemens, Erlangen, Germany). There were 11 children in whom interval CTCA was performed.

**Results:** Median age at diagnosis of KD was 37 months [range 4-96 months]. Median interval between the first and second CTCA examination was 34.5 months [range 6-61 months]). Findings of CTCA at presentation revealed 21 aneurysms: left main coronary artery (LCA) – 5; left anterior descending artery (LAD) – 8; right coronary artery (RCA) – 4 and left circumflex artery (LCx) -4. Giant aneurysms were present in 6 patients (LAD – 5; RCA-3). Thrombosis was noted in 1 patient with giant aneurysm in LAD. Interval CTCA was completely normalized in 5/11 (45.4%) patients. Remaining 6 patients showed persistent (albeit regressed/remodelled) coronary artery aneurysms: LCA-5; LAD-4; RCA-3; LCx-2. Two patients had shown mural calcifications. Interval CTCA in 1 patient showed focal stenosis just beyond remodelled aneurysm in LAD. Two patients in whom CTCA was performed at intervals of 14 months and 72 months after diagnosis of KD, revealed long segment stenosis in LAD and ostioproximal segment of LCx.

**Conclusion:** Children with KD and CAAs require prospective long-term follow-up as they may develop complications like thrombosis, stenosis and calcifications. CTCA provides more detailed and comprehensive evaluation in comparison to 2DE.

**Patient Consent:** Yes, I received consent

**Disclosure of Interest**: None declared

## Poster session: Miscellaneous rheumatic diseases

### P386. Comparison of clinical characteristics and risk factors of recurrence in Kikuchi-Fujimoto disease between children and adult

#### J. G. Ahn^1^, J. Y. Baek^2^

##### ^1^Department of Pediatrics, Severance Children’s Hospital, Yonsei University College of Medicine, ^2^Department of Pediatrics, Severance Children’s Hospital, Yonsei University College of Medicine, Seoul, Korea, Republic Of

###### **Correspondence:** J. G. Ahn

**Introduction:** Kikuchi-Fujimoto disease (KFD) is a rare, benign and self-limited disease that is characterized by cervical lymphadenopathy and fever.

**Objectives:** We analyzed the differences in clinical manifestations and risk factors of recurrence between children and adults.

**Methods:** We retrospectively reviewed the medical records of patients who were diagnosed with KFD at a tertiary referral hospital between 2005 and 2019. We divided the patient groups into children (<19 years of age) and adults (≥19 years of age).

**Results:** During the 14-year study period, a total of 127 patients were diagnosed with KFD. Among them, 34 (26.8%) were children and 93 (73.2%) were adults. There were no clinically significant differences between children and adults except for a longer fever duration before diagnosis and higher frequency of myalgia in adults. For lymph node evaluation, ultrasound was mainly used in children (61.8%), whereas CT was most often used in adults (78.5%). Antibiotics were used more in children than in adults (76.5% vs. 54.8%, *P*=0.027). In adults, multivariable logistic regression analysis showed that ANA positivity (OR: 7.813; 95% CI=1.818–33.333; *P=*0.006) was a risk factor of recurrence.

**Conclusion:** KFD in children and adults showed similar clinical features, but showed differences in evaluation method and frequency of use of antibiotics. In adults, ANA positivity was associated with recurrence of KFD.

**Patient Consent:** Not applicable (there are no patient data)

**Disclosure of Interest**: None declared

### P387. The role of the paediatric rheumatologist in the management of children with Non-Specific Orbital Inflammation (NSOI): a single centre experience

#### H. Alkhdher^1^, O. Kul Cinar^1^, S. Gore ^2^, S. Compeyrot-Lacassagne^1,3^

##### ^1^Rheumatology Department, ^2^ Ophthalmology Department , ^3^Biomedical Research Centre, Great Ormond Street Hospital, London, United Kingdom

###### **Correspondence:** O. Kul Cinar

**Introduction:** NSOI is a term used to describe a heterogenous group of non-infectious, non-neoplastic disorders characterized by orbital inflammation with no identifiable local or systemic causes. Although rare in children, orbital inflammation may be associated with systemic inflammatory conditions; thus, careful evaluation and follow up is essential.

**Objectives:** We aimed to describe the clinical assessment and work-up of patients presenting with non-specific orbital inflammation, and to identify the role of the Paediatric Rheumatologist in the monitoring of extra-ocular involvement and the management of immunosuppressive treatment.

**Methods:** A retrospective descriptive case series of 15 children who had swelling of the lacrimal gland and a diagnosis of NSOI and were followed in the Paediatric Rheumatology and ophthalmology department at Great Ormond Street Hospital between September 2000 and March 2022.

**Results:** 15 patients, median age at referral of 12 years and 9 months (range 4 years and 9 months – 15 years and 11 months) were identified. Orbital imaging was performed in all 15 patients: CT orbit (1/15), MRI Orbit (11/15), both MRI and CT orbit (3/15). The most common manifestation on orbital imaging was lacrimal gland involvement in 87% (13/15), of which 46% (6/13) had bilateral involvement.

All 15 patients had an orbital biopsy with the following histopathological diagnoses: NSOI 87% (13/15), no significant pathology: 13% (2/15).

Ophthalmic symptoms were present in all 15 patients, with the most common being (eyelid swelling and/or erythema, pain, blurred vision). Around half of the patients 53% (8/15) reported extra-orbital symptoms/findings: 20% (3/15) abdominal pain, 13% (2/15) arthralgia, 7% (1/15) headaches, 7% (1/15) arthralgia with weight loss and malaise, 7% (1/15) right facial nerve palsy. An associated underlying diagnosis was found during follow up in 13% (2/15) patients: 1/15 Inflammatory bowel disease (IBD), 1/15 SRP positive necrotizing myositis with thyroiditis.

Work-up of all patients by the Paediatric Rheumatology team included a comprehensive history with a detailed system review and laboratory investigations including baseline blood tests with inflammatory markers (FBC, liver and renal functions, CRP, ESR) and an extensive autoimmune screen. All patients had a negative ANCA and 20% (3/15) were found to have a positive Antinuclear Antibody (ANA). ACE was raised in 27% (4/15). Faecal calprotectin was tested in 3/15 and was raised in 2.

The mainstay of treatment for NSOI is corticosteroids (CS) used in 80% (12/15). 3/15 did not receive CS, 2 underwent ophthalmic surgery and 1 was lost to follow up. 17% (2/12) achieved and maintained remission with a short course of steroids only, 8% (1/12) is awaiting intra-orbital CS injections and 75% 9/12 required disease-modifying antirheumatic drugs (DMARDS) with methotrexate being the most used in 67% (6/9), followed by mycophenolate mofetil in 33% (3/9). Whilst a short course of CS’s was effective in 75% (9/12) of patient, around 78% (7/9) relapsed when stopped. Anti-TNFα agents (infliximab and adalimumab) were used in 2 patients.

In our study, 13% (2/15) lost to follow-up, 60% (9/15) achieved remission and 27% (4/15) had recurrent flares. 50% (2/4) patients with on-going dacryoadenitis are awaiting intra-orbital CS injections.

**Conclusion:** Although NSOI is a rare entity in the paediatric population, careful clinical monitoring by a Paediatric Rheumatologist/Paediatrician is warranted to rule out underlying systemic inflammatory conditions. Treatment with short course of steroids is usually effective but recurrence is frequent requiring DMARDS or Anti-TNF agents.

**Patient Consent:** Not applicable (there are no patient data)

**Disclosure of Interest**: None declared

### P388. Successful treatment of Satayoshi syndrome with dantrolene: a case report

#### B. Arabshahi, C. Saldarriaga, A. Hoang

##### Pediatrics, Inova LJ Murphy Children’s Hospital , Fairfax, United States

###### **Correspondence:** B. Arabshahi

**Introduction:** Satoyoshi syndrome is a rare multisystemic disorder of unknown etiology, which presents with diarrhea, alopecia and musculoskeletal symptoms. This disease is often incapacitating, difficult to diagnose, and may lead to severe malnutrition and death if left untreated. There are very limited reports of Satayoshi Syndrome in literature and approaches to treatment have been variable and often unsuccessful.

**Objectives:** We present the case of a 15-year-old male with alopecia and severe progressive bilateral calf spasms, and assess response to treatment with dantrolene and systemic corticosteroids.

**Methods:** Our patient underwent extensive workup for neuromuscular diseases including MRI of the lumbar spine and lower extremities, EMG of bilateral calves, serum paraneoplastic autoantibody panel, inflammatory markers, muscle enzymes, and genetics evaluation -including metabolic myopathy panel and organic acids. Once all other causes were excluded, patient was diagnosed with Satayoshi Syndrome and started treatment with corticosteroids and dantrolene. Prednisone was initiated at 40mg daily and tapered off over the course of 6 weeks, and dantrolene was started at a dose of 25mg daily and titrated up to three times a day.

**Results:** Patient had full resolution of myalgia with no recurrence in muscle spasms on the above treatment regimen. One year post steroid discontinuation, patient remains asymptomatic and independent with his activities of daily living, and has had no recurrence in muscle spasms despite weaning dantrolene dose down to 25mg daily.

**Conclusion:** We present this case report of Satayoshi Syndrome to highlight the importance of prompt diagnosis and treatment of this debilitating and potentially fatal disease, and propose dantrolene as an effective steroid-sparing agent for management of this condition.

Written informed consent for publication of patient’s clinical details was obtained from the patient/guardian. A copy of the consent form is available for review.

**Patient Consent:** Yes, I received consent

**Disclosure of Interest**: None declared

### P389. Neonatal HLH : rare is not so rare

#### J. N. Bathia^1^, P. Pal ^1^, K. U. Ghosh^2^, P. Khemka^2^, R. Hassan^2^, S. Kabir^2^

##### ^1^Department of Pediatric Rheumatology, ^2^Department of Neonatology, Institute of Child Health, Kolkata, India

###### **Correspondence:** J. N. Bathia

**Introduction:** Hemophagocytic lymphohistocytosis (HLH), a rare clinical entity in neonatal period with an incidence of around 1 in 50,000-1,50,000 live births.

**Objectives:** We present a case series of 5 neonates who were diagnosed as neonatal HLH

**Methods:** We noted the data of five neonates admitted in the neonatal intensive care unit of Institute of Child Health, Kolkata during the last one year and were diagnosed as HLH.

**Results:** The five neonates presented to NICU at 7,11,15,20 and 27 days respectively with a mean age of 16 days. All five had fever, two of them had rashes and two had purpuric spots. Hepatosplenomegaly, bicytopenia, deranged liver function tests was seen in all. At diagnosis the mean hemoglobin was 7.54gm/dL, total leucocyte count 9 x10^3/^μL, platelet 26.4 x10^3/^μL , mean ferritin 10350 ng/ml, mean triglyceride 279mg/ dl. They were all initiated on dexamethasone and cyclosporine as per HLH 2004 protocol. in unresponsive cases methyl prednisolone was also used. Due to lack of response to dexamethasone and cyclosporine, one patient was given low dose etoposide on two occasions. Whole exome sequencing could be done in 3 of 5 patients. 2 showed PRF1 gene mutation, one homozygous and the other compound heterozygous, thus confirming the diagnosis of primary HLH however both of them succumbed. Two others in whom whole exome sequencing could not be done also succumbed to the illness. The fifth patient had no pathogenic variant on whole exome sequencing. This patient was diagnosed as secondary infection associated HLH and could be successfully discharged after appropriate treatment of the infection.

**Conclusion:** Neonatal HLH is a definite entity. Babies presenting with fever, multisystem involvement, cytopenias, rashes, hepatopathy should have an estimation of ferritin. and one must look beyond sepsis. Majority of the cases are primary. Genetic analysis, though expensive, should be offered. Secondary HLH has a good prognosis with timely management. Stem cell transplantation is the definitive treatment for primary HLH and is presently a viable option in many Indian centres. HLH is a definite entity in neonates and not so rare if looked for.

**Patient Consent:** Yes, I received consent

**Disclosure of Interest**: None declared

### P390. Empowering primary care providers to manage Benign Joint Hypermobility Syndrome (BJHS) with an educational packet

#### S. Brooks^1^, R. Carrasco^1^, K. Lewis^2^

##### ^1^Pediatric Rheumatology, Pediatric Rheumatology Consultants of Austin, ^2^St. David’s Medical Center Nursing Administratio, Austin, United States

###### **Correspondence:** S. Brooks

**Introduction:** Benign joint hypermobility syndrome (BJHS) is an under-diagnosed connective tissue disorder found in 7-36% of the pediatric population which can cause frequent joint injury, fatigue, and arthritis-like pains. Many BJHS patients present to pediatric rheumatology offices to rule out inflammatory arthropathy. There is no single sub-specialty that owns the diagnosis of BJHS in the United States, and therefore treatment and coordination of care (i.e., physical therapy) is primarily provided by primary care providers (PCPs). Diagnosis, treatment, and guidance for self-management of BJHS can be overwhelming for PCPs with lack of knowledge and confidence regarding the syndrome.

**Objectives:** The purpose of developing this educational packet is to provide succinct and necessary information regarding diagnosis and treatment of BJHS to empower PCPs to manage BJHS with less reliance upon subspecialists.

**Methods:** We utilized pediatric rheumatology, physiotherapy/physical therapy, occupational therapy, and pain management journals, textbooks, and resources to develop an evidenced-based educational packet for PCPs which addresses general overview of BJHS, diagnostic criteria, treatment plans, genetic testing information, and a physiotherapy home treatment guide developed by Ashford and St. Peter’s Hospitals. In the future, a feedback survey will be created and disseminated to PCPs who share a patient with a new diagnosis of BJHS. The survey will be completed prior to receiving the packet and measure the PCPs clinical confidence in managing BJHS. A secondary survey will be sent 3 days after receiving the packet and will measure the PCPs clinical confidence in managing BJHS after receiving the packet.

**Results:** The data regarding effectiveness of the developed packet has not yet been gained as this is a preliminary abstract. We anticipate that by distributing the BJHS educational packets, they will be used in the following ways. First, PCPs will utilize user friendly treatment plans which are outlined in the packet. Second, PCPs will coordinate with subspecialists, including physical therapy and occupational therapy, to provide care. Third, PCPs will practice utilizing diagnostic criteria for BJHS when presented with a patient who has joint pain without noted swelling on exam. We plan to seek if PCP confidence levels in managing BJHS significantly change prior to and post receiving and utilizing the BJHS packet.

**Conclusion:** A succinct yet comprehensive educational packet was developed to provide research and resources for primary care providers to manage the care of BJHS patients with less reliance on subspecialists. Improvement measures in clinical confidence of PCPs in managing BJHS after receiving the packet have not yet been conducted.

**Disclosure of Interest**: None declared

### P391. A girl with painful deformities, immune dysregulation and persistent inflammation

#### G. Capata^1^, S. Pastore^2^, A. Taddio^1,2^, A. Tesser^2^, V. Boz^1^, A. Tommasini^1,2^

##### ^1^Department of Medical, Surgical and Health Sciences, University of Trieste, ^2^Department of Pediatrics, IRCCS Burlo Garofolo, Trieste, Italy

###### **Correspondence:** A. Taddio

**Introduction:** Histiocytosis-Lymphadenopathy (HL) plus syndrome is caused by biallelic mutation of SLC29A3 gene encoding ENT3, a nucleoside transporter largely expressed on the outer mitochondrial membrane and lysosomal membrane of different cells, including monocytes/macrophages, glial cells, ocular and inner ear cells and epithelial cells. ENT3 plays a crucial role in maintaining intracellular homeostasis by transporting nucleotides to the cytoplasm. SLC29A3 mutation leads to the accumulation of nucleosides within the lysosomes and mitochondria resulting in stimulation of TLR7 especially in immune and glial cells. The spectrum of clinical features is characterized by multisystemic manifestations with variable expression and unclear pathogenesis. Features such as histiocytosis, recurrent fever, type 1 diabetes mellitus, thyroiditis and arthritis might be caused by both immune dysregulatory and autoinflammatory patterns. Pathogenesis of other manifestations such as exocrine pancreatic insufficiency, sensorineural hearing loss, hypertrichosis and skin hyperpigmentation is yet to be defined.

**Objectives:** To describe clinical and laboratory features and response to treatment in a girl with HL plus syndrome.

**Methods:** Clinical assessment was performed in a 21-year-old girl presenting to our Pediatric Rheumatology and Immunology Service with infancy-onset painful deformative contractions of hands and feet leading to progressive limitation in daily activities, bilateral sensorineural hearing loss, insulin-dependent type 1 diabetes, pancreatic insufficiency and hypothyroidism. At the age of 15 she underwent a genetic analysis that was diagnostic of HL plus syndrome due to the finding of compound heterozygous mutation in SLC29A3. Laboratory investigation included IFN score.

**Results:** At our evaluation, she complained severe walking difficulties due to painful deformities in her feet. Blood tests showed high inflammation indexes (ESR 47 mm/h, CRP 15.7 mg/L, IFN score 23.7). Hematological, cardiological and pneumological involvement were unremarkable. The association of deforming arthritis with predominant interferonic inflammation recalled Jaccoud arthropathy, a known clinical manifestation of adult SLE. On the other hand, recent studies have shown that IL-6 inhibition could be involved in controlling inflammatory phenomena in HL plus syndrome. Therefore, we started a treatment with baricitinib, a non-selective JAK inhibitor which can simultaneously counteract the effects of interferon and IL-6. At 6 month-follow-up visit, a minimal clinical improvement was reported with persistently active inflammatory indexes. Therefore, a treatment with hydroxychloroquine (HCQ) was added in order to obtain a synergic effect with baricitinib as HCQ acts as a lysosomal stabilizer by reducing the stimulation of TLRs. After 3 months, our patient reported such a significant clinical improvement that she was able to start a physiotherapeutic program and, for the first time in recent years, inflammation indexes were normalized.

**Conclusion:** To the best of our knowledge, this is the first case of HL plus syndrome treated with JAK inhibitors together with HCQ. We hypothesized that this treatment may have a great potential in this rare syndrome by targeting simultaneously IL-6 and IFN inflammation. Moreover, since the disease arises from the mutation of a lysosomal transporter, it may be interesting to disclose the potential benefit of HCQ as a lysosomal active medication. Longer follow-up and further studies are needed in order to confirm our hypothesis.

**Patient Consent:** Yes, I received consent

**Disclosure of Interest**: None declared

### P392. Adolescent with idiopathic myositis ossification in deltoid: a case report

#### N. Choon Seong

##### Rheumatology, Hospital Selayang, Selayang, Malaysia

###### **Correspondence:** N. Choon Seong

**Introduction:** Focal myositis belongs to rare type V idiopathic inflammatory myopathy. Though it is rare, prognosis is generally good.

**Objectives:** Herein, I presented a case of left deltoid focal myositis, of which responded well to nonsteroidal anti-inflammatory drugs.

**Methods:** Case Report

**Results:** A 17 years old girl presented with two months’ history of left arm myalgia without proximal muscle weakness. No history of trauma. No constitutional symptoms. No dysphagia. No symptoms suggestive of connective tissue disease. Physical examination revealed localized redness and tenderness over left deltoid but did not show any cutaneous signs of dermatomyositis. Inflammatory marker of erythrocyte sedimentation rate was elevated (17 mm/hr). Creatinine kinase level was normal. Antinuclear antibody, myositis panel, extractable nuclear antigen as well as culture for tuberculosis and fungal were negative. MRI showed diffuse short tau inversion recovery hyperintense signal over the left deltoid muscle. No evidence of enhancing mass and no collection. Muscle biopsy demonstrated inflammatory infiltrate in the perimysium and endomysium with presence of CD4+ T cells. No granuloma. Sarcoma was ruled out. A diagnosis of focal deltoid myositis was made. She was started on nonsteroidal anti-inflammatory drugs and so far no relapse was seen.

**Conclusion:** The current case has shown the importance of excluding differential diagnosis before reaching conclusion on such a rare entity. Second line agent may not be required in this case as the pain did not recur and there were no extra-skeletal manifestations.

**Trial registration identifying number:** -

**Patient Consent:** Yes, I received consent

**Disclosure of Interest**: None declared

### P393. With juvenile Sjogren’s syndrome followed in the pediatric rheumatology clinic clinical characteristics and follow-up of patient

#### T. Coşkuner^1^, B. Topatan^2^, B. Sözeri^1^

##### ^1^Pediatric Rheumatology, ^2^Pediatric Health and Disease, University of Health Sciences, Umraniye Training and Research Hospital, Istanbul, Turkey

###### **Correspondence:** B. Sözeri

**Introduction:** Juvenile Sjogren’s syndrome (JSS) is a chronic inflammation of exocrine glands such as salivary and lacrimal glands. This disease is very rare in childhood, and the onset clinic differs from that in adults. It is more common in girls than in boys (5:1-7:1), with an average onset of 10 years of age. There are no confirmed diagnostic criteria for the pediatric group yet, so diagnostic criteria published for adult patients in 2016 are used.

**Objectives:** The aim of this study is to evaluate the clinical findings, treatment options and treatment results of pediatric patients followed up with the diagnosis of JSS.

**Methods:** Seventeen pediatric patients (16 female patients) who were followed up in Ümraniye pediatric rheumatology clinic after 2016 and diagnosed according to 2016 ACR/EULAR criteria were included in the study. Patients with sicca symptoms that did not meet the diagnostic criteria were excluded from the study.

**Results:** The median age of diagnosis of the patients included in the study was 14 years and the mean age of symptom onset was 13 years. The most common symptoms were found to be dry mouth, musculoskeletal system complaints and dry eyes. Fever was seen in two patients, and Raynaud’s phenomenon in four patients. 18% of patients had another autoimmune disease with overlapping clinical features. There was one patient with a history of recurrent parotitis. While ANA positivity was detected in 88% of the patients, the titer was >1/160 in all of them, and a granular pattern was detected. Anti SSA /RO antibody positivity was observed in 76% of the patients. Only 2 patients had rheumatoid factor positivity. In 3 of 5 patients who underwent parotid ultrasonography, involvement compatible with parotitis was observed. Minor salivary gland biopsy was performed in all patients; while no feature was found in biopsy in 3 patients (17.6%), it was grade 2 in four patients, grade 3 in five patients, and grade 4 and above in five patients. Complaint and age at diagnosis were not found to be significant in terms of clinical and laboratory findings (p>0.05). While 23% of the patients required systemic steroid treatment, methotrexate and/or mycophenolatmofetil treatment was used in 52% of the patients. Rituximab treatment was used in 2 patients because of the persistence of clinical findings in one patient and the development of renal involvement in 1 patient.

**Conclusion:** Sjogren’s syndrome progresses with various systemic findings in addition to dry mouth and eyes in pediatric patients as in adults. There may be a need for immunosuppressive therapy.

**Disclosure of Interest**: None declared

### P394. Importance of nursing consultation to adolescents with chronic rheumatological disease: pilot study

#### J. C. O. A. Ferreira^1^, A. A. Costa^2^, A. C. S. Monteiro^2^, H. S. Robba^2^, R. C. D. Vaz^1^

##### ^1^Clinical Research Center, ^2^Nursing Division, Instituto Da CRIANÇA DO HCFMUSP, São Paulo, Brazil

###### **Correspondence:** J. C. O. A. Ferreira

**Introduction:** The care of adolescents with chronic rheumatologic disease must include all the care demands of this phase marked by the need to consolidate identity, develop body image, establish social relationships and achieve independence. The nursing consultation contributes to the identification of difficulties faced by adolescents, reveals points of improvement in care and impacts on adherence to treatment.

**Objectives:** To characterize the profile of adolescents with chronic rheumatologic disease; To highlight the importance of nursing consultations for adolescents with chronic rheumatologic disease and To identify points of improvement in patient care.

**Methods:** Cross-sectional pilot study with adolescents with rheumatologic disease. Instruments were used to characterize the population studied, including sociodemographic and anthropometric data, vital signs, pain, drug addiction and adherence. Approval by the local Ethics Committee number 3.163.835. The parents and adolescents provided written informed consent.

**Results:** Partial results: initial data from thirteen adolescents revealed considerable dependence on parents/guardians, especially regarding medication. Attending the adolescent alone in part of the consultation was essential to identify problems such as bullying and depression, medication adherence and resistance to the use of sunscreen.

**Table:** 

Variables	Adolescents N = 13 (%)
**Sociodemographic data**	
Female gender	9 (69)
Vaccination card updated	11 (85)
Remission disease	11 (85)
Previous hospitalization	10 (77)
Appointments schedule	9 (69)
Consultation with patient accompanied	12 (92)
Physical activity	7 (54)
**Findings in the consultation with the adolescent**	
Domestic violence	1 (8)
Bullying	6 (46)
Depression	2 (15)
Suicidal ideation	4 (31 )
Live well with the disease	10 (77)
Pain	6 (46)
**CRAFFT Screening Questions**	
Did you drink alcohol (more than a few sips)?	2 (15)
Have you ever been in a CAR driven by someone who was “high” or had been drinking alcohol or using drugs?	3 (23)
**Medication Compliance (Morisky and Green Test)**	
Do you ever forget to take your medicine?	8 (62)
Are you sometimes careless about the timing of your medication?	8 (62)
When you are feeling well, do you ever stop taking your medicine?	2 (15)
When you feel bad about your medication, do you sometimes stop taking it?	2 (15)
**PedsQL**^**TM**^**4.0**	
I worry about what will happen to me	8 (62)

**Conclusion:** This work, although hampered due COVID-19 pandemic that caused the interruption of study, has already shown the need for nursing consultations for adolescents with specific instruments that allow diagnosing possible problems, understanding concerns and getting to know this clientele. The continuation of the project will use the results to develop a practical guide for the care of adolescents with rheumatologic disease.

**Patient Consent:** Yes, I received consent

**Disclosure of Interest**: None declared

### P395. Juvenile Sjogren’s syndrome: the need for validated paediatric diagnostic criteria. a retrospective review from a tertiary rheumatology centre

#### C. Foley^1^, D. McKenna^1^, K. Gallagher^1^, C. Ciurtin^2^, M. Al Obaidi^1,3^

##### ^1^Great Ormond Street Hospital, ^2^Centre for Adolescent Aheumatology Versus Arthritis Department of Medicine, University College London (UCL), ^3^NIHR Biomedical Research Centre GOS Institute of Child Health, GOSH NHS Foundation Trust, London, United Kingdom

###### **Correspondence:** C. Foley

**Introduction:** Sjögren’s syndrome (SS) is a chronic multisystem autoimmune disease, characterised by inflammation of the exocrine glands, predominantly salivary and lacrimal glands resulting in xerostomia and xerophthalmia.

Juvenile-onset SS (jSS) is rare; however, it is probably underrecognised and underdiagnosed. There are no studies reporting accurate incidence or prevalence of jSS.

Diagnosing jSS can be challenging. The cardinal signs and symptoms are non-specific. There is no gold standard biomarker of disease. Between 1965–2002 11 diagnostic criteria were developed, none of which have gained universal acceptance or been validated in a paediatric population. Until recently, the most widely used criteria were those developed by the American-European Consensus Group (AECG).

It remains well recognised that international consensus on classification is important for standardisation, particularly in relation to research and monitoring treatment outcomes. However, there remains a paucity of validated criteria for diagnosis of jSS.

Paediatric focussed criteria are required as features of SS in children differ from those in adults. Children experience less dryness and more frequently experience systemic symptoms and parotid enlargement. Applying adult criteria to a paediatric population may lead to mis- and/or under-diagnosis. Bartunkova *et al.* (1999) proposed paediatric criteria for diagnosis of jSS. These have not been validated.

**Objectives:** Describe clinical, laboratory and imaging characteristics of children diagnosed with jSS at Great Ormond Street Hospital (GOSH) over a 10-year period

Apply the AECG and proposed paediatric criteria to our cohort and report percentage fulfilling each criteria

**Methods:** Retrospective chart review of children diagnosed with jSS, January 2012-January 2022 at GOSH. Demographic, clinical, laboratory and imaging findings were documented. Features at presentation were compared to the AECG and proposed paediatric diagnostic criteria. Percentage of the cohort fulfilling each criteria was calculated.

**Results:** 28 cases of jSS were diagnosed from January 2012-January 2022, 22 female (79%). Referring clinicians to Paediatric Rheumatology included Paediatricians (n=21,75%), Ophthalmologists (n=1,3.6%), Rheumatologists (n=1,3.6%), Dermatologists (n=1, 3.6%), Nephrologists (n=2,7.1%) and Oral surgeons (n=2,7.1%). Main reasons for referral included facial swelling, lymphadenopathy, rash, constitutional symptoms, and positive investigation findings suggestive of jSS i.e. autoantibody profile, US salivary glands. Median age at diagnosis was 11 years (3.4-14.8).

The most common presenting feature was facial swelling (n-18,64%), followed by lethargy (n=12,43%) and rash (n=11,39%). Reports of dry eyes (n=7,25%) and mouth (n=9,32%) were reported less frequently. Laboratory features identified included anaemia, elevated amylase and ESR, positive ANA and Ro, and hyper IgG. All children who had an USS of their salivary glands had features consistent with a diagnosis of jSS (n=24,86%). 11 children had salivary gland biopsy, 10 (91%) of which provided tissue confirmation of the jSS diagnosis. A total of 8/28 (28.6%) cases satisfied the AECG criteria; 22/28 (78.6%) satisfied the proposed paediatric diagnostic criteria.

**Conclusion:** A low percentage of our cohort fulfilled the AECG diagnostic criteria, therefore we would suggest application of adult criteria for diagnosis of jSS maybe unhelpful. Inclusion of recurrent parotitis and additional laboratory features in the proposed paediatric criteria may increase sensitivity. Our cohort highlights the need for validated jSS diagnostic criteria as paediatric and adult presentations of SS can differ.

**Patient Consent:** Not applicable (there are no patient data)

**Disclosure of Interest**: None declared

### P396. A 9-year-old girl with persistent back pain and erythema nodosum

#### M. F. Gicchino, S. Colosimo, M. Bartiromo, A. Amodio, A. N. Olivieri

##### Department of Woman, Child and General and Specialistic Surgery, University of the Study of Campania Luigi Vanvitelli, Naples, Italy

###### **Correspondence:** M. F. Gicchino

**Introduction:** Back pain is a relatively common presenting symptom in children and adolescents. Typical causes include spinal deformities (such as scoliosis), spondylolysis, bulging or herniated intervertebral disks, functional pain (such as fibromyalgia), spondylodiscitis, post-traumatic injuries, benign or malignant tumors, osteomyelitis, juvenile idiopathic arthritis. Erythema nodosum (EN) is the most common panniculitis in childhood. EN lesions are localized at lower limbs in particular on the pretibial region, upper limbs and trunk are rarely involved. EN can be associated with general symptoms such as fever, weakness, sever pain, but skin lesions resolve without skin damage. EN can be associated with infectious diseases (beta-hemolytic Streptococcus, Chlamydia and Mycoplasma Pneumoniae, Epstein-Barr virus, Mycobacterium Tuberculosis), inflammatory bowel diseases, coeliac disease, rheumatologic diseases, malignant tumors.

**Objectives:** To report the case of a 9-year-old patient with back pain and erythema nodosum. To report the case of a 9-year-old patient with back pain.

**Methods:** A 9-year-old girl comes to our Department because of back pain and asthenia. Two weeks before the back pain onset the patient got a trauma due to a fall from a swing. Then the patient presented fever (with a temperature up to 38 ° Celsius) for 2-3 days, associated with epistaxis, asthenia and lumbar pain. At first these symptoms were treated with non steroidal antinflammatory drugs (NSAIDs). Since symptoms got worse she underwent to an orthopedic evaluation that found scoliosis in dorsal and lumbar column and bilateral valgus foot. For the persistence of both back pain and asthenia the patient came to our Department. Our clinical examination revealed: pain in lumbosacral column and on the pressure of the right sacroiliac joint; erythema nodosum on the pretibial region of the left lower limb.

**Results:** Complete blood count, liver and kidney function, iron levels was in the norm. Both Antinuclear antibodies and ENA were negative. Virological screening (including Sars-Cov2) was negative. Thyroid profile was in the norm and coeliac disease screening was negative. Inflammatory parameters were slight increased: erythrocyte sedimentation rate (ERS) was 39mm/h (normal value<15mm/h); C-reactive protein (CRP) was 3,7mg/dL (normal value<1mg/dL). Fecal calprotectin was in the normal range. Throat swab was negative. Chest x-ray, magnetic resonance imaging (MRI) of the lumbosacral column and sacroiliac joints, mantoux intradermal reaction and quantiFERON test were also performed. Chest x**-** ray was in the norm. MRI revealed: reduction of physiological lordosis in supine position; increased representation of intracanal epidural tissue from L4 region to the sacrum; fluid effusion in correspondence of the right sacro-iliac joint. Mantoux intradermal reaction was positive at 48 hours, with a diameter of 25 millimeters. QuantiFERON test was positive.

**Conclusion:** According to clinical features, laboratory and radiological findings Tubercular sacroileitis was diagnosed. Patient was entrusted to the care of our colleagues expert in infectious disease and treatment with ethambutol, pyrazinamide, rifampicin and isoniazid was started. A follow up spine MRI performed two months later was in the norm. In a child presenting with back pain also uncommon causes should be considered such as tubercular infection, so it is very important an accurate and complete medical examinations, in fact in the case described the detection of erythema nodusum on the left lower limb made us think about a possible tubercular infection.

**Patient Consent:** Yes, I received consent

**Disclosure of Interest**: None declared

### P397. A 3-year-old child with persistent fever

#### M. F. Gicchino, A. Amodio, F. G. Abbate, A. N. Olivieri

##### Department of Woman, Child and General and Specialistic Surgery, University of the Study of Campania Luigi Vanvitelli, Naples, Italy

###### **Correspondence:** M. F. Gicchino

**Introduction:** Fever is a common symptom of many clinical conditions, infection is the most common cause especially in children. Fever of unknown origin (FUO) is defined as a well-documented fever of at least three weeks’ duration without an apparent source after 1 week of investigation. FUO is a challenging problem in clinical practice, and patients presenting with FUO often undergo to unnecessary laboratory tests and antimicrobial therapies. Among children the most common causes of FUO are: infectious diseases, malignancy, autoimmune diseases, immunodeficit, Kawasaki disease, autoinflammatory syndromes.

**Objectives:** To describe a case of FUO in a 3 years old child.

**Methods:** A 3-year-old child came to our department for episodes of recurrent nocturnal fever for about 8 months (Temperature was up to 39 °Celsius). Fever was treated with antibiotics and non steroidal anti-inflammatory drugs as prescribed by her pediatrician. Psoriasis was diagnosed at the age of two years. On physical examination we found: erythematous and desquamated plaques on the scalp and on the left retro auricular region, and lymphadenitis in left latero-cervical region. We did not find any sign of arthritis and/or enthesitis.

Laboratory tests revealed: microcytic anemia (Hemoglobin 9,5 mg/dL; Mean Corpuscular Volume 68) elevation of platelets (689/mm^3^, normal value <450/mm^3^), erythrocyte sedimentation rate (ESR) (25mm / h, normal value <20 mm/h), and C-reactive protein (CRP) (4mg / dL , normal value <1mg/dL) and serum amyloid A (SAA) (80 mg/L, normal value <6.4mg/L). Liver and kidney function, iron levels, C3/C4 levels, LDH , blood coagulation profile, lymphocyte subpopulations were in the norm. Both Antinuclear antibodies and ENA were negative. Virological screening (including Sars-Cov2) and QuantiFERON test were negative. Thyroid profile was in the norm and coeliac disease screening was negative.

Both blood and urine cultures were negative, fecal calprotectin was in the normal range. Both heart and abdomen ultrasound were in the norm. The dosage of vanillylmandelic acid in the 24-hour urine was negative, so a neuroblastoma was ruled out. Bone marrow aspirate did not reveal any blastic cells. The patient finally underwent skin biopsy of the erythematous-desquamative lesions.

**Results:** Skin biopsy revealed **:** a widespread infiltrate in papillary dermis region that was composed of medium-large cells with round-oval nuclei and some incisions. Some eosinophilic granulocytes were found among cell population. A huge area of microulceration was found on epidermis. Immunoassayed sections with CD1 A, S100 and CD68 were positive. So, the morphological and phenotypic findings were compatible with Langerhans cell histiocytosis (LCH).

**Conclusion:** According to clinical condition and histopathological finding a Langerhans cell histiocytosis was diagnosed.

LCH is a rare condition in childhood characterized by the proliferation and accumulation of a particular kind of immune system cells called Langerhans cells or histiocytes . This disease could affect any organ or system and the most affected sites in children are bones and skin. When disease involves the skin LCH most commonly presents as a seborrheic dermatitis or eczematous eruption on the scalp or trunk.

The clinical presentation is quite heterogeneous, ranging from an isolated and asymptomatic skin or bone lesion to a life threating multisystem disease, characterized by fever, skin rash , anemia, thrombocytopenia, lymphadenopathy and hepatosplenomegaly. Prognosis depends on involvement of risk organs (liver, spleen, bone marrow) at diagnosis and particularly on presence of organ dysfunction and response to initial therapy. Treatment is systemic, incorporating steroids and cytostatic drugs.

**Patient Consent:** Yes, I received consent

**Disclosure of Interest**: None declared

### P398. Retrospective evaluation of pediatric sarcoidosis patients

#### V. Guliyeva^1^, F. G. demirkan^2^, R. E. Yiğit^3^, E. Esen^4^, Y. Bayındır^5^, R. Torun^6^, D. Gezgin Yıldırım^7^, G. Kılbas^8^, G. Otar Yener^9^, M. Cakan^10^, F. Demir^11^, K. Özturk^12^, E. Baglan^13^, B. Bora Makay^14^, S. A. Bakkaloglu Ezgü^15^, A. Pac Kısaaslan^16^, Y. Bilginer^17^, S. Yuksel^8^, S. Ozen^18^, B. Sozeri^19^, N. Aktay Ayaz^1^

##### ^1^Pediatric Rheumatology, Faculty of Medicine, Istanbul University, Istanbul, Turkey., ^2^İstanbul School of Medicine, ^3^Department of Pediatric Rheumatology, Ümraniye Research and Training Hospital, University of Health Science, Istanbul, Turkey., İstanbul, ^4^Department of Pediatric Rheumatology, Erciyes University School of Medicine, Kayseri, ^5^Division of Pediatric Rheumatology, Department of Pediatrics, Hacettepe University, Ankara, Turkey, ankara, ^6^Divisions of Pediatric Rheumatology, Dokuz Eylül University Faculty of Medicine, İzmir, Turkey, izmir, ^7^Department of Pediatric Rheumatology, Gazi University Faculty of Medicine, Ankara, Turkey., ankara, ^8^Pediatric Rheumatology, Faculty of Medicine, Pamukkale University, Denizli, Turkey., Denizli, ^9^Department of Pediatric Rheumatology, Sanliurfa Training and Research Hospital, Sanliurfa, Turkey, Sanliurfa, ^10^Department of Pediatric Rheumatology, University of Health Science, Umraniye Training and Research Hospital, Istanbul, Turkey., ^11^acibadem hospital pediatric rheumatology department, ^12^Pediatric Rheumatology, Istanbul Medeniyet University, Göztepe Prof. Dr. Süleyman Yalçın City Hospital, Istanbul, Turkey., İstanbul, ^13^Pediatric Rheumatology, University of Health Sciences, Dr. Sami Ulus Maternity and Child Health and Diseases Research and Training Hospital, Ankara, Turkey., ankara, ^14^Department of Pediatrics, Division of Rheumatology, Dokuz Eylül University Faculty of Medicine, İzmir, Turkey., izmir, ^15^Department of Paediatric Rheumatology, Faculty of Medicine, Gazi University, Ankara, Turkey., ankara, ^16^Department of Pediatric Rheumatology, Erciyes University School of Medicine, Kayseri, Turkey., Kayseri, ^17^Department of Pediatrics, division of Pediatric Rheumatology, Faculty of Medicine, Hacettepe University, Ankara, Turkey., ^18^Pediatric Rheumatology, Faculty of Medicine, Hacettepe University, Ankara, Turkey., ankara, ^19^Pediatric Rheumatology, University of Health Sciences, Ümraniye Research and Training Hospital, Istanbul, Turkey., İstanbul, Turkey

###### **Correspondence:** V. Guliyeva

**Introduction:** Sarcoidosis is a very rare granulomatous condition among childhood rheumatic diseases. It may affect various organ systems resulting in different clinical presentations in pediatric patients.

**Objectives:** we aimed to evaluate the demographic and clinical characteristics of pediatric sarcoidosis patients and treatments used.

**Methods:** Pediatric patients diagnosed with sarcoidosis in 12 centers between 2011-2021 were included in the study. Patient records were retrieved from the Academy of Pediatric Rheumatology (PeRA)-research group (RG) database. Demographic features and clinical spectrum were evaluated retrospectively.

**Results:** The mean age of 12.3±4.6 years and 33 (63.5%) of them were females. Demographics are outlined in Table 1. The clinical findings were ocular symptoms (40.4%), arthritis (25%), dermatologic symptoms (13.5%),multi-organ involvement (11.5%),and features of other systems (9.6%).In 9 (17.3%) patients uveitis developed under treatment. The development of uveitis in BS/EOS group was significantly more frequent (p=0.01). Arthritis, lymphadenopathy, and anemia was significantly more common in the EOS/ BS group (p=0.014). Lung involvement was present in 16 (30.8%) patients. 8 (15.4%) patients had stage I, 5 (9.6%) stage II, 2 (3.8%) stage III and 1 (1.9%) stage IV lung involvement had. Six (11.5%) patients showed a low functional vital capacity (FVC). Bronchoscopy was peculiar in two cases

The elevated ACE levels with lung involvement was not significant (p=0.2).

Fourteen (26.9%) patients received pulse methylprednisolone. Methotrexate was preferred in 47 (90.3%) patients. The median duration of methotrexate was 24 months. Methotrexate was given to 20 (42.5%)of the patients as monotherapy. Fourteen patients received biologic agents in addition to methotrexate. Remission was in 15 patients (30.7%),partial response was in 28 (53.8%) cases and refractory disease was present in 4 (7.7%) patients. Flare occurred in 13 (25%) patients.

**Conclusion:** This is the largest series of pediatric sarcoidosis in the literature according to our knowledge. Sarcoidosis is very rare in children and its presentation and prognosis are different from adults;can develop at any age and requires long-term follow-up

**Patient Consent:** Yes, I received consent

**Disclosure of Interest**: None declared

**Table 1 (abstract P398).** See text for description

Age at disease onset (month); median (min–max)	83 (5–204)
Age at diagnosis (years); median (min–max)	8.5 (1.5– 17)
**Laboratory Findings**	
Hemoglobin level, (mg/dl)	12 ± 1.6
Anemia, n (%)	12(23.1%)
Eosinophilia, n (%)	4(7.6%)
Elevated levels of acute phase reactants, n (%)	29(55.8%)
Elevated levels of ACE, n (%)	26(50%)
**Biopsy, n (%)**	**19/52 (36.5 %)**

### P399. Case report of Behcet’s disease in pediatric population

#### A. Guzman, E. Faugier, H. W. Bermudez, S. Rodríguez Aguayo, N. D. L. R. Linne, M. A. Barba Aguilar, M. I. De la Cera Rodríguez, E. . Mercedes Pérez, H. F. Menchaca Aguayo, P. Ramos Tiñini, A. K. Primero Nieto

##### Reumatologia Pediatrica, Hospital Infantil De Mexico Federico Gómez, CDMX, Mexico

###### **Correspondence:** A. Guzman

**Introduction:** Behcet’s disease (BD) is a systemic vasculitis that can affect any type and size of blood vessel and involve almost any type of organ including gastrointestinal, nervous, musculoskeletal and cardiovascular. (1) although most of the patients develops the disease between the second and fourth decade of life, up to 15-20% develops in the pediatric age, for this reason is important to be aware of this pathology, therefore we present three cases of BD that we have documented in our institution. (2)

**Objectives:** Describe three cases of BD on pediatric population and review of the literature

**Methods:** Description of three cases of BD

**Results:** Case 1: Female starts at 10 years old with painful oral ulcers 4 episodes per year, is managed symptomatically and receiving multiple antibiotic and antiviral schemes, at 11 years old presents genital ulcer biopsy with inflammatory and necrotic infiltrate, starts therapy with Prednisone for 1mgkgdosis for 14 days with remission of the lesions. After 2 years she presented new oral and genital lesions, she was sent to the Rheumatology service, PCR for herpes virus was negative and HLB-51 negative, acneiform lesions were documented, Patergia positive and EB diagnosis was integrated, other organ involvement was ruled out, she started treatment with steroid 2mgkgdosis and Thalidomide.

Case 2: 13-year-old female with a history of oral and genital ulcers with a 1-year evolution, accompanied by arthralgias, headache and acneiform lesions. History of cervical lymphadenopathy and solitary thyroid nodule of 1 year of evolution, assessed by Oncology and Endocrinology, cervical lymph node biopsy with nonspecific inflammatory result, malignancy data is ruled out, thyroid function tests without alterations. When episodes of genital and oral ulcers persist, infectious causes were ruled out and she was sent to our service. Brain magnetic resonance angiography was performed with no alterations, genital ulcer biopsy with inflammatory and necrotic tissue. Ophthalmologic disease is ruled out. HLA-B51 negative and Patergia positive. Diagnosis of EB is integrated, therapy with short course of steroid and colchicine is initiated with adequate response.

Case 3: 15-year-old male with pain on ejaculation of 3 months of evolution, is evaluated by urologist who during urethrocystoscopy evidences penile ulcers. Oral ulcers, conjunctival hyperemia, photophobia and headache were documented, he was sent to Rheumatology. Cerebral magnetic resonance angiography showed stenosis of the left internal carotid artery associated with vasculitis. HLA-B51 negative. Diagnosis of EB was documented. Therapy was started with methylprednisolone and Cyclophosphamide doses, followed by Prednisone and Azathioprine, 6 doses of Cyclophosphamide were completed with adequate evolution.

**Conclusion:** As can be seen in the previously reported cases, EB is a pathology that can affect different organs, and it can take a long period of time for the expression of symptoms to conclude its diagnosis, being important to make known the heterogeneity of the disease to give the appropriate follow-up to make the diagnosis and timely treatment.

**Patient Consent:** Yes, I received consent

**Disclosure of Interest**: None declared

### P400. Blood transcriptomics to facilitate diagnosis and stratification in paediatric rheumatic diseases

#### M. K. Ha^1^, E. Bartholomeus^1^, L. Van Os^2^, J. Dandelooy^2^, J. Leysen^2^, O. Aerts^1,2^, V. Siozopoulou^2^, E. DeSmet^2^, J. Gielen^1,2^, K. Guerti^2^, M. De Maeseneer^3^, N. Herregods^4^, B. Lechkar^2^, R. Wittoek^4,5^, E. Geens^5^, L. Claes^2^, M. Zaqout^2^, W. Dewals^2^, A. Lemay^6^, D. Tuerlinckx^7^, D. Weynants^7^, K. Vanlede^8^, G. Van Berlaer^3^, M. Raes^9^, H. Verhelst^4^, T. Boiy^2^, P. Van Damme^10^, A. Jansen^2^, M. Meuwissen^1,2^, V. Sabato^1,2^, G. Van Camp^10^, A. Suls^10^, J. Van der Werff ten Bosch^3^, J. Dehoorne^4^, R. Joos^2,4,5^, K. Laukens^11^, P. Meysman^11^, B. Ogunjimi^1,2,3,5^

##### ^1^Faculty of Medicine and Health Sciences, University of Antwerp, ^2^University Hospital Antwerp, Antwerp, ^3^University Hospital Brussels, Brussels, ^4^University Hospital Ghent, Ghent, ^5^Antwerp Hospital Network, Antwerp, ^6^Turnhout General Hospital, Turnhout, ^7^University Hospital Namur, Namur, ^8^Nikolaas General Hospital, Sint-Niklaas, ^9^Jessa Hospital, Hasselt, ^10^University of Antwerp, Antwerp, Belgium, ^11^Department of Computer Science, University of Antwerp, Antwerp, Belgium

###### **Correspondence:** M. K. Ha

**Introduction:** Children who present with rheumatic symptoms often pose several challenges to their physicians due to the lack of optimal diagnostic criteria caused by the heterogeneity of many rheumatic diseases, variable clinical presentations, and complex pathophysiology.

**Objectives:** The objective of this study is to improve the early diagnosis of paediatric rheumatic diseases via whole blood transcriptomics combined with machine learning. We aim to investigate the gene expression of whole blood from children with rheumatic diseases in comparison with viral infection cases during active infection and convalescence, then apply machine learning on the transcriptomic data to develop classification models for identifying different disease groups.

**Methods:** Whole blood was collected from a cohort of 46 children during active viral infection (35 of whom also had blood collected at a second time point during convalescence) and 48 paediatric rheumatic patients including children with (i) auto-inflammatory diseases, (ii) juvenile idiopathic arthritis (JIA), (iii) chronic recurrent multifocal osteomyelitis (CRMO), (iii) an interferonopathy (IFN) such as juvenile dermatomyositis or systemic lupus erythematosus, (iv) vasculitis or (v) diseases related to the human leukocyte antigen B51 serotype. RNA sequencing was performed on the participants’ whole blood and the resultant transcriptomic data were used to develop classification models with the Random Forest algorithm. The performance of these classifiers was evaluated by leave-one-out cross-validation. Analyses of differentially expressed genes (DEG), gene ontology (GO), and interferon-stimulated gene (ISG) score were also conducted based on the transcriptomic data.

**Results:** Our first classifier could differentiate paediatric rheumatic patients from viral infection and convalescence cases with high area-under-the-curve (AUC) values (AUC = 0.8 ± 0.1 and 0.7 ± 0.1, respectively). Three other classifiers could distinguish CRMO, JIA, and IFN from viral infection and convalescence cases with AUC ≥ 0.8. DEG and GO analyses indicate that the pathophysiology of CRMO, IFN, and JIA involves innate immune responses including myeloid leukocyte and granulocyte activation, neutrophil activation and degranulation. IFN patients in particular had the highest mean ISG score among all disease groups.

**Conclusion:** Overall, our study indicates that blood transcriptomics combined with machine learning is a promising tool for paediatric rheumatic disease classification and diagnosis. Application of machine learning on other clinical and molecular data has potentials to assist paediatric rheumatologists in predicting the course of diseases, identifying important risk factors, and estimating treatment responses.

**Patient Consent:** Yes, I received consent

**Disclosure of Interest**: M. Ha: None declared, E. Bartholomeus: None declared, L. Van Os: None declared, J. Dandelooy: None declared, J. Leysen: None declared, O. Aerts: None declared, V. Siozopoulou: None declared, E. DeSmet: None declared, J. Gielen: None declared, K. Guerti: None declared, M. De Maeseneer: None declared, N. Herregods: None declared, B. Lechkar: None declared, R. Wittoek: None declared, E. Geens: None declared, L. Claes: None declared, M. Zaqout: None declared, W. Dewals: None declared, A. Lemay: None declared, D. Tuerlinckx: None declared, D. Weynants: None declared, K. Vanlede: None declared, G. Van Berlaer: None declared, M. Raes: None declared, H. Verhelst: None declared, T. Boiy: None declared, P. Van Damme: None declared, A. Jansen: None declared, M. Meuwissen: None declared, V. Sabato: None declared, G. Van Camp: None declared, A. Suls: None declared, J. Van der Werff ten Bosch: None declared, J. Dehoorne: None declared, R. Joos: None declared, K. Laukens: None declared, P. Meysman: None declared, B. Ogunjimi Grant / Research Support with: Abbvie, Pfizer, and Roche

### P401. Interleukin-1 inhibitors - a new hope for fibrodysplasia ossificans progressiva?

#### R. Haviv^1,2^, E. C. Hsiao^3^, L. Zeitlin^4^, N. Rabinowicz^1^, F. De Benedetti^5^, G. Prencipe^5^, Y. Uziel^1,2^

##### ^1^Pediatric Rheumatology Unit, Meir Medical Center, Kfar Saba, ^2^Sackler Faculty of Medicine, Tel Aviv University, Tel Aviv, Israel, ^3^Division of Endocrinology and Metabolism, University of California at San Francisco, San Francisco, United States, ^4^Pediatric Orthopedic Department, Dana-Dwek Children’s Hospital, Sourasky Medical Center, Tel Aviv, Israel, ^5^Division of Rheumatology, Bambino Gesù Children’s Hospital IRCCS, Rome, Italy

###### **Correspondence:** R. Haviv

**Introduction:** Fibrodysplasia ossificans progressiva (FOP) is the most catastrophic form of heterotopic ossification (HO), due to ongoing intracellular signaling through the BMP pathway. It results in rapid, irreversible bone accumulation and loss of mobility. Unfortunately, there is no effective therapy to cure the disease or dramatically change its course [1-16].

The recurrent, paroxysmal appearance of inflammatory “lumps” and the fact that studies of patients with FOP and other forms of HO, indicate that several cytokines, including interleukin-1 (IL-1), are increased, indicate a similarity between FOP and other auto-inflammatory diseases [17-26]. We hypothesized that anti-IL1 agents would ameliorate disease progression.

**Objectives:** To report our long-term experience using canakinumab (CKB, anti-IL-1β) to prevent FOP flares.

**Methods:** Patient data were analyzed, to characterize the efficacy of canakinumab in ameliorating FOP progression.

**Results:** Four FOP patients are currently being treated with 4mg/kg/month CKB at Meir Medical Center, Kfar Saba, Israel and the University of California, San Francisco, USA, with a total experience of over 9 patient years. Two patients were also treated with 100mg anakinra (ANA), daily, before switching to CKB. Markedly lower rates of HO flares were documented, with a decrease from 0.7–3.1 to approximately 0.27 flares per month (see Table 1). In general, no new HO sites were documented, but some existing sites kept growing under treatment, although at a much slower rate. Response to corticosteroids was improved and some lumps diminished under CKB alone.

**Conclusion:** Our data suggest that FOP may be an auto-inflammatory disease and flares are mediated by IL1β. Anti-IL1 agents may have a role in ameliorating FOP progression, which offers new hope to FOP patients.

**Patient Consent:** Yes, I received consent

**Disclosure of Interest**: None declared

**Table 1 (abstract P401).** Rate of HO flares in 4 FOP patients before and during anti-IL1 treatment

Patient number and gender	Age at diagnosis	Age at starting anti-IL1 treatment	Flare rate before anti-IL1	Flare rate during anti-IL1 treatment	Decrease
**1. Male**	13.5 years	15 years (ANA) 15.4 years (CKB)	40/13=3.07/month (prior to ANA)	ANA 100mg/day – 3/4=0.75/month. CKB ~ 2.7mg/kg monthly – 5/4=1.25/month. Split CKB 1.35mg/kg twice monthly – 13/16=0.81/month. After augmenting CKB dose to >2mg/kg twice monthly – 3/11=0.27/month Weighted mean of flares under anti-IL1 Tx – 0.68/month	91%
**2. Female**	3 years	5.7 years	23/18=1.28/month	CKB 4mg/kg monthly – 6/22=0.27/month, of which 3/6=0.5 were not treated with steroids, at least 5/6=0.83 were within known sites and 3/6=0.5 were within surgical site (anterior right neck)	79%
**3. Female**	16 months	23 months	4/4=1/month	CKB 4mg/kg monthly – 3/11=0.27/month, of which all were post-traumatic forehead swellings, all were partially treated with steroids (1-2 doses) and all became non-palpable over time	73%
**4. Female**	3 years	14.3 years (ANA) 14.5 years (CKB)	9/13=0.69 /month (before ANA)	ANA 100 mg daily, followed by CKB 3.5 mg/kg/month – 5/18=0.27/month, with significantly decreased pain and induration at flare sites	61%

### P402. In pediatric rheumatologic disease, methotrexate leads to mildly changed bloodwork on the second day after administration

#### B. Hügle, N. Fischer, J.-P. Haas

##### German Center for Pediatric and Adolescent Rheumatology, Garmisch-Partenkirchen, Germany

###### **Correspondence:** B. Hügle

**Introduction:** Methotrexate (MTX) is the most commonly used DMARD in the treatment of juvenile idiopathic arthritis (JIA). Recommendations regarding MTX monitoring recommend measurement of aspartate aminotransferase (GOT), alanine aminotransferase (GPT) and differential blood count. MTX is given as a single weekly dose creating a serum drug level during the following approximate 24 hours. There have been concerns that blood work taken during that time would show a transient increase in blood parameters, especially transaminases [5].

**Objectives:** The aim of this retrospective study was to determine levels of transaminases and blood counts comparing them by the number of days following MTX administration.

**Methods:** The database of the German Center for Pediatric and Adolescent Rheumatology was searched for patients with pediatric rheumatologic diseases admitted between 2018 and 2021 and treated with MTX. Weekday of blood sampling and last methotrexate dose was determined to calculate the time difference in days. Laboratory values for GOT, GPT, lymphocyte and neutrophil count were determined and normalized. Statistical analysis using ANOVA was performed, as well as Student’s test for levels beyond normal range.

**Results:** A one-way ANOVA revealed that there was a statistically significant difference for GOT (F(6, 966)=8.535, p<0.0001), GPT (F(6,966)=3.657, p=0.001) and neutrophil count (F(6,966)=4.841, p<0.0001) in days of difference, with the highest/lowest values on day 2 after adminstratio However, abnormal values were not found significantly more frequently on any day for all parameters.

**Conclusion:** In a large cohort of children with pediatric rheumatic disease significant change in bloodwork is found on the second day, rather than the first. his effect is too small to merit any clinical note of caution. Bloodwork can be done on any day of the week irrespective of MTX administration.

**Patient Consent:** Not applicable (there are no patient data)

**Disclosure of Interest**: None declared

### P403. The musculoskeletal manifestations of paediatric Inflammatory Bowel Disease (IBD): the gosh experience

#### R. Jashmi, S. Compeyrot-Lacassagne

##### Rheumatology, Great Ormond Street Hospital, London, United Kingdom

###### **Correspondence:** R. Jashmi

**Introduction:** Children with Inflammatory bowel disease (IBD) have extra-intestinal manifestations. Musculoskeletal (MSK) manifestations are known to be the most common extra-intestinal manifestations of IBD. Though it is well described that IBD can have systemic manifestations, the exact mechanism of joint disease and its relation to intestinal disease is still unclear. Patient and disease characteristics, treatments, and outcomes of p-IBD-associated MSK disease are not well established.

**Objectives:** This study aims to describe the musculoskeletal manifestations seen in children with inflammatory bowel disease.

**Methods:** A retrospective cohort study was carried out between February 2011 and April 2022 looking for all children with inflammatory bowel disease who presented at any time of their disease with inflammatory musculoskeletal manifestations (chronic arthritis or chronic non-infectious osteomyelitis (CRMO) at Great Ormond Street Hospital for children.

**Results:** Out of the 770 patients followed at GOSH for IBD, twenty-four patients (3.1%) were found to have inflammatory musculoskeletal manifestations of IBD. Median age at diagnosis of musculoskeletal manifestations was ten years. Most of the patients were male (66.7%) and had Crohn’s disease (19/24). Twenty-one patients had chronic arthritis, nine patients (42.9%) had axial arthritis and twelve patients (57.1%) had peripheral arthritis. Six patients had chronic non-infectious osteomyelitis (CNO). Three patients had both.

Twenty-two patients had confirmed chronic arthritis or CNO by imaging. Two patients had florid peripheral arthritis on examination and imaging was not requested. Eight patients had ultrasound findings: arthritis and tenosynovitis. Twenty patients had MRI changes (on focal or WB-MRI): Bone marrow oedema, enthesitis, synovitis and bone erosions.

There was a positive family history in five patients (20.8%): mostly IBD and psoriasis.

Seven patients (29.2%) were found to have skin manifestations mostly described as psoriasis (3/7) and Keratosis Pilaris (2/7). Twenty-three patients (95.8%) had constitutional symptoms.

Twenty-one patients (87.5%) had raised inflammatory markers. Seven patients were ANA positive, 2 patients Rheumatoid factor positive and 3 patients HLA-B27 positive.

Six patients received enteral feeds, 19 azathioprine and 5 sulfasalazine. 17/24 patients were treated with biologics. Anti-TNF was the most used biologics (infliximab was used in 13 patients and adalimumab was used 9 patients). Ustekinumab was used in four patients only.

There was a statistically significant positive correlation between age of diagnosis of IBD and age of diagnosis of arthropathy (r= 0.593, P= 0.002), suggesting that they occur at the same age.

There was no statistically significant association between IBD remission and arthropathy remission (Fisher’s exact, P= 0.082).

**Conclusion:** This small study described the musculoskeletal manifestations (chronic arthritis and CNO) of patients with IBD. There is a significant correlation between age of diagnosis of IBD and arthropathy. Larger studies are needed to identify predictors of outcome in children with musculoskeletal manifestations of IBD.

**Patient Consent:** Not applicable (there are no patient data)

**Disclosure of Interest**: None declared

### P404. Withdrawn

### P405. Ultra-high frequency ultrasonography of labial glands in pediatric Sjögren’s syndrome: a preliminary study

#### E. Marrani^1^, G. Fulvio^2^, C. Virgili^1^, R. Izzetti^3^, V. Dini^4^, T. Oranges^5^, C. Baldini^2^, G. Simonini^1,6^

##### ^1^Pediatric Rheumatology, AOU Meyer, Firenze, ^2^Unit of Rheumatology, ^3^Dentistry and Oral surgery, ^4^Dermatology Unit, Azienda Ospedaliero-Universitaria Pisana, Pisa, ^5^Dermatology Unit, AOU Meyer, ^6^NEUROFARBA, University of Firenze, firenze, Italy

###### **Correspondence:** E. Marrani

**Introduction:** Sjögren’s Syndrome (SS) is a chronic autoimmune disease, primarily affecting lacrimal and salivary glands. The 2016 American-European Consensus Group criteria are not validated in pediatric population. Therefore, the diagnosis of pediatric SS mostly relies on clinical suspect, resulting in a significant diagnostic delay. Recently, Ultra-High Frequency Ultrasound (UHFUS) of labial glands has been proposed as a diagnostic method in adults with suspected SS.

**Objectives:** The aim of the study is to evaluate the potential role of Ultra-High Frequency Ultrasound (UHFUS) of minor salivary glands for the diagnosis of Sjogren’s Syndrome in a cohort of pediatric population.

**Methods:** Consecutive paediatric patients with pediatric-onset SS seen at AOU Meyer between 1/4/2021 and 30/04/2022 were evaluated with UHFUS. To be eligible patients should be received a clinical diagnosis of Sjogren’s Syndrome before the age of 16 years, according to a combined set of clinical, serological, and instrumental findings. Clinical, radiological, and histopathological findings were retrospectively collected using a dedicated CRF. Intraoral UHFUS scan of the lip mucosa was performed with Vevo MD equipment, using a 70 MHz probe with a standardised protocol and the images were independently reviewed by two operators. Lips salivary glands were assessed by using a four-grade semiquantitative scoring system (0-3), similar to the OMERACT scoring system used for major salivary glands.

**Results:** Twelve patients with paediatric SS were included in the study (n = 12, 11 females; 10 Caucasian, 2 Asian), with a median age at the diagnosis of 14.1 years (range 7.75-17) and a median disease duration of 13.5 months (range 1-94) . Eleven patients were ANA positive; Ro/SSA were positive in 6/12 while none tested positive for LLA/SSB. Minor salivary gland biopsy was performed in 9/12, showing inflammatory chronic sialadenitis in 8/12. Treatment with hydroxychloroquine was ongoing in 11/12. When applying UHFUS to this cohort of patients, all patients showed a UHFUS grade of ≥1 with 8/12 showing a mild glandular alteration (i.e. grade 1), 2/12 a moderate glandular alteration (i.e. grade 2) and finally 2/12 a severe glandular alteration (i.e. grade 3). A moderate intraglandular vascularization was seen in 9/12, with only 3/12 showing a mild intraglandular vascularization. Due to limited size of the sample, the relationship between histological findings, autoantibodies status and UHFUS grade could not be performed

**Conclusion:** Pediatric SS is a rare condition, and its prevalence is under-estimated due to the lack of standardized diagnostic criteria and the subtle early clinical presentation. New approaches to a faster diagnosis are urgently needed in clinical practice. This preliminary pilot study seems to report UHFUS as feasibility technique to identify salivary gland alterations in children with a clinical suspect of SS. This technique might contribute to drive guided lip biopsy, thus reducing the rate of false negative. Further studies are currently in progress in our clinics to identify the exact role of UHFUS and its potential predictive role of the various patterns observed in pediatric SS.

**Patient Consent:** Yes, I received consent

**Disclosure of Interest**: None declared

### P406. Maternal and neonatal outcomes in pregnant women with rheumatic diseases - a retrospective cohort study

#### L. Martínez, E. Moreno, M. López, A. Pujol

##### Pediatric Rheumatology Unit - Rheumatology Department, Vall d’Hebron University Hospital, Barcelona, Spain

###### **Correspondence:** L. Martínez

**Introduction:** Inflammatory and autoimmune rheumatic diseases especially affect women of childbearing age.

These diseases are associated with a greater risk of pregnancy complications including preeclampsia, preterm delivery and low birthweight infants. Frequency, variety, and severity of these complications are dependent on maternal diagnosis, serologic profile and disease activity at the moment of pregnancy. In general, it is recommended to achieve remission or low disease activity before pregnancy.

In our center, female patients with rheumatic diseases who are planning pregnancy or are pregnant are followed by a multidisciplinary team which includes obstetricians, pediatric rheumatologists and neonatologists. In 2019, a follow-up and treatment protocol was established, including assessment of babies in the pediatric rheumatology unit in the first year of life as well as cardiologic evaluation in cases where it was indicated.

**Objectives:** We herein present data of the center experience in daily practice following protocol implementation.**Methods:** We conducted a retrospective observational study based on data obtained from clinical practice. Maternal diagnosis and serological profile, treatments administered during pregnancy and complications of previous and recent pregnancies affecting both mother and/or children were collected.

**Results:** A total of 23 women and 26 children (3 twin pregnancies) were included in the present study. 4 out of 23 mothers (17.3%) had previous pregnancy complications including spontaneous abortions (2/23) and stillbirths after intrauterine growth retardation (2/23). Regarding current pregnancies, 7 of the 23 pregnancies presented complications (table 1). Out of the 26 babies evaluated in the pediatric rheumatology unit, 3 presented neonatal lupus with skin involvement, all of them being born to mothers with Anti Ro+. In all cases, lesions were self-limited before 3 months of age. Of these cases, only one mother had received preventive treatment with hydroxychloroquine during pregnancy. The 12 babies born to mothers with Anti Ro, La, U1 RNP antibodies underwent evaluation by pediatric cardiology at birth and at one year of life and none presented atrioventricular block. Eight of these mothers received preventive treatment with hydroxychloroquique. None of the babies presented infections that could be associated with the immunosuppressive maternal treatment during pregnancy. A 7-month-old girl born to a mother with rheumatoid arthritis, presented with pyelonephritis due to *Escherichia coli*: the mother had not received immunosuppressive treatment during pregnancy.

**Conclusion:** In our series, 25% of the children (3/12) who were born to mothers with anti-Ro/La or RNP antibodies developed neonatal lupus with cutaneous involvement. In all cases lesions were resolved in the first three months of life without secondary scarring.

There were no cases of atrioventricular block in those children and 66.7% (8/12) of their mothers were receiving preventive treatment with hydroxicloroquine during pregnancy. As atrioventricular block is a low-prevalence complication, larger series studies are needed to establish the efficacy of this treatment for the prevention of atrioventricular block.

**Patient Consent:** Yes, I received consent

**Disclosure of Interest**: None declared

**Table 1 (abstract P406).** Incidences of pregnancy/delivery in relation to maternal disease and immunity

RA		Fetal tocographic registry alterations: labor induction, low birth weight, preterm (Twin pregnancy)
JIA	ANA+	Preeclampsia, preterm, underweight (twin pregnancy)
RA	ANA+	HELLP
JIA		Preeclampsia, intrauterine growth retardation
SLE	ANA+	Preeclampsia, intrauterine growth retardation preterm, underweight
SLE	ANA+ Anti-DNA +	Preeclampsia, low birth weight
Sjögren	ANA+, Anti Ro+, Anti-La+	Threatened preterm labor, low birth weight
SLE	ANA+, Anti- Ro+, lupus anticoagulant +	Cesarean (Stationary labor)

### P407. Primary sjogren’s syndrome in children: a experience from a single center

#### D. Patro^1^, L. B^2^, I. Rajarathinam^2^, J. Raghuram^2,3^, A. P. Rao^1,2^

##### ^1^Pediatric Rheumatology, Manipal Hospital, Bengaluru, ^2^Pediatric Rheumatology, Indira Gandhi Institute of Child Health, ^3^Pediatric Rheumatology, Aster Whitefield, Bengaluru, India

###### **Correspondence:** D. Patro

**Introduction:** Primary Sjogren’s Syndrome(pSS) is a rare disorder often known as autoimmune exocrinopathy or epithelitis. Unlike in adults, it does not present with sicca-like manifestations in children which leads to a delay in diagnosis.

**Objectives:** 1. To describe the varied clinical manifestation of pediatric Sjogren in our cohort.

2. To assess the EULAR Sjogren’s Syndrome Disease Activity Index (ESSDAI) at the onset.

**Methods:** We reviewed 9 cases of pSS which fulfilled the 2016 ACR- EULAR Classification criteria and Proposed Juvenile pSS by Bartunkova et al.

**Results:** In our series, the majority were females (89%). The mean age of presentation was 11.5 years (±2.45). The findings are summarized in the table below. Extra-glandular manifestations were more common at presentation. 66.7% of patients had joint manifestations followed by renal tubular acidosis (RTA) in 44.4%. 2 patients each initially presented with Immune-mediated thrombocytopenia (ITP) and Ig A vasculitis (IAV). The mean ESSDAI score at onset was 6.88 ± 1.98. The most frequently scored domain in ESSDAI is constitutional followed by the articular and glandular domain. Activity level was more in the articular and hematological domain. The drawback of our study was objective involvement of the salivary gland could not do due to invasiveness and financial constraints.

**Conclusion:** Sjogren’s should be kept in the differential of U-CTD with positive surrogate markers in a resource-poor setting which helps in bridging the gap between diagnosis and timely interventional. ESSDAI score at the onset with involvement of multiple domains guides us for aggressive immunosuppression.

**Patient Consent:** Yes, I received consent

**Disclosure of Interest**: None declared

**Table 1 (abstract P407).** See text for description

Patient	A	B	C	D	E	F	G	H	I
Age of presentation	7	9	15	11	12	14	12	13	11
Delay in months Median: 24 (IQR:10-36)	24	108	6	36	2	36	24	18	10
Presenting complaints	MSK ILD	MSK Red Eye Cataract Caries	ITP MSK HTN NPS Caries	MSK NPS PNS	ITP	PP MSK	IAV PP	IAV MSK PP	PP
Initial Diagnosis	p-JIA	U-CTD	SLE	p-JIA	U-CTD	RTA	IAV RTA	IAV AIPS RTA	FS
Symptoms									
1. Oral dryness	1	1	1	2	1	1	-	1	
2. Ocular dryness	2							2	2
3. Parotid swelling			3			3		3	
Objective evidence									
1. Schirmer’s test ≤5 mm (Y/N)	(N)	(Y)	(N)	(Y)	(N)	(N)	(N)	(Y)	(Y)
2. Minor Salivary gland foci ≥1 (Y/N)	(Y)	(N)	(N)	(N)	ND	ND	ND	ND	ND
Additional Test positive									
1. RF Positive	1	3	5	3	5	2	3	2	2
2. RTA	2	5		4		3	5	3	3
3. Globulin	3			5				5	5
4. Amylase	4								
5. Cytopenia	5								
Immuno-modulation used	MTX IAS LF TOF	MTX IAS	MF	MTX IAS MF	IVIG MF	TOF	AZA	AZA	MF
ESSDAI Score at onset	10	9	6	8	6	5	4	9	5

Abbreviation used: AIPS: Autoimmune polyglandular syndrome; AZA: Azathioprine; FS: Fanconi syndrome; MSK: Musculoskeletal; IAS: Intra-articular steroids; ILD: Interstitial lung disease; IVIG: Intravenous Immunoglobulin; LF: Leflunomide; MF: Mycophenolate mofetil; MTX: Methotrexate; NPS: Neuro-psychiatric; ND: Not done; PP: Paraparesis; p-JIA: Polyarticular juvenile idiopathic arthritis; PNS: Peripheral nervous system; RF: Rheumatoid factor; TOF: Tofacitinib; U-CTD: Undifferentiated Connective Tissue Disorder

### P408. Foreign body synovitis (case report)

#### F. Phoya^1^, W. Slamang^2^, C. Scott^1^, A. Horn^3^, S. Singh^4^

##### ^1^Rheamatology, University of Cape town, ^2^Rheamatology, Red Cross Hospital , ^3^orthopaedic, University of Cape town, ^4^Histopathology , Red Cross Hospital, Cape town, South Africa

###### **Correspondence:** F. Phoya

**Introduction:** Joint pain or swelling with limitation of movement is one of the most common presenting complaints in paediatric A&E departments. The wide range of differential diagnoses make it challenging to narrow down a particular cause, which can lead to delays in the initiation of appropriate management.

Arthritis is characterized by joint pain, swelling, limitation of movement and sometimes periarticular erythema. These symptoms may be the initial presentation of a number of diseases, the differential diagnosis in children including septic arthritis, Lyme arthritis, foreign body synovitis, pigmented villonodular synovitis, hemarthrosis, avascular necrosis, malignancy, juvenile idiopathic arthritis (JIA) and rarely crystalline arthritis among others.

An accurate history, physical examination and initial investigations are invaluable in differentiating a chronic inflammatory arthritis like JIA, from the other causes. A previous history of trauma may be helpful in acute cases but may also detract from the underlying diagnosis. Skill and care is therefore required in identifying the most pertinent information in each case.

**Objectives:** Here we focud on synovitis in a young child caused by a missed foreign body.

**Methods:** We present a case to highlight the importance of a thorough history, including that of previous trauma, and the utility of ultrasound as a go to investigation when there are inconclusive findings on traditional tests like plain radiograph

**Results:** n/a

**Conclusion:** With a clear history of trauma or penetrating injury, every effort should be made to rule out a foreign body, particularly when there is no improvement after treatment. While plain radiographs are not very sensitive at picking up radiolucentforeign bodies, ultrasound may be a helpful tool to narrow down the diagnosis and also to localize the exact position of a foreign body. Although MRI has been shown to be sensitive and the investigation of choice, it might not always be easily available or accessible in many settings

**Patient Consent:** Yes, I received consent

**Disclosure of Interest**: None declared

### P409. Paediatric musculoskeletal education in the Republic of Ireland

#### B. Power^1,2^, S. McGlacken-Byrne^2^, E. MacDermott^3^, O. Killeen^4^

##### ^1^Dept. of Rheumatology, Children’s Health Ireland, Crumlin, Dublin, ^2^Clinical Education, National University of Ireland, Galway, ^3^Dept.of Rheumatology, ^4^Dept of Rheumatology, Children’s Health Ireland, Crumlin, Dublin, Ireland

###### **Correspondence:** B. Power

**Introduction:** In July 2020, The Paediatric Task Force for Global Musculoskeletal Health expressed a ‘call to action’ amongst the musculoskeletal community. Internationally, suspected deficits in paediatric musculoskeletal education and training have been cited as potential factors contributing to delayed referral of patients to specialist rheumatological services. Resultant delays may lead to delayed diagnosis and treatment leading to a reduction in physical function, persistent pain, and potentially irreversible joint damage.

**Objectives:** Our study aimed to investigate the provision of paediatric musculoskeletal education in the Republic of Ireland. In addition, we wished to evaluate the relationship between paediatric musculoskeletal education and doctors’ confidence in clinical examination skills and knowledge of common rheumatological disorders of childhood.

**Methods:** A quantitative, cross-sectional, questionnaire-based study was performed. This was based on previously validated instruments. All Consultant General Paediatricians and paediatric doctors currently enrolled in formal paediatric training in the Republic of Ireland (July 2020 to July 2021 training year) were included. The questionnaire was distributed to the participants using the online survey tool, SurveyMonkey. The data was analysed using standard statistical software (SPSS). Kruskal-Wallis and Chi-Square tests were used to investigate potential associations between educational experience, confidence in skills and knowledge-base of physicians. A p value of <0.05 was considered statistically significant. Ethical approval was granted by the Research and Ethics Committee of the Royal College of Physicians of Ireland.

**Results:** In total, 471 doctors were included (82 Basic Specialist Trainees, 150 Higher Specialist Trainees and 239 Consultant Paediatricians). There was an overall response rate of 41% (n=192). Forty-nine percent (n=94) and 48% (n=92) recalled receiving teaching on paediatric musculoskeletal examination skills during undergraduate and postgraduate training respectively. Seven percent (n=14) were ‘not at all confident’ in musculoskeletal assessment, and 61% (n=117) were ‘confident in some aspects only’. Doctors were less confident in musculoskeletal examination than all other bodily systems, except for ophthalmology assessment. There was a statistically significant association between receiving postgraduate musculoskeletal skeletal education and higher confidence in clinical skills, x2 (3, N=191) = 25.655, p = 0.000, but no significant association between receiving undergraduate musculoskeletal skeletal education and confidence, x2 (3, N=192) = 1.844, p = 0.606. There was no significant association between confidence in clinical skills and knowledge of rheumatic disorders, H(2) = .792, p=0.673.

**Conclusion:** Our study has been the first study to explore paediatric musculoskeletal education in the Republic of Ireland, the self-reported confidence of paediatric doctors in paediatric musculoskeletal examination skills, and their knowledge of rheumatic diseases of childhood. Using a learner-centred educational approach, our research has offered valuable feedback from learners within the speciality of paediatrics. Our research has identified deficiencies in the paediatric training curriculum which will help to inform future curriculum planning for undergraduate and postgraduate clinical education in paediatric rheumatology. This study will encourage further reflection and deliberation on paediatric musculoskeletal education, which will ultimately improve patient safety and quality of care for paediatric patients in the Republic of Ireland.

**Patient Consent:** Yes, I received consent

**Disclosure of Interest**: None declared

### P410. Prednisone effectiveness in children with Sydenham’s chorea based on a 27-year-experience of a Tertiary Center

#### G. Rogani^1^, A. Petaccia^1^, G. B. Beretta^1^, S. Testa^1^, E. A. Conti^1^, F. Chironi^1^, F. Lucioni^1^, F. Baldo^1^, A. M. Cappellari^2^

##### ^1^Pediatric Rheumatology, ^2^Neuroscience, Fondazione IRCCS Ca’Granda Ospedale Maggiore Policlinico, Milan, Italy

###### **Correspondence:** G. Rogani

**Introduction:** Sydenham’s Chorea (SC) is a neuropsychiatric autoimmune disorder occurring in up to 40% of patients with acute rheumatic fever and there are no guidelines for its treatment.

**Objectives:** The aim of the study is to evaluate the efficacy of prednisone therapy in children with SC in term of time of remission and recurrence rate, compared to other treatments. Furthermore, correlation has been investigated between duration of symptoms and the following clinical and biochemical features at chorea onset: age, throat culture positivity for streptococcus, presence of hemichorea, psychiatric symptoms, carditis and/or arthritis, reactive C protein (RCP), erythrocyte sedimentation rate (ESR) and anti-streptolysin-O (ASO) titer.

**Methods:** This is an observational, retrospective, single-center study involving all patients diagnosed with SC, according to 2015 revised Jones criteria, admitted to Pediatric Rheumatology Unit of Policlinic Hospital of Milan (Italy), between January 1995 and March 2022. Clinical data of patients were collected from medical records. For statistical analysis Pearson chi squared, Pearson correlation coefficient and the non-parametric Mann Whitney test were used, when appropriate. P-value lower than 0.05 were considered significant.

**Results:** Of 59 enrolled patients (74% female; median age 9.3, range 7.4-10.6 years), 49 were considered eligible (10 were excluded due to incomplete data) and, among these, 79% received steroid therapy. The remaining patients were treated with symptomatic drugs (neuroleptics or valproate). The analysis shows that time of remission is significantly shorter in patients treated with prednisone therapy (median time 31 vs 41 days, p=0.023). Moreover, the presence of arthritis at SC onset was associated to longer symptoms duration (median time 90.5 versus 39 days, p=0,02). Recurrence occurred in 12% among the eligible patients and seemed to be related only to a younger age at onset (p=0,01).

**Conclusion:** Our study demonstrates the superiority of prednisone compared to symptomatic treatments for SC remission. Major strengths of this study are the large number of investigated patients and the long follow-up time (27 years).

**Disclosure of Interest**: None declared

**Table 1 (abstract P410).** See text for description

	Prednisone therapy	Symptomatic therapy	P value
Median time of remission (Interquartile range)	31 days (13 – 41 days)	41 days (33 – 135 days)	0.023

### P411. Case series of three patients with pachydermotactily

#### I. Rukavina^1^, M. Frković^1^, I. Brnadić^2^, M. Jelusic^1^

##### ^1^Department of Pediatrics, ^2^Department of Pathology, Clinical Hospital Center Zagreb, Zagreb, Croatia

###### **Correspondence:** I. Rukavina

**Introduction:** Pachydermodactily (PDD) is a rare digital fibromatosis of unknown origine mostly found in adolescent boys. It is manifested by simetrical, painless swelling of the periarticular soft tissues of the fingers without joint involvement. It is thought to be related to repetitive mechanical trauma where patients also can have different cutaneous responses to the attrition of the skin (for example neurodermitis). Treatment is often not indicated given its benign prognosis.

**Objectives:** These reports aim to define characteristics and increase awareness of the disease that would differentiate it from other disorders that may produce PIP joint swelling, particularly juvenile idiopathic arthritis.

**Methods:** We present clinical, laboratorial and radiological characteristics of 3 male adolescents (aged 17 to 18 years) with simetrical periarticular soft tissue thickening of the fingers.

**Results:** First patient is a 17-year-old boy who noticed bilateral second to fifth PIP joint and thumb swelling that have developed through several years together with hyperkeratose nodulations. No pain was noted neither morning stiffness. He does hard physical work at his family farm. Hand MRI showed periarticular thickening of the PIP joints bilaterally with discrete subcutaneous edema and no signs of synovitis. Histological analysis of the skin showed hyperkeratosis with discrete perivascular inflammation in papilar dermis. Results of the other diagnostic tests specific for rheumatic diseases were normal. Another patient is a 18- year-old boy who developed painless swelling of the PIP joints as well as second and third MTP joints bilaterally. He was examined by dermatologist and the diagnose of contact allergic dermatitis was made. Results of the diagnostic tests for rheumatic diseases were normal. Histological analysis of the skin showed hyperkeratosis with proliferation of fibroblasts and increase in the bands of collagen in the dermis. The third patient is a 17-year-old boy who have developed bilateral second to fifth PIP joint and second to third MTP joint skin thickening with hyperkeratose nodulations two years ago. All joints are painless with normal range of motion and grip strenght. Ultrasound showed just soft tissue thickening with no signs of synovitis. Rheumatic diseases laboratorial tests were normal.

According to clinical picture, results of laboratorial tests, radiological and histological findings the diagnose of pachydemodactily was made.

**Conclusion:** Although PPD is a rare disease, probably it is very underdiagnosed. It is triggered or at least aggravated with mechanical trauma of the fingers that can be unconcious at some patients (rubbing hands or cracking knuckles). It is needed to exclude other rheumatological disease that can have similar manifestations. Make a diagnose in a short period will reduce unnecessery diagnostic tests, treatment and patient anxiety.

**Patient Consent:** Yes, I received consent

**Disclosure of Interest**: None declared

### P412. Neuromyelitis optica spectrum disorder in pediatric sjogren’s syndrome

#### M. C. Santos^1^, F. D. Kuchiki^1^, F. Arita^2^, I. Lacerda^2^, G. Oliveira^1^, C. Benavides^1^

##### ^1^Pediatric Rheumatology, ^2^Pediatric Neurology, Irmandade Da Santa Casa De Sao Paulo, São Paulo, Brazil

###### **Correspondence:** M. C. Santos

**Introduction:** Sjögren Syndrome is a chronic autoimmune disease characterized by inflammation of exocrine glands, mainly lacrimal and salivary exocrine glands. At least one third of patients present with systemic extraglandular manifestations like articular, pulmonary and neurological. The prevalence and clinical manifestations of neurological involvement in SS have been reported variably. Central nervous system (CNS) involvement has a variable reported frequency between 0,3 and 48%. Neuromyelitis optica spectrum disorder (NMOSD) is one of the CNS manifestations. There is a discussion about the relationship between the Sjögren syndrome and NMOS. It may be just an association or NMOSD may be a neurological manifestationof Sjögren syndrome.

**Objectives:** The objective of this study is to present two Sjögren syndrome cases which had NMOSD asneurological manifestation.

**Methods:** Chart review to check medical history, laboratory tests and imaging of these children with Sjogren Syndrome, according to the American College of Rheumatology SICCA Classification criteria (ACR/SICCA). An informed consent form was signed.

**Results:** Patient, eleven years old girl, carrier of common variable immunodeficiency, admitted with history of repeated mump, paresthesia of lower limbs, headache and seizures. The laboratory test showed elevated levels of anti- Ro antibodies. Added to this, it was observed hypergamaglobulinemia, hyperproteinrrachia and increase of spinal fluid pressure. The MRI shows papilledema and multiple bilateral encephalic lesions of white and gray matter, corresponding to residual lesions of vasculitis. It was performed biopsy of salivary gland to confirm the diagnosis os pediatric Sjögren syndrome. Corticosteroid, IVIG and immunosupressives drugs were introduced and in one of them, it was necessary the use of plasmaferese and . Fews months later, patient started with headache and visual loss. A new exam showed image suggesting a diagnosis of optic neuritis. Added methylprednisolone (30 mg/kg/day for 3 days) with the medications described above and patient evaluated visual improvement, at the beginning.

Patient, nine years old girl, who presented Sjögren syndrome (elevated level of anti Ro antibodies , altered sialometry and biopsy of salivary gland showing alterations compatible with Sjögren syndrome). She started with headache, evolving with a sensory motor surge in the right upper limb. Cranial magnetic resonance imaging showed areas of signal alteration in periventricular white matter, mainly in the radiated crowns, bilaterally with total improvement after pulse therapy with methylprednisolone. After 5 months, the patient started with headache, evolving with a sensory motor surge in the right upper limb. Cranial magnetic resonance imaging showed areas of signal alteration in periventricular white matter, mainly in the radiated crowns, bilaterally with total improvement after pulse therapy with methylprednisolone. After 9 months, he evolved with low visual acuity. Pulse therapy with methylprednisolone, rituximab and plasmapheresis were performed, with excellent response.

Both patients didn’t present important symptoms of sicca syndrome.

**Conclusion:** Conclusion: NMOSD may be a neurological manifestation in Sjögren syndrome patients, and can represent the initial manifestation of Sjögren Syndrome, even when mild complaints of SICCA symptoms are absent. Complaints like headache, visual loss, should be valued and investigated for an early diagnosis. Therefore, the detailed history, antibodies test, images and biopsy are necessary to a prompt diagnosis, stablish the appropriate treatment and avoid sequelae.

**Patient Consent:** Not applicable (there are no patient data)

**Disclosure of Interest**: None declared

### P413. A case of synovial chondromatosis of the shoulder in a young girl

#### M. Trevisan^1^, L. Di Lenarda^2^, S. Pastore^3^, A. Saccari^3^, G. Canton^4^, U. Lucangelo^5^, L. Murena^4^, A. Taddio^3^

##### ^1^Department of Medicine, Surgery and Health Science, University of Trieste, IRCCS Burlo Garofolo, ^2^Department of Medicine, Surgery and Health Science, University of Trieste, ^3^Institute for Maternal and Child Health, IRCCS Burlo Garofolo, ^4^Orthopaedics and Traumatology Unit, Cattinara Hospital, Azienda Sanitaria Universitaria Giuliano-Isontina (ASUGI), ^5^Department of Perioperative Medicine, Intensive Care and Emergency, Cattinara Hospital, Trieste, Italy

###### **Correspondence:** M. Trevisan

**Introduction:** Primary Synovial Chondromatosis (PSC) is a rare benign tumor of the synovial membrane in which cartilage metaplasia produces calcific loose bodies within the articular space. Only a few cases are reported in the pediatric population and its etiology remains unknown. This condition typically affects large weight-bearing joints with pain, swelling and decrease range of motion. Due to its slow progressions, delayed diagnosis is frequent and differential diagnosis should consider other chronic arthritis and malignancies. While arthroscopic removal of loose bodies is the current treatment up to now, the association of partial or complete synovectomy is debated.

**Objectives:** N/A

**Methods:** N/A

**Results:** Case presentation: We report about a 14-year-old girl with a long-lasting right shoulder pain, especially during movements or exercise, localized tenderness and hypotonia of the glenohumeral joint. No previous trauma was mentioned. Blood exams, Mantoux test and plain radiography of the right shoulder were unremarkable. Ultrasound imaging revealed echogenic and calcified bodies stretching the glenohumeral joint and dislocating the long head of biceps tendon. Magnetic resonance showed a “rice-grain” pattern of the right shoulder. From an arthroscopic surgery, multiple loose white bodies were removed within the synovial membrane, and synovial chondromatosis was confirmed by histological analysis. At one month follow up visit, the patient completely recovered without pain.

**Conclusion:** Synovial chondromatosis is a very uncommon cause of mono articular pain in children, especially when it affects shoulder. Pediatricians should keep in mind this condition to avoid delayed diagnosis and treatment, even in consideration of the low risk of malignant transformation. Through this case, we would highlight common diagnostic pitfalls and treatment of synovial chondromatosis.

**Patient Consent:** Yes, I received consent

**Disclosure of Interest**: None declared

### P414. Role of the paediatric rheumatologist in the management of tubulointerstitial nephritis and uveitis, a single-centre experience

#### I. Turtsevich, S. Compeyrot-Lacassagne

##### Great Ormond Street Hospital NHS Trust, London, United Kingdom

###### **Correspondence:** I. Turtsevich

**Introduction:** Tubulo-interstitial nephritis and uveitis (TINU) is a rare still under-recognised autoimmune condition associated with the renal and ocular involvement of unknown aetiology. Although rare in incidence, there is a hypothesis that the immune-mediated mechanisms are not limited to the renal and ocular inflammation, but may also cause multisystem involvement.

**Objectives:** To explore other organ systems involvement and the role of the rheumatologist in the management of TINU as a multisystem entity.

**Methods:** Patients aged 0-18 with the established diagnosis of TINU, diagnosed and treated at Great Ormond Street Hospital for Children NHS Trust between January 2010 and March 2022 were involved in the study. TIN was defined as the presence of AKI, elevated tubular markers (RBP and NAG) and confirmed by the kidney biopsy. Uveitis was confirmed by the routine ophthalmology assessment on the slit lamp examination. All the relevant data were extracted from the electronic patients records and were fully anonymised. The data analysis was carried out by using IBM SPSS, v.26.

**Results:** A total of 19 patients were included in the study, female gender being prevalent (p=0.002). The mean age of the patient was 13.8±2.3 years old with the mean disease onset being at 11.8±2.1 years. The cohort was multi-ethnic with the prevalence of children from Black African ethnicity (p<0.001). Most of the patients were initially presented with either TIN or uveitis (47.4% and 42.1%, respectively, p=0.104) and in the majority of the cases, the trigger was not identified (0=0.021).

Roughly two third of patients complained of fatigue, following weight loss, fever and abdominal/flank pain. Other symptoms also included arterial hypertension, dysuria and polyuria, cervical lymphadenopathy and polydipsia. The minority reported skin rash and arthralgia/arthritis.

A large proportion of the patients were found to have bilateral anterior uveitis (78.9%, p<0.001) and the rest had unilateral anterior uveitis, retinal vasculitis, bilateral panuveitis and papilledema. Kidney biopsy was performed in 12/19 patients with TIN being confirmed in 10 patients, while 2 other patients were found to have either immune-mediated glomerulonephritis or changes were non-specific (p<0.001).

Out of 19, three patients developed irreversible hearing impairment and no other extrarenal/extraocular involvement has been found.

The level of inflammatory markers, including ESR and CRP were elevated in 12 patients, while creatinine was raised in all the patients.

Tubular markers were measured in 17 patients with NAG being high in 84.2% and RBP in 73.7% of the children.

Glucosuria was found in 68.4% of patients, whereas proteinuria and UA:UC ratio was positive in 52.6% of the TINU children. Other positive findings included microscopic haematuria and pyuria.

Further laboratory analysis revealed that third of TINU patients had elevated Ig A, G and M. Furthermore, ANA was positive in 6 patients, 2 of them had hearing loss (p>0.05). ENA or ANCA were negative for all the patients.

All patients received systemic GCs and Steroid eye drops. Eleven patients with active TIN were escalated to MMF, while those who remained active for uveitis had their treatment escalated to either Methotrexate (2 patients) or anti-TNF treatment (1 patient) having good response to the treatment.

**Conclusion:** The role of the paediatric rheumatologist in the management of TINU is to identify any extrarenal/extraocular impairment, particularly hearing loss by performing an extended autoimmune screening and audiology earlier in the disease course. Further analysis is needed to explore early predictor markers for the hearing loss as one of the extrarenal/extraocular involvement and possible early escalation to steroid-sparing DMARDs.

**Disclosure of Interest**: None declared

### P415. CACP syndrome (camptodactyly, arthropathy, coxa vara, pericarditis). clinical case –16 years follow-up

#### E. Vrtikova, E. Košková

##### National Institute of Rheumatic Dieseses, Piešťany, Slovakia

###### **Correspondence:** E. Vrtikova

**Introduction:** CACP syndrome is rare condition characterized by congenital camptodactyly and early childhood onset of non-inflammatory synovial hyperplasia. This syndrome with autosomal recessive inheritance is caused by a mutation in the PRG4 gene, which is responsible for the synthesis of lubricin, a protein that lubricates the joints. In some patients only coxa vara is present, in others pericarditis is the leading sign, some have both of these symptoms.

**Objectives:** Our patient, a 18 year old girl, was diagnosed with camptodactyly as an infant and the defect was resolved surgically by the age of 15 months. At the age of 18 months frequent swelling and “cracking” of the knee was observed. Even though the rheumatologist confirmed the presence of swelling of elbows, wrists, knees, and ankles, the mobility was intact and there was no limitation of range of motion. Ultrasonography showed a non-inflammatory synovial hyperplasia and an accumulation of the intraarticular fluid. Markers of inflammation in serum were normal, inflammation was excluded also by analysis of the synovial fluid. She was diagnosed with the CACP syndrome at the age of 30 months. Her parents were negative for consanguinity.

Radiographs of the hip joints detected early signs of dysplasia. At the age of 6 years the X-ray of pelvis confirmed the presence of the coxa vara. The early course of the disease was managed by intensive physiotherapy. Over the following years there was a progression of the femoral varus deformity with pathological attitude and accentuation of lumbar lordosis, resulting to difficult walking, muscle weakness, and increased fatigue. Last year, she underwent a combined periacetabular osteotomy and proximal femoral derotation osteotomy with consequent improvement in mobility.

**Methods:** Cese report

**Results:**

1. The CACP syndrome may be misdiagnosed as a juvenile idiopathic arthritis. Immunosuppressive therapy is not effective but adequate pain treatment and rehabilitation has an irreplaceable role.
2. Musculoskeletal ultrasonography and cytological examination of synovial fluid seem to be very important for the diagnosis of the CACP syndrome. In our patient the findings of ultrasonography, together with the history of camptodactyly in infancy and negative markers of inflammation were crucial in the differential diagnosis.

**Conclusion:** Over the following years coxa vara and limited mobility developed in our patient. Even though the disease does not affect life expectancy, its character often requires surgery and joint replacement in the early adult.

**Patient Consent:** Yes, I received consent

**Disclosure of Interest**: None declared

## Poster session: Psycho-social aspects and rehabilitation

### P416. The children’s emotional state and quality of life depends on subtypes of juvenile idiopathic arthritis

#### L. Bohmat^1,2^, A. Fadieieva^1,2^, N. S. Shevchenko^1,2^

##### ^1^Department of rheumatology and comorbid states, SI “Institute for Children and Adolescents Health Care of the NAMS of Ukraine”, ^2^Department of pediatrics № 2, V.N.Karazin Kharkiv national university, Kharkiv, Ukraine

###### **Correspondence:** A. Fadieieva

**Introduction:** Juvenile idiopathic arthritis (JIA) is a chronic childhood disease that aggravates not only the physical component of health but also the emotional state of patients. According to modern guidelines, it is necessary both to consider the objective manifestations of the disease and to assess the subjective feelings of patients. Such actions are important for the realization of comprehensive care for patients with JIA.

**Objectives:** The aim was to study the emotional area of quality of life (QoL) of patients with JIA with different subtypes of the disease in one week.

**Methods:** 118 children with JIA (47 with polyarticular, 43 with oligoarticular, 28 with uveitis-associated (JIA-u) subtypes) aged from 2 to 18 years were examined, including 77 girls and 41 boys. The JADAS27 questionnaire was used to assess the disease activity, and the PedsQLTM 4.0 Generic Core, validated for Ukraine, was used for the QoL level. The general result of the questionnaire was analyzed, as well as the emotional subscale of the questionnaire. The questionnaire consists of 23 questions and has 4 age versions (for children from 2 to 18 years). The evaluation of the obtained results was performed according to the method of the Likert scale, where the result of 100 points means the best level of QoL. Disease activity was defined as high at scores above 4.2 for oligoarthritis and 8.5 for polyarthritis. Statistical processing of the material was performed using Microsoft Excel 2016.

**Results:** The disease activity according to JADAS27 in the subgroup with polyarthritis was 6.4 ± 0.6 points, with oligoarthritis - 3.5 ± 0.5 points, with JIA-u - 2.6 ± 0.5 points. It was high in 12 patients with polyarthritis, in 10 - with oligoarthritis, and in 9 - with JIA-u.

The overall rate of QoL in the group of children with JIA for 7 days of observation in the hospital was at the level of 70.9 ± 1.4, the emotional component is slightly lower - 66.3 ± 1.75. The lowest rates of total QoL were observed in the group with polyarthritis - 65.6 ± 2.9 (p≤0.01). The tendency to decrease the total QoL was also observed in the group with JIA - 69.4 ± 2.3, while children with oligoarthritis had better indicators - 77.9 ± 2.3. The assessment of the emotional component of QoL differed from the overall result, showing the worst level of emotional state in children with JIA-u - 56.6 ± 2.2 (p≤0.01). The indicators of emotional state in the groups with polyarthritis and oligoarthritis corresponded to the level of the general index of QoL (65.8 ± 3.1 and 73.3 ± 2.3, respectively). Given the importance of disease activity, the association of activity in the group as a whole with the overall QoL score and emotional component (-0.409 and -0.197, p≤0.05, respectively) was assessed.

**Conclusion:** The most significant reduction in QoL in children with polyarticular and uveitis-associated subtypes of JIA was found. The emotional component of QoL was the lowest in patients with JIA-u and did not correlate with disease activity.

**Disclosure of Interest**: None declared

### P417. Exploring art therapists perceptions of using art therapy with children managing juvenile idiopathic arthritis: a vignette study

#### D. Ghio, U. Toher, E. Griffiths, T. Epton

##### Division of Psychology and Mental Health , University of Manchester, Manchester, United Kingdom

###### **Correspondence:** D. Ghio

**Introduction:** Art Therapy, a type of psychotherapy using the creation of images and objects to improve wellbeing and facilitate communication about complex issues which is especially useful for younger populations. This type of therapy has grown in popularity to support children with long term conditions including Juvenile Idiopathic Arthritis (JIA). Managing JIA can be explored during Art Therapy using goal-setting techniques, but little is known about the processes of change occurring during therapy with different goals and what are the barriers and facilitators to getting children to participate in Art Therapy and attain their goals.

**Objectives:** This current study has two objectives; (a) to explore the perceptions of Art Therapists on goal-based processes in Art Therapy, with children managing their JIA; (b), to identify the perceived barrier and facilitators for children when accessing the therapy and to attain their goals.

**Methods:** Semi-structured interviews were conducted with three art therapists exploring two vignettes of hypothetical children aged 12 presenting with either physical-emotional issues (anxiety with hospital procedures and appointments and needle phobia) or social-emotional issues (social isolation from peers, lonely and low mood) in relation to JIA. These were developed from qualitative research of the psychosocial issues faced by children with JIA. The transcripts were analysed using framework analysis to identify barriers and facilitators.

**Results:** In total there were six vignette cases discussed that explored different approaches to either physical-emotional issues or social-emotional issues. In general, there were barriers to partaking in the therapy, due to the nature of the condition e.g. flare ups of pain and fatigue, or due to the process of the referral which is either through the parents or healthcare professionals. In the latter situation, the child may need more support in setting their own goals. These goals, the Therapists explained, needed to be flexible and adaptable for the children because of fluctuations in the child’s environment that may be out of their control. Regarding the different issues there were specific barriers for example the coordination with school but also facilitators for example identifying own skills and developing problem solving skills.

**Conclusion:** Art Therapy can be a useful for providing an outlet for exploring both physical-emotional issues and social-emotional issues empowering children with skills for problem solving in their management of JIA. The Art Therapy needs to be flexible and adaptable in how it is delivered but there is also a need to be flexible with goal setting to take into consideration the context of these behaviours that are dependent on other factors (e.g. school). Further work into children and parents’ experiences of Art Therapy will be useful to understand how children implement these skills and incorporate them in their everyday life.

**Patient Consent:** Not applicable (there are no patient data)

**Disclosure of Interest**: None declared

### P418. Survey of experienced knowledge among adolescents with JIA using the meps-questionnaire

#### A. Haavisto Olow^1^, A.-L. Lagerkvist^1,2^

##### ^1^Queen Silvia Children’s Hospital, ^2^Health and Rehabilitation, Gothenburg University, Gothenburg, Sweden

###### **Correspondence:** A. Haavisto Olow

**Introduction:** As a health care team we prepare adolescents with juvenile idiopathic arthritis (JIA) for transition into adult care and promote the adolescents’ ability to self-manage. Disease education and information is an important part of this and should be adapted to the adolescents age and maturity. To know what to target in the education we need to find out how the adolescents experience their knowledge.

**Objectives:** The objectives were to explore what knowledge adolescents with JIA have about their disease and if the knowledge differed when divided according to gender or subtype of JIA.

**Methods:** All adolescents with JIA, aged 13-17 years, who were followed at Queen Silvia Children’s Hospital were sent the youth version of the Medical Issues, Exercise, Pain, Social Support questionnaire (MEPS). The adolescents gender, their subtype of JIA and their ongoing medical treatment were noted from patient records.

**Results:** Thirty-seven of the 70 adolescents invited chose to participate, 73% were female. The results for the medical issues domain showed the adolescents experience an average knowledge of medical background of JIA (median 48 mm), a low knowledge of joint anatomy (median 31 mm) and of blood samples (median 18 mm). It also showed that most adolescents don’t fear giving blood sampling (median 4 mm) or getting corticosteroid injections (median 21 mm). In the exercise domain the adolescents answered that they have a high participation in both physical education in school (median 86 mm) and exercise in the spare time (median 90 mm). The pain domain showed a high self-efficacy for symptom management (median 72 mm) and pain alleviation skill (median 84 mm). In the social support domain the adolescents report a low value for exchanging experiences with other youth with JIA (median 3 mm). Further, they have low knowledge of community support measures (median 8 mm). When compared between genders the boys reported a less sufficient knowledge of signs of joint inflammation than girls (median 23 mm vs. 54 mm, p=0.023). The boys also gave lower value to JIA-contacts than girls (median 13 mm vs. 52 mm, p=0.007). Significant differences were seen in the comparison between subtypes in knowledge of medical background (p=0.016), joint inflammation (p=0.012), signs of joint inflammation (p=0.042), and value of JIA-contacts (p=0.038). A pair-wise comparison between all subtypes revealed significant differences between adolescents with enthesitis-related and polyarticular JIA, where those with enthesitis-related JIA experienced their knowledge as significantly closer to insufficient on knowledge of medical background (median 12 mm and 81 mm, p=0.017), joint inflammation (median 16 mm and 81 mm, p=0.01), and signs of joint inflammation (median 46 mm and 97 mm, p=0.035).

**Conclusion:** This study shows that the adolescents’ knowledge of their disease differs in different domains and that there are significant differences in knowledge between girls and boys as well as between different subtypes on certain items. This information is helpful for the health care team working with the adolescents with JIA as we can use it to further develop the education in areas where the knowledge is experienced as inadequate.

**Patient Consent:** Yes, I received consent

**Disclosure of Interest**: None declared

### P419. Factors associated with poor medication adherence in children with rheumatic diseases

#### R. Manatpreeprem, S. Vilaiyuk, B. Lerkvaleekul

##### Pediatric department, Faculty of Medicine Ramathibodi Hospital, Mahidol University, Bangkok, Thailand

###### **Correspondence:** R. Manatpreeprem

**Introduction:** Children with rheumatic diseases usually require long-term treatment and multiple medications, which increases the risk of noncompliance. Failure to take regular medications leads to poorer health outcomes. The Pediatric Rheumatology Adherence Questionnaire (PRAQ), a tool for assessing medication adherence in rheumatic patients, could effectively detect poor medication adherence in these individuals.

**Objectives:** This study aimed to determine the associated factors for poor medication adherence in children with rheumatic diseases.

**Methods:** This was a cross-sectional study design. Patients with rheumatic diseases, who had taken at least one medication and had been followed up at Pediatric Rheumatology Clinic with their caregivers, were included in the study. Patients with poor medication adherence were characterized as those who had taken less than 80% of their drugs, as determined by the pills count pharmacy refill technique. The original PRAQ was translated and validated into the Thai version and was completed by caregivers and/or literacy patients over the age of thirteen. Interviewing for additional obstacles to taking medications was conducted. Descriptive and logistic regression analyses were performed.

**Results:** From a total of 210 patients, 52.9% had juvenile idiopathic arthritis (JIA) and 47.1% had connective tissue diseases. The patients’ mean age was 14.1±4.7 years, with a median (IQR) disease duration of 4.3 (2.1-7.0) years. With a mean total PRAQ score of 11.0±3.5, poor medication adherence was discovered in 24.8% of patients. Polyarticular JIA and enthesitis-related arthritis (ERA) were two JIA subtypes that associated with poor treatment adherence (OR 4.99 ,95%CI 1.45-17.21, and OR 4.95, 95%CI 1.56-15.71, respectively). Patients who had disease duration longer than 5 years (OR 2.88, 95%CI 1.11-7.46), PRAQ scores ≥ 10 (OR 2.88, 95%CI 1.11-7.46), and forgetting to take medications (OR 15.48, 95%CI 5.66-42.33) were the predictors of poor drug adherence.

**Conclusion:** Poor medication adherence was found in one-fourth of children with rheumatic illnesses, particularly polyarticular JIA and ERA. Inadequate adherence was related to longer disease duration, high PRAQ scores, and forgetting to take prescriptions.

**Patient Consent:** Yes, I received consent

**Disclosure of Interest**: None declared

### P420. Present and accounted for: the workplace productivity loss for parents of children with juvenile idiopathic arthritis

#### L. Grazziotin^1^, G. R. Currie^1^, S. Cantarutti^1^, S. M. Benseler^1^, J. F. Swart^2^, M. M. Kip^3^, M. J. IJzerman^4^, M. Twilt^1^, S. J. Vastert^2^, N. M. Wulffraat^2^, R. S. Yeung^5^, D. A. Marshall^1^ on behalf of on behalf of UCAN CAN-DU and UCAN Cure Consortium

##### ^1^University of Calgary, Calgary, Canada, ^2^University Medical Center Utrecht, ^3^University Medical Center Utrecht, Utrecht, ^4^University of Twente, Enschede, Netherlands, ^5^University of Toronto, Toronto, Canada

###### **Correspondence:** M. M. Kip

**Introduction:** Parents of children with juvenile idiopathic arthritis (JIA) face impacts on their time at work and daily life activities due to caregiving for their children. Work productivity impacts include both absenteeism (missing work due to their child’s JIA) and presenteeism (reduced productivity while at work).

**Objectives:** To estimate changes in work commitment, impact on work (presenteeism and absenteeism) and usual activities for caregivers of children with JIA.

**Methods:** Productivity loss was captured in standardized instruments, including the validated Work Productivity and Activity Impairment Questionnaire (WPAIQ): Specific Health Problems. The UCAN CANDU is an on-going prospective, multicentre study including all pediatric rheumatology clinics in Canada and the Netherlands which focused on personalized care strategies in JIA through biological monitoring systems. Caregivers participating in the UCAN CANDU study also completed questionnaires related to demographic characteristics. Results were reported using means, standard deviations, and proportions.

**Results:** 244 caregivers were included in this study. Approximately 12% of primary parent respondents and 3% of their spouses/partners reported a change in their work commitment. The mean absenteeism and presenteeism rates reported for any reason were 15.8% and 18.1%, respectively. The mean overall work impairment reported was 28.7%, mostly due to their child’s JIA (25.8%).

**Conclusion:** JIA creates considerable socioeconomic burden affecting paid work and usual activities for parents. Our study shows that caregivers’ work impairment can primarily be attributed to their child’s JIA, and that the productivity impact extends beyond absence from work, into impaired productivity of employees while at work. The societal and economic implications of this require further study.

**Patient Consent:** Not applicable (there are no patient data)

**Disclosure of Interest**: None declared

### P421. The knowledge, attitudes and practices of non-specialist healthcare workers in Kenya towards paediatric rheumatology

#### A. Migowa^1^, S. bernatsky^2^, A. Ngugi^3^, H. Foster^4^, P. Muriuki^5^, A. Lusambili^5^, S. Luchters^6^

##### ^1^bPaediatrics and Child Health, Aga Khan University Medical College East Africa (Nairobi,Kenya), Nairobi, Kenya, ^2^Rheumatology, McGill University Health Centre, Montreal, Canada, ^3^Populations Health, Aga Khan University Medical College East Africa (Nairobi,Kenya), Nairobi, Kenya, ^4^Paediatrics, New Castle University, New Castle, United Kingdom, ^5^Population Health, ^6^Institute of Human Development, Aga Khan University Medical College East Africa (Nairobi,Kenya), Nairobi, Kenya

###### **Correspondence:** A. Migowa

**Introduction:** The World Health Organization (WHO) has categorized musculoskeletal diseases into over 200 entities which are still the leading cause of disability globally(1). The prevalence of various childhood rheumatic diseases varies across different regions of the world, with the prevalence of Juvenile Idiopathic Arthritis (JIA) ranging from 0.07 to 10/1000 children and that of Systemic Lupus Erythematosus (SLE) from 0.4 to 0.6/100 000 children(2). Epidemiological data from Kenya about childhood rheumatic diseases are lacking, but on the basis of the above population data, we could estimate 300,000 children with JIA alone.

Dysfunction is not only related to disease severity, but also on illness perception(1). In Sub-Sahara Africa, as a result of other competing health interests, musculoskeletal health is often not prioritized. Due to the paucity of skilled personnel to manage paediatric rheumatic diseases, efforts towards reducing this disparity need to be bolstered. Consequently, there is an urgent need to scale-up pediatric rheumatology knowledge and skills among non-specialist clinicians by tapping into digital technology to optimize remote learning due to the paucity of paediatric rheumatologists to bridge the gap in clinical care.

**Objectives:** The objective of this study is to understand the knowledge, attitudes and practices of non-specialist clinicians towards paediatric rheumatology to help inform development of an intervention aimed at improving their clinical skills in diagnosis and management of paediatric rheumatology conditions.

**Methods:** Online focus group discussions were conducted among first tier community health workers (clinical officers), nurses, general practitioners, and pediatricians to ascertain their knowledge, attitudes and practices towards pediatric rheumatology. The focus groups were recorded and the recordings transcribed. The data coders read the transcripts and thematic coding carried out.

**Results:** A total of 68 participants were recruited of whom 77.9% ( 53 of 68) were female with a mean age of 36 years . Among participants involved 39.7% (27) were paediatricians, 14.7% (10) general practitioners, 17.6% (12) nurses , 16.7%(11) primary community health workers and 10.2% (7) pursued other specialities. The focused group discussions revealed a paucity of knowledge among non-specialist healthcare workers towards paediatric rheumatology. This often leads to confusion and yields an attitude of anxiety and fear upon encountering paediatric rheumatology patients, which is associated with delayed diagnosis, misdiagnosis and inappropriate management.

**Conclusion:** Non specialist healthcare workers expressed paucity in knowledge of pediatric rheumatology which impacts negatively on their attitude and practice in this discipline. In order to improve their knowledge, attitudes and practices participants proposed that algorithms for diagnosis and management should be availed alongside continuous medical education and ongoing mentorship, either virtually or face to face.

**Disclosure of Interest**: None declared

### P422. Effects of a physical activity promotion program and workout in teenagers with juvenile idiopathic arthritis

#### E. Prato^1^, M. Pirinu^1^, E. marrani^2^, I. Maccora^2^, M. V. Mastrolia^2^, I. Pagnini^2^, L. Baroni^1^, G. Simonini^2,3^

##### ^1^Rehabilitation Unit, ^2^Pediatric Rheumatology, Aou meyer, ^3^NEUROFARBA department, University of Firenze, Firenze, Italy

###### **Correspondence:** E. Prato

**Introduction:** Despite physical activity represents a therapeutic agent for JIA[1,2] several studies report that JIA adolescents less active than healthy peers[3]. This can cause long-term negative effects, and as matter of fact inactive lifestyle is a risk factor[4,5]. Current evidence points out the need to increase the level of physical activity and limit sedentary behaviors in this population. Different approaches have been used[1], but none of them was clearly able to increase and improve daily activities and endurance of teenagers with JIA.

**Objectives:** The study aims to assess the effect on increasing physical activity of a multifactorial protocol including an individualized program to reduce sedentary behavior in routine daily life associated with a personalized endurance enhancement program, administered through telemedicine devices.

**Methods:** Each participant was assessed at baseline (T0), followed by a three-month training program, supervised via telemedicine by a physiotherapist associated with monthly conversations to encourage the identification of individual strategies for increasing physical activity, at the end of which they were reassessed (T3m) by a blinded researcher. Enrolled subjects were then asked to continue the program, independently, without supervision, for additional 3 months, and then again reassessed (T6m). The outcome measures selected were: Habitual Activity Estimation Scale (HAES), 10 Metres Shuttle Walk Test Modified (10MSWT), process-oriented checklist to assess Fundamental Movement Skills (FMS). In addition, two individualized goals were identified for each participant through Goal Attainment Scaling (GAS), the attainment of which was assessed at T6m. For the statistical analysis was used the Mann-Whitney test.

**Results: Seven teenagers with JIA were recruited (all female, median age 15 years old, range 11-18). HAES score resulted significant increased between T6m and T0 in relation to the total activity during weekdays. The 10MSWT shows a significant improvement between T3m and T0, that was kept at T6m. FMS showed a significant improvement between T6m and T0. All participants achieved the objectives defined with the GAS (Table I).**

**Conclusion:** This study reported a positive change in all the observed variables. The program appears feasible and well accepted by adolescents and their families. It allowed participants to get involved in overcoming individual obstacles, thus achieving a greater level of physical activity. It can be assumed that the proposed protocol may represent an integrative proposal in the physiotherapeutic treatment of adolescents with JIA. Further studies, in larger cohort and long-term follow-up, are needed to assess the maintenance the achieved results over time.

**Patient Consent:** Not applicable (there are no patient data)

**Disclosure of Interest**: None declared

**Table 1 (abstract P422).** Values obtained from evaluations and P value where there is a statistically significant difference

		T0	T3m	T6m	P value
**HAES TA WD (mean value)**		5,1	6,8	8,3	DT6m-T0 0,043
**10MSWT** **(mean value)**		671	860	753	DT3m-T0 0,018 DT6m-T0 0,018
**FMS** **(mean value)**		2,6	4,9	4,4	DT3m-T0 0,017 DT6m-T0 0,026
**GAS** **(median value)**		/	/	1	

*HAES TA WD : Habitual Activity Estimation Scale – total activity during weekdays (hours/day) ; 10MSWT : 10 Metres Shuttle Walk Test Modified (metres); FMS: process-oriented checklist to assess Fundamental Movement Skills****;****GAS: Goal Attainment Scaling*

### P423. Special aspects of the communicative and emotional-volitional sphere in pediatric patients with monogenic autoinflammatory diseases (FMF, CAPS, TRAPS)

#### E. S. Fedorov^1^, N. Y. Stepanenko^1^, S. V. Feoktistova^2^, S. Salugina^1^, V. Matkava^1^, I. E. Fedorov^2^

##### ^1^Pediatrics, V.A. Nasonova Research Institute of Rheumatology, ^2^Humanities Institute, Russian New University, Moscow, Russian Federation

###### **Correspondence:** N. Y. Stepanenko

**Introduction:** Monogenic autoinflammatory diseases (AIDs) are systemic diseases leading to severe functional abnormalities of various organs and systems, which may result in a malfunction in the psychological sphere.

**Objectives:** to identify special aspects and differences in the emotional-volitional and communicative spheres in pediatric patients with monogenic AIDs.

**Methods:** 41 children with AIDs were included into the study: FMF - 15 (boys - 8, girls - 7); CAPS - 17 (boys - 9, girls - 8); TRAPS - 9 (boys - 5, girls - 4) aged 7 to 17 years inclusive. There were no significant differences in terms of age between the studied nosologic groups. The diagnosis in all the patients was confirmed on the ground detection of the corresponding pathogenic mutations. To assess the emotional sphere, the following methods were used: a clinical conversation; emotional and communicative sphere (8-color Luscher test; CMAS (adaptation by A. Prikhozhan); Spielberger-Hanin anxiety test, drawings “non-existent animal” and “house-tree-man”).

**Results:** TRAPS: violations in terms of all studied indicators. There were revealed communication disorders (88.9%), a tendency to form neurotic fears (66.7%), reduced social adaptation (55.5%), signs of aggression and a high level of personal anxiety (33.3%). The level of aggression and personal anxiety was higher in girls than in boys (F/M= 50%/20%). Communication disorders and a tendency to form neurotic fears were more characteristic of boys (F/M= 75%/100% and 50%/80%, respectively).

FMF: high rates of communication disorders (73.3%), reduced social adaptation (46.7%), neurotic fears and a high level of personal anxiety (26.7%), signs of aggression (13.3%). A reduced level of social adaptation is characteristic of girls (F/M=57.1%/37.5%), boys are more likely to have signs of aggression (F/M=0/25%). Disorders in the communicative sphere are distributed evenly.

CAPS: a relatively intact group. The highest indicators compared to other groups were detected only in terms of criterion of susceptibility to form neurotic fears (58.8%). There were also registered communication disorders (41.2%), reduced social adaptation (29.4%), a high level of personal anxiety (23.5%), signs of aggression (5.9%). Girls are more likely to have reduced social adaptation (F / M = 41% / 33.3%), susceptibility to form neurotic fears (F / M = 62.5% / 55.5%), a high level of personal anxiety (F / M =37.5% / 11.1%). Boys are more likely to have communication disorders (F/M= 25%/55.5%) and aggression (F/M= 0/11.1%).

**Conclusion:** most often, disorders in the emotional-volitional and communicative spheres compared with other studied diseases are registered in patients with TRAPS; at that, boys are more likely to have communication disorders and form neurotic fears, while girls are characterized by high level of personal anxiety and aggression. Communication disorders and reduced social adaptation are characteristic for patients with FMF; reduced social adaptation is more common in girls. Patients with CAPS are the most intact in the studied areas, having disorders only in terms of formation of a tendency to neurotic fears, which is more common in girls.

**Patient Consent:** Yes, I received consent

**Disclosure of Interest**: None declared

### P424. Procedure related pain: successful reestablishment of parent-administered subcutaneous injections at home using an education program: a report of three cases

#### L. H. Weidlich, R. Hald, S. Reinholdt

##### Paediatric Rheaumatology, Rigshospitalet, 2100 Copenhagen E., Denmark

###### **Correspondence:** L. H. Weidlich

**Introduction: Introduction:** We have experienced an increase in the number of patients for whom subcutaneous injections at home has become difficult and traumatic for both children and parents. This sometimes results in cessation of treatment and flare-up of arthritis symptoms. Since 2018 we have been developing a structured education program inspired by Julie E. Maclaren’s article “Interventions for paediatric procedure-related pain in primary care” ^1^.

**Objectives: Objectives:** To reestablish parent-administered injections at home through an education program.

**Methods: Methods:** Case report of three cases. We used a structured education program to ensure that parents gain necessary knowledge and skills to administer and cope with subcutaneous injections at home. Children 0-18 years with juvenile idiopathic arthritis in subcutaneous treatment and their parents are the target group. The program consists of consultations facilitated by a nurse with dialog, training, and various tools to educate the patient to cope with the pain and the parents to support the child during the procedure.

**Results: Results:**

Patient 1: An eight-year-old girl. The subcutaneous injections at home take hours of negotiating and is causing the patient pain. We started with a baseline consultation uncovering the difficulties and then we made some adjustments to the methods used by changing from pen to syringe. Two additional consultations were needed with further, minor adjustments made before the family tried to home administer the medication again. At a follow-up contact three months later the family confirmed that the injections are now easily administered without any physical or psychological difficulties.

Patient 2: A thirteen-year-old boy. The boy was nervous, scared, and overthinking the subcutaneous injections. At the baseline consultation we established that using local anaesthetic and a procedure agenda as a tool could be the solution for him. The procedure agenda was tested in the paediatric department successfully and the procedure lasted less than ten minutes. The boy did not find it too painful and after three additional consultations where same agenda was used, they were ready to try giving the subcutaneous injection at home again. At a follow-up contract three months later, the family confirmed that giving the subcutaneous injections still was working well at home.

Patient 3: A seven-year-old boy. The boy cannot cooperate and getting medicine has become impossible. At the baseline consultation we established that using Nitrous Oxide and local anaesthetic could make a difference. The plan was successfully used in the outpatient clinic. The boy is challenged due to an attention deficit hyperactivity disorder. The family have tried giving the subcutaneous injection at home, but it did not work out, so he is still getting the subcutaneous injections in the outpatient clinic but without using Nitrous Oxide and we are working towards mowing the treatment home again.

**Conclusion: Conclusions:** Facilitated using a structured educational program we re-established the home administered subcutaneous treatment for two of the patients and the last patient is one his way.

**Patient Consent:** Yes, I received consent

**Disclosure of Interest**: None declared

### P425. Prevalence and factors associated with depression and anxiety in patients with juvenile idiopathic arthritis

#### N. Yothakol^1^, S. Chantaratin^2^, M. Sukharomana^1^, S. Charuvanij^1^

##### ^1^Division of Rheumatology, Department of Pediatrics, ^2^Division of Child and Adolescent Psychiatry, Department of Pediatrics, Faculty of Medicine Siriraj Hospital, Mahidol University, Bangkok, Thailand

###### **Correspondence:** N. Yothakol

**Introduction:** Juvenile idiopathic arthritis (JIA) is chronic inflammatory arthritis in children that can cause significant physical, social functioning and psychological impairment. Common psychological problems in childhood and adolescents are depression and anxiety disorders, especially those with chronic illnesses.

**Objectives:** The aims of study were to describe prevalence of depression and anxiety and to investigate factors associated with depression and anxiety in patients with JIA.

**Methods:** A cross-sectional study was conducted in JIA patients, aged between 8-17 years at the Pediatric Rheumatology clinic, Faculty of Medicine Siriraj hospital, Mahidol University, Bangkok, Thailand from July 2020 to April 2021. JIA was classified by the International League of Association for Rheumatology criteria. The demographic data, JIA variable core sets and juvenile arthritis disease activity score were collected. The depression and anxiety were assessed by the Thai version of Children’s Depression Inventory (CDI) and the Screen for Child Anxiety Related Disorders (SCARED) questionnaires. The CDI score ≥ 15 was defined as significant depressive symptoms. The SCARED score of ≥ 25 indicated the clinically significant anxiety.

**Results:** Seventy patients with JIA; 39 males (55.7%) and 31 females (44.3%) were included in this study. The mean age (SD) at the time of assessment was 12.3±2.6 years. The JIA subtypes were as follows: 26 (37.1%) patients with enthesitis-related arthritis, 19 (27.1%) patients with systemic JIA, 8 (11.4%) patients with polyarticular JIA rheumatoid factor (RF)+, 7 (10%) patients with polyarticular JIA RF-, 6 (8.6%) patients with oligoarticular JIA and 4 (5.7%) patients with undifferentiated JIA. The median follow-up time was 38 (IQR 10-67.2) months. Fifty-one (72.9%) patients had inactive disease. The anxiety symptoms were found in 35 (50%) patients; 26 (74.3%) patients had separation anxiety symptoms, 13 (34.3%) patients had social anxiety symptoms and 11 (31.4%) patients had school anxiety symptoms. SCARED score had significant positive correlation with Childhood Health Assessment Questionnaire score (r=0.311, *p*=0.009). Eight (11.4%) patients had depressive symptoms. Receiving biologic treatment was significantly associated with depressive symptoms at adjusted OR 18.08 (95% CI: 2.28-143.29), *p*=0.006 by multivariable logistic regression analysis. Additionally, CDI score had significant positive correlation with SCARED score (*r*=0.526, *p*=<0.001).

**Conclusion:** Half of patients with JIA had mood symptoms. Anxiety was more prevalent than depression. Psychological assessment and appropriate interventions are necessary as part of clinical care for patients with JIA.

**Patient Consent:** Yes, I received consent

**Disclosure of Interest**: None declared

## Poster session: e-health and digital health applications

### P426. Parent views on telemedicine in pediatric rheumatology: a survey study

#### G. Kavrul Kayaalp, F. G. Demirkan, Ö. Akgün, A. Tanatar, F. Çakmak, N. Aktay Ayaz

##### Pediatric Rheumatology, İstanbul University, Faculty of Medicine, İstanbul, Turkey

###### **Correspondence:** N. Aktay Ayaz

**Introduction:** The expanding use of telemedicine due to the emerging needs for remote visits after the COVID-19 pandemic has led many patients with chronic diseases to seek alternative ways for follow-ups, even beyond the pandemic era.

**Objectives:** It is aimed to investigate the demands and opinions of the parents of children with rheumatic diseases towards telemedicine applications and to examine the factors influencing telemedicine preference.

**Methods:** A single-center, cross-sectional, web-based survey study was conducted in a university hospital. The target group was the parents of the children with rheumatic diseases of any kind. The survey included a total of 45 questions that were grouped into four different sections. Sections were on (1) sociodemographic data, (2) disease-related characteristics, (3) transportation to the clinic, (4) use of the internet, and (5) opinions on telemedicine.

**Results:** A total of 205 parents have completed the survey. Diagnoses of the patients were periodic fever syndromes and autoinflammatory diseases (56.1%), juvenile idiopathic arthritis (28.8%), systemic connective tissue diseases (9.7%), and vasculitis (5.4%).

The most common way of transportation was public transport (77%). Only 44.9 % of patients were able to arrive at the clinic in less than one hour. Of the parents, 84.9% use the internet for social media, 45.9% for the medical information, daily. An application that enables video calling was available on the smart devices of 77.5% of the parents. Only 3.8% of parents had previous telemedicine experience.

Thirty percent of respondents missed a follow-up appointment at least once over the last year. The most common reasons for missing an appointment were the fear of COVID-19 exposure (29.6%), missed school days (14.8%), and unwillingness to come to the hospital for follow-up when the child had no complaints (12.3%). Of the parents, 70.7% stated that they would prefer telemedicine if it becomes available. Sixty-five percent of parents reported that telemedicine examinations can be useful for routine check-ups when their children have no complaints, 64% when they have new complaints, 49% when they want to reach their physicians to ask questions about their diseases, and 47% for a few times when it is not possible to come to the outpatient clinic. The frequency of telemedicine preference was found to be related to the education level of the parents (The preference rate increases with the level of education) (p=0.025), missing at least one outpatient clinic visit over the last year (p=0.002), and daily use of the internet for social media (p=0.035).

The most common reasons for preferring telemedicine over face-to-face examinations were reducing missed school days (74.1%), reducing the risk of COVID-19 exposure (73.2%), and saving time (66.3%). Easier reach to the physician (67.3%) and more regular follow-ups without misses (66.8%) were the most commonly reported advantages. Although the telemedicine preference rate was high, the rate of believing that parents could receive the same quality of care as they were examined face-to-face remained at 36.1%. Of respondents, 38% have concerns about a wrong treatment decision, and 24.9% about the privacy of personal data during telemedicine visits.

**Conclusion:** Telemedicine applications are demanded by the parents of children with rheumatic disease. It can be considered as an alternative option, especially in the follow-up of patients who do not come to their appointments regularly. An increase in telemedicine experience in the field of pediatric rheumatology may provide a better evaluation of the effectiveness of telemedicine methods.

**Disclosure of Interest**: None declared

### P427. Comparison the effectiveness of online aerobic dance exercises versus physical activity counseling in patients with childhood familial mediterranean fever

#### A. Albayrak^1,2^, N. Arman^3^, E. Tarakci^3^, O. Kasapcopur^4^

##### ^1^PhD Program of Physiotherapy-Rehabilitation, Graduate Education Institute, Istanbul University-Cerrahpasa, ^2^Department of Physiotherapy and Rehabilitation, Istanbul Kent University, Faculty of Health Science, ^3^Department of Physiotherapy and Rehabilitation , Istanbul University-Cerrahpaşa, Faculty of Health Science, ^4^Department of Pediatric Rheumatology, Cerrahpaşa Medical School, Istanbul University-Cerrahpaşa, Istanbul, Turkey

###### **Correspondence:** A. Albayrak

**Introduction:** Familial Mediterranean Fever (FMF) is the most common autoinflammatory disease that its characterized by self-limiting attacks of fever, serosal inflammation and arthritis generally developing in childhood. Symptoms associated with FMF in children and adolescent include pain, fatigue, muscle weakness, myalgia that results in activity and participation restrictions. Physical activity and exercise are key components in management of children and adolescents with rheumatism.

**Objectives:** The aim of the study was to compare the effectiveness of the online aerobic dance exercises (OADE) and physical activity counseling (PAC) program with the PAC program in patients with childhood FMF (cFMF).

**Methods:** 20 patients (8-18 years) with cFMF were taken account in the study. Randomly, related patients were divided as Group I (OADE and PAC), and Group II (PAC). In the Group I, the aerobic dance exercises program was performed online for 8 weeks, three days a week, and 60 minutes a day. General physical activities were recommended, and motivational interviews were organized for both groups.

The functional capacity of the patients by the 6-Minute Walk Test, the physical fitness by the FitnessGram Physical Activity Test Battery, the fatigue symptoms by the PedsQL Multidimensional Fatigue Scale, pain at rest by the Visual Analog Scale, the quality of life by the Familial Mediterranean Fever Quality of Life Scale were evaluated. All assessments were performed before and after treatment.

**Results:** Half of the patients were female and the mean age was 13.35±3.45 years. After treatment aerobic capacity, and physical fitness level improvements were observed in Group I. Additionally, the mean change in walking distance in Group I was 64±58.63 meters. Also, the improvements in physical fitness and pain scores after treatment were statistically significant in Group II. (p<0,05). In the comparison between groups, the changes in all parameters after treatment were not statistically significant, except for the increase in pain scores in Group II (p>0,05).

**Conclusion:** This study is the first study to pay attention physical activity and exercise programs in patients with cFMF. It has been found that OADE and PAC program provide clinically significant gains in terms of functional capacity and physical fitness, and provide effective results on pain compared to only PAC program and it has been shown that online aerobic dance and physical activity counseling are applicable in the treatment of cFMF.

**Disclosure of Interest**: None declared

### P428. Is it possible to evaluate the immediate effects of postural correction and weight-bearing training on plantar pressure and balance using pedobarography in a patient with juvenile idiopathic arthritis and obstetric brachial plexus palsy?

#### N. Arman^1^, F. G. Demirkan^2^, N. Aktay Ayaz^2^

##### ^1^Department of Physiotherapy and Rehabilitation, Faculty of Health Science, Istanbul University-Cerrahpasa, ^2^Department of Pediatric Rheumatology, İstanbul Faculty of Medicine, İstanbul University, Istanbul, Turkey

###### **Correspondence:** N. Arman

**Introduction:** Juvenile idiopathic arthritis (JIA) is the most frequent chronic rheumatic disease of childhood with arthritis of one or more joints accompanied by various musculoskeletal problems. The literature shows that the pedobarography is useful for the examination of foot biomechanics.

**Objectives:** The aim of this case study was to evaluate the immediate effects of postural correction and weight-bearing training on plantar pressure and postural control using pedobarography in a patient with JIA and obstetric brachial plexus palsy (OBPP).

**Methods:** We present a case of a 16-year-old boy, born to parents related by first-degree consanguinity and was diagnosed with polyarticular juvenile idiopathic arthritis at the age of 3 years. He has not received any medication for about 4 years. There was no history of trauma and no family history of rheumatic disease. The patient had muscle weakness and deformity in the right arm due to a fracture of the clavicle and OBPP during the delivery process. On examination, arthritis was found in the left 2nd proximal interphalangeal joint and bilateral elbows, knees, wrists and ankles. There were no significant abnormalities in other systems. It was also observed that the patient had been in a depressive mood for a while due to decreased mobilization due to deformities and pain. He can walk normally, with no further complaint of joint pain. However, he reported complaints of loss of balance and tires while walking. He cannot use his right arm in daily life activities, he prefers to keep his right hand in his pocket while walking most of the time. Besides, no arm swing and kyphotic posture were observed during walking. The patient, who had never exercised before, was given a correct posture and weight transfer exercises training including proprioceptive cues and visual imagery to increase the patient’s balance and postural control skills for 20 minutes. Pedobarographic analysis including static, dynamic and balance tests was performed before and 15 minutes after training. It was utilized for the evaluation of static analysis parameters; surface, load % and P.max for foot, dynamic loading parameters; max load %, avg. speed and duration of phase for the gait cycle as dynamic analysis, evaluation of balance parameters; sway surface, sway length and average speed mm/s for sway test.

**Results:** The results of the two assessments were presented in Table 1. Although no significant change was obtained in the static analysis results, clinically significant results were found in the dynamic analysis and Sway Test.

**Conclusion:** We found clinically improvements in plantar pressure and postural control as an immediate effect after exercise training in a patient with JIA and OBPP. These results may indicate that the patient learned to use his muscles correctly due to the postural awareness gained by the exercise training. Therefore, we consider that the pedobarographic analysis can be a useful tool to objectively identify patient’s problems, make a decision, choose exercises that he/she needs to do and follow the patient’s improvements for the physiotherapists, especially when the patient has complex problems in pediatric rheumatic disease.

**Patient Consent:** Yes, I received consent

**Disclosure of Interest**: None declared

**Table 1 (abstract P428).** The results of pedobarographic analysis of the patient with JIA and OBPP before and after exercise training

Static Analysis	Before training R	After training R	Before training L	After training L
Surface, cm^2^	92	93	72	75
Load, %	61	59	39	41
P.max, gr/cm^2^	760	707	722	608
**Dynamic Analysis for the gait cycle**, Max load, %	85	92	96	100
Avg. Speed, mm/s	678	571	583	528
Duration, ms	251	228	271	251
**Sway Test**, Sway surface, mm	203,41	55,01		
Sway length, mm	900,19	585,49		
Average speed, mm/s	15,10	9,87		

### P429. Acceptability, practicality, and accuracy of the Turkish translation of video pgals in Turkish children

#### Z. Balik, Y. Bayindir, M. Kasap Cuceoglu, S. Sener, E. Aliyev, O. Basaran, Y. Bilginer, S. Ozen, E. D. Batu

##### Pediatric Rheumatology, Hacettepe University, Ankara, Turkey

###### **Correspondence:** Z. Balik

**Introduction:** Telemedicine practices have been adopted in most departments including pediatric rheumatology during the coronavirus pandemic. pGALS (Pediatric Gait, Arms, Leg and Spine/Pediatric Gait, Arms, Leg and Spine) is a simple and practical method developed to evaluate the musculoskeletal system of children. Video pGALS is the form of this examination performed on video to enable the follow-up of children with rheumatic diseases to continue without interruption during the pandemic restrictions.

**Objectives:** This study aims to evaluate the validity, applicability, and accuracy of the Turkish translation of the video pGALS.

**Methods:** Between April and May 2022, patients aged 4-18 years, who were followed up in Hacettepe University Pediatric Rheumatology Department and agreed to participate in the study, were included. The patient’s demographic data, diagnosis, complaints and physical examination findings were noted. Video pGALS was performed by a pediatric rheumatologist. A link has been created via zoom for this examination. While the doctor was connected to the link on the computer in one room, the patient’s parent was connected to this link in the other room. Normal hands-on physical examination was also performed after video PGALS, which formed the gold standard for positive findings. The acceptability (in terms of time and additional distress) of the applied examination method by the patient/patient relative was measured with a visual analog scale (VAS) created with smiling faces. Pain was evaluated with a score between 0-10, 10 being the worst.

**Results:** A total of 102 patients, 56 girls (54.9%) with a median age of 12.41 (min-max; 4.23-17.84) years were included in the study. The median age was 12.41 (min-max; 4.23-17.84) years. Twenty-two (21.6%) patients had active complaints. The most common diagnosis was juvenile idiopathic arthritis (JIA) (25.5%), followed by familial Mediterranean fever (FMF) 19 (18.6%). The median (min-max) pain score was 4 (1-8). The median duration of pGALS examination was 7 minutes (5-13 minutes). Hands-on physical examination was abnormal in 28 (27.4%) patients, whereas video pGALS was abnormal in 25 (24.5%) patients. In 3 (2.9%) patients with abnormal physical examination, we could not detect these findings with video pGALS. These patients had scoliosis, pes planus, and striae on the thigh. All those who were abnormal in video pGALS were also abnormal on physical examination. The median (min-max) VAS score given by children and parents for acceptability of pGALS in terms of duration was 0 (0–6) for both and in terms of additional discomfort was 0 (0–5) for both. The majority of patients/parents found the duration acceptable (98%/99%, respectively) and reported that pGALS caused little or no discomfort (98%/99%, respectively). The sensitivity and specificity of video pGALS for detecting musculoskeletal abnormalities were 93.7% and 100%, respectively.

**Conclusion:** The video pGALS tool is a quick, easy, and acceptable tool for evaluating musculoskeletal problems in children. The Turkish version of video pGALS was acceptable in terms of duration and additional discomfort. It can detect abnormalities and may be used for follow-up of children with rheumatic diseases to continue without interruption during the pandemic restrictions.

**References:**

1. Susan Shenoi, Kristen Hayward, Megan L Curran, *et al*. Telemedicine in pediatric rheumatology: this is the time for the community to embrace a new way of clinical practice. Pediatr Rheumatol Online J. 2020 Oct 31;18(1):85.

2. Ezgi Deniz Batu, Özge Keniş Coşkun, Hafize Emine Sönmez, *et al*. Acceptability and Practicality of the Turkish Translation of Pediatric Gait Arm Legs and Spine in Turkish Children. J Clin Rheumatol. 2017 Dec;23(8):421-424.

**Patient Consent:** Yes, I received consent

**Disclosure of Interest**: None declared

### P430. Experience of using a mobile health application as communication tool between patients and healthcare professionals

#### M. Doeleman, S. de Roock, G. de Joode-Smink, J. Swart, N. Wulffraat

##### Pediatric Immunology and Rheumatology, University Medical Center Utrecht, Utrecht, Netherlands

###### **Correspondence:** M. Doeleman

**Introduction:** E-health and mobile applications are increasingly popular in paediatric rheumatic diseases. These tools can provide a way to monitor symptoms remotely and communicate with patients or healthcare professionals asynchronously. At our hospital, we have used such a mobile e-health application, Reuma2Go, during daily clinical practice since June 2016. The app allows users to track JIA-related symptoms such as joint pain, fatigue, morning stiffness and daily activities, and gives patients the ability to communicate with our healthcare team through text messages for advice or questions regarding their disease management.

**Objectives:** Evaluate app engagement of users of the mobile application during daily clinical practice from 2018 to 2021. Assess the frequency of relevant topics in app conversations between patients and the healthcare team.

**Methods:** Data was collected via the mobile E-health application (Reuma2Go) from January 2018 to August 2021 as pseudonymized data. App engagement was analysed using descriptive statistics. Text message analysis was performed using the quanteda package in R. Relevant topics for analysis were selected based on overall document-term frequency. Topic frequency was estimated using a dictionary search with topic-specific keywords.

**Results:** From January 2018 to August 2021, a total of 398 unique patients with JIA created an account within the mobile application. In August 2021, 62% of JIA patients visiting the outpatient clinic were using the mobile application. The app was used to track symptoms on average 3291 times a year. User activity each year was highest from March until May and lower during winter and summer school holidays (December-January and July-August, respectively). Over the study period, a total of 1572 conversations were initiated by patients consisting of 4546 messages between patients and the healthcare team. During the first COVID lockdown in the Netherlands, the number of users increased 43% (from 204 to 292) and messaging frequency via the app doubled compared to the same period in the previous year. Topic frequency for the most prevalent conversation topics are presented in Table 1.

**Conclusion:** These results demonstrate our experience of using a mobile health application during daily clinical practice. Topic analysis of conversations between patients and the healthcare team illustrates specific information needs of patients visiting the outpatient clinic. Frequent topics discussed with the healthcare team were JIA-related symptoms and pain, medication, appointments, and vaccination/COVID. During the pandemic, the mobile application has allowed us to monitor our patients remotely and communicate with them asynchronously through in-app text messages. This has provided a continuity of care and maintenance of disease control without the requirement of physical appointments. Furthermore, the app allows patients and caregivers to be more actively involved in their disease management.

**Disclosure of Interest**: None declared

**Table 1 (abstract P430).** Proportion of in-app conversations related to specific topics from January 2018 to August 2021

Year Topic	2018	2019	2020	2021
***JIA-related Symptoms / Pain***	65%	63%	56%	38%
***Covid /*** ***Vaccination***	3%	1%	9%	15%
***Medication / Prescriptions***	23%	17%	27%	26%
***Appointments***	18%	18%	18%	21%
***Total conversations, n***	*329*	*295*	*576*	*372*

### P431. PGALSPLUS: a tool to facilitate assessment and identify exemplar musculoskeletal conditions

#### V. Mercer^1,2^, N. Smith^1^, S. Jandial^1,3^, H. Foster^4^

##### ^1^Newcastle University, Newcastle upon Tyne, ^2^South Tyneside and Sunderland NHS Foundation Trust, South Shields, ^3^Paediatric Rheumatology, Great North Children’s Hospital, ^4^Paediatric Rheumatology, Newcastle University, Newcastle upon Tyne, United Kingdom

###### **Correspondence:** V. Mercer

**Introduction:** Musculoskeletal (MSK) problems in children and young people (CYP) are common and often present to healthcare professionals (HCPs) in primary care and community settings who may not be trained in MSK assessment. Whilst many presentations in CYP are benign and self-limiting, HCPs need to be able to identify serious disease and have guidance on appropriate referrals to minimise delay in diagnosis. pGALS (paediatric Gait, Arms, Legs and Spine) was developed for HCPs to guide them through a simple, quick MSK assessment and has been shown to detect joint and functional problems but doesn’t provide guidance on diagnosis. We aimed to widen the scope of pGALS to support detection of a range of serious MSK conditions and support onward referral pathways

**Objectives:** To create a pGALSplus assessment and assess its feasibility and acceptability in CYP with healthy controls (HC) and exemplar conditions to reflect a spread of MSK pathologies with development based on HCP and patient/carer feedback. Chosen exemplar conditions; Juvenile Idiopathic Arthritis (JIA), Mucopolysaccharidoses (MPS), Muscular Dystrophy (MD), Developmental Coordination Disorder (DCD)

**Methods:** 3-phase mixed methods approach; Phase 1;scoping review of literature and qualitative interviews with expert HCPs focused on key clinical assessments used to inform diagnosis and progress. Results informed the initial ‘pGALSplus’ assessment with iterative development in Phase 2 with an expert working group (including paediatric rheumatologists and physiotherapists, neuromuscualr specialists). Phase 3; testing of pGALSplus in the exemplar condition groups by the research physiotherapist (VM) with feedback from HCPs, CYP and carers, and further validation through expert consensus (international e-survey [n=22] and a virtual dissemination event [n=13])

**Results:** Phase 1 data identified key additional requirements for pGALSplus through literature review and expert opinion. These were added to the pGALS examination to produce a ‘toolkit’. Phase 2; discussion with experts in MSK assessment of CYP, consensus allowed refinement and agreement on how pGALSplus would be used in practice. The agreed toolkit is colour-coded to facilitate identification of exemplar MSK conditions. Phase 3; testing of pGALSplus on 46 CYP (JIA n=10; MPS n=6; MD n=9; DCD n=10; HC n=10). Feedback from the clinician performing pGALSplus demonstrated the assessment to be *achievable* in the target age range (2-10 years), and *quick* (mean = 12.6 minutes to complete) with high levels *of acceptability* from patients and carers. Overall expert feedback (international e-survey; n=22 and stakeholder event; n=13) was positive and enabled iterative development of pGALSplus. Responders deemed pGALSplus to be a very useful addition to pGALS as it enables further assessment relevant to the practice of non specialists and those new to the paediatric specialty. The final pGALSplus comprises 26 manoeuvres as a toolkit with a colour coding approach and signposting to additional resources

**Conclusion:** Our work has demonstrated pGALSplus to be a novel evidence and consensus-based toolkit to support differentiation between key MSK conditions in CYP with high acceptability and feasibility. As community-based MSK assessment in CYP becomes more established, pGALSplus aims to increasingly facilitate appropriate assessment and inform decision making about onward referral. A visual animation of pGALSplus and resources to aid HCPs to undertake the assessment are being developed and will be free and openly available on PMM (www.pmmonline.org)

**Patient Consent:** Yes, I received consent

**Disclosure of Interest**: None declared

### P432. Paediatric Musculoskeletal Matters (PMM) - evidence-based e-resource providing the fundamentals of paediatric MSK medicine knowledge and skills for the international context

#### N. Smith^1^, S. Jandial ^2^, C. Scott^3^, H. E. Foster^4^ on behalf of on behalf of the PMM Editorial Board

##### ^1^Translational and Clinical Research Institute, Newcastle University, ^2^Paediatric Rheumatology, Great North Children’s Hospital, Newcastle Upon Tyne, United Kingdom, ^3^Paediatric Rheumatology, Red Cross War Memorial Children’s Hospital, Cape Town, South Africa, ^4^ Emerita Professor Paediatric Rheumatology, Newcastle University, Newcastle Upon Tyne, United Kingdom

###### **Correspondence:** N. Smith

**Introduction:** The PMM Portfolio (www.pmmonline.org) is a free and openly available online resource encompassing the Paediatric Musculoskeletal Matters (PMM) website [1], the paediatric Gait, Arms, Legs and Spine (pGALS) app and e-learning modules (ELM) to support teaching and learning about the essentials of paediatric musculoskeletal (MSK) knowledge and skills. The target learner audiences are non-specialists in paediatric MSK medicine. Evaluation of PMM [2] demonstrated positive feedback and worldwide reach including areas with limited access to paediatric MSK specialists. PMM has recently moved to the permanent guardianship of PReS and will remain free and open to all. The transfer enables PMM to work closer with PReS and the Global Task Force to reach new prospective audiences, support educational activities and facilitate growth of paediatric rheumatology around the world.

**Objectives:** To produce an agreed template of collated ‘Core topics’ to meet the perceived needs of target audiences for a ‘Basic Course’ in paediatric MSK medicine and use this to inform the next iteration of PMM to maximise Course support.

**Methods:** The PMM Editorial Board was formed from health care professionals in paediatric rheumatology and orthopaedics including medical and allied health representatives from the Global Task Force. ‘Core topics’ for a paediatric MSK ‘Basic Course’ were collated from the following sources (1) programmes of previous PReS Basic Courses, and existing e-learning programmes in paediatric MSK medicine; (2) previous work assessing the level of paediatric MSK knowledge and skills needed by graduating doctors, primary care doctors and general paediatricians [3-5] and (3) feedback from the recent PMM evaluation [2]. A consensus approach with the PMM Editorial Board agreed the ‘Core Topic’ and focused on the global context and especially low resource settings. The ‘Core topic’ list informed a Mapping Exercise across the whole PMM Portfolio to identify ‘gaps’ or where updates to PMM were needed.

**Results:** An agreed template of collated ‘Core topics’ included: (1) Epidemiology and impact of MSK conditions; (2) The importance of MSK wellness across the life course; (3) Clinical assessment and normal MSK development; (4) Approaches to clinical scenarios; (5) MSK features of red flag conditions; (6) The spectrum of Arthritis; (7) Mimics of inflammatory arthritis; (8) Multisystem conditions; (9) Orthopaedic conditions; (10) Management overview; (11) Work of the Paediatric Global MSK Taskforce; (12) Guidelines and Recommendations; (13) The WHO Essentials Medicine List; (14) The PMM Portfolio and how to use it; (15) Setting up MSK clinical services; (16) Case presentations. The Mapping Exercise informed where amends and updates are needed for PMM content to be developed by the PMM Editorial Board.

**Conclusion:** Work is underway to launch a further iteration of PMM to support Basic Courses in paediatric MSK Medicine. PMM will continue to be a valuable e-resource to reach target audiences and support the growth of paediatric rheumatology.

References

[1] Smith et al. Ped Rheum 2016;14(1):1 doi.org/10.1186/s12969-015-0062-4.

[2] Smith et al. Ped Rheum 2021;19(85) doi.org/10.1186/s12969-021-00567-5

[3] Goff et al. Educ Prim Care 2014;25(5):249-56 doi.org/10.1016/j.jpeds.2009.10.047.

[4] Jandial et al. BMC Med Educ 2015;15:171 doi.org/10.1186/s12909-015-0449-4.

[5] RCPCH 2018. www.rcpch.ac.uk/sites/default/files/2018-03/rcpch_curriculum_general_paediatrics_2018_syllabus_final.pdf

**Patient Consent:** Not applicable (there are no patient data)

**Disclosure of Interest**: None declared

## Poster session: Pain, fatigue, disease experience and quality of life

### P433. Health-related quality of life of Uzbek children with juvenile idiopathic arthritis

#### S. Akhmedova^1^, A. Ibragimov^2^, I. Kasimova^2^, K. Saipova^1^, D. Akhmedova^2^, S. Ouba^1^, A. Tomoyuki^1^, S. Aya^1^, J. Tanaka^1^

##### ^1^Graduated school of biomedical and health sciences, Hiroshima university, Hiroshima, Japan, ^2^Pediatric cardiology and rheumatology, Republican specialized scientific practical medical center of Pediatrics, Tashkent, Uzbekistan

###### **Correspondence:** S. Akhmedova

**Introduction:** Estimated over 2 million are currently living with (< 16 year olds) with JIA. Disease prevalence was greatest in Asia (South Asia), followed by Africa, Americas, Europe and Oceania. Due to the lack of data from Uzbekistan this study conducted in Uzbek JIA patients. As JIA influences all aspects of the child’s life and family, achievement of an optimal health-related quality of life (HRQoL) is an important goal in clinical care. HRQoL is a complex, multidimensional concept that encompasses physical, emotional, social, and behavior-related well-being and functioning. Higher health-related quality of life is important to properly administer children with Juvenile idiopathic arthritis.

**Objectives:** Research aim is to study factors affecting HRQoL of Juvenile idiopathic arthritis (JIA) patients in Uzbekistan.

**Methods:** The study sample includes 67 JIA patients (ages 2 –18 years) who hospitalized to the Republican specialized scientific practical medical center of Pediatrics in Tashkent, Uzbekistan between September, 2021 and January 2022. Children with JIA properly were questioned about their HRQoL using the Pediatric Quality of Life Inventory 4.0 (PedsQL). Functional ability was measured using the Childhood Health Assessment Questionnaire (CHAQ), and medical, sociodemographic parameters (number of swollen joints, visual analogue scale (VAS) activity, physician global assessment (PGA), erythrocyte sedimentation rate (ESR), and C-reactive protein (CRP)) were assessed. Associations between the biological, clinical factors and HRQoL were analyzed by correlation and linear regression analysis in JMP 16 statistical software and GraphPad Prisme 6.0.

**Results:**

The average age of the patients is 10±4.12, and 54% are female. The median PedsQL is 68.4 (53.3-77.7), median CHAQ is 1 (0.4-1.75). Higher pain level, lower well-being, higher disability index, higher number of swollen joints were significantly associated with an unfavorable (suboptimal) HRQoL (PedsQL total < 79.3).

By child total self reports 76.7% (n=46) children from 67 show suboptimal HRQOL. No significant difference were found between parent and child reports.

**Conclusion:** Using the methods of assessing the health-related quality of life, we were convinced that children with juvenile idiopathic arthritis in Uzbekistan are not treated at the same level as other high-income countries. This kind of methods assist correct treatment and monitoring of patients. It is important to look for these risk factors in clinical practice to be able to set the course at an early stage of the disease with targeted support measures. This study needs a period of follow up to compare effectiveness of the targeted treatment over the period and inclusion of healthy peers to compare for more accurate results.

**Patient Consent:** Yes, I received consent

**Disclosure of Interest**: None declared

**Table 1 (abstract P433).** See text for description

Characteristics	Total (n=67)	Girls=36 (54%)	Boys=31 (46%)	P value
**Mean age in years ± SD**	10±4.12	10.3±4.0	9.67±4.3	0.5406
**Joint inflammation characteristics, median (range)**				
**CRP**	12 (5.7; 48)	12 (5.85; 36)	12 (5; 48)	0.7555
**ESR**	11 (6; 25)	10.5 (7; 20)	11 (6; 40)	0.1715
**RF**	9 (8; 10)	9.5 (8.3; 10)	9 (8; 10)	0.8790
**Patient VAS**	4.5 (2;7)	4.5 (2; 7)	5 (2; 7.5)	0.9935
**CHAQ**	1 (0.4-1.8)	0.9 (0.4-2.1)	1 (0.3; 1.6)	0.6684
**PedsQL child total, median (range)**	68.4 (53.3;77.7)	62.8 (49.9;77.2)	69.5 (62.0;78.1)	0.2946
**PedsQL parent total, median (range)**	60.3 (47.7;73.4)	57.9 (46.8;73.4)	60.5 (50.5;74.8)	0.8867

### P434. Evaluation of functional capacity, pain, fatigue, physical activity and quality of life in children and adolescents with Familial Mediterranean Fever

#### A. Albayrak^1,2^, N. Arman^3^, E. Tarakci^3^, O. Kasapcopur^4^

##### ^1^PhD Program of Physiotherapy-Rehabilitation, Graduate Education Institute, Istanbul University-Cerrahpasa, ^2^Department of Physiotherapy and Rehabilitation, Istanbul Kent University, Faculty of Health Science, ^3^Department of Physiotherapy and Rehabilitation, Istanbul University-Cerrahpaşa, Faculty of Health Science, ^4^Department of Pediatric Rheumatology, , Cerrahpaşa Medical School, Istanbul University-Cerrahpaşa, Istanbul, Turkey

###### **Correspondence:** A. Albayrak

**Introduction:** Musculoskeletal findings are common in childhood Familial Mediterranean Fever (cFMF). Children and adolescents with rheumatic diseases experience significant disabilities due to pain, fatigue, decreased joint motion, muscle impairment and joint stiffness. A comprehensive and appropriate assessment is essential for planning physical activity and exercise programs in these patients.

**Objectives:** The purpose of the study was to evaluate the functional capacity, pain, fatigue, physcial activity and quality of life in patients with cFMF.

**Methods:** Twelve patients (aged 8-18 years) with cFMF were included in the study. 6-Minute Walk Test (6MWT) was used to evaluate the functional capacity. Visual Analog Scale (VAS) with 100 mm was used to evaluate pain intensity during rest. The PedsQL Multidimensional Fatigue Scale (PedsQL-MFS) was used to evaluate the fatigue symptoms. Physical Activity Questionnaire (PAQ) was used to evaluate physical activity levels. Familial Mediterranean Fever Quality of Life Scale (FMF-QoL) was used to evaluate the quality of life.

**Results:** Half of the patients were female and mean age was 13.35±3.45 years. The mean of scores of 6MWT was 595.10±97.50 meters, the mean of PedsQL-MFS scores was 67.07±21.82, the mean of PAQ scores 1.97±0.89 and the mean of FMF-QoL was 29.45±15.12. When the patient’s 6MWT scores were compared with the normal values according to age and sex, %85 of the patient’s 6MWT scores were lower than the scores of the healthy children and adolescents. Only two patients reported musculoskeletal-related pain (The patients VAS scores; 25 and 57 points). Also, the physical activity levels of the patients was categorized “low-active” in %80 of them, and “sufficiently-active” in %20 of them.

**Conclusion:** This study confirmed that the functional capacity was lower in patients with cFMF compared to their healthy peers. Besides physical inactivity, fatigue and impaired quality of life may be important factors that contribute to the worsening of their clinical condition by causing a vicious cycle in these children and adolescents. A more comprehensive evaluation of all these symptoms, especially pain may be helpful in setting a treatment plan for the patients with cFMF. Future research should focus on both physical and psychosocial factors in cFMF for understanding the vicious cycle.

**Disclosure of Interest**: None declared

### P435. The validity and reliability of the turkish version of pain catastrophizing scale-children in adolescents with Familial Mediterranean Fever

#### D. Bayraktar^1^, D. C. Sarac^1^, O. Altug Gucenmez^2^, B. Makay^2^, P. Keskinoglu^3^, S. Savci^4^, E. Unsal^2^

##### ^1^Department of Physiotherapy and Rehabilitation, Faculty of Health Sciences, Izmir Katip Celebi University, ^2^Division of Pediatric Rheumatology, Department of Pediatrics, ^3^Department of Biostatistics and Informatics, ^4^Faculty of Physical Therapy and Rehabilitation, Dokuz Eylul University, Izmir, Turkey

###### **Correspondence:** D. Bayraktar

**Introduction:** Familial Mediterranean Fever (FMF) is an auto-inflammatory chronic condition presenting with fever and pain attacks. Due to the unpredictable nature of the FMF, children with FMF may develop a catastrophizing behaviour towards pain. Thus, evaluating pain catastrophizing in children with FMF is important.

**Objectives:** The aim of the present study was to investigate the validity and reliability of Turkish Pain Catastrophizing Scale-Children (PCS-C) in adolescents with FMF.

**Methods:** PCS-C is a 13-item questionnaire (total scores range 0-52) where higher scores indicate higher levels of pain catastrophizing. PCS-C were translated into Turkish according to established guidelines. The Turkish form of PCS-C were filled up by adolescents with FMF (13-18 years). Pain levels were measured by using visual analog scales (VAS, 0-100 mm) at rest and during activity. Quality of life was evaluated by using Turkish Pediatric Quality of Life Inventory (PedsQL) 3.0 Arthritis Module. Participants were called by phone approximately a week later to evaluate the test-retest validity. Internal validity, test-retest reliability, and convergent validity were evaluated by using Cronbach’s alpha value, intra-class coefficient (ICC) value with 95% confidence intervals (95% CI), and Pearson correlation coefficients, respectively.

**Results:** A total of 65 adolescents with FMF (mean age= 15.4±1.4 years, mean body-mass index= 20.9±3.4 kg/m^2^, 36 female) were included in the study. The average time since onset of the symptoms and time since diagnosis were 100±54 months and 77±57 months, respectively. Pain levels were 30.5±30.4 mm for rest, and 47.4±34.4 mm for activity. Average PedsQL score was 80.3±13.9. Average PCS-C score was 22.5±12.9. The interval validity was excellent (Cronbach’s alpha= 0.938). Twenty-three participants (35%) responded to recalls, and test-retest reliability was good [ICC= 0.893 (95% CI= 0.766/0.953)]. Pain catastrophizing was found related to body-mass index, pain, and quality of life (p< 0.05, Table).

**Conclusion:** Turkish version of PCS-C seems a valid and reliable tool for measuring pain catastrophizing of adolescents with FMF.

**Patient Consent:** Yes, I received consent

**Disclosure of Interest**: None declared

**Table 1 (abstract P435).** The relationships between Turkish PCS-C and physical characteristics, disease-related characteristics, pain level, and quality of life

n:65	r	***p***
**Age (years)**	0.140	0.265
**Body Mass Index (kg/m**^**2**^**)**	0.324	0.008*
**Time since the onset of symptoms (months)**	0.006	0.962
**Time since the diagnosis (months)**	-0.059	0.640
**VAS at rest (0-100 mm)**	0.325	0.008*
**VAS during activity (0-100 mm)**	0.490	<0.001*
**PedsQL Arthritis Module (score 0-100)**	-0.552	<0.001*

kg/m^2^: kilogram/meter square, VAS: Visual Analog Scale, PedsQL: Pediatric Quality of Life Inventory, r: Pearson Correlation Coefficient, *p<0.05

### P436. The validity and reliability of the Turkish version of pain catastrophizing scale-parent in parents of adolescents with Familial Mediterranean Fever

#### D. Bayraktar^1^, D. C. Sarac^1^, O. Altug Gucenmez^2^, B. Makay^2^, P. Keskinoglu^3^, N. Ilcin^4^, S. Savci^4^, E. Unsal^2^

##### ^1^Department of Physiotherapy and Rehabilitation, Faculty of Health Sciences, Izmir Katip Celebi University, ^2^Division of Pediatric Rheumatology, Department of Pediatrics, ^3^Department of Biostatistics and Informatics, ^4^Faculty of Physical Therapy and Rehabilitation, Dokuz Eylul University, Izmir, Turkey

###### **Correspondence:** D. Bayraktar

**Introduction:** Pain catastrophizing may be a challenging problem in children with chronic pain conditions. Familial Mediterranean Fever (FMF) is a chronic disease with recurrent and unpredictable pain attacks which may lead to pain catastrophizing. The attitude of parents is important for these children who are coping with chronic pain. Thus, evaluating the views of the parents of children with FMF regarding pain catastrophizing is important.

**Objectives:** The aim of the present study was to investigate the validity and reliability of Turkish Pain Catastrophizing Scale-Parent (PCS-P) in the parents of the adolescents with FMF.

**Methods:** PCS-P is a 13-item questionnaire (total scores range 0-52) where higher scores indicate higher levels of pain catastrophizing. PCS-P were translated into Turkish according to established guidelines. The Turkish form of PCS-P were filled up by the parents of the adolescents with FMF (13-18 years). The views of the parents regarding their children’s pain levels and quality of life were measured by using visual analog scales (VAS, 0-100 mm), and Parent form of the Turkish Pediatric Quality of Life Inventory (PedsQL) 3.0 Arthritis Module, respectively. Parents were called by phone approximately a week later to evaluate the test-retest validity. Internal validity, test-retest reliability, and convergent validity were evaluated by using Cronbach’s alpha value, intra-class coefficient (ICC) value with 95% confidence intervals (95% CI), and Pearson correlation coefficients, respectively.

**Results:** A total of 57 parents (mean age= 41.7±5.2 years, 45 mothers) were included in the study. The parents’ views regarding their children’s pain levels were 36.1±32.9mm for rest, and 49.7±36.1 mm for activity. Average Parent PedsQL score was 79.7±15.5. Average PCS-P score was 31.2±12.4. The interval validity was excellent (Cronbach’s alpha= 0.932). Twenty-three parents (40%) responded to recalls, and test-retest reliability was good [ICC= 0.887 (95% CI= 0.751/0.950)]. Parents views regarding pain catastrophizing was found related to their opinions about the pain levels and to quality of life of their children (p< 0.05, Table).

**Conclusion:** Turkish version of PCS-P seems a valid and reliable tool for measuring the views of the parents of the adolescents with FMF regarding pain catastrophizing.

**Patient Consent:** Yes, I received consent

**Disclosure of Interest**: None declared

**Table 1 (abstract P436).** The relationships between Turkish PCS-P and parents’ age, and parents’ views regarding pain levels and quality of life of their children

n:57	r	p*
**Physical Characteristics of Parents**		
Age (years)	-0.050	0.710
**Parents’ Views Regarding the Pain Levels of Their Children**		
VAS at Rest (0-100 mm)	0.273	0.040*
VAS during Activity (0-100 mm)	0.274	0.039*
**Parents’ Views Regarding the Pain Levels of Their Children**		
Parent form of PedsQL Arthritis Module (score 0-100)	-0.420	0.001*

VAS: Visual Analog Scale, PedsQL: Pediatric Quality of Life Inventory, r: Pearson Correlation Coefficient, *p<0.05

### P437. A novel multidimensional questionnaire to monitor juvenile fibromyalgia syndrome and identify factors influencing the disease course

#### C. Lavarello^1,2^, A. Alongi^3^, B. Mori^1^, A. Ronchetti^4^, L. Nobili^2,5^, L. Chiarella^2,5^, E. Pescio^6^, A. Ravelli^2,7^, M. Gattorno^1^, C. Malattia^1,2^

##### ^1^Pediatric Clinic and Rheumatology Unit, IRCCS Giannina Gaslini, Genova, Italy, ^2^Department of Neurosciences, Rehabilitation, Ophthalmology, Genetic and Maternal Infantile Sciences (DINOGMI), University of Genoa, Genova, ^3^Pediatrics, ARNAS Civico-Di Cristina-Benfratelli, Palermo, ^4^Physical Medicine and Rehabilitation Unit, ^5^Child Neuropsychiatry Unit, ^6^Psychology Unit, ^7^Scientific Direction, IRCCS Giannina Gaslini, Genova, Italy, Genova, Italy

###### **Correspondence:** C. Lavarello

**Introduction:** Juvenile Fibromyalgia Syndrome (JFS) is a disabling condition characterized by musculoskeletal pain, fatigue, sleep and mood disturbances. Treatment is multidisciplinary and include exercise, cognitive behavior therapy and medications as appropriate. Currently, evidence for treatment efficacy is limited due to the lack of validated outcome measures. TheJuvenile Fibromyalgia Multidimensional Assessment Report (J-FiMAR) evaluates symptoms severity (pain, fatigue, headache, sleep and mood disorders) by a numerical rating scale, quality of life and functional ability.

**Objectives:** To explore the suitability of the J-FiMAR to monitor JFS. To identify factor influencing clinical course and predictors of treatment efficacy.

**Methods:** We included JFS patients followed at our center between 2018 and 2022. At each 3-6 months follow-up visit, patients filled J-FiMAR. Patient’s and physician’s global assessment (PGA and PhGA) and ongoing treatment were also recorded. Agreement between PGA and PhGA was evaluated by the concordance correlation coefficient (CCC). Relationships between PGA and PhGA, patient-reported symptoms, quality of life and functional capacity were tested by using the Spearman rank-order correlations and partial correlation networks. Mixed effects logistic regression models with random subject-specific intercepts were used to assess the effect of excerice and medications on disease course (improved/not improved) and to identify predictors of treatment efficacy.

**Results:** We included 51 patients (44 female; median disease duration 1.8 years). Patients were on regular physical activity in 83/194 (43%) follow-up visits and received drugs in 70/194 (36%).PGA and PhGA showed poor agreement (CCC 0.60, 95%Cl 0.53-0.67). A strong correlation was found between PGA and pain (r = 0.68, 95%Cl 0.59 -0.76), fatigue (r = 0.62, 95%Cl 0.52-0.71) and depression (r = 0.60, 95%Cl 0.48-0.69). Based on partial correlation networks, fatigue seems to have a key role in predicting the presence of other JFS-related symptoms. Response to treatment was observed in 29/51 (56.8%) patient. Responders showed a significant reduction in pain and depression severity. Regular aerobic exercise was the strongest independent predictor of treatment efficacy (OR 5.69, 95% Cl 1.07- 30.33, p=0.042). Higher score in the cognitive disorders domain at baseline was associated with poor response (OR 0.64, 95% Cl 0.43-0.95, p=0.027).

**Conclusion:** The J-FiMAR allows a comprehensive evaluation of JFS course. Pain and fatigue are the main symptoms that influence PGA. Our results further support that aerobic exercise is the main cornerstones in the JFS treatment and emphasize the importance of addressing cognitive disorders in adolescents with JFS as well. To identify clinical domain that affect PGA and treatment efficacy is essential for a more individualized management strategy.

**Patient Consent:** Yes, I received consent

**Disclosure of Interest**: None declared

### P438. Residual complaints in juvenile idiopathic arthritis patients with inactive disease

#### G. Gestler, A. M. Rocha, C. A. Len, A. Araujo, D. S. Jesus, R. D. Gestler, M. T. Terreri

##### Pediatrics, Universidade Federal de São Paulo, São Paulo, Brazil

###### **Correspondence:** C. A. Len

**Introduction:** Juvenile idiopathic arthritis (JIA) is an inflammatory disease. In addition to physical impairment, it compromises the patient’s quality of life with conditions that can extend into adulthood, such as limitations, noninflammatory pain, fatigue, short stature, in addition to social, emotional and educational issues. Approximately 20% to 30% of patients with JIA treated properly may have limited daily activities and impaired quality of life and health.

**Objectives:** To identify the main residual complaints of inactive JIA patients followed up in a tertiary pediatric rheumatology outpatient clinic.

**Methods:** We studied medical records of all patients with a definitive diagnosis of JIA and who were under 18 years old. From these charts, we selected all those related to patients with inactive disease (clinical and laboratory parameters) for at least 6 months, with or without the use of medication. In addition, we applied a questionnaire on possible residual complaints and also questionnaires PedsQL 4.0 and the PedsQL Fatigue Module.

**Results:** From a total of 503 electronic medical records, we identified the medical records of 117 patients with inactive disease. Among them, 76 patients (65%; 95%CI [53.9%; 76.1%]) presented no complaints and 41 (35%; ( 95% CI [23.9%; 46.1%]) presented some residual complaint, such as arthralgia (17.9%), joint limited range of motion (1.7%) or emotional problems (4.3%). Regarding the PedsQL 4. And the PedsQL Fatigue Module, we observed general tiredness in 24%, (95% CI [14%; 33.9%]); sleep-related fatigue in 31% of patients (95%CI [20.2%; 41.7%]); mental tiredness in 37%, (95% CI [25.4%; 47.8%]); physical disability in 29%, (95% CI [29.4%; 52.3%]); emotional problems in 62% (95% CI [50.7%; 73.3%]); socialization problems in 15% (95% CI [7.1%; 23.9%]) and school problems in 82% of the patients (95% CI [72.7%; 90.7%]).

**Conclusion:** We observed a higher-than-expected prevalence of residual complaints in JIA patients with inactive disease. We also observed lower scores in the health-related quality of life questionnaires (PedsQL 4.0 and PedsQL-Fatigue Module). Our data reinforce the importance of the multidisciplinary team in the care of JIA patients, since these patients can present multiple needs even in the phases of apparently controlled disease.

**Patient Consent:** Yes, I received consent

**Disclosure of Interest**: None declared

### P439. Determination of gait normality through dynamic baropodometry in healthy adolescents

#### C. A. Len^1^, M. C. Costa^1^, E. S. Lima^2^, J. Natour^2^, M. T. Terreri^1^

##### ^1^Pediatrics, ^2^Medicine, Universidade Federal de São Paulo, São Paulo, Brazil

###### **Correspondence:** C. A. Len

**Introduction:** In our clinic we work in a line of research on the gait of children with rheumatic diseases and idiopathic musculoskeletal pain. Our aim is to develop a multiprofessional treatment program based on rehabilitation and to help patients with some measures that can improve the quality of life of them. However, there are no studies in the literature on the standardization of gait in healthy children and adolescents.

**Objectives:** 1) To determine the parameters of normal gait in healthy adolescents, through the use of dynamic baropodometry, 2) To correlate gait parameters with functional capacity, lifestyle and the quality of life.

**Methods:** The sample consisted of healthy adolescents between 14 and 17 years old of both sexes, selected among those enrolled in a recreation center and friends of these students. All subjects were evaluated in two steps: 1) Health related questionnaires (Childhood Health Assessment Questionnaire – CHAQ and Assessment of Physical Activity and Sedentary Lifestyle in Children and Adolescents – IPAQ), and 2) gait evaluation - dynamic baropodometry (walking track FootWalk Pro, AMcube®, France) with the Footwork Pro software (IST Informatique – Intelligence Service et Tecnic, France). We measured gait speed, cadence and step time in all subjects.

**Results:** We included 102 healthy adolescents aged between 14 and 17 years. Of these, 55 were aged between 14 and 15 years and 47 aged between 16 and 17 years. Regarding sex, 54 were female. All participants had normal physical capacity according to the CHAQ. Regarding the IPAQ questionnaire for adolescents, we observed that no individual was sedentary: 18.6% were irregularly active, 45.5% were active and 35.5% were very active. Regarding the dynamic baropodometry parameters, we observed an average gait speed on the right foot of 318.335 mm/s, an average gait speed on the left foot of 319.268 mm/s, a cadence (average) of 1.457 steps/second, a right foot step time of 747.661 seconds and a left foot step time of 738.331 seconds. We did not observe any correlation between the dynamic baropodometry parameters and the results obtained in the CHAQ and IPAQ questionnaires (p > 0.05).

**Conclusion:** In our study, we established the normality of the gait patterns of healthy adolescents, according to dynamic baropodometry, a method commonly used to study the gait of children and adolescents with health problems, including neurological and rheumatological problems, among others. As expected, we did not observe any correlation between the dynamic baropodometry parameters and the results of the CHAQ and IPAQ questionnaires, since all individuals had normal functional capacity. These data will be useful for the continuation of our line of research, since gait may be compromised in chronic rheumatic patients.

**Patient Consent:** Yes, I received consent

**Disclosure of Interest**: None declared

### P440. Using Virtual Reality (VR) to help children cope with joint puncture related to JIA

#### A. Pieler, H. K. Ipsen

##### H.C. Andersen Childrens Hospital, Odense, Denmark

###### **Correspondence:** A. Pieler

**Introduction:** Virtual Reality goggles became an option as a resource to help children overcome pain related procedures at the H.C. Andersens Children’s Hospital.

When steroid injections related to treatment is necessary in our outpatient clinic, we can offer local anesthesia or N_2_O. If general anesthesia is needed, the patient must come back another day.

**Objectives:** To determine if VR can be used as distraction during joint puncture as an alternative to N_2_O and general anesthesia.

**Methods:** VR was offered to some patients based on the health professionals assessment of the childrens cooperation before the joint puncture.

On a tablet connected to the goggles it was possible to see, what the child was seeing. That way it was possible to guide the child or comment on what they were doing and seeing.

**Results:** 6 children aged 12-14 agreed to try VR. Two of them had earlier tried N_2_O. Both of them preferred VR to N_2_O.

The child could use the VR how they felt like it. Trying games and movies and shifting between them.

All procedures carried out with VR was successful. One child removed the goggles prior to the injection, as he needed to watch, but reported feeling VR helped cope with nerves up to.

The children felt helped by VR and would like to use them again, should there be a need for it.

**Conclusion:** It is possible to use VR to help children cope with joint puncture and it is now an offer in our outpatient clinic alongside general anesthesia and N_2_O.

It is an individual assessment by health professionals what is suitable in each case.

**Disclosure of Interest**: None declared

### P441. Impact of oral and subcutaneous methotrexate on the quality of life of children with juvenile idiopathic arthritis

#### M. Couto^1^, M. J. Santos^2^, S. N. Sousa^2^

##### ^1^Faculdade de Medicina da Universidade de Lisboa, ^2^Serviço de Reumatologia, Hospital Garcia de Orta, Lisboa, Portugal

###### **Correspondence:** S. N. Sousa

**Introduction:** Methotrexate (MTX), administered either orally or subcutaneously, remains the mainstay of Juvenile Idiopathic Arthritis (JIA) treatment due to its effectiveness and acceptable safety profile. Even though, less than half of patients remain in treatment beyond two years.

Both routes of administration are effective, but a greater bioavailability with subcutaneous route, especially at higher doses, has been reported.

Despite this, fear of injections and other concerns, can compromise the quality of life (QoL) and hamper medication adherence.

**Objectives:** To evaluate the impact of orally and subcutaneously administered MTX on the QoL of children and adolescents with JIA, as well as the effectiveness and side effects with both routes of administration.

**Methods:** Children and adolescents followed at a Pediatric Rheumatology Centre were included. The QoL was evaluated at last appointment using the EQ-5D-Y questionnaire (0–1; higher scores indicate better QoL) and the QoL section and the well-being VAS of Juvenile Arthritis Multidimensional Assessment Report (JAMAR) (0–30, higher scores indicate worse QoL); satisfaction with treatment was measured on a Numeric Rating Scale (0-10). Effectiveness was assessed by the improvement in disease activity and function at 6 and 12 months after starting MTX. Side effects that led to change of dose or route of administration or MTX discontinuation were also collected.

**Results:** A total of 26 children and adolescents with JIA were evaluated. Of those, 15 were receiving MTX at last appointment, 9 orally and 6 subcutaneously (Table). 17 treatment courses with subcutaneous and 29 with oral MTX were analyzed.

Quality of life was similar for both routes of MTX administration. The median scores of EQ-5D-Y were 0.85 and 1 (p=0.864) and the median scores of JAMAR (children) were 4 and 3.5 (p=0.818) for subcutaneous and oral routes of administration, respectively. The results for physical subdimension of JAMAR were 2.5 and 1 (p=0.937), for subcutaneous and oral routes, respectively and for psychosocial subdimension were 1 (p=0.937) for both routes and the reported well-being on the VAS scale of JAMAR (0 corresponding to very well and 10 to very poor) was 0 (p=0.818) for both routes of administration. Similar results were reported by parents. Regarding satisfaction with medication, the median value was 10 (min 5 – max 10).

Subcutaneous MTX was superior to oral MTX, in terms of JADAS (p=0.02) and ESR (p=0.015) reduction at 6 months and reduction of pain at 6 (p=0.046) and 12 (p=0.047) months. However, the dosage of subcutaneous MTX was significantly higher (SC 0.58 ± 0.031; Oral 0.45 ± 0.03; p=0.008). Both subcutaneous and oral MTX were well tolerated.

**Conclusion:** Conclusion: In this cohort of children with well-controlled JIA, the route of MTX administration does not appear to have a negative impact on QoL. MTX was effective and well tolerated regardless of the route of administration.

**Disclosure of Interest**: None declared

**Table 1 (abstract P441).** Demographic and clinical characteristics of children and adolescents with JIA

Variables	N = 26	Oral MTX N = 9	Subcutaneous MTX N = 6
Female N (%)	17 (65.4)	4 (44.4)	5 (83.3)
Age (mean ± SD) *years*	11.9 ± 0.8	10.9 ± 1.7	12.2 ± 1.4
Disease duration (mean ± SD) *years*	7.5 ± 0.8	7.6 ± 1.4	10.8 ± 1.5
**JIA ILAR category N (%)**			
Persistent oligoarticular arthritis	8 (30.8)	3 (33.3)	2 (33.3)
Extended oligoarticular arthritis	8 (30.8)	2 (22.2)	4 (66.7)
JADAS	1.3 (4.9)	2 (7.3)	1 (5.3)
ESR mm/h/ CRP mg/dL	11 (9)/0.06 (0.15)	11 (12)/0.06 (0.2)	8 (12)/0.06 (0.3)
Swollen/ tender joints	0/0	0/0	0/0

## Poster session: Patient/parent organisation initiatives

### P442. ThinkJIA: A campaign to raise awareness that children and young people get arthritis, to enable families and frontline health professionals to recognise symptoms and refer to specialist services

#### R. Beesley^1,2^

##### ^1^European Network for Children with Arthritis, Geneva, Switzerland, ^2^Juvenile Arthritis Research, Tonbridge, United Kingdom

###### **Correspondence:** R. Beesley

**Introduction:** Awareness that Children and Young People (CYP) may develop arthritis (Juvenile Idiopathic Arthritis, JIA) is low within the general population and amongst non-rheumatology front-line health professionals. This can lead to delays in diagnosis and reduced access to treatment, and consequential risk of joint damage and permanent eye complications through undiagnosed JIA-associated uveitis.

**Objectives:** To develop an awareness campaign to enable frontline health professionals and families to remember that CYP can develop arthritis, to be aware of the main signs and symptoms, and to pursue early referral to speciality services.

**Methods:** An awareness-raising campaign was developed by UK charity Juvenile Arthritis Research working with parents of CYP with JIA, adults with JIA, teachers, campaigners, paediatric rheumatologists, ophthalmologists, and other interested lay and professional individuals. The campaign, called #ThinkJIA, includes postcards with key messages and a supporting website, www.thinkjia.org

Resources are aimed at both the general population, to help improve recognition of the signs and symptoms and encourage early attendance at primary care health services; and at non-rheumatology front-line health professionals to support recognition of JIA symptoms and the need for prompt referral. Whilst the key messaging is common to both audiences, the language used differs to reflect the lay and medical professional expectations.

Once draft campaign resources were developed, they were shared with clinical professionals and tested by parents and school teachers.

**Results:** Of the pilot group of parents and teachers receiving draft resources, 100% of parents and 68% of teachers agreed that they would feel confident in seeking medical attention if their child, or a child in their school, started showing signs of JIA. Following feedback from recipients in the pilot group resources have been updated with clearer imagery. These are also supported by posters for use in health clinics and general circulation.

**Conclusion:** The development of the #ThinkJIA awareness-raising resources has helped ensure frontline health professionals and the general population can have access to information about JIA. This can help facilitate early referral and improved access to treatment. The broad rollout of awareness resources is essential to appropriately support CYP with JIA and ensure they receive the treatment they require promptly.

[Acknowledgements: Thanks to Prof Helen Foster for her vital role in the initial development of these resources, and to all those involved in the development and pilot testing]

**Patient Consent:** Not applicable (there are no patient data)

**Disclosure of Interest**: None declared

### P443. Parliamentary inquiry into childhood rheumatic disease: a description of the inquiry process as a novel form of advocacy, and of preliminary inquiry outcomes

#### R. A. James^1^, G. Murray^2^, W. Renton^3^, G. Tiller^3^

##### ^1^Rheumatology, Queensland Children’s Hospital, Brisbane, ^2^Rheumatology, Women’s and Children’s Hospital, Adelaide, ^3^Rheumatology, Royal Children’s Hospital, Melbourne, Australia

###### **Correspondence:** R. A. James

**Introduction:** In December 2021, following advocacy from the Juvenile Arthritis Foundation of Australia (JAFA) and the Australian Paediatric Rheumatology Group (APRG), the Australian Parliament called an Inquiry into childhood rheumatic diseases.

**Objectives:** To describe how a patient advocacy group, with the support of healthcare professionals, successfully advocated for a Parliamentary Inquiry into childhood rheumatic diseases, as well as the process and early outcomes of that inquiry.

**Methods:** Terms of reference of the Inquiry centred around 5 key themes: research; disease impacts; access to care; best practice care and professional awareness. Submissions were invited over a two month period and were followed by public hearings at which JAFA, the APRG and others were invited to present.

The APRG submission was structured as a collaborative writing effort by a multidisciplinary team of 16 volunteers, with a core group of 4 editors. Virtual meetings were conducted on a weekly basis, and more frequently for the editing group. Consumer input from JAFA was sought around content and strategy. Both JAFA and the APRG actively promoted the Inquiry to a broad spectrum of interested parties, inviting further submissions.

**Results:** 127 submissions were received: 34 from named individuals, 30 from organisations and 63 as de-identified or confidential submissions. This reflects a strong response compared to previous inquiries into other conditions (TABLE 1).

Submitting organisations included patient advocacy groups, professional organisations, health services, pharmaceutical companies and others. Submissions were received from across Australia and internationally. The vast majority were from patients and caregivers.

Common themes amongst these submissions included the mental health impacts of childhood rheumatic disease; impacts on family and household members; medication access; difficulties with accessing care in rural or remote areas; the desire for care to be repatriated to local services; and workforce shortages.

In March 2022 the Committee tabled an interim report in the Australian Parliament which made 15 key recommendations. These included tripling the Australian workforce over an 8 year period; the creation of Centres of Excellence in major cities; a hub-and-spoke model for outreach to regional areas; improved access to community-based therapies and social security supports; priority funding for key areas of workforce need; improved access to medications; and the creation of a disease registry.

The Australian Government will formally respond to this report after the May 2022 election. In the interim, concrete and emergent outcomes continue to be observed including streamlined methods for prescribing biologic medications; the development of new Australian Standards of Care for JIA; attention and interest of hospital executives resulting in increased clinical staffing in some centres; matured institutional relationships and priorities; and greater collaboration between patient and professional organisations over existing funding streams.

**Conclusion:** An Australian Parliamentary Inquiry into childhood rheumatic disease was well-received and attracted many submissions from interested parties. The resulting report made sound recommendations across a number of areas of recognised service shortfalls. Concrete and emergent outcomes from this inquiry are continuing, and will lead to improvements for patients, families and healthcare professionals in this field.

**Patient Consent:** Not applicable (there are no patient data)

**Disclosure of Interest**: None declared

**Table 1 (abstract P443).** See text for description

Condition	Year of Parliamentary Inquiry	Estimated number of Australians with Condition	Submissions Received
Childhood Rheumatic Disease	2022	10,000	127
Paediatric & Adult allergy	2019	4,600,000	257
Hepatitis C	2015	220,000	106
Skin Cancer	2014	>1000,000 *per year*	63

### P444. A parent guide to rasing a child with juvenile arthritis

#### C. McCormack^1^, G. Faller^2^, P. Ambaram^3^, K. Webb^4,5^, B. Mistry^6^, A. Eckstein^1^, L. Tomasella^7^

##### ^1^Arthritis Kids South Africa, ^2^Paediatric rheumatology, WITS Gordon Medical Centre, ^3^Paediatric rheumatology and Honorary Lecturer, University of the Witwatersrand, Johannesburg, ^4^Paediatric rheumatology, University of Cape Town, Cape Town, South Africa, ^5^ Crick African Network, Francis Crick Institute, London, United Kingdom, ^6^Paediatric rheumatology, Chris Hani Baragwanath Academic Hospital, ^7^Global Rheumatology Alliance, Johannesburg, South Africa

###### **Correspondence:** C. McCormack

**Introduction:** South African parents and caregivers (PaC) of children with juvenile idiopathic arthritis (JIA) face numerous challenges in accessing credible information about their child’s condition, including language barriers, disparate education levels, poor literacy rates, limited access to affordable internet, financial barriers, and cultural beliefs about this disease.

**Objectives:** Arthritis Kids South Africa’s (AKSA) objective is to ensure PaC of children with JIA can access credible information and support about this disease regardless of socioeconomic status, language, level of basic education, and literacy rates. We also want to ensure that cultural beliefs do not hamper efforts to achieve disease management.

**Methods:** Relevant topics to be included were identified informally in workshops with paediatric rheumatologists, patients, parents, and allied healthcare professionals (HCP). With the support of this team, individual articles were written for each topic. Certain portions were translated to three of the eleven official South African languages, namely, isiZulu, Sepedi and Afrikaans. Thereafter, an informal qualitative process took place in two paediatric rheumatology clinics to test PaC understanding of concepts included.

**Results:** The resulting collection of articles, including a host of interviews with relevant allied HCP, patients and parents, was made available to PaC via the AKSA website and, where funding allowed, in printed form. The collection precipitated the development of the first parent guide for JIA in South Africa in two South African languages. Topics include ‘My child has JIA, what’s next?’, ‘When to call your child’s doctor?’, ‘Symptoms and flares’, ‘Living with JIA’, ‘Stress and anxiety’, ‘What about food?’, ‘Exercise lowers inflammation, ‘Alternative therapies’, and ‘Frequently asked questions’. The booklet includes information on the immune system, autoimmunity, nutrition, specific school issues and the prognosis for JIA in accessible language and with images. It includes a section where a parent may record treatment notes, record growth, track symptoms and ask their health team to write a plan for a flare or an infection.

It was recognised that translations alone would not adequately address the language barrier in South Africa due to differing literacy rates in both own and English languages. Access to the internet and financial barriers also prevent the satisfactory dissemination of the support material.

To overcome these challenges, we created a combination of audio and video supplements to the written library in the form of an animated video and podcasts. These are made available in up to five of the eleven South African languages.

**Conclusion:** We have created the first South African ‘Parent Guide to JIA’ with supplemental video and podcast content to assist paediatric rheumatologists, nurses and other relevant HCP to better explain concepts related to the diagnosis, treatment and long term management of JIA. Parents can share these tools with extended family members, schools, and broader social circles. We anticipate that this will improve parents’ experience, empower them as partners in their children’s care and ultimately improve the outcomes of their children with JIA.

**Patient Consent:** Not applicable (there are no patient data)

**Disclosure of Interest**: C. McCormack Grant / Research Support with: AKSA received a grant from Roche SA to translate materials in the support library. Janssen Pharmaceutica SA paid for an advert in the printed guide which funded printing costs., G. Faller: None declared, P. Ambaram: None declared, K. Webb: None declared, B. Mistry: None declared, A. Eckstein: None declared, L. Tomasella: None declared

## Poster session: COVID-19 (Coronavirus)

### P445. Azerbaijani data on Multisystem Inflammatory Syndrome in Children (MIS-C)

#### A. Aliyeva^1,2^, N. Rahimova^3^, L. Gulmammedova^3^, S. Nesirova^3^, L. Mirsalayeva^3^, K. Eshrefova^3^, E. Rahimov^4^, A. Musayev^5^ on behalf of This Working Group members consist of 2 working place that I have mentioned above, investigation lead and organize by Dr. Aytan Alieva.

##### ^1^Pediatric reumatology, Baku Medical Plaza, ^2^Pediatric reumatology, ^3^Pediatric, Baku/Azerbaijan Scientific Research Institute of Pediatrics, ^4^Pediatric, Baku Medical Plaza, ^5^Pediatric Surgeon, Baku/Azerbaijan Scientific Research Institute of Pediatrics, Baku, Azerbaijan

###### **Correspondence:** A. Aliyeva

**Introduction:** Children’s Multisystem Inflammatory Syndrome (MIS-C) is hyperinflammatory response that occurs following infection with COVID-19 and can result in serious consequences

**Objectives:** The aim of this study was to analyze the demographic, clinical laboratory characteristics and treatment modalities of MIS-C patients from Azerbaijan

**Methods:** Methods: The trial comprised patients under the age of 18 who were diagnosed with MIS-C and treated at two locations (^1^ Baku/Azerbaijan Scientific Research Institute of Pediatrics

^2^ Baku/Azerbaijan Baku Medikal Plaza) between December 2020 and March 2022.

The patients’ information was gathered retrospectively from their medical records. The following data were collected: age, gender, admission symptoms, laboratory and clinical findings, and complications.

**Results:** Results: The study included 80 patients, 60%of whom were male (n:48) and 40% were female (n:32). The median age at the time of diagnosis was 6 years (1-14). Six individuals (7.5%) had PFAPA syndrome, two (2.5%) had juvenile idiopathic arthritis (JIA), one (1.25%) had biotinidase deficiency, one (1.25%) had diabetes mellitus, and one (1.25%) had leukemia. Among them, 85% (n=68) patients were followed at pediatric inpatient department, while 15% (n=12) were treated at intensive care unit. As a consequence, 74 patients (92.5%) fully recovered, 5 patients (6.25%) were unresponsive, so they required more aggressive treatment, and 1 patient (1.25%) died. The median duration of hospitalization was 9 (5-27) days.

Involvement of the heart was found in 81.25 % (n=65) of the patients: in10.7% (n=7) of patients coronary aneurysm, in 57.5 % (n=46) mitral and tricuspid insufficiency, in 31.25 % (n=25) pericarditis.

Gastrointestinal abnormalities, stomach pain, and lymphoadenopathy were found in 12.5% (n=10) of the participants.

As a result, 74 patients (92.5%) received complete treatment. The therapy was repeated in 5 patients (6.25%) due to recurrence of symptoms, and 1 patient (1.25%) was lost.

61 patients (76.25%) received 2g/kg IVIG, whereas 19 patients (23.75%) were unable to acquire IVIG. All patients received steroid medication, with 30 patients (37.5%) receiving pulse steroid, 80 patients (100%) receiving antibiotic therapy, and 10 patients (8%) receiving anakinra treatment.

All of the patients were given low-dose aspirin, and 50 (62.5%) of them were also given heparin.

**Conclusion:** Conclusion: One of the most serious consequences seen during the COVID-19 epidemic is MIS-C. Serious complications, such as heart involvement and coronary aneurysm, might occur if left untreated. In terms of prognosis, early diagnosis and treatment are critical.

Keywords: COVID-19, hyperinflammation, MIS-C,

**Patient Consent:** Yes, I received consent

**Disclosure of Interest**: None declared

### P446. SARS-COV-2 infection in children and adolescents with rheumatological diseases, the largest tertiary center experience from Saudi Arabia

#### J. Alqanatish^1,2,3^, A. Ahmed^2,4,5^, A. Alfadhel ^2,4,5^, A. Albelali^2,4,5^, S. Alghnam^2^

##### ^1^Pediatric Rheumatology, King Saud bin Abdulaziz University for Health Sciences, ^2^King Abdullah International Medical Research Center (KAIMRC), ^3^King Abdullah Specialist Children’s Hospital,National Guard Health Affairs, ^4^Pediatrics, King Saud bin Abdulaziz University for Health Sciences, ^5^King Abdullah Specialist Children’s Hospital, National Guard Health Affairs., Riyadh, Saudi Arabia

###### **Correspondence:** J. Alqanatish

**Introduction:** Pediatric population are considered at a lower risk of contracting SARS-CoV-2 infection. Only about 10% of SARS-CoV-2 cases were among children, however; they may suffer severe complications such as multisystem inflammatory syndrome in children (MIS-C). Data in adults with rheumatological diseases showed favorable clinical outcome of COVID-19 but pediatric data are limited.

**Objectives:** We report on the clinical characteristics of SARS-COV2 infection in children and adolescents with Rheumatological diseases. To our knowledge, this is the largest experience reported from a tertiary center in Saudi Arabia.

**Methods:** We reviewed the medical records of children and adolescents who were diagnosed with a rheumatological diseases with or without COVID-19 infection and visited rheumatology clinic or day care unit at King Abdullah Specialized Children Hospital (KASCH), Riyadh, during pandemic. Moreover, we conducted phone calls to patients’ families to verify and add to the information in medical records. We described these patients’ clinical characteristics analyzed the factors associated with SARS-CoV-2 infection and associated complications, as well as clinical outcome.

**Results:** Among 496 patients included in the study, 117 (23.6%) contracted COVID-19. Gender, biologics, conventional or targeted DMARDs and comorbidities were not associated with the likelihood of infection; however, those who had COVID-19 infection were younger (average age=11.1 vs. 13.8 years; P<0.01). The most common symptom among COVID-19 patients was fever (n=82,70%).Overall, 38 (7.8%) patients suffered any complication, but that varied with COVID-19 status. Of patients who had COVID-19, 66% suffered complications or death, compared with only 0.76% in patients who did not have COVID-19 (P<0.01).

**Conclusion:** In our cohort, younger, compared to older, children with Rheumatological diseases have more commonly contracted COVID-19 infection. Nevertheless, no other clear associations were shown between patients’ demographics or comorbidities and the likelihood of infection. Although, only few patients suffered any complication but that was still more common in patients with COVID19 infection.

**Patient Consent:** Not applicable (there are no patient data)

**Disclosure of Interest**: None declared

### P447. SARS-COV-2 infection and vaccination in children with rheumatic diseases:an Italian experience

#### F. Anselmi, B. Macri, E. Barbieri, R. Ragucci, V. Aiello, R. Naddei, M. Alessio

##### Department of Translational Medicine, Section of Pediatrics, University of Naples Federico II, napoli, Italy, Naples, Italy

###### **Correspondence:** F. Anselmi

**Introduction:** Since the spread of SARS-CoV-2 infection, a great concern has been addressed to rheumatic patients, especially given that they often require immunosuppressive therapies. So far few studies have described clinical course of SARS-CoV-2infection in children affected by rheumatic diseases and their response to COVID19 vaccination.

**Objectives:** to evaluate the impact of SARS-CoV-2 infection and COVID19 vaccination on our patients with chronic rheumatic diseases.

**Methods:** In this single-centre,retrospective,cohort study,we analyzed 78 patients(M30;age11±4,1y) who developed SARS-CoV-2 infection between 2020 and 2022.A survey was submitted to their families to investigate the childrens’ vaccination status and course of infection (duration,symptoms/severity,time to negative test). Data were analyzed through SPSS2019.

**Results:** Clinical data of our cohort are shown in Table 1.COVID19 infection resulted significantly more frequent in the age group0-11 compared to the age group12-18(75,6%vs51,5%,p<0,05). No significant differences in symptoms were observed between age,sex or diagnosis groups,except for sore throat(47,9%vs20%,p<0,05) and ageusia(16,7%vs0%,p<0,02) which resulted more frequent in females. No significative correlation was found between vaccination status and development or duration of symptoms. Neverthless,23(59%)vaccinated patients vs 7(17,9%)unvaccinated showed a negative swab in less than 10 days(p<0.0001),while 14 unvaccinated patients(35,9%)vs 4 vaccinated(10,3%)resulted negative after more than 14 days(p<0.0001). Likewise, patients who contracted COVID19 before vaccination showed a longer time to negative test conversion compared to those who contracted it before vaccination (respectively 25,5% vs 63% tested negative in less than 10 days, p<0.005;31,4% vs 7,4%tested negative in more than 14days,p<0.005);Moreover,100%(4) of patients that relapsed after the infection were unvaccinated**.** Among patients under double immunosuppressive therapy (methotrexate+etanercept) fever ad arthromyalgia were significantly less frequent(2.5%vs18,4%,p <0.05).

**Conclusion:** In our cohort most patients with rheumatological diseases developed paucisymptomatic/asymptomatic COVID19 infection, regardless their vaccination status,consistently with general pediatric population reports. Nevertheless our vaccinated children showed a significantly shorter time to negative PCRtest compared to unvaccinated patients. Only 4patients relapsed after infection and all of them were not vaccinated. Therefore in our experience the antiSarsCov2 vaccination helped reducing the duration of the infection and possibly the risk of disease relapsing. Infection was more frequent in children under 12years,but this may be due to the fact that vaccination in children>12years started earlier. Further studies on larger case series are needed to evaluate the effects of COVID19infectionand vaccine on children with rheumatological diseases.

**Disclosure of Interest**: None declared

**Table 1 (abstract P447).** See text for description

Diagnosis(%)	Therapy(%)	Symptoms(%)
JIA(67,9) CRMO(5,1) Autoinflammatory(9) Dermatomyositis(5,1) Other(12,8)	Biologicaldrugs(32,1) Methotrexate(23,1) Methotrexate+biological(1,3) Other(7,7) Off-therapy(26)	Fatigue(52,6) Fever(51,3) Arthromyalgia (51,3) Headache(51,3) Rhinitis(47,4) Sore throat(37,2) Cough(38,5) Anosmia/ageusia(11,5) GI symptoms(11) Respiratoryfailure(1,3)

### P448. MIS-C and Kawasaki disease: a comparison based on our experience

#### R. Asaro^1^, A. Cilona^2^, C. Alizzi^2^, G. Corsello^3^

##### ^1^Dipartimento di scienze per la promozione della salute materno infantile G D’Alessandro Università degli studi di Palermo , ^2^Dipartimento di scienze per la promozione della salute materno infantile G D’Alessandro Università degli studi di Palermo , Palermo, Italy, ^3^dipartimento di scienze per la promozione della salute materno infantile “G. D’Alessandro” Università degli studi di Palermo, Italy , Palermo, Italy

###### **Correspondence:** R. Asaro

**Introduction:** Following the onset of the COVID-19 pandemic, there has been an increase in the incidence of autoinflammatory syndromes in pediatric age. In addition to the already known Kawasaki disease (KD), a new pathological picture has been characterized: the multisystem inflammatory syndrome in children (MIS-C). Usually MIS-C affects children> 5 years of age and adolescents, KD younger children. They are characterized by persistent fever, conjunctival hyperemia, skin rash, edema of the hands and feet, stomatitis but in MIS-C is common the finding of organ dysfunction and inflammatory markers are higher. Differential diagnosis is based on SARS-CoV-2 serology and exposure history

**Objectives:** In 2021 and 2022 at our O.U. of General Pediatrics of Hospital “G. Di Cristina”, a significant incidence of MIS-C and KD was recorded. The purpose of this work is to describe the similarities and differences between the two groups of patients using the following table.

**Methods:** Children diagnosed with **MIS-C** had an average age of 7 and IgG positive for Sars-Cov2.All presented with high fever, conjunctival hyperaemia, diffuse maculopapular rash and increased inflammation indices. They all had pulmonary involvement on chest CT. Three of them had also a cardiac involvement. All of them showed improvement in clinical conditions after therapy with IV Ig, corticosteroids and ASA.

Patients with KD had an average age of 13 months and IgG negative for SarsCov2.In addition to the same symptoms as MIS-C, two of them had edema of the hands and feet and stomatitis. None showed pulmonary or cardiac involvement on imaging, but only an increase in cardiac enzymes. High-risk patients (male, age <1 year, high inflammation indices) were treated following the specific protocol with intravenous Ig at 2g/kg, corticosteroids at 2 mg/kg and ASA at 50 mg/kg with benefit

**Results:**

**Conclusion:** In accordance with the international scientific literature, in the last two years our O.U. reported an increase in the incidence of KD and placed numerous diagnoses of MIS-C following the spread of COVID-19 infection. Our experience suggest that Sars-Cov2 may act as a superantigen by triggering an abnormal immune system response in predisposed children.

**Disclosure of Interest**: None declared

**Table 1 (abstract P448).** See text for description

Patients Diagnosis	AGE	SARS-Cov2 Serology	Symptoms	Chest Imaging	Echocardiogram	Therapy
LB **MIS-C**	11years	**negative**	High fever, conjunctival hyperemia, skin lesions, DESATURATION	thickening areolae ground-glass appearance. Pleural effusion	Normal	Ig ev, ASA, corticosteroids,oxygen therapy
P. **MIS-C**	1year	**Positive**	Fever conjunctival hyperemia, papular maculo rash	thickening areolae ground-glass appearance. No plaural effusion	Pericardial effusion	Ig ev, ASA, corticosteroids
C.**MIS-C**	6years	**Positive**	Fever conjunctival hyperemia, papular maculo rash DESATURATION	Areolae ground-glassappearance. Pleural effusion	Pericardial effusion	Ig ev, ASA, corticosteroids. oxygen therapy
M.**MIS-C**	10years	**Positive**	Fever papular maculo rash COLLIQUATIVE LYMPHADENOPATHY		Refraction of the walls of the right coronary	Ig ev, corticosteroids, ASA
B. **MIS-C complicated By myocarditis**	11years	Positive	fever, conjunctival hyperemia, palmar erythema	normal	Reduction of systolic function.myocarditis.	**Ig ev, steroids, asa, ANAKINRA EV**
G.**KD**	1year,male	negative	High fever, hyporeactivity, micropapular rash	parenchymal consolidation, pleural flap.	Normal	**Intravenous Ig corticosteroids ASA**
M. **KD**	2years,female	negative	Fever, conjunctival hyperemia, diffuse macular exanthema, edema of hands and feet.		Normal	Ig ev, ASA, corticosteroids
L..**KD**	10months,male	negative	Fever, diffuse maculopapular rash. Stomatitis.		Normal	**Intravenous Ig corticosteroids and ASA**
I.**KD**	6months,male	Active Sars-Cov2 infection	fever, mucositis, rash		Normal	**Intravenous IG.corticosteroids, and ASA**

### P449. Gastrointestinal involvement in multisystem inflammatory syndrome associated with COVID-19 in children

#### I. Avrusin^1^, K. Belozerov^1^, L. Bregel^2,3^, O. Efremova^3^, E. Dondurei^4,5^, A. Vilnits^6,7^, T. Kornishina^1^, V. Masalova^1^, T. Burtseva^8,9^, O. Kalashnikova^10^, V. Chasnyk^1^, M. Kostik^1^

##### ^1^Hospital Pediatrics, Saint Petersburg State Pediatric Medical University, Saint Petersburg, ^2^Irkutsk State Medical Academy of Postgraduate Education, branch of Russian Medical Academy of Continuous Professional Education, ^3^Irkutsk Regional Children’s Clinical Hospital, Irkutsk, ^4^Scientific Research Institute of Influenza n.a. A.A. Smorodintsev, ^5^Children’s City Clinical Hospital # 5 n.a. N.F. Filatov, ^6^Pediatric Research and Clinical Center for Infection Diseases, ^7^Saint Petersburg State Pediatric Medical University, Saint Petersburg, ^8^Pediatrics and Pediatric Surgery, North-Eastern Federal University (NEFU), ^9^Yakut Research Center of Complex Medical Problems, Yakutsk, ^10^Hospital Pediatrics, Saint Petersburg State Pediatric Medical University, Saint Petersbug, Russian Federation

###### **Correspondence:** M. Kostik

**Introduction:** COVID-19 in children is often asymptomatic or with only mild symptoms. However, since April 2020 there are many reports that the new coronavirus infection might be associated with pediatric hyperinflammatory condition, that fully or partially meets the criteria for Kawasaki disease (KD). This phenomenon was later called multisystem inflammatory syndrome in children (MIS-C).

**Objectives:** Our study aimed to evaluate main clinical and laboratorial features and course of MIS-C and find factors associated with gastrointestinal (GI) involvement.

**Methods:** The retrospective study included 162 children (96 male, 66 female), aged from 4 months to 17 years (median 8.2 years), who met the WHO criteria for MIS-C.

**Results:** GI involvement is one of the most frequent early manifestations and main features that helps to distinguish MIS-C from Kawasaki disease.

Main GI symptoms were abdominal pain, vomiting, ans diarrhea. Also, in 3 patients there were peritoneal symptoms that required diagnostic laparoscopy and appendix removal (1.8%).

Patients with MIS-C were divided into two groups: with (n=125, 77.2%) and without GI involvement (n=37, 22.8%). The predominance of boys (64.8%) was noted in the group of patients with GI involvement compared to the group without it (40.5%, p=0.008). Differences in the incidence of facial edema (56.5% vs. 30.8%, p=0.022), hepatomegaly (69.6% vs. 47.1%, p=0.016), splenomegaly (46% vs. 23.5%, p=0.02), neurological symptoms (51.6% vs. 33.3%, p=0.053), hypotension/shock (51.2% vs. 18.9%, p=0.0005), signs of myocardial damage (35% vs. 19.4%, p=0.078) compared to children without GI involvement were found, respectively. Patients with GI involvement had higher level of CRP (157.7 mg/l vs. 106.1 mg/l, p=0.077) and troponin (7 pg/ml vs. 1 pg/ml, p= 0.065).

Signs associated with GI damage, their sensitivity, specificity and odds ratio (OR) are presented in the table.

Of the initial 6 predictors included in the multifactorial regression model, only 2 variables were significantly associated with GI damage: male gender, and hypotension/shock.

**Conclusion:** GI involvement is an important early predictor of the severity of MIS-C, requiring careful clinical and laboratory monitoring. GI disorders are significantly more common in males, and also associated with such characteristics of MIS-C as face swelling, CNS lesion, hepatomegaly and splenomegaly, hypotension / shock.

**Acknowledgements**: This work was supported by the RSF grant № 20-45-01005

**Disclosure of Interest**: None declared

**Table 1 (abstract P449).** See text for description

Parameter	***Se***	***Sp***	OR (95%CI)	р
Male gender	64.8	59.5	2.7 (1.3; 5.7)	0.008
CNS involvement	57.6	66.7	2.1 (0.98; 4.65)	0.053
Face swelling	56.5	69.2	2.9 (1.1; 7.5)	0.022
Hepatomegaly	69.6	52.9	2.6 (1.2; 5.7)	0.016
Splenomegaly	45.9	76.5	2.8 (1.2; 6.6)	0.02
Hypotension/Shock	51.2	81.1	4.5 (1.8; 11.0)	0.0005

### P450. When to use biologic drugs in the treatment of Multisystem Inflammatory Syndrome in Children (MIS-C): a single center cohort study

#### O. Basaran^1^, E. D. Batu^1^, U. Kaya Akca^1^, E. Atalay^1^, M. Kasap Cuceoglu^1^, S. Sener^1^, Z. Balik^1^, S. Kesici^2^, T. Karagoz^3^, Y. Ozsurekci^4^, Y. Bilginer^1^, A. B. Cengiz^4^, S. Ozen^1^

##### ^1^Department of Pediatric Rheumatology, ^2^Department of Pediatric Intensive Care Unit, ^3^Department of Pediatric Cardiology, ^4^Department of Pediatric Infectious Diseases, Hacettepe University, Ankara, Turkey

###### **Correspondence:** E. D. Batu

**Introduction:** Multisystem inflammatory syndrome in children (MIS-C) is a newly identified and life-threatening condition associated with SARS-CoV-2 infection. Although our understanding of COVID-19 and MIS-C has been evolving, optimal treatment for MIS-C remains unclear. intravenous immune globulin (IVIG)±corticosteroids is widely accepted as the first step management in MIS-C.

**Objectives:** To evaluate and compare the demographic and clinical features of MIS-C patients who had IVIG plus steroid and who had additional biologic drugs, to analyze the indications for biologic drugs in MIS-C treatment.

**Methods:** This retrospective cohort study included 79 MIS-C patients from a tertiary-care pediatric hospital who were followed up between July 2020 to November 2021. We used univariate and multivariate regression analysis for finding out the variables predicting biologic use. The Receiver Operating Characteristics (ROC) curve was used to demonstrate the best performing cut-off value for CRP levels.

**Results:** Of 79 patients (median age 8.51 years, 33 girls), 51 (64.5%) received steroid plus IVIG plus biologic agents, while 28 (35.5%) had only steroid and IVIG. Compared with patients who had IVIG and steroid, patients having additional biologic therapy were more likely to have hypotension (6.9% vs. 93.1%, p<0.001) and pulmonary infiltration (0% vs. 100%, p<0.001), and need more inotropic agents (5.0% vs. 100%, p<0.001) and required O_2_ support (0% vs. 100%, p<0.001). Patients who received biological agents had significantly lower hemoglobin levels (p=0.01), thrombocyte (p<0.001) and lymphocyte counts (p=0.02), albumin (p<0.001) levels, and higher C-reactive protein (CRP) (p<0.001), ferritin (p<0.001), brain natriuretic peptide (BNP) (p<0.001), d-dimer (p=0.02), troponin (p<0.001) levels and longer median inpatient stay (p<0.001) and duration of fever (p=0.02). All patients were discharged with healing and none of the patients had an exitus. The multivariate regression analysis disclosed only CRP level as being independently associated with biologic use in MIS-C treatment. In the ROC analysis, the area under curve was 0.709 with a standard error of 0.064 and 95% confidence interval of 0.585-0.834 (p=0.002). CRP≥12 mg/dl was the best performing cut-off value with a sensitivity of 80.4% and specificity of 60.7% for predicting biologic use.

**Conclusion:** We suggest that the CRP level at MIS-C diagnosis may help to decide on the indication of biologic treatment. Identifying the MIS-C patients who require biologic treatment in the acute disease period would improve the outcome of these patients.

**Patient Consent:** No, I have not receive consent

**Disclosure of Interest**: None declared

### P451. The characteristics of patients with COVID-19-associated pediatric vasculitis: an international, multicenter study

#### E. D. Batu^1^, S. Sener^1^, G. Ozomay Baykal^2^, E. Arslanoglu Aydin^3^, S. Ozdel^3^, A. Gagro^4^, F. G. Demirkan^5^, E. Esen^6^, N. Akpinar Tekgoz^7^, K. Ozturk^8^, O. Vougiouka^9^, H. E. Sonmez^10^, M. Heshin-Bekenstein^11^, M. C. Maggio^12^, U. Kaya Akca^13^, M. Jelusic^14^, A. Pac Kisaarslan^6^, B. Celikel Acar^7^, N. Aktay Ayaz^5^, B. Sozeri^2^, S. Ozen^1^ on behalf of PReS Vasculitis Working Party

##### ^1^Hacettepe University Faculty of Medicine, Ankara, ^2^Umraniye Research and Training Hospital, Istanbul, ^3^Dr. Sami Ulus Maternity and Child Health and Diseases Research and Training Hospital, Ankara, Turkey, ^4^Children’s Hospital Zagreb, Zagreb, Croatia, ^5^Istanbul University Faculty of Medicine, Istanbul, ^6^Erciyes University Faculty of Medicine, Kayseri, ^7^Ankara City Hospital, Ankara, ^8^Istanbul Medeniyet University, Goztepe Research and Training Hospital, Istanbul, Turkey, ^9^Athens University School of Medicine, Athens, Greece, ^10^Kocaeli University Faculty of Medicine, Kocaeli, Turkey, ^11^Dana Dwek Children’s Hospital, Tel Aviv Medical Center, Tel Aviv, Israel, ^12^University of Palermo, Palermo, Italy, ^13^Aydın Obstetrics and Pediatrics Hospital, Aydın, Turkey, ^14^University of Zagreb, School of Medicine, Zagreb, Croatia

###### Correspondence: E. D. Batu

**Introduction:** COVID-19 has been associated with some rheumatic manifestations such as arthritis and vasculitis. Kawasaki disease (KD)-like vasculitis in MIS-C (multisystem inflammatory syndrome in children) forms the majority of COVID-19 associated pediatric vasculitides. Non-MIS-C COVID-19 associated pediatric vasculitis is very rare and only isolated case reports and case series have been reported in the literature to date.

**Objectives:** To analyze the characteristics, treatment and outcome in COVID-19-associated pediatric vasculitis other than KD-like vasculitis in MIS-C.

**Methods:** The inclusion criteria were as follows: 1) <18 years old; 2) evidence of SARS-CoV-2 exposure (history of COVID-19 or contact OR positive PCR OR serology); 3) evidence of vasculitis diagnosis (angiographic or histopathological evidence of vasculitis OR meeting diagnostic/classification criteria for vasculitis). Patients with MISC were excluded.

**Results:** A total of 42 patients (median age at vasculitis onset 9 years; M/F=1) were included from 14 centers and six countries. The most frequent phenotype was IgA vasculitis (IgAV) (n=28) followed by skin vasculitis (n=4) and Takayasu arteritis (n=3). Other vasculitis subtypes were polyarteritis nodosa (n=1), granulomatous polyangiitis (n=1), and unclassified vasculitis (n=5). The history of COVID-19 was present in 28 (66.7%) patients. SARS-CoV-2 PCR was positive in 16 out of 37 patients (43.2%) and SARS-CoV-2 serology was positive in all 24 of 24 (100%) patients. The median duration between exposure to SARS-CoV-2 and onset of vasculitis-associated symptoms was 15 days. The median duration of follow-up was 3 months. Skin (90.5%%) and musculoskeletal (73.8%) involvements were the most common manifestations of vasculitis. Gastrointestinal system and renal involvements were present in 59.5% and 42.9% of the patients, respectively. The majority of patients received corticosteroids (73.8%), while 21.4% used immunosuppressive drugs. 8 (19%) patients received antiplatelet or anticoagulant therapy. Outcome data was available for 40 patients (2 were recently diagnosed). Complete and partial remission was achieved in 80% and 17.5% of patients while one patient had refractory disease course. None of the patients died.

Regarding IgA vasculitis (M/F=1.8; median age at vasculitis onset 9.8 years), all patients had skin manifestations while 18 (64.3%) and 12 (42.9%) had gastrointestinal system and renal involvement, respectively. 17 (60.7%) patients received NSAIDs and corticosteroids were used in the treatment of 18 (64.3%) patients. Remission was achieved in all but one who had refractory disease course with severe gastrointestinal and renal vasculitis.

**Conclusion:** Although COVID-19 associated pediatric vasculitis is very rare, analyzing its characteristic features is important to raise awareness among physicians. COVID-19-associated IgAV seems similar to non-COVID-19 associated IgAV. However, the disease course may be slightly more severe. The temporal association of SARS-CoV-2 and vasculitis is similar to what is observed in MIS-C which suggests an immune reaction triggered by the virus.

**Patient Consent:** Yes, I received consent

**Disclosure of Interest**: None declared

### P452. Cardiac MRI in long term follow-up of MIS-C: a retrospective multicentre study

#### S. Benvenuto^1^, G. Simonini^2^, S. Della Paolera^3^, S. Abu-Rumeileh^2^, M. V. Mastrolia^2^, A. Manerba^4^, D. Chicco^3^, T. Caiffa^3^, M. Cattalini^4^, A. Taddio^1^

##### ^1^University of Trieste, Trieste, ^2^Rheumatology Unit, Meyer Children’s Hospital, University of Florence, Florence, ^3^Institute for Maternal and Child Health, IRCCS “Burlo Garofolo”, Trieste, ^4^ASST Spedali Civili di Brescia, Brescia, Italy

###### **Correspondence:** S. Benvenuto

**Introduction:** Heart involvement is a common feature of multisystem inflammatory syndrome in children (MIS-C), but exhaustive data on long-term cardiac recovery are lacking.

**Objectives:** To evaluate the long-term outcome of cardiac involvement in children affected by MIS-C, assessed through cardiac MRI.

**Methods:** Children referring to three Italian tertiary pediatric centers (Institute for Maternal and Child Health, IRCCS “Burlo Garofolo”, Trieste; ASST Spedali Civili, Brescia; Meyer Children’s University Hospital, Florence) between February 2020 and November 2021 with a diagnosis of MIS-C, who underwent cardiac MRI during a follow-up visit were enrolled. Demographic, clinical, laboratory, treatment and outcome data were collected in an anonymized database.

**Results:** Twenty mostly male (75%) MIS-C patients, aged 9-17 (median 12), were included. Most common clinical features at onset were fever (100%), gastrointestinal (70%) and/or mucocutaneous (70%) manifestations, hypotension and/or shock (55%). All presented laboratory evidence of myocardial involvement, either as elevated troponin or NT-proBNP, and most (15/18 assessed, 83%) showed reduced left ventricular ejection fraction (LVEF) on echocardiography on admission. Half of them required admission to pediatric intensive care unit, while 40% required inotrope agents; a nonfatal cardiac arrest was observed. A good clinical, laboratory and echocardiographic response to treatment was observed in all of the patients, with normal (15/19 assessed, 79%) or improved echocardiography at discharge.

Cardiac MRI was performed after a median interval of 3 months from discharge, with a maximum of 9 months. Mild residual left ventricular dysfunction was found in 20%, all showing normal LVEF on echocardiography at discharge. A thin layer of pericardial effusion or myocardial edema were found in 5% of patients, in line with previous similar studies^1^. Finally, in contrast to all of the previous observations, late gadolinium enhancement (LGE) as a sign of minimal myocardial scars was found in 25% of our patients. Persisting myocardial scars are typical findings of “classic” viral myocarditis^2^, which has however been associated with a worse cardiac outcome when compared to MIS-C myocarditis^3^. Of note, one patient in our cohort who had been evaluated at two consecutive time points showed partial resolution of a myocardial scar after 7 months from its first finding.

**Conclusion:** Despite the severity of heart involvement in the acute MIS-C phase, the long-term cardiac outcome is good. Larger longitudinal studies are needed in order to confirm this positive evolution and to establish the effective need and duration for physical activity restrictions or specific cardioprotective treatment.

**References**

1. Bartoszek M, et al. Cardiac Magnetic Resonance Follow-Up of Children After Pediatric Inflammatory Multisystem Syndrome Temporally Associated With SARS-CoV-2 With Initial Cardiac Involvement. J Magn Reson Imaging. 2022 Mar;55(3):883-891

2. Malek LA, et al. Children With Acute Myocarditis Often Have Persistent Subclinical Changes as Revealed by Cardiac Magnetic Resonance. J Magn Reson Imaging. 2020 Aug;52(2):488-496

3. Patel T, et al. Comparison of Multisystem Inflammatory Syndrome in Children-Related Myocarditis, Classic Viral Myocarditis, and COVID-19 Vaccine-Related Myocarditis in Children. J Am Heart Assoc. 2022 May 3;11(9):e024393

**Patient Consent:** Not applicable (there are no patient data)

**Disclosure of Interest**: None declared

### P453. Spontaneous nlrp3 inflammasome-driven IL-1b secretion is induced in severe COVID-19 patients and responds to anakinra treatment

#### A. Bertoni^1,2^, F. Penco^1^, H. Mollica^1^, P. Bocca^1^, I. Prigione^1^, A. Corcione^1^, D. Cangelosi^3^, F. Schena^1^, G. Del Zotto^4^, A. Amaro^5^, N. Paladino^1^, E. Pontali^6^, M. Feasi^6^, S. Signa^1^, M. Bustaffa^1^, R. Caorsi^1^, R. De Palma^7^, U. Pfeffer^5^, P. Uva^3^, A. Rubartelli^1^, M. Gattorno^1^, S. Volpi^1^

##### ^1^Centro Malattie Autoinfiammatorie ed Immunodeficienze, IRCCS Istituto Giannina Gaslini, ^2^DINOGMI, Università degli Studi di Genova, ^3^Clinical Bioinformatics Unit, ^4^Department of Research and Diagnostics, IRCCS Istituto Giannina Gaslini, ^5^IRCCS Ospedale Policlinico San Martino, ^6^Ente Ospedaliero Ospedale Galliera, ^7^Department of Internal Medicine, IRCCS Ospedale Policlinico San Martino, Genova, Italy

###### **Correspondence:** A. Bertoni

**Introduction:**

Coronavirus disease 2019 (COVID-19) is an acute respiratory illness caused by severe acute respiratory syndrome coronavirus 2 (SARS-CoV-2). COVID-19 severe pneumonia has been associated to systemic inflammation. Recently several reports have highlighted the role of innate immune cells, particularly monocytes, and of monocyte-derived cytokines such as IL-1b, in COVID-19 and showed a correlation between the degree of involvement of innate immunity and disease severity and outcome. Stimulation of peripheral blood mononuclear cells (PBMC) from patients with severe COVID-19 have shown an increased secretion of IL-1β. In support of a key role of IL-1β in patients with severe COVID-19 we and others have recently reported the potential efficacy of blocking IL-1β by using anti-IL1 drugs. NLRP3, activated by inflammatory signals, oligomerizes and recruits pro-caspase-1 and the adaptor molecule apoptotic speck like protein-containing a CARD (ASC), resulting in activation of caspase-1, processing of pro-IL-1β and secretion of the mature cytokine. Recently NLRP3 activation and ASC speck formation in peripheral blood cells and CD14+ cells from autoptic lung tissue of patients with COVID-19 has been reported. However, the mechanism underlying this activation is unknown.

**Objectives:** To dissect the mechanisms underlying SARS-CoV-2 associated inflammation in severe COVID-19 patients, which is reminiscent of a so-called cytokine storm syndrome, we analyzed the involvement of IL-1β, a pivotal cytokine driving inflammatory phenotypes, whose maturation and secretion are regulated by NLRP3 inflammasome.

**Methods:** We analyzed NLRP3 activations using confocal microscopy, plasma cytokines levels, cytokines secretion following in vitro stimulation of blood circulating monocytes and whole blood RNA sequencing. The role of ORF3a SARS-CoV2 protein was assessed by confocal microscopy analysis after nucleofection of a monocytic cell line.

**Results:** We observed that circulating monocytes isolated from COVID-19 patients revealed ASC specks formation, signal of NLRP3 activation. Furthermore this cells spontaneously secrete IL-1β in vitro. This spontaneous activation reverts following patient’s treatment with the IL-1 receptor antagonist anakinra. Transfection of THP-1, a monocytic cell line, with cDNA coding for the ORF3a SARS-CoV2 protein, leading to ASC speck formation.

**Conclusion:** These results provide further evidence that NLRP3 and IL-1β targeting could represent an effective strategy in this disease and suggest a mechanistic explanation for the strong inflammatory manifestations associated to COVID-19.

**Patient Consent:** Yes, I received consent

**Disclosure of Interest**: None declared

### P454. High incidence of MIS-C and other autoimmune diseases after SARS-COV-2 infection compared to COVID-19 vaccination in pediatric population from South Central Europe

#### M. Bizjak^1^, N. Emeršič^1^, M. Zajc Avramovič^1^, T. Vesel Tajnšek^1^, G. Markelj^1^, V. Berce^2^, N. Toplak^1,3^, A. Taddio^4,5^, T. Avčin^1,3^

##### ^1^Department of Allergology, Rheumatology and Clinical Immunology, Children’s Hospital, University Medical Centre Ljubljana, Ljubljana, ^2^Division of Pediatrics, University Medical Centre Maribor, Maribor, ^3^Department of Pediatrics, Faculty of Medicine, University of Ljubljana, Ljubljana, Slovenia, ^4^Institute for Maternal and Child Health, IRCCS “Burlo Garofolo”, ^5^University of Trieste, Trieste, Italy

###### **Correspondence:** N. Emeršič

**Introduction:** Children are less likely than adults to develop serious disease upon infection with SARS-CoV-2 but are at increased risk for inflammatory and autoimmune diseases linked to the virus. The reported incidence of multisystem inflammatory syndrome in children (MIS-C) varied from 0.2 to 11.4/100,000 persons under 21 years. It is yet unknown whether MIS-C can recur after SARS-CoV-2 reinfection or COVID-19 vaccination.

**Objectives:** To estimate the incidence and describe the spectrum of inflammatory and autoimmune diseases linked to SARS-CoV-2 infection and COVID-19 vaccination in pediatric patients from two neighbouring South Central European countries and regions, Slovenia and Friuli Venezia Giulia (FVG), Italy.

**Methods:** We performed a multi-centre prospective cohort study of all children (under 18 years) newly diagnosed with MIS-C or other inflammatory/autoimmune diseases linked to SARS-CoV-2 infection, who were admitted to the pediatric tertiary care hospitals in Slovenia or FVG, Italy during the period from January 1, 2020, to December 31, 2021. These hospitals serve a combined population of 546,032 children. Only patients with positive anti-SARS-CoV-2 antibodies and/or positive SARS-CoV-2 PCR test within 3 months prior to disease onset were considered for estimating the disease incidence. We obtained the number of patients with serious adverse events (SAE) after COVID-19 vaccination and with severe COVID-19 in the same population. This study was conducted as a part of the EU interregional Italy-Slovenia project CATTEDRA (Cross border cooperation for innovative diagnosis of rare diseases in paediatrics).

**Results:** 192 children were diagnosed with inflammatory and autoimmune diseases linked to SARS-CoV-2. The most common disease was MIS-C in 112 patients, followed by vasculitis in 74 patients of whom the vast majority presented with chilblain-like lesions. The neurological cases included patients with a rare Guillain-Barré syndrome variant with predominant cranial nerve involvement, peripheral facial palsy, autoimmune encephalitis and long COVID. One patient presented with acute myocarditis and one with acute pericarditis. Median age at diagnosis was 11.9 years (IQR 7.6 -14.7). All included patients were White. Incidence of MIS-C was one in 860 children after SARS-CoV-2 infection and one in 5460 of all children. Cumulative incidence of MIS-C since the start of the pandemic was 18.3/100,000 children. Until December 31, 2021, 95,191 children (17 %) received at least one dose of COVID-19 vaccine. Two patients with MIS-C and one patient with myositis presented after COVID-19 vaccination. All 3 had evidence of recent SARS-CoV-2 infection in form of positive anti-N SARS-CoV-2 antibodies. In the same period, 15 children were hospitalised with severe COVID-19. Eight patients from our cohort were vaccinated against COVID-19 median 8 months after MIS-C and further 20 patients had a SARS-CoV-2 reinfection 1.2-15 months after MIS-C. None of them experienced SAE or recurrence of MIS-C.

**Conclusion:** MIS-C was the most common manifestation and its incidence in this predominantly white population was higher than previously reported. Based on our limited experience, MIS-C does not seem to recur after SARS-CoV-2 reinfection or COVID-19 vaccination. Autoimmune diseases were much more common after SARS-CoV-2 infection than after COVID-19 vaccination. Hospitalisations due to MIS-C were seven times as frequent as hospitalisations due to severe COVID-19 in children.

**Disclosure of Interest**: None declared

### P455. Comparison of demographics, presentation and short-term outcomes in mis-c across different variants of SARS-COV-2 in Cape town, South Africa

#### C. Butters^1,2^, D. R. Abraham^3^, T. Spracklen^2^, J. Day^2^, H. Rabie^3^, C. Scott^2^, K. Webb^2^

##### ^1^Pathology, ^2^Paediatrics and Child Health, University of Cape Town, ^3^Tygerberg Hospital, Stellenbosch University, Cape Town, South Africa

###### **Correspondence:** J. Day

**Introduction:** The impact of the variants of concern (VOC) of SARS-CoV-2 on Multisystem Inflammatory Syndrome in Children (MIS-C) on disease phenotype and severity is not yet understood.

**Objectives:** Describe the demographic and clinical features of MIS-C across the four variant specific SARS-CoV-2 waves.

**Methods:** Children with MIS-C admitted to the Red Cross War Memorial Children’s Hospital (RXH) and Tygerberg Hospital (TBH) between 22 June 2020 and 27 March 2022 were recruited. Demographic, clinical and outcome data were recorded.

**Results:** There were four SARS-CoV-2 waves in Cape Town, South Africa during the time period driven by the wildtype, beta, delta and omicron variants respectively. During the time period, 129 children had confirmed MIS-C. There was no significant difference in age across the four waves, however children in the fourth wave tended to be lower (Table 1). There was no difference in sex distribution or the number of children with comorbidities. The presence of clinical signs and symptoms remained relatively constant with only diarrhoea being less prevalent in the wave driven by Omicron (p=0.028). Blood levels of inflammatory, coagulation and cardiac markers, markers, immune cells, and sodium were unchanged throughout the four waves. Abnormal ECHO findings were common, but no more so in one wave than another. However, there was a trend towards an increase in the presence of coronary artery involvement in the second and third waves (p=0.056) and reduced ejection fraction in the first and fourth waves (p=0.065). There was no difference in the need for ICU admission, inotropic support or length of hospital stay. There was only one death in the wave driven by the Omicron variant.

**Conclusion:** Overall, there does not appear to be a difference in the demographic, clinical or outcomes of MIS-C across the four waves of SARS-CoV-2 infection in Cape Town, South Africa. In this setting MIS-C remains a severe disease with a stable phenotype.

**Patient Consent:** Yes, I received consent

**Disclosure of Interest**: None declared

**Table 1 (abstract P455).** See text for description

	Totals	Wave 1	Wave 2	Wave 3	Wave 4	p Value
**VOC**		Wildtype	Beta	Delta	Omicron	
**Cases n (%)**	129	49 (38.0)	21 (26.2)	43 (33.3)	16 (24.2)	
**Age in years median (IQR)**	6.85 (3.39, 9.28)	7.37 (3.39, 10.00)	7.30 (4.17, 7.84)	6.37 (4.20, 9.11)	4.93 (2.63, 8.27)	**0.815**
**Diarrhea** **n (%)**	63 (48.8)	28 (57.1)	13 (61.9)	19 (44.2)	3 (18.8)	**0.028**
**Abnormal ECHO any abnormality (%)**	90/127 (70.9)	37 (75.7)	14 (66.7)	28/42 (66.7)	11/15 (73.3	**0.724**
**Coronary artery (%)**	10/128 (7.8)	1 (2.0)	3 (14.3)	6/42 (14.9)	0 (0.0)	**0.056**
**EF (median)**	59 (47, 65)	51 (43, 63)	64 (46, 68)	61 (53, 68)	58 (53, 63)	**0.065**
**Duration hospitalization**	7 (6, 10)	7 (6, 10)	6 (5, 9)	7 (6, 9)	7 (5, 14)	**0.670**

1 Highest

2 Lowest

### P456. An update of the international registry on COVID-19 related hyperinflammation in children and young adults (hyperped-covid)

#### R. Caorsi^1^, A. Consolaro^1,2^, C. Bracaglia^3^, F. Minoia^4^, M. Cattalini^5^, P. Brogan^6^, C. Wouters^7^, A. Taddio^8^, F. Candotti^9^, I. Meyts^10^, F. De Benedetti^3^, N. Ruperto^11^, M. Gattorno^1^ on behalf of on behalf of the Steering Committee for HyperPED-COVID registry for RITA-ERN, ISSAID, PRES, ESID, PRINTO

##### ^1^Clinica pediatrica e reumatologia, Gaslini Institute, Genoa, Italy, ^2^Dinogmi, Università degli studi di Genova, Genova, ^3^Division of Rheumatology, IRCCS Ospedale Pediatrico Bambino Gesù, Roma, ^4^Pediatric rheumatology, Fondazione IRCCS Ca’ Granda-Ospedale Maggiore Policlinico, Milano, ^5^Pediatrics Clinic, University of Brescia and ASST Spedali Civili di Brescia, Brescia, Italy, ^6^UCL GOS Institute of Child Health, London, United Kingdom, ^7^Pediatric Rheumatology and Immune-inflammatory diseases, University of Leuven, Leuven, Belgium, ^8^Institute for Maternal and Child Health IRCCS “Burlo Garofolo” and Univeristy of Trieste, Trieste, Italy, ^9^Division of Immunology and Allergy, Laboratory of Inherited Immune Disorders, CHUV, Lausanne, Switzerland, ^10^Department of Microbiology and Immunology, University of Leuven, Leuven, Belgium, ^11^Printo, IRCCS Istituto Giannina Gaslini, Genova, Italy

###### **Correspondence:** R. Caorsi

**Introduction:** The Multisystem Inflammatory Syndrome in Children (MIS-C), is a serious inflammatory condition characterized by a systemic inflammation with multiorgan failure, that can occur in children and young adults after COVID-19 infection; the clinical and laboratory features are similar to those usually observed in Kawasaki disease, cytokine storm syndrome and macrophage activation syndrome.

**Objectives:** to create an International multicenter collection of patients with MIS-C involving the main pediatric networks committed in the care of patients with hyperinflammatory conditions. Primary endpoint of the project is to collect information on clinical presentation, laboratory parameters, clinical outcome and response to treatment of patients with MIS-C.

**Methods:** a steering committee constituted by representatives of ERN-RITA, PRES, ESID and ISSAID and with the coordination of PRINTO developed a shared form to collect clinical manifestations, laboratory features, response to treatment and outcome of patients with MIS-C. The registry is available online on PRINTO and ESID websites (www.printo.it , www.ESID.org).

**Results:** Currently, more than 1000 patients from 44 centers of 20 countries worldwide have been included in the study; completed data are available for 688 patients. At the time of admission, 56 (8%) patients were younger than 2 years, 173 (25%) between 2 and 6 years, 279 (41%) between 6 and 12 years, while 180 (26%) patients were older than 12 years. 234 (34%) patients required ICU admission; 51 (7.4%) patients presented long term sequaele and 7 (1%) patients died.

Mucocutaneous manifestation were observed in 586 patients (85.2 %), hematological in 583 (84.7%), gastrointestinal in 556 (80.8%), cardiovascular in 360 (52.3%), lymphoid organ in 356 (51.7%), respiratory in 238 (34.6%), musculoskeletal in 217 (31.5%), neurological in 131 (19%), genito-urinary in 75 (10.9%).

Most common treatment were Ig infusion (576 patients, 84%) and corticosteroids (572 patients, 83%); 78 patients (11%) were treated with biologics.

**Conclusion:** The first analysis of the registry confirms that MISC is a severe inflammatory condition, requiring anti-inflammatory treatment. Even if the mortality and the complication rate is low, one third of patients required intensive care unit admission. Further analysis will be performed in order to identify predictors of outcome.

**Patient Consent:** Yes, I received consent

**Disclosure of Interest**: None declared

### P457. MIS-C and severe COVID-19 in children from the amazonian intensive care unit: poor outcome and high mortality rate

#### E. Farias^1^, G. Clemente^2^, M. T. Terreri^2^, M. Pavão Junior^1^, S. Sales^1^, L. Nascimento^1^, D. Pavão^1^, V. Santos^1^, A. Pinheiro^1^, M. Alves^1^, P. Carvalho^3^

##### ^1^Pediatrics, Centro Universitário Metropolitano da Amazônia, Belém, ^2^Pediatrics, UNIVERSIDADE FEDERAL DE SÃO PAULO, São Paulo, ^3^Pediatrics, Fundação Hospital das Clínicas Gaspar Viana, Belém, Brazil

###### **Correspondence:** G. Clemente

**Introduction:** Some children and adolescents may develop critical disease related to SARS-CoV-2, such as severe coronavirus disease (COVID-19) or multisystem inflammatory syndrome in children (MIS- C).

**Objectives:** Our aim was to study the outcome of patients with critical disease related to SARS-CoV-2 hospitalized in Paediatric Intensive Care Units in the Brazilian Amazonian region.

**Methods:** This multicentre cohort included children and adolescents with critical disease related to SARS-CoV-2 current or recent infection who were admitted into three Amazonian PICUs between April 2020 and January 2022. Exclusion criteria were patients who had positive microbiology other than SARS-CoV-2, had no criteria for PICU admission, or were immunocompromised.

**Results:** A total of 192 patients were included: 65 (33.9%) from the MIS-C group and 127 (66.1%) from the severe COVID-19 group. The total median age was 3.5 (0.8-8.6) years; 4.4 (1.0-9.3) years in MIS-C and 2.3 (0.75-8.4) years in severe COVID-19 group (p=0.241). Previous history of complex chronic condition was present in 123 (64.1%) patients. Almost half of the patients (94 [49%]) required mechanical ventilation support. The median mechanical ventilation time was 4 (3-9) days, and median length of hospital stay was 14 (10-20) days. Death occurred in 35 (18.2%) patients, 16 (24.6%) in MIS-C group and 19 (15%) in severe COVID-19 group, with no statistical difference. The results of SARS-CoV-2 tests and outcomes are described in Table 1.

**Conclusion:** In the Brazilian Amazonian region, we observed elevated requirement for mechanical ventilation support, long length of hospital stay, and high number of deaths in paediatric patients with critical disease related to SARS-CoV-2.

**Patient Consent:** Yes, I received consent

**Disclosure of Interest**: None declared

**Table 1 (abstract P457).** Results of SARS-CoV-2 tests and outcomes of children and adolescents with critical disease related to SARS-CoV-2 (severe COVID-19 and MIS-C)

	Severe COVID-19 (n=127)	MIS-C (n=65)	All patients (n=192)	***p*** value
**SARS-CoV-2 tests**				
SARS-CoV-2 RT-PCR positive	56/75 (74.7)	21/61 (34.4)	77/136 (56.2)	0.013
SARS-CoV-2 Antigen positive	60/83 (72.3)	28/52 (53.8)	88/135 (65.2)	0.32
SARS-CoV-2 ELISA IgG positive	4/47 (8.5)	16 /21(76.2)	20/68 (29.4)	<0.001
SARS-CoV-2 ELISA IgM positive	11/47 (23.4)	4 /21 (17.4)	15/68 (22.1)	0.05
**Outcomes**				
Length of PICU stay, in days	3 (2-9)	6 (3-12)	5 (2-10.5)	<0.001
Length of hospital stay, in days	14 (10-20)	15 (10-19)	14 (10-20)	0.15
Deaths	19 (15)	16 (24.6)	35 (18.2)	0.13

### P458. The role of SARS-COV-2 vaccination and SARS-COV-2 reinfection in children with previous multisystem inflammatory syndrome (MIS-C)

#### F. Corona^1^, F. Burlo^1^, S. Pastore^2^, M. V. Mastrolia^3^, F. Ricci^4,5^, S. A. Rumeileh^3^, M. Cattalini^4,5^, G. Simonini^3^, A. Taddio^1,2^

##### ^1^Department of Medicine, Surgery, and Health Sciences, University of Trieste, ^2^Institute for Maternal and Child Health - IRCCS “Burlo Garofolo” , Trieste, ^3^Pediatric Rheumatology Unit, Meyer Children’s University Hospital, School of Human Health Science, Florence, ^4^Pediatrics Clinic, University of Brescia, ^5^ASST Spedali Civili, Brescia, Italy

###### **Correspondence:** A. Taddio

**Introduction:** Multisystem inflammatory syndrome in children (MIS-C) is a rare hyperinflammatory and life-threatening complication that usually occurs 2–6 weeks after SARS-CoV-2 infection.

**Objectives:** Many studies have investigated the short and long-term consequences of this condition, but the potential risks of SARS-CoV2 vaccination or reinfection in those children who have previously been hospitalized for MIS-C are still lacking.

**Methods:** This is a multicenter retrospective study which investigated the rate and even the consequences of SARS-CoV-2 vaccination or reinfection in children who were hospitalized for MIS-C at Institute for Maternal and Child Health Burlo Garofolo in Trieste, at Meyer Children’s University Hospital in Firenze, and at the Paediatric Clinic of ASST Spedali Civili in Brescia, Italy. The parents of these children were asked if they were vaccinated or if they were reinfected with SARS-CoV-2 after MIS-C diagnosis.

**Results:** Overall, 69 children were investigated. The median age was 10 years (range: 1-19). There were 43 males and 26 females. They developed MIS-C from April 2020 to April 2022. Afterwards, 23 patients were vaccinated against SARS-CoV2 infection. Ten patients could not be vaccinated because younger than 5 years-old. The median time from MIS-C to the first dose of vaccine was ten months (range: 6-15). Just 7 patients complained mild symptoms soon after the vaccination, such as headache, fatigue or pain at the injection site. Moreover, 9 patients overall got another Covid infection after MIS-C. The median time from MIS-C to reinfection was 13 months (2-18). Two of them were hospitalized for fever and gastrointestinal or upper airway symptoms respectively and were discharges 2 days after, 1 was asymptomatic, 7 developed mild symptoms such as sore throat, cough and mild fever, and 2 of the latter were vaccinated against SARS-CoV2. No one reported a new MIS-C diagnosis.

**Conclusion:** This study reports that MIS-C was not a risk factor for severe complications in case of SARS-CoV2 reinfection or vaccination. All the symptoms complained by children in case of reinfection or vaccination were mild. In particular, no new MIS-C cases were reported in this cohort of patients.

**Disclosure of Interest**: None declared

### P459. The cytokine profile of cerebrospinal fluid in multi-system inflammatory syndrome in children

#### J. Day^1^, U. Rohlwink^2^, T. Spracklen^1^, C. Butters^1^, R. Stander^1^, H. Facey-Thomas^1^, A. Davis^3^, C. Scott^1^, L. Zühlke^4^, K. Webb^1^

##### ^1^Paediatric Rheumatology, ^2^Neuroscience, University of Cape Town, Cape Town, South Africa, ^3^School of Life and Medical Sciences, UCL, London, United Kingdom, ^4^Paediatrics, University of Cape Town, Cape Town, South Africa

###### **Correspondence:** J. Day

**Introduction:** Multi-inflammatory syndrome in children (MIS-C) is characterized by hyperinflammation following infection or exposure to SARS-CoV-2 in children and young people. Approximately a third of MIS-C cases have central nervous system manifestations, the pathogenesis of which is unknown. Here, we characterize the cytokine profile of the cerebrospinal fluid (CSF) of a small cohort of children with MISC with neurological manifestations.

**Objectives:** To characterise the cytokine milieu of cerebrospinal fluid in those with MIS-C compared to controls.

**Methods:** CSF was acquired via lumbar puncture, as part of in-hospital clinical care, from 3 children who met the criteria for MIS-C with neurological manifestations and 3 control patients presenting for procedures requiring CSF; such as revision of a blocked ventriculoperitoneal shunt. Cytokine concentrations were measured via chemiluminescence and analyzed using SPSS statistical software.

**Results:**

**Conclusion:** In a small number of patients, we show that certain inflammatory cytokines are raised in the CSF of children with MIS-C and neurological manifestations. Understanding the specific cytokine profile of this condition may be key in efforts to understand pathogenesis, and identify potential treatment targets. These data underpin the need for further studies investigating the mechanism of CNS disease in MIS-C.

**Patient Consent:** Yes, I received consent

**Disclosure of Interest**: None declared

**Table 1 (abstract P459).** See text for description

	MIS-C	CONTROL		
CSF CYTOKINE	Mean	Range	Mean	Range
IL 6	48.6	16-97.5	6.2	3.1-13
CXCL10	108.2	27-223.5	15.3	0-45.8
IL 1B	0.3	0-1	0	0
G-CSF	860.4	0-2406.6	32.3	7.3-53.6
IL10	8.5	0.75-19.4	0.25	0-0.75
IL 17A	1.3	0.5-2.7	0.3	0-0.8

### P460. Determination of factors to distinguish Multisystem Inflammatory Syndrome (MIS-C) from other acute febrile illness in the emergency department

#### M. M. N. F. Soares, A. L. C. Bessa, L. R. M. P. Crivelenti, V. P. Baldão, D. C. Aragon, M. C. Cervi, A. K. Matsuno, R. A. P. de La Ossa, L. M. De Carvalho

##### ^1^Pediatrics, Ribeirão Preto Medical School, University of São Paulo, Ribeirão Preto, Brazil

###### **Correspondence:** L. M. De Carvalho

**Introduction:** Until the moment, Multisystem Inflammatory Syndrome (MIS-C), temporally associated with SARS-CoV-2 infection is a challenging diagnosis in pediatric practice. As the signs and symptoms associated with the syndrome are very common to other serious febrile illness, the confirmation of the previous infection by RT-PCR or serology for SARS-CoV-2, is not enough in some cases, given the co-infection cases and coincidental relationship. More sensitive and specific criteria for syndrome classification are necessary.

**Objectives:** To establish clinical and laboratory criteria to differentiate, at admission, patients with MIS-C from patients with other febrile syndromes unrelated to SARS-CoV-2 infection.

**Methods:** Cross-sectional observational study of children and adolescents under 18 years of age with febrile syndromes who manifested with signs and symptoms characteristic of MIS-C, attended at the Clinical Hospital of the Ribeirão Preto Medical School - São Paulo University, in the period of March of 2020 to October 2021. The diagnosis of MIS-C was performed based on CDC and/or WHO criteria. Data from patients with MIS-C and other febrile syndromes not associated with SARS-CoV-2 at admission were compared using Fisher’s exact and Wilcoxon tests, with a level of significance of 5%. The measure of association used was the relative risk (RR) and the adjusted relative risk (RRaj) for the variables that were significantly different between the groups with 95% CI. The Hospital Ethics and Research Committee approved the study.

**Results:** During the study period, 44 children and adolescents under 18 years of age presented any of the clinical and laboratory criteria for MIS-C at admission. MIS-C temporally associated with SARS-Cov-2 infection was confirmed in 23 patients (group 1). The final diagnosis of the others febrile syndromes (group 2) were: acute viral gastroenteritis, atypical Kawasaki, acute viral bronchiolitis, viral myocarditis, mononucleosis-like syndrome, pneumonia, post-streptococcal glomerulonephritis, mesenteric adenitis, type 1 diabetes mellitus and staphylococcal shock. The groups were clinical and laboratory similar, except for older age, presence of skin rash, previous antibiotic use, hyponatremia and increase in C-reactive protein (CRP) which were more frequent in the group 1. CRP above 6.4 mg/dl increased the risk of the patient being in the MIS-C group at 3 times, even when the value was adjusted for previous antibiotic use.

**Conclusion:** The clinical and laboratory criteria associated with MIS-C are similar to other diseases. In our study, the CRP above 6.4 mg/dl at admission was the main risk factor associated with MIS-C.

**Disclosure of Interest**: None declared

### P461. Prospective cohort of patients with pediatric multisystem inflammatory syndrome temporally associated with SARS-COV-2 infection followed up in a tertiary hospital

#### A. L. C. Bessa^1^, M. M. N. F. Soares^1^, L. R. M. P. Crivelenti^1^, W. A. D. O. Borghi^1^, P. H. F. Muccillo^1^, M. C. Cervi^1^, R. A. P. de La Ossa^1^, L. M. De Carvalho

##### ^1^Pediatrics, Ribeirão Preto Medical School, University of São Paulo, Ribeirão Preto, Brazil

###### **Correspondence:** L. M. De Carvalho

**Introduction:** Multisystem inflammatory syndrome in children (MIS-C) is a potentially severe pediatric hyperinflammation disease associated with SARS-CoV-2 infection. The acute manifestations of the disease have been extensively described but the evolution profile studies are still limited.

**Objectives:** To describe the medium-term evolution profile of a cohort of MIS-C patients followed in a tertiary care clinic.

**Methods:** Prospective cohort study of 12 months follow-up of patients under 18 years of age in a specialized multidisciplinary outpatient clinic in a Brazilian hospital, diagnosed as MIS-C between March 2020 and December 2021. Assessments of anthropometric data, clinical complaints, biochemical markers, and imaging tests were performed at a mean of 1, 3, 6, 9 and, 12 months after admission. For continuous data comparison were used Whitney test (2 groups) or the Kruskal-Wallis test (3 or more groups). A significance level of 5% was established.

**Results:** Twenty-two patients met CDC and/or WHO criteria for MIS-C and had laboratory evidence of SARS-CoV-2 infection. Patients generally had a good outcome during the follow-up period. The average length of stay of patients was 8 days, with an average of 4 days in an intensive care unit. The mean time of outpatient follow-up was nine months, with 9 patients followed up for one year. There were no deaths. One patient evolved with pulmonary pneumatocele. Thirty-two percent of patients had cardiovascular changes at presentation, including left ventricular systolic dysfunction, valvular insufficiency, pericardial effusion, and hypokinesis of the cardiac wall. Only one patient maintained cardiac chamber enlargement during follow-up, without clinical repercussions. Coronary changes were not described. One of patients developed stage 1 arterial hypertension. Fourteen percent of the patients had transient renal changes. Gastrointestinal symptoms were reported in 82% of the patients and 9% had abdominal pain after 6 months of follow-up. One of patients developed chronic arthritis during follow-up. All patients had elevated markers of systemic inflammation and troponin and NT-pro-BNP concentrations at baseline, with normalization of these tests within one month, except for 2 patients who maintained elevated VHS for 6 months.

**Conclusion:** This study supports the idea of ​​an optimistic short and medium-term outcome for MIS-C, despite the severe initial presentation.

**Disclosure of Interest**: None declared

### P462. Patients with systemic juvenile idiopathic arthritis treated with IL-1 inhibitors: what happens if SARS-COV2 infection occurs?

#### A. De Matteis^1^, M. Pardeo^1^, G. Marucci^1^, F. De Benedetti^1^, C. Bracaglia

##### ^1^Division of Rheumatology, IRCCS Ospedale Pediatrico Bambino Gesù, Roma, Italy, Roma, Italy

###### **Correspondence:** C. Bracaglia

**Introduction:** Systemic juvenile idiopathic arthritis (sJIA) is a polygenic autoinflammatory disease in which inflammatory cytokines, such as IL-1 and IL-6, play a pivotal role. Macrophage activation syndrome (MAS) is the most severe potentially life-threatening complication of sJIA and it can be triggered by infections. Only few reports describe the clinical course of SARS-CoV-2 infection in sJIA patients during treatment with cytokine inhibitors. Moreover, the role of IL-1 inhibitors in preventing the development of MIS-C is not known.

**Objectives:** We report 9 cases of SARS-CoV-2 infections occurred in sJIA patients during IL-1 inhibition treatment.

**Methods:** Demographic, clinical and laboratory features of the 9 patients (7 female, median age 7 years) were summarized in table 1.

**Results:** All patients were in clinical inactive disease (CID) at time of SARS-CoV-2 infection and the infection was pauci-symptomatic. Two patients were hospitalized to be monitored. No episodes of MAS, MIS-C nor lung involvement occurred. Three patients were in treatment with canakinumab and 6 with anakinra; 2 patients were also on low dose of prednisone. Five out of 9 patients continued IL-1 inhibitors at the same dose, 3 patients increased the dose, while one patient shifted from canakinumab to anakinra (table 1).

**Conclusion:** In our sJIA patients treated with IL-1 inhibitors, SARS-CoV-2 infection was not associated with disease flare or with worse clinical outcome. SARS-CoV-2 infection did not trigger MAS in any of those patients and none of them developed MIS-C. Considering the role of IL-1 in the pathogenesis of the cytokine storm syndrome, we may hypothesize the preventing role of IL-1 inhibitors in the disease flare during SARS-CoV-2 infection.

References:

Demir F, Ulu K, Çağlayan Ş, Coşkuner T, Sözeri B. Clinical course of COVID-19 in children with rheumatic disease under biologic therapy. Clin Exp Rheumatol. 2021

de Carvalho JF. COVID-19 in a pregnant ASIA syndrome patient. Eur Rev Med Pharmacol Sci. 2021

Calvo C, Udaondo C; Rheumatic Diseases EPICO-AEP Working Group. COVID-19 in Children With Rheumatic Diseases in the Spanish National Cohort EPICO-AEP. J Rheumatol. 2021

**Patient Consent:** Yes, I received consent

**Disclosure of Interest**: A. De Matteis: None declared, M. Pardeo: None declared, G. Marucci: None declared, F. De Benedetti Consultant with: Abbvie, SOBI, Novimmune, Novartis, Roche, Pfizer, Employee with: SOBI, C. Bracaglia Consultant with: SOBI and Novartis

**Table 1 (abstract P462).** Patients’ clinical and laboratory features

Pt (age in yrs)	Pt. 1 (14)	Pt. 2 (15)	Pt. 3 (11)	Pt. 4 (17)	Pt. 5 (9)	Pt. 6 (10)	Pt. 7 (20)	Pt. 8 (9)	Pt. 9 (18)
Age at sJIA onset (yrs)	13	6	6	13	4	10	2	4	7
History of MAS	Yes	No	No	Yes	Yes	No	Yes	Yes	Yes
Treatment at time of infection (dose)	Anakinra (2 mg/Kg/day)	Canakinumab (4 mg/kg every 7 weeks)	Canakinumab (7 mg/kg every 4 weeks) Prednisone (0,1 mg/kg/day)	Anakinra (2 mg/kg/day)	Canakinumab (5 mg/kg every 4 weeks)	Anakinra (2 mg/kg/day)	Anakinra (3 mg/kg/day Prednisone (0,2 mg/kg/day)	Anakinra (4 mg/kg/day)	Canakinumab (3 mg/kg every 12 weeks)
Comorbidity	No	No	cardiopathy	epilepsy, polineuropathy	No	No	No	No	epilepsy
**SARS-COV2 infection**									
Symptoms	Rhinitis	Fever, ageusia, anosmia, arthralgia, vomiting	Headache	Rhinitis	Fever, rhinitis	Fever, myalgia	Fever, rhinitis, pharyngodynia	Rhinitis	Fever
Laboratory analysis	Not done	In the normal range	Not done	↑ CRP	↓ PLTs, ↓ WBC	Not done	Not done	Not done	Not done
Hospitalization	No	Yes	No	No	Yes	No	No	No	No
Additional treatments (dose)	Azithromycin Increased dose of Anakinra (3,5 mg/kg/day)	Anakinra (2 mg/kg/day)	No	No	No	Increased dose of Anakinra (4 mg/kg/day)	No	No	Increase dose of Canakinumab (3 mg/kg every 4 weeks)

### P463. COVID-19-associated vasculitis/vasculopathy

#### N. Emeršič^1^, M. Debeljak^2,3^, A. Koren Jeverica^1^, G. Markelj^1^, N. Toplak^1,3^, T. Vesel Tajnšek^1^, M. Zajc Avramovič^1^, T. Avčin^1,3^

##### ^1^Department of Allergology, Rheumatology and Clinical Immunology, University Medical Centre Ljubljana, Children’s Hospital, ^2^Clinical Institute for Special Laboratory Diagnostics, University Children’s Hospital, University Medical Centre Ljubljana, ^3^Department of Pediatrics, Faculty of Medicine, University of Ljubljana, Ljubljana, Slovenia

###### **Correspondence:** N. Emeršič

**Introduction:** Since the onset of the 2019 coronavirus disease pandemic (COVID-19) caused by the severe acute respiratory syndrome virus, coronavirus type 2 (SARS-CoV-2), many articles have been published describing non-infectious manifestations associated with the infection, including COVID-19-associated vasculitis/vasculopathy. In children, most of these vascular complications are chilblain-like lesions.

**Objectives:** To determine possible risk factors, which contribute to the development of vasculitis/vasculopathy in children, to determine the role of genetics, immunological profile of the patients and possible endothelial cells changes, to evaluate long term outcome of these complications and to offer possible treatment options.

**Methods:** In our prospective cohort study, we included children up to 18 years of age with COVID-associated vasculitis/vasculopathy, who were examined at Children’s hospital University Medical centre Ljubljana, Slovenia from March 2020 until May 2022. Data collection included epidemiological, clinical information, associated diseases, extended blood analyses and genetic information, which were sistematically evaluated and compared. All children were tested for specific anti-SARS-CoV-2 immunoglobulin antibodies and had genetic analyses for TREX1, type I interferonophaties and monogenic vasculitides. Changes on the skin were dermatoscopically observed and evaluated with thermographic camera. We searched for similarities and differences from known vasculitidies and systemic connective tissue diseases with emphasis on clinical and immunological profile of the patients. Approval from the institutional Ethics Committee has been obtained.

**Results:** 45 children were diagnosed with COVID-19-associated vasculitis/vasculopathy. The estimated incidence was one in 1522 children after SARS-CoV-2 infection. All included patients were Caucasian. The mean age at diagnosis was 12 years (2 months-17,5 years). 29/45 patients (64%) were female. The most common changes were chilblain-like lesions. 20/44 (45%) patients were tested positive for SARS-CoV-2 antibodies. In 24/45 (53%) patients, only lower extremities were affected, in 6/45 (13 %) patients only upper extremities and in 10/45 (22%) patients upper and lower extremities were affected, one girl had changes only on her face and another on the other parts of the body. The skin changes were usually mildly painful, some of the patients complained of pruritis. Vasculitis was more severe in younger children, where other organs, not just skin, may have been involved. A 1-year old girl developed necrotizing stomatitis, vasculitic rash, skin desquamation on the fingers and toes, and persistent hypertension. A 2-month-old boy developed skin and severe gastrointestinal vasculitis. Both patients with severe vasculitis had negative genetic analysis for TREX1, type I interferonophaties and monogenic vasculitides. A 13-years old boy developed Raynaud’s phenomenon after the second dose of SARS-CoV-2 vaccination. In 10 patients the problems recurred. In majority of patients there were some dilated capillary loops along fingernails. The affected parts of the limbs photographed with thermographic camera showed elevated temperature compared to unaffected areas.

**Conclusion:** In children, COVID-19-associated vasculitis is the second most common autoimmune manifestation after SARS-CoV-2 infection. Most patients have uneventful disease course with complete resolution. 4,4% (2/45 patients) had severe vasculitis with internal organ involvement or mutilating skin and musculoskeletal lesions.

**Patient Consent:** Yes, I received consent

**Disclosure of Interest**: None declared

### P464. Inflammatory markers linked to cardiovascular involvement in Multisystemic Inflammatory Syndrome in Children (MIS-C) among the paediatric population of a third level hospital at Northeast Mexico

#### J. M. Galindo Hayashi^1^, K. González Moctezuma^1^, L. F. González González^1^, G. R. Ayala Villegas^1^, J. G. Rodriguez Chong^1^, A. V. Villarreal Treviño^2^

##### ^1^Pediatrics, ^2^Pediatric Rheumathology, Programa Multicentro TecSalud, Monterrey, Mexico

###### **Correspondence:** J. M. Galindo Hayashi

**Introduction:** Cardiovascular involvement has been found in 86.5% of patients with multisystem inflammatory syndrome in children (MIS-c) (1). One of MIS-c´s diagnostic criteria are elevation of inflammatory markers (2).

**Objectives:** To analyse inflammatory markers to determine which of them are linked with cardiovascular involvement in MIS-c among the paediatric population of a third level hospital in northeast Mexico.

**Methods:** The researchers of this study conducted a cross-sectional retrospective study. The World Health Organization (WHO) MIS-c criteria were used to choose the eligible patients to analyse. Cardiovascular involvement was confirmed by transthoracic echocardiography (TTE).

The researchers dichotomize MIS-c patients in two groups, those with and without cardiovascular involvement. Descriptive statistics were performed by calculating the mean and standard deviation (SD) for each group. To test stastitical significance between inflammatory markers in the two groups we used the Mann-Whitney *U* test (*U*). The following markers were analysed: CRP, ERS, Ferritin, D-dimer, albumin, CK, CK-MB, neutrophils, lymphocytes, DHL, leukocytes, platelets, and fibrinogen.

**Results:** 70 patients met the eligibility criteria, six were excluded for not having an TTE, and four for having an incomplete medical record. From the 60 patients left, 58.2% had cardiovascular involvement confirmed by TTE (n=25). We found higher prevalence of cardiovascular involvement in males (56%) and the mean age was 7 years and 1 month.

The mean, standard deviation (SD) and p values are represented in the following table (Table 1).

**Conclusion:** Markers such as BNP and IL-6 have shown association to cardiovascular involvement in MIS-c (3). Nevertheless, these markers aren’t commonly available in health centres in third world countries.

A panel of specialists with expertise in MIS-C, concluded that some patients with mild symptoms may require only close monitoring without immunomodulatory treatment (4). These could set ground of exclusive use of IVIG and/or steroids in patients with suggestive inflammatory markers of cardiovascular involvement.

This study found that there is suggestive evidence that platelets and neutrophils could be used as markers of risk of cardiovascular involvement in MIS-c.

The results of this study could serve as precedent to a prospective randomized experimental trial to evaluate if a series of inflammatory markers and clinical features could predict accurately cardiovascular involvement in MIS-c. With the final purpose of prognosticating which patients would benefit the most from IVIG and/or steroid therapy in centres with limited resources.

**Trial registration identifying number:** Not applicable.

**Patient Consent:** Yes, I received consent

**Disclosure of Interest**: None declared

**Table 1 (abstract P464).** See text for description

	CRP (mg/dL) Mean (SD)	Ferritin (ng/dL) Mean (SD)	D-dimer (mg/dL) Mean (SD)	Fibrinogen (mg/dL) Mean (SD)	Leukocytes (/mm3) Mean (SD)	Lymphocytes (/mm3) Mean (SD)	Neutrophils (/mm3) Mean (SD)	Platelets (/mm3) Mean (SD)
**With cardiovascular involvement** (n=25)	18 (±12)	1,068 (±716)	9,887 (±7,035)	800 (±223)	16,034 (±10,974)	2,028 (±2,478)	12,978 (±9,359)	192,208 (±85,423)
**Without cardiovascular involvement** (n=35)	12 (±9.8)	2,541 (±2,883)	5,548 (±7,656)	627 (±282.2)	11,257 (±6,870)	1,716 (±1,493)	8,642 (±6,428)	190,950 (±136,514)
U. Mann	p=0.05	p= 0.26	p=0.21	p=0.06	p=0.05	p=0.49	p=0.04*	p≤0.01*

The laboratory exams were taken at admission, prior any intervention (corticosteroids or IVIG). CK-MB was not included for having a critical *U* distribution. CK, ESR, albumin and DHL had a non-significant p. *CRP: C-Reactive protein, ESR: Erythrocyte sedimentation rate, CK: Creatine Kinase, CK-MB Creatine Kinase-MB, U: Mann-Whitney distribution, SD: Standard deviation*

### P465. Multisystem Inflammatory Syndrome in Children (MIS-C) with rapid progression: a case report

#### G. A. Turrubiates-Hernández^1^, A. V. Villarreal-Treviño^2^, J. M. Galindo-Hayashi^1^

##### ^1^Pediatrics, ^2^Rheumatology, Tecnologico de Monterrey, Escuela de Medicina y Ciencias de la Salud, Monterrey, Mexico

###### **Correspondence:** J. M. Galindo-Hayashi

**Introduction:** MIS-C is a novel disease reported for the first-time weeks after the first cases of COVID-19 in 2020 were known. It is a heterogeneous vasculitis which can be difficult to diagnose since it can overlap to other entities.

**Objectives:** NA

**Methods:** NA

**Results:** A previously healthy 8-year-old female presented to the ER with a history of 5 days of cervicalvolume increase, diarrhea, gastric content vomit and a 24hour subjective report of temperature rise with diffuse colic abdominal pain, hyporexia, myalgias, arthralgias, non-purulent conjunctivitis, hands and feet edema and malaise. Hours before admission, she presented palpebral hyperemia, pruriginous maculopapular wheal-like lesions on the neck, trunk, abdomen, legs and genitals as well as cracked injected lips.

Three weeks prior, she presented with a mild upper respiratory tract infection. History of recent contact with patients COVID-19 positive was denied. On arrival, she was tachypneic (35 bpm), oxygen saturation of 95% under room air, tachycardic (136 bpm), hypotense (77/32) and with a 37°C temperature. Initial blood tests revealed kidney and liver dysfunction, leukocytosis, neutrophilia, lymphopenia, thrombocytopenia, elevated CRP, ferritin, and D-dimer, with a negative SARS COV 2 rapid antigen test (Table 1). Abdominal ultrasound revealed increased echogenicity of liver and kidneys. Echocardiography showed mild pericardial effusion and pericarditis with ejection fraction of 60%.

Treatment was initiated with methylprednisolone (30mg/kg/dose), norepinephrine (0.05mcg/kg/min), spironolactone (12.5mg q12h) and rivaroxaban (10mg/day), she was transferred to the PICU after subspecialists evaluation. On day 7 of illness kidney dysfunction showed recovery and IVIG at 2g/kg/dose was started. On day 8 noradrenaline was withdrawn and oral feeding was started. The 3-day methylprednisolone bolus course ended and the shift to 1.5mg/kg/day with following weaning was made. On day 10 hand and feet edema subsided and abdominal pain disappeared. On day 11 the patient was transferred from the ICU to the general ward. Fever was never reported. She demonstrated a good clinical course and was diagnosed with MIS-C based on CDC criteria. On day 19 she was discharged with normal liver, kidney, and cardiac function.

Treatment with antibiotics was not started since impaired consciousness, high fever, and livedo reticularis, which are usually noted in sepsis, were not present. The principal differential diagnosis was Kawasaki disease but, since 5 day- fever was not present, the patients age was not in the typical period, the history of an upper respiratory tract infection within the previous month, and blood test results, mild hyponatremia and elevated accute phase reactants, plus no other apparent cause of symptoms, MIS-C was suspected and the patient was treated as such.

In patients with MIS-C diagnosis fever lasting more than 48 hours is nearly present in 100%, interestingly, in our patient subjective temperature rise was reported just one time and never again during her hospitalization, furthermore the clinical course progressed fast towards shock, which was rapidly reversed with immunomodulatory treatment.

**Conclusion:** Our case study illustrates the importance of suspecting MIS-C when caring for patients under 21 years of age presenting with shock, even without clearly documented fever.

**Trial registration identifying number:** NA

**Patient Consent:** Yes, I received consent

**Disclosure of Interest**: None declared

**Table 1 (abstract P465).** See text for description

Day of illness	5	6	8	10	12	15
WBC (10x3/μL)	14.1	13.5	14.4	12.2	15.5	11.8
Neutrophils (10x3/μL)	13.2	12.3	12.7	9.3	12.1	9.5
Platelets (10x3/μL)	136	115	117	148	258	416
CRP (mg/dL)	8.57		9.59	2.85	1.43	0.49
Albumin (g/dL)	3	2.6	1.9	2.3	2.9	3.5
Creatinine (mg/dL)	1.08	0.57	0.3	0.33	0.32	1.8
Na (mEq/L)	132	135	137	135	135	
Ferritin (ng/mL)	588.6		359	266.8	283.2	357.9
D-Dimer (ng/mL)	4254		1557		3587	1589

### P466. Multisystem inflammatory syndrome in children: a year and a half of experience of university children’s hospital, Sofia, Bulgaria

#### M. Ganeva, A. Teltcharova, A. Dimitrova, D. Hristova, K. Temelkova, T. Vasilev, V. Kostova, S. Stefanov

##### ^1^Department of Pediatric Rheumatology and Cardiology, University Children’s Hospital, Medical University Sofia, Sofia, Bulgaria

###### **Correspondence:** M. Ganeva

**Introduction:** The novel coronavirus disease 2019 caused by severe acute respiratory syndrome coronavirus 2 (SARS-CoV-2) faced us with a great challenge - multisystem inflammatory syndrome in children (MIS-C). A patient is diagnosed with MIS-C based on the presence of fever, involvement of 2 or more organ systems, laboratory evidence of inflammation and evidence of SARS-CoV-2 infection or exposure and when other plausible diagnosis are excluded.

**Objectives:** To describe the demographic, epidemiological, clinical and laboratory parameters in patients diagnosed with MIS-C.

**Methods:** Demographic and clinical parameters of MIS-C patients admitted between November 2020 and May 2022 were recorded. Blood samples of MIS-C patients were collected at the time of admission to the Department of pediatric rheumatology and cardiology, Sofia, Bulgaria. Heart ultrasound was performed in all patients.

**Results:** Thirty-one MIS-C patients (15 girls and 16 boys) with a mean age of 7.35 years (1-17 years) were admitted for a period of 18 months. Nine patients had a history of household contact. Eight children had a history of SARS-CoV-2 illness. In all of the patients IgG SARS-CoV-2 antibodies were detected. Eighteen MIS-C patients were positive for IgM SARS-CoV-2 antibodies. Fever was observed in all children with a mean duration before admission of 5.67 days (3-10 days). Skin rash was noticed in 25 (80.6%) children. Kawasaki-like features were detected with 25 (80.6%) of the children having conjunctivitis. In ten MIS-C patients edema of the hands was noticed and twelve had erythema of the palms and soles. Eight of the children experienced cervical lymphadenitis. Periorbital edema and erythema were detected in ten children. Twenty-two patients (70.9%) had gastrointestinal symptoms - vomitting was observed in nine children, diarrhea in 5 patients and eight suffered from both. Myocarditis was diagnosed in nineteen (61.3%) children with eleven of them requiring inotropic support. Serositis was detected in sixteen (51.6%) patients. Neck pain was observed among eight (29%) children. Acute kidney injury occurred in two (6.4%) patients. All children had elevated CRP and ferritin at the time of admission with mean value of accordingly 193.47 mg/l (14.75-387.95 mg/l) and 1124.34 ng/ml (182.7-5708 ng/ml). ESR was not elevated in four of the patients. Lymphopenia was noted in twenty (64%) children. Seventeen (54.8%) patients had low platelets (92.4x10^9^/l (27-149 x10^9^/l)) at the time of admission. Hypoalbuminemia was detected in twenty-three children. Intravenous immunoglobulin treatment was performed in all but four children. Corticosteroids with an initial dose of 1.76 mg/kg were used in all MIS-C patients. Pulse steroid therapy was performed in one child. Low-molecular-weight heparin was injected subcutaneously in twenty-one children. Antiplatelet therapy was prescribed to twenty-six MIS-C patients. The mean hospital stay was 11.3 (6-23) days. All patients with myocarditis had favourable outcome and complete recovery.

**Conclusion:** Our data demonstrate that mucocutaneous and gastrointestinal symptoms are most commonly observed in MIS-C patients with over half of the children being diagnosed with myocarditis in our study cohort. Favourable outcome and complete recovery was observed in all myocarditis patients. Acute kidney injury was observed in two patients.

**Patient Consent:** Yes, I received consent

**Disclosure of Interest**: None declared

### P467. Immunogenicity and safety of SARS-COV-2 vaccine in patients with juvenile idiopathic arthritis

#### M. F. Gicchino, F. G. Abbate, A. Amodio, E. Miraglia del Giudice, A. N. Olivieri

##### ^1^Department of Woman, Child and General and Specialistic Surgery, University of the Study of Campania Luigi Vanvitelli, Naples, Italy

###### **Correspondence:** M. F. Gicchino

**Introduction:** Severe acute respiratory syndrome coronavirus-2 (SARS-CoV-2) continues to be an important health problem since December 2019 but the management of the infection and the pandemic trend considerably improved thanks to mass vaccination. The vaccination against SARS-CoV2 in patients with autoimmune inflammatory rheumatic disease is very important because these patients have a higher risk to get the infection and even more with a worse course. The two vaccines approved for them are the mRNA based BNT162b2 (BioNTech-Pfizer) and mRNA-1273(Moderna). Since May 2021 these vaccines have been approved also for children aged 12 and older, but there is still no data enough regarding their use in patients affected from rheumatological diseases as Juvenile Idiopathic Arthritis.

**Objectives:** To evaluate the immunogenicity and safety of vaccination against SARS-CoV2 infection in JIA patients.

**Methods:** We performed a retrospective observational study comparing the immunogenicity and safety of SARS-CoV-2 mRNA vaccine in JIA patients compared with healthy controls. We enrolled 36 patients and 33 healthy controls. For each patient we recorded JIA subtype, disease activity (according to JADAS-10), pharmacological treatment. According to EULAR/PRES recommendations all patients were in remission during vaccination and no one suspended pharmacological treatment. For both patients and controls we evaluated the side effects due to vaccination with telephone surveys. IgG antibodies against SARS-CoV-2 were quantified by CLIA method. Blood samples for the evaluation of Sars-Cov-2 antibodies were collected from all the subjects at the time of enrolment as well as one month after the second vaccination.

**Results:** In table are reported baseline characteristic, antibodies levels and side effects after immunization with Sars-Cov-2 vaccines among children affected by JIA compared with controls. In our cohort we recorded: 15 oligoarthritis, 13 polyarthritis (11RF negative, 2 RF positive polyarthritis), 4 enthesis’s related arthritis, 4 systemic arthritis. JIA treatments was assigned according to ACR recommendations, in particular 6 patients were in treatment with NSAIDs, 7 with methotrexate, 9 with methotrexate and biological drugs, 14 with only biological drug. Regarding antibodies levels we did not found any statistically significant differences comparing the average level of antibodies between patients and controls. Evaluating JIA subtype we found that patients with systemic JIA produced lower levels of antibodies compared with patients with oligoarthritis (p = 0.05) polyarthritis (p = 0.03) and entesitis related arthritis (p 0.02). Investigating the influence of treatments on Sars-Cov-2 antibodies production, no statistically significant difference was detected between the different treatment arms. No disease flare was noted based on evaluation of the JADAS-10 at baseline (before first vaccination) as well as in follow-up visits. As for side effects, local pain and need of NSAIDs were more frequent in healthy control than patients, the other symptoms were comparable in both groups.

**Conclusion:** In this study we did not find any difference in safety and immunogenicity of Sars-Cov-2 vaccines between JIA patients and healthy controls. Although our cohort is small it may be concluded that the vaccine has an adequate safety and tolerability profile. Further research is needed to investigate if the differences we observed effect the long-term protection offered by vaccine

**Patient Consent:** Yes, I received consent

**Disclosure of Interest**: None declared

**Table 1 (abstract P467).** See text for description

	Case (36)	Controls (33)	P value
Male (n)	9	8	0.95
Age (yrs)	14.5 (± 4.1)	14.8 (± 3.7)	0.32
Antibodies (ml)	1417 (± 738)	1311 (± 1199)	0.65
Local pain/ swelling	21	33	0.006
NSAIDs/paracetamol	7	14	0.04
Fever >38°	6	7	0.63
Arthralgia/Myalgia	4	8	0.16
Asthenia	7	14	0.04
Gastrointestinal symptoms	5	1	0.14

### P468. Clinical and paraclinical characteristics of pediatric patients with multisystemic inflammatory syndrome associated to COVID-19 and Kawasaki disease in a tertiary public healthcare center in Mexico Northeast

#### K. González-Moctezuma^1^, A. V. Villarreal-Teviño^2^, M. M. Rangel-Fuentes^2^, J. E. Mares-Gil^2^, O. Tamez-Rivera^1,2^, B. D. J. Fortuna-Reyna^2^, A. Rojas-Martínez^3^, G. Vargas-Duarte^1^

##### ^1^Pediatrics, Tecnológico de Monterrey, Escuela de Medicina y Ciencias de la Salud, ^2^Pediatrics, Hospital Regional Materno Infantil, Secretaria de Salud Nuevo León, ^3^Escuela de Medicina y Ciencias de la Salud, Tecnológico de Monterrey, Monterrey, Mexico

###### **Correspondence:** K. González-Moctezuma

**Introduction:** The precise risk factors which predisposed children to present Multisystemic Inflammatory Syndrome associated to COVID-19 (MIS-C) are certainly unknown; likewise its wide spectrum of clinical presentations and complications.

**Objectives:** To analyze and describe the different phenotypes of clinical, paraclinical and sociodemographic presentation in pediatric patients with diagnosis of MIS-C and Kawasaki disease (KD) in our center.

**Methods:** This was a cross-sectional descriptive, observational and retrospective study. Criteria inclusion were patients who met World Health Organization definition of MIS-C and KD who were diagnosed and hospitalized between 0-15 years old from June 2020 to November 2021. Patients with incomplete medical records or incomplete diagnosis criteria were excluded. Information was collected by clinical record review and a data base was created using clinical and paraclinical variables which were then separated in groups according to their phenotype of presentation: Classic MIS-C: Main gastrointestinal involvement with abdominal pain, vomit or diarrhea; Kawasaki like: Main mucocutaneous involvement with dermatosis and conjunctival injection; Lethal MIS-C:Hemodynamic compromise with refractory shock, vasoactive agents and pediatric intensive care unit admission; Kawasaki disease (KD): Diagnosis criteria for KD and absent history of COVID-19 disease or contact (negative PCR-SARS-CoV-2, negative antigenic and serologic tests).

**Results:** n=63 patients with MIS-C (classic=20, KW-like=25, lethal=18) and n=14 patients with KD. Male gender predisposition was observed in lethal group with 72%(n=13). The median age at Kawasaki-like phenotype were children under 5 years old (IQR:2-7), in classic phenotype was 7 years (IQR:5-11) and lethal phenotype was 10 years (IQR:7-14). 61 patients were previously healthy children. Overweight or obesity was present in 15% (n=3) of MIS-C, 16% (n=4) of KW-like and 33% (n=6) of lethal phenotype. 6 patients from the lethal phenotype died, having all of them seizures previous to their death and representing a global mortality rate of 10%. The median number of inflammatory markers was higher in lethal phenotype, while median number of platelet count was the lowest in this group, expected thrombocytosis was observed in KD. Biological results, median and IQR values are described on the following table (table 1).

**Conclusion:** Malnutrition, male gender and adolescent group of age were related to severity. The higher number of inflammatory markers at their admission to the hospital was directly proportional to severe form of disease; unlike platelet count which was inversely proportional presenting severe thrombocytopenia and bleeding in the lethal phenotype group. Seizures were highly related to mortality. It is fundamental to stay alert and create affordable strategies for early prevention, diagnosis, treatment and management of complications to support our children while we continue fighting against COVID-19 and this life threatening condition.

**Patient Consent:** Not applicable (there are no patient data)

**Disclosure of Interest**: None declared

**Table 1 (abstract P468).** Laboratory Tests Results

Variables	MIS-C (n=20)	KW-like (n=25)	KD (n=14)	MIS-C lethal (n=18)
Biological results: median, (IQR)				
**Platelet count, x10**^**9**^**/L**	169.5 (81.45-280)	151 (78.5-222.5)	373 (173.8-550)	110.4 (32.5-159.3)
**C-reactive protein mg/dl**	14 (2.5-22.8)	10 (3-20)	8 (2-16.8)	20 (14-23.7)
**D-dimer ng/ml**	4,692 (1,789.3-6,156)	4,235 (2,494.5-6,099)	1,647 (1,266-2,119)	5,137 (3,766-16,271)
**Ferritin ng/ml**	581 (296.5-1,034.8)	362 (245.8-1147.5)	199 (99.8-417.5)	1,759 (489.2-5,896.3)

### P469. Multysystem infalmmatory syndrome– not every crown is COVID-19 related

#### V. Gotloib^1,2^, A. ziv^1,2^, R. haviv^1,2^, Y. uziel^1,2^

##### ^1^Pediatric rheumatology, Meir hospital, kfar saba, ^2^Sackler School of Medicine , Tel Aviv, Israel

###### **Correspondence:** V. Gotloib

**Introduction:** Following the Coronavirus Disease 19 (COVID-19) pandemic, a new syndrome was described in April 2020, of a hyperinflammatory condition termed Multisystem Inflammatory Syndrome in Children (MIS-C). This life-threating, multi-organ failure can present as a severe disease that requires urgent diagnosis and therapy. As in many rheumatologic diseases, no single parameter or analysis can be used to diagnose this syndrome; rather, it requires a combination of clinical and laboratory criteria along with the elimination of other etiologies, mainly infectious.

**Objectives:** To introduce four cases that at presentation were diagnosed as MIS-C, but after broader analysis were found to be different illnesses.

**Methods:** we collected data regarding demographics, diagnosis, evaluation, treatment and follow up from the patients’ electronic medical records.

**Results: Case 1**: A 16-year-old boy presented with high fever, headache, new onset of psychosis, increased inflammatory markers and cytopenia 3 weeks after exposure to a patient infected with COVID-19, Empiric treatment with doxycycline was initiated due to suspicion of an atypical infectious agent. After severe clinical deterioration occurred with severe shock, MIS-C was suspected and he was treated accordingly with steroids and supportive therapy. The final diagnosis was Borreliosis with Jarisch-Herxheimers reaction.

**Case 2**: A 7-year-old boy, presented with fever, gastrointestinal symptoms, valvulitis diagnosed by echocardiography, serositis, cytopenia, elevated inflammatory markers, acute renal failure, with hematuria and proteinuria, 2 weeks after COVID-19 infection. Improvement under steroid therapy was noted. The final diagnosis was systemic lupus erythematosus.

**Case 3**: A 14-year-old boy presented with high fever, chills, headache, rash, tachycardia, elevated inflammatory markers and elevated coagulation marker, 1 month after recovering from COVID-19. Corticosteroid and antibiotic therapy led to improvement. The final diagnosis was rickettsiosis.

**Case 4**: A 4-year-old girl, presented with high fever, strawberry tongue, skin rash, abdominal and chest pain, pleural effusion, serositis, hemodynamic shock and elevated inflammatory markers, 3 weeks after recovering from COVID-19. The final diagnosis was gas empyema and streptococcal toxic shock syndrome.

**Conclusion:** In many cases, anamnesis of a recent COVID-19 infection brings us to think of a post-COVID-19 syndrome, but as these cases illustrate, it is very important to maintain a broad differential diagnosis with emphasis on so-called “older” diseases, that can “mimic” MIS-C.

**Patient Consent:** Not applicable (there are no patient data)

**Disclosure of Interest**: None declared

### P470. Monitoring of SARS-COV-2 antibody levels in children and late adolescents with inflammatory rheumatic diseases

#### F. Haslak^1^, D. Ozbey^2^, M. Yildiz^1^, A. Gunalp^1^, A. Adrovic^1^, S. Sahin^1^, O. Koker^1^, A. Aliyeva^1^, V. Guliyeva^1^, G. Yalcin^1^, G. Inanli^1^, B. S. Kocazeybek^2^, O. Kasapcopur^1^, K. Barut^1^

##### ^1^Pediatric Rheumatology, ^2^Department of Microbiology, Istanbul University-Cerrahpasa, Cerrahpasa Medical Faculty, Istanbul, Turkey

###### **Correspondence:** M. Yildiz

**Introduction:** Although current studies mostly indicate that the presence of inflammatory rheumatic disease (IRD) in childhood is not a significant risk factor for severe coronavirus disease-2019 (COVID-19), data on monitoring the seropositivity status of this patient group are limited. Chronic inflammation and immunosuppressive medications may affect the serological response in children with IRD, and considering the vaccination needs of these patients, it is seen that there is a need for studies that monitor their serological status.

**Objectives:** We aimed to monitor the Severe Acute Coronavirus-2 (SARS-CoV-2) antibody levels of patients with IRD and healthy children who were previously found seropositive.

**Methods:** In addition to the participants who were found to be seropositive in one of our previous studies, patients under 21 found seropositive between March 2020 and October 2020 were included in the study. Antibody levels of all the subjects were measured again at the third and sixth months by ELISA method. In this process, their symptoms were also questioned in terms of COVID-19.

**Results:** The study included 35 participants (female/male: 1.69) (Healthy control group: 10, patient group not receiving biological therapy: 19, patient group receiving biological therapy: 6). Their mean age was 14.27 ± 5.49 years. Of the participants, 13 (37.1%) had a history of symptomatic infection, and 4 (11.4%) had a history of hospitalization. At the end of the six-month observation, a significant decrease was found in the immunoglobulin (Ig) G levels of the participants (p=0.002). While no significant decrease was observed in the IgG levels of the participants from the beginning to the 3rd month (p=0.085), a significant decrease was observed from the 3rd to the 6th month (p<0.001). Age, gender, presence of IRD and use of biological agents did not affect this decrease.

**Conclusion:** It has been shown that antibodies acquired by infection in both healthy children and children with IRD were at an acceptable level in the first 3 months but decreased rapidly in the second trimester. This data can be used to schedule vaccination programs. Although it has been shown that IRD and biologic drugs do not affect the decrease in antibody levels, it indicates that no additional precautions are required in terms of vaccination in this patient group, but due to the limited number of patients, the data of our study should be confirmed with studies involving a larger number of patients.

**Patient Consent:** Yes, I received consent

**Disclosure of Interest**: None declared

### P471. Safety and humoral response following the second and third doses of the BNT162B2 MRNA COVID-19 vaccine in adolescents with juvenile-onset autoimmune inflammatory rheumatic diseases

#### M. Heshin-Bekenstein^1,2^, A. Ziv^3^, N. Toplak^4^, S. Lazauskas^5^, D. Kadishevich^6^, E. Ben Nun^6^, A. Miller^7^, Y. Aviel Butbul^7^, E. Saiag^8^, G. Shefer^9^, S. Pel^10^, O. Elkayam^1,10^, Y. Uziel^1,3^

##### ^1^Sackler School of Medicine, Tel Aviv University, ^2^Pediatric Rheumatology, TEL AVIV MEDICAL CENTER, Tel Aviv, ^3^Pediatric Rheumatology Unit, Meir Medical Center, Kfar Saba, Israel, ^4^Department of Allergology, Rheumatology and Clinical Immunology, University Children’s Hospital, University Medical Center Ljubljana, Ljubljana, Slovenia, ^5^Tel Aviv Medical Center, Tel Aviv, ^6^Pediatrics, Meir Medical Center, Kfar Saba, ^7^Pediatric Rheumatology Unit, Rambam Medical Center, Haifa, ^8^Hospital Management, Information and Operation Branc, ^9^Department of Endocrinology Metabolism and Hypertension, ^10^Rheumatology Department, Tel Aviv Medical Center, Tel Aviv, Israel

###### **Correspondence:** M. Heshin-Bekenstein

**Introduction:** Long term data on the safety and dynamics of the immune response to the BNT162b2 mRNA COVID-19 vaccine in adolescents with juvenile-onset autoimmune inflammatory rheumatic diseases (AIIRD) are limited.

**Objectives:** To explore the safety profile and immune response over time of the second and third doses of the BNT162b2 mRNA vaccine in adolescents with juvenile-onset AIIRD and in comparison to healthy controls.

**Methods:** This international prospective longitudinal multicenter study included adolescents with AIIRD (N=121) and healthy controls (N=49), with mean ages of 15.4 and 13.4 years, respectively. 60% of the AIIRD patients are on chronic immunomodulatory therapy. Participants were vaccinated with either a 2-dose mRNA vaccine: AIIRD n=93 (77.5%); controls n=19 (39%), or a 3-dose vaccine AIIRD n=27 (22.5%); controls n=30 (61%) and were followed to monitor for vaccine side effects, disease activity and medication change from baseline to time-points post-vaccinations. In addition, antibody titers were measured serially for part of the adolescents from both cohorts, with serum anti-spike protein S1/S2 IgG titers measured at three-time points: 2-9 weeks following the second vaccine, six-month time point and prior to the third vaccine, and 2-9 weeks following the third vaccine dose. Seropositivity was defined as an anti-S1/S2 IgG titer ≥ 15 BAU/ml.

**Results:** The safety profile was good, with the vast majority of patients reporting mild or no side effects. The rheumatic disease has stayed stable at 97% and 100% after the second and third vaccines, with no worsening of the disease activity. The two-dose-vaccine induced similar, high immunogenicity rates among adolescent patients and controls, with seropositivity rates of 93% vs. 100%, respectively (p=0.55). S1/S2 IgG titers were significantly higher in the controls compared with AIIRD patients (377±78 vs. 246±137 BAU/ml). As expected, at the 6-month measurement, before the third vaccine dose, the AIIRD cohort seropositivity rates were lower compared with the healthy controls, 86.7% vs. 100%, respectively (p=0.3), with similar S1/S2 titers (221±159 vs. 220±93 BAU/ml, p=0.98). Following the third vaccine, the seropositivity rate increased to 100% in both AIIRD and control groups, with similar S1/S2 IgG titers among AIIRD patients compared with controls, 398±6 vs. 400±0 BAU/ml (p=0.35).

**Conclusion:** This study reports the long-term safety and immune response induced by the two- and three-dose BNTb262 mRNA COVID-19 vaccine in adolescents with AIIRD compared with healthy controls. The safety profile in both cohorts was good following all three vaccine doses, with an adequate immunogenic response, though adolescents with AIIRD had lower seropositivity rates prior to the third vaccine as compared to the healthy adolescents (86.7% vs 100%). The third vaccine dose restored the immune response in both AIIRD and healthy adolescents.

**Patient Consent:** Yes, I received consent

**Disclosure of Interest**: None declared

### P472. The risk factors for a severe disease course in multisystem inflammatory syndrome in children - a multicenter retrospective study

#### K. Kaidar^1^, Y. Dizitzer^2^, P. J. Hashkes^3^, L. Wagner-Weiner^4^, M. Tesher^4^, Y. Butbul Aviel^5^, I. Kanteman ^5^, Y. Berkun^6^, E. M. Eisenstein^7^, M. H. Saied^8^, O. Goldzweig^9^, M. Heshin-Bekenstein^10^, E. Ling^11^, M. Feldon^12^, Y. Levinsky^13^, R. Tal^14^, L. Harel^15^, G. Amarilyo^16^

##### ^1^pediatric rheumatology, ^2^ward C, SCHNEIDER MEDICAL HOSPITAL, Petach TIkva, ^3^pediatric rheumatology, Shaare Zedek Medical Centre, Jerusalem, Israel, ^4^pediatric rheumatology, University of Chicago Medical Centre, Chicago , United States, ^5^pediatric rheumatology, Meyer Children’s Hospital of Haifa, Haifa, ^6^pediatrics, Hadassah-Hebrew University Medical Center, Mount Scopus, , ^7^pediatrics, Hadassah-Hebrew University Medical Center, Mount Scopus , Jerusalem, ^8^Pediatric Rheumatology, Carmel Medical Centre, Haifa, ^9^pediatric Rheumatology, Kaplan Medical Center, Rehovot, ^10^pediatric rheumatology, Dana Children’s Hospital of Tel Aviv Medical Center , Tel Aviv, ^11^Pediatrics Department B, Saban Pediatric Medical Center, Beer Sheva, ^12^Pediatric rheumatology, Shamir medical center, Rehovot, ^13^pediatric Rheumatology, Schneider Children’s Medical Center, lPetach Tikva, ^14^Pediatric Rheumatology, Schneider Children’s Medical Center, Petach Tikvah, ^15^pediatric Rheumatology, Schneider Children’s Medical Center , Petach Tikva, ^16^pediatric rheumatology, Schneider Children’s Medical Centre, Petach Tikvah, Israel

###### **Correspondence:** K. Kaidar

**Introduction:** Multisystem Inflammatory Syndrome in Children (MIS- C) associated with COVID-19, presents as a cytokine storm with features of Kawasaki disease. It is characterized by protracted fevers, gastrointestinal, cardiac and neurological symptoms. Laboratory tests are characterized by neutrophilia, lymphopenia, hypoalbuminemia and high inflammatory and myocardial damage markers. Many cases present with shock and require intensive care admission.

**Objectives:** To identify predictors of a severe clinical course of multisystem inflammatory syndrome in children (MIS-C), as defined by the need for ionotropic support.

**Methods:** This retrospective study included patients diagnosed with MIS-C in nine Israeli and one US medical centre. Univariate and multivariate regression models assessed odds ratio (OR) of demographic, clinical, laboratory and imaging variables during admission and hospitalization for severe disease.

**Results:** Of 100 patients, 61 (61%) were male; mean age 9.65±4.48 years. Sixty-five patients were hypotensive, 44 required ionotropic support. Eleven fulfilled Kawasaki disease diagnostic criteria; 87 had gastrointestinal symptoms on admission. Echocardiographic evaluation showed 10 patients with acute coronary ectasia or aneurism, and 37 with left ventricular dysfunction. In a univariate model, left ventricular dysfunction was associated with severe disease (OR 4.178 [95%CI 1.760-9.917], while conjunctivitis (OR 0.403 [95%CI 0.173-0.938]) and mucosal changes (OR 0.333 [95%CI 0.119-0.931]) at admission were protective. Laboratory markers for a severe disease course were low values of haemoglobin, platelets, albumin and potassium; and high leukocytes, neutrophils, troponin and brain natriuretic peptide. In multivariate analysis, central nervous system involvement and fever >39.5°C were associated with severe disease. Mucosal involvement showed 6.2-fold lower risk for severe disease. Low haemoglobin and platelet count, and elevated C-reactive protein and troponin levels were identified as risk factors.

**Conclusion:** Key clinical and laboratory parameters of MIS-C were identified as risk factors for severe disease; and may prompt earlier, more aggressive treatment decisions. Patients presenting with a Kawasaki-like phenotype were less likely to require ionotropic support.

**Patient Consent:** Yes, I received consent

**Disclosure of Interest**: None declared

### P473. A case of Juvenile Idiopathic Arthritis (JIA) deterioration after BNT162B2 booster vaccination (3rd vaccination)

#### K. Kim

##### Pediatrics, Hanyang University Myongji Hospital, Goyang-si, Korea, Republic Of

###### **Correspondence:** K. Kim

**Introduction:** Vaccine is an effective public health measurement to control the global COVID-19 pandemic. Patients with rheumatoid arthritis (RA) are twofold more vulnerable to infections that result in hospitalization and impaired quality of life.

With consideration to the benefits of mRNA vaccination outweighing the risks, EULAR recommends that patients with RA should receive COVID-19 vaccines without needing major adjustment to their ongoing treatment regimens.

**Objectives:** However, concerns about the safety of the vaccines are a major hurdle to widespread vaccination to the adolescent and children.

Here, we experienced a JIA flare-up more than 5 months, the day after receiving the booster shot of BNT162b2 mRNA vaccine.

**Methods:** In 2012, at the age of 10 years of boy, he was diagnosed with oligo JIA and received DMARD, but his symptoms gradually worsened, and in 2014, it changed to an extended type of oligo JIA. The symptoms improved after add on the Etenercept SC injection. All medications were discontinued in 2017 because no more symptoms occurred. However, it relapsed in 2019 and resumed DMARD plus Etenercept. After that, he has reached remission until he was COVID-19 mRNA vaccinated.

After the first and second COVID-19 mRNA vaccinations, there was no adverse reaction, so he was vaccinated for the booster shot (3^rd^ vaccination) in Dec 2021, but the Right knee swelled the next day. He is on medication state, MTX 3tab weekly, Folic Acid 1tab daily and Enbrel 1.0 twice/week since May 2019.

He was given the third vaccination on Dec. 16, 2021. The next day after the vaccination, his right knee was swollen and felt pain on Dec. 17, 2021. The swollen joint was not improved with NSAID. So, prednisolone 15mg (3tab) was added on Jan. 3, 2022. Nevertheless, the swollen right joint worsened. The US on January 5^th^ showed large amount of joint effusion on right knee and US guided aspiration and US guided mixed fluid (triamcinolone acetonide + ropivacaine + saline) injection was done. It worked for the first seven days, but after that, the right joint was swollen again and complaining of pain. These symptoms continued for more than 5 months after mRNA booster vaccination.

**Results:**

**Table:** 

	9/18/2021	12/27/2021	1/3/2021	1/26/2022	4/23/2022	5/30/2022
CBC	7000	11600	10400	10300	9700	9000
Seg %	32.6	75.0	65.7	76.2	43.1	67.7
Lymph %	57.8	15.2	20.0	15.9	47.2	23.3
Hs-CRP	0.03 mg/dL	6.51 mg/dL	10.7 mg/dL	2.32 mg/dL	1.36 mg/dL	1.24 mg/dL
ESR	2 mm/hr	32 mm/hr	55 mm/hr	37 mm/hr	15 mm/hr	14 mm/hr

**Conclusion:** We experienced that after full vaccination (3^rd^ booster vaccination) with BNT162b2 patient with JIA, showed an increased risk of possible arthritis flare-up.

So, To understand the association between arthritis flare and vaccination is important to overcome vaccine hesitancy.

**Patient Consent:** Yes, I received consent

**Disclosure of Interest**: None declared

### P474. MIS-C/PIMS-TS: clinical manifestations and therapy

#### B. Koren, V. Berce

##### Pediatric clinic, University Medical Centre , Maribor, Slovenia

###### **Correspondence:** B. Koren

**Introduction:** In children, coronavirus disease 2019 (COVID-19) is usually mild. However, in rare cases, children can be severely affected and develop a significant systemic inflammatory response, which has been termed multisystem inflammatory syndrome in children (MIS-C) or pediatric inflammatory multisystem syndrome temporally associated with severe acute respiratory syndrome (SARS) associated coronavirus 2 (SARS-CoV-2) (PIMS-TS).

**Objectives:** To evaluate clinical manifestations and therapy in pediatric patients diagnosed with MIS-C.

**Methods:** A single small center study was performed. Included were patients with MIS-C treated in inpatient service of our pediatric clinic from November 2020 till March 2022. WHO/CDC diagnostic criteria for MIS-C were used. Data was collected retrospectively from patients’ medical records.

**Results:** A total of 12 patients were included. The mean age of all patients was 8.9 years (range 1-15 years). Female and male patients were equally affected (ratio 1:1).

3 patients (25%) had positive PCR swab for COVID-19 and 10 patients (83.3%) had positive serology to COVID-19.

The clinical characteristics of patients with MIS-C are presented in Table 1.

In addition to fever and myocardial dysfunction, which were present in all patients, gastrointestinal involvement was the most common clinical manifestation.

3 boys aged 4, 5 and 15 years also fulfilled diagnostic criteria for typical and 1 girl aged 1 year fulfilled diagnostic criteria for atypical Kawasaki disease.

All of our patients were treated with intravenous immune globulin (IVIG) and aspirin. 9 patients (75%) were put on glucocorticosteroids. Moreover, among them 3 (25%) needed pulse doses. Anticoagulation with a low- molecular- weight heparin (LMWH) was used in 11 patients (92%). As MIS-C can present with signs and symptoms that mimic those of septic shock and toxic shock syndrome, 3 patients (25%) were put on antibiotic therapy. 1 girl who presented with COVID-19 pneumonia received antiviral therapy with remdesivir. We didn’t use biologics in any of our patients.

All 4 patients (33.3%) presenting with shock required pediatric intensive care treatment.

**Conclusion:** The results of our study are consistent with data from the literature. All of our patients with MIS-C achieved full recovery.

Because the disease can present as a severe shock- like illness early diagnosis and prompt treatment are of significant importance. Long- term follow- up data is limited, but the prognosis looks favourable as most children have a full clinical recovery.

**References**:

1. Riphagen S, Gomez X, Gonzalez- Martinez C, et al. Hyperinflammatory shock in children during COVID-19 pandemic. Lancet 2020; 395:1607.

2. Son MBF, Friedman K. COVID-19: Multisystem inflammatory syndrome in children (MIS-C) clinical features, evaluation and diagnosis. Available at: https://www.uotodate.com/contents/covid-19-multisystem-inflammatory-syndrome-in-children-mis-c-clinical-features-evaluation-amd-diagnosis (Accessed on April 8, 2022).

3. Licciardi F, Pruccoli G, Demina M, et al. SARS-CoV-2- Induced Kawasaki- like hyperinflammatory syndrome: A novel COVID phenotype in children. Pediatrics 2020; 146.

**Patient Consent:** Not applicable (there are no patient data)

**Disclosure of Interest**: None declared

**Table 1 (abstract P474).** See text for description

Clinical manifestation	Number of patients (n,%)
Fever	12 (100%)
Gastrointestinal involvement (abdominal pain, vomiting amd/or diarrhea)	11 (92%)
Rash	5 (41.7%)
Conjunctivitis	6 (50%)
Sore throat	2 (16.7%)
Neurocognitive symptoms (headache, confusion, vertigo)	2 (16.7%)
Shock	4 (33.3%)
Myocardial dysfunction (by echocardiogram and/ or elevated troponin or brain natriuretic peptide (BNP))	12 (100%)
Serositis	5 (41.7%)

### P475. Capillary leak syndrome as a new, common phenomenon in children with MIS-C - a retrospective, population-based cohort from Southern Sweden

#### P. Król^1,2^, M. Mossberg^1^, B. Månsson^3^, R. Kahn^1,2^

##### ^1^Department of Pediatrics, ^2^Wallenberg Centre for Molecular Medicine, ^3^Department of Rheumatology, Lund University, Lund, Sweden

###### **Correspondence:** P. Król

**Introduction:** Multisystem Inflammatory Syndrome in Children (MIS-C) is a rare entity caused by a previous, often inapparent or mild, COVID-19 infection. The pathogenesis of MIS-C is still discussed, but a few theories has been suggested (1). One could be endothelial dysfunction, that might lead to systemic capillary leak syndrome (CLS), a feature of MIS-C.

**Objectives:** The aim of this study was to compare clinical characteristics and laboratory parameters between MIS-C patients with and without CLS symptoms.

**Methods:** This retrospective, population-based cohort study from southern Sweden includes all individuals between 0-19 years diagnosed with MIS-C from the beginning of pandemic in 2020 to end of July 2021. Inclusion criteria were: 1) diagnosis according to the WHO case definition for MIS-C diagnosis (2), 2) patient and families agreed to be registered in the National Pediatric Rheumatology Register by signing the informed consent, 3) Living in the study area at time of diagnosis. Exclusion criteria were that patient/family moved to another region or country. Clinical variables included MIS-C symptoms, admission to standard ward or ICU/PICU, length of stay and treatment (IvIg or IvIg + corticosteroids or IvIg + corticosteroids + anakinra). Laboratory parameters (CRP, leukocyte- and lymphocyte count, platelets, proBNP and albumin) were measured daily. CLS was defined as having at least two of four symptoms; 1) oedema; 2) treatment-required hypotension; 3) hemoconcentration and 4) hypoalbuminemia.

**Results:** In total, 31 children with MIS-C were identified (39% females), and 24 out of these met the criteria for CLS. The median age at diagnosis was 10,6 years. Children were hospitalized for a median of 10 days (range 4-48). Six children were treated at ICU/PICU, all with CLS. The clinical characteristics are shown in Table 1. Children in CLS group had higher CRP and proBNP and lower platelets and albumin levels the first days of hospitalization as compared to the non-CLS group. Leukocyte and lymphocyte count was similar in both groups. 58% of patients from CLS group were treated with IvIg + steroids and remaining 42 % with IvIg + steroids + anakinra. Six of seven patients from non-CLS group were treated with IvIg + steroids and one patient with only IvIg. No patient from the non-CLS group were treated with IvIg + steroid + anakinra.

**Conclusion:** CLS is a severe and common feature of MIS-C. Seventy seven percent of our MIS-C patients developed this complication during the first days of hospitalization. In our cohort patients with CLS compared to non CLS patients had more severe disease course, higher inflammatory parameters, lower platelet count and need of more aggressive anti-inflammatory treatment (42% patients with triple-combination, compared to none). It is important to identify and treat CLS early and we suggest that MIS-C children with low platelet count and severe inflammatory phenotype might be at higher risk to develop CLS.

References.

1. McMurray JC, May JW, Cunningham MW, Jones OY. Multisystem Inflammatory Syndrome in Children (MIS-C), a Post-viral Myocarditis and Systemic Vasculitis-A Critical Review of Its Pathogenesis and Treatment. Front Pediatr. 2020 Dec; 8: 626182

2. World Health Organization. Multisystem inflammatory syndrome in children and adolescents temporally related to COVID-29. World Health Organization; 2020.

**Patient Consent:** Yes, I received consent

**Disclosure of Interest**: None declared

**Table 1 (abstract P475).** Clinical symptoms of patients with MIS-C

Symptom; n (%)	All patients; n=31	CLS; n=24	Non-CLS; n=7
Rash	19 (61)	15 (62)	4 (57)
Conjunctivitis	19 (61)	17 (71)	2 (29)
Abdominal pain	23 (74)	19 (79)	4 (57)
Vomiting	21 (68)	17 (71)	4 (57)
Diarrhea	17 (55)	13 (54)	4 (57)
**Capillary leak symptoms, n (%)**	**24 (77)**		
Hypotension	16 (52)	16(67)	0
Oedema	20 (65)	20 (83)	0

### P476. Epidemiology of Multisystem Inflammatory Syndrome in Children (MIS-C) in Southern Italy

#### F. La Torre^1^, M. P. Elicio^2^, V. A. Monno^2^, M. Chironna^3^, A. Campanozzi^4^, F. Moramarco^5^, A. Civino^6^, V. Cecinati^7^, U. Vairo^8^, L. Milella^9^, D. Loconsole^3^, V. Mastrorilli^2^, F. Cardinale^2^

##### ^1^Department of Pediatrics, Giovanni XXIII Pediatric Hospital, University of Bari, ^2^Department of Pediatrics, Giovanni XXIII Pediatric Hospital, University of Bari, ^3^Department of Interdisciplinary Medicine, Hygiene Section, University of Bari, Bari, ^4^Department of Medical and Surgical Sciences, Pediatric Section, University of Foggia, Foggia, ^5^Department of Pediatrics, Antonio Perrino Hospital, Brindisi, ^6^Division of Pediatric Rheumatology and Immunology, Vito Fazzi Hospital, Lecce, ^7^Department of Pediatrics , SS Annunziata Hospital , Taranto, ^8^Division of Pediatric Cardiology, ^9^Division of Intensive Care, Giovanni XXIII Pediatric Hospital, Bari, Italy

###### **Correspondence:** F. La Torre

**Introduction:** Multisystem inflammatory syndrome in children (MIS-C) is a newly identified clinical entity not yet very well known in terms of epidemiology, pathogenesis and long-term outcome. So far very few studies worldwide have investigated the incidence of MIS-C. Most studies published come from US, reflecting mainly north-American racial and genetic backgrounds. Data about the incidence of MIS-C in Europe are lacking.

**Objectives:** The aim of the study was to gain insight into the epidemiology of MIS-C in Apulia region in southern Italy. Our primary goal was to estimate the incidence of newly identified cases of MIS-C in children 0-18 years of age, during a period of six months, encompassing the second pandemic wave. We also evaluated the percentage of children with previous SARS-CoV2 infection who developed MIS-C in the following weeks, the incidence rate of MIS-C in terms of cases/month/year, and the cumulative incidence per 100,000 residents.

**Methods:** An observatory network between all pediatric departments in Apulia was created since October 2020. Data from all cases of MIS-C, between 0 and 18 years, hospitalized in all regional pediatric departments, in a period of six months, spanning from November 1, 2020 to April 30, 2021, were collected. The data collected were analyzed using STATA MP17 software. Continuous variables were expressed as mean ± standard deviation and range, categorical variables as proportions. The data source used was the “Infections Regional Informative System (IRIS) - Apulia. The number of residents aged 0-18 years in Apulia relating to the 2020-2021 was obtained from the ISTAT (Italian National Statistics Institute) registry.

**Results:** During the time frame considered, 22 new cases of MIS-C were admitted to five community pediatric departments in Apulia (Bari, Foggia, Brindisi, Lecce and Taranto). The cumulative incidence of MIS-C was 3.27 per 100,000 residents between 0 and 18 years of age and the incidence rate of MIS-C among pediatric subjects with previous SARS-CoV-2 infection was 0.07%. The age group most affected was between 6 and 10 years with a cumulative incidence of 5.67 per 100,000 residents between 0 and 18 years.

The Table show the cumulative incidence of multisystem inflammatory syndrome in children (MIS-C) by the age groups:

Most newly diagnosed cases of MIS-C followed the peak of SARS-CoV2 infections after 2-6 weeks and in particular a greater number of cases was registered in December 2020 and between late February and early April 2021 with a higher incidence rate per month, expressed in cases/month/year, on March 2021.

**Conclusion:** Our paper represents the first epidemiological study of MIS-C in Europe. The strength of our study was the creation of a network among all the pediatric departments in a well defined geographical region which allowed us to catch all cases of MIS-C that occurred in the examined period. This makes our epidemiological data more accurate as possible and it confirms in Europe the US data

**Patient Consent:** Yes, I received consent

**Disclosure of Interest**: None declared

**Table 1 (abstract P476).** See text for description

Age group, y	Number of cases	Cumulative incidence (x 100,000 residents 0-18 years)
≤ 5	9	5,02
6-10	10	5,67
11-15	3	1,56
16-18	0	0,00
0-18	22	3,27

### P477. Cardiac magnetic resonance to evaluate cardiovascular involvement in children with covid-19 temporally related Multisystem Inflammatory Syndrome (MIS-C): can we arrive to the heart of the problem?

#### M. C. Maggio^1^, A. Lembo^2^, F. Finazzo^3^, A. Alaimo^4^, M. C. Failla^5^, S. Giordano^5^, G. Corsello^2^

##### ^1^PROMISE “G. D’Alessandro”, ^2^University Department PROMISE “G. D’Alessandro”, University of Palermo, ^3^U.O.C. of Paediatric Radiology, Children Hospital “G. Di Cristina”, ^4^U.O.C. of Paediatric Cardiologic Division, Children Hospital “G. Di Cristina”, ^5^U.O.C. of Paediatric Infectious Diseases, Children Hospital “G. Di Cristina”, ARNAS, Palermo, Italy

###### **Correspondence:** M. C. Maggio

**Introduction:** MIS-C is a hyperinflammatory syndrome following SARS-CoV-2 exposure. A transitory myocardial impairment as myocarditis, occurs with full recovery in most of them. Coronary lesions, valvular insufficiency and pericardial effusion are variable associated. The pathogenesis of the heart involvement is still object of study, linked to cytokines storm.

**Objectives:** To evaluate the usefulness of cardiac magnetic resonance (CMR) in highlighting heart involvement in children affected by MIS-C.

**Methods:** We describe a case series of 18 children (age:1-14 years;11F;7M), admitted to a tertiary care Children Hospital with diagnostic criteria of MIS-C. All the patients were followed by ECG and echocardiogram. CMR was performed during the hospitalization or after the discharge, with a 1,5 Tesla scanner. The CMR study was not performed during the acute phase in some patients, clinically unstable and needing general sedation. The protocol included, before intravenous contrast media injection, retrospective ECG-Gated fiesta cine sequences (short axis, 4,3,2 chamber views), sequences for edema, nd hyperemia T2 -short tau inversion recovery (Stir). Myocardial edema was evaluated following the Lake Louise criteria. Because poor references for normal value in native T1 mapping and T2 relaxation time in children, myocardial edema was defined by increased signal intensity on T2-weighted imaging and myocardial damage by non-ischemic patterns late gadolinium enhancement. The study for evaluating myocyte necrosis and fibrosis considered late gadolinium-enhanced 2D inversion recovery sequences performed at 6 min following intravenous contrast medium administration.

**Results:** ECG evidenced arrythmia in 8 patients. All resolved arrythmia in the subacute phase. Transthoracic echocardiography demonstrated in all the patients signs of cardiac involvement: 2 showed coronaritis without aneurisms; 4 showed pericarditis; 12 showed valvular insufficiency: 7 Mitral insufficiency; 1 Tricuspid insufficiency; 2 Mitral and Tricuspid insufficiency; 2 Mitral and Aortic insufficiency. In 16/18 patients, T2-Stir sequences didn’t show myocardial edema and/or hyperemia and/or fibrosis. In 2 patients CMR showed late gadolinium enhancement in a non-ischemic pattern. 1 patient was studied in subacute phase, with evidence of ventricular myocardial oedema, persisting as delayed enhancement during the follow-up. 1 patient was studied 1 month after the hospitalization, showing myocardial fibrosis. 2 children showed pericardial effusion; during the follow-up, pericardial effusion resolved. 1 patient showed a persistent aortic insufficiency. In the other patients, cardiac lesions resolved during the follow-up.

**Conclusion:** CMR is an excellent noninvasive diagnostic tool for the diagnosis and follow-up of heart involvement. Furthermore, CMR can predict prognosis and recognize children at risk to develop arrhythmias and unfavorable events. Above all, CMR is a standardized imaging highlight some features of myocardial damages: edema, inflammation, pericardial effusion, contractile scar impairment and necrosis. Most of CMR exams didn’t detect myocardial edema, probably because the CMR was performed during the clinical recovery phase. In 2 patients CMR showed late gadolinium-enhanced in a non-ischemic pattern. CMR was an accurate and reliable method to follow children with cardiac involvement in MIS-C and guide the targeted therapeutic strategy.

**Disclosure of Interest**: None declared

### P478. The impact of COVID-19 pandemic on children with juvenile idiopathic arthritis under immunosuppressive and biological therapy: a single-center experience

#### M. Manojlovic^1^, J. Vojinovic^1,2^

##### ^1^Paediatric Clinic, Department of Rheumatology, University Clinical Center of Nis, ^2^University of Nis, Medical Faculty, Niš, Serbia

###### **Correspondence:** M. Manojlovic

**Introduction:** During COVID-19 pandemic it was observed that, despite the use of immunosuppressive and biological disease-modifying antirheumatic drugs (bDMARDs), children with paediatric rheumatic diseases are not susceptible to infections.

**Objectives:** The aim of the research was to further broaden current knowledge of whether COVID-19 affects the course of juvenile idiopathic arthritis (JIA) in children under immunosuppressive treatment. In addition, we analysed epidemiological risk and exposure to COVID-19, as well as patient interest in COVID-19 vaccination.

**Methods:** The survey was conducted by interviewing parents and patients. The questionnaire consisted of 4 domains: first, regarding COVID-19 infection or exposition; Second, assessment of underlying disease-related information (disease duration, length of the treatment, current medication, disease status at the last visit, comorbidity, and concomitant drugs) and COVID-19 influence on clinical course; And in last domain, possible interest in COVID-19 vaccination was evaluated.

**Results:** Patients who were under immunosuppressive therapy and followed up at the Paediatric Rheumatology Department were asked to provide one-on-one communication and informed about the research. Patients with incomplete medical data and those whose parents refused to participate were excluded from the study. In our cohort (n=68), the mean age was 11±4.82 years, and 69% (n=47) of the patients were female. The patients were categorised into two groups: bDMARDs (n=45) and nonbiological DMARDs (n=23). In bDMARDs group 60% were tested positive for COVID-19, and five of them (11,1%) had disease deterioration after COVID-19 infection, requiring therapy change. Two of them had their drugs changed, and one had drug dose increased. There was one case (4,3%) with exacerbation of underlying disease due to not responding to regular check-ups and self-initiated breaks in therapy, thus dose of the drug was increased. Similarly, in group of children treated with nonbiological DMARDs, 65% were diagnosed with COVID-19 and worsening of the underlying disease has been reported in one case (4,3%), after second reinfection, requiring drug change. There was no statistically significant difference between those two groups (p<0,05). None of the patients diagnosed with COVID-19, had any severe symptoms or required hospitalisation. In bDMARDs group, parents of three children were motivated for COVID-19 vaccination, while one child was vaccinated (2,2%). The rest of the parents (91,1%) had negative attitude regarding vaccination. In nonbiological DMARDs group one child (4,5%) was vaccinated, while remaining (95,6%) had negative attitude towards vaccination.

**Conclusion:** Children with JIA taking immune-modulatory drugs in COVID-19 era should be recommended to maintain the chronic therapy aiming optimal control of active disease. In our cochort most of patients under both biological and nonbiological DMARDs did not have disease exacerbation, however, some of them required therapy change.

**Disclosure of Interest**: None declared

### P479. Current approach to covid-19 and other vaccinations in children with previous Multisystem Inflammatory Syndrome (MIS-C): an international survey

#### F. Minoia^1^, F. Lucioni^1^, M. Heshin-Bekenstein^2^, S. Vastert^3^, C. Kessel^4^, Y. Uziel^5^, L. Lamot^6^, N. Ruperto^7^, M. Gattorno^7^, C. Bracaglia^8^, N. Toplak^9^ on behalf of the MAS/sJIA and Vaccination WPs

##### ^1^Fondazione IRCCS Ca’ Granda Ospedale Maggiore Policlinico, Milan, Italy, ^2^Dana Dwek Children’s Hospital, Tel Aviv Medical Center, Tel Aviv, Israel, ^3^University Medical Center, Utrecht, Netherlands, ^4^University Children’s Hospital Muenster, Muenster, Germany, ^5^Meir Medical Center; Sackler School of medicine, Tel Aviv University, Kfar Saba, Israel, ^6^University Hospital Center, Zagreb, Croatia, ^7^IRCCS Istituto Giannina Gaslini, Genoa, ^8^IRCCS Ospedale Pediatrico Bambino Gesù, Rome, Italy, ^9^University Children’s Hospital, University Medical Centre, MF, UL, Ljubljana, Slovenia

###### **Correspondence:** F. Minoia

**Introduction:** Following the Coronavirus Disease-19 (COVID-19) pandemic outbreaks, the hyperinflammatory condition termed Multisystem Inflammatory Syndrome in Children (MIS-C) has become a healthcare issue worldwide. Since December 2020 the mRNA vaccine against SARS-CoV-2 has become available with a good safety profile. However, evidence regarding safety and vaccination strategies in children with previous MIS-C is still lacking

**Objectives:** To investigate the current approach in different international centres to COVID-19 and other vaccinations among children with previous MIS-C

**Methods:** Physician all around the globe who take care of patients with MIS-C worldwide were invited to anonymously complete a 15-question web-based survey. The survey was open from October 6^th^ to December 31^st^ 2021. Centre, country and specialty of the participants were collected. Participants were asked to describe their current vaccination strategy for MIS-C patients and to provide the most important variables affecting their decision-making process

**Results:** A total of 290 replies from 237 centres in 61 countries were collected. Most respondents (86%) were pediatric rheumatologists. The anti-COVID-19 vaccine was available in 85% of the countries covered by the survey. Sixty-seven centres (28%) from 22 countries had already vaccinated MIS-C patients (< 5 patients: 52%; 5-10 patients: 29%; > 10 patients: 20%), without adverse reactions in most cases (89%). Six centres reported complications after the anti-COVID-19 vaccine: 2 unknown, 3 mild symptoms (fever, sore arm) and only 1 centre reported a MIS-C like reaction. MIS-C patients re-infected by SARS-CoV-2 were seen in 15% of centres and 2 (8%) reported a MIS-C flair. Most centres (84%) were in favour of vaccinating MIS-C patients against SARS-CoV-2, waiting 3-6 months (40%), 6-12 months (52%) or > 12 months (8%) after a MIS-C episode. The variables with the greatest impact on the decision not to vaccinate MIS-C patients were current lack of evidence (51%), patient/parent decision (40%), fear of MIS-C relapse (36%), history of severe MIS-C with myocarditis (35%) and excessive proximity to MIS-C episode (33%). The most relevant parameters in the vaccination strategy were time from MIS-C episode (78%), ongoing immunosuppressive treatment (35%), SARS-CoV-2 serologic status (32%) and MIS-C features (31%). Almost all centres favoured continuing regular vaccination with non-live (99%) and live (93%) vaccines, waiting 3-6 months and 6-12 months, respectively

**Conclusion:** When vaccinating MIS-C patients against SARS-CoV-2, the experience reported by the international pediatric rheumatology community to date, is overall reassuring. However, lack of evidence still affects the vaccination strategy of many centres worldwide. Large, prospective studies are needed to properly evaluate the safety of anti-COVID-19 vaccination among MIS-C patients

**Patient Consent:** Not applicable (there are no patient data)

**Disclosure of Interest**: F. Minoia Consultant with: SOBI, F. Lucioni: None declared, M. Heshin-Bekenstein: None declared, S. Vastert: None declared, C. Kessel Grant / Research Support with: Novartis, Consultant with: SOBI and Novartis, Y. Uziel: None declared, L. Lamot: None declared, N. Ruperto: None declared, M. Gattorno: None declared, C. Bracaglia Consultant with: Novartis and SOBI, N. Toplak: None declared

### P480. Analysis of the dynamics of changes in laboratory parameters in patients with PIMS-TS - one-year follow-up, single-center case-control study, preliminary results

#### V. Opoka-Winiarska^1^, E. Grywalska ^2^, I. Morawska^3^, K. Gosik^2^, O. Kądziołka^4^, J. Roliński^3^

##### ^1^Department of Paediatric Pulmonology and Rheumatology, ^2^Department of Experimental Immunology, ^3^Department of Clinical Immunology and Immunotherapy, Medical University of Lublin, ^4^Department of Pediatrics, Pulmonary Diseases and Rheumatology, University Children’s Hospital in Lublin, Lublin, Poland

###### **Correspondence:** I. Morawska

**Introduction:** The pediatric inflammatory syndrome temporarily associated with SARS-CoV-2 (PIMS-TS) is a severe disease entity with an incompletely established pathogenesis. Typically, the symptoms develop 4-6 weeks after exposure to the SARS-CoV-2 virus, but the clinical course is heterogeneous. The pathogenesis and the course of the disease is still incompletely established and long follow-up of patients after illness is still carefully followed.

Programmed cell death-1 protein (PD-1 or CD279) is a member of CD28 superfamily and expressed on CD4+ and CD8+ T cells, NK cells, B cells and activated monocytes. Binding to the ligands PD-L1 and PD-L2 on T cells, B cells, dendritic cells (DCs) and macrophages, PD-1 conducts its inhibitory function to regulate T cell activation, tolerance, and immune-mediated tissue damage.

The aim of the study was to evaluate the expression of selected suppressive molecules by assessing the PD-1 serum concentration in PIMS-TS patients in relation to laboratory parameters at the time of diagnosis and annual follow-up.

**Objectives:** Our study aimed to analyze expression of the PD-1 receptor on CD8+, CD4+ and CD19+ lymphocytes, in patients diagnosed with PIMS-TS, diagnosed at the Department of Lung Diseases and Pediatric Rheumatology at the Medical University of Lublin from January 2021 to April 2022. The study was approved by the bioethics committee no KE-0254/236/2020.

**Methods:** The study included 31 patients diagnosed with PIMS-TS and 36 healthy volunteers (HV), from whom, after obtaining consent, whole blood was collected, and appropriate laboratory tests were performed immediately. The observation period was one year. The tests were performed at diagnosis (21 tests), then 6 weeks (31 tests), 3 months (29 tests), 6 months (21 tests) and a year (9 tests) after diagnosis.

Laboratory and immunological parameters were assessed using standard methods by a hospital laboratory, and flow cytometry was used to assess PD-1 receptor expression. Statistical analysis of the results was performed using GraphPad Prism 8 and the following tests: ANOVA Kruskal-Wallis and multiple comparisons with multiple comparison correction: two-stage linear step-up procedure of Benjamini, Krieger, and Yekutieli were used to determine statistical significance (p<0.05).

**Results:** The highest % of PD1+CD4+ (median% 6.62 vs% 3.97 control) was observed in patients before treatment initiation, and the highest % of PD1 +CD8+ (median% 8.79 vs% 5.42 control) was observed 6 weeks after treatment initiation, however, these relationships are not statistically significant. PIMS-TS patients were characterized by an increased level of leukocytes compared to the healthy group (p=0.0029) and lower values ​​of lymphocytes (p=0.0006); both parameters normalized up to 6 weeks after the onset of the disease. We did not find statistical differences in the levels of IgG and IgM anti-SARS-CoV-2 between the groups of patients in particular follow-up periods or between patients and controls.

**Conclusion:** Our research showed interesting finding the overexpression of the PD-1 receptor on T lymphocytes and the change of its expression over time. We believe this topic requires in-depth statistical analyzes and evaluation of the usefulness of this parameter as an additional diagnostic factor. We showed no differences in the serological response to the SARS-CoV-2 virus between individual groups of patients and the control group. This allows for a hypothesis about the existence of factors predisposing to the development of PIMS-TS.

**Patient Consent:** Yes, I received consent

**Disclosure of Interest**: None declared

### P481. COVID-19 and children: complications and late outcomes at G. Zhvania academic clinic of pediatry of TSMU

#### E. Nakhutsrishvili^1^, T. Kutubidze^2^

##### ^1^Pediatric Rheumaatology, TSMU G. Zhvania’s Pediatric Academy Clinic, Tbilisi, Georgia, ^2^Tbilisi State Medical University, Tbilisi, Georgia

###### **Correspondence:** E. Nakhutsrishvili

**Introduction:** Covid - 19 has become a challenge for doctors around the world, including pediatricians since December 2019. Most children with COVID-19 have mild symptoms or have no symptoms at all. However, some children can get severely ill from COVID-19. They might require hospitalization, intensive care.

**Objectives:** In 2020-2021 at the Academic Pediatric Clinic named after G. Zhvania of Tbilisi State Medical University, we observed 60 children with post-covid complications and late Covid syndrome. More than half (32 children-53.3%) were under 5 years of age, with a predominance of boys (33 children-55%) who had had a Covid-19 infection 1.5-2 months before contacting us with a positive antibody reaction.

Most of them (51 children-85%) were healthy before the disease. vasculopathy, immune thrombocytopenia, iron deficiency anemia, coagulopathy, pneumonia-atelectasis, primary diabetes, exacerbation of the underlying disease - arthralgia, arthritis, and abnormal manifestations of sleep disturbance, general weakness and dizziness were noted. Separately, it is necessary to highlight the multisystem inflammatory syndrome in chirdren - MIS-C (8 children - 13%) proceeding with clinical signs of Kawasaki disease (mucocutaneous-lymphatic syndrome) with hectic temperature, polyserositis, hepatosplenomegaly, high rates of inflammation markers, a tendency to hypercoagulability. One patient had a coronary artery aneurysm. In 3 cases, the ANA and ANF titer was increased (up to 1: 640) and also nucleic cytoplasmic fluorescence with linear fibrils (actin-like fluorescence), which indicates immune reactions in Covid infection, which can explain the positive effect of corticosteroid therapy in the treatment of these patients. Only 22 patients (36%) were hospitalized, the rest were observed on an outpatient basis.

**Methods:** We studied 60 patients with post-Covid19 complications and “long Covid” syndrome.

**Results:** Majority of patients had no pre-existing diseases. MIS-C, arthritis/arthralgia, diabetis type 1, exacerbation of preexisting diseases, pneumonia/atelectasis, vasculopathy, ITP, Iron deficiency anemia, coagulopathy, sleep disturbances etc

**Conclusion:** The evidence that COVID-19 can have long-term impact children as well, including those with asymptomatic/symptomatic COVID-19. There is a need for more high-quality pediatric SARS-CoV-2 research and observation in dynamics.

**Disclosure of Interest**: None declared

### P482. Safety of the COVID-19 vaccination in children with juvenile idiopathic arthritis – a questionnaire study from two pediatric rheumatology centers in Poland

#### V. Opoka-Winiarska^1^, J. Lipińska^2^, K. Orczyk^2^, A. Michalak^3,4^, J. Burzyński^4^, O. Kądziołka^1^, E. Smolewska^2^

##### ^1^Department of Paediatric Pulmonology and Rheumatology, Medical Universiy of Lublin, Poland, Lublin, ^2^Department of Pediatric Cardiology and Rheumatology, Medical University of Lodz, Poland, Łódź, ^3^Department of Pediatrics, Diabetology, Endocrinology and Nephrology, ^4^Department of Biostatistics and Translational Medicine, Medical University of Lodz, Poland, Lodz, Poland

###### **Correspondence:** V. Opoka-Winiarska

**Introduction:** The safety of COVID-19 vaccines in children with JIA with long-term treatment has been drawing the researchers’ interest in the current pandemic reality.

**Objectives:** Therefore the principal objective of the study is to assess how often and what adverse events after COVID-19 vaccines develop the patients with juvenile idiopathic arthritis (JIA) and how these vaccines influence the disease activity.

**Methods:** The study project involved a 3-month follow-up of 43 patients aged 12.8 to 16.9 years (median -14.6 years), 17 boys and 26 girls, already diagnosed with JIA, who have been admitted to one of two pediatric rheumatology centers in Poland (Lodz and Lublin) for a regular check-up visit. Majority of the study group children (32.56%) had ERA-JIA subtype, polyarticular or oligoarticular JIA had equal 27.91%, systemic JIA – 6.98% and other (not classified) JIA subtype – 2.7% children.

The patients’ history regarding SARS-CoV-2 infection, exposure to these virus and current vaccination record was collected using appropriate validated questionnaire. All study group received *Pfizer COVID-19* vaccine. Furthermore, each patient was evaluated in terms of disease activity using Visual Activity Scale (VAS) and Juvenile Arthritis Disease Activity Score 27-Joint Count (JADAS27). The study group was subdivided into two age groups: 5-11 years and 12-18 years.

**Results:** Whole study group have been vaccinated for the obligatory vaccines according to polish mandatory vaccination calendar and more than 1/3 have got one or more additional recommended vaccinations. All parents of study group children and the majority of siblings have been vaccinated for COVID-19. The HLA-B27 antigen presence was confirmed in 27.91% (12/43) of children with JIA. Ten out of 43 (22.72%) children with JIA had COVID-19 infection before getting COVID-19 vaccine. More than a half (24/43 - 55.81%) of the children with JIA were treated with classical DMARDs (methotrexate, sulfasalazine), 3/43 (6.98%) - cyclosporine A. Eleven out of 43 (25.58%) had biological treatment – 2 with etanercept, 6- with adalimumab and 3 – tocilizumab. After first dose of COVID-19 vaccine 26/43 (58.14%) children presented typical adverse events: arm pain or oedema at the application side or weakness. The same number of children had side effects after second dose of COVID-19 vaccine, however the spectrum of the symptoms was wider (arm pain or oedema at the application side, weakness, headache, fever, lymphadenopathy, arrhythmia). The number of patients with active disease according to JADAS-27 was even lower after COVID-19 vaccination than before, however it was not a statistically significant difference (p>0.05). Furthermore, the grade of JADAS-27 activity (0-3) was higher in study group before COVID-19 vaccination (p=0.47).

**Conclusion:** The analysis indicated that children with JIA with remission without treatment or on the long-term treatment – classical or even biological, may be safely vaccinated for COVID-19. COVID-19 vaccination does not interfere with the JIA treatment and does not exacerbate the rheumatoid process. However, our results need to be confirmed in greater population.

Our results may be helpful in everyday practice of pediatric rheumatologist who may refer to the findings of this study during conversation with patients or parents regarding the safety of COVID-19 vaccines.

**Patient Consent:** Yes, I received consent

**Disclosure of Interest**: None declared

### P483. Relapse of acral chilblain-like lesions during covid-19 waves in a cohort of children and adolescents

#### F. Peri^1^, S. Pastore^2^, A. Taddio^2^, F. Biscaro^3^, S. Martelossi^3^, A. Tommasini^4^

##### ^1^Department of Medicine, Surgery, and Health Sciences, University of Trieste, Italy., ^2^Institute of Maternal and Child Health, IRCCS Burlo Garofolo, Trieste, Italy., Trieste, ^3^Pediatric Department, Presidio Ospedaliero di Treviso, Treviso, Italy., Treviso, ^4^Institute of Maternal and Child Health, IRCCS Burlo Garofolo, Trieste, Italy, Trieste, Italy

###### **Correspondence:** A. Taddio

**Introduction:** Chilblain-like lesions has been a peculiar phenomenon described in children and adolescents during COVID-19 pandemic. Little is known about its recurrence and whether this condition may be prodromic to more complex rheumatological disorders.

**Objectives:** To follow-up a cohort of children and adolescents that previously presented with chilblain-like lesions during early 2020 and 2021.

**Methods:** We performed a telephonic survey of 31 patients diagnosed with chilblain-like lesions during the first two waves of COVID-19 at two north-eastern Italian hospitals. We collected data regarding new episodes, other signs and symptoms of systemic disease and need for treatment.

**Results:** We reached 27/31 patients, most of them previously evaluated during early 2021. Seventeen patients (17/27, 63%) presented with new episodes of chilblain-like lesions. Relapse happened during winter months in all the patients; only two patients referred persistence of the condition also during the summer. Type I interferon signature tested positive (> 2) in 8/17 relapsed patients who underwent this examination. Most cases had a benign course with gradual resolution over several weeks with no need for specific treatment. Five adolescents received topical and systemic treatment due to painful lesions with functional impairment of hands and feet. Four patients had a minor relief from application of nifedipine cream and three patients had a progressive symptoms resolution after hydroxychloroquine treatment. None developed any signs or symptoms suggestive of a definite systemic rheumatologic disorder.

**Conclusion:** Chilblain-like lesions presented a recurrent pattern triggered by cold environmental temperatures in our cohort of children and adolescents. None of them developed other worrisome symptoms and most cases did not need specific treatment. We noted a recurrence trend in patient with a positive type I interferon signature test.

**Patient Consent:** Yes, I received consent

**Disclosure of Interest**: None declared

### P484. COVID-19 and autoimmunity (antiphospholipid antibodies syndrome)

#### A. K. Primero Nieto^1^, E. Faugier Fuentes^2^, A. Barba Aguilar^1^, E. Mercedes Pérez^2^, A. Guzmán Revilla^2^, H. Bermúdez Canales^2^, P. Ramos Tiñini^1^, H. Menchaca Aguayo^2^, S. Rodriguez Aguayo^2^

##### ^1^Reumatología, Hospital Infantil de Mexico, ^2^Reumatología, Hospital Infantil de México, Ciudad de México, Mexico

###### **Correspondence:** A. K. Primero Nieto

**Introduction:** The occurrence of autoimmune diseases can be generated by a variety of factors through hyperstimulation of the immune status. The factors can be classified as: genetic, environmental and hormonal. Viruses are an important component in the environmental factors affecting the immune system.

**Objectives:** To describe a clinical case, who developed autoimmunity following SARS-CoV-2 infection.

**Methods:** Clinical Case

**Results:** Male adolescent aged 15 years. No personal pathologic history. Admitted to our unit with clinical manifestations of epistaxis of 6 days of evolution, asthenia, adynamia, headache, fever, jaundice, acholia and choluria. He was admitted to the ER with shock data requiring endotracheal intubation, with laboratory tests reporting autoimmune hemolytic anemia (AIHA) of warm antibodies; with positive nasal antigen test for SARS-COV-2. She received treatment with immunoglobulin and 5 pulses of methylprednisolone, with subsequent full dose of steroid and tapering schedule. During her follow-up, persistent thrombocytopenia was documented, so an approach for autoimmune diseases was initiated. She presented triple positive marker integrating probable Antiphospholipid Antibody Syndrome (APS); due to hematologic condition (thrombocytopenia) she was started on azathioprine, with adequate response at the time.

**Conclusion:** This case describes the association of SARS-CoV-2 infection with autoimmune diseases such as APS and AHAI. Follow-up and subsequent treatment are described.

The ongoing pandemic induced by SARS-CoV-2, which has spread through many countries, has generated curiosity in knowing the possible mechanisms of inducing hyperstimulation of the immune system (1). The receptors of this virus are angiotensin-converting enzyme-2 (ACE-2) and transmembrane serine protease-2 (TMPRSS2), which are used to penetrate cells. ACE-2 is also widely expressed in endothelial cells. The synthesis of multiple autoantibodies is known, with an effect of occurrence of autoimmune diseases, possibly pre-existing. (2) The association of severely ill SARS-CoV-2 infected patients with circulating B2-glycoprotein or anticardiolipin (aCL) autoantibodies has also been investigated. It was found in recent study that patients with severe COVID-19 had significantly higher aCL antibody levels than patients with moderate disease. These observations undoubtedly suggest the possible existence of molecular mimicry due to the similarity between the peptide regions identified in the b2GPI molecule (and recognized by anti-b2GPI) and the membrane proteins of various viruses and bacteria. (3) This is an example of antibody production secondary or parallel to SARS-CoV-2 infection, which triggers disease and may require treatment and long-term follow-up. It is therefore important to consider autoimmunity as a consequence of SARS-CoV-2 infection.

**Disclosure of Interest**: None declared

### P485. Observational study on COVID-19 vaccination in paediatric patients with rheumatic diseases

#### M. T. Riccio, V. Aiello, F. Anselmi, B. Macri, M. Alessio, R. Naddei

##### Department of Translational Medicine, Section of Pediatrics, University of Naples Federico II, napoli, Italy

###### **Correspondence:** M. T. Riccio

**Introduction:** In December 2020, two anti-Sars-CoV2 vaccines were approved for pediatric population, which were initially intended for adult and frail population. The BNT162b2 and mRNA-1273 vaccines are chemically modified mRNA vaccines expressing the prefusion glycoprotein spike of Sars-CoV2 packed in lipid nanoparticles. This vaccine has been shown to be 94.1% efficacious to prevent symptomatic COVID-19 infection. In May 2021, these two vaccines were approved for teenagers aged 12 to 18 years, and in December 2021 for children aged 5 to 11 years. To date, many studies have been conducted on the efficacy of these vaccines in the adult population, while less is known in the pediatric population, particularly in children with chronic diseases being treated with background medication

**Objectives:** To examine how children with chronic rheumatic diseases undergoing treatment with background medication respond to Sars-Cov2 vaccination.

**Methods:** We administered a questionnaire to patients followed by the pediatric rheumatology unit of the maternal and child department of the AOU FEderico II in Naples. The questionnaire asked, how many doses were administered, if there were any side effects following the vaccination and if so which effects. After the second dose of vaccine had been given, we measured the anti-Sars-Cov2 antibodies, we assessed the presence or absence of antibodies. Once the data was collected, we divided the patients into two groups: a case group in which the therapy was not interrupted during the vaccination, and a control group that stopped the therapy.

**Results:** We recruited 84 patients, of whom 83 were given the Pfizer vaccine and one modern. All received a single dose of vaccine, while 79/84 received a second dose. After the first vaccine dose, 54/84 reported collateral effects (4 fever, 31 injection site reactions, 2 menstrual cycle changes alterations, 3 headache, 8 muscle pain, 2 asthenia, 1 lymphadenomegaly). In 44 patients the symptoms resolved, 7 used symptomatic therapy. After the second dose the side effects occurred in 70/79 patients (12 fever, 3 headache, 1 cough, 2 chills, 5 pain, 4 asthenia, 24 injection site reactions, 1 lymphadenomegaly). In 36 patients the symptoms resolved spontaneously, in 15 patients symptomatic therapy was used. 11 patients discontinued immunosuppressive therapy.

**Conclusion:** A great number of patients with chronic rheumatic diseases adhered to the vaccination campaign, no different or more severe collateral effects were reported in these than in the healthy population, and immunosuppressive drug administration and failure to discontinue did not lead to more severe collateral effects. Finally, we can state that anti-SarsCov2 vaccines can also be used in children with rheumatological diseases. The serological response of these patients is currently being evaluated.

**Patient Consent:** Yes, I received consent

**Disclosure of Interest**: None declared

### P486. COVID-19 infection and auto-inflammatory diseases in children

#### Z. Kolkhidova, S. Salugina, I. Nikishina, M. Kaleda, E. Fedorov, A. Shapovalenko

##### Pediatric Department, V. A. Nasonova Research Institute of Rheumatology, Moscow, Russian Federation

###### **Correspondence:** S. Salugina

**Introduction:**

It is known that the factors of severe COVID-19 are various comorbid conditions, including autoinflammatory diseases (AIDs) and concomitant immunosuppressive therapy. It is still unclear how COVID-19 affects the course and outcomes of AIDs. There is also data on the onset of AIDs after COVID-19. In the literature, information about the relationship between AIDs and COVID-19 is presented in some publications.

**Objectives:**

To study the frequency and features of the course of COVID-19 in children with various AIDs according to the Federal Rheumatology Center, to assess its impact on the course and outcomes of AIDs.

**Methods:**

The retrospective analysis included 36 pts (pts) with monogenic and multifactorial AIDs who had a COVID-19 confirmed by PCR test (12 pts), positive IgG (24) and/or IgM (2) antibodies.

**Results:**

For the period from 2020 to 2021, among 320 pts with rheumatic disease who underwent COVID-19, 36 (11.3%) had AIDs. Among them, Cryopyrin-associated periodic syndrome (CAPS) – 7 (20%), Familial Mediterranean Fever (FMF) -6 (17%), Blau-syndrome (BS) -2 (5%), most of them were pts with systemic juvenile idiopathic arthritis (sJIA) - 21 (58%). The number of boys and girls was the same. Median (Me) age was 13 years (y) [min 2; max 17]. The characteristics of pts are presented in Table 1. Me duration of AIDs at the time of COVID-19 was 8 y. All pts received stable therapy, which included DMARDs - 21 and Biologics (B) – 24 pts. The mean duration of therapy was 5.6 y. None of the pts received glucocorticoids. Half of pts (19) had mild clinical manifestations of COVID-19 (fever-11, rhinitis-5, anosmia-7, sore throat-1, cough-6, arthralgia-1, rash-1, dyspepsia-1), 17 (47%) children were asymptomatic. Pts with symptoms of COVID-19 compared with asymptomatic pts had a shorter duration of illness (5 vs 8 y) and received B for a longer period of time (5 vs 3 y). All pts were treated as outpatients except one girl, who required hospitalization due to initial signs of multisystem inflammatory syndrome with a good outcome. None of the pts demanded oxygenation support. At the time of, remission of AIDs was in 20 (56%), low activity in 10 (28%), high activity in 6 (16%) pts. After the COVID-19, a relapse and worsening of the disease developed in 11 (30%) pts, among them mainly pts with sJIA - 9 (82%). 3 pts required temporary withdrawal of DMARDs and B. Two girls after COVID-19 showed the development of sJIA 2 weeks and 3 months after infection.

**Conclusion:**

According to our study, pts with AIDs mostly had a mild or asymptomatic course of COVID-19, especially during remission. The therapy with DMARDs and B were continued in the majority of pts. Disease exacerbation after COVID-19 was noted in one-third of pts, more often with sJIA. We have noted 2 cases of sJIA after COVID-19 infection, thus not excluding the role of COVID-19 as a new triggering factor. Careful monitoring of the course of COVID-19 in children with AIDs, as well as long-term follow-up after COVID-19, is necessary.

**Patient Consent:** Yes, I received consent

**Disclosure of Interest**: None declared

**Table 1 (abstract P486).** Clinical characteristics of children with COVID-19 and AIDs

	sJIA (n=21)	CAPS (n=7)	FMF (n=6)	BS (n=2)
Sex (F/M)	15/6	2/5	1/5	0/2
Age, years (Me[min;max])	13 [6;17]	8[2;17]	12[8;17]	10 [3;17]
COVID-19 with symptoms	11(52%)	5(71%)	2(33%)	1(50%)
COVID-19 asymptomatic	10(48%)	2(29%)	4(67%)	1(50%)
**Treatment of RD**				
DMARDs (methotrexate/csA)	15/2	1	4	1
Colchicine	0	0	6	0
Biologics	16	5	2	1
Etanercept	2	0	1	0
Adalimumab	0	0	1	0
Infliximab	0	0	0	1
Tocilizumab	9	1	0	0
Sarilumab	3	0	0	0
Canakinumab	2	4	0	0
**The activity stage of AID by the start of COVID-19**				
Remission	14(67%)	4(57%)	1(16%)	1(50%)
Low disease activity	5(24%)	2(29%)	2(37%)	1(50%)
High disease activity	2(9%)	1(14%)	3(50%)	0
Worsening/flare of AID after COVID-19	9(43%)	0	1(17%)	1(50%)

### P487. Painful cervical involvement and febrile torticollis: a frequent symptom of PIMS

#### A. Schvartz^1^, R. Kechiche^1^, F. Bajolle^2^, S. Poignant^3^, R. Basmaci^4^, C. Pajot^5^, I. Melki^6^, L. Morin^7^, C. Adamsbaum^8^, N. Matsa^9^, M. Hofer^10^, I. Kone Paut^1,9^, C. Galeotti^1,9^ on behalf of French Covid-19 Paediatric Inflammation Consortium

##### ^1^Service de Rhumatologie Pédiatrique, CHU Bicêtre, APHP, Le Kremlin Bicêtre, ^2^Unité médico-chirurgicale de cardiologie pédiatrique, Hopital Necker Enfants Malades, APHP, Paris, ^3^Service de Pédiatrie Générale, CHU Nantes, Nantes, ^4^Service de Pédiatrie-Urgences, Hopital Louis Mourier, APHP, Colombes, ^5^Service de Néphrologie, Médecine Interne et Hypertension pédiatrique, CHU Toulouse, Toulouse, ^6^Service de Pédiatrie générale, Maladies infectieuses et Médecine Interne, CHU Robert Debré, APHP, Paris, ^7^Service de Réanimation Pédiatrique et Médecine Néonatale, ^8^Service de Radiologie Pédiatrique, ^9^Centre de Référence des Maladies AutoInflammatoires et des Amyloses, Ceremaia, CHU Bicêtre, APHP, Le Kremlin Bicêtre, France, ^10^Service de Rhumatologie Pédiatrique de Suisse Romande, Hôpital Universitaire de Lausanne, Lausanne, Switzerland

###### **Correspondence:** A. Schvartz

**Introduction:** Pediatric Inflammatory Multisystemic Syndrome associated to SARS-CoV2 (PIMS) or Multisystem inflammatory syndrome in children (*MIS*-*C*) happens 4 to 6 weeks after a SARS-CoV2 primary infection. PIMS has a wide clinical spectrum and this can lead to a late diagnosis responsible for poorer clinical outcome. Its early diagnostic recognition as well as its early management is important to avoid cardiac complications related to this pathology.

**Objectives:** We intend to describe a symptom not described enough, associated with PIMS which can lead to a better understanding of the wide spectrum of the disease, and help for faster diagnosis.

**Methods:** The JIR Cohort database, an international registry collecting data on patients with pediatric inflammatory diseases, was consulted to include patients between 03/15/20 and 12/31/2021.

**Results:** Of the 140 patients in whom a diagnosis of PIMS was retained, we present a series of 38 patients (27%) who presented at diagnosis or during evolution, febrile torticollis or painful cervical involvement. These patients were on average 8.2 years old (0.6-15.2). The proportion of boys was 14 out of 38 (37%). Twenty-four patients out of 33 (73%) were hospitalized in intensive care. Ten patients out of 38 (26%) underwent cervical imaging, 5 (50%) had abnormalities such as collection or infiltration of the soft tissues. At the therapeutic level, 27/38 patients (71%) received corticosteroid therapy, 33/38 (87%) immunoglobulins, and 26/38 (68%) antibiotic therapy.

**Conclusion:** PIMS is a pathology with significant clinical heterogeneity and severe consequences in case of delay in diagnosis and therapeutic management. In this epidemic context, it is important to consider PIMS in any patient with febrile torticollis, especially if he does not respond to antibiotics.

**Patient Consent:** Yes, I received consent

**Disclosure of Interest**: None declared

### P488. Relapse of JIA-related anterior uveitis: an observational single-centre study during SARS-COV2 vaccination campaign

#### C. Sembenini, G. Martini, D. Martino, A. Meneghel, F. Tirelli, M. Fastiggi, F. Zulian, M. E. Zannin

##### Paediatric Rheumatology Unit, Department for Woman and Child Health, University of Padova, Italy , ^2^Ophtalmology Unit University of Padua, Padua, Italy

###### **Correspondence:** C. Sembenini

**Introduction:** Juvenile idiopathic arthritis-related uveitis (JIA-AU) has been rarely reported as triggered by vaccines. Since the start of the vaccination campaign against SARS-CoV2, uveitis following the administration of the vaccine has been rarely reported.

**Objectives:** To evaluate the possible association between anti-Covid19 vaccination and relapses of JIA-AU in paediatric patients

**Methods:** A single-centre observational study was conducted in paediatric patients with JIA-AU followed at our Centre between April 2021 and March 2022.

**Results:** Out of 77 patients affected by JIA-AU, who underwent anti-SARS-CoV2 vaccination, 66 (86%) did not have any complication while 11 (14%) developed a relapse of AU within 6 weeks (mean 24 days, range: 3-42 days) after 2^nd^ or 3^rd^vaccine booster. The mean age of relapsed patients was 16.6 years (range 11-24); 10 presented unilateral relapse, one bilateral. All patients presenting JIA-UA relapse after vaccination, were on stable dose of treatment (all in methotrexate combined with adalimumab in 6 cases and abatacept in 2) and had been in AU remission for >12 months. In view of the vaccine, 10 patients had temporarily stopped systemic treatment: 7 patients for 2 weeks and 3 patients for > 2 weeks. The course of uveitis relapse was very benign as all patients went back into full clinical remission with just topical steroid therapy. No patients experienced arthritis relapse.

**Conclusion:** Recurrence of JIA-AU after Sars-Cov2 vaccination was observed in a minority of patients, was mild, transient and regressed only with topical treatment. These findings underline the need for a close ophthalmological follow-up of all patients with JIA-related AU who underwent Sars-Cov2 vaccination. Further observations are needed to establish whether the vaccine itself or the combined temporary suspension of the systemic treatment are responsible of the AU relapses.

**Disclosure of Interest**: None declared

### P489. Differences between mis-c presenting with Kawasaki-like symptoms and classical Kawasaki disease

#### S. Sener^1^, E. D. Batu^1^, U. Kaya Akca^1^, E. Atalay^1^, M. Kasap Cuceoglu^1^, Z. Balik^1^, Y. Bayindir^1^, E. Aliyev^1^, O. Basaran^1^, T. Karagoz^2^, Y. Ozsurekci^3^, Y. Bilginer^1^, S. Ozen^1^

##### ^1^Pediatric Rheumatology, ^2^Pediatric Cardiology, ^3^Pediatric Infectious Diseases, Hacettepe University, Ankara, Turkey

###### **Correspondence:** M. Kasap Cuceoglu

**Introduction:** Multisystem inflammatory syndrome in children (MIS-C) is a cytokine storm syndrome that may mimic Kawasaki disease (KD) or may present as a shock-like syndrome.

**Objectives:** In the presented study we describe the features that may help distinguish KD from MIS-C based on our clinical experience in pediatric patients.

**Methods:** We retrospectively evaluated MIS-C patients with KD-like symptoms and those with KD, followed between July 2020 and October 2021.

**Results:** Fifteen KD patients and 33 MIS-C patients with KD-like phenotype were included. The median age of MIS-C patients was older than KD patients at the time of diagnosis (7.6 vs. 3.4 years; p=0.004). Thirty MIS-C patients met the criteria for complete KD and the others for incomplete KD. Rash (p=0.044), oral mucosal changes (p=0.044), and cervical lymphadenopathy (p<0.001) were more frequent in KD. In contrast, MIS-C patients had more gastrointestinal symptoms (p<0.001), respiratory symptoms (p=0.044), and hypotension (p=0.002). MIS-C patients had lower lymphocyte and platelet counts than KD patients (p<0.001 for both). MIS-C patients often had high ferritin, D-dimer, troponin, and brain natriuretic peptide levels (p<0.001). MIS-C patients had significantly decreased left ventricular systolic functions (p=0.010). All patients received intravenous immunoglobulin as initial therapy. While most MIS-C patients were also given corticosteroid therapy, only five KD patients with refractory fever received corticosteroid therapy.

**Conclusion:** As the COVID-19 epidemic continues to spread, there is an urgent need to improve our understanding of this new disease. Although both KD and MIS-C can be perceived as cytokine storms of children, they have distinctive features.

**Trial registration identifying number:** not applicable

**Patient Consent:** Yes, I received consent

**Disclosure of Interest**: None declared

### P490. Disease relapse and protective antibodies after sars-cov-2 infection compared to vaccination against SARS-COV-2 in children with rheumatic diseases

#### T. Šinkovec Savšek^1^, M. Zajc Avramovič^1^, T. Avšič Županc^2^, T. Avčin^1,3^, N. Toplak^1,3^

##### ^1^Dpt of Allergol., Rheumatol., Clin. Immunol., UCH, UMC Ljubljana, ^2^Dpt of Microbiol., Immunol., Faculty of Medicine, Univ. Lj., ^3^Faculty of Medicine, Univ. Lj., Ljubljana, Slovenia

###### **Correspondence:** T. Šinkovec Savšek

**Introduction:** Children with rheumatic diseases (RD) are often immunocompromised because of the disease and/or the therapy they receive. At the beginning of COVID-19 pandemic there was a great concern about the possibility of severe SARS-CoV-2 infection in these children. The best method of protection is vaccination, so as soon as vaccine was licenced, we aimed to vaccinate them. However, a lot of them already got the infection before vaccination.

**Objectives:** Primary objective was to investigate the clinical presentation (CP) of SARS-CoV-2 infection in children with RD, the relapse rate (RR) of RD after infection and vaccination and to follow immunogenicity after infection and vaccination. Secondary objective was to follow safety and efficacy of vaccination against SARS-CoV-2 and to investigate if the immunogenicity and efficacy depended on the therapy. Adverse events (AE) after SARS-CoV-2 vaccination were studied and compared to healthy children.

**Methods:** This was partially a retrospective study where we collected data about children who had SARS-CoV-2 infection at regular visits at rheumatology outpatient clinic in UMC Ljubljana. Children who were vaccinated against SARS-CoV-2 were followed prospectively. Serology was followed on a long term in both groups. For ach child we collected demographic data, diagnosis, therapy, time of infection or vaccination, CP of infection, AE after vaccination, the outcome of infection, serology (IgA, IgG) and possible relapse of RD after infection or vaccination. For serology anti-SARS-CoV-2 ELISA IgG and anti-SARS-CoV-2 ELISA IgA from manufacturer Euroimmun, Medizinishe Labordiagnostika AG, Lübeck, Germany was used. For statistical analysis Fisher’s exact test and T-test were used.

**Results:** We collected data for 93 children with RD after infection (73% female) and for 43 after vaccination (63% female). In 93 children we registered 104 SARS-CoV-2 infections, 11 children got the infection twice. In 15% of events the infection was asymptomatic, 66% experienced mild CP, 18% moderate CP, 1% needed hospitalization. No statistically significant (SS) difference was detected on comparison of the CP between different therapy treatments for RD. 12% of children had a relapse after infection and 5% after vaccination, but the difference was not SS (p=0.23).

No SS difference was detected in AE after vaccination between children with RD and a healthy control group which consisted of 92 children (59% female), p=0.48 (1. dose), p=0.86 (2. dose). After vaccination 44% of children got infected with SARS-CoV-2, on average 4.4±1.7 months after vaccination; 47% of those children were treated with TNFα inhibitors. A SS difference was detected in serology between children treated with TNFα inhibitors and those treated with other or no medication (IgG p=1,68*10^-3^, IgA p=1,49*10^-2^).

**Conclusion:** RR was 12% after infection and 5% after vaccination. There was a trend towards higher RR after infection. The CP of SARS-CoV-2 infection in children with RD was mostly mild, no child had respiratory distress, and no one needed oxygen. One child was hospitalized because of dehydration. AE after vaccination were comparable between children with RD and healthy control group. A SS difference was detected in serology between children after infection and vaccination.

**Patient Consent:** Yes, I received consent

**Disclosure of Interest**: None declared

**Table 1 (abstract P490).** See text for description

	IgG			IgA		
Time frame (months)	After infection	After vaccination	p value	After infection	After vaccination	p value
< 3	2.62±2.73	8.84±2.64	1.29*10^-6^	1.71±2.32	5.84±4.45	1.23*10^-3^
3-6	3.08±2.20	6.99±2.77	2.51*10^-4^	2.62±2.84	2.68±2.68	0.95
6-12	1.93±1.66	8.87±2.87	1.06*10^-10^	2.33±2.50	8.03±5.72	2.65*10^-4^

### P491. IL-27 Expression is up-regulated in in south african patients with multisystem inflammatory syndrome in children

#### T. F. Spracklen^1^, S. Mendelsohn^1^, C. Butters^1^, H. Facey-Thomas^1^, R. Stander^1^, M. Erasmus^1^, J. Day^1^, C. Scott^1^, L. Zühlke^2^, T. Scriba^1^, K. Webb^1^

##### ^1^University of Cape Town, ^2^South African Medical Research Council, Cape Town, South Africa

###### **Correspondence:** T. F. Spracklen

**Introduction:** Multisystem inflammatory syndrome in children (MIS-C) is a severe acute inflammatory reaction to SARS-CoV-2 infection in children. Little is known about how MIS-C develops or its optimal treatment, particularly in Africa.

**Objectives:** This study sought to characterise differential expression of immune-related genes in a cohort of South African MIS-C patients and controls.

**Methods:** The cohort included 30 pre-treatment MIS-C cases and 54 healthy non-inflammatory paediatric controls. Febrile controls (n = 36) included patients with systemic lupus erythematosus (n = 7), Kawasaki disease (n = 8) and other inflammatory or infective conditions (n = 19). Longitudinal post-treatment MIS-C specimens were available for intravenous immunoglobulin (IVIG; n = 10) or IVIG and methylprednisolone (n = 30). Expression of 80 immune-related genes was determined by real-time quantitative PCR. Differentially expressed genes (DEGs) were identified through nonparametric pairwise comparisons between groups, adjusted by Holms correction. Receiver operating characteristic (ROC) analysis was used to investigate discriminatory capacity of variables.

**Results:** A total of 29 DEGs were identified in pre-treatment MIS-C compared to healthy controls. Only one gene, *IL27*, was differentially up-regulated in MIS-C compared to other febrile conditions (p = 0.00056), and none were significantly down-regulated. *IL27* expression could reasonably differentiate MIS-C from these febrile conditions (area under the ROC curve: 0.759; 95% CI: 0.632-0.886). Post-treatment follow-up data was available for 40 MIS-C patients for up to 1,200 hours after first treatment, and showed that *IL27* and the majority of other DEGs resolved to normal levels in the timeframe with IVIG and/or methylprednisolone. All patients clinically recovered.

**Conclusion:** These data indicate a role for IL-27 in development of MIS-C in South African children. IL-27 is a cytokine involved in differentiation of T helper 1 cells. Low serum levels of IL-27 have been described as a good prognosis indicator for adult COVID-19, and the up-regulation of this gene in our cohort suggests that IL-27 may be a key undescribed contributor to MIS-C phenotypes after SARS-CoV-2 exposure in children.

**Patient Consent:** Yes, I received consent

**Disclosure of Interest**: None declared

### P492. Usefulness of anakinra in multisystem inflammatory syndrome in children: results from an Italian survey

#### A. Taddio^1,2^, S. Della Paolera^1^, F. Ricci^3^, M. Giangreco^2^, L. Abbagnato^4^, A. Meneghel^5^, A. Smarrazzo^6^, A. Mauro^7^, F. Biscaro^8^, A. Meini^3^, R. M. Dellepiane^9^, M. Fabi^10^, I. Floretta^11^, F. La Torre^12^, L. Mambelli^13^, L. Verdoni^14^, M. C. Maggio^15^, S. Stucchi^16^, M. Tardi^7^, R. Sottile^17^, F. Zunica^3^, G. Zuccotti^18^, D. Montin^19^, R. Caorsi^20^, A. Consolaro^20^, G. Simonini^21^, M. Gattorno^22^, M. Cattalini^3^ on behalf of on behalf of Italian Pediatric Rheumatology Society - ReumaPED

##### ^1^University of Trieste, ^2^Institute for Maternal and Child Health - IRCCS Burlo Garofolo, Trieste, ^3^Pediatrics Clinic, ASST Spedali Civili di Brescia, University of Brescia, Brescia, ^4^U.O.C. Pediatria ASST Lariana Sant’Anna, Como, ^5^Department of Woman’s and Child’s Health, University of Padova, Padova, ^6^UOC Pediatria Multispecialistica, Dipartimento di Emergenza, Accettazione e Pediatria Generale, IRCCS Ospedale Pediatrico Bambino Gesù, Roma, ^7^Pediatric Emergency Unit, Santobono-Pausilipon Children’s Hospital, Napoli, ^8^Division of Pediatrics, Presidio Ospedaliero di Treviso, Treviso, ^9^Pediatric Intermediate Care Unit, Fondazione IRCCS Ca’ Granda Ospedale Maggiore Policlinico, Milano, ^10^Department of Pediatrics, University of Bologna, IRCCS Sant’Orsola-Malpighi Hospital, Bologna, ^11^Ospedale Santa Chiara, Trento, ^12^Pediatric Rheumatology Center, Pediatric Unit, “Giovanni XXIII”, Pediatric Hospital, Bari, ^13^UOC di Pediatria e Neonatologia, Ospedale di Ravenna, AUSL della Romagna, Ravenna, ^14^Paediatric Department, Hospital Papa Giovanni XXIII, Bergamo, ^15^Department of Health Promotion Sciences Maternal and Infantile Care, Internal Medicine and Medical Specialities “G. D’Alessandro”, University of Palermo, Palermo, ^16^S.C. Pediatria, ASST Grande Ospedale Metropolitano Niguarda, Milano, ^17^Department of Paediatrics, Pediatria 2, Santobono-Pausilipon Children’s Hospital, Napoli, ^18^Department of Pediatrics, University of Milan, Children’s Hospital V Buzzi, Milano, ^19^Department of Pediatrics and Public Health, University of Turin, Torino, ^20^Clinica Pediatrica e Reumatologia, IRCCS Istituto Giannina Gaslini and DINOGMI, Università di Genova, Genova, ^21^Pediatric Rheumatology Unit, AOU Meyer, University of Florence, Firenze, ^22^Center for Autoinflammatory diseases and Immunodeficiencies, IRCCS Istituto Giannina Gaslini, , Genova, Italy

###### **Correspondence:** A. Taddio

**Introduction:** Multisystem Inflammatory Syndrome in children (MIS-C) is systemic mulkti-organ inflammation following SARS-CoV-2 infection in children. No clear data are available about the best treatment approach: while the use of immunoglobulin is widely accepted, the use of corticosteroids is controversial and the role of biologic treatment is not defined yet.

**Objectives:** The main aim of this study was to define the better treatment option in MIS-C children and in particular to analyze the role of anakinra in this subgroup of patients.

**Methods:** This is a multicenter retrospective cohort study. MIS-C patients were defined if they fulfilled the RCPCH clinical definitions. Patients were treated according to the attending physician’s decision. The patients were then divided in 4 groups on the basis of the treatment options that the patients underwent at time of admittance: immunoglobulins (IVIG), IVIG plus methylprednisolone (≤ 2 mg/kg/day), IVIG plus high dose methylprednisolone (> 2 mg/kg/day) and anakinra with or without other drugs (IVIG and/or methylprednisolone). Primary outcomes were defined as the presence of at least one of the following clinical characteristics: death, the failure of initial treatment needing additional treatment for clinical worsening based on treating physician decision and cardiac involvement at the end of follow-up. We then divided our population into 2 groups: patients with good outcome (absence of any of the primary outcomes) and patients with bad outcome (presence of at least 1 primary outcome).

**Results:** At univariate analysis, persistent heart involvement at discharge was not associated with any clinical or laboratory test at time of diagnosis but but it was more frequent in those not receiving anakinra as initial treatment (3/20 vs 66/189; p=0.047). The need of vasoactive agents was associated with heart involvement at time of diagnosis as well as with the presence of hypotension/non-cardiogenic shock; similarly heart and pulmonary involvement were also associated with ventilatory support and intensive care admittance. Comparing the different treatment approaches adjusting for the propensity score we found that early treatment with anakinra was associated with higher probability to need intensive care admittance (p=0.0045; OR: 1,237-3,181), vaso-active agents use (p<0.0001; OR: 1,828-5,538) and initial treatment failure (p<0.0001; 8,003-30,112); however anakinra use was also associated to lower probability to have persistent heart disease at the end of follow-up (0,17-0,513) while no difference was found between high and low dose of corticosteroids at time of admission.

**Conclusion:** The early treatment with anakinra seems to be safe and very useful in patient with MIS-C. However, anakinra could be considered the best treatment options for those patients with higher probability to develop a severe disease; unfortunately it is not clear which are the clinical or laboratory characteristics at onset which may predict a more severe course.

**Disclosure of Interest**: None declared

### P493. No differences found in humoral and cellular immune response to mrna SARS-COV-2 BNT162B2 vaccine in teenagers with rheumatic diseases and healthy peers

#### C. Udaondo^1^, C. Cámara^2^, L. Miguel ^2^, R. Alcobendas^1^, C. Muñoz - Gomez^1^, C. Millan - Longo^1^, B. Diaz - Delgado^1^, I. Falces - Romero^3^, M. Diaz - Almiron^4^, J. Cano Ochando^5^, A. Mendez - Echevarria^6^, A. Remesal^1^, C. Calvo^6^

##### ^1^Paediatric Rheumatology, ^2^Immunology, ^3^Microbiology and Parasitology, ^4^Biostatitics, Hospital La Paz, ^5^National Microbiology Centre, Instituto de Salud Carlos III, ^6^Paediatric and Infectious Diseases, Hospital La Paz, Madrid, Spain

###### **Correspondence:** C. Udaondo

**Introduction:** Immunossupressive treatments for rheumatic diseases (RD) could potentially interfere with vaccine response. There is a lack of information about efficacy of the mRNA SARS-CoV-2 vaccine in teenagers with rheumatic diseases (RD) including cellular response.

**Objectives:** To evaluate safety and efficacy of the BNT162b2 vaccine, including humoral and cellular response, in teenagers with RD and immunosuppressive treatment compared with a control group.

**Methods:** Teenagers from 12 to 18 years with RD followed at a Pediatric Rheumatology unit in Madrid and non-RD teenagers receiving BNT162b2 mRNA vaccination were assessed 3 weeks after complete vaccination. Humoral response was measured by total antiSpike antibodies, and cellular response by IFN-γ and IL-2 in blood stimulated with SARS-CoV-2 Spike and M proteins.

**Results:** A total of 40 adolescents with RD and 24 healthy peers were recruited. The most frequent diagnosis was juvenile idiopathic arthritis (26/40, 65%) followed by Lupus (6/40, 15%). 60% of cases (23/40) received Tumour Necrosis Factor Inhibitors and 35% (14/40) methotrexate. No differences in spike-specific humoral or cellular response were found between groups (median IFN-γ response; 529 pg/ml in controls vs. 398 in RD patients, p 0.78, and median IL-2 in controls: 635 pg/ml vs. 497 in RD patients, p 0.22). 95% of RD patients and 96% of controls had maximum value of humoral response (IgG antibodies above 10 index). 40% of patients (26/64) had previous SARS-CoV-2 infection, 9 controls and 17 RD patients without differences. 70% of infections were asymptomatic. COVID-19 recovered individuals had higher IFN-γ than naive subjects in both groups (controls: median 859 pg/ml in recovered vs. 450 in naïve p 0.017, RD patients: 850 vs. 278 p 0.024). No serious adverse events or flares were reported following vaccination.

**Conclusion:** Standard of care treatment for teenagers with RD did not affect the humoral and the cellular immunity to BNT162b2 mRNA vaccination compared to a control group, suggesting that no treatment discontinuation or additional dosing would be required compared to healthy peers. Previous SARS-CoV-2 infection was the most relevant factor in the immune response.

**Patient Consent:** Not applicable (there are no patient data)

**Disclosure of Interest**: None declared

### P494. Multisystem inflammatory syndrome in children related with SARS-COV 2 (MIS-C) multicenter study in Mexico

#### A. V. Villarreal Treviño^1,2^ on behalf of COLIBRI, V. Ramírez Nova^3^, I. Pelaez Ballestas^3^, S. G. Rosiles De la Garza^2^, F. Moctezuma Rios^3^, M.-G. Rodríguez Maldonado^4^, P. A. León^5^, M. De la O Cavazos^2^, N. Rubio Pérez^2^ on behalf of COLIBRI, R. Hernández Martínez^5^, R. C. Calderón Zamora^2^, K. Ruiz Lopez^3^, J.-F. Heredia Gozález^3^, F. García Rodríguez^2^ on behalf of COLIBRI on behalf of COLIBRI

##### ^1^Pediatrics, Hospital Materno Infantil SSA, Guadalupe, NL, ^2^Pediatrics, Hospital Universitario “Dr. José E. González”, Monterrey, ^3^Rheumatology, Hospital General de México, Eduardo Liceaga “Dr. Eduardo Liceaga”, ^4^Rheumatology, ^5^Pediatrics Intern Medicin, Cento Médico Nacional “La Raza”, Instituto Mexicano del Seguro Social, Mexico City, Mexico

###### **Correspondence:** A. V. Villarreal Treviño

**Introduction:** An emerging entity described during the Coronavirus disease 2019 pandemic was initially reported as cases of older school-aged children and adolescents presenting with prolonged fever, shock, abdominal pain, and cardiac dysfunction after SARS-CoV-2 infections, termed as Multisystem Inflammatory Syndrome in children (MIS-C). European and North American cohorts described this syndrome is more frequent in males, adolescents and from racial and ethnic minorities.

**Objectives:** This study aims to describe epidemiological and clinical characteristics of Mexican patients with MIS-C at admission at four centers in Mexico.

**Methods:** This is a cross-sectional retrospective study of four Hospitals of specialized-care in Mexico, 2 in the center of the country (Mexico City) and 2 in northeast (Monterrey). Patients classified with MIS-C according to the WHO definition, between April 2020 and April 2022 were included. Epidemiological and clinical characteristics at the diagnosis were collected and descriptive analysis using percentages, median, intercuartile ranges (IQR), and a Kaplan-Meier survival analysis was performed.

**Results:** 106 participants, n=78 (73.5%) form the northeast and n=33.(31.1%) from center. n=67 (84.5%) were men, with a median age of 66.5 months (IQR 27-120). The median time from onset to care at the center was 6 days (IQR 4-8).

MIS(-C) was characterized by fever n=106 (100%), mucocutaneus n=91 (85.8%), gastrointestinal n=62 (58%) and cardiocirculatory manifestations n=42 (39.6%) and increased inflammatory biomarkers n= 89 (90.8%) with a C reactive Protein median 17.3mg/dL (IQR 3-68.8), n=15.9 (15%)presented overweight according to the Body Mass Index percentile, schock n=29(27.4%), n=37 (35%) needed intensive care with a median length of stay at this unity of 7 (IQR 3-9) days. The laboratory data are shown in Table 1. The n=95 (90%( received Human Intravenous Immunoglobulin, n=81 anticoagulants. The median of hospitalization days were 8 (IQP 5-13), mortality higher than other cohorts n=6 (8%), Macrophage activation Syndrome reported in n=6 (8%) and n=5(5.7%) had evidence of thrombosis. Kaplan-Meier curve of time to discharge or death compared by the presence of hemodynamic alterations on admission, the curve shows higher day of hospital stay and death in patients with hemodynamic alterations at admission p< 0.01.

**Conclusion:** This cohort showed more severe disease in MIS-C patients with hemodynamic alterations at admission. Mexican children showed similarities to other cohorts in the literature, sever cases at northeast population with increased mortality were observed. Tanslational studies and long-term follow-up of the patients are necessary to clarify the pathophysiology and predictive factors of severity to establish the long-term prognosis of this new entity.

**Patient Consent:** Yes, I received consent

**Disclosure of Interest**: None declared

**Table 1 (abstract P494).** See text for description

Laboratory n=106	Median (IQR)
Haemoglobin (mg/dL)	11.7(10.1 – 12.9)
Leucocyte (x10^3^ /uL)	9.790 (6.350 – 15.500)
Neutrophils(x10^3^ /uL)	7.300 (3.104 – 12.750)
Lymphocytes (x10^3^ /uL)	1.075 (3,104 - 12,750)
Platelets (x10^3^ /uL)	161.000 (91.840 – 272.000)
Seric ferritin (ng/dL)	464 (91,840 – 272,000)
Aspartate aminotransferasa AST (U/L)	46 (21 – 86)
Triglycerides (mg/dL)	155 (21 – 86)

### P495. COVID-19 in children with juvenile idiopathic arthritis on DMARDs treatment

#### T. Marushko, O. Vovk, Y.-E. Kulchytska

##### ^1^Pediatrics-2, Shupyk National Healthcare University of Ukraine, Kyiv, Ukraine

###### **Correspondence:** O. Vovk

**Introduction:** The occurrence of infectious diseases in children with juvenile idiopathic arthritis on immunosuppressive treatment has its own peculiarities, so they may be at higher risk of severe coronavirus disease development following the infection with SARS-CoV-2.

**Objectives:** To evaluate the COVID-19 infection incidence in children with juvenile idiopathic arthritis and to study the COVID-19 infection course in children with JIA who are being treated with disease-modifying antirheumatic drugs (DMARDs).

**Methods:** We analyzed 63 clinical cases of Covid-19 infection in children with JIA who received DMARDs. Covid-19 infection in patients with JIA was diagnosed by a positive PCR test for SARS-CoV-2 and/or a positive serology test and presence of an infected family member.

**Results:** We examined 63 children diagnosed with JIA, 73% of whom had SARS-CoV-2 infection. 32 patients received methotrexate, and 14 patients received biologic DMARDs (bDMARDs) (adalimumab) and methotrexate. There were no cases of severe Covid-19 disease course that required inpatient treatment. 12.4% of Covid-19 infected children on methotrexate treatment had no clinical symptoms of infectious disease. 1 patient from the group receiving adalimumab and methotrexate also had no clinical symptoms of infectious disease. 46.9% of patients on methotrexate had a mild course of coronavirus infection (rhinopharyngitis). 14.2% of children receiving bDMARDS and methotrexate had a mild course. 4 patients were diagnosed with viral pneumonia (1 child on methotrexate and 3 children on bDMARDS and methotrexate). 39.1% of patients, due to SARS-CoV-2 infection, were diagnosed with flare in the form of arthritis, which required intra-articular administration of glucocorticoids in 33.3% of cases and nonsteroidal anti-inflammatory drugs treatment in 44.4% of cases.

**Conclusion:** JIA patients, compared with healthy children, are more vulnerable to SARS-CoV-2 infection. The clinical course of COVID-19 infection in children with JIA who have received immunosuppressive drugs such as conventional synthetic or biologic DMARDs may have a mild course of SARS-CoV-2 infection. However, it was found that SARS-CoV-2 infection had a significant impact on JIA activity, requiring the use of intra-articular glucocorticoid administration and prescription of nonsteroidal anti-inflammatory drugs. To better investigate the associations between JIA, pharmacological management, clinical manifestations, and possible consequences of SARS-CoV-2 infection in these patients, further studies with a larger number of cases are needed.

**Patient Consent:** Yes, I received consent

**Disclosure of Interest**: None declared

### P496. Parental social media habits and its effect on children vaccination against COVID-19

#### M. Yıldız, F. Haslak, A. Gunalp, A. Adrovic, S. Sahin, K. Barut, O. Kasapcopur

##### Pediatric Rheumatology, Cerrahpasa Medical School, İstanbul, Turkey

###### **Correspondence:** M. Yıldız

**Introduction:** The systems related to the internet and internet network have been in a great change and development since they were first used. While there was a one-sided flow of data in the first years of these systems, two-sided data transfer is possible today, which gives the opportunity to use internet systems for different purposes. One of the most important results of these changes has been the establishment of social media networks, the basis of which is personal data sharing. Although social media networks are quite new, they occupy a prominent place in people’s daily lives in today’s modern world.

**Objectives:** The aim of this study is to assess the parental social media habits and its effect their willingness to allow their children to receive COVID-19 vaccination.

**Methods:** A web-based questionnaire was circulated among the families of patients under 12 who have been following up at Istanbul University- Cerrahpasa pediatric rheumatology department. The families were asked about their social media habits and their intention to vaccinate to their children against COVID-19.

**Results:** Parents of 138 children (75 females) with rheumatological diseases were completed the form. The families stated that the most common sources they used for getting information about COVID-19 were television/radio [99 (71.7%)], social media [71 (51.4%)] and health professionals [69 (50%)]. Instagram [123 (89.1%)], Facebook [96 (69.6)] and Twitter [45 (32.6)] was the most used social media platforms among the families. A total of 60.9% of the parents stated that they spend more than an hour a day on social media platforms. To stay informed was the first reason for using social media platforms, which is stated by 55 (39.9) of the parents**.** Other common reasons for using social media were to keep track of daily events (35.5%) and to keep touch with friends (17.4%). Majority of the parents (90.6%) were following a medical physician on social media and 89.1% of them stated that the statements about medical conditions by the physicians has a significant effect on their decisions if the statements are related to their field of expertise. When the parents were asked about their intention to vaccinate their children in the case the Ministry of Health recommends vaccinating children under the age of 12; 57 (41.3%) of parents answered “yes”, 14 (10.1%) “no” and the remaining “not decided yet”. There were no significant differences between the parents stated they would vaccinate their children and would not vaccinate their children according to social media accounts they have, the number of accounts, time spent on social media (p value for all > 0.05).

**Conclusion:** The results of our study pointed out that there is no direct connection between the parental social media habits and willingness to their children. The fact that vast majority of families follow at least one doctor on social media and that declare the statements made by the doctors they follow on social media are important in their decision-making processes may be emphasizing the importance of the use of social media by physicians. Further studies are needed.

**Patient Consent:** Yes, I received consent

**Disclosure of Interest**: None declared

### P497. Effectiveness of the BNT162B2 MRNA COVID-19 vaccine in adolescents with juvenile-onset autoimmune inflammatory rheumatic diseases

#### A. Ziv^1,2^, M. Heshin-Bekenstein ^2,3^, R. Haviv^4,5^, S. Kivity ^5,6^, D. Netzer ^7^, S. Yaron^7^, Y. Schur^7^, T. Egert^8^, Y. Egert^9^, Y. Sela ^10^, P. J. Hashkes ^11,12^, Y. Uziel ^5,13^

##### ^1^Pediatrics, MEIR HOSPITAL, Kfar Saba, ^2^Sackler School of Medicine , Tel Aviv University, Tel Aviv , ^3^Pediatric Rheumatology Service, Dana Children’s Hospital, Tel Aviv Medical Center, Tel Aviv, Israel, Tel Aviv, ^4^Pediatric Rheumatology Unit, Meir Medical Center, Kfar Saba, ^5^Sackler School of Medicine , Tel Aviv, ^6^Rheumatology Unit, Meir Medical Center, Kfar Saba, ^7^Community Medical Services Division, Clalit Health Services, Tel Aviv, ^8^Inbar, NPO, Ramat Gan, ^9^Inbar, NPO, Ramat Gan, ^10^The Research Center for Internet Psychology, School of Communications, Reichman University, Herzliya, ^11^Pediatric Rheumatology Unit, Shaare Zedek Medical Center, Jerusalem, ^12^Faculty of Medicine, Hebrew University of Jerusalem, Jerusalem, ^13^Pediatric Rheumatology Unit, Meir Medical Center, Kfar Saba, Israel

###### **Correspondence:** A. Ziv

**Introduction:** The effectiveness of the BNT162b2 mRNA COVID-19 vaccine for adolescents with juvenile-onset autoimmune inflammatory rheumatic diseases (AIIRD) is unknown. Several studies suggest attenuated immunogenicity in patients with AIIRD.

**Objectives:** This study evaluated the effectiveness of the BNT162b2 mRNA COVID-19 vaccine in preventing COVID-19 infection in adolescents with juvenile-onset AIIRD compared to healthy controls.

**Methods:** We used data from Clalit Health Services, the largest healthcare organization in Israel, to conduct an observational cohort study from June to December 2021, involving adolescents ages 12–18 years, diagnosed with AIIRD. Study outcomes included documented COVID-19 infection in relation to vaccination status and immunomodulatory therapy. We estimated vaccine effectiveness as one minus the risk ratio. Healthy adolescents in the same age group served as controls.

**Results:** A total of 1,639 adolescents with AIIRD (juvenile idiopathic arthritis, systemic lupus erythematosus, or familial Mediterranean fever) were included and compared to 524,471 adolescents in the same age range. There was no difference in COVID-19 infection rates after the second dose of vaccine for those with AIIRD and healthy controls (2.1% vs. 2.1% respectively, p=0.99). The estimated vaccine effectiveness for adolescents with AIIRD was 76.3% after the first dose, 94.8% after the second and 99.2% after the third dose.

**Conclusion:** We found that the BNT162b2 mRNA vaccine is highly effective against COVID-19 in adolescents with AIIRD, similar to healthy controls. Immunomodulatory therapy did not affect its effectiveness. These results can encourage adolescents with AIIRD to get vaccinated against COVID-19.

**Patient Consent:** Not applicable (there are no patient data)

**Disclosure of Interest**: None declared

**Table 1 (abstract P497).** Vaccination status and COVID-19 infection among adolescents with AIIRD

Vaccine doses	Total	0	1	2	3
Adolescents with AIIRD	1,639	451 (27.5%)	179 (10.9%)	745 (45.5%)	264 (16.1%)
COVID-19 infection	277	234	22	20	1
Infections / vaccinated*	16.9%	51.9%	12.3%	2.7%	0.4%
Vaccine effectiveness**			76.3%	94.8%	99.2%

* Percentage of COVID-19 infection per vaccine dose

** Vaccine effectiveness calculated according to the formula: VE=(NVAC-VAC)/NVAC

(VE - Vaccine effectiveness, NVAC - Attack rate among the unvaccinated, VAC - Attack rate among the vaccinated)

## Publication only abstracts

### PU01. Tuberculosis after Tocilizumab in SJIA

#### J. N. Bathia, P. Pal

##### Department of Pediatric Rheumatology, Institute of Child Health, Kolkata, India

###### **Correspondence:** J. N. Bathia

**Introduction:** Tuberculosis is a major cause of morbidity in low an middle income countries. Antitumour necrosis factor targeted agents (anti TNFs) are known to increase the risk of reactivation of latent tuberculosis infection (LTBI). However, the risk in non anti TNF biologics such as Interleukin 6 inhibitor Tocilizumab (TCZ) has been noted to be very low or absent. Sporadic cases have been reported. One may be question if it is primary TB infection or LTBI reactivation post TCZ

**Objectives:** We present a 11 years old girl who was diagnosed with systemic onset juvenile idiopathic arthritis and given TCZ. However, she developed fever after the first dose which was subsequently diagnosed as tuberculosis.

**Methods:** This 11 years old girl initially presented with a history of intermittent for 3 months which managed conservatively by antibiotics and antipyretics. On examination we found that there was left sided cervical lymphadenopathy. Other examination was within normal limits. The patient was admitted for almost a month however the fever was still persistent. Investigations revealed anemia, progressively increasing leucocytosis with a neutrophilic preponderance. Urine and blood cultures were negative. Bone marrow examination and cervical lymph node biopsy was normal. tuberculosis was ruled out. USG of abdomen and CT scan of thorax were within normal limits. As there was a maternal history of Carcinoma a PET scan was done which was also normal. The child received multiple antibiotics however the fever persisted. She was discharged later on as the fever spikes decreased. Two months after that the fever spikes increased however this time there was an evanescent rash, cervical lymphadenopathy and and left knee diagnosis. Finally a diagnosis of Systemic onset juvenile idiopathic arthritis was made and the child started on subcutaneous methotrexate. Due to relapses leflunomide was added an subsequently TCZ administered. The fever reappeared along with cough.

**Results:** A chest Xray was done which showed mediastinal widening. Contrast enhanced CT scan of thorax showed Nodular opacity in RLL with few small pulmonary nodules and conglomerated Mediastinal necrotic lymph nodes. The tuberculin sensitivity test was also positive. All this pointed towards pulmonary tuberculosis and anti tubercular therapy was started.

**Conclusion:** Tocilizumab is a humanized anti-IL-6 receptor antibody which inhibits the binding of IL-6 to its receptors, IL 6 has both pro- and anti- inflammatory action and is involved in Th17 and Th22 cell differentiation which is critical for anti-mycobacterial activity. It is produced early during mycobacterial infection. It has been note that administration of tocilizumab is associated with a very low or absent risk of tuberculosis reactivation. In our case the child was initially screened for tuberculosis before the diagnosis of sJIA. However, after administration of TCZ she still developed tuberculosis. If this reactivation of latent tuberculosis or primary infection remains debatable.

**Disclosure of Interest**: None declared

### PU02. Chronic non bacterial osteomyelitis in 11 Indian children - a single center experience

#### D. B. Pandya, M. Aggarwal, S. Sawhney

##### Pediatric & Adolescent Rheumatology Department, Sir Gangaram Hospital , New Delhi , India

###### **Correspondence:** D. B. Pandya

**Introduction:**

Chronic non-bacterial osteomyelitis (CNO) is an autoinflammatory bone disorder usually affecting children and adolescents. CNO covers a wide clinical spectrum from rather mild, time-limited, monofocal bone inflammation to severe chronically active or recurrent multifocal bone inflammation. Clinical signs of bone inflammation include localized skin redness (rare), warmth and/or swelling, and pain. CNO is a diagnosis of exclusion. MRI is a choice of investigation in diagnosis , follow up and exclusion of other close mimics. Management usually involves non-steroidal anti-inflammatory drugs (NSAIDs), corticosteroids, disease-modifying anti-rheumatic drugs (DMARDs, usually methotrexate or sulfasalazine), anti-TNF agents or bisphosphonates and anti IL-1 agents especially in monogenic forms of CNO.

**Objectives:**

We aim to unveil characteristics of CNO in eleven Indian children at our center in India.

**Methods:**

This is a retrospective analysis of 11 children who visited our unit between Aug 2013 to March 2022 and were diagnosed with CNO after excluding close mimics. Our collected data includes demographics , clinical presentation , management and follow up details.

**Results:**

This Table Shows Characteristics of 11 Indian Children with CNO at our Unit

**Conclusion:**

Pre-adolescent girls are most commonly affected. Tibia is the most common bone involved and metaphysis is the most common site of involvement. Some of these patients presented with low grade fever and skin lesions. MRI and Bone Scan remains the first imaging modality. Most of the patients responded to adequate course of NSAIDs. Few patients required bisphosphonates and biologics to control the disease.

**Trial registration identifying number:** 1. Curr Osteoporos Rep. 2017; 15(6): 542–554. Published online 2017 Oct 27. doi: 10.1007/s11914-017-0405-9

Chronic Recurrent Multifocal Osteomyelitis : Presentation , Pathogenesis & Treatment

Sigrun R. Hofmann,^1^ Franz Kapplusch,^1^ Hermann J. Girschick,^2^ Henner Morbach,^3^ Jessica Pablik,^4^ Polly J. Ferguson,^5^ and Christian M. Hedrich^1,6,7^

**Patient Consent:** Yes, I received consent

**Disclosure of Interest**: None declared

**Table 1 (abstract PU02).** See text for description

Mean Age at Onset	9.8 years (2-16)
Sex Ratio F : M	2.6 :1
Average Diagnostic delay	7 months
Fever at Onset	3 / 11 (27%)
Bone Involvement	
Tibia	9/11 (82%)
Clavicles	4 / 11 (36%)
Metacarpals & Metatarsals	2 / 11 (18%)
Spinal leions	2/11 (18%)
Calcaneum , Ribs , Femur	2/11 (18%)
Scapula , Navicular , Humerus , Ulna	1 / 11 (9%)
Which region of bone was involved	
Metaphysis	7 / 11 (63%)
Epiphysis & Diaphysis	4 / 11 (36%)
Type of Lesion at Onset	
Mixed	6 / 11 (54%)
Lytic or Sclerotic	5 / 11 (46%)
Skin Lesions	3 / 11 (27%)
Elevated CRP +/- ESR at Onset	6 / 11 (54%)
Imaging modality used for Diagnosis	
Bone Scan	8 / 11 (72%)
MRI	8 / 11 (72%)
CT Scan	4 / 11 (36%)
PET Scan	2 / 11 (18%)
Bone Biopsy	6 / 11 ( 54% )
Medications used in our Patients	
NSAIDs	11 / 11 (100%)
Bisphosphonates (BPs)	5 / 11 (46%)
BPs used in Spinal Lesion	2 / 11 (18%)
MTX	3 / 11 ( 27%)
TNF Inhibitors	2 / 11 (18%)
TNFi used in Spinal Lesion	1 / 11 (9%)
Follow Up Details	
Average number of flares	3
Status not known due to lost on follow up	4 / 11 (36%)
Inactive disease as per last follow up	5 / 11 (54%)
Flare as per last follow up	2 / 11 (18%)

### PU03. Clinical and laboratory presentation of Neonatal Lupus Erythematosus (NLE) - case report

#### D. S. Lazarevic^1,2^, J. Vucic^3^, Z. Milosevic-Anđelkovic^3^, H. Stamenkovic^1,2^, T. Stankovic^1,2^, J. Vojinovic^1,2^

##### ^1^Pediatrics, Faculty of Medicine, University of Nis, Nis, Serbia, ^2^Pediatric Rheumatology and Immunology, ^3^Neonatology Department, Clinic of Pediatrics, Clinical Center Nis, Nis, Serbia

###### **Correspondence:** D. S. Lazarevic

**Introduction:** Neonatal lupus erythematosus (NLE) is a rare acquired autoimmune condition that is present at birth with diverse clinical presentation. It is transient disorder with transplacental passage of mother’s antibodies. Often is misdiagnosed as intrauterine infection or sepsis

**Objectives:** Clinical and laboratory presentation of newborn with NLE.

**Methods:** We report a child with NLE in mother without positive history on autoimmune diseases.

**Results:** We present premature male newborn, small for gestation age with clinical symptoms starting just after birth with leucopenia and thrombocytopenia, but without elevated inflammatory markers. Antibiotics were administrated despite all bacterial cultures were sterile. In the first weeks of life severe thrombocytopenia required administration of immunoglobulins and platelets transfusion, while erythematous rash on face has appeared. A detailed hematological and the most common neonatal virological diagnostic testing were performed. AIDS and syphilis were excluded. Serological tests have confirmed active herpes simplex infection and parenteral acyclovir were started until polymerase chain reaction (PCR) results were negative. Suspected immunodeficiencies and neonatal alloimmune thrombocytopenia were ruled out, as immunoglobulin levels and flowcytometry were normal. Within the first month skin rash have changed and extended on whole face and neck. Skin lesions becomes much reddish, annular and clearly demarked. Clinical spectrum with hematological disorders were enough to suspect on NLE. Anti-Ro, Anti-La and ANA antibodies in child have confirmed our diagnosis, while mother has and Anti dsDNA antibodies associated. ECG Holter monitoring have excluded congenital heart block. Only supportive treatment was advised.

**Conclusion:** Neonatal lupus erythematosus should be considered in newborns with annular skin lesions, hematological disorders and without congenital heart block even in clinically healthy mothers.

**Patient Consent:** Yes, I received consent

**Disclosure of Interest**: None declared

### PU04. Juvenile systemic lupus erythematosus – experience of a tertiary hospital

#### M. Bastos Gomes^1^, A. Costa Azevedo^1^, A. L. Carvalho^2^, I. Cardoso^3^, F. Aguiar^4,5^, M. Rodrigues^4,5^, S. Ganhão^4^, I. Brito^4,5^

##### ^1^Pediatric Department, Unidade Local de Saúde do Alto Minho, Viana do Castelo, ^2^Pediatric Department, Hospital de Braga, Braga, ^3^Pediatric Department, Centro Hospitalar Vila Nova de Gaia/Espinho, Vila Nova de Gaia, ^4^Pediatric and Young Adult Rheumatology Unit, Centro Hospitalar Universitário de São João, ^5^Faculdade de Medicina da Universidade do Porto, Porto, Portugal

###### **Correspondence:** M. Rodrigues

**Introduction:** Juvenile systemic lupus erythematosus (jSLE) is a systemic autoimmune disease, which can affect several organs and systems, diagnosed in pediatric age. Despite representing 15-20% of SLE cases, it is characterized by a more severe course.

**Objectives:** Characterize the population of patients with jSLE followed at a tertiary university hospital.

**Methods:** Retrospective study, including the patients with jSLE followed at the Pediatric and Young Adult Rheumatology Unit, diagnosed between 1987 and 2021. Demographic, clinical and laboratory data were collected. Disease activity was evaluated using Systemic Lupus Erythematosus Disease Activity Index 2000 (SLEDAI-2K) and SLE Disease Activity Score (SLE-DAS) (2018), and the disease damage was evaluated using the Systemic Lupus International Collaborating Clinics (SLICC) / American College of Rheumatology (ACR) damage index.

**Results:** A total of 44 patients were included, 93% female, with a median age at diagnosis of 15 years (IQR 12-16) and median follow-up of 8 years (IQR 5-13). There were 2 deaths (sudden death; both with pulmonary hypertension (HTP)). The clinical manifestations were: mucocutaneous (72%), hematological (70%), osteoarticular (59%), renal (41% - class IV: 63%, class V: 21%), constitutional symptoms (27%), vascular (22%), serositis (16%), gastrointestinal (14%) and pulmonary (2%) manifestations. All patients had positive anti-nuclear antibodies, 59% with high title (>1:1000). Ongoing treatment was: glucocorticoids (70%), hydroxychloroquine (78%), mycophenolate mofetil (41%) and azathioprine (20%). 13% received rituximab. SLEDAI-2K at diagnosis was significantly higher than at last patient assessment (10 vs 2, respectively, p<0.001). Regarding disease activity, 87% have SLEDAI≤4 and 42% SLEDAI=0; SLE-DAS ≤2.08 in 80%, SLE-DAS ]2.08-7.64] in 9% and SLE-DAS>7.64 in 11%. SLICC/ACR damage index was ≥ 1 in 13%.

**Conclusion:** The most frequent manifestations in this cohort were mucocutaneous and hematological, which is in agreement with the literature. Major organs were commonly affected, including 41% with renal involvement, corroborating the greater morbidity associated with this age group. Deceased patients had risk factors: one received a delayed diagnosis and had shrinking lung syndrome and HTP, the other had an overlap with systemic sclerosis and had severe early-onset HTP. Most patients have low disease activity, which is possible in the face of early diagnosis and timely and effective immunomodulatory and/or immunosuppressive therapy, as well as regular follow-up in a specialized unit.

**Patient Consent:** Yes, I received consent

**Disclosure of Interest**: None declared

### PU05. The rare cause of back pain – the crucial role of ultrasound as the first step to diagnose Takayasu arteritis

#### Z. Pytelová^1^, J. Čivrný^2^, T. Klimas^2^, D. Klepárník^1^, K. Bouchalová^1^

##### ^1^Paediatric Rheumatology, Department of Paediatrics, ^2^Department of Imaging, Faculty of Medicine and Dentistry, Palacký University Olomouc and Olomouc Hospital, Olomouc, Czech Republic

###### **Correspondence:** Z. Pytelová

**Introduction:** Takayasu arteritis is a rare large-vessel vasculitis affecting the aorta, its major branches and the pulmonary artery, and common symptoms are non-specific myalgia, weight loss and fever or absence of peripheral pulse and claudication in later stages.

**Objectives:** We aim to describe a case of a rare disease with early diagnosis and highlight the importance of thorough ultrasound imaging.

**Methods:** We present a case report.

**Results:** A 13-years-old girl was admitted for back pain lasting 1.5 months. The pain woke the girl up from sleep and there was no effect of NSAIDs. For a psoriatic lesion on her elbow, she was examined by a dermatologist once.

On the day of admission, there were elevated inflammatory markers (CRP 172 mg/l, erythrocyte sedimentation rate, ESR 137 mm per hour), normocytic anemia and coagulopathy. During ultrasound imaging of the abdomen, the radiologist described signs of arteritis of the abdominal aorta. Immediately, CT angiography was performed with findings of involvement of abdominal aorta appropriate for inflammation process. The finding was assessed as Takayasu arteritis type 3 according to angiographic classification. MR angiography proved the diagnosis and will be used during follow-up. The patient received 5 pulses of corticosteroid therapy (methylprednisolone) followed by oral corticosteroid (prednisone). Infectious causes of inflammation were excluded. We observed a prompt improvement in clinical features.

On the 12^th^ day, we started treatment with tocilizumab 162 mg administered subcutaneously once a week.

After 4 weeks, ultrasound finding was normal and we observed a decrease in inflammatory markers.

At the time of abstract submission, she is symptoms-free, on therapy with tocilizumab, and her CRP and ESR are normal.

**Conclusion:** Takayasu arteritis is rare large-vessel vasculitis. Common symptoms are non-specific and thus, there could be a delay in diagnosis. In this case, our radiologist noticed inflammatory changes in the abdominal aorta during the standard abdominal ultrasound and it led to a diagnosis. This case highlights both the importance of doing thorough ultrasound imaging and the effect of therapy – high-dose corticosteroid followed by biologics led to clinical improvement.

**Patient Consent:** Yes, I received consent

**Disclosure of Interest**: None declared

